# Multiregional genetic evolution of metastatic uveal melanoma

**DOI:** 10.1038/s41525-021-00233-5

**Published:** 2021-08-16

**Authors:** Daniel A. Rodriguez, Jessica Yang, Michael A. Durante, Alexander N. Shoushtari, Stergios J. Moschos, Kazimierz O. Wrzeszczynski, J. William Harbour, Richard D. Carvajal

**Affiliations:** 1 Bascom Palmer Eye Institute, University of Miami Miller School of Medicine, Miami, FL USA; 2 Sylvester Comprehensive Cancer Center, University of Miami Miller School of Medicine, Miami, FL USA; 3 Interdisciplinary Stem Cell Institute, University of Miami Miller School of Medicine, Miami, FL USA; 4 Memorial Sloan Kettering Cancer Center, New York, NY USA; 5 Lindeberger Comprehensive Cancer Center, UNC School of Medicine, Chapel Hill, NC USA; 6 New York Genome Center, New York, NY USA; 7 Columbia University Irving Medical Center, Herbert Irving Comprehensive Cancer Center, New York, NY USA

**Keywords:** Cancer genomics, Eye cancer, Uveal diseases

## Abstract

Uveal melanoma (UM) is the most common primary intraocular malignancy in adults and leads to deadly metastases for which there is no approved treatment. Genetic events driving early tumor development are well-described, but those occurring later during metastatic progression remain poorly understood. We performed multiregional genomic sequencing on 22 tumors collected from two patients with widely metastatic UM who underwent rapid autopsy. We observed multiple seeding events from the primary tumors, metastasis-to-metastasis seeding, polyclonal seeding, and late driver variants in *ATM*, *KRAS*, and other genes previously unreported in UM. These findings reveal previously unrecognized temporal and anatomic complexity in the genetic evolution of metastatic uveal melanoma, and they highlight the distinction between early and late phases of UM genetic evolution with implications for novel therapeutic approaches.

## Introduction

UM is the most common primary cancer of the eye and leads to metastatic death in up to 50% of patients. The primary tumor arises through an initiating mutation in one of several genes in the Ga_q/11_ signaling pathway (*GNAQ*, *GNA11*, *PLCB4*, or *CYSLTR2*), followed by a “BSE” progression mutation in *BAP1*, *SF3B1* (and rarely other splicing factors), or *EIF1AX*, associated with high, intermediate, and low metastatic risk, respectively^1–7^. These canonical mutations arise early in a punctuated burst or selective sweep within the primary tumor and are often accompanied or followed by copy number variations (CNVs) involving chromosomes 1, 3, 6, and 8^4,7^. In contrast to primary UM, little is known about the genetic evolution of metastatic UM. Published studies to date evaluating metastatic UM either lack matching primary tumors, include only a small number of liver metastases or use targeted sequencing panels^8–11^, limiting the ability to assess tumor evolution over time and across anatomic locations. Here, we performed multiregional genomic sequencing of 22 tumors from two patients with widely metastatic UM involving 10 different organs and tissues using whole-exome sequencing (WES) or whole-genome sequencing (WGS).

## Results

### Patient 1

**Fig. 1:**
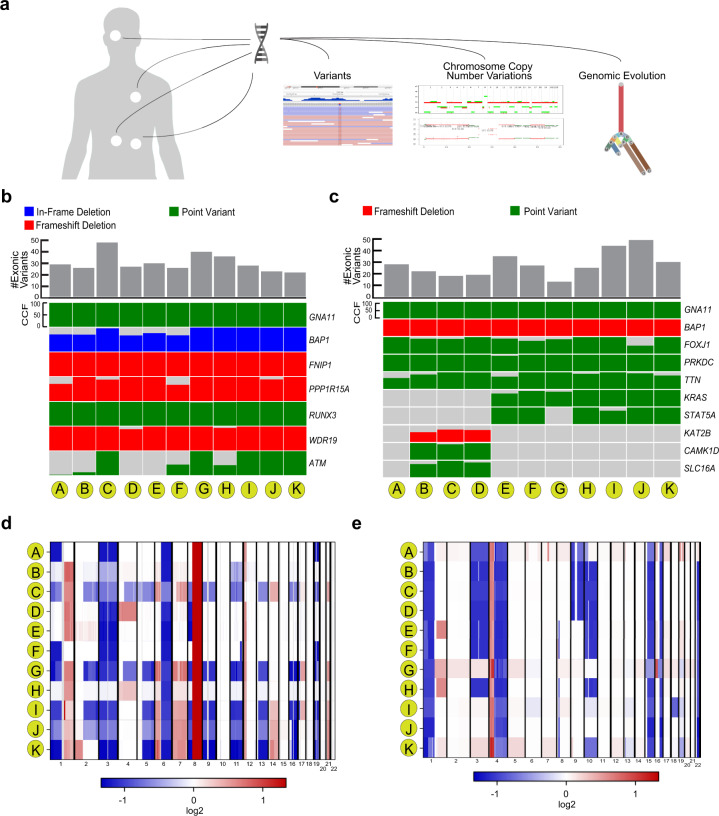
Molecular landscape of 22 tumors collected after rapid autopsy of two patients with metastatic uveal melanoma. **a** Schematic of multiple metastasis and molecular studies. **b**, **c** Co-variant plot of deleterious variants found in Patients 1 and 2, respectively. The height of each colored bar represents the cancer cell fraction of that variant in the indicated sample. For Patient 1, letters correspond to tumors harvested from anatomic sites including: periocular tumor (A), liver segment 4/5 (B), liver segment 4B (C), periportal lymph node (D), 12th rib (E), perisplenic nodule (F), liver segment 2 (G), lung (H), supraclavicular lymph node (I), subcutaneous left abdomen (J), and subcutaneous right cheek (K). For Patient 2, letters correspond to tumor harvested from the indicated anatomic sites including: primary tumor (A), left upper lung (B), right upper lung (C), subcutaneous right chest (D), liver lesion 1 (E), liver lesion 2 (F), right caval lymph node (G), subcutaneous left chest (H), adrenal (I), omentum (J), and retroperitoneum (K). **d**, **e** Copy number variation (CNV) plot of all chromosomes for Patients 1 and 2, respectively. Blue indicates a loss and red indicated a gain. Chromosome numbers are labeled on the horizontal axis. Letters represent the same sample names in panels (**b**) and (**c**) for each respective patient.

A 51-year-old Caucasian man was diagnosed with UM involving the choroid of the left eye and underwent plaque brachytherapy. Three years later, he developed biopsy-proven liver metastasis (Supplementary Table 1 and Fig. 1). He was initially treated with ipilimumab and cyclophosphamide. Four months later, treatment was switched to vorinostat on clinical trial (NCT01587352) for disease progression. After new lesions were detected in the liver, lung, and around the spleen, his therapy was changed to pembrolizumab. Six months later, further disease progression was noted, and he was treated with everolimus and pasireotide on a clinical trial14. Several months later, further disease progression was detected in the liver, with new lesions in the peritoneum and the left 12th rib, and his treatment was changed to binimetinib and sotrastaurin on a clinical trial (NCT01801358). He was subsequently treated with sunitinib and sirolimus. He ultimately received temozolomide but experienced continued disease progression with the development of numerous subcutaneous metastases. He died 21 months after the initial detection of metastatic disease. The radiographic chronology of his disease course is summarized in Supplementary Table 2.

### Patient 2

A 69-year old woman was diagnosed with a stage T3b uveal melanoma involving the ciliary body and choroid of the right eye, treated by enucleation (Supplementary Tables 1 and 3 and Supplementary Fig. 2). Twenty-one months later, small pulmonary nodules were identified and were initially managed by expectant observation. One year later, two biopsy confirmed liver metastases were identified, and treatment with ipilimumab was initiated. Following further disease progression in the liver, she was treated with temozolomide and Yttrium-90 hepatic radioembolization. She then experienced rapid extrahepatic tumor progression and died one year after initial metastatic dissemination. The radiographic chronology of her disease course is summarized in Supplementary Table 3.

### Variant and copy number analysis

Among the two patients, 22 tumor samples were collected via rapid autopsy and processed for genomic analysis (Fig. 1a). In-Patient 1, a periocular tumor was obtained that most likely arose by direct extension from the primary tumor through the sclera, which occurs in about 8% of cases^12^, thereby representing the closest available surrogate for the primary tumor. WGS was performed on tumor samples from Patient 1 and WES was performed on tumor samples from Patient 2. Somatic variants across all 22 samples were tabulated (Supplementary Tables 4–9). The number of exonic variants per tumor ranged from 21 to 47 (median, 27 variants) in Patient 1, and from 13 to 49 (median, 27 variants) in Patient 2. In both patients, canonical mutations in *GNA11* and *BAP1* were present in all tumor samples (Fig. 1b, c and Supplementary Fig. 3). Primary and metastatic tumors also harbored non-canonical variants, some of which were present at or near 100% of tumor cells, while others were present in smaller clones or in a subset of tumors (Fig. 1b, c). CNVs and their cancer cell fractions (CCF) were assessed for all tumor samples (Fig. 1d, e, Supplementary Figs. 4–7, and Supplementary Table 10). CCFs for PPP1R15A or ATM are the same or higher for all metastatic tumors compared to the periocular tumor, further suggesting that this lesion is a direct extension of the primary lesion (Supplementary Table 6). Loss of heterozygosity (LOH) for chromosome 3, which unmasks *BAP1* mutations in metastasizing Class 2 UM^3^, was detected in all tumor samples for both patients, consistent with a canonical aberration that arises early in tumor evolution^4^. There were several additional examples of LOH unmasking variants on the other chromosomal homolog in Patient 1: loss of 1p (including the *RUNX3* locus) in 7 tumors, loss of 5q (*FNIP1*) in 5 tumors, loss of 11q (*ATM*) in 5 tumors, and loss of 19q (*PPP1R15A*) in 7 tumors. Interestingly, 5 tumors from Patient 1 and 4 tumors from Patient 2 exhibited isodisomy 3 in which the retained uniparental, *BAP1*-mutant copy of chromosome 3 underwent duplication. Similar uniparental isodisomy was observed for 1p, 6q, and 8p in some late metastases, suggesting that restoration of heterozygosity may provide a selective advantage during tumor evolution^13,14^. In addition, 10 and 13 nonsynonymous exonic variants were found exclusively in the periocular tumor in Patient 1 (Supplementary Tables 5 and 11) and in the primary tumor in Patient 2 (Supplementary Tables 8 and 11), respectively. These include variants in *BAIAP3*, *TAT*, *THOP1*, and *ZBTB42* in Patient 1 and *ACVR1B*, *ANKRD11*, *CHD4*, *HNRNPM*, *NCK2*, *PAK1IP1*, and *RPS6KA2* in Patient 2.

### Multiregional analysis

**Fig. 2:**
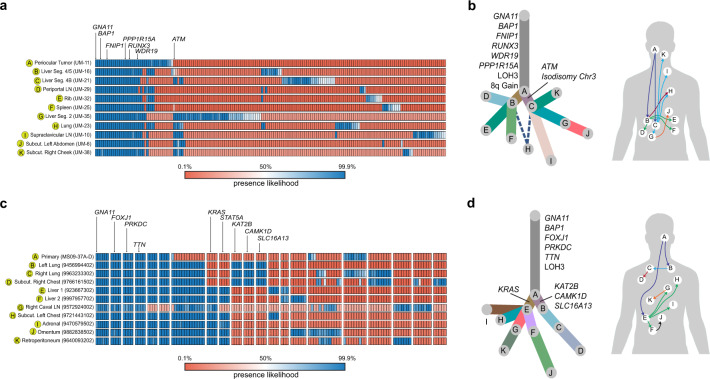
Multiregional tumor seeding analysis. **a** Treeomic heatmap of Patient 1, showing the posterior probability of the presence of variants, with a color legend indicating probability of variant in each tumor sample. **b** Clonality tree and body map in Patient 1, indicating the inferred pattern of metastatic spread. Letters correspond to tumors harvested from indicated anatomic sites including: periocular tumor (A), liver segment 4/5 (B), liver segment 4B (C), periportal lymph node (D), rib (E), spleen (F), liver segment 2 (G), lung (H), supraclavicular lymph node (I), subcutaneous left abdomen (J), and subcutaneous right cheek (K). The dotted line represents a possible polyclonal seeding event. **c** Treeomic heatmap snapshots of Patient 2, showing the posterior probability of the presence of variants, with a color legend indicating probability of variant in each tumor sample. **d** Clonality tree and body map in Patient 2, indicating the inferred pattern of metastatic spread. Letters correspond to tumors harvested from indicated anatomic sites including: primary tumor (A), left lung (B), right lung (C), subcutaneous right chest (D), liver lesion 1 (E), liver lesion 2 (F), right caval lymph node (G), subcutaneous left chest (H), adrenal (I), omentum (J), and retroperitoneum (K).

Multiregional analysis of all 22 tumors was performed using variants and CNVs to reconstruct metastatic evolution over time and anatomic location (Fig. 2a–d and Supplementary Tables 4–10). In both patients, canonical aberrations (*GNA11* and *BAP1* variants, and LOH3) were present in most or all cancer cells from early and late tumors, consistent with punctuated evolution prior to the most recent common tumor ancestor^4^. In-Patient 1, variants in *FNIP1*, *PPP1R15A*, *WDR19*, and *RUNX3* were present in ~100% of cancer cells across all samples. An *ATM*^*G2020C*^ variant was present at ~2% of cancer cell fraction (CCF) in the periocular tumor and expanded to ~100% CCF in one metastatic branch (Branch 1) compared to only 0–44% CCF in the other branch (Branch 2). In Branch 1, expansion of the *ATM* variant during subsequent metastasis-to-metastasis seeding was accompanied by LOH for the other *ATM* allele (Fig. 2a, b and Supplementary Table 6). In Branch 2, by contrast, the *ATM* variant did not expand substantially and was not accompanied by LOH for the other allele, suggesting that it did not drive this metastatic branch. In addition to the *ATM* variant, the two metastatic branches can be distinguished by other variants that are present at ~100% CCF in Branch 1 and mostly absent in Branch 2 (e.g., *WWOX, CTB-178M22.1, CDH26*), and other variants that are present at ~100% CCF in Branch 2 but mostly absent in Branch 1 (e.g., *DBH, CD6, PGAP2*). Interestingly, one lung metastasis (UM-23) harbored the first set of variants at 23–41% and the second set of variants at 76–97%, consistent with polyclonal seeding from two liver metastases (UM-21 and UM-16), with UM-21 from Branch 1 contributing ~25% and UM-16 from Branch 2 contributing ~75% to UM-23 (Supplementary Table 6).

In-Patient 2, non-canonical deleterious variants in *FOXJ1*, *PRKDC*, and *TTN* were detected in ~100% CCF across all tumor samples (Fig. 2c and Supplementary Table 9). There were also two independent seeding events from the primary tumor, one giving rise to a lung metastasis with variants in *KAT2B*, *CAMK1D*, and *SLC16A*, and the other giving rise to a liver metastasis with a subclonal *KRAS*^*G12V*^ variant which expanded to ~100% CCF in 6 downstream metastases that arose shortly before the patient’s death (Fig. 2d).

## Discussion

Here we present a genome-wide sequencing analysis of multi-organ metastatic disease in UM, revealing complex evolutionary events such as recurrent seeding from the eye, metastasis-to-metastasis seeding, and polyclonal seeding. The asymmetric clonal expansion was also observed, with an *ATM* variant in Patient 1 seemingly functioning as a driver in one branch and as a passenger in another. Such evolutionary events are potential mechanisms of heterogeneous treatment response and resistance^15^, and they would likely have been missed using small sequencing panels and small numbers of metastatic samples.

The periocular tumor in Patient 1 and the primary tumor in Patient 2 contained several genetic alterations that were not present in any of their associated metastases (Supplementary Tables 5, 8, and 11). As a possible explanation, the clone that disseminated from the primary tumor may not have contained all of the genomic aberrations found elsewhere in the primary tumor. Alternatively, the primary tumor may have acquired these alterations after the metastatic dissemination had occurred.

In a recent study using a targeted gene panel to analyze metastatic liver tumors and matched primary tumors^10^, it was suggested that LOH for *GNAQ* (but not *GNA11*) is a “tertiary driver” that is required to fully activate mutant *GNAQ* because it is less potent than mutant *GNA11*^10^. However, our findings do not support this claim. LOH for *GNAQ* was present in tumors from both patients despite neither having a *GNAQ* mutation. On the other hand, LOH for mutant *GNA11* on chromosome 19p was observed in 7 tumors from Patient 1. In light of these findings, along with recent studies failing to show a worse prognosis associated with *GNA11* mutations^7,16^, it seems more likely that LOH involving *GNAQ* and *GNA11* is not targeting these genes but rather, one or more tumor suppressor genes on chromosomes 9q and 19p, respectively.

The prior study also suggested that 8q gain is an early aberration that drives metastasis through a progressive increase in copy number. We observed this pattern In Patient 1, where early metastatic tumors contained up to 4 copies of 8q and later tumors up to 12 copies (Supplementary Table 10). However, this pattern was not observed in Patient 2, where only 2 late metastases contained small subclones with one extra copy of 8q. Previous studies have shown that 8q gain occurs frequently, not only in metastasizing class 2 UM, but also in class 1 UM that do not metastasize (albeit usually at a lower dosage), and that 8q gain does not always occur early but can also arise later in tumor evolution^4,17^. As such, while 8q gain can evidently provide a selective advantage during tumor evolution in some cases, it is not a required early event and is not necessary for UM metastasis. Of note, 8q gain is most strongly associated with poor prognosis when it is accompanied by 8p loss, which may occur through the formation of an isochromosome 8q, unmasking a metastasis modifier locus on 8p^18^. Metastatic tumors from both patients in this study demonstrated loss of 8p, including an interstitial deletion spanning this metastasis modifier locus in 7 metastatic tumors from Patient 2 (Fig. 1e and Supplementary Table 10). Further work is needed to clarify the mechanistic role of 8p loss versus 8q gain in UM progression.

Finally, this analysis underscores a key distinction in UM between early recurrent drivers (e.g., primary Gα_q/11_ and secondary BSE mutations) versus late variable “tertiary” variants^10^, which are rare or even one-off events that may provide a selective advantage in specific cases but are not generally necessary for UM development or progression. The *KRAS*^*G12V*^ variant, for example, arose in Patient 2 as a late driver concurrent with her rapid disease progression, yet this variant has never before been reported in UM. Given the genetic heterogeneity in these metastatic tumors and the frequent emergence of resistance to empiric monotherapy, our findings suggest that combination therapies may be necessary and that serial biopsies of metastatic lesions and/or serial sampling of circulating cell-free DNA (cfDNA) will be important to identify new potentially druggable driver mutations that emerge over time. Our study was limited by small sample size, primarily due to technical challenges associated with rapid autopsy tissue procurement, and inadequate information to correlate specific treatments with specific genetic aberrations. Future studies with more patients, serial biopsies in relation to disease progression and therapies administered, and newer technologies such as single-cell sequencing^19^ will continue to shed light on tumor evolution and uncover mechanisms of treatment resistance and opportunities for novel treatment approaches in UM.

## Methods

### Oversight

The rapid autopsy for Patient 1 was performed at Memorial Sloan Kettering Cancer Center Department of Pathology. Patient 2 was performed at the University of North Carolina School of Medicine Department of Pathology and Laboratory Medicine. Both patients provided written informed consent. Both autopsies were conducted understudy protocols approved by the respective institutional review board. Genomic sequencing of the samples was performed understudy protocols approved by the institutional review boards of Memorial Sloan Kettering Cancer Center and Columbia University.

### Patient and specimen collection and processing

All tumor specimens were freshly frozen, with the exception of the primary tumor (specimen A; Supplementary Table 1) from Patient 2, which was formalin-fixed paraffin-embedded (FFPE). FFPE-derived DNA was repaired prior to library preparation using the PreCR repair mix (NEB, M0309L). The process also removes moieties from the 3′ end of DNA leaving a 3′ hydroxyl group compatible with the formation of phosphodiester bonds with 5′ phosphate groups. DNA integrity was subsequently assessed using the Fragment Analyzer (Advanced Analytical, Agilent).

### Radiographic imaging

Imaging studies performed on Patient 1 and Patient 2 throughout the disease course were available for central radiographic review and documentation of the time course for the development of the harvested lesions (Supplementary Tables 2 and 3).

For Patient 1, tumor specimen B (liver segment 4/5) was the first lesion to be radiographically observed. Specimen C (liver segment 4B) was the second observed 3 months later. Three lesions, including specimen E (bone, 12th rib), specimen G (liver segment 2), and specimen F (peritoneal perisplenic nodule) were the third group of lesions to be identified based upon imaging performed 5 months after tumor specimen B was first identified. Specimen H (lung nodule) was identified 2 months later. Four of the lesions, including specimen J (soft tissue subcutaneous nodule, left abdomen), the specimen I (left supraclavicular lymph node), specimen A (periocular tumor), and specimen K (soft tissue subcutaneous nodule, right cheek), were not identified on any of the imaging studies performed. As specimen J (soft tissue subcutaneous nodule, left abdomen), the specimen I (left supraclavicular lymph node), and specimen D (periportal lymph node) were harvested from locations covered by all scans, these three lesions likely represent developmentally late lesions. Specimen A (periocular tumor) and specimen K (soft tissue subcutaneous nodule, right cheek) were harvested from the head and neck region and would have only been potentially visible on the PET/CT images; thus, these lesions would have developed into clinically evident tumors after the last PET/CT scan performed 11 months after tumor specimen B was first identified.

For Patient 2, tumor specimens B (left upper lobe lung mass) and C (right upper lobe lung mass) were the first lesions to be radiographically observed; however, imaging was limited to the chest at this initial timepoint. Specimens E (liver mass) and F (liver mass) were first observed during chest, abdominal and pelvic imaging 11 months later. Six of the lesions, including specimen I (adrenal mass), G (right caval lymph node), H (subcutaneous nodule, left chest), D (subcutaneous nodule, right chest), J (omental mass), and K (retroperitoneal mass) were not identified on any of the imaging studies available for review and likely represent developmentally late lesions.

### DNA library preparation

Genomic sequencing was performed using WES in Patient 1 and WGS in Patient 2. WGS libraries were prepared using the KAPA Hyper Library Preparation Kit in accordance with the manufacturer’s instructions. Briefly, 100–200 ηg of DNA was sheared using a Covaris LE220 sonicator (adaptive focused acoustics). DNA fragments were end-repaired, adenylated, and ligated to Illumina sequencing adapters. Ligated DNA libraries underwent bead-based size selection and were enriched with PCR amplification using 7 cycles. Final libraries were evaluated using fluorescent-based assays including PicoGreen (Life Technologies) or Qubit Fluorometer (Invitrogen) and Fragment Analyzer (Advanced Analytics) or BioAnalyzer (Agilent 2100). Libraries with adapter dimer evident in the final library QC underwent an additional bead-based size selection. All were subsequently sequenced on an Illumina HiSeq X sequencer (v2.5 chemistry) using 2 × 150 bp cycles. WES libraries were prepared using the Agilent SureSelect XT library preparation kit in accordance with the manufacturer’s instructions. Briefly, 1500 ng of DNA was sheared using a Covaris LE220 sonicator (adaptive focused acoustics). DNA fragments were end-repaired, adenylated, ligated to Illumina sequencing adapters. Ligated DNA libraries were enriched with PCR amplification using 6 cycles. Exome capture was performed on the SciClone with 750 ng of the pre-capture library using the SureSelect XT V4 Human All Exon probe set (Agilent) following the manufacturer’s recommendations. Enriched fragments are uniquely indexed during the final amplification process. Final libraries were quantified using fluorescent-based assays including PicoGreen (Life Technologies) or Qubit Fluorometer (invitrogen) and Fragment Analyzer (Advanced Analytics) or BioAnalyzer (Agilent 2100). All libraries were sequenced on an Illumina HiSeq2500 sequencer (v4 chemistry) using 2 × 125 bp cycles.

### Sequence alignment

FASTQ files containing WGS or WES data were processed by checking for quality using FASTQC and paired-end 2 × 150 bp reads were aligned to the human genome (hg19/GRCH37) using the Burrows–Wheeler Aligner (BWA v.0.7.8). Aligned reads were marked for duplicates using Picard and realigned using ABRA^20^. Alignments then underwent read mate fixing and reordering. Unknown or unplaced contigs and mitochondrial genes were excluded from further analysis.

### Variant calling

Post processed alignments underwent variant calling for SNPs and Indels using MuTect2^21^. Mutect2 was used in order to detect low coverage SNPs and to leverage its capability of handling tumors with purity less than 100%, presence of subclonal variants, and/or copy number variations. Variants for each tumor specimen were called against a matched blood specimen for each patient. Variants that were called and marked as passed were aggregated. These calls were further filtered by keeping variants that had an alternate tumor read count of ≥3 or variants that had an alternate tumor read count that was >20% of the total read count. For all sequencing specimens, the BAM files and raw MuTect2 calls were investigated manually for canonical UM variants (*GNA11*, *GNAQ*, *BAP1*, *SF3B1*, *EIF1AX*, *CYSLTR2*, and *PLCB4*), if present these variants were added to our final list. For all called variants Annovar was used for annotation^22^. Following annotation, variants were further filtered out if the minor allele frequency (MAF) was 1% greater in the 1000 Genomes Project population (2015 August), Exome Sequencing Project, or listed in dbSNP (v138). Functional consequences of variants were assessed by four predictor tools: SIFT^23^, Polyphen2 HDIV^24^, FATHMM^25^, and MetaLR^26^. Single nucleotide variants were considered deleterious if at least two out of the four tools predicted it as deleterious or possibly deleterious. Deletions and insertions were assessed using SIFTindel^27^ and were considered deleterious if predicted to be deleterious to the protein product or to result in nonsense-mediated decay (NMD).

### Copy number variations

Copy number gains and losses were determined using CNVKit^28^. Copy number subclones, monosomy, and other duplication events were assessed using cgpBattenberg^29^. This was also used to assess the normal contamination and cellularity of each specimen.

### Metastatic seeding reconstruction

We utilized Treeomics (v1.7.13)^30^ in order to leverage both driver and passenger variants identified in the WGS and WES of each specimen to infer evolutionary phylogeny. Due to the large amounts of variants captured for the primary tumor in Patient 2, only variants shared in at least one other sample were used. This approach estimated the posterior probabilities for the presence or absence of a variant in a specimen based on a Bayesian binomial likelihood model. We calculated the Jaccard similarity coefficients for the various specimens present in each individual patient in order to assess the similarity of specimens and length of evolution after seeding. Variant clusters at phylogenic branching events were assessed in order to infer metastatic spread. CCF was determined for sets of variants in order to infer possible polyclonal seeding events.

### Reporting summary

Further information on research design is available in the Nature Research Reporting Summary linked to this article.

## Supplementary information

Supplementary Information

Reporting Summary

## Data Availability

All sequencing data generated in this study have been deposited in and are available from the dbGaP database under dbGaP accession phs002491.v1.p1.

## Code availability

All software used is publicly available including FastQC v0.11.3, BWA v.0.7.8, Picard v1.128, ABRA v0.94c, MuTect2, CNVKit, Annovar, cgpBattenberg, Treeomics v1.7.13. dbSNP v138 and Human genome hg19/GRCH37 were used throughout the analysis.
