# Supplementary material for: Multiregional genetic evolution of metastatic uveal melanoma
Source: NPJ Genom Med. 2021 Aug 16;6:70. doi: 10.1038/s41525-021-00233-5 (PMC8368296; doi:10.1038/s41525-021-00233-5)
Supplement: Supplementary file 2 — Reporting Summary [file 41525_2021_233_MOESM2_ESM.pdf]

nature research

Corresponding author(s): J. William Harbour

Last updated by author(s): May 16, 2021

Reporting Summary

Nature Research wishes to improve the reproducibility of the work that we publish. This form provides structure for consistency and transparency in reporting. For further information on Nature Research policies, see our Editorial Policies and the Editorial Policy Checklist.

Statistics

For all statistical analyses, confirm that the following items are present in the figure legend, table legend, main text, or Methods section.

n/a	Confirmed	
☐	☒	The exact sample size (*n*) for each experimental group/condition, given as a discrete number and unit of measurement
☐	☒	A statement on whether measurements were taken from distinct samples or whether the same sample was measured repeatedly
☐	☒	The statistical test(s) used AND whether they are one- or two-sided<br>*Only common tests should be described solely by name; describe more complex techniques in the Methods section.*
☒	☐	A description of all covariates tested
☐	☒	A description of any assumptions or corrections, such as tests of normality and adjustment for multiple comparisons
☐	☒	A full description of the statistical parameters including central tendency (e.g. means) or other basic estimates (e.g. regression coefficient) AND variation (e.g. standard deviation) or associated estimates of uncertainty (e.g. confidence intervals)
☒	☐	For null hypothesis testing, the test statistic (e.g. *F*, *t*, *r*) with confidence intervals, effect sizes, degrees of freedom and *P* value noted<br>*Give P values as exact values whenever suitable.*
☐	☒	For Bayesian analysis, information on the choice of priors and Markov chain Monte Carlo settings
☒	☐	For hierarchical and complex designs, identification of the appropriate level for tests and full reporting of outcomes
☒	☐	Estimates of effect sizes (e.g. Cohen's *d*, Pearson's *r*), indicating how they were calculated

Our web collection on statistics for biologists contains articles on many of the points above.

Software and code

Policy information about availability of computer code

Data collection	dbSNP v138, Human genome hg19/GRCH37
Data analysis	FastQC v0.11.3, BWA v.0.7.8, Picard v1.128, ABRA v0.94c, MuTect2, CNVKit, Annovar, cgpBattenberg, Treeomics v1.7.13

For manuscripts utilizing custom algorithms or software that are central to the research but not yet described in published literature, software must be made available to editors and reviewers. We strongly encourage code deposition in a community repository (e.g. GitHub). See the Nature Research guidelines for submitting code & software for further information.

Data

Policy information about availability of data

All manuscripts must include a data availability statement. This statement should provide the following information, where applicable:
- Accession codes, unique identifiers, or web links for publicly available datasets
- A list of figures that have associated raw data
- A description of any restrictions on data availability

All sequencing data generated in this study has been deposited in and are available from the dbGaP database under dbGaP accession phs002491.v1.p1.

Field-specific reporting

Please select the one below that is the best fit for your research. If you are not sure, read the appropriate sections before making your selection.

☒ Life sciences ☐ Behavioural & social sciences ☐ Ecological, evolutionary & environmental sciences

For a reference copy of the document with all sections, see nature.com/documents/nr-reporting-summary-flat.pdf

Life sciences study design

All studies must disclose on these points even when the disclosure is negative.

Sample size	The study was exploratory in nature and a pre-determined sample size was not specified. Cases were obtained as part of a prospective study. The final sample size was determined by the number of cases that became available.
Data exclusions	No data was excluded from the analysis.
Replication	Given the limited availability of sample and time and overall nature of the study, replication experiments were not conducted.
Randomization	Randomization is not applicable to our study design.
Blinding	Blinding was not applicable to our study design.

Reporting for specific materials, systems and methods

We require information from authors about some types of materials, experimental systems and methods used in many studies. Here, indicate whether each material, system or method listed is relevant to your study. If you are not sure if a list item applies to your research, read the appropriate section before selecting a response.

Materials & experimental systems

n/a	Involved in the study	
☒	☐	Antibodies
☒	☐	Eukaryotic cell lines
☒	☐	Palaeontology and archaeology
☒	☐	Animals and other organisms
☐	☒	Human research participants
☒	☐	Clinical data
☒	☐	Dual use research of concern

Methods

n/a	Involved in the study	
☒	☐	ChIP-seq
☒	☐	Flow cytometry
☒	☐	MRI-based neuroimaging

Human research participants

Policy information about studies involving human research participants

Population characteristics	Our report involved 22 tumor samples from 2 Patients, 1 male and 1 female. 11 tumors sampled from each patient; 1 primary tumor and 10 metastatic tumors.
Recruitment	The rapid autopsy for Patient 1 was performed at Memorial Sloan Kettering Cancer Center Department of Pathology. Patient 2 was performed at the University of North Carolina School of Medicine Department of Pathology and Laboratory Medicine. Both patients provided written informed consent and were recruited by treating physicians.
Ethics oversight	Both autopsies were conducted under study protocols approved by the respective institutional review board. Genomic sequencing of the samples was performed under study protocols approved by the institutional review boards of Memorial Sloan Kettering Cancer Center and Columbia University.

Note that full information on the approval of the study protocol must also be provided in the manuscript.
