# Supplementary material for: Multiregional genetic evolution of metastatic uveal melanoma
Source: NPJ Genom Med. 2021 Aug 16;6:70. doi: 10.1038/s41525-021-00233-5 (PMC8368296; doi:10.1038/s41525-021-00233-5)
Supplement: Supplementary file 1 — Supplementary Information [file 41525_2021_233_MOESM1_ESM.pdf]

Supplementary Material

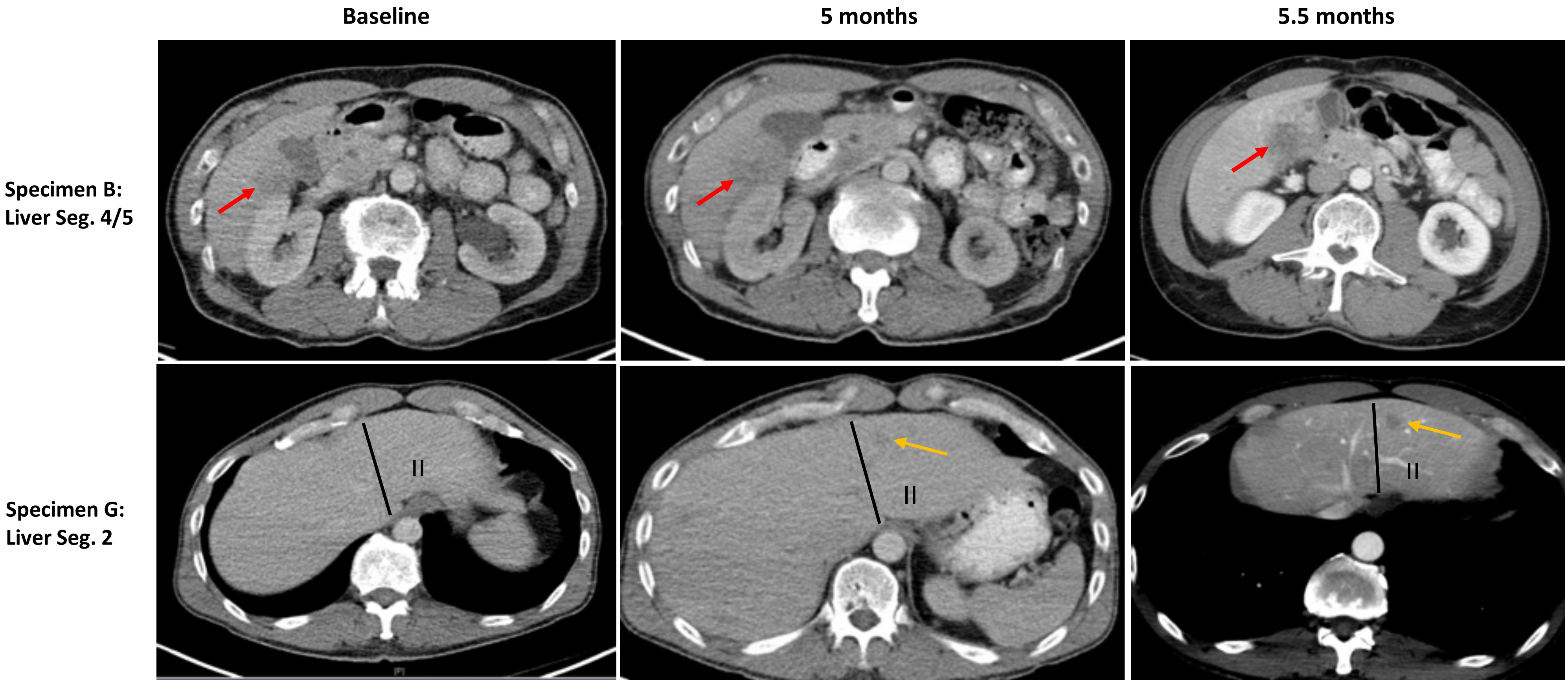

Supplementary Figure 1. Radiographic documentation of time course for the development of harvested lesions for Patient 1. Specimen B (Liver Seg. 4/5) was the first lesion observed on imaging (red arrows). Specimen G (Liver Seg. 2) was not identified until 5 months after the baseline scan (yellow arrows).

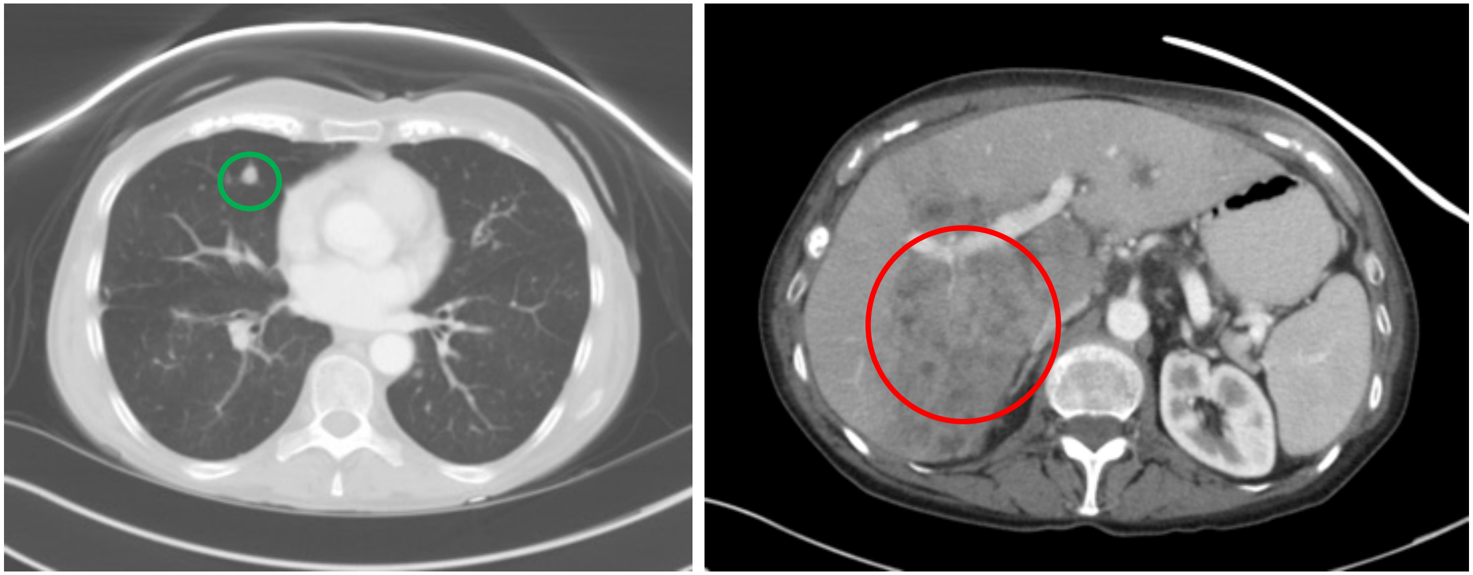

Supplementary Figure 2. Axial CT images of lung (green circle) and liver (red circle) lesions obtained at 21 months for Patient 2.

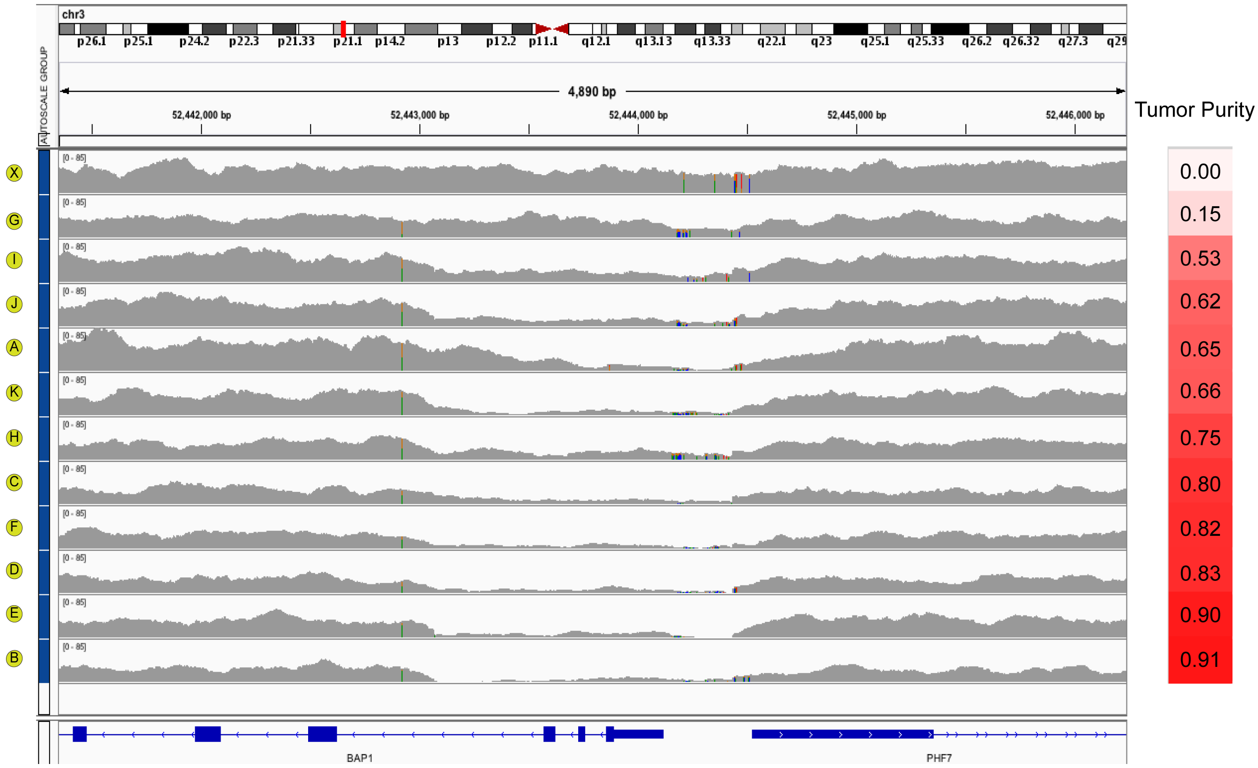

Supplementary Figure 3. IGV snapshot of DNA sequencing tracks around the first five exons of the *BAP1* locus in the 12 specimens from Patient 2, showing a large deletion encompassing the first three exons. Tumor purity refers to the inferred proportion of tumor cells in the indicated specimen harboring the BAP1 mutation. Samples include: primary tumor (A), left upper lung (B), right upper lung (C), subcutaneous right chest (D), liver lesion 1 (E), liver lesion 2 (F), right caval lymph node (G), subcutaneous left chest (H), adrenal (I), omentum (J), retroperitoneum (K), and normal tissue (X).

Patient 1

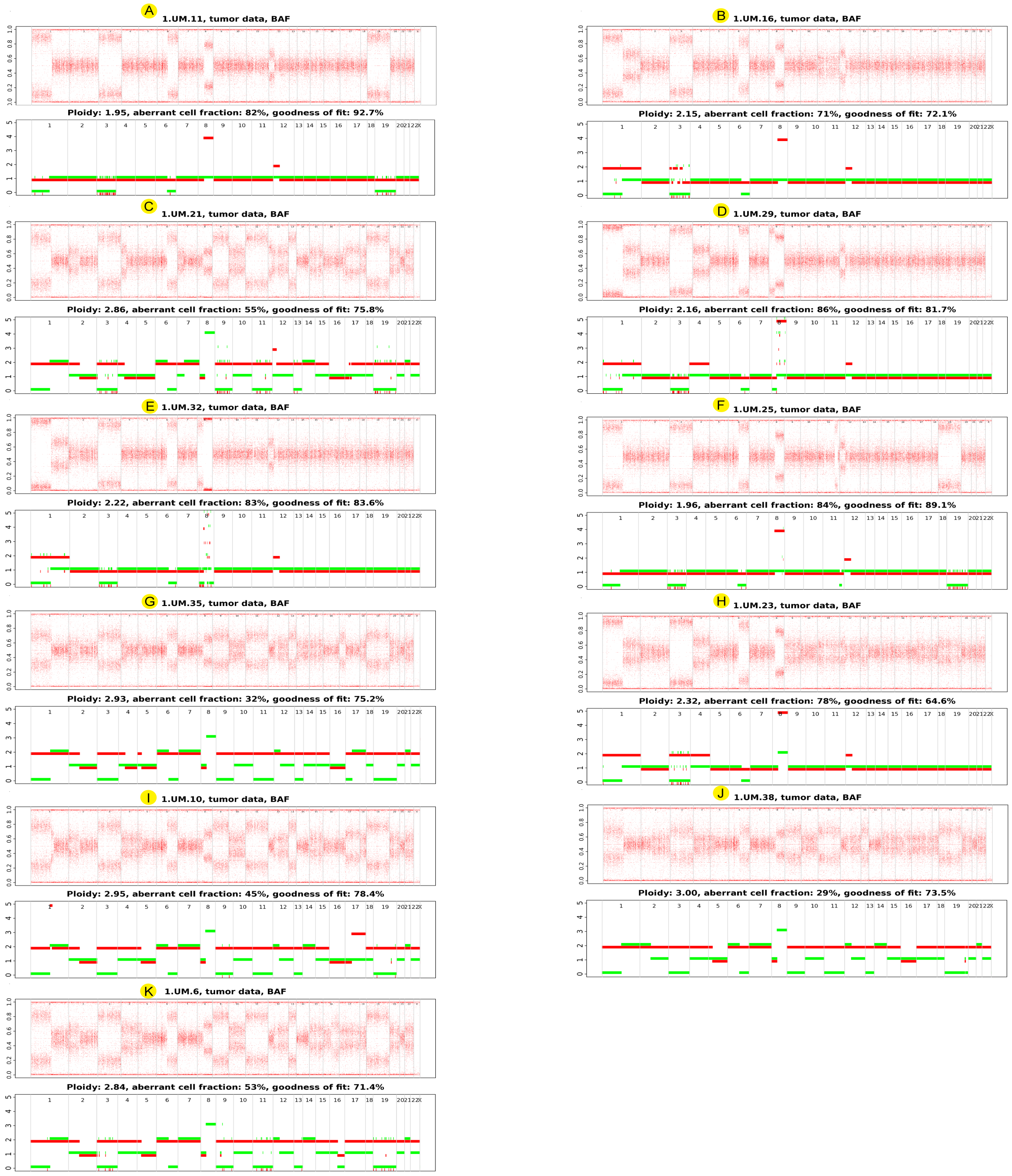

Supplementary Figure 4. Chromosome copy number variation (CNV) plots for samples from Patient 1 depicting minor allele frequency (MAF) (top panel) and ASCAT (bottom panel) plots for each sample. Samples include: periocular tumor (A), liver segment 4/5 (B), liver segment 4B (C), periportal lymph node (D), 12th rib (E), perisplenic nodule (F), liver segment 2 (G), lung (H), supraclavicular lymph node (I), subcutaneous left abdomen (J), and subcutaneous right cheek (K).

Patient 1

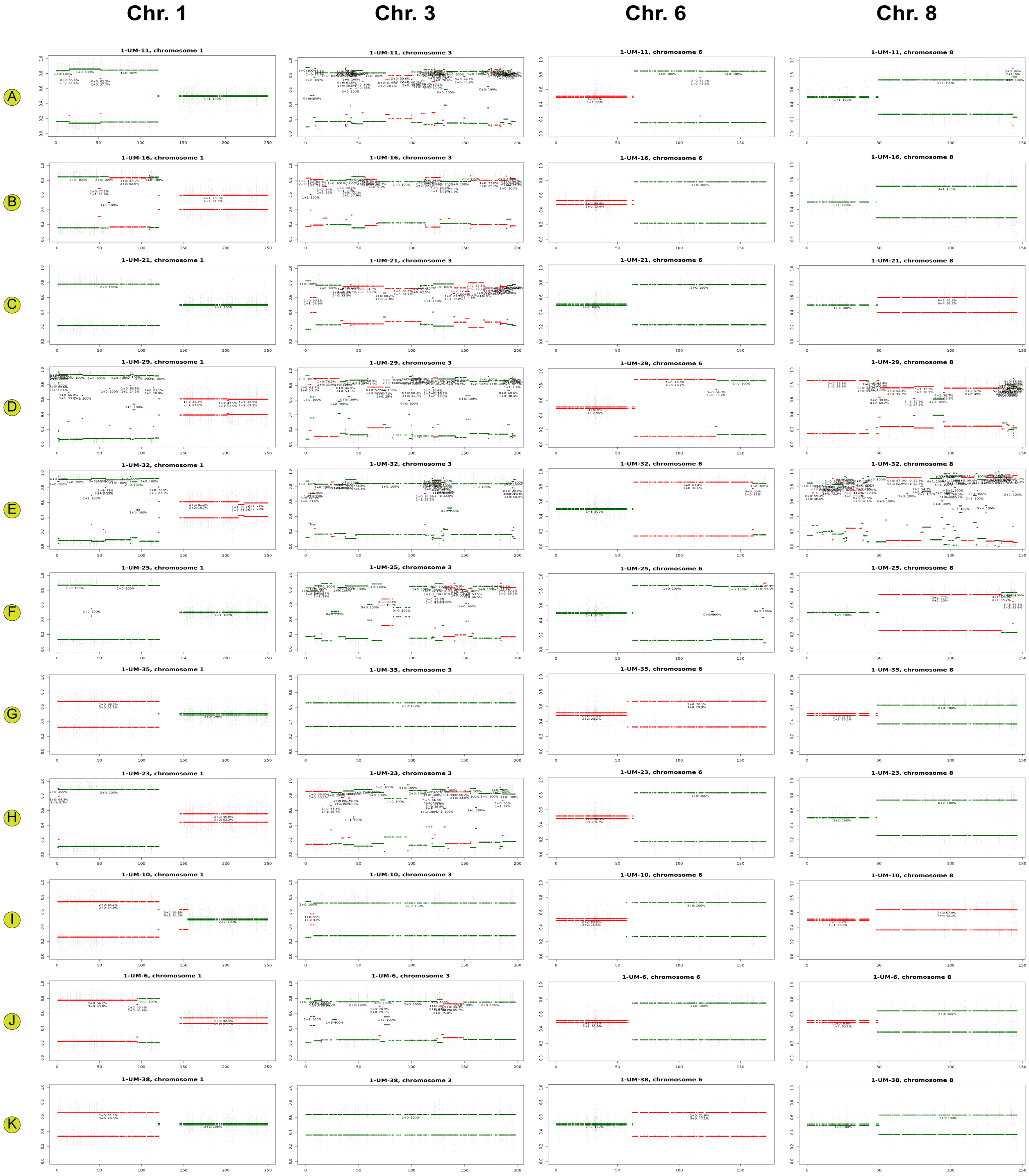

Supplementary Figure 5. Clonality analysis using cgpBattenberg on samples from Patient 1 to estimate copy number subclonality for chromosomes 1, 3, 6, and 8. Samples include: periocular tumor (A), liver segment 4/5 (B), liver segment 4B (C), periportal lymph node (D), 12th rib (E), perisplenic nodule (F), liver segment 2 (G), lung (H), supraclavicular lymph node (I), subcutaneous left abdomen (J), and subcutaneous right cheek (K).

Patient 2

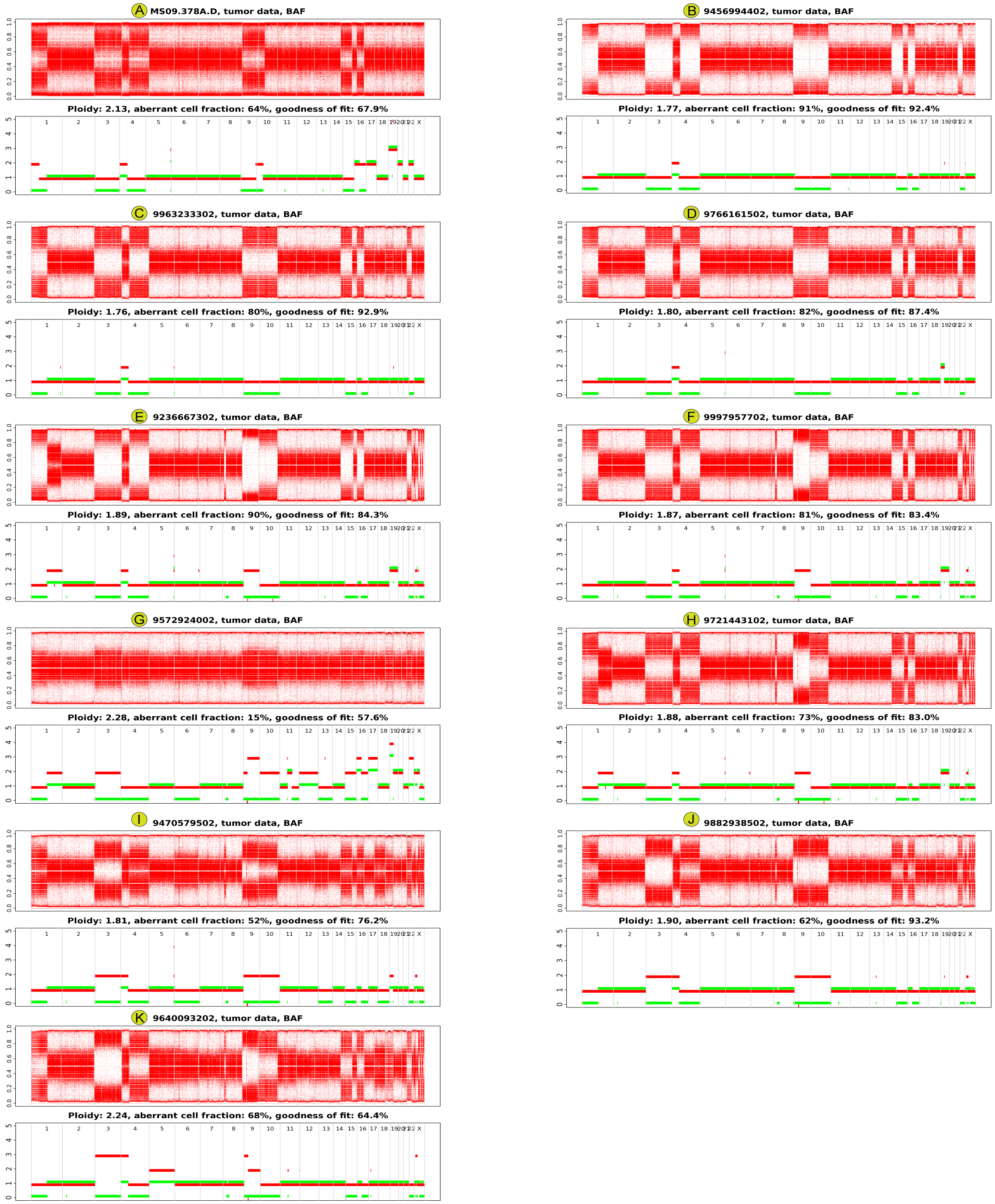

Supplementary Figure 6. Chromosome copy number variation plots for samples from Patient 2 depicting minor allele frequency (MAF) (top panel) and ASCAT (bottom panel) plots for each sample. Samples include: primary tumor (A), left upper lung (B), right upper lung (C), subcutaneous right chest (D), liver lesion 1 (E), liver lesion 2 (F), right caval lymph node (G), subcutaneous left chest (H), adrenal (I), omentum (J), and retroperitoneum (K).

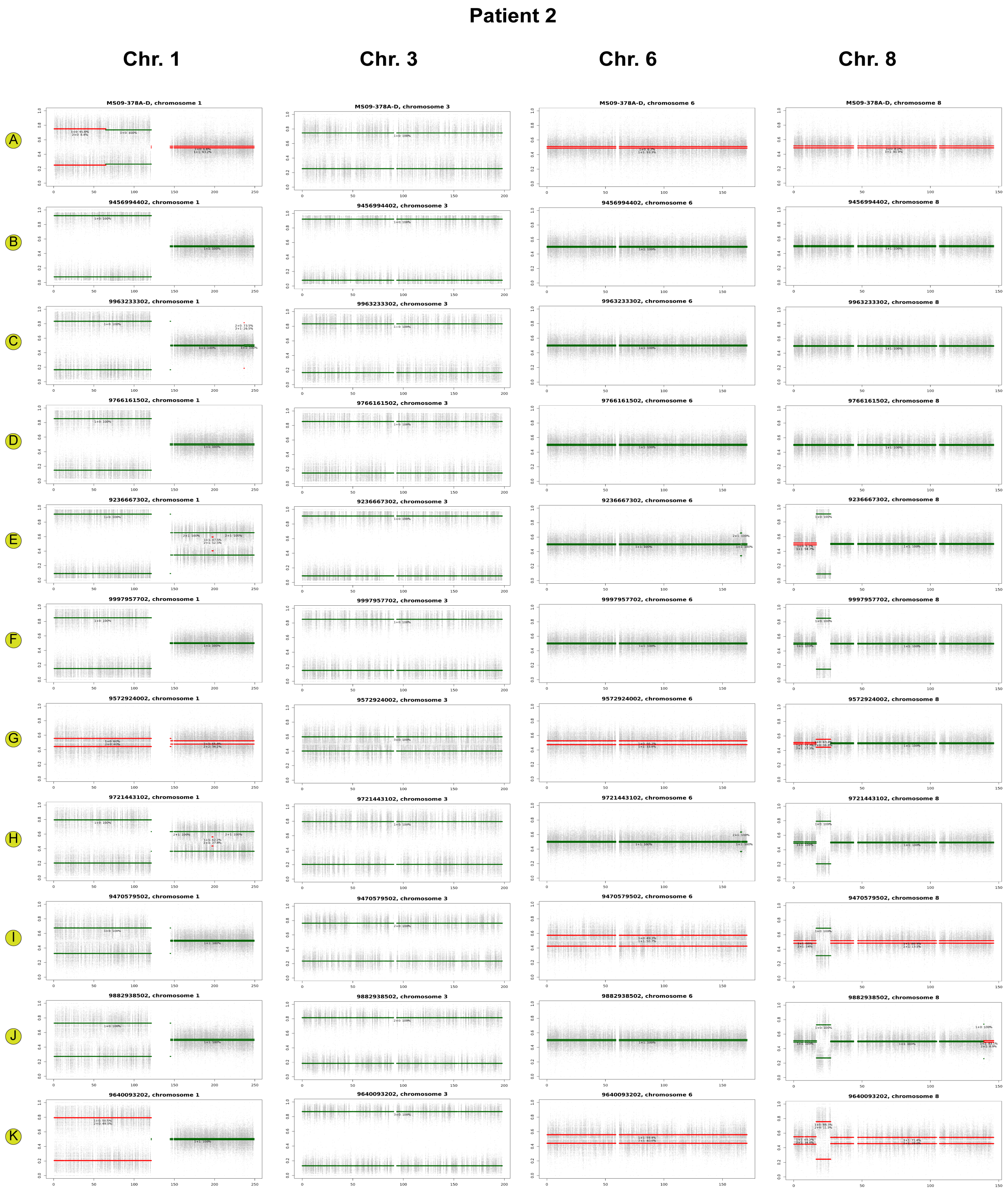

Supplementary Figure 7. Clonality analysis using cgpBattenberg on samples from Patient 2 to estimate chromosome copy number subclonality for chromosomes 1, 3, 6, and 8. Samples include: primary tumor (A), left upper lung (B), right upper lung (C), subcutaneous right chest (D), liver lesion 1 (E), liver lesion 2 (F), right caval lymph node (G), subcutaneous left chest (H), adrenal (I), omentum (J), and retroperitoneum (K).

Supplementary Table 1. Samples collected

Patient	Autopsy ID	Specimen ID	Specimen Site	Specimen Subsite	Specimen Type	Mean Coverage
1	UM-11	A	Eye	Periocular tumor	Fresh frozen	185.38
	UM-16	B	Liver	Segment 4/5	Fresh frozen	187.03
	UM-21	C	Liver	Segment 4B	Fresh frozen	186.00
	UM-35	G	Liver	Segment 2	Fresh frozen	186.69
	UM-23	H	Lung	Lung nodule	Fresh frozen	185.38
	UM-32	E	Bone	12th rib	Fresh frozen	184.42
	UM-25	F	Peritoneal	Perisplenic nodule	Fresh frozen	187.00
	UM-10	I	Lymph node	Left supraclavicular lymph node	Fresh frozen	185.16
	UM-29	D	Lymph node	Periportal lymph node	Fresh frozen	185.67
	UM-6	J	Skin	Subcutaneous nodule, left abdomer	Fresh frozen	186.04
	UM-38	K	Skin	Subcutaneous nodule, right cheek	Fresh frozen	187.57
2	MS09-37A-D	A	Eye	Primary tumor	Paraffin embedded	49.89
	9236667302	E	Liver	Liver mass	Fresh frozen	45.57
	9997957702	F	Liver	Liver mass	Fresh frozen	47.10
	9456994402	B	Lung	Left upper lobe lung mass	Fresh frozen	40.20
	9963233302	C	Lung	Right upper lobe lung mass	Fresh frozen	46.31
	9470579502	I	Adrenal gland	Adrenal mass	Fresh frozen	46.50
	9572924002	G	Lymph node	Right caval lymph node	Fresh frozen	43.41
	9721443102	H	Skin	Subcutaneous nodule, left chest	Fresh frozen	47.12
	9766161502	D	Skin	Subcutaneous nodule, right chest	Fresh frozen	41.20
	9882938502	J	Omentum	Omental mass	Fresh frozen	46.73
	9640093202	K	Retroperitoneum	Retroperitoneal mass	Fresh frozen	41.01

Supplementary Table 2. Patient 1 findings on radiographic imaging

				Imaging Studies and Timepoint (Months) Relative to the Baseline PET/CT Scan											
				0	3 months	5 months	5 months	7 months	10 months	11 months	14 months	15 months	17 months	19 months	19 months
Radiographically Determined Order of Development	Metastatic map labeling	Specimen Site	Date of First Possible/Definite Radiographic Development	PET CT w/ Contrast	PET CT w/ Contrast	PET CT w/ Contrast[a]	CT Chest/Abd/ Pelvis w/ Contrast	CT Chest/Abd/ Pelvis w/ Contrast	CT Chest/Abd/ Pelvis w/ Contrast	PET CT and CT Chest/Abd/ Pelvis w/ Contrast	CT Chest/Abd/ Pelvis w/ Contrast	CT Chest/Abd/ Pelvis w/ Contrast	CT Chest/Abd/ Pelvis w/ Contrast	MR Abd/Pelvis w/ and w/o Contrast[b]	CT Chest w/ Contrast
1	B	Liver Segment 4/5	10/9/12	Present	Present, increased	Present, slightly increased	Present, increased	Present, stable	Present, increased	Present, increased	Present, increased	Present, increased	Present, increased	Present, increased	N/A
2	C	Liver Segment 4B	1/22/13	NS	Present	Present	Present, increased	Present, stable	Present, increased	Present, increased	Present, increased	Present, increased	Present, increased	Present, increased	N/A
3	E	Bone, 12th Rib	3/2/13	NS	NS	Possibly present on PET	NS	Possibly present in retrospect	Possibly present in retrospect	Present[c]	Present, stable	Present, increased	Present, increased	Present, increased	Present, increased
3	G	Liver Segment 2	3/2/13	NS	NS	Possibly present	Present	Present, stable	Present, increased	Present, increased	Present, increased	Present, increased	Present, increased	Present, increased	N/A
3	F	Peritoneal Perisplenic Nodule	3/20/13	NS	NS	NS	Present	Present, increased	Present, increased	Present, stable	Present, increased	Present, increased	Present, increased	Present, increased	N/A
4	H	Lung Nodule	5/14/13	NS	NS[d]	NS[d]	NS[d]	Possibly present[e]	Possibly present[f]	Possibly present, decreased	Possibly present, stable[g]	Possibly present, stable/decreased	Possibly present, stable	N/A	Possibly present, few increased in size
5	A	Periocular tumor	NS[h]	NS	NS	NS	N/A	N/A	N/A	NS	N/A	N/A	N/A	N/A	N/A
5	K	Soft Tissue Subcutaneous Nodule, Right Cheek	NS[h]	NS	NS	NS	N/A	N/A	N/A	NS	N/A	N/A	N/A	N/A	N/A
6	D	Periportal Lymph Node	NS	NS	NS	NS	NS	NS	NS	NS	NS	NS	NS	NS	N/A
6	J	Soft Tissue Subcutaneous Nodule, Left Abdomen	NS	NS	NS	NS	NS	NS	NS	NS	NS	NS	NS	NS	N/A
6	I	Left Supraclavicular Lymph Node	NS	NS	NS	NS	NS	NS	NS	NS	NS	NS	NS	N/A	NS

NS - not seen. N/A - not applicable. [a]Limited image quality. [b]Multiple new/increased subcutaneous and intramuscular metastases. [c]Possibly present on prior PET from 3/2/13, but very limited images and data from this study. Heterogeneity with possible lytic area on corresponding CT, stable when compared to prior CT scans (only noted in retrospect). [d]No suspicious nodules, patchy left lung infiltrates with interval decrease between 1/22/13 and 3/20/13. [e]Near resolution of prior infiltrates/groundglass opacities, several new sub-cm bilateral nodules. [f]Several prior pulmonary nodules resolved, but new sub-cm cavitary nodules. [g]New LLL sub-cm nodule. [h]Due to location, would only have been potentially visable on total body PET/CT imaging, with the last such study performed on 09/26/2013

Supplementary Table 3. Patient 2 findings on radiographic imaging

Radiographically Determined Order of Development	Specimen ID	Specimen Site	Date of First Possible/Definite Radiographic Development	Imaging Studies and Timepoint (Months) Relative to the Baseline CT Chest		
				0	11 Months	21 Months
				CT Chest	CT CAP	CT CAP
1	A	Primary tumor	1/1/09	N/A	N/A	N/A
2	B	Left upper lobe lung mass	10/1/10	Possibly present	Possibly present	Present
2	C	Right upper lobe lung mass	10/1/10	Possibly present	Possibly present	Present
3	E	Liver mass	9/1/11	NS	Possibly present	Present
3	F	Liver mass	9/1/11	NS	Possibly present	Present
4	I	Adrenal mass	NS	NS	NS	NS
4	G	Right caval lymph node	NS	NS	NS	NS
4	H	Subcutaneous nodule, left chest	NS	NS	NS	NS
4	D	Subcutaneous nodule, right ches	NS	NS	NS	NS
4	J	Omental mass	NS	NS	NS	NS
4	K	Retroperitoneal mass	NS	NS	NS	NS

NS - not seen. N/A - not applicable.

Supplementary Table 4. Patient 1 variant locations

Mutation Type	UM-10	UM-11	UM-16	UM-21	UM-23	UM-25	UM-29	UM-32	UM-35	UM-38	UM-6	Total	Average	Median	Min	Max
splicing	0	0	2	0	0	1	1	0	4	0	0	8	0.7	0.0	0.0	4.0
exonic	27	28	25	47	35	25	26	29	39	21	22	324	29.5	27.0	21.0	47.0
intronic	20	21	18	22	25	20	16	17	29	17	15	220	20.0	20.0	15.0	29.0
UTR3	1	1	2	0	1	1	3	2	2	0	0	13	1.2	1.0	0.0	3.0
intergenic	3	3	1	1	1	1	2	2	4	1	2	21	1.9	2.0	1.0	4.0
ncRNA_intron	1	0	1	1	1	1	1	0	2	0	1	9	0.8	1.0	0.0	2.0
downstream	0	0	0	0	0	1	0	0	0	0	0	1	0.1	0.0	0.0	1.0
UTR5	0	0	1	0	0	0	0	0	2	0	0	3	0.3	0.0	0.0	2.0
ncRNA_exoni	1	0	1	1	1	1	2	1	0	0	0	8	0.7	1.0	0.0	2.0
upstream	0	0	1	1	0	0	0	0	0	0	0	2	0.2	0.0	0.0	1.0
total	53	53	52	73	64	51	51	51	82	39	40	609	55.4	52.0	39.0	82.0

Supplementary Table 5. Patient 1 variant spreadsheet

Chr	Pos	Ref	Alt	Func.refGene	Gene.refGene	ExonicFunc.refGene	CLINSIG	CLNDBN
7	87737433	T	C	intronic	*ADAM22*	.	.	.
3	52443618	TTGACAC CTGCGA	T	exonic	*BAP1*	nonframeshift_deletion	.	.
12	1991962	T	A	intronic	*CACNA2D4*	.	.	.
2	202672594	C	T	exonic	*CDK15*	nonsynonymous_SNV	.	.
16	72137069	G	A	exonic	*DHX38*	synonymous_SNV	.	.
20	32699819	G	GC	intronic	*EIF2S2*	.	.	.
19	46133236	G	C	exonic	*EML2*	synonymous_SNV	.	.
5	131008335	TCTGA	T	exonic	*FNIP1*	frameshift_deletion	.	.
19	3118942	A	T	exonic	*GNA11*	nonsynonymous_SNV	.	.
1	22168912	C	G	intronic	*HSPG2*	.	.	.
3	19295441	G	C	intronic	*KCNH8*	.	.	.
2	136594566	G	T	exonic	*LCT*	nonsynonymous_SNV	.	.
17	17072418	C	G	intronic	*MPRIP*	.	.	.
19	49376879	AG	A	exonic	*PPP1R15A*	frameshift_deletion	.	.
1	25254076	C	G	exonic	*RUNX3*	nonsynonymous_SNV	.	.
8	118183310	G	A	exonic	*SLC30A8*	synonymous_SNV	.	.
2	220499339	C	G	intronic	*SLC4A3*	.	.	.
17	26913492	TTAA	T	exonic	*SPAG5*	nonframeshift_deletion	.	.
9	93657773	C	A	intronic	*SYK*	.	.	.

22	22550478	A	C	intergenic	*TOP3B,VPREB1*	.	.	.
4	39276552	CAAAAAGAA	C	exonic	*WDR19*	frameshift_deletion	.	.
4	88047567	G	T	intronic	*AFF1*	.	.	.
19	56369444	G	A	exonic	*NLRP4*	nonsynonymous_SNV	.	.
8	182999	C	A	intronic	*ZNF596*	.	.	.
11	108186601	G	T	exonic	*ATM*	nonsynonymous_SNV	.	.
16	79245631	C	T	exonic	*WWOX*	nonsynonymous_SNV	.	.
7	2966354	G	A	intronic	*CARD11*	.	.	.
11	60739451	C	G	intronic	*CD6*	.	.	.
20	58562535	G	A	exonic	*CDH26*	nonsynonymous_SNV	.	.
9	136504958	G	T	intronic	*DBH*	.	.	.
11	3846749	A	G	UTR3	*PGAP2*	.	.	.
5	167589809	T	A	intronic	*TENM2*	.	.	.
16	49670012	G	A	exonic	*ZNF423*	synonymous_SNV	.	.
19	50837232	C	T	ncRNA_exonic	*NAPSB*	.	.	.
14	21796857	T	TAGATGC	intronic	*RPGRIP1*	.	.	.
12	133795438	C	A	UTR3	*ANHX*	.	.	.
17	45127835	C	CCAA	intergenic	*ARL17A,CDC27*	.	.	.
14	99988497	C	T	exonic	*CCDC85C*	synonymous_SNV	.	.
19	1979877	G	A	intronic	*CSNK1G2*	.	.	.
3	58395490	G	GTT	intronic	*PXK*	.	.	.
12	96259689	T	G	intronic	*SNRPF*	.	.	.
12	32903685	A	C	exonic	*YARS2*	synonymous_SNV	.	.

7	5567522	TCTGAGGAGGGAAGGGGACAGGCAGTGAGGACCCTGGATGTGACAGCTCCCCACACACCACAGGACCCCACAGCCGACCTGCCCAGGTCAGCTCAGGCAGGAAAGACACCCAC	T	splicing	*ACTB*	.	.	.
17	79477859	TCTGCAAAGACAGCCAGGCACGGCTTCAGCTCACAGAGCGCCCCCCAGTCAGCTCTCCCGAGCCAGGCAGAGGGCCACCAACCCCTCGACTCAC	T	splicing	*ACTG1*	.	.	.
19	39207763	A	G	exonic	*ACTN4*	nonsynonymous_SNV	.	.
8	143562702	A	G	exonic	*ADGRB1*	nonsynonymous_SNV	.	.
5	90046497	T	C	exonic	*ADGRV1*	nonsynonymous_SNV	.	.
2	27278969	T	G	exonic	*AGBL5*	nonsynonymous_SNV	.	.

1	109395112	T	C	exonic	*AKNAD1*	nonsynonymous_SNV	.	.
4	73957966	G	C	exonic	*ANKRD17*	nonsynonymous_SNV	.	.
10	37506752	G	A	exonic	*ANKRD30A*	synonymous_SNV	.	.
1	155308009	C	T	exonic	*ASH1L*	nonsynonymous_SNV	.	.
5	160030837	T	C	intronic	*ATP10B*	.	.	.
12	57039583	G	A	intronic	*ATP5B*	.	.	.
7	99026833	G	C	intronic	*ATP5J2-PTCD1,PTCD1*	.	.	.
6	31504235	A	G	ncRNA_intronic	*ATP6V1G2-DDX39B*	.	.	.
8	54741996	A	G	intronic	*ATP6V1H*	.	.	.
17	8111119	G	A	exonic	*AURKB*	nonsynonymous_SNV	.	.
8	104213046	A	C	intronic	*BAALC*	.	.	.
16	1398005	G	A	exonic	*BAIAP3*	nonsynonymous_SNV	.	.
14	74514785	C	T	intronic	*BBOF1*	.	.	.
16	31121043	G	A	exonic	*BCKDK*	nonsynonymous_SNV	.	.
X	70749536	A	T	ncRNA_intronic	*BCYRN1*	.	.	.
11	27911050	C	G	intergenic	*BDNF,KIF18A*	.	.	.
15	60060556	T	G	intergenic	*BNIP2,FOXB1*	.	.	.
17	8092440	C	T	exonic	*BORCS6*	nonsynonymous_SNV	.	.
X	79947484	A	G	intronic	*BRWD3*	.	.	.
12	14975932	T	G	exonic	*C12orf60*	nonsynonymous_SNV	.	.
1	150246585	A	C	intronic	*C1orf54*	.	.	.
5	39315953	T	C	exonic	*C9*	nonsynonymous_SNV	.	.

3	62518628	G	A	exonic	*CADPS*	nonsynonymous_SNV	.	.
19	1229293	T	C	downstream	*CBARP,STK11*	.	.	.
22	21989890	G	A	intronic	*CCDC116*	.	.	.
22	21989696	C	A	intronic	*CCDC116*	.	.	.
1	46089078	G	A	exonic	*CCDC17*	nonsynonymous_SNV	.	.
19	46521622	A	C	exonic	*CCDC61*	nonsynonymous_SNV	.	.
20	30607263	T	TCC	intronic	*CCM2L*	.	.	.
1	111441896	A	C	UTR3	*CD53*	.	.	.
18	25593780	G	A	exonic	*CDH2*	nonsynonymous_SNV	.	.
3	49828911	GAAA	G	intronic	*CDHR4*	.	.	.
14	54878251	A	G	exonic	*CDKN3*	synonymous_SNV	.	.
1	26603023	C	T	intronic	*CEP85*	.	.	.
12	97159023	C	CA	intronic	*CFAP54*	.	.	.
10	90966256	C	T	exonic	*CH25H*	nonsynonymous_SNV	.	.
17	48541856	A	G	UTR3	*CHAD*	.	.	.
15	34355352	A	G	exonic	*CHRM5*	nonsynonymous_SNV	.	.
19	19656453	G	A	exonic	*CILP2*	synonymous_SNV	.	.
1	87041118	T	A	exonic	*CLCA4*	nonsynonymous_SNV	.	.
4	56301737	G	A	exonic	*CLOCK*	nonsynonymous_SNV	.	.
6	75843042	C	T	exonic	*COL12A1*	nonsynonymous_SNV	.	.
8	139706079	A	AGATCGG	intronic	*COL22A1*	.	.	.
7	130288676	C	T	intronic	*COPG2*	.	.	.
12	69326412	A	G	intronic	*CPM*	.	.	.
16	1716655	G	A	intronic	*CRAMP1*	.	.	.
3	9983008	A	C	intronic	*CRELD1*	.	.	.
8	75925143	T	G	exonic	*CRISPLD1*	nonsynonymous_SNV	.	.
1	36934821	C	A	exonic	*CSF3R*	synonymous_SNV	.	.

4	1219325	A	G	exonic	*CTBP1*	nonsynonymous_SNV	.	.
7	99312197	C	T	exonic	*CYP3A7,CYP3A7-CYP3A51P*	nonsynonymous_SNV	.	.
17	76695075	G	C	intronic	*CYTH1*	.	.	.
12	109283841	G	A	intronic	*DAO*	.	.	.
6	24301999	A	C	exonic	*DCDC2*	nonsynonymous_SNV	.	.
6	30856437	T	C	intronic	*DDR1*	.	.	.
1	197616172	C	G	intronic	*DENND1B*	.	.	.
14	24458243	G	A	exonic	*DHRS4L2*	synonymous_SNV	.	.
10	79576278	A	G	intronic	*DLG5*	.	.	.
16	20976081	C	T	exonic	*DNAH3*	nonsynonymous_SNV	.	.
1	172222670	CTCT	C	intronic	*DNM3*	.	.	.
19	10286133	A	G	intronic	*DNMT1*	.	.	.
5	169494668	G	A	exonic	*DOCK2*	nonsynonymous_SNV	.	.
7	154587494	C	CAAAAA	intronic	*DPP6*	.	.	.
8	33827054	T	C	intergenic	*DUSP26,LINC01288*	.	.	.
10	82098831	T	C	intronic	*DYDC1*	.	.	.
13	78474853	CTAAAACGACATTTAA	C	ncRNA_intronic	*EDNRB-AS1*	.	.	.
16	22268549	T	C	intronic	*EEF2K*	.	.	.
2	31483409	C	T	exonic	*EHD3*	nonsynonymous_SNV	.	.
1	32690066	C	A	exonic	*EIF3I*	nonsynonymous_SNV	.	.
7	73457382	G	A	intronic	*ELN*	.	.	.
17	48617488	T	C	intronic	*EPN3*	.	.	.
1	75038882	C	A	exonic	*ERICH3*	nonsynonymous_SNV	.	.
8	95677132	T	TTCC	intronic	*ESRP1*	.	.	.
11	44151610	A	G	exonic	*EXT2*	synonymous_SNV	.	.

15	93306273	ATGCCATTATTATGCAT	A	intergenic	*FAM174B,ASB9P1*	.	.	.
12	110206485	C	T	exonic	*FAM222A*	nonsynonymous_SNV	.	.
6	116784436	T	C	intronic	*FAM26F*	.	.	.
22	40417404	A	G	exonic	*FAM83F*	nonsynonymous_SNV	.	.
2	242396201	G	A	exonic	*FARP2*	nonsynonymous_SNV	.	.
11	92534379	C	T	exonic	*FAT3*	nonsynonymous_SNV	.	.
1	159272149	A	C	UTR5	*FCER1A*	.	.	.
12	32777904	C	T	intronic	*FGD4*	.	.	.
12	4554363	G	A	intronic	*FGF6*	.	.	.
2	186659101	C	G	exonic	*FSIP2*	stopgain	.	.
12	132824508	G	A	exonic	*GALNT9*	synonymous_SNV	.	.
6	33554544	G	A	ncRNA_exonic	*GGNBP1,LINC00336*	.	.	.
7	2854602	G	A	UTR5	*GNA12*	.	.	.
9	36249261	C	G	exonic	*GNE*	nonsynonymous_SNV	.	.
12	133385016	C	A	exonic	*GOLGA3*	synonymous_SNV	.	.
3	194118797	C	T	exonic	*GP5*	nonsynonymous_SNV	.	.
13	27333262	G	A	exonic	*GPR12*	nonsynonymous_SNV	.	.
2	175333752	A	C	exonic	*GPR155*	nonsynonymous_SNV	.	.
6	167570854	C	T	exonic	*GPR31*	nonsynonymous_SNV	.	.
1	174417345	C	T	exonic	*GPR52*	synonymous_SNV	.	.
16	85690108	C	T	exonic	*GSE1*	synonymous_SNV	.	.
1	154247862	T	C	intronic	*HAX1*	.	.	.
4	89311863	T	C	exonic	*HERC6*	nonsynonymous_SNV	.	.
10	71139553	G	A	intronic	*HK1*	.	.	.
3	148789320	T	C	intronic	*HLTF*	.	.	.

1	186060010	A	G	exonic	*HMCN1*	nonsynonymous_SNV	.	.
2	138727752	C	G	exonic	*HNMT*	stopgain	.	.
11	62483191	A	C	ncRNA_intronic	*HNRNPUL2-BSCL2*	.	.	.
2	176993893	G	A	upstream	*HOXD8*	.	.	.
21	44949820	A	C	exonic	*HSF2BP*	synonymous_SNV	.	.
X	114141325	C	T	exonic	*HTR2C*	nonsynonymous_SNV	.	.
4	8307707	C	A	exonic	*HTRA3*	synonymous_SNV	.	.
4	8307709	A	G	exonic	*HTRA3*	nonsynonymous_SNV	.	.
21	33312421	T	C	intronic	*HUNK*	.	.	.
2	191231402	T	C	intronic	*INPP1*	.	.	.
2	234085917	T	C	exonic	*INPP5D*	unknown	.	.
X	107978049	C	T	exonic	*IRS4*	nonsynonymous_SNV	.	.
15	42139801	CT	C	intronic	*JMJD7-PLA2G4B,PLA2G4B*	.	.	.
8	73848917	G	A	exonic	*KCNB2*	nonsynonymous_SNV	.	.
8	73848267	A	G	exonic	*KCNB2*	nonsynonymous_SNV	.	.
1	245851519	C	T	exonic	*KIF26B*	nonsynonymous_SNV	.	.
1	245850847	C	T	exonic	*KIF26B*	nonsynonymous_SNV	.	.
12	53074047	C	T	exonic	*KRT1*	nonsynonymous_SNV	.	.
17	39197485	A	G	exonic	*KRTAP1-1*	synonymous_SNV	.	.

6	30782218	C	CTGTTTG TT	ncRNA_exonic	*LINC00243*	.	.	.
10	90530748	G	A	exonic	*LIPN*	synonymous_SNV	.	.
7	123302716	G	A	exonic	*LMOD2*	nonsynonymous_SNV	.	.
1	43260262	GA	G	exonic	*LOC100129924*	frameshift_deletion	.	.
2	114647738	G	C	ncRNA_intronic	*LOC101060091*	.	.	.
10	70987200	C	T	ncRNA_intronic	*LOC101928994*	.	.	.
2	38709144	T	C	ncRNA_intronic	*LOC101929596*	.	.	.
2	38709145	C	T	ncRNA_intronic	*LOC101929596*	.	.	.
6	160913933	T	C	ncRNA_exonic	*LPAL2*	.	.	.
8	105509427	G	A	exonic	*LRP12*	synonymous_SNV	.	.
6	25466073	T	G	intronic	*LRRC16A*	.	.	.
7	150034647	C	T	exonic	*LRRC61*	synonymous_SNV	.	.
9	131669792	C	T	exonic	*LRRC8A*	stopgain	.	.
20	6031380	G	A	intronic	*LRRN4*	.	.	.
4	156296003	G	A	intronic	*MAP9*	.	.	.
15	42110756	A	T	exonic	*MAPKBP1*	nonsynonymous_SNV	.	.
3	152163048	T	G	intronic	*MBNL1*	.	.	.
10	103557874	T	C	exonic	*MGEA5*	nonsynonymous_SNV	.	.
16	58077215	G	A	exonic	*MMP15*	nonsynonymous_SNV	.	.
17	17069027	C	T	intronic	*MPRIP*	.	.	.
8	125711803	A	G	exonic	*MTSS1*	nonsynonymous_SNV	.	.
12	78583895	A	G	exonic	*NAV3*	nonsynonymous_SNV	.	.

3	47047404	C	T	intronic	*NBEAL2*	.	.	.
9	100423222	G	A	intronic	*NCBP1*	.	.	.
20	33338071	T	A	exonic	*NCOA6*	nonsynonymous_SNV	.	.
12	124873151	T	C	intronic	*NCOR2*	.	.	.
9	140108331	G	C	splicing	*NDOR1*	.	.	.
1	161183515	C	T	exonic	*NDUFS2*	nonsynonymous_SNV	.	.
18	56016727	A	G	intronic	*NEDD4L*	.	.	.
10	71332247	C	T	exonic	*NEUROG3*	nonsynonymous_SNV	.	.
9	94172596	G	A	exonic	*NFIL3*	nonsynonymous_SNV	.	.
9	33464972	G	A	exonic	*NOL6*	nonsynonymous_SNV	.	.
1	200182945	G	A	intergenic	*NR5A2,LINC00862*	.	.	.
1	86847989	T	G	exonic	*ODF2L*	nonsynonymous_SNV	.	.
19	15932940	C	T	intergenic	*OR10H1,UCA1*	.	.	.
1	248512771	G	A	exonic	*OR14C36*	nonsynonymous_SNV	.	.
17	3119201	G	A	exonic	*OR1A1*	nonsynonymous_SNV	.	.
7	143826443	A	T	exonic	*OR2A14*	nonsynonymous_SNV	.	.
11	5906305	G	C	exonic	*OR52E4*	nonsynonymous_SNV	.	.
9	77742549	A	G	intronic	*OSTF1*	.	.	.
18	77960583	A	G	intronic	*PARD6G*	.	.	.
3	183560237	T	A	splicing	*PARL*	.	.	.
5	140751665	G	A	exonic	*PCDHGB3*	synonymous_SNV	.	.

7	82595261	A	T	exonic	*PCLO*	synonymous_SNV	.	.
15	101983697	G	A	intronic	*PCSK6*	.	.	.
7	550556	C	T	exonic	*PDGFA*	nonsynonymous_SNV	.	.
X	19377039	G	A	exonic	*PDHA1*	nonsynonymous_SNV	.	.
2	197781173	T	G	exonic	*PGAP1*	nonsynonymous_SNV	.	.
8	110457482	A	G	exonic	*PKHD1L1*	nonsynonymous_SNV	.	.
8	96166422	C	A	exonic	*PLEKHF2*	synonymous_SNV	.	.
19	4511354	G	A	exonic	*PLIN4*	nonsynonymous_SNV	.	.
7	102194766	A	T	intronic	*POLR2J3,SPDYE2,SPDYE2B*	.	.	.
22	41929829	A	G	intronic	*POLR3H*	.	.	.
12	63359457	C	T	intergenic	*PPM1H,AVPR1A*	.	.	.
19	45899479	G	A	exonic	*PPP1R13L*	stopgain	.	.
11	129784784	C	T	exonic	*PRDM10*	nonsynonymous_SNV	.	.
4	121737527	C	T	intronic	*PRDM5*	.	.	.
3	64085080	G	A	exonic	*PRICKLE2*	nonsynonymous_SNV	.	.
8	48794487	C	T	exonic	*PRKDC*	unknown	.	.
16	68349904	G	A	exonic	*PRMT7*	nonsynonymous_SNV	.	.
19	36293974	G	A	exonic	*PRODH2*	synonymous_SNV	.	.
14	24613507	C	T	intronic	*PSME2*	.	.	.
8	97311953	C	T	exonic	*PTDSS1*	nonsynonymous_SNV	.	.
1	202157000	A	G	ncRNA_intronic	*PTPRVP*	.	.	.

1	31452917	G	A	exonic	*PUM1*	nonsynonymous_SNV	.	.
1	220427736	C	T	intronic	*RAB3GAP2*	.	.	.
8	117887121	G	GCCCCCC CCCCCCC	ncRNA_exonic	*RAD21-AS1*	.	.	.
17	17700485	T	C	exonic	*RAI1*	nonsynonymous_SNV	.	.
2	33784048	A	G	exonic	*RASGRP3*	nonsynonymous_SNV	.	.
13	49054183	T	C	exonic	*RB1*	synonymous_SNV	.	.
5	150078064	A	G	exonic	*RBM22*	nonsynonymous_SNV	.	.
6	117199135	T	C	intronic	*RFX6*	.	.	.
1	1007222	G	C	exonic	*RNF223*	nonsynonymous_SNV	.	.
5	175956183	A	G	intronic	*RNF44*	.	.	.
11	498952	C	T	intronic	*RNH1*	.	.	.
1	114340555	T	C	exonic	*RSBN1*	synonymous_SNV	.	.
18	56819914	A	C	exonic	*SEC11C*	nonsynonymous_SNV	.	.
3	10354431	A	G	intronic	*SEC13*	.	.	.
1	31883227	T	C	UTR5	*SERINC2*	.	.	.
8	19231205	C	T	intronic	*SH2D4A*	.	.	.
11	70333486	G	A	exonic	*SHANK2*	nonsynonymous_SNV	.	.
11	64323484	A	G	exonic	*SLC22A11*	nonsynonymous_SNV	.	.
22	41172317	A	G	intronic	*SLC25A17*	.	.	.
4	42020534	C	T	intronic	*SLC30A9*	.	.	.
2	196545043	G	A	exonic	*SLC39A10*	nonsynonymous_SNV	.	.
17	33768221	C	A	exonic	*SLFN13*	nonsynonymous_SNV	.	.
1	31766006	A	T	intronic	*SNRNP40*	.	.	.
10	106924028	C	T	intronic	*SORCS3*	.	.	.

1	118533416	A	G	intronic	*SPAG17*	.	.	.
8	48626053	C	T	exonic	*SPIDR*	nonsynonymous_SNV	.	.
19	38778478	A	G	intronic	*SPINT2*	.	.	.
12	56863265	A	G	exonic	*SPRYD4*	synonymous_SNV	.	.
17	17719523	T	C	exonic	*SREBF1*	nonsynonymous_SNV	.	.
20	2124511	C	T	UTR3	*STK35*	.	.	.
12	27468192	G	A	intronic	*STK38L*	.	.	.
1	24684656	C	T	UTR3	*STPG1*	.	.	.
1	110581391	T	C	intronic	*STRIP1*	.	.	.
7	142919510	C	T	exonic	*TAS2R40*	synonymous_SNV	.	.
16	71604270	G	A	exonic	*TAT*	nonsynonymous_SNV	.	.
17	28890404	A	G	exonic	*TBC1D29*	synonymous_SNV	.	.
16	30100270	G	A	exonic	*TBX6*	synonymous_SNV	.	.
3	169482868	C	T	upstream	*TERC*	.	.	.
7	115624476	A	G	exonic	*TFEC*	nonsynonymous_SNV	.	.
19	2810650	T	G	exonic	*THOP1*	nonsynonymous_SNV	.	.
X	48752253	A	G	intronic	*TIMM17B*	.	.	.
9	75387472	G	A	splicing	*TMC1*	.	.	.
17	7340322	T	C	exonic	*TMEM102*	nonsynonymous_SNV	.	.
14	106067952	C	T	intergenic	*TMEM121,MIR8071-2*	.	.	.
14	21967110	C	T	UTR3	*TOX4*	.	.	.
1	211545916	A	C	exonic	*TRAF5*	nonsynonymous_SNV	.	.
12	112578723	C	T	exonic	*TRAFD1*	nonsynonymous_SNV	.	.
2	3392368	C	A	exonic	*TRAPPC12*	nonsynonymous_SNV	.	.
16	88926368	G	A	exonic	*TRAPPC2L*	nonsynonymous_SNV	.	.
17	73517973	G	A	exonic	*TSEN54*	nonsynonymous_SNV	.	.
2	178416956	G	A	exonic	*TTC30B*	nonsynonymous_SNV	.	.
2	179635146	G	A	exonic	*TTN*	synonymous_SNV	.	.

12	49579774	C	CAGCTTG CGAATTC GGTCCAA CACGAGG TCAATGAT CTCCTTG CCAA	splicing	*TUBA1A*	.	.	.
2	220116819	T	C	exonic	*TUBA4A*	nonsynonymous_SNV	.	.
22	50664585	A	T	exonic	*TUBGCP6*	nonsynonymous_SNV	.	.
15	41861222	T	C	splicing	*TYRO3*	.	.	.
15	41861223	G	C	intronic	*TYRO3*	.	.	.
9	35381100	A	G	exonic	*UNC13B*	nonsynonymous_SNV	.	.
14	94149067	A	G	intronic	*UNC79*	.	.	.
2	210860212	A	G	exonic	*UNC80*	nonsynonymous_SNV	.	.
2	61433096	C	A	intronic	*USP34*	.	.	.
9	136699397	G	A	exonic	*VAV2*	nonsynonymous_SNV	.	.
9	79985250	T	A	splicing	*VPS13A*	.	.	.
15	42459746	CAAA	C	intronic	*VPS39*	.	.	.
12	6105191	C	T	exonic	*VWF*	nonsynonymous_SNV	.	.
1	27739272	T	C	intronic	*WASF2*	.	.	.
10	49983781	C	G	exonic	*WDFY4*	synonymous_SNV	.	.
15	53815478	C	T	exonic	*WDR72*	nonsynonymous_SNV	.	.
8	145168298	T	G	intronic	*WDR97*	.	.	.
21	43412922	C	T	exonic	*ZBTB21*	nonsynonymous_SNV	.	.
14	105267779	T	G	exonic	*ZBTB42*	nonsynonymous_SNV	.	.
22	41753378	C	T	exonic	*ZC3H7B*	nonsynonymous_SNV	.	.
16	87445813	A	G	exonic	*ZCCHC14*	synonymous_SNV	.	.
8	135614834	C	T	exonic	*ZFAT*	synonymous_SNV	.	.
7	6730745	A	G	exonic	*ZNF12*	nonsynonymous_SNV	.	.
11	9549119	T	C	exonic	*ZNF143*	nonsynonymous_SNV	.	.

19	57868540	A	G	exonic	*ZNF304*	nonsynonymous_SNV	.	.
4	10447312	T	G	exonic	*ZNF518B*	nonsynonymous_SNV	.	.
19	58757820	G	T	intronic	*ZNF544*	.	.	.
16	30583455	G	A	exonic	*ZNF688*	nonsynonymous_SNV	.	.
19	22942382	T	C	exonic	*ZNF99*	nonsynonymous_SNV	.	.
19	56702320	T	C	exonic	*ZSCAN5B*	nonsynonymous_SNV	.	.

* (1 = Variant Present, 0 = Variant not Present)

LJB23_SIFT_pred	LJB23_Polyphen2_HDIV_pred	LJB23_FATHMM_pred	LJB23_LR_pred	cosmic76	Total number of samples with variant	UM-10*	UM-11*
.	.	.	.	.	11	1	1
.	.	.	.	.	11	1	1
.	.	.	.	.	11	1	1
T	B	T	T	.	11	1	1
.	.	.	.	.	11	1	1
.	.	.	.	.	11	1	1
.	.	.	.	.	11	1	1
SIFTindel: Causes nonsense mediated decay,	.	.	.	.	11	1	1
D	D	D	D	ID\x3dCOSM52969\x3bOCCURENCE\x3d6(meninges),130(eye),13(skin)	11	1	1
.	.	.	.	.	11	1	1
.	.	.	.	.	11	1	1
T	B	T	T	.	11	1	1
D	.	.	.	.	11	1	1
SIFTindel: Causes nonsense mediated decay,	.	.	.	.	11	1	1
D	D	D	D	.	11	1	1
.	.	.	.	ID\x3dCOSM4395221\x3bOCCURENCE\x3d1(skin)	11	1	1
.	.	.	.	.	11	1	1
SIFTindel: Neutral	.	.	.	.	11	1	1
.	.	.	.	.	11	1	1

.	.	.	.	.	11	1	1
SIFTindel: Causes nonsense mediated decay,	.	.	.	.	11	1	1
.	.	.	.	.	10	1	1
T	B	D	T	ID\x3dCOSM258949,COSM5070869,COSM78178\x3bOCCURENCE\x3d1(breast),1(large_intestine),1(ovary)	10	1	1
.	.	.	.	.	10	1	1
D	D	T	T	.	8	1	0
T	P	T	T	.	7	1	0
.	.	.	.	.	6	0	1
.	.	.	.	.	6	0	1
T	B	T	T	.	6	1	0
.	.	.	.	.	6	0	1
.	.	.	.	.	6	0	1
.	.	.	.	.	6	1	0
.	.	.	.	.	6	1	0
.	.	.	.	.	4	0	0
.	.	.	.	.	3	0	1
.	.	.	.	.	2	0	0
.	.	.	.	.	2	1	1
.	.	.	T	.	2	0	0
.	.	.	.	.	2	0	0
.	.	.	.	.	2	0	0
.	.	.	.	.	2	1	0
.	.	.	.	.	2	0	1

.	.	.	.	.	1	0	0
.	.	.	.	.	1	0	0
T	B	T	T	.	1	0	0
D	D	T	T	.	1	0	0
T	D	T	T	.	1	0	0
D	D	T	T	.	1	0	0

T	B	T	T	.	1	0	0
D	D	T	T	.	1	0	0
.	.	.	.	ID\x3dCOSM3867175,COSM3867174\x3bOCCURENCE\x3d1(skin)	1	0	0
T	B	D	T	.	1	0	0
.	.	.	.	.	1	0	0
.	.	.	.	.	1	0	1
.	.	.	.	.	1	0	1
.	.	.	.	.	1	0	0
.	.	.	.	.	1	0	0
D	D	T	T	ID\x3dCOSM4664757,COSM4664756\x3bOCCURENCE\x3d1(large_intestine)	1	0	0
.	.	.	.	.	1	1	0
D	D	T	D	ID\x3dCOSM3506072\x3bOCCURENCE\x3d1(skin)	1	0	1
.	.	.	.	.	1	0	0
T	D	T	T	.	1	0	0
.	.	.	.	.	1	0	0
.	.	.	.	.	1	0	0
.	.	.	.	.	1	0	0
D	D	.	T	.	1	0	0
.	.	.	.	.	1	0	0
T	B	T	T	.	1	0	0
.	.	.	.	.	1	0	0
T	B	T	T	.	1	0	0

D	D	T	T	ID\x3dCOSM3714668,COSM3714669\x3bOCCURENCE\x3d1(ovary),2(upper_aerodigestive_tract)	1	0	0
D	.	.	T	.	1	0	0
.	.	.	.	.	1	0	0
.	.	.	.	.	1	0	0
D	B	T	T	.	1	0	0
T	D	.	T	.	1	1	0
.	.	.	.	.	1	1	0
.	.	.	.	.	1	0	0
T	D	T	T	.	1	0	0
.	.	.	.	.	1	0	0
.	.	.	.	.	1	0	0
.	.	.	.	.	1	0	0
.	.	.	.	.	1	1	0
T	P	T	T	ID\x3dCOSM4016490\x3bOCCURENCE\x3d1(stomach)	1	0	0
.	.	.	.	.	1	0	0
T	B	T	T	.	1	0	0
.	.	.	.	.	1	0	0
D	D	T	T	.	1	0	0
T	B	T	T	.	1	0	0
T	P	T	T	.	1	0	0
.	.	.	.	.	1	1	0
.	.	.	.	.	1	1	0
.	.	.	.	.	1	1	0
.	.	.	.	.	1	0	0
.	.	.	.	.	1	0	0
T	B	T	T	.	1	0	0
.	.	.	.	.	1	0	0

T	P	D	D	.	1	0	0
D	P	T	T	ID\x3dCOSM4587667\x3bOCCURENCE\x3d1(bone)	1	0	0
.	.	.	.	.	1	0	0
.	.	.	.	.	1	1	0
T	P	D	T	.	1	1	0
.	.	.	.	.	1	0	0
.	.	.	.	.	1	0	0
.	.	.	.	.	1	0	0
.	.	.	.	.	1	0	0
T	B	T	T	.	1	0	0
.	.	.	.	.	1	0	0
.	.	.	.	.	1	0	0
D	D	T	T	ID\x3dCOSM3661707\x3bOCCURENCE\x3d2(liver)	1	0	0
.	.	.	.	.	1	0	0
.	.	.	.	.	1	0	1
.	.	.	.	.	1	0	0
.	.	.	.	.	1	0	0
.	.	.	.	.	1	0	0
D	D	D	D	.	1	0	0
T	P	T	T	.	1	0	1
.	.	.	.	.	1	0	0
.	.	.	.	.	1	0	0
D	D	T	T	.	1	0	0
.	.	.	.	.	1	0	0
.	.	.	.	.	1	0	0

.	.	.	.	.	1	0	0
T	P	T	T	.	1	0	0
.	.	.	.	.	1	0	0
T	D	T	T	.	1	0	0
T	B	T	T	ID\x3dCOSM442641\x3bOCCURENCE\x3d1(breast)	1	0	0
T	B	T	T	.	1	1	0
.	.	.	.	.	1	0	0
.	.	.	.	.	1	0	0
.	.	.	.	.	1	0	0
D	.	.	.	.	1	0	0
.	.	.	T	.	1	0	0
.	.	.	.	.	1	0	0
.	.	.	.	.	1	0	0
T	D	D	D	.	1	0	0
.	.	.	.	.	1	0	0
T	B	D	D	ID\x3dCOSM3783693,COSM3783694\x3bOCCURENCE\x3d1(prostate)	1	0	0
D	D	T	T	.	1	0	0
D	D	T	T	.	1	0	0
T	B	T	T	.	1	0	0
.	.	.	.	.	1	0	1
.	.	.	T	.	1	1	0
.	.	.	.	.	1	0	1
T	B	D	T	.	1	0	0
.	.	.	.	.	1	0	0
.	.	.	.	.	1	0	0

T	B	T	T	.	1	0	0
T	.	.	.	.	1	0	0
.	.	.	.	.	1	1	0
D	.	.	T	.	1	0	0
.	.	.	.	.	1	1	0
D	B	T	T	.	1	0	0
.	.	.	.	.	1	0	0
T	B	T	T	ID\x3dCOSM1310309\x3bOCCURENCE\x3d2(central_nervous_system),2(skin),1(urinary_tract),1(prostate)	1	0	0
.	.	.	.	.	1	0	0
.	.	.	.	.	1	0	0
D	D	T	D	.	1	0	0
T	D	T	T	ID\x3dCOSM4959360\x3bOCCURENCE\x3d2(liver)	1	0	1
.	.	.	.	.	1	0	0
D	D	D	D	.	1	0	0
T	P	D	D	.	1	0	0
D	P	T	T	.	1	0	0
T	B	T	T	.	1	0	0
D	D	D	T	.	1	0	0
.	.	.	.	ID\x3dCOSM3717344\x3bOCCURENCE\x3d6(liver),1(upper_aerodigestive_tract)	1	0	0

.	.	.	.	.	1	0	0
.	.	.	.	.	1	0	0
D	D	D	D	ID\x3dCOSM240501\x3bOCCURENCE\x3d1(endometrium),1(stomach),1(urinary_tract),1(prostate)	1	0	0
.	.	.	.	.	1	0	0
.	.	.	.	.	1	0	0
.	.	.	.	.	1	0	0
.	.	.	.	.	1	0	0
.	.	.	.	.	1	0	0
.	.	.	.	.	1	0	0
.	.	.	.	.	1	0	0
.	.	.	.	.	1	0	0
.	.	.	.	.	1	0	0
T	.	.	.	ID\x3dCOSM5345713\x3bOCCURENCE\x3d1(large_intestine)	1	0	0
.	.	.	.	.	1	0	0
.	.	.	.	.	1	0	0
D	D	T	T	.	1	0	0
.	.	.	.	.	1	0	0
T	P	T	T	.	1	0	0
D	D	T	T	.	1	0	0
.	.	.	T	.	1	0	0
T	D	T	T	.	1	0	0
.	.	.	D	ID\x3dCOSM1992733,COSM1992734\x3bOCCURENCE\x3d1(large_intestine)	1	0	0

.	.	.	.	.	1	0	1
.	.	.	.	.	1	0	0
D	P	T	T	.	1	0	0
.	.	.	.	.	1	0	0
.	.	.	.	.	1	0	0
T	P	D	D	.	1	0	0
.	.	.	.	.	1	0	0
T	B	D	T	.	1	0	0
T	D	.	T	.	1	0	1
D	D	T	T	ID\x3dCOSM4397722,COSM4397721\x3bOCCURENCE\x3d1(skin)	1	0	0
.	.	.	.	.	1	1	0
T	P	T	T	.	1	0	0
.	.	.	.	ID\x3dCOSM992353\x3bOCCURENCE\x3d1(endometrium)	1	0	0
D	B	T	T	.	1	0	0
T	B	T	T	ID\x3dCOSM3516002\x3bOCCURENCE\x3d1(skin)	1	0	0
T	B	T	T	.	1	0	0
T	B	T	T	.	1	0	0
.	.	.	.	.	1	0	0
.	.	.	.	.	1	0	0
.	.	.	.	.	1	0	0
.	.	.	.	ID\x3dCOSM3994055,COSM3994056\x3bOCCURENCE\x3d1(kidney)	1	0	0

.	.	.	.	.		1	0	0
.	.	.	.	.		1	0	1
T	D	T	T	ID\x3dCOSM5170205,COSM5170204\x3bOCCURENCE\x3d1(large_intestine)		1	1	0
D	D	D	D	.		1	0	0
T	D	T	T	.		1	1	0
T	B	T	T	.		1	0	0
.	.	.	.	.		1	0	0
T	B	T	T	ID\x3dCOSM1725391,COSM1725390\x3bOCCURENCE\x3d1(liver)		1	0	1
.	.	.	.	.		1	0	0
.	.	.	.	.		1	0	0
.	.	.	.	ID\x3dCOSM3955049\x3bOCCURENCE\x3d1(lung)		1	0	0
T	.	.	.	.		1	0	0
D	D	T	T	.		1	0	0
.	.	.	.	.		1	0	1
T	P	D	T	.		1	0	0
T	P	T	T	.		1	0	0
T	B	T	T	ID\x3dCOSM704398\x3bOCCURENCE\x3d1(lung)		1	1	0
.	.	.	.	.		1	0	1
.	.	.	.	.		1	0	0
T	B	T	T	.		1	0	0
.	.	.	.	.		1	0	0

D	D	T	T	ID\x3dCOSM4031340\x3bOCCURENCE\x3d1(stomach)	1	0	0
.	.	.	.	.	1	0	0
.	.	.	.	.	1	1	0
T	D	T	T	.	1	0	0
T	B	T	T	.	1	0	0
.	.	.	.	ID\x3dCOSM1942461,COSM1942462\x3bOCCURENCE\x3d1(large_intestine)	1	0	0
D	D	T	T	.	1	0	0
.	.	.	.	.	1	0	0
D	.	D	T	.	1	1	0
.	.	.	T	.	1	0	0
.	.	.	.	.	1	0	1
.	.	.	.	.	1	0	0
T	P	.	T	ID\x3dCOSM5415370\x3bOCCURENCE\x3d1(prostate)	1	0	0
.	.	.	.	.	1	0	0
.	.	.	.	.	1	0	0
.	.	.	.	.	1	0	0
T	B	T	T	.	1	0	0
T	B	T	T	.	1	0	0
.	.	.	.	.	1	0	0
.	.	.	.	.	1	0	0
T	D	T	T	.	1	0	0
D	D	D	D	.	1	0	0
.	.	.	.	.	1	0	0
.	.	.	.	.	1	0	0

.	.	.	.	.	1	0	0
D	D	.	T	.	1	0	0
.	.	.	.	.	1	0	0
.	.	.	.	.	1	0	0
.	.	.	T	.	1	0	0
T	B	T	T	.	1	1	0
.	.	.	.	.	1	0	0
.	.	.	.	.	1	0	0
.	.	.	.	.	1	0	0
.	.	.	.	.	1	0	0
D	D	D	D	.	1	0	1
.	.	.	.	.	1	0	0
.	.	.	.	.	1	0	0
.	.	.	.	.	1	0	0
D	P	T	T	.	1	0	0
D	D	T	T	.	1	0	1
.	.	.	.	.	1	0	0
.	.	.	.	.	1	0	0
T	B	T	T	.	1	0	0
.	.	.	.	.	1	0	0
.	.	.	.	.	1	0	0
D	D	T	T	.	1	0	0
D	D	T	T	ID\x3dCOSM4038813\x3bOCCURENCE\x3d1(stomach)	1	0	0
D	P	T	T	.	1	0	0
T	P	D	D	ID\x3dCOSM974796\x3bOCCURENCE\x3d3(endometrium)	1	0	0
T	B	T	T	.	1	0	0
T	B	T	T	.	1	0	0
.	.	.	.	.	1	0	1

.	.	.	.	.	1	0	0
D	B	T	T	.	1	0	0
T	D	T	T	.	1	0	0
.	.	.	.	.	1	0	0
.	.	.	.	.	1	0	0
D	D	T	D	.	1	0	0
.	.	.	.	.	1	0	0
D	B	T	T	.	1	0	1
.	.	.	.	.	1	0	0
T	B	T	T	.	1	0	0
.	.	.	.	.	1	0	0
.	.	.	.	.	1	0	0
T	B	T	T	.	1	0	0
.	.	.	.	.	1	0	0
.	.	.	T	.	1	0	1
D	D	T	T	.	1	0	0
.	.	.	.	.	1	1	0
T	B	T	T	.	1	0	0
D	D	T	D	.	1	0	1
T	B	T	T	.	1	1	0
.	.	.	.	.	1	0	0
.	.	.	.	ID\x3dCOSM3318686,COSM3318687\x3bOCCURENCE\x3d1(stomach),1(large_intestine)	1	0	0
D	D	D	D	.	1	0	0
T	D	T	T	.	1	0	0

T	B	T	T	.	1	0	0
D	D	T	T	.	1	0	0
.	.	.	.	.	1	0	0
D	B	T	T	.	1	0	1
T	P	T	T	.	1	0	0
T	B	T	T	.	1	0	0

UM-16*	UM-21*	UM-23*	UM-25*	UM-29*	UM-32*	UM-35*	UM-38*	UM-6*
1	1	1	1	1	1	1	1	1
1	1	1	1	1	1	1	1	1
1	1	1	1	1	1	1	1	1
1	1	1	1	1	1	1	1	1
1	1	1	1	1	1	1	1	1
1	1	1	1	1	1	1	1	1
1	1	1	1	1	1	1	1	1
1	1	1	1	1	1	1	1	1
1	1	1	1	1	1	1	1	1
1	1	1	1	1	1	1	1	1
1	1	1	1	1	1	1	1	1
1	1	1	1	1	1	1	1	1
1	1	1	1	1	1	1	1	1
1	1	1	1	1	1	1	1	1
1	1	1	1	1	1	1	1	1
1	1	1	1	1	1	1	1	1
1	1	1	1	1	1	1	1	1
1	1	1	1	1	1	1	1	1
1	1	1	1	1	1	1	1	1

1	1	1	1	1	1	1	1	1
1	1	1	1	1	1	1	1	1
1	1	1	1	1	1	0	1	1
1	0	1	1	1	1	1	1	1
1	1	1	1	0	1	1	1	1
1	1	1	1	0	0	1	1	1
1	1	1	0	0	0	1	1	1
0	0	1	1	0	0	1	1	1
1	0	1	1	1	1	0	0	0
0	1	1	0	0	0	1	1	1
1	0	1	1	1	1	0	0	0
1	0	1	1	1	1	0	0	0
0	1	1	0	0	0	1	1	1
1	0	1	0	0	0	1	1	1
1	0	1	0	1	1	0	0	0
0	0	0	0	1	0	1	0	0
1	0	0	0	1	0	0	0	0
0	0	0	0	0	0	0	0	0
0	0	0	0	0	0	1	0	1
1	0	1	0	0	0	0	0	0
0	0	0	1	0	0	0	0	1
0	0	1	0	0	0	0	0	0
1	0	0	0	0	0	0	0	0

0	0	0	0	0	0	1	0	0
0	0	0	0	0	0	1	0	0
0	1	0	0	0	0	0	0	0
0	1	0	0	0	0	0	0	0
0	1	0	0	0	0	0	0	0
0	0	0	0	0	0	1	0	0

0	0	0	0	0	0	1	0	0
0	0	0	0	1	0	0	0	0
0	0	0	1	0	0	0	0	0
1	0	0	0	0	0	0	0	0
0	0	0	0	1	0	0	0	0
0	0	0	0	0	0	0	0	0
0	0	0	0	0	0	0	0	0
0	0	0	1	0	0	0	0	0
0	0	0	0	0	0	1	0	0
0	0	1	0	0	0	0	0	0
0	0	0	0	0	0	0	0	0
0	0	0	0	0	0	0	0	0
0	0	0	1	0	0	0	0	0
0	0	0	1	0	0	0	0	0
0	0	1	0	0	0	0	0	0
0	0	0	0	0	0	1	0	0
0	0	0	0	0	0	1	0	0
0	0	0	1	0	0	0	0	0
0	0	0	0	0	0	1	0	0
0	0	0	0	0	0	1	0	0
0	0	1	0	0	0	0	0	0
0	0	0	0	0	1	0	0	0

0	0	0	0	0	0	1	0	0
0	0	0	1	0	0	0	0	0
0	0	1	0	0	0	0	0	0
0	0	0	0	1	0	0	0	0
0	0	0	0	0	0	0	0	1
0	0	0	0	0	0	0	0	0
0	0	0	0	0	0	0	0	0
0	0	0	0	0	1	0	0	0
0	0	0	0	1	0	0	0	0
0	0	0	1	0	0	0	0	0
0	1	0	0	0	0	0	0	0
0	0	1	0	0	0	0	0	0
0	0	0	0	0	0	0	0	0
0	0	0	1	0	0	0	0	0
0	0	0	0	0	0	1	0	0
0	1	0	0	0	0	0	0	0
1	0	0	0	0	0	0	0	0
0	0	0	0	1	0	0	0	0
0	0	1	0	0	0	0	0	0
0	0	1	0	0	0	0	0	0
0	0	0	0	0	0	0	0	0
0	0	0	0	0	0	0	0	0
0	0	0	0	0	0	0	0	0
0	0	0	1	0	0	0	0	0
0	0	0	0	0	0	1	0	0
0	1	0	0	0	0	0	0	0
0	1	0	0	0	0	0	0	0

0	1	0	0	0	0	0	0	0
0	1	0	0	0	0	0	0	0
0	0	0	0	0	0	0	1	0
0	0	0	0	0	0	0	0	0
0	0	0	0	0	0	0	0	0
0	0	0	0	0	0	1	0	0
0	0	0	1	0	0	0	0	0
1	0	0	0	0	0	0	0	0
0	1	0	0	0	0	0	0	0
0	0	0	0	0	1	0	0	0
0	1	0	0	0	0	0	0	0
0	1	0	0	0	0	0	0	0
1	0	0	0	0	0	0	0	0
0	0	0	0	0	0	0	1	0
0	0	0	0	0	0	0	0	0
0	0	0	0	0	0	1	0	0
0	0	0	0	0	0	0	0	1
0	0	0	0	0	0	1	0	0
0	0	1	0	0	0	0	0	0
0	0	0	0	0	0	0	0	0
0	1	0	0	0	0	0	0	0
0	1	0	0	0	0	0	0	0
0	0	1	0	0	0	0	0	0
0	0	1	0	0	0	0	0	0
0	1	0	0	0	0	0	0	0

0	0	0	0	0	0	0	0	1
0	0	0	1	0	0	0	0	0
0	0	1	0	0	0	0	0	0
0	1	0	0	0	0	0	0	0
0	0	1	0	0	0	0	0	0
0	0	0	0	0	0	0	0	0
0	0	0	0	0	0	1	0	0
1	0	0	0	0	0	0	0	0
0	0	0	1	0	0	0	0	0
0	0	0	0	1	0	0	0	0
0	1	0	0	0	0	0	0	0
0	0	0	0	1	0	0	0	0
1	0	0	0	0	0	0	0	0
0	0	0	1	0	0	0	0	0
0	0	0	0	0	0	1	0	0
0	0	0	0	0	0	1	0	0
0	0	0	0	0	1	0	0	0
0	0	0	0	0	1	0	0	0
0	0	0	0	0	0	1	0	0
0	0	0	0	0	0	0	0	0
0	0	0	0	0	0	0	0	0
0	0	0	0	0	0	0	0	0
0	1	0	0	0	0	0	0	0
1	0	0	0	0	0	0	0	0
0	1	0	0	0	0	0	0	0

0	1	0	0	0	0	0	0	0
0	0	1	0	0	0	0	0	0
0	0	0	0	0	0	0	0	0
0	1	0	0	0	0	0	0	0
0	0	0	0	0	0	0	0	0
0	1	0	0	0	0	0	0	0
0	1	0	0	0	0	0	0	0
0	1	0	0	0	0	0	0	0
0	0	0	0	0	0	1	0	0
0	0	0	0	0	0	1	0	0
0	0	0	1	0	0	0	0	0
0	0	0	0	0	0	0	0	0
0	0	0	0	0	0	1	0	0
0	0	0	0	1	0	0	0	0
0	0	0	0	0	0	1	0	0
0	0	1	0	0	0	0	0	0
0	0	0	0	0	0	0	1	0
0	0	0	0	1	0	0	0	0
0	0	0	1	0	0	0	0	0

0	1	0	0	0	0	0	0	0
0	0	0	0	0	1	0	0	0
0	0	0	1	0	0	0	0	0
1	0	0	0	0	0	0	0	0
1	0	0	0	0	0	0	0	0
0	0	0	0	1	0	0	0	0
0	0	0	0	0	0	1	0	0
0	0	0	0	0	0	1	0	0
0	0	0	1	0	0	0	0	0
0	0	0	0	1	0	0	0	0
1	0	0	0	0	0	0	0	0
0	0	0	0	0	0	0	1	0
0	1	0	0	0	0	0	0	0
0	0	0	0	0	0	0	1	0
0	0	1	0	0	0	0	0	0
1	0	0	0	0	0	0	0	0
0	0	0	0	0	0	0	1	0
0	0	0	0	0	0	1	0	0
0	0	0	0	0	1	0	0	0
0	1	0	0	0	0	0	0	0
0	1	0	0	0	0	0	0	0
0	0	1	0	0	0	0	0	0

0	0	0	0	0	0	0	0	0
0	0	0	0	0	1	0	0	0
0	0	1	0	0	0	0	0	0
0	0	0	0	0	0	1	0	0
1	0	0	0	0	0	0	0	0
0	0	0	0	1	0	0	0	0
0	0	0	0	0	0	1	0	0
0	1	0	0	0	0	0	0	0
0	0	0	0	0	0	0	0	0
0	0	0	0	0	0	1	0	0
0	0	0	0	0	0	0	0	0
0	0	1	0	0	0	0	0	0
0	0	0	0	1	0	0	0	0
0	0	0	0	1	0	0	0	0
0	0	0	0	0	0	0	0	1
0	0	1	0	0	0	0	0	0
0	1	0	0	0	0	0	0	0
0	0	0	0	0	1	0	0	0
0	0	1	0	0	0	0	0	0
1	0	0	0	0	0	0	0	0
0	0	0	0	0	1	0	0	0

0	0	0	0	0	0	0	1	0
0	0	0	0	0	0	0	0	0
0	0	0	0	0	0	0	0	0
0	0	0	0	0	0	1	0	0
0	0	0	0	0	0	0	0	0
0	0	0	0	0	0	1	0	0
0	0	0	0	0	1	0	0	0
0	0	0	0	0	0	0	0	0
0	0	0	0	1	0	0	0	0
0	0	0	0	0	0	1	0	0
0	0	0	0	0	0	1	0	0
0	0	1	0	0	0	0	0	0
0	0	0	0	1	0	0	0	0
0	0	0	0	0	0	0	0	0
0	0	0	0	0	1	0	0	0
0	0	0	0	0	0	1	0	0
0	0	0	0	0	0	0	0	0
0	0	0	0	0	0	0	0	0
0	0	0	0	0	0	0	0	1
0	1	0	0	0	0	0	0	0
0	1	0	0	0	0	0	0	0

0	0	0	0	0	0	0	1	0
0	0	0	0	0	0	1	0	0
0	0	0	0	0	0	0	0	0
0	1	0	0	0	0	0	0	0
0	0	0	0	0	1	0	0	0
0	1	0	0	0	0	0	0	0
0	0	0	0	0	0	1	0	0
0	0	0	0	0	1	0	0	0
0	0	0	0	0	0	0	0	0
1	0	0	0	0	0	0	0	0
0	0	0	0	0	0	0	0	0
0	1	0	0	0	0	0	0	0
0	0	0	0	0	0	1	0	0
0	0	0	0	1	0	0	0	0
0	0	0	0	0	0	1	0	0
0	0	1	0	0	0	0	0	0
1	0	0	0	0	0	0	0	0
0	0	0	0	0	1	0	0	0
0	0	0	0	0	0	1	0	0
0	0	0	0	0	0	0	0	1
0	0	0	0	1	0	0	0	0
0	0	1	0	0	0	0	0	0
0	1	0	0	0	0	0	0	0
0	1	0	0	0	0	0	0	0

0	1	0	0	0	0	0	0	0
0	0	0	0	0	1	0	0	0
0	0	0	0	0	0	0	1	0
0	1	0	0	0	0	0	0	0
0	0	1	0	0	0	0	0	0
0	0	0	0	0	0	0	0	0
0	1	0	0	0	0	0	0	0
0	0	0	0	1	0	0	0	0
0	0	0	0	0	0	1	0	0
1	0	0	0	0	0	0	0	0
0	0	0	0	0	0	0	0	0
0	1	0	0	0	0	0	0	0
0	0	0	1	0	0	0	0	0
1	0	0	0	0	0	0	0	0
0	1	0	0	0	0	0	0	0
0	0	0	0	0	0	0	0	0
0	0	1	0	0	0	0	0	0
0	0	0	0	0	0	1	0	0
0	0	0	0	0	0	1	0	0
0	0	0	0	0	1	0	0	0
0	0	0	0	0	0	1	0	0
0	0	0	0	0	1	0	0	0
0	0	0	0	1	0	0	0	0
0	1	0	0	0	0	0	0	0
0	0	0	1	0	0	0	0	0
0	0	0	0	0	0	0	0	1
0	0	0	0	0	0	1	0	0
0	0	0	0	0	0	0	0	0

0	0	0	0	0	0	1	0	0
0	0	0	0	0	0	1	0	0
0	1	0	0	0	0	0	0	0
0	0	0	1	0	0	0	0	0
0	0	0	1	0	0	0	0	0
0	0	0	0	0	0	1	0	0
0	0	0	0	0	0	1	0	0
0	0	0	0	0	0	0	0	0
0	0	0	0	0	0	1	0	0
0	0	1	0	0	0	0	0	0
0	0	0	0	1	0	0	0	0
0	0	0	0	0	1	0	0	0
0	0	0	0	1	0	0	0	0
0	0	0	0	0	1	0	0	0
0	0	0	0	0	0	0	0	0
0	0	1	0	0	0	0	0	0
0	0	0	0	0	0	0	0	0
0	0	0	0	0	0	0	0	1
0	0	0	0	0	0	0	0	0
0	0	0	0	0	0	0	0	0
0	0	0	0	0	0	1	0	0
0	0	0	0	0	1	0	0	0
0	0	0	0	0	0	1	0	0
0	0	1	0	0	0	0	0	0

0	0	0	0	0	1	0	0	0
0	1	0	0	0	0	0	0	0
1	0	0	0	0	0	0	0	0
0	0	0	0	0	0	0	0	0
0	0	0	0	0	1	0	0	0
0	1	0	0	0	0	0	0	0

Supplementary Table 6. Patient 1 variant cancer cell fractions

	UM-06	UM-10	UM-11	UM-16	UM-21	UM-23	UM-25	UM-29	UM-32	UM-35	UM-38
BAP1	0.99	1.00	0.71	0.70	0.97	1.00	0.68	0.67	0.76	1.00	1.00
FNIP1	1.00	1.00	1.00	1.00	1.00	1.00	1.00	1.00	1.00	1.00	1.00
GNA11	1.00	1.00	1.00	1.00	1.00	1.00	1.00	1.00	1.00	1.00	1.00
PPP1R15A	0.90	1.00	0.71	1.00	0.89	1.00	0.68	1.00	1.00	1.00	1.00
RUNX3	1.00	1.00	1.00	0.98	1.00	0.99	0.99	1.00	1.00	0.97	1.00
WDR19	1.00	1.00	1.00	1.00	1.00	0.94	1.00	0.89	1.00	1.00	1.00
ATM	1.00	1.00	0.02	0.12	1.00	0.41	0.44	0.00	0.00	1.00	1.00
WWOX	1.00	1.00	0.00	0.10	0.85	0.30	0.00	0.00	0.00	0.78	1.00
CTB-178M22.1	1.00	1.00	0.00	0.05	1.00	0.23	0.00	0.00	0.00	1.00	1.00
CDH26	1.00	1.00	0.00	0.04	1.00	0.25	0.00	0.00	0.00	1.00	0.86
DBH	0.00	0.00	0.55	0.94	0.00	0.77	1.00	1.00	1.00	0.00	0.00
CD6	0.00	0.00	0.83	0.97	0.00	0.97	0.95	1.00	1.00	0.00	0.00
PGAP2	0.00	0.00	1.00	1.00	0.00	0.76	1.00	0.93	1.00	0.00	0.00

Periocular tumor
Branch #1
Branch #2
Polyclonal seeding

Supplementary Table 7. Patient 2 variant locations

Variant Type	9236667302	9456994402	9470579502	9572924002	9640093202	9721443102	9766161502	9882938502	9963233302
intronic	822	546	1258	533	792	785	566	1451	593
intergenic	1742	1243	2585	1114	1688	1620	1160	2962	1316
ncRNA_intronic	183	116	237	126	175	183	114	302	130
exonic	35	22	44	13	30	25	19	49	18
downstream	15	8	14	9	13	8	11	22	8
ncRNA_exonic	19	11	14	10	14	12	9	14	15
upstream	17	11	12	10	15	11	15	11	14
UTR3	23	11	22	9	21	17	9	24	13
splicing	0	0	1	0	0	0	0	2	3
UTR5	7	3	8	2	5	5	4	5	4
upstream/downstream	0	0	0	0	0	0	1	1	0
Total	2863	1971	4195	1826	2753	2666	1908	4843	2114

*Excluding variants in MS09-378A-D not present in any over sample

9997957702	MS09-378A-D	MS09-378A-D corrected*	Total	Average	Total corrected*	Average corrected*	Average of mets	Median of mets	Min of mets	Max of mets
606	7555	277	15507	1409.7	8229	748.1	795.2	695.5	533	1451
1290	17444	512	34164	3105.8	17232	1566.5	1672	1468	1114	2962
122	1457	54	3145	285.9	1742	158.4	168.8	152.5	114	302
27	28	12	310	28.2	294	26.7	28.2	26	13	49
7	56	2	171	15.5	117	10.6	11.5	10	7	22
11	27	8	156	14.2	137	12.5	12.9	13	9	19
11	42	7	169	15.4	134	12.2	12.7	11.5	10	17
14	28	5	191	17.4	168	15.3	16.3	15.5	9	24
0	0	0	6	0.5	6	0.5	0.6	0	0	3
2	6	3	51	4.6	48	4.4	4.5	4.5	2	8
0	0	0	2	0.2	2	0.2	0.2	0	0	1
2090	26643	880	53872	4897.5	28109	2555.4	2722.9	2390	1826	4843

Supplementary Table 8. Patient 2 variant spreadsheet

Chr	Pos	Ref	Alt	Func.refGene	Gene.refGene	ExonicFunc.refGene	CLINSIG	CLNDBN
2	111770680	TATA	T	intronic	*ACOXL*	.	.	.
3	65065268	A	G	intergenic	*ADAMTS9-AS2,MAGI1*	.	.	.
8	132107618	G	T	intergenic	*ADCY8,EFR3A*	.	.	.
8	132132260	C	T	intergenic	*ADCY8,EFR3A*	.	.	.
8	132547453	T	C	intergenic	*ADCY8,EFR3A*	.	.	.
18	1153261	A	T	intergenic	*ADCYAP1,LINC00470*	.	.	.
18	1238296	C	T	intergenic	*ADCYAP1,LINC00470*	.	.	.
12	131488855	C	T	intronic	*ADGRD1*	.	.	.
4	63159214	C	T	intergenic	*ADGRL3-AS1,TECRL*	.	.	.
1	80824498	A	C	intergenic	*ADGRL4,LOC101927412*	.	.	.
9	88230024	ACCACTAAGAATAGGATTAAGTTAAAT	A	intronic	*AGTPBP1*	.	.	.
14	32835735	T	C	intronic	*AKAP6*	.	.	.
14	33272043	C	T	intronic	*AKAP6*	.	.	.
9	117109798	A	G	intronic	*AKNA*	.	.	.
10	62442084	C	T	intronic	*ANK3*	.	.	.
2	70013777	T	C	intronic	*ANXA4*	.	.	.
14	32591478	TGCC	T	intronic	*ARHGAP5*	.	.	.
10	28115356	G	A	intronic	*ARMC4*	.	.	.
9	119252551	TAAAAAAAAAAAAAAAAAAAAAAAAAA	T	intronic	*ASTN2*	.	.	.
13	70783596	G	T	intergenic	*ATXN8OS,LINC00348*	.	.	.
3	52442921	G	A	intronic	*BAP1*	.	.	.

2	160450492	G	C	intronic	*BAZ2B*	.	.	.
17	59056891	A	G	intronic	*BCAS3*	.	.	.
1	45253138	C	T	exonic	*BEST4*	synonymous_SNV	.	.
12	26274653	C	T	UTR3	*BHLHE41*	.	.	.
15	60181179	G	A	intergenic	*BNIP2,FOXB1*	.	.	.
1	92481414	C	T	intergenic	*BRDT,EPHX4*	.	.	.
X	79949540	A	C	intronic	*BRWD3*	.	.	.
14	57952490	C	A	intronic	*C14orf105*	.	.	.
14	99188672	C	A	intergenic	*C14orf177,BCL11B*	.	.	.
2	119915093	C	G	intronic	*C1QL2*	.	.	.
5	24149880	A	T	ncRNA_intronic	*C5orf17*	.	.	.
8	86308343	G	T	intergenic	*CA1,CA3*	.	.	.
17	50252886	C	T	intergenic	*CA10,C17orf112*	.	.	.
12	2580892	A	G	intronic	*CACNA1C*	.	.	.
9	138779112	G	C	intronic	*CAMSAP1*	.	.	.
11	10390979	T	A	ncRNA_intronic	*CAND1.11*	.	.	.
19	48743307	C	T	intronic	*CARD8*	.	.	.
6	92409809	T	C	intergenic	*CASC6,EPHA7*	.	.	.
6	93787785	C	T	intergenic	*CASC6,EPHA7*	.	.	.
X	41944688	G	A	intergenic	*CASK,PPP1R2P9*	.	.	.
X	150040774	T	C	intronic	*CD99L2*	.	.	.
6	44663536	T	C	intergenic	*CDC5L,SUPT3H*	.	.	.
16	66419416	G	A	intronic	*CDH5*	.	.	.
20	48846321	C	T	intergenic	*CEBPB,LINC01272*	.	.	.
15	79021513	G	A	intergenic	*CHRNB4,LOC646938*	.	.	.
11	99419758	G	A	intronic	*CNTN5*	.	.	.
3	1702974	T	A	intergenic	*CNTN6,CNTN4*	.	.	.
7	51704780	T	C	intergenic	*COBL,POM121L12*	.	.	.
11	14502815	A	G	intronic	*COPB1*	.	.	.
4	8724784	G	T	intergenic	*CPZ,HMX1*	.	.	.
1	152516003	G	T	intergenic	*CRCT1,LCE3E*	.	.	.
8	113888138	C	T	intronic	*CSMD3*	.	.	.
7	143455113	T	C	upstream	*CTAGE6*	.	.	.

5	99369604	G	A	intergenic	*CTD-2151A2.1,LOC100133050*	.	.	.
2	137334524	G	T	intergenic	*CXCR4,THSD7B*	.	.	.
21	25631098	A	G	intergenic	*D21S2088E,LOC101927869*	.	.	.
13	73083301	A	G	intergenic	*DACH1,MZT1*	.	.	.
1	69369361	T	C	intergenic	*DEPDC1-AS1,LRRC7*	.	.	.
14	24476270	G	A	UTR5	*DHRS4L1*	.	.	.
X	2152823	C	T	intronic	*DHRSX*	.	.	.
X	97512089	A	T	intergenic	*DIAPH2,XRCC6P5*	.	.	.
10	842043	G	A	intergenic	*DIP2C,LARP4B*	.	.	.
11	31860318	TAGGTTTTTAAAAAGTTATTACTTTTTTAAA	T	ncRNA_intronic	*DKFZp686K1684*	.	.	.
10	124519007	T	C	ncRNA_intronic	*DMBT1P1*	.	.	.
1	225470994	G	A	intronic	*DNAH14*	.	.	.
16	21118319	A	G	intronic	*DNAH3*	.	.	.
20	54017404	C	G	intergenic	*DOK5,LINC01441*	.	.	.
7	154476994	C	T	intronic	*DPP6*	.	.	.
12	77526178	G	A	intergenic	*E2F7,NAV3*	.	.	.
5	83704761	G	T	intergenic	*EDIL3,NBPF22P*	.	.	.
5	85127708	G	A	intergenic	*EDIL3,NBPF22P*	.	.	.
10	119416700	C	T	intergenic	*EMX2,RAB11FIP2*	.	.	.
X	130113731	C	A	intergenic	*ENOX2,LINC01201*	.	.	.
4	75229389	A	G	intergenic	*EPGN,EREG*	.	.	.
10	50727103	G	A	intronic	*ERCC6,ERCC6-PGBD3,PGBD3*	.	.	.
X	71443653	G	A	intronic	*ERCC6L,PIN4*	.	.	.
2	68009506	G	T	intergenic	*ETAA1,LOC101927701*	.	.	.
8	72280515	G	A	intergenic	*EYA1,MSC*	.	.	.
1	28313242	C	T	intronic	*EYA3*	.	.	.
6	66300612	C	T	intronic	*EYS*	.	.	.
5	175896450	C	G	intronic	*FAF2*	.	.	.

X	94196288	T	C	intergenic	*FAM133A,BRDTP1*	.	.	.
5	93484784	T	C	intergenic	*FAM172A,KIAA0825*	.	.	.
X	103441913	C	T	intergenic	*FAM199X,ESX1*	.	.	.
17	42004743	T	C	intergenic	*FAM215A,PPY*	.	.	.
X	35265594	T	C	intergenic	*FAM47B,MAGEB16*	.	.	.
1	166426052	T	C	intergenic	*FAM78B,FMO9P*	.	.	.
4	126318772	T	A	intronic	*FAT4*	.	.	.
13	102632710	G	A	intronic	*FGF14*	.	.	.
12	27114299	G	A	intronic	*FGFR1OP2*	.	.	.
17	74133748	C	G	exonic	*FOXJ1*	nonsynonymous_SNV	.	.
4	46202654	T	C	intergenic	*GABRG1,GABRA2*	.	.	.
X	49439414	C	G	intergenic	*GAGE1,PAGE1*	.	.	.
15	58158728	A	G	intergenic	*GCOM1,ALDH1A2*	.	.	.
19	48134657	G	T	intronic	*GLTSCR1*	.	.	.
6	2453559	G	A	intergenic	*GMDS-AS1,LINC01600*	.	.	.
19	3118942	A	T	exonic	*GNA11*	nonsynonymous_SNV	.	.
9	80699036	C	T	intergenic	*GNAQ,CEP78*	.	.	.
4	46025162	C	T	intergenic	*GNPDA2,GABRG1*	.	.	.
7	50726794	T	A	intronic	*GRB10*	.	.	.
4	43284801	TACA	T	intergenic	*GRXCR1,LVCAT1*	.	.	.
4	43443282	A	G	intergenic	*GRXCR1,LVCAT1*	.	.	.
2	189450110	G	A	intronic	*GULP1*	.	.	.
5	21610282	A	C	intergenic	*GUSBP1,CDH12*	.	.	.
11	2078651	C	G	intergenic	*H19,IGF2*	.	.	.
4	174935317	C	T	intergenic	*HAND2-AS1,LOC101928509*	.	.	.
2	197261414	A	G	intronic	*HECW2*	.	.	.
1	221313349	G	A	intergenic	*HLX,C1orf140*	.	.	.
4	95293909	C	T	intergenic	*HPGDS,PDLIM5*	.	.	.

10	100935938	C	A	intronic	*HPSE2*	.	.	.
4	11531300	T	G	intergenic	*HS3ST1,LOC101929019*	.	.	.
2	8844126	C	T	intergenic	*ID2,KIDINS220*	.	.	.
10	94301921	A	G	intronic	*IDE*	.	.	.
17	59960081	T	G	intronic	*INTS2*	.	.	.
21	46342833	G	A	ncRNA_exonic	*ITGB2-AS1*	.	.	.
7	27995654	G	A	intronic	*JAZF1*	.	.	.
12	75463755	C	T	intronic	*KCNC2*	.	.	.
17	21382149	C	A	intergenic	*KCNJ12,C17orf51*	.	.	.
17	52076561	G	A	intergenic	*KIF2B,TOM1L1*	.	.	.
17	52096556	A	C	intergenic	*KIF2B,TOM1L1*	.	.	.
6	39559739	T	C	intronic	*KIF6*	.	.	.
17	38868404	T	G	intergenic	*KRT24,KRT25*	.	.	.
2	143847914	C	G	intergenic	*KYNU,ARHGAP15*	.	.	.
4	20096340	A	T	intergenic	*LCORL,SLIT2*	.	.	.
X	140473419	A	T	intergenic	*LDOC1,SPANXA2-OT1*	.	.	.
1	205358440	G	T	intronic	*LEMD1*	.	.	.
6	64152771	C	T	intergenic	*LGSN,PTP4A1*	.	.	.
5	38486406	A	T	intronic	*LIFR*	.	.	.
1	218205888	C	A	intergenic	*LINC00210,LOC101929631*	.	.	.
16	34323328	G	A	intergenic	*LINC00273,UBE2MP1*	.	.	.
8	47756101	G	A	ncRNA_intronic	*LINC00293*	.	.	.
8	47832341	T	C	intergenic	*LINC00293,LOC100287846*	.	.	.
13	106391153	CA	C	ncRNA_intronic	*LINC00343*	.	.	.
13	68488563	A	G	intergenic	*LINC00364,LINC00550*	.	.	.
13	59901579	C	T	intergenic	*LINC00374,DIAPH3*	.	.	.
6	170378920	G	A	intergenic	*LINC00574,LOC102724511*	.	.	.
2	216719593	C	T	intergenic	*LINC00607,MREG*	.	.	.
6	58675306	T	C	intergenic	*LINC00680-GUSBP4,NONE*	.	.	.

10	26097254	A	G	intergenic	*LINC00836,LOC101929073*	.	.	.
8	126958344	CTAGTTAAACCATGTGTTTTACATGCTGGCAAAGGTCAGTTGTTTTACTGTCCT	C	ncRNA_exonic	*LINC00861*	.	.	.
11	115751213	T	C	intergenic	*LINC00900,BUD13*	.	.	.
7	63495838	C	T	intergenic	*LINC01005,ZNF727*	.	.	.
1	239019969	G	A	intergenic	*LINC01139,CHRM3*	.	.	.
1	239592704	T	A	intergenic	*LINC01139,CHRM3*	.	.	.
2	71118790	G	A	intergenic	*LINC01143,VAX2*	.	.	.
10	130985457	G	A	intergenic	*LINC01163,MGMT*	.	.	.
15	21931359	C	A	intergenic	*LINC01193,LOC646214*	.	.	.
4	101897494	C	G	intergenic	*LINC01216,PPP3CA*	.	.	.
2	5719269	C	A	intergenic	*LINC01249,LINC01248*	.	.	.
4	190644279	C	T	intergenic	*LINC01262,LINC01596*	.	.	.
X	50976402	T	C	intergenic	*LINC01284,NUDT10*	.	.	.
2	4539524	C	A	intergenic	*LINC01304,LINC01249*	.	.	.
13	105025187	C	T	intergenic	*LINC01309,DAOA-AS1*	.	.	.
1	4425122	C	G	intergenic	*LINC01346,LOC284661*	.	.	.
9	109446741	G	A	intergenic	*LINC01505,ZNF462*	.	.	.
9	83002216	A	G	intergenic	*LINC01507,TLE1*	.	.	.

16	51961175	TA	T	intergenic	*LINC01571,C16orf97*	.	.	.
11	30017840	G	C	intergenic	*LINC01616,KCNA4*	.	.	.
6	1288826	A	G	intergenic	*LINC01622,FOXQ1*	.	.	.
5	96378207	C	G	intergenic	*LNPEP,LIX1*	.	.	.
13	28165874	T	C	intronic	*LNX2*	.	.	.
1	163566705	T	C	intergenic	*LOC100422212,PBX1*	.	.	.
18	71649706	C	G	intergenic	*LOC100505817,FBXO15*	.	.	.
5	2697534	G	A	intergenic	*LOC100506858,IRX2*	.	.	.
2	82076187	A	T	intergenic	*LOC100507201,LOC1720*	.	.	.
4	142452738	C	A	intergenic	*LOC100507639,IL15*	.	.	.
14	23401066	G	A	ncRNA_intronic	*LOC101926933*	.	.	.
10	103012263	A	C	downstream	*LOC101927419*	.	.	.
10	107479384	C	T	ncRNA_intronic	*LOC101927549*	.	.	.
16	60212457	G	A	intergenic	*LOC101927580,LOC729159*	.	.	.
2	78273257	A	G	ncRNA_intronic	*LOC101927967*	.	.	.
2	79023031	G	C	intergenic	*LOC101927967,REG3G*	.	.	.
5	172184943	C	A	intronic	*LOC101928093*	.	.	.
3	35026536	C	T	ncRNA_intronic	*LOC101928135*	.	.	.
X	5468879	T	C	intergenic	*LOC101928201,NLGN4X*	.	.	.
X	5718754	G	A	intergenic	*LOC101928201,NLGN4X*	.	.	.
7	125985193	A	G	intergenic	*LOC101928283,GRM8*	.	.	.
7	125992473	T	A	intergenic	*LOC101928283,GRM8*	.	.	.
X	107191399	A	G	intergenic	*LOC101928335,TEX13B*	.	.	.

1	30099748	C	T	intergenic	*LOC101928460,LOC101929406*	.	.	.
6	88613512	G	C	ncRNA_intronic	*LOC101928911*	.	.	.
5	4968673	G	A	intergenic	*LOC101929153,LINC01020*	.	.	.
4	27674005	A	G	intergenic	*LOC101929199,MIR4275*	.	.	.
12	4809806	A	T	ncRNA_exonic	*LOC101929549*	.	.	.
1	96718023	C	T	intergenic	*LOC102723661,LOC101928241*	.	.	.
8	79382923	T	C	intergenic	*LOC102724874,PKIA*	.	.	.
18	42123027	T	G	ncRNA_exonic	*LOC105667213*	.	.	.
10	8959268	G	A	intergenic	*LOC105755953,LOC101928272*	.	.	.
11	58707652	G	A	ncRNA_intronic	*LOC283194*	.	.	.
18	61967672	T	A	ncRNA_intronic	*LOC284294*	.	.	.
18	61967673	C	T	ncRNA_intronic	*LOC284294*	.	.	.
8	20917702	T	C	intergenic	*LOC286114,LOC101929172*	.	.	.
21	16141169	C	A	intergenic	*LOC388813,NRIP1*	.	.	.
5	50670193	A	T	ncRNA_exonic	*LOC642366*	.	.	.
2	22112293	C	G	intergenic	*LOC645949,LOC102723362*	.	.	.
11	50462913	G	A	intergenic	*LOC646813,OR4A5*	.	.	.
11	51254288	GA	G	intergenic	*LOC646813,OR4A5*	.	.	.
2	14920814	C	A	intergenic	*LOC653602,NBAS*	.	.	.
18	22421737	C	G	intergenic	*LOC729950,ZNF521*	.	.	.
11	40724949	G	A	intronic	*LRRC4C*	.	.	.
11	25021155	G	A	intronic	*LUZP2*	.	.	.
X	52018976	T	C	intergenic	*MAGED4,MIR8088*	.	.	.
6	119531899	G	C	intronic	*MAN1A1*	.	.	.
5	109089558	G	A	intronic	*MAN2A1*	.	.	.
1	36630953	G	T	intronic	*MAP7D1*	.	.	.
X	20130819	C	T	intronic	*MAP7D2*	.	.	.

14	47338072	C	A	intronic	*MDGA2*	.	.	.
6	78855496	G	C	intergenic	*MEI4,IRAK1BP1*	.	.	.
7	141844897	TG	T	intronic	*MGAM2*	.	.	.
6	31404891	G	A	intergenic	*MICA,HCP5*	.	.	.
13	55649885	G	T	intergenic	*MIR1297,MIR5007*	.	.	.
X	39823662	A	G	intergenic	*MIR3937,BCOR*	.	.	.
9	92820405	T	C	intergenic	*MIR4290HG,LINC01508*	.	.	.
9	96673963	C	G	intergenic	*MIR4291,BARX1*	.	.	.
18	36049621	T	C	intergenic	*MIR4318,MIR924HG*	.	.	.
3	24753650	A	T	intergenic	*MIR4792,RARB*	.	.	.
1	16217238	C	T	ncRNA_intronic	*MIR5096*	.	.	.
3	104057535	G	T	intergenic	*MIR548A3,ALCAM*	.	.	.
21	19998846	A	T	ncRNA_intronic	*MIR548XHG*	.	.	.
21	20940580	G	A	intergenic	*MIR548XHG,LINC00320*	.	.	.
21	21790045	C	T	intergenic	*MIR548XHG,LINC00320*	.	.	.
10	122551252	T	A	ncRNA_intronic	*MIR5694,WDR11-AS1*	.	.	.
11	23584820	A	G	intergenic	*MIR8054,LUZP2*	.	.	.
11	24476103	T	C	intergenic	*MIR8054,LUZP2*	.	.	.
3	96500050	A	C	intergenic	*MIR8060,EPHA6*	.	.	.
14	45758674	A	G	intergenic	*MIS18BP1,LINC00871*	.	.	.
8	16087075	A	T	intergenic	*MSR1,FGF20*	.	.	.
8	10099224	A	G	intronic	*MSRA*	.	.	.
17	8419073	G	A	intronic	*MYH10*	.	.	.
5	17063521	G	A	intergenic	*MYO10,LOC285696*	.	.	.
2	1933471	C	T	intronic	*MYT1L*	.	.	.
9	88569215	C	G	intronic	*NAA35*	.	.	.
X	72518927	G	A	intergenic	*NAP1L2,CDX4*	.	.	.
X	72430629	A	C	intergenic	*NAP1L6,NAP1L2*	.	.	.
5	85779743	A	T	intergenic	*NBPF22P,COX7C*	.	.	.
X	43885665	C	T	intergenic	*NDP,EFHC2*	.	.	.
11	21328862	C	A	intronic	*NELL1*	.	.	.

2	219939293	C	T	downstream	*NHEJ1*	.	.	.
17	5506072	A	T	intergenic	*NLRP1,LOC339166*	.	.	.
18	31852899	G	A	intergenic	*NOL4,DTNA*	.	.	.
1	195823975	A	T	intergenic	*NONE,KCNT2*	.	.	.
8	47293532	G	A	intergenic	*NONE,LINC00293*	.	.	.
8	47304503	A	G	intergenic	*NONE,LINC00293*	.	.	.
19	27791000	G	A	intergenic	*NONE,LINC00662*	.	.	.
3	84068704	G	T	intergenic	*NONE,LINC00971*	.	.	.
2	91793845	G	A	intergenic	*NONE,LOC654342*	.	.	.
5	71574	G	T	intergenic	*NONE,PLEKHG4B*	.	.	.
5	104007	C	A	intergenic	*NONE,PLEKHG4B*	.	.	.
16	17177889	C	T	intergenic	*NPIPA8,XYLT1*	.	.	.
7	108058269	C	T	intronic	*NRCAM*	.	.	.
14	80410479	G	A	intergenic	*NRXN3,DIO2*	.	.	.
3	94305752	C	T	intergenic	*NSUN3,MIR6730*	.	.	.
11	131667214	A	G	intronic	*NTM*	.	.	.
7	56253373	G	A	intergenic	*NUPR2,LOC650226*	.	.	.
1	248234948	G	A	intronic	*OR2L13*	.	.	.
22	44533587	G	T	intronic	*PARVB*	.	.	.
2	194396851	T	A	intergenic	*PCGEM1,LOC101927406*	.	.	.
1	233448769	T	A	intergenic	*PCNXL2,KIAA1804*	.	.	.
3	34379884	A	G	intergenic	*PDCD6IP,LOC101928135*	.	.	.
5	58281638	ATAAAATATTTCACGAAGCTACATATCACAAGTTTACAAGTTATTAGAAGGAT	A	intronic	*PDE4D*	.	.	.
4	157797622	C	T	intronic	*PDGFC*	.	.	.
7	10506762	T	C	intergenic	*PER4,NDUFA4*	.	.	.
20	52977201	T	C	intergenic	*PFDN4,DOK5*	.	.	.
8	75826412	C	T	intergenic	*PI15,CRISPLD1*	.	.	.

1	204459037	C	A	UTR5	*PIK3C2B*	.	.	.
5	67868694	G	A	intergenic	*PIK3R1,LOC101928885*	.	.	.
17	65552052	A	ATC	intronic	*PITPNC1*	.	.	.
2	159322223	C	G	intronic	*PKP4*	.	.	.
20	9172931	G	A	intronic	*PLCB4*	.	.	.
1	242864237	A	G	intergenic	*PLD5,LINC01347*	.	.	.
1	242926916	A	G	intergenic	*PLD5,LINC01347*	.	.	.
1	209446747	C	T	intergenic	*PLXNA2,MIR205HG*	.	.	.
X	7992852	G	T	intergenic	*PNPLA4,MIR651*	.	.	.
13	28282883	G	T	intergenic	*POLR1D,GSX1*	.	.	.
7	53122193	C	G	intergenic	*POM121L12,LINC01446*	.	.	.
3	87899309	G	C	intergenic	*POU1F1,HTR1F*	.	.	.
X	27682375	G	C	intergenic	*PPP4R3CP,DCAF8L2*	.	.	.
10	6642706	C	T	intergenic	*PRKCQ-AS1,LOC101928150*	.	.	.
8	48812978	A	G	exonic	*PRKDC*	unknown	.	.
X	126505508	C	T	intergenic	*PRR32,ACTRT1*	.	.	.
X	126694565	C	T	intergenic	*PRR32,ACTRT1*	.	.	.
1	171585719	G	T	intergenic	*PRRC2C,MYOC*	.	.	.
12	28383610	T	G	intergenic	*PTHLH,CCDC91*	.	.	.
9	96928562	C	G	intergenic	*PTPDC1,MIRLET7A1*	.	.	.
6	129001990	C	T	intergenic	*PTPRK,LAMA2*	.	.	.
2	37582925	G	C	intronic	*QPCT*	.	.	.
5	36421849	G	A	intergenic	*RANBP3L,SLC1A3*	.	.	.
12	114604820	G	A	intergenic	*RBM19,TBX5*	.	.	.
20	56069903	G	A	intergenic	*RBM38,CTCFL*	.	.	.
2	86586076	T	C	intergenic	*REEP1,KDM3A*	.	.	.
2	108523918	G	A	intergenic	*RGPD4,SLC5A7*	.	.	.
6	72960799	A	G	intronic	*RIMS1*	.	.	.
17	74253807	G	T	intergenic	*RNF157,UBALD2*	.	.	.
X	36788015	G	T	intergenic	*RP11-87M18.2,FAM47C*	.	.	.

2	154499654	T	G	intergenic	*RPRM,GALNT13*	.	.	.
1	201546699	CTTTGTCAAAAATCAGTTGGGCATAT	C	intergenic	*RPS10P7,NAV1*	.	.	.
X	56177552	C	G	intergenic	*RRAGB,KLF8*	.	.	.
22	23410657	G	C	intronic	*RSPH14*	.	.	.
1	237899716	TGCACCACTGCACTCCAGGCTGGGCGATGGAGTGA	T	intronic	*RYR2*	.	.	.
16	23239430	C	T	intergenic	*SCNN1G,SCNN1B*	.	.	.
2	20447264	T	C	intergenic	*SDC1,PUM2*	.	.	.
7	85300658	A	G	intergenic	*SEMA3D,GRM3*	.	.	.
7	45789235	A	G	ncRNA_intronic	*SEPT7P2*	.	.	.
2	223408942	C	T	intronic	*SGPP2*	.	.	.
11	70571959	G	A	intronic	*SHANK2*	.	.	.
16	13618839	G	T	intergenic	*SHISA9,ERCC4*	.	.	.
11	111516301	T	G	intronic	*SIK2*	.	.	.
6	111523251	G	A	intronic	*SLC16A10*	.	.	.
4	10023662	C	G	intronic	*SLC2A9*	.	.	.
4	41998023	G	A	intronic	*SLC30A9*	.	.	.
2	165767837	T	C	intronic	*SLC38A11*	.	.	.
17	26752219	G	T	intergenic	*SLC46A1,SLC13A2*	.	.	.
14	70651525	G	A	intronic	*SLC8A3*	.	.	.
15	67361086	G	A	intronic	*SMAD3*	.	.	.
11	93278473	G	A	intergenic	*SMCO4,CEP295*	.	.	.
17	2148143	G	A	intronic	*SMG6*	.	.	.
1	99257084	G	GTGTAACAC	intergenic	*SNX7,PLPPR5*	.	.	.
2	215269278	T	A	intronic	*SPAG16*	.	.	.
5	136590879	C	T	intronic	*SPOCK1*	.	.	.
14	65240049	C	A	exonic	*SPTB*	nonsynonymous_SNV	.	.
X	38021785	T	C	intronic	*SRPX*	.	.	.
5	111016408	GTTAGAC	G	ncRNA_intronic	*STARD4-AS1*	.	.	.
X	67964190	G	A	intergenic	*STARD8,EFNB1*	.	.	.

11	8598488	C	T	intronic	*STK33*	.	.	.
10	70590930	ATAGATATTTATTTATCTATTTATTTATTTTTGAGACGATGTT	A	intronic	*STOX1*	.	.	.
1	185243965	T	C	intronic	*SWT1*	.	.	.
20	13513540	A	C	intronic	*TASP1*	.	.	.
6	121537092	T	C	intronic	*TBC1D32*	.	.	.
10	133071462	G	A	intronic	*TCERG1L*	.	.	.
21	10806339	A	G	intergenic	*TEKT4P2,TPTE*	.	.	.
17	56639138	C	T	intronic	*TEX14*	.	.	.
5	126004646	C	T	intergenic	*TEX43,LMNB1*	.	.	.
6	50967731	G	T	intergenic	*TFAP2B,PKHD1*	.	.	.
6	54193490	T	G	intronic	*TINAG*	.	.	.
4	167503239	A	G	intergenic	*TLL1,SPOCK3*	.	.	.
X	12890141	G	A	intronic	*TLR7*	.	.	.
17	10674517	G	T	ncRNA_intronic	*TMEM220-AS1*	.	.	.
7	5458846	G	T	intronic	*TNRC18*	.	.	.
7	22877507	C	T	intergenic	*TOMM7,SNORD93*	.	.	.
8	60218201	T	C	intergenic	*TOX,CA8*	.	.	.
17	3506251	A	C	intronic	*TRPV1*	.	.	.
2	122619284	T	C	intergenic	*TSN,NONE*	.	.	.
6	41011774	C	G	exonic	*TSPO2*	synonymous_SNV	.	.
2	179499927	A	T	exonic	*TTN*	nonsynonymous_SNV	.	.
8	15402379	G	C	intronic	*TUSC3*	.	.	.
2	238912170	G	A	ncRNA_intronic	*UBE2F-SCLY*	.	.	.
16	34476901	C	T	intergenic	*UBE2MP1,LINC01566*	.	.	.
2	136537178	C	G	intronic	*UBXN4*	.	.	.
4	70314849	C	A	intergenic	*UGT2B28,UGT2B4*	.	.	.
1	16158297	CT	C	intergenic	*UQCRHL,FLJ37453*	.	.	.
1	230038133	C	T	intergenic	*URB2,GALNT2*	.	.	.
1	101318298	G	C	intergenic	*VCAM1,EXTL2*	.	.	.
16	78336337	A	G	intronic	*WWOX*	.	.	.

1	168616219	T	G	intergenic	*XCL1,DPT*	.	.	.
8	56266685	T	C	intronic	*XKR4*	.	.	.
1	180666730	A	G	intronic	*XPR1*	.	.	.
12	58562861	T	A	intergenic	*XRCC6BP1,LOC101927653*	.	.	.
16	31603425	G	T	intergenic	*YBX3P1,CLUHP3*	.	.	.
5	112913937	T	G	intronic	*YTHDC2*	.	.	.
5	113285074	G	A	intergenic	*YTHDC2,KCNN2*	.	.	.
22	32344534	T	A	intronic	*YWHAH*	.	.	.
9	89525033	A	G	intergenic	*ZCCHC6,GAS1*	.	.	.
20	45909079	G	C	intronic	*ZMYND8*	.	.	.
10	44063267	A	C	intronic	*ZNF239*	.	.	.
8	81645144	G	A	intronic	*ZNF704*	.	.	.
19	23087957	C	G	intergenic	*ZNF99,ZNF728*	.	.	.
X	58094075	G	C	intergenic	*ZXDA,NONE*	.	.	.
5	126211027	T	C	intronic	*43893*	.	.	.
5	178269717	G	A	intergenic	*AACSP1,ZNF354B*	.	.	.
3	151638668	G	A	ncRNA_intronic	*AADACL2-AS1*	.	.	.
3	151809785	T	C	intergenic	*AADACL2-AS1,MBNL1-AS1*	.	.	.
14	78460918	C	G	intergenic	*ADCK1,NONE*	.	.	.
4	100287378	G	A	intergenic	*ADH1C,ADH7*	.	.	.
13	114104794	G	T	intronic	*ADPRHL1*	.	.	.
1	50060838	G	A	intronic	*AGBL4*	.	.	.
14	33364258	A	G	intergenic	*AKAP6,NPAS3*	.	.	.
11	43928398	C	G	intronic	*ALKBH3*	.	.	.
7	38676979	T	C	intergenic	*AMPH,FAM183BP*	.	.	.
16	89489424	A	C	intronic	*ANKRD11*	.	.	.
12	99185753	C	A	intronic	*ANKS1B*	.	.	.
12	99817822	C	G	intronic	*ANKS1B*	.	.	.
20	57090046	G	T	upstream	*APCDD1L,APCDD1L-AS1*	.	.	.
3	195357605	AC	A	intergenic	*APOD,SDHAP2*	.	.	.
2	144132830	GCTCT	G	intronic	*ARHGAP15*	.	.	.
18	6881294	CT	C	intronic	*ARHGAP28*	.	.	.
11	129187101	C	T	intergenic	*ARHGAP32,BARX2*	.	.	.
18	77036329	C	G	intronic	*ATP9B*	.	.	.

6	16583896	C	T	intronic	*ATXN1*	.	.	.
7	69969431	G	A	intronic	*AUTS2*	.	.	.
11	129313296	A	G	intronic	*BARX2*	.	.	.
13	23591805	G	A	intergenic	*BASP1P1,SGCG*	.	.	.
4	122793088	C	T	intergenic	*BBS7,TRPC3*	.	.	.
17	59188852	G	A	intronic	*BCAS3*	.	.	.
3	165714407	T	A	intergenic	*BCHE,ZBBX*	.	.	.
6	82006698	C	G	intergenic	*BCKDHB,FAM46A*	.	.	.
4	42341429	A	G	intergenic	*BEND4,SHISA3*	.	.	.
17	76151922	G	A	intronic	*C17orf99*	.	.	.
1	244728877	G	T	intronic	*C1orf101*	.	.	.
20	3343595	G	A	intronic	*C20orf194*	.	.	.
2	54641433	A	G	intergenic	*C2orf73,SPTBN1*	.	.	.
8	61240746	T	C	intergenic	*CA8,LINC01301*	.	.	.
6	93593342	AG	A	intergenic	*CASC6,EPHA7*	.	.	.
17	42978516	T	G	intronic	*CCDC103*	.	.	.
7	64672987	T	C	intergenic	*CCT6P3,ZNF92*	.	.	.
17	72465941	G	C	intronic	*CD300A*	.	.	.
5	20186795	C	A	intronic	*CDH18*	.	.	.
9	90734130	A	C	intergenic	*CDK20,SPATA31C2*	.	.	.
13	26904755	C	G	intronic	*CDK8*	.	.	.
6	126711904	A	G	intergenic	*CENPW,MIR588*	.	.	.
5	122799144	T	G	intergenic	*CEP120,CSNK1G3*	.	.	.
X	35949309	T	G	intronic	*CFAP47*	.	.	.
3	126693281	A	C	intergenic	*CHCHD6,PLXNA1*	.	.	.
20	40106778	T	C	intronic	*CHD6*	.	.	.
16	19375746	G	A	intergenic	*CLEC19A,TMC5*	.	.	.
6	123327484	G	A	intronic	*CLVS2*	.	.	.
16	66719490	C	G	intronic	*CMTM4*	.	.	.
11	99964169	AT	A	intronic	*CNTN5*	.	.	.
4	46824716	C	T	intronic	*COX7B2*	.	.	.
12	94290929	G	A	intergenic	*CRADD,PLXNC1*	.	.	.
8	5189719	G	A	intergenic	*CSMD1,LOC100287015*	.	.	.
8	115441187	T	C	intergenic	*CSMD3,TRPS1*	.	.	.
8	68020753	A	T	intronic	*CSPP1*	.	.	.
X	119689793	T	C	intronic	*CUL4B*	.	.	.

13	73215184	G	A	intergenic	*DACH1,MZT1*	.	.	.
11	102945568	C	T	intronic	*DCUN1D5*	.	.	.
7	14863764	A	AC	intronic	*DGKB*	.	.	.
8	13060345	G	A	intronic	*DLC1*	.	.	.
3	197102771	C	A	intergenic	*DLG1-AS1,BDH1*	.	.	.
X	32671045	T	C	intronic	*DMD*	.	.	.
3	57357391	C	T	intronic	*DNAH12*	.	.	.
2	230424519	G	C	intronic	*DNER*	.	.	.
2	115486358	A	G	intronic	*DPP10*	.	.	.
11	117306875	G	A	intronic	*DSCAML1*	.	.	.
2	25863651	G	A	intronic	*DTNB*	.	.	.
12	12724418	C	A	intergenic	*DUSP16,CREBL2*	.	.	.
8	29499269	T	C	intergenic	*DUSP4,LINC00589*	.	.	.
6	12297000	G	T	UTR3	*EDN1*	.	.	.
7	110170771	G	A	intergenic	*EIF3IP1,IMMP2L*	.	.	.
5	60181555	A	G	intronic	*ERCC8*	.	.	.
5	137882896	G	C	intergenic	*ETF1,HSPA9*	.	.	.
8	72305316	A	G	intergenic	*EYA1,MSC*	.	.	.
6	6220316	A	G	intronic	*F13A1*	.	.	.
2	111779	C	T	intergenic	*FAM110C,SH3YL1*	.	.	.
7	23086790	G	A	intergenic	*FAM126A,KLHL7-AS1*	.	.	.
5	93455014	G	A	intergenic	*FAM172A,KIAA0825*	.	.	.
20	49346964	G	C	intergenic	*FAM65C,PARD6B*	.	.	.
1	144016820	C	A	intergenic	*FAM72C,NBPF20*	.	.	.
20	37557578	T	C	intronic	*FAM83D*	.	.	.
12	133083184	A	G	intronic	*FBRSL1*	.	.	.
6	37063750	G	A	intergenic	*FGD2,PIM1*	.	.	.
3	192468316	G	T	intergenic	*FGF12,MB21D2*	.	.	.
13	102963738	A	AC	ncRNA_intronic	*FGF14-IT1*	.	.	.
2	216299043	G	A	intronic	*FN1*	.	.	.
3	171929296	TA	T	intronic	*FNDC3B*	.	.	.
14	89773195	T	A	intronic	*FOXN3*	.	.	.
19	52279100	C	T	intergenic	*FPR2,FPR3*	.	.	.
X	12160030	G	T	intronic	*FRMPD4*	.	.	.
4	162823540	A	G	intronic	*FSTL5*	.	.	.
5	161673420	CAGAGT	C	intergenic	*GABRG2,CCNG1*	.	.	.

10	8176572	T	C	intergenic	*GATA3,LINC00708*	.	.	.
1	89878725	G	A	ncRNA_intronic	*GBP1P1*	.	.	.
19	45592361	G	C	intronic	*GEMIN7*	.	.	.
5	179779022	G	GTGGC	intronic	*GFPT2*	.	.	.
22	23002539	G	C	intergenic	*GGTLC2,MIR650*	.	.	.
12	104434227	G	A	intronic	*GLT8D2*	.	.	.
6	2301087	A	G	ncRNA_intronic	*GMDS-AS1*	.	.	.
X	136279316	G	C	intergenic	*GPR101,ZIC3*	.	.	.
6	103274434	C	A	intergenic	*GRIK2,HACE1*	.	.	.
12	66934157	A	G	intronic	*GRIP1*	.	.	.
11	88517436	T	C	intronic	*GRM5*	.	.	.
3	119627445	C	A	intronic	*GSK3B*	.	.	.
14	81660655	A	G	intronic	*GTF2A1*	.	.	.
1	224174699	T	C	ncRNA_intronic	*GTF2IP20*	.	.	.
2	189290727	C	G	intronic	*GULP1*	.	.	.
8	122626460	C	T	exonic	*HAS2*	synonymous_SNV	.	.
6	22708743	T	C	intergenic	*HDGFL1,NRSN1*	.	.	.
4	145570095	A	G	intronic	*HHIP*	.	.	.
6	55594344	C	T	intergenic	*HMGCLL1,BMP5*	.	.	.
8	77245235	G	C	intergenic	*HNF4G,LINC01111*	.	.	.
16	26066789	C	A	intronic	*HS3ST4*	.	.	.
13	97074713	G	A	intronic	*HS6ST3*	.	.	.
11	113783617	G	C	intronic	*HTR3B*	.	.	.
7	46576510	C	G	intergenic	*IGFBP3,TNS3*	.	.	.
8	58055066	C	T	intergenic	*IMPAD1,LINC01606*	.	.	.
13	102344397	G	A	intronic	*ITGBL1*	.	.	.
6	39197278	T	C	upstream	*KCNK5*	.	.	.
7	128547259	C	T	intronic	*KCP*	.	.	.
19	34427510	C	T	intergenic	*KCTD15,LSM14A*	.	.	.
1	245566103	GAAATTACAACTT	G	intronic	*KIF26B*	.	.	.
13	33665614	G	T	intergenic	*KL,STARD13*	.	.	.
3	160221418	T	C	intronic	*KPNA4*	.	.	.
1	32618830	T	G	intronic	*KPNA6*	.	.	.
12	52966184	A	G	intronic	*KRT74*	.	.	.
2	211283902	G	A	ncRNA_intronic	*LANCL1-AS1*	.	.	.
4	18551536	T	C	intergenic	*LCORL,SLIT2*	.	.	.

18	13327010	A	G	intronic	*LDLRAD4*	.	.	.
4	1861020	CTAGGGATCTAGTTAGGGAGGAAGT	C	intergenic	*LETM1,WHSC1*	.	.	.
11	129914611	CAAAAAAATACGAAAACCCGTCAGG	C	intergenic	*LINC00167,APLP2*	.	.	.
21	24047497	C	T	intergenic	*LINC00308,D21S2088E*	.	.	.
13	106488255	G	C	intergenic	*LINC00343,LINC00460*	.	.	.
13	106583577	C	T	intergenic	*LINC00343,LINC00460*	.	.	.
13	71868231	G	A	intergenic	*LINC00348,DACH1*	.	.	.
13	64282646	G	A	ncRNA_intronic	*LINC00395*	.	.	.
9	105659001	G	A	intergenic	*LINC00587,CYLC2*	.	.	.
6	166521542	T	C	intergenic	*LINC00602,T*	.	.	.
3	28619877	CT	C	ncRNA_intronic	*LINC00693*	.	.	.
17	41400791	A	G	intergenic	*LINC00854,LINC00910*	.	.	.
8	127009486	G	T	intergenic	*LINC00861,LOC101927657*	.	.	.
8	127128252	T	C	intergenic	*LINC00861,LOC101927657*	.	.	.
3	94778743	A	T	intergenic	*LINC00879,MTHFD2P1*	.	.	.
11	115975058	C	T	intergenic	*LINC00900,BUD13*	.	.	.
18	75851212	TC	T	intergenic	*LINC01029,SALL3*	.	.	.
5	53741347	G	C	intergenic	*LINC01033,HSPB3*	.	.	.
X	45578785	C	A	intergenic	*LINC01204,LOC392452*	.	.	.
2	242845425	C	T	ncRNA_intronic	*LINC01237*	.	.	.
13	105728357	C	T	intergenic	*LINC01309,DAOA-AS1*	.	.	.
22	49290783	T	C	ncRNA_exonic	*LINC01310*	.	.	.

7	67272404	T	C	intergenic	*LINC01372,LOC102723427*	.	.	.
11	38808722	C	G	intergenic	*LINC01493,LRRC4C*	.	.	.
11	39850589	G	T	intergenic	*LINC01493,LRRC4C*	.	.	.
6	82800810	T	G	intergenic	*LINC01526,IBTK*	.	.	.
6	139941774	C	T	intergenic	*LINC01625,LOC100132735*	.	.	.
18	47118789	C	A	UTR3	*LIPG*	.	.	.
22	47939008	C	A	intergenic	*LL22NC03-75H12.2,LINC00898*	.	.	.
6	140146877	G	A	ncRNA_intronic	*LOC100132735*	.	.	.
5	121622482	C	T	intergenic	*LOC100505841,SNCAIP*	.	.	.
6	10117990	G	T	intergenic	*LOC100506207,TFAP2A*	.	.	.
12	67979220	T	A	intergenic	*LOC100507175,DYRK2*	.	.	.
12	74714846	G	A	intergenic	*LOC100507377,ATXN7L3B*	.	.	.
12	43349721	G	GT	intergenic	*LOC101927058,ADAMTS20*	.	.	.
6	97850738	C	G	ncRNA_intronic	*LOC101927314,MIR548H3*	.	.	.
5	125043634	A	G	intergenic	*LOC101927460,LOC102546228*	.	.	.
X	43081187	C	T	ncRNA_intronic	*LOC101927501*	.	.	.
12	127680890	A	T	intergenic	*LOC101927592,LOC101927616*	.	.	.
6	114952954	A	C	intergenic	*LOC101927768,FRK*	.	.	.
3	20809058	C	A	intergenic	*LOC101927829,VENTXP7*	.	.	.
7	125343290	C	A	intergenic	*LOC101928283,GRM8*	.	.	.

4	34446346	C	T	intergenic	*LOC101928622,ARAP2*	.	.	.
5	86471121	G	C	ncRNA_intronic	*LOC101929380*	.	.	.
5	53999881	A	G	ncRNA_intronic	*LOC102467080*	.	.	.
13	65495619	A	T	intergenic	*LOC102723968,LINC01052*	.	.	.
2	83550257	G	T	intergenic	*LOC1720,FUNDC2P2*	.	.	.
11	134927743	C	T	intergenic	*LOC283177,NONE*	.	.	.
20	38108644	T	C	intergenic	*LOC339568,LINC01370*	.	.	.
21	26491309	C	T	intergenic	*LOC339622,LINC00158*	.	.	.
20	2240959	G	A	intergenic	*LOC388780,TGM3*	.	.	.
4	8973193	C	T	intergenic	*LOC650293,USP17L10*	.	.	.
2	91950430	G	T	intergenic	*LOC654342,GGT8P*	.	.	.
12	57588939	C	A	intronic	*LRP1*	.	.	.
8	105741053	G	A	intergenic	*LRP12,ZFPM2*	.	.	.
2	142114838	T	A	intronic	*LRP1B*	.	.	.
2	143584095	ATATT	A	intergenic	*LRP1B,KYNU*	.	.	.
17	28989790	C	T	intergenic	*LRRC37BP1,SUZ12P1*	.	.	.
11	26168187	A	G	intergenic	*LUZP2,ANO3*	.	.	.
2	99895318	A	G	intergenic	*LYG2,LYG1*	.	.	.
7	2196034	C	T	intronic	*MAD1L1*	.	.	.
6	137065331	TTCACCAATTGTAAC	T	intronic	*MAP3K5*	.	.	.
14	36813719	A	T	intergenic	*MBIP,SFTA3*	.	.	.
3	169470943	T	C	intergenic	*MECOM,TERC*	.	.	.
6	79492845	C	T	intergenic	*MEI4,IRAK1BP1*	.	.	.
X	39162714	C	T	intergenic	*MID1IP1,LINC01281*	.	.	.
6	98950190	C	T	intergenic	*MIR2113,POU3F2*	.	.	.
X	75961939	C	A	ncRNA_intronic	*MIR325HG*	.	.	.
X	121389496	G	A	intergenic	*MIR3672,GRIA3*	.	.	.
4	29181579	G	C	intergenic	*MIR4275,PCDH7*	.	.	.

1	5713019	G	A	intergenic	*MIR4417,MIR4689*	.	.	.
1	55922397	G	A	intergenic	*MIR4422,PLPP3*	.	.	.
2	112120248	A	G	intergenic	*MIR4435-1,MIR4435-2HG*	.	.	.
13	88283672	ATAACTAAAATGTATTAACTTAG	A	ncRNA_intronic	*MIR4500HG*	.	.	.
18	45185629	G	A	intergenic	*MIR4527,SMAD2*	.	.	.
13	66870563	T	C	intergenic	*MIR4704,PCDH9*	.	.	.
13	56350756	G	T	intergenic	*MIR5007,PRR20D*	.	.	.
2	108102695	G	T	intergenic	*MIR548AU,GACAT1*	.	.	.
4	9660835	T	C	intergenic	*MIR548I2,DRD5*	.	.	.
7	79571738	G	T	intergenic	*MIR548M,GNAI1*	.	.	.
21	21847498	T	C	intergenic	*MIR548XHG,LINC00320*	.	.	.
21	21978748	C	G	intergenic	*MIR548XHG,LINC00320*	.	.	.
11	23811642	A	G	intergenic	*MIR8054,LUZP2*	.	.	.
11	29084504	T	C	intergenic	*MIR8068,LINC01616*	.	.	.
18	37319879	G	A	ncRNA_intronic	*MIR924HG*	.	.	.
12	102007104	C	T	intronic	*MYBPC1*	.	.	.
13	109413100	G	A	intronic	*MYO16*	.	.	.
17	21175007	G	T	intergenic	*NATD1,MAP2K3*	.	.	.
21	14348229	G	T	intergenic	*NONE,ANKRD30BP2*	.	.	.
7	113974366	T	C	intergenic	*NONE,FOXP2*	.	.	.
8	47374378	G	T	intergenic	*NONE,LINC00293*	.	.	.
14	19096906	C	G	intergenic	*NONE,OR11H12*	.	.	.
8	32789220	C	T	intergenic	*NRG1,FUT10*	.	.	.
17	44808817	A	G	intronic	*NSF*	.	.	.
12	113392558	G	A	intronic	*OAS3*	.	.	.
11	133268113	C	T	intronic	*OPCML*	.	.	.
11	6898424	C	CCCTCCT	exonic	*OR10A4*	nonframeshift_insertion	.	.
11	48852325	G	A	intergenic	*OR4A47,TRIM49B*	.	.	.
11	4823579	T	G	intergenic	*OR51F1,OR52R1*	.	.	.

5	38698176	G	T	ncRNA_intronic	*OSMR-AS1*	.	.	.
4	109630236	C	T	intergenic	*OSTC,ETNPPL*	.	.	.
11	65957869	C	T	intronic	*PACS1*	.	.	.
9	119021594	T	C	intronic	*PAPPA*	.	.	.
11	66652227	T	C	intronic	*PC*	.	.	.
X	91288646	C	T	intronic	*PCDH11X*	.	.	.
5	140217916	C	G	intronic	*PCDHA1,PCDHA2,PCDHA3,PCDHA4,PCDHA5,PCDHA6,PCDHA7*	.	.	.
21	41294710	G	A	intronic	*PCP4*	.	.	.
5	67843826	T	G	intergenic	*PIK3R1,LOC101928885*	.	.	.
5	68166947	A	C	intergenic	*PIK3R1,LOC101928885*	.	.	.
2	209197143	C	T	intronic	*PIKFYVE*	.	.	.
X	133722486	G	T	intronic	*PLAC1*	.	.	.
3	155405147	C	G	intronic	*PLCH1*	.	.	.
6	161149154	A	G	intronic	*PLG*	.	.	.
7	132226144	G	A	intronic	*PLXNA4*	.	.	.
X	8056847	A	G	intergenic	*PNPLA4,MIR651*	.	.	.
3	157593604	A	G	intergenic	*PQLC2L,SHOX2*	.	.	.
5	126980141	G	A	intergenic	*PRRC1,CTXN3*	.	.	.
19	43430757	C	T	exonic	*PSG7*	stopgain	.	.
14	90728505	A	AG	intronic	*PSMC1*	.	.	.
8	27226971	G	A	intronic	*PTK2B*	.	.	.
20	41408718	T	C	intronic	*PTPRT*	.	.	.
5	36515286	T	G	intergenic	*RANBP3L,SLC1A3*	.	.	.
X	17368090	T	C	intergenic	*REPS2,NHS*	.	.	.
4	40277622	C	T	intergenic	*RHOH,LOC101060498*	.	.	.
5	63770660	T	A	intergenic	*RNF180,RGS7BP*	.	.	.
17	78896742	G	A	intronic	*RPTOR*	.	.	.
21	36192456	C	G	intronic	*RUNX1*	.	.	.
2	167735621	T	C	intergenic	*SCN7A,XIRP2*	.	.	.
5	134042963	G	A	intronic	*SEC24A*	.	.	.
7	85912339	A	G	intergenic	*SEMA3D,GRM3*	.	.	.
7	86111154	G	A	intergenic	*SEMA3D,GRM3*	.	.	.

X	19554173	G	A	UTR3	*SH3KBP1*	.	.	.
6	146295441	GGAGTTTGAGACCAGCCTAGCCAATATGGTGAAAC	G	intergenic	*SHPRH,GRM1*	.	.	.
11	111496801	A	G	intronic	*SIK2*	.	.	.
14	72339340	G	GT	intergenic	*SIPA1L1,RGS6*	.	.	.
7	27028616	C	A	intergenic	*SKAP2,HOXA1*	.	.	.
7	122818812	G	A	intronic	*SLC13A1*	.	.	.
12	61720460	A	G	intergenic	*SLC16A7,FAM19A2*	.	.	.
2	165790151	A	G	intronic	*SLC38A11*	.	.	.
17	42342894	C	T	intronic	*SLC4A1*	.	.	.
2	41756679	C	T	intergenic	*SLC8A1,LOC388942*	.	.	.
9	107160439	A	G	intergenic	*SMC2,OR13F1*	.	.	.
6	168852527	C	T	intronic	*SMOC2*	.	.	.
6	168852528	C	T	intronic	*SMOC2*	.	.	.
1	99290891	C	T	intergenic	*SNX7,PLPPR5*	.	.	.
5	136940063	G	A	intergenic	*SPOCK1,KLHL3*	.	.	.
19	2359246	G	A	intergenic	*SPPL2B,TMPRSS9*	.	.	.
12	121270576	A	G	intronic	*SPPL3*	.	.	.
13	81223125	C	T	intergenic	*SPRY2,LINC00377*	.	.	.
1	121125321	A	G	ncRNA_intronic	*SRGAP2-AS1,SRGAP2D*	.	.	.
12	119468227	T	C	intronic	*SRRM4*	.	.	.
4	27102877	T	C	intergenic	*STIM2,LOC101929199*	.	.	.
8	99929650	C	T	intronic	*STK3*	.	.	.
17	9233789	CCCTGGTGTTAGT	C	intronic	*STX8*	.	.	.
13	37608865	C	T	intronic	*SUPT20H*	.	.	.
14	95927017	C	T	intronic	*SYNE3*	.	.	.
7	105779673	G	A	intergenic	*SYPL1,NAMPT*	.	.	.
2	75344198	G	T	intronic	*TACR1*	.	.	.
1	222754290	T	C	intronic	*TAF1A*	.	.	.

17	27788186	C	A	intronic	*TAOK1*	.	.	.
22	47585172	A	G	intergenic	*TBC1D22A,LL22NC03-75H12.2*	.	.	.
2	21627897	C	T	intergenic	*TDRD15,LOC645949*	.	.	.
2	88553566	T	C	intergenic	*THNSL2,FOXI3*	.	.	.
21	32777505	G	C	intronic	*TIAM1*	.	.	.
19	4831972	G	C	upstream	*TICAM1*	.	.	.
12	129272298	G	A	intergenic	*TMEM132C,SLC15A4*	.	.	.
12	129702651	C	A	intronic	*TMEM132D*	.	.	.
17	32950436	G	A	intronic	*TMEM132E*	.	.	.
6	130852059	T	A	intergenic	*TMEM200A,SMLR1*	.	.	.
7	5349928	T	C	intronic	*TNRC18*	.	.	.
12	73221671	G	C	intergenic	*TRHDE,LOC101928137*	.	.	.
5	136196845	A	G	intergenic	*TRPC7,SPOCK1*	.	.	.
7	120448769	T	C	intronic	*TSPAN12*	.	.	.
22	28854255	C	T	intronic	*TTC28*	.	.	.
4	148150894	T	A	intergenic	*TTC29,EDNRA*	.	.	.
6	158771842	G	A	intronic	*TULP4*	.	.	.
15	41925621	G	C	intergenic	*TYRO3,MGA*	.	.	.
X	143236253	C	A	intergenic	*UBE2NL,SPANXN1*	.	.	.
4	39590980	TACAGGCATGAGCC	T	ncRNA_intronic	*UGDH-AS1*	.	.	.
2	234537786	T	C	intronic	*UGT1A8*	.	.	.
4	69848359	G	A	intergenic	*UGT2A3,UGT2B10*	.	.	.
3	113389871	C	T	intronic	*USF3*	.	.	.
X	129063551	C	CA	exonic	*UTP14A*	frameshift_insertion	.	.
6	145133796	A	G	intronic	*UTRN*	.	.	.
6	153072881	T	C	intronic	*VIP*	.	.	.

2	36935628	G	C	intronic	*VIT*	.	.	.
X	150599452	A	G	intergenic	*VMA21,PASD1*	.	.	.
18	816572	G	A	intergenic	*YES1,ADCYAP1*	.	.	.
2	140986417	A	G	intergenic	*YY1P2,LRP1B*	.	.	.
10	31807860	T	C	intronic	*ZEB1*	.	.	.
X	136745079	AGAAATGT	A	intergenic	*ZIC3,LINC00889*	.	.	.
3	178795032	C	T	intergenic	*ZMAT3,LOC101928739*	.	.	.
19	37450510	A	G	intronic	*ZNF568*	.	.	.
19	57006568	A	G	ncRNA_exonic	*ZNF667-AS1*	.	.	.
7	56015051	C	A	intergenic	*ZNF713,MRPS17*	.	.	.
X	58162885	G	C	intergenic	*ZXDA,NONE*	.	.	.
2	92288350	G	A	intergenic	*ACTR3BP2,NONE*	.	.	.
X	127450874	A	T	intergenic	*ACTRT1,SMARCA1*	.	.	.
6	151649442	G	C	intronic	*AKAP12*	.	.	.
19	40820256	C	G	intergenic	*AKT2,C19orf47*	.	.	.
21	27582592	A	G	intergenic	*APP,CYYR1*	.	.	.
14	32627993	A	C	UTR3	*ARHGAP5*	.	.	.
12	75206877	TA	T	intergenic	*ATXN7L3B,KCNC2*	.	.	.
16	371827	A	G	intronic	*AXIN1*	.	.	.
11	37299303	T	C	intergenic	*C11orf74,LOC103312105*	.	.	.
14	99278814	G	GGGATGGA(	intergenic	*C14orf177,BCL11B*	.	.	.
7	112547936	G	C	intronic	*C7orf60*	.	.	.
18	25675646	C	T	intronic	*CDH2*	.	.	.
8	97753890	C	T	intronic	*CPQ*	.	.	.
13	50562331	GTGTGGTGGTGCGTGCCTGTAATCCCAGCTAC	G	ncRNA_intronic	*DLEU2*	.	.	.
14	19687993	T	C	ncRNA_intronic	*DUXAP10*	.	.	.
1	149756219	G	C	intronic	*FCGR1A,HIST2H2BF*	.	.	.
6	41579732	C	A	intergenic	*FOXP4,MDFI*	.	.	.
18	75385538	G	A	intergenic	*GALR1,LINC01029*	.	.	.
6	26914897	G	A	ncRNA_intronic	*GUSBP2*	.	.	.

2	138959953	C	T	intergenic	*HNMT,SPOPL*	.	.	.
16	26414300	G	A	intergenic	*HS3ST4,C16orf82*	.	.	.
6	77120788	T	G	intergenic	*IMPG1,HTR1B*	.	.	.
14	93572574	C	A	intronic	*ITPK1*	.	.	.
18	39280201	A	G	intergenic	*KC6,PIK3C3*	.	.	.
6	108686378	A	G	intronic	*LACE1*	.	.	.
13	59117917	A	G	intergenic	*LINC00374,DIAPH3*	.	.	.
13	81935507	C	G	intergenic	*LINC00564,NONE*	.	.	.
14	28519888	A	T	intergenic	*LINC00645,FOXG1-AS1*	.	.	.
18	37776118	C	T	intergenic	*LINC01477,KC6*	.	.	.
18	38486990	G	C	intergenic	*LINC01477,KC6*	.	.	.
2	78636949	C	G	intergenic	*LOC101927967,REG3G*	.	.	.
X	5776116	C	G	intergenic	*LOC101928201,NLGN4X*	.	.	.
7	56861185	A	G	intergenic	*LOC101928401,LOC401357*	.	.	.
2	162979577	T	C	ncRNA_intronic	*LOC101929532*	.	.	.
18	70152388	T	TGCCAG	intergenic	*LOC102724913,CBLN2*	.	.	.
11	50771546	C	T	intergenic	*LOC646813,OR4A5*	.	.	.
18	22548016	C	A	intergenic	*LOC729950,ZNF521*	.	.	.
1	74520753	C	A	intronic	*LRRIQ3*	.	.	.
11	25155059	C	T	intergenic	*LUZP2,ANO3*	.	.	.
21	14862772	A	T	intergenic	*MIR3156-3,LOC102724188*	.	.	.
18	36434235	T	C	intergenic	*MIR4318,MIR924HG*	.	.	.
18	36543119	A	G	intergenic	*MIR4318,MIR924HG*	.	.	.
X	145714773	TTGGTAAAGA	T	intergenic	*MIR891A,CXorf51A*	.	.	.
13	21751524	C	G	UTR3	*MRPL57*	.	.	.
20	60780038	C	T	intergenic	*MTG2,HRH3*	.	.	.

4	20148280	A	T	intergenic	*NONE,SLIT2*	.	.	.
22	17042823	G	A	intergenic	*OR11H1,CCT8L2*	.	.	.
22	21178686	TGCAACCGGGAGG	T	exonic	*PI4KA*	nonframeshift_deletion	.	.
7	131268173	T	C	intergenic	*PODXL,LOC101928782*	.	.	.
X	448333	CCTTGTCTGTGGTCTCAGCTACTCAGGGAGGCAGAGGTTGCAGTGAGCTGTGGTCTCAG	C	intergenic	*PPP2R3B,SHOX*	.	.	.
19	43466279	T	G	intergenic	*PSG7,PSG11*	.	.	.
12	57100064	T	G	intergenic	*PTGES3,NACA*	.	.	.
6	13699593	T	C	intronic	*RANBP9*	.	.	.
1	241124461	GA	G	intronic	*RGS7*	.	.	.
6	18461706	C	A	intronic	*RNF144B*	.	.	.
6	71338572	C	A	intergenic	*SDHAF4,SMAP1*	.	.	.
19	4605323	C	CT	intergenic	*SEMA6B,TNFAIP8L1*	.	.	.
6	76362759	C	CT	intronic	*SENP6*	.	.	.
17	28099290	G	A	intronic	*SSH2*	.	.	.
17	35937043	T	A	intronic	*SYNRG*	.	.	.
21	10799220	A	AGGAATGGA	intergenic	*TEKT4P2,TPTE*	.	.	.
21	10647193	G	A	intergenic	*TEKT4P2,TPTE*	.	.	.
13	109108838	G	T	intergenic	*TNFSF13B,MYO16*	.	.	.
19	16189789	C	G	intronic	*TPM4*	.	.	.
21	17373424	T	C	intergenic	*USP25,MIR99AHG*	.	.	.
19	56673607	C	T	intergenic	*ZNF444,GALP*	.	.	.
19	19916422	A	G	intronic	*ZNF506*	.	.	.
19	30983994	C	T	intronic	*ZNF536*	.	.	.
7	149351297	A	AGGAAAAAA	intergenic	*ZNF767P,KRBA1*	.	.	.
22	41917056	A	G	intronic	*ACO2*	.	.	.
17	8095233	TAAA	T	intergenic	*BORCS6,AURKB*	.	.	.
19	13634898	G	GGGAA	intergenic	*CACNA1A,CCDC130*	.	.	.

5	123085598	T	C	intergenic	*CSNK1G3,LINC01170*	.	.	.
1	166385366	G	A	intergenic	*FAM78B,FMO9P*	.	.	.
12	66656807	C	CTTCTCTT	intergenic	*IRAK3,HELB*	.	.	.
20	62931879	C	T	ncRNA_intronic	*LINC00266-1*	.	.	.
13	63646188	A	C	intergenic	*LINC00448,LINC00376*	.	.	.
13	88876751	C	G	ncRNA_intronic	*LOC105370306*	.	.	.
18	3335053	C	G	intergenic	*MYL12B,TGIF1*	.	.	.
1	147999176	ATT	A	intronic	*NBPF8*	.	.	.
15	20381396	C	A	intergenic	*NONE,CHEK2P2*	.	.	.
16	32918393	T	C	intergenic	*SLC6A10P,TP53TG3*	.	.	.
12	21575423	G	T	intergenic	*SLCO1A2,PYROXD1*	.	.	.
4	165284278	T	A	intronic	*43891*	.	.	.
16	18680186	T	C	intergenic	*ABCC6P1,RPS15A*	.	.	.
8	132192942	G	T	intergenic	*ADCY8,EFR3A*	.	.	.
7	31136422	C	A	intronic	*ADCYAP1R1*	.	.	.
4	63131356	A	T	intergenic	*ADGRL3-AS1,TECRL*	.	.	.
4	63362616	T	G	intergenic	*ADGRL3-AS1,TECRL*	.	.	.
10	76502124	T	C	intergenic	*ADK,KAT6B*	.	.	.
10	112936166	G	C	intergenic	*ADRA2A,GPAM*	.	.	.
10	112980490	C	T	intergenic	*ADRA2A,GPAM*	.	.	.
7	15341783	A	T	intronic	*AGMO*	.	.	.
7	15349615	G	GC	intronic	*AGMO*	.	.	.
12	34497859	C	T	intergenic	*ALG10,NONE*	.	.	.
7	118473629	G	T	intergenic	*ANKRD7,LVCAT5*	.	.	.
7	119222840	A	G	intergenic	*ANKRD7,LVCAT5*	.	.	.
5	160345958	C	T	intergenic	*ATP10B,LOC285629*	.	.	.
10	117502871	A	T	intronic	*ATRNL1*	.	.	.
4	123682094	T	G	intergenic	*BBS12,FGF2*	.	.	.
3	166139405	C	G	intergenic	*BCHE,ZBBX*	.	.	.
6	81713235	G	T	intergenic	*BCKDHB,FAM46A*	.	.	.

10	50472608	C	A	intergenic	*C10orf128,C10orf71-AS1*	.	.	.
11	37127234	T	A	intergenic	*C11orf74,LOC103312105*	.	.	.
11	38381592	T	C	intergenic	*C11orf74,LOC103312105*	.	.	.
12	27285438	G	A	intergenic	*C12orf71,STK38L*	.	.	.
4	130537703	C	G	intergenic	*C4orf33,LOC101927282*	.	.	.
5	41281899	T	G	intergenic	*C6,PLCXD3*	.	.	.
6	31079699	G	A	exonic	*C6orf15*	nonsynonymous_SNV	.	.
11	15049464	A	G	intergenic	*CALCA,CALCB*	.	.	.
19	11029991	A	G	intronic	*CARM1*	.	.	.
3	105876518	A	G	intergenic	*CBLB,LINC00882*	.	.	.
2	58116348	C	T	intergenic	*CCDC85A,VRK2*	.	.	.
1	158195495	G	A	intergenic	*CD1D,CD1A*	.	.	.
5	21377743	T	C	intergenic	*CDH18,GUSBP1*	.	.	.
6	21367172	G	A	intergenic	*CDKAL1,LINC00581*	.	.	.
2	120431664	GGCCATTTCCTC	G	intergenic	*CFAP221,TMEM177*	.	.	.
16	11047431	G	A	intronic	*CLEC16A*	.	.	.
16	81520778	G	T	intronic	*CMIP*	.	.	.
8	88654341	C	A	intergenic	*CNBD1,DCAF4L2*	.	.	.
12	41248869	G	T	intronic	*CNTN1*	.	.	.
X	107772828	A	G	intronic	*COL4A5*	.	.	.
9	137611647	G	A	intronic	*COL5A1*	.	.	.
12	39350605	C	T	intergenic	*CPNE8,KIF21A*	.	.	.
8	113940819	A	C	intronic	*CSMD3*	.	.	.
8	115405432	T	A	intergenic	*CSMD3,TRPS1*	.	.	.
X	116534665	T	A	intergenic	*CT83,KLHL13*	.	.	.
4	49149682	TCCATA	T	intergenic	*CWH43,NONE*	.	.	.
4	49169502	G	T	intergenic	*CWH43,NONE*	.	.	.
4	74810871	T	A	intergenic	*CXCL1,PF4*	.	.	.
2	228832032	G	T	intergenic	*DAW1,SPHKAP*	.	.	.
14	73427148	G	A	downstream	*DCAF4*	.	.	.
18	50698952	G	A	intronic	*DCC*	.	.	.

18	51066364	C	A	intergenic	*DCC,LOC102724651*	.	.	.
9	118480825	G	A	intergenic	*DEC1,LOC101928775*	.	.	.
14	55726608	C	A	intergenic	*DLGAP5,FBXO34*	.	.	.
X	31220406	C	G	intronic	*DMD*	.	.	.
1	225224656	G	A	intronic	*DNAH14*	.	.	.
2	196901760	C	A	intronic	*DNAH7*	.	.	.
2	230485241	G	A	intronic	*DNER*	.	.	.
2	117537370	C	A	intergenic	*DPP10,DDX18*	.	.	.
2	117537371	C	A	intergenic	*DPP10,DDX18*	.	.	.
11	65707467	C	A	intergenic	*DRAP1,TSGA10IP*	.	.	.
14	19720640	G	A	intergenic	*DUXAP10,LINC01296*	.	.	.
5	85230414	G	A	intergenic	*EDIL3,NBPF22P*	.	.	.
17	13389882	G	A	intergenic	*ELAC2,HS3ST3A1*	.	.	.
16	33803930	G	T	intergenic	*ENPP7P13,LINC00273*	.	.	.
X	93607722	T	A	intergenic	*FAM133A,BRDTP1*	.	.	.
X	93851290	TA	T	intergenic	*FAM133A,BRDTP1*	.	.	.
13	107965839	A	G	intronic	*FAM155A*	.	.	.
14	40130674	C	T	intergenic	*FBXO33,LOC644919*	.	.	.
8	125091603	T	C	ncRNA_intronic	*FER1L6-AS2*	.	.	.
10	13961338	C	A	intronic	*FRMD4A*	.	.	.
4	163469969	T	C	intergenic	*FSTL5,MIR4454*	.	.	.
4	45185371	G	C	intergenic	*GNPDA2,GABRG1*	.	.	.
4	174687386	G	A	intergenic	*HAND2-AS1,LOC101928509*	.	.	.
1	210806443	G	A	intronic	*HHAT*	.	.	.
8	76543513	T	C	intergenic	*HNF4G,LINC01111*	.	.	.
2	139193071	C	G	intergenic	*HNMT,SPOPL*	.	.	.
X	104694947	G	T	intronic	*IL1RAPL2*	.	.	.
8	111261670	G	A	intergenic	*KCNV1,LINC01608*	.	.	.
9	7344083	G	T	intergenic	*KDM4C,TMEM261*	.	.	.
X	86895217	A	G	intronic	*KLHL4*	.	.	.
12	28011473	A	ATATTATTAT	intergenic	*KLHL42,PTHLH*	.	.	.

12	25398284	C	A	exonic	*KRAS*	nonsynonymous_SNV	Pathogenic|F	Carcinoma_of
22	34664723	G	T	intergenic	*LARGE,ISX*	.	.	.
9	105300085	G	A	ncRNA_intronic	*LINC00587*	.	.	.
3	13836441	C	T	intergenic	*LINC00620,WNT7A*	.	.	.
8	12754255	T	C	intergenic	*LINC00681,KIAA1456*	.	.	.
3	26656893	G	C	intergenic	*LINC00692,LRRC3B*	.	.	.
10	2676259	GCACA	G	intergenic	*LINC00701,PFKP*	.	.	.

3	94776838	G	T	intergenic	*LINC00879,MTHFD2P1*	.	.	.
3	94953881	C	T	intergenic	*LINC00879,MTHFD2P1*	.	.	.
8	67217327	G	T	intergenic	*LINC00967,RRS1-AS1*	.	.	.
7	150141709	C	T	ncRNA_intronic	*LINC00996*	.	.	.
5	27619393	C	T	intergenic	*LINC01021,LSP1P3*	.	.	.
4	166789715	A	C	intergenic	*LINC01179,TLL1*	.	.	.
15	21347115	A	T	intergenic	*LINC01193,LOC646214*	.	.	.
1	199054225	C	A	intergenic	*LINC01221,NR5A2*	.	.	.
2	4779313	G	T	intergenic	*LINC01249,LINC01248*	.	.	.
22	49648435	G	A	intergenic	*LINC01310,NONE*	.	.	.
2	34771415	T	G	intergenic	*LINC01317,LINC01320*	.	.	.
5	97394116	G	T	intergenic	*LINC01340,RGMB*	.	.	.
1	101156694	G	A	intergenic	*LINC01349,VCAM1*	.	.	.
8	84778009	C	G	intergenic	*LINC01419,RALYL*	.	.	.
X	18040638	G	C	ncRNA_intronic	*LINC01456*	.	.	.
11	39838544	A	T	intergenic	*LINC01493,LRRC4C*	.	.	.
14	29516745	G	A	intergenic	*LINC01551,MIR548AI*	.	.	.
1	238454898	C	T	intergenic	*LOC100130331,LINC01139*	.	.	.
5	175550370	G	A	ncRNA_intronic	*LOC100507387*	.	.	.
5	125216964	G	A	intergenic	*LOC101927460,LOC102546228*	.	.	.
6	115825083	T	C	intergenic	*LOC101927768,FRK*	.	.	.
12	74089550	G	T	intergenic	*LOC101928137,LOC100507377*	.	.	.
13	112959503	T	C	intergenic	*LOC101928730,LINC01044*	.	.	.

5	30203504	A	T	intergenic	*LOC101929681,CDH6*	.	.	.
5	139153860	G	T	intergenic	*LOC101929696,PSD2*	.	.	.
5	117374120	A	T	ncRNA_intronic	*LOC102467224*	.	.	.
5	71953637	T	C	ncRNA_intronic	*LOC102477328*	.	.	.
16	53416738	A	G	ncRNA_intronic	*LOC102723373*	.	.	.
7	67559826	T	C	intergenic	*LOC102723427,LOC100507468*	.	.	.
8	78395478	G	A	intergenic	*LOC102724874,PKIA*	.	.	.
9	44798500	A	G	intergenic	*LOC103908605,FAM27C*	.	.	.
2	130550878	G	T	intergenic	*LOC151121,LOC389033*	.	.	.
5	177128682	G	A	intergenic	*LOC202181,FAM153A*	.	.	.
5	9649796	C	A	ncRNA_intronic	*LOC285692*	.	.	.
3	18049176	C	A	intronic	*LOC339862*	.	.	.
16	33308279	T	TA	intergenic	*LOC390705,ENPP7P13*	.	.	.
16	32568828	C	A	intergenic	*LOC390705,TP53TG3*	.	.	.
5	18943942	A	T	intergenic	*LOC401177,CDH18*	.	.	.
11	49958132	G	A	intergenic	*LOC440040,OR4C13*	.	.	.
1	191304495	G	T	intergenic	*LOC440704,RGS18*	.	.	.
1	191571685	G	C	intergenic	*LOC440704,RGS18*	.	.	.
2	22443432	A	T	intergenic	*LOC645949,LOC102723362*	.	.	.
11	50428845	C	A	intergenic	*LOC646813,OR4A5*	.	.	.
14	43534552	C	A	intergenic	*LRFN5,FSCB*	.	.	.
20	15216305	T	C	intronic	*MACROD2*	.	.	.
20	15810190	T	C	intronic	*MACROD2*	.	.	.

7	78318604	C	T	intronic	*MAGI2*	.	.	.
7	78603387	G	C	intronic	*MAGI2*	.	.	.
6	78924092	T	A	intergenic	*MEI4,IRAK1BP1*	.	.	.
X	10836311	T	A	intronic	*MID1*	.	.	.
X	63324324	T	A	intergenic	*MIR1468,AMER1*	.	.	.
4	117395801	C	T	intergenic	*MIR1973,TRAM1L1*	.	.	.
X	76178686	G	A	ncRNA_intronic	*MIR325HG*	.	.	.
1	37737277	G	T	intergenic	*MIR4255,LINC01137*	.	.	.
4	29322485	G	A	intergenic	*MIR4275,PCDH7*	.	.	.
4	29400128	G	A	intergenic	*MIR4275,PCDH7*	.	.	.
2	89132435	G	T	intergenic	*MIR4436A,NONE*	.	.	.
13	57546217	T	A	intergenic	*MIR5007,PRR20D*	.	.	.
1	235745499	C	A	ncRNA_intronic	*MIR5096*	.	.	.
14	30119002	C	G	ncRNA_intronic	*MIR548AI*	.	.	.
10	122515657	G	T	ncRNA_intronic	*MIR5694*	.	.	.
X	63799058	GT	G	intergenic	*MTMR8,ZC4H2*	.	.	.
13	77985558	C	A	intergenic	*MYCBP2,SCEL*	.	.	.
3	175316908	G	A	intronic	*NAALADL2*	.	.	.
11	19510598	C	A	intronic	*NAV2*	.	.	.
11	21279319	G	A	intronic	*NELL1*	.	.	.
X	95584496	C	A	intergenic	*NONE,BRDTP1*	.	.	.
8	47195346	G	T	intergenic	*NONE,LINC00293*	.	.	.
8	47667717	G	T	intergenic	*NONE,LINC00293*	.	.	.
16	16933757	A	G	intergenic	*NPIPA8,XYLT1*	.	.	.
16	29431575	T	C	intergenic	*NPIPB11,LOC606724*	.	.	.
10	84866750	C	A	intergenic	*NRG3,GHITM*	.	.	.
14	79895828	G	A	intronic	*NRXN3*	.	.	.
8	126225949	C	CCCTTCCTTC	intronic	*NSMCE2*	.	.	.
11	114485341	T	C	intergenic	*NXPE4,NXPE2*	.	.	.
14	20353095	G	A	intergenic	*OR4K2,OR4K5*	.	.	.
11	93990440	G	A	intergenic	*PANX1,IZUMO1R*	.	.	.
13	58246026	C	T	intronic	*PCDH17*	.	.	.
8	52951028	A	T	intergenic	*PCMTD1,ST18*	.	.	.
2	178827975	C	A	intronic	*PDE11A*	.	.	.
10	60943664	T	A	intronic	*PHYHIPL*	.	.	.

17	7429212	T	C	intergenic	*POLR2A,TNFSF12*	.	.	.
6	57550920	G	T	intergenic	*PRIM2,GUSBP4*	.	.	.
6	57762167	T	C	intergenic	*PRIM2,GUSBP4*	.	.	.
1	214091033	G	A	ncRNA_intronic	*PROX1-AS1*	.	.	.
5	120530216	T	G	intergenic	*PRR16,LOC102467226*	.	.	.
X	126009839	G	T	intergenic	*PRR32,ACTRT1*	.	.	.
5	105946439	A	C	intergenic	*RAB9BP1,LOC102467213*	.	.	.
8	85749414	G	C	intronic	*RALYL*	.	.	.
4	161399860	C	T	intergenic	*RAPGEF2,FSTL5*	.	.	.
7	102153577	G	C	intronic	*RASA4B*	.	.	.
X	16911923	G	A	intergenic	*RBBP7,REPS2*	.	.	.
16	7413597	C	A	intronic	*RBFOX1*	.	.	.
7	103542112	G	C	intronic	*RELN*	.	.	.
1	192865128	G	A	intergenic	*RGS2,LINC01032*	.	.	.
18	40374507	G	A	intronic	*RIT2*	.	.	.
3	80948821	C	A	intergenic	*ROBO1,LOC728290*	.	.	.
X	83314870	A	T	UTR3	*RPS6KA6*	.	.	.
6	7207289	G	C	intronic	*RREB1*	.	.	.
8	145573612	C	T	intergenic	*SCRT1,TMEM249*	.	.	.
10	99575283	C	T	intergenic	*SFRP5,LINC00866*	.	.	.
18	43081309	A	C	ncRNA_intronic	*SLC14A2-AS1*	.	.	.
6	118505587	CCTT	C	intronic	*SLC35F1*	.	.	.
11	26691132	G	A	UTR3	*SLC5A12*	.	.	.
18	46540673	G	A	intergenic	*SMAD7,DYM*	.	.	.
6	86885361	C	T	intergenic	*SNHG5,HTR1E*	.	.	.
2	1415518	C	A	intergenic	*SNTG2,TPO*	.	.	.
12	23744510	G	T	intronic	*SOX5*	.	.	.
X	142311180	G	A	intergenic	*SPANXN4,SPANXN3*	.	.	.
4	177598293	A	G	intergenic	*SPCS3,VEGFC*	.	.	.
2	45640614	G	A	intronic	*SRBD1*	.	.	.
7	104806268	C	T	intronic	*SRPK2*	.	.	.
X	7437430	A	T	intergenic	*STS,VCX*	.	.	.
14	26621672	A	G	intergenic	*STXBP6,NOVA1*	.	.	.
12	79603397	G	T	intronic	*SYT1*	.	.	.

12	33638379	G	T	intergenic	*SYT10,ALG10*	.	.	.
11	120933960	C	G	intronic	*TBCEL*	.	.	.
8	67866485	T	TG	intronic	*TCF24*	.	.	.
21	10587580	A	G	intergenic	*TEKT4P2,TPTE*	.	.	.
21	10855889	A	AATGGAATG	intergenic	*TEKT4P2,TPTE*	.	.	.
5	167075540	G	A	intronic	*TENM2*	.	.	.
11	79901308	A	G	intergenic	*TENM4,LOC101928944*	.	.	.
6	51143642	C	A	intergenic	*TFAP2B,PKHD1*	.	.	.
6	130956547	G	T	intergenic	*TMEM200A,SMLR1*	.	.	.
11	57650490	A	T	intergenic	*TMX2-CTNND1,OR9Q1*	.	.	.
3	189303242	C	G	intergenic	*TPRG1,TP63*	.	.	.
12	72773801	C	A	intronic	*TRHDE*	.	.	.
2	96250137	C	A	intergenic	*TRIM43B,TRIM43*	.	.	.
12	71829785	C	A	intergenic	*TSPAN8,LGR5*	.	.	.
21	38567571	G	A	intronic	*TTC3*	.	.	.
6	80726078	C	T	intronic	*TTK*	.	.	.
19	30595593	C	A	intergenic	*URI1,ZNF536*	.	.	.
X	27188002	C	A	intergenic	*VENTXP1,PPP4R3CP*	.	.	.
X	65280181	C	A	intergenic	*VSIG4,HEPH*	.	.	.
X	113706381	C	A	intergenic	*XACT,HTR2C*	.	.	.
16	17572828	G	A	intergenic	*XYLT1,NPIPA8*	.	.	.
16	17768184	A	T	intergenic	*XYLT1,NPIPA8*	.	.	.
X	74789430	C	T	intergenic	*ZDHHC15,TTC3P1*	.	.	.
1	247352420	G	T	intergenic	*ZNF124,MIR3916*	.	.	.
10	38198776	A	T	intergenic	*ZNF248,ZNF25*	.	.	.
7	57639284	C	T	intergenic	*ZNF716,NONE*	.	.	.
19	24159816	C	A	intergenic	*ZNF726,ZNF254*	.	.	.
19	22530979	G	T	intergenic	*ZNF729,ZNF98*	.	.	.
3	102953797	C	T	intergenic	*ZPLD1,MIR548AB*	.	.	.
5	10386784	A	C	intronic	*43896*	.	.	.
4	7996058	G	A	intronic	*ABLIM2*	.	.	.
7	152978598	C	A	intergenic	*ACTR3B,LINC01287*	.	.	.

X	127475508	G	A	intergenic	*ACTRT1,SMARCA1*	.	.	.
4	100272510	G	T	intronic	*ADH1C*	.	.	.
8	26671462	C	A	intronic	*ADRA1A*	.	.	.
6	151642796	T	G	intronic	*AKAP12*	.	.	.
18	21183400	C	A	intronic	*ANKRD29*	.	.	.
18	14798178	TCAC	T	intronic	*ANKRD30B*	.	.	.
21	14656985	A	C	intergenic	*ANKRD30BP2,MIR3156-3*	.	.	.
X	11281180	C	A	intronic	*ARHGAP6*	.	.	.
4	42660359	G	C	intergenic	*ATP8A1,GRXCR1*	.	.	.
7	97918799	C	T	intronic	*BRI3*	.	.	.
14	58531629	T	C	intronic	*C14orf37*	.	.	.
19	30193138	C	G	UTR3	*C19orf12*	.	.	.
5	3064991	C	A	intergenic	*C5orf38,LINC01377*	.	.	.
17	58252086	G	A	intergenic	*CA4,USP32*	.	.	.
6	92950966	C	T	intergenic	*CASC6,EPHA7*	.	.	.
X	42499310	C	A	intergenic	*CASK,PPP1R2P9*	.	.	.
11	23140553	G	GTA	intergenic	*CCDC179,MIR8054*	.	.	.
2	57529444	C	G	intergenic	*CCDC85A,VRK2*	.	.	.
2	57870258	C	CT	intergenic	*CCDC85A,VRK2*	.	.	.
12	97253361	T	A	intronic	*CFAP54*	.	.	.
X	72860685	G	T	intronic	*CHIC1*	.	.	.
5	130035085	A	G	intergenic	*CHSY3,HINT1*	.	.	.
14	38898804	A	G	intergenic	*CLEC14A,LINC00639*	.	.	.
7	52016179	C	T	intergenic	*COBL,POM121L12*	.	.	.
8	140462634	G	A	intergenic	*COL22A1,KCNK9*	.	.	.
8	114871668	T	A	intergenic	*CSMD3,TRPS1*	.	.	.
2	81143092	A	T	intergenic	*CTNNA2,LOC100507201*	.	.	.
5	11763499	C	A	intronic	*CTNND2*	.	.	.
4	49264297	A	T	intergenic	*CWH43,NONE*	.	.	.
4	49646837	G	GA	intergenic	*CWH43,NONE*	.	.	.
4	74810406	C	T	intergenic	*CXCL1,PF4*	.	.	.

5	39496862	G	C	intergenic	*DAB2,LOC101926940*	.	.	.
18	49915913	G	C	intronic	*DCC*	.	.	.
X	134710010	G	T	intronic	*DDX26B*	.	.	.
14	80787196	A	C	ncRNA_intronic	*DIO2-AS1*	.	.	.
14	19812998	G	T	intergenic	*DUXAP10,LINC01296*	.	.	.
16	33734559	TAAAATAAAATAAAATAAAATAAAATAAA	T	intergenic	*ENPP7P13,LINC00273*	.	.	.
1	6506201	C	T	intronic	*ESPN*	.	.	.
1	157219251	C	G	intergenic	*ETV3,FCRL5*	.	.	.
22	48908767	G	C	intronic	*FAM19A5*	.	.	.
9	40607397	C	T	intergenic	*FAM74A1,SPATA31A3*	.	.	.
16	86741639	T	C	intergenic	*FOXL1,LOC440390*	.	.	.
1	74797192	T	C	exonic	*FPGT-TNNI3K,TNNI3K*	nonsynonymous_SNV	.	.
14	45341303	A	G	intergenic	*FSCB,C14orf28*	.	.	.
6	96540851	C	G	intronic	*FUT9*	.	.	.
5	161044530	A	AT	intergenic	*GABRB2,GABRA6*	.	.	.
4	46061466	C	T	intronic	*GABRG1*	.	.	.
5	161627880	T	A	intergenic	*GABRG2,CCNG1*	.	.	.
X	49383191	C	T	intergenic	*GAGE1,PAGE1*	.	.	.
18	29871087	T	G	intronic	*GAREM1*	.	.	.
15	75613411	G	C	intergenic	*GOLGA6D,COMMD4*	.	.	.

13	94175371	TATATGTACCCATATATACATATACACAATATTATCCATATATGTACATATATACACATACATATACACATATATGGATAATATTGTGTATATGTATATATGGGTACATATATACAC	T	intronic	*GPC6*	.	.	.
X	136637257	G	C	intergenic	*GPR101,ZIC3*	.	.	.
5	145936249	C	T	intergenic	*GPR151,PPP2R2B*	.	.	.
5	145936250	A	T	intergenic	*GPR151,PPP2R2B*	.	.	.
6	101856251	TGAGA	T	intronic	*GRIK2*	.	.	.
11	120725258	G	A	intronic	*GRIK4*	.	.	.
12	96365714	C	CTTTTTTTTTT	downstream	*HAL*	.	.	.
X	133667895	C	T	intergenic	*HPRT1,MIR450B*	.	.	.
5	51832055	C	T	intergenic	*ISL1,PELO*	.	.	.
8	111652472	G	C	intergenic	*KCNV1,LINC01608*	.	.	.
20	37007728	C	T	intergenic	*LBP,SNHG17*	.	.	.
X	68644066	C	A	intergenic	*LINC00269,FAM155B*	.	.	.
13	88039481	G	A	intergenic	*LINC00430,MIR4500HG*	.	.	.
10	131243780	AG	A	intergenic	*LINC01163,MGMT*	.	.	.
14	83187543	G	A	intergenic	*LINC01467,NONE*	.	.	.
9	83544264	T	TATATAGATA	intergenic	*LINC01507,TLE1*	.	.	.
8	136416580	A	G	intergenic	*LINC01591,KHDRBS3*	.	.	.
8	112949034	A	G	intergenic	*LINC01609,CSMD3*	.	.	.
4	188539843	A	T	ncRNA_intronic	*LOC100506272*	.	.	.

2	81751620	C	A	intergenic	*LOC100507201,LOC1720*	.	.	.
1	61181593	A	T	ncRNA_intronic	*LOC101926964*	.	.	.
1	81218305	G	A	intergenic	*LOC101927412,LOC101927434*	.	.	.
12	126683810	G	C	intergenic	*LOC101927464,LOC100128554*	.	.	.
12	128113991	G	A	intergenic	*LOC101927616,LOC101927637*	.	.	.
21	26116302	C	A	intergenic	*LOC101927869,LOC339622*	.	.	.
12	73563431	G	T	ncRNA_intronic	*LOC101928137*	.	.	.
12	73624764	A	C	intergenic	*LOC101928137,LOC100507377*	.	.	.
15	45641827	GAAAGAAAAGA	G	intergenic	*LOC101928414,GATM*	.	.	.
5	29980470	C	G	intergenic	*LOC101929681,CDH6*	.	.	.
18	12317	G	T	ncRNA_exonic	*LOC102723376*	.	.	.
7	68062471	C	T	intergenic	*LOC102723427,LOC100507468*	.	.	.
7	68459899	T	C	intergenic	*LOC102723427,LOC100507468*	.	.	.
4	31985034	C	A	intergenic	*LOC102723778,LOC102723828*	.	.	.
13	65084878	C	T	intergenic	*LOC102723968,LINC01052*	.	.	.
13	65904126	A	T	intergenic	*LOC102723968,LINC01052*	.	.	.
13	66349513	T	G	intergenic	*LOC102723968,LINC01052*	.	.	.
8	64813401	C	T	ncRNA_intronic	*LOC102724623*	.	.	.
22	48206212	G	T	ncRNA_intronic	*LOC284930*	.	.	.
22	48486852	G	A	intergenic	*LOC284930,MIR3201*	.	.	.
X	3943595	C	A	intergenic	*LOC389906,LOC101928201*	.	.	.

7	56925181	A	T	intergenic	*LOC401357,LOC100130849*	.	.	.
6	161002160	T	A	intronic	*LPA*	.	.	.
6	161002161	A	T	intronic	*LPA*	.	.	.
11	40529051	G	C	intronic	*LRRC4C*	.	.	.
7	16585399	A	T	intronic	*LRRC72*	.	.	.
6	6353797	G	A	ncRNA_intronic	*LY86-AS1*	.	.	.
20	14048258	T	C	intronic	*MACROD2*	.	.	.
X	142020341	G	T	intergenic	*MAGEC2,SPANXN4*	.	.	.
7	78603629	A	T	intronic	*MAGI2*	.	.	.
17	12418906	A	G	intergenic	*MAP2K4,LINC00670*	.	.	.
14	55571231	A	G	intergenic	*MAPK1IP1L,LGALS3*	.	.	.
8	6531122	G	T	ncRNA_intronic	*MCPH1-AS1*	.	.	.
17	38188097	G	A	intronic	*MED24*	.	.	.
12	95876500	G	A	intronic	*METAP2*	.	.	.
3	158609540	G	GTATATATAT	intergenic	*MFSD1,IQCJ*	.	.	.
6	156340041	G	T	intergenic	*MIR1202,ARID1B*	.	.	.
22	48797781	C	T	intergenic	*MIR3201,FAM19A5*	.	.	.
5	7175455	C	A	intergenic	*MIR4278,MIR4454*	.	.	.
7	63365228	T	C	intergenic	*MIR4283-2,LINC01005*	.	.	.
18	36394084	A	G	intergenic	*MIR4318,MIR924HG*	.	.	.
2	53198793	C	A	intergenic	*MIR4431,ASB3*	.	.	.
X	92418999	T	C	intergenic	*MIR4454,NAP1L3*	.	.	.
13	55920154	TA	T	intergenic	*MIR5007,PRR20D*	.	.	.
13	55920156	G	C	intergenic	*MIR5007,PRR20D*	.	.	.
X	146456070	C	A	intergenic	*MIR514A2,FMR1-AS1*	.	.	.
21	21773313	G	T	intergenic	*MIR548XHG,LINC00320*	.	.	.
16	5934432	T	A	intergenic	*MIR8065,RBFOX1*	.	.	.
8	89713980	A	G	intergenic	*MMP16,LOC101929709*	.	.	.

8	142709943	G	A	intergenic	*MROH5,MIR1302-7*	.	.	.
1	145208188	A	AGTT	intronic	*NBPF20,NBPF9*	.	.	.
1	73131131	C	A	intergenic	*NEGR1,LINC01360*	.	.	.
11	98462074	C	T	intergenic	*NONE,CNTN5*	.	.	.
11	98720962	G	T	intergenic	*NONE,CNTN5*	.	.	.
14	84354457	C	T	intergenic	*NONE,LINC00911*	.	.	.
20	29420633	G	T	intergenic	*NONE,LINC01598*	.	.	.
2	91781247	G	T	intergenic	*NONE,LOC654342*	.	.	.
4	20188388	C	G	intergenic	*NONE,SLIT2*	.	.	.
16	16784192	A	G	intergenic	*NPIPA8,XYLT1*	.	.	.
7	34770773	G	A	ncRNA_intronic	*NPSR1-AS1*	.	.	.
11	114404365	G	A	intronic	*NXPE1*	.	.	.
13	97669549	T	A	intergenic	*OXGR1,LINC00456*	.	.	.
11	93997282	C	T	intergenic	*PANX1,IZUMO1R*	.	.	.
11	74042401	G	A	UTR3	*PGM2L1*	.	.	.
2	230035357	T	TA	intronic	*PID1*	.	.	.
8	43821536	C	A	intergenic	*POTEA,NONE*	.	.	.
8	43821537	A	G	intergenic	*POTEA,NONE*	.	.	.
X	18883129	G	A	intergenic	*PPEF1,PHKA2-AS1*	.	.	.
X	42977088	T	A	intergenic	*PPP1R2P9,LOC101927501*	.	.	.
5	146468427	G	C	intergenic	*PPP2R2B,STK32A*	.	.	.
2	231958654	A	G	intronic	*PSMD1*	.	.	.
4	161138573	T	G	intergenic	*RAPGEF2,FSTL5*	.	.	.
16	6781996	A	AT	intronic	*RBFOX1*	.	.	.
12	114427360	A	G	intergenic	*RBM19,TBX5*	.	.	.
2	238740878	G	T	intronic	*RBM44*	.	.	.
X	17346632	TA	T	intergenic	*REPS2,NHS*	.	.	.
19	51960818	G	T	exonic	*SIGLEC8*	synonymous_SNV	.	.
19	18017179	C	T	intergenic	*SLC5A5,CCDC124*	.	.	.
13	88337249	G	C	intergenic	*SLITRK5,LINC00397*	.	.	.
13	86516306	G	A	intergenic	*SLITRK6,LINC00430*	.	.	.

6	169398101	T	A	intergenic	*SMOC2,LINC01615*	.	.	.
X	142848871	G	C	intergenic	*SPANXN2,UBE2NL*	.	.	.
4	168961505	G	A	intergenic	*SPOCK3,ANXA10*	.	.	.
17	40441546	G	C	exonic	*STAT5A*	nonsynonymous_SNV	.	.
6	147331469	T	G	ncRNA_intronic	*STXBP5-AS1*	.	.	.
12	119054698	C	T	intergenic	*SUDS3,SRRM4*	.	.	.
X	77516666	G	T	intergenic	*TAF9B,CYSLTR1*	.	.	.
21	10768277	TG	T	intergenic	*TEKT4P2,TPTE*	.	.	.
2	71247540	C	A	intergenic	*TEX261,OR7E91P*	.	.	.
6	50711205	C	T	intronic	*TFAP2D*	.	.	.
12	126424422	C	T	intergenic	*TMEM132B,LINC00939*	.	.	.
13	109134615	G	A	intergenic	*TNFSF13B,MYO16*	.	.	.
6	94499619	C	A	intergenic	*TSG1,MANEA-AS1*	.	.	.
2	123120130	C	T	intergenic	*TSN,CNTNAP5*	.	.	.
8	33391292	G	C	intergenic	*TTI2,RNF122*	.	.	.
Y	28582695	T	C	intergenic	*TTTY3,NONE*	.	.	.
7	1357592	G	A	intergenic	*UNCX,MICALL2*	.	.	.
6	144980804	C	T	intronic	*UTRN*	.	.	.
6	145485556	A	G	intergenic	*UTRN,EPM2A*	.	.	.
1	168637724	C	A	intergenic	*XCL1,DPT*	.	.	.
X	98902237	C	A	ncRNA_intronic	*XRCC6P5*	.	.	.
19	23159246	G	T	exonic	*ZNF728*	nonsynonymous_SNV	.	.
7	149545300	C	T	exonic	*ZNF862*	nonsynonymous_SNV	.	.
7	76077015	G	A	intergenic	*ZP3,DTX2*	.	.	.
1	142788944	G	C	intergenic	*ANKRD20A12P,LOC102723769*	.	.	.
9	42432552	TTAGCCTCCTGAG	T	intergenic	*ANKRD20A3,FAM95B1*	.	.	.
9	69494087	T	TA	intergenic	*ANKRD20A4,LOC100133920*	.	.	.
21	27661345	G	GTATCTATC	intergenic	*APP,CYYR1*	.	.	.
17	42257034	A	G	ncRNA_intronic	*ASB16-AS1*	.	.	.

2	10411766	C	CGGACACAT	intergenic	*C2orf48,HPCAL1*	.	.	.
12	29032237	G	GAGAAAGAA	intergenic	*CCDC91,FAR2*	.	.	.
18	25915896	C	G	intergenic	*CDH2,MIR302F*	.	.	.
8	5972801	A	AATTTT	intergenic	*CSMD1,LOC100287015*	.	.	.
4	191028023	T	C	intergenic	*DBET,NONE*	.	.	.
9	1920158	CTGTG	C	intergenic	*DMRT2,SMARCA2*	.	.	.
8	34228852	ATGTG	A	intergenic	*DUSP26,LINC01288*	.	.	.
9	38829898	TG	T	intergenic	*FAM201A,CNTNAP3*	.	.	.
9	46209753	G	A	intergenic	*FAM27E2,FAM27E2*	.	.	.
6	99809354	G	GTC	intergenic	*FAXC,COQ3*	.	.	.
1	166680873	T	A	intergenic	*FMO9P,POGK*	.	.	.
10	135518436	T	C	intergenic	*FRG2B,NONE*	.	.	.
5	156471622	GAAAGAGAAAGAAAGAAAGAAAGAA	G	intronic	*HAVCR1*	.	.	.
19	24619313	A	AGT	intergenic	*HAVCR1P1,NONE*	.	.	.
19	24619315	CAT	C	intergenic	*HAVCR1P1,NONE*	.	.	.
10	6127683	AAAAGAAAGAAAGAAAGAAAG	A	intergenic	*IL2RA,RBM17*	.	.	.
18	39451021	A	G	intergenic	*KC6,PIK3C3*	.	.	.
2	8271286	A	AAAG	ncRNA_intronic	*LINC00299*	.	.	.
13	89702692	C	G	intergenic	*LINC00433,LINC01047*	.	.	.
10	38892392	A	G	intergenic	*LINC00999,ACTR3BP5*	.	.	.
1	239462709	T	G	intergenic	*LINC01139,CHRM3*	.	.	.
1	88807840	C	T	intergenic	*LINC01364,PKN2-AS1*	.	.	.
21	25709103	T	A	intergenic	*LOC101927869,LOC339622*	.	.	.
7	124929106	G	GATACC	ncRNA_intronic	*LOC101928283*	.	.	.

5	95571815	GTCTATCTATCTATCTC	G	ncRNA_intronic	*LOC101929710*	.	.	.
1	143521424	C	T	intergenic	*LOC102723769,MIR6077*	.	.	.
16	33330462	TGTGGCTCACTCACTCACAGTGGGACACG	T	intergenic	*LOC390705,ENPP7P13*	.	.	.
4	9652046	T	A	intergenic	*MIR548I2,DRD5*	.	.	.
4	9662805	T	C	intergenic	*MIR548I2,DRD5*	.	.	.
4	9663027	A	G	intergenic	*MIR548I2,DRD5*	.	.	.
17	22260162	C	A	intergenic	*MTRNR2L1,NONE*	.	.	.
Y	13447881	T	TATTCCATTC	intergenic	*NONE,GYG2P1*	.	.	.
Y	58972876	G	GTCCAT	intergenic	*NONE,SPRY3*	.	.	.
7	144042114	CATAGATAGATAG	C	ncRNA_intronic	*OR2A1-AS1*	.	.	.
3	183588060	T	TCC	intronic	*PARL*	.	.	.
1	66920064	T	TCACA	intergenic	*PDE4B,SGIP1*	.	.	.
X	126521235	A	G	intergenic	*PRR32,ACTRT1*	.	.	.
19	55729171	C	A	intergenic	*PTPRH,TMEM86B*	.	.	.
13	87320568	A	C	intergenic	*SLITRK6,LINC00430*	.	.	.
13	33763507	AGGAAGGGAACGACAAACCAGAGAGTCCCTTCCCTTTCCTCCTGCTTTCCCTCCTTCCTCCTTTCGGAGGAAGGAGGGAAAGCAGGAGGAAAG	A	intronic	*STARD13*	.	.	.
17	34758502	GGCTGGC	G	intronic	*TBC1D3F*	.	.	.

21	10214733	TGTAAAAATTTGAAATCATAATTATGCTA	T	intergenic	*TEKT4P2,TPTE*	.	.	.
2	135587351	A	G	intergenic	*TMEM163,ACMSD*	.	.	.
2	170957632	C	T	intergenic	*UBR3,MYO3B*	.	.	.
2	114342554	A	T	ncRNA_intronic	*WASH2P*	.	.	.
6	157905300	TGTATATATACGTATATATATATGTGTATATATATATACATATATATACACATATATATACGTATATATACATTATAC	T	intronic	*ZDHHC14*	.	.	.
20	32323885	ATCTGAACTGTTTCCAGTTTTTACTATTTTTTTTTTTAGACCCTGTCTCAAAAAAAAAAAAAAGTAAAAACTGGAAACAGTTCAGATGTTCT	A	intronic	*ZNF341*	.	.	.

7	57716531	AGTTAAGCAGCTGAAGGTTAAAAAAAATCAAAGCTCCCCAAATATGGGTATGACAGCTGATCCAACCTCACCAT	A	intergenic	*ZNF716,NONE*	.	.	.
2	186327380	TATATATATATATTA	T	intergenic	*ZNF804A,LOC101927196*	.	.	.
16	20567517	A	C	intronic	*ACSM2B*	.	.	.
4	22551798	A	AATTTT	intergenic	*ADGRA3,GBA3*	.	.	.
10	51753896	A	G	intronic	*AGAP6*	.	.	.
X	1728625	GTGTCCCCTACTCCAGCCAAAGCTCCAGGCCGCCCCTGCCCCTACTCCAGCCAAAGCTCCAGGCCACCCC	G	intronic	*ASMT*	.	.	.
21	35303870	C	A	intergenic	*ATP5O,LINC00649*	.	.	.
2	132764815	A	G	intergenic	*C2orf27B,ANKRD30BL*	.	.	.
3	55172620	C	T	intergenic	*CACNA2D3,WNT5A*	.	.	.
9	70487501	T	A	intronic	*CBWD5*	.	.	.
5	21272179	G	A	intergenic	*CDH18,GUSBP1*	.	.	.
7	90572475	A	C	intronic	*CDK14*	.	.	.
7	43684392	G	GAC	intronic	*COA1*	.	.	.
5	64266046	T	C	intronic	*CWC27*	.	.	.
1	47385132	T	TAC	intergenic	*CYP4Z2P,CYP4A11*	.	.	.
X	85915371	T	C	intronic	*DACH2*	.	.	.
2	159704216	A	G	intergenic	*DAPL1,TANC1*	.	.	.

X	96218281	C	T	intronic	*DIAPH2*	.	.	.
11	83303036	G	A	intronic	*DLG2*	.	.	.
22	45716206	GGTGGCGCAGGCCTGTGCCACTGCACTCCAGCCTGGTGACAAAGCGAGACTCCATCAAAAAAAAACAAACAAACACGTAATTTTTCTTTTTCTTTTCTTCCTTCTTCTTCTTCTTTTTTTTTTTCTTTTTAAGACAGGGTCTCACTCTGCGACCCAGGCTGGAGTGCA	G	intronic	*FAM118A*	.	.	.
5	74285964	TAAAAAA	T	intergenic	*FAM169A,GCNT4*	.	.	.
9	38829900	TTTTTTG	T	intergenic	*FAM201A,CNTNAP3*	.	.	.
1	143920415	G	T	intergenic	*FAM72C,NBPF20*	.	.	.
1	120933972	C	G	intronic	*FCGR1B*	.	.	.
X	138009333	C	T	intronic	*FGF13*	.	.	.
X	151397599	T	A	intronic	*GABRA3*	.	.	.
X	151491393	C	T	intronic	*GABRA3*	.	.	.
16	20202822	A	AAG	intergenic	*GPR139,GP2*	.	.	.
X	122341144	A	G	intronic	*GRIA3*	.	.	.
5	70597060	A	AAG	intergenic	*GUSBP9,PMCHL2*	.	.	.
12	48726013	C	A	intergenic	*H1FNT,ZNF641*	.	.	.
X	131952698	G	A	intronic	*HS6ST2*	.	.	.
5	144530501	C	T	intergenic	*KCTD16,PRELID2*	.	.	.
6	74052026	G	GAGGGGAG	intergenic	*KHDC1,DPPA5*	.	.	.

9	111103207	TACAC	T	intergenic	*KLF4,ACTL7B*	.	.	.
7	152104490	C	T	intronic	*KMT2C*	.	.	.
13	35304859	GTATA	G	intergenic	*LINC00457,NBEA*	.	.	.
10	4735264	A	AATATAT	intergenic	*LINC00704,AKR1E2*	.	.	.
1	199560665	T	C	intergenic	*LINC01221,NR5A2*	.	.	.
8	70157409	G	A	intergenic	*LINC01592,LINC01603*	.	.	.
1	726202	C	T	intergenic	*LOC100288069,FAM87B*	.	.	.
9	69824962	G	A	intergenic	*LOC101928381,FOXD4L5*	.	.	.
4	12588049	TTGTG	T	intergenic	*LOC101929019,RAB28*	.	.	.
1	143187211	G	A	ncRNA_intronic	*LOC102723769*	.	.	.
1	143187212	G	A	ncRNA_intronic	*LOC102723769*	.	.	.
1	143536268	A	C	intergenic	*LOC102723769,MIR6077*	.	.	.
13	65535137	C	CTCTTTCTTT	intergenic	*LOC102723968,LINC01052*	.	.	.
9	44802320	T	A	intergenic	*LOC103908605,FAM27C*	.	.	.
4	158692438	ATGTG	A	intergenic	*LOC340017,FAM198B*	.	.	.
1	149335990	G	A	intergenic	*LOC388692,FCGR1C*	.	.	.
16	32323664	G	A	intergenic	*LOC390705,TP53TG3*	.	.	.
16	88177833	C	T	intergenic	*LOC400553,LOC101928880*	.	.	.
9	66531997	G	GTTTTACAT	ncRNA_intronic	*LOC403323*	.	.	.
14	19443249	T	C	intergenic	*LOC642426,POTEG*	.	.	.
4	119525056	T	C	ncRNA_intronic	*LOC729218*	.	.	.
10	30961826	C	T	intergenic	*LYZL2,SVILP1*	.	.	.
10	30961828	A	G	intergenic	*LYZL2,SVILP1*	.	.	.
5	94060905	G	GA	intronic	*MCTP1*	.	.	.
X	120942919	T	C	intergenic	*MIR3672,GRIA3*	.	.	.

2	87646936	A	ACCAG	intergenic	*MIR4771-2,LINC00152*	.	.	.
13	52904389	T	C	intergenic	*MRPS31P5,LOC103191607*	.	.	.
17	22260164	T	A	intergenic	*MTRNR2L1,NONE*	.	.	.
1	16904645	C	CTG	intronic	*NBPF1*	.	.	.
22	16140357	G	GC	intergenic	*NONE,DUXAP8*	.	.	.
Y	13279675	A	G	intergenic	*NONE,GYG2P1*	.	.	.
Y	13435523	A	G	intergenic	*NONE,GYG2P1*	.	.	.
21	9556305	A	G	intergenic	*NONE,MIR3648-1*	.	.	.
15	41674383	G	GA	intergenic	*NUSAP1,NDUFAF1*	.	.	.
11	132290879	TTCTC	T	intronic	*OPCML*	.	.	.
22	16871400	G	A	intergenic	*OR11H1,CCT8L2*	.	.	.
X	91087410	C	T	intergenic	*PABPC5,PCDH11X*	.	.	.
1	66769036	GTATA	G	intronic	*PDE4B*	.	.	.
X	85025667	AT	A	intergenic	*POF1B,MIR1321*	.	.	.
8	22322212	T	C	intronic	*PPP3CC*	.	.	.
5	35334327	GTATGTATATGTGTGTGTATATATGTACATATATATGTACATATATACACACACATATACATACACATA	G	intergenic	*PRLR,SPEF2*	.	.	.
7	142471601	C	G	intergenic	*PRSS1,PRSS3P2*	.	.	.
5	139210177	T	TTTC	intronic	*PSD2*	.	.	.
15	24641843	G	A	intergenic	*PWRN2,PWRN3*	.	.	.
1	174210076	TTGTG	T	intronic	*RABGAP1L*	.	.	.
7	102159825	C	CG	intergenic	*RASA4B,POLR2J3*	.	.	.

19	50626888	G	A	upstream	*SNAR-A10,SNAR-A11,SNAR-A14,SNAR-A3,SNAR-A4,SNAR-A5,SNAR-A6,SNAR-A7,SNAR-A8,SNAR-A9*	.	.	.
X	142426366	C	A	intergenic	*SPANXN4,SPANXN3*	.	.	.
X	142549319	T	C	intergenic	*SPANXN4,SPANXN3*	.	.	.
7	102000386	T	C	intergenic	*SPDYE6,LOC100289561*	.	.	.
11	14213792	GATATAT	G	intronic	*SPON1*	.	.	.
2	192006954	T	TAC	intronic	*STAT4*	.	.	.
21	10643879	CT	C	intergenic	*TEKT4P2,TPTE*	.	.	.
X	123810879	G	GA	intronic	*TENM1*	.	.	.
X	90226274	G	C	intergenic	*TGIF2LX,PABPC5-AS1*	.	.	.
10	51731505	C	G	intronic	*TIMM23B*	.	.	.
1	2585950	C	G	intronic	*TTC34*	.	.	.
9	141091454	T	C	intergenic	*TUBBP5,FAM157B*	.	.	.
3	41816378	T	G	intronic	*ULK4*	.	.	.
X	7814977	C	T	intergenic	*VCX,PNPLA4*	.	.	.
7	55672003	A	T	intergenic	*VOPP1,FKBP9P1*	.	.	.
19	44111568	TACAC	T	UTR3	*ZNF428*	.	.	.
9	39820449	T	G	intergenic	*ZNF658B,SPATA31A1*	.	.	.
4	164837127	C	G	intronic	*43891*	.	.	.
3	100835412	TACAC	T	intergenic	*ABI3BP,IMPG2*	.	.	.
2	111876461	T	TA	downstream	*ACOXL*	.	.	.
8	39217467	C	T	ncRNA_intronic	*ADAM5*	.	.	.
5	178684576	C	T	intronic	*ADAMTS2*	.	.	.
6	70217308	C	T	intergenic	*ADGRB3,LMBRD1*	.	.	.
12	131573658	C	G	intronic	*ADGRD1*	.	.	.

1	80156780	A	T	intergenic	*ADGRL4,LOC101927412*	.	.	.
1	5615244	G	C	intergenic	*AJAP1,MIR4417*	.	.	.
X	119029983	G	A	intronic	*AKAP14*	.	.	.
3	125836272	T	A	intronic	*ALDH1L1*	.	.	.
2	29749013	C	A	intronic	*ALK*	.	.	.
X	109648361	G	T	intronic	*AMMECR1*	.	.	.
21	15379084	T	TTCA	intergenic	*ANKRD20A11P,LIPI*	.	.	.
7	118989757	C	T	intergenic	*ANKRD7,LVCAT5*	.	.	.
10	49791990	G	C	intronic	*ARHGAP22*	.	.	.
X	130209639	C	A	intronic	*ARHGAP36*	.	.	.
3	36276709	A	T	intergenic	*ARPP21,STAC*	.	.	.
3	36370082	C	G	intergenic	*ARPP21,STAC*	.	.	.
1	76448108	C	A	intergenic	*ASB17,LOC101927342*	.	.	.
18	31169849	G	T	intronic	*ASXL3*	.	.	.
X	138941137	G	T	intergenic	*ATP11C,MIR505*	.	.	.
1	169092833	A	ATATT	intronic	*ATP1B1*	.	.	.
5	81793096	AG	A	intergenic	*ATP6AP1L,MIR3977*	.	.	.
18	76936802	C	G	intronic	*ATP9B*	.	.	.
14	99835076	G	A	intergenic	*BCL11B,SETD3*	.	.	.
X	39924455	C	A	intronic	*BCOR*	.	.	.
X	101996141	A	T	intronic	*BHLHB9*	.	.	.
20	7021662	G	T	intergenic	*BMP2,LINC01428*	.	.	.
7	34343614	G	T	intergenic	*BMPER,NPSR1-AS1*	.	.	.
X	79950433	G	T	intronic	*BRWD3*	.	.	.
11	38154756	C	T	intergenic	*C11orf74,LOC103312105*	.	.	.
12	4621154	G	A	intronic	*C12orf4*	.	.	.
16	27114096	G	T	intergenic	*C16orf82,KDM8*	.	.	.
19	30252594	A	G	intergenic	*C19orf12,CCNE1*	.	.	.
1	244688116	C	G	intronic	*C1orf101*	.	.	.
2	10409915	G	A	intergenic	*C2orf48,HPCAL1*	.	.	.
3	88425708	G	C	intergenic	*C3orf38,EPHA3*	.	.	.
6	128014123	GCACA	G	intergenic	*C6orf58,THEMIS*	.	.	.

7	109307273	G	T	intergenic	*C7orf66,EIF3IP1*	.	.	.
8	50541810	TACACAC	T	intergenic	*C8orf22,SNTG1*	.	.	.
8	96832578	G	C	intergenic	*C8orf37-AS1,LOC100500773*	.	.	.
3	53630221	T	C	intronic	*CACNA1D*	.	.	.
7	81615438	T	G	intronic	*CACNA2D1*	.	.	.
3	54350103	GGTGT	G	intronic	*CACNA2D3*	.	.	.
3	55497988	G	C	intergenic	*CACNA2D3,WNT5A*	.	.	.
10	12803068	G	A	exonic	*CAMK1D*	nonsynonymous_SNV	.	.
6	21884606	C	T	ncRNA_intronic	*CASC15*	.	.	.
6	22232901	A	G	intergenic	*CASC15,PRL*	.	.	.
X	41541669	G	T	intronic	*CASK*	.	.	.
X	42305667	A	G	intergenic	*CASK,PPP1R2P9*	.	.	.
20	54591551	C	A	intergenic	*CBLN4,MC3R*	.	.	.
20	54694926	G	A	intergenic	*CBLN4,MC3R*	.	.	.
4	24844780	A	C	intronic	*CCDC149*	.	.	.
14	70044044	C	T	intergenic	*CCDC177,SUSD6*	.	.	.
12	7618468	C	T	intergenic	*CD163L1,CD163*	.	.	.
6	14249354	A	G	intergenic	*CD83,LINC01108*	.	.	.
16	89252688	C	T	intronic	*CDH15*	.	.	.
18	64620325	A	G	intergenic	*CDH19,MIR5011*	.	.	.
18	25945295	A	C	intergenic	*CDH2,MIR302F*	.	.	.
1	214888212	GTATTGTGTTACGAGAC	G	intergenic	*CENPF,KCNK2*	.	.	.
17	63928996	T	TAC	intronic	*CEP112*	.	.	.
3	138299659	G	T	intronic	*CEP70*	.	.	.
15	93487886	AT	A	intronic	*CHD2*	.	.	.
X	85220423	C	A	intronic	*CHM*	.	.	.
12	105055182	C	CTAA	intronic	*CHST11*	.	.	.
18	25108472	G	T	intergenic	*CHST9,CDH2*	.	.	.
1	47844010	G	A	UTR3	*CMPK1*	.	.	.
5	79150951	G	C	intergenic	*CMYA5,LINC01455*	.	.	.
3	2549504	C	A	intronic	*CNTN4*	.	.	.

3	2571200	G	C	intronic	*CNTN4*	.	.	.
11	100192238	C	A	intronic	*CNTN5*	.	.	.
2	125516032	GTC	G	intronic	*CNTNAP5*	.	.	.
2	126815548	C	A	intergenic	*CNTNAP5,GYPC*	.	.	.
6	75906448	A	G	intronic	*COL12A1*	.	.	.
2	232701599	C	A	intergenic	*COPS7B,MIR1471*	.	.	.
8	68413982	G	A	intronic	*CPA6*	.	.	.
X	88853837	C	T	intergenic	*CPXCR1,TGIF2LX*	.	.	.
4	8715425	G	A	intergenic	*CPZ,HMX1*	.	.	.
7	28538050	C	G	intronic	*CREB5*	.	.	.
8	76021937	C	A	intergenic	*CRISPLD1,CASC9*	.	.	.
1	16860924	C	G	intergenic	*CROCCP3,FAM231B*	.	.	.
8	115655810	T	A	intergenic	*CSMD3,TRPS1*	.	.	.
X	116477470	G	T	intergenic	*CT83,KLHL13*	.	.	.
14	39767647	A	ATATATATAT	intronic	*CTAGE5*	.	.	.
5	151679457	G	T	intergenic	*CTB-12O2.1,NMUR2*	.	.	.
2	81226569	G	T	intergenic	*CTNNA2,LOC100507201*	.	.	.
4	49311303	ACACG	A	intergenic	*CWH43,NONE*	.	.	.
X	145956477	G	T	intergenic	*CXorf51A,MIR513C*	.	.	.
19	41358053	G	C	intergenic	*CYP2A6,CYP2A7*	.	.	.
21	15273067	G	A	intergenic	*CYP4F29P,ANKRD20A11P*	.	.	.
X	86232746	T	C	intergenic	*DACH2,KLHL4*	.	.	.
4	100755676	T	C	intronic	*DAPP1*	.	.	.
X	28438706	C	A	intergenic	*DCAF8L1,MIR6134*	.	.	.
X	28406936	C	T	intergenic	*DCAF8L1,MIR6134*	.	.	.
8	6884622	C	T	intergenic	*DEFA1,DEFA11P*	.	.	.
1	69310030	C	A	intergenic	*DEPDC1-AS1,LRRC7*	.	.	.
7	14975504	T	C	intergenic	*DGKB,AGMO*	.	.	.
17	57651745	C	G	intronic	*DHX40*	.	.	.
X	97851315	G	C	intergenic	*DIAPH2,XRCC6P5*	.	.	.

11	90880125	GTATATATACGCATATATACATCTATATACATAGATGTATATATGCGTATATATACA	G	intergenic	*DISC1FP1,FAT3*	.	.	.
11	84017260	T	A	intronic	*DLG2*	.	.	.
14	101234794	G	T	intergenic	*DLK1,MIR2392*	.	.	.
X	32741216	A	T	intronic	*DMD*	.	.	.
X	33816702	T	C	intergenic	*DMD,FAM47A*	.	.	.
7	21935872	G	A	intronic	*DNAH11*	.	.	.
1	225294079	G	A	intronic	*DNAH14*	.	.	.
17	11775762	G	A	intronic	*DNAH9*	.	.	.
2	118202104	G	T	intergenic	*DPP10,DDX18*	.	.	.
3	109112995	C	T	intergenic	*DPPA4,LINC01205*	.	.	.
21	42354983	G	A	intergenic	*DSCAM,LINC00323*	.	.	.
11	117448771	G	A	intronic	*DSCAML1*	.	.	.
8	34145774	A	T	intergenic	*DUSP26,LINC01288*	.	.	.
3	32662199	G	A	intergenic	*DYNC1LI1,CNOT10*	.	.	.
10	131762399	C	T	upstream	*EBF3*	.	.	.
13	50261367	A	T	intronic	*EBPL*	.	.	.
14	106209605	T	C	intergenic	*ELK2AP,MIR4507*	.	.	.
4	111387667	C	T	intergenic	*ELOVL6,ENPEP*	.	.	.
1	121366007	G	T	intergenic	*EMBP1,NONE*	.	.	.
2	120919036	C	G	intronic	*EPB41L5*	.	.	.
4	66555544	C	T	ncRNA_intronic	*EPHA5-AS1*	.	.	.
2	67970177	G	C	intergenic	*ETAA1,LOC101927701*	.	.	.
17	18312449	A	G	intergenic	*EVPLL,FLJ35934*	.	.	.
8	118950302	A	G	intronic	*EXT1*	.	.	.
X	154104759	T	A	intronic	*F8*	.	.	.
2	97703060	T	C	intergenic	*FAM178B,FAHD2B*	.	.	.
10	16387288	G	T	intergenic	*FAM188A,PTER*	.	.	.

9	38549582	A	G	intergenic	*FAM95C,ANKRD18A*	.	.	.
10	127613483	A	C	intronic	*FANK1*	.	.	.
12	29343399	A	AATTT	intronic	*FAR2*	.	.	.
7	100186324	A	G	intronic	*FBXO24*	.	.	.
3	192068262	G	T	intronic	*FGF12*	.	.	.
18	34222068	T	C	intronic	*FHOD3*	.	.	.
1	48060431	G	A	intergenic	*FOXD2,TRABD2B*	.	.	.
10	129570745	C	G	intergenic	*FOXI2,CLRN3*	.	.	.
19	52312779	G	T	intronic	*FPR3*	.	.	.
12	130749388	G	A	intergenic	*FZD10,PIWIL1*	.	.	.
5	162741181	C	A	intergenic	*GABRG2,CCNG1*	.	.	.
2	16542877	C	G	intergenic	*GACAT3,FAM49A*	.	.	.
7	99855654	C	T	intronic	*GATS*	.	.	.
2	76823044	C	T	intergenic	*GCFC2,LRRTM4*	.	.	.
X	14141748	T	A	intergenic	*GEMIN8,UBE2E4P*	.	.	.
6	122104909	G	T	intergenic	*GJA1,HSF2*	.	.	.
1	147392608	G	GAT	intergenic	*GJA8,GPR89B*	.	.	.
X	30722245	T	TTATATATAT	intronic	*GK*	.	.	.
6	2067729	C	A	intronic	*GMDS*	.	.	.
4	44942837	T	A	intergenic	*GNPDA2,GABRG1*	.	.	.
15	85783060	T	TGTA	upstream	*GOLGA6L3*	.	.	.
13	92559018	G	A	intronic	*GPC5*	.	.	.
13	93352065	T	A	intronic	*GPC5*	.	.	.
13	93530101	G	C	intergenic	*GPC5,LINC00363*	.	.	.
X	136403703	T	A	intergenic	*GPR101,ZIC3*	.	.	.
5	153105201	C	CAAAG	intronic	*GRIA1*	.	.	.
4	94228374	A	T	intronic	*GRID2*	.	.	.
4	93625862	C	G	intronic	*GRID2*	.	.	.
6	102425092	C	T	intronic	*GRIK2*	.	.	.
12	14092538	G	A	intronic	*GRIN2B*	.	.	.
9	105209982	T	C	intergenic	*GRIN3A,LINC00587*	.	.	.
9	105022907	C	G	intergenic	*GRIN3A,LINC00587*	.	.	.
4	43561412	G	T	intergenic	*GRXCR1,LVCAT1*	.	.	.
16	12039319	C	T	intergenic	*GSPT1,TNFRSF17*	.	.	.
6	52787937	TG	T	intergenic	*GSTA3,GSTA4*	.	.	.

X	103331515	C	G	intergenic	*H2BFXP,SLC25A53*	.	.	.
5	46084077	C	T	intergenic	*HCN1,NONE*	.	.	.
7	43537926	C	G	intronic	*HECW1*	.	.	.
15	23355470	A	G	ncRNA_intronic	*HERC2P2*	.	.	.
16	26245851	A	T	intergenic	*HS3ST4,C16orf82*	.	.	.
2	129176670	G	C	intergenic	*HS6ST1,LOC101927881*	.	.	.
X	131986038	T	C	intronic	*HS6ST2*	.	.	.
X	53815106	T	C	intergenic	*HUWE1,PHF8*	.	.	.
X	53815107	A	T	intergenic	*HUWE1,PHF8*	.	.	.
X	53872271	C	T	intergenic	*HUWE1,PHF8*	.	.	.
2	204897857	C	T	intergenic	*ICOS,PARD3B*	.	.	.
7	23418020	G	GTTTTTTTTT	intronic	*IGF2BP3*	.	.	.
X	130433622	C	A	intergenic	*IGSF1,OR13H1*	.	.	.
X	114283785	T	C	intergenic	*IL13RA2,LRCH2*	.	.	.
X	103915876	C	T	intronic	*IL1RAPL2*	.	.	.
3	137446879	C	A	intergenic	*IL20RB,SOX14*	.	.	.
5	3698878	C	A	intergenic	*IRX1,LOC101929153*	.	.	.
5	3905466	G	T	intergenic	*IRX1,LOC101929153*	.	.	.
5	50710393	C	A	intergenic	*ISL1,PELO*	.	.	.
10	65271198	G	A	intergenic	*JMJD1C-AS1,REEP3*	.	.	.
11	97507878	T	C	intergenic	*JRKL-AS1,CNTN5*	.	.	.
11	98175089	C	A	intergenic	*JRKL-AS1,CNTN5*	.	.	.
17	44159775	C	T	intronic	*KANSL1*	.	.	.
3	20167593	TC	T	exonic	*KAT2B*	frameshift_deletion	.	.
2	95974853	C	A	intronic	*KCNIP3*	.	.	.
2	156305971	C	A	intergenic	*KCNJ3,LOC101929378*	.	.	.
21	39120935	T	A	intronic	*KCNJ6*	.	.	.
10	79531897	C	A	intergenic	*KCNMA1,DLG5*	.	.	.
6	73480386	T	A	intronic	*KCNQ5*	.	.	.
6	73882963	A	C	intronic	*KCNQ5*	.	.	.
5	144232096	G	A	intergenic	*KCTD16,PRELID2*	.	.	.
4	44257412	A	T	intronic	*KCTD8*	.	.	.

8	138170310	ATCTCTC	A	intergenic	*KHDRBS3,LOC101927915*	.	.	.
8	137841505	C	G	intergenic	*KHDRBS3,LOC101927915*	.	.	.
20	16624235	A	G	intergenic	*KIF16B,SNRPB2*	.	.	.
17	52302604	T	C	intergenic	*KIF2B,TOM1L1*	.	.	.
6	39449831	G	A	intronic	*KIF6*	.	.	.
13	73708076	G	A	intergenic	*KLF5,LINC00392*	.	.	.
X	87064397	G	T	intergenic	*KLHL4,CPXCR1*	.	.	.
X	87741449	T	A	intergenic	*KLHL4,CPXCR1*	.	.	.
7	151995284	C	T	intronic	*KMT2C*	.	.	.
17	45736253	C	T	intronic	*KPNB1*	.	.	.
12	76102103	C	A	intergenic	*KRR1,PHLDA1*	.	.	.
10	27224228	G	GCAA	ncRNA_intronic	*LINC00202-1*	.	.	.
16	34233776	C	T	intergenic	*LINC00273,UBE2MP1*	.	.	.
16	33963923	CG	C	intergenic	*LINC00273,UBE2MP1*	.	.	.
21	23509663	T	TA	intergenic	*LINC00308,D21S2088E*	.	.	.
13	84820932	G	A	ncRNA_intronic	*LINC00333*	.	.	.
13	71630718	G	A	ncRNA_intronic	*LINC00348*	.	.	.
13	93755865	T	A	intergenic	*LINC00363,GPC6*	.	.	.
13	68566830	T	G	intergenic	*LINC00364,LINC00550*	.	.	.
13	59523961	A	G	intergenic	*LINC00374,DIAPH3*	.	.	.
13	22643662	A	T	intergenic	*LINC00424,LINC00540*	.	.	.
20	47123628	T	C	intergenic	*LINC00494,PREX1*	.	.	.
20	47123629	G	A	intergenic	*LINC00494,PREX1*	.	.	.
14	101903896	G	A	intergenic	*LINC00524,DIO3OS*	.	.	.
8	117323466	G	T	ncRNA_intronic	*LINC00536*	.	.	.
14	29121506	CTATA	C	intergenic	*LINC00645,FOXG1-AS1*	.	.	.
14	49368832	C	T	intergenic	*LINC00648,RPS29*	.	.	.

3	117056753	TACAC	T	intergenic	*LINC00901,IGSF11*	.	.	.
3	117735067	A	G	intergenic	*LINC00901,IGSF11*	.	.	.
12	90805125	G	A	intergenic	*LINC00936,LINC00 615*	.	.	.
9	132313322	G	A	intergenic	*LINC00963,NTMT1*	.	.	.
7	113130153	C	A	intergenic	*LINC00998,PPP1R 3A*	.	.	.
10	38907945	A	T	intergenic	*LINC00999,ACTR3 BP5*	.	.	.
5	3452463	G	A	ncRNA_intronic	*LINC01019*	.	.	.
5	28587368	G	T	intergenic	*LINC01021,LSP1P 3*	.	.	.
18	76064878	G	T	intergenic	*LINC01029,SALL3*	.	.	.
1	194719461	C	T	intergenic	*LINC01031,KCNT2*	.	.	.
13	63239590	T	C	intergenic	*LINC01075,LINC00 448*	.	.	.
13	63239592	G	A	intergenic	*LINC01075,LINC00 448*	.	.	.
4	124994485	G	C	intergenic	*LINC01091,LOC10 1927087*	.	.	.
8	77473990	T	G	intergenic	*LINC01111,ZFHX4- AS1*	.	.	.
2	177742519	C	T	intergenic	*LINC01117,HNRNP A3*	.	.	.
2	59259279	G	C	ncRNA_intronic	*LINC01122*	.	.	.
10	130348589	C	A	intergenic	*LINC01163,MGMT*	.	.	.
10	130984349	G	A	intergenic	*LINC01163,MGMT*	.	.	.
3	163400465	G	C	intergenic	*LINC01192,MIR126 3*	.	.	.
14	75797026	G	A	intergenic	*LINC01220,JDP2*	.	.	.
1	199270790	AACAC	A	intergenic	*LINC01221,NR5A2*	.	.	.
9	23338470	C	A	intergenic	*LINC01239,LOC10 1929563*	.	.	.
2	6557226	G	GATATAGAT	intergenic	*LINC01247,LINC01 246*	.	.	.
2	2952937	G	C	ncRNA_intronic	*LINC01250*	.	.	.

13	105176597	T	G	intergenic	*LINC01309,DAOA-AS1*	.	.	.
13	105329774	T	C	intergenic	*LINC01309,DAOA-AS1*	.	.	.
2	34641656	T	C	intergenic	*LINC01317,LINC01320*	.	.	.
2	34902310	T	G	upstream	*LINC01320*	.	.	.
2	36161821	T	A	intergenic	*LINC01320,LOC100288911*	.	.	.
10	110771722	GTATATGTGTATATATGTATACGTATATACACACATATATACACA	G	intergenic	*LINC01435,XPNPEP1*	.	.	.
18	15162368	C	T	intergenic	*LINC01443,LOC644669*	.	.	.
7	53790099	C	G	ncRNA_intronic	*LINC01446*	.	.	.
5	152488659	C	A	intergenic	*LINC01470,GRIA1*	.	.	.
11	38847414	T	A	intergenic	*LINC01493,LRRC4C*	.	.	.
11	39785795	G	T	intergenic	*LINC01493,LRRC4C*	.	.	.
20	51238682	T	C	ncRNA_intronic	*LINC01524*	.	.	.
3	128298509	G	T	intergenic	*LINC01565,RPN1*	.	.	.
3	191279163	GTCTC	G	ncRNA_intronic	*LINCR-0002*	.	.	.
9	30125787	A	T	intergenic	*LINGO2,LINC01242*	.	.	.
7	97832946	G	C	exonic	*LMTK2*	nonsynonymous_SNV	.	.
1	104912726	GTATA	G	intergenic	*LOC100129138,LOC101928476*	.	.	.
11	95281395	A	G	intergenic	*LOC100129203,FAM76B*	.	.	.
17	5122945	T	A	ncRNA_intronic	*LOC100130950*	.	.	.
7	7215610	G	T	intergenic	*LOC100131257,C1GALT1*	.	.	.

5	98721657	T	TATTTACA	intergenic	*LOC100289230,CTD-2151A2.1*	.	.	.
X	55437037	G	T	intergenic	*LOC100421746,MIR4536-2*	.	.	.
2	13641875	C	CTAAA	intergenic	*LOC100506474,LINC00276*	.	.	.
2	81824691	C	T	intergenic	*LOC100507201,LOC1720*	.	.	.
2	82069594	G	A	intergenic	*LOC100507201,LOC1720*	.	.	.
2	82173793	TC	T	intergenic	*LOC100507201,LOC1720*	.	.	.
11	42400483	C	A	intergenic	*LOC100507205,HNRNPKP3*	.	.	.
9	137732221	C	T	ncRNA_intronic	*LOC101448202*	.	.	.
5	42220196	C	A	intergenic	*LOC101926960,GHR*	.	.	.
8	98568756	C	A	intergenic	*LOC101927066,MTDH*	.	.	.
2	60317536	G	A	intergenic	*LOC101927285,MIR4432HG*	.	.	.
9	82169572	A	G	intergenic	*LOC101927450,TLE4*	.	.	.
9	82111804	C	T	intergenic	*LOC101927450,TLE4*	.	.	.
12	127805258	A	G	intergenic	*LOC101927592,LOC101927616*	.	.	.
12	127967153	T	A	intergenic	*LOC101927616,LOC101927637*	.	.	.
12	128666090	G	A	intergenic	*LOC101927694,MIR4419B*	.	.	.
8	2791216	C	A	intergenic	*LOC101927815,CSMD1*	.	.	.
8	138691115	C	CAAAAT	intergenic	*LOC101927915,FAM135B*	.	.	.
8	138815418	G	A	intergenic	*LOC101927915,FAM135B*	.	.	.
7	112298047	G	T	ncRNA_intronic	*LOC101928012*	.	.	.

X	4625039	G	T	intergenic	*LOC101928201,NLGN4X*	.	.	.
X	5792614	G	C	intergenic	*LOC101928201,NLGN4X*	.	.	.
11	106412654	T	C	intergenic	*LOC101928535,GUCY1A2*	.	.	.
4	34393819	G	T	intergenic	*LOC101928622,ARAP2*	.	.	.
4	35504448	TCA	T	intergenic	*LOC101928622,ARAP2*	.	.	.
14	86881004	T	C	intergenic	*LOC101928767,LOC283585*	.	.	.
4	12517909	CA	C	intergenic	*LOC101929019,RAB28*	.	.	.
4	13180770	GATAT	G	intergenic	*LOC101929019,RAB28*	.	.	.
1	31098219	C	A	intergenic	*LOC101929406,MATN1*	.	.	.
5	30212401	C	T	intergenic	*LOC101929681,CDH6*	.	.	.
5	30132196	A	T	intergenic	*LOC101929681,CDH6*	.	.	.
1	143166102	C	T	ncRNA_intronic	*LOC102723769*	.	.	.
1	143141351	T	C	ncRNA_intronic	*LOC102723769*	.	.	.
4	33221871	T	A	intergenic	*LOC102723828,LOC101928622*	.	.	.
4	33226737	C	T	intergenic	*LOC102723828,LOC101928622*	.	.	.
6	68844142	C	T	intergenic	*LOC102723883,LOC101928280*	.	.	.
13	65477881	A	C	intergenic	*LOC102723968,LINC01052*	.	.	.
18	51099447	T	A	ncRNA_intronic	*LOC102724651*	.	.	.
8	78379750	C	T	ncRNA_intronic	*LOC102724874*	.	.	.
9	44800225	A	T	intergenic	*LOC103908605,FAM27C*	.	.	.
2	240752160	C	G	intergenic	*LOC150935,MIR4786*	.	.	.

2	130205336	C	T	intergenic	*LOC151121,LOC389033*	.	.	.
2	130534839	G	T	intergenic	*LOC151121,LOC389033*	.	.	.
2	83998425	A	C	intergenic	*LOC1720,FUNDC2P2*	.	.	.
17	5936159	G	A	intergenic	*LOC339166,WSCD1*	.	.	.
5	32966895	G	T	intergenic	*LOC340113,TARS*	.	.	.
X	4178634	C	A	intergenic	*LOC389906,LOC101928201*	.	.	.
16	33304601	TTTTG	T	intergenic	*LOC390705,ENPP7P13*	.	.	.
5	19168593	T	A	intergenic	*LOC401177,CDH18*	.	.	.
11	50298550	C	T	intergenic	*LOC441601,LOC646813*	.	.	.
12	93771412	G	C	ncRNA_exonic	*LOC643339*	.	.	.
18	65440523	A	AGATAGATA	ncRNA_intronic	*LOC643542*	.	.	.
14	41947252	T	G	intergenic	*LOC644919,LRFN5*	.	.	.
2	22146344	G	A	intergenic	*LOC645949,LOC102723362*	.	.	.
11	51194166	G	T	intergenic	*LOC646813,OR4A5*	.	.	.
2	91906938	CTTT	C	intergenic	*LOC654342,GGT8P*	.	.	.
20	58708792	G	T	intergenic	*LOC729296,MIR646HG*	.	.	.
8	106083933	C	G	intergenic	*LRP12,ZFPM2*	.	.	.
2	141107395	C	G	intronic	*LRP1B*	.	.	.
2	143016196	C	G	intergenic	*LRP1B,KYNU*	.	.	.
1	74620943	A	T	intronic	*LRRIQ3*	.	.	.
X	141008535	G	T	intergenic	*MAGEC1,MAGEC2*	.	.	.
X	141460582	C	G	intergenic	*MAGEC2,SPANXN4*	.	.	.
X	75383327	C	G	intergenic	*MAGEE2,PBDC1*	.	.	.
5	109118065	G	C	intronic	*MAN2A1*	.	.	.

6	96336561	G	A	intergenic	*MANEA,FUT9*	.	.	.
17	21238983	T	A	intergenic	*MAP2K3,KCNJ12*	.	.	.
2	149076622	G	C	intronic	*MBD5*	.	.	.
15	37425373	T	TTTCTTTCTC	intergenic	*MEIS2,TMCO5A*	.	.	.
1	40439845	G	C	intergenic	*MFSD2A,CAP1*	.	.	.
X	10704176	G	A	intronic	*MID1*	.	.	.
14	37897068	T	C	intronic	*MIPOL1*	.	.	.
X	63096063	T	C	intergenic	*MIR1468,AMER1*	.	.	.
4	117456142	GTATA	G	intergenic	*MIR1973,TRAM1L1*	.	.	.
4	127086820	C	T	intergenic	*MIR2054,INTU*	.	.	.
6	120673263	C	G	intergenic	*MIR3144,TBC1D32*	.	.	.
7	57493923	C	A	intergenic	*MIR3147,ZNF716*	.	.	.
16	15387970	C	T	intergenic	*MIR3180-4,NPIPA5*	.	.	.
X	75881425	G	T	ncRNA_intronic	*MIR325HG*	.	.	.
X	120680536	G	C	intergenic	*MIR3672,GRIA3*	.	.	.
X	120856527	G	T	intergenic	*MIR3672,GRIA3*	.	.	.
7	63168600	G	A	intergenic	*MIR4283-2,LINC01005*	.	.	.
18	35306730	C	G	intergenic	*MIR4318,MIR924HG*	.	.	.
2	52934979	C	T	intergenic	*MIR4431,ASB3*	.	.	.
2	90030473	G	T	intergenic	*MIR4436A,LOC654342*	.	.	.
2	90435846	C	CACACA	intergenic	*MIR4436A,LOC654342*	.	.	.
2	90435939	G	A	intergenic	*MIR4436A,LOC654342*	.	.	.
2	90470962	A	AAAAG	intergenic	*MIR4436A,LOC654342*	.	.	.
3	109756763	G	T	intergenic	*MIR4445,PVRL3-AS1*	.	.	.
X	92580508	G	T	intergenic	*MIR4454,NAP1L3*	.	.	.
7	137823444	G	T	intergenic	*MIR4468,TRIM24*	.	.	.
18	45157369	T	A	intergenic	*MIR4527,SMAD2*	.	.	.
X	6421455	A	G	intergenic	*MIR4770,VCX3A*	.	.	.

2	87605598	ATATATATATATG	A	intergenic	*MIR4771-2,LINC00152*	.	.	.
13	55849086	G	T	intergenic	*MIR5007,PRR20D*	.	.	.
13	57615608	G	T	intergenic	*MIR5007,PRR20D*	.	.	.
5	143059714	T	C	downstream	*MIR5197*	.	.	.
14	30132359	A	G	ncRNA_intronic	*MIR548AI*	.	.	.
4	23691076	C	A	intergenic	*MIR548AJ2,PPARGC1A*	.	.	.
21	22073061	GTATATA	G	intergenic	*MIR548XHG,LINC00320*	.	.	.
20	7735651	A	T	intergenic	*MIR8062,HAO1*	.	.	.
18	36786583	C	T	downstream	*MIR924HG*	.	.	.
14	45973549	G	A	intergenic	*MIS18BP1,LINC00871*	.	.	.
3	158349385	T	C	intergenic	*MLF1,GFM1*	.	.	.
2	39126995	T	TA	intergenic	*MORN2,ARHGEF33*	.	.	.
8	16235325	G	T	intergenic	*MSR1,FGF20*	.	.	.
16	3424864	C	A	intergenic	*MTRNR2L4,ZSCAN32*	.	.	.
10	57487120	G	C	intergenic	*MTRNR2L5,ZWINT*	.	.	.
9	103543739	C	A	intergenic	*MURC,PLPPR1*	.	.	.
X	72427980	A	T	intergenic	*NAP1L6,NAP1L2*	.	.	.
1	146472880	G	A	intergenic	*NBPF20,LOC728989*	.	.	.
1	144849389	C	T	intronic	*NBPF20,NBPF9*	.	.	.
5	85807914	T	G	intergenic	*NBPF22P,COX7C*	.	.	.
1	148754482	A	C	ncRNA_intronic	*NBPF25P*	.	.	.
20	26095289	G	A	upstream	*NCOR1P1*	.	.	.
4	117095046	G	A	intergenic	*NDST4,MIR1973*	.	.	.
15	22152825	G	A	intergenic	*NF1P2,MIR5701-3*	.	.	.
X	5975687	C	T	intronic	*NLGN4X*	.	.	.
1	142606340	C	T	intergenic	*NONE,ANKRD20A12P*	.	.	.
15	20020674	G	A	intergenic	*NONE,CHEK2P2*	.	.	.
15	20030199	C	A	intergenic	*NONE,CHEK2P2*	.	.	.
15	20425743	T	A	intergenic	*NONE,CHEK2P2*	.	.	.
15	20105669	G	T	intergenic	*NONE,CHEK2P2*	.	.	.

8	47298667	A	G	intergenic	*NONE,LINC00293*	.	.	.
14	85020553	T	TTAG	intergenic	*NONE,LINC00911*	.	.	.
2	91777628	C	A	intergenic	*NONE,LOC654342*	.	.	.
5	16445	G	GT	intergenic	*NONE,PLEKHG4B*	.	.	.
X	61715532	T	C	intergenic	*NONE,SPIN4*	.	.	.
X	61853333	A	G	intergenic	*NONE,SPIN4*	.	.	.
Y	59002825	CAAA	C	intergenic	*NONE,SPRY3*	.	.	.
7	61086648	A	C	intergenic	*NONE,ZNF733P*	.	.	.
6	155951364	T	C	intergenic	*NOX3,MIR1202*	.	.	.
3	132449031	T	A	ncRNA_intronic	*NPHP3-AS1*	.	.	.
16	16555944	T	C	intergenic	*NPIPA8,XYLT1*	.	.	.
16	16883364	G	A	intergenic	*NPIPA8,XYLT1*	.	.	.
16	16900015	C	T	intergenic	*NPIPA8,XYLT1*	.	.	.
16	28771491	C	T	intronic	*NPIPB9*	.	.	.
10	129362368	C	T	intergenic	*NPS,FOXI2*	.	.	.
1	200293790	A	G	intergenic	*NR5A2,LINC00862*	.	.	.
21	17020745	T	TAAG	intergenic	*NRIP1,USP25*	.	.	.
14	80245719	G	A	intronic	*NRXN3*	.	.	.
14	80249225	A	C	intronic	*NRXN3*	.	.	.
11	131770830	C	T	intronic	*NTM*	.	.	.
11	131986514	C	A	intronic	*NTM*	.	.	.
17	9012280	G	T	intronic	*NTN1*	.	.	.
15	53091434	G	T	intergenic	*ONECUT1,WDR72*	.	.	.
11	132520984	C	T	intronic	*OPCML*	.	.	.
11	133092314	C	T	intronic	*OPCML*	.	.	.
11	123963691	GGAAGGA	G	intergenic	*OR10G7,VWA5A*	.	.	.
7	143692588	G	T	intergenic	*OR2F1,OR6B1*	.	.	.
11	59303440	G	T	intergenic	*OR4D9,OSBP*	.	.	.
11	55608940	G	A	intergenic	*OR5D16,TRIM51*	.	.	.
3	31939224	A	G	intronic	*OSBPL10*	.	.	.
3	125419725	G	T	intergenic	*OSBPL11,MIR548I1*	.	.	.
12	29604096	AATC	A	ncRNA_intronic	*OVCH1-AS1*	.	.	.
11	7755533	TAATTTTAAAAATTTTTTTAA	T	intergenic	*OVCH2,OR5P2*	.	.	.
3	8795825	G	C	intronic	*OXTR*	.	.	.
9	118929832	C	T	intronic	*PAPPA*	.	.	.

3	183588061	G	A	intronic	*PARL*	.	.	.
X	150830849	T	A	intronic	*PASD1*	.	.	.
5	140633161	C	T	intergenic	*PCDHB15,SLC25A2*	.	.	.
11	34973012	C	T	intronic	*PDHX*	.	.	.
6	12936669	C	T	intronic	*PHACTR1*	.	.	.
17	10861375	G	A	intergenic	*PIRT,SHISA6*	.	.	.
12	33515950	C	T	intergenic	*PKP2,SYT10*	.	.	.
X	133865661	G	A	intronic	*PLAC1*	.	.	.
10	124139338	A	G	intronic	*PLEKHA1*	.	.	.
7	74328582	C	G	ncRNA_intronic	*PMS2P5*	.	.	.
19	7626640	C	T	UTR3	*PNPLA6*	.	.	.
7	131513712	T	TTTC	intergenic	*PODXL,LOC101928782*	.	.	.
14	19552644	GC	G	upstream	*POTEG*	.	.	.
X	82878841	T	A	intergenic	*POU3F4,CYLC1*	.	.	.
X	82949322	T	C	intergenic	*POU3F4,CYLC1*	.	.	.
12	111266024	T	TTTC	intergenic	*PPP1CC,CCDC63*	.	.	.
X	42844223	C	T	intergenic	*PPP1R2P9,LOC101927501*	.	.	.
11	129823431	T	C	intronic	*PRDM10*	.	.	.
6	57358368	TTAAATAAAATTGATGATTAAAAGTTGATTAAAA	T	intronic	*PRIM2*	.	.	.
6	57501257	C	T	intronic	*PRIM2*	.	.	.
12	3708509	C	G	intergenic	*PRMT8,CRACR2A*	.	.	.
9	81167127	A	ATAC	intergenic	*PSAT1,LOC101927450*	.	.	.
X	23257639	G	A	ncRNA_intronic	*PTCHD1-AS*	.	.	.
11	13530335	C	G	intergenic	*PTH,FAR1*	.	.	.
1	202130166	G	GGCCCTCTC	UTR5	*PTPN7*	.	.	.
9	10523607	A	ATATATATAT	intronic	*PTPRD*	.	.	.
7	157986720	G	A	intronic	*PTPRN2*	.	.	.
15	24483466	G	T	intergenic	*PWRN2,PWRN3*	.	.	.
11	82738616	A	T	intronic	*RAB30*	.	.	.
2	130753922	G	GAATT	intergenic	*RAB6C,FAR2P1*	.	.	.

5	104471353	G	C	intergenic	*RAB9BP1,LOC102467213*	.	.	.
20	20459005	C	A	intronic	*RALGAPA2*	.	.	.
8	85828503	T	C	intronic	*RALYL*	.	.	.
12	69053250	T	A	UTR3	*RAP1B*	.	.	.
4	160762412	GTATA	G	intergenic	*RAPGEF2,FSTL5*	.	.	.
16	6718629	C	T	intronic	*RBFOX1*	.	.	.
9	4821768	T	A	intronic	*RCL1*	.	.	.
10	66271280	G	T	intergenic	*REEP3,ANXA2P3*	.	.	.
X	36803636	C	T	intergenic	*RP11-87M18.2,FAM47C*	.	.	.
8	119624890	G	C	intronic	*SAMD12*	.	.	.
7	83965811	A	AACT	intergenic	*SEMA3A,LOC101927378*	.	.	.
X	81446239	T	A	intergenic	*SH3BGRL,POU3F4*	.	.	.
19	51787518	C	A	intergenic	*SIGLECL1,IGLON5*	.	.	.
15	48562968	TATATATATATATAC	T	intronic	*SLC12A1*	.	.	.
6	111481996	C	G	intronic	*SLC16A10*	.	.	.
17	6941634	G	T	exonic	*SLC16A13*	nonsynonymous_SNV	.	.
7	95981500	C	T	intergenic	*SLC25A13,C7orf76*	.	.	.
5	110188861	G	A	intergenic	*SLC25A46,TSLP*	.	.	.
6	67797935	G	C	intergenic	*SLC25A51P1,LOC102723883*	.	.	.
12	7971706	G	A	intronic	*SLC2A14*	.	.	.
14	58076889	G	T	intronic	*SLC35F4*	.	.	.
5	168641628	G	T	intronic	*SLIT3*	.	.	.
20	57638459	A	G	intergenic	*SLMO2-ATP5E,ZNF831*	.	.	.
6	87277373	G	C	intergenic	*SNHG5,HTR1E*	.	.	.
13	85394726	T	A	intergenic	*SNORA107,LINC00375*	.	.	.
8	84061919	T	A	intergenic	*SNX16,LINC01419*	.	.	.
2	5958669	C	A	intergenic	*SOX11,LINC01105*	.	.	.

22	24712825	G	A	ncRNA_intronic	*SPECC1L-ADORA2A*	.	.	.
2	229241829	T	C	intergenic	*SPHKAP,PID1*	.	.	.
2	229616883	C	A	intergenic	*SPHKAP,PID1*	.	.	.
2	229675343	C	A	intergenic	*SPHKAP,PID1*	.	.	.
4	168295119	T	C	intergenic	*SPOCK3,ANXA10*	.	.	.
2	235294548	G	T	intergenic	*SPP2,ARL4C*	.	.	.
13	81158003	T	C	intergenic	*SPRY2,LINC00377*	.	.	.
13	81389485	G	C	intergenic	*SPRY2,LINC00377*	.	.	.
5	80845493	A	T	intronic	*SSBP2*	.	.	.
8	53334667	G	A	intergenic	*ST18,FAM150A*	.	.	.
5	111005319	G	A	ncRNA_exonic	*STARD4-AS1*	.	.	.
4	27144739	CT	C	intergenic	*STIM2,LOC101929199*	.	.	.
4	5296879	G	T	intronic	*STK32B*	.	.	.
4	4645616	CCA	C	ncRNA_intronic	*STX18-AS1*	.	.	.
4	4731463	C	A	intergenic	*STX18-AS1,LOC101928279*	.	.	.
8	110937118	G	T	intergenic	*SYBU,KCNV1*	.	.	.
20	24451880	G	A	intronic	*SYNDIG1*	.	.	.
20	13645414	C	G	intergenic	*TASP1,ESF1*	.	.	.
17	36304713	G	C	intergenic	*TBC1D3,TBC1D3*	.	.	.
7	143345031	G	C	intronic	*TCAF2*	.	.	.
13	61056234	C	A	intronic	*TDRD3*	.	.	.
9	27183707	C	T	intronic	*TEK*	.	.	.
21	10897367	G	T	intergenic	*TEKT4P2,TPTE*	.	.	.
X	124169483	A	C	intergenic	*TENM1,LOC100129520*	.	.	.
5	167134714	A	C	intronic	*TENM2*	.	.	.
11	79169204	G	A	intergenic	*TENM4,LOC101928944*	.	.	.
16	75762093	G	C	intergenic	*TERF2IP,CNTNAP4*	.	.	.
2	88823410	A	C	upstream	*TEX37*	.	.	.
2	146552133	GATATAT	G	intergenic	*TEX41,PABPC1P2*	.	.	.
2	188836439	C	G	intergenic	*TFPI,LINC01090*	.	.	.
7	11855368	T	G	intronic	*THSD7A*	.	.	.

17	8084931	C	CCTG	intergenic	*TMEM107,MIR4521*	.	.	.
12	126260198	G	T	intergenic	*TMEM132B,LINC00939*	.	.	.
12	129634853	C	T	intronic	*TMEM132D*	.	.	.
2	135501040	G	A	intergenic	*TMEM163,ACMSD*	.	.	.
2	104441074	G	A	intergenic	*TMEM182,LOC100287010*	.	.	.
6	130775168	T	G	intergenic	*TMEM200A,SMLR1*	.	.	.
21	19717951	G	A	intronic	*TMPRSS15*	.	.	.
11	117962148	C	T	intronic	*TMPRSS4*	.	.	.
12	84128820	A	C	intergenic	*TMTC2,SLC6A15*	.	.	.
1	175397046	C	A	intronic	*TNR*	.	.	.
21	10951083	G	T	intronic	*TPTE*	.	.	.
6	111812053	G	A	ncRNA_intronic	*TRAF3IP2-AS1*	.	.	.
8	141299512	T	C	intronic	*TRAPPC9*	.	.	.
6	41232275	T	TAA	intergenic	*TREML5P,TREM1*	.	.	.
8	110155281	A	T	intergenic	*TRHR,NUDCD1*	.	.	.
8	94024115	C	T	intergenic	*TRIQK,MIR8084*	.	.	.
7	141995816	A	T	intergenic	*TRY2P,MTRNR2L6*	.	.	.
7	142028918	C	A	intergenic	*TRY2P,MTRNR2L6*	.	.	.
6	95018316	G	A	intergenic	*TSG1,MANEA-AS1*	.	.	.
2	123285469	G	A	intergenic	*TSN,CNTNAP5*	.	.	.
7	120455530	G	C	intronic	*TSPAN12*	.	.	.
22	46686444	GGTGTTAGGAGGGTGGTGTGGCTGGAGGGTGA	G	intronic	*TTC38*	.	.	.
14	76386345	G	A	intronic	*TTLL5*	.	.	.
10	104539	CAG	C	intergenic	*TUBB8,ZMYND11*	.	.	.
10	120793	A	G	intergenic	*TUBB8,ZMYND11*	.	.	.
10	120794	C	T	intergenic	*TUBB8,ZMYND11*	.	.	.
1	154201471	G	C	intronic	*UBAP2L*	.	.	.
X	144254889	G	T	intergenic	*UBE2NL,SPANXN1*	.	.	.
15	55357428	C	T	intergenic	*UNC13C,RSL24D1*	.	.	.

8	36066490	T	A	intergenic	*UNC5D,KCNU1*	.	.	.
13	31285067	A	C	intergenic	*USPL1,ALOX5AP*	.	.	.
X	27031274	GTATATGTAAATATATTTACATATATGCATAACGTGAATATGTA	G	intergenic	*VENTXP1,PPP4R3CP*	.	.	.
11	124084905	T	TTAGATAGA	intergenic	*VWA5A,OR8G2*	.	.	.
13	27177244	C	A	intronic	*WASF3*	.	.	.
X	48501300	CTCTCTCTCTATA	C	intergenic	*WDR13,WAS*	.	.	.
16	79581817	TAGAG	T	intergenic	*WWOX,MAF*	.	.	.
X	98732166	G	T	ncRNA_intronic	*XRCC6P5*	.	.	.
X	99404651	C	T	intergenic	*XRCC6P5,PCDH19*	.	.	.
16	17720871	G	A	intergenic	*XYLT1,NPIPA8*	.	.	.
16	18305329	T	C	intergenic	*XYLT1,NPIPA8*	.	.	.
11	11198784	C	CTCTCAAGT	intergenic	*ZBED5-AS1,GALNT18*	.	.	.
X	64510801	G	T	intergenic	*ZC4H2,ZC3H12B*	.	.	.
5	178365310	G	A	intergenic	*ZFP2,ZNF454*	.	.	.
20	51147982	C	A	intergenic	*ZFP64,LINC01524*	.	.	.
X	136686176	C	CTT	intergenic	*ZIC3,LINC00889*	.	.	.
X	136735528	T	A	intergenic	*ZIC3,LINC00889*	.	.	.
12	133672577	A	C	intronic	*ZNF140*	.	.	.
16	32140750	T	C	intergenic	*ZNF267,HERC2P4*	.	.	.
X	129408987	G	T	intergenic	*ZNF280C,SLC25A14*	.	.	.
19	22883214	C	T	intergenic	*ZNF492,ZNF99*	.	.	.
2	133096640	C	CACACATAT	intergenic	*ZNF806,GPR39*	.	.	.
19	20642026	C	A	intergenic	*ZNF826P,ZNF737*	.	.	.
19	23010814	G	A	intergenic	*ZNF99,ZNF728*	.	.	.
3	151777973	G	T	intergenic	*AADACL2-AS1,MBNL1-AS1*	.	.	.
7	48590246	G	A	intronic	*ABCA13*	.	.	.
1	180258583	C	A	intronic	*ACBD6*	.	.	.
7	153042662	C	A	intergenic	*ACTR3B,LINC01287*	.	.	.

2	92239050	TG	T	intergenic	*ACTR3BP2,NONE*	.	.	.
10	39060463	A	G	intergenic	*ACTR3BP5,NONE*	.	.	.
X	128142062	GTATATA	G	intergenic	*ACTRT1,SMARCA1*	.	.	.
4	176382182	C	G	intergenic	*ADAM29,GPM6A*	.	.	.
5	33827350	CACCACTGGGTTGAAGATG	C	intronic	*ADAMTS12*	.	.	.
11	130521410	GCACA	G	intergenic	*ADAMTS15,MIR8052*	.	.	.
4	73251704	A	AT	intronic	*ADAMTS3*	.	.	.
3	65095423	C	CACACAG	intergenic	*ADAMTS9-AS2,MAGI1*	.	.	.
8	132719658	G	C	intergenic	*ADCY8,EFR3A*	.	.	.
8	132325489	C	T	intergenic	*ADCY8,EFR3A*	.	.	.
6	69866997	C	A	intronic	*ADGRB3*	.	.	.
X	19255447	C	A	intergenic	*ADGRG2,PDHA1*	.	.	.
4	63268167	G	T	intergenic	*ADGRL3-AS1,TECRL*	.	.	.
4	63530469	CATTATT	C	intergenic	*ADGRL3-AS1,TECRL*	.	.	.
3	186611513	T	C	intergenic	*ADIPOQ,ST6GAL1*	.	.	.
10	75438697	C	T	intronic	*AGAP5*	.	.	.
15	87450682	T	G	intronic	*AGBL1*	.	.	.
1	244009808	T	G	intergenic	*AKT3,LOC339529*	.	.	.
2	30328711	T	TG	intergenic	*ALK,YPEL5*	.	.	.
2	30333591	C	G	intergenic	*ALK,YPEL5*	.	.	.
2	73877241	C	T	ncRNA_intronic	*ALMS1P1*	.	.	.
4	145988823	T	C	intronic	*ANAPC10*	.	.	.
4	74062667	A	ATGTATG	intronic	*ANKRD17*	.	.	.
9	69488970	G	A	intergenic	*ANKRD20A4,LOC100133920*	.	.	.
10	27336668	A	AATT	intronic	*ANKRD26*	.	.	.
3	15839573	G	A	UTR5	*ANKRD28*	.	.	.
2	132954328	C	T	ncRNA_intronic	*ANKRD30BL*	.	.	.
2	97870549	T	C	intronic	*ANKRD36*	.	.	.
22	17231795	C	CA	intergenic	*ANKRD62P1-PARP4P3,XKR3*	.	.	.

4	169075132	TACAC	T	intronic	*ANXA10*	.	.	.
11	129961609	G	A	intronic	*APLP2*	.	.	.
2	21293067	G	A	intergenic	*APOB,TDRD15*	.	.	.
15	32920522	C	T	intronic	*ARHGAP11A*	.	.	.
2	144174404	A	C	intronic	*ARHGAP15*	.	.	.
2	144174406	G	C	intronic	*ARHGAP15*	.	.	.
1	157030274	A	G	intergenic	*ARHGEF11,ETV3L*	.	.	.
3	36378478	A	C	intergenic	*ARPP21,STAC*	.	.	.
5	91450137	T	TGTGTGTGC	intergenic	*ARRDC3-AS1,NR2F1-AS1*	.	.	.
X	25220802	G	T	intergenic	*ARX,MAGEB18*	.	.	.
X	1816479	G	C	intergenic	*ASMT,DHRSX*	.	.	.
9	120294568	A	G	intergenic	*ASTN2,LOC101928797*	.	.	.
5	160210391	TATATATATATGTGATGGATATATCTATCCATGGATATATCCATGGATAGATATATCCATCACATATATATATATATATC	T	intronic	*ATP10B*	.	.	.
2	176638230	C	T	intergenic	*ATP5G3,KIAA1715*	.	.	.
10	117746857	AGC	A	intergenic	*ATRNL1,GFRA1*	.	.	.
11	129441277	T	C	intergenic	*BARX2,LINC01395*	.	.	.
1	212883444	A	C	intergenic	*BATF3,NSL1*	.	.	.
7	33656105	GTATA	G	intergenic	*BBS9,BMPER*	.	.	.
20	52638824	TTGTG	T	intronic	*BCAS1*	.	.	.
2	112045999	G	A	intergenic	*BCL2L11,MIR4435-1*	.	.	.
X	70605495	G	A	ncRNA_intronic	*BCYRN1*	.	.	.
4	42155657	G	C	upstream	*BEND4*	.	.	.
20	6795484	T	A	intergenic	*BMP2,LINC01428*	.	.	.
10	47348456	T	A	intergenic	*BMS1P6,FAM35DP*	.	.	.

5	44591793	A	AT	intergenic	*BRCAT107,BRCAT54*	.	.	.
11	8955496	G	GGTTT	upstream	*C11orf16*	.	.	.
11	38530602	G	T	intergenic	*C11orf74,LOC103312105*	.	.	.
15	39337551	T	C	intergenic	*C15orf53,C15orf54*	.	.	.
17	21547770	G	GAGAAAACA	intergenic	*C17orf51,FAM27L*	.	.	.
17	21668797	G	C	intergenic	*C17orf51,FAM27L*	.	.	.
17	21523790	C	CTG	intergenic	*C17orf51,FAM27L*	.	.	.
1	226768375	G	T	intronic	*C1orf95*	.	.	.
20	19104413	C	T	intergenic	*C20orf78,SLC24A3*	.	.	.
12	22772397	A	G	intergenic	*C2CD5,ETNK1*	.	.	.
4	81681090	C	T	intronic	*C4orf22*	.	.	.
5	147435049	A	G	intergenic	*C5orf46,SPINK5*	.	.	.
7	139075488	G	C	intronic	*C7orf55-LUC7L2,LUC7L2*	.	.	.
7	108712992	CACAT	C	intergenic	*C7orf66,EIF3IP1*	.	.	.
8	69254917	C	T	intronic	*C8orf34*	.	.	.
9	97531236	A	G	intronic	*C9orf3*	.	.	.
9	74578551	T	G	intronic	*C9orf85*	.	.	.
3	86093313	C	T	intronic	*CADM2*	.	.	.
7	122564976	CATAT	C	intergenic	*CADPS2,TAS2R16*	.	.	.
6	7334990	T	A	intronic	*CAGE1*	.	.	.
1	7608694	T	C	intronic	*CAMTA1*	.	.	.
6	92746222	C	T	intergenic	*CASC6,EPHA7*	.	.	.
20	54745992	C	A	intergenic	*CBLN4,MC3R*	.	.	.
17	79635283	G	A	intronic	*CCDC137*	.	.	.
9	15767820	T	TCC	intronic	*CCDC171*	.	.	.
8	130498633	T	C	ncRNA_intronic	*CCDC26*	.	.	.
17	34730359	A	G	intergenic	*CCL4L1,TBC1D3H*	.	.	.
7	64633083	TA	T	intergenic	*CCT6P3,ZNF92*	.	.	.
5	22005841	A	G	intronic	*CDH12*	.	.	.
5	22091306	GTATA	G	intronic	*CDH12*	.	.	.
18	26221005	G	GTATTATAA	intergenic	*CDH2,MIR302F*	.	.	.
18	64103447	C	T	intergenic	*CDH7,CDH19*	.	.	.

2	202747370	GTGTGTGTGTGCTCGTGCACATGAGTGCACGAGCA	G	intronic	*CDK15*	.	.	.
17	20483016	C	T	upstream	*CDRT15L2*	.	.	.
X	100455119	A	T	intergenic	*CENPI,DRP2*	.	.	.
1	243347664	C	CTA	intronic	*CEP170*	.	.	.
5	98254203	G	A	intronic	*CHD1*	.	.	.
15	20514329	G	GATGAGATG	intergenic	*CHEK2P2,HERC2P3*	.	.	.
15	20563427	T	TCCTCTCCTC	intergenic	*CHEK2P2,HERC2P3*	.	.	.
21	19300548	C	G	intronic	*CHODL*	.	.	.
16	23843270	A	AAAAACC	intergenic	*CHP2,PRKCB*	.	.	.
2	122362710	A	C	intronic	*CLASP1*	.	.	.
13	96166664	CAGGAG	C	ncRNA_intronic	*CLDN10-AS1*	.	.	.
7	90075534	A	G	intergenic	*CLDN12,CDK14*	.	.	.
11	123185011	A	C	intergenic	*CLMP,MIR4493*	.	.	.
3	2479045	G	A	intronic	*CNTN4*	.	.	.
3	2576521	T	TGCTGCTGC	intronic	*CNTN4*	.	.	.
3	1941659	G	A	intergenic	*CNTN6,CNTN4*	.	.	.
3	2048560	A	G	intergenic	*CNTN6,CNTN4*	.	.	.
2	125543917	AAAGAAAGAAAGG	A	intronic	*CNTNAP5*	.	.	.
2	126389042	C	G	intergenic	*CNTNAP5,GYPC*	.	.	.
2	125890430	C	G	intergenic	*CNTNAP5,GYPC*	.	.	.
7	52478546	C	A	intergenic	*COBL,POM121L12*	.	.	.
8	139999267	G	A	intergenic	*COL22A1,KCNK9*	.	.	.
X	107702793	C	A	intronic	*COL4A5*	.	.	.
5	175356968	T	G	intergenic	*CPLX2,THOC3*	.	.	.
X	88844768	G	A	intergenic	*CPXCR1,TGIF2LX*	.	.	.
13	21008752	A	C	intronic	*CRYL1*	.	.	.
8	3734995	C	A	intronic	*CSMD1*	.	.	.
8	3106761	T	G	intronic	*CSMD1*	.	.	.
8	4153596	G	A	intronic	*CSMD1*	.	.	.
8	4957918	C	A	intergenic	*CSMD1,LOC100287015*	.	.	.

8	114042703	T	A	intronic	*CSMD3*	.	.	.
8	114921930	T	G	intergenic	*CSMD3,TRPS1*	.	.	.
8	115681326	CT	C	intergenic	*CSMD3,TRPS1*	.	.	.
13	38003311	A	C	intergenic	*CSNK1A1L,LINC01048*	.	.	.
X	153826753	T	A	intergenic	*CTAG1B,CTAG1B*	.	.	.
5	99216344	A	T	intergenic	*CTD-2151A2.1,LOC100133050*	.	.	.
5	99657568	G	A	intergenic	*CTD-2151A2.1,LOC100133050*	.	.	.
5	2082462	A	T	intergenic	*CTD-2194D22.4,LOC100506858*	.	.	.
5	2029718	GTGTA	G	intergenic	*CTD-2194D22.4,LOC100506858*	.	.	.
2	80430244	C	A	intronic	*CTNNA2*	.	.	.
10	69015076	A	AAATT	intronic	*CTNNA3*	.	.	.
2	181018432	G	C	intergenic	*CWC22,SCHLAP1*	.	.	.
11	107246682	C	T	intronic	*CWF19L2*	.	.	.
4	49654013	C	T	intergenic	*CWH43,NONE*	.	.	.
4	49163467	G	A	intergenic	*CWH43,NONE*	.	.	.
17	4639564	G	GAAAT	intronic	*CXCL16*	.	.	.
2	137384373	G	T	intergenic	*CXCR4,THSD7B*	.	.	.
X	30621538	G	T	intergenic	*CXorf21,GK*	.	.	.
18	47898174	A	C	intergenic	*CXXC1,SKA1*	.	.	.
X	47650340	A	G	intergenic	*CXXC1P1,ZNF81*	.	.	.
10	96648717	G	T	intergenic	*CYP2C19,CYP2C9*	.	.	.
X	77583808	C	A	upstream	*CYSLTR1*	.	.	.
21	24808703	T	TGC	intergenic	*D21S2088E,LOC101927869*	.	.	.
X	114997959	C	T	ncRNA_intronic	*DANT2*	.	.	.
X	125769547	A	C	intergenic	*DCAF12L1,PRR32*	.	.	.
18	50112448	G	A	intronic	*DCC*	.	.	.
18	50394421	ATATGTGTGTG	A	intronic	*DCC*	.	.	.

18	50711929	C	A	intronic	*DCC*	.	.	.
18	50653189	G	A	intronic	*DCC*	.	.	.
6	24275581	T	G	intronic	*DCDC2*	.	.	.
12	91582780	C	CCAGT	intergenic	*DCN,LINC01619*	.	.	.
12	92081452	A	C	intergenic	*DCN,LINC01619*	.	.	.
8	7017586	T	C	intergenic	*DEFA5,LINC00965*	.	.	.
8	6747430	CAT	C	intergenic	*DEFB1,DEFA6*	.	.	.
1	244881963	A	AATAT	intergenic	*DESI2,COX20*	.	.	.
10	826463	AGGACAGGAATCAGGTCTACAGTGCAGGGTCTGTGG	A	intergenic	*DIP2C,LARP4B*	.	.	.
2	233196946	GGA	G	intronic	*DIS3L2*	.	.	.
15	41239455	TATACAC	T	intergenic	*DLL4,CHAC1*	.	.	.
X	32406735	C	T	intronic	*DMD*	.	.	.
X	31970388	T	A	intronic	*DMD*	.	.	.
X	32373730	T	A	intronic	*DMD*	.	.	.
X	32373731	C	A	intronic	*DMD*	.	.	.
X	33693540	T	G	intergenic	*DMD,FAM47A*	.	.	.
X	33662016	A	G	intergenic	*DMD,FAM47A*	.	.	.
9	1311995	G	T	intergenic	*DMRT2,SMARCA2*	.	.	.
2	84877962	A	G	intronic	*DNAH6*	.	.	.
2	230407197	G	A	intronic	*DNER*	.	.	.
10	101695155	C	T	ncRNA_intronic	*DNMBP-AS1*	.	.	.
2	226130445	C	G	intergenic	*DOCK10,NYAP2*	.	.	.
2	225944322	C	T	intergenic	*DOCK10,NYAP2*	.	.	.
3	51067954	C	A	intronic	*DOCK3*	.	.	.
20	53429052	C	A	intergenic	*DOK5,LINC01441*	.	.	.
20	53430484	C	A	intergenic	*DOK5,LINC01441*	.	.	.
6	83860921	G	A	intronic	*DOPEY1*	.	.	.
2	115539905	AATAT	A	intronic	*DPP10*	.	.	.
2	116729485	G	T	intergenic	*DPP10,DDX18*	.	.	.
7	153670271	C	G	intronic	*DPP6*	.	.	.
12	64048618	GA	G	intronic	*DPY19L2*	.	.	.
8	29451825	AGATAGATATAGTGTGTGTGT	A	intergenic	*DUSP4,LINC00589*	.	.	.

19	57680837	GTA	G	intergenic	*DUXA,ZNF264*	.	.	.
14	102526980	T	A	intergenic	*DYNC1H1,HSP90AA1*	.	.	.
X	66403360	C	A	intergenic	*EDA2R,AR*	.	.	.
5	83779731	GA	G	intergenic	*EDIL3,NBPF22P*	.	.	.
5	84346599	G	T	intergenic	*EDIL3,NBPF22P*	.	.	.
5	84621064	GT	G	intergenic	*EDIL3,NBPF22P*	.	.	.
5	84621066	GT	G	intergenic	*EDIL3,NBPF22P*	.	.	.
13	78498831	G	A	intronic	*EDNRB*	.	.	.
13	78437432	T	C	ncRNA_intronic	*EDNRB-AS1*	.	.	.
2	233532250	A	AATTT	intronic	*EFHD1*	.	.	.
X	68156088	G	T	intergenic	*EFNB1,PJA1*	.	.	.
2	99989086	C	A	intronic	*EIF5B*	.	.	.
17	13239284	C	T	intergenic	*ELAC2,HS3ST3A1*	.	.	.
9	24508234	A	G	intergenic	*ELAVL2,IZUMO3*	.	.	.
14	106209604	C	T	intergenic	*ELK2AP,MIR4507*	.	.	.
14	106267179	G	T	intergenic	*ELK2AP,MIR4507*	.	.	.
1	121479907	C	G	intergenic	*EMBP1,NONE*	.	.	.
2	42552741	T	TA	intronic	*EML4*	.	.	.
16	33837517	A	T	intergenic	*ENPP7P13,LINC00273*	.	.	.
16	33921282	C	A	intergenic	*ENPP7P13,LINC00273*	.	.	.
16	33950642	C	G	intergenic	*ENPP7P13,LINC00273*	.	.	.
16	33953518	G	A	intergenic	*ENPP7P13,LINC00273*	.	.	.
7	38077357	G	GTATA	intergenic	*EPDR1,STARD3NL*	.	.	.
2	212783010	G	T	intronic	*ERBB4*	.	.	.
1	216742500	G	GAGGGA	intronic	*ESRRG*	.	.	.
X	93892932	A	T	intergenic	*FAM133A,BRDTP1*	.	.	.
X	93741539	C	CA	intergenic	*FAM133A,BRDTP1*	.	.	.
8	139438375	G	GTA	intronic	*FAM135B*	.	.	.
13	108363684	G	GTTTA	intronic	*FAM155A*	.	.	.
20	25744823	G	A	ncRNA_intronic	*FAM182B*	.	.	.
15	29456318	T	A	intronic	*FAM189A1*	.	.	.
22	49019627	A	T	intronic	*FAM19A5*	.	.	.

9	38997713	G	A	intergenic	*FAM201A,CNTNAP3*	.	.	.
9	38951321	GA	G	intergenic	*FAM201A,CNTNAP3*	.	.	.
X	79702170	T	C	intergenic	*FAM46D,BRWD3*	.	.	.
20	49257383	G	T	intronic	*FAM65C*	.	.	.
1	206174739	G	A	intergenic	*FAM72C,AVPR1B*	.	.	.
12	8490001	T	C	intergenic	*FAM86FP,LINC00937*	.	.	.
11	92221628	G	C	intronic	*FAT3*	.	.	.
6	99725327	T	TCATTAAAA/	UTR3	*FAXC*	.	.	.
5	108039734	CACACACACACAT	C	intergenic	*FBXL17,LINC01023*	.	.	.
2	48128227	TATATATACATATATATGTATATATGTACACACGTGTGTACATGTATATATATACACACGTGTGTAC	T	intronic	*FBXO11*	.	.	.
14	41401853	A	C	intergenic	*FBXO33,LOC644919*	.	.	.
14	40913784	G	T	intergenic	*FBXO33,LOC644919*	.	.	.
14	40981180	G	T	intergenic	*FBXO33,LOC644919*	.	.	.
3	60913944	C	T	intronic	*FHIT*	.	.	.
3	60574778	C	T	intronic	*FHIT*	.	.	.
2	164796779	CTGTG	C	intergenic	*FIGN,GRB14*	.	.	.
16	27282880	G	GCA	ncRNA_intronic	*FLJ21408*	.	.	.
9	68729743	C	T	ncRNA_intronic	*FRG1HP*	.	.	.
10	135460997	C	T	intergenic	*FRG2B,NONE*	.	.	.
9	108299155	T	G	intronic	*FSD1L*	.	.	.
4	46062274	ATGTGTG	A	intronic	*GABRG1*	.	.	.
4	46179794	A	C	intergenic	*GABRG1,GABRA2*	.	.	.
5	161637812	C	A	intergenic	*GABRG2,CCNG1*	.	.	.

5	162086126	A	C	intergenic	*GABRG2,CCNG1*	.	.	.
5	162677222	A	T	intergenic	*GABRG2,CCNG1*	.	.	.
5	161666653	T	G	intergenic	*GABRG2,CCNG1*	.	.	.
X	49220460	T	G	intronic	*GAGE12I,GAGE12J,GAGE13,GAGE2A,GAGE2C,GAGE2D,GAGE2E,GAGE4,GAGE5,GAGE7,GAGE8*	.	.	.
X	49203622	TTTTATTTA	T	intronic	*GAGE12J,GAGE13,GAGE2A,GAGE2C,GAGE2D,GAGE2E,GAGE8*	.	.	.
4	173145241	C	G	intronic	*GALNTL6*	.	.	.
5	37913947	G	C	intergenic	*GDNF-AS1,EGFLAM*	.	.	.
2	91964190	C	A	ncRNA_intronic	*GGT8P*	.	.	.
7	150338023	G	T	intergenic	*GIMAP6,GIMAP2*	.	.	.
6	121794163	T	A	intergenic	*GJA1,HSF2*	.	.	.
9	4042714	C	A	intronic	*GLIS3*	.	.	.
12	129466395	C	A	intronic	*GLT1D1*	.	.	.
15	75593063	A	G	intergenic	*GOLGA6D,COMMD4*	.	.	.
15	84921888	T	A	intergenic	*GOLGA6L4,LOC103171574*	.	.	.
15	84921889	G	A	intergenic	*GOLGA6L4,LOC103171574*	.	.	.
15	29090595	C	T	ncRNA_exonic	*GOLGA6L7P*	.	.	.
13	92224043	GTGTGTGTATATATACACACACATACGTGTGTGTATATATACACATATACT	G	intronic	*GPC5*	.	.	.
13	92956254	T	C	intronic	*GPC5*	.	.	.
13	94916244	G	GCTA	intronic	*GPC6*	.	.	.
X	136194432	T	C	intergenic	*GPR101,ZIC3*	.	.	.

X	136291983	GTCTC	G	intergenic	*GPR101,ZIC3*	.	.	.
X	136400625	C	A	intergenic	*GPR101,ZIC3*	.	.	.
X	136196726	A	G	intergenic	*GPR101,ZIC3*	.	.	.
2	11776929	G	A	intronic	*GREB1*	.	.	.
4	93672081	C	T	intronic	*GRID2*	.	.	.
6	104462982	C	A	intergenic	*GRIK2,HACE1*	.	.	.
11	120486926	C	G	intronic	*GRIK4*	.	.	.
12	14198852	C	A	intergenic	*GRIN2B,ATF7IP*	.	.	.
9	104756925	C	CATAT	intergenic	*GRIN3A,LINC0058 7*	.	.	.
6	146524328	AAAATAAA T	A	intronic	*GRM1*	.	.	.
4	43219257	G	T	intergenic	*GRXCR1,LVCAT1*	.	.	.
4	145032099	T	TAATTTATTT	UTR3	*GYPA*	.	.	.
X	103258448	T	C	intergenic	*H2BFXP,H2BFWT*	.	.	.
19	24619567	C	G	intergenic	*HAVCR1P1,NONE*	.	.	.
5	156554303	A	C	intergenic	*HAVCR2,MED7*	.	.	.
5	45295183	C	A	intronic	*HCN1*	.	.	.
5	46259730	G	T	intergenic	*HCN1,NONE*	.	.	.
5	46401492	C	G	intergenic	*HCN1,NONE*	.	.	.
X	84183623	C	G	intergenic	*HDX,UBE2DNL*	.	.	.
15	28599774	C	T	intergenic	*HERC2,GOLGA8G*	.	.	.
1	934142	A	AGGGGAGG	downstream	*HES4*	.	.	.
1	70869659	G	C	intergenic	*HHLA3,CTH*	.	.	.
1	120923116	T	C	intergenic	*HIST2H2BA,FCGR1 B*	.	.	.
6	12067099	C	CTGTGTG	intronic	*HIVEP1*	.	.	.
6	55424799	T	C	intronic	*HMGCLL1*	.	.	.
1	120321072	C	T	intergenic	*HMGCS2,REG4*	.	.	.
X	133594088	A	T	upstream	*HPRT1*	.	.	.
16	23063282	G	C	intergenic	*HS3ST2,USP31*	.	.	.
17	14873949	G	C	intergenic	*HS3ST3B1,CDRT7*	.	.	.
16	26167411	G	T	intergenic	*HS3ST4,C16orf82*	.	.	.
16	26542977	G	T	intergenic	*HS3ST4,C16orf82*	.	.	.
22	17364621	A	G	intergenic	*HSFY1P1,GAB4*	.	.	.
22	17364622	T	C	intergenic	*HSFY1P1,GAB4*	.	.	.
22	17350083	C	CA	intergenic	*HSFY1P1,GAB4*	.	.	.
7	154865092	G	A	intronic	*HTR5A*	.	.	.

6	19912127	G	GTAGA	intergenic	*ID4,MBOAT1*	.	.	.
9	21195454	T	G	intergenic	*IFNA4,IFNA7*	.	.	.
1	18503899	G	A	intronic	*IGSF21*	.	.	.
X	105050626	ATCTTGAG TTAAT	A	intergenic	*IL1RAPL2,NRK*	.	.	.
8	28738812	G	A	intronic	*INTS9*	.	.	.
5	51370516	T	C	intergenic	*ISL1,PELO*	.	.	.
3	124438998	C	A	UTR3	*KALRN*	.	.	.
15	34476506	C	CAAAAAAAA	intronic	*KATNBL1*	.	.	.
10	78968582	T	G	intronic	*KCNMA1*	.	.	.
6	73345398	G	GTTTTTTTTT	ncRNA_intronic	*KCNQ5-IT1*	.	.	.
8	36704753	C	A	intronic	*KCNU1*	.	.	.
8	111590925	A	G	intergenic	*KCNV1,LINC01608*	.	.	.
8	111237993	C	T	intergenic	*KCNV1,LINC01608*	.	.	.
18	24117981	C	T	intronic	*KCTD1*	.	.	.
5	144179624	T	A	intergenic	*KCTD16,PRELID2*	.	.	.
5	143896217	A	T	intergenic	*KCTD16,PRELID2*	.	.	.
4	44359919	TATA	T	intronic	*KCTD8*	.	.	.
8	137339168	TTCTC	T	intergenic	*KHDRBS3,LOC101 927915*	.	.	.
4	6827299	CGAGA	C	intronic	*KIAA0232*	.	.	.
5	93865959	A	C	intronic	*KIAA0825*	.	.	.
X	118283249	C	T	intronic	*KIAA1210*	.	.	.
10	24397091	G	C	intronic	*KIAA1217*	.	.	.
6	17875189	A	T	intronic	*KIF13A*	.	.	.
6	17875190	T	TTTTTTTTTT	intronic	*KIF13A*	.	.	.
17	52897648	A	C	intergenic	*KIF2B,TOM1L1*	.	.	.
10	32364413	C	CAAAAAAAA	intergenic	*KIF5B,EPC1*	.	.	.
4	55625395	A	ATATATATAT	intergenic	*KIT,KDR*	.	.	.

15	31715285	TATATATACGTATATATACGTATACGTATACGTATATATACGTATATATACGTATACGTATACGTATATATACGTATATATATGTATATATATAC	T	intronic	*KLF13*	.	.	.
9	111135098	T	TTTA	intergenic	*KLF4,ACTL7B*	.	.	.
17	20428998	A	AG	intergenic	*KRT16P3,CDRT15L2*	.	.	.
11	71434062	A	AC	intergenic	*KRTAP5-11,FAM86C1*	.	.	.
12	118321452	C	T	intronic	*KSR2*	.	.	.
6	129521232	TATATACACGTGTATATGTGTACACGTATATATATGTGTATATATATACGTGTACAC	T	intronic	*LAMA2*	.	.	.
22	35241442	TC	T	intergenic	*LARGE,ISX*	.	.	.
4	129021657	G	A	intronic	*LARP1B*	.	.	.
4	18287687	T	C	intergenic	*LCORL,SLIT2*	.	.	.
4	16632948	T	A	intronic	*LDB2*	.	.	.
4	16676410	GCACA	G	intronic	*LDB2*	.	.	.
4	109257098	T	C	intergenic	*LEF1-AS1,RPL34-AS1*	.	.	.
1	66137457	G	A	intergenic	*LEPR,PDE4B*	.	.	.
19	55141944	T	C	UTR5	*LILRB1*	.	.	.
15	88227152	G	C	intergenic	*LINC00052,NTRK3*	.	.	.
21	24402211	T	A	intergenic	*LINC00308,D21S2088E*	.	.	.

21	24606480	A	G	intergenic	*LINC00308,D21S2088E*	.	.	.
21	24385857	C	A	intergenic	*LINC00308,D21S2088E*	.	.	.
13	90475578	GTATATA	G	intergenic	*LINC00353,LINC00559*	.	.	.
13	51748799	T	C	intergenic	*LINC00371,FAM124A*	.	.	.
13	22557813	T	C	intergenic	*LINC00424,LINC00540*	.	.	.
13	87701107	G	A	intergenic	*LINC00430,MIR4500HG*	.	.	.
13	63645938	G	A	intergenic	*LINC00448,LINC00376*	.	.	.
18	1451044	C	A	intergenic	*LINC00470,METTL4*	.	.	.
5	102177909	AATAT	A	intergenic	*LINC00491,PAM*	.	.	.
10	92898026	TACAC	T	intergenic	*LINC00502,NUDT9P1*	.	.	.
10	92883433	A	AG	intergenic	*LINC00502,NUDT9P1*	.	.	.
13	54447810	A	C	ncRNA_exonic	*LINC00558*	.	.	.
6	170454418	A	G	intergenic	*LINC00574,LOC102724511*	.	.	.
X	139858691	C	T	intergenic	*LINC00632,CDR1*	.	.	.
14	62718171	C	A	intergenic	*LINC00644,KCNH5*	.	.	.
14	63062325	C	A	intergenic	*LINC00644,KCNH5*	.	.	.
8	12723214	A	C	intergenic	*LINC00681,KIAA1456*	.	.	.
10	34173540	C	G	intergenic	*LINC00838,PARD3*	.	.	.
10	44638283	AATAT	A	intergenic	*LINC00841,C10orf142*	.	.	.
10	91648379	G	GC	intergenic	*LINC00865,LINC01375*	.	.	.
1	149646040	C	G	ncRNA_intronic	*LINC00869,LOC103091866*	.	.	.
11	116044017	G	A	intergenic	*LINC00900,BUD13*	.	.	.

18	74314003	C	G	intergenic	*LINC00908,LINC00683*	.	.	.
15	96505196	GGTGT	G	intergenic	*LINC00924,NR2F2-AS1*	.	.	.
12	90661247	C	T	intergenic	*LINC00936,LINC00615*	.	.	.
10	38925209	A	G	intergenic	*LINC00999,ACTR3BP5*	.	.	.
7	128281741	A	G	ncRNA_intronic	*LINC01000*	.	.	.
5	28008648	A	G	intergenic	*LINC01021,LSP1P3*	.	.	.
5	28406671	A	G	intergenic	*LINC01021,LSP1P3*	.	.	.
5	28605913	C	G	intergenic	*LINC01021,LSP1P3*	.	.	.
5	27924735	G	C	intergenic	*LINC01021,LSP1P3*	.	.	.
1	194657808	TTATA	T	intergenic	*LINC01031,KCNT2*	.	.	.
1	195129575	G	GTA	intergenic	*LINC01031,KCNT2*	.	.	.
13	91409725	G	A	intergenic	*LINC01049,LINC00410*	.	.	.
4	190201125	A	ACT	intergenic	*LINC01060,LINC01262*	.	.	.
13	53905242	G	A	intergenic	*LINC01065,LINC00558*	.	.	.
4	180082350	C	CTCTCTA	intergenic	*LINC01098,LINC00290*	.	.	.
4	180147362	C	A	intergenic	*LINC01098,LINC00290*	.	.	.
1	170295878	G	A	intergenic	*LINC01142,LOC101928650*	.	.	.
2	115127184	T	A	intergenic	*LINC01191,DPP10*	.	.	.
5	12669824	T	G	ncRNA_intronic	*LINC01194*	.	.	.
9	30864374	C	G	intergenic	*LINC01242,ACO1*	.	.	.
2	4708414	G	A	intergenic	*LINC01249,LINC01248*	.	.	.
2	2989580	TACAC	T	ncRNA_intronic	*LINC01250*	.	.	.
4	190581064	T	TA	ncRNA_intronic	*LINC01262*	.	.	.

4	190658943	A	AATCT	intergenic	*LINC01262,LINC01596*	.	.	.
4	190626671	G	GCGACCACC	intergenic	*LINC01262,LINC01596*	.	.	.
X	39193450	A	ATTGTGTCT/	intergenic	*LINC01281,LINC01282*	.	.	.
X	39454719	T	A	intergenic	*LINC01282,MIR3937*	.	.	.
8	66497641	C	A	intergenic	*LINC01299,ARMC1*	.	.	.
8	61348050	C	A	intergenic	*LINC01301,RAB2A*	.	.	.
1	102113412	C	A	intergenic	*LINC01307,OLFM3*	.	.	.
13	105641320	C	G	intergenic	*LINC01309,DAOA-AS1*	.	.	.
2	34652775	C	CT	intergenic	*LINC01317,LINC01320*	.	.	.
2	34769919	C	T	intergenic	*LINC01317,LINC01320*	.	.	.
2	34907467	A	AAAATAAAT	ncRNA_intronic	*LINC01320*	.	.	.
2	35533950	C	A	intergenic	*LINC01320,LOC100288911*	.	.	.
5	97574126	C	T	intergenic	*LINC01340,RGMB*	.	.	.
5	98083550	C	A	intergenic	*LINC01340,RGMB*	.	.	.
1	243249053	A	G	ncRNA_intronic	*LINC01347*	.	.	.
1	185551693	C	T	ncRNA_intronic	*LINC01350*	.	.	.
7	67313262	C	A	intergenic	*LINC01372,LOC102723427*	.	.	.
7	67424554	A	G	intergenic	*LINC01372,LOC102723427*	.	.	.
5	173903480	G	T	ncRNA_intronic	*LINC01411*	.	.	.
8	84459363	T	A	intergenic	*LINC01419,RALYL*	.	.	.
8	84587273	C	A	intergenic	*LINC01419,RALYL*	.	.	.
X	56813074	TC	T	ncRNA_intronic	*LINC01420*	.	.	.
X	56813076	TTTTTC	T	ncRNA_intronic	*LINC01420*	.	.	.
21	23146428	G	GAAACCCCG	ncRNA_intronic	*LINC01425*	.	.	.
20	22264130	C	T	upstream	*LINC01427*	.	.	.
7	54509599	A	T	intergenic	*LINC01445,VSTM2A*	.	.	.

7	53758806	G	A	ncRNA_intronic	*LINC01446*	.	.	.
7	54216043	A	T	intergenic	*LINC01446,HPVC1*	.	.	.
14	82621137	A	ATATATATAT	intergenic	*LINC01467,NONE*	.	.	.
14	82860279	T	C	intergenic	*LINC01467,NONE*	.	.	.
18	38798519	G	T	intergenic	*LINC01477,KC6*	.	.	.
11	39848714	G	A	intergenic	*LINC01493,LRRC4C*	.	.	.
11	39102997	A	ATAT	intergenic	*LINC01493,LRRC4C*	.	.	.
9	84056231	G	GT	intergenic	*LINC01507,TLE1*	.	.	.
9	83538253	G	C	intergenic	*LINC01507,TLE1*	.	.	.
18	69247862	T	C	intergenic	*LINC01541,LOC102724913*	.	.	.
14	99045234	G	C	intergenic	*LINC01550,C14orf177*	.	.	.
X	47400050	TAAA	T	intergenic	*LINC01560,ARAF*	.	.	.
16	72360415	C	A	ncRNA_intronic	*LINC01572*	.	.	.
16	72616349	C	G	ncRNA_intronic	*LINC01572*	.	.	.
15	94705340	GTATGTATATATGTATATATACATGTATATATATGTGTATATATACATGTATATATATGTATATATGTATGTATA	G	intergenic	*LINC01581,MCTP2*	.	.	.
4	190804459	T	TTCTGC	ncRNA_intronic	*LINC01596*	.	.	.
8	113224777	G	T	intergenic	*LINC01609,CSMD3*	.	.	.
6	85143007	C	A	ncRNA_intronic	*LINC01611*	.	.	.
6	80548843	C	A	intergenic	*LINC01621,ELOVL4*	.	.	.
6	72513986	A	T	intergenic	*LINC01626,RIMS1*	.	.	.
10	38642700	T	G	intergenic	*LOC100129055,HSD17B7P2*	.	.	.

14	98258857	ATT	A	intergenic	*LOC100129345,LINC01550*	.	.	.
7	56948613	G	A	ncRNA_intronic	*LOC100130849*	.	.	.
6	140177617	C	T	ncRNA_intronic	*LOC100132735*	.	.	.
9	69701480	TTGA	T	intergenic	*LOC100133920,LOC101928381*	.	.	.
5	50493148	AATATAT	A	intergenic	*LOC100287592,LOC642366*	.	.	.
12	106923539	GTATA	G	ncRNA_intronic	*LOC100287944*	.	.	.
1	745305	CTAAA	C	intergenic	*LOC100288069,FAM87B*	.	.	.
1	726041	G	T	intergenic	*LOC100288069,FAM87B*	.	.	.
5	98732125	G	A	intergenic	*LOC100289230,CTD-2151A2.1*	.	.	.
1	164093570	C	CTTT	intergenic	*LOC100422212,PBX1*	.	.	.
11	4336792	T	A	intergenic	*LOC100506082,OR52B4*	.	.	.
12	20354272	T	A	intergenic	*LOC100506393,PDE3A*	.	.	.
2	13989804	T	C	intergenic	*LOC100506474,LINC00276*	.	.	.
8	12314896	C	A	ncRNA_intronic	*LOC100506990*	.	.	.
8	12314898	G	A	ncRNA_intronic	*LOC100506990*	.	.	.
11	43026624	G	A	intergenic	*LOC100507205,HNRNPKP3*	.	.	.
1	96183655	G	T	intergenic	*LOC100996635,LOC102723661*	.	.	.
19	28756328	C	A	intergenic	*LOC101927151,LOC100420587*	.	.	.
19	28836942	C	G	intergenic	*LOC101927151,LOC100420587*	.	.	.
19	28848430	C	G	intergenic	*LOC101927151,LOC100420587*	.	.	.
6	98319358	A	AAACAACAA	intergenic	*LOC101927314,MIR2113*	.	.	.

2	195474747	G	C	intergenic	*LOC101927406,LOC101927431*	.	.	.
10	108213989	C	A	intergenic	*LOC101927549,SORCS1*	.	.	.
9	86816038	TG	T	intergenic	*LOC101927575,SLC28A3*	.	.	.
4	150165590	A	G	ncRNA_intronic	*LOC101927849*	.	.	.
2	129997761	T	G	intergenic	*LOC101927881,LOC151121*	.	.	.
4	162957686	A	G	ncRNA_intronic	*LOC101928052*	.	.	.
3	35239638	ATGTG	A	ncRNA_intronic	*LOC101928135*	.	.	.
12	73769030	A	C	intergenic	*LOC101928137,LOC100507377*	.	.	.
X	4608801	C	A	intergenic	*LOC101928201,NLGN4X*	.	.	.
10	9985528	C	T	intergenic	*LOC101928272,LOC101928298*	.	.	.
7	125277719	A	T	intergenic	*LOC101928283,GRM8*	.	.	.
10	10122480	G	C	intergenic	*LOC101928298,LOC101928322*	.	.	.
2	88859411	T	G	ncRNA_intronic	*LOC101928371*	.	.	.
9	69755906	T	C	ncRNA_intronic	*LOC101928381*	.	.	.
9	69922226	A	AG	intergenic	*LOC101928381,FOXD4L5*	.	.	.
5	57210489	T	TAGAGAG	intergenic	*LOC101928539,LOC101928569*	.	.	.
4	34029757	G	T	ncRNA_intronic	*LOC101928622*	.	.	.
4	34388668	GACACACAC	G	intergenic	*LOC101928622,ARAP2*	.	.	.
4	34953369	CATAT	C	intergenic	*LOC101928622,ARAP2*	.	.	.
4	34057521	T	G	intergenic	*LOC101928622,ARAP2*	.	.	.
4	34090876	C	T	intergenic	*LOC101928622,ARAP2*	.	.	.
4	34678408	T	G	intergenic	*LOC101928622,ARAP2*	.	.	.

14	86421035	T	C	ncRNA_intronic	*LOC101928767*	.	.	.
14	87204667	A	G	intergenic	*LOC101928767,LOC283585*	.	.	.
9	118505520	C	G	ncRNA_intronic	*LOC101928775*	.	.	.
4	58937876	GTATA	G	intergenic	*LOC101928851,NONE*	.	.	.
4	12670032	C	A	intergenic	*LOC101929019,RAB28*	.	.	.
4	12900682	TATG	T	intergenic	*LOC101929019,RAB28*	.	.	.
4	15038795	T	C	ncRNA_intronic	*LOC101929095*	.	.	.
1	119835733	GTATATA	G	intergenic	*LOC101929147,HAO2*	.	.	.
2	157163913	A	AG	intergenic	*LOC101929378,NR4A2*	.	.	.
11	126976714	G	T	intergenic	*LOC101929473,LOC101929497*	.	.	.
5	31090059	G	T	intergenic	*LOC101929681,CDH6*	.	.	.
5	106248353	GTACATATATATGTACGTATATGTATATACATATATGTATATACATATACGTACATATA	G	ncRNA_intronic	*LOC102467213*	.	.	.
5	116664537	T	G	intergenic	*LOC102467223,LINC00992*	.	.	.
5	165315114	C	G	intergenic	*LOC102546299,CTB-7E3.1*	.	.	.
5	165360991	A	C	intergenic	*LOC102546299,CTB-7E3.1*	.	.	.
4	32070999	T	TATTATAA	ncRNA_intronic	*LOC102723828*	.	.	.
4	32654768	GTATA	G	intergenic	*LOC102723828,LOC101928622*	.	.	.
8	64584163	G	T	intergenic	*LOC102724612,LINC01289*	.	.	.

8	64888209	A	T	ncRNA_intronic	*LOC102724623*	.	.	.
8	65051524	A	T	ncRNA_intronic	*LOC102724623*	.	.	.
2	192588046	C	G	intergenic	*LOC105747689,SDPR*	.	.	.
2	84060503	TATAC	T	intergenic	*LOC1720,FUNDC2P2*	.	.	.
11	134370664	G	A	ncRNA_intronic	*LOC283177*	.	.	.
11	134519087	A	C	intergenic	*LOC283177,NONE*	.	.	.
19	37764195	G	C	intergenic	*LOC284412,HKR1*	.	.	.
2	30591814	G	T	intergenic	*LOC285043,LCLAT1*	.	.	.
5	159044545	G	A	intergenic	*LOC285627,LOC101927766*	.	.	.
5	135102691	G	C	intergenic	*LOC340074,SLC25A48*	.	.	.
5	26556981	G	T	intergenic	*LOC340107,CDH9*	.	.	.
7	136871309	C	A	intergenic	*LOC349160,PTN*	.	.	.
X	4527509	C	A	intergenic	*LOC389906,LOC101928201*	.	.	.
16	33438606	C	CAAAAA	intergenic	*LOC390705,ENPP7P13*	.	.	.
16	32659092	A	ATCATT	intergenic	*LOC390705,TP53TG3*	.	.	.
16	88144187	C	T	intergenic	*LOC400553,LOC101928880*	.	.	.
18	74474058	A	C	intergenic	*LOC400661,LOC100131655*	.	.	.
5	19192608	C	T	intergenic	*LOC401177,CDH18*	.	.	.

5	17903275	TGATGTAAATTACATCAGCGTACGCTGATGTAAATTACATCAGCGTACTCTGATGTAAATTACATCAGCGTACTC	T	intergenic	*LOC401177,CDH18*	.	.	.
5	17515795	A	C	intergenic	*LOC401177,CDH18*	.	.	.
7	56918264	A	C	intergenic	*LOC401357,LOC100130849*	.	.	.
7	56936425	C	G	intergenic	*LOC401357,LOC100130849*	.	.	.
11	49716916	C	A	ncRNA_intronic	*LOC440040*	.	.	.
11	49824156	G	T	ncRNA_intronic	*LOC440040*	.	.	.
12	127355717	A	C	ncRNA_intronic	*LOC440117*	.	.	.
1	190949941	G	T	intergenic	*LOC440704,RGS18*	.	.	.
2	227310855	C	CA	intergenic	*LOC646736,MIR5702*	.	.	.
16	22654595	TGAGA	T	intergenic	*LOC653786,MIR548AA2*	.	.	.
16	60440236	CTATATGCATACATATGTACGTGTCTATATGCATACATATGTACGTGTATATGCATATATATGTACGTGTG	C	intergenic	*LOC729159,MIR4426*	.	.	.
16	60605872	C	CCAA	intergenic	*LOC729159,MIR4426*	.	.	.
18	44117469	C	A	intronic	*LOXHD1*	.	.	.
14	44169892	C	G	intergenic	*LRFN5,FSCB*	.	.	.

3	4179598	TACAC	T	intergenic	*LRRN1,SETMAR*	.	.	.
11	1888570	A	C	intronic	*LSP1*	.	.	.
7	119286855	G	C	ncRNA_intronic	*LVCAT5*	.	.	.
7	119755376	T	G	intergenic	*LVCAT5,KCND2*	.	.	.
7	119885268	AATAT	A	intergenic	*LVCAT5,KCND2*	.	.	.
2	150005931	TACAC	T	intronic	*LYPD6B*	.	.	.
1	219553020	A	ATAT	intergenic	*LYPLAL1,RNU5F-1*	.	.	.
7	1974500	T	G	intronic	*MAD1L1*	.	.	.
X	30299138	TTTATTATT ATCA	T	intergenic	*MAGEB1,NR0B1*	.	.	.
X	26321538	T	A	intergenic	*MAGEB5,VENTXP1*	.	.	.
X	26434214	A	T	intergenic	*MAGEB5,VENTXP1*	.	.	.
X	75025791	T	C	intergenic	*MAGEE2,PBDC1*	.	.	.
11	95724565	C	G	intronic	*MAML2*	.	.	.
6	91539831	TATAC	T	intergenic	*MAP3K7,MIR4643*	.	.	.
1	206917404	TTCCTTCCT TCCTTCCTT C	T	intergenic	*MAPKAPK2,IL10*	.	.	.
5	66216194	G	A	intronic	*MAST4*	.	.	.
1	31194674	G	A	ncRNA_intronic	*MATN1-AS1*	.	.	.
X	131631433	C	CTTTTGCCAT	intergenic	*MBNL3,HS6ST2*	.	.	.
X	131631435	C	CAGATAAAC	intergenic	*MBNL3,HS6ST2*	.	.	.
X	21931657	C	CAGAAAGAA	intergenic	*MBTPS2,SMS*	.	.	.
18	58312327	ATGTG	A	intergenic	*MC4R,CDH20*	.	.	.
18	58937350	GTATATA	G	intergenic	*MC4R,CDH20*	.	.	.
6	100607252	ATAT	A	intergenic	*MCHR2-AS1,SIM1*	.	.	.
5	94152727	G	A	intronic	*MCTP1*	.	.	.
14	47660622	CATAT	C	intronic	*MDGA2*	.	.	.
3	169000228	C	A	intronic	*MECOM*	.	.	.
6	78845498	C	T	intergenic	*MEI4,IRAK1BP1*	.	.	.
6	78694224	C	T	intergenic	*MEI4,IRAK1BP1*	.	.	.
15	37452800	AT	A	intergenic	*MEIS2,TMCO5A*	.	.	.

2	208507350	CCACTGCACTCCAGCCTGGGTGACAGAGTGAGACCCCTTCTCAAAAAAAAAAAAAAAATGCTTGTTATTCAAACACAGTTTTTTTTTTTTTGAGATGGAGTTTCACTCTGTCACCCAGGCTGGAGTGCAGTGGTGCGATCTCAGCT	C	intergenic	*METTL21A,CCNYL1*	.	.	.
1	40465962	T	C	intergenic	*MFSD2A,CAP1*	.	.	.
7	141785567	C	G	intronic	*MGAM*	.	.	.
12	87205946	G	GT	intronic	*MGAT4C*	.	.	.
12	87205948	C	G	intronic	*MGAT4C*	.	.	.
1	65548838	TCACA	T	intergenic	*MIR101-1,AK4*	.	.	.
6	121073527	C	T	intergenic	*MIR3144,TBC1D32*	.	.	.
13	61817162	G	T	intergenic	*MIR3169,PCDH20*	.	.	.
4	185766766	A	T	ncRNA_exonic	*MIR3945HG*	.	.	.
4	30275201	G	T	intergenic	*MIR4275,PCDH7*	.	.	.
7	63191479	G	C	intergenic	*MIR4283-2,LINC01005*	.	.	.
7	63261851	G	A	intergenic	*MIR4283-2,LINC01005*	.	.	.
18	36518395	C	A	intergenic	*MIR4318,MIR924HG*	.	.	.
18	35447390	T	A	intergenic	*MIR4318,MIR924HG*	.	.	.
18	36269074	TA	T	intergenic	*MIR4318,MIR924HG*	.	.	.

X	150342087	T	C	intergenic	*MIR4330,GPR50*	.	.	.
1	192684466	CAGAG	C	upstream	*MIR4426*	.	.	.
2	90485072	T	TGGCTGGCT	intergenic	*MIR4436A,LOC654342*	.	.	.
2	90403346	A	G	intergenic	*MIR4436A,LOC654342*	.	.	.
3	25724950	G	A	intergenic	*MIR4442,NGLY1*	.	.	.
3	109796518	C	T	intergenic	*MIR4445,PVRL3-AS1*	.	.	.
5	552372	C	G	intergenic	*MIR4456,LOC100996325*	.	.	.
6	142091636	AATAT	A	intergenic	*MIR4465,NMBR*	.	.	.
3	31291404	T	TA	intergenic	*MIR466,STT3B*	.	.	.
2	87642740	C	A	intergenic	*MIR4771-2,LINC00152*	.	.	.
2	87596661	A	ACTT	intergenic	*MIR4771-2,LINC00152*	.	.	.
3	5558660	C	T	intergenic	*MIR4790,GRM7-AS3*	.	.	.
3	5914884	A	T	intergenic	*MIR4790,GRM7-AS3*	.	.	.
3	6483761	G	T	intergenic	*MIR4790,GRM7-AS3*	.	.	.
X	146857609	G	A	intergenic	*MIR514A2,FMR1-AS1*	.	.	.
20	59200296	A	G	intergenic	*MIR548AG2,LOC100506470*	.	.	.
4	23436693	G	A	ncRNA_intronic	*MIR548AJ2*	.	.	.
7	147971457	T	G	ncRNA_intronic	*MIR548T*	.	.	.
1	35134869	C	A	downstream	*MIR552*	.	.	.
20	26294354	C	T	intergenic	*MIR663AHG,NONE*	.	.	.
8	93460485	C	A	intergenic	*MIR7641-2,LOC102724710*	.	.	.
1	228771080	A	T	intergenic	*MIR7641-2,MIR7641-2*	.	.	.
11	29796882	C	A	intergenic	*MIR8068,LINC01616*	.	.	.

14	106108691	T	A	intergenic	*MIR8071-2,ELK2AP*	.	.	.
19	751985	G	C	intronic	*MISP*	.	.	.
11	102758619	T	TTTTA	intergenic	*MMP12,MMP13*	.	.	.
21	35547038	A	T	intergenic	*MRPS6,LINC00310*	.	.	.
17	55557830	C	T	intronic	*MSI2*	.	.	.
X	64915399	A	T	intronic	*MSN*	.	.	.
3	95504477	C	G	intergenic	*MTHFD2P1,MIR8060*	.	.	.
17	22047388	G	A	intergenic	*MTRNR2L1,NONE*	.	.	.
13	29694774	G	T	intronic	*MTUS2*	.	.	.
13	29786994	G	A	intronic	*MTUS2*	.	.	.
13	109581762	T	A	intronic	*MYO16*	.	.	.
8	2236113	T	A	intergenic	*MYOM2,LOC101927815*	.	.	.
11	20003805	T	TTTTTGTTTT	intronic	*NAV2*	.	.	.
1	144836560	T	C	intronic	*NBPF20,NBPF9*	.	.	.
1	148654542	C	T	ncRNA_intronic	*NBPF25P*	.	.	.
1	148005278	G	A	intronic	*NBPF8*	.	.	.
21	22589357	C	A	intronic	*NCAM2*	.	.	.
7	72650658	C	T	downstream	*NCF1B*	.	.	.
2	232333302	C	CT	intergenic	*NCL,LINC00471*	.	.	.
X	43912962	T	G	intergenic	*NDP,EFHC2*	.	.	.
1	73465537	C	CACATAT	intergenic	*NEGR1,LINC01360*	.	.	.
1	198128766	C	A	intronic	*NEK7*	.	.	.
18	77395780	C	T	intergenic	*NFATC1,CTDP1*	.	.	.
2	219943559	T	TA	intronic	*NHEJ1*	.	.	.
X	17687643	C	A	intronic	*NHS*	.	.	.
3	173982897	T	C	intronic	*NLGN1*	.	.	.
3	173302222	T	TTTTG	intronic	*NLGN1*	.	.	.
X	6050968	C	G	intronic	*NLGN4X*	.	.	.
1	142539940	A	ATGGAGTGC	intergenic	*NONE,ANKRD20A12P*	.	.	.
15	20138019	G	T	intergenic	*NONE,CHEK2P2*	.	.	.
11	98462829	G	C	intergenic	*NONE,CNTN5*	.	.	.
Y	13423716	G	A	intergenic	*NONE,GYG2P1*	.	.	.

Y	13484184	T	C	intergenic	*NONE,GYG2P1*	.	.	.
Y	13409919	G	C	intergenic	*NONE,GYG2P1*	.	.	.
Y	13428498	G	A	intergenic	*NONE,GYG2P1*	.	.	.
Y	13473818	G	C	intergenic	*NONE,GYG2P1*	.	.	.
Y	13427753	G	A	intergenic	*NONE,GYG2P1*	.	.	.
Y	13499358	T	TA	intergenic	*NONE,GYG2P1*	.	.	.
Y	13288118	C	T	intergenic	*NONE,GYG2P1*	.	.	.
Y	13263678	C	A	intergenic	*NONE,GYG2P1*	.	.	.
8	46843261	C	T	intergenic	*NONE,LINC00293*	.	.	.
10	42702256	AG	A	intergenic	*NONE,LOC441666*	.	.	.
10	42398632	A	G	intergenic	*NONE,LOC441666*	.	.	.
10	42355426	A	C	intergenic	*NONE,LOC441666*	.	.	.
10	42622747	C	CTAAA	intergenic	*NONE,LOC441666*	.	.	.
17	25279891	G	GT	intergenic	*NONE,MIR4522*	.	.	.
17	25270277	G	A	intergenic	*NONE,MIR4522*	.	.	.
X	62176510	T	C	intergenic	*NONE,SPIN4*	.	.	.
Y	58980004	C	CTCCAATCC/	intergenic	*NONE,SPRY3*	.	.	.
Y	58989154	G	T	intergenic	*NONE,SPRY3*	.	.	.
Y	58989209	G	A	intergenic	*NONE,SPRY3*	.	.	.
Y	59001280	T	A	intergenic	*NONE,SPRY3*	.	.	.
7	61066434	T	G	intergenic	*NONE,ZNF733P*	.	.	.
7	61066436	G	A	intergenic	*NONE,ZNF733P*	.	.	.
7	61560803	T	G	intergenic	*NONE,ZNF733P*	.	.	.
7	61056488	C	CTAA	intergenic	*NONE,ZNF733P*	.	.	.
6	156009385	C	G	intergenic	*NOX3,MIR1202*	.	.	.
11	89125014	C	T	intronic	*NOX4*	.	.	.
8	54046652	T	G	intergenic	*NPBWR1,OPRK1*	.	.	.
8	54110595	C	G	intergenic	*NPBWR1,OPRK1*	.	.	.
16	14909095	G	C	intergenic	*NPIPA3,ABCC6P2*	.	.	.
16	17163100	C	CACACACAT	intergenic	*NPIPA8,XYLT1*	.	.	.
16	16717121	G	T	intergenic	*NPIPA8,XYLT1*	.	.	.
7	98305467	G	C	intergenic	*NPTX2,TMEM130*	.	.	.
2	157181836	CTGTG	C	UTR3	*NR4A2*	.	.	.
10	84213316	C	G	intronic	*NRG3*	.	.	.
X	105168598	A	G	intronic	*NRK*	.	.	.
2	51955232	C	CATTATT	intergenic	*NRXN1,MIR4431*	.	.	.
2	19474068	T	G	intergenic	*NT5C1B-RDH14,MIR4757*	.	.	.

10	81535509	T	C	ncRNA_intronic	*NUTM2B-AS1*	.	.	.
11	114424149	A	G	intronic	*NXPE1*	.	.	.
7	8546063	G	A	intronic	*NXPH1*	.	.	.
7	8757524	GTATA	G	intronic	*NXPH1*	.	.	.
1	102799324	G	C	intergenic	*OLFM3,COL11A1*	.	.	.
X	67476689	C	CA	intronic	*OPHN1*	.	.	.
6	154421240	ATATATTATATAT	A	intronic	*OPRM1*	.	.	.
22	17041465	AG	A	intergenic	*OR11H1,CCT8L2*	.	.	.
9	125316102	C	T	exonic	*OR1N2*	synonymous_SNV	.	.
19	8880413	C	G	intergenic	*OR2Z1,ZNF558*	.	.	.
11	55301177	T	TC	intergenic	*OR4A15,OR4C15*	.	.	.
11	48627656	G	T	intergenic	*OR4A47,TRIM49B*	.	.	.
11	48755907	G	T	intergenic	*OR4A47,TRIM49B*	.	.	.
11	48755909	A	T	intergenic	*OR4A47,TRIM49B*	.	.	.
11	51585174	A	G	intergenic	*OR4C46,NONE*	.	.	.
1	404447	C	T	intergenic	*OR4F16,LOC101928626*	.	.	.
12	55728168	A	G	intergenic	*OR6C3,OR6C75*	.	.	.
X	38240982	G	T	intronic	*OTC*	.	.	.
11	7757721	T	TAAAGA	intergenic	*OVCH2,OR5P2*	.	.	.
X	1628157	A	G	intronic	*P2RY8*	.	.	.
2	147990486	AAATCAAAATTAAATTAATCAAAATTTTGATTAATTTAATCAAAATTAAATTAATCAAAATTTTGATTAATTT	A	intergenic	*PABPC1P2,ACVR2A*	.	.	.
1	176789214	G	A	intronic	*PAPPA2*	.	.	.
6	163040742	TCACA	T	intronic	*PARK2*	.	.	.
4	76196508	T	A	intergenic	*PARM1,LOC441025*	.	.	.
2	114181914	G	A	intergenic	*PAX8,CBWD2*	.	.	.
1	164573161	ATTTCATTTTT	A	intronic	*PBX1*	.	.	.

10	36071162	G	T	ncRNA_intronic	*PCAT5*	.	.	.
10	37270198	CACACACA CACACAT	C	intergenic	*PCAT5,ANKRD30A*	.	.	.
10	57178177	C	G	intergenic	*PCDH15,MTRNR2L 5*	.	.	.
10	56585461	G	A	intergenic	*PCDH15,MTRNR2L 5*	.	.	.
7	82726068	T	TTATATAAAT	intronic	*PCLO*	.	.	.
6	171021358	C	T	intergenic	*PDCD2,NONE*	.	.	.
11	14771445	C	CATATATATA	intronic	*PDE3B*	.	.	.
16	15076571	C	T	intronic	*PDXDC1*	.	.	.
12	41893593	T	A	intronic	*PDZRN4*	.	.	.
8	77965003	A	ATTC	intergenic	*PEX2,LOC1027248 74*	.	.	.
3	48568138	G	GTTTTC	intronic	*PFKFB4*	.	.	.
20	58360930	T	TGC	intronic	*PHACTR3*	.	.	.
X	22061695	C	T	intronic	*PHEX*	.	.	.
3	111473293	CTGTT	C	intronic	*PHLDB2,PLCXD2*	.	.	.
5	67862479	T	A	intergenic	*PIK3R1,LOC10192 8885*	.	.	.
17	10752639	A	C	intergenic	*PIRT,SHISA6*	.	.	.
19	14582530	G	A	exonic	*PKN1*	synonymous_SNV	.	.
10	20612720	G	C	intergenic	*PLXDC2,MIR4675*	.	.	.
10	20802435	G	C	intergenic	*PLXDC2,MIR4675*	.	.	.
3	126887909	G	T	intergenic	*PLXNA1,C3orf56*	.	.	.
18	57629147	T	A	intergenic	*PMAIP1,MC4R*	.	.	.
5	75193320	G	A	intergenic	*POC5,SV2C*	.	.	.
7	53390040	T	C	intergenic	*POM121L12,LINC0 1446*	.	.	.
22	24646723	A	G	upstream	*POM121L9P*	.	.	.
14	19573285	G	A	intronic	*POTEG*	.	.	.

22	16272612	C	G	intronic	*POTEH*	.	.	.
22	16278406	T	C	intronic	*POTEH*	.	.	.
22	16420663	C	A	intergenic	*POTEH,OR11H1*	.	.	.
14	91973733	GTATATGT ACTATAATT ATATATACT ATATAA	G	intronic	*PPP4R3A*	.	.	.
1	13218179	A	C	intergenic	*PRAMEF36P,PRAM EF18*	.	.	.
11	129821843	C	A	intronic	*PRDM10*	.	.	.
11	129823432	G	A	intronic	*PRDM10*	.	.	.
4	121911449	C	G	intergenic	*PRDM5,NDNF*	.	.	.
6	106058762	T	G	intergenic	*PREP,PRDM1*	.	.	.
20	47307786	C	T	intronic	*PREX1*	.	.	.
6	57611781	T	A	intergenic	*PRIM2,GUSBP4*	.	.	.
14	30512719	C	G	intergenic	*PRKD1,G2E3*	.	.	.
8	48687774	A	AAATT	intronic	*PRKDC*	.	.	.
X	126443086	C	T	intergenic	*PRR32,ACTRT1*	.	.	.
X	127123714	T	C	intergenic	*PRR32,ACTRT1*	.	.	.
1	170749621	T	TAAAA	intergenic	*PRRX1,MROH9*	.	.	.
16	2925158	A	ATGTTT	intergenic	*PRSS22,FLYWCH2*	.	.	.
7	141610458	T	G	intergenic	*PRSS37,OR9A4*	.	.	.
9	79567426	CAAAAA	C	intergenic	*PRUNE2,FOXB2*	.	.	.
2	162211668	C	G	intronic	*PSMD14*	.	.	.
18	43568401	G	GAAAAAAAA	intronic	*PSTPIP2*	.	.	.
X	23105842	A	G	ncRNA_intronic	*PTCHD1-AS*	.	.	.
10	16522409	G	A	intronic	*PTER*	.	.	.
5	40710072	A	C	intergenic	*PTGER4,TTC33*	.	.	.
2	210031205	C	T	intergenic	*PTH2R,MAP2*	.	.	.

2	120578390	ATAAAGCTGGACTTGCACATATATATATGTATACTTTACATATATATATACACAGCTTTACATACACACATGTGTGTGTATG	A	intronic	*PTPN4*	.	.	.
9	9805753	A	T	intronic	*PTPRD*	.	.	.
9	10577481	C	T	intronic	*PTPRD*	.	.	.
9	11956707	G	C	intergenic	*PTPRD-AS2,TYRP1*	.	.	.
7	158210795	G	A	intronic	*PTPRN2*	.	.	.
12	71175893	C	G	intronic	*PTPRR*	.	.	.
15	24525184	A	T	intergenic	*PWRN2,PWRN3*	.	.	.
15	24349650	G	T	intergenic	*PWRN4,PWRN2*	.	.	.
4	122433714	ATATATATATATATATATATATATATATG	A	intergenic	*QRFPR,ANXA5*	.	.	.
X	102268947	C	A	intergenic	*RAB40AL,BEX1*	.	.	.
1	174596496	A	G	intronic	*RABGAP1L*	.	.	.
8	85176844	A	G	intronic	*RALYL*	.	.	.
8	85567336	T	A	intronic	*RALYL*	.	.	.
4	82480812	A	T	intergenic	*RASGEF1B,HNRNPD*	.	.	.
16	7494890	T	TAGAC	intronic	*RBFOX1*	.	.	.
16	7605481	G	A	intronic	*RBFOX1*	.	.	.
16	6855839	C	T	intronic	*RBFOX1*	.	.	.
20	35640309	C	T	intronic	*RBL1*	.	.	.
12	114416202	G	T	intergenic	*RBM19,TBX5*	.	.	.
6	111757594	T	C	intronic	*REV3L*	.	.	.
2	88157929	TAAAAAAG	T	intronic	*RGPD1*	.	.	.
2	107055889	T	C	intronic	*RGPD3*	.	.	.
2	107055890	G	A	intronic	*RGPD3*	.	.	.

2	108461566	A	G	intronic	*RGPD4*	.	.	.
12	49476425	CCCAAAGTGCTGGGGTTACAGGCATGAGCCACCATGCCTATGCTTTTTTTAAAAAAAAATAGAATAAAATAAAATAAACGTGTAGGCCAGGTGCAGTGGCTCATGCCTGTAATCCCAGCACTTTGGGGGG	C	intergenic	*RHEBL1,DHH*	.	.	.
22	20713603	C	T	intergenic	*RIMBP3,ZNF74*	.	.	.
22	20721281	A	G	intergenic	*RIMBP3,ZNF74*	.	.	.
8	104972034	C	A	intronic	*RIMS2*	.	.	.
3	80200265	T	A	intergenic	*ROBO1,LOC728290*	.	.	.
X	46718666	C	T	intronic	*RP2*	.	.	.
6	166960442	C	CT	intronic	*RPS6KA2*	.	.	.
X	55985353	A	G	intergenic	*RRAGB,KLF8*	.	.	.
X	56026025	A	C	intergenic	*RRAGB,KLF8*	.	.	.
1	237515847	T	C	intronic	*RYR2*	.	.	.
8	119392853	C	T	intronic	*SAMD12*	.	.	.
6	147883957	CAAAA	C	intronic	*SAMD5*	.	.	.
2	181719497	T	C	ncRNA_intronic	*SCHLAP1*	.	.	.
X	18436323	C	A	intergenic	*SCML2,CDKL5*	.	.	.
3	38612895	C	T	intronic	*SCN5A*	.	.	.
5	9350971	C	T	intronic	*SEMA5A*	.	.	.
2	180111945	G	A	intronic	*SESTD1*	.	.	.
2	85892795	C	A	exonic	*SFTPB*	synonymous_SNV	.	.
8	14398783	T	A	intronic	*SGCZ*	.	.	.
8	14141023	T	TATAA	intronic	*SGCZ*	.	.	.

10	83266353	C	G	intergenic	*SH2D4B,NRG3*	.	.	.
X	695495	C	T	intergenic	*SHOX,CRLF2*	.	.	.
X	913030	G	T	intergenic	*SHOX,CRLF2*	.	.	.
3	72800647	G	GTACATATA	intronic	*SHQ1*	.	.	.
18	44866994	ATGTG	A	intergenic	*SKOR2,MIR4527*	.	.	.
12	60718945	T	TTTG	intergenic	*SLC16A7,FAM19A2*	.	.	.
12	60932435	T	A	intergenic	*SLC16A7,FAM19A2*	.	.	.
21	46957664	G	A	intronic	*SLC19A1*	.	.	.
9	87242899	T	G	intergenic	*SLC28A3,NTRK2*	.	.	.
8	145585658	G	T	downstream	*SLC52A2*	.	.	.
16	33042442	C	A	intergenic	*SLC6A10P,TP53TG3*	.	.	.
1	173476647	GCACA	G	intronic	*SLC9C2*	.	.	.
18	73189570	C	T	intergenic	*SMIM21,LOC339298*	.	.	.
19	48421186	TA	T	upstream	*SNAR-A1,SNAR-A2*	.	.	.
19	50621073	C	A	ncRNA_exonic	*SNAR-A10,SNAR-A11,SNAR-A14,SNAR-A3,SNAR-A4,SNAR-A5,SNAR-A6,SNAR-A7,SNAR-A8,SNAR-A9*	.	.	.
6	87625339	A	AAACTAAT	intergenic	*SNHG5,HTR1E*	.	.	.
15	75901656	G	A	intronic	*SNUPN*	.	.	.
11	16263161	T	G	intronic	*SOX6*	.	.	.
2	214786089	T	TGTG	intronic	*SPAG16*	.	.	.
2	214213444	G	C	intronic	*SPAG16*	.	.	.
2	215042695	GTATATA	G	intronic	*SPAG16*	.	.	.
X	142902472	A	T	intergenic	*SPANXN2,UBE2NL*	.	.	.
9	66329285	CAAAAAAAAAAAAAAA	C	intergenic	*SPATA31A5,LINC01410*	.	.	.

9	85461787	C	G	intergenic	*SPATA31D1,RASEF*	.	.	.
2	229729953	A	G	intergenic	*SPHKAP,PID1*	.	.	.
1	158621699	C	CTGTG	intronic	*SPTA1*	.	.	.
8	126028359	T	C	intronic	*SQLE*	.	.	.
11	126278955	A	AG	intronic	*ST3GAL4*	.	.	.
7	38276507	A	G	intergenic	*STARD3NL,TARP*	.	.	.
2	169214595	AAGAG	A	intergenic	*STK39,CERS6*	.	.	.
7	134973834	T	TCCCAGGC	intergenic	*STRA8,CNOT4*	.	.	.
14	26547042	AATATAT	A	intergenic	*STXBP6,NOVA1*	.	.	.
13	37658443	C	A	intergenic	*SUPT20H,CSNK1A1L*	.	.	.
6	152723910	CAGAG	C	intronic	*SYNE1*	.	.	.
3	63291897	G	T	intronic	*SYNPR*	.	.	.
X	37914314	G	T	intronic	*SYTL5*	.	.	.
7	38303849	T	G	intronic	*TARP*	.	.	.
4	38413019	CTGTG	C	intergenic	*TBC1D1,LINC01258*	.	.	.
22	47305191	TACAC	T	intronic	*TBC1D22A*	.	.	.
17	58112340	C	CA	intergenic	*TBC1D3P1-DHX40P1,MIR4737*	.	.	.
X	9544961	G	T	intronic	*TBL1X*	.	.	.
6	85838498	GTGTA	G	intergenic	*TBX18,NT5E*	.	.	.
X	79428825	G	T	intergenic	*TBX22,CHMP1B2P*	.	.	.
6	167662381	C	CTTCCTT	intergenic	*TCP10L2,UNC93A*	.	.	.
4	65431653	C	T	intergenic	*TECRL,LOC401134*	.	.	.
4	65619322	T	C	intergenic	*TECRL,LOC401134*	.	.	.
4	65619964	A	T	intergenic	*TECRL,LOC401134*	.	.	.
21	10744439	C	CG	intergenic	*TEKT4P2,TPTE*	.	.	.
21	10740290	C	A	intergenic	*TEKT4P2,TPTE*	.	.	.

21	10618747	GGTGTATATACACACATATGCATATATTGTGTATATACACACATATGCATATATT	G	intergenic	*TEKT4P2,TPTE*	.	.	.
21	10820005	GGATTTGAATAGAATA	G	intergenic	*TEKT4P2,TPTE*	.	.	.
21	10644151	A	C	intergenic	*TEKT4P2,TPTE*	.	.	.
X	123983272	TTCTC	T	intronic	*TENM1*	.	.	.
11	78686662	CATAT	C	intronic	*TENM4*	.	.	.
X	69962572	CA	C	intronic	*TEX11*	.	.	.
2	145671584	C	T	ncRNA_intronic	*TEX41*	.	.	.
X	90291766	C	A	intergenic	*TGIF2LX,PABPC5-AS1*	.	.	.
2	43490106	A	G	intronic	*THADA*	.	.	.
19	32266924	G	T	intergenic	*THEG5,LINC01533*	.	.	.
6	128223485	A	AAAAT	intronic	*THEMIS*	.	.	.
1	205210300	A	G	intronic	*TMCC2*	.	.	.
17	8084933	A	AGCTTGACA	intergenic	*TMEM107,MIR4521*	.	.	.
12	44423237	G	A	intronic	*TMEM117*	.	.	.
14	106025555	C	T	intergenic	*TMEM121,MIR8071-2*	.	.	.
11	86785633	C	A	intronic	*TMEM135*	.	.	.
2	104720467	C	A	intergenic	*TMEM182,LOC100287010*	.	.	.
2	103583841	GCACA	G	intergenic	*TMEM182,LOC100287010*	.	.	.
2	104321627	T	TA	intergenic	*TMEM182,LOC100287010*	.	.	.

14	57063077	CACACACACATATATATGTGAGAGTGTGTGTGTGTGTGTGTGTGTGTGTATATATATATATATATACAT	C	intronic	*TMEM260*	.	.	.
9	7864520	T	G	intergenic	*TMEM261,PTPRD*	.	.	.
16	32293489	G	GGAAAT	intergenic	*TP53TG3D,LOC390705*	.	.	.
12	72474909	A	T	intergenic	*TPH2,TRHDE-AS1*	.	.	.
5	722391	G	A	intergenic	*TPPP,ZDHHC11*	.	.	.
6	42382798	G	A	intronic	*TRERF1*	.	.	.
6	42478791	C	CT	intergenic	*TRERF1,UBR2*	.	.	.
6	30187732	C	A	intergenic	*TRIM26,HCG17*	.	.	.
X	100297393	G	A	intronic	*TRMT2B*	.	.	.
4	122997316	C	T	intergenic	*TRPC3,KIAA1109*	.	.	.
5	136289914	A	G	intergenic	*TRPC7,SPOCK1*	.	.	.
8	116869274	T	G	intergenic	*TRPS1,LINC00536*	.	.	.
13	45005299	GCACA	G	downstream	*TSC22D1*	.	.	.
6	94468712	C	T	ncRNA_intronic	*TSG1*	.	.	.
X	73019871	G	T	ncRNA_exonic	*TSIX*	.	.	.
X	38449469	C	G	intronic	*TSPAN7*	.	.	.
12	71568602	A	T	intergenic	*TSPAN8,LGR5*	.	.	.
2	3361136	G	T	intronic	*TSSC1*	.	.	.
4	147783403	TTATA	T	intronic	*TTC29*	.	.	.
6	167751943	TCACA	T	intronic	*TTLL2*	.	.	.
18	666848	AATGGGCACAGAGGTTTT	A	intronic	*TYMS*	.	.	.
X	143875138	TAGAG	T	intergenic	*UBE2NL,SPANXN1*	.	.	.
9	33831179	C	G	intronic	*UBE2R2*	.	.	.
2	129007241	C	T	intergenic	*UGGT1,HS6ST1*	.	.	.
15	30869574	A	G	ncRNA_intronic	*ULK4P1,ULK4P2*	.	.	.

8	35370031	CATAGATTATATATTCAGATATGTATGTATATATATATATATATATATATATATA	C	intronic	*UNC5D*	.	.	.
8	36210621	A	G	intergenic	*UNC5D,KCNU1*	.	.	.
1	216551125	GAAAGAAAGAGAAGGAAAGAAATAA	G	intronic	*USH2A*	.	.	.
13	27786226	G	A	intergenic	*USP12-AS2,LINC00412*	.	.	.
21	30413258	T	A	intronic	*USP16*	.	.	.
17	18419505	T	C	ncRNA_intronic	*USP32P2*	.	.	.
6	99964297	G	C	intergenic	*USP45,TSTD3*	.	.	.
17	49381563	A	AAAAT	intergenic	*UTP18,LOC101927274*	.	.	.
6	43798210	ATGTG	A	intergenic	*VEGFA,LINC01512*	.	.	.
X	27325337	G	GATAT	intergenic	*VENTXP1,PPP4R3CP*	.	.	.
7	54730610	C	T	intergenic	*VSTM2A-OT1,SEC61G*	.	.	.
2	215401699	TACACAC	T	intronic	*VWC2L*	.	.	.
12	6102794	TATATATGTGTGTGTGTGTGTGTATATATATATATATACACACACACACATATAC	T	intronic	*VWF*	.	.	.
1	14455	T	TAAGAG	ncRNA_exonic	*WASH7P*	.	.	.
2	63659982	G	T	intronic	*WDPCP*	.	.	.
X	54303569	G	T	intronic	*WNK3*	.	.	.
1	228172177	G	A	intergenic	*WNT9A,WNT3A*	.	.	.

8	56334085	ATCTC	A	intronic	*XKR4*	.	.	.
20	21300921	T	TTATTTATG	intronic	*XRN2*	.	.	.
11	74650888	TAGAG	T	intronic	*XRRA1*	.	.	.
16	17771783	C	A	intergenic	*XYLT1,NPIPA8*	.	.	.
20	56218638	C	T	intergenic	*ZBP1,PMEPA1*	.	.	.
X	64173516	C	CAGTT	intronic	*ZC4H2*	.	.	.
2	145234640	G	A	intronic	*ZEB2*	.	.	.
8	106970141	G	A	ncRNA_intronic	*ZFPM2-AS1*	.	.	.
4	395686	G	A	intergenic	*ZNF141,ABCA11P*	.	.	.
19	36785951	G	C	intergenic	*ZNF146,LOC100134317*	.	.	.
18	74651771	T	G	intronic	*ZNF236*	.	.	.
1	91086120	C	A	intergenic	*ZNF326,BARHL2*	.	.	.
1	91133261	GTATA	G	intergenic	*ZNF326,BARHL2*	.	.	.
2	180553217	AAACAAC	A	intronic	*ZNF385B*	.	.	.
18	32963430	G	C	intergenic	*ZNF396,INO80C*	.	.	.
16	30414604	G	GCTTCTTCT	intergenic	*ZNF48,ZNF771*	.	.	.
18	23392989	TACAC	T	intergenic	*ZNF521,SS18*	.	.	.
19	2906843	G	A	intronic	*ZNF57*	.	.	.
19	2929301	CT	C	intergenic	*ZNF57,ZNF77*	.	.	.
X	47958799	G	A	intergenic	*ZNF630,SSX6*	.	.	.
19	22391645	G	T	intergenic	*ZNF676,ZNF729*	.	.	.
22	20753453	T	C	intronic	*ZNF74*	.	.	.
7	127034758	A	AGG	intergenic	*ZNF800,LOC100506682*	.	.	.
2	133115870	A	T	intergenic	*ZNF806,GPR39*	.	.	.
7	64961944	G	T	intergenic	*ZNF92,LOC441242*	.	.	.
7	76077497	G	A	intergenic	*ZP3,DTX2*	.	.	.
3	103188634	CATTCACATATACAATATGTGAATACGATTGTGAATACAATCGT	C	intergenic	*ZPLD1,MIR548AB*	.	.	.
3	102701295	A	G	intergenic	*ZPLD1,MIR548AB*	.	.	.
2	187861514	T	C	intergenic	*ZSWIM2,CALCRL*	.	.	.
4	165156767	T	C	intronic	*43891*	.	.	.

4	164702177	T	A	intronic	*43891*	.	.	.
4	164707287	G	A	intronic	*43891*	.	.	.
4	164639096	T	C	intronic	*43891*	.	.	.
4	164754464	A	G	intronic	*43891*	.	.	.
4	164953284	T	A	intronic	*43891*	.	.	.
4	164975056	C	CA	intronic	*43891*	.	.	.
4	164998602	T	C	intronic	*43891*	.	.	.
5	126234531	G	A	intronic	*43893*	.	.	.
5	126317927	C	T	intronic	*43893*	.	.	.
5	126326168	T	C	intronic	*43893*	.	.	.
2	217188593	T	C	intronic	*43894*	.	.	.
5	10409225	G	A	intronic	*43896*	.	.	.
5	10389794	A	G	intronic	*43896*	.	.	.
2	160582021	G	A	intronic	*43897*	.	.	.
10	45972452	C	G	intronic	*43898*	.	.	.
10	46035406	G	A	intronic	*43898*	.	.	.
X	118754300	G	T	intronic	*44080*	.	.	.
7	35846814	A	G	intronic	*44081*	.	.	.
17	75469896	T	C	intronic	*44083*	.	.	.
17	75343610	A	G	intronic	*44083*	.	.	.
17	75287352	G	A	intronic	*44083*	.	.	.
2	110340360	T	C	intronic	*44084*	.	.	.
2	110340623	G	T	intronic	*44084*	.	.	.
10	52587454	T	C	intronic	*A1CF*	.	.	.
12	9276135	A	G	intergenic	*A2M,PZP*	.	.	.
12	125564332	G	T	intronic	*AACS*	.	.	.
12	125651631	TACACACACAC	T	intergenic	*AACS,TMEM132B*	.	.	.
3	151604552	A	G	ncRNA_intronic	*AADACL2-AS1*	.	.	.
3	151560315	T	G	ncRNA_intronic	*AADACL2-AS1*	.	.	.
3	151947053	G	A	intergenic	*AADACL2-AS1,MBNL1-AS1*	.	.	.
1	12793429	T	C	intergenic	*AADACL3,C1orf158*	.	.	.
1	12767170	C	T	intergenic	*AADACL4,AADACL3*	.	.	.
4	171077070	C	G	intergenic	*AADAT,LINC01612*	.	.	.
4	171077333	C	A	intergenic	*AADAT,LINC01612*	.	.	.

11	77565327	C	T	intronic	*AAMDC*	.	.	.
11	77604821	G	C	intronic	*AAMDC,INTS4*	.	.	.
11	77600335	C	T	intronic	*AAMDC,INTS4*	.	.	.
11	77600336	A	G	intronic	*AAMDC,INTS4*	.	.	.
4	57234291	T	G	intronic	*AASDH*	.	.	.
11	106051321	G	A	intergenic	*AASDHPPT,LOC101928535*	.	.	.
7	121775006	G	T	intronic	*AASS*	.	.	.
7	121881429	G	A	intergenic	*AASS,FEZF1*	.	.	.
7	121898707	T	G	intergenic	*AASS,FEZF1*	.	.	.
21	45266429	C	T	intergenic	*AATBC,AGPAT3*	.	.	.
17	35433909	G	A	intergenic	*AATF,ACACA*	.	.	.
17	79125104	T	C	intronic	*AATK*	.	.	.
16	8782557	A	G	intronic	*ABAT*	.	.	.
9	107773560	ACC	A	intergenic	*ABCA1,SLC44A1*	.	.	.
4	454851	A	T	ncRNA_intronic	*ABCA11P*	.	.	.
4	454852	T	C	ncRNA_intronic	*ABCA11P*	.	.	.
2	215905275	G	A	intronic	*ABCA12*	.	.	.
2	216115364	C	T	intergenic	*ABCA12,ATIC*	.	.	.
2	216004472	A	C	intergenic	*ABCA12,ATIC*	.	.	.
2	216004473	G	A	intergenic	*ABCA12,ATIC*	.	.	.
2	216015979	C	T	intergenic	*ABCA12,ATIC*	.	.	.
2	216060468	C	A	intergenic	*ABCA12,ATIC*	.	.	.
2	216060470	G	A	intergenic	*ABCA12,ATIC*	.	.	.
2	216152111	G	A	intergenic	*ABCA12,ATIC*	.	.	.
7	48522766	C	T	intronic	*ABCA13*	.	.	.
7	48609428	C	T	intronic	*ABCA13*	.	.	.
7	48641795	G	T	intronic	*ABCA13*	.	.	.
7	48642102	C	T	intronic	*ABCA13*	.	.	.
7	48830939	C	G	intergenic	*ABCA13,CDC14C*	.	.	.
7	48884919	A	G	intergenic	*ABCA13,CDC14C*	.	.	.
7	48713331	T	C	intergenic	*ABCA13,CDC14C*	.	.	.
7	48728523	C	T	intergenic	*ABCA13,CDC14C*	.	.	.
7	48770943	T	C	intergenic	*ABCA13,CDC14C*	.	.	.
7	48770944	G	A	intergenic	*ABCA13,CDC14C*	.	.	.
7	48771434	G	C	intergenic	*ABCA13,CDC14C*	.	.	.
7	48893887	A	G	intergenic	*ABCA13,CDC14C*	.	.	.
1	94551343	C	T	intronic	*ABCA4*	.	.	.

1	94604573	C	T	intergenic	*ABCA4,ARHGAP29*	.	.	.
17	67280538	T	C	intronic	*ABCA5*	.	.	.
17	67406726	T	C	intergenic	*ABCA5,MAP2K6*	.	.	.
17	67110268	T	C	intronic	*ABCA6*	.	.	.
17	66998625	T	C	intronic	*ABCA9*	.	.	.
17	67009170	G	A	ncRNA_intronic	*ABCA9-AS1*	.	.	.
17	67072931	G	A	intergenic	*ABCA9,ABCA6*	.	.	.
17	67072933	G	T	intergenic	*ABCA9,ABCA6*	.	.	.
7	87140844	A	G	intronic	*ABCB1*	.	.	.
1	229664897	G	A	intronic	*ABCB10*	.	.	.
2	169798285	A	G	intronic	*ABCB11*	.	.	.
7	87077555	G	A	intronic	*ABCB4*	.	.	.
7	87100410	C	CATT	intronic	*ABCB4*	.	.	.
7	87071957	CATAA	C	intronic	*ABCB4*	.	.	.
7	87097313	T	A	intronic	*ABCB4*	.	.	.
7	20756537	C	T	intronic	*ABCB5*	.	.	.
7	20731237	T	G	intronic	*ABCB5*	.	.	.
X	74328867	A	C	intronic	*ABCB7*	.	.	.
X	74320802	A	T	intronic	*ABCB7*	.	.	.
X	74343202	A	C	intronic	*ABCB7*	.	.	.
X	74347695	G	A	intronic	*ABCB7*	.	.	.
X	74468061	C	CGAGCT	intergenic	*ABCB7,UPRT*	.	.	.
X	74479638	T	TTGTC	intergenic	*ABCB7,UPRT*	.	.	.
X	74472986	C	A	intergenic	*ABCB7,UPRT*	.	.	.
X	74466859	G	T	intergenic	*ABCB7,UPRT*	.	.	.
X	74412067	A	G	intergenic	*ABCB7,UPRT*	.	.	.
X	74456835	C	A	intergenic	*ABCB7,UPRT*	.	.	.
X	74481034	G	A	intergenic	*ABCB7,UPRT*	.	.	.
X	74484385	T	G	intergenic	*ABCB7,UPRT*	.	.	.
X	74486059	T	A	intergenic	*ABCB7,UPRT*	.	.	.
X	74486939	T	C	intergenic	*ABCB7,UPRT*	.	.	.
6	43408429	A	G	intronic	*ABCC10*	.	.	.
6	43410104	G	A	intronic	*ABCC10*	.	.	.
21	15701356	T	C	intergenic	*ABCC13,HSPA13*	.	.	.
21	15704207	G	T	intergenic	*ABCC13,HSPA13*	.	.	.
10	101587736	T	TACAAGAAA	intronic	*ABCC2*	.	.	.
10	101624192	G	A	intergenic	*ABCC2,DNMBP*	.	.	.

10	101624193	A	G	intergenic	*ABCC2,DNMBP*	.	.	.
10	101624308	T	C	intergenic	*ABCC2,DNMBP*	.	.	.
10	101624928	A	G	intergenic	*ABCC2,DNMBP*	.	.	.
10	101626225	T	C	intergenic	*ABCC2,DNMBP*	.	.	.
13	95733978	G	C	intronic	*ABCC4*	.	.	.
13	95698817	G	A	intronic	*ABCC4*	.	.	.
3	183711710	A	G	intronic	*ABCC5*	.	.	.
3	183703765	C	T	intronic	*ABCC5*	.	.	.
16	18681637	T	A	intergenic	*ABCC6P1,RPS15A*	.	.	.
16	18682482	A	C	intergenic	*ABCC6P1,RPS15A*	.	.	.
12	22002217	T	C	intronic	*ABCC9*	.	.	.
12	22023930	A	G	intronic	*ABCC9*	.	.	.
12	21977175	A	G	intronic	*ABCC9*	.	.	.
12	22057954	G	GA	intronic	*ABCC9*	.	.	.
12	22188846	T	C	intergenic	*ABCC9,CMAS*	.	.	.
12	22178401	A	G	intergenic	*ABCC9,CMAS*	.	.	.
12	22119600	T	C	intergenic	*ABCC9,CMAS*	.	.	.
12	22120086	C	T	intergenic	*ABCC9,CMAS*	.	.	.
12	39954076	C	G	intronic	*ABCD2*	.	.	.
12	39960985	G	C	intronic	*ABCD2*	.	.	.
12	39978316	A	T	intronic	*ABCD2*	.	.	.
12	39978318	A	G	intronic	*ABCD2*	.	.	.
1	94962007	TTGTGTG	T	intronic	*ABCD3*	.	.	.
1	94976418	T	C	intronic	*ABCD3*	.	.	.
14	74775253	T	A	intergenic	*ABCD4,VRTN*	.	.	.
6	30553476	G	A	intronic	*ABCF1*	.	.	.
3	183938004	T	A	intergenic	*ABCF3,VWA5B2*	.	.	.
21	43725108	T	A	intergenic	*ABCG1,TFF3*	.	.	.
2	44060120	C	T	intronic	*ABCG5*	.	.	.
7	73151421	T	C	exonic	*ABHD11*	nonsynonymous_SNV	.	.
15	80999886	C	G	intronic	*ABHD17C*	.	.	.
15	80999887	T	A	intronic	*ABHD17C*	.	.	.
14	23184514	A	G	intergenic	*ABHD4,OXA1L*	.	.	.
14	23087288	C	T	intergenic	*ABHD4,OXA1L*	.	.	.
14	23142649	C	G	intergenic	*ABHD4,OXA1L*	.	.	.
14	23152086	C	A	intergenic	*ABHD4,OXA1L*	.	.	.
3	43846952	C	A	intergenic	*ABHD5,MIR138-1*	.	.	.

3	44119329	T	A	intergenic	*ABHD5,MIR138-1*	.	.	.
3	43845125	A	C	intergenic	*ABHD5,MIR138-1*	.	.	.
3	43850062	A	G	intergenic	*ABHD5,MIR138-1*	.	.	.
3	43890342	C	A	intergenic	*ABHD5,MIR138-1*	.	.	.
3	43890344	T	A	intergenic	*ABHD5,MIR138-1*	.	.	.
3	43976738	C	T	intergenic	*ABHD5,MIR138-1*	.	.	.
3	58289436	G	A	intergenic	*ABHD6,RPP14*	.	.	.
2	204235064	G	C	intronic	*ABI2*	.	.	.
3	100555101	C	CT	intronic	*ABI3BP*	.	.	.
3	100627273	C	T	intronic	*ABI3BP*	.	.	.
3	100725117	A	G	intergenic	*ABI3BP,IMPG2*	.	.	.
9	133739311	A	AT	intronic	*ABL1*	.	.	.
10	116417812	T	C	exonic	*ABLIM1*	nonsynonymous_SNV	.	.
10	116512580	G	T	intergenic	*ABLIM1,LOC101927692*	.	.	.
4	8143040	C	A	intronic	*ABLIM2*	.	.	.
4	8177108	T	C	intergenic	*ABLIM2,SH3TC1*	.	.	.
17	1168307	G	A	intergenic	*ABR,BHLHA9*	.	.	.
8	108073086	G	A	intergenic	*ABRA,ANGPT1*	.	.	.
8	108073087	A	ATTTTTG	intergenic	*ABRA,ANGPT1*	.	.	.
8	107873275	T	G	intergenic	*ABRA,ANGPT1*	.	.	.
8	107975975	A	G	intergenic	*ABRA,ANGPT1*	.	.	.
8	108016962	T	TA	intergenic	*ABRA,ANGPT1*	.	.	.
8	108053010	G	A	intergenic	*ABRA,ANGPT1*	.	.	.
6	139392786	G	A	intergenic	*ABRACL,HECA*	.	.	.
6	139407403	T	A	intergenic	*ABRACL,HECA*	.	.	.
6	139409529	C	T	intergenic	*ABRACL,HECA*	.	.	.
6	139442823	A	G	intergenic	*ABRACL,HECA*	.	.	.
6	139444989	A	G	intergenic	*ABRACL,HECA*	.	.	.
11	34221496	C	T	intronic	*ABTB2*	.	.	.
11	34391185	T	C	intergenic	*ABTB2,CAT*	.	.	.
17	35559631	T	TAC	intronic	*ACACA*	.	.	.
17	35538876	T	A	intronic	*ACACA*	.	.	.
17	35728144	C	T	intronic	*ACACA*	.	.	.
12	109681224	T	C	intronic	*ACACB*	.	.	.
12	109681225	G	A	intronic	*ACACB*	.	.	.
12	112156501	C	CT	intronic	*ACAD10*	.	.	.

12	112172799	G	A	intronic	*ACAD10*	.	.	.
2	211122644	A	G	intergenic	*ACADL,MYL1*	.	.	.
2	211130387	T	C	intergenic	*ACADL,MYL1*	.	.	.
2	211130388	G	A	intergenic	*ACADL,MYL1*	.	.	.
1	76246066	C	T	intergenic	*ACADM,RABGGTB*	.	.	.
17	7252722	T	C	intronic	*ACAP1*	.	.	.
1	180364615	T	G	intronic	*ACBD6*	.	.	.
1	180272603	T	C	intronic	*ACBD6*	.	.	.
1	180307780	T	A	intronic	*ACBD6*	.	.	.
1	180307781	G	A	intronic	*ACBD6*	.	.	.
1	180423279	T	C	intronic	*ACBD6*	.	.	.
1	180423280	G	C	intronic	*ACBD6*	.	.	.
1	180423435	A	G	intronic	*ACBD6*	.	.	.
1	180423436	T	C	intronic	*ACBD6*	.	.	.
1	180424004	T	A	intronic	*ACBD6*	.	.	.
1	180435032	A	T	intronic	*ACBD6*	.	.	.
1	180435033	C	A	intronic	*ACBD6*	.	.	.
9	19500685	G	T	intergenic	*ACER2,SLC24A2*	.	.	.
11	76580405	A	G	intronic	*ACER3*	.	.	.
11	76619415	A	T	intronic	*ACER3*	.	.	.
11	76620094	A	G	intronic	*ACER3*	.	.	.
11	76620205	C	T	intronic	*ACER3*	.	.	.
11	76620337	A	G	intronic	*ACER3*	.	.	.
11	76624984	A	C	intronic	*ACER3*	.	.	.
11	76677908	T	C	intronic	*ACER3*	.	.	.
11	76679616	C	A	intronic	*ACER3*	.	.	.
11	76680954	T	C	intronic	*ACER3*	.	.	.
11	76680955	A	G	intronic	*ACER3*	.	.	.
14	23530825	A	G	exonic	*ACIN1*	nonsynonymous_SNV	.	.
14	23542821	C	T	intronic	*ACIN1*	.	.	.
1	159193627	C	T	intergenic	*ACKR1,FCER1A*	.	.	.
1	159205108	A	T	intergenic	*ACKR1,FCER1A*	.	.	.
2	237819015	G	GTATATATAT	intergenic	*ACKR3,COPS8*	.	.	.
2	237726581	C	T	intergenic	*ACKR3,COPS8*	.	.	.
9	32445243	G	A	intronic	*ACO1*	.	.	.
6	24675644	A	G	intronic	*ACOT13*	.	.	.
6	24676305	G	A	intronic	*ACOT13*	.	.	.

2	111589952	A	G	intronic	*ACOXL*	.	.	.
2	111675262	G	T	intronic	*ACOXL*	.	.	.
2	111675263	T	G	intronic	*ACOXL*	.	.	.
2	111702904	A	G	intronic	*ACOXL*	.	.	.
2	111839174	T	C	intronic	*ACOXL*	.	.	.
2	111839175	G	A	intronic	*ACOXL*	.	.	.
2	111839392	T	C	intronic	*ACOXL*	.	.	.
1	147179856	A	G	intergenic	*ACP6,GJA5*	.	.	.
1	147181444	T	C	intergenic	*ACP6,GJA5*	.	.	.
1	147181445	G	A	intergenic	*ACP6,GJA5*	.	.	.
22	51190473	A	G	intergenic	*ACR,RPL23AP82*	.	.	.
11	125580898	G	GTA	intergenic	*ACRV1,PATE1*	.	.	.
11	125578282	T	C	intergenic	*ACRV1,PATE1*	.	.	.
11	125579645	C	G	intergenic	*ACRV1,PATE1*	.	.	.
11	125585299	T	C	intergenic	*ACRV1,PATE1*	.	.	.
11	125585300	G	A	intergenic	*ACRV1,PATE1*	.	.	.
15	78479310	A	G	intronic	*ACSBG1*	.	.	.
17	48529026	A	G	intronic	*ACSF2*	.	.	.
17	48529027	C	T	intronic	*ACSF2*	.	.	.
2	223880248	T	A	intergenic	*ACSL3,KCNE4*	.	.	.
X	108914758	G	T	intronic	*ACSL4*	.	.	.
X	108921756	T	G	intronic	*ACSL4*	.	.	.
X	108892634	A	G	intronic	*ACSL4*	.	.	.
5	131351136	A	G	intergenic	*ACSL6,IL3*	.	.	.
5	131375458	C	A	intergenic	*ACSL6,IL3*	.	.	.
16	20660867	TTCTC	T	intronic	*ACSM1*	.	.	.
16	20707360	A	G	intergenic	*ACSM1,THUMPD1*	.	.	.
16	20633412	CTGTGTG	C	intergenic	*ACSM2B,ACSM1*	.	.	.
16	20785972	G	C	intronic	*ACSM3*	.	.	.
12	7486163	G	C	intergenic	*ACSM4,CD163L1*	.	.	.
20	24994591	A	G	intronic	*ACSS1*	.	.	.
12	81490601	G	A	intronic	*ACSS3*	.	.	.
12	81524837	A	G	intronic	*ACSS3*	.	.	.
7	5606944	C	A	intergenic	*ACTB,FSCN1*	.	.	.
5	56863285	A	G	intergenic	*ACTBL2,LINCR-0003*	.	.	.
5	56843579	C	T	intergenic	*ACTBL2,LINCR-0003*	.	.	.

5	56843581	C	CT	intergenic	*ACTBL2,LINCR-0003*	.	.	.
1	18235026	C	G	intergenic	*ACTL8,LOC101927876*	.	.	.
19	8819214	C	T	intergenic	*ACTL9,OR2Z1*	.	.	.
19	8826569	T	C	intergenic	*ACTL9,OR2Z1*	.	.	.
19	8827510	C	G	intergenic	*ACTL9,OR2Z1*	.	.	.
1	236871852	T	C	intronic	*ACTN2*	.	.	.
1	236933836	G	T	intergenic	*ACTN2,MTR*	.	.	.
19	39205423	G	C	intronic	*ACTN4*	.	.	.
19	39206667	A	G	intronic	*ACTN4*	.	.	.
2	65474716	C	T	intronic	*ACTR2*	.	.	.
7	152752728	T	C	intergenic	*ACTR3B,LINC01287*	.	.	.
7	152923976	T	C	intergenic	*ACTR3B,LINC01287*	.	.	.
7	152800945	G	T	intergenic	*ACTR3B,LINC01287*	.	.	.
7	152814486	A	G	intergenic	*ACTR3B,LINC01287*	.	.	.
7	152927491	A	C	intergenic	*ACTR3B,LINC01287*	.	.	.
7	152927537	A	G	intergenic	*ACTR3B,LINC01287*	.	.	.
7	152927962	A	G	intergenic	*ACTR3B,LINC01287*	.	.	.
7	152927964	C	T	intergenic	*ACTR3B,LINC01287*	.	.	.
7	152928829	G	A	intergenic	*ACTR3B,LINC01287*	.	.	.
7	152929481	G	A	intergenic	*ACTR3B,LINC01287*	.	.	.
7	152929483	C	A	intergenic	*ACTR3B,LINC01287*	.	.	.
7	152968329	T	C	intergenic	*ACTR3B,LINC01287*	.	.	.
7	152968330	A	C	intergenic	*ACTR3B,LINC01287*	.	.	.

7	153008570	C	T	intergenic	*ACTR3B,LINC0128 7*	.	.	.
7	153010862	G	A	intergenic	*ACTR3B,LINC0128 7*	.	.	.
7	153014197	C	T	intergenic	*ACTR3B,LINC0128 7*	.	.	.
2	92263529	GAGGGAG GAAGGAAA	G	intergenic	*ACTR3BP2,NONE*	.	.	.
2	92290895	T	C	intergenic	*ACTR3BP2,NONE*	.	.	.
2	92312751	A	T	intergenic	*ACTR3BP2,NONE*	.	.	.
2	92322461	C	T	intergenic	*ACTR3BP2,NONE*	.	.	.
2	92181071	C	T	intergenic	*ACTR3BP2,NONE*	.	.	.
2	92204943	C	T	intergenic	*ACTR3BP2,NONE*	.	.	.
2	92297879	G	T	intergenic	*ACTR3BP2,NONE*	.	.	.
2	92133766	A	AT	intergenic	*ACTR3BP2,NONE*	.	.	.
2	92317280	T	A	intergenic	*ACTR3BP2,NONE*	.	.	.
2	92166835	G	T	intergenic	*ACTR3BP2,NONE*	.	.	.
2	92279823	C	T	intergenic	*ACTR3BP2,NONE*	.	.	.
2	92200469	T	TA	intergenic	*ACTR3BP2,NONE*	.	.	.
2	92288607	T	C	intergenic	*ACTR3BP2,NONE*	.	.	.
2	92176343	A	G	intergenic	*ACTR3BP2,NONE*	.	.	.
2	92254212	G	A	intergenic	*ACTR3BP2,NONE*	.	.	.
2	92138612	A	G	intergenic	*ACTR3BP2,NONE*	.	.	.
2	92139964	A	C	intergenic	*ACTR3BP2,NONE*	.	.	.
2	92140163	C	T	intergenic	*ACTR3BP2,NONE*	.	.	.
2	92192996	G	A	intergenic	*ACTR3BP2,NONE*	.	.	.
2	92293208	C	G	intergenic	*ACTR3BP2,NONE*	.	.	.
2	92305361	A	T	intergenic	*ACTR3BP2,NONE*	.	.	.
10	38991211	C	T	ncRNA_exonic	*ACTR3BP5*	.	.	.
10	39059976	A	G	intergenic	*ACTR3BP5,NONE*	.	.	.
10	39042031	T	C	intergenic	*ACTR3BP5,NONE*	.	.	.
10	39060682	T	A	intergenic	*ACTR3BP5,NONE*	.	.	.
10	39001912	GT	G	intergenic	*ACTR3BP5,NONE*	.	.	.
10	38998986	T	G	intergenic	*ACTR3BP5,NONE*	.	.	.
10	38998988	C	T	intergenic	*ACTR3BP5,NONE*	.	.	.
7	149995026	A	T	intronic	*ACTR3C*	.	.	.
12	100605835	T	C	intronic	*ACTR6*	.	.	.

12	100613842	C	A	exonic	*ACTR6*	nonsynonymous_SNV	.	.
X	127418408	T	A	intergenic	*ACTRT1,SMARCA1*	.	.	.
X	127789728	GA	G	intergenic	*ACTRT1,SMARCA1*	.	.	.
X	127806757	C	A	intergenic	*ACTRT1,SMARCA1*	.	.	.
X	127889508	C	A	intergenic	*ACTRT1,SMARCA1*	.	.	.
X	128519104	G	T	intergenic	*ACTRT1,SMARCA1*	.	.	.
X	127367623	T	A	intergenic	*ACTRT1,SMARCA1*	.	.	.
X	128373482	T	TA	intergenic	*ACTRT1,SMARCA1*	.	.	.
X	127251072	C	T	intergenic	*ACTRT1,SMARCA1*	.	.	.
X	127475399	G	GT	intergenic	*ACTRT1,SMARCA1*	.	.	.
X	127745298	C	T	intergenic	*ACTRT1,SMARCA1*	.	.	.
X	127970709	A	G	intergenic	*ACTRT1,SMARCA1*	.	.	.
X	127297424	G	T	intergenic	*ACTRT1,SMARCA1*	.	.	.
X	127838560	G	T	intergenic	*ACTRT1,SMARCA1*	.	.	.
X	127549981	GTATA	G	intergenic	*ACTRT1,SMARCA1*	.	.	.
X	127197743	G	A	intergenic	*ACTRT1,SMARCA1*	.	.	.
X	127197744	G	A	intergenic	*ACTRT1,SMARCA1*	.	.	.
X	127234016	T	C	intergenic	*ACTRT1,SMARCA1*	.	.	.
X	127338061	G	T	intergenic	*ACTRT1,SMARCA1*	.	.	.

X	127338616	T	C	intergenic	*ACTRT1,SMARCA1*	.	.	.
X	127338617	G	A	intergenic	*ACTRT1,SMARCA1*	.	.	.
X	127485635	T	C	intergenic	*ACTRT1,SMARCA1*	.	.	.
X	127523024	C	G	intergenic	*ACTRT1,SMARCA1*	.	.	.
X	127547464	C	T	intergenic	*ACTRT1,SMARCA1*	.	.	.
X	127709139	C	G	intergenic	*ACTRT1,SMARCA1*	.	.	.
X	127726272	G	A	intergenic	*ACTRT1,SMARCA1*	.	.	.
X	127803069	C	T	intergenic	*ACTRT1,SMARCA1*	.	.	.
X	127805356	G	A	intergenic	*ACTRT1,SMARCA1*	.	.	.
X	127807224	A	G	intergenic	*ACTRT1,SMARCA1*	.	.	.
X	127807805	T	G	intergenic	*ACTRT1,SMARCA1*	.	.	.
X	127807806	C	A	intergenic	*ACTRT1,SMARCA1*	.	.	.
X	127838225	T	C	intergenic	*ACTRT1,SMARCA1*	.	.	.
X	127918940	T	C	intergenic	*ACTRT1,SMARCA1*	.	.	.
X	127919639	G	T	intergenic	*ACTRT1,SMARCA1*	.	.	.
X	127925350	T	C	intergenic	*ACTRT1,SMARCA1*	.	.	.
X	127925352	G	A	intergenic	*ACTRT1,SMARCA1*	.	.	.
X	127925672	A	G	intergenic	*ACTRT1,SMARCA1*	.	.	.
X	127926997	C	T	intergenic	*ACTRT1,SMARCA1*	.	.	.

X	127926998	A	G	intergenic	*ACTRT1,SMARCA1*	.	.	.
X	127978041	C	T	intergenic	*ACTRT1,SMARCA1*	.	.	.
X	128077798	T	C	intergenic	*ACTRT1,SMARCA1*	.	.	.
X	128135978	T	C	intergenic	*ACTRT1,SMARCA1*	.	.	.
X	128156730	A	G	intergenic	*ACTRT1,SMARCA1*	.	.	.
X	128179921	G	T	intergenic	*ACTRT1,SMARCA1*	.	.	.
X	128290613	T	C	intergenic	*ACTRT1,SMARCA1*	.	.	.
X	128300834	T	C	intergenic	*ACTRT1,SMARCA1*	.	.	.
X	128378180	T	C	intergenic	*ACTRT1,SMARCA1*	.	.	.
X	128385220	A	G	intergenic	*ACTRT1,SMARCA1*	.	.	.
X	128385221	G	A	intergenic	*ACTRT1,SMARCA1*	.	.	.
X	128425848	C	T	intergenic	*ACTRT1,SMARCA1*	.	.	.
X	128425849	A	G	intergenic	*ACTRT1,SMARCA1*	.	.	.
X	128488753	T	C	intergenic	*ACTRT1,SMARCA1*	.	.	.
X	128488927	G	A	intergenic	*ACTRT1,SMARCA1*	.	.	.
X	128498977	A	G	intergenic	*ACTRT1,SMARCA1*	.	.	.
X	128535427	A	G	intergenic	*ACTRT1,SMARCA1*	.	.	.
X	128539131	T	C	intergenic	*ACTRT1,SMARCA1*	.	.	.
1	2937914	GTCTC	G	upstream	*ACTRT2*	.	.	.
2	158699946	G	A	intronic	*ACVR1*	.	.	.

2	158687210	C	T	intronic	*ACVR1*	.	.	.
2	158772771	A	T	intergenic	*ACVR1,UPP2*	.	.	.
12	52375293	G	T	intronic	*ACVR1B*	.	.	.
12	52387775	C	T	exonic	*ACVR1B*	nonsynonymous_SNV	.	.
2	158587225	T	C	intergenic	*ACVR1C,ACVR1*	.	.	.
2	54361300	T	C	intronic	*ACYP2*	.	.	.
2	54400702	C	T	intronic	*ACYP2*	.	.	.
2	54401354	A	G	intronic	*ACYP2*	.	.	.
2	54401739	T	C	intronic	*ACYP2*	.	.	.
2	54401740	G	A	intronic	*ACYP2*	.	.	.
20	43281379	TTTA	T	intergenic	*ADA,LINC01260*	.	.	.
10	127711414	C	T	intronic	*ADAM12*	.	.	.
10	127950356	A	T	intronic	*ADAM12*	.	.	.
10	127861208	T	C	intronic	*ADAM12*	.	.	.
10	127861209	G	A	intronic	*ADAM12*	.	.	.
10	128033153	G	A	intronic	*ADAM12*	.	.	.
10	128034143	G	A	intronic	*ADAM12*	.	.	.
10	128034802	G	A	intronic	*ADAM12*	.	.	.
10	128067913	C	T	intronic	*ADAM12*	.	.	.
8	39447280	T	C	intronic	*ADAM18*	.	.	.
8	39570717	A	C	intronic	*ADAM18*	.	.	.
8	39571097	A	T	intronic	*ADAM18*	.	.	.
5	157008117	G	T	intergenic	*ADAM19,SOX30*	.	.	.
5	157049363	A	G	intergenic	*ADAM19,SOX30*	.	.	.
8	39667397	C	T	intronic	*ADAM2*	.	.	.
8	39668020	G	A	intronic	*ADAM2*	.	.	.
8	39668127	T	C	intronic	*ADAM2*	.	.	.
8	39668850	T	C	intronic	*ADAM2*	.	.	.
8	39669337	G	A	intronic	*ADAM2*	.	.	.
8	39670390	G	T	intronic	*ADAM2*	.	.	.
14	70945746	A	C	ncRNA_intronic	*ADAM20P1*	.	.	.
14	70981524	C	A	intergenic	*ADAM20P1,ADAM20*	.	.	.
14	70967469	T	C	intergenic	*ADAM20P1,ADAM20*	.	.	.
14	70967470	G	T	intergenic	*ADAM20P1,ADAM20*	.	.	.

14	70970981	C	T	intergenic	*ADAM20P1,ADAM20*	.	.	.
14	70971064	G	T	intergenic	*ADAM20P1,ADAM20*	.	.	.
14	70774246	T	G	intergenic	*ADAM21P1,COX16*	.	.	.
14	70774442	A	G	intergenic	*ADAM21P1,COX16*	.	.	.
14	70775484	T	G	intergenic	*ADAM21P1,COX16*	.	.	.
7	87582515	T	C	intronic	*ADAM22*	.	.	.
7	87583723	C	G	intronic	*ADAM22*	.	.	.
7	87653717	C	T	intronic	*ADAM22*	.	.	.
7	87710082	T	G	intronic	*ADAM22*	.	.	.
2	207362748	C	T	intronic	*ADAM23*	.	.	.
2	207363167	C	G	intronic	*ADAM23*	.	.	.
4	175852967	TCACA	T	intronic	*ADAM29*	.	.	.
4	175874353	C	A	intronic	*ADAM29*	.	.	.
4	176300782	G	T	intergenic	*ADAM29,GPM6A*	.	.	.
4	176145336	A	T	intergenic	*ADAM29,GPM6A*	.	.	.
4	176312833	C	CT	intergenic	*ADAM29,GPM6A*	.	.	.
4	176521827	C	T	intergenic	*ADAM29,GPM6A*	.	.	.
4	176522576	A	G	intergenic	*ADAM29,GPM6A*	.	.	.
4	176524367	T	G	intergenic	*ADAM29,GPM6A*	.	.	.
4	176524502	T	C	intergenic	*ADAM29,GPM6A*	.	.	.
8	38997318	T	C	intronic	*ADAM32*	.	.	.
8	38997320	T	C	intronic	*ADAM32*	.	.	.
8	38998624	T	C	intronic	*ADAM32*	.	.	.
8	38999048	A	G	intronic	*ADAM32*	.	.	.
8	39033501	C	T	intronic	*ADAM32*	.	.	.
8	39293857	A	G	intergenic	*ADAM5,ADAM3A*	.	.	.
8	39294462	C	T	intergenic	*ADAM5,ADAM3A*	.	.	.
8	39294785	C	A	intergenic	*ADAM5,ADAM3A*	.	.	.
14	106496293	A	G	intergenic	*ADAM6,LINC00226*	.	.	.
14	106698293	T	C	intergenic	*ADAM6,LINC00226*	.	.	.

14	106444881	G	A	intergenic	*ADAM6,LINC00226*	.	.	.
14	106563103	T	C	intergenic	*ADAM6,LINC00226*	.	.	.
14	106616549	C	T	intergenic	*ADAM6,LINC00226*	.	.	.
14	106616776	T	G	intergenic	*ADAM6,LINC00226*	.	.	.
14	106617070	G	A	intergenic	*ADAM6,LINC00226*	.	.	.
14	106618341	A	C	intergenic	*ADAM6,LINC00226*	.	.	.
14	106618770	A	C	intergenic	*ADAM6,LINC00226*	.	.	.
14	106702164	T	C	intergenic	*ADAM6,LINC00226*	.	.	.
5	33857017	G	T	intronic	*ADAMTS12*	.	.	.
5	33543345	T	C	intronic	*ADAMTS12*	.	.	.
5	33543347	A	C	intronic	*ADAMTS12*	.	.	.
5	33543604	T	C	intronic	*ADAMTS12*	.	.	.
5	33543605	G	C	intronic	*ADAMTS12*	.	.	.
5	33560708	A	G	intronic	*ADAMTS12*	.	.	.
5	33573058	T	C	intronic	*ADAMTS12*	.	.	.
5	33860411	A	G	intronic	*ADAMTS12*	.	.	.
5	33861613	C	G	intronic	*ADAMTS12*	.	.	.
5	33861615	A	G	intronic	*ADAMTS12*	.	.	.
5	33863232	C	T	intronic	*ADAMTS12*	.	.	.
5	33916021	G	T	intergenic	*ADAMTS12,RXFP3*	.	.	.
11	130409884	G	C	intergenic	*ADAMTS15,MIR8052*	.	.	.
5	5274850	G	GATAT	intronic	*ADAMTS16*	.	.	.
5	5386498	C	G	intergenic	*ADAMTS16,ICE1*	.	.	.
15	100611376	T	A	intronic	*ADAMTS17*	.	.	.
15	100626715	T	C	intronic	*ADAMTS17*	.	.	.
15	100628480	T	C	intronic	*ADAMTS17*	.	.	.
16	77742761	T	C	intergenic	*ADAMTS18,NUDT7*	.	.	.
16	77539995	G	A	intergenic	*ADAMTS18,NUDT7*	.	.	.
16	77562005	T	A	intergenic	*ADAMTS18,NUDT7*	.	.	.

5	128869769	C	T	intronic	*ADAMTS19*	.	.	.
5	128881315	A	G	intronic	*ADAMTS19*	.	.	.
5	128915131	A	T	intronic	*ADAMTS19*	.	.	.
5	128915773	T	C	intronic	*ADAMTS19*	.	.	.
5	128947353	A	G	intronic	*ADAMTS19*	.	.	.
5	128948167	A	AC	intronic	*ADAMTS19*	.	.	.
5	128949726	A	G	intronic	*ADAMTS19*	.	.	.
5	128963795	T	C	intronic	*ADAMTS19*	.	.	.
5	129058011	C	T	intronic	*ADAMTS19*	.	.	.
5	178813020	G	T	intergenic	*ADAMTS2,RUFY1*	.	.	.
5	178880385	A	C	intergenic	*ADAMTS2,RUFY1*	.	.	.
5	178938644	G	A	intergenic	*ADAMTS2,RUFY1*	.	.	.
5	178940964	T	A	intergenic	*ADAMTS2,RUFY1*	.	.	.
5	178940965	G	A	intergenic	*ADAMTS2,RUFY1*	.	.	.
12	43779264	A	C	intronic	*ADAMTS20*	.	.	.
12	43779806	T	C	intronic	*ADAMTS20*	.	.	.
12	44085086	G	C	intergenic	*ADAMTS20,PUS7L*	.	.	.
12	44003449	T	C	intergenic	*ADAMTS20,PUS7L*	.	.	.
12	44003752	G	A	intergenic	*ADAMTS20,PUS7L*	.	.	.
12	44005916	T	C	intergenic	*ADAMTS20,PUS7L*	.	.	.
12	44083492	G	A	intergenic	*ADAMTS20,PUS7L*	.	.	.
4	73328374	G	T	intronic	*ADAMTS3*	.	.	.
4	73382738	A	G	intronic	*ADAMTS3*	.	.	.
4	73460955	T	C	intergenic	*ADAMTS3,COX18*	.	.	.
4	73499441	T	C	intergenic	*ADAMTS3,COX18*	.	.	.
4	73520829	G	T	intergenic	*ADAMTS3,COX18*	.	.	.
4	73716798	T	A	intergenic	*ADAMTS3,COX18*	.	.	.
4	73720863	T	A	intergenic	*ADAMTS3,COX18*	.	.	.
4	73735684	G	A	intergenic	*ADAMTS3,COX18*	.	.	.
4	73738028	G	A	intergenic	*ADAMTS3,COX18*	.	.	.
4	73738030	A	G	intergenic	*ADAMTS3,COX18*	.	.	.
4	73773770	T	C	intergenic	*ADAMTS3,COX18*	.	.	.
21	28326201	A	G	intronic	*ADAMTS5*	.	.	.
21	28321455	GATAT	G	intronic	*ADAMTS5*	.	.	.
21	28405208	C	T	intergenic	*ADAMTS5,MIR5009*	.	.	.
5	64728100	C	G	intronic	*ADAMTS6*	.	.	.
5	64728101	T	A	intronic	*ADAMTS6*	.	.	.

5	64617358	T	C	intronic	*ADAMTS6*	.	.	.
5	64705406	G	A	intronic	*ADAMTS6*	.	.	.
5	64781781	G	A	intergenic	*ADAMTS6,CENPK*	.	.	.
3	64595185	T	TA	intronic	*ADAMTS9*	.	.	.
3	64501505	G	A	UTR3	*ADAMTS9*	.	.	.
3	64967328	C	T	ncRNA_intronic	*ADAMTS9-AS2*	.	.	.
3	65005529	A	G	intergenic	*ADAMTS9-AS2,MAGI1*	.	.	.
3	65043308	C	T	intergenic	*ADAMTS9-AS2,MAGI1*	.	.	.
3	65050608	C	T	intergenic	*ADAMTS9-AS2,MAGI1*	.	.	.
3	65050609	A	T	intergenic	*ADAMTS9-AS2,MAGI1*	.	.	.
3	65060976	T	A	intergenic	*ADAMTS9-AS2,MAGI1*	.	.	.
3	64764946	T	C	ncRNA_intronic	*ADAMTS9-AS2,MIR548A2*	.	.	.
9	18728111	T	A	intronic	*ADAMTSL1*	.	.	.
9	18798621	A	G	intronic	*ADAMTSL1*	.	.	.
9	18826052	A	G	intronic	*ADAMTSL1*	.	.	.
9	18728459	GAA	G	intronic	*ADAMTSL1*	.	.	.
9	18517725	G	GAAC	intronic	*ADAMTSL1*	.	.	.
9	18517727	CCCT	C	intronic	*ADAMTSL1*	.	.	.
9	18913559	A	T	intergenic	*ADAMTSL1,SAXO1*	.	.	.
15	84574659	T	A	intronic	*ADAMTSL3*	.	.	.
15	84447811	C	G	intronic	*ADAMTSL3*	.	.	.
15	84447813	G	T	intronic	*ADAMTSL3*	.	.	.
15	84491345	G	A	intronic	*ADAMTSL3*	.	.	.
15	84533962	C	T	intronic	*ADAMTSL3*	.	.	.
15	84534343	G	A	intronic	*ADAMTSL3*	.	.	.
15	84534459	A	G	intronic	*ADAMTSL3*	.	.	.
15	84692835	G	A	intronic	*ADAMTSL3*	.	.	.
15	84698178	T	C	intronic	*ADAMTSL3*	.	.	.
15	84698179	G	T	intronic	*ADAMTSL3*	.	.	.
15	84735468	A	G	intergenic	*ADAMTSL3,EFTUD1P1*	.	.	.

15	84712835	A	G	intergenic	*ADAMTSL3,EFTUD1P1*	.	.	.
1	154580346	A	G	intronic	*ADAR*	.	.	.
1	154613563	T	A	intergenic	*ADAR,KCNN3*	.	.	.
1	154668034	G	T	intergenic	*ADAR,KCNN3*	.	.	.
1	154657911	C	A	intergenic	*ADAR,KCNN3*	.	.	.
10	1279164	C	T	intronic	*ADARB2*	.	.	.
10	1711718	T	A	intronic	*ADARB2*	.	.	.
10	1568737	G	C	intronic	*ADARB2*	.	.	.
10	1268379	C	T	intronic	*ADARB2*	.	.	.
10	1269037	T	A	intronic	*ADARB2*	.	.	.
10	1937940	T	A	intergenic	*ADARB2,LINC00700*	.	.	.
10	1909272	C	CATAT	intergenic	*ADARB2,LINC00700*	.	.	.
10	1810507	A	T	intergenic	*ADARB2,LINC00700*	.	.	.
10	1952781	T	C	intergenic	*ADARB2,LINC00700*	.	.	.
6	143745741	T	C	UTR3	*ADAT2*	.	.	.
14	78268189	G	GC	intronic	*ADCK1*	.	.	.
14	78691934	A	G	intergenic	*ADCK1,NONE*	.	.	.
7	140395029	GCACA	G	downstream	*ADCK2,NDUFB2-AS1*	.	.	.
1	227159747	A	G	intronic	*ADCK3*	.	.	.
7	45706122	G	A	intronic	*ADCY1*	.	.	.
7	45708046	G	A	intronic	*ADCY1*	.	.	.
1	167855330	A	G	intronic	*ADCY10*	.	.	.
1	167855934	C	T	intronic	*ADCY10*	.	.	.
5	7476871	C	A	intronic	*ADCY2*	.	.	.
5	7764010	G	A	intronic	*ADCY2*	.	.	.
5	7426969	G	T	intronic	*ADCY2*	.	.	.
5	7553441	C	T	intronic	*ADCY2*	.	.	.
5	7721362	A	T	intronic	*ADCY2*	.	.	.
2	25128260	C	T	intronic	*ADCY3*	.	.	.
3	123008588	C	T	intronic	*ADCY5*	.	.	.
8	131850664	A	G	intronic	*ADCY8*	.	.	.
8	131925617	A	C	intronic	*ADCY8*	.	.	.

8	131940800	TCA	T	intronic	*ADCY8*	.	.	.
8	131981232	C	T	intronic	*ADCY8*	.	.	.
8	132821874	C	A	intergenic	*ADCY8,EFR3A*	.	.	.
8	132757005	A	T	intergenic	*ADCY8,EFR3A*	.	.	.
8	132817367	G	C	intergenic	*ADCY8,EFR3A*	.	.	.
8	132075316	C	T	intergenic	*ADCY8,EFR3A*	.	.	.
8	132079056	G	A	intergenic	*ADCY8,EFR3A*	.	.	.
8	132093574	A	G	intergenic	*ADCY8,EFR3A*	.	.	.
8	132223223	C	T	intergenic	*ADCY8,EFR3A*	.	.	.
8	132223225	A	G	intergenic	*ADCY8,EFR3A*	.	.	.
8	132265896	T	C	intergenic	*ADCY8,EFR3A*	.	.	.
8	132265897	G	A	intergenic	*ADCY8,EFR3A*	.	.	.
8	132266954	A	G	intergenic	*ADCY8,EFR3A*	.	.	.
8	132268333	A	T	intergenic	*ADCY8,EFR3A*	.	.	.
8	132334429	A	G	intergenic	*ADCY8,EFR3A*	.	.	.
8	132336659	T	C	intergenic	*ADCY8,EFR3A*	.	.	.
8	132451532	G	A	intergenic	*ADCY8,EFR3A*	.	.	.
8	132561063	A	T	intergenic	*ADCY8,EFR3A*	.	.	.
8	132842640	G	T	intergenic	*ADCY8,EFR3A*	.	.	.
8	132842642	C	T	intergenic	*ADCY8,EFR3A*	.	.	.
8	132892331	C	T	intergenic	*ADCY8,EFR3A*	.	.	.
18	1235795	C	A	intergenic	*ADCYAP1,LINC00470*	.	.	.
18	1025991	C	A	intergenic	*ADCYAP1,LINC00470*	.	.	.
18	969771	G	C	intergenic	*ADCYAP1,LINC00470*	.	.	.
18	972028	G	A	intergenic	*ADCYAP1,LINC00470*	.	.	.
18	974183	A	T	intergenic	*ADCYAP1,LINC00470*	.	.	.
18	977621	G	C	intergenic	*ADCYAP1,LINC00470*	.	.	.
18	977622	T	A	intergenic	*ADCYAP1,LINC00470*	.	.	.
18	1026500	T	C	intergenic	*ADCYAP1,LINC00470*	.	.	.

18	1032118	T	G	intergenic	*ADCYAP1,LINC00470*	.	.	.
18	1102786	G	A	intergenic	*ADCYAP1,LINC00470*	.	.	.
18	1158517	A	T	intergenic	*ADCYAP1,LINC00470*	.	.	.
7	31110490	T	C	intronic	*ADCYAP1R1*	.	.	.
7	31189673	TA	T	intergenic	*ADCYAP1R1,NEUROD6*	.	.	.
7	31240974	C	A	intergenic	*ADCYAP1R1,NEUROD6*	.	.	.
7	31224222	C	CTATA	intergenic	*ADCYAP1R1,NEUROD6*	.	.	.
7	31229177	A	AATT	intergenic	*ADCYAP1R1,NEUROD6*	.	.	.
7	31187352	G	A	intergenic	*ADCYAP1R1,NEUROD6*	.	.	.
7	31188515	A	C	intergenic	*ADCYAP1R1,NEUROD6*	.	.	.
7	31367556	C	G	intergenic	*ADCYAP1R1,NEUROD6*	.	.	.
10	111940092	C	T	intergenic	*ADD3,MXI1*	.	.	.
6	147021534	T	C	intronic	*ADGB*	.	.	.
10	134926695	TAAGCCGCGTCGGTTATTTGAGGTCTGC	T	intronic	*ADGRA1*	.	.	.
10	134897938	T	A	downstream	*ADGRA1-AS1*	.	.	.
4	22481283	A	G	intronic	*ADGRA3*	.	.	.
4	22590560	G	T	intergenic	*ADGRA3,GBA3*	.	.	.
4	22553578	A	G	intergenic	*ADGRA3,GBA3*	.	.	.
6	69590088	G	T	intronic	*ADGRB3*	.	.	.
6	69648507	T	C	intronic	*ADGRB3*	.	.	.
6	69826145	C	T	intronic	*ADGRB3*	.	.	.
6	69830816	A	G	intronic	*ADGRB3*	.	.	.
6	69835467	C	A	intronic	*ADGRB3*	.	.	.
6	69478701	G	A	intronic	*ADGRB3*	.	.	.
6	69496982	A	C	intronic	*ADGRB3*	.	.	.

6	70143668	A	T	intergenic	*ADGRB3,LMBRD1*	.	.	.
6	70121931	A	G	intergenic	*ADGRB3,LMBRD1*	.	.	.
6	70152878	C	T	intergenic	*ADGRB3,LMBRD1*	.	.	.
6	70152879	A	G	intergenic	*ADGRB3,LMBRD1*	.	.	.
6	70153031	A	G	intergenic	*ADGRB3,LMBRD1*	.	.	.
6	70193481	C	A	intergenic	*ADGRB3,LMBRD1*	.	.	.
6	70210575	T	C	intergenic	*ADGRB3,LMBRD1*	.	.	.
6	70271308	G	GCATCAA	intergenic	*ADGRB3,LMBRD1*	.	.	.
6	70274666	T	C	intergenic	*ADGRB3,LMBRD1*	.	.	.
12	131530219	C	T	intronic	*ADGRD1*	.	.	.
12	131516409	G	T	intronic	*ADGRD1*	.	.	.
12	131554162	G	T	intronic	*ADGRD1*	.	.	.
12	131627834	T	A	intergenic	*ADGRD1,LINC01257*	.	.	.
6	47056710	G	A	intergenic	*ADGRF1,TNFRSF21*	.	.	.
6	47185004	C	T	intergenic	*ADGRF1,TNFRSF21*	.	.	.
6	47185558	A	C	intergenic	*ADGRF1,TNFRSF21*	.	.	.
6	47186997	C	A	intergenic	*ADGRF1,TNFRSF21*	.	.	.
6	47696708	G	T	intergenic	*ADGRF4,OPN5*	.	.	.
6	46832514	G	C	intronic	*ADGRF5*	.	.	.
6	46906733	A	G	intronic	*ADGRF5*	.	.	.
X	19159202	T	C	intergenic	*ADGRG2,PDHA1*	.	.	.
X	19159203	G	A	intergenic	*ADGRG2,PDHA1*	.	.	.
X	19239140	T	A	intergenic	*ADGRG2,PDHA1*	.	.	.
X	19239142	T	C	intergenic	*ADGRG2,PDHA1*	.	.	.
X	19248294	G	GT	intergenic	*ADGRG2,PDHA1*	.	.	.
X	19351910	G	A	intergenic	*ADGRG2,PDHA1*	.	.	.
X	135495347	C	T	intronic	*ADGRG4*	.	.	.
6	142761994	G	A	intronic	*ADGRG6*	.	.	.
6	142744665	G	T	intronic	*ADGRG6*	.	.	.
6	142837561	T	C	intergenic	*ADGRG6,LOC153910*	.	.	.
6	142837562	G	T	intergenic	*ADGRG6,LOC153910*	.	.	.

3	100343777	T	C	intronic	*ADGRG7*	.	.	.
3	100405785	T	A	intronic	*ADGRG7*	.	.	.
1	82438914	C	A	intronic	*ADGRL2*	.	.	.
1	82434056	A	C	intronic	*ADGRL2*	.	.	.
1	82190783	G	C	intronic	*ADGRL2*	.	.	.
1	82803945	C	T	intergenic	*ADGRL2,LINC01361*	.	.	.
1	82510629	G	T	intergenic	*ADGRL2,LINC01361*	.	.	.
1	82827055	GATAC	G	intergenic	*ADGRL2,LINC01361*	.	.	.
1	83050058	A	G	intergenic	*ADGRL2,LINC01361*	.	.	.
4	62493851	T	C	intronic	*ADGRL3*	.	.	.
4	62549981	G	A	intronic	*ADGRL3*	.	.	.
4	62644721	A	G	intronic	*ADGRL3*	.	.	.
4	62624266	T	C	intronic	*ADGRL3*	.	.	.
4	62831295	A	G	intronic	*ADGRL3*	.	.	.
4	62838451	A	G	intronic	*ADGRL3*	.	.	.
4	63387578	A	T	intergenic	*ADGRL3-AS1,TECRL*	.	.	.
4	64008793	G	A	intergenic	*ADGRL3-AS1,TECRL*	.	.	.
4	64141662	C	A	intergenic	*ADGRL3-AS1,TECRL*	.	.	.
4	63404841	A	T	intergenic	*ADGRL3-AS1,TECRL*	.	.	.
4	63373761	T	C	intergenic	*ADGRL3-AS1,TECRL*	.	.	.
4	63942970	C	T	intergenic	*ADGRL3-AS1,TECRL*	.	.	.
4	63943312	T	G	intergenic	*ADGRL3-AS1,TECRL*	.	.	.
4	64651336	A	T	intergenic	*ADGRL3-AS1,TECRL*	.	.	.
4	63432550	T	C	intergenic	*ADGRL3-AS1,TECRL*	.	.	.

4	63432551	G	C	intergenic	*ADGRL3-AS1,TECRL*	.	.	.
4	63434483	A	T	intergenic	*ADGRL3-AS1,TECRL*	.	.	.
4	63892755	G	A	intergenic	*ADGRL3-AS1,TECRL*	.	.	.
4	63965549	C	A	intergenic	*ADGRL3-AS1,TECRL*	.	.	.
4	63967551	T	C	intergenic	*ADGRL3-AS1,TECRL*	.	.	.
4	63983289	C	T	intergenic	*ADGRL3-AS1,TECRL*	.	.	.
4	64030935	C	T	intergenic	*ADGRL3-AS1,TECRL*	.	.	.
4	64047270	A	T	intergenic	*ADGRL3-AS1,TECRL*	.	.	.
4	64134002	A	T	intergenic	*ADGRL3-AS1,TECRL*	.	.	.
4	64202004	C	T	intergenic	*ADGRL3-AS1,TECRL*	.	.	.
4	64202305	G	A	intergenic	*ADGRL3-AS1,TECRL*	.	.	.
4	64374957	A	G	intergenic	*ADGRL3-AS1,TECRL*	.	.	.
4	64456928	T	A	intergenic	*ADGRL3-AS1,TECRL*	.	.	.
4	64460247	A	G	intergenic	*ADGRL3-AS1,TECRL*	.	.	.
4	64605568	A	G	intergenic	*ADGRL3-AS1,TECRL*	.	.	.
4	64711914	G	T	intergenic	*ADGRL3-AS1,TECRL*	.	.	.
4	64813847	T	A	intergenic	*ADGRL3-AS1,TECRL*	.	.	.
4	64813848	C	G	intergenic	*ADGRL3-AS1,TECRL*	.	.	.
4	64814874	G	A	intergenic	*ADGRL3-AS1,TECRL*	.	.	.

4	65062868	T	TATGCAGAT	intergenic	*ADGRL3-AS1,TECRL*	.	.	.
1	79608172	TACAC	T	intergenic	*ADGRL4,LOC101927412*	.	.	.
1	79577776	A	T	intergenic	*ADGRL4,LOC101927412*	.	.	.
1	80386592	A	C	intergenic	*ADGRL4,LOC101927412*	.	.	.
1	80078647	CT	C	intergenic	*ADGRL4,LOC101927412*	.	.	.
1	80533415	C	G	intergenic	*ADGRL4,LOC101927412*	.	.	.
1	79635064	T	G	intergenic	*ADGRL4,LOC101927412*	.	.	.
1	79882826	A	C	intergenic	*ADGRL4,LOC101927412*	.	.	.
1	79885004	C	A	intergenic	*ADGRL4,LOC101927412*	.	.	.
1	80409338	G	T	intergenic	*ADGRL4,LOC101927412*	.	.	.
1	80533902	C	G	intergenic	*ADGRL4,LOC101927412*	.	.	.
1	80598908	A	T	intergenic	*ADGRL4,LOC101927412*	.	.	.
1	80809746	T	C	intergenic	*ADGRL4,LOC101927412*	.	.	.
1	80841576	GCACACACA	G	intergenic	*ADGRL4,LOC101927412*	.	.	.
1	80980519	C	T	intergenic	*ADGRL4,LOC101927412*	.	.	.
1	80980520	G	T	intergenic	*ADGRL4,LOC101927412*	.	.	.
1	80981867	T	A	intergenic	*ADGRL4,LOC101927412*	.	.	.
5	89873369	T	A	intronic	*ADGRV1*	.	.	.
5	89874031	T	C	intronic	*ADGRV1*	.	.	.
5	89874713	T	C	intronic	*ADGRV1*	.	.	.
5	89874714	G	A	intronic	*ADGRV1*	.	.	.

5	89961173	T	C	intronic	*ADGRV1*	.	.	.
5	89961174	G	C	intronic	*ADGRV1*	.	.	.
5	90093883	G	A	intronic	*ADGRV1*	.	.	.
5	90115273	C	A	intronic	*ADGRV1*	.	.	.
5	90118635	A	G	intronic	*ADGRV1*	.	.	.
5	90128790	A	G	intronic	*ADGRV1*	.	.	.
5	90128792	G	A	intronic	*ADGRV1*	.	.	.
5	90220802	A	G	intronic	*ADGRV1*	.	.	.
5	90271351	C	A	intronic	*ADGRV1*	.	.	.
5	90372689	TA	T	intronic	*ADGRV1*	.	.	.
5	90377464	A	G	intronic	*ADGRV1*	.	.	.
5	90380196	T	C	intronic	*ADGRV1*	.	.	.
5	90388162	C	T	intronic	*ADGRV1*	.	.	.
4	100342428	A	C	intronic	*ADH7*	.	.	.
4	100342853	T	C	intronic	*ADH7*	.	.	.
4	100342855	G	A	intronic	*ADH7*	.	.	.
4	100418044	G	C	intergenic	*ADH7,C4orf17*	.	.	.
8	67376668	A	G	intronic	*ADHFE1*	.	.	.
12	1863680	AG	A	splicing	*ADIPOR2*	.	.	.
12	1876623	A	G	intronic	*ADIPOR2*	.	.	.
10	75995766	C	T	intronic	*ADK*	.	.	.
10	76110275	C	G	intronic	*ADK*	.	.	.
10	76111580	A	T	intronic	*ADK*	.	.	.
10	76111581	C	CA	intronic	*ADK*	.	.	.
10	76161263	A	G	intronic	*ADK*	.	.	.
10	76216867	A	G	intronic	*ADK*	.	.	.
10	76325944	A	T	intronic	*ADK*	.	.	.
10	76329285	A	G	intronic	*ADK*	.	.	.
10	76330387	T	C	intronic	*ADK*	.	.	.
10	76331490	G	C	intronic	*ADK*	.	.	.
10	76355800	T	A	intronic	*ADK*	.	.	.
10	76378770	TTGTGTGTGTGTGTGTGTGTGTGTG	T	intronic	*ADK*	.	.	.
10	76462182	C	T	intronic	*ADK*	.	.	.
1	203106980	C	G	intronic	*ADORA1*	.	.	.
22	24846845	A	G	ncRNA_intronic	*ADORA2A-AS1*	.	.	.

13	114101181	C	T	intronic	*ADPRHL1*	.	.	.
17	10601267	C	T	intronic	*ADPRM*	.	.	.
8	26885268	G	T	intergenic	*ADRA1A,STMN4*	.	.	.
8	26793447	T	C	intergenic	*ADRA1A,STMN4*	.	.	.
8	26794326	T	G	intergenic	*ADRA1A,STMN4*	.	.	.
8	26911782	A	G	intergenic	*ADRA1A,STMN4*	.	.	.
8	26911783	C	G	intergenic	*ADRA1A,STMN4*	.	.	.
8	26917475	T	C	intergenic	*ADRA1A,STMN4*	.	.	.
8	26983176	A	AG	intergenic	*ADRA1A,STMN4*	.	.	.
5	159394772	C	G	intronic	*ADRA1B*	.	.	.
5	159395574	G	A	intronic	*ADRA1B*	.	.	.
5	159415305	G	A	intergenic	*ADRA1B,TTC1*	.	.	.
20	4268191	C	A	intergenic	*ADRA1D,PRNP*	.	.	.
20	4396848	T	C	intergenic	*ADRA1D,PRNP*	.	.	.
20	4399473	A	T	intergenic	*ADRA1D,PRNP*	.	.	.
20	4399726	C	T	intergenic	*ADRA1D,PRNP*	.	.	.
20	4568532	C	CTGCCT	intergenic	*ADRA1D,PRNP*	.	.	.
20	4601397	C	G	intergenic	*ADRA1D,PRNP*	.	.	.
10	112982139	T	C	intergenic	*ADRA2A,GPAM*	.	.	.
10	113251593	T	G	intergenic	*ADRA2A,GPAM*	.	.	.
10	113438798	C	A	intergenic	*ADRA2A,GPAM*	.	.	.
10	113848385	A	G	intergenic	*ADRA2A,GPAM*	.	.	.
10	113685097	C	A	intergenic	*ADRA2A,GPAM*	.	.	.
10	112892796	A	G	intergenic	*ADRA2A,GPAM*	.	.	.
10	113054796	TA	T	intergenic	*ADRA2A,GPAM*	.	.	.
10	113106017	G	A	intergenic	*ADRA2A,GPAM*	.	.	.
10	113146415	A	T	intergenic	*ADRA2A,GPAM*	.	.	.
10	113147073	T	C	intergenic	*ADRA2A,GPAM*	.	.	.
10	113399903	A	G	intergenic	*ADRA2A,GPAM*	.	.	.
10	113401267	C	T	intergenic	*ADRA2A,GPAM*	.	.	.
10	113803403	T	C	intergenic	*ADRA2A,GPAM*	.	.	.
10	113825110	G	C	intergenic	*ADRA2A,GPAM*	.	.	.
4	3923115	G	C	intergenic	*ADRA2C,FAM86EP*	.	.	.
4	3814168	G	T	intergenic	*ADRA2C,FAM86EP*	.	.	.
4	3822452	T	C	intergenic	*ADRA2C,FAM86EP*	.	.	.

4	3892866	ACCCTGTGATAGGATTTTTGATATCCTAGGGAGATATTGCTCCTGACAGCAGAGTGGGCGTACAC	A	intergenic	*ADRA2C,FAM86EP*	.	.	.
4	3932186	T	C	intergenic	*ADRA2C,FAM86EP*	.	.	.
5	148360483	CAGAG	C	intergenic	*ADRB2,SH3TC2*	.	.	.
22	26043026	T	A	intronic	*ADRBK2*	.	.	.
12	19617770	C	T	intronic	*AEBP2*	.	.	.
12	20090757	C	A	intergenic	*AEBP2,LOC100506393*	.	.	.
12	19940303	GTTTTTTTTT	G	intergenic	*AEBP2,LOC100506393*	.	.	.
12	20165548	C	G	intergenic	*AEBP2,LOC100506393*	.	.	.
12	19721167	G	A	intergenic	*AEBP2,LOC100506393*	.	.	.
12	19830831	T	TA	intergenic	*AEBP2,LOC100506393*	.	.	.
12	19841041	A	G	intergenic	*AEBP2,LOC100506393*	.	.	.
4	7878725	C	G	intronic	*AFAP1*	.	.	.
4	7766381	C	T	ncRNA_intronic	*AFAP1-AS1*	.	.	.
10	116173777	C	A	intergenic	*AFAP1L2,ABLIM1*	.	.	.
X	147636094	C	T	intronic	*AFF2*	.	.	.
X	147641525	T	G	intronic	*AFF2*	.	.	.
X	147699019	A	G	intronic	*AFF2*	.	.	.
X	147775513	G	A	intronic	*AFF2*	.	.	.
X	147859736	G	A	intronic	*AFF2*	.	.	.
X	147860838	G	A	intronic	*AFF2*	.	.	.
X	148310182	T	A	intergenic	*AFF2,IDS*	.	.	.
X	148275299	C	T	intergenic	*AFF2,IDS*	.	.	.
X	148276790	T	C	intergenic	*AFF2,IDS*	.	.	.

X	148277460	C	G	intergenic	*AFF2,IDS*	.	.	.
X	148316974	T	C	intergenic	*AFF2,IDS*	.	.	.
X	148316975	G	A	intergenic	*AFF2,IDS*	.	.	.
X	148347455	A	C	intergenic	*AFF2,IDS*	.	.	.
X	148347648	G	A	intergenic	*AFF2,IDS*	.	.	.
2	100642848	A	C	intronic	*AFF3*	.	.	.
2	100358119	C	T	intronic	*AFF3*	.	.	.
2	100769123	C	T	intergenic	*AFF3,LINC01104*	.	.	.
5	132325093	A	G	intergenic	*AFF4,ZCCHC10*	.	.	.
5	132327338	ATT	A	intergenic	*AFF4,ZCCHC10*	.	.	.
18	12355493	T	C	intronic	*AFG3L2*	.	.	.
4	178444675	T	C	intergenic	*AGA,LINC01098*	.	.	.
4	178444676	G	A	intergenic	*AGA,LINC01098*	.	.	.
2	236867080	A	G	intronic	*AGAP1*	.	.	.
2	236832176	T	C	intronic	*AGAP1*	.	.	.
2	236600008	C	T	intronic	*AGAP1*	.	.	.
2	236990251	A	G	intronic	*AGAP1*	.	.	.
2	236764589	G	A	intronic	*AGAP1*	.	.	.
2	237046787	C	T	intergenic	*AGAP1,GBX2*	.	.	.
10	88767895	A	T	intronic	*AGAP11*	.	.	.
10	46340240	T	G	intronic	*AGAP4*	.	.	.
10	46336725	T	A	intronic	*AGAP4*	.	.	.
15	87056137	AT	A	intronic	*AGBL1*	.	.	.
15	87334959	TTATATA	T	intronic	*AGBL1*	.	.	.
15	87512555	C	CA	intronic	*AGBL1*	.	.	.
15	86951732	C	A	intronic	*AGBL1*	.	.	.
15	86791667	T	C	intronic	*AGBL1*	.	.	.
15	86750887	C	T	intronic	*AGBL1*	.	.	.
15	87106454	C	T	intronic	*AGBL1*	.	.	.
15	87291693	T	C	intronic	*AGBL1*	.	.	.
15	87362773	G	A	intronic	*AGBL1*	.	.	.
15	87480256	A	T	intronic	*AGBL1*	.	.	.
15	87480257	C	T	intronic	*AGBL1*	.	.	.
15	87546259	A	G	intronic	*AGBL1*	.	.	.
15	87546261	A	G	intronic	*AGBL1*	.	.	.
15	87894288	C	A	intergenic	*AGBL1,LINC00052*	.	.	.
15	87946846	A	G	intergenic	*AGBL1,LINC00052*	.	.	.
15	87722099	C	G	intergenic	*AGBL1,LINC00052*	.	.	.

15	87578021	T	TATACATGT/	intergenic	*AGBL1,LINC00052*	.	.	.
15	87656574	AG	A	intergenic	*AGBL1,LINC00052*	.	.	.
15	87656576	ACATTCTTC	A	intergenic	*AGBL1,LINC00052*	.	.	.
15	87773938	A	G	intergenic	*AGBL1,LINC00052*	.	.	.
15	87777585	A	C	intergenic	*AGBL1,LINC00052*	.	.	.
15	87796908	T	C	intergenic	*AGBL1,LINC00052*	.	.	.
15	87802760	T	A	intergenic	*AGBL1,LINC00052*	.	.	.
15	87819931	C	T	intergenic	*AGBL1,LINC00052*	.	.	.
15	87922880	T	A	intergenic	*AGBL1,LINC00052*	.	.	.
15	87923793	A	C	intergenic	*AGBL1,LINC00052*	.	.	.
15	87998028	T	C	intergenic	*AGBL1,LINC00052*	.	.	.
15	87998029	A	G	intergenic	*AGBL1,LINC00052*	.	.	.
11	47686786	G	A	intronic	*AGBL2*	.	.	.
11	47686787	T	C	intronic	*AGBL2*	.	.	.
7	134711149	C	T	intronic	*AGBL3*	.	.	.
7	134753009	C	A	intronic	*AGBL3*	.	.	.
7	134754527	T	G	intronic	*AGBL3*	.	.	.
1	50301837	G	C	intronic	*AGBL4*	.	.	.
1	50181305	C	CTGACT	intronic	*AGBL4*	.	.	.
1	49532907	C	G	intronic	*AGBL4*	.	.	.
1	50133781	A	C	intronic	*AGBL4*	.	.	.
1	50229470	T	A	intronic	*AGBL4*	.	.	.
1	49008533	C	T	intronic	*AGBL4*	.	.	.
1	49264142	C	T	intronic	*AGBL4*	.	.	.
1	49264143	T	C	intronic	*AGBL4*	.	.	.
1	49535068	G	A	intronic	*AGBL4*	.	.	.
1	49536619	T	C	intronic	*AGBL4*	.	.	.
1	49536620	G	A	intronic	*AGBL4*	.	.	.
1	49537127	A	G	intronic	*AGBL4*	.	.	.
1	49537128	C	T	intronic	*AGBL4*	.	.	.
1	49583418	G	A	intronic	*AGBL4*	.	.	.
1	49598466	A	G	intronic	*AGBL4*	.	.	.
1	49598468	T	G	intronic	*AGBL4*	.	.	.
1	49668760	C	A	intronic	*AGBL4*	.	.	.
1	49751387	G	A	intronic	*AGBL4*	.	.	.
1	49753174	C	T	intronic	*AGBL4*	.	.	.
1	49779635	G	A	intronic	*AGBL4*	.	.	.

1	50028382	G	A	intronic	*AGBL4*	.	.	.
1	50029202	G	T	intronic	*AGBL4*	.	.	.
1	50038314	C	G	intronic	*AGBL4*	.	.	.
1	50038316	A	G	intronic	*AGBL4*	.	.	.
1	50065327	G	C	intronic	*AGBL4*	.	.	.
1	50085788	A	C	intronic	*AGBL4*	.	.	.
1	50207022	T	G	intronic	*AGBL4*	.	.	.
1	50214697	A	G	intronic	*AGBL4*	.	.	.
1	49887473	T	C	ncRNA_intronic	*AGBL4-IT1*	.	.	.
2	228407719	C	T	intronic	*AGFG1*	.	.	.
2	228408539	T	A	intronic	*AGFG1*	.	.	.
2	228408541	A	G	intronic	*AGFG1*	.	.	.
7	100143813	T	A	intronic	*AGFG2*	.	.	.
5	76337809	G	A	intronic	*AGGF1*	.	.	.
5	76337810	G	T	intronic	*AGGF1*	.	.	.
7	141283582	A	G	intronic	*AGK*	.	.	.
7	15298788	A	G	intronic	*AGMO*	.	.	.
7	15312537	C	T	intronic	*AGMO*	.	.	.
7	15313445	T	C	intronic	*AGMO*	.	.	.
7	15513817	G	A	intronic	*AGMO*	.	.	.
7	15514320	G	A	intronic	*AGMO*	.	.	.
7	15579128	A	G	intronic	*AGMO*	.	.	.
7	15588897	C	A	intronic	*AGMO*	.	.	.
8	141665589	C	T	intergenic	*AGO2,PTK2*	.	.	.
21	45366258	G	C	intronic	*AGPAT3*	.	.	.
6	161594756	T	C	intronic	*AGPAT4*	.	.	.
6	161762654	C	A	intergenic	*AGPAT4,PARK2*	.	.	.
2	178321717	A	T	intronic	*AGPS*	.	.	.
2	178324062	C	A	intronic	*AGPS*	.	.	.
7	16871131	T	TGC	intergenic	*AGR2,AGR3*	.	.	.
7	16871131	T	TGTGC	intergenic	*AGR2,AGR3*	.	.	.
7	16915457	A	G	intronic	*AGR3*	.	.	.
7	16985057	T	C	intergenic	*AGR3,AHR*	.	.	.
7	17056834	C	T	intergenic	*AGR3,AHR*	.	.	.
7	17091075	T	G	intergenic	*AGR3,AHR*	.	.	.
7	17201331	C	G	intergenic	*AGR3,AHR*	.	.	.
7	17202476	A	G	intergenic	*AGR3,AHR*	.	.	.
7	17257206	G	A	intergenic	*AGR3,AHR*	.	.	.

7	17260006	G	A	intergenic	*AGR3,AHR*	.	.	.
9	88277187	T	C	intronic	*AGTPBP1*	.	.	.
9	88333574	T	A	intronic	*AGTPBP1*	.	.	.
9	88412772	G	A	intergenic	*AGTPBP1,LOC389765*	.	.	.
9	88404619	T	C	intergenic	*AGTPBP1,LOC389765*	.	.	.
9	88373843	A	C	intergenic	*AGTPBP1,LOC389765*	.	.	.
3	148497064	G	A	intergenic	*AGTR1,CPB1*	.	.	.
X	115520635	C	A	intergenic	*AGTR2,SLC6A14*	.	.	.
X	115322220	C	A	intergenic	*AGTR2,SLC6A14*	.	.	.
X	115442443	A	G	intergenic	*AGTR2,SLC6A14*	.	.	.
5	35020903	C	T	intronic	*AGXT2*	.	.	.
2	162460722	A	C	intergenic	*AHCTF1P1,SLC4A10*	.	.	.
2	162371258	T	C	intergenic	*AHCTF1P1,SLC4A10*	.	.	.
2	162371905	A	C	intergenic	*AHCTF1P1,SLC4A10*	.	.	.
2	162396393	T	C	intergenic	*AHCTF1P1,SLC4A10*	.	.	.
2	162424075	C	T	intergenic	*AHCTF1P1,SLC4A10*	.	.	.
2	162447616	A	C	intergenic	*AHCTF1P1,SLC4A10*	.	.	.
7	128941607	C	T	intronic	*AHCYL2*	.	.	.
7	128984895	T	C	intronic	*AHCYL2*	.	.	.
7	129019075	C	T	intronic	*AHCYL2*	.	.	.
6	135767560	T	C	intronic	*AHI1*	.	.	.
6	135817376	T	C	intronic	*AHI1*	.	.	.
6	135659163	C	T	intronic	*AHI1*	.	.	.
6	135791103	C	T	intronic	*AHI1*	.	.	.
6	135791104	A	G	intronic	*AHI1*	.	.	.
6	135798340	G	A	intronic	*AHI1*	.	.	.
11	62317368	GTTTTT	G	intergenic	*AHNAK,EEF1G*	.	.	.
7	17377619	C	T	intronic	*AHR*	.	.	.
7	17388912	G	C	intergenic	*AHR,KCCAT333*	.	.	.

7	17389299	C	T	intergenic	*AHR,KCCAT333*	.	.	.
7	17389300	A	G	intergenic	*AHR,KCCAT333*	.	.	.
16	31571518	T	A	intergenic	*AHSP,YBX3P1*	.	.	.
1	222861960	G	T	intronic	*AIDA*	.	.	.
X	129301546	G	A	intergenic	*AIFM1,RAB33A*	.	.	.
6	143408392	G	A	intronic	*AIG1*	.	.	.
6	143470704	T	A	intronic	*AIG1*	.	.	.
1	159079739	G	A	intergenic	*AIM2,CADM3*	.	.	.
1	159124738	A	G	intergenic	*AIM2,CADM3*	.	.	.
1	159125114	A	G	intergenic	*AIM2,CADM3*	.	.	.
4	107262190	G	A	intronic	*AIMP1*	.	.	.
1	4749140	C	A	intronic	*AJAP1*	.	.	.
1	4890942	A	T	intergenic	*AJAP1,MIR4417*	.	.	.
1	4924025	T	C	intergenic	*AJAP1,MIR4417*	.	.	.
1	5242926	T	C	intergenic	*AJAP1,MIR4417*	.	.	.
9	4778148	G	A	intergenic	*AK3,RCL1*	.	.	.
14	96898721	G	A	intronic	*AK7*	.	.	.
14	96962303	C	G	intergenic	*AK7,LOC730202*	.	.	.
6	109915695	C	T	intronic	*AK9*	.	.	.
6	109858977	A	G	intronic	*AK9*	.	.	.
6	109859721	C	T	intronic	*AK9*	.	.	.
6	109874072	G	A	intronic	*AK9*	.	.	.
6	109876558	C	A	intronic	*AK9*	.	.	.
6	109896874	T	C	intronic	*AK9*	.	.	.
6	109942280	T	C	intronic	*AK9*	.	.	.
6	109961017	C	T	intronic	*AK9*	.	.	.
6	109968156	T	G	intronic	*AK9*	.	.	.
6	109969540	A	C	intronic	*AK9*	.	.	.
6	109971509	G	A	intronic	*AK9*	.	.	.
6	110002031	G	C	intronic	*AK9*	.	.	.
6	110003867	A	T	intronic	*AK9*	.	.	.
17	19867271	C	G	intronic	*AKAP10*	.	.	.
13	42853399	CCACA	C	intronic	*AKAP11*	.	.	.
13	43120984	C	T	intergenic	*AKAP11,TNFSF11*	.	.	.
13	43103425	C	T	intergenic	*AKAP11,TNFSF11*	.	.	.
13	43038498	T	C	intergenic	*AKAP11,TNFSF11*	.	.	.
13	43046802	C	T	intergenic	*AKAP11,TNFSF11*	.	.	.
13	43047472	C	T	intergenic	*AKAP11,TNFSF11*	.	.	.

13	43078898	T	C	intergenic	*AKAP11,TNFSF11*	.	.	.
13	43093092	T	C	intergenic	*AKAP11,TNFSF11*	.	.	.
13	43094772	T	C	intergenic	*AKAP11,TNFSF11*	.	.	.
13	43095581	A	C	intergenic	*AKAP11,TNFSF11*	.	.	.
13	43095583	G	A	intergenic	*AKAP11,TNFSF11*	.	.	.
13	43131028	C	G	intergenic	*AKAP11,TNFSF11*	.	.	.
15	85953966	C	G	intronic	*AKAP13*	.	.	.
9	112868223	T	TTATA	intronic	*AKAP2,PALM2-AKAP2*	.	.	.
9	112819849	G	A	intronic	*AKAP2,PALM2-AKAP2*	.	.	.
X	49962623	T	C	intronic	*AKAP4*	.	.	.
14	32955606	T	C	intronic	*AKAP6*	.	.	.
14	32964806	A	T	intronic	*AKAP6*	.	.	.
14	32964807	A	T	intronic	*AKAP6*	.	.	.
14	32978373	A	C	intronic	*AKAP6*	.	.	.
14	32932674	G	A	intronic	*AKAP6*	.	.	.
14	32932676	C	G	intronic	*AKAP6*	.	.	.
14	32933436	G	C	intronic	*AKAP6*	.	.	.
14	32933686	T	A	intronic	*AKAP6*	.	.	.
14	32954839	A	G	intronic	*AKAP6*	.	.	.
14	32954840	C	T	intronic	*AKAP6*	.	.	.
14	32955362	C	T	intronic	*AKAP6*	.	.	.
14	32955779	T	A	intronic	*AKAP6*	.	.	.
14	32988841	G	A	intronic	*AKAP6*	.	.	.
14	32990186	C	A	intronic	*AKAP6*	.	.	.
14	32990187	A	G	intronic	*AKAP6*	.	.	.
14	33050079	A	G	intronic	*AKAP6*	.	.	.
14	33322042	C	T	intergenic	*AKAP6,NPAS3*	.	.	.
6	131505307	C	T	intronic	*AKAP7*	.	.	.
6	131522993	G	C	intronic	*AKAP7*	.	.	.
6	131550482	C	T	intronic	*AKAP7*	.	.	.
6	131556732	G	A	intronic	*AKAP7*	.	.	.
6	131557798	C	T	intronic	*AKAP7*	.	.	.
6	131849699	T	C	intergenic	*AKAP7,ARG1*	.	.	.
6	131850047	G	C	intergenic	*AKAP7,ARG1*	.	.	.
6	131850846	G	T	intergenic	*AKAP7,ARG1*	.	.	.
6	131861660	A	G	intergenic	*AKAP7,ARG1*	.	.	.

6	131863807	A	G	intergenic	*AKAP7,ARG1*	.	.	.
6	131863808	T	C	intergenic	*AKAP7,ARG1*	.	.	.
6	131864369	C	T	intergenic	*AKAP7,ARG1*	.	.	.
6	131865559	T	C	intergenic	*AKAP7,ARG1*	.	.	.
6	131865560	G	A	intergenic	*AKAP7,ARG1*	.	.	.
19	15495999	GGGATGGTCTCGATCTCCTGACCTCGTGATCCGCCCGCCTCGGCCTCCCAAAGTGCTGGGACTACAGGCGTGAGCCACCGCGCCCGGCCTTTCATGCCATTCTCTTGCCTCAGCCTCCCGAGTAGCTGGGACTACAGGCGCCCGCCACCACGTCTGGCTAATTTTTTGTATTTTTAGTAGAGATGGGGTTTCACCGTGTTAGCCA	G	intronic	*AKAP8L*	.	.	.
7	91712915	G	A	exonic	*AKAP9*	synonymous_SNV	.	.
7	91578114	G	A	intronic	*AKAP9*	.	.	.
7	91643220	ACATT	A	intronic	*AKAP9*	.	.	.

6	88460708	CGGGCAGGGGGCTGACCCCCCCACCTCCCTCCCGGACGGGGCGGCTCGCCT	C	intergenic	*AKIRIN2,LOC101928911*	.	.	.
6	88418613	A	G	intergenic	*AKIRIN2,LOC101928911*	.	.	.
6	88433300	GGAGA	G	intergenic	*AKIRIN2,LOC101928911*	.	.	.
9	117160602	G	A	intergenic	*AKNA,DFNB31*	.	.	.
10	5043017	C	A	intronic	*AKR1C2*	.	.	.
10	5142728	C	T	intronic	*AKR1C3*	.	.	.
10	5155288	C	A	intergenic	*AKR1C3,AKR1C8P*	.	.	.
10	5181886	T	A	intergenic	*AKR1C3,AKR1C8P*	.	.	.
10	5262792	T	C	intergenic	*AKR1C4,UCN3*	.	.	.
10	5324702	G	A	intergenic	*AKR1C4,UCN3*	.	.	.
10	4926374	T	A	ncRNA_intronic	*AKR1C6P*	.	.	.
10	4971489	T	C	intergenic	*AKR1C6P,AKR1C1*	.	.	.
19	40792835	C	G	intergenic	*AKT2,C19orf47*	.	.	.
1	243740292	T	C	intronic	*AKT3*	.	.	.
1	243848445	T	G	intronic	*AKT3*	.	.	.
1	243849204	T	C	intronic	*AKT3*	.	.	.
1	243867093	T	A	intronic	*AKT3*	.	.	.
1	243972891	G	C	intronic	*AKT3*	.	.	.
1	243974953	T	C	intronic	*AKT3*	.	.	.
1	243987880	C	T	intronic	*AKT3*	.	.	.
16	53545836	A	T	intergenic	*AKTIP,RPGRIP1L*	.	.	.
X	55048269	A	T	intronic	*ALAS2*	.	.	.
X	55061167	GACAC	G	intergenic	*ALAS2,PAGE2B*	.	.	.
X	55064483	T	A	intergenic	*ALAS2,PAGE2B*	.	.	.
X	55094370	C	T	intergenic	*ALAS2,PAGE2B*	.	.	.
X	55094623	A	G	intergenic	*ALAS2,PAGE2B*	.	.	.
X	55097137	G	A	intergenic	*ALAS2,PAGE2B*	.	.	.
4	74273288	A	G	intronic	*ALB*	.	.	.
3	105110311	G	A	intronic	*ALCAM*	.	.	.
19	49959857	G	C	intronic	*ALDH16A1*	.	.	.

10	97379140	G	A	intronic	*ALDH18A1*	.	.	.
15	58327750	A	G	intronic	*ALDH1A2*	.	.	.
15	58286909	T	C	intronic	*ALDH1A2*	.	.	.
15	58302743	T	C	intronic	*ALDH1A2*	.	.	.
15	58291360	G	A	intronic	*ALDH1A2*	.	.	.
15	58291361	T	C	intronic	*ALDH1A2*	.	.	.
15	58323603	C	A	intronic	*ALDH1A2*	.	.	.
3	125964569	G	A	intergenic	*ALDH1L1-AS2,KLF15*	.	.	.
12	112223357	A	G	intronic	*ALDH2*	.	.	.
12	112268221	A	G	intergenic	*ALDH2,MAPKAPK5-AS1*	.	.	.
12	112268398	G	A	intergenic	*ALDH2,MAPKAPK5-AS1*	.	.	.
11	67789715	G	A	intronic	*ALDH3B1*	.	.	.
14	74537223	A	C	intronic	*ALDH6A1*	.	.	.
5	125919779	C	T	intronic	*ALDH7A1*	.	.	.
5	125923191	C	T	intronic	*ALDH7A1*	.	.	.
6	135259825	G	A	intronic	*ALDH8A1*	.	.	.
12	34728757	C	T	intergenic	*ALG10,NONE*	.	.	.
12	34820629	C	A	intergenic	*ALG10,NONE*	.	.	.
12	34412361	G	C	intergenic	*ALG10,NONE*	.	.	.
12	34222043	C	T	intergenic	*ALG10,NONE*	.	.	.
12	34228531	C	T	intergenic	*ALG10,NONE*	.	.	.
12	34239214	T	C	intergenic	*ALG10,NONE*	.	.	.
12	34240762	G	A	intergenic	*ALG10,NONE*	.	.	.
12	34241277	G	T	intergenic	*ALG10,NONE*	.	.	.
12	34241837	G	A	intergenic	*ALG10,NONE*	.	.	.
12	34289990	C	T	intergenic	*ALG10,NONE*	.	.	.
12	34292179	G	A	intergenic	*ALG10,NONE*	.	.	.
12	34297399	A	T	intergenic	*ALG10,NONE*	.	.	.
12	34588810	C	A	intergenic	*ALG10,NONE*	.	.	.
12	34619863	C	T	intergenic	*ALG10,NONE*	.	.	.
12	34763095	C	G	intergenic	*ALG10,NONE*	.	.	.
12	34764300	C	T	intergenic	*ALG10,NONE*	.	.	.
12	34784360	T	G	intergenic	*ALG10,NONE*	.	.	.
12	34793530	C	T	intergenic	*ALG10,NONE*	.	.	.
12	34793531	A	T	intergenic	*ALG10,NONE*	.	.	.

12	34796483	G	A	intergenic	*ALG10,NONE*	.	.	.
12	34797606	G	A	intergenic	*ALG10,NONE*	.	.	.
12	34821250	A	C	intergenic	*ALG10,NONE*	.	.	.
12	34823184	C	T	intergenic	*ALG10,NONE*	.	.	.
12	34850935	T	G	intergenic	*ALG10,NONE*	.	.	.
12	38932693	T	TAAGAAGG	intergenic	*ALG10B,CPNE8*	.	.	.
12	38747053	A	G	intergenic	*ALG10B,CPNE8*	.	.	.
12	38747054	C	T	intergenic	*ALG10B,CPNE8*	.	.	.
12	38747865	G	A	intergenic	*ALG10B,CPNE8*	.	.	.
12	38801153	C	T	intergenic	*ALG10B,CPNE8*	.	.	.
12	38893523	C	T	intergenic	*ALG10B,CPNE8*	.	.	.
12	38904542	T	C	intergenic	*ALG10B,CPNE8*	.	.	.
12	38906428	T	C	intergenic	*ALG10B,CPNE8*	.	.	.
12	38908135	A	C	intergenic	*ALG10B,CPNE8*	.	.	.
12	38928330	A	T	intergenic	*ALG10B,CPNE8*	.	.	.
12	38928760	T	A	intergenic	*ALG10B,CPNE8*	.	.	.
12	38930263	C	A	intergenic	*ALG10B,CPNE8*	.	.	.
12	38930811	C	T	intergenic	*ALG10B,CPNE8*	.	.	.
12	38930812	A	G	intergenic	*ALG10B,CPNE8*	.	.	.
12	38970190	G	A	intergenic	*ALG10B,CPNE8*	.	.	.
2	30304393	A	T	intergenic	*ALK,YPEL5*	.	.	.
2	30280208	T	C	intergenic	*ALK,YPEL5*	.	.	.
2	30224821	T	C	intergenic	*ALK,YPEL5*	.	.	.
11	107452608	G	A	intergenic	*ALKBH8,ELMOD1*	.	.	.
2	3745211	GTCTATCTA	G	intronic	*ALLC*	.	.	.
2	3734572	C	T	intronic	*ALLC*	.	.	.
2	73692029	T	C	intronic	*ALMS1*	.	.	.
2	73692598	G	T	intronic	*ALMS1*	.	.	.
2	73693707	T	A	intronic	*ALMS1*	.	.	.
2	73733783	C	T	intronic	*ALMS1*	.	.	.
2	73736562	G	C	intronic	*ALMS1*	.	.	.
2	73769693	C	G	intronic	*ALMS1*	.	.	.
2	73770513	C	A	intronic	*ALMS1*	.	.	.
2	73862694	T	A	intergenic	*ALMS1,NAT8*	.	.	.
2	73872585	A	G	ncRNA_intronic	*ALMS1P1*	.	.	.
2	73894433	C	T	ncRNA_intronic	*ALMS1P1*	.	.	.
2	73894899	A	G	ncRNA_intronic	*ALMS1P1*	.	.	.

17	6897165	A	G	ncRNA_intronic	*ALOX12-AS1*	.	.	.
17	6821546	AACAC	A	intergenic	*ALOX12P2,ALOX12-AS1*	.	.	.
17	4548619	A	G	intergenic	*ALOX15,PELP1*	.	.	.
10	45889330	A	G	intronic	*ALOX5*	.	.	.
13	31325830	G	T	intronic	*ALOX5AP*	.	.	.
4	113416629	C	CCTCAACAG	intergenic	*ALPK1,NEUROG2*	.	.	.
4	113397390	A	G	intergenic	*ALPK1,NEUROG2*	.	.	.
4	113419934	T	G	intergenic	*ALPK1,NEUROG2*	.	.	.
4	113420144	T	C	intergenic	*ALPK1,NEUROG2*	.	.	.
18	56223273	C	T	intronic	*ALPK2*	.	.	.
18	56223274	A	G	intronic	*ALPK2*	.	.	.
2	202379514	T	C	intronic	*ALS2CR11*	.	.	.
2	202392730	A	T	intronic	*ALS2CR11*	.	.	.
2	202392732	T	A	intronic	*ALS2CR11*	.	.	.
2	202392937	A	C	intronic	*ALS2CR11*	.	.	.
2	202455875	G	A	intronic	*ALS2CR11*	.	.	.
2	202480440	G	T	intronic	*ALS2CR11*	.	.	.
2	202481927	A	AC	intronic	*ALS2CR11*	.	.	.
2	202159858	T	C	intronic	*ALS2CR12*	.	.	.
2	202167034	G	A	intronic	*ALS2CR12*	.	.	.
2	202169242	C	T	intronic	*ALS2CR12*	.	.	.
2	202169387	A	G	intronic	*ALS2CR12*	.	.	.
2	202237759	C	T	intergenic	*ALS2CR12,TRAK2*	.	.	.
12	85884432	T	C	intergenic	*ALX1,RASSF9*	.	.	.
12	85769289	C	T	intergenic	*ALX1,RASSF9*	.	.	.
12	85707647	G	C	intergenic	*ALX1,RASSF9*	.	.	.
12	85832796	A	C	intergenic	*ALX1,RASSF9*	.	.	.
12	86022558	T	A	intergenic	*ALX1,RASSF9*	.	.	.
12	86043716	C	T	intergenic	*ALX1,RASSF9*	.	.	.
12	86043717	A	G	intergenic	*ALX1,RASSF9*	.	.	.
12	86182402	A	G	intergenic	*ALX1,RASSF9*	.	.	.
1	110610279	T	G	intronic	*ALX3*	.	.	.
11	44347098	G	T	intergenic	*ALX4,CD82*	.	.	.
11	44413189	G	T	intergenic	*ALX4,CD82*	.	.	.
11	44415147	A	G	intergenic	*ALX4,CD82*	.	.	.
11	46595260	T	C	intronic	*AMBRA1*	.	.	.
6	111230437	G	A	intergenic	*AMD1,GTF3C6*	.	.	.

X	63414194	A	G	intronic	*AMER1*	.	.	.
2	131634022	T	G	intergenic	*AMER3,ARHGEF4*	.	.	.
12	53831248	T	G	intergenic	*AMHR2,PRR13*	.	.	.
X	109494286	G	T	intronic	*AMMECR1*	.	.	.
X	109649757	C	T	intronic	*AMMECR1*	.	.	.
X	109474923	A	G	intronic	*AMMECR1*	.	.	.
X	109475145	A	G	intronic	*AMMECR1*	.	.	.
X	109510252	C	T	intronic	*AMMECR1*	.	.	.
X	109588230	T	C	intronic	*AMMECR1*	.	.	.
2	128663058	A	G	intergenic	*AMMECR1L,SAP130*	.	.	.
2	128676325	A	G	intergenic	*AMMECR1L,SAP130*	.	.	.
X	112045441	A	G	intronic	*AMOT*	.	.	.
X	112159177	T	G	intergenic	*AMOT,LOC101928437*	.	.	.
X	112167990	C	A	intergenic	*AMOT,LOC101928437*	.	.	.
11	94682923	C	T	intergenic	*AMOTL1,CWC15*	.	.	.
11	10491897	G	C	intronic	*AMPD3*	.	.	.
7	38707042	A	G	intergenic	*AMPH,FAM183BP*	.	.	.
7	38707871	G	A	intergenic	*AMPH,FAM183BP*	.	.	.
1	104528898	A	G	intergenic	*AMY1A,LOC100129138*	.	.	.
1	104338770	T	C	intergenic	*AMY1A,LOC100129138*	.	.	.
1	104338771	A	C	intergenic	*AMY1A,LOC100129138*	.	.	.
1	104428297	G	A	intergenic	*AMY1A,LOC100129138*	.	.	.
1	104428351	G	A	intergenic	*AMY1A,LOC100129138*	.	.	.
1	104549125	A	G	intergenic	*AMY1A,LOC100129138*	.	.	.
1	104577953	C	T	intergenic	*AMY1A,LOC100129138*	.	.	.
10	73983512	G	C	intronic	*ANAPC16*	.	.	.
2	87108280	A	C	ncRNA_intronic	*ANAPC1P1*	.	.	.

2	87110022	G	A	ncRNA_intronic	*ANAPC1P1*	.	.	.
4	25533948	C	A	intergenic	*ANAPC4,SLC34A2*	.	.	.
4	25567567	A	AC	intergenic	*ANAPC4,SLC34A2*	.	.	.
4	25567568	T	A	intergenic	*ANAPC4,SLC34A2*	.	.	.
12	110830678	G	A	intronic	*ANAPC7*	.	.	.
8	108352619	T	A	intronic	*ANGPT1*	.	.	.
8	108486717	A	G	intronic	*ANGPT1*	.	.	.
8	108640530	C	A	intergenic	*ANGPT1,RSPO2*	.	.	.
8	108737717	T	A	intergenic	*ANGPT1,RSPO2*	.	.	.
8	108826822	G	A	intergenic	*ANGPT1,RSPO2*	.	.	.
8	108834098	T	C	intergenic	*ANGPT1,RSPO2*	.	.	.
8	108724873	C	T	intergenic	*ANGPT1,RSPO2*	.	.	.
8	108733722	G	A	intergenic	*ANGPT1,RSPO2*	.	.	.
8	108837347	C	T	intergenic	*ANGPT1,RSPO2*	.	.	.
8	108837348	A	G	intergenic	*ANGPT1,RSPO2*	.	.	.
20	921523	C	A	intergenic	*ANGPT4,RSPO4*	.	.	.
11	101767803	C	A	intronic	*ANGPTL5*	.	.	.
11	101768518	A	G	intronic	*ANGPTL5*	.	.	.
12	133811291	G	A	intronic	*ANHX*	.	.	.
12	133824929	A	G	intergenic	*ANHX,NONE*	.	.	.
8	41529350	T	C	intronic	*ANK1*	.	.	.
4	114036351	A	G	intronic	*ANK2*	.	.	.
4	114113979	T	A	intronic	*ANK2*	.	.	.
10	62209760	G	GAA	intronic	*ANK3*	.	.	.
10	62181192	A	T	intronic	*ANK3*	.	.	.
10	62076567	A	G	intronic	*ANK3*	.	.	.
10	62076569	G	C	intronic	*ANK3*	.	.	.
2	190579952	C	A	intronic	*ANKAR*	.	.	.
2	190580642	C	T	intronic	*ANKAR*	.	.	.
2	190582112	T	G	intronic	*ANKAR*	.	.	.
17	54518027	A	C	intronic	*ANKFN1*	.	.	.
17	54391983	A	T	intronic	*ANKFN1*	.	.	.
17	54478116	AT	A	intronic	*ANKFN1*	.	.	.
17	54241965	A	G	intronic	*ANKFN1*	.	.	.
17	54264362	G	T	intronic	*ANKFN1*	.	.	.
17	54286253	ATTT	A	intronic	*ANKFN1*	.	.	.
17	54417703	A	C	intronic	*ANKFN1*	.	.	.
17	54417705	A	T	intronic	*ANKFN1*	.	.	.

17	54460859	T	A	intronic	*ANKFN1*	.	.	.
17	54648700	G	A	intergenic	*ANKFN1,NOG*	.	.	.
17	54593618	AGTGTGT	A	intergenic	*ANKFN1,NOG*	.	.	.
5	14862146	A	T	intronic	*ANKH*	.	.	.
5	15005286	CCTTTCTTTCTTTCTTTCTTTCTTT	C	intergenic	*ANKH,LOC101929454*	.	.	.
5	139836020	A	G	intronic	*ANKHD1,ANKHD1-EIF4EBP3*	.	.	.
5	139897666	T	C	intronic	*ANKHD1,ANKHD1-EIF4EBP3*	.	.	.
5	139897667	G	A	intronic	*ANKHD1,ANKHD1-EIF4EBP3*	.	.	.
7	91938941	T	C	intronic	*ANKIB1*	.	.	.
7	92012110	T	C	intronic	*ANKIB1*	.	.	.
7	92013259	G	A	intronic	*ANKIB1*	.	.	.
7	92013802	G	C	intronic	*ANKIB1*	.	.	.
7	92013992	C	G	intronic	*ANKIB1*	.	.	.
7	92014746	A	G	intronic	*ANKIB1*	.	.	.
16	89351863	C	T	exonic	*ANKRD11*	nonsynonymous_SNV	.	.
16	89568045	C	T	intergenic	*ANKRD11,SPG7*	.	.	.
18	9162608	T	C	intronic	*ANKRD12*	.	.	.
18	9168734	C	T	intronic	*ANKRD12*	.	.	.
18	9268036	A	G	intronic	*ANKRD12*	.	.	.
18	9268407	G	A	intronic	*ANKRD12*	.	.	.
18	9269024	T	A	intronic	*ANKRD12*	.	.	.
18	9269765	G	A	intronic	*ANKRD12*	.	.	.
18	9314557	C	T	intergenic	*ANKRD12,TWSG1*	.	.	.
12	110451747	T	C	intronic	*ANKRD13A*	.	.	.
1	70766861	C	CATAT	intronic	*ANKRD13C*	.	.	.
1	70731141	A	C	intronic	*ANKRD13C*	.	.	.
4	74222235	G	A	intergenic	*ANKRD17,ALB*	.	.	.
4	74188767	C	A	intergenic	*ANKRD17,ALB*	.	.	.
4	74188769	G	C	intergenic	*ANKRD17,ALB*	.	.	.

3	197828233	A	G	intergenic	*ANKRD18DP,FAM157A*	.	.	.
3	197855452	TTTCTC	T	intergenic	*ANKRD18DP,FAM157A*	.	.	.
21	15447091	G	A	intergenic	*ANKRD20A11P,LIPI*	.	.	.
21	15363912	A	G	intergenic	*ANKRD20A11P,LIPI*	.	.	.
21	15363914	T	C	intergenic	*ANKRD20A11P,LIPI*	.	.	.
21	15364772	G	A	intergenic	*ANKRD20A11P,LIPI*	.	.	.
1	142703499	C	A	ncRNA_intronic	*ANKRD20A12P*	.	.	.
1	143124629	T	C	intergenic	*ANKRD20A12P,LOC102723769*	.	.	.
1	142965942	G	A	intergenic	*ANKRD20A12P,LOC102723769*	.	.	.
1	142800929	C	T	intergenic	*ANKRD20A12P,LOC102723769*	.	.	.
1	143121512	G	A	intergenic	*ANKRD20A12P,LOC102723769*	.	.	.
1	142899869	C	T	intergenic	*ANKRD20A12P,LOC102723769*	.	.	.
1	142960618	T	C	intergenic	*ANKRD20A12P,LOC102723769*	.	.	.
1	142961129	C	T	intergenic	*ANKRD20A12P,LOC102723769*	.	.	.
1	143129290	G	A	intergenic	*ANKRD20A12P,LOC102723769*	.	.	.
9	68380655	C	T	intergenic	*ANKRD20A3,MIR4477A*	.	.	.
9	68380657	T	G	intergenic	*ANKRD20A3,MIR4477A*	.	.	.
9	68381765	C	T	intergenic	*ANKRD20A3,MIR4477A*	.	.	.
9	68410887	C	T	intergenic	*ANKRD20A3,MIR4477A*	.	.	.

9	68387247	T	C	intergenic	*ANKRD20A3,MIR4477A*	.	.	.
9	68389981	C	CAT	intergenic	*ANKRD20A3,MIR4477A*	.	.	.
9	69444672	TAGCCTATGTG	T	intergenic	*ANKRD20A4,LOC100133920*	.	.	.
18	14224152	A	G	ncRNA_intronic	*ANKRD20A5P*	.	.	.
18	14250534	G	A	intergenic	*ANKRD20A5P,CYP4F35P*	.	.	.
18	14276224	C	A	intergenic	*ANKRD20A5P,CYP4F35P*	.	.	.
18	14269781	A	G	intergenic	*ANKRD20A5P,CYP4F35P*	.	.	.
18	14294293	G	A	intergenic	*ANKRD20A5P,CYP4F35P*	.	.	.
2	95507368	C	A	ncRNA_intronic	*ANKRD20A8P*	.	.	.
2	95428685	G	T	ncRNA_intronic	*ANKRD20A8P*	.	.	.
2	95462920	T	C	ncRNA_intronic	*ANKRD20A8P*	.	.	.
13	19440188	A	T	ncRNA_exonic	*ANKRD20A9P*	.	.	.
13	19430162	G	A	ncRNA_exonic	*ANKRD20A9P*	.	.	.
16	46570674	T	C	ncRNA_intronic	*ANKRD26P1*	.	.	.
16	46529254	T	C	ncRNA_intronic	*ANKRD26P1*	.	.	.
13	19872646	G	C	ncRNA_intronic	*ANKRD26P3*	.	.	.
13	19851884	C	A	ncRNA_intronic	*ANKRD26P3*	.	.	.
13	19853990	T	C	ncRNA_intronic	*ANKRD26P3*	.	.	.
13	19855998	T	A	ncRNA_intronic	*ANKRD26P3*	.	.	.
3	15741519	T	A	intronic	*ANKRD28*	.	.	.
10	37438247	T	G	intronic	*ANKRD30A*	.	.	.
18	14892397	C	T	intergenic	*ANKRD30B,LINC01443*	.	.	.
18	14896448	T	G	intergenic	*ANKRD30B,LINC01443*	.	.	.
18	14911158	A	C	intergenic	*ANKRD30B,LINC01443*	.	.	.
18	14911159	C	A	intergenic	*ANKRD30B,LINC01443*	.	.	.
2	132946808	T	A	ncRNA_intronic	*ANKRD30BL*	.	.	.
2	132958217	G	A	ncRNA_intronic	*ANKRD30BL*	.	.	.

2	132959542	T	A	ncRNA_intronic	*ANKRD30BL*	.	.	.
2	133016190	G	A	upstream	*ANKRD30BL*	.	.	.
2	133063184	ATT	A	intergenic	*ANKRD30BL,ZNF806*	.	.	.
2	133057694	T	A	intergenic	*ANKRD30BL,ZNF806*	.	.	.
2	133025055	C	A	intergenic	*ANKRD30BL,ZNF806*	.	.	.
2	133061448	G	A	intergenic	*ANKRD30BL,ZNF806*	.	.	.
21	14462111	A	C	ncRNA_intronic	*ANKRD30BP2*	.	.	.
21	14444962	T	A	ncRNA_intronic	*ANKRD30BP2*	.	.	.
21	14454656	C	G	ncRNA_intronic	*ANKRD30BP2*	.	.	.
21	14549811	C	A	intergenic	*ANKRD30BP2,MIR3156-3*	.	.	.
21	14505690	G	T	intergenic	*ANKRD30BP2,MIR3156-3*	.	.	.
21	14508500	G	A	intergenic	*ANKRD30BP2,MIR3156-3*	.	.	.
21	14509105	C	A	intergenic	*ANKRD30BP2,MIR3156-3*	.	.	.
21	14644351	G	T	intergenic	*ANKRD30BP2,MIR3156-3*	.	.	.
21	14763852	C	T	intergenic	*ANKRD30BP2,MIR3156-3*	.	.	.
10	45763657	C	T	intergenic	*ANKRD30BP3,OR13A1*	.	.	.
10	45766461	T	C	intergenic	*ANKRD30BP3,OR13A1*	.	.	.
10	45772248	A	G	intergenic	*ANKRD30BP3,OR13A1*	.	.	.
5	74399201	T	C	intronic	*ANKRD31*	.	.	.
5	74394596	C	A	intronic	*ANKRD31*	.	.	.
5	74395997	T	C	intronic	*ANKRD31*	.	.	.
5	74431525	C	T	intronic	*ANKRD31*	.	.	.
5	74468447	C	A	intronic	*ANKRD31*	.	.	.
5	74468604	C	T	intronic	*ANKRD31*	.	.	.
5	74468605	A	G	intronic	*ANKRD31*	.	.	.

5	74474339	T	C	intronic	*ANKRD31*	.	.	.
5	74474340	G	A	intronic	*ANKRD31*	.	.	.
5	74565185	C	T	intergenic	*ANKRD31,HMGCR*	.	.	.
5	74585446	C	G	intergenic	*ANKRD31,HMGCR*	.	.	.
5	74585971	A	T	intergenic	*ANKRD31,HMGCR*	.	.	.
2	97803931	A	C	intronic	*ANKRD36*	.	.	.
2	97829601	C	A	intronic	*ANKRD36*	.	.	.
2	97795088	T	A	intronic	*ANKRD36*	.	.	.
2	97795657	A	G	intronic	*ANKRD36*	.	.	.
2	97803615	G	T	intronic	*ANKRD36*	.	.	.
2	97834765	A	G	intronic	*ANKRD36*	.	.	.
2	97842738	G	A	intronic	*ANKRD36*	.	.	.
2	97891755	A	T	intronic	*ANKRD36*	.	.	.
2	97892459	A	T	intronic	*ANKRD36*	.	.	.
2	97893138	A	G	intronic	*ANKRD36*	.	.	.
2	97918396	A	G	intronic	*ANKRD36*	.	.	.
2	98182119	C	A	intronic	*ANKRD36B*	.	.	.
2	98182352	G	GTATA	intronic	*ANKRD36B*	.	.	.
2	98144430	T	C	intronic	*ANKRD36B*	.	.	.
2	98182032	T	G	intronic	*ANKRD36B*	.	.	.
2	98214023	C	T	intergenic	*ANKRD36B,COX5B*	.	.	.
2	98215572	A	C	intergenic	*ANKRD36B,COX5B*	.	.	.
2	98216554	C	T	intergenic	*ANKRD36B,COX5B*	.	.	.
1	168217374	A	G	upstream	*ANKRD36BP1*	.	.	.
2	89074489	A	G	ncRNA_intronic	*ANKRD36BP2*	.	.	.
2	96524070	T	A	intronic	*ANKRD36C*	.	.	.
2	96602225	G	A	intronic	*ANKRD36C*	.	.	.
2	96608325	T	C	intronic	*ANKRD36C*	.	.	.

2	96605635	CTTTTCATTCACTTATGCAAAAAAAAAGTATGCATTACACATCAAATAACTAATGAGTACTCAGA	C	intronic	*ANKRD36C*	.	.	.
2	96643867	T	C	intronic	*ANKRD36C*	.	.	.
2	96536932	T	C	intronic	*ANKRD36C*	.	.	.
2	96538418	A	G	intronic	*ANKRD36C*	.	.	.
2	96599366	C	T	intronic	*ANKRD36C*	.	.	.
2	96612062	C	T	intronic	*ANKRD36C*	.	.	.
2	96640906	T	G	intronic	*ANKRD36C*	.	.	.
11	82919565	T	G	intronic	*ANKRD42*	.	.	.
11	82912766	A	G	intronic	*ANKRD42*	.	.	.
2	198009598	T	C	intronic	*ANKRD44*	.	.	.
2	198034943	G	A	intronic	*ANKRD44*	.	.	.
2	197933322	T	C	intronic	*ANKRD44*	.	.	.
2	197933816	C	T	intronic	*ANKRD44*	.	.	.
2	197993346	C	G	intronic	*ANKRD44*	.	.	.
2	198008007	C	T	intronic	*ANKRD44*	.	.	.
1	173581866	A	G	intronic	*ANKRD45*	.	.	.
1	173590880	C	T	intronic	*ANKRD45*	.	.	.
1	173590881	A	G	intronic	*ANKRD45*	.	.	.
1	173620378	A	G	intronic	*ANKRD45*	.	.	.
4	126131997	A	G	intergenic	*ANKRD50,FAT4*	.	.	.
4	125893314	G	T	intergenic	*ANKRD50,FAT4*	.	.	.
4	126068992	A	G	intergenic	*ANKRD50,FAT4*	.	.	.
4	126071311	T	C	intergenic	*ANKRD50,FAT4*	.	.	.
4	126179779	A	G	intergenic	*ANKRD50,FAT4*	.	.	.
5	55519558	G	A	intronic	*ANKRD55*	.	.	.
5	55519651	T	C	intronic	*ANKRD55*	.	.	.
5	55520246	C	T	intronic	*ANKRD55*	.	.	.
5	55522261	C	T	intronic	*ANKRD55*	.	.	.
5	55527480	GCACACACACACACACACACACA	G	intronic	*ANKRD55*	.	.	.

5	55707345	C	A	intergenic	*ANKRD55,LOC102467147*	.	.	.
5	55588910	A	G	intergenic	*ANKRD55,LOC102467147*	.	.	.
5	55693130	A	C	intergenic	*ANKRD55,LOC102467147*	.	.	.
6	90269373	T	C	intronic	*ANKRD6*	.	.	.
18	12168861	A	G	intergenic	*ANKRD62,C18orf61*	.	.	.
18	12168862	C	T	intergenic	*ANKRD62,C18orf61*	.	.	.
18	12184327	C	T	intergenic	*ANKRD62,C18orf61*	.	.	.
18	12184658	T	G	intergenic	*ANKRD62,C18orf61*	.	.	.
22	17143939	C	T	ncRNA_intronic	*ANKRD62P1-PARP4P3*	.	.	.
22	17144335	G	A	ncRNA_intronic	*ANKRD62P1-PARP4P3*	.	.	.
22	17145172	T	C	ncRNA_intronic	*ANKRD62P1-PARP4P3*	.	.	.
22	17145623	C	G	ncRNA_intronic	*ANKRD62P1-PARP4P3*	.	.	.
22	17238342	G	GGCA	intergenic	*ANKRD62P1-PARP4P3,XKR3*	.	.	.
6	46730206	A	G	intergenic	*ANKRD66,MEP1A*	.	.	.
7	117880507	T	A	intronic	*ANKRD7*	.	.	.
7	117910627	T	C	intergenic	*ANKRD7,LVCAT5*	.	.	.
7	118344790	TC	T	intergenic	*ANKRD7,LVCAT5*	.	.	.
7	118396428	T	TA	intergenic	*ANKRD7,LVCAT5*	.	.	.
7	118677916	AG	A	intergenic	*ANKRD7,LVCAT5*	.	.	.
7	118816408	T	C	intergenic	*ANKRD7,LVCAT5*	.	.	.
7	118685057	GGTGT	G	intergenic	*ANKRD7,LVCAT5*	.	.	.
7	118319337	A	G	intergenic	*ANKRD7,LVCAT5*	.	.	.
7	117888911	C	G	intergenic	*ANKRD7,LVCAT5*	.	.	.
7	118421815	G	T	intergenic	*ANKRD7,LVCAT5*	.	.	.
7	118427684	T	A	intergenic	*ANKRD7,LVCAT5*	.	.	.
7	118110475	T	C	intergenic	*ANKRD7,LVCAT5*	.	.	.

7	117955048	G	T	intergenic	*ANKRD7,LVCAT5*	.	.	.
7	118033922	G	A	intergenic	*ANKRD7,LVCAT5*	.	.	.
7	118089351	A	T	intergenic	*ANKRD7,LVCAT5*	.	.	.
7	118605551	G	A	intergenic	*ANKRD7,LVCAT5*	.	.	.
7	118941077	T	G	intergenic	*ANKRD7,LVCAT5*	.	.	.
7	118948932	TAAAG	T	intergenic	*ANKRD7,LVCAT5*	.	.	.
7	118980743	A	T	intergenic	*ANKRD7,LVCAT5*	.	.	.
7	119104756	G	A	intergenic	*ANKRD7,LVCAT5*	.	.	.
7	119157904	T	C	intergenic	*ANKRD7,LVCAT5*	.	.	.
7	119233180	C	A	intergenic	*ANKRD7,LVCAT5*	.	.	.
6	34946862	C	T	intronic	*ANKS1A*	.	.	.
12	100340892	T	C	intronic	*ANKS1B*	.	.	.
12	99915827	T	C	intronic	*ANKS1B*	.	.	.
12	100076931	G	C	intronic	*ANKS1B*	.	.	.
12	100267800	CATAT	C	intronic	*ANKS1B*	.	.	.
12	99162967	TTTTTTG	T	intronic	*ANKS1B*	.	.	.
12	99765044	G	A	intronic	*ANKS1B*	.	.	.
12	99898390	A	T	exonic	*ANKS1B*	synonymous_SNV	.	.
12	100046693	A	T	intronic	*ANKS1B*	.	.	.
12	99609445	C	G	intronic	*ANKS1B*	.	.	.
12	99917349	TG	T	intronic	*ANKS1B*	.	.	.
12	99584849	G	T	intronic	*ANKS1B*	.	.	.
12	99635801	A	C	intronic	*ANKS1B*	.	.	.
12	99685587	T	G	intronic	*ANKS1B*	.	.	.
12	99686765	A	C	intronic	*ANKS1B*	.	.	.
12	99708643	T	C	intronic	*ANKS1B*	.	.	.
12	99710664	G	A	intronic	*ANKS1B*	.	.	.
12	99712186	T	C	intronic	*ANKS1B*	.	.	.
12	99854460	C	T	intronic	*ANKS1B*	.	.	.
12	99859700	C	T	intronic	*ANKS1B*	.	.	.
12	99886405	T	G	intronic	*ANKS1B*	.	.	.
12	100027393	T	C	intronic	*ANKS1B*	.	.	.
12	100027535	T	C	intronic	*ANKS1B*	.	.	.
12	100122391	C	T	intronic	*ANKS1B*	.	.	.
12	100149036	A	G	intronic	*ANKS1B*	.	.	.
12	100262557	C	T	intronic	*ANKS1B*	.	.	.
12	100386713	G	A	intergenic	*ANKS1B,UHRF1BP1L*	.	.	.

12	100388740	G	A	intergenic	*ANKS1B,UHRF1BP1L*	.	.	.
7	36469122	G	T	intronic	*ANLN*	.	.	.
11	69977465	G	A	intronic	*ANO1*	.	.	.
11	70018788	C	T	intronic	*ANO1*	.	.	.
3	43472074	A	T	intronic	*ANO10*	.	.	.
3	43635973	A	G	intronic	*ANO10*	.	.	.
3	43636782	G	A	intronic	*ANO10*	.	.	.
3	43725932	A	G	intergenic	*ANO10,ABHD5*	.	.	.
12	5740815	G	A	intronic	*ANO2*	.	.	.
12	5675803	G	C	intronic	*ANO2*	.	.	.
12	5946867	G	A	intronic	*ANO2*	.	.	.
11	26472867	C	T	intronic	*ANO3*	.	.	.
11	26685112	T	C	downstream	*ANO3*	.	.	.
11	26234050	T	C	intronic	*ANO3*	.	.	.
11	26235130	T	C	intronic	*ANO3*	.	.	.
11	26235131	G	C	intronic	*ANO3*	.	.	.
11	26254237	C	T	intronic	*ANO3*	.	.	.
11	26334377	G	A	intronic	*ANO3*	.	.	.
11	26339247	A	C	intronic	*ANO3*	.	.	.
11	26339248	G	A	intronic	*ANO3*	.	.	.
11	26417291	A	G	intronic	*ANO3*	.	.	.
11	26464545	T	TCA	intronic	*ANO3*	.	.	.
11	26626578	A	G	intronic	*ANO3*	.	.	.
11	26626580	G	A	intronic	*ANO3*	.	.	.
11	26627312	C	T	intronic	*ANO3*	.	.	.
12	101300767	A	G	intronic	*ANO4*	.	.	.
12	101317869	T	C	intronic	*ANO4*	.	.	.
12	101317871	A	G	intronic	*ANO4*	.	.	.
12	101423896	G	C	intronic	*ANO4*	.	.	.
12	101423898	T	C	intronic	*ANO4*	.	.	.
12	101427300	C	T	intronic	*ANO4*	.	.	.
12	101427301	C	A	intronic	*ANO4*	.	.	.
12	101427709	T	C	intronic	*ANO4*	.	.	.
12	101451616	C	T	intronic	*ANO4*	.	.	.
12	101451617	A	T	intronic	*ANO4*	.	.	.
12	101451751	C	T	intronic	*ANO4*	.	.	.
12	101452830	C	T	intronic	*ANO4*	.	.	.

12	101466410	G	C	intronic	*ANO4*	.	.	.
12	101479858	CTGTGTGT GTGTGTGT GTGTG	C	intronic	*ANO4*	.	.	.
11	22313488	G	A	intergenic	*ANO5,SLC17A6*	.	.	.
12	45637457	C	T	intronic	*ANO6*	.	.	.
12	45637458	A	G	intronic	*ANO6*	.	.	.
12	45714241	A	G	intronic	*ANO6*	.	.	.
12	45768371	G	A	intronic	*ANO6*	.	.	.
12	45788082	A	T	intronic	*ANO6*	.	.	.
12	45790526	A	C	intronic	*ANO6*	.	.	.
2	242133996	T	G	intronic	*ANO7*	.	.	.
X	8553017	C	A	intronic	*ANOS1*	.	.	.
X	8595334	G	A	intronic	*ANOS1*	.	.	.
X	8577923	C	T	intronic	*ANOS1*	.	.	.
X	8524664	C	A	intronic	*ANOS1*	.	.	.
X	8743418	C	G	intergenic	*ANOS1,FAM9A*	.	.	.
X	8746105	A	G	intergenic	*ANOS1,FAM9A*	.	.	.
15	35548108	A	G	intergenic	*ANP32AP1,DPH6*	.	.	.
15	35566722	A	T	intergenic	*ANP32AP1,DPH6*	.	.	.
15	35646358	C	T	intergenic	*ANP32AP1,DPH6*	.	.	.
15	35647204	T	C	intergenic	*ANP32AP1,DPH6*	.	.	.
12	48872564	T	G	intergenic	*ANP32D,C12orf54*	.	.	.
12	48872565	G	C	intergenic	*ANP32D,C12orf54*	.	.	.
2	69283436	C	G	intronic	*ANTXR1*	.	.	.
2	69509207	G	T	intergenic	*ANTXR1,GFPT1*	.	.	.
4	80937062	AAGGAAAG GAAAGGAA AAAGG	A	intronic	*ANTXR2*	.	.	.
4	80954155	C	CA	intronic	*ANTXR2*	.	.	.
10	47589913	A	G	downstream	*ANTXRLP1*	.	.	.
9	76302731	G	T	intergenic	*ANXA1,MIR6130*	.	.	.
9	76202297	T	C	intergenic	*ANXA1,MIR6130*	.	.	.
9	76278626	A	T	intergenic	*ANXA1,MIR6130*	.	.	.
4	169051467	A	T	intronic	*ANXA10*	.	.	.
4	169136035	T	C	intergenic	*ANXA10,DDX60*	.	.	.
9	33649562	C	T	intergenic	*ANXA2P2,PTENP1*	.	.	.

10	66613838	G	T	intergenic	*ANXA2P3,LINC01515*	.	.	.
10	66755347	G	C	intergenic	*ANXA2P3,LINC01515*	.	.	.
10	66667262	A	T	intergenic	*ANXA2P3,LINC01515*	.	.	.
10	67166195	G	A	intergenic	*ANXA2P3,LINC01515*	.	.	.
10	67061291	GTATA	G	intergenic	*ANXA2P3,LINC01515*	.	.	.
10	66596273	A	T	intergenic	*ANXA2P3,LINC01515*	.	.	.
10	66597539	T	C	intergenic	*ANXA2P3,LINC01515*	.	.	.
10	66686456	C	T	intergenic	*ANXA2P3,LINC01515*	.	.	.
10	66686696	T	A	intergenic	*ANXA2P3,LINC01515*	.	.	.
10	66863374	A	T	intergenic	*ANXA2P3,LINC01515*	.	.	.
10	66864899	C	A	intergenic	*ANXA2P3,LINC01515*	.	.	.
10	66940293	C	T	intergenic	*ANXA2P3,LINC01515*	.	.	.
10	67026351	G	A	intergenic	*ANXA2P3,LINC01515*	.	.	.
10	67286341	A	G	intergenic	*ANXA2P3,LINC01515*	.	.	.
4	122651860	A	G	intergenic	*ANXA5,TMEM155*	.	.	.
4	122675158	A	G	intergenic	*ANXA5,TMEM155*	.	.	.
10	75147631	C	T	intronic	*ANXA7*	.	.	.
10	75151743	T	C	intronic	*ANXA7*	.	.	.
10	48309421	T	G	intergenic	*ANXA8,ZNF488*	.	.	.
10	48346998	A	T	intergenic	*ANXA8,ZNF488*	.	.	.
10	47995729	A	T	intergenic	*ANXA8L1,CTSLP2*	.	.	.
7	36599067	T	C	intronic	*AOAH*	.	.	.
7	36744862	A	T	intronic	*AOAH*	.	.	.
7	36745135	G	A	intronic	*AOAH*	.	.	.

7	36754427	G	A	intronic	*AOAH*	.	.	.
7	36792277	A	C	intergenic	*AOAH,ELMO1*	.	.	.
7	36875416	C	T	intergenic	*AOAH,ELMO1*	.	.	.
7	36885975	G	A	intergenic	*AOAH,ELMO1*	.	.	.
7	36886343	T	G	intergenic	*AOAH,ELMO1*	.	.	.
7	150640890	C	T	intergenic	*AOC1,KCNH2*	.	.	.
2	201470820	T	C	intronic	*AOX1*	.	.	.
2	201471577	T	G	intronic	*AOX1*	.	.	.
22	29825403	GAAA	G	intergenic	*AP1B1,RFPL1S*	.	.	.
16	71865710	C	T	intergenic	*AP1G1,ATXN1L*	.	.	.
19	16315089	GC	G	intronic	*AP1M1*	.	.	.
19	16309839	G	A	intronic	*AP1M1*	.	.	.
19	16315907	A	G	intronic	*AP1M1*	.	.	.
X	15852449	TTAAAGAAAAAAAAAGTCTACAGCTTTACCCCATTATCACCACATAACCAATGAGAAAGACTCTTCTCATCCGAGACCAATGTTTTTGATTCCACTCCCATTCTCCCTTCCAAAA	T	intronic	*AP1S2*	.	.	.
X	15923249	T	C	intergenic	*AP1S2,GRPR*	.	.	.
X	16061699	T	A	intergenic	*AP1S2,GRPR*	.	.	.
2	224631010	C	A	intronic	*AP1S3*	.	.	.
5	77343334	A	G	intronic	*AP3B1*	.	.	.
5	77417390	T	C	intronic	*AP3B1*	.	.	.
5	77467549	G	T	intronic	*AP3B1*	.	.	.
5	77483544	C	T	intronic	*AP3B1*	.	.	.
5	115216752	A	G	intronic	*AP3S1*	.	.	.
5	115292865	A	AAG	intergenic	*AP3S1,LVRN*	.	.	.
5	115278099	A	T	intergenic	*AP3S1,LVRN*	.	.	.

5	115278461	T	A	intergenic	*AP3S1,LVRN*	.	.	.
1	114435872	G	GTTA	ncRNA_intronic	*AP4B1-AS1*	.	.	.
15	51247411	A	G	intronic	*AP4E1*	.	.	.
15	51277190	C	T	intronic	*AP4E1*	.	.	.
15	51277191	A	G	intronic	*AP4E1*	.	.	.
15	51278046	C	A	intronic	*AP4E1*	.	.	.
14	57817646	A	G	intergenic	*AP5M1,NAA30*	.	.	.
20	3821564	G	A	intergenic	*AP5S1,MAVS*	.	.	.
12	99110888	C	T	intronic	*APAF1*	.	.	.
9	72050732	A	T	intronic	*APBA1*	.	.	.
9	72050999	C	T	intronic	*APBA1*	.	.	.
9	72294346	AACACAC	A	intergenic	*APBA1,PTAR1*	.	.	.
15	29344402	T	C	intronic	*APBA2*	.	.	.
15	29232398	G	A	intronic	*APBA2*	.	.	.
4	40933830	A	T	intronic	*APBB2*	.	.	.
4	40855498	C	T	intronic	*APBB2*	.	.	.
4	41107940	C	A	intronic	*APBB2*	.	.	.
4	41116939	T	C	intronic	*APBB2*	.	.	.
20	57035802	T	C	UTR3	*APCDD1L*	.	.	.
20	57108306	C	T	ncRNA_intronic	*APCDD1L-AS1*	.	.	.
20	57178184	C	A	ncRNA_intronic	*APCDD1L-AS1*	.	.	.
1	159588662	G	T	intergenic	*APCS,CRP*	.	.	.
1	159590385	T	TGTTTGCA	intergenic	*APCS,CRP*	.	.	.
1	159577850	A	G	intergenic	*APCS,CRP*	.	.	.
1	159603479	T	A	intergenic	*APCS,CRP*	.	.	.
2	68747606	A	C	intronic	*APLF*	.	.	.
2	68747607	C	T	intronic	*APLF*	.	.	.
2	68826973	T	C	intergenic	*APLF,PROKR1*	.	.	.
2	68827519	T	C	intergenic	*APLF,PROKR1*	.	.	.
20	24983213	T	C	intergenic	*APMAP,ACSS1*	.	.	.
16	59797135	T	C	intergenic	*APOOP5,LOC101927580*	.	.	.
16	59797726	G	A	intergenic	*APOOP5,LOC101927580*	.	.	.
21	27452447	C	T	intronic	*APP*	.	.	.
21	27832548	T	C	intergenic	*APP,CYYR1*	.	.	.
12	105573124	T	A	intronic	*APPL2*	.	.	.
12	105573418	T	C	intronic	*APPL2*	.	.	.

12	105574770	T	C	intronic	*APPL2*	.	.	.
12	105574772	T	C	intronic	*APPL2*	.	.	.
12	105576527	C	A	intronic	*APPL2*	.	.	.
12	105576528	A	G	intronic	*APPL2*	.	.	.
12	105642793	C	A	intergenic	*APPL2,KCCAT198*	.	.	.
12	105642795	G	T	intergenic	*APPL2,KCCAT198*	.	.	.
12	105638764	G	T	intergenic	*APPL2,KCCAT198*	.	.	.
12	105638766	C	T	intergenic	*APPL2,KCCAT198*	.	.	.
12	105645087	G	GC	intergenic	*APPL2,KCCAT198*	.	.	.
12	105668733	T	C	intergenic	*APPL2,KCCAT198*	.	.	.
18	24476472	C	T	ncRNA_intronic	*AQP4-AS1*	.	.	.
18	24476473	A	G	ncRNA_intronic	*AQP4-AS1*	.	.	.
9	33423977	G	A	intergenic	*AQP7,AQP3*	.	.	.
9	67355770	AAAC	A	intergenic	*AQP7P1,LOC102723709*	.	.	.
9	67316698	T	C	intergenic	*AQP7P1,LOC102723709*	.	.	.
9	67316699	G	T	intergenic	*AQP7P1,LOC102723709*	.	.	.
15	58435107	G	T	intronic	*AQP9*	.	.	.
15	58545105	A	G	intergenic	*AQP9,LIPC*	.	.	.
15	58490067	T	C	intergenic	*AQP9,LIPC*	.	.	.
15	58656760	C	G	intergenic	*AQP9,LIPC*	.	.	.
15	35198487	G	A	intronic	*AQR*	.	.	.
X	66843775	G	T	intronic	*AR*	.	.	.
X	66900759	G	T	intronic	*AR*	.	.	.
X	66848024	C	T	intronic	*AR*	.	.	.
X	66848025	A	G	intronic	*AR*	.	.	.
X	66859789	G	C	intronic	*AR*	.	.	.
X	66880771	A	C	intronic	*AR*	.	.	.
X	66881329	G	T	intronic	*AR*	.	.	.
X	66924474	T	C	intronic	*AR*	.	.	.
X	66925141	C	G	intronic	*AR*	.	.	.
X	66925395	A	G	intronic	*AR*	.	.	.
X	66926283	A	G	intronic	*AR*	.	.	.
X	67006734	G	T	intergenic	*AR,OPHN1*	.	.	.
X	67041180	C	T	intergenic	*AR,OPHN1*	.	.	.
X	67041295	A	G	intergenic	*AR,OPHN1*	.	.	.

X	67051943	C	T	intergenic	*AR,OPHN1*	.	.	.
X	67052158	T	C	intergenic	*AR,OPHN1*	.	.	.
X	67080343	T	G	intergenic	*AR,OPHN1*	.	.	.
X	67104187	T	C	intergenic	*AR,OPHN1*	.	.	.
X	67104887	T	A	intergenic	*AR,OPHN1*	.	.	.
X	67105302	G	A	intergenic	*AR,OPHN1*	.	.	.
X	67107578	A	G	intergenic	*AR,OPHN1*	.	.	.
X	67165708	T	C	intergenic	*AR,OPHN1*	.	.	.
X	67167682	G	A	intergenic	*AR,OPHN1*	.	.	.
X	67184758	A	G	intergenic	*AR,OPHN1*	.	.	.
X	67225084	G	A	intergenic	*AR,OPHN1*	.	.	.
X	67229505	A	G	intergenic	*AR,OPHN1*	.	.	.
X	67230098	C	T	intergenic	*AR,OPHN1*	.	.	.
X	67240157	G	A	intergenic	*AR,OPHN1*	.	.	.
X	67240190	T	C	intergenic	*AR,OPHN1*	.	.	.
X	67240191	G	A	intergenic	*AR,OPHN1*	.	.	.
X	47425032	G	A	UTR3	*ARAF*	.	.	.
4	36086361	A	G	intronic	*ARAP2*	.	.	.
4	36073368	T	A	intronic	*ARAP2*	.	.	.
4	36157829	A	G	intronic	*ARAP2*	.	.	.
4	75372964	G	T	intergenic	*AREG,AREG*	.	.	.
4	75560259	C	A	intergenic	*AREG,BTC*	.	.	.
3	57597342	A	C	intergenic	*ARF4-AS1,DENND6A*	.	.	.
3	57597735	G	T	intergenic	*ARF4-AS1,DENND6A*	.	.	.
3	57600222	C	T	intergenic	*ARF4-AS1,DENND6A*	.	.	.
11	47192114	C	G	intronic	*ARFGAP2*	.	.	.
11	47188006	C	T	intronic	*ARFGAP2*	.	.	.
8	68249389	G	A	intronic	*ARFGEF1*	.	.	.
8	68249391	C	A	intronic	*ARFGEF1*	.	.	.
8	68265573	A	G	intergenic	*ARFGEF1,CPA6*	.	.	.
8	68266229	T	A	intergenic	*ARFGEF1,CPA6*	.	.	.
8	68268941	A	C	intergenic	*ARFGEF1,CPA6*	.	.	.
20	47551568	G	T	intronic	*ARFGEF2*	.	.	.
20	47596893	G	A	intronic	*ARFGEF2*	.	.	.
20	47597392	A	T	intronic	*ARFGEF2*	.	.	.

6	138685609	C	T	intergenic	*ARFGEF3,HEBP2*	.	.	.
6	131901142	G	A	intronic	*ARG1,MED23*	.	.	.
3	121291714	C	G	intronic	*ARGFX*	.	.	.
13	107237818	G	C	intergenic	*ARGLU1,LINC0055 1*	.	.	.
4	148910349	A	G	intronic	*ARHGAP10*	.	.	.
4	148910351	A	G	intronic	*ARHGAP10*	.	.	.
4	148683118	C	G	intronic	*ARHGAP10*	.	.	.
4	148758814	A	T	intronic	*ARHGAP10*	.	.	.
15	32920708	G	A	intronic	*ARHGAP11A*	.	.	.
2	144085389	A	G	intronic	*ARHGAP15*	.	.	.
2	144304581	T	A	intronic	*ARHGAP15*	.	.	.
2	144005958	C	A	intronic	*ARHGAP15*	.	.	.
2	144060976	C	T	intronic	*ARHGAP15*	.	.	.
2	144341942	A	C	intronic	*ARHGAP15*	.	.	.
2	144341943	T	C	intronic	*ARHGAP15*	.	.	.
2	144529548	C	T	intergenic	*ARHGAP15,LOC10 1928386*	.	.	.
6	130128890	C	T	intergenic	*ARHGAP18,TMEM2 44*	.	.	.
11	110466599	G	A	intronic	*ARHGAP20*	.	.	.
11	110571959	A	C	intronic	*ARHGAP20*	.	.	.
11	110573548	G	A	intronic	*ARHGAP20*	.	.	.
11	110882672	A	G	intergenic	*ARHGAP20,C11orf 53*	.	.	.
11	110593381	G	GTA	intergenic	*ARHGAP20,C11orf 53*	.	.	.
11	110922030	T	A	intergenic	*ARHGAP20,C11orf 53*	.	.	.
10	49691840	G	T	intronic	*ARHGAP22*	.	.	.
17	36677091	T	C	intergenic	*ARHGAP23,SRCIN 1*	.	.	.
17	36677092	G	T	intergenic	*ARHGAP23,SRCIN 1*	.	.	.
4	86654947	G	T	intronic	*ARHGAP24*	.	.	.
4	86654948	G	T	intronic	*ARHGAP24*	.	.	.
4	86425301	G	A	intronic	*ARHGAP24*	.	.	.
4	86505001	C	T	intronic	*ARHGAP24*	.	.	.

4	86546025	C	T	intronic	*ARHGAP24*	.	.	.
4	86546530	C	T	intronic	*ARHGAP24*	.	.	.
4	86546847	C	G	intronic	*ARHGAP24*	.	.	.
4	86583426	G	A	intronic	*ARHGAP24*	.	.	.
4	86642236	A	G	intronic	*ARHGAP24*	.	.	.
5	142443684	G	A	intronic	*ARHGAP26*	.	.	.
5	142195446	A	T	intronic	*ARHGAP26*	.	.	.
5	142308207	C	G	intronic	*ARHGAP26*	.	.	.
5	142563403	C	T	intronic	*ARHGAP26*	.	.	.
5	142230558	G	A	intronic	*ARHGAP26*	.	.	.
5	142366283	T	C	intronic	*ARHGAP26*	.	.	.
5	142366579	G	A	intronic	*ARHGAP26*	.	.	.
5	142366580	G	A	intronic	*ARHGAP26*	.	.	.
5	142374647	C	T	intronic	*ARHGAP26*	.	.	.
18	6873937	C	T	intronic	*ARHGAP28*	.	.	.
1	94666727	A	C	intronic	*ARHGAP29*	.	.	.
1	94754785	C	G	intergenic	*ARHGAP29,ABCD3*	.	.	.
1	94756306	A	T	intergenic	*ARHGAP29,ABCD3*	.	.	.
11	128999280	G	A	intronic	*ARHGAP32*	.	.	.
11	129031705	T	G	intronic	*ARHGAP32*	.	.	.
11	129031707	T	A	intronic	*ARHGAP32*	.	.	.
11	128853503	T	C	intronic	*ARHGAP32*	.	.	.
11	129031143	A	G	intronic	*ARHGAP32*	.	.	.
11	129162884	C	T	intergenic	*ARHGAP32,BARX2*	.	.	.
11	129077439	A	G	intergenic	*ARHGAP32,BARX2*	.	.	.
11	129114477	A	C	intergenic	*ARHGAP32,BARX2*	.	.	.
X	130381753	T	C	intergenic	*ARHGAP36,IGSF1*	.	.	.
8	145783159	G	C	intronic	*ARHGAP39*	.	.	.
11	100754198	C	T	intronic	*ARHGAP42*	.	.	.
11	100754199	A	G	intronic	*ARHGAP42*	.	.	.
11	100767762	A	G	intronic	*ARHGAP42*	.	.	.
11	100769947	G	A	intronic	*ARHGAP42*	.	.	.
11	100775197	C	T	intronic	*ARHGAP42*	.	.	.

14	32601546	A	G	intronic	*ARHGAP5*	.	.	.
14	32601547	G	A	intronic	*ARHGAP5*	.	.	.
X	11468209	C	G	intronic	*ARHGAP6*	.	.	.
X	11523966	G	T	intronic	*ARHGAP6*	.	.	.
X	11584314	G	A	intronic	*ARHGAP6*	.	.	.
X	11235735	T	C	intronic	*ARHGAP6*	.	.	.
X	11236242	A	G	intronic	*ARHGAP6*	.	.	.
X	11236895	A	G	intronic	*ARHGAP6*	.	.	.
X	11236897	C	T	intronic	*ARHGAP6*	.	.	.
X	11377726	C	A	intronic	*ARHGAP6*	.	.	.
X	11377847	C	T	intronic	*ARHGAP6*	.	.	.
X	11408367	T	C	intronic	*ARHGAP6*	.	.	.
X	11592037	A	G	intronic	*ARHGAP6*	.	.	.
X	11613982	A	C	intronic	*ARHGAP6*	.	.	.
X	11761029	A	T	intergenic	*ARHGAP6,MSL3*	.	.	.
X	11746383	G	A	intergenic	*ARHGAP6,MSL3*	.	.	.
1	157036142	T	C	intergenic	*ARHGEF11,ETV3L*	.	.	.
11	120330562	C	T	intronic	*ARHGEF12*	.	.	.
1	155976047	A	C	intergenic	*ARHGEF2,SSR2*	.	.	.
5	73099991	T	C	intronic	*ARHGEF28*	.	.	.
5	73206238	T	A	intronic	*ARHGEF28*	.	.	.
5	73596978	T	C	intergenic	*ARHGEF28,LINC01335*	.	.	.
3	56839623	G	T	intronic	*ARHGEF3*	.	.	.
3	56927662	C	T	intronic	*ARHGEF3*	.	.	.
2	39145948	G	A	upstream	*ARHGEF33*	.	.	.
2	131710244	T	C	intronic	*ARHGEF4*	.	.	.
7	144061192	A	T	exonic	*ARHGEF5*	nonsynonymous_SNV	.	.
13	111817052	T	C	intronic	*ARHGEF7*	.	.	.
13	111786474	C	A	intronic	*ARHGEF7*	.	.	.
13	111787392	G	A	intronic	*ARHGEF7*	.	.	.
X	62865885	C	T	intronic	*ARHGEF9*	.	.	.
X	62865944	G	T	intronic	*ARHGEF9*	.	.	.
X	62866916	A	G	intronic	*ARHGEF9*	.	.	.
X	62868623	G	A	intronic	*ARHGEF9*	.	.	.
X	62911637	G	A	intronic	*ARHGEF9*	.	.	.
9	34622411	C	T	exonic	*ARID3C*	synonymous_SNV	.	.

10	63737630	G	C	intronic	*ARID5B*	.	.	.
X	100230378	T	C	intronic	*ARL13A*	.	.	.
X	100242558	C	T	intronic	*ARL13A*	.	.	.
11	30402104	C	G	intergenic	*ARL14EP,MPPED2*	.	.	.
5	115396193	T	C	intergenic	*ARL14EPL,COMMD 10*	.	.	.
5	53436285	GATAGAT	G	intronic	*ARL15*	.	.	.
5	53238951	G	A	intronic	*ARL15*	.	.	.
5	53278342	G	A	intronic	*ARL15*	.	.	.
5	53278344	T	C	intronic	*ARL15*	.	.	.
5	53300962	A	G	intronic	*ARL15*	.	.	.
10	104444201	C	T	intronic	*ARL3*	.	.	.
7	13128865	T	C	intergenic	*ARL4A,ETV1*	.	.	.
7	13436541	T	TGAAC	intergenic	*ARL4A,ETV1*	.	.	.
7	13496173	G	T	intergenic	*ARL4A,ETV1*	.	.	.
7	12790462	A	C	intergenic	*ARL4A,ETV1*	.	.	.
7	12790464	T	G	intergenic	*ARL4A,ETV1*	.	.	.
7	12975414	T	C	intergenic	*ARL4A,ETV1*	.	.	.
7	12977407	G	A	intergenic	*ARL4A,ETV1*	.	.	.
7	13127439	C	T	intergenic	*ARL4A,ETV1*	.	.	.
7	13161402	A	G	intergenic	*ARL4A,ETV1*	.	.	.
7	13161628	A	G	intergenic	*ARL4A,ETV1*	.	.	.
7	13162712	GAT	G	intergenic	*ARL4A,ETV1*	.	.	.
7	13163260	A	G	intergenic	*ARL4A,ETV1*	.	.	.
7	13311498	T	A	intergenic	*ARL4A,ETV1*	.	.	.
7	13466871	C	G	intergenic	*ARL4A,ETV1*	.	.	.
7	13671074	T	C	intergenic	*ARL4A,ETV1*	.	.	.
7	13709849	C	A	intergenic	*ARL4A,ETV1*	.	.	.
7	13712207	G	A	intergenic	*ARL4A,ETV1*	.	.	.
7	13848652	G	A	intergenic	*ARL4A,ETV1*	.	.	.
7	13848653	T	C	intergenic	*ARL4A,ETV1*	.	.	.
2	235404569	C	T	UTR3	*ARL4C*	.	.	.
10	19225069	AGAATTCG GCTGT	A	intergenic	*ARL5B,MALRD1*	.	.	.
10	19108196	G	A	intergenic	*ARL5B,MALRD1*	.	.	.
10	19172483	G	A	intergenic	*ARL5B,MALRD1*	.	.	.
10	19226146	T	G	intergenic	*ARL5B,MALRD1*	.	.	.

10	19262938	A	G	intergenic	*ARL5B,MALRD1*	.	.	.
2	153791820	C	A	intergenic	*ARL6IP6,RPRM*	.	.	.
2	153941012	C	A	intergenic	*ARL6IP6,RPRM*	.	.	.
2	154180162	C	T	intergenic	*ARL6IP6,RPRM*	.	.	.
2	153795316	T	C	intergenic	*ARL6IP6,RPRM*	.	.	.
2	153875268	G	T	intergenic	*ARL6IP6,RPRM*	.	.	.
2	153979294	TC	T	intergenic	*ARL6IP6,RPRM*	.	.	.
2	154068948	T	C	intergenic	*ARL6IP6,RPRM*	.	.	.
2	153822316	C	A	intergenic	*ARL6IP6,RPRM*	.	.	.
2	153652317	G	A	intergenic	*ARL6IP6,RPRM*	.	.	.
2	153696235	G	A	intergenic	*ARL6IP6,RPRM*	.	.	.
2	154041220	A	T	intergenic	*ARL6IP6,RPRM*	.	.	.
2	154103987	G	A	intergenic	*ARL6IP6,RPRM*	.	.	.
2	154106237	T	C	intergenic	*ARL6IP6,RPRM*	.	.	.
2	154257276	G	A	intergenic	*ARL6IP6,RPRM*	.	.	.
2	154291030	A	G	intergenic	*ARL6IP6,RPRM*	.	.	.
2	154292614	G	C	intergenic	*ARL6IP6,RPRM*	.	.	.
2	154292909	G	A	intergenic	*ARL6IP6,RPRM*	.	.	.
6	35729612	A	G	intergenic	*ARMC12,CLPSL2*	.	.	.
6	109243238	C	T	ncRNA_intronic	*ARMC2-AS1*	.	.	.
10	28273379	A	C	intronic	*ARMC4*	.	.	.
10	28192313	C	T	intronic	*ARMC4*	.	.	.
10	28192314	A	G	intronic	*ARMC4*	.	.	.
10	28267993	G	A	intronic	*ARMC4*	.	.	.
3	137923520	T	C	intronic	*ARMC8*	.	.	.
X	100836982	C	T	intergenic	*ARMCX1,ARMCX6*	.	.	.
X	100837242	A	C	intergenic	*ARMCX1,ARMCX6*	.	.	.
X	100926114	T	C	intergenic	*ARMCX2,NXF5*	.	.	.
X	100966804	G	A	intergenic	*ARMCX2,NXF5*	.	.	.
X	101063324	G	A	intergenic	*ARMCX2,NXF5*	.	.	.
X	100782611	T	C	intergenic	*ARMCX4,ARMCX1*	.	.	.
X	100783916	G	A	intergenic	*ARMCX4,ARMCX1*	.	.	.
X	100784343	A	G	intergenic	*ARMCX4,ARMCX1*	.	.	.
X	100795004	G	C	intergenic	*ARMCX4,ARMCX1*	.	.	.
X	100795045	T	C	intergenic	*ARMCX4,ARMCX1*	.	.	.
X	100797930	C	G	intergenic	*ARMCX4,ARMCX1*	.	.	.
X	101871689	G	A	intronic	*ARMCX5-GPRASP2*	.	.	.

X	101896889	A	G	intronic	*ARMCX5-GPRASP2*	.	.	.
X	101897193	A	G	intronic	*ARMCX5-GPRASP2*	.	.	.
X	101920198	A	G	intronic	*ARMCX5-GPRASP2*	.	.	.
X	101944191	A	G	intronic	*ARMCX5-GPRASP2*	.	.	.
X	101944322	A	G	intronic	*ARMCX5-GPRASP2*	.	.	.
1	150792399	A	C	intronic	*ARNT*	.	.	.
12	27493060	T	G	intronic	*ARNTL2*	.	.	.
12	27493061	C	G	intronic	*ARNTL2*	.	.	.
12	27560004	T	A	ncRNA_intronic	*ARNTL2-AS1*	.	.	.
12	27562442	C	T	ncRNA_intronic	*ARNTL2-AS1*	.	.	.
3	35967970	T	C	intergenic	*ARPP21,STAC*	.	.	.
3	36384134	G	A	intergenic	*ARPP21,STAC*	.	.	.
3	35860590	A	G	intergenic	*ARPP21,STAC*	.	.	.
3	36367383	C	T	intergenic	*ARPP21,STAC*	.	.	.
3	36367764	T	C	intergenic	*ARPP21,STAC*	.	.	.
5	91827973	CATAT	C	intergenic	*ARRDC3-AS1,NR2F1-AS1*	.	.	.
5	91975364	G	T	intergenic	*ARRDC3-AS1,NR2F1-AS1*	.	.	.
5	92133066	G	C	intergenic	*ARRDC3-AS1,NR2F1-AS1*	.	.	.
5	92090382	C	A	intergenic	*ARRDC3-AS1,NR2F1-AS1*	.	.	.
5	92674209	C	CT	intergenic	*ARRDC3-AS1,NR2F1-AS1*	.	.	.
5	90853326	T	A	intergenic	*ARRDC3-AS1,NR2F1-AS1*	.	.	.
5	90855209	T	C	intergenic	*ARRDC3-AS1,NR2F1-AS1*	.	.	.
5	90860508	T	C	intergenic	*ARRDC3-AS1,NR2F1-AS1*	.	.	.
5	90973625	G	A	intergenic	*ARRDC3-AS1,NR2F1-AS1*	.	.	.

5	91106248	G	A	intergenic	*ARRDC3-AS1,NR2F1-AS1*	.	.	.
5	91325420	A	G	intergenic	*ARRDC3-AS1,NR2F1-AS1*	.	.	.
5	91428983	C	A	intergenic	*ARRDC3-AS1,NR2F1-AS1*	.	.	.
5	91428984	A	G	intergenic	*ARRDC3-AS1,NR2F1-AS1*	.	.	.
5	91462311	C	G	intergenic	*ARRDC3-AS1,NR2F1-AS1*	.	.	.
5	91489664	C	T	intergenic	*ARRDC3-AS1,NR2F1-AS1*	.	.	.
5	91491224	G	A	intergenic	*ARRDC3-AS1,NR2F1-AS1*	.	.	.
5	91492084	G	A	intergenic	*ARRDC3-AS1,NR2F1-AS1*	.	.	.
5	91577863	T	C	intergenic	*ARRDC3-AS1,NR2F1-AS1*	.	.	.
5	91593560	T	C	intergenic	*ARRDC3-AS1,NR2F1-AS1*	.	.	.
5	91594656	A	G	intergenic	*ARRDC3-AS1,NR2F1-AS1*	.	.	.
5	91595654	C	T	intergenic	*ARRDC3-AS1,NR2F1-AS1*	.	.	.
5	91595655	A	G	intergenic	*ARRDC3-AS1,NR2F1-AS1*	.	.	.
5	91723578	T	TA	intergenic	*ARRDC3-AS1,NR2F1-AS1*	.	.	.
5	91776903	G	A	intergenic	*ARRDC3-AS1,NR2F1-AS1*	.	.	.
5	91776904	A	T	intergenic	*ARRDC3-AS1,NR2F1-AS1*	.	.	.
5	92552238	A	G	intergenic	*ARRDC3-AS1,NR2F1-AS1*	.	.	.
5	92554481	G	A	intergenic	*ARRDC3-AS1,NR2F1-AS1*	.	.	.
5	78077103	A	C	intronic	*ARSB*	.	.	.
5	78136978	ACT	A	intronic	*ARSB*	.	.	.

5	78174684	T	C	intronic	*ARSB*	.	.	.
5	78157762	G	A	intronic	*ARSB*	.	.	.
5	78158907	G	A	intronic	*ARSB*	.	.	.
5	78159065	T	C	intronic	*ARSB*	.	.	.
5	78159066	G	C	intronic	*ARSB*	.	.	.
5	78239113	C	A	intronic	*ARSB*	.	.	.
5	78239614	A	G	intronic	*ARSB*	.	.	.
5	78285917	G	T	intergenic	*ARSB,DMGDH*	.	.	.
X	3015515	T	C	intronic	*ARSF*	.	.	.
X	2979944	G	A	intronic	*ARSF*	.	.	.
X	2979945	G	A	intronic	*ARSF*	.	.	.
X	2982132	C	G	intronic	*ARSF*	.	.	.
X	3053128	GGAGA	G	intergenic	*ARSF,LINC01546*	.	.	.
17	66256031	A	G	intronic	*ARSG*	.	.	.
17	66407475	T	C	intronic	*ARSG*	.	.	.
17	66276165	C	A	intronic	*ARSG,SLC16A6*	.	.	.
X	2927250	G	T	intronic	*ARSH*	.	.	.
4	114859040	T	A	intronic	*ARSJ*	.	.	.
4	114859041	G	A	intronic	*ARSJ*	.	.	.
4	115006701	AC	A	intergenic	*ARSJ,UGT8*	.	.	.
4	115006703	A	T	intergenic	*ARSJ,UGT8*	.	.	.
4	115285604	C	T	intergenic	*ARSJ,UGT8*	.	.	.
4	115346107	T	C	intergenic	*ARSJ,UGT8*	.	.	.
4	115444527	A	G	intergenic	*ARSJ,UGT8*	.	.	.
4	115461816	T	C	intergenic	*ARSJ,UGT8*	.	.	.
4	115461818	C	T	intergenic	*ARSJ,UGT8*	.	.	.
12	15012059	A	AT	intergenic	*ART4,MGP*	.	.	.
12	15012061	T	C	intergenic	*ART4,MGP*	.	.	.
12	15015318	T	A	intergenic	*ART4,MGP*	.	.	.
X	25712565	T	C	intergenic	*ARX,MAGEB18*	.	.	.
X	25883931	C	A	intergenic	*ARX,MAGEB18*	.	.	.
X	26131084	A	T	intergenic	*ARX,MAGEB18*	.	.	.
X	25532895	A	T	intergenic	*ARX,MAGEB18*	.	.	.
X	25218257	T	G	intergenic	*ARX,MAGEB18*	.	.	.
X	25236172	C	T	intergenic	*ARX,MAGEB18*	.	.	.
X	25470281	G	GA	intergenic	*ARX,MAGEB18*	.	.	.
X	25516055	G	T	intergenic	*ARX,MAGEB18*	.	.	.
X	25703957	T	A	intergenic	*ARX,MAGEB18*	.	.	.

X	26034491	T	A	intergenic	*ARX,MAGEB18*	.	.	.
X	26044240	GCACA	G	intergenic	*ARX,MAGEB18*	.	.	.
X	25365452	C	CA	intergenic	*ARX,MAGEB18*	.	.	.
X	25634822	T	C	intergenic	*ARX,MAGEB18*	.	.	.
X	25754702	T	C	intergenic	*ARX,MAGEB18*	.	.	.
X	25866188	G	T	intergenic	*ARX,MAGEB18*	.	.	.
X	25986714	G	A	intergenic	*ARX,MAGEB18*	.	.	.
X	26003593	T	C	intergenic	*ARX,MAGEB18*	.	.	.
X	25860619	C	T	intergenic	*ARX,MAGEB18*	.	.	.
X	25086043	G	T	intergenic	*ARX,MAGEB18*	.	.	.
X	25138011	A	T	intergenic	*ARX,MAGEB18*	.	.	.
X	25138012	T	C	intergenic	*ARX,MAGEB18*	.	.	.
X	25139947	T	C	intergenic	*ARX,MAGEB18*	.	.	.
X	25320492	C	T	intergenic	*ARX,MAGEB18*	.	.	.
X	25329996	A	G	intergenic	*ARX,MAGEB18*	.	.	.
X	25345261	A	C	intergenic	*ARX,MAGEB18*	.	.	.
X	25426715	A	T	intergenic	*ARX,MAGEB18*	.	.	.
X	25428342	T	C	intergenic	*ARX,MAGEB18*	.	.	.
X	25430488	A	G	intergenic	*ARX,MAGEB18*	.	.	.
X	25440124	G	T	intergenic	*ARX,MAGEB18*	.	.	.
X	25440710	G	T	intergenic	*ARX,MAGEB18*	.	.	.
X	25792926	A	T	intergenic	*ARX,MAGEB18*	.	.	.
X	25793735	T	C	intergenic	*ARX,MAGEB18*	.	.	.
X	25902947	C	T	intergenic	*ARX,MAGEB18*	.	.	.
X	25902949	G	A	intergenic	*ARX,MAGEB18*	.	.	.
X	25905712	C	G	intergenic	*ARX,MAGEB18*	.	.	.
X	25998722	G	A	intergenic	*ARX,MAGEB18*	.	.	.
10	51975866	A	G	intronic	*ASAH2*	.	.	.
10	52028077	C	T	intergenic	*ASAH2,SGMS1*	.	.	.
10	52029360	G	C	intergenic	*ASAH2,SGMS1*	.	.	.
10	52544936	C	T	intergenic	*ASAH2B,A1CF*	.	.	.
10	52545885	A	G	intergenic	*ASAH2B,A1CF*	.	.	.
8	131427022	G	T	intronic	*ASAP1*	.	.	.
8	131427024	G	T	intronic	*ASAP1*	.	.	.
8	131481264	C	T	intergenic	*ASAP1,ADCY8*	.	.	.
2	9445275	T	C	intronic	*ASAP2*	.	.	.
2	239390834	A	AC	intergenic	*ASB1,LINC01107*	.	.	.
X	63452079	C	G	intergenic	*ASB12,MTMR8*	.	.	.

X	63473322	A	T	intergenic	*ASB12,MTMR8*	.	.	.
7	123273668	AGTGT	A	intronic	*ASB15*	.	.	.
1	76385533	T	C	intronic	*ASB17*	.	.	.
15	101217111	A	AG	intergenic	*ASB7,ALDH1A3*	.	.	.
15	101217112	A	T	intergenic	*ASB7,ALDH1A3*	.	.	.
15	93420845	G	A	intergenic	*ASB9P1,LINC01578*	.	.	.
10	73935746	A	G	intronic	*ASCC1*	.	.	.
22	30237358	TA	T	intergenic	*ASCC2,MTMR3*	.	.	.
22	30237360	TTA	T	intergenic	*ASCC2,MTMR3*	.	.	.
6	101026498	T	A	intronic	*ASCC3*	.	.	.
6	101029339	A	T	intronic	*ASCC3*	.	.	.
6	101206829	C	A	intronic	*ASCC3*	.	.	.
6	101206835	C	T	intronic	*ASCC3*	.	.	.
6	101718670	TAAGA	T	intergenic	*ASCC3,GRIK2*	.	.	.
6	101358098	A	G	intergenic	*ASCC3,GRIK2*	.	.	.
6	101359060	C	T	intergenic	*ASCC3,GRIK2*	.	.	.
6	101440178	C	T	intergenic	*ASCC3,GRIK2*	.	.	.
6	101477296	C	T	intergenic	*ASCC3,GRIK2*	.	.	.
6	101495245	C	T	intergenic	*ASCC3,GRIK2*	.	.	.
6	101512956	G	A	intergenic	*ASCC3,GRIK2*	.	.	.
6	101516074	A	G	intergenic	*ASCC3,GRIK2*	.	.	.
6	101591029	T	C	intergenic	*ASCC3,GRIK2*	.	.	.
6	101591030	G	A	intergenic	*ASCC3,GRIK2*	.	.	.
6	101634225	T	C	intergenic	*ASCC3,GRIK2*	.	.	.
6	101639177	G	A	intergenic	*ASCC3,GRIK2*	.	.	.
6	101662754	C	T	intergenic	*ASCC3,GRIK2*	.	.	.
6	101695918	G	C	intergenic	*ASCC3,GRIK2*	.	.	.
6	101696105	C	A	intergenic	*ASCC3,GRIK2*	.	.	.
6	101696705	G	A	intergenic	*ASCC3,GRIK2*	.	.	.
6	101794705	T	C	intergenic	*ASCC3,GRIK2*	.	.	.
12	103355982	C	G	intergenic	*ASCL1,LOC101929058*	.	.	.
12	103461192	A	G	intergenic	*ASCL1,LOC101929058*	.	.	.
12	103451419	A	G	intergenic	*ASCL1,LOC101929058*	.	.	.

12	108282338	T	A	intergenic	*ASCL4,LOC728739*	.	.	.
12	108257039	T	C	intergenic	*ASCL4,LOC728739*	.	.	.
17	7053152	T	C	intergenic	*ASGR2,ASGR1*	.	.	.
17	31501006	C	T	intronic	*ASIC2*	.	.	.
17	31449844	T	C	intronic	*ASIC2*	.	.	.
17	32100948	A	T	intronic	*ASIC2*	.	.	.
17	31893978	C	A	intronic	*ASIC2*	.	.	.
17	31952313	G	A	intronic	*ASIC2*	.	.	.
17	31392691	G	T	intronic	*ASIC2*	.	.	.
17	32285905	C	G	intronic	*ASIC2*	.	.	.
17	31869264	A	C	intronic	*ASIC2*	.	.	.
17	32206308	T	C	intronic	*ASIC2*	.	.	.
17	32410926	G	A	intronic	*ASIC2*	.	.	.
X	1743860	A	C	intronic	*ASMT*	.	.	.
X	2120694	A	ATG	intergenic	*ASMT,DHRSX*	.	.	.
X	2044833	G	T	intergenic	*ASMT,DHRSX*	.	.	.
X	2014731	G	C	intergenic	*ASMT,DHRSX*	.	.	.
X	1940635	T	C	intergenic	*ASMT,DHRSX*	.	.	.
X	1801801	C	T	intergenic	*ASMT,DHRSX*	.	.	.
X	1917664	G	A	intergenic	*ASMT,DHRSX*	.	.	.
X	1536699	GAGAGAC	G	intronic	*ASMTL*	.	.	.
7	97547854	A	C	intergenic	*ASNS,MIR5692A1*	.	.	.
7	97531951	G	T	intergenic	*ASNS,MIR5692A1*	.	.	.
1	197080240	A	C	intronic	*ASPM*	.	.	.
9	133327539	G	A	intronic	*ASS1*	.	.	.
1	177110213	G	A	intronic	*ASTN1*	.	.	.
9	119813199	C	A	intronic	*ASTN2*	.	.	.
9	120079300	T	G	intronic	*ASTN2*	.	.	.
9	120111117	AT	A	intronic	*ASTN2*	.	.	.
9	119473611	C	T	intronic	*ASTN2*	.	.	.
9	119963483	T	A	intronic	*ASTN2*	.	.	.
9	120164807	C	G	intronic	*ASTN2*	.	.	.
9	119263513	C	T	intronic	*ASTN2*	.	.	.
9	119828712	G	A	intronic	*ASTN2*	.	.	.
9	119961421	A	C	intronic	*ASTN2*	.	.	.
9	119275992	T	C	ncRNA_intronic	*ASTN2-AS1*	.	.	.

9	119296916	A	G	ncRNA_intronic	*ASTN2-AS1*	.	.	.
9	119297667	G	A	ncRNA_intronic	*ASTN2-AS1*	.	.	.
9	120249994	C	G	intergenic	*ASTN2,LOC101928797*	.	.	.
9	120386694	G	T	intergenic	*ASTN2,LOC101928797*	.	.	.
2	26047347	TACAC	T	intronic	*ASXL2*	.	.	.
18	31323688	C	T	exonic	*ASXL3*	synonymous_SNV	.	.
18	31349739	A	G	intergenic	*ASXL3,NOL4*	.	.	.
7	117044516	A	C	intronic	*ASZ1*	.	.	.
7	117062510	T	C	intronic	*ASZ1*	.	.	.
7	117117644	A	G	intergenic	*ASZ1,CFTR*	.	.	.
7	117103720	T	C	intergenic	*ASZ1,CFTR*	.	.	.
10	89522870	T	A	intronic	*ATAD1*	.	.	.
2	24018245	G	C	intronic	*ATAD2B*	.	.	.
2	24093014	T	A	intronic	*ATAD2B*	.	.	.
2	24033884	AAAACAAAC	A	intronic	*ATAD2B*	.	.	.
1	1398893	G	A	intronic	*ATAD3C*	.	.	.
17	29165429	A	G	intronic	*ATAD5*	.	.	.
17	29160923	T	C	intronic	*ATAD5*	.	.	.
17	29170488	C	T	intronic	*ATAD5*	.	.	.
2	175948449	A	G	intronic	*ATF2*	.	.	.
1	212752495	G	A	intronic	*ATF3*	.	.	.
1	161800559	G	C	intronic	*ATF6*	.	.	.
1	161887303	C	T	intronic	*ATF6*	.	.	.
1	161944318	T	C	intergenic	*ATF6,OLFML2B*	.	.	.
12	14542682	C	CAGA	intronic	*ATF7IP*	.	.	.
16	10480448	C	T	intronic	*ATF7IP2*	.	.	.
16	10495286	C	A	intronic	*ATF7IP2*	.	.	.
5	81344319	G	T	intronic	*ATG10*	.	.	.
5	81406537	T	A	intronic	*ATG10*	.	.	.
5	81406539	A	G	intronic	*ATG10*	.	.	.
5	81418535	T	C	intronic	*ATG10*	.	.	.
5	81428069	ATTT	A	intronic	*ATG10*	.	.	.
5	81432002	A	G	intronic	*ATG10*	.	.	.
2	234211571	T	C	intergenic	*ATG16L1,SAG*	.	.	.

X	107358348	CGTGTGTGTGTGTGT	C	intronic	*ATG4A*	.	.	.
1	63479319	T	TATAC	intergenic	*ATG4C,LINC00466*	.	.	.
6	106696762	G	A	intronic	*ATG5*	.	.	.
6	106884985	C	A	intergenic	*ATG5,AIM1*	.	.	.
3	11408445	CTGTGTGTGTGTG	C	intronic	*ATG7*	.	.	.
11	108224095	CT	C	intronic	*ATM*	.	.	.
12	7048938	AT	A	intronic	*ATN1*	.	.	.
4	94978587	C	A	intergenic	*ATOH1,LOC101929210*	.	.	.
4	94805986	G	GTTGT	intergenic	*ATOH1,LOC101929210*	.	.	.
5	160240761	T	C	intronic	*ATP10B*	.	.	.
5	160031726	CAT	C	intronic	*ATP10B*	.	.	.
5	160008646	T	G	intronic	*ATP10B*	.	.	.
5	160090358	A	G	intronic	*ATP10B*	.	.	.
5	160116534	A	G	intronic	*ATP10B*	.	.	.
5	160324503	A	C	intergenic	*ATP10B,LOC285629*	.	.	.
5	160325018	T	C	intergenic	*ATP10B,LOC285629*	.	.	.
4	47505921	A	G	intronic	*ATP10D*	.	.	.
13	113469099	G	A	intronic	*ATP11A*	.	.	.
X	138834844	T	G	intronic	*ATP11C*	.	.	.
X	138860948	G	A	intronic	*ATP11C*	.	.	.
X	138962455	AAGAT	A	intergenic	*ATP11C,MIR505*	.	.	.
13	25257336	T	C	intronic	*ATP12A*	.	.	.
3	193249098	A	AGGGAG	intronic	*ATP13A4*	.	.	.
3	193141631	GAA	G	intronic	*ATP13A4*	.	.	.
1	160134924	C	T	intronic	*ATP1A4*	.	.	.
X	119520036	T	G	intergenic	*ATP1B4,LAMP2*	.	.	.
12	110795999	GC	G	intergenic	*ATP2A2,ANAPC7*	.	.	.
12	90070712	T	C	intergenic	*ATP2B1,LINC00936*	.	.	.
X	152833610	C	A	intronic	*ATP2B3*	.	.	.
3	130654805	C	T	intronic	*ATP2C1*	.	.	.

19	36056852	C	T	intergenic	*ATP4A,LOC102723617*	.	.	.
19	36082389	G	A	intergenic	*ATP4A,LOC102723617*	.	.	.
13	28533573	G	A	intergenic	*ATP5EP2,CDX2*	.	.	.
2	176739037	A	G	intergenic	*ATP5G3,KIAA1715*	.	.	.
2	176397285	T	C	intergenic	*ATP5G3,KIAA1715*	.	.	.
2	176055844	G	A	intergenic	*ATP5G3,KIAA1715*	.	.	.
2	176369620	A	G	intergenic	*ATP5G3,KIAA1715*	.	.	.
2	176467906	T	C	intergenic	*ATP5G3,KIAA1715*	.	.	.
2	176468175	T	C	intergenic	*ATP5G3,KIAA1715*	.	.	.
2	176468439	T	C	intergenic	*ATP5G3,KIAA1715*	.	.	.
2	176468440	G	A	intergenic	*ATP5G3,KIAA1715*	.	.	.
2	176488514	A	G	intergenic	*ATP5G3,KIAA1715*	.	.	.
2	176538437	T	C	intergenic	*ATP5G3,KIAA1715*	.	.	.
2	176538438	T	C	intergenic	*ATP5G3,KIAA1715*	.	.	.
2	176585382	A	G	intergenic	*ATP5G3,KIAA1715*	.	.	.
2	176777028	A	C	intergenic	*ATP5G3,KIAA1715*	.	.	.
2	176777030	C	T	intergenic	*ATP5G3,KIAA1715*	.	.	.
21	35300983	A	G	intergenic	*ATP5O,LINC00649*	.	.	.
21	35303646	C	A	intergenic	*ATP5O,LINC00649*	.	.	.
19	41938263	C	A	exonic	*ATP5SL*	nonsynonymous_SNV	.	.

5	81821799	A	G	intergenic	*ATP6AP1L,MIR3977*	.	.	.
5	82032553	G	A	intergenic	*ATP6AP1L,MIR3977*	.	.	.
5	81730120	C	A	intergenic	*ATP6AP1L,MIR3977*	.	.	.
5	81730122	G	T	intergenic	*ATP6AP1L,MIR3977*	.	.	.
5	81913021	T	G	intergenic	*ATP6AP1L,MIR3977*	.	.	.
5	81913356	T	C	intergenic	*ATP6AP1L,MIR3977*	.	.	.
5	81973762	C	T	intergenic	*ATP6AP1L,MIR3977*	.	.	.
5	81973763	A	G	intergenic	*ATP6AP1L,MIR3977*	.	.	.
5	81987281	T	C	intergenic	*ATP6AP1L,MIR3977*	.	.	.
17	40676782	G	A	intergenic	*ATP6V0A1,NAGLU*	.	.	.
7	138390492	C	T	downstream	*ATP6V0A4*	.	.	.
7	138390493	A	G	downstream	*ATP6V0A4*	.	.	.
8	87182581	G	A	intergenic	*ATP6V0D2,SLC7A13*	.	.	.
8	87182582	C	G	intergenic	*ATP6V0D2,SLC7A13*	.	.	.
8	87183298	A	G	intergenic	*ATP6V0D2,SLC7A13*	.	.	.
8	87183473	G	A	intergenic	*ATP6V0D2,SLC7A13*	.	.	.
8	87206853	T	C	intergenic	*ATP6V0D2,SLC7A13*	.	.	.
5	172450201	TATTTATTC	T	intronic	*ATP6V0E1*	.	.	.
7	149675092	C	G	intergenic	*ATP6V0E2,ACTR3C*	.	.	.
7	149838527	G	A	intergenic	*ATP6V0E2,ACTR3C*	.	.	.
7	149601987	C	T	intergenic	*ATP6V0E2,ACTR3C*	.	.	.

7	149817536	T	C	intergenic	*ATP6V0E2,ACTR3C*	.	.	.
2	71201893	A	G	intergenic	*ATP6V1B1,ANKRD53*	.	.	.
8	104143246	CTTTTTT	C	intergenic	*ATP6V1C1,BAALC-AS2*	.	.	.
8	54693964	C	T	intronic	*ATP6V1H*	.	.	.
X	77184066	C	A	intronic	*ATP7A*	.	.	.
X	77335745	A	C	intergenic	*ATP7A,PGK1*	.	.	.
4	42501959	C	A	intronic	*ATP8A1*	.	.	.
4	42814056	G	T	intergenic	*ATP8A1,GRXCR1*	.	.	.
4	42780172	C	G	intergenic	*ATP8A1,GRXCR1*	.	.	.
4	42799582	C	T	intergenic	*ATP8A1,GRXCR1*	.	.	.
4	42807475	T	C	intergenic	*ATP8A1,GRXCR1*	.	.	.
4	42847008	C	A	intergenic	*ATP8A1,GRXCR1*	.	.	.
4	42890443	TTCTC	T	intergenic	*ATP8A1,GRXCR1*	.	.	.
4	42699938	T	C	intergenic	*ATP8A1,GRXCR1*	.	.	.
4	42794085	G	A	intergenic	*ATP8A1,GRXCR1*	.	.	.
4	42795339	T	A	intergenic	*ATP8A1,GRXCR1*	.	.	.
4	42824743	C	T	intergenic	*ATP8A1,GRXCR1*	.	.	.
4	42824744	T	G	intergenic	*ATP8A1,GRXCR1*	.	.	.
13	26008648	G	A	intronic	*ATP8A2*	.	.	.
13	26148081	A	G	intronic	*ATP8A2*	.	.	.
13	26215402	T	G	intronic	*ATP8A2*	.	.	.
13	26468194	C	T	intronic	*ATP8A2*	.	.	.
13	26470207	A	C	intronic	*ATP8A2*	.	.	.
13	26470257	A	G	intronic	*ATP8A2*	.	.	.
13	26471904	A	T	intronic	*ATP8A2*	.	.	.
13	26471906	G	A	intronic	*ATP8A2*	.	.	.
13	26472289	T	TG	intronic	*ATP8A2*	.	.	.
13	26539152	T	C	intronic	*ATP8A2*	.	.	.
13	26539153	T	C	intronic	*ATP8A2*	.	.	.
18	55353440	G	GGTGCAAA(	intronic	*ATP8B1*	.	.	.
18	55406731	A	G	intronic	*ATP8B1*	.	.	.
18	55391570	C	T	intronic	*ATP8B1*	.	.	.
18	55614463	T	C	intergenic	*ATP8B1,NEDD4L*	.	.	.
18	76833633	A	G	intronic	*ATP9B*	.	.	.
18	76833635	G	A	intronic	*ATP9B*	.	.	.

18	76875025	C	T	intronic	*ATP9B*	.	.	.
18	76909919	G	A	intronic	*ATP9B*	.	.	.
18	76949204	G	A	intronic	*ATP9B*	.	.	.
18	77031916	C	T	intronic	*ATP9B*	.	.	.
18	77045600	C	G	intronic	*ATP9B*	.	.	.
18	77092245	C	T	intronic	*ATP9B*	.	.	.
17	17933664	T	A	intronic	*ATPAF2*	.	.	.
3	142310509	T	A	intergenic	*ATR,PLS1*	.	.	.
20	3507504	C	T	intronic	*ATRN*	.	.	.
10	117102669	A	G	intronic	*ATRNL1*	.	.	.
10	117197610	C	CA	intronic	*ATRNL1*	.	.	.
10	117408953	A	T	intronic	*ATRNL1*	.	.	.
10	117497739	T	A	intronic	*ATRNL1*	.	.	.
10	116892713	C	T	intronic	*ATRNL1*	.	.	.
10	116894406	T	C	intronic	*ATRNL1*	.	.	.
10	116911686	C	T	intronic	*ATRNL1*	.	.	.
10	116967169	G	A	intronic	*ATRNL1*	.	.	.
10	117015232	C	T	intronic	*ATRNL1*	.	.	.
10	117246218	A	G	intronic	*ATRNL1*	.	.	.
10	117246219	A	G	intronic	*ATRNL1*	.	.	.
10	117248564	T	C	intronic	*ATRNL1*	.	.	.
10	117293288	C	T	intronic	*ATRNL1*	.	.	.
10	117343517	G	A	intronic	*ATRNL1*	.	.	.
10	117514198	G	T	intronic	*ATRNL1*	.	.	.
10	117581614	T	C	intronic	*ATRNL1*	.	.	.
10	117581615	A	T	intronic	*ATRNL1*	.	.	.
10	117582849	G	A	intronic	*ATRNL1*	.	.	.
10	117583961	T	C	intronic	*ATRNL1*	.	.	.
10	117658533	C	T	intronic	*ATRNL1*	.	.	.
10	117683688	C	A	intronic	*ATRNL1*	.	.	.
10	117683689	A	T	intronic	*ATRNL1*	.	.	.
10	117683765	A	T	intronic	*ATRNL1*	.	.	.
10	117684390	A	G	intronic	*ATRNL1*	.	.	.
10	117795449	G	T	intergenic	*ATRNL1,GFRA1*	.	.	.
X	76817724	C	G	intronic	*ATRX*	.	.	.
X	76939129	C	T	exonic	*ATRX*	nonsynonymous_SNV	.	.
X	76991101	AAAT	A	intronic	*ATRX*	.	.	.

X	77002334	G	T	intronic	*ATRX*	.	.	.
X	76907626	C	T	exonic	*ATRX*	nonsynonymous_SNV	.	.
X	76787143	T	C	intronic	*ATRX*	.	.	.
X	76787144	G	A	intronic	*ATRX*	.	.	.
X	76806708	T	C	intronic	*ATRX*	.	.	.
X	76919514	C	T	intronic	*ATRX*	.	.	.
X	76975003	C	T	intronic	*ATRX*	.	.	.
X	76976693	C	T	intronic	*ATRX*	.	.	.
X	76980680	G	A	intronic	*ATRX*	.	.	.
X	77000654	G	GAAAAAAAA	intronic	*ATRX*	.	.	.
X	77077972	A	G	intergenic	*ATRX,MAGT1*	.	.	.
6	16601671	G	T	intronic	*ATXN1*	.	.	.
6	16395332	G	A	intronic	*ATXN1*	.	.	.
6	16702819	G	T	intronic	*ATXN1*	.	.	.
6	17094286	A	C	intergenic	*ATXN1,STMND1*	.	.	.
6	17077084	G	A	intergenic	*ATXN1,STMND1*	.	.	.
22	46184334	C	T	intronic	*ATXN10*	.	.	.
7	105547922	C	T	intergenic	*ATXN7L1,CDHR3*	.	.	.
7	105590106	A	T	intergenic	*ATXN7L1,CDHR3*	.	.	.
12	75057197	C	T	intergenic	*ATXN7L3B,KCNC2*	.	.	.
12	75129471	G	T	intergenic	*ATXN7L3B,KCNC2*	.	.	.
12	75203740	C	T	intergenic	*ATXN7L3B,KCNC2*	.	.	.
12	75074477	G	A	intergenic	*ATXN7L3B,KCNC2*	.	.	.
12	75075183	A	T	intergenic	*ATXN7L3B,KCNC2*	.	.	.
12	75180664	A	G	intergenic	*ATXN7L3B,KCNC2*	.	.	.
12	75396230	A	T	intergenic	*ATXN7L3B,KCNC2*	.	.	.
13	70685385	T	A	ncRNA_intronic	*ATXN8OS*	.	.	.
13	70713823	G	T	ncRNA_exonic	*ATXN8OS*	.	.	.
13	70694593	C	T	ncRNA_intronic	*ATXN8OS*	.	.	.
13	70975282	T	TA	intergenic	*ATXN8OS,LINC00348*	.	.	.
13	71426107	A	T	intergenic	*ATXN8OS,LINC00348*	.	.	.

13	71335684	A	C	intergenic	*ATXN8OS,LINC00348*	.	.	.
13	70992695	G	T	intergenic	*ATXN8OS,LINC00348*	.	.	.
13	71288476	C	CATAT	intergenic	*ATXN8OS,LINC00348*	.	.	.
13	71407526	G	T	intergenic	*ATXN8OS,LINC00348*	.	.	.
13	71543075	A	G	intergenic	*ATXN8OS,LINC00348*	.	.	.
13	71124708	TC	T	intergenic	*ATXN8OS,LINC00348*	.	.	.
13	71527174	ATTT	A	intergenic	*ATXN8OS,LINC00348*	.	.	.
13	70863087	C	T	intergenic	*ATXN8OS,LINC00348*	.	.	.
13	71014098	A	G	intergenic	*ATXN8OS,LINC00348*	.	.	.
13	71014100	A	T	intergenic	*ATXN8OS,LINC00348*	.	.	.
13	71122859	G	A	intergenic	*ATXN8OS,LINC00348*	.	.	.
13	71555615	TTTTC	T	intergenic	*ATXN8OS,LINC00348*	.	.	.
9	94040369	C	A	intronic	*AUH*	.	.	.
9	94051378	T	C	intronic	*AUH*	.	.	.
7	69835544	C	T	intronic	*AUTS2*	.	.	.
7	69341484	A	G	intronic	*AUTS2*	.	.	.
7	69341486	A	G	intronic	*AUTS2*	.	.	.
7	69522546	CA	C	intronic	*AUTS2*	.	.	.
7	69728211	C	A	intronic	*AUTS2*	.	.	.
7	69289539	C	T	intronic	*AUTS2*	.	.	.
7	69419376	C	T	intronic	*AUTS2*	.	.	.
7	69875837	G	GTTATTA	intronic	*AUTS2*	.	.	.
7	69875957	T	C	intronic	*AUTS2*	.	.	.
7	70024414	G	A	intronic	*AUTS2*	.	.	.
7	70507362	G	A	intergenic	*AUTS2,WBSCR17*	.	.	.
7	70355695	C	T	intergenic	*AUTS2,WBSCR17*	.	.	.

7	70581942	G	A	intergenic	*AUTS2,WBSCR17*	.	.	.
7	70569517	G	T	intergenic	*AUTS2,WBSCR17*	.	.	.
7	70305317	G	T	intergenic	*AUTS2,WBSCR17*	.	.	.
15	34246273	C	T	intronic	*AVEN*	.	.	.
10	99451487	T	A	intergenic	*AVPI1,MARVELD1*	.	.	.
12	63596129	GTATA	G	intergenic	*AVPR1A,DPY19L2*	.	.	.
12	63583050	C	T	intergenic	*AVPR1A,DPY19L2*	.	.	.
12	63583306	A	G	intergenic	*AVPR1A,DPY19L2*	.	.	.
12	63584732	A	T	intergenic	*AVPR1A,DPY19L2*	.	.	.
12	63593155	A	G	intergenic	*AVPR1A,DPY19L2*	.	.	.
12	63594284	A	C	intergenic	*AVPR1A,DPY19L2*	.	.	.
12	63594286	T	G	intergenic	*AVPR1A,DPY19L2*	.	.	.
12	63636652	G	A	intergenic	*AVPR1A,DPY19L2*	.	.	.
12	63638152	G	T	intergenic	*AVPR1A,DPY19L2*	.	.	.
12	63638534	C	T	intergenic	*AVPR1A,DPY19L2*	.	.	.
12	63733050	G	A	intergenic	*AVPR1A,DPY19L2*	.	.	.
12	63733612	G	A	intergenic	*AVPR1A,DPY19L2*	.	.	.
12	63762592	T	C	intergenic	*AVPR1A,DPY19L2*	.	.	.
12	63762593	A	G	intergenic	*AVPR1A,DPY19L2*	.	.	.
12	63774041	T	A	intergenic	*AVPR1A,DPY19L2*	.	.	.
12	63830989	A	G	intergenic	*AVPR1A,DPY19L2*	.	.	.
12	63831203	T	C	intergenic	*AVPR1A,DPY19L2*	.	.	.
12	63837286	T	C	intergenic	*AVPR1A,DPY19L2*	.	.	.
12	63882839	G	T	intergenic	*AVPR1A,DPY19L2*	.	.	.
12	63884021	G	T	intergenic	*AVPR1A,DPY19L2*	.	.	.
12	63884023	C	T	intergenic	*AVPR1A,DPY19L2*	.	.	.
12	63885869	T	A	intergenic	*AVPR1A,DPY19L2*	.	.	.
1	179402668	A	C	intronic	*AXDND1*	.	.	.
1	179342406	A	G	intronic	*AXDND1*	.	.	.
1	179430139	T	C	intronic	*AXDND1*	.	.	.
1	179431058	G	T	intronic	*AXDND1*	.	.	.
1	179431442	A	G	intronic	*AXDND1*	.	.	.
1	179431444	A	G	intronic	*AXDND1*	.	.	.
1	179432270	G	T	intronic	*AXDND1*	.	.	.
1	179468021	T	C	intronic	*AXDND1*	.	.	.
8	103938315	A	T	ncRNA_intronic	*AZIN1-AS1*	.	.	.
3	160848764	T	C	intergenic	*B3GALNT1,NMD3*	.	.	.
21	41010476	C	A	intronic	*B3GALT5*	.	.	.

6	71627439	A	G	intronic	*B3GAT2*	.	.	.
6	71637289	TA	T	intronic	*B3GAT2*	.	.	.
6	71786972	T	G	intergenic	*B3GAT2,OGFRL1*	.	.	.
6	71846273	C	A	intergenic	*B3GAT2,OGFRL1*	.	.	.
6	71686307	A	C	intergenic	*B3GAT2,OGFRL1*	.	.	.
6	71710564	G	A	intergenic	*B3GAT2,OGFRL1*	.	.	.
6	71711754	T	G	intergenic	*B3GAT2,OGFRL1*	.	.	.
6	71711756	G	C	intergenic	*B3GAT2,OGFRL1*	.	.	.
6	71850187	A	G	intergenic	*B3GAT2,OGFRL1*	.	.	.
6	71853172	A	G	intergenic	*B3GAT2,OGFRL1*	.	.	.
6	71853302	C	A	intergenic	*B3GAT2,OGFRL1*	.	.	.
6	71854453	G	A	intergenic	*B3GAT2,OGFRL1*	.	.	.
6	71855978	T	C	intergenic	*B3GAT2,OGFRL1*	.	.	.
13	31829543	T	C	intronic	*B3GLCT*	.	.	.
13	32032269	G	A	intergenic	*B3GLCT,RXFP2*	.	.	.
13	32134494	C	T	intergenic	*B3GLCT,RXFP2*	.	.	.
13	32136503	C	T	intergenic	*B3GLCT,RXFP2*	.	.	.
13	32153330	A	G	intergenic	*B3GLCT,RXFP2*	.	.	.
13	32208700	A	G	intergenic	*B3GLCT,RXFP2*	.	.	.
13	32267583	C	T	intergenic	*B3GLCT,RXFP2*	.	.	.
2	62620047	G	A	intergenic	*B3GNT2,TMEM17*	.	.	.
19	17915010	A	T	intronic	*B3GNT3*	.	.	.
2	232259354	G	A	upstream	*B3GNT7*	.	.	.
17	80930228	C	CT	intronic	*B3GNTL1*	.	.	.
17	80930249	A	G	intronic	*B3GNTL1*	.	.	.
17	80912774	G	A	intronic	*B3GNTL1*	.	.	.
12	58079351	C	A	intergenic	*B4GALNT1,OS9*	.	.	.
8	104278118	A	G	intergenic	*BAALC,FZD6*	.	.	.
8	104303936	A	G	intergenic	*BAALC,FZD6*	.	.	.
8	104304168	A	G	intergenic	*BAALC,FZD6*	.	.	.
21	30676465	G	T	intronic	*BACH1*	.	.	.
21	30698000	G	A	intronic	*BACH1*	.	.	.
6	90742074	C	T	intronic	*BACH2*	.	.	.
6	90917973	G	T	intronic	*BACH2*	.	.	.
6	90918403	T	C	intronic	*BACH2*	.	.	.
21	11100930	C	A	intergenic	*BAGE,NONE*	.	.	.
21	11131065	A	AT	intergenic	*BAGE,NONE*	.	.	.
21	11184956	C	A	intergenic	*BAGE,NONE*	.	.	.

21	11160089	T	C	intergenic	*BAGE,NONE*	.	.	.
21	11161646	C	A	intergenic	*BAGE,NONE*	.	.	.
21	11162566	A	G	intergenic	*BAGE,NONE*	.	.	.
21	11024529	CAAGAT	C	intronic	*BAGE2,BAGE3,BAGE4,BAGE5*	.	.	.
21	11036712	C	CAAA	intronic	*BAGE2,BAGE3,BAGE4,BAGE5*	.	.	.
17	79394411	C	A	intronic	*BAHCC1*	.	.	.
17	79438290	TCCAC	T	intergenic	*BAHCC1,ACTG1*	.	.	.
17	79016674	T	TTTTAA	intronic	*BAIAP2*	.	.	.
7	98033314	G	A	intergenic	*BAIAP2L1,NPTX2*	.	.	.
10	28989537	T	C	intergenic	*BAMBI,LINC01517*	.	.	.
20	17720901	A	G	intergenic	*BANF2,SNX5*	.	.	.
4	102916601	A	C	intronic	*BANK1*	.	.	.
4	102919105	T	C	intronic	*BANK1*	.	.	.
4	103152760	A	C	intergenic	*BANK1,SLC39A8*	.	.	.
4	103141934	C	A	intergenic	*BANK1,SLC39A8*	.	.	.
16	88019850	G	A	intronic	*BANP*	.	.	.
16	88012604	T	C	intronic	*BANP*	.	.	.
16	88012605	C	T	intronic	*BANP*	.	.	.
3	52443069	A	AGGGGAAA/	intronic	*BAP1*	.	.	.
3	52443069	A	AGGGG	intronic	*BAP1*	.	.	.
2	215611836	T	C	intronic	*BARD1*	.	.	.
11	129452690	G	C	intergenic	*BARX2,LINC01395*	.	.	.
11	129340046	A	C	intergenic	*BARX2,LINC01395*	.	.	.
11	129368564	A	C	intergenic	*BARX2,LINC01395*	.	.	.
11	129368565	G	A	intergenic	*BARX2,LINC01395*	.	.	.
11	129379448	A	C	intergenic	*BARX2,LINC01395*	.	.	.
11	129472211	G	A	intergenic	*BARX2,LINC01395*	.	.	.
13	23537759	T	G	intergenic	*BASP1P1,SGCG*	.	.	.
13	23518832	G	C	intergenic	*BASP1P1,SGCG*	.	.	.
14	35225413	G	A	intronic	*BAZ1A*	.	.	.
14	35365545	C	T	intergenic	*BAZ1A,IGBP1P1*	.	.	.
7	72914389	T	C	intronic	*BAZ1B*	.	.	.
7	72916409	C	T	intronic	*BAZ1B*	.	.	.
2	160213961	T	C	intronic	*BAZ2B*	.	.	.
2	160217633	A	G	intronic	*BAZ2B*	.	.	.
2	160278848	C	A	intronic	*BAZ2B*	.	.	.

2	160328786	A	G	intronic	*BAZ2B*	.	.	.
2	160391304	T	G	intronic	*BAZ2B*	.	.	.
2	160391306	A	C	intronic	*BAZ2B*	.	.	.
2	160435824	A	T	intronic	*BAZ2B*	.	.	.
14	74504551	G	A	intronic	*BBOF1*	.	.	.
11	27061718	C	G	upstream	*BBOX1*	.	.	.
11	27066394	C	T	intronic	*BBOX1*	.	.	.
11	27098653	C	A	ncRNA_intronic	*BBOX1-AS1*	.	.	.
11	27128165	T	A	ncRNA_intronic	*BBOX1-AS1*	.	.	.
11	27177626	A	G	ncRNA_intronic	*BBOX1-AS1*	.	.	.
11	27292240	A	G	intergenic	*BBOX1-AS1,CCDC34*	.	.	.
11	27345396	T	C	intergenic	*BBOX1-AS1,CCDC34*	.	.	.
11	27347873	A	C	intergenic	*BBOX1-AS1,CCDC34*	.	.	.
4	122793615	T	G	intergenic	*BBS7,TRPC3*	.	.	.
4	122793617	A	T	intergenic	*BBS7,TRPC3*	.	.	.
7	33407700	TCA	T	intronic	*BBS9*	.	.	.
7	33356326	C	G	intronic	*BBS9*	.	.	.
7	33203406	C	T	intronic	*BBS9*	.	.	.
7	33203408	G	A	intronic	*BBS9*	.	.	.
7	33203809	A	T	intronic	*BBS9*	.	.	.
7	33446789	G	A	intronic	*BBS9*	.	.	.
7	33564183	C	T	intronic	*BBS9*	.	.	.
7	33615179	T	C	intronic	*BBS9*	.	.	.
7	33616301	A	G	intronic	*BBS9*	.	.	.
7	33634603	C	T	intronic	*BBS9*	.	.	.
7	33634605	C	T	intronic	*BBS9*	.	.	.
7	33658681	A	G	intergenic	*BBS9,BMPER*	.	.	.
3	107411494	A	G	intronic	*BBX*	.	.	.
3	107488058	T	C	intronic	*BBX*	.	.	.
7	107290072	A	G	intergenic	*BCAP29,SLC26A4-AS1*	.	.	.
1	94324602	A	G	intergenic	*BCAR3,DNTTIP2*	.	.	.
1	94326111	T	C	intergenic	*BCAR3,DNTTIP2*	.	.	.
1	94333617	C	A	intergenic	*BCAR3,DNTTIP2*	.	.	.
20	52571114	A	G	intronic	*BCAS1*	.	.	.

20	52652749	C	A	intronic	*BCAS1*	.	.	.
20	52693132	C	T	intergenic	*BCAS1,CYP24A1*	.	.	.
17	58801883	C	T	intronic	*BCAS3*	.	.	.
17	58947285	C	CA	intronic	*BCAS3*	.	.	.
17	59164270	A	G	intronic	*BCAS3*	.	.	.
17	58867937	T	C	intronic	*BCAS3*	.	.	.
17	58803431	C	T	intronic	*BCAS3*	.	.	.
17	58892291	T	C	intronic	*BCAS3*	.	.	.
12	25109128	T	C	intergenic	*BCAT1,C12orf77*	.	.	.
12	50229126	T	C	ncRNA_intronic	*BCDIN3D-AS1*	.	.	.
3	166891777	A	T	intergenic	*BCHE,ZBBX*	.	.	.
3	166340538	T	A	intergenic	*BCHE,ZBBX*	.	.	.
3	166827829	G	A	intergenic	*BCHE,ZBBX*	.	.	.
3	166141734	C	T	intergenic	*BCHE,ZBBX*	.	.	.
3	166584489	A	C	intergenic	*BCHE,ZBBX*	.	.	.
3	165890290	T	C	intergenic	*BCHE,ZBBX*	.	.	.
3	166206102	T	G	intergenic	*BCHE,ZBBX*	.	.	.
3	166292827	T	C	intergenic	*BCHE,ZBBX*	.	.	.
3	166613838	A	G	intergenic	*BCHE,ZBBX*	.	.	.
3	166626718	C	T	intergenic	*BCHE,ZBBX*	.	.	.
6	80847033	A	G	intronic	*BCKDHB*	.	.	.
6	80831220	A	G	intronic	*BCKDHB*	.	.	.
6	80833498	G	C	intronic	*BCKDHB*	.	.	.
6	80833663	A	G	intronic	*BCKDHB*	.	.	.
6	80833913	T	A	intronic	*BCKDHB*	.	.	.
6	81695365	G	T	intergenic	*BCKDHB,FAM46A*	.	.	.
6	81675996	T	C	intergenic	*BCKDHB,FAM46A*	.	.	.
6	81398222	A	AAAAC	intergenic	*BCKDHB,FAM46A*	.	.	.
6	81741840	G	A	intergenic	*BCKDHB,FAM46A*	.	.	.
6	81744294	C	A	intergenic	*BCKDHB,FAM46A*	.	.	.
6	81116025	T	C	intergenic	*BCKDHB,FAM46A*	.	.	.
6	81118268	A	T	intergenic	*BCKDHB,FAM46A*	.	.	.
6	81139134	A	T	intergenic	*BCKDHB,FAM46A*	.	.	.
6	81139448	A	G	intergenic	*BCKDHB,FAM46A*	.	.	.
6	81325823	T	C	intergenic	*BCKDHB,FAM46A*	.	.	.
6	81326249	C	A	intergenic	*BCKDHB,FAM46A*	.	.	.
6	81373160	G	T	intergenic	*BCKDHB,FAM46A*	.	.	.
6	81395086	A	C	intergenic	*BCKDHB,FAM46A*	.	.	.

6	81456889	A	G	intergenic	*BCKDHB,FAM46A*	.	.	.
6	81511141	A	G	intergenic	*BCKDHB,FAM46A*	.	.	.
6	81533854	A	G	intergenic	*BCKDHB,FAM46A*	.	.	.
6	81561093	A	G	intergenic	*BCKDHB,FAM46A*	.	.	.
6	81622286	T	C	intergenic	*BCKDHB,FAM46A*	.	.	.
6	81724652	A	G	intergenic	*BCKDHB,FAM46A*	.	.	.
6	81756940	C	A	intergenic	*BCKDHB,FAM46A*	.	.	.
6	81823487	T	C	intergenic	*BCKDHB,FAM46A*	.	.	.
6	81825129	C	G	intergenic	*BCKDHB,FAM46A*	.	.	.
6	81845045	G	A	intergenic	*BCKDHB,FAM46A*	.	.	.
6	81845047	A	C	intergenic	*BCKDHB,FAM46A*	.	.	.
6	81918944	G	A	intergenic	*BCKDHB,FAM46A*	.	.	.
6	81953219	C	T	intergenic	*BCKDHB,FAM46A*	.	.	.
6	81966510	G	C	intergenic	*BCKDHB,FAM46A*	.	.	.
6	81980216	T	A	intergenic	*BCKDHB,FAM46A*	.	.	.
6	82109448	T	C	intergenic	*BCKDHB,FAM46A*	.	.	.
6	82141954	C	T	intergenic	*BCKDHB,FAM46A*	.	.	.
6	82146427	C	T	intergenic	*BCKDHB,FAM46A*	.	.	.
6	82171771	A	T	intergenic	*BCKDHB,FAM46A*	.	.	.
6	82218066	C	T	intergenic	*BCKDHB,FAM46A*	.	.	.
6	82224198	C	T	intergenic	*BCKDHB,FAM46A*	.	.	.
6	82269615	T	C	intergenic	*BCKDHB,FAM46A*	.	.	.
6	82291044	G	A	intergenic	*BCKDHB,FAM46A*	.	.	.
6	82291513	T	C	intergenic	*BCKDHB,FAM46A*	.	.	.
6	82293587	G	A	intergenic	*BCKDHB,FAM46A*	.	.	.
6	82294346	T	C	intergenic	*BCKDHB,FAM46A*	.	.	.
6	82421878	G	A	intergenic	*BCKDHB,FAM46A*	.	.	.
16	31120474	G	A	UTR5	*BCKDK*	.	.	.
2	60818909	A	G	intergenic	*BCL11A,PAPOLG*	.	.	.
14	99664630	T	C	intronic	*BCL11B*	.	.	.
14	99640686	G	A	exonic	*BCL11B*	synonymous_SNV	.	.
2	112069912	C	T	intergenic	*BCL2L11,MIR4435-1*	.	.	.
3	187571189	A	C	intergenic	*BCL6,LPP-AS2*	.	.	.
12	122503921	G	A	intergenic	*BCL7A,MLXIP*	.	.	.
11	112052901	G	A	intronic	*BCO2*	.	.	.
X	39922693	G	A	intronic	*BCOR*	.	.	.
X	40029659	A	G	intronic	*BCOR*	.	.	.

X	39994755	A	G	intronic	*BCOR*	.	.	.
X	129174922	G	A	intronic	*BCORL1*	.	.	.
22	23673758	C	G	intergenic	*BCR,CES5AP1*	.	.	.
X	70804823	T	A	ncRNA_intronic	*BCYRN1*	.	.	.
X	70496933	A	C	ncRNA_intronic	*BCYRN1*	.	.	.
X	70662652	A	G	ncRNA_intronic	*BCYRN1*	.	.	.
X	70663103	C	G	ncRNA_intronic	*BCYRN1*	.	.	.
14	96715286	C	A	intergenic	*BDKRB2,BDKRB1*	.	.	.
11	27589167	TTCTATATTTATATCTAGTATCTAGATATATCTATATTTATATCTAGTATCTAGATATA	T	ncRNA_intronic	*BDNF-AS*	.	.	.
11	27616786	T	C	ncRNA_intronic	*BDNF-AS*	.	.	.
11	27690823	C	T	ncRNA_intronic	*BDNF-AS*	.	.	.
11	27690825	C	T	ncRNA_intronic	*BDNF-AS*	.	.	.
11	27691781	T	C	ncRNA_intronic	*BDNF-AS*	.	.	.
11	27962439	G	T	intergenic	*BDNF,KIF18A*	.	.	.
X	18241888	C	A	intergenic	*BEND2,SCML2*	.	.	.
X	18251866	T	A	intergenic	*BEND2,SCML2*	.	.	.
6	107430247	A	T	intronic	*BEND3*	.	.	.
4	42144096	ATT	A	intronic	*BEND4*	.	.	.
4	42151208	A	C	intronic	*BEND4*	.	.	.
4	42326584	G	A	intergenic	*BEND4,SHISA3*	.	.	.
6	56899015	C	CATAGAT	intergenic	*BEND6,KIAA1586*	.	.	.
7	93680148	C	T	intergenic	*BET1,COL1A2*	.	.	.
7	93848505	T	A	intergenic	*BET1,COL1A2*	.	.	.
7	93850854	T	C	intergenic	*BET1,COL1A2*	.	.	.
X	102495268	G	C	intergenic	*BEX4,TCEAL8*	.	.	.
X	101442301	C	T	intergenic	*BEX5,TCP11X2*	.	.	.
X	101442715	C	G	intergenic	*BEX5,TCP11X2*	.	.	.
X	101449740	G	T	intergenic	*BEX5,TCP11X2*	.	.	.
3	4954456	T	C	ncRNA_intronic	*BHLHE40-AS1*	.	.	.
3	5012960	A	G	ncRNA_intronic	*BHLHE40-AS1*	.	.	.
3	5094585	GTGGTGT	G	intergenic	*BHLHE40,ARL8B*	.	.	.

5	78463055	C	T	intergenic	*BHMT,JMY*	.	.	.
10	60331964	C	A	intronic	*BICC1*	.	.	.
10	60354420	T	C	intronic	*BICC1*	.	.	.
10	60354422	A	G	intronic	*BICC1*	.	.	.
10	60444006	T	G	intronic	*BICC1*	.	.	.
10	60453563	C	T	intronic	*BICC1*	.	.	.
10	60712679	G	C	intergenic	*BICC1,LINC00844*	.	.	.
10	60649565	A	C	intergenic	*BICC1,LINC00844*	.	.	.
12	32377489	G	A	intronic	*BICD1*	.	.	.
12	32310068	T	C	intronic	*BICD1*	.	.	.
12	32423369	C	G	intronic	*BICD1*	.	.	.
12	32587596	G	A	intergenic	*BICD1,FGD4*	.	.	.
12	32636103	G	T	intergenic	*BICD1,FGD4*	.	.	.
12	32577378	T	C	intergenic	*BICD1,FGD4*	.	.	.
12	32578800	C	CTCTGTTTG	intergenic	*BICD1,FGD4*	.	.	.
12	32580488	A	G	intergenic	*BICD1,FGD4*	.	.	.
11	102243318	C	G	intronic	*BIRC2*	.	.	.
11	102210869	T	C	downstream	*BIRC3*	.	.	.
2	32800030	G	T	intronic	*BIRC6*	.	.	.
2	32739893	C	CTA	intronic	*BIRC6*	.	.	.
2	32761811	T	C	intronic	*BIRC6*	.	.	.
2	32762061	C	T	intronic	*BIRC6*	.	.	.
1	205430015	T	A	intergenic	*BLACAT1,CDK18*	.	.	.
20	36178046	C	A	intergenic	*BLCAP,LINC00489*	.	.	.
20	36231828	T	TGTTTCTTC	intergenic	*BLCAP,LINC00489*	.	.	.
20	36183201	G	A	intergenic	*BLCAP,LINC00489*	.	.	.

4	6747243	TTGCAGTGAGCTGAGATCACGCCACTGCACTCCAGCCTGGGTGACAGAAAGAGGATCTTTTTTTTTTTTTTTTTGAGACGGAGTCTCGCTCTGTCGCCCAGGCTGGAG	T	intergenic	*BLOC1S4,KIAA0232*	.	.	.
6	7929294	T	C	ncRNA_intronic	*BLOC1S5-TXNDC5*	.	.	.
6	7930854	G	A	ncRNA_intronic	*BLOC1S5-TXNDC5*	.	.	.
6	7947200	G	T	ncRNA_intronic	*BLOC1S5-TXNDC5*	.	.	.
6	8032748	G	A	ncRNA_intronic	*BLOC1S5-TXNDC5,EEF1E1-BLOC1S5*	.	.	.
15	45890197	A	G	intronic	*BLOC1S6*	.	.	.
15	45915930	T	A	intergenic	*BLOC1S6,SQRDL*	.	.	.
15	45916690	T	A	intergenic	*BLOC1S6,SQRDL*	.	.	.
7	43874779	A	C	intergenic	*BLVRA,URGCP-MRPS24*	.	.	.
19	40960224	C	T	intronic	*BLVRB*	.	.	.
15	40433114	A	C	intergenic	*BMF,BUB1B*	.	.	.
2	69157361	G	A	intergenic	*BMP10,GKN2*	.	.	.
X	50742049	T	C	intergenic	*BMP15,LINC01284*	.	.	.
X	50742050	G	A	intergenic	*BMP15,LINC01284*	.	.	.
X	50753140	C	G	intergenic	*BMP15,LINC01284*	.	.	.
20	6760617	G	T	UTR3	*BMP2*	.	.	.
20	7000603	T	C	intergenic	*BMP2,LINC01428*	.	.	.
20	7023276	G	T	intergenic	*BMP2,LINC01428*	.	.	.
20	7059860	G	GT	intergenic	*BMP2,LINC01428*	.	.	.
20	7047740	G	GAT	intergenic	*BMP2,LINC01428*	.	.	.
20	6961619	C	T	intergenic	*BMP2,LINC01428*	.	.	.
4	79716677	A	C	intronic	*BMP2K*	.	.	.

4	79750941	T	TTTCTTTTCT	intronic	*BMP2K*	.	.	.
14	54590418	TTCTCTC	T	intergenic	*BMP4,CDKN3*	.	.	.
14	54701531	G	C	intergenic	*BMP4,CDKN3*	.	.	.
14	54608609	A	G	intergenic	*BMP4,CDKN3*	.	.	.
14	54471637	A	G	intergenic	*BMP4,CDKN3*	.	.	.
14	54496136	A	G	intergenic	*BMP4,CDKN3*	.	.	.
14	54540495	C	T	intergenic	*BMP4,CDKN3*	.	.	.
14	54818494	G	A	intergenic	*BMP4,CDKN3*	.	.	.
6	7860766	C	T	intronic	*BMP6*	.	.	.
20	55882982	G	A	intergenic	*BMP7,MIR4325*	.	.	.
7	34003202	T	A	intronic	*BMPER*	.	.	.
7	34030857	G	C	intronic	*BMPER*	.	.	.
7	34055621	TGAGA	T	intronic	*BMPER*	.	.	.
7	34256534	TGAGA	T	intergenic	*BMPER,NPSR1-AS1*	.	.	.
7	34211854	C	A	intergenic	*BMPER,NPSR1-AS1*	.	.	.
7	34212583	A	T	intergenic	*BMPER,NPSR1-AS1*	.	.	.
7	34271946	T	A	intergenic	*BMPER,NPSR1-AS1*	.	.	.
10	88606800	T	C	intronic	*BMPR1A*	.	.	.
4	95874708	A	ATTCTT	intronic	*BMPR1B*	.	.	.
4	95873875	G	A	intronic	*BMPR1B*	.	.	.
4	95873876	G	A	intronic	*BMPR1B*	.	.	.
4	95950610	C	G	intronic	*BMPR1B*	.	.	.
4	96056770	A	T	intronic	*BMPR1B*	.	.	.
4	96058913	C	G	intronic	*BMPR1B*	.	.	.
4	95664531	A	G	downstream	*BMPR1B-AS1*	.	.	.
4	95664532	T	C	downstream	*BMPR1B-AS1*	.	.	.
2	203498174	G	A	intergenic	*BMPR2,FAM117B*	.	.	.
10	43321645	G	A	intronic	*BMS1*	.	.	.
10	43321646	C	T	intronic	*BMS1*	.	.	.
22	22657155	C	T	ncRNA_intronic	*BMS1P20*	.	.	.
22	22657282	G	A	ncRNA_intronic	*BMS1P20*	.	.	.
22	22730788	G	A	intergenic	*BMS1P20,ZNF280B*	.	.	.

22	22689681	T	C	intergenic	*BMS1P20,ZNF280B*	.	.	.
10	75462491	A	C	ncRNA_intronic	*BMS1P4*	.	.	.
10	75484658	A	T	ncRNA_intronic	*BMS1P4*	.	.	.
10	48934177	G	A	ncRNA_intronic	*BMS1P5*	.	.	.
10	46799084	AG	A	intergenic	*BMS1P5,FAM35BP*	.	.	.
10	46799086	A	T	intergenic	*BMS1P5,FAM35BP*	.	.	.
10	47350979	C	T	intergenic	*BMS1P6,FAM35DP*	.	.	.
10	47376972	T	C	intergenic	*BMS1P6,FAM35DP*	.	.	.
15	84020487	G	T	intergenic	*BNC1,SH3GL3*	.	.	.
9	16601658	G	C	intronic	*BNC2*	.	.	.
9	16641373	T	A	intronic	*BNC2*	.	.	.
9	17058484	C	G	intergenic	*BNC2,CNTLN*	.	.	.
9	17077003	G	A	intergenic	*BNC2,CNTLN*	.	.	.
5	172607372	A	G	intergenic	*BNIP1,NKX2-5*	.	.	.
15	60108688	T	C	intergenic	*BNIP2,FOXB1*	.	.	.
15	60073661	C	T	intergenic	*BNIP2,FOXB1*	.	.	.
10	133814073	A	C	intergenic	*BNIP3,JAKMIP3*	.	.	.
10	133823160	C	T	intergenic	*BNIP3,JAKMIP3*	.	.	.
10	133901929	C	T	intergenic	*BNIP3,JAKMIP3*	.	.	.
10	133904674	G	A	intergenic	*BNIP3,JAKMIP3*	.	.	.
10	133904675	T	C	intergenic	*BNIP3,JAKMIP3*	.	.	.
5	173102346	T	A	intergenic	*BOD1,LINC01484*	.	.	.
5	173102348	A	ATCG	intergenic	*BOD1,LINC01484*	.	.	.
18	54891927	T	TTTTTG	intergenic	*BOD1L2,ST8SIA3*	.	.	.
18	54896031	G	A	intergenic	*BOD1L2,ST8SIA3*	.	.	.
18	54981301	A	T	intergenic	*BOD1L2,ST8SIA3*	.	.	.
2	198606406	C	A	intronic	*BOLL*	.	.	.
2	198615701	T	C	intronic	*BOLL*	.	.	.
2	198615702	G	T	intronic	*BOLL*	.	.	.
12	12572710	C	T	intronic	*BORCS5*	.	.	.
12	12573333	G	A	intronic	*BORCS5*	.	.	.
3	138830711	C	T	ncRNA_intronic	*BPESC1*	.	.	.
7	134434285	G	A	intergenic	*BPGM,CALD1*	.	.	.

20	31821207	G	C	intergenic	*BPIFA3,BPIFA1*	.	.	.
20	31600671	TGCGCCTGCTGGCAGCAGCTAATTTTACTTTCAA	T	exonic	*BPIFB2*	nonframeshift_deletion	.	.
20	31705466	C	T	intergenic	*BPIFB4,BPIFA2*	.	.	.
20	31705467	C	G	intergenic	*BPIFB4,BPIFA2*	.	.	.
22	32853546	GTCCATCCATCCATCCA	G	upstream	*BPIFC*	.	.	.
7	140462203	C	A	intronic	*BRAF*	.	.	.
7	140462205	C	T	intronic	*BRAF*	.	.	.
7	140466025	A	G	intronic	*BRAF*	.	.	.
7	140677782	C	T	intergenic	*BRAF,MRPS33*	.	.	.
7	140704782	C	T	intergenic	*BRAF,MRPS33*	.	.	.
7	2590152	T	A	intronic	*BRAT1*	.	.	.
13	32949971	A	G	intronic	*BRCA2*	.	.	.
5	44679820	G	T	intergenic	*BRCAT107,BRCAT54*	.	.	.
5	44579813	G	C	intergenic	*BRCAT107,BRCAT54*	.	.	.
5	44625955	T	A	intergenic	*BRCAT107,BRCAT54*	.	.	.
5	44671844	T	C	intergenic	*BRCAT107,BRCAT54*	.	.	.
5	44671871	C	A	intergenic	*BRCAT107,BRCAT54*	.	.	.
5	44672915	T	C	intergenic	*BRCAT107,BRCAT54*	.	.	.
5	44748972	GT	G	ncRNA_intronic	*BRCAT54*	.	.	.
5	44771332	G	T	ncRNA_intronic	*BRCAT54*	.	.	.
5	44758154	G	A	ncRNA_intronic	*BRCAT54*	.	.	.
5	44758438	T	C	ncRNA_intronic	*BRCAT54*	.	.	.
X	154388915	T	C	intergenic	*BRCC3,VBP1*	.	.	.
X	154411533	A	G	intergenic	*BRCC3,VBP1*	.	.	.
X	154411534	G	A	intergenic	*BRCC3,VBP1*	.	.	.
X	154413358	G	A	intergenic	*BRCC3,VBP1*	.	.	.

16	50382184	G	A	intronic	*BRD7*	.	.	.
16	50382329	C	A	intronic	*BRD7*	.	.	.
X	95601422	A	T	intergenic	*BRDTP1,DIAPH2*	.	.	.
X	95601908	G	T	intergenic	*BRDTP1,DIAPH2*	.	.	.
X	95603914	A	G	intergenic	*BRDTP1,DIAPH2*	.	.	.
X	95616110	C	A	intergenic	*BRDTP1,DIAPH2*	.	.	.
X	95745845	A	C	intergenic	*BRDTP1,DIAPH2*	.	.	.
X	95746146	T	C	intergenic	*BRDTP1,DIAPH2*	.	.	.
X	95746750	T	C	intergenic	*BRDTP1,DIAPH2*	.	.	.
X	95784856	C	T	intergenic	*BRDTP1,DIAPH2*	.	.	.
X	95785228	C	A	intergenic	*BRDTP1,DIAPH2*	.	.	.
X	95844223	G	T	intergenic	*BRDTP1,DIAPH2*	.	.	.
X	95844324	A	C	intergenic	*BRDTP1,DIAPH2*	.	.	.
X	95844326	A	G	intergenic	*BRDTP1,DIAPH2*	.	.	.
X	95857175	A	G	intergenic	*BRDTP1,DIAPH2*	.	.	.
X	95858419	T	C	intergenic	*BRDTP1,DIAPH2*	.	.	.
X	95888295	TA	T	intergenic	*BRDTP1,DIAPH2*	.	.	.
2	28152166	A	AAGAACCCT	intronic	*BRE*	.	.	.
2	28152166	A	AAGAACCCT	intronic	*BRE*	.	.	.
2	28247087	C	T	intronic	*BRE*	.	.	.
2	28552555	A	T	intronic	*BRE*	.	.	.
2	28553542	G	A	intronic	*BRE*	.	.	.
2	28553544	C	A	intronic	*BRE*	.	.	.
9	122029640	C	G	intronic	*BRINP1*	.	.	.
9	122073752	A	G	intronic	*BRINP1*	.	.	.
9	122651840	GTCTC	G	intergenic	*BRINP1,LINC01613*	.	.	.
9	122669968	T	G	intergenic	*BRINP1,LINC01613*	.	.	.
9	122670287	G	T	intergenic	*BRINP1,LINC01613*	.	.	.
9	122280343	C	T	intergenic	*BRINP1,LINC01613*	.	.	.
9	122323535	T	C	intergenic	*BRINP1,LINC01613*	.	.	.
9	122404879	T	A	intergenic	*BRINP1,LINC01613*	.	.	.
1	177172360	A	G	intronic	*BRINP2*	.	.	.

1	177181088	G	T	intronic	*BRINP2*	.	.	.
1	177150202	C	T	intronic	*BRINP2*	.	.	.
1	177234410	C	T	intronic	*BRINP2*	.	.	.
1	177265955	T	C	intergenic	*BRINP2,LOC102724661*	.	.	.
1	190416429	GTATA	G	intronic	*BRINP3*	.	.	.
1	190189441	A	C	intronic	*BRINP3*	.	.	.
1	190223795	A	G	intronic	*BRINP3*	.	.	.
1	190277728	A	T	intronic	*BRINP3*	.	.	.
1	190412187	C	T	intronic	*BRINP3*	.	.	.
17	59776266	C	T	intronic	*BRIP1*	.	.	.
17	59776267	A	G	intronic	*BRIP1*	.	.	.
17	59903491	A	G	intronic	*BRIP1*	.	.	.
17	59903492	C	A	intronic	*BRIP1*	.	.	.
17	59903635	A	AC	intronic	*BRIP1*	.	.	.
17	59903637	A	C	intronic	*BRIP1*	.	.	.
3	10175927	A	AAAAAAAAA	intergenic	*BRK1,VHL*	.	.	.
14	36308869	G	C	intronic	*BRMS1L*	.	.	.
14	36309867	A	G	intronic	*BRMS1L*	.	.	.
14	36446612	C	T	intergenic	*BRMS1L,LINC00609*	.	.	.
14	36391984	G	T	intergenic	*BRMS1L,LINC00609*	.	.	.
14	36441526	TCCCCCCTCCTCCTCCCTC	T	intergenic	*BRMS1L,LINC00609*	.	.	.
X	80024263	T	C	intronic	*BRWD3*	.	.	.
X	80279521	A	T	intergenic	*BRWD3,HMGN5*	.	.	.
X	80334775	A	G	intergenic	*BRWD3,HMGN5*	.	.	.
X	80079075	G	A	intergenic	*BRWD3,HMGN5*	.	.	.
X	80113777	G	T	intergenic	*BRWD3,HMGN5*	.	.	.
X	80113953	T	C	intergenic	*BRWD3,HMGN5*	.	.	.
X	80144950	T	C	intergenic	*BRWD3,HMGN5*	.	.	.
X	80164665	A	C	intergenic	*BRWD3,HMGN5*	.	.	.
X	80188248	C	A	intergenic	*BRWD3,HMGN5*	.	.	.
X	80188250	C	A	intergenic	*BRWD3,HMGN5*	.	.	.
X	80195588	G	A	intergenic	*BRWD3,HMGN5*	.	.	.
X	80235388	G	A	intergenic	*BRWD3,HMGN5*	.	.	.

X	80235685	T	G	intergenic	*BRWD3,HMGN5*	.	.	.
X	80236973	G	A	intergenic	*BRWD3,HMGN5*	.	.	.
X	80284441	C	T	intergenic	*BRWD3,HMGN5*	.	.	.
X	80286170	A	T	intergenic	*BRWD3,HMGN5*	.	.	.
X	80286172	T	C	intergenic	*BRWD3,HMGN5*	.	.	.
X	80294061	A	G	intergenic	*BRWD3,HMGN5*	.	.	.
X	80294122	C	T	intergenic	*BRWD3,HMGN5*	.	.	.
X	80321533	G	A	intergenic	*BRWD3,HMGN5*	.	.	.
X	80321796	A	G	intergenic	*BRWD3,HMGN5*	.	.	.
X	80325170	A	C	intergenic	*BRWD3,HMGN5*	.	.	.
X	80325481	T	G	intergenic	*BRWD3,HMGN5*	.	.	.
X	80341638	T	C	intergenic	*BRWD3,HMGN5*	.	.	.
11	13417834	C	T	intronic	*BTBD10*	.	.	.
11	13418493	A	T	intronic	*BTBD10*	.	.	.
11	13419128	C	T	intronic	*BTBD10*	.	.	.
11	13506018	C	T	intergenic	*BTBD10,PTH*	.	.	.
11	13506019	T	C	intergenic	*BTBD10,PTH*	.	.	.
12	107716707	T	C	intronic	*BTBD11*	.	.	.
20	11936182	C	G	intergenic	*BTBD3,LOC101929486*	.	.	.
20	12251964	T	C	intergenic	*BTBD3,LOC101929486*	.	.	.
20	11961963	TGC	T	intergenic	*BTBD3,LOC101929486*	.	.	.
20	11955235	C	A	intergenic	*BTBD3,LOC101929486*	.	.	.
20	12172812	C	T	intergenic	*BTBD3,LOC101929486*	.	.	.
20	12292674	T	C	intergenic	*BTBD3,LOC101929486*	.	.	.
20	12466269	GTCTGTCTGTCTGTCTATCTATCTATCTA	G	intergenic	*BTBD3,LOC101929486*	.	.	.
20	11983891	C	A	intergenic	*BTBD3,LOC101929486*	.	.	.
20	12798422	T	G	intergenic	*BTBD3,LOC101929486*	.	.	.

20	12739538	A	T	intergenic	*BTBD3,LOC101929486*	.	.	.
20	11934667	G	A	intergenic	*BTBD3,LOC101929486*	.	.	.
20	12106999	C	T	intergenic	*BTBD3,LOC101929486*	.	.	.
20	12254777	G	A	intergenic	*BTBD3,LOC101929486*	.	.	.
20	12289690	C	T	intergenic	*BTBD3,LOC101929486*	.	.	.
20	12404532	G	A	intergenic	*BTBD3,LOC101929486*	.	.	.
20	12406390	G	C	intergenic	*BTBD3,LOC101929486*	.	.	.
20	12423826	C	A	intergenic	*BTBD3,LOC101929486*	.	.	.
20	12423827	T	A	intergenic	*BTBD3,LOC101929486*	.	.	.
20	12460219	G	C	intergenic	*BTBD3,LOC101929486*	.	.	.
20	12460325	T	C	intergenic	*BTBD3,LOC101929486*	.	.	.
20	12460691	A	G	intergenic	*BTBD3,LOC101929486*	.	.	.
20	12474333	T	C	intergenic	*BTBD3,LOC101929486*	.	.	.
20	12567771	A	C	intergenic	*BTBD3,LOC101929486*	.	.	.
20	12645811	C	A	intergenic	*BTBD3,LOC101929486*	.	.	.
20	12750471	A	C	intergenic	*BTBD3,LOC101929486*	.	.	.
20	12750472	C	T	intergenic	*BTBD3,LOC101929486*	.	.	.
20	12799748	T	C	intergenic	*BTBD3,LOC101929486*	.	.	.
6	38431046	C	A	intronic	*BTBD9*	.	.	.
6	38513351	C	G	intronic	*BTBD9*	.	.	.

6	38261045	T	C	intronic	*BTBD9*	.	.	.
6	38261338	C	T	intronic	*BTBD9*	.	.	.
6	38261693	T	C	intronic	*BTBD9*	.	.	.
6	38261694	C	T	intronic	*BTBD9*	.	.	.
6	38277378	G	A	intronic	*BTBD9*	.	.	.
6	38393006	T	C	intronic	*BTBD9*	.	.	.
6	38393007	G	A	intronic	*BTBD9*	.	.	.
4	75805639	CATAT	C	intergenic	*BTC,PARM1*	.	.	.
4	75753439	A	C	intergenic	*BTC,PARM1*	.	.	.
4	75755591	G	A	intergenic	*BTC,PARM1*	.	.	.
1	52553084	T	C	UTR3	*BTF3L4*	.	.	.
1	52596658	T	C	intergenic	*BTF3L4,ZFYVE9*	.	.	.
13	77518208	C	T	intergenic	*BTF3P11,IRG1*	.	.	.
12	92717604	G	T	intergenic	*BTG1,CLLU1OS*	.	.	.
12	92557788	C	T	intergenic	*BTG1,CLLU1OS*	.	.	.
21	18973300	T	A	intronic	*BTG3*	.	.	.
21	19034927	C	T	intergenic	*BTG3,C21orf91-OT1*	.	.	.
21	19025106	C	T	intergenic	*BTG3,C21orf91-OT1*	.	.	.
21	19057461	CAA	C	intergenic	*BTG3,C21orf91-OT1*	.	.	.
21	19144870	C	A	intergenic	*BTG3,C21orf91-OT1*	.	.	.
21	19146677	T	C	intergenic	*BTG3,C21orf91-OT1*	.	.	.
1	228709513	A	G	intergenic	*BTNL10,MIR7641-2*	.	.	.
1	228711036	A	G	intergenic	*BTNL10,MIR7641-2*	.	.	.
1	228712195	G	A	intergenic	*BTNL10,MIR7641-2*	.	.	.
5	180439051	G	A	intergenic	*BTNL3,BTNL9*	.	.	.
5	180443725	G	A	intergenic	*BTNL3,BTNL9*	.	.	.
5	180443727	C	A	intergenic	*BTNL3,BTNL9*	.	.	.
5	180343988	C	T	intronic	*BTNL8*	.	.	.
5	180343989	A	G	intronic	*BTNL8*	.	.	.
5	180333007	C	G	intronic	*BTNL8*	.	.	.

5	180334169	G	A	intronic	*BTNL8*	.	.	.
5	180358209	G	A	intronic	*BTNL8*	.	.	.
5	180358554	C	T	intronic	*BTNL8*	.	.	.
10	103134494	G	C	intronic	*BTRC*	.	.	.
2	111449331	C	A	intergenic	*BUB1,ACOXL*	.	.	.
10	125072003	C	G	intergenic	*BUB3,GPR26*	.	.	.
6	41895884	T	C	intronic	*BYSL*	.	.	.
17	56419325	T	A	ncRNA_intronic	*BZRAP1-AS1*	.	.	.
10	63472665	GC	G	intronic	*C10orf107*	.	.	.
10	63473013	C	T	intronic	*C10orf107*	.	.	.
10	63484901	G	A	intronic	*C10orf107*	.	.	.
10	63496303	T	C	intronic	*C10orf107*	.	.	.
10	63496304	A	C	intronic	*C10orf107*	.	.	.
10	63568848	G	T	intergenic	*C10orf107,ARID5B*	.	.	.
10	63609634	G	A	intergenic	*C10orf107,ARID5B*	.	.	.
10	77561735	T	C	intronic	*C10orf11*	.	.	.
10	77800328	TTCTC	T	intronic	*C10orf11*	.	.	.
10	77558224	A	G	intronic	*C10orf11*	.	.	.
10	77655422	T	C	intronic	*C10orf11*	.	.	.
10	77725632	TGGGTTTATACACATTATAG	T	intronic	*C10orf11*	.	.	.
10	77435810	T	C	intronic	*C10orf11*	.	.	.
10	77751991	A	G	intronic	*C10orf11*	.	.	.
10	78227574	G	A	intronic	*C10orf11*	.	.	.
10	77560273	C	G	intronic	*C10orf11*	.	.	.
10	77560274	A	C	intronic	*C10orf11*	.	.	.
10	77614689	A	T	intronic	*C10orf11*	.	.	.
10	77616726	G	A	intronic	*C10orf11*	.	.	.
10	77927697	T	G	intronic	*C10orf11*	.	.	.
10	77981365	T	G	intronic	*C10orf11*	.	.	.
10	77981366	T	G	intronic	*C10orf11*	.	.	.
10	78186935	T	C	intronic	*C10orf11*	.	.	.
10	78186937	A	G	intronic	*C10orf11*	.	.	.
10	78474516	G	T	intergenic	*C10orf11,KCNMA1*	.	.	.
10	78424272	T	C	intergenic	*C10orf11,KCNMA1*	.	.	.
10	78374397	G	C	intergenic	*C10orf11,KCNMA1*	.	.	.
10	78374398	C	A	intergenic	*C10orf11,KCNMA1*	.	.	.

10	78542870	A	T	intergenic	*C10orf11,KCNMA1*	.	.	.
10	78582865	T	C	intergenic	*C10orf11,KCNMA1*	.	.	.
10	78609024	T	C	intergenic	*C10orf11,KCNMA1*	.	.	.
10	78611140	C	A	intergenic	*C10orf11,KCNMA1*	.	.	.
10	21435434	C	G	exonic	*C10orf113*	nonsynonymous_SNV	.	.
10	29355168	T	C	intergenic	*C10orf126,LYZL1*	.	.	.
10	44816450	G	T	intergenic	*C10orf142,CXCL12*	.	.	.
10	71414307	G	A	intergenic	*C10orf35,COL13A1*	.	.	.
10	71458220	T	C	intergenic	*C10orf35,COL13A1*	.	.	.
10	71458221	G	A	intergenic	*C10orf35,COL13A1*	.	.	.
10	75703909	A	T	intergenic	*C10orf55,VCL*	.	.	.
10	103785043	A	T	intronic	*C10orf76*	.	.	.
10	103796031	G	A	intronic	*C10orf76*	.	.	.
10	103700256	C	T	intronic	*C10orf76*	.	.	.
10	128223467	C	T	intergenic	*C10orf90,DOCK1*	.	.	.
10	128543249	G	A	intergenic	*C10orf90,DOCK1*	.	.	.
10	128279939	T	G	intergenic	*C10orf90,DOCK1*	.	.	.
10	128453608	T	C	intergenic	*C10orf90,DOCK1*	.	.	.
10	128453609	T	C	intergenic	*C10orf90,DOCK1*	.	.	.
10	128575706	C	T	intergenic	*C10orf90,DOCK1*	.	.	.
10	128576255	T	A	intergenic	*C10orf90,DOCK1*	.	.	.
11	130639304	C	CAGGGTG	intergenic	*C11orf44,LOC100507431*	.	.	.
11	47015648	G	A	intronic	*C11orf49*	.	.	.
11	47017107	A	G	intronic	*C11orf49*	.	.	.
11	47132690	G	A	intronic	*C11orf49*	.	.	.
11	108296092	C	A	intronic	*C11orf65*	.	.	.
11	108313166	A	G	intronic	*C11orf65*	.	.	.
11	108313167	A	G	intronic	*C11orf65*	.	.	.
11	108314060	G	T	intronic	*C11orf65*	.	.	.
11	108321192	A	T	intronic	*C11orf65*	.	.	.
11	108322377	G	A	intronic	*C11orf65*	.	.	.

11	101962837	CATATATATATATATATACACACATACATATATATGTATGTGTGTAT	C	intergenic	*C11orf70,YAP1*	.	.	.
11	86023431	C	T	intronic	*C11orf73*	.	.	.
11	86051452	T	C	intronic	*C11orf73*	.	.	.
11	86077413	G	A	intergenic	*C11orf73,CCDC81*	.	.	.
11	86077843	A	G	intergenic	*C11orf73,CCDC81*	.	.	.
11	36676700	G	A	intronic	*C11orf74*	.	.	.
11	36623307	G	A	intronic	*C11orf74*	.	.	.
11	36664424	G	A	intronic	*C11orf74*	.	.	.
11	36665421	A	G	intronic	*C11orf74*	.	.	.
11	38203070	G	GA	intergenic	*C11orf74,LOC103312105*	.	.	.
11	36759190	A	ATT	intergenic	*C11orf74,LOC103312105*	.	.	.
11	36955482	G	A	intergenic	*C11orf74,LOC103312105*	.	.	.
11	36765928	AC	A	intergenic	*C11orf74,LOC103312105*	.	.	.
11	38402311	A	G	intergenic	*C11orf74,LOC103312105*	.	.	.
11	38441180	A	C	intergenic	*C11orf74,LOC103312105*	.	.	.
11	36882030	T	C	intergenic	*C11orf74,LOC103312105*	.	.	.
11	38323076	G	T	intergenic	*C11orf74,LOC103312105*	.	.	.
11	37069191	AATAT	A	intergenic	*C11orf74,LOC103312105*	.	.	.
11	36708469	A	T	intergenic	*C11orf74,LOC103312105*	.	.	.
11	37050546	A	G	intergenic	*C11orf74,LOC103312105*	.	.	.
11	38287022	T	A	intergenic	*C11orf74,LOC103312105*	.	.	.

11	38621801	T	A	intergenic	*C11orf74,LOC1033 12105*	.	.	.
11	37727426	C	A	intergenic	*C11orf74,LOC1033 12105*	.	.	.
11	37898627	G	T	intergenic	*C11orf74,LOC1033 12105*	.	.	.
11	38082980	G	T	intergenic	*C11orf74,LOC1033 12105*	.	.	.
11	38510437	C	A	intergenic	*C11orf74,LOC1033 12105*	.	.	.
11	36697162	A	G	intergenic	*C11orf74,LOC1033 12105*	.	.	.
11	36698596	A	C	intergenic	*C11orf74,LOC1033 12105*	.	.	.
11	36700437	A	C	intergenic	*C11orf74,LOC1033 12105*	.	.	.
11	36700664	C	A	intergenic	*C11orf74,LOC1033 12105*	.	.	.
11	37041282	T	A	intergenic	*C11orf74,LOC1033 12105*	.	.	.
11	37052607	A	G	intergenic	*C11orf74,LOC1033 12105*	.	.	.
11	37170242	C	A	intergenic	*C11orf74,LOC1033 12105*	.	.	.
11	37186656	A	ATTTGTGTG	intergenic	*C11orf74,LOC1033 12105*	.	.	.
11	37227867	A	G	intergenic	*C11orf74,LOC1033 12105*	.	.	.
11	37227868	G	C	intergenic	*C11orf74,LOC1033 12105*	.	.	.
11	37229560	A	G	intergenic	*C11orf74,LOC1033 12105*	.	.	.
11	37382609	G	A	intergenic	*C11orf74,LOC1033 12105*	.	.	.
11	37383585	A	C	intergenic	*C11orf74,LOC1033 12105*	.	.	.
11	37383586	G	A	intergenic	*C11orf74,LOC1033 12105*	.	.	.

11	37450801	G	A	intergenic	*C11orf74,LOC1033 12105*	.	.	.
11	37454351	T	A	intergenic	*C11orf74,LOC1033 12105*	.	.	.
11	37454560	C	T	intergenic	*C11orf74,LOC1033 12105*	.	.	.
11	37560061	A	G	intergenic	*C11orf74,LOC1033 12105*	.	.	.
11	37639873	T	G	intergenic	*C11orf74,LOC1033 12105*	.	.	.
11	37707557	A	G	intergenic	*C11orf74,LOC1033 12105*	.	.	.
11	37707558	T	C	intergenic	*C11orf74,LOC1033 12105*	.	.	.
11	37840779	G	A	intergenic	*C11orf74,LOC1033 12105*	.	.	.
11	37840781	A	T	intergenic	*C11orf74,LOC1033 12105*	.	.	.
11	38155576	CA	C	intergenic	*C11orf74,LOC1033 12105*	.	.	.
11	38253958	T	C	intergenic	*C11orf74,LOC1033 12105*	.	.	.
11	38439260	T	C	intergenic	*C11orf74,LOC1033 12105*	.	.	.
11	38439261	G	A	intergenic	*C11orf74,LOC1033 12105*	.	.	.
11	38493069	T	A	intergenic	*C11orf74,LOC1033 12105*	.	.	.
11	38511164	A	G	intergenic	*C11orf74,LOC1033 12105*	.	.	.
11	38595759	G	T	intergenic	*C11orf74,LOC1033 12105*	.	.	.
11	66546421	A	G	intronic	*C11orf80*	.	.	.
11	64720591	G	A	intronic	*C11orf85*	.	.	.
11	109337492	A	C	intergenic	*C11orf87,ZC3H12C*	.	.	.
11	109844776	C	T	intergenic	*C11orf87,ZC3H12C*	.	.	.

11	109765769	T	C	intergenic	*C11orf87,ZC3H12C*	.	.	.
11	109483383	A	G	intergenic	*C11orf87,ZC3H12C*	.	.	.
11	109846384	C	A	intergenic	*C11orf87,ZC3H12C*	.	.	.
11	109319760	G	A	intergenic	*C11orf87,ZC3H12C*	.	.	.
11	109320355	C	A	intergenic	*C11orf87,ZC3H12C*	.	.	.
11	109332324	G	A	intergenic	*C11orf87,ZC3H12C*	.	.	.
11	109339853	A	G	intergenic	*C11orf87,ZC3H12C*	.	.	.
11	109340402	A	G	intergenic	*C11orf87,ZC3H12C*	.	.	.
11	109341197	A	C	intergenic	*C11orf87,ZC3H12C*	.	.	.
11	109386367	G	A	intergenic	*C11orf87,ZC3H12C*	.	.	.
11	109400363	T	C	intergenic	*C11orf87,ZC3H12C*	.	.	.
11	109413636	G	A	intergenic	*C11orf87,ZC3H12C*	.	.	.
11	109444676	C	A	intergenic	*C11orf87,ZC3H12C*	.	.	.
11	109725206	A	G	intergenic	*C11orf87,ZC3H12C*	.	.	.
11	109725208	A	G	intergenic	*C11orf87,ZC3H12C*	.	.	.
11	111407055	C	CCT	intronic	*C11orf88*	.	.	.
12	40107153	T	C	intronic	*C12orf40*	.	.	.
12	40058404	T	G	intronic	*C12orf40*	.	.	.
12	40095868	G	A	intronic	*C12orf40*	.	.	.
12	40122773	G	A	intergenic	*C12orf40,SLC2A13*	.	.	.
12	40122962	T	C	intergenic	*C12orf40,SLC2A13*	.	.	.

12	40123017	A	T	intergenic	*C12orf40,SLC2A13*	.	.	.
12	40144122	A	T	intergenic	*C12orf40,SLC2A13*	.	.	.
12	40144605	A	C	intergenic	*C12orf40,SLC2A13*	.	.	.
12	103817650	G	A	intronic	*C12orf42*	.	.	.
12	103760427	G	A	intronic	*C12orf42*	.	.	.
12	103763641	A	G	intronic	*C12orf42*	.	.	.
12	103806724	T	G	intronic	*C12orf42*	.	.	.
12	103821237	A	G	intronic	*C12orf42*	.	.	.
12	103821238	G	A	intronic	*C12orf42*	.	.	.
12	103822021	A	G	intronic	*C12orf42*	.	.	.
12	103822129	G	T	intronic	*C12orf42*	.	.	.
12	103823147	T	A	intronic	*C12orf42*	.	.	.
12	103830464	T	C	intronic	*C12orf42*	.	.	.
12	103858736	A	G	intronic	*C12orf42*	.	.	.
12	103878411	A	C	intronic	*C12orf42*	.	.	.
12	103880477	A	G	intronic	*C12orf42*	.	.	.
12	103880515	C	T	intronic	*C12orf42*	.	.	.
12	103880933	A	T	intronic	*C12orf42*	.	.	.
12	103881443	G	A	intronic	*C12orf42*	.	.	.
12	88408518	C	A	intronic	*C12orf50*	.	.	.
12	88375599	T	G	intronic	*C12orf50*	.	.	.
12	88391533	A	G	intronic	*C12orf50*	.	.	.
12	64691565	C	T	intronic	*C12orf56*	.	.	.
12	64692828	C	T	intronic	*C12orf56*	.	.	.
12	27270756	G	T	intergenic	*C12orf71,STK38L*	.	.	.
12	27245928	T	A	intergenic	*C12orf71,STK38L*	.	.	.
12	27269102	T	G	intergenic	*C12orf71,STK38L*	.	.	.
12	27319757	T	G	intergenic	*C12orf71,STK38L*	.	.	.
12	27321441	T	C	intergenic	*C12orf71,STK38L*	.	.	.
12	27380235	C	T	intergenic	*C12orf71,STK38L*	.	.	.
12	105922236	G	A	intergenic	*C12orf75,CASC18*	.	.	.
12	106089803	C	T	intergenic	*C12orf75,CASC18*	.	.	.
12	106079170	G	T	intergenic	*C12orf75,CASC18*	.	.	.
12	105997125	G	A	intergenic	*C12orf75,CASC18*	.	.	.
12	25160601	GCC	G	intergenic	*C12orf77,LRMP*	.	.	.

14	57991551	T	G	intergenic	*C14orf105,SLC35F4*	.	.	.
14	57983146	T	C	intergenic	*C14orf105,SLC35F4*	.	.	.
14	57980539	C	A	intergenic	*C14orf105,SLC35F4*	.	.	.
14	58007101	G	T	intergenic	*C14orf105,SLC35F4*	.	.	.
14	58009598	C	G	intergenic	*C14orf105,SLC35F4*	.	.	.
14	58017935	A	G	intergenic	*C14orf105,SLC35F4*	.	.	.
14	23575410	CGGCCT	C	intergenic	*C14orf119,CEBPE*	.	.	.
14	96544963	GAGTTCTC	G	intronic	*C14orf132*	.	.	.
14	96567057	C	A	intergenic	*C14orf132,BDKRB2*	.	.	.
14	99196303	G	C	intergenic	*C14orf177,BCL11B*	.	.	.
14	99242741	A	T	intergenic	*C14orf177,BCL11B*	.	.	.
14	99242742	T	G	intergenic	*C14orf177,BCL11B*	.	.	.
14	99269883	A	T	intergenic	*C14orf177,BCL11B*	.	.	.
14	58508588	A	G	intronic	*C14orf37*	.	.	.
14	58509024	T	C	intronic	*C14orf37*	.	.	.
14	58509025	G	A	intronic	*C14orf37*	.	.	.
14	58511154	A	C	intronic	*C14orf37*	.	.	.
14	58582093	G	A	intronic	*C14orf37*	.	.	.
14	60912604	T	C	intronic	*C14orf39*	.	.	.
15	93079488	T	C	intergenic	*C15orf32,LINC00930*	.	.	.
15	45285671	G	T	intergenic	*C15orf43,SORD*	.	.	.
15	45285713	A	G	intergenic	*C15orf43,SORD*	.	.	.
15	45287621	T	C	intergenic	*C15orf43,SORD*	.	.	.
15	45287622	G	C	intergenic	*C15orf43,SORD*	.	.	.
15	39104752	GTTT	G	intergenic	*C15orf53,C15orf54*	.	.	.
15	39433238	TACAC	T	intergenic	*C15orf53,C15orf54*	.	.	.

15	39068112	G	A	intergenic	*C15orf53,C15orf54*	.	.	.
15	39092458	C	G	intergenic	*C15orf53,C15orf54*	.	.	.
15	39105763	T	A	intergenic	*C15orf53,C15orf54*	.	.	.
15	39105874	G	A	intergenic	*C15orf53,C15orf54*	.	.	.
15	39124145	G	GT	intergenic	*C15orf53,C15orf54*	.	.	.
15	39124529	C	CA	intergenic	*C15orf53,C15orf54*	.	.	.
15	39144780	C	T	intergenic	*C15orf53,C15orf54*	.	.	.
15	39329790	A	G	intergenic	*C15orf53,C15orf54*	.	.	.
15	39585002	A	T	intergenic	*C15orf54,THBS1*	.	.	.
16	31528716	T	C	intergenic	*C16orf58,AHSP*	.	.	.
16	19643117	A	G	intronic	*C16orf62*	.	.	.
16	19681011	A	T	intronic	*C16orf62*	.	.	.
16	19681012	G	T	intronic	*C16orf62*	.	.	.
16	9232465	G	A	intergenic	*C16orf72,MIR548X*	.	.	.
16	9279316	G	A	intergenic	*C16orf72,MIR548X*	.	.	.
16	9301013	G	GTATT	intergenic	*C16orf72,MIR548X*	.	.	.
16	27211761	A	G	intergenic	*C16orf82,KDM8*	.	.	.
16	52103879	G	C	intronic	*C16orf97*	.	.	.
17	41872161	G	A	intergenic	*C17orf105,MPP3*	.	.	.
17	51503165	A	G	intergenic	*C17orf112,KIF2B*	.	.	.
17	51479754	T	C	intergenic	*C17orf112,KIF2B*	.	.	.
17	51532506	C	G	intergenic	*C17orf112,KIF2B*	.	.	.
17	51227068	A	AT	intergenic	*C17orf112,KIF2B*	.	.	.
17	51286469	C	A	intergenic	*C17orf112,KIF2B*	.	.	.
17	51664400	A	C	intergenic	*C17orf112,KIF2B*	.	.	.
17	51387130	T	C	intergenic	*C17orf112,KIF2B*	.	.	.
17	51555552	GCTCT	G	intergenic	*C17orf112,KIF2B*	.	.	.
17	51578076	T	C	intergenic	*C17orf112,KIF2B*	.	.	.
17	51647829	G	A	intergenic	*C17orf112,KIF2B*	.	.	.
17	51689628	AACACACA CAC	A	intergenic	*C17orf112,KIF2B*	.	.	.
17	51888279	T	C	intergenic	*C17orf112,KIF2B*	.	.	.
17	51889151	G	T	intergenic	*C17orf112,KIF2B*	.	.	.
17	21518621	G	T	intergenic	*C17orf51,FAM27L*	.	.	.
17	21560967	A	ATATATGTG	intergenic	*C17orf51,FAM27L*	.	.	.
17	21556480	G	A	intergenic	*C17orf51,FAM27L*	.	.	.
17	21702182	A	T	intergenic	*C17orf51,FAM27L*	.	.	.
17	21740226	C	G	intergenic	*C17orf51,FAM27L*	.	.	.

17	21551185	C	T	intergenic	*C17orf51,FAM27L*	.	.	.
17	21472725	T	A	intergenic	*C17orf51,FAM27L*	.	.	.
17	21493453	T	A	intergenic	*C17orf51,FAM27L*	.	.	.
17	21525031	GC	G	intergenic	*C17orf51,FAM27L*	.	.	.
17	21796086	G	A	intergenic	*C17orf51,FAM27L*	.	.	.
17	36999881	G	C	intergenic	*C17orf98,RPL23*	.	.	.
18	43779480	G	A	intronic	*C18orf25*	.	.	.
18	43779481	T	C	intronic	*C18orf25*	.	.	.
18	52019536	G	T	intergenic	*C18orf54,DYNAP*	.	.	.
18	12205583	C	T	ncRNA_intronic	*C18orf61*	.	.	.
19	48690943	T	C	intronic	*C19orf68*	.	.	.
19	51156515	C	T	intronic	*C19orf81*	.	.	.
2	68345251	A	G	intergenic	*C1D,WDR92*	.	.	.
2	68296059	T	C	intergenic	*C1D,WDR92*	.	.	.
7	7236627	C	T	intronic	*C1GALT1*	.	.	.
7	7238221	G	A	intronic	*C1GALT1*	.	.	.
X	119786635	T	G	intergenic	*C1GALT1C1,CT47B1*	.	.	.
X	119784547	G	A	intergenic	*C1GALT1C1,CT47B1*	.	.	.
X	119948918	G	T	intergenic	*C1GALT1C1,CT47B1*	.	.	.
X	119954932	C	T	intergenic	*C1GALT1C1,CT47B1*	.	.	.
1	244654809	C	T	intronic	*C1orf101*	.	.	.
1	244663010	A	G	intronic	*C1orf101*	.	.	.
1	172425845	T	A	intronic	*C1orf105*	.	.	.
1	172425846	A	T	intronic	*C1orf105*	.	.	.
1	200885446	C	T	downstream	*C1orf106*	.	.	.
1	221603142	ATCTC	A	intergenic	*C1orf140,DUSP10*	.	.	.
1	221834202	A	G	intergenic	*C1orf140,DUSP10*	.	.	.
1	221730822	A	G	intergenic	*C1orf140,DUSP10*	.	.	.
1	221607712	A	AAAAAAAAA	intergenic	*C1orf140,DUSP10*	.	.	.
1	221557478	T	C	intergenic	*C1orf140,DUSP10*	.	.	.
1	221560106	G	A	intergenic	*C1orf140,DUSP10*	.	.	.
1	67607190	C	A	intergenic	*C1orf141,IL23R*	.	.	.
1	67616562	T	A	intergenic	*C1orf141,IL23R*	.	.	.
1	67620795	C	T	intergenic	*C1orf141,IL23R*	.	.	.

1	92701647	A	G	intronic	*C1orf146*	.	.	.
1	57199115	T	C	intronic	*C1orf168*	.	.	.
1	51571727	G	A	intronic	*C1orf185*	.	.	.
1	184517109	T	C	intronic	*C1orf21*	.	.	.
1	60706405	A	G	intergenic	*C1orf87,LOC101926964*	.	.	.
1	60790726	A	G	intergenic	*C1orf87,LOC101926964*	.	.	.
1	60791805	G	T	intergenic	*C1orf87,LOC101926964*	.	.	.
1	60845233	G	A	intergenic	*C1orf87,LOC101926964*	.	.	.
1	60847856	T	C	intergenic	*C1orf87,LOC101926964*	.	.	.
1	60847857	G	A	intergenic	*C1orf87,LOC101926964*	.	.	.
1	60931038	G	A	intergenic	*C1orf87,LOC101926964*	.	.	.
1	34845809	C	A	intergenic	*C1orf94,MIR552*	.	.	.
1	34745784	C	T	intergenic	*C1orf94,MIR552*	.	.	.
1	34955867	G	A	intergenic	*C1orf94,MIR552*	.	.	.
1	34957368	G	A	intergenic	*C1orf94,MIR552*	.	.	.
1	35021519	G	A	intergenic	*C1orf94,MIR552*	.	.	.
1	35021606	T	G	intergenic	*C1orf94,MIR552*	.	.	.
1	35021970	C	T	intergenic	*C1orf94,MIR552*	.	.	.
1	35050294	G	T	intergenic	*C1orf94,MIR552*	.	.	.
2	119947833	GTATA	G	intergenic	*C1QL2,STEAP3*	.	.	.
10	16600944	C	G	intergenic	*C1QL3,RSU1*	.	.	.
5	34102866	T	C	ncRNA_intronic	*C1QTNF3-AMACR*	.	.	.
5	34178188	A	G	intergenic	*C1QTNF3-AMACR,RAI14*	.	.	.
5	34189226	C	A	intergenic	*C1QTNF3-AMACR,RAI14*	.	.	.
5	34184491	G	A	intergenic	*C1QTNF3-AMACR,RAI14*	.	.	.
5	34184492	C	G	intergenic	*C1QTNF3-AMACR,RAI14*	.	.	.

5	34224060	C	T	intergenic	*C1QTNF3-AMACR,RAI14*	.	.	.
5	34319245	A	C	intergenic	*C1QTNF3-AMACR,RAI14*	.	.	.
5	34386116	T	C	intergenic	*C1QTNF3-AMACR,RAI14*	.	.	.
5	34431440	A	T	intergenic	*C1QTNF3-AMACR,RAI14*	.	.	.
5	34641964	G	A	intergenic	*C1QTNF3-AMACR,RAI14*	.	.	.
12	7274466	C	A	downstream	*C1RL-AS1*	.	.	.
6	31898602	G	A	intronic	*C2*	.	.	.
20	61143623	C	T	ncRNA_exonic	*C20orf166-AS1*	.	.	.
20	3280619	G	A	intronic	*C20orf194*	.	.	.
20	18847332	G	A	intergenic	*C20orf78,SLC24A3*	.	.	.
20	18843646	T	C	intergenic	*C20orf78,SLC24A3*	.	.	.
20	18848487	T	G	intergenic	*C20orf78,SLC24A3*	.	.	.
20	18916628	A	C	intergenic	*C20orf78,SLC24A3*	.	.	.
20	18920740	C	T	intergenic	*C20orf78,SLC24A3*	.	.	.
20	18920741	A	C	intergenic	*C20orf78,SLC24A3*	.	.	.
20	19144405	A	G	intergenic	*C20orf78,SLC24A3*	.	.	.
20	19144911	G	A	intergenic	*C20orf78,SLC24A3*	.	.	.
20	19146009	G	T	intergenic	*C20orf78,SLC24A3*	.	.	.
20	19146010	T	A	intergenic	*C20orf78,SLC24A3*	.	.	.
21	35799590	T	C	intergenic	*C21orf140,KCNE1*	.	.	.
21	33986329	T	C	intergenic	*C21orf59,SYNJ1*	.	.	.
21	34381532	G	T	intergenic	*C21orf62,OLIG2*	.	.	.
21	34207444	C	T	intergenic	*C21orf62,OLIG2*	.	.	.

22	50136414	T	C	intergenic	*C22orf34,BRD1*	.	.	.
22	50137641	T	C	intergenic	*C22orf34,BRD1*	.	.	.
11	73772379	C	T	intronic	*C2CD3*	.	.	.
11	73773284	T	C	intronic	*C2CD3*	.	.	.
11	73793079	A	G	intronic	*C2CD3*	.	.	.
11	73794068	C	T	intronic	*C2CD3*	.	.	.
15	62426891	C	T	intergenic	*C2CD4A,C2CD4B*	.	.	.
2	132636743	C	T	intergenic	*C2orf27B,ANKRD30BL*	.	.	.
2	132636744	A	G	intergenic	*C2orf27B,ANKRD30BL*	.	.	.
2	132818748	C	G	intergenic	*C2orf27B,ANKRD30BL*	.	.	.
2	132876816	C	G	intergenic	*C2orf27B,ANKRD30BL*	.	.	.
2	132812342	A	G	intergenic	*C2orf27B,ANKRD30BL*	.	.	.
2	132851176	AT	A	intergenic	*C2orf27B,ANKRD30BL*	.	.	.
2	132598082	C	A	intergenic	*C2orf27B,ANKRD30BL*	.	.	.
2	132759926	T	A	intergenic	*C2orf27B,ANKRD30BL*	.	.	.
2	132883782	A	G	intergenic	*C2orf27B,ANKRD30BL*	.	.	.
2	70384454	C	T	intronic	*C2orf42*	.	.	.
2	201139078	C	T	intergenic	*C2orf47,SPATS2L*	.	.	.
2	200979404	T	G	intergenic	*C2orf47,SPATS2L*	.	.	.
2	200979594	T	A	intergenic	*C2orf47,SPATS2L*	.	.	.
2	200981245	C	T	intergenic	*C2orf47,SPATS2L*	.	.	.
2	201068940	G	C	intergenic	*C2orf47,SPATS2L*	.	.	.
2	201070139	A	G	intergenic	*C2orf47,SPATS2L*	.	.	.
2	201070422	T	G	intergenic	*C2orf47,SPATS2L*	.	.	.
2	201070797	C	T	intergenic	*C2orf47,SPATS2L*	.	.	.
2	201070798	A	G	intergenic	*C2orf47,SPATS2L*	.	.	.
2	201115593	T	C	intergenic	*C2orf47,SPATS2L*	.	.	.
2	10299232	A	C	intronic	*C2orf48*	.	.	.
2	10403864	G	T	intergenic	*C2orf48,HPCAL1*	.	.	.

2	241851470	G	A	intergenic	*C2orf54,LOC200772*	.	.	.
2	197678974	G	T	intergenic	*C2orf66,PGAP1*	.	.	.
19	6708564	C	T	intronic	*C3*	.	.	.
19	6728444	A	G	intergenic	*C3,GPR108*	.	.	.
3	14793483	G	A	intronic	*C3orf20*	.	.	.
3	155509747	T	A	intronic	*C3orf33*	.	.	.
3	37431558	T	C	intronic	*C3orf35*	.	.	.
3	37435597	G	C	intronic	*C3orf35*	.	.	.
3	37468073	C	G	intronic	*C3orf35*	.	.	.
3	37468074	G	T	intronic	*C3orf35*	.	.	.
3	88908709	A	C	intergenic	*C3orf38,EPHA3*	.	.	.
3	88966174	T	C	intergenic	*C3orf38,EPHA3*	.	.	.
3	88800872	T	G	intergenic	*C3orf38,EPHA3*	.	.	.
3	88904240	G	C	intergenic	*C3orf38,EPHA3*	.	.	.
3	88952052	C	T	intergenic	*C3orf38,EPHA3*	.	.	.
3	88952053	A	G	intergenic	*C3orf38,EPHA3*	.	.	.
3	89118376	A	G	intergenic	*C3orf38,EPHA3*	.	.	.
3	89141449	G	A	intergenic	*C3orf38,EPHA3*	.	.	.
3	89141713	G	GA	intergenic	*C3orf38,EPHA3*	.	.	.
3	63809201	C	T	ncRNA_exonic	*C3orf49*	.	.	.
3	144003688	A	G	intergenic	*C3orf58,PLOD2*	.	.	.
3	144242766	A	G	intergenic	*C3orf58,PLOD2*	.	.	.
3	145293728	C	T	intergenic	*C3orf58,PLOD2*	.	.	.
3	145635219	T	G	intergenic	*C3orf58,PLOD2*	.	.	.
3	144575502	TCCTC	T	intergenic	*C3orf58,PLOD2*	.	.	.
3	145716515	T	C	intergenic	*C3orf58,PLOD2*	.	.	.
3	49305136	ATTTT	A	downstream	*C3orf62*	.	.	.
3	58983737	C	T	ncRNA_intronic	*C3orf67-AS1*	.	.	.
3	58841332	A	C	ncRNA_intronic	*C3orf67-AS1*	.	.	.
3	58935221	G	A	ncRNA_intronic	*C3orf67-AS1*	.	.	.
3	59209270	T	C	intergenic	*C3orf67,FHIT*	.	.	.
3	59344525	G	A	intergenic	*C3orf67,FHIT*	.	.	.
3	59344762	G	T	intergenic	*C3orf67,FHIT*	.	.	.
3	153512987	TAATAAA	T	intergenic	*C3orf79,ARHGEF26-AS1*	.	.	.
3	153315483	A	G	intergenic	*C3orf79,ARHGEF26-AS1*	.	.	.

3	153691413	C	T	intergenic	*C3orf79,ARHGEF26-AS1*	.	.	.
3	153666149	G	GA	intergenic	*C3orf79,ARHGEF26-AS1*	.	.	.
3	153246566	C	A	intergenic	*C3orf79,ARHGEF26-AS1*	.	.	.
3	49230959	A	G	intergenic	*C3orf84,CCDC36*	.	.	.
3	49230961	T	C	intergenic	*C3orf84,CCDC36*	.	.	.
19	10182011	G	T	ncRNA_intronic	*C3P1*	.	.	.
1	207315539	C	T	intronic	*C4BPA*	.	.	.
1	207383452	C	A	intergenic	*C4BPA,CD55*	.	.	.
1	207385066	C	T	intergenic	*C4BPA,CD55*	.	.	.
1	207385872	C	A	intergenic	*C4BPA,CD55*	.	.	.
1	207386206	T	C	intergenic	*C4BPA,CD55*	.	.	.
1	207435789	C	CCATTTTGA(	intergenic	*C4BPA,CD55*	.	.	.
4	37498102	G	C	intronic	*C4orf19*	.	.	.
4	37506781	T	G	intronic	*C4orf19*	.	.	.
4	37461818	T	C	intronic	*C4orf19*	.	.	.
4	37461819	G	A	intronic	*C4orf19*	.	.	.
4	81267434	G	T	intronic	*C4orf22*	.	.	.
4	81320078	G	T	intronic	*C4orf22*	.	.	.
4	81363596	A	G	intronic	*C4orf22*	.	.	.
4	81578784	A	G	intronic	*C4orf22*	.	.	.
4	81594253	A	G	intronic	*C4orf22*	.	.	.
4	81595241	C	T	intronic	*C4orf22*	.	.	.
4	81611992	G	A	intronic	*C4orf22*	.	.	.
4	81759534	G	GT	intronic	*C4orf22*	.	.	.
4	81937540	A	AGGTG	intergenic	*C4orf22,BMP3*	.	.	.
4	170760718	G	T	intergenic	*C4orf27,LOC100506085*	.	.	.
4	113083695	G	A	intronic	*C4orf32*	.	.	.
4	113084229	G	T	intronic	*C4orf32*	.	.	.
4	113084280	A	G	intronic	*C4orf32*	.	.	.
4	130487505	A	T	intergenic	*C4orf33,LOC101927282*	.	.	.
4	130044504	T	G	intergenic	*C4orf33,LOC101927282*	.	.	.

4	130105102	A	G	intergenic	*C4orf33,LOC101927282*	.	.	.
4	130105815	A	T	intergenic	*C4orf33,LOC101927282*	.	.	.
4	130209055	C	A	intergenic	*C4orf33,LOC101927282*	.	.	.
4	130209056	C	G	intergenic	*C4orf33,LOC101927282*	.	.	.
4	130210110	A	G	intergenic	*C4orf33,LOC101927282*	.	.	.
4	130237070	T	C	intergenic	*C4orf33,LOC101927282*	.	.	.
4	130512255	C	T	intergenic	*C4orf33,LOC101927282*	.	.	.
4	130513354	A	C	intergenic	*C4orf33,LOC101927282*	.	.	.
4	130514261	C	A	intergenic	*C4orf33,LOC101927282*	.	.	.
4	130514263	G	A	intergenic	*C4orf33,LOC101927282*	.	.	.
4	130546247	G	C	intergenic	*C4orf33,LOC101927282*	.	.	.
4	130546298	A	T	intergenic	*C4orf33,LOC101927282*	.	.	.
4	130577391	T	A	intergenic	*C4orf33,LOC101927282*	.	.	.
4	130577654	C	T	intergenic	*C4orf33,LOC101927282*	.	.	.
4	130577656	A	C	intergenic	*C4orf33,LOC101927282*	.	.	.
4	87832439	G	C	intergenic	*C4orf36,LOC100506746*	.	.	.
4	159907720	A	G	intronic	*C4orf45*	.	.	.
4	159908567	T	A	intronic	*C4orf45*	.	.	.
4	159910180	T	C	intronic	*C4orf45*	.	.	.
4	159910181	G	A	intronic	*C4orf45*	.	.	.
4	159911080	T	C	intronic	*C4orf45*	.	.	.
4	186369394	C	T	intronic	*C4orf47,CCDC110*	.	.	.

5	24104429	C	G	ncRNA_intronic	*C5orf17*	.	.	.
5	24178563	T	C	downstream	*C5orf17*	.	.	.
5	24028497	A	G	ncRNA_intronic	*C5orf17*	.	.	.
5	24038519	C	A	ncRNA_intronic	*C5orf17*	.	.	.
5	24083643	ATGTGTG	A	ncRNA_intronic	*C5orf17*	.	.	.
5	24283426	A	C	intergenic	*C5orf17,CDH10*	.	.	.
5	24285948	A	C	intergenic	*C5orf17,CDH10*	.	.	.
5	24306021	T	C	intergenic	*C5orf17,CDH10*	.	.	.
5	24306022	G	C	intergenic	*C5orf17,CDH10*	.	.	.
5	24315940	T	C	intergenic	*C5orf17,CDH10*	.	.	.
5	24315941	T	C	intergenic	*C5orf17,CDH10*	.	.	.
5	31544552	G	A	intronic	*C5orf22*	.	.	.
5	43483953	A	T	UTR5	*C5orf28*	.	.	.
5	43451653	G	A	intronic	*C5orf28*	.	.	.
5	43473551	C	T	intronic	*C5orf28*	.	.	.
5	43473731	A	G	intronic	*C5orf28*	.	.	.
5	102595751	CGTGTGTGTGTGTGTGTGTGTGTGTGTTT	C	intronic	*C5orf30*	.	.	.
5	102829837	C	T	intergenic	*C5orf30,LOC102467212*	.	.	.
5	102716261	C	T	intergenic	*C5orf30,LOC102467212*	.	.	.
5	102716805	A	G	intergenic	*C5orf30,LOC102467212*	.	.	.
5	102842214	C	T	intergenic	*C5orf30,LOC102467212*	.	.	.
5	102842215	A	G	intergenic	*C5orf30,LOC102467212*	.	.	.
5	3166544	C	A	intergenic	*C5orf38,LINC01377*	.	.	.
5	2762546	G	A	intergenic	*C5orf38,LINC01377*	.	.	.
5	2907409	T	A	intergenic	*C5orf38,LINC01377*	.	.	.
5	3133508	G	A	intergenic	*C5orf38,LINC01377*	.	.	.

5	3138999	A	G	intergenic	*C5orf38,LINC01377*	.	.	.
5	3139829	A	G	intergenic	*C5orf38,LINC01377*	.	.	.
5	3039966	G	A	intergenic	*C5orf38,LINC01377*	.	.	.
5	3160091	T	G	intergenic	*C5orf38,LINC01377*	.	.	.
5	2943754	C	T	intergenic	*C5orf38,LINC01377*	.	.	.
5	37261614	T	G	intergenic	*C5orf42,NUP155*	.	.	.
5	147322263	C	A	intergenic	*C5orf46,SPINK5*	.	.	.
5	173459287	A	G	intergenic	*C5orf47,HMP19*	.	.	.
5	173459913	T	C	intergenic	*C5orf47,HMP19*	.	.	.
5	157151064	C	G	intergenic	*C5orf52,THG1L*	.	.	.
5	157151388	C	T	intergenic	*C5orf52,THG1L*	.	.	.
5	169663581	C	T	intronic	*C5orf58*	.	.	.
5	126390529	T	C	intronic	*C5orf63*	.	.	.
5	126390781	T	G	intronic	*C5orf63*	.	.	.
5	126439038	T	C	intergenic	*C5orf63,MEGF10*	.	.	.
5	126440447	C	G	intergenic	*C5orf63,MEGF10*	.	.	.
5	126483296	G	A	intergenic	*C5orf63,MEGF10*	.	.	.
5	126493126	T	C	intergenic	*C5orf63,MEGF10*	.	.	.
5	126511045	C	T	intergenic	*C5orf63,MEGF10*	.	.	.
5	126581600	T	C	intergenic	*C5orf63,MEGF10*	.	.	.
5	61006367	A	C	intergenic	*C5orf64,LOC100506526*	.	.	.
5	134569836	G	A	intronic	*C5orf66*	.	.	.
5	55814376	CCTCT	C	intronic	*C5orf67*	.	.	.
5	56027049	C	T	intergenic	*C5orf67,MAP3K1*	.	.	.
5	56073712	C	T	intergenic	*C5orf67,MAP3K1*	.	.	.
5	56075146	T	G	intergenic	*C5orf67,MAP3K1*	.	.	.
5	41233041	T	C	intronic	*C6*	.	.	.
5	41197948	T	C	intronic	*C6*	.	.	.
5	41210297	G	A	intronic	*C6*	.	.	.
5	41210423	A	T	intronic	*C6*	.	.	.
6	32327554	C	T	intronic	*C6orf10*	.	.	.
6	34685938	C	T	intergenic	*C6orf106,SNRPC*	.	.	.

6	34693513	G	A	intergenic	*C6orf106,SNRPC*	.	.	.
6	165702628	T	C	intronic	*C6orf118*	.	.	.
6	165699269	T	TG	intronic	*C6orf118*	.	.	.
6	49568469	T	G	intergenic	*C6orf141,RHAG*	.	.	.
6	49571367	A	C	intergenic	*C6orf141,RHAG*	.	.	.
6	107359121	T	C	intronic	*C6orf203*	.	.	.
6	127918439	T	C	intergenic	*C6orf58,THEMIS*	.	.	.
6	127973407	T	A	intergenic	*C6orf58,THEMIS*	.	.	.
6	127998184	A	G	intergenic	*C6orf58,THEMIS*	.	.	.
6	127998185	A	C	intergenic	*C6orf58,THEMIS*	.	.	.
6	36871001	TTGTG	T	intronic	*C6orf89*	.	.	.
6	36910470	T	C	intergenic	*C6orf89,PI16*	.	.	.
6	36910471	G	A	intergenic	*C6orf89,PI16*	.	.	.
6	159389289	T	C	intergenic	*C6orf99,RSPH3*	.	.	.
6	159389290	G	A	intergenic	*C6orf99,RSPH3*	.	.	.
7	112486579	A	T	intronic	*C7orf60*	.	.	.
7	100057956	T	C	intronic	*C7orf61*	.	.	.
7	109278087	T	A	intergenic	*C7orf66,EIF3IP1*	.	.	.
7	109043526	TGTGC	T	intergenic	*C7orf66,EIF3IP1*	.	.	.
7	108612559	G	C	intergenic	*C7orf66,EIF3IP1*	.	.	.
7	109234284	A	C	intergenic	*C7orf66,EIF3IP1*	.	.	.
7	108903299	A	T	intergenic	*C7orf66,EIF3IP1*	.	.	.
7	108967288	G	A	intergenic	*C7orf66,EIF3IP1*	.	.	.
7	109402134	T	G	intergenic	*C7orf66,EIF3IP1*	.	.	.
7	109490678	A	G	intergenic	*C7orf66,EIF3IP1*	.	.	.
7	109331134	C	CTT	intergenic	*C7orf66,EIF3IP1*	.	.	.
7	108600522	T	G	intergenic	*C7orf66,EIF3IP1*	.	.	.
7	109407958	G	A	intergenic	*C7orf66,EIF3IP1*	.	.	.
7	109425261	A	G	intergenic	*C7orf66,EIF3IP1*	.	.	.
7	108620088	A	G	intergenic	*C7orf66,EIF3IP1*	.	.	.
7	108671912	C	T	intergenic	*C7orf66,EIF3IP1*	.	.	.
7	108671913	A	G	intergenic	*C7orf66,EIF3IP1*	.	.	.
7	108682304	G	A	intergenic	*C7orf66,EIF3IP1*	.	.	.
7	108682555	C	A	intergenic	*C7orf66,EIF3IP1*	.	.	.
7	108725057	T	A	intergenic	*C7orf66,EIF3IP1*	.	.	.
7	108725058	C	T	intergenic	*C7orf66,EIF3IP1*	.	.	.
7	108735531	G	C	intergenic	*C7orf66,EIF3IP1*	.	.	.
7	108825755	CTTTT	C	intergenic	*C7orf66,EIF3IP1*	.	.	.

7	108850643	G	A	intergenic	*C7orf66,EIF3IP1*	.	.	.
7	108850645	A	G	intergenic	*C7orf66,EIF3IP1*	.	.	.
7	108955368	C	T	intergenic	*C7orf66,EIF3IP1*	.	.	.
7	109023881	C	A	intergenic	*C7orf66,EIF3IP1*	.	.	.
7	109024314	G	A	intergenic	*C7orf66,EIF3IP1*	.	.	.
7	109130952	C	CT	intergenic	*C7orf66,EIF3IP1*	.	.	.
7	109197664	C	T	intergenic	*C7orf66,EIF3IP1*	.	.	.
7	109197996	A	G	intergenic	*C7orf66,EIF3IP1*	.	.	.
7	109331201	A	T	intergenic	*C7orf66,EIF3IP1*	.	.	.
7	109422927	AAGAG	A	intergenic	*C7orf66,EIF3IP1*	.	.	.
7	109502761	G	A	intergenic	*C7orf66,EIF3IP1*	.	.	.
7	109552566	C	T	intergenic	*C7orf66,EIF3IP1*	.	.	.
7	109553142	C	T	intergenic	*C7orf66,EIF3IP1*	.	.	.
7	50273550	C	T	intergenic	*C7orf72,IKZF1*	.	.	.
7	96182128	A	G	intergenic	*C7orf76,LOC100506136*	.	.	.
7	96200185	A	G	intergenic	*C7orf76,LOC100506136*	.	.	.
7	96200556	A	AT	intergenic	*C7orf76,LOC100506136*	.	.	.
7	96201920	T	A	intergenic	*C7orf76,LOC100506136*	.	.	.
7	96210500	C	T	intergenic	*C7orf76,LOC100506136*	.	.	.
7	96210501	A	G	intergenic	*C7orf76,LOC100506136*	.	.	.
7	96213408	T	C	intergenic	*C7orf76,LOC100506136*	.	.	.
7	124449951	T	C	intergenic	*C7orf77,POT1*	.	.	.
8	50730296	C	T	intergenic	*C8orf22,SNTG1*	.	.	.
8	50084664	C	A	intergenic	*C8orf22,SNTG1*	.	.	.
8	50096226	A	T	intergenic	*C8orf22,SNTG1*	.	.	.
8	50706393	T	A	intergenic	*C8orf22,SNTG1*	.	.	.
8	50759725	G	A	intergenic	*C8orf22,SNTG1*	.	.	.
8	50114526	T	G	intergenic	*C8orf22,SNTG1*	.	.	.
8	50178279	T	C	intergenic	*C8orf22,SNTG1*	.	.	.
8	50178280	A	C	intergenic	*C8orf22,SNTG1*	.	.	.
8	50364692	A	G	intergenic	*C8orf22,SNTG1*	.	.	.

8	50418356	A	G	intergenic	*C8orf22,SNTG1*	.	.	.
8	50442340	GT	G	intergenic	*C8orf22,SNTG1*	.	.	.
8	50444362	A	T	intergenic	*C8orf22,SNTG1*	.	.	.
8	50591293	T	C	intergenic	*C8orf22,SNTG1*	.	.	.
8	50788362	A	G	intergenic	*C8orf22,SNTG1*	.	.	.
8	50788364	T	C	intergenic	*C8orf22,SNTG1*	.	.	.
8	50789265	A	G	intergenic	*C8orf22,SNTG1*	.	.	.
8	146299112	A	G	intergenic	*C8orf33,NONE*	.	.	.
8	146298399	G	T	intergenic	*C8orf33,NONE*	.	.	.
8	146298400	T	C	intergenic	*C8orf33,NONE*	.	.	.
8	69657645	A	G	intronic	*C8orf34*	.	.	.
8	69727588	C	T	intronic	*C8orf34*	.	.	.
8	69667088	A	T	intronic	*C8orf34*	.	.	.
8	69590185	G	T	intronic	*C8orf34*	.	.	.
8	69317012	G	C	intronic	*C8orf34*	.	.	.
8	69526149	G	A	intronic	*C8orf34*	.	.	.
8	69530126	A	G	intronic	*C8orf34*	.	.	.
8	69658230	T	C	intronic	*C8orf34*	.	.	.
8	69801307	C	T	intergenic	*C8orf34,LINC01592*	.	.	.
8	96413772	GTCTC	G	ncRNA_intronic	*C8orf37-AS1*	.	.	.
8	96546946	G	A	ncRNA_intronic	*C8orf37-AS1*	.	.	.
8	96299980	A	G	ncRNA_intronic	*C8orf37-AS1*	.	.	.
8	96390993	G	A	ncRNA_intronic	*C8orf37-AS1*	.	.	.
8	96589366	T	C	ncRNA_intronic	*C8orf37-AS1*	.	.	.
8	96604023	T	C	ncRNA_intronic	*C8orf37-AS1*	.	.	.
8	96604696	C	T	ncRNA_intronic	*C8orf37-AS1*	.	.	.
8	96729821	T	A	ncRNA_intronic	*C8orf37-AS1*	.	.	.
8	96769656	T	A	ncRNA_intronic	*C8orf37-AS1*	.	.	.
8	96769720	C	T	ncRNA_intronic	*C8orf37-AS1*	.	.	.
8	96910731	AATAT	A	intergenic	*C8orf37-AS1,LOC100500773*	.	.	.
8	96895003	C	T	intergenic	*C8orf37-AS1,LOC100500773*	.	.	.
8	40035598	G	C	intergenic	*C8orf4,ZMAT4*	.	.	.
8	40365035	G	A	intergenic	*C8orf4,ZMAT4*	.	.	.

8	40366685	T	C	intergenic	*C8orf4,ZMAT4*	.	.	.
8	40366686	G	A	intergenic	*C8orf4,ZMAT4*	.	.	.
8	40367030	T	G	intergenic	*C8orf4,ZMAT4*	.	.	.
8	67627596	G	A	intronic	*C8orf44-SGK3,SGK3*	.	.	.
8	67747068	G	A	intronic	*C8orf44-SGK3,SGK3*	.	.	.
8	67772510	A	T	UTR3	*C8orf44-SGK3,SGK3*	.	.	.
8	67772511	C	G	UTR3	*C8orf44-SGK3,SGK3*	.	.	.
8	13552080	A	T	intergenic	*C8orf48,SGCZ*	.	.	.
8	13882955	TAAC	T	intergenic	*C8orf48,SGCZ*	.	.	.
8	13683296	C	G	intergenic	*C8orf48,SGCZ*	.	.	.
8	13735936	A	AT	intergenic	*C8orf48,SGCZ*	.	.	.
8	13756046	GTGTA	G	intergenic	*C8orf48,SGCZ*	.	.	.
8	13861325	G	A	intergenic	*C8orf48,SGCZ*	.	.	.
8	13931955	C	T	intergenic	*C8orf48,SGCZ*	.	.	.
8	94310942	GTATA	G	intergenic	*C8orf87,LINC00535*	.	.	.
8	94213685	T	A	intergenic	*C8orf87,LINC00535*	.	.	.
8	94214780	C	T	intergenic	*C8orf87,LINC00535*	.	.	.
8	74159898	A	T	intronic	*C8orf89*	.	.	.
8	74167610	C	T	intronic	*C8orf89*	.	.	.
5	39348502	A	G	intronic	*C9*	.	.	.
9	72588560	C	T	intergenic	*C9orf135,MAMDC2*	.	.	.
9	88851494	T	A	intronic	*C9orf153*	.	.	.
9	97501725	G	A	intronic	*C9orf3*	.	.	.
9	116200877	C	T	intergenic	*C9orf43,RGS3*	.	.	.
9	74689019	C	T	intergenic	*C9orf57,GDA*	.	.	.
9	74690756	T	C	intergenic	*C9orf57,GDA*	.	.	.
9	74714035	T	C	intergenic	*C9orf57,GDA*	.	.	.
9	86563543	C	A	intronic	*C9orf64*	.	.	.
9	27635913	G	C	intergenic	*C9orf72,LINGO2*	.	.	.
9	27585536	C	T	intergenic	*C9orf72,LINGO2*	.	.	.
9	27779752	T	C	intergenic	*C9orf72,LINGO2*	.	.	.

9	74613769	A	G	intergenic	*C9orf85,C9orf57*	.	.	.
9	16227773	T	C	intronic	*C9orf92*	.	.	.
9	16227914	T	C	intronic	*C9orf92*	.	.	.
9	16383656	C	T	intergenic	*C9orf92,BNC2*	.	.	.
8	86331809	C	T	intergenic	*CA1,CA3*	.	.	.
8	86332821	C	T	intergenic	*CA1,CA3*	.	.	.
8	86334222	G	A	intergenic	*CA1,CA3*	.	.	.
8	86334931	A	G	intergenic	*CA1,CA3*	.	.	.
8	86334932	G	A	intergenic	*CA1,CA3*	.	.	.
17	49810430	C	G	intronic	*CA10*	.	.	.
17	49846023	G	A	intronic	*CA10*	.	.	.
17	49965722	C	T	intronic	*CA10*	.	.	.
17	49839376	A	C	intronic	*CA10*	.	.	.
17	50098119	T	G	intronic	*CA10*	.	.	.
17	49889048	A	T	intronic	*CA10*	.	.	.
17	49974415	T	G	intronic	*CA10*	.	.	.
17	49974416	T	C	intronic	*CA10*	.	.	.
17	50885092	A	G	intergenic	*CA10,C17orf112*	.	.	.
17	50244247	A	T	intergenic	*CA10,C17orf112*	.	.	.
17	50458312	C	G	intergenic	*CA10,C17orf112*	.	.	.
17	50891439	G	GT	intergenic	*CA10,C17orf112*	.	.	.
17	50718275	CAGA	C	intergenic	*CA10,C17orf112*	.	.	.
17	50864772	C	A	intergenic	*CA10,C17orf112*	.	.	.
17	51018919	G	T	intergenic	*CA10,C17orf112*	.	.	.
17	50502574	C	A	intergenic	*CA10,C17orf112*	.	.	.
17	50502575	C	G	intergenic	*CA10,C17orf112*	.	.	.
17	50627516	G	T	intergenic	*CA10,C17orf112*	.	.	.
17	50693646	C	T	intergenic	*CA10,C17orf112*	.	.	.
8	86436682	C	T	intergenic	*CA2,REXO1L2P*	.	.	.
8	86493007	C	G	intergenic	*CA2,REXO1L2P*	.	.	.
8	86532298	G	C	intergenic	*CA2,REXO1L2P*	.	.	.
8	86559909	C	A	intergenic	*CA2,REXO1L2P*	.	.	.
16	87957960	G	GAC	intronic	*CA5A*	.	.	.
8	61155271	C	T	intronic	*CA8*	.	.	.
2	231667089	C	T	intronic	*CAB39*	.	.	.
2	231690776	T	G	intergenic	*CAB39,ITM2C*	.	.	.
22	24510025	TTTGTTG	T	intronic	*CABIN1*	.	.	.
22	24464913	GCC	G	intronic	*CABIN1*	.	.	.

19	13545245	C	T	intronic	*CACNA1A*	.	.	.
19	13763094	C	T	intergenic	*CACNA1A,CCDC130*	.	.	.
19	13768505	A	C	intergenic	*CACNA1A,CCDC130*	.	.	.
9	141036178	C	T	intergenic	*CACNA1B,TUBBP5*	.	.	.
9	141036433	C	T	intergenic	*CACNA1B,TUBBP5*	.	.	.
12	2760099	G	T	intronic	*CACNA1C*	.	.	.
12	2649548	A	G	intronic	*CACNA1C*	.	.	.
12	2736846	C	G	intronic	*CACNA1C*	.	.	.
12	2190027	A	G	intronic	*CACNA1C*	.	.	.
12	2213733	T	C	intronic	*CACNA1C*	.	.	.
12	2213419	A	G	intronic	*CACNA1C*	.	.	.
12	2811568	T	C	intergenic	*CACNA1C,LOC283440*	.	.	.
12	2813464	A	G	intergenic	*CACNA1C,LOC283440*	.	.	.
12	2856132	C	T	intergenic	*CACNA1C,LOC283440*	.	.	.
12	2856133	A	G	intergenic	*CACNA1C,LOC283440*	.	.	.
3	53751782	T	C	intronic	*CACNA1D*	.	.	.
1	181584131	T	C	intronic	*CACNA1E*	.	.	.
1	181729205	G	T	intronic	*CACNA1E*	.	.	.
1	181653352	G	A	intronic	*CACNA1E*	.	.	.
1	181822482	C	A	intergenic	*CACNA1E,ZNF648*	.	.	.
1	181823195	C	T	intergenic	*CACNA1E,ZNF648*	.	.	.
1	181853764	C	T	intergenic	*CACNA1E,ZNF648*	.	.	.
1	181856991	A	AC	intergenic	*CACNA1E,ZNF648*	.	.	.
1	181861364	C	T	intergenic	*CACNA1E,ZNF648*	.	.	.
1	181861365	A	G	intergenic	*CACNA1E,ZNF648*	.	.	.
1	181915830	T	C	intergenic	*CACNA1E,ZNF648*	.	.	.
1	181949200	C	T	intergenic	*CACNA1E,ZNF648*	.	.	.
17	48658611	G	A	intronic	*CACNA1G*	.	.	.
17	48702297	A	C	intronic	*CACNA1G*	.	.	.
16	1265456	C	T	intronic	*CACNA1H*	.	.	.

22	40081447	G	GAGATCGGA	intronic	*CACNA1I*	.	.	.
22	40088740	A	T	intergenic	*CACNA1I,ENTHD1*	.	.	.
1	201021358	C	T	intronic	*CACNA1S*	.	.	.
7	81607524	G	A	intronic	*CACNA2D1*	.	.	.
7	81691981	C	T	intronic	*CACNA2D1*	.	.	.
7	81853651	G	C	intronic	*CACNA2D1*	.	.	.
7	81925121	A	C	intronic	*CACNA2D1*	.	.	.
7	82029335	C	T	intronic	*CACNA2D1*	.	.	.
7	82322798	A	AAAAT	intergenic	*CACNA2D1,PCLO*	.	.	.
7	82378536	GGTGTGT	G	intergenic	*CACNA2D1,PCLO*	.	.	.
7	82185146	C	G	intergenic	*CACNA2D1,PCLO*	.	.	.
7	82185147	T	C	intergenic	*CACNA2D1,PCLO*	.	.	.
7	82210221	A	G	intergenic	*CACNA2D1,PCLO*	.	.	.
7	82210367	G	C	intergenic	*CACNA2D1,PCLO*	.	.	.
7	82239796	T	C	intergenic	*CACNA2D1,PCLO*	.	.	.
7	82239797	G	A	intergenic	*CACNA2D1,PCLO*	.	.	.
7	82241294	C	T	intergenic	*CACNA2D1,PCLO*	.	.	.
7	82241295	A	G	intergenic	*CACNA2D1,PCLO*	.	.	.
7	82265362	A	G	intergenic	*CACNA2D1,PCLO*	.	.	.
7	82265677	A	C	intergenic	*CACNA2D1,PCLO*	.	.	.
3	50445171	C	A	intronic	*CACNA2D2*	.	.	.
3	50425035	G	A	exonic	*CACNA2D2*	synonymous_SNV	.	.
3	54889151	A	G	intronic	*CACNA2D3*	.	.	.
3	54792621	T	C	intronic	*CACNA2D3*	.	.	.
3	54268637	C	A	intronic	*CACNA2D3*	.	.	.
3	54506668	C	T	intronic	*CACNA2D3*	.	.	.
3	54506669	A	G	intronic	*CACNA2D3*	.	.	.
3	54506819	A	T	intronic	*CACNA2D3*	.	.	.
3	54726178	C	T	intronic	*CACNA2D3*	.	.	.
3	55211702	T	G	intergenic	*CACNA2D3,WNT5A*	.	.	.
3	55415355	G	A	intergenic	*CACNA2D3,WNT5A*	.	.	.
3	55196863	A	G	intergenic	*CACNA2D3,WNT5A*	.	.	.
3	55346545	A	G	intergenic	*CACNA2D3,WNT5A*	.	.	.

3	55425747	C	T	intergenic	*CACNA2D3,WNT5A*	.	.	.
12	2010752	C	T	intronic	*CACNA2D4*	.	.	.
10	18625603	C	G	intronic	*CACNB2*	.	.	.
2	152855656	A	ATCAC	intronic	*CACNB4*	.	.	.
2	152768784	G	T	intronic	*CACNB4*	.	.	.
2	152768786	G	T	intronic	*CACNB4*	.	.	.
2	152769642	C	G	intronic	*CACNB4*	.	.	.
2	152769643	A	T	intronic	*CACNB4*	.	.	.
22	37076717	A	ACCACTATA(	intronic	*CACNG2*	.	.	.
16	24411558	T	C	intergenic	*CACNG3,RBBP6*	.	.	.
16	24509197	C	A	intergenic	*CACNG3,RBBP6*	.	.	.
17	64968779	C	T	intronic	*CACNG4*	.	.	.
19	54541847	G	A	intergenic	*CACNG6,VSTM1*	.	.	.
10	120555426	A	G	intergenic	*CACUL1,NANOS1*	.	.	.
10	120555627	G	C	intergenic	*CACUL1,NANOS1*	.	.	.
10	120623547	G	A	intergenic	*CACUL1,NANOS1*	.	.	.
10	120640883	G	A	intergenic	*CACUL1,NANOS1*	.	.	.
11	115324777	A	C	intronic	*CADM1*	.	.	.
11	115080646	C	T	intronic	*CADM1*	.	.	.
11	115263580	A	G	intronic	*CADM1*	.	.	.
11	115108186	T	C	intronic	*CADM1*	.	.	.
11	115132568	C	T	intronic	*CADM1*	.	.	.
11	115345176	T	A	intronic	*CADM1*	.	.	.
11	115345177	T	A	intronic	*CADM1*	.	.	.
3	85489581	G	A	intronic	*CADM2*	.	.	.
3	85777203	A	C	intronic	*CADM2*	.	.	.
3	85622307	TTTTATTTATTTATTTATTTATTTATTTA	T	intronic	*CADM2*	.	.	.
3	86008300	ATCTC	A	intronic	*CADM2*	.	.	.
3	85669410	T	A	intronic	*CADM2*	.	.	.
3	85342035	A	G	intronic	*CADM2*	.	.	.
3	85448221	T	C	intronic	*CADM2*	.	.	.
3	85642439	C	T	intronic	*CADM2*	.	.	.
3	85754016	ATTT	A	intronic	*CADM2*	.	.	.
3	85995277	T	C	intronic	*CADM2*	.	.	.

3	86068998	C	T	intronic	*CADM2*	.	.	.
3	86089847	A	G	intronic	*CADM2*	.	.	.
3	86092527	A	C	intronic	*CADM2*	.	.	.
3	85863339	T	C	ncRNA_intronic	*CADM2-AS2*	.	.	.
3	86330262	G	A	intergenic	*CADM2,VGLL3*	.	.	.
3	86457287	T	G	intergenic	*CADM2,VGLL3*	.	.	.
3	86546380	A	T	intergenic	*CADM2,VGLL3*	.	.	.
3	86672743	C	CAAATTAT	intergenic	*CADM2,VGLL3*	.	.	.
3	86738851	A	G	intergenic	*CADM2,VGLL3*	.	.	.
3	86144894	T	C	intergenic	*CADM2,VGLL3*	.	.	.
3	86147723	A	C	intergenic	*CADM2,VGLL3*	.	.	.
3	86349025	T	C	intergenic	*CADM2,VGLL3*	.	.	.
3	86349241	T	C	intergenic	*CADM2,VGLL3*	.	.	.
3	86350914	G	A	intergenic	*CADM2,VGLL3*	.	.	.
3	86351463	G	A	intergenic	*CADM2,VGLL3*	.	.	.
3	86634433	G	A	intergenic	*CADM2,VGLL3*	.	.	.
3	86756212	C	T	intergenic	*CADM2,VGLL3*	.	.	.
3	86856534	T	A	intergenic	*CADM2,VGLL3*	.	.	.
3	86887367	A	T	intergenic	*CADM2,VGLL3*	.	.	.
3	86887368	C	T	intergenic	*CADM2,VGLL3*	.	.	.
3	86936692	T	C	intergenic	*CADM2,VGLL3*	.	.	.
3	86936693	G	A	intergenic	*CADM2,VGLL3*	.	.	.
3	62847603	G	A	intronic	*CADPS*	.	.	.
3	62842452	A	T	intronic	*CADPS*	.	.	.
3	62892052	A	T	intergenic	*CADPS,LINC00698*	.	.	.
3	62999604	T	C	intergenic	*CADPS,LINC00698*	.	.	.
7	121971692	C	CA	intronic	*CADPS2*	.	.	.
7	122231363	T	A	intronic	*CADPS2*	.	.	.
7	122297285	A	G	intronic	*CADPS2*	.	.	.
7	122341321	T	A	intronic	*CADPS2*	.	.	.
7	122477398	A	C	intronic	*CADPS2*	.	.	.
7	122480121	A	T	intronic	*CADPS2*	.	.	.
7	122513937	G	A	intronic	*CADPS2*	.	.	.
7	122514833	A	G	intronic	*CADPS2*	.	.	.
6	7364947	C	T	intronic	*CAGE1*	.	.	.
8	91221853	T	C	intergenic	*CALB1,LINC00534*	.	.	.

8	91221854	G	A	intergenic	*CALB1,LINC00534*	.	.	.
11	15016502	C	A	intergenic	*CALCA,CALCB*	.	.	.
11	15019507	T	C	intergenic	*CALCA,CALCB*	.	.	.
11	15108703	A	T	intergenic	*CALCB,INSC*	.	.	.
11	15108704	C	T	intergenic	*CALCB,INSC*	.	.	.
7	93120292	A	G	intronic	*CALCR*	.	.	.
7	93274202	G	A	intergenic	*CALCR,MIR4652*	.	.	.
7	93290724	G	A	intergenic	*CALCR,MIR4652*	.	.	.
7	93290726	G	A	intergenic	*CALCR,MIR4652*	.	.	.
7	93336257	C	A	intergenic	*CALCR,MIR4652*	.	.	.
2	188221462	T	A	intronic	*CALCRL*	.	.	.
7	134538866	A	T	intronic	*CALD1*	.	.	.
7	134662874	G	A	intergenic	*CALD1,AGBL3*	.	.	.
7	134665361	G	A	intergenic	*CALD1,AGBL3*	.	.	.
7	134665994	G	C	intergenic	*CALD1,AGBL3*	.	.	.
14	90894381	A	G	intergenic	*CALM1,LINC00642*	.	.	.
10	5643616	G	A	intergenic	*CALML3,ASB13*	.	.	.
7	71610904	C	T	intronic	*CALN1*	.	.	.
7	71616653	A	C	intronic	*CALN1*	.	.	.
7	71690114	A	G	intronic	*CALN1*	.	.	.
7	71702924	T	A	intronic	*CALN1*	.	.	.
7	71266014	A	G	intronic	*CALN1*	.	.	.
7	71392256	G	A	intronic	*CALN1*	.	.	.
7	71561845	A	G	intronic	*CALN1*	.	.	.
7	71561990	C	T	intronic	*CALN1*	.	.	.
7	71612610	G	T	intronic	*CALN1*	.	.	.
7	71612762	A	G	intronic	*CALN1*	.	.	.
7	71761852	A	G	intronic	*CALN1*	.	.	.
10	12624479	T	C	intronic	*CAMK1D*	.	.	.
10	12901404	C	T	intergenic	*CAMK1D,CCDC3*	.	.	.
10	12901890	A	G	intergenic	*CAMK1D,CCDC3*	.	.	.
10	12902044	A	G	intergenic	*CAMK1D,CCDC3*	.	.	.
7	44374361	TC	T	intergenic	*CAMK2B,NUDCD3*	.	.	.
7	44394939	A	G	intergenic	*CAMK2B,NUDCD3*	.	.	.
4	114401563	A	G	intronic	*CAMK2D*	.	.	.
4	114544894	CTGTGTG	C	intronic	*CAMK2D*	.	.	.
10	75591221	G	A	intronic	*CAMK2G*	.	.	.
5	110693963	A	G	intronic	*CAMK4*	.	.	.

5	110592938	A	G	intronic	*CAMK4*	.	.	.
5	110592940	C	A	intronic	*CAMK4*	.	.	.
5	110755169	T	G	intronic	*CAMK4*	.	.	.
2	44986606	T	G	intronic	*CAMKMT*	.	.	.
2	44653849	T	G	intronic	*CAMKMT*	.	.	.
2	44789898	A	C	intronic	*CAMKMT*	.	.	.
2	44818609	G	A	intronic	*CAMKMT*	.	.	.
2	44821965	T	C	intronic	*CAMKMT*	.	.	.
2	44821966	G	T	intronic	*CAMKMT*	.	.	.
2	45162973	CGA	C	intergenic	*CAMKMT,SIX3-AS1*	.	.	.
2	45021541	G	A	intergenic	*CAMKMT,SIX3-AS1*	.	.	.
9	138736980	T	C	intronic	*CAMSAP1*	.	.	.
1	200768582	C	T	intronic	*CAMSAP2*	.	.	.
1	7122826	G	A	intronic	*CAMTA1*	.	.	.
1	7552617	ACG	A	intronic	*CAMTA1*	.	.	.
1	7659358	C	T	intronic	*CAMTA1*	.	.	.
1	7659839	C	T	intronic	*CAMTA1*	.	.	.
1	7662822	A	G	intronic	*CAMTA1*	.	.	.
1	7699241	G	A	intronic	*CAMTA1*	.	.	.
12	67819287	A	G	intergenic	*CAND1,LOC100507175*	.	.	.
12	67802405	A	T	intergenic	*CAND1,LOC100507175*	.	.	.
11	10364274	T	A	ncRNA_intronic	*CAND1.11*	.	.	.
11	10406079	A	T	ncRNA_intronic	*CAND1.11*	.	.	.
6	17441208	G	A	intronic	*CAP2*	.	.	.
6	17546655	T	A	intronic	*CAP2*	.	.	.
2	85650312	GTTT	G	intergenic	*CAPG,SH2D6*	.	.	.
6	44127908	T	C	intronic	*CAPN11*	.	.	.
2	31041948	A	C	intergenic	*CAPN13,GALNT14*	.	.	.
2	31073191	G	A	intergenic	*CAPN13,GALNT14*	.	.	.
2	31075747	T	C	intergenic	*CAPN13,GALNT14*	.	.	.
2	31075748	A	C	intergenic	*CAPN13,GALNT14*	.	.	.
2	31098671	G	A	intergenic	*CAPN13,GALNT14*	.	.	.
1	223956183	A	G	intronic	*CAPN2*	.	.	.
1	223831229	T	A	intronic	*CAPN8*	.	.	.

1	230958155	G	A	intergenic	*CAPN9,C1orf198*	.	.	.
19	36639541	C	T	intronic	*CAPNS1*	.	.	.
11	34079769	T	C	intronic	*CAPRIN1*	.	.	.
12	30903483	A	T	intronic	*CAPRIN2*	.	.	.
12	75707099	G	A	intronic	*CAPS2*	.	.	.
5	35938770	G	T	UTR5	*CAPSL*	.	.	.
5	35914973	C	G	intronic	*CAPSL*	.	.	.
5	35915702	C	A	intronic	*CAPSL*	.	.	.
12	18922064	A	G	intergenic	*CAPZA3,PLEKHA5*	.	.	.
12	19077345	T	C	intergenic	*CAPZA3,PLEKHA5*	.	.	.
12	19045905	C	T	intergenic	*CAPZA3,PLEKHA5*	.	.	.
12	19045907	A	T	intergenic	*CAPZA3,PLEKHA5*	.	.	.
12	19046868	G	T	intergenic	*CAPZA3,PLEKHA5*	.	.	.
12	19257985	C	T	intergenic	*CAPZA3,PLEKHA5*	.	.	.
1	19807139	A	C	intronic	*CAPZB*	.	.	.
22	37927951	G	T	intergenic	*CARD10,CDC42EP1*	.	.	.
7	2953442	G	GGCAAGA	intronic	*CARD11*	.	.	.
7	3041177	G	A	intronic	*CARD11*	.	.	.
7	3141063	T	A	intergenic	*CARD11,LOC100129603*	.	.	.
11	105413522	T	C	intergenic	*CARD18,GRIA4*	.	.	.
11	105224664	G	A	intergenic	*CARD18,GRIA4*	.	.	.
11	105241143	C	T	intergenic	*CARD18,GRIA4*	.	.	.
11	105165234	C	G	intergenic	*CARD18,GRIA4*	.	.	.
11	105165235	A	G	intergenic	*CARD18,GRIA4*	.	.	.
11	105286802	C	T	intergenic	*CARD18,GRIA4*	.	.	.
11	105286804	GT	G	intergenic	*CARD18,GRIA4*	.	.	.
11	105315644	A	AATTTTAATT	intergenic	*CARD18,GRIA4*	.	.	.
19	48762651	G	A	intergenic	*CARD8-AS1,ZNF114*	.	.	.
2	203810744	A	G	intronic	*CARF*	.	.	.
2	203812947	A	G	intronic	*CARF*	.	.	.
13	111287468	G	A	intronic	*CARKD*	.	.	.
5	148809668	GAGC	G	ncRNA_intronic	*CARMN*	.	.	.
9	77643057	G	A	exonic	*CARNMT1*	nonsynonymous_SNV	.	.
5	71388396	C	A	intergenic	*CARTPT,MAP1B*	.	.	.

5	71094984	T	C	intergenic	*CARTPT,MAP1B*	.	.	.
5	71224017	G	A	intergenic	*CARTPT,MAP1B*	.	.	.
5	71247892	C	T	intergenic	*CARTPT,MAP1B*	.	.	.
5	71348215	T	A	intergenic	*CARTPT,MAP1B*	.	.	.
5	71349174	A	T	intergenic	*CARTPT,MAP1B*	.	.	.
12	25326053	C	A	intronic	*CASC1*	.	.	.
12	25326054	C	A	intronic	*CASC1*	.	.	.
12	25266213	CTGTTGTT GTTGTTGT	C	intronic	*CASC1*	.	.	.
12	25327937	G	A	intronic	*CASC1*	.	.	.
6	22107629	T	A	ncRNA_intronic	*CASC15*	.	.	.
6	21694333	C	CT	ncRNA_intronic	*CASC15*	.	.	.
6	22185535	C	T	ncRNA_intronic	*CASC15*	.	.	.
6	22140113	C	T	ncRNA_intronic	*CASC15,NBAT1*	.	.	.
16	52846161	T	C	intergenic	*CASC16,CHD9*	.	.	.
16	52966759	T	A	intergenic	*CASC16,CHD9*	.	.	.
16	53066635	C	T	intergenic	*CASC16,CHD9*	.	.	.
16	52882025	A	G	intergenic	*CASC16,CHD9*	.	.	.
16	52933970	A	G	intergenic	*CASC16,CHD9*	.	.	.
16	52934652	A	C	intergenic	*CASC16,CHD9*	.	.	.
16	52980544	A	G	intergenic	*CASC16,CHD9*	.	.	.
17	69108989	C	A	ncRNA_intronic	*CASC17*	.	.	.
17	69131110	G	A	ncRNA_intronic	*CASC17*	.	.	.
17	69737381	G	A	intergenic	*CASC17,LOC1027 23505*	.	.	.
17	69967981	C	T	intergenic	*CASC17,LOC1027 23505*	.	.	.
17	69612845	T	C	intergenic	*CASC17,LOC1027 23505*	.	.	.
17	69730844	A	G	intergenic	*CASC17,LOC1027 23505*	.	.	.
17	69934838	G	A	intergenic	*CASC17,LOC1027 23505*	.	.	.
17	69205208	T	C	intergenic	*CASC17,LOC1027 23505*	.	.	.
17	69642242	T	C	intergenic	*CASC17,LOC1027 23505*	.	.	.

17	69808441	T	C	intergenic	*CASC17,LOC102723505*	.	.	.
17	69808442	T	A	intergenic	*CASC17,LOC102723505*	.	.	.
17	69840150	T	C	intergenic	*CASC17,LOC102723505*	.	.	.
12	106175668	T	A	intergenic	*CASC18,NUAK1*	.	.	.
10	119919669	A	T	ncRNA_intronic	*CASC2*	.	.	.
10	119826949	C	T	ncRNA_intronic	*CASC2*	.	.	.
10	119827590	C	G	ncRNA_intronic	*CASC2*	.	.	.
10	119984186	A	T	intergenic	*CASC2,FAM204A*	.	.	.
10	120061804	T	C	intergenic	*CASC2,FAM204A*	.	.	.
20	6506045	GGTGTGTGTGT	G	ncRNA_intronic	*CASC20*	.	.	.
17	38307244	C	T	intronic	*CASC3*	.	.	.
15	44653347	A	G	intronic	*CASC4*	.	.	.
15	40904615	G	A	intronic	*CASC5*	.	.	.
15	40972520	T	C	intergenic	*CASC5,RAD51-AS1*	.	.	.
6	92391455	T	C	ncRNA_intronic	*CASC6*	.	.	.
6	92393356	AG	A	ncRNA_intronic	*CASC6*	.	.	.
6	93254180	A	G	intergenic	*CASC6,EPHA7*	.	.	.
6	93381446	A	G	intergenic	*CASC6,EPHA7*	.	.	.
6	93114410	A	G	intergenic	*CASC6,EPHA7*	.	.	.
6	93242198	T	C	intergenic	*CASC6,EPHA7*	.	.	.
6	93397815	T	C	intergenic	*CASC6,EPHA7*	.	.	.
6	93761079	CA	C	intergenic	*CASC6,EPHA7*	.	.	.
6	92762751	T	C	intergenic	*CASC6,EPHA7*	.	.	.
6	92894712	G	A	intergenic	*CASC6,EPHA7*	.	.	.
6	92639230	C	A	intergenic	*CASC6,EPHA7*	.	.	.
6	92681291	G	A	intergenic	*CASC6,EPHA7*	.	.	.
6	93167848	G	C	intergenic	*CASC6,EPHA7*	.	.	.
6	92816561	T	C	intergenic	*CASC6,EPHA7*	.	.	.
6	93499193	T	A	intergenic	*CASC6,EPHA7*	.	.	.
6	93655014	G	T	intergenic	*CASC6,EPHA7*	.	.	.
6	93140538	G	A	intergenic	*CASC6,EPHA7*	.	.	.
6	93760543	T	TAAAAAAAA	intergenic	*CASC6,EPHA7*	.	.	.
6	92435871	C	T	intergenic	*CASC6,EPHA7*	.	.	.

6	92503967	A	G	intergenic	*CASC6,EPHA7*	.	.	.
6	92668020	C	T	intergenic	*CASC6,EPHA7*	.	.	.
6	92668489	T	C	intergenic	*CASC6,EPHA7*	.	.	.
6	92681805	T	C	intergenic	*CASC6,EPHA7*	.	.	.
6	92719865	T	A	intergenic	*CASC6,EPHA7*	.	.	.
6	92790217	A	T	intergenic	*CASC6,EPHA7*	.	.	.
6	92792731	T	C	intergenic	*CASC6,EPHA7*	.	.	.
6	93065416	T	G	intergenic	*CASC6,EPHA7*	.	.	.
6	93113272	T	TC	intergenic	*CASC6,EPHA7*	.	.	.
6	93133635	A	G	intergenic	*CASC6,EPHA7*	.	.	.
6	93334944	A	G	intergenic	*CASC6,EPHA7*	.	.	.
6	93336501	A	G	intergenic	*CASC6,EPHA7*	.	.	.
6	93350476	A	G	intergenic	*CASC6,EPHA7*	.	.	.
6	93361917	A	G	intergenic	*CASC6,EPHA7*	.	.	.
6	93442346	T	C	intergenic	*CASC6,EPHA7*	.	.	.
6	93442713	C	CTG	intergenic	*CASC6,EPHA7*	.	.	.
6	93581584	T	C	intergenic	*CASC6,EPHA7*	.	.	.
6	93891137	G	A	intergenic	*CASC6,EPHA7*	.	.	.
8	128557381	C	A	intergenic	*CASC8,CASC11*	.	.	.
8	128643080	AAAATATAAATAAATAAATAAATAAAT	A	intergenic	*CASC8,CASC11*	.	.	.
8	128567734	G	A	intergenic	*CASC8,CASC11*	.	.	.
8	128610550	T	C	intergenic	*CASC8,CASC11*	.	.	.
8	76428223	T	C	intergenic	*CASC9,HNF4G*	.	.	.
8	76268621	A	C	intergenic	*CASC9,HNF4G*	.	.	.
8	76268846	A	T	intergenic	*CASC9,HNF4G*	.	.	.
8	76269481	T	A	intergenic	*CASC9,HNF4G*	.	.	.
8	76269482	A	G	intergenic	*CASC9,HNF4G*	.	.	.
X	41750392	C	G	intronic	*CASK*	.	.	.
X	41424033	T	C	intronic	*CASK*	.	.	.
X	41424307	G	A	intronic	*CASK*	.	.	.
X	41426830	T	A	intronic	*CASK*	.	.	.
X	41501398	A	T	intronic	*CASK*	.	.	.
X	41670671	T	C	intronic	*CASK*	.	.	.
X	42522692	C	T	intergenic	*CASK,PPP1R2P9*	.	.	.
X	42031738	T	A	intergenic	*CASK,PPP1R2P9*	.	.	.

X	42181840	T	G	intergenic	*CASK,PPP1R2P9*	.	.	.
X	41868761	C	T	intergenic	*CASK,PPP1R2P9*	.	.	.
X	42032577	A	C	intergenic	*CASK,PPP1R2P9*	.	.	.
X	42063135	A	G	intergenic	*CASK,PPP1R2P9*	.	.	.
X	42168680	T	C	intergenic	*CASK,PPP1R2P9*	.	.	.
X	42168681	G	A	intergenic	*CASK,PPP1R2P9*	.	.	.
X	42170058	C	G	intergenic	*CASK,PPP1R2P9*	.	.	.
X	42170395	G	A	intergenic	*CASK,PPP1R2P9*	.	.	.
X	42172438	T	C	intergenic	*CASK,PPP1R2P9*	.	.	.
X	42196667	G	C	intergenic	*CASK,PPP1R2P9*	.	.	.
X	42198004	C	T	intergenic	*CASK,PPP1R2P9*	.	.	.
X	42200214	A	G	intergenic	*CASK,PPP1R2P9*	.	.	.
X	42307430	G	A	intergenic	*CASK,PPP1R2P9*	.	.	.
X	42471945	A	G	intergenic	*CASK,PPP1R2P9*	.	.	.
X	42513984	C	T	intergenic	*CASK,PPP1R2P9*	.	.	.
X	42567886	A	T	intergenic	*CASK,PPP1R2P9*	.	.	.
10	115460333	A	T	intronic	*CASP7*	.	.	.
10	115447605	T	TTTCTTTCTT	intronic	*CASP7*	.	.	.
1	116322729	T	C	intergenic	*CASQ2,NHLH2*	.	.	.
1	116323335	G	C	intergenic	*CASQ2,NHLH2*	.	.	.
20	55001094	C	T	intronic	*CASS4*	.	.	.
5	96043895	T	A	intronic	*CAST*	.	.	.
1	10726148	C	G	intronic	*CASZ1*	.	.	.
1	10895272	G	A	intergenic	*CASZ1,C1orf127*	.	.	.
5	134334570	C	T	intronic	*CATSPER3*	.	.	.
14	92143383	A	G	intronic	*CATSPERB*	.	.	.
14	92205938	G	A	intergenic	*CATSPERB,TC2N*	.	.	.
20	32099140	G	A	intronic	*CBFA2T2*	.	.	.
16	88986872	A	T	intronic	*CBFA2T3*	.	.	.
16	88996807	C	A	intronic	*CBFA2T3*	.	.	.
16	89012945	C	T	intronic	*CBFA2T3,LOC100129697*	.	.	.
3	105559071	C	T	intronic	*CBLB*	.	.	.
3	106111136	G	A	intergenic	*CBLB,LINC00882*	.	.	.
3	106427211	T	C	intergenic	*CBLB,LINC00882*	.	.	.
3	106118059	G	A	intergenic	*CBLB,LINC00882*	.	.	.
3	106333317	G	T	intergenic	*CBLB,LINC00882*	.	.	.
3	105590176	ATGTGTG	A	intergenic	*CBLB,LINC00882*	.	.	.

3	105856336	A	G	intergenic	*CBLB,LINC00882*	.	.	.
3	105869268	C	T	intergenic	*CBLB,LINC00882*	.	.	.
3	106038027	T	G	intergenic	*CBLB,LINC00882*	.	.	.
3	106257337	A	G	intergenic	*CBLB,LINC00882*	.	.	.
3	106257339	C	T	intergenic	*CBLB,LINC00882*	.	.	.
3	106621990	G	A	intergenic	*CBLB,LINC00882*	.	.	.
3	106665340	C	T	intergenic	*CBLB,LINC00882*	.	.	.
18	70343033	A	G	intergenic	*CBLN2,NETO1*	.	.	.
18	70406842	A	G	intergenic	*CBLN2,NETO1*	.	.	.
18	70342992	A	C	intergenic	*CBLN2,NETO1*	.	.	.
20	54630533	GT	G	intergenic	*CBLN4,MC3R*	.	.	.
20	54630535	G	C	intergenic	*CBLN4,MC3R*	.	.	.
20	54598220	G	A	intergenic	*CBLN4,MC3R*	.	.	.
20	54629437	G	A	intergenic	*CBLN4,MC3R*	.	.	.
20	54629438	G	A	intergenic	*CBLN4,MC3R*	.	.	.
20	54683821	G	C	intergenic	*CBLN4,MC3R*	.	.	.
9	157923	C	A	intronic	*CBWD1*	.	.	.
9	195581	C	T	intergenic	*CBWD1,C9orf66*	.	.	.
9	182630	T	C	intergenic	*CBWD1,C9orf66*	.	.	.
2	114209951	T	G	intronic	*CBWD2*	.	.	.
9	69338505	A	G	intergenic	*CBWD5,ANKRD20A4*	.	.	.
9	69338507	A	G	intergenic	*CBWD5,ANKRD20A4*	.	.	.
9	70578666	CATAT	C	intergenic	*CBWD5,CBWD5*	.	.	.
9	70701503	C	A	intergenic	*CBWD5,CBWD5*	.	.	.
22	39528030	C	T	UTR3	*CBX7*	.	.	.
22	39617888	A	T	intergenic	*CBX7,PDGFB*	.	.	.
22	39553419	T	A	intergenic	*CBX7,PDGFB*	.	.	.
19	14034337	A	G	intronic	*CC2D1A*	.	.	.
18	57149395	C	T	intronic	*CCBE1*	.	.	.
18	57440878	T	C	intergenic	*CCBE1,PMAIP1*	.	.	.
18	57563214	C	T	intergenic	*CCBE1,PMAIP1*	.	.	.
1	89467935	A	G	intergenic	*CCBL2,GBP3*	.	.	.
18	66424117	GGTGTGT	G	intronic	*CCDC102B*	.	.	.
18	66501669	CATAT	C	intronic	*CCDC102B*	.	.	.
18	66536895	T	C	intronic	*CCDC102B*	.	.	.
18	66536943	T	G	intronic	*CCDC102B*	.	.	.

18	66787311	G	A	intergenic	*CCDC102B,DOK6*	.	.	.
18	66997051	A	G	intergenic	*CCDC102B,DOK6*	.	.	.
18	66863306	A	G	intergenic	*CCDC102B,DOK6*	.	.	.
18	66729198	CATAT	C	intergenic	*CCDC102B,DOK6*	.	.	.
18	67026113	CACAG	C	intergenic	*CCDC102B,DOK6*	.	.	.
18	66843546	C	T	intergenic	*CCDC102B,DOK6*	.	.	.
18	66921185	G	T	intergenic	*CCDC102B,DOK6*	.	.	.
4	110483017	A	G	intronic	*CCDC109B*	.	.	.
5	114706144	T	C	intergenic	*CCDC112,FEM1C*	.	.	.
5	114778313	G	C	intergenic	*CCDC112,FEM1C*	.	.	.
5	114787881	G	A	intergenic	*CCDC112,FEM1C*	.	.	.
5	114736320	G	A	intergenic	*CCDC112,FEM1C*	.	.	.
5	114736321	C	A	intergenic	*CCDC112,FEM1C*	.	.	.
5	114777663	A	G	intergenic	*CCDC112,FEM1C*	.	.	.
5	114780096	T	C	intergenic	*CCDC112,FEM1C*	.	.	.
5	114780642	G	T	intergenic	*CCDC112,FEM1C*	.	.	.
5	114780644	A	T	intergenic	*CCDC112,FEM1C*	.	.	.
5	114782947	T	C	intergenic	*CCDC112,FEM1C*	.	.	.
13	44427572	C	G	intronic	*CCDC122*	.	.	.
7	23657129	A	G	intronic	*CCDC126*	.	.	.
7	23661984	T	C	intronic	*CCDC126*	.	.	.
7	31597820	C	A	intronic	*CCDC129*	.	.	.
7	31671139	AC	A	intronic	*CCDC129*	.	.	.
7	31627491	G	C	intronic	*CCDC129*	.	.	.
7	31677493	G	T	intronic	*CCDC129*	.	.	.
3	42808616	T	C	intronic	*CCDC13*	.	.	.
3	42779764	A	C	ncRNA_intronic	*CCDC13-AS1*	.	.	.
2	223182154	T	C	intergenic	*CCDC140,SGPP2*	.	.	.
2	223231152	G	A	intergenic	*CCDC140,SGPP2*	.	.	.
2	179910388	T	G	intronic	*CCDC141*	.	.	.
2	179901854	A	G	intronic	*CCDC141*	.	.	.
2	179914301	A	T	intronic	*CCDC141*	.	.	.
2	179907857	A	G	intronic	*CCDC141*	.	.	.
2	179926253	T	G	intergenic	*CCDC141,SESTD1*	.	.	.
2	179927165	T	G	intergenic	*CCDC141,SESTD1*	.	.	.
2	179943805	G	C	intergenic	*CCDC141,SESTD1*	.	.	.
2	179943807	C	G	intergenic	*CCDC141,SESTD1*	.	.	.
2	179945183	A	C	intergenic	*CCDC141,SESTD1*	.	.	.

17	16635843	T	C	intronic	*CCDC144A*	.	.	.
17	16666199	C	T	intronic	*CCDC144A*	.	.	.
17	18442517	T	G	ncRNA_exonic	*CCDC144B*	.	.	.
17	20771242	C	T	intronic	*CCDC144NL*	.	.	.
17	20785508	A	G	ncRNA_intronic	*CCDC144NL-AS1*	.	.	.
17	20820560	C	T	intergenic	*CCDC144NL-AS1,LOC339260*	.	.	.
7	76799811	T	C	intronic	*CCDC146*	.	.	.
7	76778031	A	G	intronic	*CCDC146*	.	.	.
2	159208416	G	C	intronic	*CCDC148*	.	.	.
2	159292390	G	C	intronic	*CCDC148*	.	.	.
2	159185399	G	T	intronic	*CCDC148*	.	.	.
2	159186313	T	C	intronic	*CCDC148*	.	.	.
2	159187081	G	T	intronic	*CCDC148*	.	.	.
2	159220124	C	CA	intronic	*CCDC148*	.	.	.
2	159225962	T	C	intronic	*CCDC148*	.	.	.
2	159255515	C	T	intronic	*CCDC148*	.	.	.
2	159257068	C	T	intronic	*CCDC148*	.	.	.
2	159257263	C	T	intronic	*CCDC148*	.	.	.
2	159257265	T	C	intronic	*CCDC148*	.	.	.
2	159277535	C	A	intronic	*CCDC148*	.	.	.
2	159292794	G	A	intronic	*CCDC148*	.	.	.
2	159293132	C	T	intronic	*CCDC148*	.	.	.
2	159293134	G	A	intronic	*CCDC148*	.	.	.
2	159294003	A	G	intronic	*CCDC148*	.	.	.
2	159084425	C	T	ncRNA_intronic	*CCDC148-AS1*	.	.	.
4	24944752	A	C	intronic	*CCDC149*	.	.	.
11	124832903	T	C	intronic	*CCDC15*	.	.	.
2	197555838	C	T	intronic	*CCDC150*	.	.	.
2	197602918	TC	T	intergenic	*CCDC150,GTF3C3*	.	.	.
2	197603151	C	T	intergenic	*CCDC150,GTF3C3*	.	.	.
2	197617648	A	T	intergenic	*CCDC150,GTF3C3*	.	.	.
2	197619809	A	G	intergenic	*CCDC150,GTF3C3*	.	.	.
2	197620705	G	A	intergenic	*CCDC150,GTF3C3*	.	.	.
2	197621958	C	A	intergenic	*CCDC150,GTF3C3*	.	.	.
2	197621960	A	G	intergenic	*CCDC150,GTF3C3*	.	.	.
4	77297507	G	C	intronic	*CCDC158*	.	.	.
X	133393552	A	G	intergenic	*CCDC160,PHF6*	.	.	.

X	133464548	A	C	intergenic	*CCDC160,PHF6*	.	.	.
X	133476958	G	T	intergenic	*CCDC160,PHF6*	.	.	.
X	133487380	A	T	intergenic	*CCDC160,PHF6*	.	.	.
X	133504070	A	G	intergenic	*CCDC160,PHF6*	.	.	.
X	133480566	G	A	intergenic	*CCDC160,PHF6*	.	.	.
13	36750900	T	C	intronic	*CCDC169-SOHLH2,SOHLH2*	.	.	.
13	36815455	C	G	intronic	*CCDC169,CCDC169-SOHLH2*	.	.	.
13	36844572	T	G	intronic	*CCDC169,CCDC169-SOHLH2*	.	.	.
9	15912266	CTTGTACCTTATATTCT	C	intronic	*CCDC171*	.	.	.
9	15767822	T	TCCCTCCCT(	intronic	*CCDC171*	.	.	.
9	15557346	C	T	intronic	*CCDC171*	.	.	.
9	15598097	C	G	intronic	*CCDC171*	.	.	.
9	15598553	T	A	intronic	*CCDC171*	.	.	.
9	15625061	T	C	intronic	*CCDC171*	.	.	.
9	15671971	C	A	intronic	*CCDC171*	.	.	.
9	15674380	C	T	intronic	*CCDC171*	.	.	.
9	15790691	T	A	intronic	*CCDC171*	.	.	.
9	15991186	A	T	intergenic	*CCDC171,C9orf92*	.	.	.
9	15992572	C	G	intergenic	*CCDC171,C9orf92*	.	.	.
9	16007090	C	T	intergenic	*CCDC171,C9orf92*	.	.	.
9	16007091	A	G	intergenic	*CCDC171,C9orf92*	.	.	.
9	16116045	T	C	intergenic	*CCDC171,C9orf92*	.	.	.
14	59983018	T	C	intronic	*CCDC175*	.	.	.
14	59983019	G	A	intronic	*CCDC175*	.	.	.
14	60021257	T	C	intronic	*CCDC175*	.	.	.
14	60023387	T	C	intronic	*CCDC175*	.	.	.
14	60045103	C	T	intergenic	*CCDC175,RTN1*	.	.	.
18	30693091	G	T	intronic	*CCDC178*	.	.	.
18	30829478	C	T	intronic	*CCDC178*	.	.	.
18	30994735	G	A	intronic	*CCDC178*	.	.	.
18	30994736	A	C	intronic	*CCDC178*	.	.	.
18	31110251	A	G	intergenic	*CCDC178,ASXL3*	.	.	.
11	23137123	C	G	intergenic	*CCDC179,MIR8054*	.	.	.

11	23137124	C	A	intergenic	*CCDC179,MIR8054*	.	.	.
11	23348064	T	C	intergenic	*CCDC179,MIR8054*	.	.	.
11	23010589	T	C	intergenic	*CCDC179,MIR8054*	.	.	.
11	23115637	C	G	intergenic	*CCDC179,MIR8054*	.	.	.
11	22940471	CTATA	C	intergenic	*CCDC179,MIR8054*	.	.	.
11	23128794	G	T	intergenic	*CCDC179,MIR8054*	.	.	.
11	23367387	T	C	intergenic	*CCDC179,MIR8054*	.	.	.
11	22951067	G	C	intergenic	*CCDC179,MIR8054*	.	.	.
11	23029808	G	A	intergenic	*CCDC179,MIR8054*	.	.	.
11	23239016	C	T	intergenic	*CCDC179,MIR8054*	.	.	.
11	23239549	C	A	intergenic	*CCDC179,MIR8054*	.	.	.
11	23239550	A	G	intergenic	*CCDC179,MIR8054*	.	.	.
11	23241347	A	C	intergenic	*CCDC179,MIR8054*	.	.	.
11	23241511	C	T	intergenic	*CCDC179,MIR8054*	.	.	.
11	23283515	C	A	intergenic	*CCDC179,MIR8054*	.	.	.
11	23301425	G	A	intergenic	*CCDC179,MIR8054*	.	.	.
11	23347659	G	A	intergenic	*CCDC179,MIR8054*	.	.	.
11	23431192	G	A	intergenic	*CCDC179,MIR8054*	.	.	.
11	23433094	T	A	intergenic	*CCDC179,MIR8054*	.	.	.

1	169379995	A	G	intronic	*CCDC181*	.	.	.
1	169374198	A	G	intronic	*CCDC181*	.	.	.
1	169379375	A	C	intronic	*CCDC181*	.	.	.
1	169381843	TGG	T	intronic	*CCDC181*	.	.	.
10	115882895	A	T	UTR3	*CCDC186*	.	.	.
1	162936372	T	C	intergenic	*CCDC190,RGS4*	.	.	.
8	130381368	C	T	ncRNA_intronic	*CCDC26*	.	.	.
8	130412840	G	A	ncRNA_intronic	*CCDC26*	.	.	.
8	130660282	A	T	ncRNA_intronic	*CCDC26*	.	.	.
8	130747952	C	A	intergenic	*CCDC26,GSDMC*	.	.	.
10	13128648	G	A	intronic	*CCDC3*	.	.	.
1	43027892	T	G	intronic	*CCDC30*	.	.	.
3	49261806	A	G	intronic	*CCDC36*	.	.	.
17	78043808	G	A	intronic	*CCDC40*	.	.	.
12	102440199	G	A	intronic	*CCDC53*	.	.	.
12	119807216	A	G	intronic	*CCDC60*	.	.	.
12	119820985	G	A	intronic	*CCDC60*	.	.	.
12	119973501	A	T	intronic	*CCDC60*	.	.	.
12	119974134	A	C	intronic	*CCDC60*	.	.	.
12	120029918	C	A	intergenic	*CCDC60,TMEM233*	.	.	.
12	111313272	A	G	intronic	*CCDC63*	.	.	.
3	56611408	T	C	intronic	*CCDC66*	.	.	.
3	56633996	T	G	intronic	*CCDC66*	.	.	.
3	56636142	G	A	intronic	*CCDC66*	.	.	.
11	93151088	C	A	intronic	*CCDC67*	.	.	.
11	93173269	T	A	intergenic	*CCDC67,SMCO4*	.	.	.
11	93174985	G	T	intergenic	*CCDC67,SMCO4*	.	.	.
11	93195654	A	C	intergenic	*CCDC67,SMCO4*	.	.	.
18	52674285	A	T	intergenic	*CCDC68,LOC101927229*	.	.	.
10	32772419	G	T	intronic	*CCDC7*	.	.	.
10	32814167	C	T	intronic	*CCDC7*	.	.	.
10	32851504	G	A	intronic	*CCDC7*	.	.	.
10	32875314	T	C	intronic	*CCDC7*	.	.	.
10	32875315	T	C	intronic	*CCDC7*	.	.	.
10	32891211	G	A	intronic	*CCDC7*	.	.	.
10	32926021	G	A	intronic	*CCDC7*	.	.	.
10	32955990	A	G	intronic	*CCDC7*	.	.	.

10	32959571	C	T	intronic	*CCDC7*	.	.	.
10	32991936	T	C	intronic	*CCDC7*	.	.	.
10	32995025	C	A	intronic	*CCDC7*	.	.	.
10	32995027	A	C	intronic	*CCDC7*	.	.	.
10	32995313	T	C	intronic	*CCDC7*	.	.	.
10	33006008	C	T	intronic	*CCDC7*	.	.	.
10	33006629	C	A	intronic	*CCDC7*	.	.	.
10	33006734	AGCTTT	A	intronic	*CCDC7*	.	.	.
10	33048772	T	A	intronic	*CCDC7*	.	.	.
10	33125553	A	G	intronic	*CCDC7*	.	.	.
10	33128697	G	A	intronic	*CCDC7*	.	.	.
7	106313562	A	G	intergenic	*CCDC71L,PIK3CG*	.	.	.
7	106484150	G	T	intergenic	*CCDC71L,PIK3CG*	.	.	.
7	106314559	T	A	intergenic	*CCDC71L,PIK3CG*	.	.	.
7	106314807	T	C	intergenic	*CCDC71L,PIK3CG*	.	.	.
7	106315407	C	T	intergenic	*CCDC71L,PIK3CG*	.	.	.
7	106316068	G	A	intergenic	*CCDC71L,PIK3CG*	.	.	.
7	106327085	C	T	intergenic	*CCDC71L,PIK3CG*	.	.	.
7	106328470	A	G	intergenic	*CCDC71L,PIK3CG*	.	.	.
7	106353126	C	A	intergenic	*CCDC71L,PIK3CG*	.	.	.
7	106353127	G	A	intergenic	*CCDC71L,PIK3CG*	.	.	.
7	106354925	C	A	intergenic	*CCDC71L,PIK3CG*	.	.	.
7	106364875	G	A	intergenic	*CCDC71L,PIK3CG*	.	.	.
7	106381814	TAAAATA	T	intergenic	*CCDC71L,PIK3CG*	.	.	.
7	106395648	C	T	intergenic	*CCDC71L,PIK3CG*	.	.	.
11	32646111	C	T	intronic	*CCDC73*	.	.	.
11	32684167	T	C	intronic	*CCDC73*	.	.	.
11	32684815	T	G	intronic	*CCDC73*	.	.	.
11	32756356	A	G	intronic	*CCDC73*	.	.	.
11	32757928	C	T	intronic	*CCDC73*	.	.	.
11	32763539	C	A	intronic	*CCDC73*	.	.	.
11	32764117	A	G	intronic	*CCDC73*	.	.	.
11	32767110	G	C	intronic	*CCDC73*	.	.	.
11	32788478	A	G	intronic	*CCDC73*	.	.	.
2	132309966	A	T	intergenic	*CCDC74A,POTEKP*	.	.	.
2	132309968	C	A	intergenic	*CCDC74A,POTEKP*	.	.	.

2	132319322	G	A	intergenic	*CCDC74A,POTEKP*	.	.	.
12	543707	G	T	intronic	*CCDC77*	.	.	.
3	112381150	T	C	intergenic	*CCDC80,LOC101929694*	.	.	.
3	112381151	G	T	intergenic	*CCDC80,LOC101929694*	.	.	.
11	86109846	T	C	intronic	*CCDC81*	.	.	.
11	85637237	C	T	intergenic	*CCDC83,PICALM*	.	.	.
11	85651291	C	A	intergenic	*CCDC83,PICALM*	.	.	.
11	85651403	A	C	intergenic	*CCDC83,PICALM*	.	.	.
11	85651405	T	C	intergenic	*CCDC83,PICALM*	.	.	.
2	56548138	C	T	intronic	*CCDC85A*	.	.	.
2	56902721	G	T	intergenic	*CCDC85A,VRK2*	.	.	.
2	57211627	C	T	intergenic	*CCDC85A,VRK2*	.	.	.
2	57530546	C	A	intergenic	*CCDC85A,VRK2*	.	.	.
2	57012421	GTATA	G	intergenic	*CCDC85A,VRK2*	.	.	.
2	57532636	C	A	intergenic	*CCDC85A,VRK2*	.	.	.
2	57347942	CAA	C	intergenic	*CCDC85A,VRK2*	.	.	.
2	57541865	T	C	intergenic	*CCDC85A,VRK2*	.	.	.
2	57509592	G	T	intergenic	*CCDC85A,VRK2*	.	.	.
2	56844432	ATATT	A	intergenic	*CCDC85A,VRK2*	.	.	.
2	57754287	ATCTC	A	intergenic	*CCDC85A,VRK2*	.	.	.
2	57884071	G	A	intergenic	*CCDC85A,VRK2*	.	.	.
2	56650329	A	C	intergenic	*CCDC85A,VRK2*	.	.	.
2	56780473	A	G	intergenic	*CCDC85A,VRK2*	.	.	.
2	56821326	G	A	intergenic	*CCDC85A,VRK2*	.	.	.
2	56860185	T	G	intergenic	*CCDC85A,VRK2*	.	.	.
2	56860726	C	T	intergenic	*CCDC85A,VRK2*	.	.	.
2	56861767	C	A	intergenic	*CCDC85A,VRK2*	.	.	.
2	56889719	C	T	intergenic	*CCDC85A,VRK2*	.	.	.
2	56953053	T	C	intergenic	*CCDC85A,VRK2*	.	.	.
2	56953282	C	T	intergenic	*CCDC85A,VRK2*	.	.	.
2	56987015	G	A	intergenic	*CCDC85A,VRK2*	.	.	.
2	56987642	C	A	intergenic	*CCDC85A,VRK2*	.	.	.
2	57045641	A	T	intergenic	*CCDC85A,VRK2*	.	.	.
2	57048880	T	C	intergenic	*CCDC85A,VRK2*	.	.	.
2	57121053	C	A	intergenic	*CCDC85A,VRK2*	.	.	.

2	57121788	T	G	intergenic	*CCDC85A,VRK2*	.	.	.
2	57142987	G	C	intergenic	*CCDC85A,VRK2*	.	.	.
2	57143849	T	C	intergenic	*CCDC85A,VRK2*	.	.	.
2	57226809	T	G	intergenic	*CCDC85A,VRK2*	.	.	.
2	57294689	T	C	intergenic	*CCDC85A,VRK2*	.	.	.
2	57324251	G	C	intergenic	*CCDC85A,VRK2*	.	.	.
2	57498647	C	T	intergenic	*CCDC85A,VRK2*	.	.	.
2	57499658	T	C	intergenic	*CCDC85A,VRK2*	.	.	.
2	57499659	G	A	intergenic	*CCDC85A,VRK2*	.	.	.
2	57512599	A	C	intergenic	*CCDC85A,VRK2*	.	.	.
2	57534072	T	A	intergenic	*CCDC85A,VRK2*	.	.	.
2	57536090	G	T	intergenic	*CCDC85A,VRK2*	.	.	.
2	57620524	TATAAATAA	T	intergenic	*CCDC85A,VRK2*	.	.	.
2	57647795	A	G	intergenic	*CCDC85A,VRK2*	.	.	.
2	57651798	C	T	intergenic	*CCDC85A,VRK2*	.	.	.
2	57758574	T	A	intergenic	*CCDC85A,VRK2*	.	.	.
2	57819516	G	C	intergenic	*CCDC85A,VRK2*	.	.	.
2	57832924	A	G	intergenic	*CCDC85A,VRK2*	.	.	.
2	57838998	G	A	intergenic	*CCDC85A,VRK2*	.	.	.
2	57840943	G	A	intergenic	*CCDC85A,VRK2*	.	.	.
2	57875730	T	C	intergenic	*CCDC85A,VRK2*	.	.	.
2	57957427	C	T	intergenic	*CCDC85A,VRK2*	.	.	.
2	58059345	G	A	intergenic	*CCDC85A,VRK2*	.	.	.
2	55691395	G	A	intergenic	*CCDC88A,CFAP36*	.	.	.
2	55693701	C	T	intergenic	*CCDC88A,CFAP36*	.	.	.
11	82979600	T	C	intronic	*CCDC90B*	.	.	.
12	28697605	G	T	intronic	*CCDC91*	.	.	.
12	28427992	T	A	intronic	*CCDC91*	.	.	.
12	28428335	C	T	intronic	*CCDC91*	.	.	.
12	28428959	A	G	intronic	*CCDC91*	.	.	.
12	28630315	T	C	intronic	*CCDC91*	.	.	.
12	28630316	G	A	intronic	*CCDC91*	.	.	.
12	28631037	G	A	intronic	*CCDC91*	.	.	.
12	28631038	G	A	intronic	*CCDC91*	.	.	.
12	28676012	A	G	intronic	*CCDC91*	.	.	.
12	28917331	C	A	intergenic	*CCDC91,FAR2*	.	.	.
12	29291742	G	T	intergenic	*CCDC91,FAR2*	.	.	.

12	28827357	C	T	intergenic	*CCDC91,FAR2*	.	.	.
12	28829636	T	C	intergenic	*CCDC91,FAR2*	.	.	.
12	29154755	C	T	intergenic	*CCDC91,FAR2*	.	.	.
12	29170734	C	T	intergenic	*CCDC91,FAR2*	.	.	.
12	29247579	G	C	intergenic	*CCDC91,FAR2*	.	.	.
12	29248065	T	C	intergenic	*CCDC91,FAR2*	.	.	.
12	29248368	G	A	intergenic	*CCDC91,FAR2*	.	.	.
3	42348601	A	G	intergenic	*CCK,LYZL4*	.	.	.
3	42326215	G	A	intergenic	*CCK,LYZL4*	.	.	.
3	42326933	C	T	intergenic	*CCK,LYZL4*	.	.	.
4	26494416	A	C	intergenic	*CCKAR,TBC1D19*	.	.	.
4	26578904	CGTGT	C	intergenic	*CCKAR,TBC1D19*	.	.	.
17	32792796	T	C	intergenic	*CCL1,C17orf102*	.	.	.
17	32715225	G	C	intergenic	*CCL1,C17orf102*	.	.	.
17	32584630	C	G	downstream	*CCL2*	.	.	.
17	34348891	A	AAT	intergenic	*CCL23,CCL18*	.	.	.
7	75451314	G	A	intergenic	*CCL24,RHBDD2*	.	.	.
5	43404367	C	T	intronic	*CCL28*	.	.	.
5	43405411	A	AG	intronic	*CCL28*	.	.	.
17	34506652	G	A	intergenic	*CCL4,CCL3L3*	.	.	.
17	34199019	G	A	UTR3	*CCL5*	.	.	.
13	37229026	C	T	intergenic	*CCNA1,SERTM1*	.	.	.
13	37229027	A	G	intergenic	*CCNA1,SERTM1*	.	.	.
15	59420814	G	A	intergenic	*CCNB2,MYO1E*	.	.	.
6	100006271	A	G	intronic	*CCNC*	.	.	.
6	42014696	A	C	intronic	*CCND3*	.	.	.
19	30374503	C	G	intergenic	*CCNE1,URI1*	.	.	.
4	78179308	A	G	intergenic	*CCNG2,CXCL13*	.	.	.
4	78218355	C	T	intergenic	*CCNG2,CXCL13*	.	.	.
4	78246526	T	G	intergenic	*CCNG2,CXCL13*	.	.	.
4	78246527	T	C	intergenic	*CCNG2,CXCL13*	.	.	.
4	78382602	G	A	intergenic	*CCNG2,CXCL13*	.	.	.
4	78408544	G	A	intergenic	*CCNG2,CXCL13*	.	.	.
5	87458690	G	T	intergenic	*CCNH,TMEM161B*	.	.	.
5	86942255	G	T	intergenic	*CCNH,TMEM161B*	.	.	.
5	87293130	AT	A	intergenic	*CCNH,TMEM161B*	.	.	.
5	87154865	C	G	intergenic	*CCNH,TMEM161B*	.	.	.
5	87276829	G	C	intergenic	*CCNH,TMEM161B*	.	.	.

5	86784027	C	A	intergenic	*CCNH,TMEM161B*	.	.	.
5	86762956	A	T	intergenic	*CCNH,TMEM161B*	.	.	.
5	86769871	G	A	intergenic	*CCNH,TMEM161B*	.	.	.
5	86770826	G	T	intergenic	*CCNH,TMEM161B*	.	.	.
5	86771466	T	G	intergenic	*CCNH,TMEM161B*	.	.	.
5	86771467	A	C	intergenic	*CCNH,TMEM161B*	.	.	.
5	86771845	T	G	intergenic	*CCNH,TMEM161B*	.	.	.
5	86780259	A	C	intergenic	*CCNH,TMEM161B*	.	.	.
5	86781135	A	T	intergenic	*CCNH,TMEM161B*	.	.	.
5	86781825	T	C	intergenic	*CCNH,TMEM161B*	.	.	.
5	86813124	T	A	intergenic	*CCNH,TMEM161B*	.	.	.
5	86813738	A	G	intergenic	*CCNH,TMEM161B*	.	.	.
5	86844034	C	A	intergenic	*CCNH,TMEM161B*	.	.	.
5	86844036	A	T	intergenic	*CCNH,TMEM161B*	.	.	.
5	86845619	A	C	intergenic	*CCNH,TMEM161B*	.	.	.
5	86845832	A	C	intergenic	*CCNH,TMEM161B*	.	.	.
5	86861278	C	A	intergenic	*CCNH,TMEM161B*	.	.	.
5	86861279	C	T	intergenic	*CCNH,TMEM161B*	.	.	.
5	86921193	C	T	intergenic	*CCNH,TMEM161B*	.	.	.
5	86921195	A	G	intergenic	*CCNH,TMEM161B*	.	.	.
5	86988673	T	C	intergenic	*CCNH,TMEM161B*	.	.	.
5	86988907	T	TG	intergenic	*CCNH,TMEM161B*	.	.	.
5	86988908	A	G	intergenic	*CCNH,TMEM161B*	.	.	.
5	86992106	A	C	intergenic	*CCNH,TMEM161B*	.	.	.
5	87215463	T	C	intergenic	*CCNH,TMEM161B*	.	.	.
5	87218170	G	A	intergenic	*CCNH,TMEM161B*	.	.	.
5	87218172	G	A	intergenic	*CCNH,TMEM161B*	.	.	.
5	87218387	A	G	intergenic	*CCNH,TMEM161B*	.	.	.
5	87289251	A	AC	intergenic	*CCNH,TMEM161B*	.	.	.
5	87289946	C	T	intergenic	*CCNH,TMEM161B*	.	.	.
5	87307193	T	C	intergenic	*CCNH,TMEM161B*	.	.	.
5	87386404	A	ACGCGCG	intergenic	*CCNH,TMEM161B*	.	.	.
5	87402519	A	C	intergenic	*CCNH,TMEM161B*	.	.	.
5	87460187	A	G	intergenic	*CCNH,TMEM161B*	.	.	.
5	87464687	A	G	intergenic	*CCNH,TMEM161B*	.	.	.
5	87464923	A	G	intergenic	*CCNH,TMEM161B*	.	.	.
5	87464924	A	C	intergenic	*CCNH,TMEM161B*	.	.	.
5	87465891	T	G	intergenic	*CCNH,TMEM161B*	.	.	.

5	87465892	T	G	intergenic	*CCNH,TMEM161B*	.	.	.
5	87466435	T	G	intergenic	*CCNH,TMEM161B*	.	.	.
5	87466436	T	A	intergenic	*CCNH,TMEM161B*	.	.	.
5	87484150	T	C	intergenic	*CCNH,TMEM161B*	.	.	.
5	54547517	C	A	intergenic	*CCNO,DHX29*	.	.	.
2	135695108	T	G	intronic	*CCNT2*	.	.	.
2	135670219	G	GGT	ncRNA_intronic	*CCNT2-AS1*	.	.	.
10	35764459	C	T	intronic	*CCNY*	.	.	.
10	35765659	A	G	intronic	*CCNY*	.	.	.
10	42938659	T	C	ncRNA_intronic	*CCNYL2*	.	.	.
3	33018666	A	G	intergenic	*CCR4,GLB1*	.	.	.
3	46475764	G	T	intergenic	*CCRL2,LTF*	.	.	.
1	229549825	T	C	intergenic	*CCSAP,ACTA1*	.	.	.
4	91117944	A	G	intronic	*CCSER1*	.	.	.
4	91622934	T	C	intronic	*CCSER1*	.	.	.
4	91845399	T	C	intronic	*CCSER1*	.	.	.
4	92471312	A	G	intronic	*CCSER1*	.	.	.
4	91710158	TACAC	T	intronic	*CCSER1*	.	.	.
4	92124142	A	C	intronic	*CCSER1*	.	.	.
4	91076774	A	C	intronic	*CCSER1*	.	.	.
4	91076989	A	G	intronic	*CCSER1*	.	.	.
4	91125932	A	G	intronic	*CCSER1*	.	.	.
4	91225041	G	A	intronic	*CCSER1*	.	.	.
4	91245986	C	A	intronic	*CCSER1*	.	.	.
4	91329772	C	A	intronic	*CCSER1*	.	.	.
4	91401259	G	T	intronic	*CCSER1*	.	.	.
4	91411982	C	T	intronic	*CCSER1*	.	.	.
4	91412863	C	A	intronic	*CCSER1*	.	.	.
4	91622138	G	A	intronic	*CCSER1*	.	.	.
4	91622556	T	A	intronic	*CCSER1*	.	.	.
4	91622557	A	G	intronic	*CCSER1*	.	.	.
4	91623682	C	A	intronic	*CCSER1*	.	.	.
4	91623683	A	G	intronic	*CCSER1*	.	.	.
4	91624352	A	T	intronic	*CCSER1*	.	.	.
4	91784108	T	A	intronic	*CCSER1*	.	.	.
4	91784109	A	C	intronic	*CCSER1*	.	.	.
4	91834969	CAA	C	intronic	*CCSER1*	.	.	.
4	91835707	C	T	intronic	*CCSER1*	.	.	.

4	91838142	T	C	intronic	*CCSER1*	.	.	.
4	92097740	A	T	intronic	*CCSER1*	.	.	.
4	92098183	C	T	intronic	*CCSER1*	.	.	.
4	92099125	C	T	intronic	*CCSER1*	.	.	.
4	92404210	G	A	intronic	*CCSER1*	.	.	.
4	93041430	T	C	intergenic	*CCSER1,LOC101929194*	.	.	.
4	92545302	G	GTTATCACT(	intergenic	*CCSER1,LOC101929194*	.	.	.
4	92957053	G	T	intergenic	*CCSER1,LOC101929194*	.	.	.
4	92711300	A	T	intergenic	*CCSER1,LOC101929194*	.	.	.
4	92782251	A	T	intergenic	*CCSER1,LOC101929194*	.	.	.
4	93039831	G	C	intergenic	*CCSER1,LOC101929194*	.	.	.
4	93070142	A	G	intergenic	*CCSER1,LOC101929194*	.	.	.
4	93070327	G	A	intergenic	*CCSER1,LOC101929194*	.	.	.
4	93123816	A	T	intergenic	*CCSER1,LOC101929194*	.	.	.
4	93171926	T	C	intergenic	*CCSER1,LOC101929194*	.	.	.
4	93172702	GTTTT	G	intergenic	*CCSER1,LOC101929194*	.	.	.
4	93173028	T	C	intergenic	*CCSER1,LOC101929194*	.	.	.
4	93173029	G	A	intergenic	*CCSER1,LOC101929194*	.	.	.
4	93173050	A	T	intergenic	*CCSER1,LOC101929194*	.	.	.
4	93173051	C	T	intergenic	*CCSER1,LOC101929194*	.	.	.
10	86178475	T	C	intronic	*CCSER2*	.	.	.
10	86246185	T	C	intronic	*CCSER2*	.	.	.
10	86246683	A	G	intronic	*CCSER2*	.	.	.

10	86729622	C	A	intergenic	*CCSER2,LINC01519*	.	.	.
10	86312282	A	C	intergenic	*CCSER2,LINC01519*	.	.	.
10	86910170	G	C	intergenic	*CCSER2,LINC01519*	.	.	.
10	86399747	C	T	intergenic	*CCSER2,LINC01519*	.	.	.
10	86410153	C	T	intergenic	*CCSER2,LINC01519*	.	.	.
10	86489346	G	C	intergenic	*CCSER2,LINC01519*	.	.	.
10	86491910	T	C	intergenic	*CCSER2,LINC01519*	.	.	.
10	86536251	C	A	intergenic	*CCSER2,LINC01519*	.	.	.
10	86631036	C	A	intergenic	*CCSER2,LINC01519*	.	.	.
10	86677875	T	C	intergenic	*CCSER2,LINC01519*	.	.	.
10	86677876	C	A	intergenic	*CCSER2,LINC01519*	.	.	.
12	69998221	C	T	intergenic	*CCT2,LRRC10*	.	.	.
2	62128258	C	T	intergenic	*CCT4,COMMD1*	.	.	.
17	33284382	C	T	intronic	*CCT6B*	.	.	.
7	64617474	A	G	intergenic	*CCT6P3,ZNF92*	.	.	.
7	64746794	T	G	intergenic	*CCT6P3,ZNF92*	.	.	.
7	6853669	T	C	intronic	*CCZ1B*	.	.	.
7	7051246	A	ATAG	intergenic	*CCZ1B,MIR3683*	.	.	.
7	6885232	C	A	intergenic	*CCZ1B,MIR3683*	.	.	.
7	6867892	G	T	intergenic	*CCZ1B,MIR3683*	.	.	.
7	6867894	A	C	intergenic	*CCZ1B,MIR3683*	.	.	.
7	7013525	T	C	intergenic	*CCZ1B,MIR3683*	.	.	.
6	74746795	G	T	intergenic	*CD109,LOC101928516*	.	.	.
6	74543700	C	T	intergenic	*CD109,LOC101928516*	.	.	.

6	74556874	A	G	intergenic	*CD109,LOC101928516*	.	.	.
6	74557612	C	T	intergenic	*CD109,LOC101928516*	.	.	.
6	74626763	A	G	intergenic	*CD109,LOC101928516*	.	.	.
6	74630794	A	C	intergenic	*CD109,LOC101928516*	.	.	.
6	74718643	A	T	intergenic	*CD109,LOC101928516*	.	.	.
6	74743000	A	C	intergenic	*CD109,LOC101928516*	.	.	.
12	7630324	A	T	intronic	*CD163*	.	.	.
12	7656347	C	T	UTR5	*CD163*	.	.	.
12	7629660	T	C	intronic	*CD163*	.	.	.
12	7663864	G	GAGAGAGA(	intergenic	*CD163,APOBEC1*	.	.	.
12	7657925	A	G	intergenic	*CD163,APOBEC1*	.	.	.
12	7660650	C	T	intergenic	*CD163,APOBEC1*	.	.	.
12	7683515	G	A	intergenic	*CD163,APOBEC1*	.	.	.
12	7685668	T	A	intergenic	*CD163,APOBEC1*	.	.	.
12	7698874	G	A	intergenic	*CD163,APOBEC1*	.	.	.
12	7699043	G	A	intergenic	*CD163,APOBEC1*	.	.	.
12	7699045	T	C	intergenic	*CD163,APOBEC1*	.	.	.
12	7714873	T	C	intergenic	*CD163,APOBEC1*	.	.	.
12	7714992	T	C	intergenic	*CD163,APOBEC1*	.	.	.
12	7715187	C	T	intergenic	*CD163,APOBEC1*	.	.	.
12	7751279	T	C	intergenic	*CD163,APOBEC1*	.	.	.
12	7560672	CATAT	C	intronic	*CD163L1*	.	.	.
12	7547143	A	T	intronic	*CD163L1*	.	.	.
12	7569919	T	C	intronic	*CD163L1*	.	.	.
12	7576354	C	A	intronic	*CD163L1*	.	.	.
12	7618154	G	A	intergenic	*CD163L1,CD163*	.	.	.
5	66806191	A	G	intergenic	*CD180,LOC101928858*	.	.	.
5	66607657	A	T	intergenic	*CD180,LOC101928858*	.	.	.
5	66555927	T	A	intergenic	*CD180,LOC101928858*	.	.	.

5	66699638	T	C	intergenic	*CD180,LOC101928858*	.	.	.
5	66887902	A	T	intergenic	*CD180,LOC101928858*	.	.	.
5	66925438	G	T	intergenic	*CD180,LOC101928858*	.	.	.
5	66977521	C	T	intergenic	*CD180,LOC101928858*	.	.	.
5	67002873	G	A	intergenic	*CD180,LOC101928858*	.	.	.
5	67057053	G	A	intergenic	*CD180,LOC101928858*	.	.	.
5	67057227	A	G	intergenic	*CD180,LOC101928858*	.	.	.
5	67075103	C	A	intergenic	*CD180,LOC101928858*	.	.	.
1	158268566	G	A	intergenic	*CD1C,CD1B*	.	.	.
1	158269652	A	T	intergenic	*CD1C,CD1B*	.	.	.
1	158288235	A	G	intergenic	*CD1C,CD1B*	.	.	.
1	158289367	C	A	intergenic	*CD1C,CD1B*	.	.	.
1	158186608	A	T	intergenic	*CD1D,CD1A*	.	.	.
1	158359607	CA	C	intergenic	*CD1E,OR10T2*	.	.	.
1	158357465	T	C	intergenic	*CD1E,OR10T2*	.	.	.
1	158357467	A	T	intergenic	*CD1E,OR10T2*	.	.	.
1	158357613	G	C	intergenic	*CD1E,OR10T2*	.	.	.
2	71075024	G	C	intergenic	*CD207,LINC01143*	.	.	.
2	71107867	C	G	intergenic	*CD207,LINC01143*	.	.	.
2	71108519	T	C	intergenic	*CD207,LINC01143*	.	.	.
18	67598583	T	C	intronic	*CD226*	.	.	.
1	160823003	G	T	intronic	*CD244*	.	.	.
1	167462255	A	T	intronic	*CD247*	.	.	.
11	66083318	C	T	exonic	*CD248*	nonsynonymous_SNV	.	.
9	5472930	A	T	intergenic	*CD274,PDCD1LG2*	.	.	.
9	5475507	C	T	intergenic	*CD274,PDCD1LG2*	.	.	.
2	204729878	G	A	intergenic	*CD28,CTLA4*	.	.	.
6	47489177	A	G	intronic	*CD2AP*	.	.	.
6	47473350	CG	C	intronic	*CD2AP*	.	.	.

6	47473352	G	A	intronic	*CD2AP*	.	.	.
6	47584074	C	A	intronic	*CD2AP*	.	.	.
6	47478859	T	A	intronic	*CD2AP*	.	.	.
6	47525902	T	A	intronic	*CD2AP*	.	.	.
6	47526357	T	C	intronic	*CD2AP*	.	.	.
6	47526827	T	G	intronic	*CD2AP*	.	.	.
6	47534562	A	G	intronic	*CD2AP*	.	.	.
17	72495470	A	C	intergenic	*CD300A,CD300LB*	.	.	.
17	72535015	T	C	intergenic	*CD300LB,CD300C*	.	.	.
19	51729229	C	T	exonic	*CD33*	nonsynonymous_SNV	.	.
7	80322588	T	C	intergenic	*CD36,SEMA3C*	.	.	.
7	80324119	G	T	intergenic	*CD36,SEMA3C*	.	.	.
4	15813232	C	G	intronic	*CD38*	.	.	.
4	15822360	G	A	intronic	*CD38*	.	.	.
4	15823170	T	C	intronic	*CD38*	.	.	.
4	15847540	C	T	intronic	*CD38*	.	.	.
1	207945633	G	A	intronic	*CD46*	.	.	.
1	207945634	T	A	intronic	*CD46*	.	.	.
1	207947455	G	T	intronic	*CD46*	.	.	.
1	207952029	C	T	intronic	*CD46*	.	.	.
3	107812244	T	C	intergenic	*CD47,LINC01215*	.	.	.
11	33749860	G	A	intronic	*CD59*	.	.	.
12	56133428	C	CTA	intergenic	*CD63,GDF11*	.	.	.
12	9970245	A	G	intergenic	*CD69,KLRF1*	.	.	.
5	149787442	C	T	intronic	*CD74*	.	.	.
3	119271777	T	C	intronic	*CD80*	.	.	.
1	160552505	T	G	intergenic	*CD84,SLAMF1*	.	.	.
1	160553094	C	T	intergenic	*CD84,SLAMF1*	.	.	.
12	6414725	CTA	C	intergenic	*CD9,PLEKHG6*	.	.	.
12	6380150	C	T	intergenic	*CD9,PLEKHG6*	.	.	.
3	111363568	A	C	intronic	*CD96*	.	.	.
10	12326398	C	T	intergenic	*CDC123,CAMK1D*	.	.	.
1	100871937	C	T	intronic	*CDC14A*	.	.	.
7	49307095	G	A	intergenic	*CDC14C,VWC2*	.	.	.
7	49111724	G	A	intergenic	*CDC14C,VWC2*	.	.	.
7	49149003	A	G	intergenic	*CDC14C,VWC2*	.	.	.
7	49171829	G	T	intergenic	*CDC14C,VWC2*	.	.	.

7	49705088	G	A	intergenic	*CDC14C,VWC2*	.	.	.
7	49762282	C	A	intergenic	*CDC14C,VWC2*	.	.	.
7	49810393	AGGAAGGGAGGGGAGGGGAGGGGAAGGGAGGGGAGGGGAGG	A	intergenic	*CDC14C,VWC2*	.	.	.
7	49084374	G	A	intergenic	*CDC14C,VWC2*	.	.	.
7	48989391	C	A	intergenic	*CDC14C,VWC2*	.	.	.
7	49044463	T	C	intergenic	*CDC14C,VWC2*	.	.	.
7	49044771	A	T	intergenic	*CDC14C,VWC2*	.	.	.
7	49046914	A	G	intergenic	*CDC14C,VWC2*	.	.	.
7	49064736	T	C	intergenic	*CDC14C,VWC2*	.	.	.
7	49064737	G	A	intergenic	*CDC14C,VWC2*	.	.	.
7	49498097	C	T	intergenic	*CDC14C,VWC2*	.	.	.
17	45213923	A	T	intronic	*CDC27*	.	.	.
17	45225537	C	T	intronic	*CDC27*	.	.	.
17	45234625	C	T	exonic	*CDC27*	nonsynonymous_SNV	.	.
1	227186454	C	A	intronic	*CDC42BPA*	.	.	.
1	227383096	C	T	intronic	*CDC42BPA*	.	.	.
1	227679888	C	A	intergenic	*CDC42BPA,ZNF678*	.	.	.
1	227705637	C	A	intergenic	*CDC42BPA,ZNF678*	.	.	.
1	227592301	C	T	intergenic	*CDC42BPA,ZNF678*	.	.	.
1	227665978	T	C	intergenic	*CDC42BPA,ZNF678*	.	.	.
1	227740939	A	C	intergenic	*CDC42BPA,ZNF678*	.	.	.
1	227742652	A	G	intergenic	*CDC42BPA,ZNF678*	.	.	.

1	227742653	T	G	intergenic	*CDC42BPA,ZNF678*	.	.	.
2	37929275	T	A	intergenic	*CDC42EP3,LINC00211*	.	.	.
2	37953967	T	C	intergenic	*CDC42EP3,LINC00211*	.	.	.
2	38031768	T	C	intergenic	*CDC42EP3,LINC00211*	.	.	.
2	38032395	T	C	intergenic	*CDC42EP3,LINC00211*	.	.	.
17	71285902	GGA	G	intronic	*CDC42EP4*	.	.	.
17	71284978	C	T	intronic	*CDC42EP4*	.	.	.
5	130602948	G	A	intronic	*CDC42SE2*	.	.	.
5	130668346	C	T	intronic	*CDC42SE2*	.	.	.
5	130668347	A	G	intronic	*CDC42SE2*	.	.	.
5	130670697	T	G	intronic	*CDC42SE2*	.	.	.
5	130683427	A	G	intronic	*CDC42SE2*	.	.	.
6	44369474	C	T	intronic	*CDC5L*	.	.	.
6	44500232	C	G	intergenic	*CDC5L,SUPT3H*	.	.	.
6	44552638	G	T	intergenic	*CDC5L,SUPT3H*	.	.	.
6	44714373	C	T	intergenic	*CDC5L,SUPT3H*	.	.	.
6	44714375	T	A	intergenic	*CDC5L,SUPT3H*	.	.	.
6	44717299	G	C	intergenic	*CDC5L,SUPT3H*	.	.	.
6	44718525	T	C	intergenic	*CDC5L,SUPT3H*	.	.	.
6	44719433	T	C	intergenic	*CDC5L,SUPT3H*	.	.	.
6	44732334	A	T	intergenic	*CDC5L,SUPT3H*	.	.	.
6	44732432	G	A	intergenic	*CDC5L,SUPT3H*	.	.	.
6	44738368	G	A	intergenic	*CDC5L,SUPT3H*	.	.	.
1	92077604	C	T	intergenic	*CDC7,TGFBR3*	.	.	.
1	92089754	G	T	intergenic	*CDC7,TGFBR3*	.	.	.
1	193209368	A	G	intronic	*CDC73*	.	.	.
1	193132280	GTTGTGT	G	intronic	*CDC73*	.	.	.
1	193249690	AACACAC	A	intergenic	*CDC73,LINC01031*	.	.	.
8	25598960	A	T	intergenic	*CDCA2,EBF2*	.	.	.
8	25581277	T	C	intergenic	*CDCA2,EBF2*	.	.	.
14	105476892	T	A	UTR3	*CDCA4*	.	.	.
11	64851157	A	G	exonic	*CDCA5*	nonsynonymous_SNV	.	.

2	174609544	GTCTC	G	intergenic	*CDCA7,SP3*	.	.	.
2	174676678	T	A	intergenic	*CDCA7,SP3*	.	.	.
2	174373611	TACACACA CACACAC	T	intergenic	*CDCA7,SP3*	.	.	.
2	174429727	A	T	intergenic	*CDCA7,SP3*	.	.	.
2	174430403	A	T	intergenic	*CDCA7,SP3*	.	.	.
2	174564152	T	A	intergenic	*CDCA7,SP3*	.	.	.
2	174564153	T	C	intergenic	*CDCA7,SP3*	.	.	.
2	174595384	G	A	intergenic	*CDCA7,SP3*	.	.	.
7	22094341	G	T	intergenic	*CDCA7L,RAPGEF5*	.	.	.
7	22100811	AG	A	intergenic	*CDCA7L,RAPGEF5*	.	.	.
16	68840147	C	T	intronic	*CDH1*	.	.	.
16	68802106	C	T	intronic	*CDH1*	.	.	.
5	24532564	A	C	intronic	*CDH10*	.	.	.
5	24771466	A	AT	intergenic	*CDH10,LOC340107*	.	.	.
5	24702848	T	C	intergenic	*CDH10,LOC340107*	.	.	.
5	22545879	G	T	intronic	*CDH12*	.	.	.
5	21771499	G	A	intronic	*CDH12*	.	.	.
5	22523928	G	C	intronic	*CDH12*	.	.	.
5	22573808	AT	A	intronic	*CDH12*	.	.	.
5	22204420	T	C	intronic	*CDH12*	.	.	.
5	22320278	C	T	intronic	*CDH12*	.	.	.
5	21954020	G	GATA	intronic	*CDH12*	.	.	.
5	22754138	A	T	intronic	*CDH12*	.	.	.
5	22027538	A	G	intronic	*CDH12*	.	.	.
5	22027760	A	G	intronic	*CDH12*	.	.	.
5	22074526	A	T	intronic	*CDH12*	.	.	.
5	22279859	T	C	intronic	*CDH12*	.	.	.
5	22280064	A	G	intronic	*CDH12*	.	.	.
5	22359819	T	C	intronic	*CDH12*	.	.	.
5	22360460	C	G	intronic	*CDH12*	.	.	.
5	22362322	A	T	intronic	*CDH12*	.	.	.
5	22362838	T	C	intronic	*CDH12*	.	.	.
5	22478555	T	A	intronic	*CDH12*	.	.	.

5	22624265	A	AAATC	intronic	*CDH12*	.	.	.
5	22625097	G	T	intronic	*CDH12*	.	.	.
5	22625099	G	A	intronic	*CDH12*	.	.	.
5	22807092	T	C	intronic	*CDH12*	.	.	.
5	22822222	C	G	intronic	*CDH12*	.	.	.
5	23290454	A	G	intergenic	*CDH12,PRDM9*	.	.	.
5	23116470	A	C	intergenic	*CDH12,PRDM9*	.	.	.
5	23432923	T	A	intergenic	*CDH12,PRDM9*	.	.	.
5	22943001	C	G	intergenic	*CDH12,PRDM9*	.	.	.
5	22943002	T	G	intergenic	*CDH12,PRDM9*	.	.	.
5	22943299	T	C	intergenic	*CDH12,PRDM9*	.	.	.
5	23380834	G	GT	intergenic	*CDH12,PRDM9*	.	.	.
5	23380836	C	CA	intergenic	*CDH12,PRDM9*	.	.	.
16	83595144	G	A	intronic	*CDH13*	.	.	.
16	83760040	C	T	intronic	*CDH13*	.	.	.
16	83375594	CCACACAC ACA	C	intronic	*CDH13*	.	.	.
16	83669140	G	A	intronic	*CDH13*	.	.	.
5	20203255	A	G	intronic	*CDH18*	.	.	.
5	20449101	AATAT	A	intronic	*CDH18*	.	.	.
5	20473666	G	A	intronic	*CDH18*	.	.	.
5	19945824	G	A	intronic	*CDH18*	.	.	.
5	19709439	G	T	intronic	*CDH18*	.	.	.
5	19505549	G	T	intronic	*CDH18*	.	.	.
5	19551020	G	C	intronic	*CDH18*	.	.	.
5	19587073	T	A	intronic	*CDH18*	.	.	.
5	19587640	A	C	intronic	*CDH18*	.	.	.
5	20037797	C	G	intronic	*CDH18*	.	.	.
5	20039256	A	G	intronic	*CDH18*	.	.	.
5	20059792	G	A	intronic	*CDH18*	.	.	.
5	20135770	T	C	intronic	*CDH18*	.	.	.
5	20136078	G	A	intronic	*CDH18*	.	.	.
5	20138932	G	A	intronic	*CDH18*	.	.	.
5	20139207	A	G	intronic	*CDH18*	.	.	.
5	20139208	C	T	intronic	*CDH18*	.	.	.
5	20139584	G	T	intronic	*CDH18*	.	.	.
5	20192881	G	A	intronic	*CDH18*	.	.	.
5	20215169	G	T	intronic	*CDH18*	.	.	.

5	20215170	C	T	intronic	*CDH18*	.	.	.
5	20293362	T	G	intronic	*CDH18*	.	.	.
5	20390414	G	A	intronic	*CDH18*	.	.	.
5	20413280	G	A	intronic	*CDH18*	.	.	.
5	20858397	G	C	intergenic	*CDH18,GUSBP1*	.	.	.
5	20591285	G	T	intergenic	*CDH18,GUSBP1*	.	.	.
5	20997172	C	T	intergenic	*CDH18,GUSBP1*	.	.	.
5	20836993	C	T	intergenic	*CDH18,GUSBP1*	.	.	.
5	21380995	A	C	intergenic	*CDH18,GUSBP1*	.	.	.
5	21364059	A	G	intergenic	*CDH18,GUSBP1*	.	.	.
5	20990124	G	A	intergenic	*CDH18,GUSBP1*	.	.	.
5	21315903	G	T	intergenic	*CDH18,GUSBP1*	.	.	.
5	21373133	G	T	intergenic	*CDH18,GUSBP1*	.	.	.
5	20726932	A	G	intergenic	*CDH18,GUSBP1*	.	.	.
5	20877875	G	A	intergenic	*CDH18,GUSBP1*	.	.	.
5	20988543	T	A	intergenic	*CDH18,GUSBP1*	.	.	.
5	20988545	T	C	intergenic	*CDH18,GUSBP1*	.	.	.
5	20989293	A	G	intergenic	*CDH18,GUSBP1*	.	.	.
5	21026421	G	A	intergenic	*CDH18,GUSBP1*	.	.	.
5	21046489	T	C	intergenic	*CDH18,GUSBP1*	.	.	.
5	21230440	T	C	intergenic	*CDH18,GUSBP1*	.	.	.
5	21316236	T	A	intergenic	*CDH18,GUSBP1*	.	.	.
5	21357526	T	C	intergenic	*CDH18,GUSBP1*	.	.	.
5	21376446	C	G	intergenic	*CDH18,GUSBP1*	.	.	.
5	21408013	G	C	intergenic	*CDH18,GUSBP1*	.	.	.
5	21408464	T	C	intergenic	*CDH18,GUSBP1*	.	.	.
5	21435527	A	AT	intergenic	*CDH18,GUSBP1*	.	.	.
18	64255842	C	CATATATATA	intronic	*CDH19*	.	.	.
18	64280859	C	T	intergenic	*CDH19,MIR5011*	.	.	.
18	64372182	C	T	intergenic	*CDH19,MIR5011*	.	.	.
18	64664350	G	A	intergenic	*CDH19,MIR5011*	.	.	.
18	64664351	T	C	intergenic	*CDH19,MIR5011*	.	.	.

18	25736672	CATGTCACCTTCCCAGGTATAGCATGTCACCTTCCCAGGTATAGCATGTCACCTTCCCAGGTACAGA	C	intronic	*CDH2*	.	.	.
18	25753156	A	C	intronic	*CDH2*	.	.	.
18	26866701	G	A	intergenic	*CDH2,MIR302F*	.	.	.
18	27212197	T	C	intergenic	*CDH2,MIR302F*	.	.	.
18	26806069	G	T	intergenic	*CDH2,MIR302F*	.	.	.
18	26595116	ATGTG	A	intergenic	*CDH2,MIR302F*	.	.	.
18	27693339	T	TA	intergenic	*CDH2,MIR302F*	.	.	.
18	26000105	G	A	intergenic	*CDH2,MIR302F*	.	.	.
18	27241876	T	G	intergenic	*CDH2,MIR302F*	.	.	.
18	26035929	G	T	intergenic	*CDH2,MIR302F*	.	.	.
18	26451303	T	A	intergenic	*CDH2,MIR302F*	.	.	.
18	26522874	G	A	intergenic	*CDH2,MIR302F*	.	.	.
18	27473727	A	G	intergenic	*CDH2,MIR302F*	.	.	.
18	25802927	C	T	intergenic	*CDH2,MIR302F*	.	.	.
18	25803158	T	C	intergenic	*CDH2,MIR302F*	.	.	.
18	25804045	T	C	intergenic	*CDH2,MIR302F*	.	.	.
18	25804046	G	A	intergenic	*CDH2,MIR302F*	.	.	.
18	25875487	C	T	intergenic	*CDH2,MIR302F*	.	.	.
18	25876134	C	T	intergenic	*CDH2,MIR302F*	.	.	.
18	25884475	G	A	intergenic	*CDH2,MIR302F*	.	.	.
18	25922935	C	T	intergenic	*CDH2,MIR302F*	.	.	.
18	26013174	G	A	intergenic	*CDH2,MIR302F*	.	.	.
18	26013176	G	C	intergenic	*CDH2,MIR302F*	.	.	.
18	26157854	G	A	intergenic	*CDH2,MIR302F*	.	.	.
18	26240488	A	G	intergenic	*CDH2,MIR302F*	.	.	.
18	26621446	T	C	intergenic	*CDH2,MIR302F*	.	.	.
18	26631958	G	A	intergenic	*CDH2,MIR302F*	.	.	.
18	27055700	G	GATA	intergenic	*CDH2,MIR302F*	.	.	.
18	27147498	T	C	intergenic	*CDH2,MIR302F*	.	.	.
18	27147796	T	G	intergenic	*CDH2,MIR302F*	.	.	.

18	27171076	G	A	intergenic	*CDH2,MIR302F*	.	.	.
18	27181751	C	A	intergenic	*CDH2,MIR302F*	.	.	.
18	27281624	C	T	intergenic	*CDH2,MIR302F*	.	.	.
18	27480472	T	A	intergenic	*CDH2,MIR302F*	.	.	.
18	27529467	A	G	intergenic	*CDH2,MIR302F*	.	.	.
18	59186471	T	C	intronic	*CDH20*	.	.	.
18	59189445	A	T	intronic	*CDH20*	.	.	.
18	59189446	T	C	intronic	*CDH20*	.	.	.
18	59190778	A	T	intronic	*CDH20*	.	.	.
18	59228638	C	T	intergenic	*CDH20,LINC01544*	.	.	.
18	59229302	G	C	intergenic	*CDH20,LINC01544*	.	.	.
18	59235998	T	C	intergenic	*CDH20,LINC01544*	.	.	.
18	59235999	G	A	intergenic	*CDH20,LINC01544*	.	.	.
18	59368227	T	C	intergenic	*CDH20,LINC01544*	.	.	.
20	44907223	A	G	intronic	*CDH22*	.	.	.
20	44813769	TACACACACACACACAC	T	intronic	*CDH22*	.	.	.
10	73185611	G	A	intronic	*CDH23*	.	.	.
16	68688462	T	C	intronic	*CDH3*	.	.	.
16	68688463	G	T	intronic	*CDH3*	.	.	.
20	60502154	GTGTTT	G	intronic	*CDH4*	.	.	.
20	60007549	C	G	intronic	*CDH4*	.	.	.
20	60404435	G	C	intronic	*CDH4*	.	.	.
20	60411245	A	G	intronic	*CDH4*	.	.	.
20	59904405	C	CCCACGGG	intronic	*CDH4*	.	.	.
20	59849687	G	C	intronic	*CDH4*	.	.	.
20	59881300	C	T	intronic	*CDH4*	.	.	.
5	31254537	A	T	intronic	*CDH6*	.	.	.
5	31317897	C	T	exonic	*CDH6*	nonsynonymous_SNV	.	.
5	31360282	GGTGTGTGTGTGTGTGCGTGT	G	intergenic	*CDH6,DROSHA*	.	.	.
18	63854161	G	A	intergenic	*CDH7,CDH19*	.	.	.
18	63960073	A	T	intergenic	*CDH7,CDH19*	.	.	.
18	63961468	G	T	intergenic	*CDH7,CDH19*	.	.	.
18	63961507	T	C	intergenic	*CDH7,CDH19*	.	.	.

16	63371506	A	G	intergenic	*CDH8,CDH11*	.	.	.
16	63700922	G	C	intergenic	*CDH8,CDH11*	.	.	.
16	63186122	A	G	intergenic	*CDH8,CDH11*	.	.	.
16	63477165	T	C	intergenic	*CDH8,CDH11*	.	.	.
16	62503550	G	A	intergenic	*CDH8,NONE*	.	.	.
16	62073829	A	T	intergenic	*CDH8,NONE*	.	.	.
16	62124756	G	A	intergenic	*CDH8,NONE*	.	.	.
16	62825790	G	A	intergenic	*CDH8,NONE*	.	.	.
5	26974214	G	T	intronic	*CDH9*	.	.	.
5	26926371	C	T	intronic	*CDH9*	.	.	.
5	26998794	G	A	intronic	*CDH9*	.	.	.
5	27442264	GTATA	G	intergenic	*CDH9,LINC01021*	.	.	.
5	27133180	GTGTATATA	G	intergenic	*CDH9,LINC01021*	.	.	.
5	27202869	A	T	intergenic	*CDH9,LINC01021*	.	.	.
17	37757483	T	C	intergenic	*CDK12,NEUROD2*	.	.	.
7	40090749	A	G	intronic	*CDK13*	.	.	.
7	40022509	G	A	intronic	*CDK13*	.	.	.
7	90609505	G	A	intronic	*CDK14*	.	.	.
7	90492985	A	ATGTGTGTG	intronic	*CDK14*	.	.	.
7	90767008	GGTGTGT	G	intronic	*CDK14*	.	.	.
7	90600554	A	T	intronic	*CDK14*	.	.	.
7	90360599	T	C	intronic	*CDK14*	.	.	.
7	90437297	C	T	intronic	*CDK14*	.	.	.
7	90440204	T	C	intronic	*CDK14*	.	.	.
7	90705488	G	A	intronic	*CDK14*	.	.	.
7	90706626	T	C	intronic	*CDK14*	.	.	.
7	90844124	G	C	intergenic	*CDK14,FZD1*	.	.	.
7	90844126	G	C	intergenic	*CDK14,FZD1*	.	.	.
7	90885519	C	T	intergenic	*CDK14,FZD1*	.	.	.
1	205516074	G	T	intergenic	*CDK18,LOC284578*	.	.	.
6	111077959	T	C	intronic	*CDK19*	.	.	.
9	123258679	G	A	intronic	*CDK5RAP2*	.	.	.
9	123259148	G	A	intronic	*CDK5RAP2*	.	.	.
9	123314158	T	G	intronic	*CDK5RAP2*	.	.	.
9	123335663	A	C	intronic	*CDK5RAP2*	.	.	.
7	92405568	A	G	intronic	*CDK6*	.	.	.

7	92405569	G	C	intronic	*CDK6*	.	.	.
7	92405882	T	C	intronic	*CDK6*	.	.	.
6	21131977	G	A	intronic	*CDKAL1*	.	.	.
6	20894897	T	C	intronic	*CDKAL1*	.	.	.
6	20591329	C	T	intronic	*CDKAL1*	.	.	.
6	20618306	T	C	intronic	*CDKAL1*	.	.	.
6	20890139	C	T	intronic	*CDKAL1*	.	.	.
6	21055975	A	G	intronic	*CDKAL1*	.	.	.
6	21475585	C	T	intergenic	*CDKAL1,LINC00581*	.	.	.
6	21239917	C	G	intergenic	*CDKAL1,LINC00581*	.	.	.
6	21239918	A	G	intergenic	*CDKAL1,LINC00581*	.	.	.
14	50815141	T	C	intronic	*CDKL1*	.	.	.
14	50829906	G	A	intronic	*CDKL1*	.	.	.
14	50879574	A	G	intergenic	*CDKL1,MAP4K5*	.	.	.
5	133667858	A	G	intronic	*CDKL3*	.	.	.
5	133762893	C	T	intergenic	*CDKN2AIPNL,LOC102546229*	.	.	.
10	14879487	AG	A	intronic	*CDNF*	.	.	.
5	115159111	G	GTTTGT	intergenic	*CDO1,ATG12*	.	.	.
5	115159112	A	T	intergenic	*CDO1,ATG12*	.	.	.
5	115159243	A	T	intergenic	*CDO1,ATG12*	.	.	.
5	115159766	C	A	intergenic	*CDO1,ATG12*	.	.	.
5	115159767	A	C	intergenic	*CDO1,ATG12*	.	.	.
5	115160541	A	T	intergenic	*CDO1,ATG12*	.	.	.
5	115160542	T	C	intergenic	*CDO1,ATG12*	.	.	.
11	125855633	A	G	intronic	*CDON*	.	.	.
11	125855921	G	T	intronic	*CDON*	.	.	.
11	125978433	C	T	intergenic	*CDON,RPUSD4*	.	.	.
X	139902367	A	C	intergenic	*CDR1,MIR320D2*	.	.	.
17	15497603	C	T	intronic	*CDRT1*	.	.	.
17	14174009	G	T	intergenic	*CDRT15,HS3ST3B1*	.	.	.
17	14184618	G	A	intergenic	*CDRT15,HS3ST3B1*	.	.	.

17	14196231	G	A	intergenic	*CDRT15,HS3ST3B1*	.	.	.
17	20497797	C	A	intergenic	*CDRT15L2,LOC100287072*	.	.	.
17	20504300	C	T	intergenic	*CDRT15L2,LOC100287072*	.	.	.
17	15675414	TTGG	T	intergenic	*CDRT15P2,MEIS3P1*	.	.	.
17	15343135	C	T	intronic	*CDRT4,TVP23C-CDRT4*	.	.	.
17	15041977	G	A	intergenic	*CDRT8,PMP22*	.	.	.
17	15063231	C	T	intergenic	*CDRT8,PMP22*	.	.	.
17	15108962	C	T	intergenic	*CDRT8,PMP22*	.	.	.
20	5181062	T	C	intergenic	*CDS2,PROKR2*	.	.	.
20	5257473	G	A	intergenic	*CDS2,PROKR2*	.	.	.
20	5257866	A	C	intergenic	*CDS2,PROKR2*	.	.	.
X	72703340	G	A	intergenic	*CDX4,MAP2K4P1*	.	.	.
X	72712554	A	T	intergenic	*CDX4,MAP2K4P1*	.	.	.
X	72712903	C	T	intergenic	*CDX4,MAP2K4P1*	.	.	.
19	45054721	T	TAC	ncRNA_intronic	*CEACAM22P*	.	.	.
19	42142392	A	G	intergenic	*CEACAM4,CEACAM7*	.	.	.
19	42163843	T	C	intergenic	*CEACAM4,CEACAM7*	.	.	.
19	42165560	A	G	intergenic	*CEACAM4,CEACAM7*	.	.	.
19	42165561	G	A	intergenic	*CEACAM4,CEACAM7*	.	.	.
19	42166115	A	AG	intergenic	*CEACAM4,CEACAM7*	.	.	.
19	42210235	C	T	intergenic	*CEACAM7,CEACAM5*	.	.	.
19	33818620	A	T	intergenic	*CEBPA-AS1,CEBPG*	.	.	.

8	48662183	CTGCAGTGAGCCGTGATCGTGCCACTGCACTCCAGCCTGGGTGACAGAGTGAGCCCCCCACTTTTTTTTTTTTTTGAGAGGGAGTCTCACTCTGTCAGCCAGGCTGGAG	C	intergenic	*CEBPD,PRKDC*	.	.	.
8	48668762	C	T	intergenic	*CEBPD,PRKDC*	.	.	.
8	48668763	T	G	intergenic	*CEBPD,PRKDC*	.	.	.
10	11369073	G	T	ncRNA_intronic	*CELF2-AS1*	.	.	.
18	34921537	C	T	intronic	*CELF4*	.	.	.
18	35181678	T	C	intergenic	*CELF4,MIR4318*	.	.	.
19	3278192	C	T	intronic	*CELF5*	.	.	.
19	3280090	G	T	intronic	*CELF5*	.	.	.
9	135962458	ATGCCCCTGTGCCCCTCACAAGTGACTCTGAGGC	A	ncRNA_exonic	*CELP*	.	.	.
22	46931873	C	T	exonic	*CELSR1*	nonsynonymous_SNV	.	.
15	81255590	T	G	intergenic	*CEMIP,MESDC2*	.	.	.
15	81255592	T	C	intergenic	*CEMIP,MESDC2*	.	.	.
19	59102181	G	A	intergenic	*CENPBD1P1,NONE*	.	.	.
4	68398312	A	G	intronic	*CENPC*	.	.	.
4	104111077	G	A	intronic	*CENPE*	.	.	.
4	104111825	G	T	intronic	*CENPE*	.	.	.
4	104160001	G	A	intergenic	*CENPE,LOC101929448*	.	.	.

4	104161495	G	A	intergenic	*CENPE,LOC101929448*	.	.	.
4	104163268	A	T	intergenic	*CENPE,LOC101929448*	.	.	.
4	104163765	G	A	intergenic	*CENPE,LOC101929448*	.	.	.
4	104172756	A	G	intergenic	*CENPE,LOC101929448*	.	.	.
4	104174340	A	G	intergenic	*CENPE,LOC101929448*	.	.	.
4	104201757	G	A	intergenic	*CENPE,LOC101929448*	.	.	.
4	104217177	G	A	intergenic	*CENPE,LOC101929448*	.	.	.
4	104317235	G	A	intergenic	*CENPE,LOC101929448*	.	.	.
1	214784141	G	A	intronic	*CENPF*	.	.	.
1	214784142	T	G	intronic	*CENPF*	.	.	.
1	215139725	T	C	intergenic	*CENPF,KCNK2*	.	.	.
1	214896618	T	C	intergenic	*CENPF,KCNK2*	.	.	.
1	214897277	T	C	intergenic	*CENPF,KCNK2*	.	.	.
1	214898139	A	G	intergenic	*CENPF,KCNK2*	.	.	.
1	214994810	G	C	intergenic	*CENPF,KCNK2*	.	.	.
1	214994811	T	A	intergenic	*CENPF,KCNK2*	.	.	.
1	215104630	C	G	intergenic	*CENPF,KCNK2*	.	.	.
X	100384509	G	A	intronic	*CENPI*	.	.	.
X	100439360	T	A	intergenic	*CENPI,DRP2*	.	.	.
13	25498194	A	G	intergenic	*CENPJ,TPTE2P1*	.	.	.
1	173783790	T	C	intronic	*CENPL*	.	.	.
1	173783791	G	A	intronic	*CENPL*	.	.	.
1	173786405	A	C	intronic	*CENPL*	.	.	.
2	25024210	G	A	intronic	*CENPO*	.	.	.
9	95335077	T	C	intronic	*CENPP*	.	.	.
6	49442336	T	A	intronic	*CENPQ*	.	.	.
6	49444763	T	C	intronic	*CENPQ*	.	.	.
6	49444765	T	C	intronic	*CENPQ*	.	.	.
X	51441423	CCTCT	C	intergenic	*CENPVP2,CENPVP2*	.	.	.

6	126729242	TTTAGTAAATGGTGCTGGAACAACTGGGTAGCCATATGCATAAAGTTGAAA	T	intergenic	*CENPW,MIR588*	.	.	.
6	126693283	A	G	intergenic	*CENPW,NONE*	.	.	.
1	3758805	G	A	intronic	*CEP104*	.	.	.
17	64070340	C	A	intronic	*CEP112*	.	.	.
17	63688507	G	A	intronic	*CEP112*	.	.	.
17	64055699	T	A	intronic	*CEP112*	.	.	.
17	64076767	T	A	intronic	*CEP112*	.	.	.
17	64154931	A	C	intronic	*CEP112*	.	.	.
17	64154933	A	T	intronic	*CEP112*	.	.	.
17	64155167	T	C	intronic	*CEP112*	.	.	.
17	64156198	T	C	intronic	*CEP112*	.	.	.
17	64157283	C	T	intronic	*CEP112*	.	.	.
5	122783820	A	G	intergenic	*CEP120,CSNK1G3*	.	.	.
5	122811964	A	G	intergenic	*CEP120,CSNK1G3*	.	.	.
5	122793956	C	G	intergenic	*CEP120,CSNK1G3*	.	.	.
11	101903848	A	T	intergenic	*CEP126,C11orf70*	.	.	.
11	101903850	T	C	intergenic	*CEP126,C11orf70*	.	.	.
14	81087081	C	A	intronic	*CEP128*	.	.	.
14	81057262	C	G	intronic	*CEP128*	.	.	.
14	81058654	G	A	intronic	*CEP128*	.	.	.
14	81058656	G	A	intronic	*CEP128*	.	.	.
14	81059379	A	C	intronic	*CEP128*	.	.	.
14	81116654	G	T	intronic	*CEP128*	.	.	.
17	79198633	G	A	intergenic	*CEP131,ENTHD2*	.	.	.
6	84839973	A	T	intronic	*CEP162*	.	.	.
6	84890966	A	G	intronic	*CEP162*	.	.	.
6	84914599	G	A	intronic	*CEP162*	.	.	.
6	84914639	C	T	intronic	*CEP162*	.	.	.
6	85075983	G	T	intergenic	*CEP162,LINC01611*	.	.	.
11	117270714	T	C	intronic	*CEP164*	.	.	.
11	117272692	A	G	intronic	*CEP164*	.	.	.

1	243314998	G	A	intronic	*CEP170*	.	.	.
1	243302593	G	A	intronic	*CEP170*	.	.	.
18	13027052	C	G	intronic	*CEP192*	.	.	.
18	13169109	A	AGAGAG	intergenic	*CEP192,LDLRAD4*	.	.	.
20	34085770	C	T	exonic	*CEP250*	stopgain	.	.
12	88496418	TTGTG	T	intronic	*CEP290*	.	.	.
1	179967725	A	G	intronic	*CEP350*	.	.	.
1	179967726	T	C	intronic	*CEP350*	.	.	.
1	180118677	C	T	intergenic	*CEP350,QSOX1*	.	.	.
4	175279202	CAAACAAA ACA	C	intergenic	*CEP44,MIR4276*	.	.	.
4	175266833	A	C	intergenic	*CEP44,MIR4276*	.	.	.
4	175335847	G	A	intergenic	*CEP44,MIR4276*	.	.	.
10	95281790	T	C	intronic	*CEP55*	.	.	.
10	95281791	G	A	intronic	*CEP55*	.	.	.
10	95285908	T	A	intronic	*CEP55*	.	.	.
10	95319260	C	A	intergenic	*CEP55,FFAR4*	.	.	.
10	95298657	A	T	intergenic	*CEP55,FFAR4*	.	.	.
10	95298799	T	C	intergenic	*CEP55,FFAR4*	.	.	.
10	95299455	A	C	intergenic	*CEP55,FFAR4*	.	.	.
11	95541958	G	A	intronic	*CEP57*	.	.	.
11	95542720	C	A	intronic	*CEP57*	.	.	.
6	109430988	T	C	intronic	*CEP57L1*	.	.	.
6	109432931	G	A	intronic	*CEP57L1*	.	.	.
6	109433860	C	A	intronic	*CEP57L1*	.	.	.
6	109454963	G	A	intronic	*CEP57L1*	.	.	.
6	109470480	G	A	intronic	*CEP57L1*	.	.	.
3	134299269	A	AAAAAAAAT	intergenic	*CEP63,KY*	.	.	.
2	65285622	A	T	intronic	*CEP68*	.	.	.
5	649037	T	G	intronic	*CEP72*	.	.	.
5	649024	G	T	intronic	*CEP72*	.	.	.
12	94831888	G	A	intronic	*CEP83*	.	.	.
12	94878444	T	C	intergenic	*CEP83-AS1,MIR5700*	.	.	.
12	94879303	T	C	intergenic	*CEP83-AS1,MIR5700*	.	.	.
6	118829220	T	C	intronic	*CEP85L*	.	.	.
6	118934819	C	A	intronic	*CEP85L*	.	.	.

6	118934820	T	G	intronic	*CEP85L*	.	.	.
6	118837772	A	G	intronic	*CEP85L*	.	.	.
6	118838190	C	T	intronic	*CEP85L*	.	.	.
19	33404208	TATA	T	intronic	*CEP89*	.	.	.
3	101465160	A	G	intronic	*CEP97*	.	.	.
2	182421747	C	T	intronic	*CERKL*	.	.	.
2	182528191	C	G	intergenic	*CERKL,NEUROD1*	.	.	.
19	8298240	G	A	intronic	*CERS4*	.	.	.
2	169332597	C	T	intronic	*CERS6*	.	.	.
16	55878720	C	T	intergenic	*CES1,CES5A*	.	.	.
16	67039552	C	G	intronic	*CES4A*	.	.	.
16	56055589	GTGTGTGTGTGTGTGTGTGTGTGTGTA	G	intergenic	*CES5A,LOC283856*	.	.	.
1	161450813	TTCTC	T	intergenic	*CFAP126,FCGR2A*	.	.	.
2	120380458	A	G	intronic	*CFAP221*	.	.	.
10	134696554	C	T	intronic	*CFAP46*	.	.	.
10	134634957	CTGTGTGTGCTGATGTGTGT	C	intronic	*CFAP46*	.	.	.
10	134746602	G	A	intronic	*CFAP46*	.	.	.
X	36313552	C	A	intronic	*CFAP47*	.	.	.
X	36035672	A	AT	intronic	*CFAP47*	.	.	.
X	36060769	C	T	intronic	*CFAP47*	.	.	.
X	36300647	T	C	intronic	*CFAP47*	.	.	.
X	36136687	C	T	intronic	*CFAP47*	.	.	.
X	36106629	TTCTC	T	intronic	*CFAP47*	.	.	.
X	36215008	C	G	intronic	*CFAP47*	.	.	.
X	35958509	A	T	intronic	*CFAP47*	.	.	.
X	36030497	A	G	intronic	*CFAP47*	.	.	.
X	36096932	C	T	intronic	*CFAP47*	.	.	.
X	36098038	A	T	intronic	*CFAP47*	.	.	.
X	36145965	A	T	intronic	*CFAP47*	.	.	.
X	36145967	T	A	intronic	*CFAP47*	.	.	.
X	36191843	C	G	intronic	*CFAP47*	.	.	.
X	36222155	G	A	intronic	*CFAP47*	.	.	.
X	36248895	A	G	intronic	*CFAP47*	.	.	.

X	36251035	A	T	intronic	*CFAP47*	.	.	.
X	36365058	G	C	intronic	*CFAP47*	.	.	.
X	36365098	T	C	intronic	*CFAP47*	.	.	.
X	36365415	T	A	intronic	*CFAP47*	.	.	.
17	9504407	G	T	intronic	*CFAP52*	.	.	.
12	97252971	G	A	intronic	*CFAP54*	.	.	.
12	97208244	A	T	intronic	*CFAP54*	.	.	.
12	96993532	A	G	intronic	*CFAP54*	.	.	.
12	96996064	C	T	intronic	*CFAP54*	.	.	.
12	97039152	C	T	intronic	*CFAP54*	.	.	.
12	97118594	G	A	intronic	*CFAP54*	.	.	.
12	97139456	G	A	intronic	*CFAP54*	.	.	.
12	97209050	C	T	intronic	*CFAP54*	.	.	.
10	106228125	GTATA	G	intergenic	*CFAP58,LOC101927523*	.	.	.
20	20095957	C	CCAGCACTT	intronic	*CFAP61*	.	.	.
20	20222429	C	CATT	intronic	*CFAP61*	.	.	.
7	89921332	G	C	intronic	*CFAP69*	.	.	.
7	89944708	C	A	intergenic	*CFAP69,LOC101927446*	.	.	.
7	89953537	T	C	intergenic	*CFAP69,LOC101927446*	.	.	.
7	89959889	C	T	intergenic	*CFAP69,LOC101927446*	.	.	.
4	2432335	G	T	intronic	*CFAP99*	.	.	.
4	2431162	C	T	intronic	*CFAP99*	.	.	.
1	196667214	C	A	intronic	*CFH*	.	.	.
1	196831567	G	C	intergenic	*CFHR1,CFHR4*	.	.	.
1	196832397	A	C	intergenic	*CFHR1,CFHR4*	.	.	.
1	196832399	T	C	intergenic	*CFHR1,CFHR4*	.	.	.
1	196850060	G	C	intergenic	*CFHR1,CFHR4*	.	.	.
1	196850187	A	G	intergenic	*CFHR1,CFHR4*	.	.	.
1	196850475	T	TA	intergenic	*CFHR1,CFHR4*	.	.	.
1	196850476	C	CA	intergenic	*CFHR1,CFHR4*	.	.	.
1	196851562	G	A	intergenic	*CFHR1,CFHR4*	.	.	.
1	196915508	T	C	intronic	*CFHR2*	.	.	.
1	196907319	T	C	intergenic	*CFHR4,CFHR2*	.	.	.
1	196907320	G	A	intergenic	*CFHR4,CFHR2*	.	.	.

1	196956245	A	G	intronic	*CFHR5*	.	.	.
1	196956502	T	C	intronic	*CFHR5*	.	.	.
1	196956714	C	T	intronic	*CFHR5*	.	.	.
1	196958700	G	T	intronic	*CFHR5*	.	.	.
4	110699234	G	A	intronic	*CFI*	.	.	.
7	117157936	C	G	intronic	*CFTR*	.	.	.
7	117193278	GACACACACAC	G	intronic	*CFTR*	.	.	.
6	87851078	G	A	intergenic	*CGA,ZNF292*	.	.	.
3	88147274	T	C	intronic	*CGGBP1*	.	.	.
1	151509763	G	A	exonic	*CGN*	nonsynonymous_SNV	.	.
14	55011008	C	T	intergenic	*CGRRF1,SAMD4A*	.	.	.
19	4437990	G	A	intronic	*CHAF1A*	.	.	.
7	56179360	G	A	intergenic	*CHCHD2,NUPR2*	.	.	.
7	132589289	T	A	intronic	*CHCHD3*	.	.	.
7	132829204	C	T	intergenic	*CHCHD3,EXOC4*	.	.	.
7	132829205	A	G	intergenic	*CHCHD3,EXOC4*	.	.	.
7	132895347	T	A	intergenic	*CHCHD3,EXOC4*	.	.	.
2	113366302	T	G	intergenic	*CHCHD5,FLJ42351*	.	.	.
2	113368790	C	A	intergenic	*CHCHD5,FLJ42351*	.	.	.
3	126511087	ATAGAATATATATTCTAATATAGAATATATTAGAATATATATTC	A	intronic	*CHCHD6*	.	.	.
3	126481294	C	G	intronic	*CHCHD6*	.	.	.
8	57174706	C	T	intergenic	*CHCHD7,SDR16C5*	.	.	.
1	146781316	G	A	intergenic	*CHD1L,LINC00624*	.	.	.
1	146783595	T	C	intergenic	*CHD1L,LINC00624*	.	.	.
1	146800774	A	G	intergenic	*CHD1L,LINC00624*	.	.	.
1	146816865	G	A	intergenic	*CHD1L,LINC00624*	.	.	.
1	146817143	C	T	intergenic	*CHD1L,LINC00624*	.	.	.
1	146817202	T	C	intergenic	*CHD1L,LINC00624*	.	.	.
1	146833492	G	A	intergenic	*CHD1L,LINC00624*	.	.	.

12	6680087	C	T	exonic	*CHD4*	nonsynonymous_SNV	.	.
20	40219007	A	T	intronic	*CHD6*	.	.	.
20	40219008	G	C	intronic	*CHD6*	.	.	.
20	40597275	A	G	intergenic	*CHD6,PTPRT*	.	.	.
20	40507417	C	G	intergenic	*CHD6,PTPRT*	.	.	.
20	40310855	T	G	intergenic	*CHD6,PTPRT*	.	.	.
20	40331608	T	A	intergenic	*CHD6,PTPRT*	.	.	.
20	40331610	A	ATCG	intergenic	*CHD6,PTPRT*	.	.	.
20	40370416	G	C	intergenic	*CHD6,PTPRT*	.	.	.
20	40388695	C	T	intergenic	*CHD6,PTPRT*	.	.	.
20	40388696	A	G	intergenic	*CHD6,PTPRT*	.	.	.
20	40431787	A	C	intergenic	*CHD6,PTPRT*	.	.	.
20	40595857	A	G	intergenic	*CHD6,PTPRT*	.	.	.
8	61735231	G	A	exonic	*CHD7*	nonsynonymous_SNV	.	.
8	61738504	T	C	intronic	*CHD7*	.	.	.
16	53378667	C	A	intergenic	*CHD9,LOC643802*	.	.	.
16	53378668	C	T	intergenic	*CHD9,LOC643802*	.	.	.
16	53381737	T	C	intergenic	*CHD9,LOC643802*	.	.	.
15	20557490	C	A	intergenic	*CHEK2P2,HERC2P3*	.	.	.
15	20581484	A	G	intergenic	*CHEK2P2,HERC2P3*	.	.	.
15	20514339	A	AATGAGATG	intergenic	*CHEK2P2,HERC2P3*	.	.	.
15	20546544	G	GCCAAGCCA	intergenic	*CHEK2P2,HERC2P3*	.	.	.
15	20516056	ATG	A	intergenic	*CHEK2P2,HERC2P3*	.	.	.
15	20498509	T	C	intergenic	*CHEK2P2,HERC2P3*	.	.	.
15	20521330	G	A	intergenic	*CHEK2P2,HERC2P3*	.	.	.
15	20523841	T	C	intergenic	*CHEK2P2,HERC2P3*	.	.	.
15	20534548	C	CATAT	intergenic	*CHEK2P2,HERC2P3*	.	.	.

15	20555919	CGTGTGTGTGTGTGTGTGTGT	C	intergenic	*CHEK2P2,HERC2P3*	.	.	.
15	20578738	A	AG	intergenic	*CHEK2P2,HERC2P3*	.	.	.
1	111880280	C	T	intergenic	*CHIA,PIFO*	.	.	.
X	72840270	C	T	intronic	*CHIC1*	.	.	.
X	72840810	A	G	intronic	*CHIC1*	.	.	.
X	72893739	C	T	intronic	*CHIC1*	.	.	.
X	72893741	G	A	intronic	*CHIC1*	.	.	.
X	72893928	A	T	intronic	*CHIC1*	.	.	.
X	72894325	C	A	intronic	*CHIC1*	.	.	.
X	72917395	A	G	intergenic	*CHIC1,TSIX*	.	.	.
X	72925759	T	C	intergenic	*CHIC1,TSIX*	.	.	.
X	72913702	C	G	intergenic	*CHIC1,TSIX*	.	.	.
X	72917584	G	C	intergenic	*CHIC1,TSIX*	.	.	.
X	72920055	A	C	intergenic	*CHIC1,TSIX*	.	.	.
X	72920733	G	A	intergenic	*CHIC1,TSIX*	.	.	.
X	72926667	G	A	intergenic	*CHIC1,TSIX*	.	.	.
X	72965099	G	A	intergenic	*CHIC1,TSIX*	.	.	.
X	72993135	A	T	intergenic	*CHIC1,TSIX*	.	.	.
1	203230242	A	G	intergenic	*CHIT1,LINC01353*	.	.	.
11	67836647	C	A	intronic	*CHKA*	.	.	.
3	355615	A	G	intronic	*CHL1*	.	.	.
3	372616	G	A	intronic	*CHL1*	.	.	.
3	410226	G	A	ncRNA_intronic	*CHL1-AS1*	.	.	.
X	85235270	A	T	intronic	*CHM*	.	.	.
X	85171218	C	T	intronic	*CHM*	.	.	.
X	85171465	C	T	intronic	*CHM*	.	.	.
X	85260151	T	C	intronic	*CHM*	.	.	.
X	85292107	A	T	intronic	*CHM*	.	.	.
X	85400179	C	A	intergenic	*CHM,DACH2*	.	.	.
X	79578290	C	T	ncRNA_intronic	*CHMP1B2P*	.	.	.
X	79579333	A	T	ncRNA_intronic	*CHMP1B2P*	.	.	.
8	82688440	C	T	intergenic	*CHMP4C,SNX16*	.	.	.
9	33271069	C	CAAAT	intronic	*CHMP5*	.	.	.
2	175685077	C	A	intronic	*CHN1*	.	.	.
2	175847183	G	A	intronic	*CHN1*	.	.	.

7	29208857	T	C	intronic	*CHN2*	.	.	.
7	29237590	C	A	UTR5	*CHN2*	.	.	.
7	29208830	A	G	intronic	*CHN2*	.	.	.
21	19321727	GA	G	intronic	*CHODL*	.	.	.
21	19319861	GACAC	G	intronic	*CHODL*	.	.	.
21	19345274	A	G	intronic	*CHODL*	.	.	.
21	19345535	A	G	intronic	*CHODL*	.	.	.
21	19345632	C	T	intronic	*CHODL*	.	.	.
12	102105288	T	C	intronic	*CHPT1*	.	.	.
X	110012059	CA	C	intronic	*CHRDL1*	.	.	.
X	110061243	A	T	intergenic	*CHRDL1,PAK3*	.	.	.
X	110075563	T	A	intergenic	*CHRDL1,PAK3*	.	.	.
X	110143525	T	C	intergenic	*CHRDL1,PAK3*	.	.	.
X	110144425	A	C	intergenic	*CHRDL1,PAK3*	.	.	.
11	62710974	G	A	intergenic	*CHRM1,SLC22A6*	.	.	.
7	136558246	A	G	intronic	*CHRM2*	.	.	.
1	239888580	T	C	intronic	*CHRM3*	.	.	.
1	239802717	C	G	intronic	*CHRM3*	.	.	.
1	240168129	G	A	intergenic	*CHRM3,RPS7P5*	.	.	.
2	175618552	A	AAGCTCCCA	intronic	*CHRNA1*	.	.	.
2	175622935	G	A	intronic	*CHRNA1*	.	.	.
20	61987722	C	T	splicing	*CHRNA4*	.	.	.
8	42626622	T	C	intergenic	*CHRNA6,THAP1*	.	.	.
8	42682899	T	G	intergenic	*CHRNA6,THAP1*	.	.	.
15	32372111	G	A	intronic	*CHRNA7*	.	.	.
15	32372112	T	G	intronic	*CHRNA7*	.	.	.
15	32435451	T	C	intronic	*CHRNA7*	.	.	.
4	40368698	A	T	intergenic	*CHRNA9,RBM47*	.	.	.
4	40365092	C	T	intergenic	*CHRNA9,RBM47*	.	.	.
12	104859881	G	A	intronic	*CHST11*	.	.	.
12	104966816	G	A	intronic	*CHST11*	.	.	.
10	125926147	A	G	intergenic	*CHST15,OAT*	.	.	.
3	142949976	T	A	intergenic	*CHST2,SLC9A9*	.	.	.
10	73798884	G	A	intergenic	*CHST3,SPOCK2*	.	.	.
X	46447148	A	C	intronic	*CHST7*	.	.	.
X	46451466	A	T	intronic	*CHST7*	.	.	.
X	46451467	G	A	intronic	*CHST7*	.	.	.
19	34141251	C	T	intronic	*CHST8*	.	.	.

19	34180960	G	A	intronic	*CHST8*	.	.	.
18	24604164	C	A	intronic	*CHST9*	.	.	.
18	24706562	G	A	intronic	*CHST9*	.	.	.
18	24670599	A	G	intronic	*CHST9*	.	.	.
18	24527348	A	C	intronic	*CHST9*	.	.	.
18	24670538	A	T	intronic	*CHST9*	.	.	.
18	24678536	G	A	intronic	*CHST9*	.	.	.
18	24742946	G	A	intronic	*CHST9*	.	.	.
18	25487404	T	A	intergenic	*CHST9,CDH2*	.	.	.
18	25246686	A	T	intergenic	*CHST9,CDH2*	.	.	.
18	25275369	G	A	intergenic	*CHST9,CDH2*	.	.	.
18	25274546	C	T	intergenic	*CHST9,CDH2*	.	.	.
18	25274548	G	A	intergenic	*CHST9,CDH2*	.	.	.
18	25432836	G	A	intergenic	*CHST9,CDH2*	.	.	.
5	129393593	CAGAG	C	intronic	*CHSY3*	.	.	.
5	129386712	G	T	intronic	*CHSY3*	.	.	.
5	129333605	A	T	intronic	*CHSY3*	.	.	.
5	130245643	A	C	intergenic	*CHSY3,HINT1*	.	.	.
5	130052097	T	C	intergenic	*CHSY3,HINT1*	.	.	.
5	129562674	C	A	intergenic	*CHSY3,HINT1*	.	.	.
5	129583763	T	C	intergenic	*CHSY3,HINT1*	.	.	.
5	129755342	A	C	intergenic	*CHSY3,HINT1*	.	.	.
5	129755343	T	C	intergenic	*CHSY3,HINT1*	.	.	.
5	129757090	T	C	intergenic	*CHSY3,HINT1*	.	.	.
5	129782259	A	C	intergenic	*CHSY3,HINT1*	.	.	.
5	129782301	A	C	intergenic	*CHSY3,HINT1*	.	.	.
5	129848283	C	T	intergenic	*CHSY3,HINT1*	.	.	.
5	129861059	G	A	intergenic	*CHSY3,HINT1*	.	.	.
5	129913618	G	A	intergenic	*CHSY3,HINT1*	.	.	.
5	129929250	T	C	intergenic	*CHSY3,HINT1*	.	.	.
5	129945816	A	C	intergenic	*CHSY3,HINT1*	.	.	.
5	129947392	A	C	intergenic	*CHSY3,HINT1*	.	.	.
5	129948502	G	A	intergenic	*CHSY3,HINT1*	.	.	.
5	129999813	A	AC	intergenic	*CHSY3,HINT1*	.	.	.
5	130011029	T	C	intergenic	*CHSY3,HINT1*	.	.	.
5	130156204	C	T	intergenic	*CHSY3,HINT1*	.	.	.
5	130266860	A	T	intergenic	*CHSY3,HINT1*	.	.	.
5	130268203	C	T	intergenic	*CHSY3,HINT1*	.	.	.

5	130268520	T	G	intergenic	*CHSY3,HINT1*	.	.	.
5	130269596	T	C	intergenic	*CHSY3,HINT1*	.	.	.
5	130270609	G	A	intergenic	*CHSY3,HINT1*	.	.	.
5	130292438	T	C	intergenic	*CHSY3,HINT1*	.	.	.
5	130352755	C	T	intergenic	*CHSY3,HINT1*	.	.	.
5	130365326	A	G	intergenic	*CHSY3,HINT1*	.	.	.
5	130366991	C	T	intergenic	*CHSY3,HINT1*	.	.	.
5	130387547	T	A	intergenic	*CHSY3,HINT1*	.	.	.
5	130427186	G	T	intergenic	*CHSY3,HINT1*	.	.	.
5	130453503	T	C	intergenic	*CHSY3,HINT1*	.	.	.
5	130454228	A	G	intergenic	*CHSY3,HINT1*	.	.	.
5	130464148	C	T	intergenic	*CHSY3,HINT1*	.	.	.
5	130464331	T	C	intergenic	*CHSY3,HINT1*	.	.	.
5	130471134	C	A	intergenic	*CHSY3,HINT1*	.	.	.
5	130473250	A	G	intergenic	*CHSY3,HINT1*	.	.	.
14	65403162	A	C	intronic	*CHURC1-FNTB*	.	.	.
14	65527008	CTTTTTAAAAAGTTTG	C	intronic	*CHURC1-FNTB,FNTB,MAX*	.	.	.
1	150257744	G	A	intronic	*CIART*	.	.	.
16	10996102	G	T	intronic	*CIITA*	.	.	.
12	54272048	T	A	intergenic	*CISTR,HOXC13-AS*	.	.	.
12	54273126	G	C	intergenic	*CISTR,HOXC13-AS*	.	.	.
12	120141213	A	C	intronic	*CIT*	.	.	.
12	120317520	C	CAA	intergenic	*CIT,CCDC64*	.	.	.
6	139776453	T	C	intergenic	*CITED2,LINC01625*	.	.	.
12	106654858	AAAAAAAAAT	A	intergenic	*CKAP4,TCP11L2*	.	.	.
19	45820853	T	C	intronic	*CKM*	.	.	.
5	80574633	C	T	ncRNA_intronic	*CKMT2-AS1,RNU5D-1,RNU5E-1*	.	.	.
5	80574635	G	A	ncRNA_intronic	*CKMT2-AS1,RNU5D-1,RNU5E-1*	.	.	.
2	122272392	G	A	intronic	*CLASP1*	.	.	.

2	122309097	A	G	intronic	*CLASP1*	.	.	.
3	33749984	ATATATATA TATATG	A	intronic	*CLASP2*	.	.	.
3	33605121	A	G	intronic	*CLASP2*	.	.	.
3	33622559	T	A	intronic	*CLASP2*	.	.	.
19	40240495	T	C	intergenic	*CLC,LEUTX*	.	.	.
1	87015813	C	A	intronic	*CLCA4*	.	.	.
1	87077010	C	T	intergenic	*CLCA4,CLCA3P*	.	.	.
1	87077011	G	A	intergenic	*CLCA4,CLCA3P*	.	.	.
1	87093452	C	A	intergenic	*CLCA4,CLCA3P*	.	.	.
1	87096623	T	G	intergenic	*CLCA4,CLCA3P*	.	.	.
1	87097139	G	A	intergenic	*CLCA4,CLCA3P*	.	.	.
4	170591138	C	A	intronic	*CLCN3*	.	.	.
4	170591348	A	C	intronic	*CLCN3*	.	.	.
4	170591349	G	A	intronic	*CLCN3*	.	.	.
X	10337107	A	G	intergenic	*CLCN4,MID1*	.	.	.
X	10405611	G	T	intergenic	*CLCN4,MID1*	.	.	.
X	10306992	T	C	intergenic	*CLCN4,MID1*	.	.	.
X	10386383	T	C	intergenic	*CLCN4,MID1*	.	.	.
X	49862721	C	G	UTR3	*CLCN5*	.	.	.
X	49845757	G	T	intronic	*CLCN5*	.	.	.
X	49772170	C	T	intronic	*CLCN5*	.	.	.
X	49810179	T	A	intronic	*CLCN5*	.	.	.
X	49933509	C	A	intergenic	*CLCN5,AKAP4*	.	.	.
16	1530270	G	A	intergenic	*CLCN7,PTX4*	.	.	.
7	90184906	T	C	intergenic	*CLDN12,CDK14*	.	.	.
7	90155125	C	T	intergenic	*CLDN12,CDK14*	.	.	.
7	90175072	T	C	intergenic	*CLDN12,CDK14*	.	.	.
7	90175507	T	C	intergenic	*CLDN12,CDK14*	.	.	.
7	90175508	G	T	intergenic	*CLDN12,CDK14*	.	.	.
7	90192007	T	G	intergenic	*CLDN12,CDK14*	.	.	.
21	38050597	C	T	intergenic	*CLDN14,SIM2*	.	.	.
21	37956517	G	A	intergenic	*CLDN14,SIM2*	.	.	.
21	37956518	A	G	intergenic	*CLDN14,SIM2*	.	.	.
21	37991217	T	C	intergenic	*CLDN14,SIM2*	.	.	.
21	37991218	T	C	intergenic	*CLDN14,SIM2*	.	.	.
21	31539810	T	A	upstream	*CLDN17*	.	.	.

4	184295257	T	G	intergenic	*CLDN24,CDKN2AIP*	.	.	.
X	9963000	G	T	intergenic	*CLDN34,WWC3*	.	.	.
X	9968798	C	T	intergenic	*CLDN34,WWC3*	.	.	.
17	6985754	T	C	intergenic	*CLEC10A,ASGR2*	.	.	.
12	10140941	T	A	intergenic	*CLEC12A,CLEC1B*	.	.	.
14	38968803	C	A	intergenic	*CLEC14A,LINC00639*	.	.	.
14	38747250	A	G	intergenic	*CLEC14A,LINC00639*	.	.	.
14	38894288	C	T	intergenic	*CLEC14A,LINC00639*	.	.	.
14	38974251	C	T	intergenic	*CLEC14A,LINC00639*	.	.	.
14	38765316	G	T	intergenic	*CLEC14A,LINC00639*	.	.	.
14	39022601	G	A	intergenic	*CLEC14A,LINC00639*	.	.	.
14	38734913	T	C	intergenic	*CLEC14A,LINC00639*	.	.	.
14	38735417	G	C	intergenic	*CLEC14A,LINC00639*	.	.	.
14	38736400	T	C	intergenic	*CLEC14A,LINC00639*	.	.	.
14	38794895	G	A	intergenic	*CLEC14A,LINC00639*	.	.	.
14	38794896	T	A	intergenic	*CLEC14A,LINC00639*	.	.	.
14	38851098	C	T	intergenic	*CLEC14A,LINC00639*	.	.	.
14	38851100	A	G	intergenic	*CLEC14A,LINC00639*	.	.	.
14	38932436	C	T	intergenic	*CLEC14A,LINC00639*	.	.	.
14	38932438	A	T	intergenic	*CLEC14A,LINC00639*	.	.	.
14	38933575	A	G	intergenic	*CLEC14A,LINC00639*	.	.	.

14	39055094	G	A	intergenic	*CLEC14A,LINC00639*	.	.	.
14	39108341	G	A	intergenic	*CLEC14A,LINC00639*	.	.	.
14	39142127	T	C	intergenic	*CLEC14A,LINC00639*	.	.	.
14	39142128	G	A	intergenic	*CLEC14A,LINC00639*	.	.	.
14	39142260	T	C	intergenic	*CLEC14A,LINC00639*	.	.	.
14	39142608	A	AT	intergenic	*CLEC14A,LINC00639*	.	.	.
14	39144274	A	G	intergenic	*CLEC14A,LINC00639*	.	.	.
14	39145113	T	C	intergenic	*CLEC14A,LINC00639*	.	.	.
14	39182606	G	A	intergenic	*CLEC14A,LINC00639*	.	.	.
14	39184735	G	GATCGGAA	intergenic	*CLEC14A,LINC00639*	.	.	.
14	39217203	G	C	intergenic	*CLEC14A,LINC00639*	.	.	.
16	11082361	T	C	intronic	*CLEC16A*	.	.	.
16	70243089	C	G	intergenic	*CLEC18C,SMG1P7*	.	.	.
16	70236107	C	CCTGT	intergenic	*CLEC18C,SMG1P7*	.	.	.
12	10257898	T	A	intergenic	*CLEC1A,CLEC7A*	.	.	.
12	9844888	C	T	intronic	*CLEC2D*	.	.	.
16	78105024	C	T	intergenic	*CLEC3A,WWOX*	.	.	.
12	8300993	A	G	intergenic	*CLEC4A,ZNF705A*	.	.	.
12	8300994	G	A	intergenic	*CLEC4A,ZNF705A*	.	.	.
12	8301753	C	T	intergenic	*CLEC4A,ZNF705A*	.	.	.
12	8302071	C	T	intergenic	*CLEC4A,ZNF705A*	.	.	.
12	8304516	A	G	intergenic	*CLEC4A,ZNF705A*	.	.	.
12	8316387	T	C	intergenic	*CLEC4A,ZNF705A*	.	.	.
12	8676160	G	C	intergenic	*CLEC4D,CLEC4E*	.	.	.
12	8733244	T	A	intergenic	*CLEC4E,AICDA*	.	.	.

19	7884834	TAAA	T	intergenic	*CLEC4GP1,EVI5L*	.	.	.
12	8639084	A	G	intergenic	*CLEC6A,CLEC4D*	.	.	.
X	154545876	G	A	intronic	*CLIC2*	.	.	.
X	154554240	C	A	intronic	*CLIC2*	.	.	.
X	154531786	T	C	intronic	*CLIC2*	.	.	.
X	154552975	C	T	intronic	*CLIC2*	.	.	.
X	154552976	A	G	intronic	*CLIC2*	.	.	.
6	45882508	T	TCTC	intronic	*CLIC5*	.	.	.
6	45916985	A	G	intronic	*CLIC5*	.	.	.
6	46036931	G	T	intronic	*CLIC5*	.	.	.
6	46037255	C	T	intronic	*CLIC5*	.	.	.
6	46092446	A	T	intergenic	*CLIC5,ENPP4*	.	.	.
5	157223895	CATAT	C	intronic	*CLINT1*	.	.	.
5	157216005	T	A	intronic	*CLINT1*	.	.	.
5	157466509	G	GTGTGTGTG	intergenic	*CLINT1,LOC101927697*	.	.	.
5	157590213	CTGTT	C	intergenic	*CLINT1,LOC101927697*	.	.	.
5	157321830	G	A	intergenic	*CLINT1,LOC101927697*	.	.	.
5	157291875	T	C	intergenic	*CLINT1,LOC101927697*	.	.	.
5	157381652	C	T	intergenic	*CLINT1,LOC101927697*	.	.	.
5	157611932	T	C	intergenic	*CLINT1,LOC101927697*	.	.	.
5	157611933	G	T	intergenic	*CLINT1,LOC101927697*	.	.	.
12	122889739	C	T	intronic	*CLIP1*	.	.	.
12	122900698	C	T	intronic	*CLIP1*	.	.	.
12	122946133	C	T	intergenic	*CLIP1,ZCCHC8*	.	.	.
7	73856826	T	G	intergenic	*CLIP2,GTF2IRD1*	.	.	.
2	29389401	AC	A	intronic	*CLIP4*	.	.	.
12	92883431	C	T	intergenic	*CLLU1,C12orf74*	.	.	.
12	93072995	C	A	intergenic	*CLLU1,C12orf74*	.	.	.
12	93092956	G	A	intergenic	*CLLU1,C12orf74*	.	.	.
14	95760743	G	A	intronic	*CLMN*	.	.	.
11	123154896	C	CCTTCCTTC(	intergenic	*CLMP,MIR4493*	.	.	.

11	123188487	G	A	intergenic	*CLMP,MIR4493*	.	.	.
4	10617848	A	G	intronic	*CLNK*	.	.	.
4	10626106	CG	C	intronic	*CLNK*	.	.	.
4	10516331	C	T	intronic	*CLNK*	.	.	.
4	11257739	T	G	intergenic	*CLNK,MIR572*	.	.	.
4	10771763	C	A	intergenic	*CLNK,MIR572*	.	.	.
4	11104159	A	G	intergenic	*CLNK,MIR572*	.	.	.
4	11144608	G	T	intergenic	*CLNK,MIR572*	.	.	.
4	11176481	T	C	intergenic	*CLNK,MIR572*	.	.	.
4	11315731	T	C	intergenic	*CLNK,MIR572*	.	.	.
4	11025841	G	A	intergenic	*CLNK,MIR572*	.	.	.
4	11108747	G	A	intergenic	*CLNK,MIR572*	.	.	.
4	10811295	A	G	intergenic	*CLNK,MIR572*	.	.	.
4	11220951	C	G	intergenic	*CLNK,MIR572*	.	.	.
4	11222251	AAGTTGTA TTGC	A	intergenic	*CLNK,MIR572*	.	.	.
4	11259724	A	T	intergenic	*CLNK,MIR572*	.	.	.
4	11281531	T	A	intergenic	*CLNK,MIR572*	.	.	.
4	11119075	C	G	intergenic	*CLNK,MIR572*	.	.	.
4	10738976	G	GT	intergenic	*CLNK,MIR572*	.	.	.
4	10777063	T	A	intergenic	*CLNK,MIR572*	.	.	.
4	10778119	A	C	intergenic	*CLNK,MIR572*	.	.	.
4	10781091	C	T	intergenic	*CLNK,MIR572*	.	.	.
4	10786052	C	T	intergenic	*CLNK,MIR572*	.	.	.
4	10802822	C	G	intergenic	*CLNK,MIR572*	.	.	.
4	10850390	A	ACAGT	intergenic	*CLNK,MIR572*	.	.	.
4	11046244	T	C	intergenic	*CLNK,MIR572*	.	.	.
4	11046245	G	C	intergenic	*CLNK,MIR572*	.	.	.
4	11047365	C	G	intergenic	*CLNK,MIR572*	.	.	.
4	11048324	G	A	intergenic	*CLNK,MIR572*	.	.	.
4	11048341	G	A	intergenic	*CLNK,MIR572*	.	.	.
4	11065376	T	C	intergenic	*CLNK,MIR572*	.	.	.
4	11066406	T	C	intergenic	*CLNK,MIR572*	.	.	.
4	11079510	G	A	intergenic	*CLNK,MIR572*	.	.	.
4	11103987	A	G	intergenic	*CLNK,MIR572*	.	.	.
4	11104530	A	T	intergenic	*CLNK,MIR572*	.	.	.
11	77332339	AACACACA CACACAC	A	intronic	*CLNS1A*	.	.	.

11	72212626	G	A	intergenic	*CLPB,LINC01537*	.	.	.
5	1328641	G	A	intronic	*CLPTM1L*	.	.	.
5	1328583	G	A	intronic	*CLPTM1L*	.	.	.
3	150751698	C	G	ncRNA_intronic	*CLRN1-AS1*	.	.	.
3	139890909	G	A	intronic	*CLSTN2*	.	.	.
3	140072084	CATATATAT	C	intronic	*CLSTN2*	.	.	.
3	139927913	T	G	intronic	*CLSTN2*	.	.	.
3	140341979	T	C	intergenic	*CLSTN2,TRIM42*	.	.	.
3	140353424	T	A	intergenic	*CLSTN2,TRIM42*	.	.	.
9	36193329	TA	T	intronic	*CLTA*	.	.	.
22	19239943	C	A	intronic	*CLTCL1*	.	.	.
18	606558	C	A	intronic	*CLUL1*	.	.	.
18	599880	G	A	intronic	*CLUL1*	.	.	.
8	62392109	T	A	intronic	*CLVS1*	.	.	.
8	62392111	G	GT	intronic	*CLVS1*	.	.	.
8	62347570	GT	G	intronic	*CLVS1*	.	.	.
8	62252609	G	A	intronic	*CLVS1*	.	.	.
8	62388001	C	T	intronic	*CLVS1*	.	.	.
8	62388284	C	A	intronic	*CLVS1*	.	.	.
8	62396202	A	C	intronic	*CLVS1*	.	.	.
8	62398155	A	T	intronic	*CLVS1*	.	.	.
6	123481784	G	T	intergenic	*CLVS2,TRDN*	.	.	.
6	123438842	C	G	intergenic	*CLVS2,TRDN*	.	.	.
6	123450196	T	A	intergenic	*CLVS2,TRDN*	.	.	.
14	24989533	A	G	intergenic	*CMA1,CTSG*	.	.	.
14	25030240	A	G	intergenic	*CMA1,CTSG*	.	.	.
14	25038100	G	A	intergenic	*CMA1,CTSG*	.	.	.
6	25161672	C	G	intergenic	*CMAHP,LOC101928663*	.	.	.
6	25210866	A	G	intergenic	*CMAHP,LOC101928663*	.	.	.
6	25211947	T	G	intergenic	*CMAHP,LOC101928663*	.	.	.
6	25211949	A	C	intergenic	*CMAHP,LOC101928663*	.	.	.
12	22301417	T	C	intergenic	*CMAS,ST8SIA1*	.	.	.
12	22304149	T	C	intergenic	*CMAS,ST8SIA1*	.	.	.

12	22304627	T	C	intergenic	*CMAS,ST8SIA1*	.	.	.
12	22304628	G	A	intergenic	*CMAS,ST8SIA1*	.	.	.
12	22331792	A	G	intergenic	*CMAS,ST8SIA1*	.	.	.
5	10297337	A	G	intronic	*CMBL*	.	.	.
5	10312403	A	G	intergenic	*CMBL,MARCH6*	.	.	.
3	28315047	A	C	intronic	*CMC1*	.	.	.
3	99731351	C	T	intronic	*CMSS1,FILIP1L*	.	.	.
3	99770313	TA	T	intronic	*CMSS1,FILIP1L*	.	.	.
5	79173108	A	G	intergenic	*CMYA5,LINC01455*	.	.	.
5	79211387	C	A	intergenic	*CMYA5,LINC01455*	.	.	.
8	88373917	AATAT	A	intronic	*CNBD1*	.	.	.
8	87894892	AT	A	intronic	*CNBD1*	.	.	.
8	87894894	T	C	intronic	*CNBD1*	.	.	.
8	87968142	A	G	intronic	*CNBD1*	.	.	.
8	88276912	C	T	intronic	*CNBD1*	.	.	.
8	88287049	A	G	intronic	*CNBD1*	.	.	.
8	88287169	G	A	intronic	*CNBD1*	.	.	.
8	88315445	T	C	intronic	*CNBD1*	.	.	.
8	88375681	G	C	intronic	*CNBD1*	.	.	.
8	88384390	A	G	intronic	*CNBD1*	.	.	.
8	88391191	G	A	intronic	*CNBD1*	.	.	.
8	88792459	C	A	intergenic	*CNBD1,DCAF4L2*	.	.	.
8	88594291	T	C	intergenic	*CNBD1,DCAF4L2*	.	.	.
8	88400180	G	C	intergenic	*CNBD1,DCAF4L2*	.	.	.
8	88402446	T	C	intergenic	*CNBD1,DCAF4L2*	.	.	.
8	88551133	G	A	intergenic	*CNBD1,DCAF4L2*	.	.	.
8	88567022	A	G	intergenic	*CNBD1,DCAF4L2*	.	.	.
8	88662520	A	G	intergenic	*CNBD1,DCAF4L2*	.	.	.
8	88733128	C	T	intergenic	*CNBD1,DCAF4L2*	.	.	.
8	88733129	A	G	intergenic	*CNBD1,DCAF4L2*	.	.	.
8	88733141	A	C	intergenic	*CNBD1,DCAF4L2*	.	.	.
8	88807225	G	GAA	intergenic	*CNBD1,DCAF4L2*	.	.	.
8	88807226	TGC	T	intergenic	*CNBD1,DCAF4L2*	.	.	.
8	88810732	A	T	intergenic	*CNBD1,DCAF4L2*	.	.	.
20	34615730	T	A	intronic	*CNBD2*	.	.	.
20	34615731	G	GATCGGAA	intronic	*CNBD2*	.	.	.

X	150996764	C	T	intergenic	*CNGA2,MAGEA4*	.	.	.
2	99024328	C	T	intergenic	*CNGA3,INPP4A*	.	.	.
8	87798198	T	A	intergenic	*CNGB3,CNBD1*	.	.	.
8	87860750	T	G	intergenic	*CNGB3,CNBD1*	.	.	.
1	225061784	T	C	intergenic	*CNIH3,DNAH14*	.	.	.
1	224559182	A	G	intronic	*CNIH4*	.	.	.
X	21585480	A	T	intronic	*CNKSR2*	.	.	.
X	21469623	C	A	intronic	*CNKSR2*	.	.	.
X	21469624	C	T	intronic	*CNKSR2*	.	.	.
X	21565744	T	A	intronic	*CNKSR2*	.	.	.
X	21567573	A	G	intronic	*CNKSR2*	.	.	.
X	21568464	A	G	intronic	*CNKSR2*	.	.	.
10	104792138	A	G	intronic	*CNNM2*	.	.	.
10	104792139	T	C	intronic	*CNNM2*	.	.	.
16	58607210	CAAAA	C	intronic	*CNOT1*	.	.	.
12	70655678	T	C	intronic	*CNOT2*	.	.	.
12	70693034	A	G	intronic	*CNOT2*	.	.	.
6	42899288	G	A	intronic	*CNPY3*	.	.	.
6	89096400	C	T	intergenic	*CNR1,LOC101928936*	.	.	.
6	89182087	T	C	intergenic	*CNR1,LOC101928936*	.	.	.
6	88966584	C	A	intergenic	*CNR1,LOC101928936*	.	.	.
6	89047490	C	G	intergenic	*CNR1,LOC101928936*	.	.	.
6	89062323	G	A	intergenic	*CNR1,LOC101928936*	.	.	.
6	89065209	T	A	intergenic	*CNR1,LOC101928936*	.	.	.
6	89065415	C	T	intergenic	*CNR1,LOC101928936*	.	.	.
6	89103478	G	A	intergenic	*CNR1,LOC101928936*	.	.	.

1	246779820	ACTGCACTCCAGCCTGAGCAACAGAATGAGACTTATTTTTTTCTTTTTTCTTTTTTGAGTTGGAGTCTCACTCTGTTGCCAGGCTGGAGTGCAGTGGCCCAGTCTTGGCTCC	A	intronic	*CNST*	.	.	.
9	17211457	T	C	intronic	*CNTLN*	.	.	.
12	41113663	G	A	intronic	*CNTN1*	.	.	.
12	41245963	C	T	intronic	*CNTN1*	.	.	.
12	41341213	A	C	intronic	*CNTN1*	.	.	.
12	41210569	G	C	intronic	*CNTN1*	.	.	.
12	41210570	A	T	intronic	*CNTN1*	.	.	.
12	41343899	GGTGTGTGTGTGTGT	G	intronic	*CNTN1*	.	.	.
12	41478551	G	A	intergenic	*CNTN1,PDZRN4*	.	.	.
12	41532500	T	C	intergenic	*CNTN1,PDZRN4*	.	.	.
12	41532501	G	A	intergenic	*CNTN1,PDZRN4*	.	.	.
12	41553223	C	A	intergenic	*CNTN1,PDZRN4*	.	.	.
12	41573850	A	G	intergenic	*CNTN1,PDZRN4*	.	.	.
12	41573851	G	T	intergenic	*CNTN1,PDZRN4*	.	.	.
3	74499302	A	T	intronic	*CNTN3*	.	.	.
3	74849422	C	A	intergenic	*CNTN3,MIR4444-1*	.	.	.
3	74635966	G	C	intergenic	*CNTN3,MIR4444-1*	.	.	.
3	74658632	C	A	intergenic	*CNTN3,MIR4444-1*	.	.	.
3	74617842	C	A	intergenic	*CNTN3,MIR4444-1*	.	.	.
3	75011344	A	G	intergenic	*CNTN3,MIR4444-1*	.	.	.
3	74636035	A	G	intergenic	*CNTN3,MIR4444-1*	.	.	.
3	75048480	G	C	intergenic	*CNTN3,MIR4444-1*	.	.	.
3	74737347	G	A	intergenic	*CNTN3,MIR4444-1*	.	.	.
3	74848791	A	T	intergenic	*CNTN3,MIR4444-1*	.	.	.

3	2793065	G	A	intronic	*CNTN4*	.	.	.
3	3048841	T	C	intronic	*CNTN4*	.	.	.
3	2272830	G	C	intronic	*CNTN4*	.	.	.
3	2670834	C	A	intronic	*CNTN4*	.	.	.
3	2587429	T	G	intronic	*CNTN4*	.	.	.
3	3035873	C	A	intronic	*CNTN4*	.	.	.
3	2323569	T	G	intronic	*CNTN4*	.	.	.
3	2296593	C	T	intronic	*CNTN4*	.	.	.
3	2299046	C	T	intronic	*CNTN4*	.	.	.
3	2299047	T	C	intronic	*CNTN4*	.	.	.
3	2338074	T	C	intronic	*CNTN4*	.	.	.
3	2466875	C	T	intronic	*CNTN4*	.	.	.
3	2468101	A	G	intronic	*CNTN4*	.	.	.
3	2711696	C	A	intronic	*CNTN4*	.	.	.
3	2160374	A	AT	ncRNA_intronic	*CNTN4-AS2*	.	.	.
11	99239948	A	AATATATATA	intronic	*CNTN5*	.	.	.
11	99580734	G	GCCTT	intronic	*CNTN5*	.	.	.
11	99637033	A	G	intronic	*CNTN5*	.	.	.
11	100023002	C	G	intronic	*CNTN5*	.	.	.
11	98962820	G	GAAAA	intronic	*CNTN5*	.	.	.
11	99050328	G	A	intronic	*CNTN5*	.	.	.
11	99444129	G	A	intronic	*CNTN5*	.	.	.
11	99448606	C	T	intronic	*CNTN5*	.	.	.
11	99451171	A	G	intronic	*CNTN5*	.	.	.
11	99583518	C	G	intronic	*CNTN5*	.	.	.
11	99583520	T	A	intronic	*CNTN5*	.	.	.
11	99814173	C	T	intronic	*CNTN5*	.	.	.
11	99814174	A	C	intronic	*CNTN5*	.	.	.
11	99835249	A	C	intronic	*CNTN5*	.	.	.
11	99835250	G	A	intronic	*CNTN5*	.	.	.
11	99835635	T	C	intronic	*CNTN5*	.	.	.
11	99835960	T	C	intronic	*CNTN5*	.	.	.
11	100025909	T	C	intronic	*CNTN5*	.	.	.
11	100027551	G	A	intronic	*CNTN5*	.	.	.
11	100131831	T	C	intronic	*CNTN5*	.	.	.
11	100157713	T	C	intronic	*CNTN5*	.	.	.
11	100372414	A	C	intergenic	*CNTN5,LOC100128386*	.	.	.

11	100295524	G	A	intergenic	*CNTN5,LOC100128386*	.	.	.
11	100301721	A	G	intergenic	*CNTN5,LOC100128386*	.	.	.
11	100327559	C	T	intergenic	*CNTN5,LOC100128386*	.	.	.
11	100336348	C	T	intergenic	*CNTN5,LOC100128386*	.	.	.
11	100365493	C	T	intergenic	*CNTN5,LOC100128386*	.	.	.
11	100460404	G	T	intergenic	*CNTN5,LOC100128386*	.	.	.
11	100460625	G	A	intergenic	*CNTN5,LOC100128386*	.	.	.
11	100477223	G	C	intergenic	*CNTN5,LOC100128386*	.	.	.
3	1205291	G	A	intronic	*CNTN6*	.	.	.
3	1587472	G	C	intergenic	*CNTN6,CNTN4*	.	.	.
3	1642914	C	A	intergenic	*CNTN6,CNTN4*	.	.	.
3	1737252	G	T	intergenic	*CNTN6,CNTN4*	.	.	.
3	2112959	T	A	intergenic	*CNTN6,CNTN4*	.	.	.
3	1588396	C	A	intergenic	*CNTN6,CNTN4*	.	.	.
3	1802578	C	A	intergenic	*CNTN6,CNTN4*	.	.	.
3	1893448	C	CTGTT	intergenic	*CNTN6,CNTN4*	.	.	.
3	1522824	G	A	intergenic	*CNTN6,CNTN4*	.	.	.
3	1693002	A	G	intergenic	*CNTN6,CNTN4*	.	.	.
3	1697383	T	C	intergenic	*CNTN6,CNTN4*	.	.	.
3	1697555	C	T	intergenic	*CNTN6,CNTN4*	.	.	.
3	1958225	T	G	intergenic	*CNTN6,CNTN4*	.	.	.
3	1958227	T	G	intergenic	*CNTN6,CNTN4*	.	.	.
3	1978851	A	G	intergenic	*CNTN6,CNTN4*	.	.	.
3	1979323	T	C	intergenic	*CNTN6,CNTN4*	.	.	.
7	146636266	A	AT	intronic	*CNTNAP2*	.	.	.
7	146188084	C	T	intronic	*CNTNAP2*	.	.	.
7	146623963	A	T	intronic	*CNTNAP2*	.	.	.
7	146683986	G	GT	intronic	*CNTNAP2*	.	.	.
7	146801312	A	T	intronic	*CNTNAP2*	.	.	.
7	147285994	G	T	intronic	*CNTNAP2*	.	.	.

7	147484101	AAGAG	A	intronic	*CNTNAP2*	.	.	.
7	146048636	T	C	intronic	*CNTNAP2*	.	.	.
7	146550574	C	A	intronic	*CNTNAP2*	.	.	.
7	146222366	G	GT	intronic	*CNTNAP2*	.	.	.
7	146287396	C	T	intronic	*CNTNAP2*	.	.	.
7	146655437	G	A	intronic	*CNTNAP2*	.	.	.
7	146817493	G	T	intronic	*CNTNAP2*	.	.	.
7	146824998	T	A	intronic	*CNTNAP2*	.	.	.
7	146148900	C	CTA	intronic	*CNTNAP2*	.	.	.
7	146674374	A	G	intronic	*CNTNAP2*	.	.	.
7	147422547	T	A	intronic	*CNTNAP2*	.	.	.
7	147492844	G	A	intronic	*CNTNAP2*	.	.	.
7	147616356	A	C	intronic	*CNTNAP2*	.	.	.
7	148058976	G	A	intronic	*CNTNAP2*	.	.	.
7	146065573	C	G	intronic	*CNTNAP2*	.	.	.
7	146726014	C	A	intronic	*CNTNAP2*	.	.	.
7	146860866	G	A	intronic	*CNTNAP2*	.	.	.
7	147314030	C	A	intronic	*CNTNAP2*	.	.	.
7	145858858	G	A	intronic	*CNTNAP2*	.	.	.
7	146002985	G	A	intronic	*CNTNAP2*	.	.	.
7	146126849	T	C	intronic	*CNTNAP2*	.	.	.
7	146126850	G	A	intronic	*CNTNAP2*	.	.	.
7	146648526	T	C	intronic	*CNTNAP2*	.	.	.
7	146664496	C	T	intronic	*CNTNAP2*	.	.	.
7	146664682	A	T	intronic	*CNTNAP2*	.	.	.
7	146666587	T	C	intronic	*CNTNAP2*	.	.	.
7	146892888	A	G	intronic	*CNTNAP2*	.	.	.
7	146892889	T	C	intronic	*CNTNAP2*	.	.	.
7	147431321	C	T	intronic	*CNTNAP2*	.	.	.
7	147431323	A	G	intronic	*CNTNAP2*	.	.	.
7	147432035	A	T	intronic	*CNTNAP2*	.	.	.
7	147547279	C	T	intronic	*CNTNAP2*	.	.	.
7	147547281	G	C	intronic	*CNTNAP2*	.	.	.
7	148148194	C	T	intergenic	*CNTNAP2,C7orf33*	.	.	.
7	148229103	G	C	intergenic	*CNTNAP2,C7orf33*	.	.	.
9	39146480	A	G	intronic	*CNTNAP3*	.	.	.
9	44130727	T	C	intergenic	*CNTNAP3B,XLOC_007697*	.	.	.

9	44063285	G	T	intergenic	*CNTNAP3B,XLOC_007697*	.	.	.
9	44058595	T	C	intergenic	*CNTNAP3B,XLOC_007697*	.	.	.
9	43909977	C	A	ncRNA_intronic	*CNTNAP3P2*	.	.	.
16	76325277	A	T	intronic	*CNTNAP4*	.	.	.
16	76400427	T	A	intronic	*CNTNAP4*	.	.	.
16	76610909	G	T	intergenic	*CNTNAP4,LOC101928203*	.	.	.
16	76643065	T	G	intergenic	*CNTNAP4,LOC101928203*	.	.	.
2	125603947	G	T	intronic	*CNTNAP5*	.	.	.
2	125309932	G	T	intronic	*CNTNAP5*	.	.	.
2	124883304	T	C	intronic	*CNTNAP5*	.	.	.
2	125035755	C	A	intronic	*CNTNAP5*	.	.	.
2	125414946	A	T	intronic	*CNTNAP5*	.	.	.
2	125513463	A	G	intronic	*CNTNAP5*	.	.	.
2	125536055	C	G	intronic	*CNTNAP5*	.	.	.
2	125189085	GATAT	G	intronic	*CNTNAP5*	.	.	.
2	125665734	G	T	intronic	*CNTNAP5*	.	.	.
2	125094270	AG	A	intronic	*CNTNAP5*	.	.	.
2	125653193	C	G	intronic	*CNTNAP5*	.	.	.
2	124798198	CTGTGTGTGTGTG	C	intronic	*CNTNAP5*	.	.	.
2	125017930	C	T	intronic	*CNTNAP5*	.	.	.
2	125389283	C	G	intronic	*CNTNAP5*	.	.	.
2	125389285	A	C	intronic	*CNTNAP5*	.	.	.
2	125616581	G	A	intronic	*CNTNAP5*	.	.	.
2	125990226	G	C	intergenic	*CNTNAP5,GYPC*	.	.	.
2	127041366	A	C	intergenic	*CNTNAP5,GYPC*	.	.	.
2	125685804	G	C	intergenic	*CNTNAP5,GYPC*	.	.	.
2	125705847	C	A	intergenic	*CNTNAP5,GYPC*	.	.	.
2	126307242	T	A	intergenic	*CNTNAP5,GYPC*	.	.	.
2	126714137	A	T	intergenic	*CNTNAP5,GYPC*	.	.	.
2	126821649	T	TG	intergenic	*CNTNAP5,GYPC*	.	.	.
2	126821650	T	C	intergenic	*CNTNAP5,GYPC*	.	.	.
2	126969597	G	T	intergenic	*CNTNAP5,GYPC*	.	.	.
2	126742154	G	C	intergenic	*CNTNAP5,GYPC*	.	.	.

2	127033829	A	T	intergenic	*CNTNAP5,GYPC*	.	.	.
2	126680941	CATGCAT	C	intergenic	*CNTNAP5,GYPC*	.	.	.
2	126924620	A	G	intergenic	*CNTNAP5,GYPC*	.	.	.
2	127259728	A	G	intergenic	*CNTNAP5,GYPC*	.	.	.
2	126160085	G	A	intergenic	*CNTNAP5,GYPC*	.	.	.
2	125787888	T	G	intergenic	*CNTNAP5,GYPC*	.	.	.
2	126016643	A	AT	intergenic	*CNTNAP5,GYPC*	.	.	.
2	126174881	A	C	intergenic	*CNTNAP5,GYPC*	.	.	.
2	126380844	A	T	intergenic	*CNTNAP5,GYPC*	.	.	.
2	126382353	A	G	intergenic	*CNTNAP5,GYPC*	.	.	.
2	126520470	TGAGAGA	T	intergenic	*CNTNAP5,GYPC*	.	.	.
2	126525126	C	T	intergenic	*CNTNAP5,GYPC*	.	.	.
2	126526477	A	C	intergenic	*CNTNAP5,GYPC*	.	.	.
2	126635612	A	G	intergenic	*CNTNAP5,GYPC*	.	.	.
2	126832970	A	AC	intergenic	*CNTNAP5,GYPC*	.	.	.
2	126844557	T	C	intergenic	*CNTNAP5,GYPC*	.	.	.
2	126844765	C	G	intergenic	*CNTNAP5,GYPC*	.	.	.
2	126860935	T	C	intergenic	*CNTNAP5,GYPC*	.	.	.
2	126861388	T	A	intergenic	*CNTNAP5,GYPC*	.	.	.
2	126876797	A	G	intergenic	*CNTNAP5,GYPC*	.	.	.
2	126876799	A	G	intergenic	*CNTNAP5,GYPC*	.	.	.
2	126920256	G	A	intergenic	*CNTNAP5,GYPC*	.	.	.
2	126922110	A	G	intergenic	*CNTNAP5,GYPC*	.	.	.
2	126927429	T	TAGATCGGA	intergenic	*CNTNAP5,GYPC*	.	.	.
2	126955273	T	A	intergenic	*CNTNAP5,GYPC*	.	.	.
2	126956592	T	C	intergenic	*CNTNAP5,GYPC*	.	.	.
2	127280640	A	T	intergenic	*CNTNAP5,GYPC*	.	.	.
2	127338319	T	C	intergenic	*CNTNAP5,GYPC*	.	.	.
9	123854441	T	C	intronic	*CNTRL*	.	.	.
9	123891112	A	G	intronic	*CNTRL*	.	.	.
9	123892057	A	G	intronic	*CNTRL*	.	.	.
7	43713241	C	G	intronic	*COA1*	.	.	.
7	51262710	G	T	intronic	*COBL*	.	.	.
7	51369161	G	A	intronic	*COBL*	.	.	.
7	51474087	AC	A	intergenic	*COBL,POM121L12*	.	.	.
7	51883486	T	C	intergenic	*COBL,POM121L12*	.	.	.
7	52900303	T	C	intergenic	*COBL,POM121L12*	.	.	.
7	52922375	C	A	intergenic	*COBL,POM121L12*	.	.	.

7	52012493	G	T	intergenic	*COBL,POM121L12*	.	.	.
7	52450842	G	T	intergenic	*COBL,POM121L12*	.	.	.
7	52615636	G	A	intergenic	*COBL,POM121L12*	.	.	.
7	52625828	G	C	intergenic	*COBL,POM121L12*	.	.	.
7	52497408	C	G	intergenic	*COBL,POM121L12*	.	.	.
7	51903641	C	T	intergenic	*COBL,POM121L12*	.	.	.
7	52575307	G	C	intergenic	*COBL,POM121L12*	.	.	.
7	52882308	T	A	intergenic	*COBL,POM121L12*	.	.	.
7	52212190	G	T	intergenic	*COBL,POM121L12*	.	.	.
7	51765934	A	G	intergenic	*COBL,POM121L12*	.	.	.
7	51780310	G	T	intergenic	*COBL,POM121L12*	.	.	.
7	52825853	C	G	intergenic	*COBL,POM121L12*	.	.	.
7	52940566	A	T	intergenic	*COBL,POM121L12*	.	.	.
7	52304497	G	T	intergenic	*COBL,POM121L12*	.	.	.
7	52659756	G	T	intergenic	*COBL,POM121L12*	.	.	.
7	51599983	A	C	intergenic	*COBL,POM121L12*	.	.	.
7	51601248	G	A	intergenic	*COBL,POM121L12*	.	.	.
7	51601361	A	C	intergenic	*COBL,POM121L12*	.	.	.
7	51621951	G	A	intergenic	*COBL,POM121L12*	.	.	.
7	51623573	T	C	intergenic	*COBL,POM121L12*	.	.	.
7	51663804	T	C	intergenic	*COBL,POM121L12*	.	.	.
7	51856225	G	A	intergenic	*COBL,POM121L12*	.	.	.
7	51883897	C	T	intergenic	*COBL,POM121L12*	.	.	.
7	51904624	A	G	intergenic	*COBL,POM121L12*	.	.	.
7	51904990	T	C	intergenic	*COBL,POM121L12*	.	.	.
7	51959841	C	A	intergenic	*COBL,POM121L12*	.	.	.
7	51960249	G	A	intergenic	*COBL,POM121L12*	.	.	.
7	52172761	C	T	intergenic	*COBL,POM121L12*	.	.	.
7	52358716	T	C	intergenic	*COBL,POM121L12*	.	.	.
7	52648876	A	G	intergenic	*COBL,POM121L12*	.	.	.
7	52712590	A	G	intergenic	*COBL,POM121L12*	.	.	.
7	52962685	G	T	intergenic	*COBL,POM121L12*	.	.	.
7	52997553	G	T	intergenic	*COBL,POM121L12*	.	.	.
7	52998390	G	A	intergenic	*COBL,POM121L12*	.	.	.
2	165611568	G	A	intronic	*COBLL1*	.	.	.
1	230789831	A	G	intronic	*COG2*	.	.	.
1	230790753	A	G	intronic	*COG2*	.	.	.
7	107048416	A	G	intronic	*COG5*	.	.	.

7	107116821	G	T	intronic	*COG5*	.	.	.
7	106992025	T	C	intronic	*COG5*	.	.	.
7	107153362	C	A	intronic	*COG5*	.	.	.
7	107160886	T	C	intronic	*COG5*	.	.	.
7	107160887	G	A	intronic	*COG5*	.	.	.
7	107174151	C	T	intronic	*COG5*	.	.	.
13	40319573	T	C	intronic	*COG6*	.	.	.
13	40622166	C	G	intergenic	*COG6,LINC00332*	.	.	.
13	40573027	T	G	intergenic	*COG6,LINC00332*	.	.	.
13	40427052	T	C	intergenic	*COG6,LINC00332*	.	.	.
16	23417611	C	A	intronic	*COG7*	.	.	.
1	103395484	A	T	intronic	*COL11A1*	.	.	.
1	103746135	G	A	intergenic	*COL11A1,LOC101 928436*	.	.	.
1	103753185	C	A	intergenic	*COL11A1,LOC101 928436*	.	.	.
10	71605238	C	T	intronic	*COL13A1*	.	.	.
8	121212291	T	TATATATATC	intronic	*COL14A1*	.	.	.
8	121215776	G	A	intronic	*COL14A1*	.	.	.
8	121246040	A	G	intronic	*COL14A1*	.	.	.
8	121343191	G	A	intronic	*COL14A1*	.	.	.
21	46878378	C	A	intronic	*COL18A1*	.	.	.
21	46854220	C	T	intronic	*COL18A1*	.	.	.
6	70610748	A	G	intronic	*COL19A1*	.	.	.
6	70922703	C	A	downstream	*COL19A1*	.	.	.
6	70648346	T	C	intronic	*COL19A1*	.	.	.
6	70775356	G	A	intronic	*COL19A1*	.	.	.
6	70775805	T	C	intronic	*COL19A1*	.	.	.
7	94108293	CTT	C	intergenic	*COL1A2,CASD1*	.	.	.
6	56003411	C	T	intronic	*COL21A1*	.	.	.
6	55963503	A	G	intronic	*COL21A1*	.	.	.
6	56056785	G	T	intronic	*COL21A1*	.	.	.
6	56170932	C	G	intergenic	*COL21A1,DST*	.	.	.
6	56143490	A	C	intergenic	*COL21A1,DST*	.	.	.
8	139784630	C	T	intronic	*COL22A1*	.	.	.
8	139797363	G	A	intronic	*COL22A1*	.	.	.
8	139717803	C	A	intronic	*COL22A1*	.	.	.
8	139718402	A	G	intronic	*COL22A1*	.	.	.

8	139720135	A	G	intronic	*COL22A1*	.	.	.
8	139754527	T	G	intronic	*COL22A1*	.	.	.
8	140101207	T	C	intergenic	*COL22A1,KCNK9*	.	.	.
8	140060159	T	C	intergenic	*COL22A1,KCNK9*	.	.	.
8	140607429	C	A	intergenic	*COL22A1,KCNK9*	.	.	.
8	140116801	C	A	intergenic	*COL22A1,KCNK9*	.	.	.
8	140116802	T	A	intergenic	*COL22A1,KCNK9*	.	.	.
8	140537937	C	A	intergenic	*COL22A1,KCNK9*	.	.	.
8	140474740	G	C	intergenic	*COL22A1,KCNK9*	.	.	.
5	177996209	T	C	intronic	*COL23A1*	.	.	.
5	177708101	G	A	intronic	*COL23A1*	.	.	.
1	86240578	C	T	intronic	*COL24A1*	.	.	.
1	86253633	C	T	intronic	*COL24A1*	.	.	.
1	86290978	A	T	intronic	*COL24A1*	.	.	.
1	86297966	C	T	intronic	*COL24A1*	.	.	.
1	86338554	A	C	intronic	*COL24A1*	.	.	.
1	86338752	C	A	intronic	*COL24A1*	.	.	.
1	86338754	G	A	intronic	*COL24A1*	.	.	.
1	86390296	C	T	intronic	*COL24A1*	.	.	.
1	86391539	A	C	intronic	*COL24A1*	.	.	.
1	86539087	G	A	intronic	*COL24A1*	.	.	.
1	86797643	G	A	intergenic	*COL24A1,ODF2L*	.	.	.
1	86799941	T	C	intergenic	*COL24A1,ODF2L*	.	.	.
4	110214577	C	A	intronic	*COL25A1*	.	.	.
4	109775597	A	T	intronic	*COL25A1*	.	.	.
4	110061846	C	A	intronic	*COL25A1*	.	.	.
4	110273142	CACAG	C	intergenic	*COL25A1,SEC24B-AS1*	.	.	.
4	110264595	T	C	intergenic	*COL25A1,SEC24B-AS1*	.	.	.
4	110264596	G	C	intergenic	*COL25A1,SEC24B-AS1*	.	.	.
4	110264799	T	C	intergenic	*COL25A1,SEC24B-AS1*	.	.	.
7	101126723	C	T	intronic	*COL26A1*	.	.	.
7	101126724	A	G	intronic	*COL26A1*	.	.	.
7	7566268	T	A	intronic	*COL28A1*	.	.	.
7	7430372	C	T	intronic	*COL28A1*	.	.	.

5	74684650	G	A	intronic	*COL4A3BP*	.	.	.
5	74785885	C	G	intronic	*COL4A3BP*	.	.	.
5	74785886	G	C	intronic	*COL4A3BP*	.	.	.
5	74791687	C	T	intronic	*COL4A3BP*	.	.	.
2	227911920	T	C	intronic	*COL4A4*	.	.	.
2	227932139	T	A	intronic	*COL4A4*	.	.	.
2	227934771	C	T	intronic	*COL4A4*	.	.	.
2	227938964	T	C	intronic	*COL4A4*	.	.	.
2	227941399	G	A	intronic	*COL4A4*	.	.	.
X	107755500	T	C	intronic	*COL4A5*	.	.	.
X	107695397	G	C	intronic	*COL4A5*	.	.	.
X	107774157	C	T	intronic	*COL4A5*	.	.	.
X	107786304	A	G	intronic	*COL4A5*	.	.	.
X	107873423	T	A	intronic	*COL4A5*	.	.	.
X	107875223	A	G	intronic	*COL4A5*	.	.	.
X	107926242	A	G	intronic	*COL4A5*	.	.	.
X	107400280	G	T	exonic	*COL4A6*	nonsynonymous_SNV	.	.
X	107594969	G	T	intronic	*COL4A6*	.	.	.
X	107522915	A	T	intronic	*COL4A6*	.	.	.
X	107523002	T	A	intronic	*COL4A6*	.	.	.
X	107523370	A	G	intronic	*COL4A6*	.	.	.
X	107573278	T	TTA	intronic	*COL4A6*	.	.	.
2	190134808	T	C	intergenic	*COL5A2,WDR75*	.	.	.
2	190135589	T	C	intergenic	*COL5A2,WDR75*	.	.	.
2	190135590	C	A	intergenic	*COL5A2,WDR75*	.	.	.
2	190188846	A	T	intergenic	*COL5A2,WDR75*	.	.	.
2	190189276	C	A	intergenic	*COL5A2,WDR75*	.	.	.
2	190189278	G	A	intergenic	*COL5A2,WDR75*	.	.	.
2	190218472	G	T	intergenic	*COL5A2,WDR75*	.	.	.
2	190268242	T	C	intergenic	*COL5A2,WDR75*	.	.	.
2	190268243	A	C	intergenic	*COL5A2,WDR75*	.	.	.
2	190299625	G	A	intergenic	*COL5A2,WDR75*	.	.	.
2	190300995	A	G	intergenic	*COL5A2,WDR75*	.	.	.
2	238371086	T	C	intergenic	*COL6A3,MLPH*	.	.	.
2	238371790	A	C	intergenic	*COL6A3,MLPH*	.	.	.
3	15233111	T	A	ncRNA_intronic	*COL6A4P1*	.	.	.
3	130172398	T	G	intronic	*COL6A5*	.	.	.

3	130290536	A	T	intronic	*COL6A6*	.	.	.
6	70943938	AGTGT	A	intronic	*COL9A1*	.	.	.
6	71027523	C	T	intergenic	*COL9A1,EVADR*	.	.	.
8	120130485	A	T	intergenic	*COLEC10,MAL2*	.	.	.
8	120214402	C	A	intergenic	*COLEC10,MAL2*	.	.	.
8	120122737	A	G	intergenic	*COLEC10,MAL2*	.	.	.
8	120124370	G	A	intergenic	*COLEC10,MAL2*	.	.	.
18	465494	G	T	intronic	*COLEC12*	.	.	.
18	336571	C	T	intronic	*COLEC12*	.	.	.
18	569305	G	A	intergenic	*COLEC12,CETN1*	.	.	.
5	115458406	C	T	intronic	*COMMD10*	.	.	.
5	115506666	G	A	intronic	*COMMD10*	.	.	.
5	115539637	G	A	intronic	*COMMD10*	.	.	.
5	115668320	G	T	intergenic	*COMMD10,LOC101927190*	.	.	.
5	115668391	T	G	intergenic	*COMMD10,LOC101927190*	.	.	.
19	18926082	G	T	intergenic	*COMP,UPF1*	.	.	.
10	77033464	G	GA	intergenic	*COMTD1,ZNF503-AS1*	.	.	.
11	14504325	G	A	intronic	*COPB1*	.	.	.
3	139082830	A	G	intronic	*COPB2*	.	.	.
7	130266206	C	T	intronic	*COPG2*	.	.	.
7	130268296	T	C	intronic	*COPG2*	.	.	.
7	130271978	A	T	intronic	*COPG2*	.	.	.
7	130321077	T	G	intronic	*COPG2*	.	.	.
7	130321341	T	C	intronic	*COPG2*	.	.	.
8	67959188	C	G	intronic	*COPS5*	.	.	.
2	232753742	A	G	intergenic	*COPS7B,MIR1471*	.	.	.
2	232738186	C	T	intergenic	*COPS7B,MIR1471*	.	.	.
2	238078239	T	C	intergenic	*COPS8,COL6A3*	.	.	.
2	238153132	C	T	intergenic	*COPS8,COL6A3*	.	.	.
2	238096323	C	T	intergenic	*COPS8,COL6A3*	.	.	.
2	238131253	C	T	intergenic	*COPS8,COL6A3*	.	.	.
2	238156321	A	G	intergenic	*COPS8,COL6A3*	.	.	.
16	19087565	A	C	intronic	*COQ7*	.	.	.
4	47618303	C	T	intronic	*CORIN*	.	.	.
4	47651965	A	T	intronic	*CORIN*	.	.	.

4	47655747	G	A	intronic	*CORIN*	.	.	.
4	47608757	A	T	intronic	*CORIN*	.	.	.
4	47613069	T	G	intronic	*CORIN*	.	.	.
15	69032256	T	C	intergenic	*CORO2B,ANP32A*	.	.	.
16	4469555	T	C	intergenic	*CORO7-PAM16,DNAJA3*	.	.	.
17	13975093	T	A	intronic	*COX10*	.	.	.
17	13981728	T	C	intronic	*COX10*	.	.	.
17	13992613	G	A	intronic	*COX10*	.	.	.
17	13992930	C	A	intronic	*COX10*	.	.	.
17	14051702	T	G	intronic	*COX10*	.	.	.
17	14064604	A	G	intronic	*COX10*	.	.	.
17	14064955	T	A	intronic	*COX10*	.	.	.
17	14065757	A	C	intronic	*COX10*	.	.	.
17	14067342	T	A	intronic	*COX10*	.	.	.
17	14067436	G	T	intronic	*COX10*	.	.	.
17	14079132	G	A	intronic	*COX10*	.	.	.
20	30230667	G	A	intronic	*COX4I2*	.	.	.
15	75228998	C	T	intronic	*COX5A*	.	.	.
19	36144834	A	G	intronic	*COX6B1*	.	.	.
19	36144835	C	A	intronic	*COX6B1*	.	.	.
19	36153102	G	T	intergenic	*COX6B1,UPK1A*	.	.	.
4	46886314	T	C	intronic	*COX7B2*	.	.	.
4	46879364	G	A	intronic	*COX7B2*	.	.	.
4	46875521	C	T	intronic	*COX7B2*	.	.	.
5	86021495	C	G	intergenic	*COX7C,LOC100505878*	.	.	.
5	85960940	A	G	intergenic	*COX7C,LOC100505878*	.	.	.
5	86024697	A	G	intergenic	*COX7C,LOC100505878*	.	.	.
5	86025379	GT	G	intergenic	*COX7C,LOC100505878*	.	.	.
5	86026198	T	C	intergenic	*COX7C,LOC100505878*	.	.	.
3	148603478	T	C	intronic	*CPA3*	.	.	.
7	129933753	G	A	intronic	*CPA4*	.	.	.
7	129983187	A	T	intergenic	*CPA4,CPA5*	.	.	.

8	68530888	T	C	intronic	*CPA6*	.	.	.
8	68484170	G	GATGTGATA	intronic	*CPA6*	.	.	.
8	68502870	G	A	intronic	*CPA6*	.	.	.
8	68505226	G	A	intronic	*CPA6*	.	.	.
8	68727649	A	C	intergenic	*CPA6,PREX2*	.	.	.
8	68727651	C	A	intergenic	*CPA6,PREX2*	.	.	.
19	17076953	T	G	intronic	*CPAMD8*	.	.	.
3	148578519	A	T	downstream	*CPB1*	.	.	.
3	148578520	G	A	downstream	*CPB1*	.	.	.
17	28736199	A	C	intronic	*CPD*	.	.	.
17	28780002	C	T	intronic	*CPD*	.	.	.
10	93812361	C	T	intronic	*CPEB3*	.	.	.
10	93894366	G	A	intronic	*CPEB3*	.	.	.
10	93894752	A	C	intronic	*CPEB3*	.	.	.
7	120708230	A	T	intronic	*CPED1*	.	.	.
7	120863327	G	A	intronic	*CPED1*	.	.	.
7	120892760	C	T	intronic	*CPED1*	.	.	.
7	120955018	A	T	intergenic	*CPED1,WNT16*	.	.	.
7	120955019	A	T	intergenic	*CPED1,WNT16*	.	.	.
4	800656	T	C	intronic	*CPLX1*	.	.	.
5	175356970	G	A	intergenic	*CPLX2,THOC3*	.	.	.
12	69291357	G	A	intronic	*CPM*	.	.	.
12	69291358	T	A	intronic	*CPM*	.	.	.
12	69322065	C	T	intronic	*CPM*	.	.	.
3	194066369	A	G	intronic	*CPN2*	.	.	.
3	131751643	C	G	intronic	*CPNE4*	.	.	.
6	36814261	C	T	intergenic	*CPNE5,PPIL1*	.	.	.
12	39082726	T	C	intronic	*CPNE8*	.	.	.
12	39129163	A	G	intronic	*CPNE8*	.	.	.
12	39199770	C	T	intronic	*CPNE8*	.	.	.
12	39213071	T	C	intronic	*CPNE8*	.	.	.
12	39646815	C	A	intergenic	*CPNE8,KIF21A*	.	.	.
12	39396720	T	C	intergenic	*CPNE8,KIF21A*	.	.	.
12	39360102	T	C	intergenic	*CPNE8,KIF21A*	.	.	.
12	39586283	T	A	intergenic	*CPNE8,KIF21A*	.	.	.
12	39589138	T	C	intergenic	*CPNE8,KIF21A*	.	.	.
12	39599672	A	T	intergenic	*CPNE8,KIF21A*	.	.	.
12	39604600	T	C	intergenic	*CPNE8,KIF21A*	.	.	.

12	39656917	G	A	intergenic	*CPNE8,KIF21A*	.	.	.
3	9762757	T	C	intronic	*CPNE9*	.	.	.
2	207885643	T	C	intergenic	*CPO,KLF7*	.	.	.
3	98403793	T	G	intergenic	*CPOX,ST3GAL6-AS1*	.	.	.
16	12764746	C	T	intronic	*CPPED1*	.	.	.
8	97736718	GACACACAC	G	intronic	*CPQ*	.	.	.
8	97736362	TTCTC	T	intronic	*CPQ*	.	.	.
8	97675979	TAG	T	intronic	*CPQ*	.	.	.
2	211497133	T	G	intronic	*CPS1*	.	.	.
2	211407012	A	G	intronic	*CPS1*	.	.	.
2	211374390	G	A	intronic	*CPS1*	.	.	.
2	211378392	C	T	intronic	*CPS1*	.	.	.
2	211479442	A	G	intronic	*CPS1*	.	.	.
2	211816857	T	C	intergenic	*CPS1,ERBB4*	.	.	.
2	212201754	A	T	intergenic	*CPS1,ERBB4*	.	.	.
2	211635763	G	A	intergenic	*CPS1,ERBB4*	.	.	.
2	211636559	G	A	intergenic	*CPS1,ERBB4*	.	.	.
2	211845622	C	G	intergenic	*CPS1,ERBB4*	.	.	.
2	211846275	A	C	intergenic	*CPS1,ERBB4*	.	.	.
14	92620202	A	C	intronic	*CPSF2*	.	.	.
14	92762108	T	C	intergenic	*CPSF2,SLC24A4*	.	.	.
14	92696523	C	G	intergenic	*CPSF2,SLC24A4*	.	.	.
14	92697202	A	G	intergenic	*CPSF2,SLC24A4*	.	.	.
7	29108579	G	A	intronic	*CPVL*	.	.	.
X	88005825	G	T	intronic	*CPXCR1*	.	.	.
X	88284216	G	T	intergenic	*CPXCR1,TGIF2LX*	.	.	.
X	88136684	C	A	intergenic	*CPXCR1,TGIF2LX*	.	.	.
X	88707740	C	T	intergenic	*CPXCR1,TGIF2LX*	.	.	.
X	88231127	A	G	intergenic	*CPXCR1,TGIF2LX*	.	.	.
X	88061738	A	T	intergenic	*CPXCR1,TGIF2LX*	.	.	.
X	88724649	T	G	intergenic	*CPXCR1,TGIF2LX*	.	.	.
X	88953216	A	T	intergenic	*CPXCR1,TGIF2LX*	.	.	.
X	88037161	G	A	intergenic	*CPXCR1,TGIF2LX*	.	.	.
X	88042823	T	TTG	intergenic	*CPXCR1,TGIF2LX*	.	.	.
X	88042824	CAA	C	intergenic	*CPXCR1,TGIF2LX*	.	.	.
X	88058043	C	A	intergenic	*CPXCR1,TGIF2LX*	.	.	.

X	88073741	A	C	intergenic	*CPXCR1,TGIF2LX*	.	.	.
X	88074430	A	G	intergenic	*CPXCR1,TGIF2LX*	.	.	.
X	88182591	G	A	intergenic	*CPXCR1,TGIF2LX*	.	.	.
X	88184134	T	C	intergenic	*CPXCR1,TGIF2LX*	.	.	.
X	88184135	C	A	intergenic	*CPXCR1,TGIF2LX*	.	.	.
X	88241155	G	T	intergenic	*CPXCR1,TGIF2LX*	.	.	.
X	88316206	G	A	intergenic	*CPXCR1,TGIF2LX*	.	.	.
X	88369797	G	A	intergenic	*CPXCR1,TGIF2LX*	.	.	.
X	88499234	A	G	intergenic	*CPXCR1,TGIF2LX*	.	.	.
X	88549362	G	C	intergenic	*CPXCR1,TGIF2LX*	.	.	.
X	88579100	G	A	intergenic	*CPXCR1,TGIF2LX*	.	.	.
X	88774767	T	G	intergenic	*CPXCR1,TGIF2LX*	.	.	.
X	89065368	G	T	intergenic	*CPXCR1,TGIF2LX*	.	.	.
10	125518877	G	T	intronic	*CPXM2*	.	.	.
10	125752332	T	C	intergenic	*CPXM2,CHST15*	.	.	.
10	125752333	G	A	intergenic	*CPXM2,CHST15*	.	.	.
10	125752512	T	C	intergenic	*CPXM2,CHST15*	.	.	.
4	8821122	C	T	intergenic	*CPZ,HMX1*	.	.	.
4	8710199	G	A	intergenic	*CPZ,HMX1*	.	.	.
4	8730169	C	T	intergenic	*CPZ,HMX1*	.	.	.
1	207678153	G	A	intronic	*CR1*	.	.	.
1	207668641	G	C	upstream	*CR1*	.	.	.
1	207668643	C	T	upstream	*CR1*	.	.	.
1	207834641	A	T	intronic	*CR1L*	.	.	.
15	78671460	A	G	intergenic	*CRABP1,IREB2*	.	.	.
12	3815868	G	A	intronic	*CRACR2A*	.	.	.
12	3897311	T	C	intergenic	*CRACR2A,PARP11*	.	.	.
12	94398161	G	T	intergenic	*CRADD,PLXNC1*	.	.	.
12	94398886	T	C	intergenic	*CRADD,PLXNC1*	.	.	.
12	94399805	T	C	intergenic	*CRADD,PLXNC1*	.	.	.
12	94400497	T	C	intergenic	*CRADD,PLXNC1*	.	.	.
12	94460485	G	A	intergenic	*CRADD,PLXNC1*	.	.	.
15	92062542	G	T	intergenic	*CRAT37,SLCO3A1*	.	.	.
15	92126454	A	G	intergenic	*CRAT37,SLCO3A1*	.	.	.
12	128567320	C	A	intergenic	*CRAT8,LOC100996679*	.	.	.
1	197289392	T	A	intronic	*CRB1*	.	.	.

1	197208064	G	A	intronic	*CRB1*	.	.	.
1	197209963	T	A	intronic	*CRB1*	.	.	.
1	197261296	G	A	intronic	*CRB1*	.	.	.
1	197261943	T	A	intronic	*CRB1*	.	.	.
3	3833968	A	G	intergenic	*CRBN,LRRN1*	.	.	.
3	3358829	T	C	intergenic	*CRBN,LRRN1*	.	.	.
3	3357120	T	C	intergenic	*CRBN,LRRN1*	.	.	.
3	3662602	C	A	intergenic	*CRBN,LRRN1*	.	.	.
3	3452086	T	C	intergenic	*CRBN,LRRN1*	.	.	.
3	3453069	T	C	intergenic	*CRBN,LRRN1*	.	.	.
3	3521926	A	C	intergenic	*CRBN,LRRN1*	.	.	.
3	3586125	C	T	intergenic	*CRBN,LRRN1*	.	.	.
7	65605599	G	A	intronic	*CRCP*	.	.	.
1	152501974	C	T	intergenic	*CRCT1,LCE3E*	.	.	.
1	152523640	A	G	intergenic	*CRCT1,LCE3E*	.	.	.
11	46343075	G	A	downstream	*CREB3L1*	.	.	.
7	137653061	G	C	intronic	*CREB3L2*	.	.	.
7	137653063	T	C	intronic	*CREB3L2*	.	.	.
7	137722768	T	C	intergenic	*CREB3L2,AKR1D1*	.	.	.
7	137724606	C	T	intergenic	*CREB3L2,AKR1D1*	.	.	.
7	137726336	A	C	intergenic	*CREB3L2,AKR1D1*	.	.	.
7	137741856	G	A	intergenic	*CREB3L2,AKR1D1*	.	.	.
19	4154850	G	T	intronic	*CREB3L3*	.	.	.
7	28453970	A	G	intronic	*CREB5*	.	.	.
7	28742500	C	T	intronic	*CREB5*	.	.	.
7	28637936	G	A	intronic	*CREB5*	.	.	.
7	28869285	G	A	intergenic	*CREB5,TRIL*	.	.	.
8	67092185	C	T	intergenic	*CRH,LINC00967*	.	.	.
5	76261968	C	T	intronic	*CRHBP*	.	.	.
5	76283825	G	C	intergenic	*CRHBP,AGGF1*	.	.	.
2	36640851	G	T	intronic	*CRIM1*	.	.	.
2	36627356	G	A	intronic	*CRIM1*	.	.	.
6	49864981	T	C	intergenic	*CRISP1,DEFB133*	.	.	.
6	49691951	G	A	intergenic	*CRISP2,CRISP3*	.	.	.
6	49733931	A	T	intergenic	*CRISP3,PGK2*	.	.	.
6	49739020	A	T	intergenic	*CRISP3,PGK2*	.	.	.
8	75943980	A	G	intronic	*CRISPLD1*	.	.	.
8	75966090	C	G	intergenic	*CRISPLD1,CASC9*	.	.	.

8	76024004	G	A	intergenic	*CRISPLD1,CASC9*	.	.	.
8	75973248	A	G	intergenic	*CRISPLD1,CASC9*	.	.	.
8	75992779	G	A	intergenic	*CRISPLD1,CASC9*	.	.	.
8	76006220	G	A	intergenic	*CRISPLD1,CASC9*	.	.	.
8	76017512	TCTTTC	T	intergenic	*CRISPLD1,CASC9*	.	.	.
8	76063715	T	C	intergenic	*CRISPLD1,CASC9*	.	.	.
8	76088616	T	C	intergenic	*CRISPLD1,CASC9*	.	.	.
8	76115221	T	C	intergenic	*CRISPLD1,CASC9*	.	.	.
8	76115222	A	C	intergenic	*CRISPLD1,CASC9*	.	.	.
8	76116786	C	T	intergenic	*CRISPLD1,CASC9*	.	.	.
X	1322213	T	C	intronic	*CRLF2*	.	.	.
X	1361559	G	A	intergenic	*CRLF2,CSF2RA*	.	.	.
4	5887562	TCATTTGACAAGTTAATGCAAACCCACTAACAGCCAATGGGTTTGCCTTAACTTGTCAAATGACTACTATGGAACACGTTAAGGTGCAGC	T	intronic	*CRMP1*	.	.	.
4	5868832	C	A	intronic	*CRMP1*	.	.	.
4	5839405	T	TAAATAAAA	intronic	*CRMP1*	.	.	.
1	152462152	C	T	intergenic	*CRNN,LCE5A*	.	.	.
1	16838476	G	A	intergenic	*CROCCP3,FAM231B*	.	.	.
1	16857136	C	T	intergenic	*CROCCP3,FAM231B*	.	.	.
1	159731098	C	T	intergenic	*CRP,DUSP23*	.	.	.
5	79668321	T	C	intergenic	*CRSP8P,ZFYVE16*	.	.	.
5	79688683	A	T	intergenic	*CRSP8P,ZFYVE16*	.	.	.
5	79689759	C	T	intergenic	*CRSP8P,ZFYVE16*	.	.	.
12	107478183	A	G	intronic	*CRY1*	.	.	.
12	107607590	G	A	intergenic	*CRY1,BTBD11*	.	.	.
12	107606892	T	C	intergenic	*CRY1,BTBD11*	.	.	.

22	27023331	T	C	intronic	*CRYBA4*	.	.	.
22	25622520	T	C	intronic	*CRYBB2*	.	.	.
22	25673760	T	C	intergenic	*CRYBB2,IGLL3P*	.	.	.
22	25892319	G	A	intergenic	*CRYBB2P1,ADRBK2*	.	.	.
7	151136277	G	A	intronic	*CRYGN*	.	.	.
13	21024850	A	G	intronic	*CRYL1*	.	.	.
13	21091605	G	A	intronic	*CRYL1*	.	.	.
16	21341090	A	G	intergenic	*CRYM-AS1,SNX29P1*	.	.	.
16	21344019	A	G	intergenic	*CRYM-AS1,SNX29P1*	.	.	.
1	115299666	A	G	intronic	*CSDE1*	.	.	.
5	149476393	C	G	intronic	*CSF1R*	.	.	.
5	131445047	AGCATTAG	A	intergenic	*CSF2,P4HA2-AS1*	.	.	.
5	131444121	C	T	intergenic	*CSF2,P4HA2-AS1*	.	.	.
5	131459971	C	T	intergenic	*CSF2,P4HA2-AS1*	.	.	.
5	131490449	A	G	intergenic	*CSF2,P4HA2-AS1*	.	.	.
X	1393519	TGGTATGTAA	T	intronic	*CSF2RA*	.	.	.
1	37074350	G	A	intergenic	*CSF3R,GRIK3*	.	.	.
8	19518977	G	A	intronic	*CSGALNACT1*	.	.	.
8	4383317	G	A	intronic	*CSMD1*	.	.	.
8	4671718	TTGTG	T	intronic	*CSMD1*	.	.	.
8	4517138	G	T	intronic	*CSMD1*	.	.	.
8	2894637	G	T	intronic	*CSMD1*	.	.	.
8	3136470	AG	A	intronic	*CSMD1*	.	.	.
8	3225154	G	GA	intronic	*CSMD1*	.	.	.
8	3252581	A	T	intronic	*CSMD1*	.	.	.
8	4337974	C	A	intronic	*CSMD1*	.	.	.
8	4680587	T	C	intronic	*CSMD1*	.	.	.
8	3017525	G	T	intronic	*CSMD1*	.	.	.
8	3137306	G	A	intronic	*CSMD1*	.	.	.
8	3814735	A	T	intronic	*CSMD1*	.	.	.
8	3994025	C	A	intronic	*CSMD1*	.	.	.
8	3994026	C	A	intronic	*CSMD1*	.	.	.
8	4686141	C	G	intronic	*CSMD1*	.	.	.
8	4718409	A	G	intronic	*CSMD1*	.	.	.

8	3818170	G	C	intronic	*CSMD1*	.	.	.
8	3128497	C	T	intronic	*CSMD1*	.	.	.
8	3236725	T	C	intronic	*CSMD1*	.	.	.
8	3237040	A	C	intronic	*CSMD1*	.	.	.
8	3237252	T	C	intronic	*CSMD1*	.	.	.
8	3309111	C	T	intronic	*CSMD1*	.	.	.
8	3365228	A	G	intronic	*CSMD1*	.	.	.
8	3538885	G	A	intronic	*CSMD1*	.	.	.
8	3783022	C	T	intronic	*CSMD1*	.	.	.
8	3820465	C	T	intronic	*CSMD1*	.	.	.
8	4036539	C	G	intronic	*CSMD1*	.	.	.
8	4158404	A	G	intronic	*CSMD1*	.	.	.
8	5510396	G	A	intergenic	*CSMD1,LOC100287015*	.	.	.
8	5277359	G	A	intergenic	*CSMD1,LOC100287015*	.	.	.
8	5316653	GT	G	intergenic	*CSMD1,LOC100287015*	.	.	.
8	5562403	G	A	intergenic	*CSMD1,LOC100287015*	.	.	.
8	5739986	C	A	intergenic	*CSMD1,LOC100287015*	.	.	.
8	4995722	CATAT	C	intergenic	*CSMD1,LOC100287015*	.	.	.
8	5706155	A	G	intergenic	*CSMD1,LOC100287015*	.	.	.
8	5090317	T	A	intergenic	*CSMD1,LOC100287015*	.	.	.
8	5008391	C	T	intergenic	*CSMD1,LOC100287015*	.	.	.
8	5009448	T	C	intergenic	*CSMD1,LOC100287015*	.	.	.
8	5047282	G	C	intergenic	*CSMD1,LOC100287015*	.	.	.
8	5054632	G	A	intergenic	*CSMD1,LOC100287015*	.	.	.
8	5087551	T	C	intergenic	*CSMD1,LOC100287015*	.	.	.

8	5087553	A	G	intergenic	*CSMD1,LOC100287015*	.	.	.
8	5198976	C	T	intergenic	*CSMD1,LOC100287015*	.	.	.
8	5354859	A	T	intergenic	*CSMD1,LOC100287015*	.	.	.
8	5355040	A	T	intergenic	*CSMD1,LOC100287015*	.	.	.
8	5596533	C	T	intergenic	*CSMD1,LOC100287015*	.	.	.
8	5596938	A	G	intergenic	*CSMD1,LOC100287015*	.	.	.
8	5787455	G	C	intergenic	*CSMD1,LOC100287015*	.	.	.
8	5788005	C	T	intergenic	*CSMD1,LOC100287015*	.	.	.
8	5788056	C	A	intergenic	*CSMD1,LOC100287015*	.	.	.
8	5932929	T	A	intergenic	*CSMD1,LOC100287015*	.	.	.
8	5946756	C	G	intergenic	*CSMD1,LOC100287015*	.	.	.
8	6113098	A	G	intergenic	*CSMD1,LOC100287015*	.	.	.
8	6243607	A	G	intergenic	*CSMD1,LOC100287015*	.	.	.
8	6245955	C	T	intergenic	*CSMD1,LOC100287015*	.	.	.
1	34558338	G	C	intronic	*CSMD2*	.	.	.
8	113695425	C	T	intronic	*CSMD3*	.	.	.
8	113975871	C	A	intronic	*CSMD3*	.	.	.
8	114123470	T	A	intronic	*CSMD3*	.	.	.
8	114156824	AT	A	intronic	*CSMD3*	.	.	.
8	114186337	T	C	intronic	*CSMD3*	.	.	.
8	113897575	T	C	intronic	*CSMD3*	.	.	.
8	113909871	C	G	intronic	*CSMD3*	.	.	.
8	113477646	C	A	intronic	*CSMD3*	.	.	.
8	113741381	G	T	intronic	*CSMD3*	.	.	.

8	113270499	T	A	intronic	*CSMD3*	.	.	.
8	113547254	A	G	intronic	*CSMD3*	.	.	.
8	113608789	C	T	intronic	*CSMD3*	.	.	.
8	113708910	C	T	intronic	*CSMD3*	.	.	.
8	113726809	C	A	intronic	*CSMD3*	.	.	.
8	113726810	A	G	intronic	*CSMD3*	.	.	.
8	113728474	C	T	intronic	*CSMD3*	.	.	.
8	113886536	A	C	intronic	*CSMD3*	.	.	.
8	114101056	C	T	intronic	*CSMD3*	.	.	.
8	114281438	T	A	intronic	*CSMD3*	.	.	.
8	114308215	C	T	intronic	*CSMD3*	.	.	.
8	114580372	GT	G	intergenic	*CSMD3,TRPS1*	.	.	.
8	116385080	G	A	intergenic	*CSMD3,TRPS1*	.	.	.
8	114550186	C	A	intergenic	*CSMD3,TRPS1*	.	.	.
8	115205026	A	T	intergenic	*CSMD3,TRPS1*	.	.	.
8	115328897	C	T	intergenic	*CSMD3,TRPS1*	.	.	.
8	114533507	C	A	intergenic	*CSMD3,TRPS1*	.	.	.
8	114659332	C	T	intergenic	*CSMD3,TRPS1*	.	.	.
8	114797516	G	T	intergenic	*CSMD3,TRPS1*	.	.	.
8	115152643	G	GA	intergenic	*CSMD3,TRPS1*	.	.	.
8	115162539	A	T	intergenic	*CSMD3,TRPS1*	.	.	.
8	115245244	G	C	intergenic	*CSMD3,TRPS1*	.	.	.
8	114456603	G	GTTTC	intergenic	*CSMD3,TRPS1*	.	.	.
8	114985530	C	A	intergenic	*CSMD3,TRPS1*	.	.	.
8	115145787	C	A	intergenic	*CSMD3,TRPS1*	.	.	.
8	114812086	C	A	intergenic	*CSMD3,TRPS1*	.	.	.
8	115819436	C	A	intergenic	*CSMD3,TRPS1*	.	.	.
8	114497480	G	C	intergenic	*CSMD3,TRPS1*	.	.	.
8	114686968	C	A	intergenic	*CSMD3,TRPS1*	.	.	.
8	114723071	G	C	intergenic	*CSMD3,TRPS1*	.	.	.
8	115084199	G	T	intergenic	*CSMD3,TRPS1*	.	.	.
8	115288663	T	C	intergenic	*CSMD3,TRPS1*	.	.	.
8	115526832	G	T	intergenic	*CSMD3,TRPS1*	.	.	.
8	115526833	A	T	intergenic	*CSMD3,TRPS1*	.	.	.
8	115571365	A	G	intergenic	*CSMD3,TRPS1*	.	.	.
8	115619606	C	A	intergenic	*CSMD3,TRPS1*	.	.	.
8	116348899	C	G	intergenic	*CSMD3,TRPS1*	.	.	.
8	114885663	G	GT	intergenic	*CSMD3,TRPS1*	.	.	.

8	114872699	C	T	intergenic	*CSMD3,TRPS1*	.	.	.
8	114947291	G	GAAAAT	intergenic	*CSMD3,TRPS1*	.	.	.
8	114623172	T	A	intergenic	*CSMD3,TRPS1*	.	.	.
8	114677719	C	T	intergenic	*CSMD3,TRPS1*	.	.	.
8	114686009	C	T	intergenic	*CSMD3,TRPS1*	.	.	.
8	114844228	T	C	intergenic	*CSMD3,TRPS1*	.	.	.
8	115116599	T	C	intergenic	*CSMD3,TRPS1*	.	.	.
8	115205673	A	T	intergenic	*CSMD3,TRPS1*	.	.	.
8	115205674	T	C	intergenic	*CSMD3,TRPS1*	.	.	.
8	115644513	G	T	intergenic	*CSMD3,TRPS1*	.	.	.
8	115939644	G	A	intergenic	*CSMD3,TRPS1*	.	.	.
8	116023286	G	A	intergenic	*CSMD3,TRPS1*	.	.	.
8	116148908	T	C	intergenic	*CSMD3,TRPS1*	.	.	.
8	116384702	T	A	intergenic	*CSMD3,TRPS1*	.	.	.
4	70978941	C	T	intergenic	*CSN1S2AP,CSN1S2BP*	.	.	.
5	148938780	T	A	intergenic	*CSNK1A1,ARHGEF37*	.	.	.
13	37942058	G	T	intergenic	*CSNK1A1L,LINC01048*	.	.	.
13	38023490	A	G	intergenic	*CSNK1A1L,LINC01048*	.	.	.
13	37714912	G	A	intergenic	*CSNK1A1L,LINC01048*	.	.	.
13	37715140	C	T	intergenic	*CSNK1A1L,LINC01048*	.	.	.
13	37762723	T	C	intergenic	*CSNK1A1L,LINC01048*	.	.	.
15	64564318	T	C	intronic	*CSNK1G1*	.	.	.
5	123012106	G	T	intergenic	*CSNK1G3,LINC01170*	.	.	.
5	123013006	T	A	intergenic	*CSNK1G3,LINC01170*	.	.	.
5	123103339	C	T	intergenic	*CSNK1G3,LINC01170*	.	.	.
5	123104325	A	G	intergenic	*CSNK1G3,LINC01170*	.	.	.

5	123104327	T	C	intergenic	*CSNK1G3,LINC01170*	.	.	.
5	123268792	T	C	intergenic	*CSNK1G3,LINC01170*	.	.	.
5	123310642	C	T	intergenic	*CSNK1G3,LINC01170*	.	.	.
20	530037	T	C	intergenic	*CSNK2A1,TCF15*	.	.	.
8	68075400	A	G	intronic	*CSPP1*	.	.	.
12	51472719	A	C	intronic	*CSRNP2*	.	.	.
2	166380359	G	C	intronic	*CSRNP3*	.	.	.
2	166362854	A	G	intronic	*CSRNP3*	.	.	.
2	166388511	G	T	intronic	*CSRNP3*	.	.	.
20	23760503	G	T	intergenic	*CST1,CST2*	.	.	.
20	23443735	G	C	intergenic	*CST11,CST8*	.	.	.
20	23694824	T	C	intergenic	*CST4,CST1*	.	.	.
20	23676117	A	G	intergenic	*CST4,CST1*	.	.	.
20	23697339	T	C	intergenic	*CST4,CST1*	.	.	.
20	23878698	C	T	intergenic	*CST5,GGTLC1*	.	.	.
20	23960375	C	T	intergenic	*CST5,GGTLC1*	.	.	.
20	23606479	C	T	intergenic	*CST9,CST3*	.	.	.
20	23596956	A	G	intergenic	*CST9,CST3*	.	.	.
20	23597580	A	T	intergenic	*CST9,CST3*	.	.	.
20	23575022	C	A	intergenic	*CST9L,CST9*	.	.	.
20	23425921	A	T	downstream	*CSTL1*	.	.	.
20	23425923	A	C	downstream	*CSTL1*	.	.	.
X	120151518	G	T	intergenic	*CT47A1,GLUD2*	.	.	.
X	134390715	G	T	intergenic	*CT55,ZNF75D*	.	.	.
X	134395118	G	A	intergenic	*CT55,ZNF75D*	.	.	.
X	134395120	G	A	intergenic	*CT55,ZNF75D*	.	.	.
X	134413551	T	C	intergenic	*CT55,ZNF75D*	.	.	.
X	116862356	C	T	intergenic	*CT83,KLHL13*	.	.	.
X	116795974	A	AGACAGT	intergenic	*CT83,KLHL13*	.	.	.
X	115901201	C	A	intergenic	*CT83,KLHL13*	.	.	.
X	116451103	C	G	intergenic	*CT83,KLHL13*	.	.	.
X	117009193	T	C	intergenic	*CT83,KLHL13*	.	.	.
X	115645811	C	T	intergenic	*CT83,KLHL13*	.	.	.
X	115660724	T	G	intergenic	*CT83,KLHL13*	.	.	.
X	115815821	T	C	intergenic	*CT83,KLHL13*	.	.	.

X	115815822	G	A	intergenic	*CT83,KLHL13*	.	.	.
X	115850459	G	A	intergenic	*CT83,KLHL13*	.	.	.
X	115870478	C	T	intergenic	*CT83,KLHL13*	.	.	.
X	115907880	A	G	intergenic	*CT83,KLHL13*	.	.	.
X	115998652	C	T	intergenic	*CT83,KLHL13*	.	.	.
X	116129759	T	C	intergenic	*CT83,KLHL13*	.	.	.
X	116196340	A	G	intergenic	*CT83,KLHL13*	.	.	.
X	116196341	C	T	intergenic	*CT83,KLHL13*	.	.	.
X	116289737	C	G	intergenic	*CT83,KLHL13*	.	.	.
X	116301581	T	C	intergenic	*CT83,KLHL13*	.	.	.
X	116302232	A	G	intergenic	*CT83,KLHL13*	.	.	.
X	116389525	A	G	intergenic	*CT83,KLHL13*	.	.	.
X	116406870	A	G	intergenic	*CT83,KLHL13*	.	.	.
X	116441987	C	A	intergenic	*CT83,KLHL13*	.	.	.
X	116494181	A	G	intergenic	*CT83,KLHL13*	.	.	.
X	116550864	A	G	intergenic	*CT83,KLHL13*	.	.	.
X	116551569	C	T	intergenic	*CT83,KLHL13*	.	.	.
X	116567118	A	G	intergenic	*CT83,KLHL13*	.	.	.
X	116567717	G	A	intergenic	*CT83,KLHL13*	.	.	.
X	116579519	T	C	intergenic	*CT83,KLHL13*	.	.	.
X	116582852	A	G	intergenic	*CT83,KLHL13*	.	.	.
X	116582854	T	C	intergenic	*CT83,KLHL13*	.	.	.
X	116599905	A	T	intergenic	*CT83,KLHL13*	.	.	.
X	116639265	G	A	intergenic	*CT83,KLHL13*	.	.	.
X	116639899	C	A	intergenic	*CT83,KLHL13*	.	.	.
X	116729427	C	T	intergenic	*CT83,KLHL13*	.	.	.
X	116730143	G	C	intergenic	*CT83,KLHL13*	.	.	.
X	116768882	G	A	intergenic	*CT83,KLHL13*	.	.	.
X	116795034	G	C	intergenic	*CT83,KLHL13*	.	.	.
X	116803055	C	T	intergenic	*CT83,KLHL13*	.	.	.
X	116892367	A	C	intergenic	*CT83,KLHL13*	.	.	.
X	116945003	A	G	intergenic	*CT83,KLHL13*	.	.	.
X	116945004	A	G	intergenic	*CT83,KLHL13*	.	.	.
X	116956499	T	C	intergenic	*CT83,KLHL13*	.	.	.
X	153896722	A	C	intergenic	*CTAG2,GAB3*	.	.	.
X	153897071	G	A	intergenic	*CTAG2,GAB3*	.	.	.
18	20030053	C	T	intergenic	*CTAGE1,LOC101927571*	.	.	.

18	20201335	G	A	intergenic	*CTAGE1,LOC101927571*	.	.	.
18	20228738	C	T	intergenic	*CTAGE1,LOC101927571*	.	.	.
18	20299882	T	C	intergenic	*CTAGE1,LOC101927571*	.	.	.
14	39797367	G	A	intronic	*CTAGE5*	.	.	.
5	151363663	G	GT	ncRNA_intronic	*CTB-12O2.1*	.	.	.
5	151373527	A	G	ncRNA_intronic	*CTB-12O2.1*	.	.	.
5	151401774	A	T	ncRNA_intronic	*CTB-12O2.1*	.	.	.
5	151402071	T	A	ncRNA_intronic	*CTB-12O2.1*	.	.	.
5	151403339	C	A	ncRNA_intronic	*CTB-12O2.1*	.	.	.
5	151413260	T	A	ncRNA_intronic	*CTB-12O2.1*	.	.	.
5	151536690	A	G	ncRNA_intronic	*CTB-12O2.1*	.	.	.
5	151554863	T	C	ncRNA_intronic	*CTB-12O2.1*	.	.	.
5	151592844	T	C	ncRNA_intronic	*CTB-12O2.1*	.	.	.
5	166591719	G	A	intergenic	*CTB-7E3.1,TENM2*	.	.	.
5	166371324	C	T	intergenic	*CTB-7E3.1,TENM2*	.	.	.
4	1268163	G	T	intergenic	*CTBP1-AS2,MAEA*	.	.	.
4	1257286	ATTTT	A	intergenic	*CTBP1-AS2,MAEA*	.	.	.
10	127004042	A	G	intergenic	*CTBP2,TEX36-AS1*	.	.	.
10	126927233	G	C	intergenic	*CTBP2,TEX36-AS1*	.	.	.
10	126976430	A	T	intergenic	*CTBP2,TEX36-AS1*	.	.	.
10	127197473	A	T	intergenic	*CTBP2,TEX36-AS1*	.	.	.
10	127215373	T	C	intergenic	*CTBP2,TEX36-AS1*	.	.	.
5	60493921	A	AG	ncRNA_intronic	*CTC-436P18.1*	.	.	.
5	60540482	A	T	intergenic	*CTC-436P18.1,ZSWIM6*	.	.	.
20	56131726	G	A	intergenic	*CTCFL,PCK1*	.	.	.
5	98896925	T	G	ncRNA_intronic	*CTD-2151A2.1*	.	.	.
5	98917634	C	T	intergenic	*CTD-2151A2.1,LOC100133050*	.	.	.

5	99434151	A	T	intergenic	*CTD-2151A2.1,LOC100133050*	.	.	.
5	99602072	C	G	intergenic	*CTD-2151A2.1,LOC100133050*	.	.	.
5	99705693	T	C	intergenic	*CTD-2151A2.1,LOC100133050*	.	.	.
5	99675456	T	C	intergenic	*CTD-2151A2.1,LOC100133050*	.	.	.
5	99427928	C	T	intergenic	*CTD-2151A2.1,LOC100133050*	.	.	.
5	98969703	A	G	intergenic	*CTD-2151A2.1,LOC100133050*	.	.	.
5	99047025	G	A	intergenic	*CTD-2151A2.1,LOC100133050*	.	.	.
5	99094940	G	C	intergenic	*CTD-2151A2.1,LOC100133050*	.	.	.
5	99094942	G	C	intergenic	*CTD-2151A2.1,LOC100133050*	.	.	.
5	99096883	C	G	intergenic	*CTD-2151A2.1,LOC100133050*	.	.	.
5	99096885	A	G	intergenic	*CTD-2151A2.1,LOC100133050*	.	.	.
5	99097185	G	A	intergenic	*CTD-2151A2.1,LOC100133050*	.	.	.

5	99097383	A	AG	intergenic	*CTD-2151A2.1,LOC100133050*	.	.	.
5	99295044	A	T	intergenic	*CTD-2151A2.1,LOC100133050*	.	.	.
5	99297733	G	A	intergenic	*CTD-2151A2.1,LOC100133050*	.	.	.
5	99297735	C	T	intergenic	*CTD-2151A2.1,LOC100133050*	.	.	.
5	99407654	A	G	intergenic	*CTD-2151A2.1,LOC100133050*	.	.	.
5	99553862	C	CAAAAAAAA	intergenic	*CTD-2151A2.1,LOC100133050*	.	.	.
5	99618788	A	G	intergenic	*CTD-2151A2.1,LOC100133050*	.	.	.
5	99652494	A	C	intergenic	*CTD-2151A2.1,LOC100133050*	.	.	.
5	99653543	C	T	intergenic	*CTD-2151A2.1,LOC100133050*	.	.	.
5	169819378	G	T	ncRNA_intronic	*CTD-2270F17.1*	.	.	.
18	77441937	TGTGGTG	T	intronic	*CTDP1*	.	.	.
18	77620315	G	C	intergenic	*CTDP1,KCNG2*	.	.	.
15	44748676	G	A	intronic	*CTDSPL2*	.	.	.
15	44762132	G	A	intronic	*CTDSPL2*	.	.	.
6	132342034	C	T	intergenic	*CTGF,MIR548AJ1*	.	.	.
1	70975836	T	C	intergenic	*CTH,LOC101927244*	.	.	.
1	71076137	C	A	intergenic	*CTH,LOC101927244*	.	.	.

1	71120865	A	T	intergenic	*CTH,LOC101927244*	.	.	.
18	46381917	G	T	intronic	*CTIF*	.	.	.
18	46179638	G	C	intronic	*CTIF*	.	.	.
18	46248115	C	T	intronic	*CTIF*	.	.	.
2	80008277	G	T	intronic	*CTNNA2*	.	.	.
2	80652630	G	C	intronic	*CTNNA2*	.	.	.
2	80544258	A	C	intronic	*CTNNA2*	.	.	.
2	80629378	C	A	intronic	*CTNNA2*	.	.	.
2	80854442	G	T	intronic	*CTNNA2*	.	.	.
2	80443188	G	T	intronic	*CTNNA2*	.	.	.
2	80493249	C	A	intronic	*CTNNA2*	.	.	.
2	80603324	A	T	intronic	*CTNNA2*	.	.	.
2	80638893	T	A	intronic	*CTNNA2*	.	.	.
2	80820931	C	T	intronic	*CTNNA2*	.	.	.
2	80852382	G	A	intronic	*CTNNA2*	.	.	.
2	79862364	C	T	intronic	*CTNNA2*	.	.	.
2	79919725	T	C	intronic	*CTNNA2*	.	.	.
2	80106484	T	A	intronic	*CTNNA2*	.	.	.
2	80107676	C	G	intronic	*CTNNA2*	.	.	.
2	80107948	A	G	intronic	*CTNNA2*	.	.	.
2	80218240	C	G	intronic	*CTNNA2*	.	.	.
2	80322927	A	G	intronic	*CTNNA2*	.	.	.
2	80440163	C	T	intronic	*CTNNA2*	.	.	.
2	80441089	A	T	intronic	*CTNNA2*	.	.	.
2	80795691	CGTGTGTGTGT	C	intronic	*CTNNA2*	.	.	.
2	80823036	A	T	intronic	*CTNNA2*	.	.	.
2	81017775	T	A	intergenic	*CTNNA2,LOC100507201*	.	.	.
2	81652362	C	T	intergenic	*CTNNA2,LOC100507201*	.	.	.
2	81069668	G	C	intergenic	*CTNNA2,LOC100507201*	.	.	.
2	81394453	G	C	intergenic	*CTNNA2,LOC100507201*	.	.	.
2	81473500	C	A	intergenic	*CTNNA2,LOC100507201*	.	.	.

2	81065066	A	C	intergenic	*CTNNA2,LOC100507201*	.	.	.
2	81192955	G	C	intergenic	*CTNNA2,LOC100507201*	.	.	.
2	81195291	GTTTGTCT	G	intergenic	*CTNNA2,LOC100507201*	.	.	.
2	81379888	C	A	intergenic	*CTNNA2,LOC100507201*	.	.	.
2	81478520	T	A	intergenic	*CTNNA2,LOC100507201*	.	.	.
2	81436188	G	A	intergenic	*CTNNA2,LOC100507201*	.	.	.
2	80980389	A	T	intergenic	*CTNNA2,LOC100507201*	.	.	.
2	81074545	A	AT	intergenic	*CTNNA2,LOC100507201*	.	.	.
2	81252910	G	T	intergenic	*CTNNA2,LOC100507201*	.	.	.
2	81036983	T	G	intergenic	*CTNNA2,LOC100507201*	.	.	.
2	81044987	A	G	intergenic	*CTNNA2,LOC100507201*	.	.	.
2	81044988	A	C	intergenic	*CTNNA2,LOC100507201*	.	.	.
2	81048013	T	A	intergenic	*CTNNA2,LOC100507201*	.	.	.
2	81183723	T	A	intergenic	*CTNNA2,LOC100507201*	.	.	.
2	81479462	A	G	intergenic	*CTNNA2,LOC100507201*	.	.	.
2	81585499	A	G	intergenic	*CTNNA2,LOC100507201*	.	.	.
10	67735431	C	G	intronic	*CTNNA3*	.	.	.
10	67834335	T	G	intronic	*CTNNA3*	.	.	.
10	68204469	T	A	intronic	*CTNNA3*	.	.	.
10	67954348	T	C	intronic	*CTNNA3*	.	.	.
10	67801429	T	G	intronic	*CTNNA3*	.	.	.
10	67867326	A	C	intronic	*CTNNA3*	.	.	.

10	68476212	G	A	intronic	*CTNNA3*	.	.	.
10	68636913	G	A	intronic	*CTNNA3*	.	.	.
10	68986998	CA	C	intronic	*CTNNA3*	.	.	.
10	68893064	G	GATATATAT/	intronic	*CTNNA3*	.	.	.
10	67714100	C	T	intronic	*CTNNA3*	.	.	.
10	67715139	T	C	intronic	*CTNNA3*	.	.	.
10	67715140	C	T	intronic	*CTNNA3*	.	.	.
10	68093952	C	CTG	intronic	*CTNNA3*	.	.	.
10	68124994	A	G	intronic	*CTNNA3*	.	.	.
10	68144375	T	C	intronic	*CTNNA3*	.	.	.
10	68144516	C	A	intronic	*CTNNA3*	.	.	.
10	68144718	T	A	intronic	*CTNNA3*	.	.	.
10	68146305	C	T	intronic	*CTNNA3*	.	.	.
10	68187937	A	G	intronic	*CTNNA3*	.	.	.
10	68188341	G	C	intronic	*CTNNA3*	.	.	.
10	68188342	G	T	intronic	*CTNNA3*	.	.	.
10	68337256	G	T	intronic	*CTNNA3*	.	.	.
10	68337257	G	T	intronic	*CTNNA3*	.	.	.
10	68377379	T	A	intronic	*CTNNA3*	.	.	.
10	68633186	G	A	intronic	*CTNNA3*	.	.	.
10	68942232	G	T	intronic	*CTNNA3*	.	.	.
10	68994187	C	T	intronic	*CTNNA3*	.	.	.
10	69132100	G	A	intronic	*CTNNA3*	.	.	.
10	69167832	T	C	intronic	*CTNNA3*	.	.	.
10	69259778	C	T	intronic	*CTNNA3*	.	.	.
10	69269821	C	G	intronic	*CTNNA3*	.	.	.
10	69270339	C	G	intronic	*CTNNA3*	.	.	.
10	69271697	C	G	intronic	*CTNNA3*	.	.	.
10	69271698	C	A	intronic	*CTNNA3*	.	.	.
10	69289367	T	A	intronic	*CTNNA3*	.	.	.
10	69320538	G	A	intronic	*CTNNA3*	.	.	.
10	69323054	C	A	intronic	*CTNNA3*	.	.	.
10	69323055	A	G	intronic	*CTNNA3*	.	.	.
10	69336939	T	G	intronic	*CTNNA3*	.	.	.
10	69399012	C	A	intronic	*CTNNA3*	.	.	.
10	69399013	A	G	intronic	*CTNNA3*	.	.	.
10	69399230	C	T	intronic	*CTNNA3*	.	.	.
10	69400504	A	C	intronic	*CTNNA3*	.	.	.

10	69400789	C	T	intronic	*CTNNA3*	.	.	.
10	69401425	G	C	intronic	*CTNNA3*	.	.	.
10	69402877	T	C	intronic	*CTNNA3*	.	.	.
10	69403650	A	C	intronic	*CTNNA3*	.	.	.
10	69404054	T	A	intronic	*CTNNA3*	.	.	.
10	69432011	G	A	intronic	*CTNNA3*	.	.	.
10	69464094	G	A	intergenic	*CTNNA3,DNAJC12*	.	.	.
10	69464862	T	C	intergenic	*CTNNA3,DNAJC12*	.	.	.
10	69479109	T	G	intergenic	*CTNNA3,DNAJC12*	.	.	.
10	69479216	A	C	intergenic	*CTNNA3,DNAJC12*	.	.	.
10	69479217	C	A	intergenic	*CTNNA3,DNAJC12*	.	.	.
10	69498868	G	C	intergenic	*CTNNA3,DNAJC12*	.	.	.
10	69499757	C	T	intergenic	*CTNNA3,DNAJC12*	.	.	.
10	69501014	C	A	intergenic	*CTNNA3,DNAJC12*	.	.	.
10	69501193	A	C	intergenic	*CTNNA3,DNAJC12*	.	.	.
10	69511421	A	T	intergenic	*CTNNA3,DNAJC12*	.	.	.
9	111750562	T	C	intronic	*CTNNAL1*	.	.	.
1	9958594	A	AAAAT	intronic	*CTNNBIP1*	.	.	.
20	36346598	G	A	intronic	*CTNNBL1*	.	.	.
5	11084778	G	T	intronic	*CTNND2*	.	.	.
5	11662225	GTGTGTATATATATATACATATATGTATATATACACATATATATGTGTATATATGTGTATATATACATATA	G	intronic	*CTNND2*	.	.	.
5	11562160	CA	C	intronic	*CTNND2*	.	.	.
5	11157047	T	C	intronic	*CTNND2*	.	.	.
5	11317001	G	A	intronic	*CTNND2*	.	.	.
5	11667535	T	C	intronic	*CTNND2*	.	.	.
5	11667537	T	C	intronic	*CTNND2*	.	.	.
5	11667607	A	AT	intronic	*CTNND2*	.	.	.
5	11670953	C	T	intronic	*CTNND2*	.	.	.
5	11670955	C	G	intronic	*CTNND2*	.	.	.
5	11740914	C	G	intronic	*CTNND2*	.	.	.

5	11939224	AACAC	A	intergenic	*CTNND2,LINC01194*	.	.	.
5	12416660	A	G	intergenic	*CTNND2,LINC01194*	.	.	.
5	12561481	C	A	intergenic	*CTNND2,LINC01194*	.	.	.
5	12165254	G	C	intergenic	*CTNND2,LINC01194*	.	.	.
5	12061432	A	G	intergenic	*CTNND2,LINC01194*	.	.	.
5	12176924	C	A	intergenic	*CTNND2,LINC01194*	.	.	.
5	12447603	C	A	intergenic	*CTNND2,LINC01194*	.	.	.
5	11995482	T	C	intergenic	*CTNND2,LINC01194*	.	.	.
5	12058824	G	C	intergenic	*CTNND2,LINC01194*	.	.	.
5	12098615	C	T	intergenic	*CTNND2,LINC01194*	.	.	.
5	12158510	A	G	intergenic	*CTNND2,LINC01194*	.	.	.
5	12159064	G	A	intergenic	*CTNND2,LINC01194*	.	.	.
5	12326142	A	ATGCT	intergenic	*CTNND2,LINC01194*	.	.	.
5	12351416	C	T	intergenic	*CTNND2,LINC01194*	.	.	.
5	12351417	A	G	intergenic	*CTNND2,LINC01194*	.	.	.
5	12379904	A	T	intergenic	*CTNND2,LINC01194*	.	.	.
8	11819930	C	A	intergenic	*CTSB,DEFB136*	.	.	.
11	88025967	A	G	downstream	*CTSC*	.	.	.
11	88175466	G	A	intergenic	*CTSC,GRM5-AS1*	.	.	.
11	88194728	A	G	intergenic	*CTSC,GRM5-AS1*	.	.	.
14	25053635	G	A	intergenic	*CTSG,GZMH*	.	.	.
14	25068032	C	T	intergenic	*CTSG,GZMH*	.	.	.

15	79236649	C	T	intronic	*CTSH*	.	.	.
4	156936770	G	A	intergenic	*CTSO,PDGFC*	.	.	.
4	157401745	AACAC	A	intergenic	*CTSO,PDGFC*	.	.	.
4	157291949	T	C	intergenic	*CTSO,PDGFC*	.	.	.
4	157538389	AAACAAC	A	intergenic	*CTSO,PDGFC*	.	.	.
7	117425126	A	T	intronic	*CTTNBP2*	.	.	.
7	117791911	C	T	intergenic	*CTTNBP2,LSM8*	.	.	.
7	117563325	G	T	intergenic	*CTTNBP2,LSM8*	.	.	.
7	117563020	T	C	intergenic	*CTTNBP2,LSM8*	.	.	.
7	117563021	A	C	intergenic	*CTTNBP2,LSM8*	.	.	.
7	117563088	T	C	intergenic	*CTTNBP2,LSM8*	.	.	.
7	117606635	T	A	intergenic	*CTTNBP2,LSM8*	.	.	.
7	117606788	C	T	intergenic	*CTTNBP2,LSM8*	.	.	.
7	117607536	T	C	intergenic	*CTTNBP2,LSM8*	.	.	.
7	117608738	T	C	intergenic	*CTTNBP2,LSM8*	.	.	.
7	117766629	G	A	intergenic	*CTTNBP2,LSM8*	.	.	.
7	117767175	G	A	intergenic	*CTTNBP2,LSM8*	.	.	.
5	127065844	G	A	intergenic	*CTXN3,LINC01184*	.	.	.
5	127073690	G	A	intergenic	*CTXN3,LINC01184*	.	.	.
5	127085873	T	C	intergenic	*CTXN3,LINC01184*	.	.	.
5	127087969	T	A	intergenic	*CTXN3,LINC01184*	.	.	.
5	127215862	T	C	intergenic	*CTXN3,LINC01184*	.	.	.
2	225352701	T	C	intronic	*CUL3*	.	.	.
2	225353304	G	A	intronic	*CUL3*	.	.	.
2	225606339	G	T	intergenic	*CUL3,DOCK10*	.	.	.
2	225486646	C	T	intergenic	*CUL3,DOCK10*	.	.	.
10	101497299	G	GGAA	intronic	*CUTC*	.	.	.
7	101473790	T	C	intronic	*CUX1*	.	.	.
12	111511254	C	T	intronic	*CUX2*	.	.	.
2	181061632	C	A	intergenic	*CWC22,SCHLAP1*	.	.	.
2	181177511	T	C	intergenic	*CWC22,SCHLAP1*	.	.	.
2	180947702	G	T	intergenic	*CWC22,SCHLAP1*	.	.	.
2	181098317	A	T	intergenic	*CWC22,SCHLAP1*	.	.	.
2	181164450	A	G	intergenic	*CWC22,SCHLAP1*	.	.	.
2	181166122	A	G	intergenic	*CWC22,SCHLAP1*	.	.	.
2	181357483	C	T	intergenic	*CWC22,SCHLAP1*	.	.	.
2	181357784	G	T	intergenic	*CWC22,SCHLAP1*	.	.	.
2	181392667	T	C	intergenic	*CWC22,SCHLAP1*	.	.	.

2	181394817	C	A	intergenic	*CWC22,SCHLAP1*	.	.	.
2	181452581	T	C	intergenic	*CWC22,SCHLAP1*	.	.	.
2	181452582	G	A	intergenic	*CWC22,SCHLAP1*	.	.	.
2	181453053	G	A	intergenic	*CWC22,SCHLAP1*	.	.	.
5	64126367	T	C	intronic	*CWC27*	.	.	.
5	64231838	T	G	intronic	*CWC27*	.	.	.
5	64233253	T	C	intronic	*CWC27*	.	.	.
5	64233254	G	T	intronic	*CWC27*	.	.	.
5	64233413	G	A	intronic	*CWC27*	.	.	.
5	64233414	A	G	intronic	*CWC27*	.	.	.
5	64282969	T	TGTATA	intronic	*CWC27*	.	.	.
5	64304419	T	TG	intronic	*CWC27*	.	.	.
5	64304420	A	G	intronic	*CWC27*	.	.	.
11	107211814	C	T	intronic	*CWF19L2*	.	.	.
11	107313370	A	G	intronic	*CWF19L2*	.	.	.
11	107246435	C	G	intronic	*CWF19L2*	.	.	.
11	107349948	G	T	intergenic	*CWF19L2,ALKBH8*	.	.	.
11	107332155	C	T	intergenic	*CWF19L2,ALKBH8*	.	.	.
4	48995855	C	T	intronic	*CWH43*	.	.	.
4	48995857	T	C	intronic	*CWH43*	.	.	.
4	49010162	G	C	intronic	*CWH43*	.	.	.
4	49042091	T	C	intronic	*CWH43*	.	.	.
4	49153036	C	G	intergenic	*CWH43,NONE*	.	.	.
4	49626813	C	T	intergenic	*CWH43,NONE*	.	.	.
4	49117580	C	G	intergenic	*CWH43,NONE*	.	.	.
4	49121293	C	G	intergenic	*CWH43,NONE*	.	.	.
4	49200486	T	A	intergenic	*CWH43,NONE*	.	.	.
4	49154896	C	G	intergenic	*CWH43,NONE*	.	.	.
4	49290271	A	G	intergenic	*CWH43,NONE*	.	.	.
4	49324130	C	G	intergenic	*CWH43,NONE*	.	.	.
4	49631181	A	T	intergenic	*CWH43,NONE*	.	.	.
4	49657599	G	T	intergenic	*CWH43,NONE*	.	.	.

4	49601305	CCCTCACA TAGGATTC CAGAACAC TTCTGCTG GTATCTGA ATGTTTGTA	C	intergenic	*CWH43,NONE*	.	.	.
4	49615766	CAA	C	intergenic	*CWH43,NONE*	.	.	.
4	49208168	A	G	intergenic	*CWH43,NONE*	.	.	.
4	49297536	G	T	intergenic	*CWH43,NONE*	.	.	.
4	49516178	C	G	intergenic	*CWH43,NONE*	.	.	.
4	49114264	A	C	intergenic	*CWH43,NONE*	.	.	.
4	49157187	CCATTG	C	intergenic	*CWH43,NONE*	.	.	.
4	49235864	G	A	intergenic	*CWH43,NONE*	.	.	.
4	49526522	C	T	intergenic	*CWH43,NONE*	.	.	.
4	49639972	G	A	intergenic	*CWH43,NONE*	.	.	.
4	49653928	C	T	intergenic	*CWH43,NONE*	.	.	.
4	49069464	G	T	intergenic	*CWH43,NONE*	.	.	.
4	49073045	G	C	intergenic	*CWH43,NONE*	.	.	.
4	49073046	T	C	intergenic	*CWH43,NONE*	.	.	.
4	49073477	A	G	intergenic	*CWH43,NONE*	.	.	.
4	49081259	C	G	intergenic	*CWH43,NONE*	.	.	.
4	49118383	G	C	intergenic	*CWH43,NONE*	.	.	.
4	49146072	G	T	intergenic	*CWH43,NONE*	.	.	.
4	49146073	G	A	intergenic	*CWH43,NONE*	.	.	.
4	49158055	T	G	intergenic	*CWH43,NONE*	.	.	.
4	49171914	G	T	intergenic	*CWH43,NONE*	.	.	.
4	49171915	T	G	intergenic	*CWH43,NONE*	.	.	.
4	49172141	A	G	intergenic	*CWH43,NONE*	.	.	.
4	49207900	T	G	intergenic	*CWH43,NONE*	.	.	.
4	49318444	C	A	intergenic	*CWH43,NONE*	.	.	.
4	49647669	T	TGGAAA	intergenic	*CWH43,NONE*	.	.	.
4	49649759	A	C	intergenic	*CWH43,NONE*	.	.	.
10	45165900	GGAGAGA	G	intergenic	*CXCL12,TMEM72-AS1*	.	.	.
10	45170411	A	G	intergenic	*CXCL12,TMEM72-AS1*	.	.	.

10	44961967	T	G	intergenic	*CXCL12,TMEM72-AS1*	.	.	.
10	45199639	A	T	intergenic	*CXCL12,TMEM72-AS1*	.	.	.
10	44902996	G	A	intergenic	*CXCL12,TMEM72-AS1*	.	.	.
10	44903941	C	T	intergenic	*CXCL12,TMEM72-AS1*	.	.	.
10	44911036	T	G	intergenic	*CXCL12,TMEM72-AS1*	.	.	.
10	44944375	A	G	intergenic	*CXCL12,TMEM72-AS1*	.	.	.
10	44947556	G	A	intergenic	*CXCL12,TMEM72-AS1*	.	.	.
10	45019273	T	A	intergenic	*CXCL12,TMEM72-AS1*	.	.	.
10	45030265	T	C	intergenic	*CXCL12,TMEM72-AS1*	.	.	.
10	45037614	A	G	intergenic	*CXCL12,TMEM72-AS1*	.	.	.
4	78451764	T	C	intronic	*CXCL13*	.	.	.
4	78467993	T	C	intronic	*CXCL13*	.	.	.
4	78467994	G	A	intronic	*CXCL13*	.	.	.
4	78579267	G	A	intergenic	*CXCL13,CNOT6L*	.	.	.
4	78581033	G	A	intergenic	*CXCL13,CNOT6L*	.	.	.
4	78581628	T	C	intergenic	*CXCL13,CNOT6L*	.	.	.
4	78581629	G	A	intergenic	*CXCL13,CNOT6L*	.	.	.
4	74996662	T	C	intergenic	*CXCL2,MTHFD2L*	.	.	.
4	74660667	A	C	intergenic	*CXCL8,CXCL6*	.	.	.
4	74661549	A	G	intergenic	*CXCL8,CXCL6*	.	.	.
4	74700899	G	T	intergenic	*CXCL8,CXCL6*	.	.	.
2	136871530	G	A	downstream	*CXCR4*	.	.	.
2	137493005	T	G	intergenic	*CXCR4,THSD7B*	.	.	.
2	137005260	T	C	intergenic	*CXCR4,THSD7B*	.	.	.
2	137519521	C	A	intergenic	*CXCR4,THSD7B*	.	.	.
2	137157611	C	T	intergenic	*CXCR4,THSD7B*	.	.	.
2	137223166	A	C	intergenic	*CXCR4,THSD7B*	.	.	.
2	137021071	T	C	intergenic	*CXCR4,THSD7B*	.	.	.

2	137021945	G	C	intergenic	*CXCR4,THSD7B*	.	.	.
2	137358658	T	A	intergenic	*CXCR4,THSD7B*	.	.	.
2	137377847	T	G	intergenic	*CXCR4,THSD7B*	.	.	.
2	137439008	T	C	intergenic	*CXCR4,THSD7B*	.	.	.
2	137468279	A	G	intergenic	*CXCR4,THSD7B*	.	.	.
X	30667961	G	A	intergenic	*CXorf21,GK*	.	.	.
X	30627538	A	T	intergenic	*CXorf21,GK*	.	.	.
X	45350510	G	C	intergenic	*CXorf36,LINC01204*	.	.	.
X	45351638	T	C	intergenic	*CXorf36,LINC01204*	.	.	.
X	45295669	T	A	intergenic	*CXorf36,LINC01204*	.	.	.
X	45075952	C	T	intergenic	*CXorf36,LINC01204*	.	.	.
X	45140941	T	C	intergenic	*CXorf36,LINC01204*	.	.	.
X	45176920	C	A	intergenic	*CXorf36,LINC01204*	.	.	.
X	45176922	G	A	intergenic	*CXorf36,LINC01204*	.	.	.
X	45198300	T	C	intergenic	*CXorf36,LINC01204*	.	.	.
X	45277439	G	T	intergenic	*CXorf36,LINC01204*	.	.	.
X	45290657	T	A	intergenic	*CXorf36,LINC01204*	.	.	.
X	71026809	T	C	intergenic	*CXorf49,NHSL2*	.	.	.
X	71039804	A	G	intergenic	*CXorf49,NHSL2*	.	.	.
X	71054937	C	T	intergenic	*CXorf49,NHSL2*	.	.	.
X	71054938	A	T	intergenic	*CXorf49,NHSL2*	.	.	.
X	71058791	T	A	intergenic	*CXorf49,NHSL2*	.	.	.
X	71058966	A	G	intergenic	*CXorf49,NHSL2*	.	.	.
X	71059975	C	T	intergenic	*CXorf49,NHSL2*	.	.	.
X	71061108	G	A	intergenic	*CXorf49,NHSL2*	.	.	.
X	71120836	T	C	intergenic	*CXorf49,NHSL2*	.	.	.
X	145966224	G	T	intergenic	*CXorf51A,MIR513C*	.	.	.

X	146157399	G	T	intergenic	*CXorf51A,MIR513C*	.	.	.
X	146199372	C	CAAAAAAAA	intergenic	*CXorf51A,MIR513C*	.	.	.
X	146014169	G	T	intergenic	*CXorf51A,MIR513C*	.	.	.
X	146217999	A	C	intergenic	*CXorf51A,MIR513C*	.	.	.
X	146217527	C	T	intergenic	*CXorf51A,MIR513C*	.	.	.
X	146217528	A	G	intergenic	*CXorf51A,MIR513C*	.	.	.
X	146229382	G	A	intergenic	*CXorf51A,MIR513C*	.	.	.
X	105897082	A	C	intronic	*CXorf57*	.	.	.
X	105897685	G	A	intronic	*CXorf57*	.	.	.
X	139066583	G	C	intergenic	*CXorf66,LOC389895*	.	.	.
X	139080588	A	AT	intergenic	*CXorf66,LOC389895*	.	.	.
X	139080590	T	C	intergenic	*CXorf66,LOC389895*	.	.	.
X	139085932	G	A	intergenic	*CXorf66,LOC389895*	.	.	.
X	139098662	A	AGATCGGAA	intergenic	*CXorf66,LOC389895*	.	.	.
X	51195499	G	A	intergenic	*CXorf67,NUDT11*	.	.	.
X	51156945	G	A	intergenic	*CXorf67,NUDT11*	.	.	.
18	47893473	A	G	intergenic	*CXXC1,SKA1*	.	.	.
18	47894205	C	T	intergenic	*CXXC1,SKA1*	.	.	.
X	47582437	C	T	ncRNA_intronic	*CXXC1P1*	.	.	.
X	47667921	C	A	intergenic	*CXXC1P1,ZNF81*	.	.	.
X	47650429	C	T	intergenic	*CXXC1P1,ZNF81*	.	.	.
6	84652278	G	A	intronic	*CYB5R4*	.	.	.
6	84609374	A	T	intronic	*CYB5R4*	.	.	.
6	84609553	C	T	intronic	*CYB5R4*	.	.	.
6	84654251	A	G	intronic	*CYB5R4*	.	.	.
6	84655458	G	T	intronic	*CYB5R4*	.	.	.

X	37638309	A	T	upstream	*CYBB*	.	.	.
2	172498837	T	G	intergenic	*CYBRD1,DYNC1I2*	.	.	.
2	172501734	G	T	intergenic	*CYBRD1,DYNC1I2*	.	.	.
5	156853725	A	G	intergenic	*CYFIP2,NIPAL4*	.	.	.
5	156854363	C	A	intergenic	*CYFIP2,NIPAL4*	.	.	.
5	156855663	T	C	intergenic	*CYFIP2,NIPAL4*	.	.	.
5	156856404	C	G	intergenic	*CYFIP2,NIPAL4*	.	.	.
X	83164080	A	G	intergenic	*CYLC1,RPS6KA6*	.	.	.
X	83164929	C	G	intergenic	*CYLC1,RPS6KA6*	.	.	.
X	83212660	T	C	intergenic	*CYLC1,RPS6KA6*	.	.	.
X	83304133	T	G	intergenic	*CYLC1,RPS6KA6*	.	.	.
16	50908642	ATGTG	A	intergenic	*CYLD,LOC101927334*	.	.	.
2	38363746	T	C	ncRNA_intronic	*CYP1B1-AS1*	.	.	.
2	38435901	T	C	intergenic	*CYP1B1-AS1,ATL2*	.	.	.
2	38330404	C	A	intergenic	*CYP1B1,CYP1B1-AS1*	.	.	.
10	94944877	C	T	intergenic	*CYP26A1,MYOF*	.	.	.
10	95046391	GGTGT	G	intergenic	*CYP26A1,MYOF*	.	.	.
2	72404721	G	C	intergenic	*CYP26B1,EXOC6B*	.	.	.
2	127968300	A	T	intergenic	*CYP27C1,ERCC3*	.	.	.
2	127970588	T	A	intergenic	*CYP27C1,ERCC3*	.	.	.
2	127986470	G	A	intergenic	*CYP27C1,ERCC3*	.	.	.
19	41609224	G	A	intergenic	*CYP2A13,CYP2F1*	.	.	.
19	41372485	A	G	intergenic	*CYP2A6,CYP2A7*	.	.	.
19	41394705	C	T	intergenic	*CYP2A7,CYP2G1P*	.	.	.
19	41541755	T	A	intergenic	*CYP2B6,CYP2A13*	.	.	.
19	41541757	A	C	intergenic	*CYP2B6,CYP2A13*	.	.	.
19	41430241	C	T	ncRNA_exonic	*CYP2B7P*	.	.	.
19	41431541	G	A	ncRNA_intronic	*CYP2B7P*	.	.	.
10	96451900	A	G	intronic	*CYP2C18*	.	.	.
10	96457567	G	A	intronic	*CYP2C18*	.	.	.
10	96457568	G	A	intronic	*CYP2C18*	.	.	.
10	96473679	G	A	intronic	*CYP2C18*	.	.	.

10	96503548	T	C	intergenic	*CYP2C18,CYP2C19*	.	.	.
10	96514971	C	A	intergenic	*CYP2C18,CYP2C19*	.	.	.
10	96515730	T	A	intergenic	*CYP2C18,CYP2C19*	.	.	.
10	96529886	G	T	intronic	*CYP2C19*	.	.	.
10	96580484	G	A	intronic	*CYP2C19*	.	.	.
10	96559281	G	A	intronic	*CYP2C19*	.	.	.
10	96571087	A	T	intronic	*CYP2C19*	.	.	.
10	96586291	T	C	intronic	*CYP2C19*	.	.	.
10	96657653	A	T	intergenic	*CYP2C19,CYP2C9*	.	.	.
10	96655025	G	A	intergenic	*CYP2C19,CYP2C9*	.	.	.
10	96670449	A	C	intergenic	*CYP2C19,CYP2C9*	.	.	.
10	96670867	G	A	intergenic	*CYP2C19,CYP2C9*	.	.	.
10	96820889	T	G	intronic	*CYP2C8*	.	.	.
10	96821413	C	T	intronic	*CYP2C8*	.	.	.
10	96822123	A	G	intronic	*CYP2C8*	.	.	.
10	96843393	T	C	intergenic	*CYP2C8,ACSM6*	.	.	.
10	96866717	C	T	intergenic	*CYP2C8,ACSM6*	.	.	.
10	96868782	C	G	intergenic	*CYP2C8,ACSM6*	.	.	.
10	96868784	A	C	intergenic	*CYP2C8,ACSM6*	.	.	.
10	96869446	C	T	intergenic	*CYP2C8,ACSM6*	.	.	.
10	96898115	A	T	intergenic	*CYP2C8,ACSM6*	.	.	.
10	96908990	G	C	intergenic	*CYP2C8,ACSM6*	.	.	.
10	96913742	T	A	intergenic	*CYP2C8,ACSM6*	.	.	.
10	96916885	T	G	intergenic	*CYP2C8,ACSM6*	.	.	.
10	96921919	T	C	intergenic	*CYP2C8,ACSM6*	.	.	.
10	96934708	T	A	intergenic	*CYP2C8,ACSM6*	.	.	.
10	96737862	C	T	intronic	*CYP2C9*	.	.	.
10	96705888	G	A	intronic	*CYP2C9*	.	.	.
10	96705889	T	G	intronic	*CYP2C9*	.	.	.
10	96710241	T	A	intronic	*CYP2C9*	.	.	.
10	96715433	A	G	intronic	*CYP2C9*	.	.	.
10	96717796	T	A	intronic	*CYP2C9*	.	.	.
10	96717798	T	A	intronic	*CYP2C9*	.	.	.
10	96725800	A	C	intronic	*CYP2C9*	.	.	.
10	135361316	G	A	intergenic	*CYP2E1,SYCE1*	.	.	.

19	41657690	T	C	intergenic	*CYP2F1,CYP2S1*	.	.	.
1	60361429	G	A	intronic	*CYP2J2*	.	.	.
1	60423353	GACAC	G	intergenic	*CYP2J2,C1orf87*	.	.	.
1	60440729	ATT	A	intergenic	*CYP2J2,C1orf87*	.	.	.
11	14905925	T	TAG	intronic	*CYP2R1*	.	.	.
11	14905950	T	A	intronic	*CYP2R1*	.	.	.
11	14916541	A	G	intergenic	*CYP2R1,CALCA*	.	.	.
11	14933624	C	A	intergenic	*CYP2R1,CALCA*	.	.	.
7	99252704	C	A	intronic	*CYP3A5*	.	.	.
7	99287059	C	G	intronic	*CYP3A7-CYP3A51P*	.	.	.
7	99288079	G	T	intronic	*CYP3A7-CYP3A51P*	.	.	.
7	99342452	G	A	intergenic	*CYP3A7-CYP3A51P,CYP3A4*	.	.	.
14	100169899	C	A	intronic	*CYP46A1*	.	.	.
1	47428675	A	G	intergenic	*CYP4A11,CYP4X1*	.	.	.
1	47428822	C	T	intergenic	*CYP4A11,CYP4X1*	.	.	.
1	47428823	A	G	intergenic	*CYP4A11,CYP4X1*	.	.	.
1	47453353	A	T	intergenic	*CYP4A11,CYP4X1*	.	.	.
1	47453385	C	A	intergenic	*CYP4A11,CYP4X1*	.	.	.
1	47464480	T	C	intergenic	*CYP4A11,CYP4X1*	.	.	.
1	47464482	T	C	intergenic	*CYP4A11,CYP4X1*	.	.	.
1	47622121	C	G	intergenic	*CYP4A22,LINC00853*	.	.	.
1	47633854	T	C	intergenic	*CYP4A22,LINC00853*	.	.	.
1	47292240	C	T	intergenic	*CYP4B1,CYP4Z2P*	.	.	.
1	47292241	A	G	intergenic	*CYP4B1,CYP4Z2P*	.	.	.
19	16041953	T	G	intronic	*CYP4F11*	.	.	.
19	15835206	TTATATA	T	intergenic	*CYP4F12,OR10H2*	.	.	.
19	15998473	C	A	intronic	*CYP4F2*	.	.	.
19	16010158	C	T	intergenic	*CYP4F2,CYP4F11*	.	.	.
19	15707491	C	T	intergenic	*CYP4F22,CYP4F8*	.	.	.
19	15707996	C	A	intergenic	*CYP4F22,CYP4F8*	.	.	.
19	15898945	CTGTG	C	intergenic	*CYP4F24P,OR10H5*	.	.	.

19	15760787	C	G	exonic	*CYP4F3*	nonsynonymous_SNV	.	.
2	131474674	C	G	intergenic	*CYP4F30P,GPR148*	.	.	.
18	14344785	G	C	intergenic	*CYP4F35P,CXADRP3*	.	.	.
18	14425719	T	G	intergenic	*CYP4F35P,CXADRP3*	.	.	.
18	14426105	C	A	intergenic	*CYP4F35P,CXADRP3*	.	.	.
19	15726980	T	TCC	intronic	*CYP4F8*	.	.	.
1	47516644	T	C	downstream	*CYP4X1*	.	.	.
1	47552746	T	C	intronic	*CYP4Z1*	.	.	.
1	47592278	T	G	intergenic	*CYP4Z1,CYP4A22*	.	.	.
1	47313933	A	C	ncRNA_intronic	*CYP4Z2P*	.	.	.
1	47382816	G	T	intergenic	*CYP4Z2P,CYP4A11*	.	.	.
1	47385971	C	T	intergenic	*CYP4Z2P,CYP4A11*	.	.	.
1	47385972	A	G	intergenic	*CYP4Z2P,CYP4A11*	.	.	.
7	91814874	A	T	intergenic	*CYP51A1-AS1,KRIT1*	.	.	.
8	59463509	T	C	intergenic	*CYP7A1,SDCBP*	.	.	.
8	65660550	T	C	intronic	*CYP7B1*	.	.	.
8	66020213	A	G	intergenic	*CYP7B1,LINC00251*	.	.	.
8	65848491	C	T	intergenic	*CYP7B1,LINC00251*	.	.	.
8	65965007	C	T	intergenic	*CYP7B1,LINC00251*	.	.	.
8	65823491	A	G	intergenic	*CYP7B1,LINC00251*	.	.	.
8	65824167	A	C	intergenic	*CYP7B1,LINC00251*	.	.	.
8	65824863	T	C	intergenic	*CYP7B1,LINC00251*	.	.	.

8	65825061	T	C	intergenic	*CYP7B1,LINC00251*	.	.	.
8	65840783	T	C	intergenic	*CYP7B1,LINC00251*	.	.	.
8	65840784	G	A	intergenic	*CYP7B1,LINC00251*	.	.	.
1	86112825	G	A	intergenic	*CYR61,ZNHIT6*	.	.	.
X	77530099	A	G	intronic	*CYSLTR1*	.	.	.
X	77699486	T	C	intergenic	*CYSLTR1,ZCCHC5*	.	.	.
X	77907180	T	C	intergenic	*CYSLTR1,ZCCHC5*	.	.	.
X	77663774	G	T	intergenic	*CYSLTR1,ZCCHC5*	.	.	.
X	77601809	T	A	intergenic	*CYSLTR1,ZCCHC5*	.	.	.
X	77601810	T	C	intergenic	*CYSLTR1,ZCCHC5*	.	.	.
X	77745986	T	G	intergenic	*CYSLTR1,ZCCHC5*	.	.	.
X	77745987	T	C	intergenic	*CYSLTR1,ZCCHC5*	.	.	.
X	77901076	CGTGTGTGTGT	C	intergenic	*CYSLTR1,ZCCHC5*	.	.	.
13	49244379	A	T	intronic	*CYSLTR2*	.	.	.
13	49244381	A	G	intronic	*CYSLTR2*	.	.	.
13	49246641	T	C	intronic	*CYSLTR2*	.	.	.
13	49274022	C	A	intronic	*CYSLTR2*	.	.	.
13	49274230	A	G	intronic	*CYSLTR2*	.	.	.
13	49274898	C	T	intronic	*CYSLTR2*	.	.	.
13	49330603	T	C	intergenic	*CYSLTR2,FNDC3A*	.	.	.
5	139579780	T	C	intronic	*CYSTM1*	.	.	.
17	76774414	T	A	intronic	*CYTH1*	.	.	.
17	76708564	G	A	intronic	*CYTH1*	.	.	.
7	6210681	C	T	intronic	*CYTH3*	.	.	.
7	6301300	G	A	intronic	*CYTH3*	.	.	.
7	6326674	C	T	intergenic	*CYTH3,FAM220A*	.	.	.
2	158278331	T	C	intronic	*CYTIP*	.	.	.
2	158374584	G	A	intergenic	*CYTIP,ACVR1C*	.	.	.
2	158376439	G	A	intergenic	*CYTIP,ACVR1C*	.	.	.
21	28127842	A	G	intergenic	*CYYR1,ADAMTS1*	.	.	.
21	28187169	C	G	intergenic	*CYYR1,ADAMTS1*	.	.	.
21	28038744	C	T	intergenic	*CYYR1,ADAMTS1*	.	.	.
21	25661492	C	T	intergenic	*D21S2088E,LOC101927869*	.	.	.

21	24836534	A	C	intergenic	*D21S2088E,LOC101927869*	.	.	.
21	25137698	A	G	intergenic	*D21S2088E,LOC101927869*	.	.	.
21	25244189	C	T	intergenic	*D21S2088E,LOC101927869*	.	.	.
21	25450057	C	T	intergenic	*D21S2088E,LOC101927869*	.	.	.
21	25480563	C	A	intergenic	*D21S2088E,LOC101927869*	.	.	.
21	25520524	G	T	intergenic	*D21S2088E,LOC101927869*	.	.	.
21	25443462	GTCTC	G	intergenic	*D21S2088E,LOC101927869*	.	.	.
21	25637598	T	A	intergenic	*D21S2088E,LOC101927869*	.	.	.
21	24829560	C	CAAAAAAAA	intergenic	*D21S2088E,LOC101927869*	.	.	.
21	24885504	G	C	intergenic	*D21S2088E,LOC101927869*	.	.	.
21	25021294	A	G	intergenic	*D21S2088E,LOC101927869*	.	.	.
21	25260016	A	T	intergenic	*D21S2088E,LOC101927869*	.	.	.
21	25089718	A	T	intergenic	*D21S2088E,LOC101927869*	.	.	.
21	25521970	T	C	intergenic	*D21S2088E,LOC101927869*	.	.	.
21	25628790	T	C	intergenic	*D21S2088E,LOC101927869*	.	.	.
21	25633906	C	T	intergenic	*D21S2088E,LOC101927869*	.	.	.
21	25633907	C	T	intergenic	*D21S2088E,LOC101927869*	.	.	.
14	59878667	A	T	intergenic	*DAAM1,GPR135*	.	.	.
14	59880566	C	T	intergenic	*DAAM1,GPR135*	.	.	.
1	57608271	TCACACACA	T	intronic	*DAB1*	.	.	.

1	58152755	A	T	intronic	*DAB1*	.	.	.
1	58491937	A	G	intronic	*DAB1*	.	.	.
1	58602482	C	T	intronic	*DAB1*	.	.	.
1	58033502	G	C	intronic	*DAB1*	.	.	.
1	58273869	GCACACA	G	intronic	*DAB1*	.	.	.
1	58443635	G	C	intronic	*DAB1*	.	.	.
1	58517832	A	G	intronic	*DAB1*	.	.	.
1	58919757	C	A	intergenic	*DAB1,OMA1*	.	.	.
5	39380650	A	G	intronic	*DAB2*	.	.	.
5	39387551	A	G	intronic	*DAB2*	.	.	.
5	39414497	G	A	intronic	*DAB2*	.	.	.
5	39449139	A	T	intergenic	*DAB2,LOC101926940*	.	.	.
9	124558958	C	T	intergenic	*DAB2IP,TTLL11*	.	.	.
13	72241291	G	A	intronic	*DACH1*	.	.	.
13	72100838	GTATA	G	intronic	*DACH1*	.	.	.
13	72016889	A	G	intronic	*DACH1*	.	.	.
13	72060095	A	G	intronic	*DACH1*	.	.	.
13	72166626	C	CAAAAAAAA	intronic	*DACH1*	.	.	.
13	72284584	TTGTGTGTGTGTGTG	T	intronic	*DACH1*	.	.	.
13	73003457	A	AAT	intergenic	*DACH1,MZT1*	.	.	.
13	72946479	A	G	intergenic	*DACH1,MZT1*	.	.	.
13	72979111	G	A	intergenic	*DACH1,MZT1*	.	.	.
13	72526181	C	G	intergenic	*DACH1,MZT1*	.	.	.
13	72526913	C	T	intergenic	*DACH1,MZT1*	.	.	.
13	72802002	C	T	intergenic	*DACH1,MZT1*	.	.	.
13	72932411	C	A	intergenic	*DACH1,MZT1*	.	.	.
13	73279339	T	C	intergenic	*DACH1,MZT1*	.	.	.
X	85848213	A	C	intronic	*DACH2*	.	.	.
X	85956551	A	T	intronic	*DACH2*	.	.	.
X	86084962	C	T	intronic	*DACH2*	.	.	.
X	85437931	T	C	intronic	*DACH2*	.	.	.
X	85438712	G	T	intronic	*DACH2*	.	.	.
X	85536571	T	G	intronic	*DACH2*	.	.	.
X	85536573	C	A	intronic	*DACH2*	.	.	.
X	85538451	G	A	intronic	*DACH2*	.	.	.
X	85589461	A	G	intronic	*DACH2*	.	.	.

X	85589583	T	C	intronic	*DACH2*	.	.	.
X	85592693	T	A	intronic	*DACH2*	.	.	.
X	85650419	C	T	intronic	*DACH2*	.	.	.
X	85707700	C	A	intronic	*DACH2*	.	.	.
X	85718526	C	T	intronic	*DACH2*	.	.	.
X	85846182	A	T	intronic	*DACH2*	.	.	.
X	85862638	T	G	intronic	*DACH2*	.	.	.
X	85901970	T	C	intronic	*DACH2*	.	.	.
X	85973888	A	G	intronic	*DACH2*	.	.	.
X	85973889	C	T	intronic	*DACH2*	.	.	.
X	86622811	G	A	intergenic	*DACH2,KLHL4*	.	.	.
X	86662301	G	C	intergenic	*DACH2,KLHL4*	.	.	.
X	86662996	CTGCGTA	C	intergenic	*DACH2,KLHL4*	.	.	.
X	86518119	G	A	intergenic	*DACH2,KLHL4*	.	.	.
X	86193710	A	G	intergenic	*DACH2,KLHL4*	.	.	.
X	86150436	G	A	intergenic	*DACH2,KLHL4*	.	.	.
X	86153784	G	C	intergenic	*DACH2,KLHL4*	.	.	.
X	86153785	T	C	intergenic	*DACH2,KLHL4*	.	.	.
X	86154943	A	G	intergenic	*DACH2,KLHL4*	.	.	.
X	86226925	T	G	intergenic	*DACH2,KLHL4*	.	.	.
X	86452433	C	T	intergenic	*DACH2,KLHL4*	.	.	.
X	86511450	A	G	intergenic	*DACH2,KLHL4*	.	.	.
X	86511965	T	A	intergenic	*DACH2,KLHL4*	.	.	.
X	86511966	T	C	intergenic	*DACH2,KLHL4*	.	.	.
X	86601489	T	C	intergenic	*DACH2,KLHL4*	.	.	.
X	86669435	A	G	intergenic	*DACH2,KLHL4*	.	.	.
X	86700332	T	G	intergenic	*DACH2,KLHL4*	.	.	.
X	86700979	C	T	intergenic	*DACH2,KLHL4*	.	.	.
X	86701439	A	G	intergenic	*DACH2,KLHL4*	.	.	.
X	86721455	G	A	intergenic	*DACH2,KLHL4*	.	.	.
14	59188203	G	T	intergenic	*DACT1,LINC01500*	.	.	.
14	59268838	GT	G	intergenic	*DACT1,LINC01500*	.	.	.
6	168802867	G	A	intergenic	*DACT2,SMOC2*	.	.	.
X	115032399	A	G	ncRNA_intronic	*DANT2*	.	.	.
X	115294628	A	G	intergenic	*DANT2,AGTR2*	.	.	.
X	115164449	G	A	intergenic	*DANT2,AGTR2*	.	.	.
X	115165763	T	G	intergenic	*DANT2,AGTR2*	.	.	.
X	115184750	G	T	intergenic	*DANT2,AGTR2*	.	.	.

X	115199056	T	A	intergenic	*DANT2,AGTR2*	.	.	.
X	115254725	G	A	intergenic	*DANT2,AGTR2*	.	.	.
X	115256644	G	T	intergenic	*DANT2,AGTR2*	.	.	.
X	115257318	G	C	intergenic	*DANT2,AGTR2*	.	.	.
13	106243595	G	GC	intergenic	*DAOA-AS1,LINC00343*	.	.	.
13	106243597	T	C	intergenic	*DAOA-AS1,LINC00343*	.	.	.
13	106343175	C	A	intergenic	*DAOA-AS1,LINC00343*	.	.	.
13	106357003	A	AATTT	intergenic	*DAOA-AS1,LINC00343*	.	.	.
5	10714218	T	C	intronic	*DAP*	.	.	.
5	10737232	C	T	intronic	*DAP*	.	.	.
5	10865321	G	T	intergenic	*DAP,CTNND2*	.	.	.
5	10814801	C	T	intergenic	*DAP,CTNND2*	.	.	.
5	10814803	G	C	intergenic	*DAP,CTNND2*	.	.	.
2	159803839	A	G	intergenic	*DAPL1,TANC1*	.	.	.
2	159732075	C	A	intergenic	*DAPL1,TANC1*	.	.	.
2	159697969	T	G	intergenic	*DAPL1,TANC1*	.	.	.
2	136666810	C	T	intronic	*DARS*	.	.	.
2	136695469	G	C	intronic	*DARS*	.	.	.
2	136696493	A	C	intronic	*DARS*	.	.	.
2	136696892	C	G	intronic	*DARS*	.	.	.
2	136697733	T	C	intronic	*DARS*	.	.	.
2	136699462	G	A	intronic	*DARS*	.	.	.
1	173819692	C	T	intronic	*DARS2*	.	.	.
2	228830167	C	T	intergenic	*DAW1,SPHKAP*	.	.	.
2	228831137	A	G	intergenic	*DAW1,SPHKAP*	.	.	.
2	228831353	A	G	intergenic	*DAW1,SPHKAP*	.	.	.
3	16846152	T	C	intergenic	*DAZL,PLCL2*	.	.	.
3	16722828	G	A	intergenic	*DAZL,PLCL2*	.	.	.
4	191038318	C	T	intergenic	*DBET,NONE*	.	.	.
1	100668867	C	A	intronic	*DBT*	.	.	.
1	100669852	C	T	intronic	*DBT*	.	.	.
11	20216035	T	C	intergenic	*DBX1,HTATIP2*	.	.	.
11	20254710	T	C	intergenic	*DBX1,HTATIP2*	.	.	.
11	20347009	A	G	intergenic	*DBX1,HTATIP2*	.	.	.

11	20369145	C	T	intergenic	*DBX1,HTATIP2*	.	.	.
11	20369305	T	G	intergenic	*DBX1,HTATIP2*	.	.	.
9	34146225	TTGCAGTGAGCTGAGATCATGCCACTGCACTCCAGCCTGGCAACAGAGGGACTCTCCATCTCAAAAAAACTAAATAAATAAAATAAAAAATAAAACCTGCTTAGAAAACCAAACTCTCTCTCTCTCTTTTTTTTTTTTTTTTTTTTTTGAGGCAGGGTCTCCCTCTGTTGCCCAGGCTGGAG	T	intergenic	*DCAF12,UBAP1*	.	.	.
X	125697961	G	A	intergenic	*DCAF12L1,PRR32*	.	.	.
X	125774777	C	G	intergenic	*DCAF12L1,PRR32*	.	.	.
X	125822143	C	A	intergenic	*DCAF12L1,PRR32*	.	.	.
X	125635324	G	T	intergenic	*DCAF12L2,DCAF12L1*	.	.	.
X	125534757	A	C	intergenic	*DCAF12L2,DCAF12L1*	.	.	.
X	125670324	T	G	intergenic	*DCAF12L2,DCAF12L1*	.	.	.
X	125604543	A	G	intergenic	*DCAF12L2,DCAF12L1*	.	.	.
X	125491063	A	C	intergenic	*DCAF12L2,DCAF12L1*	.	.	.

X	125497191	C	T	intergenic	*DCAF12L2,DCAF12L1*	.	.	.
X	125497369	T	A	intergenic	*DCAF12L2,DCAF12L1*	.	.	.
X	125497759	C	T	intergenic	*DCAF12L2,DCAF12L1*	.	.	.
X	125567477	ATAAATAAATAAC	A	intergenic	*DCAF12L2,DCAF12L1*	.	.	.
X	125682108	C	T	intergenic	*DCAF12L2,DCAF12L1*	.	.	.
2	172312229	A	G	intronic	*DCAF17*	.	.	.
2	172312527	G	T	intronic	*DCAF17*	.	.	.
2	172348954	C	T	intergenic	*DCAF17,CYBRD1*	.	.	.
2	172348955	A	G	intergenic	*DCAF17,CYBRD1*	.	.	.
8	89004937	G	T	intergenic	*DCAF4L2,MMP16*	.	.	.
8	89040429	G	A	intergenic	*DCAF4L2,MMP16*	.	.	.
8	88959447	G	C	intergenic	*DCAF4L2,MMP16*	.	.	.
8	88892782	T	G	intergenic	*DCAF4L2,MMP16*	.	.	.
X	28459204	A	G	intergenic	*DCAF8L1,MIR6134*	.	.	.
X	28414256	A	G	intergenic	*DCAF8L1,MIR6134*	.	.	.
X	28087918	G	GTTTA	intergenic	*DCAF8L1,MIR6134*	.	.	.
X	28431702	CT	C	intergenic	*DCAF8L1,MIR6134*	.	.	.
X	28097609	A	G	intergenic	*DCAF8L1,MIR6134*	.	.	.
X	28480753	T	C	intergenic	*DCAF8L1,MIR6134*	.	.	.
X	28065947	G	A	intergenic	*DCAF8L1,MIR6134*	.	.	.
X	28147295	G	A	intergenic	*DCAF8L1,MIR6134*	.	.	.
X	28147296	T	A	intergenic	*DCAF8L1,MIR6134*	.	.	.
X	28147565	A	G	intergenic	*DCAF8L1,MIR6134*	.	.	.
X	28380670	G	C	intergenic	*DCAF8L1,MIR6134*	.	.	.
X	28381403	G	C	intergenic	*DCAF8L1,MIR6134*	.	.	.
X	28406123	C	T	intergenic	*DCAF8L1,MIR6134*	.	.	.
X	28407193	T	C	intergenic	*DCAF8L1,MIR6134*	.	.	.
X	28472422	G	A	intergenic	*DCAF8L1,MIR6134*	.	.	.
X	28476158	T	G	intergenic	*DCAF8L1,MIR6134*	.	.	.
X	28491503	C	T	intergenic	*DCAF8L1,MIR6134*	.	.	.
X	28500285	G	T	intergenic	*DCAF8L1,MIR6134*	.	.	.
X	28500286	C	T	intergenic	*DCAF8L1,MIR6134*	.	.	.

X	27779780	C	A	intergenic	*DCAF8L2,MAGEB10*	.	.	.
X	27782255	C	G	intergenic	*DCAF8L2,MAGEB10*	.	.	.
3	98580721	G	C	intronic	*DCBLD2*	.	.	.
3	98570746	G	A	intronic	*DCBLD2*	.	.	.
3	99107019	T	C	intergenic	*DCBLD2,MIR548G*	.	.	.
3	99069166	GTATA	G	intergenic	*DCBLD2,MIR548G*	.	.	.
3	99016435	G	A	intergenic	*DCBLD2,MIR548G*	.	.	.
3	99022255	A	G	intergenic	*DCBLD2,MIR548G*	.	.	.
18	50936617	C	A	intronic	*DCC*	.	.	.
18	50305316	T	C	intronic	*DCC*	.	.	.
18	49917236	A	G	intronic	*DCC*	.	.	.
18	49926748	T	C	intronic	*DCC*	.	.	.
18	50305871	A	G	intronic	*DCC*	.	.	.
18	50305872	T	C	intronic	*DCC*	.	.	.
18	50393109	G	T	intronic	*DCC*	.	.	.
18	50567816	C	T	intronic	*DCC*	.	.	.
18	50567817	A	G	intronic	*DCC*	.	.	.
18	50567842	T	G	intronic	*DCC*	.	.	.
18	50567843	T	G	intronic	*DCC*	.	.	.
18	50622494	A	G	intronic	*DCC*	.	.	.
18	50701431	G	A	intronic	*DCC*	.	.	.
18	50781932	A	G	intronic	*DCC*	.	.	.
18	50783014	A	G	intronic	*DCC*	.	.	.
18	50786777	A	G	intronic	*DCC*	.	.	.
18	51020228	T	C	intronic	*DCC*	.	.	.
12	55058907	T	C	intergenic	*DCD,MUCL1*	.	.	.
12	55108228	C	G	intergenic	*DCD,MUCL1*	.	.	.
12	55108546	G	C	intergenic	*DCD,MUCL1*	.	.	.
12	55172680	C	T	intergenic	*DCD,MUCL1*	.	.	.
12	55175580	T	C	intergenic	*DCD,MUCL1*	.	.	.
12	55201141	C	G	intergenic	*DCD,MUCL1*	.	.	.
12	55204192	G	A	intergenic	*DCD,MUCL1*	.	.	.
12	55207132	C	T	intergenic	*DCD,MUCL1*	.	.	.
12	55207134	G	T	intergenic	*DCD,MUCL1*	.	.	.
12	55207709	A	G	intergenic	*DCD,MUCL1*	.	.	.
11	31351899	C	G	intronic	*DCDC1*	.	.	.

11	31377840	C	T	intronic	*DCDC1*	.	.	.
11	31379455	G	A	intronic	*DCDC1*	.	.	.
6	24203423	G	A	intronic	*DCDC2*	.	.	.
6	24369182	C	T	intronic	*DCDC2*	.	.	.
6	24379633	A	G	intronic	*DCDC2*	.	.	.
6	24399521	G	A	intergenic	*DCDC2,MRS2*	.	.	.
2	3985825	T	G	intergenic	*DCDC2C,LINC01304*	.	.	.
11	30908065	T	A	intronic	*DCDC5*	.	.	.
11	30983218	G	T	intronic	*DCDC5*	.	.	.
11	30895125	C	T	intronic	*DCDC5*	.	.	.
11	30957268	G	T	intronic	*DCDC5*	.	.	.
11	30971297	G	C	intronic	*DCDC5*	.	.	.
11	30998191	C	A	intronic	*DCDC5*	.	.	.
11	31131464	G	T	intergenic	*DCDC5,DCDC1*	.	.	.
11	31205272	A	G	intergenic	*DCDC5,DCDC1*	.	.	.
11	31205615	T	G	intergenic	*DCDC5,DCDC1*	.	.	.
11	31206607	T	C	intergenic	*DCDC5,DCDC1*	.	.	.
11	31273162	CAGAG	C	intergenic	*DCDC5,DCDC1*	.	.	.
4	155370050	G	A	intronic	*DCHS2*	.	.	.
4	71894168	A	G	intronic	*DCK*	.	.	.
4	71880604	C	A	intronic	*DCK*	.	.	.
4	71913081	T	C	intergenic	*DCK,SLC4A4*	.	.	.
4	71913287	A	G	intergenic	*DCK,SLC4A4*	.	.	.
4	71952920	A	G	intergenic	*DCK,SLC4A4*	.	.	.
4	71952921	C	T	intergenic	*DCK,SLC4A4*	.	.	.
4	72009687	G	T	intergenic	*DCK,SLC4A4*	.	.	.
4	72045696	A	G	intergenic	*DCK,SLC4A4*	.	.	.
4	72045697	A	C	intergenic	*DCK,SLC4A4*	.	.	.
4	72046273	T	C	intergenic	*DCK,SLC4A4*	.	.	.
13	36648420	G	A	intronic	*DCLK1*	.	.	.
13	36727554	G	A	intergenic	*DCLK1,SOHLH2*	.	.	.
3	36845298	G	GA	intergenic	*DCLK3,TRANK1*	.	.	.
1	114465585	A	G	intergenic	*DCLRE1B,HIPK1-AS1*	.	.	.
12	91871782	G	T	intergenic	*DCN,LINC01619*	.	.	.
12	91696757	T	C	intergenic	*DCN,LINC01619*	.	.	.
12	92143833	G	A	intergenic	*DCN,LINC01619*	.	.	.

12	92062310	AGTGT	A	intergenic	*DCN,LINC01619*	.	.	.
12	91757638	C	G	intergenic	*DCN,LINC01619*	.	.	.
12	91854902	C	CAAAAAAAA	intergenic	*DCN,LINC01619*	.	.	.
12	91626295	G	A	intergenic	*DCN,LINC01619*	.	.	.
12	91693278	C	A	intergenic	*DCN,LINC01619*	.	.	.
12	91695083	A	G	intergenic	*DCN,LINC01619*	.	.	.
12	91855462	A	G	intergenic	*DCN,LINC01619*	.	.	.
12	91855883	G	C	intergenic	*DCN,LINC01619*	.	.	.
12	91911898	A	G	intergenic	*DCN,LINC01619*	.	.	.
12	91953627	A	C	intergenic	*DCN,LINC01619*	.	.	.
12	92144620	A	T	intergenic	*DCN,LINC01619*	.	.	.
3	53500015	T	TTTTTTTTTTT	intergenic	*DCP1A,CACNA1D*	.	.	.
3	53430298	A	T	intergenic	*DCP1A,CACNA1D*	.	.	.
3	53477841	A	T	intergenic	*DCP1A,CACNA1D*	.	.	.
3	53493872	G	T	intergenic	*DCP1A,CACNA1D*	.	.	.
3	53505580	G	A	intergenic	*DCP1A,CACNA1D*	.	.	.
11	126203778	C	G	intronic	*DCPS*	.	.	.
8	105377752	A	G	intergenic	*DCSTAMP,DPYS*	.	.	.
8	105375187	C	T	intergenic	*DCSTAMP,DPYS*	.	.	.
8	105389545	G	A	intergenic	*DCSTAMP,DPYS*	.	.	.
13	95134341	A	G	intergenic	*DCT,TGDS*	.	.	.
4	183918795	C	T	intergenic	*DCTD,FAM92A1P2*	.	.	.
4	183925985	C	A	intergenic	*DCTD,FAM92A1P2*	.	.	.
12	57925135	G	A	intronic	*DCTN2*	.	.	.
5	150100128	C	A	intronic	*DCTN4*	.	.	.
5	150126235	G	A	intronic	*DCTN4*	.	.	.

13	114125554	CAGAAGACCCAACTTCACAAGGGACCCAACTCCACAGGGGACCCAACTCCACAGAAGACACAACTCCACAGAAGACCCAACTCCACAGGGGACCCAACTCCAT	C	intronic	*DCUN1D2*	.	.	.
4	52805329	G	A	intergenic	*DCUN1D4,LRRC66*	.	.	.
4	52814769	C	T	intergenic	*DCUN1D4,LRRC66*	.	.	.
4	52817393	G	T	intergenic	*DCUN1D4,LRRC66*	.	.	.
4	52852347	T	G	intergenic	*DCUN1D4,LRRC66*	.	.	.
4	52852617	T	G	intergenic	*DCUN1D4,LRRC66*	.	.	.
X	110558217	T	G	intronic	*DCX*	.	.	.
X	110608628	G	A	intronic	*DCX*	.	.	.
X	110608629	C	T	intronic	*DCX*	.	.	.
X	110706988	A	G	intergenic	*DCX,LINC00890*	.	.	.
X	110713291	C	A	intergenic	*DCX,LINC00890*	.	.	.
1	86000124	A	T	intronic	*DDAH1*	.	.	.
7	50574091	C	T	intronic	*DDC*	.	.	.
7	50639619	T	C	intergenic	*DDC,GRB10*	.	.	.
14	53550506	T	G	intronic	*DDHD1*	.	.	.
14	53556627	G	A	intronic	*DDHD1*	.	.	.
14	53556826	C	A	intronic	*DDHD1*	.	.	.
1	162718747	T	C	intronic	*DDR2*	.	.	.
11	108752070	C	G	intronic	*DDX10*	.	.	.
11	108687614	A	G	intronic	*DDX10*	.	.	.
11	109139465	G	A	intergenic	*DDX10,C11orf87*	.	.	.
11	108851638	T	A	intergenic	*DDX10,C11orf87*	.	.	.
11	109228864	CTT	C	intergenic	*DDX10,C11orf87*	.	.	.
11	108821131	G	T	intergenic	*DDX10,C11orf87*	.	.	.
11	108876526	C	T	intergenic	*DDX10,C11orf87*	.	.	.

11	108901466	A	G	intergenic	*DDX10,C11orf87*	.	.	.
11	108982015	C	T	intergenic	*DDX10,C11orf87*	.	.	.
11	109058024	T	A	intergenic	*DDX10,C11orf87*	.	.	.
11	109115293	C	A	intergenic	*DDX10,C11orf87*	.	.	.
11	109115295	G	A	intergenic	*DDX10,C11orf87*	.	.	.
11	109186535	T	C	intergenic	*DDX10,C11orf87*	.	.	.
11	109272691	T	C	intergenic	*DDX10,C11orf87*	.	.	.
12	31343150	A	G	intergenic	*DDX11,FAM60A*	.	.	.
12	31344172	C	T	intergenic	*DDX11,FAM60A*	.	.	.
12	31352628	T	C	intergenic	*DDX11,FAM60A*	.	.	.
9	11042	A	C	upstream	*DDX11L5*	.	.	.
12	9619694	C	T	intergenic	*DDX12P,KLRB1*	.	.	.
12	9634309	G	C	intergenic	*DDX12P,KLRB1*	.	.	.
12	9732323	T	C	intergenic	*DDX12P,KLRB1*	.	.	.
12	9732821	A	G	intergenic	*DDX12P,KLRB1*	.	.	.
2	118585309	G	A	intronic	*DDX18*	.	.	.
2	118596876	G	A	intergenic	*DDX18,CCDC93*	.	.	.
2	118656402	T	C	intergenic	*DDX18,CCDC93*	.	.	.
2	118659369	T	G	intergenic	*DDX18,CCDC93*	.	.	.
2	118664416	A	T	intergenic	*DDX18,CCDC93*	.	.	.
2	118665305	C	T	intergenic	*DDX18,CCDC93*	.	.	.
14	94533783	C	T	intronic	*DDX24*	.	.	.
X	134801798	T	TG	intergenic	*DDX26B,CT45A1*	.	.	.
X	134796123	C	T	intergenic	*DDX26B,CT45A1*	.	.	.
X	134816817	A	G	intergenic	*DDX26B,CT45A1*	.	.	.
X	134817091	A	G	intergenic	*DDX26B,CT45A1*	.	.	.
X	134817093	T	C	intergenic	*DDX26B,CT45A1*	.	.	.
X	134842967	T	A	intergenic	*DDX26B,CT45A1*	.	.	.
5	55101954	C	T	intronic	*DDX4*	.	.	.
5	134137006	A	G	intronic	*DDX46*	.	.	.
10	70677741	C	T	intronic	*DDX50*	.	.	.
17	36017068	C	T	intergenic	*DDX52,HNF1B*	.	.	.
17	36039795	C	A	intergenic	*DDX52,HNF1B*	.	.	.
17	36039984	G	A	intergenic	*DDX52,HNF1B*	.	.	.
9	32511136	G	A	intronic	*DDX58*	.	.	.
4	169250961	T	C	intergenic	*DDX60,DDX60L*	.	.	.
9	118226791	T	C	intergenic	*DEC1,LOC101928775*	.	.	.

9	118498014	A	T	intergenic	*DEC1,LOC101928775*	.	.	.
9	118470112	T	C	intergenic	*DEC1,LOC101928775*	.	.	.
9	118488448	G	GC	intergenic	*DEC1,LOC101928775*	.	.	.
8	91027073	A	G	intronic	*DECR1*	.	.	.
8	91036277	G	A	intronic	*DECR1*	.	.	.
8	91039286	G	C	intronic	*DECR1*	.	.	.
8	91039288	C	A	intronic	*DECR1*	.	.	.
8	91060149	T	C	intronic	*DECR1*	.	.	.
8	91060892	T	G	intronic	*DECR1*	.	.	.
8	91061709	T	G	intronic	*DECR1*	.	.	.
8	91062397	A	T	intronic	*DECR1*	.	.	.
6	35303179	T	A	intergenic	*DEF6,PPARD*	.	.	.
6	35303181	G	T	intergenic	*DEF6,PPARD*	.	.	.
6	35305153	G	T	intergenic	*DEF6,PPARD*	.	.	.
6	35305466	C	T	intergenic	*DEF6,PPARD*	.	.	.
8	6800960	G	A	intergenic	*DEFA4,DEFA8P*	.	.	.
8	6931263	G	A	intergenic	*DEFA5,LINC00965*	.	.	.
8	6944465	G	T	intergenic	*DEFA5,LINC00965*	.	.	.
8	6984380	C	T	intergenic	*DEFA5,LINC00965*	.	.	.
8	6990080	C	A	intergenic	*DEFA5,LINC00965*	.	.	.
8	6990081	A	G	intergenic	*DEFA5,LINC00965*	.	.	.
8	7749600	C	T	intergenic	*DEFB103A,DEFB4A*	.	.	.
8	7343040	A	T	intronic	*DEFB106A,DEFB106B*	.	.	.
11	71561908	T	C	intergenic	*DEFB108B,LOC100133315*	.	.	.
6	50017076	A	C	upstream	*DEFB112*	.	.	.
6	50444787	C	G	intergenic	*DEFB112,TFAP2D*	.	.	.
6	50027659	A	T	intergenic	*DEFB112,TFAP2D*	.	.	.
6	50488677	T	C	intergenic	*DEFB112,TFAP2D*	.	.	.
6	50515679	CATAT	C	intergenic	*DEFB112,TFAP2D*	.	.	.
6	50093082	G	GCACACA	intergenic	*DEFB112,TFAP2D*	.	.	.
6	50386906	A	G	intergenic	*DEFB112,TFAP2D*	.	.	.
6	50459492	A	G	intergenic	*DEFB112,TFAP2D*	.	.	.

6	50543247	G	A	intergenic	*DEFB112,TFAP2D*	.	.	.
6	50049523	T	G	intergenic	*DEFB112,TFAP2D*	.	.	.
6	50049789	T	C	intergenic	*DEFB112,TFAP2D*	.	.	.
6	50193887	C	T	intergenic	*DEFB112,TFAP2D*	.	.	.
6	50193888	A	G	intergenic	*DEFB112,TFAP2D*	.	.	.
6	50243045	A	C	intergenic	*DEFB112,TFAP2D*	.	.	.
6	50243963	A	G	intergenic	*DEFB112,TFAP2D*	.	.	.
6	50243964	G	A	intergenic	*DEFB112,TFAP2D*	.	.	.
6	50314438	A	G	intergenic	*DEFB112,TFAP2D*	.	.	.
6	50315808	G	C	intergenic	*DEFB112,TFAP2D*	.	.	.
6	50316636	A	C	intergenic	*DEFB112,TFAP2D*	.	.	.
6	50361945	G	C	intergenic	*DEFB112,TFAP2D*	.	.	.
6	50455082	T	A	intergenic	*DEFB112,TFAP2D*	.	.	.
6	50455310	C	A	intergenic	*DEFB112,TFAP2D*	.	.	.
6	50455715	C	T	intergenic	*DEFB112,TFAP2D*	.	.	.
6	50455716	T	A	intergenic	*DEFB112,TFAP2D*	.	.	.
6	50456682	C	A	intergenic	*DEFB112,TFAP2D*	.	.	.
6	50589766	C	T	intergenic	*DEFB112,TFAP2D*	.	.	.
6	50589958	C	G	intergenic	*DEFB112,TFAP2D*	.	.	.
6	50613101	A	C	intergenic	*DEFB112,TFAP2D*	.	.	.
6	50613376	A	G	intergenic	*DEFB112,TFAP2D*	.	.	.
6	50614754	T	C	intergenic	*DEFB112,TFAP2D*	.	.	.
6	50614756	A	G	intergenic	*DEFB112,TFAP2D*	.	.	.
20	29851276	C	T	intergenic	*DEFB115,DEFB116*	.	.	.
20	29854094	T	G	intergenic	*DEFB115,DEFB116*	.	.	.
20	29922896	A	G	intergenic	*DEFB116,DEFB118*	.	.	.
20	30032518	T	C	intronic	*DEFB123*	.	.	.
8	11871680	G	A	intergenic	*DEFB134,DEFB130*	.	.	.
6	18377286	T	C	intergenic	*DEK,RNF144B*	.	.	.
6	18355738	T	TG	intergenic	*DEK,RNF144B*	.	.	.
6	18356227	T	C	intergenic	*DEK,RNF144B*	.	.	.
6	18356228	G	A	intergenic	*DEK,RNF144B*	.	.	.
9	126593765	CAAGAAAAAACTTTT	C	intronic	*DENND1A*	.	.	.

9	126609210	C	A	intronic	*DENND1A*	.	.	.
9	126235908	C	T	intronic	*DENND1A*	.	.	.
9	126484342	C	A	intronic	*DENND1A*	.	.	.
1	197640248	A	T	intronic	*DENND1B*	.	.	.
1	197640250	A	T	intronic	*DENND1B*	.	.	.
1	197593446	A	G	intronic	*DENND1B*	.	.	.
1	197704989	C	T	intronic	*DENND1B*	.	.	.
1	197706579	G	A	intronic	*DENND1B*	.	.	.
1	197542402	T	C	intronic	*DENND1B*	.	.	.
1	197543982	G	T	intronic	*DENND1B*	.	.	.
1	197544861	G	A	intronic	*DENND1B*	.	.	.
1	197544862	T	C	intronic	*DENND1B*	.	.	.
1	197595632	C	G	intronic	*DENND1B*	.	.	.
1	197670282	T	C	intronic	*DENND1B*	.	.	.
1	197764604	A	G	intergenic	*DENND1B,C1orf53*	.	.	.
1	197764606	T	C	intergenic	*DENND1B,C1orf53*	.	.	.
1	197804666	T	A	intergenic	*DENND1B,C1orf53*	.	.	.
1	197824897	C	A	intergenic	*DENND1B,C1orf53*	.	.	.
7	140306226	A	G	intergenic	*DENND2A,ADCK2*	.	.	.
7	140365540	C	T	intergenic	*DENND2A,ADCK2*	.	.	.
9	19265883	A	G	intronic	*DENND4C*	.	.	.
12	31690369	T	C	intronic	*DENND5B*	.	.	.
3	57637039	G	A	intronic	*DENND6A*	.	.	.
1	69485804	G	A	intergenic	*DEPDC1-AS1,LRRC7*	.	.	.
1	70034749	GACAC	G	intergenic	*DEPDC1-AS1,LRRC7*	.	.	.
1	69334195	G	T	intergenic	*DEPDC1-AS1,LRRC7*	.	.	.
1	69385047	C	A	intergenic	*DEPDC1-AS1,LRRC7*	.	.	.
1	69467359	T	C	intergenic	*DEPDC1-AS1,LRRC7*	.	.	.
1	70108494	CATAGATAG	C	intergenic	*DEPDC1-AS1,LRRC7*	.	.	.
1	69316660	T	TAC	intergenic	*DEPDC1-AS1,LRRC7*	.	.	.

1	69049306	C	CA	intergenic	*DEPDC1-AS1,LRRC7*	.	.	.
1	69148726	T	G	intergenic	*DEPDC1-AS1,LRRC7*	.	.	.
1	69456631	C	T	intergenic	*DEPDC1-AS1,LRRC7*	.	.	.
1	69533347	G	A	intergenic	*DEPDC1-AS1,LRRC7*	.	.	.
1	69533547	T	C	intergenic	*DEPDC1-AS1,LRRC7*	.	.	.
1	69583051	T	C	intergenic	*DEPDC1-AS1,LRRC7*	.	.	.
1	69583538	C	T	intergenic	*DEPDC1-AS1,LRRC7*	.	.	.
1	69810107	T	C	intergenic	*DEPDC1-AS1,LRRC7*	.	.	.
1	69810109	C	T	intergenic	*DEPDC1-AS1,LRRC7*	.	.	.
1	69855831	G	GT	intergenic	*DEPDC1-AS1,LRRC7*	.	.	.
1	69911317	T	C	intergenic	*DEPDC1-AS1,LRRC7*	.	.	.
1	69911318	G	A	intergenic	*DEPDC1-AS1,LRRC7*	.	.	.
1	69912405	A	G	intergenic	*DEPDC1-AS1,LRRC7*	.	.	.
5	59913697	C	T	intronic	*DEPDC1B*	.	.	.
5	59914363	G	A	intronic	*DEPDC1B*	.	.	.
5	59921231	C	T	intronic	*DEPDC1B*	.	.	.
5	60026966	G	A	intergenic	*DEPDC1B,ELOVL7*	.	.	.
5	60028043	G	C	intergenic	*DEPDC1B,ELOVL7*	.	.	.
5	60028077	A	G	intergenic	*DEPDC1B,ELOVL7*	.	.	.
5	60029087	G	A	intergenic	*DEPDC1B,ELOVL7*	.	.	.

5	60029427	T	C	intergenic	*DEPDC1B,ELOVL7*	.	.	.
22	32156574	C	T	intronic	*DEPDC5*	.	.	.
22	32245624	G	A	intronic	*DEPDC5*	.	.	.
8	120927058	T	A	intronic	*DEPTOR*	.	.	.
8	120905314	C	T	intronic	*DEPTOR*	.	.	.
8	120950039	C	A	intronic	*DEPTOR*	.	.	.
8	120885135	G	A	upstream	*DEPTOR*	.	.	.
12	16097627	A	G	intronic	*DERA*	.	.	.
12	16338792	C	T	intergenic	*DERA,SLC15A5*	.	.	.
22	24195318	A	AT	intergenic	*DERL3,SLC2A11*	.	.	.
1	244838134	T	C	intronic	*DESI2*	.	.	.
1	244956323	TCTGTCGCC	T	intergenic	*DESI2,COX20*	.	.	.
1	244962508	ACAAGCACACACATTTAAAAATGTGTGAGCAAGCACACACATTTAAAAATGTGTGAG	A	intergenic	*DESI2,COX20*	.	.	.
7	24751466	T	C	intronic	*DFNA5*	.	.	.
7	24814316	T	C	intergenic	*DFNA5,OSBPL3*	.	.	.
7	24821966	C	G	intergenic	*DFNA5,OSBPL3*	.	.	.
X	69435031	T	G	intergenic	*DGAT2L6,AWAT1*	.	.	.
X	69433712	G	A	intergenic	*DGAT2L6,AWAT1*	.	.	.
7	14592997	G	A	intronic	*DGKB*	.	.	.
7	14633281	A	G	intronic	*DGKB*	.	.	.
7	14337946	T	G	intronic	*DGKB*	.	.	.
7	14338603	CA	C	intronic	*DGKB*	.	.	.
7	14397646	T	A	intronic	*DGKB*	.	.	.
7	15103230	A	G	intergenic	*DGKB,AGMO*	.	.	.
7	15104003	G	A	intergenic	*DGKB,AGMO*	.	.	.
13	42634641	C	T	intronic	*DGKH*	.	.	.
X	50164110	C	T	intronic	*DGKK*	.	.	.
X	50284792	AG	A	intergenic	*DGKK,SHROOM4*	.	.	.
X	50332237	T	A	intergenic	*DGKK,SHROOM4*	.	.	.

X	50232787	G	A	intergenic	*DGKK,SHROOM4*	.	.	.
X	50234466	G	T	intergenic	*DGKK,SHROOM4*	.	.	.
4	962067	C	T	intronic	*DGKQ*	.	.	.
4	962068	A	C	splicing	*DGKQ*	.	.	.
5	79947414	G	GAGA	intronic	*DHFR*	.	.	.
14	24124361	G	A	intergenic	*DHRS2,DHRS4-AS1*	.	.	.
14	24156939	G	C	intergenic	*DHRS2,DHRS4-AS1*	.	.	.
14	24156941	G	A	intergenic	*DHRS2,DHRS4-AS1*	.	.	.
14	24157564	T	C	intergenic	*DHRS2,DHRS4-AS1*	.	.	.
14	24157566	C	G	intergenic	*DHRS2,DHRS4-AS1*	.	.	.
14	24208965	A	T	intergenic	*DHRS2,DHRS4-AS1*	.	.	.
14	24226327	A	T	intergenic	*DHRS2,DHRS4-AS1*	.	.	.
14	24227122	T	C	intergenic	*DHRS2,DHRS4-AS1*	.	.	.
14	24227655	T	G	intergenic	*DHRS2,DHRS4-AS1*	.	.	.
14	24227656	T	G	intergenic	*DHRS2,DHRS4-AS1*	.	.	.
14	24228613	G	A	intergenic	*DHRS2,DHRS4-AS1*	.	.	.
14	24291035	C	T	intergenic	*DHRS2,DHRS4-AS1*	.	.	.
14	24291693	T	C	intergenic	*DHRS2,DHRS4-AS1*	.	.	.
14	24487690	G	A	intronic	*DHRS4L1*	.	.	.
14	24497069	T	C	intronic	*DHRS4L1*	.	.	.
14	24442891	G	A	intronic	*DHRS4L2*	.	.	.
14	24443764	C	G	intronic	*DHRS4L2*	.	.	.
14	24445374	A	C	intronic	*DHRS4L2*	.	.	.
X	2171892	A	G	intronic	*DHRSX*	.	.	.
X	2171894	T	G	intronic	*DHRSX*	.	.	.

20	37659995	G	T	intronic	*DHX35*	.	.	.
17	40262231	T	A	intronic	*DHX58*	.	.	.
X	96053630	A	G	intronic	*DIAPH2*	.	.	.
X	96213511	T	A	intronic	*DIAPH2*	.	.	.
X	96066273	A	T	intronic	*DIAPH2*	.	.	.
X	96067609	A	G	intronic	*DIAPH2*	.	.	.
X	96344948	C	T	intronic	*DIAPH2*	.	.	.
X	96547089	TG	T	intronic	*DIAPH2*	.	.	.
X	98455490	C	A	intergenic	*DIAPH2,XRCC6P5*	.	.	.
X	98573831	T	C	intergenic	*DIAPH2,XRCC6P5*	.	.	.
X	98505611	A	G	intergenic	*DIAPH2,XRCC6P5*	.	.	.
X	98330604	C	T	intergenic	*DIAPH2,XRCC6P5*	.	.	.
X	97297915	G	C	intergenic	*DIAPH2,XRCC6P5*	.	.	.
X	96907033	G	T	intergenic	*DIAPH2,XRCC6P5*	.	.	.
X	96907093	G	A	intergenic	*DIAPH2,XRCC6P5*	.	.	.
X	97053253	C	T	intergenic	*DIAPH2,XRCC6P5*	.	.	.
X	97173636	C	G	intergenic	*DIAPH2,XRCC6P5*	.	.	.
X	97175655	T	C	intergenic	*DIAPH2,XRCC6P5*	.	.	.
X	97181870	T	A	intergenic	*DIAPH2,XRCC6P5*	.	.	.
X	97182626	T	C	intergenic	*DIAPH2,XRCC6P5*	.	.	.
X	97299132	C	A	intergenic	*DIAPH2,XRCC6P5*	.	.	.
X	97299656	G	C	intergenic	*DIAPH2,XRCC6P5*	.	.	.
X	97300982	G	A	intergenic	*DIAPH2,XRCC6P5*	.	.	.
X	97301218	T	C	intergenic	*DIAPH2,XRCC6P5*	.	.	.
X	97301219	G	A	intergenic	*DIAPH2,XRCC6P5*	.	.	.
X	97348210	A	G	intergenic	*DIAPH2,XRCC6P5*	.	.	.
X	97382555	T	G	intergenic	*DIAPH2,XRCC6P5*	.	.	.
X	97490544	T	C	intergenic	*DIAPH2,XRCC6P5*	.	.	.
X	97493122	C	G	intergenic	*DIAPH2,XRCC6P5*	.	.	.
X	97494215	G	A	intergenic	*DIAPH2,XRCC6P5*	.	.	.
X	97559550	C	T	intergenic	*DIAPH2,XRCC6P5*	.	.	.
X	97577886	G	C	intergenic	*DIAPH2,XRCC6P5*	.	.	.
X	97577887	T	A	intergenic	*DIAPH2,XRCC6P5*	.	.	.
X	97662718	C	T	intergenic	*DIAPH2,XRCC6P5*	.	.	.
X	97671886	G	C	intergenic	*DIAPH2,XRCC6P5*	.	.	.
X	97697726	A	C	intergenic	*DIAPH2,XRCC6P5*	.	.	.
X	97702083	T	C	intergenic	*DIAPH2,XRCC6P5*	.	.	.
X	97816065	A	T	intergenic	*DIAPH2,XRCC6P5*	.	.	.

X	97818503	T	A	intergenic	*DIAPH2,XRCC6P5*	.	.	.
X	97924476	T	C	intergenic	*DIAPH2,XRCC6P5*	.	.	.
X	97949133	G	A	intergenic	*DIAPH2,XRCC6P5*	.	.	.
X	97949134	A	G	intergenic	*DIAPH2,XRCC6P5*	.	.	.
X	97950883	T	C	intergenic	*DIAPH2,XRCC6P5*	.	.	.
X	97950884	G	A	intergenic	*DIAPH2,XRCC6P5*	.	.	.
X	97951392	T	C	intergenic	*DIAPH2,XRCC6P5*	.	.	.
X	98089702	A	G	intergenic	*DIAPH2,XRCC6P5*	.	.	.
X	98090750	A	G	intergenic	*DIAPH2,XRCC6P5*	.	.	.
X	98090752	A	G	intergenic	*DIAPH2,XRCC6P5*	.	.	.
X	98495529	G	A	intergenic	*DIAPH2,XRCC6P5*	.	.	.
X	98578404	G	A	intergenic	*DIAPH2,XRCC6P5*	.	.	.
X	98578405	G	A	intergenic	*DIAPH2,XRCC6P5*	.	.	.
13	60629518	T	A	intronic	*DIAPH3*	.	.	.
13	60686634	G	C	intronic	*DIAPH3*	.	.	.
13	60283669	T	C	intronic	*DIAPH3*	.	.	.
13	60424688	T	C	intronic	*DIAPH3*	.	.	.
13	60620577	C	G	intronic	*DIAPH3*	.	.	.
13	60721903	G	A	ncRNA_intronic	*DIAPH3-AS2*	.	.	.
1	210075909	C	A	intergenic	*DIEXF,SYT14*	.	.	.
14	80664101	A	C	UTR3	*DIO2*	.	.	.
14	80715753	C	T	ncRNA_intronic	*DIO2-AS1*	.	.	.
14	80764142	A	G	ncRNA_intronic	*DIO2-AS1*	.	.	.
14	80931231	G	A	intergenic	*DIO2-AS1,CEP128*	.	.	.
14	80955409	C	A	intergenic	*DIO2-AS1,CEP128*	.	.	.
14	102021138	G	C	ncRNA_exonic	*DIO3OS*	.	.	.
21	47965065	T	C	exonic	*DIP2A*	nonsynonymous_SNV	.	.
21	47959301	C	T	intronic	*DIP2A*	.	.	.
12	50995386	A	G	intronic	*DIP2B*	.	.	.
12	51149465	C	T	intergenic	*DIP2B,ATF1*	.	.	.
10	509028	T	TC	intronic	*DIP2C*	.	.	.
10	509029	A	C	intronic	*DIP2C*	.	.	.
10	509685	A	AC	intronic	*DIP2C*	.	.	.
10	509686	G	T	intronic	*DIP2C*	.	.	.
10	512480	G	GA	intronic	*DIP2C*	.	.	.
10	826629	A	G	intergenic	*DIP2C,LARP4B*	.	.	.
2	189633250	G	A	intronic	*DIRC1*	.	.	.

2	189823808	G	A	intergenic	*DIRC1,COL3A1*	.	.	.
2	218209059	C	G	ncRNA_intronic	*DIRC3*	.	.	.
2	218541128	T	C	ncRNA_intronic	*DIRC3*	.	.	.
2	232900652	A	T	intronic	*DIS3L2*	.	.	.
2	232900653	G	C	intronic	*DIS3L2*	.	.	.
2	232897024	A	G	intronic	*DIS3L2*	.	.	.
2	232897515	G	A	intronic	*DIS3L2*	.	.	.
2	232898961	T	C	intronic	*DIS3L2*	.	.	.
2	232898962	G	A	intronic	*DIS3L2*	.	.	.
2	232899978	T	C	intronic	*DIS3L2*	.	.	.
2	233052299	C	T	intronic	*DIS3L2*	.	.	.
2	233210301	A	C	intergenic	*DIS3L2,ALPP*	.	.	.
2	233221630	T	C	intergenic	*DIS3L2,ALPP*	.	.	.
2	233224884	G	A	intergenic	*DIS3L2,ALPP*	.	.	.
11	90500544	G	A	ncRNA_intronic	*DISC1FP1*	.	.	.
11	90435486	C	G	ncRNA_intronic	*DISC1FP1*	.	.	.
11	90002003	T	A	ncRNA_intronic	*DISC1FP1*	.	.	.
11	90031267	C	A	ncRNA_intronic	*DISC1FP1*	.	.	.
11	90031269	C	G	ncRNA_intronic	*DISC1FP1*	.	.	.
11	90032851	A	T	ncRNA_intronic	*DISC1FP1*	.	.	.
11	90032852	A	C	ncRNA_intronic	*DISC1FP1*	.	.	.
11	90032882	C	T	ncRNA_intronic	*DISC1FP1*	.	.	.
11	90033588	C	T	ncRNA_intronic	*DISC1FP1*	.	.	.
11	90039961	T	C	ncRNA_intronic	*DISC1FP1*	.	.	.
11	90132122	C	G	ncRNA_intronic	*DISC1FP1*	.	.	.
11	90313663	G	C	ncRNA_intronic	*DISC1FP1*	.	.	.
11	90315325	C	T	ncRNA_intronic	*DISC1FP1*	.	.	.
11	90324516	C	T	ncRNA_intronic	*DISC1FP1*	.	.	.
11	90369916	G	T	ncRNA_intronic	*DISC1FP1*	.	.	.
11	90423621	A	G	ncRNA_intronic	*DISC1FP1*	.	.	.
11	90506597	A	G	ncRNA_intronic	*DISC1FP1*	.	.	.
11	90506602	G	A	ncRNA_intronic	*DISC1FP1*	.	.	.
11	90613734	C	A	ncRNA_intronic	*DISC1FP1*	.	.	.
11	90615570	A	T	ncRNA_intronic	*DISC1FP1*	.	.	.
11	90657616	C	T	intergenic	*DISC1FP1,FAT3*	.	.	.
11	91007636	A	T	intergenic	*DISC1FP1,FAT3*	.	.	.
11	91216755	T	A	intergenic	*DISC1FP1,FAT3*	.	.	.
11	91453138	G	T	intergenic	*DISC1FP1,FAT3*	.	.	.

11	91565603	T	C	intergenic	*DISC1FP1,FAT3*	.	.	.
11	91862986	G	C	intergenic	*DISC1FP1,FAT3*	.	.	.
11	91281446	A	G	intergenic	*DISC1FP1,FAT3*	.	.	.
11	90695769	A	T	intergenic	*DISC1FP1,FAT3*	.	.	.
11	90962862	G	T	intergenic	*DISC1FP1,FAT3*	.	.	.
11	91676150	A	G	intergenic	*DISC1FP1,FAT3*	.	.	.
11	91557405	C	T	intergenic	*DISC1FP1,FAT3*	.	.	.
11	92008202	G	C	intergenic	*DISC1FP1,FAT3*	.	.	.
11	90707655	C	T	intergenic	*DISC1FP1,FAT3*	.	.	.
11	90714910	T	A	intergenic	*DISC1FP1,FAT3*	.	.	.
11	90750635	T	C	intergenic	*DISC1FP1,FAT3*	.	.	.
11	90750637	A	G	intergenic	*DISC1FP1,FAT3*	.	.	.
11	90767275	G	T	intergenic	*DISC1FP1,FAT3*	.	.	.
11	90769660	A	C	intergenic	*DISC1FP1,FAT3*	.	.	.
11	90770543	TA	T	intergenic	*DISC1FP1,FAT3*	.	.	.
11	90806459	G	A	intergenic	*DISC1FP1,FAT3*	.	.	.
11	90958088	T	G	intergenic	*DISC1FP1,FAT3*	.	.	.
11	90958089	A	G	intergenic	*DISC1FP1,FAT3*	.	.	.
11	90985117	A	G	intergenic	*DISC1FP1,FAT3*	.	.	.
11	90990340	T	A	intergenic	*DISC1FP1,FAT3*	.	.	.
11	91053150	A	G	intergenic	*DISC1FP1,FAT3*	.	.	.
11	91053534	C	T	intergenic	*DISC1FP1,FAT3*	.	.	.
11	91079619	G	A	intergenic	*DISC1FP1,FAT3*	.	.	.
11	91079621	A	C	intergenic	*DISC1FP1,FAT3*	.	.	.
11	91113592	C	A	intergenic	*DISC1FP1,FAT3*	.	.	.
11	91134985	A	C	intergenic	*DISC1FP1,FAT3*	.	.	.
11	91185908	C	T	intergenic	*DISC1FP1,FAT3*	.	.	.
11	91205988	A	T	intergenic	*DISC1FP1,FAT3*	.	.	.
11	91311572	G	C	intergenic	*DISC1FP1,FAT3*	.	.	.
11	91313562	G	A	intergenic	*DISC1FP1,FAT3*	.	.	.
11	91353865	A	ATTTTTCTCC	intergenic	*DISC1FP1,FAT3*	.	.	.
11	91743086	C	T	intergenic	*DISC1FP1,FAT3*	.	.	.
11	91745798	G	GC	intergenic	*DISC1FP1,FAT3*	.	.	.
11	91827687	A	C	intergenic	*DISC1FP1,FAT3*	.	.	.
11	91906475	C	A	intergenic	*DISC1FP1,FAT3*	.	.	.
11	91906795	T	G	intergenic	*DISC1FP1,FAT3*	.	.	.
11	91907715	T	C	intergenic	*DISC1FP1,FAT3*	.	.	.
1	223003289	A	G	intronic	*DISP1*	.	.	.

1	223032462	A	G	intronic	*DISP1*	.	.	.
1	223044373	T	C	intronic	*DISP1*	.	.	.
1	223044558	C	T	intronic	*DISP1*	.	.	.
1	223076645	TG	T	intronic	*DISP1*	.	.	.
1	223134732	A	G	intronic	*DISP1*	.	.	.
11	111878074	C	T	intronic	*DIXDC1*	.	.	.
11	111879909	G	A	intronic	*DIXDC1*	.	.	.
11	111881674	G	A	intronic	*DIXDC1*	.	.	.
11	111881902	C	A	intronic	*DIXDC1*	.	.	.
15	30613891	TTTTATTTATTTA	T	intergenic	*DKFZP434L187,CHRFAM7A*	.	.	.
7	56574547	C	T	intergenic	*DKFZp434L192,LOC101928401*	.	.	.
7	56576191	C	T	intergenic	*DKFZp434L192,LOC101928401*	.	.	.
6	163804722	C	T	intergenic	*DKFZp451B082,CAHM*	.	.	.
7	30421303	T	TC	intergenic	*DKFZP586I1420,LINC01176*	.	.	.
7	30422005	C	T	intergenic	*DKFZP586I1420,LINC01176*	.	.	.
7	30422006	G	A	intergenic	*DKFZP586I1420,LINC01176*	.	.	.
11	32040195	T	A	intergenic	*DKFZp686K1684,LOC100506675*	.	.	.
11	31930658	A	G	intergenic	*DKFZp686K1684,LOC100506675*	.	.	.
11	31972787	T	C	intergenic	*DKFZp686K1684,LOC100506675*	.	.	.
10	54206338	T	C	intergenic	*DKK1,LINC01468*	.	.	.
10	54120707	A	G	intergenic	*DKK1,LINC01468*	.	.	.
10	54121007	C	G	intergenic	*DKK1,LINC01468*	.	.	.
10	54122971	T	C	intergenic	*DKK1,LINC01468*	.	.	.
10	54130207	C	T	intergenic	*DKK1,LINC01468*	.	.	.
10	54130653	A	C	intergenic	*DKK1,LINC01468*	.	.	.
10	54132462	C	T	intergenic	*DKK1,LINC01468*	.	.	.
4	107896789	C	T	intronic	*DKK2*	.	.	.
4	108274188	A	T	intergenic	*DKK2,PAPSS1*	.	.	.

4	108405625	A	T	intergenic	*DKK2,PAPSS1*	.	.	.
11	12101236	G	GC	intergenic	*DKK3,MICAL2*	.	.	.
11	12101238	A	C	intergenic	*DKK3,MICAL2*	.	.	.
11	111919057	C	T	intronic	*DLAT*	.	.	.
11	111919058	A	G	intronic	*DLAT*	.	.	.
8	13130800	G	T	intronic	*DLC1*	.	.	.
8	13205611	CAAACAAAT	C	intronic	*DLC1*	.	.	.
8	13206858	A	G	intronic	*DLC1*	.	.	.
8	13039532	T	G	intronic	*DLC1*	.	.	.
8	13041694	A	G	intronic	*DLC1*	.	.	.
8	13203192	G	A	intronic	*DLC1*	.	.	.
8	13233154	C	T	intronic	*DLC1*	.	.	.
8	13275499	C	T	intronic	*DLC1*	.	.	.
8	13275941	A	T	intronic	*DLC1*	.	.	.
8	13276439	G	A	intronic	*DLC1*	.	.	.
8	13299604	A	C	intronic	*DLC1*	.	.	.
8	13421838	T	A	intergenic	*DLC1,C8orf48*	.	.	.
8	13377745	G	A	intergenic	*DLC1,C8orf48*	.	.	.
13	51085644	G	C	ncRNA_intronic	*DLEU1*	.	.	.
13	50739770	C	T	ncRNA_intronic	*DLEU1*	.	.	.
13	50740075	G	A	ncRNA_intronic	*DLEU1*	.	.	.
13	51078339	A	G	ncRNA_intronic	*DLEU1*	.	.	.
13	51129288	C	T	intergenic	*DLEU1,DLEU7*	.	.	.
13	51407945	T	C	ncRNA_intronic	*DLEU7-AS1*	.	.	.
3	197117814	A	G	intergenic	*DLG1-AS1,BDH1*	.	.	.
3	197104051	T	G	intergenic	*DLG1-AS1,BDH1*	.	.	.
11	83525943	T	C	intronic	*DLG2*	.	.	.
11	84869836	C	CGCCG	intronic	*DLG2*	.	.	.
11	83445673	T	C	intronic	*DLG2*	.	.	.
11	84190401	T	A	intronic	*DLG2*	.	.	.
11	84968529	G	T	intronic	*DLG2*	.	.	.
11	85296757	C	T	intronic	*DLG2*	.	.	.
11	83841581	G	GTA	intronic	*DLG2*	.	.	.
11	84392733	A	G	intronic	*DLG2*	.	.	.
11	84521608	C	A	intronic	*DLG2*	.	.	.
11	84423474	T	A	intronic	*DLG2*	.	.	.
11	84350442	G	T	intronic	*DLG2*	.	.	.

11	83223873	G	A	intronic	*DLG2*	.	.	.
11	83223874	C	T	intronic	*DLG2*	.	.	.
11	83224427	A	T	intronic	*DLG2*	.	.	.
11	83224705	A	G	intronic	*DLG2*	.	.	.
11	83226178	C	T	intronic	*DLG2*	.	.	.
11	83553853	T	A	intronic	*DLG2*	.	.	.
11	83553854	G	GA	intronic	*DLG2*	.	.	.
11	83749469	T	C	intronic	*DLG2*	.	.	.
11	83763563	T	C	intronic	*DLG2*	.	.	.
11	83865959	T	G	intronic	*DLG2*	.	.	.
11	83895533	A	G	intronic	*DLG2*	.	.	.
11	83913571	C	T	intronic	*DLG2*	.	.	.
11	84075988	A	T	intronic	*DLG2*	.	.	.
11	84152917	G	A	intronic	*DLG2*	.	.	.
11	84328583	A	G	intronic	*DLG2*	.	.	.
11	84437922	C	T	intronic	*DLG2*	.	.	.
11	84438973	G	T	intronic	*DLG2*	.	.	.
11	84439684	T	A	intronic	*DLG2*	.	.	.
11	84550459	T	G	intronic	*DLG2*	.	.	.
11	84573012	C	T	intronic	*DLG2*	.	.	.
11	84588804	G	A	intronic	*DLG2*	.	.	.
11	84588805	T	C	intronic	*DLG2*	.	.	.
11	84699068	G	A	intronic	*DLG2*	.	.	.
11	84703762	A	C	intronic	*DLG2*	.	.	.
11	84704117	T	C	intronic	*DLG2*	.	.	.
11	84717795	C	T	intronic	*DLG2*	.	.	.
11	84984583	C	T	intronic	*DLG2*	.	.	.
11	85062519	T	C	intronic	*DLG2*	.	.	.
11	85063152	G	A	intronic	*DLG2*	.	.	.
11	85063274	A	G	intronic	*DLG2*	.	.	.
11	85064292	T	C	intronic	*DLG2*	.	.	.
11	85109260	G	T	intronic	*DLG2*	.	.	.
11	85127110	G	A	intronic	*DLG2*	.	.	.
11	85129716	C	A	intronic	*DLG2*	.	.	.
11	85139077	C	G	intronic	*DLG2*	.	.	.
11	85145297	G	A	intronic	*DLG2*	.	.	.
11	85179685	T	C	intronic	*DLG2*	.	.	.
11	85216215	A	G	intronic	*DLG2*	.	.	.

11	85255277	C	T	intronic	*DLG2*	.	.	.
11	85255959	C	T	intronic	*DLG2*	.	.	.
11	85302523	G	A	intronic	*DLG2*	.	.	.
X	69737690	G	T	intergenic	*DLG3,TEX11*	.	.	.
X	69738230	G	A	intergenic	*DLG3,TEX11*	.	.	.
X	69738810	T	G	intergenic	*DLG3,TEX11*	.	.	.
18	4359030	G	C	intronic	*DLGAP1*	.	.	.
18	4413309	A	C	intronic	*DLGAP1*	.	.	.
18	3614650	A	G	intronic	*DLGAP1*	.	.	.
18	3697083	G	C	intronic	*DLGAP1*	.	.	.
18	3697085	C	CT	intronic	*DLGAP1*	.	.	.
18	3698486	C	T	intronic	*DLGAP1*	.	.	.
18	3737948	A	G	intronic	*DLGAP1*	.	.	.
18	3945749	A	T	intronic	*DLGAP1*	.	.	.
18	4090317	G	A	intronic	*DLGAP1*	.	.	.
18	3893947	A	G	ncRNA_intronic	*DLGAP1-AS3*	.	.	.
18	4009963	G	T	ncRNA_intronic	*DLGAP1-AS4*	.	.	.
18	4287354	A	G	ncRNA_intronic	*DLGAP1-AS5*	.	.	.
18	4807756	A	G	intergenic	*DLGAP1,C18orf42*	.	.	.
18	4786664	C	G	intergenic	*DLGAP1,C18orf42*	.	.	.
18	4594270	G	T	intergenic	*DLGAP1,C18orf42*	.	.	.
18	4617216	C	G	intergenic	*DLGAP1,C18orf42*	.	.	.
18	4697478	G	A	intergenic	*DLGAP1,C18orf42*	.	.	.
18	4699719	T	A	intergenic	*DLGAP1,C18orf42*	.	.	.
18	4700609	T	C	intergenic	*DLGAP1,C18orf42*	.	.	.
18	4895032	C	T	intergenic	*DLGAP1,C18orf42*	.	.	.
18	5126911	A	G	intergenic	*DLGAP1,C18orf42*	.	.	.
18	5126913	T	G	intergenic	*DLGAP1,C18orf42*	.	.	.
18	5127396	T	C	intergenic	*DLGAP1,C18orf42*	.	.	.
8	1578768	C	T	intronic	*DLGAP2*	.	.	.
8	1544774	G	T	ncRNA_intronic	*DLGAP2-AS1*	.	.	.
8	1545714	T	C	ncRNA_intronic	*DLGAP2-AS1*	.	.	.
8	1545715	G	A	ncRNA_intronic	*DLGAP2-AS1*	.	.	.
8	1557761	G	A	ncRNA_intronic	*DLGAP2-AS1*	.	.	.
8	1558803	G	A	ncRNA_intronic	*DLGAP2-AS1*	.	.	.
1	35377789	T	C	intronic	*DLGAP3*	.	.	.
20	34955392	A	T	intronic	*DLGAP4*	.	.	.
14	55705137	G	A	intergenic	*DLGAP5,FBXO34*	.	.	.

2	173133453	T	G	intergenic	*DLX2-AS1,ITGA6*	.	.	.
7	96656531	G	C	intergenic	*DLX5,SDHAF3*	.	.	.
7	96722842	G	A	intergenic	*DLX5,SDHAF3*	.	.	.
7	96683868	C	T	intergenic	*DLX5,SDHAF3*	.	.	.
7	96683869	A	G	intergenic	*DLX5,SDHAF3*	.	.	.
7	96685564	T	G	intergenic	*DLX5,SDHAF3*	.	.	.
7	96685566	C	T	intergenic	*DLX5,SDHAF3*	.	.	.
7	96690981	G	A	intergenic	*DLX5,SDHAF3*	.	.	.
7	96692310	A	G	intergenic	*DLX5,SDHAF3*	.	.	.
7	96629171	TGTGC	T	ncRNA_intronic	*DLX6-AS1*	.	.	.
10	124389858	A	G	exonic	*DMBT1*	synonymous_SNV	.	.
10	124570160	A	G	intergenic	*DMBT1P1,CUZD1*	.	.	.
X	32522751	A	C	intronic	*DMD*	.	.	.
X	31168389	T	A	intronic	*DMD*	.	.	.
X	31440119	C	CAT	intronic	*DMD*	.	.	.
X	31710272	C	G	intronic	*DMD*	.	.	.
X	31775382	G	A	intronic	*DMD*	.	.	.
X	31949726	T	A	intronic	*DMD*	.	.	.
X	32046911	A	G	intronic	*DMD*	.	.	.
X	32208056	T	C	intronic	*DMD*	.	.	.
X	32334205	T	A	intronic	*DMD*	.	.	.
X	32985011	G	A	intronic	*DMD*	.	.	.
X	33206617	GA	G	intronic	*DMD*	.	.	.
X	33254444	C	A	intronic	*DMD*	.	.	.
X	33089850	T	C	intronic	*DMD*	.	.	.
X	32371362	T	C	intronic	*DMD*	.	.	.
X	33181669	GTATA	G	intronic	*DMD*	.	.	.
X	32109835	A	T	intronic	*DMD*	.	.	.
X	32578312	A	AATAT	intronic	*DMD*	.	.	.
X	32588544	A	T	intronic	*DMD*	.	.	.
X	33153177	G	A	intronic	*DMD*	.	.	.
X	33198931	G	T	intronic	*DMD*	.	.	.
X	31484305	T	C	intronic	*DMD*	.	.	.
X	31708166	G	A	intronic	*DMD*	.	.	.
X	31803952	G	T	intronic	*DMD*	.	.	.
X	31803953	T	A	intronic	*DMD*	.	.	.
X	31830402	T	G	intronic	*DMD*	.	.	.
X	32294855	C	G	intronic	*DMD*	.	.	.

X	32294856	A	G	intronic	*DMD*	.	.	.
X	32296644	C	T	intronic	*DMD*	.	.	.
X	32296645	G	T	intronic	*DMD*	.	.	.
X	32330908	C	A	intronic	*DMD*	.	.	.
X	32425122	T	C	intronic	*DMD*	.	.	.
X	32452580	T	TAGATCG	intronic	*DMD*	.	.	.
X	32505046	G	A	intronic	*DMD*	.	.	.
X	32505188	A	G	intronic	*DMD*	.	.	.
X	32672114	A	AT	intronic	*DMD*	.	.	.
X	32674044	C	T	intronic	*DMD*	.	.	.
X	32751526	G	A	intronic	*DMD*	.	.	.
X	32751715	A	G	intronic	*DMD*	.	.	.
X	32820273	T	C	intronic	*DMD*	.	.	.
X	32821337	T	A	intronic	*DMD*	.	.	.
X	32824943	T	C	intronic	*DMD*	.	.	.
X	32982774	G	A	intronic	*DMD*	.	.	.
X	33177751	T	C	intronic	*DMD*	.	.	.
X	33177752	G	A	intronic	*DMD*	.	.	.
X	34070926	C	T	intergenic	*DMD,FAM47A*	.	.	.
X	33789936	C	G	intergenic	*DMD,FAM47A*	.	.	.
X	33550726	C	A	intergenic	*DMD,FAM47A*	.	.	.
X	33628126	C	A	intergenic	*DMD,FAM47A*	.	.	.
X	33731129	CATAT	C	intergenic	*DMD,FAM47A*	.	.	.
X	33700967	G	T	intergenic	*DMD,FAM47A*	.	.	.
X	33718354	C	A	intergenic	*DMD,FAM47A*	.	.	.
X	34084260	A	G	intergenic	*DMD,FAM47A*	.	.	.
X	33411648	G	A	intergenic	*DMD,FAM47A*	.	.	.
X	33459905	G	A	intergenic	*DMD,FAM47A*	.	.	.
X	33460206	C	T	intergenic	*DMD,FAM47A*	.	.	.
X	33473035	A	G	intergenic	*DMD,FAM47A*	.	.	.
X	33473036	A	G	intergenic	*DMD,FAM47A*	.	.	.
X	33526087	G	A	intergenic	*DMD,FAM47A*	.	.	.
X	33654200	C	T	intergenic	*DMD,FAM47A*	.	.	.
X	33658550	T	C	intergenic	*DMD,FAM47A*	.	.	.
X	33770529	C	T	intergenic	*DMD,FAM47A*	.	.	.
X	33770530	A	G	intergenic	*DMD,FAM47A*	.	.	.
X	33921721	T	C	intergenic	*DMD,FAM47A*	.	.	.
4	88719327	T	C	intergenic	*DMP1,IBSP*	.	.	.

9	1420694	G	A	intergenic	*DMRT2,SMARCA2*	.	.	.
9	1110316	T	A	intergenic	*DMRT2,SMARCA2*	.	.	.
9	1066859	C	T	intergenic	*DMRT2,SMARCA2*	.	.	.
9	1066860	A	G	intergenic	*DMRT2,SMARCA2*	.	.	.
9	1175341	C	CAAAAAAAA	intergenic	*DMRT2,SMARCA2*	.	.	.
9	1229349	G	A	intergenic	*DMRT2,SMARCA2*	.	.	.
9	1398112	G	A	intergenic	*DMRT2,SMARCA2*	.	.	.
9	1779495	G	T	intergenic	*DMRT2,SMARCA2*	.	.	.
9	1780223	C	T	intergenic	*DMRT2,SMARCA2*	.	.	.
9	1821216	C	T	intergenic	*DMRT2,SMARCA2*	.	.	.
9	1862357	G	A	intergenic	*DMRT2,SMARCA2*	.	.	.
9	1864442	G	A	intergenic	*DMRT2,SMARCA2*	.	.	.
9	985717	A	G	intronic	*DMRT3*	.	.	.
5	118452836	G	A	intronic	*DMXL1*	.	.	.
15	51761156	AAGAG	A	intronic	*DMXL2*	.	.	.
15	51863421	G	A	intronic	*DMXL2*	.	.	.
3	52357005	G	A	intronic	*DNAH1*	.	.	.
7	21618710	C	A	intronic	*DNAH11*	.	.	.
7	21878500	C	A	intronic	*DNAH11*	.	.	.
7	21894423	A	G	intronic	*DNAH11*	.	.	.
7	21610765	C	T	intronic	*DNAH11*	.	.	.
7	21622673	C	A	intronic	*DNAH11*	.	.	.
7	21662104	C	A	intronic	*DNAH11*	.	.	.
7	21678309	GGTGTGTG TGT	G	intronic	*DNAH11*	.	.	.
1	225514033	T	C	intronic	*DNAH14*	.	.	.
1	225244350	G	T	intronic	*DNAH14*	.	.	.
1	225353256	T	C	intronic	*DNAH14*	.	.	.
1	225471429	A	G	intronic	*DNAH14*	.	.	.
1	225183537	T	TA	intronic	*DNAH14*	.	.	.
1	225129263	C	T	intronic	*DNAH14*	.	.	.
1	225129264	A	G	intronic	*DNAH14*	.	.	.
1	225131495	G	A	intronic	*DNAH14*	.	.	.
1	225164169	G	A	intronic	*DNAH14*	.	.	.
1	225195960	T	C	intronic	*DNAH14*	.	.	.
1	225227901	G	A	intronic	*DNAH14*	.	.	.
1	225292744	G	T	intronic	*DNAH14*	.	.	.
1	225293525	T	G	intronic	*DNAH14*	.	.	.

1	225358930	T	A	intronic	*DNAH14*	.	.	.
1	225402364	T	C	intronic	*DNAH14*	.	.	.
1	225403038	A	C	intronic	*DNAH14*	.	.	.
1	225408179	C	T	intronic	*DNAH14*	.	.	.
1	225423522	G	T	intronic	*DNAH14*	.	.	.
1	225423524	T	G	intronic	*DNAH14*	.	.	.
1	225431906	A	C	intronic	*DNAH14*	.	.	.
1	225500991	G	T	intronic	*DNAH14*	.	.	.
1	225571916	C	T	intronic	*DNAH14*	.	.	.
5	13747743	T	C	intronic	*DNAH5*	.	.	.
5	13748046	T	A	intronic	*DNAH5*	.	.	.
5	13796344	G	A	intronic	*DNAH5*	.	.	.
2	84873516	A	C	intronic	*DNAH6*	.	.	.
2	84787974	C	A	intronic	*DNAH6*	.	.	.
2	84890965	T	C	intronic	*DNAH6*	.	.	.
2	84893385	T	G	intronic	*DNAH6*	.	.	.
2	196793627	T	C	intronic	*DNAH7*	.	.	.
2	196812718	C	T	intronic	*DNAH7*	.	.	.
2	196812719	A	G	intronic	*DNAH7*	.	.	.
2	196820020	T	G	intronic	*DNAH7*	.	.	.
2	196957151	A	AAAG	intergenic	*DNAH7,STK17B*	.	.	.
2	196958227	C	A	intergenic	*DNAH7,STK17B*	.	.	.
6	38724316	A	T	intronic	*DNAH8*	.	.	.
6	38725497	T	G	intronic	*DNAH8*	.	.	.
17	11659697	C	T	intronic	*DNAH9*	.	.	.
17	11806066	C	T	exonic	*DNAH9*	synonymous_SNV	.	.
17	11727610	G	T	intronic	*DNAH9*	.	.	.
17	11749193	G	A	intronic	*DNAH9*	.	.	.

17	72298005	GGAAGGAA GGAAGGAA GGAAGGAA GGAAGGAA GGAAGGAA GGAAAGAA GGAAGGAA GGAAGGAA GGAAGGAA GGAAGGAA GGAAGGAA GGAAGGAA GGAAAGAA GGAAGGAA GGAAGGAA GGAAGGAA GGAAGGAA GGAAGGAA GGAAAGAA GGAAGGAA AGAAGGAA GGAAGGAA GGAAGGAA GGAAGGAA GGAAGGAA GGAAA	G	intronic	*DNAI2*	.	.	.
17	72312232	T	C	intergenic	*DNAI2,KIF19*	.	.	.
7	157242956	C	T	intergenic	*DNAJB6,LOC101927914*	.	.	.
7	108277051	AACACAC	A	intergenic	*DNAJB9,C7orf66*	.	.	.
7	108235744	C	T	intergenic	*DNAJB9,C7orf66*	.	.	.
7	108256006	C	T	intergenic	*DNAJB9,C7orf66*	.	.	.
7	108281638	A	C	intergenic	*DNAJB9,C7orf66*	.	.	.
7	108369307	A	AT	intergenic	*DNAJB9,C7orf66*	.	.	.
7	108370192	T	G	intergenic	*DNAJB9,C7orf66*	.	.	.

7	108400740	C	G	intergenic	*DNAJB9,C7orf66*	.	.	.
7	108400742	T	C	intergenic	*DNAJB9,C7orf66*	.	.	.
7	108427313	C	T	intergenic	*DNAJB9,C7orf66*	.	.	.
10	22072403	A	G	intronic	*DNAJC1*	.	.	.
10	22129627	G	A	intronic	*DNAJC1*	.	.	.
10	22366263	G	C	intergenic	*DNAJC1,EBLN1*	.	.	.
2	183634087	A	C	intronic	*DNAJC10*	.	.	.
2	183634567	T	A	intronic	*DNAJC10*	.	.	.
2	183636237	C	T	intronic	*DNAJC10*	.	.	.
2	183637312	C	CT	intronic	*DNAJC10*	.	.	.
2	183647153	A	C	intergenic	*DNAJC10,FRZB*	.	.	.
2	183647489	A	G	intergenic	*DNAJC10,FRZB*	.	.	.
11	31422453	T	C	intronic	*DNAJC24*	.	.	.
2	25222824	C	T	ncRNA_intronic	*DNAJC27-AS1*	.	.	.
21	34864312	A	G	upstream	*DNAJC28*	.	.	.
21	34873268	A	C	intergenic	*DNAJC28,GART*	.	.	.
13	96378705	T	C	intronic	*DNAJC3*	.	.	.
13	96389991	T	C	intronic	*DNAJC3*	.	.	.
13	96391992	C	T	intronic	*DNAJC3*	.	.	.
8	66940318	C	T	intronic	*DNAJC5B*	.	.	.
8	66954900	A	C	intronic	*DNAJC5B*	.	.	.
8	66984113	A	G	intronic	*DNAJC5B*	.	.	.
2	230262179	C	CAAAAAAAA	intronic	*DNER*	.	.	.
2	230262179	C	CAAAAAAAA	intronic	*DNER*	.	.	.
2	230430887	G	T	intronic	*DNER*	.	.	.
2	230543440	T	A	intronic	*DNER*	.	.	.
2	230249072	G	A	intronic	*DNER*	.	.	.
2	230249960	C	T	intronic	*DNER*	.	.	.
2	230249961	A	G	intronic	*DNER*	.	.	.
12	32869634	TTATATATA TACTATATA TAGAGTAT ATATACACT ATATATAGT ATATATATA	T	intronic	*DNM1L*	.	.	.
15	100351092	ATGTG	A	intergenic	*DNM1P46,ADAMTS 17*	.	.	.

19	10844137	G	C	intronic	*DNM2*	.	.	.
19	10844138	G	T	intronic	*DNM2*	.	.	.
1	172006085	A	G	intronic	*DNM3*	.	.	.
1	171995951	G	A	intronic	*DNM3*	.	.	.
1	172160098	C	T	intronic	*DNM3*	.	.	.
1	172160540	C	G	intronic	*DNM3*	.	.	.
1	172286091	G	A	intronic	*DNM3*	.	.	.
1	172287145	T	C	intronic	*DNM3*	.	.	.
1	172288617	T	A	intronic	*DNM3*	.	.	.
1	172369372	C	A	intronic	*DNM3*	.	.	.
10	101766895	T	G	intronic	*DNMBP*	.	.	.
19	10326698	G	A	intergenic	*DNMT1,S1PR2*	.	.	.
10	129050305	A	G	intronic	*DOCK1*	.	.	.
10	129209915	A	G	intronic	*DOCK1*	.	.	.
10	128941170	T	C	intronic	*DOCK1,FAM196A*	.	.	.
2	225881315	CACATAGA TACATATAT CTATGTGC ACATAGAT ACATATATC TATGTGCA CATAGAT	C	intronic	*DOCK10*	.	.	.
2	225776602	G	A	intronic	*DOCK10*	.	.	.
2	225992565	T	C	intergenic	*DOCK10,NYAP2*	.	.	.
2	226156335	C	T	intergenic	*DOCK10,NYAP2*	.	.	.
2	226196554	T	A	intergenic	*DOCK10,NYAP2*	.	.	.
X	117695772	G	T	intronic	*DOCK11*	.	.	.
5	169481436	A	G	intronic	*DOCK2*	.	.	.
5	169248755	T	C	intronic	*DOCK2*	.	.	.
5	169249727	A	G	intronic	*DOCK2*	.	.	.
5	169251357	T	C	intronic	*DOCK2*	.	.	.
5	169251358	G	A	intronic	*DOCK2*	.	.	.
5	169464717	C	T	intronic	*DOCK2*	.	.	.
5	169464718	A	G	intronic	*DOCK2*	.	.	.
5	169323165	A	C	intronic	*DOCK2,FAM196B*	.	.	.
5	169316937	G	A	intronic	*DOCK2,FAM196B*	.	.	.
3	51183818	G	C	intronic	*DOCK3*	.	.	.

3	51326334	GGTGT	G	intronic	*DOCK3*	.	.	.
3	50781091	T	C	intronic	*DOCK3*	.	.	.
3	51361203	A	G	intronic	*DOCK3*	.	.	.
3	51401974	T	A	intronic	*DOCK3*	.	.	.
3	51404146	T	C	intronic	*DOCK3*	.	.	.
7	111764023	G	T	intronic	*DOCK4*	.	.	.
7	111766632	A	G	intronic	*DOCK4*	.	.	.
8	25100453	G	C	intronic	*DOCK5*	.	.	.
8	25122199	T	C	intronic	*DOCK5*	.	.	.
13	99478962	G	A	intronic	*DOCK9*	.	.	.
13	99797206	G	A	intergenic	*DOCK9-AS2,UBAC2-AS1*	.	.	.
13	99820020	G	A	intergenic	*DOCK9-AS2,UBAC2-AS1*	.	.	.
20	53160846	A	G	intronic	*DOK5*	.	.	.
20	53256965	G	A	intronic	*DOK5*	.	.	.
20	53515376	C	A	intergenic	*DOK5,LINC01441*	.	.	.
20	53973863	C	T	intergenic	*DOK5,LINC01441*	.	.	.
20	53650281	T	C	intergenic	*DOK5,LINC01441*	.	.	.
20	53448196	G	T	intergenic	*DOK5,LINC01441*	.	.	.
20	53448197	C	T	intergenic	*DOK5,LINC01441*	.	.	.
20	53718435	G	C	intergenic	*DOK5,LINC01441*	.	.	.
20	53779417	G	A	intergenic	*DOK5,LINC01441*	.	.	.
20	53587295	A	G	intergenic	*DOK5,LINC01441*	.	.	.
20	53970056	A	T	intergenic	*DOK5,LINC01441*	.	.	.
20	53574842	G	T	intergenic	*DOK5,LINC01441*	.	.	.
20	53492402	T	C	intergenic	*DOK5,LINC01441*	.	.	.
20	53499416	A	G	intergenic	*DOK5,LINC01441*	.	.	.
20	53500221	A	G	intergenic	*DOK5,LINC01441*	.	.	.
20	53500607	T	C	intergenic	*DOK5,LINC01441*	.	.	.
20	53501959	A	G	intergenic	*DOK5,LINC01441*	.	.	.
20	53502394	C	A	intergenic	*DOK5,LINC01441*	.	.	.
20	53517420	T	A	intergenic	*DOK5,LINC01441*	.	.	.
20	53519185	A	C	intergenic	*DOK5,LINC01441*	.	.	.
20	53591228	T	C	intergenic	*DOK5,LINC01441*	.	.	.
20	53797387	T	TAAAAA	intergenic	*DOK5,LINC01441*	.	.	.
20	53839494	A	G	intergenic	*DOK5,LINC01441*	.	.	.
20	53857823	T	C	intergenic	*DOK5,LINC01441*	.	.	.

20	53924597	G	C	intergenic	*DOK5,LINC01441*	.	.	.
20	53926948	T	C	intergenic	*DOK5,LINC01441*	.	.	.
20	53973068	T	C	intergenic	*DOK5,LINC01441*	.	.	.
20	53973221	G	A	intergenic	*DOK5,LINC01441*	.	.	.
18	67120202	A	C	intronic	*DOK6*	.	.	.
18	67114069	C	T	intronic	*DOK6*	.	.	.
18	67114071	G	T	intronic	*DOK6*	.	.	.
18	67114400	A	C	intronic	*DOK6*	.	.	.
18	67230111	A	G	intronic	*DOK6*	.	.	.
19	2203024	G	A	intronic	*DOT1L*	.	.	.
6	30930437	C	G	intergenic	*DPCR1,MUC21*	.	.	.
11	65113845	C	T	intronic	*DPF2*	.	.	.
14	73339718	A	G	intronic	*DPF3*	.	.	.
15	35681006	C	T	intronic	*DPH6*	.	.	.
15	35732996	C	A	intronic	*DPH6*	.	.	.
2	115926483	A	T	intronic	*DPP10*	.	.	.
2	115458444	G	T	intronic	*DPP10*	.	.	.
2	115514410	T	A	intronic	*DPP10*	.	.	.
2	116285928	G	C	intronic	*DPP10*	.	.	.
2	116560715	G	A	intronic	*DPP10*	.	.	.
2	115443957	CA	C	intronic	*DPP10*	.	.	.
2	115803603	G	A	intronic	*DPP10*	.	.	.
2	116329421	TTGTG	T	intronic	*DPP10*	.	.	.
2	115247982	G	A	intronic	*DPP10*	.	.	.
2	115304604	T	C	intronic	*DPP10*	.	.	.
2	115824483	A	AT	intronic	*DPP10*	.	.	.
2	115835964	A	T	intronic	*DPP10*	.	.	.
2	116050975	A	T	intronic	*DPP10*	.	.	.
2	116112892	G	A	intronic	*DPP10*	.	.	.
2	115371324	T	A	intronic	*DPP10*	.	.	.
2	115449010	A	T	intronic	*DPP10*	.	.	.
2	115762083	T	A	intronic	*DPP10*	.	.	.
2	116013703	A	C	intronic	*DPP10*	.	.	.
2	116013704	G	A	intronic	*DPP10*	.	.	.
2	116088377	G	C	intronic	*DPP10*	.	.	.
2	116090683	G	C	intronic	*DPP10*	.	.	.
2	116293498	G	A	intronic	*DPP10*	.	.	.
2	116348182	C	T	intronic	*DPP10*	.	.	.

2	116348238	C	T	intronic	*DPP10*	.	.	.
2	116348939	G	T	intronic	*DPP10*	.	.	.
2	116349345	A	G	intronic	*DPP10*	.	.	.
2	116547901	C	T	intronic	*DPP10*	.	.	.
2	115902338	T	C	ncRNA_intronic	*DPP10-AS1*	.	.	.
2	116955147	T	C	intergenic	*DPP10,DDX18*	.	.	.
2	118393596	G	T	intergenic	*DPP10,DDX18*	.	.	.
2	117352379	G	T	intergenic	*DPP10,DDX18*	.	.	.
2	117472345	G	T	intergenic	*DPP10,DDX18*	.	.	.
2	117669476	G	GA	intergenic	*DPP10,DDX18*	.	.	.
2	118444297	C	T	intergenic	*DPP10,DDX18*	.	.	.
2	117302466	C	A	intergenic	*DPP10,DDX18*	.	.	.
2	117532693	T	C	intergenic	*DPP10,DDX18*	.	.	.
2	116685208	AT	A	intergenic	*DPP10,DDX18*	.	.	.
2	116763391	CA	C	intergenic	*DPP10,DDX18*	.	.	.
2	116919024	G	T	intergenic	*DPP10,DDX18*	.	.	.
2	117126497	C	T	intergenic	*DPP10,DDX18*	.	.	.
2	117159896	A	AAT	intergenic	*DPP10,DDX18*	.	.	.
2	117207217	G	A	intergenic	*DPP10,DDX18*	.	.	.
2	117394137	C	A	intergenic	*DPP10,DDX18*	.	.	.
2	117453079	A	G	intergenic	*DPP10,DDX18*	.	.	.
2	117467836	G	T	intergenic	*DPP10,DDX18*	.	.	.
2	118430408	C	G	intergenic	*DPP10,DDX18*	.	.	.
2	117034405	C	T	intergenic	*DPP10,DDX18*	.	.	.
2	118144253	T	C	intergenic	*DPP10,DDX18*	.	.	.
2	118480230	G	A	intergenic	*DPP10,DDX18*	.	.	.
2	116633273	G	A	intergenic	*DPP10,DDX18*	.	.	.
2	116650149	A	G	intergenic	*DPP10,DDX18*	.	.	.
2	116718241	C	T	intergenic	*DPP10,DDX18*	.	.	.
2	116718242	A	G	intergenic	*DPP10,DDX18*	.	.	.
2	116719816	C	G	intergenic	*DPP10,DDX18*	.	.	.
2	116721078	T	G	intergenic	*DPP10,DDX18*	.	.	.
2	116794000	A	C	intergenic	*DPP10,DDX18*	.	.	.
2	116795954	G	A	intergenic	*DPP10,DDX18*	.	.	.
2	116847569	T	C	intergenic	*DPP10,DDX18*	.	.	.
2	116859993	G	A	intergenic	*DPP10,DDX18*	.	.	.
2	116860886	C	T	intergenic	*DPP10,DDX18*	.	.	.
2	116861157	T	C	intergenic	*DPP10,DDX18*	.	.	.

2	116880350	T	C	intergenic	*DPP10,DDX18*	.	.	.
2	116892423	A	G	intergenic	*DPP10,DDX18*	.	.	.
2	116896919	A	G	intergenic	*DPP10,DDX18*	.	.	.
2	116992982	C	T	intergenic	*DPP10,DDX18*	.	.	.
2	116993806	G	A	intergenic	*DPP10,DDX18*	.	.	.
2	117092650	T	A	intergenic	*DPP10,DDX18*	.	.	.
2	117093176	C	T	intergenic	*DPP10,DDX18*	.	.	.
2	117117355	C	G	intergenic	*DPP10,DDX18*	.	.	.
2	117120294	C	T	intergenic	*DPP10,DDX18*	.	.	.
2	117125916	C	T	intergenic	*DPP10,DDX18*	.	.	.
2	117126073	A	T	intergenic	*DPP10,DDX18*	.	.	.
2	117186104	C	T	intergenic	*DPP10,DDX18*	.	.	.
2	117186105	A	G	intergenic	*DPP10,DDX18*	.	.	.
2	117314889	A	G	intergenic	*DPP10,DDX18*	.	.	.
2	117360715	A	G	intergenic	*DPP10,DDX18*	.	.	.
2	117469513	A	T	intergenic	*DPP10,DDX18*	.	.	.
2	117499806	C	T	intergenic	*DPP10,DDX18*	.	.	.
2	117627562	A	G	intergenic	*DPP10,DDX18*	.	.	.
2	117627564	T	C	intergenic	*DPP10,DDX18*	.	.	.
2	117688682	G	A	intergenic	*DPP10,DDX18*	.	.	.
2	117741891	T	C	intergenic	*DPP10,DDX18*	.	.	.
2	117761095	A	AG	intergenic	*DPP10,DDX18*	.	.	.
2	117763445	C	T	intergenic	*DPP10,DDX18*	.	.	.
2	117916508	A	C	intergenic	*DPP10,DDX18*	.	.	.
2	117919262	C	T	intergenic	*DPP10,DDX18*	.	.	.
2	117992052	G	A	intergenic	*DPP10,DDX18*	.	.	.
2	118163524	T	C	intergenic	*DPP10,DDX18*	.	.	.
2	118163525	G	A	intergenic	*DPP10,DDX18*	.	.	.
2	118323050	T	C	intergenic	*DPP10,DDX18*	.	.	.
2	118325106	A	G	intergenic	*DPP10,DDX18*	.	.	.
2	118358173	C	T	intergenic	*DPP10,DDX18*	.	.	.
2	118383337	G	A	intergenic	*DPP10,DDX18*	.	.	.
2	118447464	G	A	intergenic	*DPP10,DDX18*	.	.	.
2	118566057	C	T	intergenic	*DPP10,DDX18*	.	.	.
2	162955549	T	C	intergenic	*DPP4,LOC101929532*	.	.	.
7	153815929	G	C	intronic	*DPP6*	.	.	.
7	154509091	AACAC	A	intronic	*DPP6*	.	.	.

7	153759895	A	G	intronic	*DPP6*	.	.	.
7	153759896	G	A	intronic	*DPP6*	.	.	.
7	154219429	G	C	intronic	*DPP6*	.	.	.
7	154540544	C	A	intronic	*DPP6*	.	.	.
7	153670791	G	T	intronic	*DPP6*	.	.	.
7	154394902	G	A	intronic	*DPP6*	.	.	.
7	153961817	C	T	intronic	*DPP6*	.	.	.
7	153961818	A	G	intronic	*DPP6*	.	.	.
7	153760489	C	T	intronic	*DPP6*	.	.	.
7	154026942	A	G	intronic	*DPP6*	.	.	.
7	154159782	C	T	intronic	*DPP6*	.	.	.
7	154162611	T	C	intronic	*DPP6*	.	.	.
7	154162613	T	C	intronic	*DPP6*	.	.	.
7	154461248	TTGTGTGTGTGTG	T	intronic	*DPP6*	.	.	.
19	4676741	C	A	intronic	*DPP9*	.	.	.
19	4733532	T	C	intergenic	*DPP9,MIR7-3HG*	.	.	.
3	109124501	A	G	intergenic	*DPPA4,LINC01205*	.	.	.
1	168714534	T	C	intergenic	*DPT,LINC00626*	.	.	.
1	168746469	G	A	intergenic	*DPT,LINC00626*	.	.	.
7	32640060	A	G	ncRNA_intronic	*DPY19L1P1*	.	.	.
7	32644226	G	A	ncRNA_intronic	*DPY19L1P1*	.	.	.
7	32737981	A	T	ncRNA_intronic	*DPY19L1P1*	.	.	.
7	32753379	A	T	ncRNA_intronic	*DPY19L1P1*	.	.	.
12	64025175	A	C	intronic	*DPY19L2*	.	.	.
12	64025177	T	A	intronic	*DPY19L2*	.	.	.
12	64026131	G	A	intronic	*DPY19L2*	.	.	.
12	64026265	G	A	intronic	*DPY19L2*	.	.	.
12	64033489	C	T	intronic	*DPY19L2*	.	.	.
12	64067596	T	C	intergenic	*DPY19L2,TMEM5*	.	.	.
12	64113771	T	C	intergenic	*DPY19L2,TMEM5*	.	.	.
7	35194212	A	T	ncRNA_intronic	*DPY19L2P1*	.	.	.
7	102890482	T	C	ncRNA_intronic	*DPY19L2P2*	.	.	.
7	102898333	G	A	ncRNA_intronic	*DPY19L2P2*	.	.	.
7	102922650	G	T	intergenic	*DPY19L2P2,PMPCB*	.	.	.
7	29725722	C	T	ncRNA_intronic	*DPY19L2P3*	.	.	.
19	32901594	T	C	intronic	*DPY19L3*	.	.	.

19	32901595	G	A	intronic	*DPY19L3*	.	.	.
1	98242844	A	G	intronic	*DPYD*	.	.	.
1	98155647	T	C	intronic	*DPYD*	.	.	.
1	97695779	G	T	ncRNA_intronic	*DPYD-AS1*	.	.	.
1	97732490	A	G	ncRNA_intronic	*DPYD-AS1*	.	.	.
1	97754399	C	G	ncRNA_intronic	*DPYD-AS1*	.	.	.
8	105469848	C	A	intronic	*DPYS*	.	.	.
8	26492328	C	T	exonic	*DPYSL2*	synonymous_SNV	.	.
8	26534289	A	T	intergenic	*DPYSL2,ADRA1A*	.	.	.
12	102390335	T	C	intergenic	*DRAM1,CCDC53*	.	.	.
11	113406328	G	A	intergenic	*DRD2,TMPRSS5*	.	.	.
11	113530612	C	T	intergenic	*DRD2,TMPRSS5*	.	.	.
11	113530613	A	G	intergenic	*DRD2,TMPRSS5*	.	.	.
11	113533398	G	A	intergenic	*DRD2,TMPRSS5*	.	.	.
10	50653855	G	A	intergenic	*DRGX,ERCC6*	.	.	.
10	50612760	C	T	intergenic	*DRGX,ERCC6*	.	.	.
10	50629082	A	T	intergenic	*DRGX,ERCC6*	.	.	.
10	50631349	C	G	intergenic	*DRGX,ERCC6*	.	.	.
18	28762386	C	G	intergenic	*DSC1,DSG1*	.	.	.
18	28876374	AGC	A	intergenic	*DSC1,DSG1*	.	.	.
18	28876376	C	CAAT	intergenic	*DSC1,DSG1*	.	.	.
18	28865267	G	T	intergenic	*DSC1,DSG1*	.	.	.
21	41398767	T	A	intronic	*DSCAM*	.	.	.
21	41466378	G	T	intronic	*DSCAM*	.	.	.
21	42158548	T	A	intronic	*DSCAM*	.	.	.
21	42126764	G	T	intronic	*DSCAM*	.	.	.
21	41466862	T	A	intronic	*DSCAM*	.	.	.
21	41887146	T	C	intronic	*DSCAM*	.	.	.
21	42015678	C	CAG	intronic	*DSCAM*	.	.	.
21	41479574	A	T	intronic	*DSCAM*	.	.	.
21	41479575	T	A	intronic	*DSCAM*	.	.	.
21	41484710	C	A	intronic	*DSCAM*	.	.	.
21	41591018	C	A	intronic	*DSCAM*	.	.	.
21	41689950	T	C	intronic	*DSCAM*	.	.	.
21	41722188	T	G	intronic	*DSCAM*	.	.	.
21	42193319	GCACACACACACA	G	intronic	*DSCAM*	.	.	.

21	41992053	GAGAGAGAAAGAGAGAGAAAAAAGAAAAAGAAAGAAAGAGAGAGAGAAAGAGAGAGAAAAAAGAAAAAGAAAGAA	G	ncRNA_intronic	*DSCAM-IT1*	.	.	.
21	42439097	CAT	C	intergenic	*DSCAM,LINC00323*	.	.	.
21	42489847	C	T	intergenic	*DSCAM,LINC00323*	.	.	.
21	42489848	A	G	intergenic	*DSCAM,LINC00323*	.	.	.
11	117305605	C	A	intronic	*DSCAML1*	.	.	.
21	38604363	T	C	intronic	*DSCR3*	.	.	.
21	39457930	G	C	intronic	*DSCR4*	.	.	.
6	116666562	G	A	intronic	*DSE*	.	.	.
6	116666855	G	C	intronic	*DSE*	.	.	.
6	116682380	G	A	intronic	*DSE*	.	.	.
18	29087857	A	G	intronic	*DSG2*	.	.	.
18	29087859	G	A	intronic	*DSG2*	.	.	.
6	56428806	A	G	intronic	*DST*	.	.	.
6	56448620	T	C	intronic	*DST*	.	.	.
6	56448621	G	A	intronic	*DST*	.	.	.
6	56645937	T	A	intronic	*DST*	.	.	.
1	205165059	G	A	intronic	*DSTYK*	.	.	.
20	18587538	A	T	intronic	*DTD1*	.	.	.
14	31929632	G	C	intergenic	*DTD2,GPR33*	.	.	.
4	36350945	C	T	intergenic	*DTHD1,MIR1255B1*	.	.	.
4	36384226	C	G	intergenic	*DTHD1,MIR1255B1*	.	.	.
4	36376346	C	G	intergenic	*DTHD1,MIR1255B1*	.	.	.

4	36382051	G	A	intergenic	*DTHD1,MIR1255B1*	.	.	.
4	36382616	T	A	intergenic	*DTHD1,MIR1255B1*	.	.	.
18	32182912	A	C	intronic	*DTNA*	.	.	.
18	32081293	T	C	intronic	*DTNA*	.	.	.
18	32244963	A	T	intronic	*DTNA*	.	.	.
18	32329631	TAATAATAA TAAC	T	intronic	*DTNA*	.	.	.
18	32115431	A	C	intronic	*DTNA*	.	.	.
18	32115651	G	A	intronic	*DTNA*	.	.	.
18	32133697	A	G	intronic	*DTNA*	.	.	.
18	32245908	A	G	intronic	*DTNA*	.	.	.
18	32267814	C	G	intronic	*DTNA*	.	.	.
18	32304776	A	C	intronic	*DTNA*	.	.	.
18	32479947	T	C	intergenic	*DTNA,MAPRE2*	.	.	.
2	25914543	C	T	intergenic	*DTNB,ASXL2*	.	.	.
6	15645784	A	T	intronic	*DTNBP1*	.	.	.
6	15942392	T	C	intergenic	*DTNBP1,MYLIP*	.	.	.
6	16103516	A	T	intergenic	*DTNBP1,MYLIP*	.	.	.
5	118259700	G	C	intronic	*DTWD2*	.	.	.
5	118243997	C	A	intronic	*DTWD2*	.	.	.
5	118184606	T	A	intronic	*DTWD2*	.	.	.
5	118356071	A	G	intergenic	*DTWD2,DMXL1*	.	.	.
5	118328684	A	T	intergenic	*DTWD2,DMXL1*	.	.	.
7	76642377	T	A	ncRNA_intronic	*DTX2P1-UPK3BP1-PMS2P11*	.	.	.
7	76661642	C	T	intergenic	*DTX2P1-UPK3BP1-PMS2P11,PMS2P9*	.	.	.
1	221876863	C	T	intronic	*DUSP10*	.	.	.
1	221939799	AGTGTGT	A	intergenic	*DUSP10,LOC101929771*	.	.	.
1	221980361	T	C	intergenic	*DUSP10,LOC101929771*	.	.	.
10	76857029	C	T	intronic	*DUSP13*	.	.	.
6	332737	C	T	intronic	*DUSP22*	.	.	.
6	374008	C	A	intergenic	*DUSP22,IRF4*	.	.	.

8	33775654	C	T	intergenic	*DUSP26,LINC01288*	.	.	.
8	34205533	A	G	intergenic	*DUSP26,LINC01288*	.	.	.
8	34621804	A	C	intergenic	*DUSP26,LINC01288*	.	.	.
8	34120178	A	T	intergenic	*DUSP26,LINC01288*	.	.	.
8	34280274	G	T	intergenic	*DUSP26,LINC01288*	.	.	.
8	33652021	A	T	intergenic	*DUSP26,LINC01288*	.	.	.
8	34129348	T	C	intergenic	*DUSP26,LINC01288*	.	.	.
8	34352497	T	G	intergenic	*DUSP26,LINC01288*	.	.	.
8	34420563	C	A	intergenic	*DUSP26,LINC01288*	.	.	.
8	33733060	C	CAT	intergenic	*DUSP26,LINC01288*	.	.	.
8	33960202	C	T	intergenic	*DUSP26,LINC01288*	.	.	.
8	34290042	G	C	intergenic	*DUSP26,LINC01288*	.	.	.
8	33710393	A	ATACTTAAT	intergenic	*DUSP26,LINC01288*	.	.	.
8	33755140	C	T	intergenic	*DUSP26,LINC01288*	.	.	.
8	33915121	G	T	intergenic	*DUSP26,LINC01288*	.	.	.
8	33916035	G	A	intergenic	*DUSP26,LINC01288*	.	.	.
8	33917478	C	T	intergenic	*DUSP26,LINC01288*	.	.	.
8	33962036	T	C	intergenic	*DUSP26,LINC01288*	.	.	.
8	34049812	G	A	intergenic	*DUSP26,LINC01288*	.	.	.

8	34157000	A	G	intergenic	*DUSP26,LINC01288*	.	.	.
8	34159392	A	T	intergenic	*DUSP26,LINC01288*	.	.	.
8	34160264	T	C	intergenic	*DUSP26,LINC01288*	.	.	.
8	34396693	A	AG	intergenic	*DUSP26,LINC01288*	.	.	.
8	34400293	A	G	intergenic	*DUSP26,LINC01288*	.	.	.
8	34583316	G	A	intergenic	*DUSP26,LINC01288*	.	.	.
8	34599628	G	C	intergenic	*DUSP26,LINC01288*	.	.	.
8	34599650	G	C	intergenic	*DUSP26,LINC01288*	.	.	.
8	34600558	A	G	intergenic	*DUSP26,LINC01288*	.	.	.
8	34600748	C	T	intergenic	*DUSP26,LINC01288*	.	.	.
8	29309207	C	G	intergenic	*DUSP4,LINC00589*	.	.	.
8	29310472	G	C	intergenic	*DUSP4,LINC00589*	.	.	.
8	29457947	T	C	intergenic	*DUSP4,LINC00589*	.	.	.
10	112275738	A	T	intergenic	*DUSP5,SMC3*	.	.	.
1	228847644	C	T	intergenic	*DUSP5P1,RHOU*	.	.	.
1	228847645	A	G	intergenic	*DUSP5P1,RHOU*	.	.	.
14	19728378	CTTT	C	intergenic	*DUXAP10,LINC01296*	.	.	.
14	19747417	GAAAA	G	intergenic	*DUXAP10,LINC01296*	.	.	.
14	19818251	T	C	intergenic	*DUXAP10,LINC01296*	.	.	.
14	19938477	C	T	intergenic	*DUXAP10,POTEM*	.	.	.
22	16174942	G	A	ncRNA_intronic	*DUXAP8*	.	.	.
18	46900901	A	C	intronic	*DYM*	.	.	.
18	46631042	A	G	intronic	*DYM*	.	.	.
18	46632123	C	T	intronic	*DYM*	.	.	.
18	46632255	C	T	intronic	*DYM*	.	.	.

18	46681134	C	CTTATTA	intronic	*DYM*	.	.	.
18	46724015	G	A	intronic	*DYM*	.	.	.
18	46945471	G	A	intronic	*DYM*	.	.	.
18	46948862	A	T	intronic	*DYM*	.	.	.
18	46948864	G	A	intronic	*DYM*	.	.	.
18	46951088	T	C	intronic	*DYM*	.	.	.
18	46952152	G	A	intronic	*DYM*	.	.	.
18	52410007	A	T	intergenic	*DYNAP,RAB27B*	.	.	.
18	52300815	A	G	intergenic	*DYNAP,RAB27B*	.	.	.
7	95415109	A	G	intronic	*DYNC1I1*	.	.	.
7	95465446	C	T	intronic	*DYNC1I1*	.	.	.
2	172549107	A	G	intronic	*DYNC1I2*	.	.	.
2	172612544	G	A	intergenic	*DYNC1I2,SLC25A12*	.	.	.
2	172615534	T	C	intergenic	*DYNC1I2,SLC25A12*	.	.	.
3	32602902	T	C	intronic	*DYNC1LI1*	.	.	.
3	32717703	A	G	intergenic	*DYNC1LI1,CNOT10*	.	.	.
3	32687259	A	G	intergenic	*DYNC1LI1,CNOT10*	.	.	.
11	103109311	TACACAC	T	intronic	*DYNC2H1*	.	.	.
11	103225197	T	C	intronic	*DYNC2H1*	.	.	.
11	103107799	T	C	intronic	*DYNC2H1*	.	.	.
11	103009184	T	C	intronic	*DYNC2H1*	.	.	.
11	103134658	A	T	intronic	*DYNC2H1*	.	.	.
11	103258175	C	A	intronic	*DYNC2H1*	.	.	.
11	103258839	G	A	intronic	*DYNC2H1*	.	.	.
11	103259940	C	CT	intronic	*DYNC2H1*	.	.	.
11	103479870	CAGAGAG	C	intergenic	*DYNC2H1,MIR4693*	.	.	.
11	103484885	T	A	intergenic	*DYNC2H1,MIR4693*	.	.	.
11	103486100	G	A	intergenic	*DYNC2H1,MIR4693*	.	.	.
11	103486335	C	T	intergenic	*DYNC2H1,MIR4693*	.	.	.

11	103486987	T	C	intergenic	*DYNC2H1,MIR4693*	.	.	.
11	103572409	ATGTGTGTGTGTGTGTGTG	A	intergenic	*DYNC2H1,MIR4693*	.	.	.
11	103631677	T	C	intergenic	*DYNC2H1,MIR4693*	.	.	.
11	103631679	C	T	intergenic	*DYNC2H1,MIR4693*	.	.	.
11	103632544	G	A	intergenic	*DYNC2H1,MIR4693*	.	.	.
11	103686225	A	G	intergenic	*DYNC2H1,MIR4693*	.	.	.
17	56201993	G	A	intergenic	*DYNLL2,OR4D1*	.	.	.
17	56210155	A	G	intergenic	*DYNLL2,OR4D1*	.	.	.
X	37814794	T	C	intergenic	*DYNLT3,HYPM*	.	.	.
X	37776862	T	G	intergenic	*DYNLT3,HYPM*	.	.	.
21	38961209	G	C	intergenic	*DYRK1A,KCNJ6*	.	.	.
2	71848838	A	G	intronic	*DYSF*	.	.	.
2	72159198	T	C	intergenic	*DYSF,CYP26B1*	.	.	.
2	71952689	CTGTG	C	intergenic	*DYSF,CYP26B1*	.	.	.
2	72293662	T	C	intergenic	*DYSF,CYP26B1*	.	.	.
2	72295989	T	C	intergenic	*DYSF,CYP26B1*	.	.	.
2	207576393	G	T	intronic	*DYTN*	.	.	.
2	207596397	C	T	intergenic	*DYTN,MDH1B*	.	.	.
20	18367407	C	CATAG	intronic	*DZANK1*	.	.	.
3	108387584	G	T	intronic	*DZIP3*	.	.	.
3	108400194	T	C	intronic	*DZIP3*	.	.	.
8	86124970	C	A	intronic	*E2F5*	.	.	.
8	86097964	T	A	intronic	*E2F5*	.	.	.
2	11590408	T	A	intronic	*E2F6*	.	.	.
12	78076470	G	A	intergenic	*E2F7,NAV3*	.	.	.
12	78079272	C	CAA	intergenic	*E2F7,NAV3*	.	.	.
12	77893626	GTGTA	G	intergenic	*E2F7,NAV3*	.	.	.
12	77486108	C	T	intergenic	*E2F7,NAV3*	.	.	.
12	77486691	G	T	intergenic	*E2F7,NAV3*	.	.	.
12	77487987	G	T	intergenic	*E2F7,NAV3*	.	.	.
12	77655519	T	C	intergenic	*E2F7,NAV3*	.	.	.

12	77660679	T	G	intergenic	*E2F7,NAV3*	.	.	.
12	77683669	T	C	intergenic	*E2F7,NAV3*	.	.	.
12	77684063	T	C	intergenic	*E2F7,NAV3*	.	.	.
12	77684064	G	A	intergenic	*E2F7,NAV3*	.	.	.
12	77684640	T	A	intergenic	*E2F7,NAV3*	.	.	.
12	77685092	G	C	intergenic	*E2F7,NAV3*	.	.	.
12	77711482	A	G	intergenic	*E2F7,NAV3*	.	.	.
12	77818037	C	A	intergenic	*E2F7,NAV3*	.	.	.
12	77854341	A	T	intergenic	*E2F7,NAV3*	.	.	.
12	77861379	T	C	intergenic	*E2F7,NAV3*	.	.	.
12	77861380	G	A	intergenic	*E2F7,NAV3*	.	.	.
12	77921507	G	A	intergenic	*E2F7,NAV3*	.	.	.
12	77922739	A	G	intergenic	*E2F7,NAV3*	.	.	.
11	19246044	G	GTATA	UTR3	*E2F8*	.	.	.
3	121570148	C	T	intronic	*EAF2*	.	.	.
5	158355294	G	A	intronic	*EBF1*	.	.	.
5	158409801	TA	T	intronic	*EBF1*	.	.	.
5	158243362	A	G	intronic	*EBF1*	.	.	.
5	158417214	A	G	intronic	*EBF1*	.	.	.
5	158324221	C	T	intronic	*EBF1*	.	.	.
5	158371763	A	G	intronic	*EBF1*	.	.	.
8	25865626	A	G	intronic	*EBF2*	.	.	.
8	25879524	T	C	intronic	*EBF2*	.	.	.
8	25963178	C	T	intergenic	*EBF2,PPP2R2A*	.	.	.
20	2698475	G	T	intronic	*EBF4*	.	.	.
10	74903029	C	T	intronic	*ECD*	.	.	.
6	127696056	C	T	intergenic	*ECHDC1,KIAA0408*	.	.	.
6	127729462	T	G	intergenic	*ECHDC1,KIAA0408*	.	.	.
6	127730657	A	C	intergenic	*ECHDC1,KIAA0408*	.	.	.
6	127732344	T	A	intergenic	*ECHDC1,KIAA0408*	.	.	.
6	127748344	G	T	intergenic	*ECHDC1,KIAA0408*	.	.	.
6	127748346	TAAAA	T	intergenic	*ECHDC1,KIAA0408*	.	.	.

6	139189074	C	G	intronic	*ECT2L*	.	.	.
X	68886049	T	C	intronic	*EDA*	.	.	.
X	68853794	C	A	intronic	*EDA*	.	.	.
X	68869449	G	T	intronic	*EDA*	.	.	.
X	68901812	C	A	intronic	*EDA*	.	.	.
X	68902249	C	A	intronic	*EDA*	.	.	.
X	69132630	G	T	intronic	*EDA*	.	.	.
X	69244994	C	G	intronic	*EDA*	.	.	.
X	68881010	A	G	intronic	*EDA*	.	.	.
X	68899627	T	A	intronic	*EDA*	.	.	.
X	68899989	G	T	intronic	*EDA*	.	.	.
X	68971981	A	G	intronic	*EDA*	.	.	.
X	69003477	T	C	intronic	*EDA*	.	.	.
X	69054290	G	T	intronic	*EDA*	.	.	.
X	69077747	C	T	intronic	*EDA*	.	.	.
X	65974071	CATATATATATATATGTATGTATGTGTGTGTGTATATATATATATATATATACACACACACATACATACAT	C	intergenic	*EDA2R,AR*	.	.	.
X	66640645	A	G	intergenic	*EDA2R,AR*	.	.	.
X	65864247	C	A	intergenic	*EDA2R,AR*	.	.	.
X	66638913	G	C	intergenic	*EDA2R,AR*	.	.	.
X	66235819	G	T	intergenic	*EDA2R,AR*	.	.	.
X	66244048	T	C	intergenic	*EDA2R,AR*	.	.	.
X	66312002	G	A	intergenic	*EDA2R,AR*	.	.	.
X	66047368	C	A	intergenic	*EDA2R,AR*	.	.	.
X	66615985	C	T	intergenic	*EDA2R,AR*	.	.	.
X	66416278	CA	C	intergenic	*EDA2R,AR*	.	.	.
X	65866972	T	C	intergenic	*EDA2R,AR*	.	.	.
X	65866973	G	A	intergenic	*EDA2R,AR*	.	.	.
X	65907293	C	T	intergenic	*EDA2R,AR*	.	.	.
X	65907716	A	G	intergenic	*EDA2R,AR*	.	.	.
X	65908384	A	G	intergenic	*EDA2R,AR*	.	.	.

X	65909841	T	C	intergenic	*EDA2R,AR*	.	.	.
X	65909843	A	T	intergenic	*EDA2R,AR*	.	.	.
X	65934339	T	G	intergenic	*EDA2R,AR*	.	.	.
X	65940284	C	G	intergenic	*EDA2R,AR*	.	.	.
X	65961485	G	A	intergenic	*EDA2R,AR*	.	.	.
X	65982330	T	G	intergenic	*EDA2R,AR*	.	.	.
X	65983240	T	C	intergenic	*EDA2R,AR*	.	.	.
X	66011562	T	G	intergenic	*EDA2R,AR*	.	.	.
X	66012345	G	A	intergenic	*EDA2R,AR*	.	.	.
X	66013483	C	G	intergenic	*EDA2R,AR*	.	.	.
X	66013484	A	G	intergenic	*EDA2R,AR*	.	.	.
X	66023087	T	C	intergenic	*EDA2R,AR*	.	.	.
X	66029368	A	G	intergenic	*EDA2R,AR*	.	.	.
X	66031937	C	A	intergenic	*EDA2R,AR*	.	.	.
X	66031938	A	G	intergenic	*EDA2R,AR*	.	.	.
X	66107626	T	C	intergenic	*EDA2R,AR*	.	.	.
X	66165137	T	A	intergenic	*EDA2R,AR*	.	.	.
X	66165845	G	T	intergenic	*EDA2R,AR*	.	.	.
X	66166498	T	C	intergenic	*EDA2R,AR*	.	.	.
X	66166821	C	T	intergenic	*EDA2R,AR*	.	.	.
X	66172934	T	C	intergenic	*EDA2R,AR*	.	.	.
X	66174920	G	A	intergenic	*EDA2R,AR*	.	.	.
X	66190938	G	A	intergenic	*EDA2R,AR*	.	.	.
X	66193292	T	A	intergenic	*EDA2R,AR*	.	.	.
X	66195127	C	A	intergenic	*EDA2R,AR*	.	.	.
X	66195681	C	G	intergenic	*EDA2R,AR*	.	.	.
X	66195682	A	C	intergenic	*EDA2R,AR*	.	.	.
X	66199874	T	C	intergenic	*EDA2R,AR*	.	.	.
X	66213637	C	T	intergenic	*EDA2R,AR*	.	.	.
X	66248477	A	G	intergenic	*EDA2R,AR*	.	.	.
X	66248754	A	G	intergenic	*EDA2R,AR*	.	.	.
X	66282527	T	C	intergenic	*EDA2R,AR*	.	.	.
X	66283555	C	T	intergenic	*EDA2R,AR*	.	.	.
X	66301647	G	A	intergenic	*EDA2R,AR*	.	.	.
X	66362140	G	C	intergenic	*EDA2R,AR*	.	.	.
X	66366011	C	G	intergenic	*EDA2R,AR*	.	.	.
X	66371096	G	C	intergenic	*EDA2R,AR*	.	.	.
X	66371097	C	A	intergenic	*EDA2R,AR*	.	.	.

X	66407380	A	G	intergenic	*EDA2R,AR*	.	.	.
X	66409753	C	T	intergenic	*EDA2R,AR*	.	.	.
X	66435842	G	A	intergenic	*EDA2R,AR*	.	.	.
X	66435844	T	C	intergenic	*EDA2R,AR*	.	.	.
X	66438622	A	G	intergenic	*EDA2R,AR*	.	.	.
X	66441662	T	C	intergenic	*EDA2R,AR*	.	.	.
X	66472186	G	A	intergenic	*EDA2R,AR*	.	.	.
X	66474948	T	TG	intergenic	*EDA2R,AR*	.	.	.
X	66475721	A	G	intergenic	*EDA2R,AR*	.	.	.
X	66475723	A	G	intergenic	*EDA2R,AR*	.	.	.
X	66535999	A	G	intergenic	*EDA2R,AR*	.	.	.
X	66536300	C	T	intergenic	*EDA2R,AR*	.	.	.
X	66542925	C	T	intergenic	*EDA2R,AR*	.	.	.
X	66543219	T	C	intergenic	*EDA2R,AR*	.	.	.
X	66543220	G	C	intergenic	*EDA2R,AR*	.	.	.
X	66546069	T	A	intergenic	*EDA2R,AR*	.	.	.
X	66583127	G	A	intergenic	*EDA2R,AR*	.	.	.
X	66617814	A	G	intergenic	*EDA2R,AR*	.	.	.
X	66618143	G	A	intergenic	*EDA2R,AR*	.	.	.
X	66633302	C	T	intergenic	*EDA2R,AR*	.	.	.
X	66670168	T	A	intergenic	*EDA2R,AR*	.	.	.
X	66680298	C	A	intergenic	*EDA2R,AR*	.	.	.
X	66693873	C	T	intergenic	*EDA2R,AR*	.	.	.
X	66694015	A	G	intergenic	*EDA2R,AR*	.	.	.
X	66713998	T	A	intergenic	*EDA2R,AR*	.	.	.
X	66733047	A	C	intergenic	*EDA2R,AR*	.	.	.
X	66733609	T	A	intergenic	*EDA2R,AR*	.	.	.
X	66734982	C	A	intergenic	*EDA2R,AR*	.	.	.
2	109555767	G	C	intronic	*EDAR*	.	.	.
2	109555768	T	A	intronic	*EDAR*	.	.	.
2	109629153	G	A	intergenic	*EDAR,SH3RF3-AS1*	.	.	.
2	109675858	C	T	intergenic	*EDAR,SH3RF3-AS1*	.	.	.
2	109730766	G	T	intergenic	*EDAR,SH3RF3-AS1*	.	.	.
1	236569241	T	C	intronic	*EDARADD*	.	.	.
1	236617448	C	T	intronic	*EDARADD*	.	.	.

1	236617449	A	G	intronic	*EDARADD*	.	.	.
3	5261216	A	T	UTR3	*EDEM1*	.	.	.
5	83276495	AATATAT	A	intronic	*EDIL3*	.	.	.
5	83237457	A	G	UTR3	*EDIL3*	.	.	.
5	83315331	C	G	intronic	*EDIL3*	.	.	.
5	83351175	G	T	intronic	*EDIL3*	.	.	.
5	83351192	T	A	intronic	*EDIL3*	.	.	.
5	83351193	A	T	intronic	*EDIL3*	.	.	.
5	83396131	T	C	intronic	*EDIL3*	.	.	.
5	83605724	A	G	intronic	*EDIL3*	.	.	.
5	83615663	C	T	intronic	*EDIL3*	.	.	.
5	83863828	C	G	intergenic	*EDIL3,NBPF22P*	.	.	.
5	84377912	C	A	intergenic	*EDIL3,NBPF22P*	.	.	.
5	85100324	G	A	intergenic	*EDIL3,NBPF22P*	.	.	.
5	85163426	G	A	intergenic	*EDIL3,NBPF22P*	.	.	.
5	85227557	T	A	intergenic	*EDIL3,NBPF22P*	.	.	.
5	85420728	C	A	intergenic	*EDIL3,NBPF22P*	.	.	.
5	84944888	C	CAAAAAAA	intergenic	*EDIL3,NBPF22P*	.	.	.
5	84948068	T	C	intergenic	*EDIL3,NBPF22P*	.	.	.
5	84107048	ATCTTGGC	A	intergenic	*EDIL3,NBPF22P*	.	.	.
5	85338462	A	T	intergenic	*EDIL3,NBPF22P*	.	.	.
5	84775289	T	G	intergenic	*EDIL3,NBPF22P*	.	.	.
5	83725110	A	T	intergenic	*EDIL3,NBPF22P*	.	.	.
5	83849716	T	A	intergenic	*EDIL3,NBPF22P*	.	.	.
5	83991120	A	T	intergenic	*EDIL3,NBPF22P*	.	.	.
5	84235317	A	T	intergenic	*EDIL3,NBPF22P*	.	.	.
5	84271319	T	C	intergenic	*EDIL3,NBPF22P*	.	.	.
5	84271320	G	A	intergenic	*EDIL3,NBPF22P*	.	.	.
5	84272355	A	G	intergenic	*EDIL3,NBPF22P*	.	.	.
5	84359717	A	G	intergenic	*EDIL3,NBPF22P*	.	.	.
5	84388665	A	T	intergenic	*EDIL3,NBPF22P*	.	.	.
5	84397843	C	T	intergenic	*EDIL3,NBPF22P*	.	.	.
5	84397844	C	T	intergenic	*EDIL3,NBPF22P*	.	.	.
5	84465042	C	T	intergenic	*EDIL3,NBPF22P*	.	.	.
5	84729446	C	A	intergenic	*EDIL3,NBPF22P*	.	.	.
5	84729877	A	T	intergenic	*EDIL3,NBPF22P*	.	.	.
5	84732292	T	A	intergenic	*EDIL3,NBPF22P*	.	.	.
5	85019452	C	T	intergenic	*EDIL3,NBPF22P*	.	.	.

5	85020098	T	C	intergenic	*EDIL3,NBPF22P*	.	.	.
5	85020099	G	A	intergenic	*EDIL3,NBPF22P*	.	.	.
5	85020281	G	A	intergenic	*EDIL3,NBPF22P*	.	.	.
5	85088365	A	G	intergenic	*EDIL3,NBPF22P*	.	.	.
5	85088960	A	G	intergenic	*EDIL3,NBPF22P*	.	.	.
5	85295362	G	A	intergenic	*EDIL3,NBPF22P*	.	.	.
5	85376272	C	T	intergenic	*EDIL3,NBPF22P*	.	.	.
5	85376274	A	G	intergenic	*EDIL3,NBPF22P*	.	.	.
5	85442929	C	A	intergenic	*EDIL3,NBPF22P*	.	.	.
5	85561253	A	G	intergenic	*EDIL3,NBPF22P*	.	.	.
5	85561255	G	T	intergenic	*EDIL3,NBPF22P*	.	.	.
5	85563533	C	T	intergenic	*EDIL3,NBPF22P*	.	.	.
5	85564233	C	T	intergenic	*EDIL3,NBPF22P*	.	.	.
5	85564815	T	C	intergenic	*EDIL3,NBPF22P*	.	.	.
6	12584237	CAA	C	intergenic	*EDN1,PHACTR1*	.	.	.
6	12523958	T	C	intergenic	*EDN1,PHACTR1*	.	.	.
6	12542950	C	T	intergenic	*EDN1,PHACTR1*	.	.	.
6	12552208	T	A	intergenic	*EDN1,PHACTR1*	.	.	.
20	57922697	G	A	intergenic	*EDN3,PHACTR3*	.	.	.
20	58144437	G	A	intergenic	*EDN3,PHACTR3*	.	.	.
20	58005475	A	T	intergenic	*EDN3,PHACTR3*	.	.	.
13	78538226	C	G	intronic	*EDNRB*	.	.	.
13	78415432	T	G	ncRNA_intronic	*EDNRB-AS1*	.	.	.
13	78415433	C	T	ncRNA_intronic	*EDNRB-AS1*	.	.	.
10	127452878	G	T	downstream	*EDRF1*	.	.	.
12	93186202	TA	T	intronic	*EEA1*	.	.	.
12	93187039	G	A	intronic	*EEA1*	.	.	.
12	93228349	T	A	intronic	*EEA1*	.	.	.
12	93255007	A	G	intronic	*EEA1*	.	.	.
12	93341885	C	G	intergenic	*EEA1,LOC643339*	.	.	.
6	74295015	T	TTTTTTTTTT	intergenic	*EEF1A1,SLC17A5*	.	.	.
13	32508315	C	T	ncRNA_intronic	*EEF1DP3*	.	.	.
6	8249087	A	C	intergenic	*EEF1E1-BLOC1S5,SLC35B3*	.	.	.
6	8310487	G	A	intergenic	*EEF1E1-BLOC1S5,SLC35B3*	.	.	.

6	8310489	G	A	intergenic	*EEF1E1-BLOC1S5,SLC35B3*	.	.	.
16	5419377	A	T	intergenic	*EEF2KMT,LINC01570*	.	.	.
16	5574512	C	A	intergenic	*EEF2KMT,LINC01570*	.	.	.
16	5188553	A	ACTT	intergenic	*EEF2KMT,LINC01570*	.	.	.
3	127965656	A	G	intronic	*EEFSEC*	.	.	.
7	36295696	C	G	intronic	*EEPD1*	.	.	.
8	49817606	CAGAG	C	intergenic	*EFCAB1,SNAI2*	.	.	.
14	90371231	G	A	intronic	*EFCAB11*	.	.	.
17	45457853	G	A	intronic	*EFCAB13*	.	.	.
17	45459246	T	TCC	intronic	*EFCAB13*	.	.	.
1	47159641	A	G	intronic	*EFCAB14*	.	.	.
1	47195934	A	T	intergenic	*EFCAB14,CYP4B1*	.	.	.
1	47195935	C	A	intergenic	*EFCAB14,CYP4B1*	.	.	.
1	47199642	A	C	intergenic	*EFCAB14,CYP4B1*	.	.	.
17	60479060	A	G	intronic	*EFCAB3*	.	.	.
17	60479491	T	C	intronic	*EFCAB3*	.	.	.
17	28277288	T	C	intronic	*EFCAB5*	.	.	.
17	28340872	T	G	intronic	*EFCAB5*	.	.	.
17	28352057	C	T	intronic	*EFCAB5*	.	.	.
6	52318077	C	G	intronic	*EFHC1*	.	.	.
6	52307856	C	T	intronic	*EFHC1*	.	.	.
6	52308801	T	C	intronic	*EFHC1*	.	.	.
6	52308802	G	A	intronic	*EFHC1*	.	.	.
6	52343217	C	G	intronic	*EFHC1*	.	.	.
X	44067082	A	G	intronic	*EFHC2*	.	.	.
X	44067084	C	T	intronic	*EFHC2*	.	.	.
X	44114763	A	G	intronic	*EFHC2*	.	.	.
X	44115752	T	C	intronic	*EFHC2*	.	.	.
X	44117190	C	T	intronic	*EFHC2*	.	.	.
X	44356395	A	T	intergenic	*EFHC2,FUNDC1*	.	.	.
5	107185456	G	A	intergenic	*EFNA5,FBXL17*	.	.	.
5	107058145	C	T	intergenic	*EFNA5,FBXL17*	.	.	.
5	107058146	A	G	intergenic	*EFNA5,FBXL17*	.	.	.

X	68244225	G	A	intergenic	*EFNB1,PJA1*	.	.	.
X	68161287	A	G	intergenic	*EFNB1,PJA1*	.	.	.
X	68273316	G	A	intergenic	*EFNB1,PJA1*	.	.	.
X	68273317	T	C	intergenic	*EFNB1,PJA1*	.	.	.
X	68273531	T	G	intergenic	*EFNB1,PJA1*	.	.	.
8	132975644	C	T	intronic	*EFR3A*	.	.	.
2	25347183	G	C	intronic	*EFR3B*	.	.	.
15	82499627	GTATA	G	intronic	*EFTUD1*	.	.	.
15	82485181	A	C	intronic	*EFTUD1*	.	.	.
3	168441595	C	T	ncRNA_intronic	*EGFEM1P*	.	.	.
3	168080123	G	A	ncRNA_intronic	*EGFEM1P*	.	.	.
5	38341445	G	A	intronic	*EGFLAM*	.	.	.
5	38391987	T	TCC	intronic	*EGFLAM*	.	.	.
5	38391989	GTC	G	intronic	*EGFLAM*	.	.	.
7	55107174	G	C	intronic	*EGFR*	.	.	.
7	55125843	T	C	intronic	*EGFR*	.	.	.
7	55296873	G	A	intergenic	*EGFR,ELDR*	.	.	.
14	34663535	A	T	intergenic	*EGLN3,SPTSSA*	.	.	.
14	34734271	T	C	intergenic	*EGLN3,SPTSSA*	.	.	.
14	34632423	A	G	intergenic	*EGLN3,SPTSSA*	.	.	.
14	34459217	A	AGTGTGTGT	intergenic	*EGLN3,SPTSSA*	.	.	.
14	34434034	G	A	intergenic	*EGLN3,SPTSSA*	.	.	.
14	34612342	T	A	intergenic	*EGLN3,SPTSSA*	.	.	.
14	34613639	G	A	intergenic	*EGLN3,SPTSSA*	.	.	.
14	34763772	G	A	intergenic	*EGLN3,SPTSSA*	.	.	.
14	34862280	C	A	intergenic	*EGLN3,SPTSSA*	.	.	.
14	34863269	C	G	intergenic	*EGLN3,SPTSSA*	.	.	.
14	34865462	G	A	intergenic	*EGLN3,SPTSSA*	.	.	.
14	34865558	G	T	intergenic	*EGLN3,SPTSSA*	.	.	.
14	34865991	G	A	intergenic	*EGLN3,SPTSSA*	.	.	.
10	64645028	G	A	intergenic	*EGR2,NRBF2*	.	.	.
10	64712894	G	A	intergenic	*EGR2,NRBF2*	.	.	.
10	64820715	G	A	intergenic	*EGR2,NRBF2*	.	.	.
10	64820773	C	T	intergenic	*EGR2,NRBF2*	.	.	.
2	63246965	G	GGGAGGGA	intronic	*EHBP1*	.	.	.
2	62939656	T	G	intronic	*EHBP1*	.	.	.
2	63135014	T	C	intronic	*EHBP1*	.	.	.
2	63065571	A	G	intronic	*EHBP1*	.	.	.

2	63067315	C	T	intronic	*EHBP1*	.	.	.
2	63149225	G	A	intronic	*EHBP1*	.	.	.
2	31503640	G	A	intergenic	*EHD3,XDH*	.	.	.
3	184924767	A	G	intronic	*EHHADH*	.	.	.
19	40085763	C	T	intergenic	*EID2,LGALS13*	.	.	.
19	40085765	C	T	intergenic	*EID2,LGALS13*	.	.	.
2	88912900	A	G	intronic	*EIF2AK3*	.	.	.
15	40276042	G	A	intronic	*EIF2AK4*	.	.	.
15	40277314	A	G	intronic	*EIF2AK4*	.	.	.
X	24121535	A	T	intergenic	*EIF2S3,ZFX-AS1*	.	.	.
X	24157958	C	A	intergenic	*EIF2S3,ZFX-AS1*	.	.	.
X	24157959	A	G	intergenic	*EIF2S3,ZFX-AS1*	.	.	.
22	36951209	C	T	intergenic	*EIF3D,CACNG2*	.	.	.
22	36952265	T	A	intergenic	*EIF3D,CACNG2*	.	.	.
8	109223774	T	C	intronic	*EIF3E*	.	.	.
8	109382382	G	T	intergenic	*EIF3E,EMC2*	.	.	.
8	109394746	G	A	intergenic	*EIF3E,EMC2*	.	.	.
11	8031150	G	A	intergenic	*EIF3F,CASC23*	.	.	.
7	110081167	G	T	intergenic	*EIF3IP1,IMMP2L*	.	.	.
7	110177067	T	C	intergenic	*EIF3IP1,IMMP2L*	.	.	.
7	109603457	C	A	intergenic	*EIF3IP1,IMMP2L*	.	.	.
7	109685040	A	G	intergenic	*EIF3IP1,IMMP2L*	.	.	.
7	110062658	G	T	intergenic	*EIF3IP1,IMMP2L*	.	.	.
7	109671215	T	C	intergenic	*EIF3IP1,IMMP2L*	.	.	.
7	109762722	C	T	intergenic	*EIF3IP1,IMMP2L*	.	.	.
7	109764550	C	T	intergenic	*EIF3IP1,IMMP2L*	.	.	.
7	109765165	C	T	intergenic	*EIF3IP1,IMMP2L*	.	.	.
7	110008216	A	G	intergenic	*EIF3IP1,IMMP2L*	.	.	.
7	110079113	C	A	intergenic	*EIF3IP1,IMMP2L*	.	.	.
7	110097969	G	A	intergenic	*EIF3IP1,IMMP2L*	.	.	.
7	110097971	T	C	intergenic	*EIF3IP1,IMMP2L*	.	.	.
7	110098975	T	C	intergenic	*EIF3IP1,IMMP2L*	.	.	.
7	110114632	A	T	intergenic	*EIF3IP1,IMMP2L*	.	.	.
7	110298054	A	G	intergenic	*EIF3IP1,IMMP2L*	.	.	.
2	233445337	C	T	intronic	*EIF4E2*	.	.	.
3	71765389	CTTT	C	intronic	*EIF4E3*	.	.	.
1	21160010	A	T	intronic	*EIF4G3*	.	.	.
1	21381659	TAC	T	intronic	*EIF4G3*	.	.	.

1	21487500	A	C	intronic	*EIF4G3*	.	.	.
14	103831832	T	TTTCC	intergenic	*EIF5,MARK3*	.	.	.
18	48507466	G	C	intronic	*ELAC1*	.	.	.
18	48528255	C	T	intergenic	*ELAC1,SMAD4*	.	.	.
17	13141604	C	A	intergenic	*ELAC2,HS3ST3A1*	.	.	.
17	13040879	A	G	intergenic	*ELAC2,HS3ST3A1*	.	.	.
17	13061824	G	T	intergenic	*ELAC2,HS3ST3A1*	.	.	.
17	13174181	AAAATAAAT	A	intergenic	*ELAC2,HS3ST3A1*	.	.	.
17	12957141	C	T	intergenic	*ELAC2,HS3ST3A1*	.	.	.
17	13181160	C	G	intergenic	*ELAC2,HS3ST3A1*	.	.	.
19	8089790	T	C	intergenic	*ELAVL1,CCL25*	.	.	.
19	8089791	G	A	intergenic	*ELAVL1,CCL25*	.	.	.
9	24525029	C	CCATTTA	intergenic	*ELAVL2,IZUMO3*	.	.	.
9	24487843	C	A	intergenic	*ELAVL2,IZUMO3*	.	.	.
9	23937284	C	T	intergenic	*ELAVL2,IZUMO3*	.	.	.
9	24051072	G	A	intergenic	*ELAVL2,IZUMO3*	.	.	.
9	24213912	T	C	intergenic	*ELAVL2,IZUMO3*	.	.	.
9	24280261	A	G	intergenic	*ELAVL2,IZUMO3*	.	.	.
9	24280777	A	C	intergenic	*ELAVL2,IZUMO3*	.	.	.
9	24347700	A	G	intergenic	*ELAVL2,IZUMO3*	.	.	.
1	50805214	G	A	intergenic	*ELAVL4,DMRTA2*	.	.	.
1	50830264	C	G	intergenic	*ELAVL4,DMRTA2*	.	.	.
7	55386632	G	C	intergenic	*ELDR,LANCL2*	.	.	.
13	41556992	A	G	intronic	*ELF1*	.	.	.
13	41630835	G	C	intergenic	*ELF1,WBP4*	.	.	.
13	41617320	T	A	intergenic	*ELF1,WBP4*	.	.	.
11	34607173	G	A	intergenic	*ELF5,EHF*	.	.	.
7	1807277	AACACACACACACAC	A	intergenic	*ELFN1,MAD1L1*	.	.	.
14	106233651	C	A	intergenic	*ELK2AP,MIR4507*	.	.	.
14	106233652	A	G	intergenic	*ELK2AP,MIR4507*	.	.	.
14	106145970	C	T	intergenic	*ELK2AP,MIR4507*	.	.	.
14	106238012	A	G	intergenic	*ELK2AP,MIR4507*	.	.	.
1	205584549	T	A	UTR3	*ELK4*	.	.	.
7	36975305	C	T	intronic	*ELMO1*	.	.	.
7	37397303	G	T	intronic	*ELMO1*	.	.	.
7	37437391	A	G	intronic	*ELMO1*	.	.	.

7	37628538	A	T	intergenic	*ELMO1,GPR141*	.	.	.
7	37679715	T	C	intergenic	*ELMO1,GPR141*	.	.	.
7	37679716	G	A	intergenic	*ELMO1,GPR141*	.	.	.
20	45047143	C	T	intergenic	*ELMO2,ZNF663P*	.	.	.
6	10987497	A	G	intronic	*ELOVL2*	.	.	.
6	80666273	A	G	intergenic	*ELOVL4,TTK*	.	.	.
6	80668230	T	C	intergenic	*ELOVL4,TTK*	.	.	.
6	53169341	G	A	intronic	*ELOVL5*	.	.	.
4	111000415	T	C	intronic	*ELOVL6*	.	.	.
4	111220763	G	A	intergenic	*ELOVL6,ENPEP*	.	.	.
4	111299006	A	G	intergenic	*ELOVL6,ENPEP*	.	.	.
4	111323495	C	A	intergenic	*ELOVL6,ENPEP*	.	.	.
4	111247473	T	C	intergenic	*ELOVL6,ENPEP*	.	.	.
4	111261962	A	G	intergenic	*ELOVL6,ENPEP*	.	.	.
4	111309953	A	G	intergenic	*ELOVL6,ENPEP*	.	.	.
4	111310350	G	A	intergenic	*ELOVL6,ENPEP*	.	.	.
4	111310562	C	T	intergenic	*ELOVL6,ENPEP*	.	.	.
4	111331036	G	T	intergenic	*ELOVL6,ENPEP*	.	.	.
4	111356332	G	A	intergenic	*ELOVL6,ENPEP*	.	.	.
4	111356333	C	T	intergenic	*ELOVL6,ENPEP*	.	.	.
5	60159029	T	C	intergenic	*ELOVL7,ERCC8*	.	.	.
8	28099099	C	T	intergenic	*ELP3,PNOC*	.	.	.
8	28068623	T	C	intergenic	*ELP3,PNOC*	.	.	.
8	28068625	C	T	intergenic	*ELP3,PNOC*	.	.	.
8	28072793	T	C	intergenic	*ELP3,PNOC*	.	.	.
8	28072794	G	C	intergenic	*ELP3,PNOC*	.	.	.
11	31647922	A	G	intronic	*ELP4*	.	.	.
11	31567704	A	G	intronic	*ELP4*	.	.	.
11	31597488	A	T	intronic	*ELP4*	.	.	.
11	31598271	G	A	intronic	*ELP4*	.	.	.
11	31716470	G	A	intronic	*ELP4*	.	.	.
11	31718315	G	A	intronic	*ELP4*	.	.	.
11	31765378	T	TA	intronic	*ELP4*	.	.	.
3	47546286	A	C	intronic	*ELP6*	.	.	.
5	49889193	C	A	intergenic	*EMB,PARP8*	.	.	.
5	49777608	C	A	intergenic	*EMB,PARP8*	.	.	.
5	49823490	A	G	intergenic	*EMB,PARP8*	.	.	.
5	49938344	G	T	intergenic	*EMB,PARP8*	.	.	.

5	49749170	C	A	intergenic	*EMB,PARP8*	.	.	.
5	49749732	G	A	intergenic	*EMB,PARP8*	.	.	.
5	49751963	C	A	intergenic	*EMB,PARP8*	.	.	.
5	49783568	G	T	intergenic	*EMB,PARP8*	.	.	.
5	49792062	G	A	intergenic	*EMB,PARP8*	.	.	.
5	49794821	C	G	intergenic	*EMB,PARP8*	.	.	.
5	49827811	A	G	intergenic	*EMB,PARP8*	.	.	.
5	49828033	C	T	intergenic	*EMB,PARP8*	.	.	.
5	49839012	A	T	intergenic	*EMB,PARP8*	.	.	.
5	49839013	A	C	intergenic	*EMB,PARP8*	.	.	.
5	49892345	A	T	intergenic	*EMB,PARP8*	.	.	.
5	49893156	G	A	intergenic	*EMB,PARP8*	.	.	.
5	49895012	C	T	intergenic	*EMB,PARP8*	.	.	.
5	49958614	G	A	intergenic	*EMB,PARP8*	.	.	.
1	121279642	C	T	ncRNA_intronic	*EMBP1*	.	.	.
1	121370309	C	G	intergenic	*EMBP1,NONE*	.	.	.
1	121480542	A	C	intergenic	*EMBP1,NONE*	.	.	.
1	121387080	C	T	intergenic	*EMBP1,NONE*	.	.	.
1	121386916	C	G	intergenic	*EMBP1,NONE*	.	.	.
1	121352923	A	C	intergenic	*EMBP1,NONE*	.	.	.
1	121434252	T	G	intergenic	*EMBP1,NONE*	.	.	.
1	121480636	C	T	intergenic	*EMBP1,NONE*	.	.	.
1	121318909	A	T	intergenic	*EMBP1,NONE*	.	.	.
1	121321104	G	A	intergenic	*EMBP1,NONE*	.	.	.
1	121321847	T	C	intergenic	*EMBP1,NONE*	.	.	.
1	121323916	GT	G	intergenic	*EMBP1,NONE*	.	.	.
1	121325859	C	T	intergenic	*EMBP1,NONE*	.	.	.
1	121343836	C	G	intergenic	*EMBP1,NONE*	.	.	.
1	121472961	T	C	intergenic	*EMBP1,NONE*	.	.	.
8	109779233	C	T	intergenic	*EMC2,TMEM74*	.	.	.
8	109708153	T	G	intergenic	*EMC2,TMEM74*	.	.	.
8	109513754	G	T	intergenic	*EMC2,TMEM74*	.	.	.
8	109514232	A	G	intergenic	*EMC2,TMEM74*	.	.	.
8	109515933	T	A	intergenic	*EMC2,TMEM74*	.	.	.
8	109540990	G	A	intergenic	*EMC2,TMEM74*	.	.	.
8	109541932	G	T	intergenic	*EMC2,TMEM74*	.	.	.
18	2905394	G	A	intronic	*EMILIN2*	.	.	.
18	2889511	T	C	intronic	*EMILIN2*	.	.	.

18	2861162	G	T	intronic	*EMILIN2*	.	.	.
18	2862634	C	G	intronic	*EMILIN2*	.	.	.
18	2863501	T	G	intronic	*EMILIN2*	.	.	.
20	39993031	C	T	intronic	*EMILIN3*	.	.	.
11	62379373	C	CAG	intronic	*EML3*	.	.	.
14	89124562	T	C	exonic	*EML5*	synonymous_SNV	.	.
14	89162228	C	T	intronic	*EML5*	.	.	.
14	89120557	C	T	intronic	*EML5*	.	.	.
14	89120558	A	G	intronic	*EML5*	.	.	.
14	89121580	T	G	intronic	*EML5*	.	.	.
12	13403015	C	T	intergenic	*EMP1,LINC01559*	.	.	.
12	13489155	G	A	intergenic	*EMP1,LINC01559*	.	.	.
11	76248491	A	G	intronic	*EMSY*	.	.	.
11	76194562	G	GC	intronic	*EMSY*	.	.	.
11	76195426	T	G	intronic	*EMSY*	.	.	.
10	119621993	G	A	intergenic	*EMX2,RAB11FIP2*	.	.	.
10	119592640	C	T	intergenic	*EMX2,RAB11FIP2*	.	.	.
10	119594681	G	T	intergenic	*EMX2,RAB11FIP2*	.	.	.
10	119275548	C	A	ncRNA_intronic	*EMX2OS*	.	.	.
2	119635704	T	C	intergenic	*EN1,MARCO*	.	.	.
1	225679043	T	C	UTR3	*ENAH*	.	.	.
1	225893412	C	T	intergenic	*ENAH,SRP9*	.	.	.
11	94838810	T	C	intronic	*ENDOD1*	.	.	.
18	697049	G	A	intronic	*ENOSF1*	.	.	.
13	43824450	A	T	intronic	*ENOX1*	.	.	.
13	43938650	T	A	intronic	*ENOX1*	.	.	.
13	43879771	G	T	intronic	*ENOX1*	.	.	.
13	44075057	A	ATATT	intronic	*ENOX1*	.	.	.
13	44080291	T	C	intronic	*ENOX1*	.	.	.
13	44286956	A	G	intronic	*ENOX1*	.	.	.
13	44287307	T	C	intronic	*ENOX1*	.	.	.
X	129978561	G	GA	intronic	*ENOX2*	.	.	.
X	129952487	G	A	intronic	*ENOX2*	.	.	.
X	130140389	C	A	intergenic	*ENOX2,LINC01201*	.	.	.
X	130140390	A	G	intergenic	*ENOX2,LINC01201*	.	.	.
6	132241198	C	T	intergenic	*ENPP1,CTGF*	.	.	.

8	120576924	AT	A	intronic	*ENPP2*	.	.	.
8	120576926	AT	A	intronic	*ENPP2*	.	.	.
8	120705524	A	G	intergenic	*ENPP2,TAF2*	.	.	.
6	132000047	C	T	intronic	*ENPP3*	.	.	.
6	46184276	G	A	intergenic	*ENPP5,RCAN2*	.	.	.
6	46182173	T	A	intergenic	*ENPP5,RCAN2*	.	.	.
6	46182310	A	G	intergenic	*ENPP5,RCAN2*	.	.	.
4	185104653	TACAC	T	intronic	*ENPP6*	.	.	.
4	185009207	A	G	downstream	*ENPP6*	.	.	.
4	185009208	A	G	downstream	*ENPP6*	.	.	.
16	33571262	G	T	upstream	*ENPP7P13*	.	.	.
16	33573113	GTTGCCCCCGCCGCCGCGGCTTT	G	ncRNA_intronic	*ENPP7P13*	.	.	.
16	33848177	GCTTA	G	intergenic	*ENPP7P13,LINC00273*	.	.	.
16	33592899	G	T	intergenic	*ENPP7P13,LINC00273*	.	.	.
16	33620710	T	A	intergenic	*ENPP7P13,LINC00273*	.	.	.
16	33744605	G	T	intergenic	*ENPP7P13,LINC00273*	.	.	.
16	33784273	T	C	intergenic	*ENPP7P13,LINC00273*	.	.	.
16	33784274	G	T	intergenic	*ENPP7P13,LINC00273*	.	.	.
16	33836968	T	A	intergenic	*ENPP7P13,LINC00273*	.	.	.
16	33925009	T	A	intergenic	*ENPP7P13,LINC00273*	.	.	.
16	33837773	A	T	intergenic	*ENPP7P13,LINC00273*	.	.	.
16	33837774	G	C	intergenic	*ENPP7P13,LINC00273*	.	.	.
16	33848395	A	G	intergenic	*ENPP7P13,LINC00273*	.	.	.
16	33873433	G	C	intergenic	*ENPP7P13,LINC00273*	.	.	.

16	33862854	A	T	intergenic	*ENPP7P13,LINC00273*	.	.	.
16	33635356	G	T	intergenic	*ENPP7P13,LINC00273*	.	.	.
16	33643317	T	C	intergenic	*ENPP7P13,LINC00273*	.	.	.
16	33771155	A	G	intergenic	*ENPP7P13,LINC00273*	.	.	.
16	33771157	G	A	intergenic	*ENPP7P13,LINC00273*	.	.	.
16	33823853	C	A	intergenic	*ENPP7P13,LINC00273*	.	.	.
16	33824182	C	A	intergenic	*ENPP7P13,LINC00273*	.	.	.
16	33825379	T	G	intergenic	*ENPP7P13,LINC00273*	.	.	.
16	33825661	T	TTC	intergenic	*ENPP7P13,LINC00273*	.	.	.
16	33825662	AAG	A	intergenic	*ENPP7P13,LINC00273*	.	.	.
16	33910138	T	A	intergenic	*ENPP7P13,LINC00273*	.	.	.
16	33912757	T	C	intergenic	*ENPP7P13,LINC00273*	.	.	.
16	33929641	G	A	intergenic	*ENPP7P13,LINC00273*	.	.	.
16	33930293	C	T	intergenic	*ENPP7P13,LINC00273*	.	.	.
10	97483527	C	T	intronic	*ENTPD1*	.	.	.
10	97830131	C	A	ncRNA_intronic	*ENTPD1-AS1*	.	.	.
10	97534066	A	T	ncRNA_intronic	*ENTPD1-AS1*	.	.	.
10	97535439	A	C	ncRNA_intronic	*ENTPD1-AS1*	.	.	.
10	97536255	C	A	ncRNA_intronic	*ENTPD1-AS1*	.	.	.
10	97615779	C	T	ncRNA_intronic	*ENTPD1-AS1*	.	.	.
10	97688569	G	A	ncRNA_intronic	*ENTPD1-AS1*	.	.	.
10	97689979	C	T	ncRNA_intronic	*ENTPD1-AS1*	.	.	.
10	97691026	C	T	ncRNA_intronic	*ENTPD1-AS1*	.	.	.
14	74470444	C	T	intronic	*ENTPD5*	.	.	.

14	74451834	T	C	intronic	*ENTPD5*	.	.	.
9	140337129	T	C	intergenic	*ENTPD8,NSMF*	.	.	.
22	41564512	C	T	exonic	*EP300*	stopgain	.	.
12	132495310	C	A	intronic	*EP400*	.	.	.
12	132607811	G	A	ncRNA_intronic	*EP400NL*	.	.	.
2	46627442	T	A	intergenic	*EPAS1,LOC101805491*	.	.	.
2	46631298	A	AT	intergenic	*EPAS1,LOC101805491*	.	.	.
6	131231098	A	G	intronic	*EPB41L2*	.	.	.
6	131412468	G	T	intergenic	*EPB41L2,AKAP7*	.	.	.
18	5402966	C	G	intronic	*EPB41L3*	.	.	.
18	5736149	G	C	intergenic	*EPB41L3,MIR3976HG*	.	.	.
5	111603693	G	C	intronic	*EPB41L4A*	.	.	.
5	111704160	T	C	intronic	*EPB41L4A*	.	.	.
5	111601403	G	A	intronic	*EPB41L4A*	.	.	.
5	111542770	A	AC	intronic	*EPB41L4A*	.	.	.
5	111728025	A	C	intronic	*EPB41L4A*	.	.	.
5	111728258	T	C	intronic	*EPB41L4A*	.	.	.
5	111728850	C	T	intronic	*EPB41L4A*	.	.	.
5	111729196	A	G	intronic	*EPB41L4A*	.	.	.
5	111775289	C	A	intergenic	*EPB41L4A-AS2,LOC102467214*	.	.	.
5	111800224	C	G	intergenic	*EPB41L4A-AS2,LOC102467214*	.	.	.
5	111842043	G	A	intergenic	*EPB41L4A-AS2,LOC102467214*	.	.	.
5	111842296	T	G	intergenic	*EPB41L4A-AS2,LOC102467214*	.	.	.

5	111843629	A	T	intergenic	*EPB41L4A-AS2,LOC102467214*	.	.	.
5	111843630	A	T	intergenic	*EPB41L4A-AS2,LOC102467214*	.	.	.
5	111852480	G	A	intergenic	*EPB41L4A-AS2,LOC102467214*	.	.	.
5	111852481	C	T	intergenic	*EPB41L4A-AS2,LOC102467214*	.	.	.
5	111854549	C	G	intergenic	*EPB41L4A-AS2,LOC102467214*	.	.	.
5	111877410	A	G	intergenic	*EPB41L4A-AS2,LOC102467214*	.	.	.
5	111879430	A	G	intergenic	*EPB41L4A-AS2,LOC102467214*	.	.	.
9	112011352	A	AATAT	intronic	*EPB41L4B*	.	.	.
2	120780122	G	T	intronic	*EPB41L5*	.	.	.
2	120781302	G	T	intronic	*EPB41L5*	.	.	.
2	120781662	G	A	intronic	*EPB41L5*	.	.	.
2	120829550	T	C	intronic	*EPB41L5*	.	.	.
2	120839232	T	G	intronic	*EPB41L5*	.	.	.
2	120876915	A	G	intronic	*EPB41L5*	.	.	.
2	120943530	A	G	intergenic	*EPB41L5,TMEM185B*	.	.	.
2	149441744	C	T	intronic	*EPC2*	.	.	.
2	149465082	G	A	intronic	*EPC2*	.	.	.
2	149467007	A	C	intronic	*EPC2*	.	.	.
2	149629936	A	G	intergenic	*EPC2,KIF5C*	.	.	.
2	149548870	T	A	intergenic	*EPC2,KIF5C*	.	.	.
7	37967004	C	A	intronic	*EPDR1*	.	.	.
7	38084749	C	A	intergenic	*EPDR1,STARD3NL*	.	.	.
18	43432245	AACAC	A	UTR3	*EPG5*	.	.	.

18	43559791	A	G	intergenic	*EPG5,PSTPIP2*	.	.	.
1	38234268	G	A	intergenic	*EPHA10,MANEAL*	.	.	.
1	38237061	A	G	intergenic	*EPHA10,MANEAL*	.	.	.
3	89510081	G	C	intronic	*EPHA3*	.	.	.
3	89475251	T	C	intronic	*EPHA3*	.	.	.
3	89461875	C	T	intronic	*EPHA3*	.	.	.
3	89394613	C	A	intronic	*EPHA3*	.	.	.
3	89394614	C	A	intronic	*EPHA3*	.	.	.
3	89183772	C	G	intronic	*EPHA3*	.	.	.
3	89935185	AC	A	intergenic	*EPHA3,NONE*	.	.	.
3	89935187	A	G	intergenic	*EPHA3,NONE*	.	.	.
3	89662234	G	T	intergenic	*EPHA3,NONE*	.	.	.
3	89654716	A	T	intergenic	*EPHA3,NONE*	.	.	.
3	90037519	C	A	intergenic	*EPHA3,NONE*	.	.	.
3	90134137	A	G	intergenic	*EPHA3,NONE*	.	.	.
3	89799749	A	T	intergenic	*EPHA3,NONE*	.	.	.
3	89799750	G	A	intergenic	*EPHA3,NONE*	.	.	.
3	89881789	C	T	intergenic	*EPHA3,NONE*	.	.	.
3	89883646	C	T	intergenic	*EPHA3,NONE*	.	.	.
3	89886653	G	A	intergenic	*EPHA3,NONE*	.	.	.
3	89946613	G	A	intergenic	*EPHA3,NONE*	.	.	.
3	89948814	T	G	intergenic	*EPHA3,NONE*	.	.	.
3	89948816	C	A	intergenic	*EPHA3,NONE*	.	.	.
3	90012061	A	G	intergenic	*EPHA3,NONE*	.	.	.
3	90012062	G	A	intergenic	*EPHA3,NONE*	.	.	.
3	90142815	GTT	G	intergenic	*EPHA3,NONE*	.	.	.
3	90237716	G	A	intergenic	*EPHA3,NONE*	.	.	.
3	90239586	A	G	intergenic	*EPHA3,NONE*	.	.	.
3	90321865	A	C	intergenic	*EPHA3,NONE*	.	.	.
3	90326137	T	A	intergenic	*EPHA3,NONE*	.	.	.
3	90362222	C	A	intergenic	*EPHA3,NONE*	.	.	.
3	90362223	T	G	intergenic	*EPHA3,NONE*	.	.	.
3	90368380	A	T	intergenic	*EPHA3,NONE*	.	.	.
3	90424993	A	C	intergenic	*EPHA3,NONE*	.	.	.
3	90432412	A	G	intergenic	*EPHA3,NONE*	.	.	.
2	222592560	C	T	intergenic	*EPHA4,PAX3*	.	.	.
2	222604517	T	C	intergenic	*EPHA4,PAX3*	.	.	.
2	222741130	C	T	intergenic	*EPHA4,PAX3*	.	.	.

2	222741131	A	G	intergenic	*EPHA4,PAX3*	.	.	.
2	222852051	A	G	intergenic	*EPHA4,PAX3*	.	.	.
2	222854215	T	A	intergenic	*EPHA4,PAX3*	.	.	.
2	222866954	T	C	intergenic	*EPHA4,PAX3*	.	.	.
4	66235134	T	G	intronic	*EPHA5*	.	.	.
4	66254591	G	A	intronic	*EPHA5*	.	.	.
4	66409661	C	T	intronic	*EPHA5*	.	.	.
4	66409662	A	G	intronic	*EPHA5*	.	.	.
4	66899266	A	G	intergenic	*EPHA5-AS1,MIR1269A*	.	.	.
4	67017699	GTTC	G	intergenic	*EPHA5-AS1,MIR1269A*	.	.	.
4	67133558	A	G	intergenic	*EPHA5-AS1,MIR1269A*	.	.	.
4	66610019	A	G	intergenic	*EPHA5-AS1,MIR1269A*	.	.	.
4	66592503	G	C	intergenic	*EPHA5-AS1,MIR1269A*	.	.	.
4	66743007	A	G	intergenic	*EPHA5-AS1,MIR1269A*	.	.	.
4	66890554	G	A	intergenic	*EPHA5-AS1,MIR1269A*	.	.	.
4	66890556	A	T	intergenic	*EPHA5-AS1,MIR1269A*	.	.	.
4	66981247	G	A	intergenic	*EPHA5-AS1,MIR1269A*	.	.	.
4	66998836	A	T	intergenic	*EPHA5-AS1,MIR1269A*	.	.	.
4	67135314	G	A	intergenic	*EPHA5-AS1,MIR1269A*	.	.	.
4	67135315	G	C	intergenic	*EPHA5-AS1,MIR1269A*	.	.	.
3	96978345	C	A	intronic	*EPHA6*	.	.	.
3	97042258	A	G	intronic	*EPHA6*	.	.	.
3	96991332	A	G	intronic	*EPHA6*	.	.	.
3	96658930	C	T	intronic	*EPHA6*	.	.	.
3	96701005	A	G	intronic	*EPHA6*	.	.	.
3	96701683	T	G	intronic	*EPHA6*	.	.	.

3	97050150	G	A	intronic	*EPHA6*	.	.	.
3	97224815	C	T	intronic	*EPHA6*	.	.	.
3	97224816	A	G	intronic	*EPHA6*	.	.	.
3	97224929	T	C	intronic	*EPHA6*	.	.	.
3	97261979	A	AT	intronic	*EPHA6*	.	.	.
3	97412508	C	T	intronic	*EPHA6*	.	.	.
6	94117857	T	G	intronic	*EPHA7*	.	.	.
6	94006802	T	G	intronic	*EPHA7*	.	.	.
6	94268990	TA	T	intergenic	*EPHA7,TSG1*	.	.	.
6	94190910	G	A	intergenic	*EPHA7,TSG1*	.	.	.
6	94244368	T	G	intergenic	*EPHA7,TSG1*	.	.	.
6	94257008	G	T	intergenic	*EPHA7,TSG1*	.	.	.
6	94257366	T	C	intergenic	*EPHA7,TSG1*	.	.	.
6	94257738	T	C	intergenic	*EPHA7,TSG1*	.	.	.
6	94328815	G	C	intergenic	*EPHA7,TSG1*	.	.	.
6	94328816	G	A	intergenic	*EPHA7,TSG1*	.	.	.
3	134929472	T	A	intronic	*EPHB1*	.	.	.
3	134557908	G	A	intronic	*EPHB1*	.	.	.
3	135357603	G	C	intergenic	*EPHB1,PPP2R3A*	.	.	.
3	135398664	C	A	intergenic	*EPHB1,PPP2R3A*	.	.	.
3	135387468	ATTT	A	intergenic	*EPHB1,PPP2R3A*	.	.	.
3	135523778	G	A	intergenic	*EPHB1,PPP2R3A*	.	.	.
3	135649348	A	G	intergenic	*EPHB1,PPP2R3A*	.	.	.
3	135221054	T	A	intergenic	*EPHB1,PPP2R3A*	.	.	.
3	135537281	A	G	intergenic	*EPHB1,PPP2R3A*	.	.	.
3	135027839	ATGTGTG	A	intergenic	*EPHB1,PPP2R3A*	.	.	.
1	23146069	C	G	intronic	*EPHB2*	.	.	.
8	27361024	G	A	intronic	*EPHX2*	.	.	.
6	146051797	G	A	intronic	*EPM2A*	.	.	.
6	145989267	A	T	intronic	*EPM2A*	.	.	.
6	145996936	T	C	intronic	*EPM2A*	.	.	.
6	145998646	T	A	intronic	*EPM2A*	.	.	.
6	145998876	T	C	intronic	*EPM2A*	.	.	.
6	145999647	T	G	intronic	*EPM2A*	.	.	.
6	146002813	A	C	intronic	*EPM2A*	.	.	.
6	146031340	C	T	intronic	*EPM2A*	.	.	.
1	51848338	G	A	intronic	*EPS15*	.	.	.
1	51943180	C	T	intronic	*EPS15*	.	.	.

1	51943182	G	T	intronic	*EPS15*	.	.	.
19	16554947	T	A	intronic	*EPS15L1*	.	.	.
12	15895994	G	A	intronic	*EPS8*	.	.	.
12	15896815	T	C	intronic	*EPS8*	.	.	.
12	15921114	T	C	intronic	*EPS8*	.	.	.
1	110302778	G	C	intronic	*EPS8L3*	.	.	.
1	110355870	T	TCTC	intergenic	*EPS8L3,CSF1*	.	.	.
13	43518473	A	C	intronic	*EPSTI1*	.	.	.
12	91412856	A	G	intergenic	*EPYC,KERA*	.	.	.
9	27317841	C	T	intergenic	*EQTN,MOB3B*	.	.	.
9	27317842	C	T	intergenic	*EQTN,MOB3B*	.	.	.
9	27319661	C	T	intergenic	*EQTN,MOB3B*	.	.	.
5	96182669	T	C	intergenic	*ERAP1,ERAP2*	.	.	.
5	96178206	G	T	intergenic	*ERAP1,ERAP2*	.	.	.
5	96233792	C	T	intronic	*ERAP2*	.	.	.
5	65357090	G	GCTC	intronic	*ERBB2IP*	.	.	.
5	65301635	G	A	intronic	*ERBB2IP*	.	.	.
5	65302902	T	A	intronic	*ERBB2IP*	.	.	.
5	65354903	G	A	intronic	*ERBB2IP*	.	.	.
5	65355624	G	C	intronic	*ERBB2IP*	.	.	.
5	65356170	T	A	intronic	*ERBB2IP*	.	.	.
5	65423507	A	AGTGCTG	intergenic	*ERBB2IP,SREK1*	.	.	.
5	65406072	C	A	intergenic	*ERBB2IP,SREK1*	.	.	.
2	212977133	G	A	intronic	*ERBB4*	.	.	.
2	213229638	AGTGTGT	A	intronic	*ERBB4*	.	.	.
2	213216711	T	TA	intronic	*ERBB4*	.	.	.
2	212449258	G	T	intronic	*ERBB4*	.	.	.
2	212444421	C	G	intronic	*ERBB4*	.	.	.
2	212790255	C	T	intronic	*ERBB4*	.	.	.
2	212886207	G	A	intronic	*ERBB4*	.	.	.
2	212886208	C	T	intronic	*ERBB4*	.	.	.
2	212923283	T	C	intronic	*ERBB4*	.	.	.
2	212924281	T	C	intronic	*ERBB4*	.	.	.
2	213031862	T	C	intronic	*ERBB4*	.	.	.
2	213100450	T	A	intronic	*ERBB4*	.	.	.
2	213658767	C	CCTCTCT	intergenic	*ERBB4,LOC102725079*	.	.	.

2	213448130	T	C	intergenic	*ERBB4,LOC102725079*	.	.	.
2	213448244	T	C	intergenic	*ERBB4,LOC102725079*	.	.	.
2	213448422	T	C	intergenic	*ERBB4,LOC102725079*	.	.	.
2	213620637	T	C	intergenic	*ERBB4,LOC102725079*	.	.	.
2	213620861	T	C	intergenic	*ERBB4,LOC102725079*	.	.	.
12	1501303	T	C	intronic	*ERC1*	.	.	.
12	1347196	A	G	intronic	*ERC1*	.	.	.
12	1503201	AAAAAAAAAACCAC	A	intronic	*ERC1*	.	.	.
12	1593050	G	A	intronic	*ERC1*	.	.	.
12	1228708	TGTGTGTGTGTGTGTGTGTGTGTGTGTGA	T	intronic	*ERC1*	.	.	.
12	1290781	G	A	intronic	*ERC1*	.	.	.
3	55935673	A	C	intronic	*ERC2*	.	.	.
3	55863205	C	A	intronic	*ERC2*	.	.	.
3	56394822	G	T	intronic	*ERC2*	.	.	.
3	55853709	G	T	intronic	*ERC2*	.	.	.
3	56498861	C	T	intronic	*ERC2*	.	.	.
3	55870897	G	A	intronic	*ERC2*	.	.	.
3	56100287	G	T	intronic	*ERC2*	.	.	.
3	56100288	T	C	intronic	*ERC2*	.	.	.
3	56100976	A	G	intronic	*ERC2*	.	.	.
19	45862635	C	A	intronic	*ERCC2*	.	.	.
19	45880938	G	A	intergenic	*ERCC2,PPP1R13L*	.	.	.
10	50786150	T	C	intergenic	*ERCC6-PGBD3,CHAT*	.	.	.
10	50774607	A	T	intergenic	*ERCC6-PGBD3,CHAT*	.	.	.
10	50791061	A	ATATCTTTCT	intergenic	*ERCC6-PGBD3,CHAT*	.	.	.

10	50734151	ATTTATATATT	A	intronic	*ERCC6,ERCC6-PGBD3*	.	.	.
X	71457985	A	G	intronic	*ERCC6L,PIN4*	.	.	.
X	71441693	G	A	intronic	*ERCC6L,PIN4*	.	.	.
5	60172660	T	C	intronic	*ERCC8*	.	.	.
5	60174055	T	A	intronic	*ERCC8*	.	.	.
5	60208112	A	T	intronic	*ERCC8*	.	.	.
5	60208259	T	C	intronic	*ERCC8*	.	.	.
5	60208260	G	A	intronic	*ERCC8*	.	.	.
4	75259920	C	A	intergenic	*EREG,AREG*	.	.	.
4	75259922	A	T	intergenic	*EREG,AREG*	.	.	.
21	39982450	CGTGTGT	C	intronic	*ERG*	.	.	.
21	40038667	G	A	intergenic	*ERG,LINC00114*	.	.	.
20	34139604	G	A	intronic	*ERGIC3*	.	.	.
1	44863921	A	G	intergenic	*ERI3,RNF220*	.	.	.
1	44863923	A	T	intergenic	*ERI3,RNF220*	.	.	.
8	572804	A	T	intronic	*ERICH1*	.	.	.
8	990357	A	G	ncRNA_intronic	*ERICH1-AS1*	.	.	.
8	958506	T	C	ncRNA_intronic	*ERICH1-AS1*	.	.	.
8	990628	A	G	ncRNA_intronic	*ERICH1-AS1*	.	.	.
8	1056422	C	T	ncRNA_intronic	*ERICH1-AS1*	.	.	.
8	968183	G	C	ncRNA_intronic	*ERICH1-AS1*	.	.	.
8	1058874	A	G	ncRNA_intronic	*ERICH1-AS1*	.	.	.
8	701039	A	G	ncRNA_intronic	*ERICH1-AS1*	.	.	.
8	1213597	A	T	intergenic	*ERICH1-AS1,LOC286083*	.	.	.
8	1134506	G	C	intergenic	*ERICH1-AS1,LOC286083*	.	.	.
8	1191339	G	C	intergenic	*ERICH1-AS1,LOC286083*	.	.	.
8	99088344	C	T	intronic	*ERICH5*	.	.	.
13	46131972	C	T	intronic	*ERICH6B*	.	.	.
13	46228234	A	T	intergenic	*ERICH6B,LINC01055*	.	.	.
13	46241939	T	C	intergenic	*ERICH6B,LINC01055*	.	.	.
13	46191538	C	G	intergenic	*ERICH6B,LINC01055*	.	.	.

13	46194222	C	A	intergenic	*ERICH6B,LINC01055*	.	.	.
13	46211133	C	T	intergenic	*ERICH6B,LINC01055*	.	.	.
13	46211135	T	G	intergenic	*ERICH6B,LINC01055*	.	.	.
13	46212764	G	A	intergenic	*ERICH6B,LINC01055*	.	.	.
13	46237207	T	C	intergenic	*ERICH6B,LINC01055*	.	.	.
13	46240970	C	T	intergenic	*ERICH6B,LINC01055*	.	.	.
2	158257823	T	A	intergenic	*ERMN,CYTIP*	.	.	.
9	5824623	A	G	intronic	*ERMP1*	.	.	.
17	62166745	T	A	intronic	*ERN1*	.	.	.
1	236465847	T	C	intergenic	*ERO1B,EDARADD*	.	.	.
9	102748579	A	T	intronic	*ERP44*	.	.	.
9	102745277	A	G	intronic	*ERP44*	.	.	.
9	102753027	A	T	intronic	*ERP44*	.	.	.
9	102755351	A	G	intronic	*ERP44*	.	.	.
9	102755353	A	G	intronic	*ERP44*	.	.	.
1	8126425	C	T	intergenic	*ERRFI1,LOC102724539*	.	.	.
19	53525356	T	A	intergenic	*ERVV-1,ERVV-2*	.	.	.
5	54295872	A	G	intergenic	*ESM1,LOC102467081*	.	.	.
5	54313245	G	A	intergenic	*ESM1,LOC102467081*	.	.	.
1	17025975	G	A	ncRNA_exonic	*ESPNP*	.	.	.
6	152034929	T	G	intronic	*ESR1*	.	.	.
6	152035903	C	T	intronic	*ESR1*	.	.	.
6	152035904	A	G	intronic	*ESR1*	.	.	.
6	152036558	A	C	intronic	*ESR1*	.	.	.
6	152036560	A	G	intronic	*ESR1*	.	.	.
6	152169099	T	C	intronic	*ESR1*	.	.	.
6	152192663	C	T	intronic	*ESR1*	.	.	.
6	152192664	A	G	intronic	*ESR1*	.	.	.
6	152352480	T	C	intronic	*ESR1*	.	.	.

6	152353875	T	C	intronic	*ESR1*	.	.	.
6	152400562	GAAAGGAA TAGCATCA ACATCAAC AA	G	intronic	*ESR1*	.	.	.
6	152402980	T	A	intronic	*ESR1*	.	.	.
8	95675040	A	G	intronic	*ESRP1*	.	.	.
14	76921313	T	TGGTGAGGA	intronic	*ESRRB*	.	.	.
14	77119047	G	T	intergenic	*ESRRB,VASH1*	.	.	.
1	216709209	A	G	intronic	*ESRRG*	.	.	.
1	216701966	A	ATT	intronic	*ESRRG*	.	.	.
1	216992323	A	G	intronic	*ESRRG*	.	.	.
1	217143550	G	C	intronic	*ESRRG*	.	.	.
1	217586858	A	T	intergenic	*ESRRG,GPATCH2*	.	.	.
1	217389341	C	T	intergenic	*ESRRG,GPATCH2*	.	.	.
1	217430744	G	A	intergenic	*ESRRG,GPATCH2*	.	.	.
1	217519820	G	A	intergenic	*ESRRG,GPATCH2*	.	.	.
1	217558206	T	A	intergenic	*ESRRG,GPATCH2*	.	.	.
X	103774493	C	T	intergenic	*ESX1,IL1RAPL2*	.	.	.
X	103678534	A	T	intergenic	*ESX1,IL1RAPL2*	.	.	.
X	103583295	G	A	intergenic	*ESX1,IL1RAPL2*	.	.	.
X	103605675	T	A	intergenic	*ESX1,IL1RAPL2*	.	.	.
X	103608107	A	G	intergenic	*ESX1,IL1RAPL2*	.	.	.
X	103608471	G	C	intergenic	*ESX1,IL1RAPL2*	.	.	.
2	67636886	T	C	intronic	*ETAA1*	.	.	.
2	67761819	C	G	intergenic	*ETAA1,LOC101927 701*	.	.	.
2	67996621	C	A	intergenic	*ETAA1,LOC101927 701*	.	.	.
2	67697324	C	T	intergenic	*ETAA1,LOC101927 701*	.	.	.
2	67802202	A	G	intergenic	*ETAA1,LOC101927 701*	.	.	.
2	67819047	C	A	intergenic	*ETAA1,LOC101927 701*	.	.	.
2	67819104	T	C	intergenic	*ETAA1,LOC101927 701*	.	.	.

2	67821046	A	G	intergenic	*ETAA1,LOC101927701*	.	.	.
2	67850830	A	G	intergenic	*ETAA1,LOC101927701*	.	.	.
15	76552660	C	G	intronic	*ETFA*	.	.	.
12	22829695	T	C	intronic	*ETNK1*	.	.	.
12	22830337	A	G	intronic	*ETNK1*	.	.	.
12	23270054	ATGTG	A	intergenic	*ETNK1,LOC101928441*	.	.	.
12	23132359	T	TAA	intergenic	*ETNK1,LOC101928441*	.	.	.
12	23244778	A	G	intergenic	*ETNK1,LOC101928441*	.	.	.
12	23289160	T	C	intergenic	*ETNK1,LOC101928441*	.	.	.
12	23303951	T	C	intergenic	*ETNK1,LOC101928441*	.	.	.
12	23310169	C	T	intergenic	*ETNK1,LOC101928441*	.	.	.
12	23310422	C	T	intergenic	*ETNK1,LOC101928441*	.	.	.
12	23310423	A	G	intergenic	*ETNK1,LOC101928441*	.	.	.
11	128520970	T	C	intergenic	*ETS1,FLI1*	.	.	.
7	13970894	T	C	intronic	*ETV1*	.	.	.
7	14121642	T	C	intergenic	*ETV1,DGKB*	.	.	.
7	14122504	A	T	intergenic	*ETV1,DGKB*	.	.	.
7	14083679	C	G	intergenic	*ETV1,DGKB*	.	.	.
7	14084035	C	T	intergenic	*ETV1,DGKB*	.	.	.
7	14084625	T	C	intergenic	*ETV1,DGKB*	.	.	.
1	157162191	G	A	intergenic	*ETV3,FCRL5*	.	.	.
1	157181509	T	C	intergenic	*ETV3,FCRL5*	.	.	.
1	157181809	G	C	intergenic	*ETV3,FCRL5*	.	.	.
1	157181810	G	A	intergenic	*ETV3,FCRL5*	.	.	.
1	157226139	A	C	intergenic	*ETV3,FCRL5*	.	.	.
1	157265674	C	T	intergenic	*ETV3,FCRL5*	.	.	.
1	157269113	C	A	intergenic	*ETV3,FCRL5*	.	.	.
1	157290511	G	C	intergenic	*ETV3,FCRL5*	.	.	.

1	157290512	C	T	intergenic	*ETV3,FCRL5*	.	.	.
1	157291229	T	C	intergenic	*ETV3,FCRL5*	.	.	.
1	157309715	C	T	intergenic	*ETV3,FCRL5*	.	.	.
1	157309957	C	T	intergenic	*ETV3,FCRL5*	.	.	.
1	157311247	G	A	intergenic	*ETV3,FCRL5*	.	.	.
17	41645549	T	C	intergenic	*ETV4,MEOX1*	.	.	.
17	41645550	G	A	intergenic	*ETV4,MEOX1*	.	.	.
17	41656102	TACACAC	T	intergenic	*ETV4,MEOX1*	.	.	.
12	12089683	G	A	intergenic	*ETV6,BCL2L14*	.	.	.
4	5730006	A	G	intronic	*EVC*	.	.	.
4	5564495	T	A	UTR3	*EVC2*	.	.	.
4	5648304	T	C	intronic	*EVC2*	.	.	.
4	5600332	T	C	intronic	*EVC2*	.	.	.
4	5601054	G	T	intronic	*EVC2*	.	.	.
4	5643911	C	T	intronic	*EVC2*	.	.	.
1	93035489	C	T	intronic	*EVI5*	.	.	.
1	92997826	G	A	intronic	*EVI5*	.	.	.
1	93105349	G	A	intronic	*EVI5*	.	.	.
1	93152748	T	G	intronic	*EVI5*	.	.	.
1	93185572	A	G	intronic	*EVI5*	.	.	.
1	93186225	A	G	intronic	*EVI5*	.	.	.
1	93262325	TTTTA	T	intergenic	*EVI5,RPL5*	.	.	.
1	93261194	A	G	intergenic	*EVI5,RPL5*	.	.	.
19	7908280	TAG	T	intronic	*EVI5L*	.	.	.
7	27480169	G	A	intergenic	*EVX1,HIBADH*	.	.	.
7	27518615	T	TTA	intergenic	*EVX1,HIBADH*	.	.	.
7	27331849	A	G	intergenic	*EVX1,HIBADH*	.	.	.
7	27331908	A	G	intergenic	*EVX1,HIBADH*	.	.	.
15	41521441	C	T	intronic	*EXD1*	.	.	.
14	69685782	A	G	intronic	*EXD2*	.	.	.
14	69685783	A	G	intronic	*EXD2*	.	.	.
4	56788282	C	T	intergenic	*EXOC1,CEP135*	.	.	.
6	591397	T	C	intronic	*EXOC2*	.	.	.
6	687879	A	G	intronic	*EXOC2*	.	.	.
6	508469	C	T	intronic	*EXOC2*	.	.	.
6	765263	T	C	intergenic	*EXOC2,LOC101927691*	.	.	.

6	824789	T	C	intergenic	*EXOC2,LOC101927691*	.	.	.
19	45743525	C	CTTAA	intergenic	*EXOC3L2,MARK4*	.	.	.
14	103583530	A	T	intergenic	*EXOC3L4,TNFAIP2*	.	.	.
7	133143625	A	C	intronic	*EXOC4*	.	.	.
7	133121429	T	C	intronic	*EXOC4*	.	.	.
7	133227922	AT	A	intronic	*EXOC4*	.	.	.
7	133502130	C	T	exonic	*EXOC4*	nonsynonymous_SNV	.	.
7	133134539	G	A	intronic	*EXOC4*	.	.	.
7	133683518	G	T	intronic	*EXOC4*	.	.	.
7	133008511	G	A	intronic	*EXOC4*	.	.	.
7	133014070	T	C	intronic	*EXOC4*	.	.	.
7	133064927	T	G	intronic	*EXOC4*	.	.	.
7	133209291	A	T	intronic	*EXOC4*	.	.	.
7	133650535	T	G	intronic	*EXOC4*	.	.	.
7	133650776	T	C	intronic	*EXOC4*	.	.	.
7	133652752	GA	G	intronic	*EXOC4*	.	.	.
7	133676524	A	G	intronic	*EXOC4*	.	.	.
14	57704613	C	A	intronic	*EXOC5*	.	.	.
10	94801656	G	C	intronic	*EXOC6*	.	.	.
2	72443564	T	C	intronic	*EXOC6B*	.	.	.
2	72443565	G	A	intronic	*EXOC6B*	.	.	.
2	72595883	T	A	intronic	*EXOC6B*	.	.	.
2	72634225	G	A	intronic	*EXOC6B*	.	.	.
2	72698598	G	T	intronic	*EXOC6B*	.	.	.
2	72872734	C	T	intronic	*EXOC6B*	.	.	.
2	72874137	G	A	intronic	*EXOC6B*	.	.	.
2	72875325	T	C	intronic	*EXOC6B*	.	.	.
2	72931603	A	G	intronic	*EXOC6B*	.	.	.
8	118943704	G	T	intronic	*EXT1*	.	.	.
8	119057863	G	T	intronic	*EXT1*	.	.	.
11	44236317	A	T	intronic	*EXT2*	.	.	.
8	72113709	G	C	intronic	*EYA1*	.	.	.
8	72394938	G	T	intergenic	*EYA1,MSC*	.	.	.
8	72394405	G	T	intergenic	*EYA1,MSC*	.	.	.
8	72366567	G	A	intergenic	*EYA1,MSC*	.	.	.

8	72368482	A	G	intergenic	*EYA1,MSC*	.	.	.
8	72497163	T	C	intergenic	*EYA1,MSC*	.	.	.
8	72655176	C	T	intergenic	*EYA1,MSC*	.	.	.
8	72736141	G	C	intergenic	*EYA1,MSC*	.	.	.
20	45711692	C	T	intronic	*EYA2*	.	.	.
20	45750919	A	T	intronic	*EYA2*	.	.	.
1	28418675	A	AAT	intergenic	*EYA3,PTAFR*	.	.	.
1	28428517	C	T	intergenic	*EYA3,PTAFR*	.	.	.
6	133671780	G	C	intronic	*EYA4*	.	.	.
6	133677195	AAGAG	A	intronic	*EYA4*	.	.	.
6	133794079	G	T	intronic	*EYA4*	.	.	.
6	64951095	T	G	intronic	*EYS*	.	.	.
6	66313622	C	T	intronic	*EYS*	.	.	.
6	66196422	T	G	intronic	*EYS*	.	.	.
6	64440384	T	A	intronic	*EYS*	.	.	.
6	64447933	T	C	intronic	*EYS*	.	.	.
6	64551986	T	C	intronic	*EYS*	.	.	.
6	64552596	A	AT	intronic	*EYS*	.	.	.
6	64561383	T	A	intronic	*EYS*	.	.	.
6	64561385	T	C	intronic	*EYS*	.	.	.
6	64583836	G	T	intronic	*EYS*	.	.	.
6	64901301	C	T	intronic	*EYS*	.	.	.
6	64901303	A	C	intronic	*EYS*	.	.	.
6	64901907	T	C	intronic	*EYS*	.	.	.
6	64909374	G	A	intronic	*EYS*	.	.	.
6	65024779	G	A	intronic	*EYS*	.	.	.
6	65024798	A	G	intronic	*EYS*	.	.	.
6	65054840	T	C	intronic	*EYS*	.	.	.
6	65102433	A	C	intronic	*EYS*	.	.	.
6	65104659	A	C	intronic	*EYS*	.	.	.
6	65116730	T	C	intronic	*EYS*	.	.	.
6	65116731	G	A	intronic	*EYS*	.	.	.
6	65160010	G	A	intronic	*EYS*	.	.	.
6	65280577	T	G	intronic	*EYS*	.	.	.
6	65283808	T	C	intronic	*EYS*	.	.	.
6	65346120	A	G	intronic	*EYS*	.	.	.
6	65373777	C	T	intronic	*EYS*	.	.	.
6	65441028	A	G	intronic	*EYS*	.	.	.

6	65563877	C	A	intronic	*EYS*	.	.	.
6	65563879	G	A	intronic	*EYS*	.	.	.
6	65564224	A	C	intronic	*EYS*	.	.	.
6	66148689	T	C	intronic	*EYS*	.	.	.
6	66148691	C	G	intronic	*EYS*	.	.	.
6	66197834	T	G	intronic	*EYS*	.	.	.
6	66222650	G	A	intronic	*EYS*	.	.	.
6	66304722	C	T	intronic	*EYS*	.	.	.
6	66330199	C	T	intronic	*EYS*	.	.	.
7	148562076	G	A	intronic	*EZH2*	.	.	.
7	148656826	A	G	intergenic	*EZH2,GHET1*	.	.	.
4	187307122	T	C	ncRNA_intronic	*F11-AS1*	.	.	.
4	187319571	A	T	ncRNA_intronic	*F11-AS1*	.	.	.
1	197014257	C	A	intronic	*F13B*	.	.	.
5	76077857	C	CTCTCTCTCT	intergenic	*F2R,F2RL1*	.	.	.
1	95040062	A	T	intergenic	*F3,LINC01057*	.	.	.
1	169524406	C	T	intronic	*F5*	.	.	.
X	154098756	C	T	intronic	*F8*	.	.	.
X	154149599	G	A	intronic	*F8*	.	.	.
X	154204788	C	T	intronic	*F8*	.	.	.
X	154204789	A	G	intronic	*F8*	.	.	.
X	154680132	C	T	intergenic	*F8A1,F8A1*	.	.	.
X	154636537	A	G	intergenic	*F8A1,F8A1*	.	.	.
X	154656134	G	C	intergenic	*F8A1,F8A1*	.	.	.
X	154656240	A	C	intergenic	*F8A1,F8A1*	.	.	.
X	138658508	T	A	intergenic	*F9,MCF2*	.	.	.
X	57483869	G	T	intronic	*FAAH2*	.	.	.
X	57327055	G	A	intronic	*FAAH2*	.	.	.
X	57345329	T	A	intronic	*FAAH2*	.	.	.
X	57345550	A	G	intronic	*FAAH2*	.	.	.
X	57404298	G	T	intronic	*FAAH2*	.	.	.
X	57408980	T	C	intronic	*FAAH2*	.	.	.
X	57410337	T	C	intronic	*FAAH2*	.	.	.
X	57410338	G	A	intronic	*FAAH2*	.	.	.
X	57441689	T	C	intronic	*FAAH2*	.	.	.
X	57442205	A	G	intronic	*FAAH2*	.	.	.
X	57442207	C	T	intronic	*FAAH2*	.	.	.
X	57467543	T	C	intronic	*FAAH2*	.	.	.

X	57501859	T	C	intronic	*FAAH2*	.	.	.
X	57501860	T	C	intronic	*FAAH2*	.	.	.
X	57533910	G	C	intergenic	*FAAH2,ZXDB*	.	.	.
X	57549022	T	G	intergenic	*FAAH2,ZXDB*	.	.	.
X	57584402	C	G	intergenic	*FAAH2,ZXDB*	.	.	.
X	57584403	A	C	intergenic	*FAAH2,ZXDB*	.	.	.
X	57589476	G	A	intergenic	*FAAH2,ZXDB*	.	.	.
X	57594384	C	A	intergenic	*FAAH2,ZXDB*	.	.	.
X	57597789	C	G	intergenic	*FAAH2,ZXDB*	.	.	.
X	57597791	T	C	intergenic	*FAAH2,ZXDB*	.	.	.
X	57599641	G	A	intergenic	*FAAH2,ZXDB*	.	.	.
8	82479503	TATC	T	intergenic	*FABP12,IMPA1*	.	.	.
4	120246302	A	T	intergenic	*FABP2,LINC01061*	.	.	.
4	120302777	A	G	intergenic	*FABP2,LINC01061*	.	.	.
8	82235645	T	C	intergenic	*FABP5,PMP2*	.	.	.
11	61591753	C	T	intronic	*FADS2*	.	.	.
1	51054336	G	T	intronic	*FAF1*	.	.	.
1	51219650	G	A	intronic	*FAF1*	.	.	.
1	50958767	C	T	intronic	*FAF1*	.	.	.
1	50978411	AT	A	intronic	*FAF1*	.	.	.
1	51095593	A	G	intronic	*FAF1*	.	.	.
1	51226785	A	G	intronic	*FAF1*	.	.	.
1	51274606	C	G	intronic	*FAF1*	.	.	.
1	51352830	G	T	intronic	*FAF1*	.	.	.
1	51353931	C	G	intronic	*FAF1*	.	.	.
1	51371332	G	T	intronic	*FAF1*	.	.	.
1	51374586	T	A	intronic	*FAF1*	.	.	.
1	51374588	C	T	intronic	*FAF1*	.	.	.
5	175942145	G	A	intergenic	*FAF2,RNF44*	.	.	.
5	175942147	G	T	intergenic	*FAF2,RNF44*	.	.	.
2	97772160	G	A	intergenic	*FAHD2B,ANKRD36*	.	.	.
2	96681770	G	A	ncRNA_intronic	*FAHD2CP*	.	.	.
12	50296198	T	A	intronic	*FAIM2*	.	.	.
1	150493119	T	C	intergenic	*FALEC,ADAMTSL4*	.	.	.
10	14725809	G	A	intronic	*FAM107B*	.	.	.
10	14763915	C	T	intronic	*FAM107B*	.	.	.
8	58967785	T	C	intronic	*FAM110B*	.	.	.
8	58976519	A	G	intronic	*FAM110B*	.	.	.

8	59009094	G	A	intronic	*FAM110B*	.	.	.
8	59089387	CA	C	intergenic	*FAM110B,LOC101929528*	.	.	.
8	59089561	G	A	intergenic	*FAM110B,LOC101929528*	.	.	.
2	193387	C	A	intergenic	*FAM110C,SH3YL1*	.	.	.
2	108369	G	C	intergenic	*FAM110C,SH3YL1*	.	.	.
2	93882	T	C	intergenic	*FAM110C,SH3YL1*	.	.	.
5	153402501	C	G	intronic	*FAM114A2*	.	.	.
2	203609657	G	A	intronic	*FAM117B*	.	.	.
9	96248345	T	A	intronic	*FAM120A*	.	.	.
6	170645235	T	C	intronic	*FAM120B*	.	.	.
6	170645874	G	T	intronic	*FAM120B*	.	.	.
6	170646156	G	A	intronic	*FAM120B*	.	.	.
6	170663125	A	G	intronic	*FAM120B*	.	.	.
X	54132176	G	A	intronic	*FAM120C*	.	.	.
X	54170943	C	A	intronic	*FAM120C*	.	.	.
X	54171639	G	A	intronic	*FAM120C*	.	.	.
X	133940468	G	A	intronic	*FAM122C*	.	.	.
X	133965199	G	C	intronic	*FAM122C*	.	.	.
7	23072460	A	G	intergenic	*FAM126A,KLHL7-AS1*	.	.	.
2	201891236	G	T	intronic	*FAM126B*	.	.	.
2	201892982	A	G	intronic	*FAM126B*	.	.	.
2	201892983	C	G	intronic	*FAM126B*	.	.	.
2	201893346	C	T	intronic	*FAM126B*	.	.	.
2	201893725	T	C	intronic	*FAM126B*	.	.	.
2	201893726	G	A	intronic	*FAM126B*	.	.	.
2	201901247	A	G	intronic	*FAM126B*	.	.	.
X	134196585	G	GTTTGGCTG	intergenic	*FAM127B,SMIM10L2B*	.	.	.
X	94363765	C	A	intergenic	*FAM133A,BRDTP1*	.	.	.
X	94444131	T	C	intergenic	*FAM133A,BRDTP1*	.	.	.
X	94508501	G	T	intergenic	*FAM133A,BRDTP1*	.	.	.
X	93932278	A	T	intergenic	*FAM133A,BRDTP1*	.	.	.
X	94347302	G	T	intergenic	*FAM133A,BRDTP1*	.	.	.
X	94107883	GTATA	G	intergenic	*FAM133A,BRDTP1*	.	.	.
X	94852547	T	G	intergenic	*FAM133A,BRDTP1*	.	.	.

X	94759917	C	A	intergenic	*FAM133A,BRDTP1*	.	.	.
X	94759919	T	A	intergenic	*FAM133A,BRDTP1*	.	.	.
X	94240424	T	A	intergenic	*FAM133A,BRDTP1*	.	.	.
X	93536370	T	A	intergenic	*FAM133A,BRDTP1*	.	.	.
X	93555420	C	T	intergenic	*FAM133A,BRDTP1*	.	.	.
X	93778724	A	ATATGGAAT	intergenic	*FAM133A,BRDTP1*	.	.	.
X	93892716	A	G	intergenic	*FAM133A,BRDTP1*	.	.	.
X	93968259	T	A	intergenic	*FAM133A,BRDTP1*	.	.	.
X	93968260	T	C	intergenic	*FAM133A,BRDTP1*	.	.	.
X	94020103	AC	A	intergenic	*FAM133A,BRDTP1*	.	.	.
X	94020105	T	G	intergenic	*FAM133A,BRDTP1*	.	.	.
X	94020875	G	A	intergenic	*FAM133A,BRDTP1*	.	.	.
X	94034607	A	G	intergenic	*FAM133A,BRDTP1*	.	.	.
X	94034609	T	C	intergenic	*FAM133A,BRDTP1*	.	.	.
X	94046194	C	T	intergenic	*FAM133A,BRDTP1*	.	.	.
X	94046594	A	C	intergenic	*FAM133A,BRDTP1*	.	.	.
X	94046647	T	G	intergenic	*FAM133A,BRDTP1*	.	.	.
X	94046648	T	G	intergenic	*FAM133A,BRDTP1*	.	.	.
X	94047898	C	G	intergenic	*FAM133A,BRDTP1*	.	.	.
X	94097532	T	C	intergenic	*FAM133A,BRDTP1*	.	.	.
X	94120424	T	C	intergenic	*FAM133A,BRDTP1*	.	.	.
X	94151066	C	T	intergenic	*FAM133A,BRDTP1*	.	.	.
X	94151067	A	G	intergenic	*FAM133A,BRDTP1*	.	.	.
X	94152048	A	G	intergenic	*FAM133A,BRDTP1*	.	.	.
X	94200095	A	G	intergenic	*FAM133A,BRDTP1*	.	.	.
X	94243908	A	C	intergenic	*FAM133A,BRDTP1*	.	.	.
X	94455584	A	G	intergenic	*FAM133A,BRDTP1*	.	.	.
X	94456886	A	T	intergenic	*FAM133A,BRDTP1*	.	.	.
X	94482167	T	G	intergenic	*FAM133A,BRDTP1*	.	.	.
X	94516027	G	C	intergenic	*FAM133A,BRDTP1*	.	.	.
X	94517412	G	A	intergenic	*FAM133A,BRDTP1*	.	.	.
X	94609184	C	T	intergenic	*FAM133A,BRDTP1*	.	.	.
X	94609686	G	A	intergenic	*FAM133A,BRDTP1*	.	.	.
X	94625171	A	G	intergenic	*FAM133A,BRDTP1*	.	.	.
X	94687086	G	A	intergenic	*FAM133A,BRDTP1*	.	.	.
X	94813846	T	C	intergenic	*FAM133A,BRDTP1*	.	.	.
X	94868495	G	C	intergenic	*FAM133A,BRDTP1*	.	.	.
X	93058334	T	C	intergenic	*FAM133A,NONE*	.	.	.

X	93352700	C	T	intergenic	*FAM133A,NONE*	.	.	.
X	93263845	T	C	intergenic	*FAM133A,NONE*	.	.	.
X	93015957	AT	A	intergenic	*FAM133A,NONE*	.	.	.
X	92973591	G	T	intergenic	*FAM133A,NONE*	.	.	.
X	93163004	G	A	intergenic	*FAM133A,NONE*	.	.	.
X	93163006	G	T	intergenic	*FAM133A,NONE*	.	.	.
X	93194014	T	C	intergenic	*FAM133A,NONE*	.	.	.
X	93194015	G	A	intergenic	*FAM133A,NONE*	.	.	.
X	93226615	C	G	intergenic	*FAM133A,NONE*	.	.	.
X	93244950	T	C	intergenic	*FAM133A,NONE*	.	.	.
X	93338047	G	A	intergenic	*FAM133A,NONE*	.	.	.
X	93486100	A	G	intergenic	*FAM133A,NONE*	.	.	.
5	16476653	C	T	intronic	*FAM134B*	.	.	.
5	16610845	A	C	intronic	*FAM134B*	.	.	.
6	71206241	C	G	intronic	*FAM135A*	.	.	.
6	71208237	T	C	intronic	*FAM135A*	.	.	.
8	139499883	T	TGGGGGC	intronic	*FAM135B*	.	.	.
8	139152601	G	T	intronic	*FAM135B*	.	.	.
8	139296405	T	C	intronic	*FAM135B*	.	.	.
8	139447355	G	C	intronic	*FAM135B*	.	.	.
8	139505334	T	A	intronic	*FAM135B*	.	.	.
8	139167293	A	G	intronic	*FAM135B*	.	.	.
8	139167295	T	C	intronic	*FAM135B*	.	.	.
8	139167873	A	G	intronic	*FAM135B*	.	.	.
8	139167874	A	G	intronic	*FAM135B*	.	.	.
8	139251579	C	T	intronic	*FAM135B*	.	.	.
8	139251581	A	T	intronic	*FAM135B*	.	.	.
8	139456058	G	T	intronic	*FAM135B*	.	.	.
8	139456059	A	G	intronic	*FAM135B*	.	.	.
8	139459664	G	A	intronic	*FAM135B*	.	.	.
8	139517588	C	T	intergenic	*FAM135B,COL22A1*	.	.	.
8	139531816	C	CG	intergenic	*FAM135B,COL22A1*	.	.	.
8	139517570	GACAC	G	intergenic	*FAM135B,COL22A1*	.	.	.
2	70541108	T	A	intergenic	*FAM136A,TGFA*	.	.	.
2	70634372	T	G	intergenic	*FAM136A,TGFA*	.	.	.

2	70656682	A	G	intergenic	*FAM136A,TGFA*	.	.	.
9	41871	A	T	intergenic	*FAM138C,PGM5P3-AS1*	.	.	.
4	89834333	T	A	intronic	*FAM13A*	.	.	.
4	89794905	A	C	intronic	*FAM13A*	.	.	.
4	89794906	G	A	intronic	*FAM13A*	.	.	.
4	89795351	T	A	intronic	*FAM13A*	.	.	.
4	89981162	G	A	intergenic	*FAM13A,TIGD2*	.	.	.
4	89983201	G	A	intergenic	*FAM13A,TIGD2*	.	.	.
5	137300761	C	G	intronic	*FAM13B*	.	.	.
5	137300762	A	G	intronic	*FAM13B*	.	.	.
5	137309196	C	T	intronic	*FAM13B*	.	.	.
5	137388791	A	G	intergenic	*FAM13B,WNT8A*	.	.	.
10	61059666	A	G	intronic	*FAM13C*	.	.	.
10	61307454	CA	C	intergenic	*FAM13C,SLC16A9*	.	.	.
10	61170869	C	T	intergenic	*FAM13C,SLC16A9*	.	.	.
10	61172080	T	C	intergenic	*FAM13C,SLC16A9*	.	.	.
10	61181839	T	A	intergenic	*FAM13C,SLC16A9*	.	.	.
10	61183312	T	A	intergenic	*FAM13C,SLC16A9*	.	.	.
10	61184116	A	C	intergenic	*FAM13C,SLC16A9*	.	.	.
10	61184908	A	G	intergenic	*FAM13C,SLC16A9*	.	.	.
10	61262316	G	A	intergenic	*FAM13C,SLC16A9*	.	.	.
10	61262318	G	A	intergenic	*FAM13C,SLC16A9*	.	.	.
10	61291087	A	G	intergenic	*FAM13C,SLC16A9*	.	.	.
10	61293102	A	T	intergenic	*FAM13C,SLC16A9*	.	.	.
10	61325689	C	T	intergenic	*FAM13C,SLC16A9*	.	.	.
10	61325691	G	T	intergenic	*FAM13C,SLC16A9*	.	.	.
4	187102487	T	C	intergenic	*FAM149A,FLJ38576*	.	.	.
4	187105110	G	T	intergenic	*FAM149A,FLJ38576*	.	.	.
8	53456538	C	G	intronic	*FAM150A*	.	.	.
8	53465260	C	A	intronic	*FAM150A*	.	.	.
2	623920	A	G	intergenic	*FAM150B,TMEM18*	.	.	.
2	487017	G	C	intergenic	*FAM150B,TMEM18*	.	.	.
2	545802	C	A	intergenic	*FAM150B,TMEM18*	.	.	.
2	612656	G	T	intergenic	*FAM150B,TMEM18*	.	.	.
2	361950	C	A	intergenic	*FAM150B,TMEM18*	.	.	.

2	348663	G	T	intergenic	*FAM150B,TMEM18*	.	.	.
2	371849	CT	C	intergenic	*FAM150B,TMEM18*	.	.	.
5	79825224	G	A	intronic	*FAM151B*	.	.	.
5	79825485	T	C	intronic	*FAM151B*	.	.	.
5	79827276	G	A	intronic	*FAM151B*	.	.	.
5	79829597	T	A	intronic	*FAM151B*	.	.	.
5	79845594	G	C	intergenic	*FAM151B,ANKRD34B*	.	.	.
5	175503309	G	T	intronic	*FAM153B*	.	.	.
5	177453572	T	A	ncRNA_intronic	*FAM153C*	.	.	.
5	177454106	T	C	ncRNA_intronic	*FAM153C*	.	.	.
5	177520736	C	A	intergenic	*FAM153C,N4BP3*	.	.	.
5	177520737	A	G	intergenic	*FAM153C,N4BP3*	.	.	.
13	108283093	AATAT	A	intronic	*FAM155A*	.	.	.
13	108151559	G	A	intronic	*FAM155A*	.	.	.
13	108341871	C	A	intronic	*FAM155A*	.	.	.
13	108429860	T	C	intronic	*FAM155A*	.	.	.
13	108116574	TA	T	intronic	*FAM155A*	.	.	.
13	108229994	G	T	intronic	*FAM155A*	.	.	.
13	108491937	G	A	intronic	*FAM155A*	.	.	.
13	107837741	T	C	intronic	*FAM155A*	.	.	.
13	108284658	T	C	intronic	*FAM155A*	.	.	.
13	108187409	C	T	intronic	*FAM155A*	.	.	.
13	108380657	A	T	intronic	*FAM155A*	.	.	.
13	108480900	G	A	ncRNA_intronic	*FAM155A-IT1*	.	.	.
13	108805417	G	A	intergenic	*FAM155A,LIG4*	.	.	.
13	108640692	T	C	intergenic	*FAM155A,LIG4*	.	.	.
X	53000860	A	T	intronic	*FAM156A*	.	.	.
X	53015267	G	A	intronic	*FAM156A*	.	.	.
X	52958788	C	T	intergenic	*FAM156A,FAM156A*	.	.	.
X	52958789	A	T	intergenic	*FAM156A,FAM156A*	.	.	.
3	197894468	G	T	intronic	*FAM157A*	.	.	.
16	90216257	C	T	ncRNA_intronic	*FAM157C*	.	.	.
4	152394842	C	G	intronic	*FAM160A1*	.	.	.
4	152473588	T	A	intronic	*FAM160A1*	.	.	.
2	62082227	A	G	upstream	*FAM161A*	.	.	.

3	122104525	T	C	intronic	*FAM162A*	.	.	.
6	117096030	G	A	intergenic	*FAM162B,GPRC6A*	.	.	.
6	117096032	G	A	intergenic	*FAM162B,GPRC6A*	.	.	.
6	117097588	C	T	intergenic	*FAM162B,GPRC6A*	.	.	.
1	179761061	G	T	intronic	*FAM163A*	.	.	.
8	11261673	T	C	ncRNA_intronic	*FAM167A-AS1*	.	.	.
8	11290023	G	T	ncRNA_intronic	*FAM167A-AS1*	.	.	.
11	73214514	T	G	intronic	*FAM168A*	.	.	.
2	131831158	G	A	intronic	*FAM168B*	.	.	.
5	74090273	C	T	intronic	*FAM169A*	.	.	.
5	74201211	A	T	intergenic	*FAM169A,GCNT4*	.	.	.
15	99148583	C	T	intergenic	*FAM169B,IRAIN*	.	.	.
15	99086949	T	C	intergenic	*FAM169B,IRAIN*	.	.	.
5	119527396	T	TCCACTTGT(	intergenic	*FAM170A,PRR16*	.	.	.
5	119650734	T	C	intergenic	*FAM170A,PRR16*	.	.	.
5	119678604	G	GA	intergenic	*FAM170A,PRR16*	.	.	.
5	119190258	G	C	intergenic	*FAM170A,PRR16*	.	.	.
5	119311369	A	G	intergenic	*FAM170A,PRR16*	.	.	.
5	119340618	G	C	intergenic	*FAM170A,PRR16*	.	.	.
5	119277318	A	T	intergenic	*FAM170A,PRR16*	.	.	.
5	119627814	G	A	intergenic	*FAM170A,PRR16*	.	.	.
5	119004285	A	G	intergenic	*FAM170A,PRR16*	.	.	.
5	119024207	C	T	intergenic	*FAM170A,PRR16*	.	.	.
5	119058954	A	G	intergenic	*FAM170A,PRR16*	.	.	.
5	119462900	C	A	intergenic	*FAM170A,PRR16*	.	.	.
5	119514647	C	T	intergenic	*FAM170A,PRR16*	.	.	.
5	119611076	T	A	intergenic	*FAM170A,PRR16*	.	.	.
5	119611387	T	A	intergenic	*FAM170A,PRR16*	.	.	.
5	119638464	G	A	intergenic	*FAM170A,PRR16*	.	.	.
5	119638466	C	T	intergenic	*FAM170A,PRR16*	.	.	.
5	119762042	G	C	intergenic	*FAM170A,PRR16*	.	.	.
10	15315002	A	C	intronic	*FAM171A1*	.	.	.
10	15315297	C	T	intronic	*FAM171A1*	.	.	.
10	15459966	G	A	intergenic	*FAM171A1,ITGA8*	.	.	.
2	187660497	C	T	intergenic	*FAM171B,ZSWIM2*	.	.	.

2	187679551	T	C	intergenic	*FAM171B,ZSWIM2*	.	.	.
2	187679833	C	A	intergenic	*FAM171B,ZSWIM2*	.	.	.
2	187682475	A	C	intergenic	*FAM171B,ZSWIM2*	.	.	.
5	93101625	G	C	intronic	*FAM172A*	.	.	.
5	93163688	C	T	intronic	*FAM172A*	.	.	.
5	93188075	A	G	intronic	*FAM172A*	.	.	.
5	93468707	A	G	intergenic	*FAM172A,KIAA0825*	.	.	.
5	93469617	A	G	intergenic	*FAM172A,KIAA0825*	.	.	.
5	93469618	C	T	intergenic	*FAM172A,KIAA0825*	.	.	.
3	101248228	A	G	intergenic	*FAM172BP,TRMT10C*	.	.	.
3	101257160	A	G	intergenic	*FAM172BP,TRMT10C*	.	.	.
3	101258007	G	C	intergenic	*FAM172BP,TRMT10C*	.	.	.
3	101258009	A	G	intergenic	*FAM172BP,TRMT10C*	.	.	.
5	99892663	A	G	intronic	*FAM174A*	.	.	.
5	99894162	C	T	intronic	*FAM174A*	.	.	.
5	99909622	A	G	intronic	*FAM174A*	.	.	.
5	99911344	G	T	intronic	*FAM174A*	.	.	.
5	100049066	A	G	intergenic	*FAM174A,ST8SIA4*	.	.	.
5	99976083	T	C	intergenic	*FAM174A,ST8SIA4*	.	.	.
5	99996583	G	T	intergenic	*FAM174A,ST8SIA4*	.	.	.
5	100039209	T	C	intergenic	*FAM174A,ST8SIA4*	.	.	.
5	100039210	G	A	intergenic	*FAM174A,ST8SIA4*	.	.	.
5	100040134	T	C	intergenic	*FAM174A,ST8SIA4*	.	.	.
5	100119805	A	G	intergenic	*FAM174A,ST8SIA4*	.	.	.

5	100122599	A	G	intergenic	*FAM174A,ST8SIA4*	.	.	.
15	93211278	A	G	intergenic	*FAM174B,ASB9P1*	.	.	.
15	93300253	T	C	intergenic	*FAM174B,ASB9P1*	.	.	.
4	84421890	T	C	intergenic	*FAM175A,GPAT3*	.	.	.
10	126495656	C	T	intronic	*FAM175B*	.	.	.
10	126567383	G	A	intergenic	*FAM175B,ZRANB1*	.	.	.
2	97640148	T	A	intronic	*FAM178B*	.	.	.
2	97742063	A	AAAAAAC	intergenic	*FAM178B,FAHD2B*	.	.	.
14	45540521	T	C	intronic	*FAM179B*	.	.	.
14	45457423	A	G	intronic	*FAM179B*	.	.	.
14	45540113	CTGTTGT	C	intronic	*FAM179B*	.	.	.
20	26063195	A	AATAAG	ncRNA_intronic	*FAM182A*	.	.	.
20	26077670	A	G	intergenic	*FAM182A,NCOR1P1*	.	.	.
20	25753636	C	T	ncRNA_exonic	*FAM182B*	.	.	.
6	119299001	G	T	intronic	*FAM184A*	.	.	.
4	17646072	A	G	intronic	*FAM184B*	.	.	.
4	17680683	C	T	intronic	*FAM184B*	.	.	.
4	17680684	A	G	intronic	*FAM184B*	.	.	.
4	17705479	G	A	intronic	*FAM184B*	.	.	.
4	17731864	T	G	intronic	*FAM184B*	.	.	.
4	17733027	T	C	intronic	*FAM184B*	.	.	.
4	17733028	A	C	intronic	*FAM184B*	.	.	.
4	17736514	C	T	intronic	*FAM184B*	.	.	.
4	17736633	A	G	intronic	*FAM184B*	.	.	.
4	17786682	C	T	intergenic	*FAM184B,DCAF16*	.	.	.
4	17794022	A	T	intergenic	*FAM184B,DCAF16*	.	.	.
4	17799927	A	T	intergenic	*FAM184B,DCAF16*	.	.	.
7	102422573	A	G	intronic	*FAM185A*	.	.	.
7	102433807	C	T	intronic	*FAM185A*	.	.	.
12	50016007	CATT	C	intergenic	*FAM186B,PRPF40B*	.	.	.
10	16036231	C	T	intergenic	*FAM188A,PTER*	.	.	.
10	15916889	G	C	intergenic	*FAM188A,PTER*	.	.	.
10	15917890	G	A	intergenic	*FAM188A,PTER*	.	.	.
10	16096041	A	G	intergenic	*FAM188A,PTER*	.	.	.
10	16126156	A	G	intergenic	*FAM188A,PTER*	.	.	.

10	16217634	T	G	intergenic	*FAM188A,PTER*	.	.	.
10	16260488	T	C	intergenic	*FAM188A,PTER*	.	.	.
10	16260489	G	A	intergenic	*FAM188A,PTER*	.	.	.
10	16385234	C	T	intergenic	*FAM188A,PTER*	.	.	.
10	16457962	G	A	intergenic	*FAM188A,PTER*	.	.	.
15	29505266	T	G	intronic	*FAM189A1*	.	.	.
9	71940646	A	G	intronic	*FAM189A2*	.	.	.
9	72024465	C	T	intergenic	*FAM189A2,APBA1*	.	.	.
9	72029755	G	T	intergenic	*FAM189A2,APBA1*	.	.	.
4	2629112	A	AAACAGGTG	intronic	*FAM193A*	.	.	.
4	2713447	G	A	intronic	*FAM193A*	.	.	.
3	43082926	A	G	intronic	*FAM198A*	.	.	.
X	103481076	C	T	intergenic	*FAM199X,ESX1*	.	.	.
3	68531571	T	C	intronic	*FAM19A1*	.	.	.
3	68217932	CA	C	intronic	*FAM19A1*	.	.	.
3	68270523	C	A	intronic	*FAM19A1*	.	.	.
3	68352302	T	G	intronic	*FAM19A1*	.	.	.
3	68226940	C	A	intronic	*FAM19A1*	.	.	.
3	68079800	T	A	intronic	*FAM19A1*	.	.	.
3	68079802	C	T	intronic	*FAM19A1*	.	.	.
3	68182631	T	G	intronic	*FAM19A1*	.	.	.
3	68182773	T	G	intronic	*FAM19A1*	.	.	.
3	68525155	A	G	intronic	*FAM19A1*	.	.	.
3	68689254	T	C	intergenic	*FAM19A1,FAM19A4*	.	.	.
3	68630956	C	T	intergenic	*FAM19A1,FAM19A4*	.	.	.
3	68630957	A	G	intergenic	*FAM19A1,FAM19A4*	.	.	.
12	62492472	A	T	intronic	*FAM19A2*	.	.	.
12	62128201	C	T	intronic	*FAM19A2*	.	.	.
12	62373974	C	A	intronic	*FAM19A2*	.	.	.
12	62502487	A	G	intronic	*FAM19A2*	.	.	.
12	62503285	T	C	intronic	*FAM19A2*	.	.	.
12	62503324	A	G	intronic	*FAM19A2*	.	.	.
12	62503325	A	G	intronic	*FAM19A2*	.	.	.
12	62594241	T	G	intergenic	*FAM19A2,USP15*	.	.	.
12	62594242	T	C	intergenic	*FAM19A2,USP15*	.	.	.

1	113294480	C	T	intergenic	*FAM19A3,LINC01356*	.	.	.
3	68861657	G	A	intronic	*FAM19A4*	.	.	.
22	48928030	A	G	intronic	*FAM19A5*	.	.	.
22	48953305	C	T	intronic	*FAM19A5*	.	.	.
4	15702188	G	A	intergenic	*FAM200B,BST1*	.	.	.
9	38999437	T	TCACAGAA	intergenic	*FAM201A,CNTNAP3*	.	.	.
9	38730000	G	T	intergenic	*FAM201A,CNTNAP3*	.	.	.
9	38770020	C	CAGAT	intergenic	*FAM201A,CNTNAP3*	.	.	.
9	38794105	T	C	intergenic	*FAM201A,CNTNAP3*	.	.	.
9	38794106	G	C	intergenic	*FAM201A,CNTNAP3*	.	.	.
10	120104043	C	T	intergenic	*FAM204A,LINC00867*	.	.	.
9	34846839	C	T	intergenic	*FAM205BP,FAM205C*	.	.	.
9	34881171	C	A	intergenic	*FAM205BP,FAM205C*	.	.	.
21	46407612	A	G	intergenic	*FAM207A,LINC00163*	.	.	.
17	66549058	A	G	intronic	*FAM20A*	.	.	.
17	66606247	GGTGTGT	G	intergenic	*FAM20A,LINC01482*	.	.	.
1	179039242	G	A	intronic	*FAM20B*	.	.	.
18	13692841	G	A	intronic	*FAM210A*	.	.	.
18	13694604	T	A	intronic	*FAM210A*	.	.	.
15	52996800	T	C	intergenic	*FAM214A,ONECUT1*	.	.	.
10	46257327	A	T	intronic	*FAM21C*	.	.	.
10	51801774	C	T	ncRNA_intronic	*FAM21EP*	.	.	.
12	110198602	G	A	ncRNA_intronic	*FAM222A-AS1*	.	.	.
15	49786069	GTATA	G	intronic	*FAM227B*	.	.	.
15	49795541	T	C	intronic	*FAM227B*	.	.	.
10	124678662	C	G	intergenic	*FAM24A,C10orf88*	.	.	.

10	124679399	G	T	intergenic	*FAM24A,C10orf88*	.	.	.
6	116800377	T	C	intergenic	*FAM26F,TRAPPC3L*	.	.	.
6	116808973	T	C	intergenic	*FAM26F,TRAPPC3L*	.	.	.
6	116809576	T	C	intergenic	*FAM26F,TRAPPC3L*	.	.	.
6	116809712	T	C	intergenic	*FAM26F,TRAPPC3L*	.	.	.
9	67920430	C	T	intergenic	*FAM27B,ANKRD20A1*	.	.	.
17	21873588	A	ATGCC	intergenic	*FAM27L,FLJ36000*	.	.	.
17	21892108	T	C	intergenic	*FAM27L,FLJ36000*	.	.	.
17	21835302	A	T	intergenic	*FAM27L,FLJ36000*	.	.	.
17	21884078	A	C	intergenic	*FAM27L,FLJ36000*	.	.	.
10	47550392	C	T	intergenic	*FAM35DP,ANTXRLP1*	.	.	.
10	47550393	T	C	intergenic	*FAM35DP,ANTXRLP1*	.	.	.
7	121079029	G	C	intergenic	*FAM3C,PTPRZ1*	.	.	.
7	121082475	G	A	intergenic	*FAM3C,PTPRZ1*	.	.	.
7	121350742	A	C	intergenic	*FAM3C,PTPRZ1*	.	.	.
7	121362408	C	T	intergenic	*FAM3C,PTPRZ1*	.	.	.
7	121467521	A	ATACCTGAG	intergenic	*FAM3C,PTPRZ1*	.	.	.
3	58620475	A	G	intronic	*FAM3D*	.	.	.
3	58662360	G	A	intergenic	*FAM3D,C3orf67*	.	.	.
1	826117	T	C	intergenic	*FAM41C,LOC100130417*	.	.	.
1	825987	C	T	intergenic	*FAM41C,LOC100130417*	.	.	.
X	129734848	T	G	intergenic	*FAM45BP,ENOX2*	.	.	.
X	129734849	G	C	intergenic	*FAM45BP,ENOX2*	.	.	.
1	118260364	C	A	intergenic	*FAM46C,GDAP2*	.	.	.
1	118308909	A	G	intergenic	*FAM46C,GDAP2*	.	.	.
1	118309403	T	C	intergenic	*FAM46C,GDAP2*	.	.	.
1	118309404	G	T	intergenic	*FAM46C,GDAP2*	.	.	.
X	79631050	T	G	intronic	*FAM46D*	.	.	.
X	79612619	T	C	intronic	*FAM46D*	.	.	.

X	79612620	G	A	intronic	*FAM46D*	.	.	.
X	79613258	G	A	intronic	*FAM46D*	.	.	.
X	79613876	A	C	intronic	*FAM46D*	.	.	.
X	79637728	A	G	intronic	*FAM46D*	.	.	.
X	79640325	A	T	intronic	*FAM46D*	.	.	.
X	79640699	T	C	intronic	*FAM46D*	.	.	.
X	79665061	C	G	intronic	*FAM46D*	.	.	.
X	79797482	G	T	intergenic	*FAM46D,BRWD3*	.	.	.
X	79715725	G	T	intergenic	*FAM46D,BRWD3*	.	.	.
X	79910947	A	AG	intergenic	*FAM46D,BRWD3*	.	.	.
X	79870489	G	T	intergenic	*FAM46D,BRWD3*	.	.	.
X	79728351	C	T	intergenic	*FAM46D,BRWD3*	.	.	.
X	79728619	T	G	intergenic	*FAM46D,BRWD3*	.	.	.
X	79757632	C	T	intergenic	*FAM46D,BRWD3*	.	.	.
X	79849227	C	G	intergenic	*FAM46D,BRWD3*	.	.	.
X	79852811	A	G	intergenic	*FAM46D,BRWD3*	.	.	.
X	79852812	G	T	intergenic	*FAM46D,BRWD3*	.	.	.
X	79853087	A	G	intergenic	*FAM46D,BRWD3*	.	.	.
X	79853366	T	C	intergenic	*FAM46D,BRWD3*	.	.	.
X	79909517	C	G	intergenic	*FAM46D,BRWD3*	.	.	.
X	79911026	G	A	intergenic	*FAM46D,BRWD3*	.	.	.
X	79911092	T	A	intergenic	*FAM46D,BRWD3*	.	.	.
X	34193567	A	T	intergenic	*FAM47A,TMEM47*	.	.	.
X	34186698	C	G	intergenic	*FAM47A,TMEM47*	.	.	.
X	34542046	GACACAC	G	intergenic	*FAM47A,TMEM47*	.	.	.
X	34177485	C	T	intergenic	*FAM47A,TMEM47*	.	.	.
X	34177815	C	T	intergenic	*FAM47A,TMEM47*	.	.	.
X	34184688	A	G	intergenic	*FAM47A,TMEM47*	.	.	.
X	34186834	C	A	intergenic	*FAM47A,TMEM47*	.	.	.
X	34331964	CTGTGTGTG	C	intergenic	*FAM47A,TMEM47*	.	.	.
X	34613283	A	G	intergenic	*FAM47A,TMEM47*	.	.	.
X	34613284	A	G	intergenic	*FAM47A,TMEM47*	.	.	.
X	35587663	A	T	intergenic	*FAM47B,MAGEB16*	.	.	.
X	35152684	T	TGCATC	intergenic	*FAM47B,MAGEB16*	.	.	.

X	35291024	G	C	intergenic	*FAM47B,MAGEB16*	.	.	.
X	35297591	G	C	intergenic	*FAM47B,MAGEB16*	.	.	.
X	34979806	AC	A	intergenic	*FAM47B,MAGEB16*	.	.	.
X	35172825	G	A	intergenic	*FAM47B,MAGEB16*	.	.	.
X	35801868	T	G	intergenic	*FAM47B,MAGEB16*	.	.	.
X	35481105	G	T	intergenic	*FAM47B,MAGEB16*	.	.	.
X	35612876	T	C	intergenic	*FAM47B,MAGEB16*	.	.	.
X	35676762	A	T	intergenic	*FAM47B,MAGEB16*	.	.	.
X	35516113	G	T	intergenic	*FAM47B,MAGEB16*	.	.	.
X	35516114	C	T	intergenic	*FAM47B,MAGEB16*	.	.	.
X	35158057	T	C	intergenic	*FAM47B,MAGEB16*	.	.	.
X	35351135	C	G	intergenic	*FAM47B,MAGEB16*	.	.	.
X	35351136	C	T	intergenic	*FAM47B,MAGEB16*	.	.	.
X	35430388	T	C	intergenic	*FAM47B,MAGEB16*	.	.	.
X	35430389	G	A	intergenic	*FAM47B,MAGEB16*	.	.	.
X	35433106	T	A	intergenic	*FAM47B,MAGEB16*	.	.	.
X	35538917	AACACACACAC	A	intergenic	*FAM47B,MAGEB16*	.	.	.
X	35812415	A	AAC	intergenic	*FAM47B,MAGEB16*	.	.	.
X	37035976	T	A	intergenic	*FAM47C,FTH1P18*	.	.	.

2	17521516	C	T	intergenic	*FAM49A,RAD51AP2*	.	.	.
2	17332333	G	T	intergenic	*FAM49A,RAD51AP2*	.	.	.
2	16991362	C	CT	intergenic	*FAM49A,RAD51AP2*	.	.	.
2	16882337	G	C	intergenic	*FAM49A,RAD51AP2*	.	.	.
2	16924656	A	T	intergenic	*FAM49A,RAD51AP2*	.	.	.
2	17179417	G	A	intergenic	*FAM49A,RAD51AP2*	.	.	.
2	17247364	G	A	intergenic	*FAM49A,RAD51AP2*	.	.	.
2	17247744	T	G	intergenic	*FAM49A,RAD51AP2*	.	.	.
2	17334222	A	G	intergenic	*FAM49A,RAD51AP2*	.	.	.
2	17544025	C	T	intergenic	*FAM49A,RAD51AP2*	.	.	.
2	17646231	A	G	intergenic	*FAM49A,RAD51AP2*	.	.	.
2	17647144	T	A	intergenic	*FAM49A,RAD51AP2*	.	.	.
2	17670139	C	CA	intergenic	*FAM49A,RAD51AP2*	.	.	.
8	130945178	A	G	intronic	*FAM49B*	.	.	.
8	130986354	A	G	intergenic	*FAM49B,MIR5194*	.	.	.
6	3987939	C	T	intergenic	*FAM50B,PRPF4B*	.	.	.
4	1645210	G	T	intronic	*FAM53A*	.	.	.
12	31523339	A	G	intergenic	*FAM60A,DENND5B*	.	.	.
12	31483616	C	T	intergenic	*FAM60A,DENND5B*	.	.	.
12	31483617	A	G	intergenic	*FAM60A,DENND5B*	.	.	.
6	24928871	T	C	intronic	*FAM65B*	.	.	.
6	24812566	C	T	intronic	*FAM65B*	.	.	.

6	24970439	ACATCTGGTTC	A	intronic	*FAM65B*	.	.	.
6	25075112	T	G	intergenic	*FAM65B,CMAHP*	.	.	.
8	12269800	A	G	intergenic	*FAM66A,FAM90A25P*	.	.	.
12	8362292	A	C	intergenic	*FAM66C,FAM90A1*	.	.	.
8	7885350	G	A	intergenic	*FAM66E,MIR548I3*	.	.	.
8	7906908	AAAAAT	A	intergenic	*FAM66E,MIR548I3*	.	.	.
14	67680597	G	C	intronic	*FAM71D*	.	.	.
14	67681124	G	A	intronic	*FAM71D*	.	.	.
1	206171416	A	T	intergenic	*FAM72C,AVPR1B*	.	.	.
1	143906352	CTATTAT	C	intronic	*FAM72C,FAM72D*	.	.	.
1	144018389	A	AGCCCAC	intergenic	*FAM72C,NBPF20*	.	.	.
9	40660347	G	C	intergenic	*FAM74A1,SPATA31A3*	.	.	.
9	40390422	A	T	intergenic	*FAM74A1,SPATA31A3*	.	.	.
9	40738045	A	G	intergenic	*FAM74A3,ZNF658*	.	.	.
1	166471913	A	T	intergenic	*FAM78B,FMO9P*	.	.	.
1	166485681	C	A	intergenic	*FAM78B,FMO9P*	.	.	.
1	166374700	T	A	intergenic	*FAM78B,FMO9P*	.	.	.
1	166381048	C	T	intergenic	*FAM78B,FMO9P*	.	.	.
1	166563158	A	T	intergenic	*FAM78B,FMO9P*	.	.	.
1	166181977	A	G	intergenic	*FAM78B,FMO9P*	.	.	.
1	166182438	C	T	intergenic	*FAM78B,FMO9P*	.	.	.
1	166183481	G	A	intergenic	*FAM78B,FMO9P*	.	.	.
1	166218716	T	A	intergenic	*FAM78B,FMO9P*	.	.	.
1	166236324	G	A	intergenic	*FAM78B,FMO9P*	.	.	.
1	166238338	A	G	intergenic	*FAM78B,FMO9P*	.	.	.
1	166247208	C	T	intergenic	*FAM78B,FMO9P*	.	.	.
1	166280912	A	G	intergenic	*FAM78B,FMO9P*	.	.	.
1	166282231	T	C	intergenic	*FAM78B,FMO9P*	.	.	.
1	166283199	C	T	intergenic	*FAM78B,FMO9P*	.	.	.
1	166284713	T	G	intergenic	*FAM78B,FMO9P*	.	.	.
1	166284715	T	G	intergenic	*FAM78B,FMO9P*	.	.	.
1	166371957	C	T	intergenic	*FAM78B,FMO9P*	.	.	.
1	166373948	C	A	intergenic	*FAM78B,FMO9P*	.	.	.
1	166374327	C	T	intergenic	*FAM78B,FMO9P*	.	.	.

1	166374488	C	T	intergenic	*FAM78B,FMO9P*	.	.	.
1	166381410	G	A	intergenic	*FAM78B,FMO9P*	.	.	.
1	166382688	G	A	intergenic	*FAM78B,FMO9P*	.	.	.
1	166383743	G	A	intergenic	*FAM78B,FMO9P*	.	.	.
1	166464376	A	G	intergenic	*FAM78B,FMO9P*	.	.	.
1	166472555	G	C	intergenic	*FAM78B,FMO9P*	.	.	.
1	166516771	G	A	intergenic	*FAM78B,FMO9P*	.	.	.
15	59759118	G	A	intronic	*FAM81A*	.	.	.
15	59812964	G	A	intronic	*FAM81A*	.	.	.
6	54762027	A	AAAAG	intronic	*FAM83B*	.	.	.
6	54730279	G	A	intronic	*FAM83B*	.	.	.
6	54796770	A	T	intronic	*FAM83B*	.	.	.
6	54843582	G	A	intergenic	*FAM83B,HCRTR2*	.	.	.
6	54915666	T	A	intergenic	*FAM83B,HCRTR2*	.	.	.
6	54923278	G	T	intergenic	*FAM83B,HCRTR2*	.	.	.
6	54956720	C	G	intergenic	*FAM83B,HCRTR2*	.	.	.
6	54989240	G	T	intergenic	*FAM83B,HCRTR2*	.	.	.
6	54992733	A	C	intergenic	*FAM83B,HCRTR2*	.	.	.
6	54992735	C	T	intergenic	*FAM83B,HCRTR2*	.	.	.
8	144857511	CACACACACACACAG	C	intergenic	*FAM83H-AS1,SCRIB*	.	.	.
8	127625931	A	G	intergenic	*FAM84B,PCAT1*	.	.	.
8	127664831	G	A	intergenic	*FAM84B,PCAT1*	.	.	.
8	128014626	A	G	intergenic	*FAM84B,PCAT1*	.	.	.
11	67569210	C	G	ncRNA_intronic	*FAM86C2P*	.	.	.
11	67689963	G	A	intergenic	*FAM86C2P,UNC93B1*	.	.	.
11	67666391	A	C	intergenic	*FAM86C2P,UNC93B1*	.	.	.
3	75533668	C	G	intergenic	*FAM86DP,MIR1324*	.	.	.
3	75535439	T	C	intergenic	*FAM86DP,MIR1324*	.	.	.
4	4083449	G	A	intergenic	*FAM86EP,OTOP1*	.	.	.
4	4108285	G	T	intergenic	*FAM86EP,OTOP1*	.	.	.
4	4019509	C	CCTT	intergenic	*FAM86EP,OTOP1*	.	.	.
4	4003627	C	T	intergenic	*FAM86EP,OTOP1*	.	.	.
4	4017995	G	A	intergenic	*FAM86EP,OTOP1*	.	.	.

4	4044540	A	G	intergenic	*FAM86EP,OTOP1*	.	.	.
4	4054084	A	C	intergenic	*FAM86EP,OTOP1*	.	.	.
12	8490517	C	T	intergenic	*FAM86FP,LINC00937*	.	.	.
12	8456520	A	T	intergenic	*FAM86FP,LINC00937*	.	.	.
12	8485862	G	A	intergenic	*FAM86FP,LINC00937*	.	.	.
3	129878880	C	T	intergenic	*FAM86HP,COL6A4P2*	.	.	.
8	325210	A	G	downstream	*FAM87A*	.	.	.
X	8883827	C	T	intergenic	*FAM9A,FAM9B*	.	.	.
X	8903868	C	G	intergenic	*FAM9A,FAM9B*	.	.	.
X	9125396	C	T	intergenic	*FAM9B,TBL1X*	.	.	.
X	13150506	C	A	intergenic	*FAM9C,GS1-600G8.3*	.	.	.
X	13204263	G	T	intergenic	*FAM9C,GS1-600G8.3*	.	.	.
15	31211984	C	G	intronic	*FAN1*	.	.	.
16	89879420	G	A	intronic	*FANCA*	.	.	.
9	98016909	G	T	intronic	*FANCC*	.	.	.
9	97872597	C	T	UTR3	*FANCC*	.	.	.
9	97982245	G	C	intronic	*FANCC*	.	.	.
9	98022091	C	T	intronic	*FANCC*	.	.	.
9	98195607	T	C	intergenic	*FANCC,PTCH1*	.	.	.
11	22652004	C	A	intergenic	*FANCF,GAS2*	.	.	.
2	58402814	C	T	intronic	*FANCL*	.	.	.
2	58402816	G	C	intronic	*FANCL*	.	.	.
2	58497771	G	A	intergenic	*FANCL,LINC01122*	.	.	.
2	58739158	G	T	intergenic	*FANCL,LINC01122*	.	.	.
2	58554388	C	A	intergenic	*FANCL,LINC01122*	.	.	.
2	58558315	A	G	intergenic	*FANCL,LINC01122*	.	.	.
10	127658872	A	C	intronic	*FANK1*	.	.	.
10	127587683	G	A	intronic	*FANK1*	.	.	.
10	127613283	T	A	intronic	*FANK1*	.	.	.
10	127637975	A	G	intronic	*FANK1*	.	.	.
10	127638012	A	G	intronic	*FANK1*	.	.	.
11	13908514	GCTCT	G	intergenic	*FAR1,SPON1*	.	.	.

11	13844417	A	T	intergenic	*FAR1,SPON1*	.	.	.
11	13888261	G	T	intergenic	*FAR1,SPON1*	.	.	.
11	13956118	C	T	intergenic	*FAR1,SPON1*	.	.	.
11	13956273	A	G	intergenic	*FAR1,SPON1*	.	.	.
12	29398735	C	T	intronic	*FAR2*	.	.	.
12	29477522	G	A	intronic	*FAR2*	.	.	.
12	29477524	C	T	intronic	*FAR2*	.	.	.
12	29478663	T	C	intronic	*FAR2*	.	.	.
12	29478731	A	G	intronic	*FAR2*	.	.	.
12	29490030	C	CAT	intergenic	*FAR2,ERGIC2*	.	.	.
2	130818669	C	A	intergenic	*FAR2P1,POTEF*	.	.	.
13	98988673	G	A	intronic	*FARP1*	.	.	.
2	242323275	G	A	intronic	*FARP2*	.	.	.
6	5721411	A	T	intronic	*FARS2*	.	.	.
6	5472436	G	A	intronic	*FARS2*	.	.	.
6	5756305	G	A	intronic	*FARS2*	.	.	.
6	5773724	C	A	intergenic	*FARS2,NRN1*	.	.	.
6	5899390	C	G	intergenic	*FARS2,NRN1*	.	.	.
6	5858864	C	T	intergenic	*FARS2,NRN1*	.	.	.
6	5859155	A	T	intergenic	*FARS2,NRN1*	.	.	.
6	5914348	C	CAT	intergenic	*FARS2,NRN1*	.	.	.
2	223452245	C	A	intronic	*FARSB*	.	.	.
1	172672633	T	C	intergenic	*FASLG,TNFSF18*	.	.	.
1	172798674	G	A	intergenic	*FASLG,TNFSF18*	.	.	.
1	172878882	GAGACTAT CCCACGTA CACAGGAT AGGCTCCA	G	intergenic	*FASLG,TNFSF18*	.	.	.
1	172846838	C	T	intergenic	*FASLG,TNFSF18*	.	.	.
1	172848487	T	C	intergenic	*FASLG,TNFSF18*	.	.	.
1	173003630	A	G	intergenic	*FASLG,TNFSF18*	.	.	.
1	173003648	T	G	intergenic	*FASLG,TNFSF18*	.	.	.
2	207759830	T	G	intergenic	*FASTKD2,CPO*	.	.	.
2	207762129	C	T	intergenic	*FASTKD2,CPO*	.	.	.
2	207762678	G	C	intergenic	*FASTKD2,CPO*	.	.	.
2	207763594	A	G	intergenic	*FASTKD2,CPO*	.	.	.
2	207794539	A	AC	intergenic	*FASTKD2,CPO*	.	.	.
2	207794742	T	A	intergenic	*FASTKD2,CPO*	.	.	.

4	187810399	C	CACTCCTCC	intergenic	*FAT1,LOC339975*	.	.	.
4	187923836	A	G	intergenic	*FAT1,LOC339975*	.	.	.
4	187953672	C	A	intergenic	*FAT1,LOC339975*	.	.	.
5	151027669	T	G	intergenic	*FAT2,SPARC*	.	.	.
11	92392260	A	G	intronic	*FAT3*	.	.	.
11	92092691	T	A	intronic	*FAT3*	.	.	.
11	92160431	T	A	intronic	*FAT3*	.	.	.
11	92164820	G	T	intronic	*FAT3*	.	.	.
11	92164861	A	G	intronic	*FAT3*	.	.	.
11	92198645	C	CT	intronic	*FAT3*	.	.	.
11	92250972	T	C	intronic	*FAT3*	.	.	.
11	92250973	G	A	intronic	*FAT3*	.	.	.
11	92251455	T	A	intronic	*FAT3*	.	.	.
11	92297698	T	C	intronic	*FAT3*	.	.	.
11	92297699	T	A	intronic	*FAT3*	.	.	.
11	92300802	T	C	intronic	*FAT3*	.	.	.
1	16101670	A	G	UTR3	*FBLIM1*	.	.	.
5	167970916	G	A	intergenic	*FBLL1,PANK3*	.	.	.
5	127604024	C	T	intronic	*FBN2*	.	.	.
5	127606356	G	A	intronic	*FBN2*	.	.	.
5	127631684	C	T	intronic	*FBN2*	.	.	.
5	127658032	C	T	intronic	*FBN2*	.	.	.
5	127658899	A	G	intronic	*FBN2*	.	.	.
5	127658900	T	G	intronic	*FBN2*	.	.	.
5	127824767	G	A	intronic	*FBN2*	.	.	.
5	127999034	A	AT	intergenic	*FBN2,SLC27A6*	.	.	.
5	128238300	T	A	intergenic	*FBN2,SLC27A6*	.	.	.
5	128108422	T	C	intergenic	*FBN2,SLC27A6*	.	.	.
5	127987820	C	T	intergenic	*FBN2,SLC27A6*	.	.	.
5	127988248	T	C	intergenic	*FBN2,SLC27A6*	.	.	.
5	127988393	A	G	intergenic	*FBN2,SLC27A6*	.	.	.
5	128276915	G	T	intergenic	*FBN2,SLC27A6*	.	.	.
5	128280655	C	T	intergenic	*FBN2,SLC27A6*	.	.	.
5	128286960	G	A	intergenic	*FBN2,SLC27A6*	.	.	.
5	128294230	AT	A	intergenic	*FBN2,SLC27A6*	.	.	.
7	102595723	T	C	intronic	*FBXL13*	.	.	.
7	102600699	C	T	intronic	*FBXL13*	.	.	.
7	102604220	T	C	intronic	*FBXL13*	.	.	.

7	102658415	C	A	intronic	*FBXL13*	.	.	.
7	102659155	G	A	intronic	*FBXL13*	.	.	.
7	102661589	C	T	intronic	*FBXL13*	.	.	.
7	102680596	G	A	intronic	*FBXL13*	.	.	.
7	102680708	C	A	intronic	*FBXL13*	.	.	.
7	102682072	C	T	intronic	*FBXL13*	.	.	.
5	107214420	C	T	intronic	*FBXL17*	.	.	.
5	107717940	G	A	upstream	*FBXL17*	.	.	.
5	107206770	A	AAAAAAAAA	intronic	*FBXL17*	.	.	.
5	107660441	ATGTG	A	intronic	*FBXL17*	.	.	.
5	107368341	T	C	intronic	*FBXL17*	.	.	.
5	107383624	C	T	intronic	*FBXL17*	.	.	.
5	107547516	C	T	intronic	*FBXL17*	.	.	.
5	107778100	C	T	intergenic	*FBXL17,LINC01023*	.	.	.
5	107793507	C	G	intergenic	*FBXL17,LINC01023*	.	.	.
5	107918567	G	C	intergenic	*FBXL17,LINC01023*	.	.	.
5	107739114	A	G	intergenic	*FBXL17,LINC01023*	.	.	.
5	107760897	A	C	intergenic	*FBXL17,LINC01023*	.	.	.
5	107763247	C	T	intergenic	*FBXL17,LINC01023*	.	.	.
5	107834754	C	T	intergenic	*FBXL17,LINC01023*	.	.	.
7	5533019	C	T	intronic	*FBXL18*	.	.	.
3	33326604	G	A	intronic	*FBXL2*	.	.	.
17	37445236	G	A	intronic	*FBXL20*	.	.	.
6	99354769	A	G	intronic	*FBXL4*	.	.	.
6	99361982	G	A	intronic	*FBXL4*	.	.	.
6	99704621	A	C	intergenic	*FBXL4,FAXC*	.	.	.
6	99505782	G	A	intergenic	*FBXL4,FAXC*	.	.	.
6	99638210	TCACA	T	intergenic	*FBXL4,FAXC*	.	.	.
6	99491709	T	C	intergenic	*FBXL4,FAXC*	.	.	.
6	99647366	T	C	intergenic	*FBXL4,FAXC*	.	.	.
6	99647368	T	A	intergenic	*FBXL4,FAXC*	.	.	.

4	15649059	T	G	intronic	*FBXL5*	.	.	.
4	15619346	C	T	intronic	*FBXL5*	.	.	.
5	15833901	C	T	intronic	*FBXL7*	.	.	.
5	15578500	G	A	intronic	*FBXL7*	.	.	.
5	15782185	G	C	intronic	*FBXL7*	.	.	.
5	15867015	T	C	intronic	*FBXL7*	.	.	.
5	15760156	AT	A	intronic	*FBXL7*	.	.	.
5	15874713	C	G	intronic	*FBXL7*	.	.	.
5	16065631	TGAGA	T	intergenic	*FBXL7,MARCH11*	.	.	.
5	16009519	A	G	intergenic	*FBXL7,MARCH11*	.	.	.
2	48505861	A	G	intergenic	*FBXO11,FOXN2*	.	.	.
2	48404083	T	G	intergenic	*FBXO11,FOXN2*	.	.	.
2	48315418	A	T	intergenic	*FBXO11,FOXN2*	.	.	.
2	48139701	A	C	intergenic	*FBXO11,FOXN2*	.	.	.
2	48140562	A	T	intergenic	*FBXO11,FOXN2*	.	.	.
2	48284061	G	C	intergenic	*FBXO11,FOXN2*	.	.	.
2	48478457	C	T	intergenic	*FBXO11,FOXN2*	.	.	.
18	71813038	T	C	intronic	*FBXO15*	.	.	.
18	71767182	C	G	intronic	*FBXO15*	.	.	.
10	5988695	C	T	intergenic	*FBXO18,IL15RA*	.	.	.
8	398519	T	G	intronic	*FBXO25*	.	.	.
8	391389	C	T	intronic	*FBXO25*	.	.	.
8	391390	A	G	intronic	*FBXO25*	.	.	.
8	391634	C	T	intronic	*FBXO25*	.	.	.
1	224308403	G	A	intronic	*FBXO28*	.	.	.
1	224354452	T	C	intergenic	*FBXO28,DEGS1*	.	.	.
11	33818470	G	A	intergenic	*FBXO3-AS1,LMO2*	.	.	.
8	124595173	A	G	intergenic	*FBXO32,KLHL38*	.	.	.
8	124594778	T	A	intergenic	*FBXO32,KLHL38*	.	.	.
8	124619790	C	CA	intergenic	*FBXO32,KLHL38*	.	.	.
14	40579692	C	A	intergenic	*FBXO33,LOC644919*	.	.	.
14	40983583	C	A	intergenic	*FBXO33,LOC644919*	.	.	.
14	40688853	G	A	intergenic	*FBXO33,LOC644919*	.	.	.
14	40917995	A	G	intergenic	*FBXO33,LOC644919*	.	.	.

14	41105361	GA	G	intergenic	*FBXO33,LOC644919*	.	.	.
14	41105363	GAGA	G	intergenic	*FBXO33,LOC644919*	.	.	.
14	41421588	T	C	intergenic	*FBXO33,LOC644919*	.	.	.
14	40584268	G	T	intergenic	*FBXO33,LOC644919*	.	.	.
14	40914267	A	T	intergenic	*FBXO33,LOC644919*	.	.	.
14	40928329	AT	A	intergenic	*FBXO33,LOC644919*	.	.	.
14	41103027	A	C	intergenic	*FBXO33,LOC644919*	.	.	.
14	41103028	G	T	intergenic	*FBXO33,LOC644919*	.	.	.
14	41380702	T	A	intergenic	*FBXO33,LOC644919*	.	.	.
14	41246571	T	C	intergenic	*FBXO33,LOC644919*	.	.	.
14	40888881	C	T	intergenic	*FBXO33,LOC644919*	.	.	.
14	39947637	A	G	intergenic	*FBXO33,LOC644919*	.	.	.
14	39952272	T	C	intergenic	*FBXO33,LOC644919*	.	.	.
14	39991938	C	T	intergenic	*FBXO33,LOC644919*	.	.	.
14	40065778	T	C	intergenic	*FBXO33,LOC644919*	.	.	.
14	40065973	C	T	intergenic	*FBXO33,LOC644919*	.	.	.
14	40106504	C	T	intergenic	*FBXO33,LOC644919*	.	.	.
14	40127669	A	G	intergenic	*FBXO33,LOC644919*	.	.	.
14	40127858	C	T	intergenic	*FBXO33,LOC644919*	.	.	.

14	40130521	G	A	intergenic	*FBXO33,LOC644919*	.	.	.
14	40370469	TTCTC	T	intergenic	*FBXO33,LOC644919*	.	.	.
14	40398059	G	T	intergenic	*FBXO33,LOC644919*	.	.	.
14	40398230	C	T	intergenic	*FBXO33,LOC644919*	.	.	.
14	40399459	C	T	intergenic	*FBXO33,LOC644919*	.	.	.
14	40462164	A	G	intergenic	*FBXO33,LOC644919*	.	.	.
14	40462165	T	G	intergenic	*FBXO33,LOC644919*	.	.	.
14	40572908	C	T	intergenic	*FBXO33,LOC644919*	.	.	.
14	40594596	G	GTTTGTTTA	intergenic	*FBXO33,LOC644919*	.	.	.
14	40790836	A	G	intergenic	*FBXO33,LOC644919*	.	.	.
14	40791006	A	T	intergenic	*FBXO33,LOC644919*	.	.	.
14	40801102	C	T	intergenic	*FBXO33,LOC644919*	.	.	.
14	40822122	C	T	intergenic	*FBXO33,LOC644919*	.	.	.
14	41013003	A	G	intergenic	*FBXO33,LOC644919*	.	.	.
14	41013937	G	A	intergenic	*FBXO33,LOC644919*	.	.	.
14	41078228	G	A	intergenic	*FBXO33,LOC644919*	.	.	.
14	41222767	A	G	intergenic	*FBXO33,LOC644919*	.	.	.
14	41269669	T	C	intergenic	*FBXO33,LOC644919*	.	.	.
14	41288030	A	G	intergenic	*FBXO33,LOC644919*	.	.	.

2	230831450	G	A	intronic	*FBXO36*	.	.	.
5	42083223	A	G	intergenic	*FBXO4,LOC101926960*	.	.	.
5	42099847	G	T	intergenic	*FBXO4,LOC101926960*	.	.	.
5	42074821	A	G	intergenic	*FBXO4,LOC101926960*	.	.	.
5	42083163	A	T	intergenic	*FBXO4,LOC101926960*	.	.	.
5	42151614	G	C	intergenic	*FBXO4,LOC101926960*	.	.	.
5	42151635	C	A	intergenic	*FBXO4,LOC101926960*	.	.	.
1	16629735	C	T	intronic	*FBXO42*	.	.	.
5	171345178	A	G	intronic	*FBXW11*	.	.	.
5	171310830	T	C	intronic	*FBXW11*	.	.	.
5	171456812	T	A	intergenic	*FBXW11,STK10*	.	.	.
3	48431808	T	G	intronic	*FBXW12*	.	.	.
10	103392769	G	A	intronic	*FBXW4*	.	.	.
12	117361569	G	A	intronic	*FBXW8*	.	.	.
1	207172984	T	C	intergenic	*FCAMR,C1orf116*	.	.	.
14	75194872	T	C	intronic	*FCF1*	.	.	.
19	40438169	G	A	intronic	*FCGBP*	.	.	.
19	40443295	GGTGT	G	intergenic	*FCGBP,PSMC4*	.	.	.
19	40466422	A	C	intergenic	*FCGBP,PSMC4*	.	.	.
1	161549117	GAACAAAGAAAGAAAGAAAGAAAGA	G	intergenic	*FCGR3A,FCGR2C*	.	.	.
5	72267492	A	G	intronic	*FCHO2*	.	.	.
5	72272676	T	C	intronic	*FCHO2*	.	.	.
5	72273100	T	C	intronic	*FCHO2*	.	.	.
5	72290152	C	T	intronic	*FCHO2*	.	.	.
11	72618042	C	T	intronic	*FCHSD2*	.	.	.
11	72618043	A	G	intronic	*FCHSD2*	.	.	.
11	72618223	A	G	intronic	*FCHSD2*	.	.	.
11	72640298	A	G	intronic	*FCHSD2*	.	.	.
11	72689024	T	C	intronic	*FCHSD2*	.	.	.

11	72702163	C	T	intronic	*FCHSD2*	.	.	.
11	72702164	T	G	intronic	*FCHSD2*	.	.	.
11	72776381	G	A	intronic	*FCHSD2*	.	.	.
11	72777220	A	G	intronic	*FCHSD2*	.	.	.
11	72777221	G	C	intronic	*FCHSD2*	.	.	.
11	72777564	T	C	intronic	*FCHSD2*	.	.	.
9	137844567	C	A	intergenic	*FCN1,OLFM1*	.	.	.
1	157652745	C	A	intronic	*FCRL3*	.	.	.
1	157651430	T	C	intronic	*FCRL3*	.	.	.
1	157651431	G	A	intronic	*FCRL3*	.	.	.
1	157651859	T	C	intronic	*FCRL3*	.	.	.
1	157675982	C	A	intergenic	*FCRL3,FCRL2*	.	.	.
1	157699021	A	C	intergenic	*FCRL3,FCRL2*	.	.	.
1	157705427	G	A	intergenic	*FCRL3,FCRL2*	.	.	.
1	157568894	TATATATATATATATATATATATATATATATAA	T	intergenic	*FCRL4,FCRL3*	.	.	.
1	157487809	A	G	intronic	*FCRL5*	.	.	.
1	157518245	A	G	intronic	*FCRL5*	.	.	.
1	157518688	G	C	intronic	*FCRL5*	.	.	.
1	157518689	C	T	intronic	*FCRL5*	.	.	.
1	157529455	T	C	intergenic	*FCRL5,FCRL4*	.	.	.
8	11673940	A	G	intronic	*FDFT1*	.	.	.
8	11674275	A	T	intronic	*FDFT1*	.	.	.
11	110398324	A	G	intergenic	*FDX1,ARHGAP20*	.	.	.
15	68578812	TCACACA	T	intronic	*FEM1B*	.	.	.
5	114862016	A	G	intronic	*FEM1C*	.	.	.
5	108485435	A	G	intronic	*FER*	.	.	.
5	108304648	C	G	intronic	*FER*	.	.	.
5	108269211	G	T	intronic	*FER*	.	.	.
5	108310710	T	C	intronic	*FER*	.	.	.
5	108362472	C	T	intronic	*FER*	.	.	.
5	108438869	C	T	intronic	*FER*	.	.	.
5	108497390	A	C	intronic	*FER*	.	.	.
5	108497391	G	T	intronic	*FER*	.	.	.
5	108607427	C	T	intergenic	*FER,PJA2*	.	.	.

5	108607824	C	T	intergenic	*FER,PJA2*	.	.	.
5	108608449	T	C	intergenic	*FER,PJA2*	.	.	.
8	124872265	T	A	intronic	*FER1L6*	.	.	.
8	124872266	T	A	intronic	*FER1L6*	.	.	.
8	125127136	GTATATATA TA	G	ncRNA_intronic	*FER1L6-AS2*	.	.	.
8	125128740	G	A	ncRNA_intronic	*FER1L6-AS2*	.	.	.
8	125109327	TATATATAT AC	T	ncRNA_intronic	*FER1L6-AS2*	.	.	.
7	19705581	G	T	intergenic	*FERD3L,TWISTNB*	.	.	.
7	19258302	G	A	intergenic	*FERD3L,TWISTNB*	.	.	.
7	19270260	T	C	intergenic	*FERD3L,TWISTNB*	.	.	.
7	19397876	G	A	intergenic	*FERD3L,TWISTNB*	.	.	.
7	19398510	C	A	intergenic	*FERD3L,TWISTNB*	.	.	.
7	19499799	T	TAGATCGGA	intergenic	*FERD3L,TWISTNB*	.	.	.
7	19533281	T	A	intergenic	*FERD3L,TWISTNB*	.	.	.
7	19533560	T	C	intergenic	*FERD3L,TWISTNB*	.	.	.
7	19535004	ATGTGTGT ATATG	A	intergenic	*FERD3L,TWISTNB*	.	.	.
7	19582618	T	C	intergenic	*FERD3L,TWISTNB*	.	.	.
7	19582983	T	C	intergenic	*FERD3L,TWISTNB*	.	.	.
7	19597118	T	A	intergenic	*FERD3L,TWISTNB*	.	.	.
7	19725967	C	G	intergenic	*FERD3L,TWISTNB*	.	.	.
20	6295205	T	C	intergenic	*FERMT1,CASC20*	.	.	.
20	6380920	A	G	intergenic	*FERMT1,CASC20*	.	.	.
20	6125640	G	A	intergenic	*FERMT1,CASC20*	.	.	.
20	6203169	A	T	intergenic	*FERMT1,CASC20*	.	.	.
14	53462567	G	A	intergenic	*FERMT2,DDHD1*	.	.	.
2	36847856	C	A	intergenic	*FEZ2,VIT*	.	.	.
10	95350402	C	A	downstream	*FFAR4*	.	.	.
6	36985105	T	C	intronic	*FGD2*	.	.	.
6	37085928	T	C	intergenic	*FGD2,PIM1*	.	.	.
6	37085929	G	A	intergenic	*FGD2,PIM1*	.	.	.
12	32733564	A	G	intronic	*FGD4*	.	.	.
5	44309982	C	CAT	intronic	*FGF10*	.	.	.
3	191864061	A	ATATAT	intronic	*FGF12*	.	.	.
3	192177627	AGTGT	A	intronic	*FGF12*	.	.	.
3	192234940	G	A	intronic	*FGF12*	.	.	.

3	192369145	G	A	intronic	*FGF12*	.	.	.
3	192372508	C	T	intronic	*FGF12*	.	.	.
3	192462295	G	C	intergenic	*FGF12,MB21D2*	.	.	.
X	137852376	T	TTAATATCTC	intronic	*FGF13*	.	.	.
X	137716007	A	G	intronic	*FGF13*	.	.	.
X	137754631	C	T	intronic	*FGF13*	.	.	.
X	137820275	T	C	intronic	*FGF13*	.	.	.
X	137952670	G	T	intronic	*FGF13*	.	.	.
X	137990190	GTAGAGTA GGATTATT GGTGATCT TTTTCTTCT TAATCATTT TCTTTTTTT TTTTTTTTTT GAGACGGA GACTCGTT CTTTCACCC AGGTTGGA CTGCAGTG GCGCTATC TCGGCT	G	intronic	*FGF13*	.	.	.
X	137805001	A	C	intronic	*FGF13*	.	.	.
X	137817602	G	A	intronic	*FGF13*	.	.	.
X	137852376	T	TTAATATCTC	intronic	*FGF13*	.	.	.
X	138205092	G	A	intronic	*FGF13*	.	.	.
X	137741865	C	T	intronic	*FGF13*	.	.	.
X	137864449	A	T	intronic	*FGF13*	.	.	.
X	137890433	T	C	intronic	*FGF13*	.	.	.
X	137890771	G	A	intronic	*FGF13*	.	.	.
X	137892006	C	T	intronic	*FGF13*	.	.	.
X	137892777	A	G	intronic	*FGF13*	.	.	.
X	137962644	T	C	intronic	*FGF13*	.	.	.
X	137962646	A	T	intronic	*FGF13*	.	.	.
X	138081981	T	C	intronic	*FGF13*	.	.	.
X	138086127	G	A	intronic	*FGF13*	.	.	.
X	138146951	T	C	intronic	*FGF13*	.	.	.
X	138148308	T	C	intronic	*FGF13*	.	.	.

X	138242848	C	T	intronic	*FGF13*	.	.	.
X	138245015	T	C	intronic	*FGF13*	.	.	.
X	138245016	G	A	intronic	*FGF13*	.	.	.
X	138259302	T	C	intronic	*FGF13*	.	.	.
X	138259303	G	A	intronic	*FGF13*	.	.	.
X	137795428	A	T	ncRNA_intronic	*FGF13-AS1*	.	.	.
X	138380311	C	A	intergenic	*FGF13,SRD5A1P1*	.	.	.
X	138526871	A	C	intergenic	*FGF13,SRD5A1P1*	.	.	.
X	138526872	G	A	intergenic	*FGF13,SRD5A1P1*	.	.	.
X	138517685	A	G	intergenic	*FGF13,SRD5A1P1*	.	.	.
X	138343900	A	G	intergenic	*FGF13,SRD5A1P1*	.	.	.
X	138344985	G	A	intergenic	*FGF13,SRD5A1P1*	.	.	.
X	138347490	T	G	intergenic	*FGF13,SRD5A1P1*	.	.	.
X	138457653	A	G	intergenic	*FGF13,SRD5A1P1*	.	.	.
X	138520335	A	C	intergenic	*FGF13,SRD5A1P1*	.	.	.
X	138522755	G	A	intergenic	*FGF13,SRD5A1P1*	.	.	.
X	138522770	T	C	intergenic	*FGF13,SRD5A1P1*	.	.	.
13	102555588	GGTGT	G	intronic	*FGF14*	.	.	.
13	102712333	G	A	intronic	*FGF14*	.	.	.
13	102872525	C	T	intronic	*FGF14*	.	.	.
13	102600780	G	A	intronic	*FGF14*	.	.	.
13	102646214	C	T	intronic	*FGF14*	.	.	.
13	102700076	GC	G	intronic	*FGF14*	.	.	.
13	102808040	G	A	intronic	*FGF14*	.	.	.
13	102809086	C	T	intronic	*FGF14*	.	.	.
13	103009278	CATAT	C	ncRNA_intronic	*FGF14-IT1*	.	.	.
13	102974464	A	G	ncRNA_intronic	*FGF14-IT1*	.	.	.
13	103121288	T	G	intergenic	*FGF14,TPP2*	.	.	.
X	76714781	C	G	intergenic	*FGF16,ATRX*	.	.	.
X	76743238	T	C	intergenic	*FGF16,ATRX*	.	.	.
X	76754798	A	G	intergenic	*FGF16,ATRX*	.	.	.
X	76755770	T	A	intergenic	*FGF16,ATRX*	.	.	.
5	170924640	T	TCTTCTC	intergenic	*FGF18,SMIM23*	.	.	.
5	171025391	GTTTT	G	intergenic	*FGF18,SMIM23*	.	.	.
11	69626014	G	A	intronic	*FGF3*	.	.	.
11	69888954	G	A	intergenic	*FGF3,LOC101928443*	.	.	.

11	69656406	A	ACAACAGCA	intergenic	*FGF3,LOC101928443*	.	.	.
11	69815821	G	A	intergenic	*FGF3,LOC101928443*	.	.	.
13	22334416	A	G	intergenic	*FGF9,LINC00424*	.	.	.
13	22436850	G	C	intergenic	*FGF9,LINC00424*	.	.	.
13	22436851	T	A	intergenic	*FGF9,LINC00424*	.	.	.
6	167416972	G	A	intronic	*FGFR1OP*	.	.	.
10	123485737	ATGTG	A	intergenic	*FGFR2,ATE1*	.	.	.
10	123407348	G	C	intergenic	*FGFR2,ATE1*	.	.	.
10	123407350	C	A	intergenic	*FGFR2,ATE1*	.	.	.
4	1051460	C	T	intergenic	*FGFRL1,RNF212*	.	.	.
4	155553269	C	T	intergenic	*FGG,LRAT*	.	.	.
1	59837438	G	A	intronic	*FGGY*	.	.	.
1	59839713	G	A	intronic	*FGGY*	.	.	.
1	59846177	A	G	intronic	*FGGY*	.	.	.
1	59846470	G	T	intronic	*FGGY*	.	.	.
1	60002252	G	A	intronic	*FGGY*	.	.	.
1	60080124	C	T	intronic	*FGGY*	.	.	.
1	60081382	G	A	intronic	*FGGY*	.	.	.
1	27965096	A	T	intergenic	*FGR,IFI6*	.	.	.
4	154023399	C	T	intergenic	*FHDC1,TRIM2*	.	.	.
3	61225237	A	G	intronic	*FHIT*	.	.	.
3	59902832	C	T	intronic	*FHIT*	.	.	.
3	59948925	G	A	intronic	*FHIT*	.	.	.
3	60683319	C	G	intronic	*FHIT*	.	.	.
3	60861321	A	G	intronic	*FHIT*	.	.	.
3	61197274	C	A	intronic	*FHIT*	.	.	.
3	60470548	C	T	intronic	*FHIT*	.	.	.
3	60914954	C	T	intronic	*FHIT*	.	.	.
3	60916014	T	C	intronic	*FHIT*	.	.	.
3	61173742	C	A	intronic	*FHIT*	.	.	.
3	61191662	C	T	intronic	*FHIT*	.	.	.
3	61193290	C	T	intronic	*FHIT*	.	.	.
3	61193291	C	G	intronic	*FHIT*	.	.	.
3	61201733	T	A	intronic	*FHIT*	.	.	.
3	61246366	T	C	intergenic	*FHIT,PTPRG*	.	.	.
2	106004483	G	A	intronic	*FHL2*	.	.	.

2	106182375	A	C	intergenic	*FHL2,LOC285000*	.	.	.
2	106185055	C	T	intergenic	*FHL2,LOC285000*	.	.	.
6	97088375	T	C	intergenic	*FHL5,GPR63*	.	.	.
6	97090258	T	G	intergenic	*FHL5,GPR63*	.	.	.
6	97090509	C	A	intergenic	*FHL5,GPR63*	.	.	.
18	34131917	T	G	intronic	*FHOD3*	.	.	.
18	34266146	T	C	intronic	*FHOD3*	.	.	.
12	108910860	G	A	exonic	*FICD*	synonymous_SNV	.	.
6	110067283	C	A	intronic	*FIG4*	.	.	.
6	110072873	T	C	intronic	*FIG4*	.	.	.
6	110074241	T	G	intronic	*FIG4*	.	.	.
6	110163841	T	A	intergenic	*FIG4,GPR6*	.	.	.
2	164525525	AATAT	A	intronic	*FIGN*	.	.	.
2	164718061	A	G	intergenic	*FIGN,GRB14*	.	.	.
2	164986545	A	G	intergenic	*FIGN,GRB14*	.	.	.
2	165139694	T	C	intergenic	*FIGN,GRB14*	.	.	.
2	164974432	TTATA	T	intergenic	*FIGN,GRB14*	.	.	.
2	165114333	C	T	intergenic	*FIGN,GRB14*	.	.	.
2	165319176	T	TCG	intergenic	*FIGN,GRB14*	.	.	.
2	165319177	TAG	T	intergenic	*FIGN,GRB14*	.	.	.
2	164636713	A	G	intergenic	*FIGN,GRB14*	.	.	.
2	165185193	T	C	intergenic	*FIGN,GRB14*	.	.	.
2	164943998	T	A	intergenic	*FIGN,GRB14*	.	.	.
2	164978322	C	T	intergenic	*FIGN,GRB14*	.	.	.
2	165120942	T	A	intergenic	*FIGN,GRB14*	.	.	.
6	76173023	C	T	intronic	*FILIP1*	.	.	.
6	76216521	C	T	intergenic	*FILIP1,SENP6*	.	.	.
6	76216522	A	G	intergenic	*FILIP1,SENP6*	.	.	.
6	76283373	A	AC	intergenic	*FILIP1,SENP6*	.	.	.
4	54273219	T	C	intronic	*FIP1L1*	.	.	.
X	130944809	T	C	ncRNA_intronic	*FIRRE*	.	.	.
X	130892779	G	A	ncRNA_intronic	*FIRRE*	.	.	.
X	131047586	A	T	intergenic	*FIRRE,STK26*	.	.	.
X	131047588	A	C	intergenic	*FIRRE,STK26*	.	.	.
X	131125807	T	A	intergenic	*FIRRE,STK26*	.	.	.
X	131127183	T	C	intergenic	*FIRRE,STK26*	.	.	.
X	131127653	C	T	intergenic	*FIRRE,STK26*	.	.	.
X	131127654	A	G	intergenic	*FIRRE,STK26*	.	.	.

11	35654565	A	T	intergenic	*FJX1,TRIM44*	.	.	.
6	35682546	G	A	intronic	*FKBP5*	.	.	.
7	55773257	T	G	upstream	*FKBP9P1*	.	.	.
7	55832978	C	T	intergenic	*FKBP9P1,SEPT14*	.	.	.
9	108334511	GTATATA	G	intronic	*FKTN*	.	.	.
5	174593714	G	A	intergenic	*FLJ16171,DRD1*	.	.	.
5	174458653	C	T	intergenic	*FLJ16171,DRD1*	.	.	.
5	174475694	A	G	intergenic	*FLJ16171,DRD1*	.	.	.
4	102651081	A	AAAT	intergenic	*FLJ20021,BANK1*	.	.	.
4	102431063	G	C	intergenic	*FLJ20021,BANK1*	.	.	.
4	102490903	G	A	intergenic	*FLJ20021,BANK1*	.	.	.
4	102660421	T	A	intergenic	*FLJ20021,BANK1*	.	.	.
4	102674055	T	C	intergenic	*FLJ20021,BANK1*	.	.	.
16	35041487	G	T	intergenic	*FLJ26245,NONE*	.	.	.
16	35260257	G	T	intergenic	*FLJ26245,NONE*	.	.	.
16	35060033	C	T	intergenic	*FLJ26245,NONE*	.	.	.
16	35068488	C	T	intergenic	*FLJ26245,NONE*	.	.	.
16	35068489	A	G	intergenic	*FLJ26245,NONE*	.	.	.
16	35109214	G	T	intergenic	*FLJ26245,NONE*	.	.	.
16	35197911	T	G	intergenic	*FLJ26245,NONE*	.	.	.
16	35224698	A	G	intergenic	*FLJ26245,NONE*	.	.	.
16	35235043	T	G	intergenic	*FLJ26245,NONE*	.	.	.
16	35261904	A	C	intergenic	*FLJ26245,NONE*	.	.	.
16	35263300	T	C	intergenic	*FLJ26245,NONE*	.	.	.
5	55365623	ATT	A	intergenic	*FLJ31104,ANKRD55*	.	.	.
20	24194611	ATCTC	A	ncRNA_intronic	*FLJ33581*	.	.	.
20	24382871	T	A	intergenic	*FLJ33581,SYNDIG1*	.	.	.
20	24299043	G	T	intergenic	*FLJ33581,SYNDIG1*	.	.	.
20	24208144	G	T	intergenic	*FLJ33581,SYNDIG1*	.	.	.
20	24339940	G	C	intergenic	*FLJ33581,SYNDIG1*	.	.	.
20	24372667	G	T	intergenic	*FLJ33581,SYNDIG1*	.	.	.

20	24398438	C	A	intergenic	*FLJ33581,SYNDIG1*	.	.	.
20	24377170	A	G	intergenic	*FLJ33581,SYNDIG1*	.	.	.
20	24378307	T	G	intergenic	*FLJ33581,SYNDIG1*	.	.	.
17	21934532	T	C	intergenic	*FLJ36000,MTRNR2L1*	.	.	.
17	21934533	G	C	intergenic	*FLJ36000,MTRNR2L1*	.	.	.
17	21997978	T	C	intergenic	*FLJ36000,MTRNR2L1*	.	.	.
17	21998683	G	GC	intergenic	*FLJ36000,MTRNR2L1*	.	.	.
17	22013170	A	T	intergenic	*FLJ36000,MTRNR2L1*	.	.	.
17	47354912	A	G	intergenic	*FLJ40194,MIR6129*	.	.	.
7	132337361	C	T	ncRNA_intronic	*FLJ40288*	.	.	.
7	132360869	A	G	ncRNA_intronic	*FLJ40288*	.	.	.
9	13454386	G	T	intergenic	*FLJ41200,LINC00583*	.	.	.
9	13615936	A	T	intergenic	*FLJ41200,LINC00583*	.	.	.
9	13541290	C	T	intergenic	*FLJ41200,LINC00583*	.	.	.
9	13700365	T	C	intergenic	*FLJ41200,LINC00583*	.	.	.
9	13748541	C	G	intergenic	*FLJ41200,LINC00583*	.	.	.
9	13748543	A	G	intergenic	*FLJ41200,LINC00583*	.	.	.
9	13748988	C	A	intergenic	*FLJ41200,LINC00583*	.	.	.
12	65327114	AGTGT	A	ncRNA_intronic	*FLJ41278*	.	.	.
12	65354956	C	G	ncRNA_intronic	*FLJ41278*	.	.	.
12	65428978	T	C	intergenic	*FLJ41278,WIF1*	.	.	.
12	65425076	A	G	intergenic	*FLJ41278,WIF1*	.	.	.

12	65427326	A	G	intergenic	*FLJ41278,WIF1*	.	.	.
2	239873793	C	G	intergenic	*FLJ43879,HDAC4*	.	.	.
X	71371133	C	A	intronic	*FLJ44635*	.	.	.
X	71396000	A	G	intergenic	*FLJ44635,PIN4*	.	.	.
17	75906759	T	A	intergenic	*FLJ45079,TNRC6C*	.	.	.
17	75907922	A	C	intergenic	*FLJ45079,TNRC6C*	.	.	.
17	48003918	C	A	intergenic	*FLJ45513,DLX4*	.	.	.
3	182258441	G	A	intergenic	*FLJ46066,ATP11B*	.	.	.
8	93757339	G	T	ncRNA_intronic	*FLJ46284*	.	.	.
6	139028453	A	T	intergenic	*FLJ46906,GVQW2*	.	.	.
6	139030668	C	A	intergenic	*FLJ46906,GVQW2*	.	.	.
3	58171526	G	A	intergenic	*FLNB,DNASE1L3*	.	.	.
7	128474548	CAGAA	C	intronic	*FLNC*	.	.	.
14	86029634	C	A	intronic	*FLRT2*	.	.	.
14	86371299	A	C	intergenic	*FLRT2,LOC101928767*	.	.	.
14	86270996	A	G	intergenic	*FLRT2,LOC101928767*	.	.	.
14	86372780	C	T	intergenic	*FLRT2,LOC101928767*	.	.	.
14	86372781	C	G	intergenic	*FLRT2,LOC101928767*	.	.	.
13	28910651	C	T	intronic	*FLT1*	.	.	.
13	29011782	A	G	intronic	*FLT1*	.	.	.
13	28996356	G	T	intronic	*FLT1*	.	.	.
13	29021496	A	AT	intronic	*FLT1*	.	.	.
13	29205703	A	G	intergenic	*FLT1,POMP*	.	.	.
13	29087325	C	G	intergenic	*FLT1,POMP*	.	.	.
13	28708302	G	C	intergenic	*FLT3,PAN3-AS1*	.	.	.
1	213080262	G	A	intergenic	*FLVCR1,VASH2*	.	.	.
16	2939205	CTTTACTTTT	C	intronic	*FLYWCH2*	.	.	.
15	33060290	T	C	UTR3	*FMN1*	.	.	.
15	33097485	C	T	intronic	*FMN1*	.	.	.
15	33097487	A	T	intronic	*FMN1*	.	.	.
1	240523417	C	CA	intronic	*FMN2*	.	.	.
1	240426080	G	T	intronic	*FMN2*	.	.	.
1	240384410	C	T	intronic	*FMN2*	.	.	.

1	240384740	T	G	intronic	*FMN2*	.	.	.
1	240428059	G	A	intronic	*FMN2*	.	.	.
1	240576458	T	A	intronic	*FMN2*	.	.	.
1	240578481	T	A	intronic	*FMN2*	.	.	.
12	50104527	C	T	intergenic	*FMNL3,TMBIM6*	.	.	.
1	171074202	T	A	intronic	*FMO3*	.	.	.
1	171074636	G	A	intronic	*FMO3*	.	.	.
1	171334192	A	G	intergenic	*FMO4,PRRC2C*	.	.	.
1	146664871	T	C	intronic	*FMO5*	.	.	.
1	146664236	G	A	intronic	*FMO5*	.	.	.
1	146664364	A	T	intronic	*FMO5*	.	.	.
1	146665123	T	G	intronic	*FMO5*	.	.	.
1	146703822	T	C	intergenic	*FMO5,CHD1L*	.	.	.
1	146702350	T	C	intergenic	*FMO5,CHD1L*	.	.	.
1	146703236	G	GT	intergenic	*FMO5,CHD1L*	.	.	.
1	171148373	A	C	intergenic	*FMO6P,FMO2*	.	.	.
1	166789556	G	A	intergenic	*FMO9P,POGK*	.	.	.
1	166636628	A	C	intergenic	*FMO9P,POGK*	.	.	.
1	166665331	A	C	intergenic	*FMO9P,POGK*	.	.	.
1	166665974	C	G	intergenic	*FMO9P,POGK*	.	.	.
1	166676390	A	T	intergenic	*FMO9P,POGK*	.	.	.
1	166731182	A	T	intergenic	*FMO9P,POGK*	.	.	.
1	166752315	C	T	intergenic	*FMO9P,POGK*	.	.	.
1	166756693	C	T	intergenic	*FMO9P,POGK*	.	.	.
1	166769198	A	G	intergenic	*FMO9P,POGK*	.	.	.
1	166779132	C	T	intergenic	*FMO9P,POGK*	.	.	.
1	166779134	A	C	intergenic	*FMO9P,POGK*	.	.	.
1	166779993	G	T	intergenic	*FMO9P,POGK*	.	.	.
1	203384291	T	A	intergenic	*FMOD,PRELP*	.	.	.
1	203384293	C	T	intergenic	*FMOD,PRELP*	.	.	.
1	203385598	C	T	intergenic	*FMOD,PRELP*	.	.	.
1	203385600	G	C	intergenic	*FMOD,PRELP*	.	.	.
1	203426128	G	A	intergenic	*FMOD,PRELP*	.	.	.
X	147051791	T	C	intergenic	*FMR1,FMR1NB*	.	.	.
X	147068221	T	C	intronic	*FMR1NB*	.	.	.
X	147068243	T	C	intronic	*FMR1NB*	.	.	.
X	147079082	A	T	intronic	*FMR1NB*	.	.	.
X	147079760	A	G	intronic	*FMR1NB*	.	.	.

X	147100444	G	A	intronic	*FMR1NB*	.	.	.
X	147439117	CATAGATA G	C	intergenic	*FMR1NB,AFF2*	.	.	.
X	147512414	G	C	intergenic	*FMR1NB,AFF2*	.	.	.
X	147183146	C	A	intergenic	*FMR1NB,AFF2*	.	.	.
X	147165534	G	C	intergenic	*FMR1NB,AFF2*	.	.	.
X	147165535	G	A	intergenic	*FMR1NB,AFF2*	.	.	.
X	147245542	C	T	intergenic	*FMR1NB,AFF2*	.	.	.
X	147251212	T	C	intergenic	*FMR1NB,AFF2*	.	.	.
X	147258645	A	T	intergenic	*FMR1NB,AFF2*	.	.	.
X	147320827	A	G	intergenic	*FMR1NB,AFF2*	.	.	.
X	147321600	A	T	intergenic	*FMR1NB,AFF2*	.	.	.
X	147384995	T	C	intergenic	*FMR1NB,AFF2*	.	.	.
X	147431218	T	G	intergenic	*FMR1NB,AFF2*	.	.	.
X	147432608	G	T	intergenic	*FMR1NB,AFF2*	.	.	.
X	147439117	CATAGATA GATAGATA GATAG	C	intergenic	*FMR1NB,AFF2*	.	.	.
X	147440406	T	C	intergenic	*FMR1NB,AFF2*	.	.	.
9	132690054	A	C	intronic	*FNBP1*	.	.	.
9	132734263	A	G	intronic	*FNBP1*	.	.	.
9	132735872	T	C	intronic	*FNBP1*	.	.	.
6	159692389	T	C	exonic	*FNDC1*	nonsynonymous_SN V	.	.
6	159705860	C	A	intergenic	*FNDC1,LOC102724 053*	.	.	.
6	159706523	C	G	intergenic	*FNDC1,LOC102724 053*	.	.	.
6	159787877	T	C	intergenic	*FNDC1,LOC102724 053*	.	.	.
13	49629288	G	T	intronic	*FNDC3A*	.	.	.
13	49590490	G	T	intronic	*FNDC3A*	.	.	.
13	49697522	G	A	intronic	*FNDC3A*	.	.	.
13	49727822	G	A	intronic	*FNDC3A*	.	.	.
1	109281176	T	C	intronic	*FNDC7*	.	.	.
5	130993459	T	G	intronic	*FNIP1*	.	.	.
5	131059753	C	T	intronic	*FNIP1*	.	.	.
5	131061111	A	ACATC	intronic	*FNIP1*	.	.	.

5	131068454	A	G	intronic	*FNIP1*	.	.	.
4	159800931	A	G	intronic	*FNIP2*	.	.	.
9	20851330	T	C	intronic	*FOCAD*	.	.	.
9	20883071	A	G	intronic	*FOCAD*	.	.	.
9	20919530	A	T	intronic	*FOCAD*	.	.	.
9	20920050	T	G	intronic	*FOCAD*	.	.	.
11	49181554	A	C	intronic	*FOLH1*	.	.	.
11	49563677	TTTTTGTAA AGTGAACA A	T	intergenic	*FOLH1,LOC440040*	.	.	.
11	49333901	C	G	intergenic	*FOLH1,LOC440040*	.	.	.
11	71912567	T	C	intergenic	*FOLR1,FOLR2*	.	.	.
15	60322760	C	T	intergenic	*FOXB1,ANXA2*	.	.	.
1	48076277	G	T	intergenic	*FOXD2,TRABD2B*	.	.	.
9	116010	C	CT	downstream	*FOXD4*	.	.	.
6	1419997	C	T	intergenic	*FOXF2,FOXCUT*	.	.	.
10	129586189	A	G	intergenic	*FOXI2,CLRN3*	.	.	.
10	129608848	C	T	intergenic	*FOXI2,CLRN3*	.	.	.
2	88806798	T	C	intergenic	*FOXI3,TEX37*	.	.	.
1	42717249	T	C	intronic	*FOXJ3*	.	.	.
7	4737943	GTATA	G	intronic	*FOXK1*	.	.	.
7	4766266	C	A	intronic	*FOXK1*	.	.	.
16	86782884	G	A	intergenic	*FOXL1,LOC440390*	.	.	.
16	86923256	A	AATGGATGG	intergenic	*FOXL1,LOC440390*	.	.	.
16	86743940	G	A	intergenic	*FOXL1,LOC440390*	.	.	.
14	90074788	C	CCCAG	intronic	*FOXN3*	.	.	.
14	89759378	C	A	intronic	*FOXN3*	.	.	.
14	89754413	ATAT	A	intronic	*FOXN3*	.	.	.
14	89980249	TACACAC	T	intronic	*FOXN3*	.	.	.
13	41299512	G	C	intergenic	*FOXO1,MIR320D1*	.	.	.
13	41250997	G	C	intergenic	*FOXO1,MIR320D1*	.	.	.
13	41265592	A	C	intergenic	*FOXO1,MIR320D1*	.	.	.
13	41284668	C	T	intergenic	*FOXO1,MIR320D1*	.	.	.
3	71258938	G	A	intronic	*FOXP1*	.	.	.

3	71609235	C	A	intronic	*FOXP1*	.	.	.
3	71272320	A	G	intronic	*FOXP1*	.	.	.
3	71063101	G	T	intronic	*FOXP1*	.	.	.
3	71593509	CAAAGG	C	intronic	*FOXP1*	.	.	.
3	71715214	G	A	intergenic	*FOXP1,EIF4E3*	.	.	.
3	71715490	G	A	intergenic	*FOXP1,EIF4E3*	.	.	.
7	114266586	ATCTC	A	intronic	*FOXP2*	.	.	.
7	114311842	A	C	intronic	*FOXP2*	.	.	.
7	114132454	A	G	intronic	*FOXP2*	.	.	.
7	114156195	T	C	intronic	*FOXP2*	.	.	.
7	114404276	T	A	intergenic	*FOXP2,MDFIC*	.	.	.
7	114534475	T	C	intergenic	*FOXP2,MDFIC*	.	.	.
6	41493601	A	T	ncRNA_intronic	*FOXP4-AS1*	.	.	.
6	41579391	A	G	intergenic	*FOXP4,MDFI*	.	.	.
X	55704792	G	T	intergenic	*FOXR2,RRAGB*	.	.	.
X	55697115	G	T	intergenic	*FOXR2,RRAGB*	.	.	.
X	55656510	G	C	intergenic	*FOXR2,RRAGB*	.	.	.
X	55656511	T	C	intergenic	*FOXR2,RRAGB*	.	.	.
X	55692088	A	G	intergenic	*FOXR2,RRAGB*	.	.	.
X	55692297	A	C	intergenic	*FOXR2,RRAGB*	.	.	.
X	55697629	T	G	intergenic	*FOXR2,RRAGB*	.	.	.
X	55710606	T	A	intergenic	*FOXR2,RRAGB*	.	.	.
X	55731996	A	G	intergenic	*FOXR2,RRAGB*	.	.	.
X	55734010	T	A	intergenic	*FOXR2,RRAGB*	.	.	.
X	55734012	A	T	intergenic	*FOXR2,RRAGB*	.	.	.
1	75015424	C	A	intergenic	*FPGT-TNNI3K,ERICH3*	.	.	.
1	75006757	C	G	intronic	*FPGT-TNNI3K,TNNI3K*	.	.	.
4	79071534	C	G	intronic	*FRAS1*	.	.	.
4	79152448	T	TTA	intronic	*FRAS1*	.	.	.
4	79217334	A	G	intronic	*FRAS1*	.	.	.
4	79037997	T	A	intronic	*FRAS1*	.	.	.
4	79049947	T	C	intronic	*FRAS1*	.	.	.
4	79049948	G	A	intronic	*FRAS1*	.	.	.
4	79113044	T	C	intronic	*FRAS1*	.	.	.
4	79223932	C	T	intronic	*FRAS1*	.	.	.
4	79425007	A	G	intronic	*FRAS1*	.	.	.

4	79452194	A	G	intronic	*FRAS1*	.	.	.
4	79452342	A	T	intronic	*FRAS1*	.	.	.
4	79452344	T	G	intronic	*FRAS1*	.	.	.
9	14952396	C	CA	intergenic	*FREM1,LOC389705*	.	.	.
9	14952534	T	G	intergenic	*FREM1,LOC389705*	.	.	.
13	39392589	G	T	intronic	*FREM2*	.	.	.
13	39392590	A	T	intronic	*FREM2*	.	.	.
13	39409500	A	G	intronic	*FREM2*	.	.	.
13	39409586	T	G	intronic	*FREM2*	.	.	.
13	39409884	A	G	intronic	*FREM2*	.	.	.
13	39411833	C	G	intronic	*FREM2*	.	.	.
4	144583386	C	T	intronic	*FREM3*	.	.	.
4	144596870	T	C	intronic	*FREM3*	.	.	.
4	144776540	C	CAG	intergenic	*FREM3,GYPE*	.	.	.
4	144675351	A	T	intergenic	*FREM3,GYPE*	.	.	.
4	144677098	C	T	intergenic	*FREM3,GYPE*	.	.	.
4	190871187	G	T	intronic	*FRG1*	.	.	.
4	190941803	G	A	intergenic	*FRG1,FRG2*	.	.	.
20	29615944	G	T	ncRNA_intronic	*FRG1BP,FRG1DP*	.	.	.
20	29611463	C	A	upstream	*FRG1BP,FRG1DP*	.	.	.
20	29617578	T	C	ncRNA_intronic	*FRG1BP,FRG1DP*	.	.	.
9	68454808	C	T	ncRNA_intronic	*FRG1JP*	.	.	.
9	68444563	C	T	ncRNA_intronic	*FRG1JP*	.	.	.
4	190976679	C	T	intergenic	*FRG2,DBET*	.	.	.
10	135465878	C	CCTT	intergenic	*FRG2B,NONE*	.	.	.
10	135466504	T	A	intergenic	*FRG2B,NONE*	.	.	.
10	135511762	A	G	intergenic	*FRG2B,NONE*	.	.	.
6	116367582	A	G	intronic	*FRK*	.	.	.
6	168584123	A	G	intergenic	*FRMD1,LOC101929420*	.	.	.
9	86065130	A	G	intronic	*FRMD3*	.	.	.
9	86131736	A	C	intronic	*FRMD3*	.	.	.
9	86133387	A	G	intronic	*FRMD3*	.	.	.
9	86134087	T	C	intronic	*FRMD3*	.	.	.
9	86134327	T	C	intronic	*FRMD3*	.	.	.
9	86164860	A	T	intergenic	*FRMD3,IDNK*	.	.	.

3	69778440	A	T	intergenic	*FRMD4B,MITF*	.	.	.
3	69548842	CAGAG	C	intergenic	*FRMD4B,MITF*	.	.	.
3	69600960	G	A	intergenic	*FRMD4B,MITF*	.	.	.
3	69466710	G	A	intergenic	*FRMD4B,MITF*	.	.	.
3	69515293	C	T	intergenic	*FRMD4B,MITF*	.	.	.
3	69682178	G	A	intergenic	*FRMD4B,MITF*	.	.	.
15	44507716	A	G	intergenic	*FRMD5,CASC4*	.	.	.
14	52055050	T	A	ncRNA_intronic	*FRMD6-AS2*	.	.	.
14	52270220	G	C	intergenic	*FRMD6,GNG2*	.	.	.
14	52324945	T	C	intergenic	*FRMD6,GNG2*	.	.	.
X	131279148	CATAT	C	intergenic	*FRMD7,RAP2C*	.	.	.
X	64841716	G	T	intergenic	*FRMD8P1,MSN*	.	.	.
X	64837895	A	T	intergenic	*FRMD8P1,MSN*	.	.	.
10	49438756	T	G	intronic	*FRMPD2*	.	.	.
X	106830146	A	G	intronic	*FRMPD3*	.	.	.
X	106755275	ACACACAC ACACGCG	A	downstream	*FRMPD3-AS1*	.	.	.
X	12180180	T	TAAAAAAG	intronic	*FRMPD4*	.	.	.
X	12184977	C	T	intronic	*FRMPD4*	.	.	.
X	12491562	T	G	intronic	*FRMPD4*	.	.	.
X	12492627	C	T	intronic	*FRMPD4*	.	.	.
X	12559068	C	A	intronic	*FRMPD4*	.	.	.
X	12559575	C	A	intronic	*FRMPD4*	.	.	.
X	12682078	T	A	intronic	*FRMPD4*	.	.	.
12	69918898	C	T	intronic	*FRS2*	.	.	.
12	69872441	A	G	intronic	*FRS2*	.	.	.
13	32756395	A	C	intronic	*FRY*	.	.	.
4	48587395	T	G	intronic	*FRYL*	.	.	.
4	48698282	A	T	intronic	*FRYL*	.	.	.
4	48715193	A	G	intronic	*FRYL*	.	.	.
4	48717177	C	A	intronic	*FRYL*	.	.	.
4	48733955	C	T	intronic	*FRYL*	.	.	.
4	48734283	C	T	intronic	*FRYL*	.	.	.
4	48736726	G	A	intronic	*FRYL*	.	.	.
4	48759697	C	G	intronic	*FRYL*	.	.	.
4	48815003	T	C	intergenic	*FRYL,OCIAD1*	.	.	.
2	183742581	T	A	intergenic	*FRZB,NCKAP1*	.	.	.
2	183744266	T	A	intergenic	*FRZB,NCKAP1*	.	.	.

2	183744989	C	T	intergenic	*FRZB,NCKAP1*	.	.	.
14	45060270	C	T	intergenic	*FSCB,C14orf28*	.	.	.
14	45014278	C	G	intergenic	*FSCB,C14orf28*	.	.	.
14	45297148	A	T	intergenic	*FSCB,C14orf28*	.	.	.
14	45052276	A	G	intergenic	*FSCB,C14orf28*	.	.	.
14	45152597	C	G	intergenic	*FSCB,C14orf28*	.	.	.
14	45165533	T	C	intergenic	*FSCB,C14orf28*	.	.	.
14	45167031	T	C	intergenic	*FSCB,C14orf28*	.	.	.
14	45324353	A	G	intergenic	*FSCB,C14orf28*	.	.	.
14	45327612	C	T	intergenic	*FSCB,C14orf28*	.	.	.
19	4320709	G	A	intronic	*FSD1*	.	.	.
9	108254731	G	C	intronic	*FSD1L*	.	.	.
2	49348110	C	T	intronic	*FSHR*	.	.	.
2	49371669	G	C	intronic	*FSHR*	.	.	.
2	49377244	C	T	intronic	*FSHR*	.	.	.
2	49216853	A	G	intronic	*FSHR*	.	.	.
2	49355109	G	A	intronic	*FSHR*	.	.	.
2	49311571	G	A	intronic	*FSHR*	.	.	.
2	49361587	T	C	intronic	*FSHR*	.	.	.
2	49361588	G	A	intronic	*FSHR*	.	.	.
2	49856439	C	A	intergenic	*FSHR,NRXN1*	.	.	.
2	49429745	A	G	intergenic	*FSHR,NRXN1*	.	.	.
2	49435572	A	G	intergenic	*FSHR,NRXN1*	.	.	.
2	50098472	G	A	intergenic	*FSHR,NRXN1*	.	.	.
2	50138156	CA	C	intergenic	*FSHR,NRXN1*	.	.	.
2	49523224	C	T	intergenic	*FSHR,NRXN1*	.	.	.
2	50102416	T	G	intergenic	*FSHR,NRXN1*	.	.	.
2	49441832	G	A	intergenic	*FSHR,NRXN1*	.	.	.
2	49456475	G	T	intergenic	*FSHR,NRXN1*	.	.	.
2	49456477	T	A	intergenic	*FSHR,NRXN1*	.	.	.
2	49511033	T	A	intergenic	*FSHR,NRXN1*	.	.	.
2	49528007	T	A	intergenic	*FSHR,NRXN1*	.	.	.
2	49531466	A	G	intergenic	*FSHR,NRXN1*	.	.	.
2	49603281	C	T	intergenic	*FSHR,NRXN1*	.	.	.
2	49656381	G	T	intergenic	*FSHR,NRXN1*	.	.	.
2	49656777	G	C	intergenic	*FSHR,NRXN1*	.	.	.
2	49710609	C	A	intergenic	*FSHR,NRXN1*	.	.	.
2	49712584	A	G	intergenic	*FSHR,NRXN1*	.	.	.

2	49772575	C	T	intergenic	*FSHR,NRXN1*	.	.	.
2	49773888	C	T	intergenic	*FSHR,NRXN1*	.	.	.
2	50053224	A	G	intergenic	*FSHR,NRXN1*	.	.	.
2	50075785	A	T	intergenic	*FSHR,NRXN1*	.	.	.
2	50075787	A	T	intergenic	*FSHR,NRXN1*	.	.	.
2	50103348	G	A	intergenic	*FSHR,NRXN1*	.	.	.
15	40021884	C	T	intronic	*FSIP1*	.	.	.
15	40082470	T	C	intergenic	*FSIP1,GPR176*	.	.	.
2	186892260	G	A	intergenic	*FSIP2,LINC01473*	.	.	.
2	186865056	G	A	intergenic	*FSIP2,LINC01473*	.	.	.
2	186894951	T	G	intergenic	*FSIP2,LINC01473*	.	.	.
2	186890369	G	A	intergenic	*FSIP2,LINC01473*	.	.	.
2	186713047	A	G	intergenic	*FSIP2,LINC01473*	.	.	.
2	186731285	G	A	intergenic	*FSIP2,LINC01473*	.	.	.
2	186732245	C	T	intergenic	*FSIP2,LINC01473*	.	.	.
2	186733025	A	T	intergenic	*FSIP2,LINC01473*	.	.	.
2	186810722	G	A	intergenic	*FSIP2,LINC01473*	.	.	.
2	186810724	A	G	intergenic	*FSIP2,LINC01473*	.	.	.
2	186879255	T	C	intergenic	*FSIP2,LINC01473*	.	.	.
5	52796497	C	A	intergenic	*FST,NDUFS4*	.	.	.
5	52794709	G	C	intergenic	*FST,NDUFS4*	.	.	.
3	120288768	G	C	intergenic	*FSTL1,NDUFB4*	.	.	.
5	132965066	T	C	intergenic	*FSTL4,WSPAR*	.	.	.
5	132966263	A	G	intergenic	*FSTL4,WSPAR*	.	.	.
5	132966583	A	G	intergenic	*FSTL4,WSPAR*	.	.	.
4	162306294	T	C	UTR3	*FSTL5*	.	.	.
4	162407323	T	A	intronic	*FSTL5*	.	.	.
4	162518060	C	T	intronic	*FSTL5*	.	.	.
4	162539872	T	G	intronic	*FSTL5*	.	.	.
4	162753834	C	T	intronic	*FSTL5*	.	.	.
4	162757291	C	T	intronic	*FSTL5*	.	.	.
4	163160349	A	C	intergenic	*FSTL5,MIR4454*	.	.	.
4	163319842	G	A	intergenic	*FSTL5,MIR4454*	.	.	.
4	163389580	T	C	intergenic	*FSTL5,MIR4454*	.	.	.
4	163399169	T	A	intergenic	*FSTL5,MIR4454*	.	.	.
4	163538649	T	C	intergenic	*FSTL5,MIR4454*	.	.	.
4	163591979	C	A	intergenic	*FSTL5,MIR4454*	.	.	.
4	163591981	G	A	intergenic	*FSTL5,MIR4454*	.	.	.

4	163887352	C	T	intergenic	*FSTL5,MIR4454*	.	.	.
4	163921258	A	G	intergenic	*FSTL5,MIR4454*	.	.	.
4	163921259	A	G	intergenic	*FSTL5,MIR4454*	.	.	.
4	163922879	C	T	intergenic	*FSTL5,MIR4454*	.	.	.
4	163922880	A	G	intergenic	*FSTL5,MIR4454*	.	.	.
4	163923126	T	C	intergenic	*FSTL5,MIR4454*	.	.	.
4	163976731	G	A	intergenic	*FSTL5,MIR4454*	.	.	.
4	163979245	A	G	intergenic	*FSTL5,MIR4454*	.	.	.
4	163979246	G	A	intergenic	*FSTL5,MIR4454*	.	.	.
2	200660315	T	A	ncRNA_intronic	*FTCDNL1*	.	.	.
2	200754132	C	A	intergenic	*FTCDNL1,C2orf69*	.	.	.
X	37170586	T	TAC	intergenic	*FTH1P18,PRRG1*	.	.	.
X	37163228	A	G	intergenic	*FTH1P18,PRRG1*	.	.	.
X	37163230	G	A	intergenic	*FTH1P18,PRRG1*	.	.	.
X	37164304	A	G	intergenic	*FTH1P18,PRRG1*	.	.	.
X	31131036	G	T	intergenic	*FTHL17,DMD*	.	.	.
X	31128491	G	A	intergenic	*FTHL17,DMD*	.	.	.
X	31098451	C	T	intergenic	*FTHL17,DMD*	.	.	.
5	121257214	A	G	intergenic	*FTMT,SRFBP1*	.	.	.
5	121257216	A	T	intergenic	*FTMT,SRFBP1*	.	.	.
16	53801381	G	A	intronic	*FTO*	.	.	.
16	53801382	T	A	intronic	*FTO*	.	.	.
X	73308123	C	T	ncRNA_intronic	*FTX*	.	.	.
X	73496015	C	T	ncRNA_intronic	*FTX*	.	.	.
X	73428597	G	A	ncRNA_intronic	*FTX*	.	.	.
X	73295568	T	TA	ncRNA_intronic	*FTX*	.	.	.
X	73363529	A	G	ncRNA_intronic	*FTX*	.	.	.
X	73287185	T	G	ncRNA_intronic	*FTX,JPX*	.	.	.
X	73261675	T	G	ncRNA_intronic	*FTX,JPX*	.	.	.
X	44440879	T	C	intergenic	*FUNDC1,DUSP21*	.	.	.
X	44440880	G	A	intergenic	*FUNDC1,DUSP21*	.	.	.
X	154257831	T	C	intronic	*FUNDC2*	.	.	.
X	154257832	C	A	intronic	*FUNDC2*	.	.	.
2	84621743	A	G	intergenic	*FUNDC2P2,SUCLG1*	.	.	.
2	84583460	A	C	intergenic	*FUNDC2P2,SUCLG1*	.	.	.

2	84628347	T	C	intergenic	*FUNDC2P2,SUCLG1*	.	.	.
2	84628719	T	C	intergenic	*FUNDC2P2,SUCLG1*	.	.	.
19	5885891	C	T	intergenic	*FUT5,NDUFA11*	.	.	.
19	5835922	C	T	intronic	*FUT6*	.	.	.
14	66083948	TATA	T	intronic	*FUT8*	.	.	.
14	65961277	A	G	intronic	*FUT8*	.	.	.
14	65990504	C	A	intronic	*FUT8*	.	.	.
14	65990948	A	G	intronic	*FUT8*	.	.	.
14	66583090	A	T	intergenic	*FUT8,LINC00238*	.	.	.
14	66661097	C	A	intergenic	*FUT8,LINC00238*	.	.	.
14	66235558	G	A	intergenic	*FUT8,LINC00238*	.	.	.
14	66360382	G	C	intergenic	*FUT8,LINC00238*	.	.	.
14	66363987	A	G	intergenic	*FUT8,LINC00238*	.	.	.
14	66496836	A	T	intergenic	*FUT8,LINC00238*	.	.	.
14	66497122	G	C	intergenic	*FUT8,LINC00238*	.	.	.
14	66613697	C	A	intergenic	*FUT8,LINC00238*	.	.	.
14	66614033	G	A	intergenic	*FUT8,LINC00238*	.	.	.
14	66614034	T	C	intergenic	*FUT8,LINC00238*	.	.	.
14	66653466	C	T	intergenic	*FUT8,LINC00238*	.	.	.
14	66669580	A	G	intergenic	*FUT8,LINC00238*	.	.	.
14	66670058	T	C	intergenic	*FUT8,LINC00238*	.	.	.
14	66672196	C	A	intergenic	*FUT8,LINC00238*	.	.	.
14	66745303	A	G	intergenic	*FUT8,LINC00238*	.	.	.
14	66756278	T	A	intergenic	*FUT8,LINC00238*	.	.	.
14	66803529	A	T	intergenic	*FUT8,LINC00238*	.	.	.
14	66804956	G	A	intergenic	*FUT8,LINC00238*	.	.	.
6	96649654	C	T	intronic	*FUT9*	.	.	.
6	96587728	G	A	intronic	*FUT9*	.	.	.
6	96505843	C	A	intronic	*FUT9*	.	.	.
6	96575627	G	T	intronic	*FUT9*	.	.	.
6	96684081	T	C	intergenic	*FUT9,UFL1*	.	.	.
6	96788723	C	A	intergenic	*FUT9,UFL1*	.	.	.
6	96902955	TG	T	intergenic	*FUT9,UFL1*	.	.	.
6	96720124	C	T	intergenic	*FUT9,UFL1*	.	.	.
6	96886782	C	A	intergenic	*FUT9,UFL1*	.	.	.
6	96886783	A	G	intergenic	*FUT9,UFL1*	.	.	.

6	96888395	A	G	intergenic	*FUT9,UFL1*	.	.	.
6	96888752	T	C	intergenic	*FUT9,UFL1*	.	.	.
6	96922039	A	G	intergenic	*FUT9,UFL1*	.	.	.
6	96922782	A	G	intergenic	*FUT9,UFL1*	.	.	.
6	96943956	A	G	intergenic	*FUT9,UFL1*	.	.	.
6	96944023	C	G	intergenic	*FUT9,UFL1*	.	.	.
6	96944024	A	G	intergenic	*FUT9,UFL1*	.	.	.
5	39277965	A	G	intergenic	*FYB,C9*	.	.	.
5	39278869	A	T	intergenic	*FYB,C9*	.	.	.
5	39279096	T	G	intergenic	*FYB,C9*	.	.	.
3	45972039	A	G	intronic	*FYCO1*	.	.	.
6	112028447	T	C	intronic	*FYN*	.	.	.
6	112107761	GGTATACA CCCA	G	intronic	*FYN*	.	.	.
6	112243007	C	T	intergenic	*FYN,WISP3*	.	.	.
6	112252449	T	A	intergenic	*FYN,WISP3*	.	.	.
6	112323096	G	A	intergenic	*FYN,WISP3*	.	.	.
6	112349882	C	T	intergenic	*FYN,WISP3*	.	.	.
7	91030898	G	A	intergenic	*FZD1,MTERF1*	.	.	.
7	91249775	G	A	intergenic	*FZD1,MTERF1*	.	.	.
7	91300177	T	TAG	intergenic	*FZD1,MTERF1*	.	.	.
7	91017276	CAT	C	intergenic	*FZD1,MTERF1*	.	.	.
7	91047898	A	G	intergenic	*FZD1,MTERF1*	.	.	.
7	91106696	A	G	intergenic	*FZD1,MTERF1*	.	.	.
7	91107097	C	T	intergenic	*FZD1,MTERF1*	.	.	.
7	91333496	T	G	intergenic	*FZD1,MTERF1*	.	.	.
7	91333498	A	T	intergenic	*FZD1,MTERF1*	.	.	.
7	91354551	A	C	intergenic	*FZD1,MTERF1*	.	.	.
7	91363042	G	A	intergenic	*FZD1,MTERF1*	.	.	.
7	91363044	C	T	intergenic	*FZD1,MTERF1*	.	.	.
7	91392991	A	G	intergenic	*FZD1,MTERF1*	.	.	.
7	91393685	T	G	intergenic	*FZD1,MTERF1*	.	.	.
7	91423520	C	T	intergenic	*FZD1,MTERF1*	.	.	.
7	91469601	C	T	intergenic	*FZD1,MTERF1*	.	.	.
7	91469602	A	G	intergenic	*FZD1,MTERF1*	.	.	.
7	91477136	C	T	intergenic	*FZD1,MTERF1*	.	.	.
14	31034540	G	T	intronic	*G2E3*	.	.	.
4	144265610	T	A	intronic	*GAB1*	.	.	.

11	78066670	C	G	intronic	*GAB2*	.	.	.
22	17459951	T	C	intronic	*GAB4*	.	.	.
22	17459952	G	C	intronic	*GAB4*	.	.	.
22	17461095	T	C	intronic	*GAB4*	.	.	.
22	17479102	C	T	intronic	*GAB4*	.	.	.
22	17480266	C	T	intronic	*GAB4*	.	.	.
22	17496377	G	A	intergenic	*GAB4,CECR7*	.	.	.
22	17507196	T	C	intergenic	*GAB4,CECR7*	.	.	.
22	17507415	T	A	intergenic	*GAB4,CECR7*	.	.	.
22	17507689	G	C	intergenic	*GAB4,CECR7*	.	.	.
22	17507690	T	C	intergenic	*GAB4,CECR7*	.	.	.
9	101393468	A	T	intronic	*GABBR2*	.	.	.
15	50624049	C	T	intronic	*GABPB1*	.	.	.
5	161291252	G	A	intronic	*GABRA1*	.	.	.
5	161330399	A	G	intergenic	*GABRA1,LINC01202*	.	.	.
5	161331191	T	G	intergenic	*GABRA1,LINC01202*	.	.	.
5	161331849	C	T	intergenic	*GABRA1,LINC01202*	.	.	.
4	46290605	G	T	intronic	*GABRA2*	.	.	.
4	46350863	G	A	intronic	*GABRA2*	.	.	.
4	46651240	T	C	intergenic	*GABRA2,COX7B2*	.	.	.
4	46608440	A	G	intergenic	*GABRA2,COX7B2*	.	.	.
4	46722449	G	T	intergenic	*GABRA2,COX7B2*	.	.	.
4	46624306	G	A	intergenic	*GABRA2,COX7B2*	.	.	.
4	46427841	G	C	intergenic	*GABRA2,COX7B2*	.	.	.
4	46617811	G	T	intergenic	*GABRA2,COX7B2*	.	.	.
4	46517859	T	C	intergenic	*GABRA2,COX7B2*	.	.	.
4	46410613	T	C	intergenic	*GABRA2,COX7B2*	.	.	.
4	46410615	G	A	intergenic	*GABRA2,COX7B2*	.	.	.
4	46425272	T	C	intergenic	*GABRA2,COX7B2*	.	.	.
4	46467488	A	G	intergenic	*GABRA2,COX7B2*	.	.	.
4	46574978	A	G	intergenic	*GABRA2,COX7B2*	.	.	.
4	46665121	A	G	intergenic	*GABRA2,COX7B2*	.	.	.
4	46665685	C	T	intergenic	*GABRA2,COX7B2*	.	.	.
4	46667995	T	C	intergenic	*GABRA2,COX7B2*	.	.	.
4	46704479	C	T	intergenic	*GABRA2,COX7B2*	.	.	.

4	46727042	T	G	intergenic	*GABRA2,COX7B2*	.	.	.
X	151345513	G	T	intronic	*GABRA3*	.	.	.
X	151342105	T	G	intronic	*GABRA3*	.	.	.
X	151541278	C	A	intronic	*GABRA3*	.	.	.
X	151362174	A	G	intronic	*GABRA3*	.	.	.
X	151458326	TC	T	intronic	*GABRA3*	.	.	.
X	151458883	C	T	intronic	*GABRA3*	.	.	.
X	151461105	A	G	intronic	*GABRA3*	.	.	.
X	151461397	G	A	intronic	*GABRA3*	.	.	.
X	151555096	G	A	intronic	*GABRA3*	.	.	.
X	151585615	G	A	intronic	*GABRA3*	.	.	.
X	151587373	T	A	intronic	*GABRA3*	.	.	.
X	151714339	A	G	intergenic	*GABRA3,GABRQ*	.	.	.
X	151755312	G	T	intergenic	*GABRA3,GABRQ*	.	.	.
X	151777004	T	C	intergenic	*GABRA3,GABRQ*	.	.	.
X	151777005	T	C	intergenic	*GABRA3,GABRQ*	.	.	.
4	46946937	C	A	intronic	*GABRA4*	.	.	.
15	27150782	C	T	intronic	*GABRA5*	.	.	.
5	161181748	A	G	intergenic	*GABRA6,GABRA1*	.	.	.
5	161175172	T	A	intergenic	*GABRA6,GABRA1*	.	.	.
4	47303284	T	C	intronic	*GABRB1*	.	.	.
4	47217431	T	C	intronic	*GABRB1*	.	.	.
4	47360421	T	TAGAG	intronic	*GABRB1*	.	.	.
4	47137986	G	A	intronic	*GABRB1*	.	.	.
4	47152394	CACAG	C	intronic	*GABRB1*	.	.	.
4	47248294	CATAT	C	intronic	*GABRB1*	.	.	.
4	47234283	C	A	intronic	*GABRB1*	.	.	.
4	47081970	T	C	intronic	*GABRB1*	.	.	.
4	47197458	TAGATGATAGATAGATTAGATGATAGATAGATAGATA	T	intronic	*GABRB1*	.	.	.
4	47245464	A	G	intronic	*GABRB1*	.	.	.
4	47264257	T	A	intronic	*GABRB1*	.	.	.
4	47330335	G	T	intronic	*GABRB1*	.	.	.
4	47444514	A	C	intergenic	*GABRB1,COMMD8*	.	.	.
5	160735739	C	T	intronic	*GABRB2*	.	.	.

5	160735740	T	G	intronic	*GABRB2*	.	.	.
5	161064014	T	G	intergenic	*GABRB2,GABRA6*	.	.	.
5	161034086	A	G	intergenic	*GABRB2,GABRA6*	.	.	.
5	161081877	G	A	intergenic	*GABRB2,GABRA6*	.	.	.
5	161081919	A	T	intergenic	*GABRB2,GABRA6*	.	.	.
15	26871986	G	C	intronic	*GABRB3*	.	.	.
15	26949289	C	T	intronic	*GABRB3*	.	.	.
X	151271672	G	A	intergenic	*GABRE,MAGEA10-MAGEA5*	.	.	.
4	46114500	G	A	intronic	*GABRG1*	.	.	.
4	46108170	C	T	intronic	*GABRG1*	.	.	.
4	46118959	A	G	intronic	*GABRG1*	.	.	.
4	46050387	AGAAGGAAGGAAG	A	intronic	*GABRG1*	.	.	.
4	46063916	C	A	intronic	*GABRG1*	.	.	.
4	46065626	T	C	intronic	*GABRG1*	.	.	.
4	46168127	G	A	intergenic	*GABRG1,GABRA2*	.	.	.
4	46234141	C	T	intergenic	*GABRG1,GABRA2*	.	.	.
4	46237640	A	AG	intergenic	*GABRG1,GABRA2*	.	.	.
4	46237672	T	A	intergenic	*GABRG1,GABRA2*	.	.	.
5	162179873	C	A	intergenic	*GABRG2,CCNG1*	.	.	.
5	161650340	G	A	intergenic	*GABRG2,CCNG1*	.	.	.
5	162161236	A	T	intergenic	*GABRG2,CCNG1*	.	.	.
5	161663016	CCATCATATTGTGA	C	intergenic	*GABRG2,CCNG1*	.	.	.
5	162592084	C	A	intergenic	*GABRG2,CCNG1*	.	.	.
5	162717005	A	G	intergenic	*GABRG2,CCNG1*	.	.	.
5	162127246	G	A	intergenic	*GABRG2,CCNG1*	.	.	.
5	162054722	C	CCCTAAAAG	intergenic	*GABRG2,CCNG1*	.	.	.
5	162046813	T	C	intergenic	*GABRG2,CCNG1*	.	.	.
5	162105853	C	T	intergenic	*GABRG2,CCNG1*	.	.	.
5	162389642	GCACA	G	intergenic	*GABRG2,CCNG1*	.	.	.
5	161596954	A	G	intergenic	*GABRG2,CCNG1*	.	.	.
5	161731951	T	C	intergenic	*GABRG2,CCNG1*	.	.	.
5	161733367	A	G	intergenic	*GABRG2,CCNG1*	.	.	.
5	161774012	A	G	intergenic	*GABRG2,CCNG1*	.	.	.
5	161774884	T	A	intergenic	*GABRG2,CCNG1*	.	.	.
5	161774885	G	A	intergenic	*GABRG2,CCNG1*	.	.	.

5	161864668	T	A	intergenic	*GABRG2,CCNG1*	.	.	.
5	161864669	A	T	intergenic	*GABRG2,CCNG1*	.	.	.
5	162054449	A	C	intergenic	*GABRG2,CCNG1*	.	.	.
5	162054450	C	A	intergenic	*GABRG2,CCNG1*	.	.	.
5	162055558	A	G	intergenic	*GABRG2,CCNG1*	.	.	.
5	162057955	T	A	intergenic	*GABRG2,CCNG1*	.	.	.
5	162115644	C	T	intergenic	*GABRG2,CCNG1*	.	.	.
5	162117975	T	A	intergenic	*GABRG2,CCNG1*	.	.	.
5	162118677	A	T	intergenic	*GABRG2,CCNG1*	.	.	.
5	162118912	T	C	intergenic	*GABRG2,CCNG1*	.	.	.
5	162119954	G	A	intergenic	*GABRG2,CCNG1*	.	.	.
5	162140143	T	C	intergenic	*GABRG2,CCNG1*	.	.	.
5	162164221	C	T	intergenic	*GABRG2,CCNG1*	.	.	.
5	162275418	C	G	intergenic	*GABRG2,CCNG1*	.	.	.
5	162277066	C	G	intergenic	*GABRG2,CCNG1*	.	.	.
5	162277304	C	A	intergenic	*GABRG2,CCNG1*	.	.	.
5	162284218	T	C	intergenic	*GABRG2,CCNG1*	.	.	.
5	162400799	T	A	intergenic	*GABRG2,CCNG1*	.	.	.
5	162503654	C	T	intergenic	*GABRG2,CCNG1*	.	.	.
5	162544667	C	T	intergenic	*GABRG2,CCNG1*	.	.	.
5	162547210	C	T	intergenic	*GABRG2,CCNG1*	.	.	.
5	162548425	G	T	intergenic	*GABRG2,CCNG1*	.	.	.
5	162549016	T	G	intergenic	*GABRG2,CCNG1*	.	.	.
5	162595588	G	A	intergenic	*GABRG2,CCNG1*	.	.	.
5	162641065	T	C	intergenic	*GABRG2,CCNG1*	.	.	.
5	162641067	G	A	intergenic	*GABRG2,CCNG1*	.	.	.
5	162642039	T	A	intergenic	*GABRG2,CCNG1*	.	.	.
5	162643803	T	C	intergenic	*GABRG2,CCNG1*	.	.	.
5	162670608	G	T	intergenic	*GABRG2,CCNG1*	.	.	.
15	27890106	A	C	intergenic	*GABRG3,OCA2*	.	.	.
15	27831073	C	T	intergenic	*GABRG3,OCA2*	.	.	.
15	27875766	T	C	intergenic	*GABRG3,OCA2*	.	.	.
5	170227408	A	G	intronic	*GABRP*	.	.	.
X	151844038	A	G	intergenic	*GABRQ,MAGEA6*	.	.	.
X	151846322	C	A	intergenic	*GABRQ,MAGEA6*	.	.	.
6	90013441	T	C	intronic	*GABRR2*	.	.	.
6	90013442	G	A	intronic	*GABRR2*	.	.	.
2	16370241	G	T	intergenic	*GACAT3,FAM49A*	.	.	.

2	16603071	C	G	intergenic	*GACAT3,FAM49A*	.	.	.
2	16263470	A	T	intergenic	*GACAT3,FAM49A*	.	.	.
10	26632186	TACAC	T	intergenic	*GAD2,APBB1IP*	.	.	.
10	26673109	C	A	intergenic	*GAD2,APBB1IP*	.	.	.
10	26651597	G	T	intergenic	*GAD2,APBB1IP*	.	.	.
10	26694991	C	T	intergenic	*GAD2,APBB1IP*	.	.	.
19	2506583	A	G	intergenic	*GADD45B,GNG7*	.	.	.
19	2480914	G	GACTAAAGC	intergenic	*GADD45B,GNG7*	.	.	.
3	31117968	G	A	intergenic	*GADL1,MIR466*	.	.	.
3	30991289	CATAT	C	intergenic	*GADL1,MIR466*	.	.	.
3	31036193	G	T	intergenic	*GADL1,MIR466*	.	.	.
3	31098251	A	C	intergenic	*GADL1,MIR466*	.	.	.
3	31098253	T	C	intergenic	*GADL1,MIR466*	.	.	.
X	49444547	G	T	intergenic	*GAGE1,PAGE1*	.	.	.
X	49387442	C	T	intergenic	*GAGE1,PAGE1*	.	.	.
X	49393967	T	C	intergenic	*GAGE1,PAGE1*	.	.	.
X	49446858	GGTGT	G	intergenic	*GAGE1,PAGE1*	.	.	.
4	863535	G	GCA	intronic	*GAK*	.	.	.
14	88449340	A	G	intronic	*GALC*	.	.	.
14	88463609	C	T	intergenic	*GALC,GPR65*	.	.	.
15	49499741	G	T	intronic	*GALK2*	.	.	.
15	49554531	G	A	intronic	*GALK2*	.	.	.
15	49554799	C	T	intronic	*GALK2*	.	.	.
15	49556361	T	C	intronic	*GALK2*	.	.	.
15	49609607	T	C	intronic	*GALK2*	.	.	.
18	33391143	A	G	intergenic	*GALNT1,MIR187*	.	.	.
18	33303524	A	G	intergenic	*GALNT1,MIR187*	.	.	.
18	33374172	G	A	intergenic	*GALNT1,MIR187*	.	.	.
18	33375517	C	T	intergenic	*GALNT1,MIR187*	.	.	.
18	33394973	T	A	intergenic	*GALNT1,MIR187*	.	.	.
18	33448809	C	G	intergenic	*GALNT1,MIR187*	.	.	.
18	33480093	T	C	intergenic	*GALNT1,MIR187*	.	.	.
18	33480815	A	G	intergenic	*GALNT1,MIR187*	.	.	.
5	153627585	C	T	intronic	*GALNT10*	.	.	.
7	151757804	C	T	intronic	*GALNT11*	.	.	.
7	151760706	C	A	intronic	*GALNT11*	.	.	.
2	155029164	AGTGT	A	intronic	*GALNT13*	.	.	.
2	155178830	G	T	intronic	*GALNT13*	.	.	.

2	155241968	A	T	intronic	*GALNT13*	.	.	.
2	154936371	T	C	intronic	*GALNT13*	.	.	.
2	154984195	ATATATATATCCATATCCACACACACACGTGTGTGTGTGTGGATATGGATATATGTGTATATGTGCATATATGTATATGGG	A	intronic	*GALNT13*	.	.	.
2	155072461	T	A	intronic	*GALNT13*	.	.	.
2	154984163	AGATATGGATATATATGGTCATACATATAGTCATATATATATCCATATCCACACACACACGTGTGTGTGTGTG	A	intronic	*GALNT13*	.	.	.
2	154878582	A	G	intronic	*GALNT13*	.	.	.
2	154939661	A	G	intronic	*GALNT13*	.	.	.
2	31285784	TA	T	intronic	*GALNT14*	.	.	.
2	31285786	C	T	intronic	*GALNT14*	.	.	.
11	11297972	G	T	intronic	*GALNT18*	.	.	.
1	230238352	T	C	intronic	*GALNT2*	.	.	.
1	230236261	C	T	intronic	*GALNT2*	.	.	.
1	230236262	A	G	intronic	*GALNT2*	.	.	.
1	230433969	G	A	intergenic	*GALNT2,PGBD5*	.	.	.
2	166682202	T	C	intergenic	*GALNT3,LOC100506124*	.	.	.
2	158130711	G	A	intronic	*GALNT5*	.	.	.
2	158131899	A	G	intronic	*GALNT5*	.	.	.
4	174202523	G	A	intronic	*GALNT7*	.	.	.

4	173451478	T	G	intronic	*GALNTL6*	.	.	.
4	172889066	G	T	intronic	*GALNTL6*	.	.	.
4	173218409	G	C	intronic	*GALNTL6*	.	.	.
4	173700126	ACTCT	A	intronic	*GALNTL6*	.	.	.
4	173836461	C	T	intronic	*GALNTL6*	.	.	.
4	174062577	A	G	intergenic	*GALNTL6,GALNT7*	.	.	.
4	174003744	A	T	intergenic	*GALNTL6,GALNT7*	.	.	.
4	174003746	C	G	intergenic	*GALNTL6,GALNT7*	.	.	.
18	75062710	G	A	intergenic	*GALR1,LINC01029*	.	.	.
18	75315641	T	A	intergenic	*GALR1,LINC01029*	.	.	.
18	75464119	C	T	intergenic	*GALR1,LINC01029*	.	.	.
18	75481512	A	T	intergenic	*GALR1,LINC01029*	.	.	.
18	75465087	G	A	intergenic	*GALR1,LINC01029*	.	.	.
18	75005845	T	G	intergenic	*GALR1,LINC01029*	.	.	.
18	74998413	C	T	intergenic	*GALR1,LINC01029*	.	.	.
18	74999418	T	A	intergenic	*GALR1,LINC01029*	.	.	.
18	75000497	A	G	intergenic	*GALR1,LINC01029*	.	.	.
18	75137832	C	T	intergenic	*GALR1,LINC01029*	.	.	.
18	75187869	T	C	intergenic	*GALR1,LINC01029*	.	.	.
18	75192469	T	C	intergenic	*GALR1,LINC01029*	.	.	.
18	75292799	G	A	intergenic	*GALR1,LINC01029*	.	.	.
18	75293009	A	T	intergenic	*GALR1,LINC01029*	.	.	.
18	75293693	T	C	intergenic	*GALR1,LINC01029*	.	.	.
18	75294189	T	G	intergenic	*GALR1,LINC01029*	.	.	.
18	75335019	C	T	intergenic	*GALR1,LINC01029*	.	.	.
18	75352115	C	T	intergenic	*GALR1,LINC01029*	.	.	.
18	75354197	A	C	intergenic	*GALR1,LINC01029*	.	.	.
15	42641824	A	G	intronic	*GANC*	.	.	.
15	42595986	G	T	intronic	*GANC*	.	.	.
15	42597165	A	G	intronic	*GANC*	.	.	.
15	42597368	A	G	intronic	*GANC*	.	.	.
5	57814127	T	A	intergenic	*GAPT,LOC101928600*	.	.	.
5	57835078	T	C	intergenic	*GAPT,LOC101928600*	.	.	.
9	128078902	A	T	intronic	*GAPVD1*	.	.	.
9	128198371	G	T	intergenic	*GAPVD1,MAPKAP1*	.	.	.

18	30032885	G	T	intronic	*GAREM1*	.	.	.
18	29977408	A	G	intronic	*GAREM1*	.	.	.
18	29978423	C	T	intronic	*GAREM1*	.	.	.
18	29980106	T	C	intronic	*GAREM1*	.	.	.
18	29980909	A	T	intronic	*GAREM1*	.	.	.
18	30059523	C	T	intergenic	*GAREM1,WBP11P1*	.	.	.
9	130142901	C	CACTG	intronic	*GARNL3*	.	.	.
11	22820531	G	A	intronic	*GAS2*	.	.	.
11	22837744	CTG	C	intergenic	*GAS2,SVIP*	.	.	.
9	99984217	G	T	intergenic	*GAS2L1P2,LOC100499484*	.	.	.
9	99979988	A	AT	intergenic	*GAS2L1P2,LOC100499484*	.	.	.
12	101030539	G	A	intergenic	*GAS2L3,ANO4*	.	.	.
13	114534381	C	T	ncRNA_intronic	*GAS6-AS1*	.	.	.
13	114568047	C	T	ncRNA_exonic	*GAS6-AS2*	.	.	.
17	9903086	C	CA	intronic	*GAS7*	.	.	.
17	9899566	G	T	intronic	*GAS7*	.	.	.
10	8159804	A	G	intergenic	*GATA3,LINC00708*	.	.	.
10	8196998	A	G	intergenic	*GATA3,LINC00708*	.	.	.
19	19511833	C	T	intronic	*GATAD2A*	.	.	.
19	19496589	G	T	upstream	*GATAD2A*	.	.	.
4	152669438	G	A	intronic	*GATB*	.	.	.
7	99855596	T	C	intronic	*GATS*	.	.	.
4	22777727	A	C	intronic	*GBA3*	.	.	.
4	23057391	GGTGT	G	intergenic	*GBA3,MIR548AJ2*	.	.	.
4	23181618	T	C	intergenic	*GBA3,MIR548AJ2*	.	.	.
4	22865332	T	A	intergenic	*GBA3,MIR548AJ2*	.	.	.
4	22887833	C	G	intergenic	*GBA3,MIR548AJ2*	.	.	.
4	22889883	C	T	intergenic	*GBA3,MIR548AJ2*	.	.	.
4	22922528	T	C	intergenic	*GBA3,MIR548AJ2*	.	.	.
4	22965351	T	G	intergenic	*GBA3,MIR548AJ2*	.	.	.
4	23080805	G	A	intergenic	*GBA3,MIR548AJ2*	.	.	.
1	151333884	G	A	intergenic	*GBAT2,SELENBP1*	.	.	.
3	81677984	A	G	intronic	*GBE1*	.	.	.
3	81760809	A	G	intronic	*GBE1*	.	.	.

3	82671345	A	T	intergenic	*GBE1,LINC00971*	.	.	.
3	83082367	G	A	intergenic	*GBE1,LINC00971*	.	.	.
3	83864731	A	G	intergenic	*GBE1,LINC00971*	.	.	.
3	82982779	GA	G	intergenic	*GBE1,LINC00971*	.	.	.
3	83230893	A	T	intergenic	*GBE1,LINC00971*	.	.	.
3	83317193	GACAC	G	intergenic	*GBE1,LINC00971*	.	.	.
3	83502693	G	C	intergenic	*GBE1,LINC00971*	.	.	.
3	83666457	G	T	intergenic	*GBE1,LINC00971*	.	.	.
3	83419531	A	G	intergenic	*GBE1,LINC00971*	.	.	.
3	83429966	G	T	intergenic	*GBE1,LINC00971*	.	.	.
3	83813673	A	G	intergenic	*GBE1,LINC00971*	.	.	.
3	82880377	T	C	intergenic	*GBE1,LINC00971*	.	.	.
3	82951568	C	T	intergenic	*GBE1,LINC00971*	.	.	.
3	82951570	T	G	intergenic	*GBE1,LINC00971*	.	.	.
3	83003378	T	C	intergenic	*GBE1,LINC00971*	.	.	.
3	83087476	G	A	intergenic	*GBE1,LINC00971*	.	.	.
3	83339059	A	T	intergenic	*GBE1,LINC00971*	.	.	.
3	83449969	C	T	intergenic	*GBE1,LINC00971*	.	.	.
3	83449970	T	C	intergenic	*GBE1,LINC00971*	.	.	.
3	83586571	G	A	intergenic	*GBE1,LINC00971*	.	.	.
3	83586572	C	G	intergenic	*GBE1,LINC00971*	.	.	.
3	83751149	T	A	intergenic	*GBE1,LINC00971*	.	.	.
3	83751151	C	G	intergenic	*GBE1,LINC00971*	.	.	.
3	83797297	A	G	intergenic	*GBE1,LINC00971*	.	.	.
3	81895923	C	G	intergenic	*GBE1,NONE*	.	.	.
3	81939908	T	C	intergenic	*GBE1,NONE*	.	.	.
3	82233343	A	G	intergenic	*GBE1,NONE*	.	.	.
3	82436886	A	AT	intergenic	*GBE1,NONE*	.	.	.
3	81988480	C	CT	intergenic	*GBE1,NONE*	.	.	.
3	82085078	G	T	intergenic	*GBE1,NONE*	.	.	.
3	82320441	C	T	intergenic	*GBE1,NONE*	.	.	.
3	82450743	G	A	intergenic	*GBE1,NONE*	.	.	.
3	82451478	C	A	intergenic	*GBE1,NONE*	.	.	.
3	82451480	T	C	intergenic	*GBE1,NONE*	.	.	.
10	104043129	A	G	intronic	*GBF1*	.	.	.
9	136066820	C	CTG	intergenic	*GBGT1,OBP2B*	.	.	.
1	89943153	T	A	intergenic	*GBP1P1,LRRC8B*	.	.	.
1	89943154	A	G	intergenic	*GBP1P1,LRRC8B*	.	.	.

1	89692104	C	T	intergenic	*GBP4,GBP5*	.	.	.
1	89693628	A	G	intergenic	*GBP4,GBP5*	.	.	.
1	89711600	C	T	intergenic	*GBP4,GBP5*	.	.	.
1	89711602	C	T	intergenic	*GBP4,GBP5*	.	.	.
1	89838566	T	C	intronic	*GBP6*	.	.	.
1	89854782	T	C	intergenic	*GBP6,GBP1P1*	.	.	.
1	89625036	A	T	intronic	*GBP7*	.	.	.
4	72741888	C	CCTTT	intergenic	*GC,NPFFR2*	.	.	.
4	72759020	G	A	intergenic	*GC,NPFFR2*	.	.	.
4	72760525	T	C	intergenic	*GC,NPFFR2*	.	.	.
4	72762848	A	C	intergenic	*GC,NPFFR2*	.	.	.
4	72814754	G	A	intergenic	*GC,NPFFR2*	.	.	.
4	72816452	T	C	intergenic	*GC,NPFFR2*	.	.	.
4	72818285	G	C	intergenic	*GC,NPFFR2*	.	.	.
4	72865126	T	C	intergenic	*GC,NPFFR2*	.	.	.
4	72867204	T	C	intergenic	*GC,NPFFR2*	.	.	.
2	75911032	T	G	intronic	*GCFC2*	.	.	.
2	76265365	C	T	intergenic	*GCFC2,LRRTM4*	.	.	.
2	76755047	C	CT	intergenic	*GCFC2,LRRTM4*	.	.	.
2	76143555	G	T	intergenic	*GCFC2,LRRTM4*	.	.	.
2	76485929	C	A	intergenic	*GCFC2,LRRTM4*	.	.	.
2	76781464	AT	A	intergenic	*GCFC2,LRRTM4*	.	.	.
2	76354567	T	A	intergenic	*GCFC2,LRRTM4*	.	.	.
2	76362800	T	C	intergenic	*GCFC2,LRRTM4*	.	.	.
2	76436423	T	TC	intergenic	*GCFC2,LRRTM4*	.	.	.
2	76452830	G	A	intergenic	*GCFC2,LRRTM4*	.	.	.
2	76452832	C	T	intergenic	*GCFC2,LRRTM4*	.	.	.
2	76575423	A	G	intergenic	*GCFC2,LRRTM4*	.	.	.
2	76669887	A	G	intergenic	*GCFC2,LRRTM4*	.	.	.
14	55317736	G	A	intronic	*GCH1*	.	.	.
14	55357110	GA	G	intronic	*GCH1*	.	.	.
6	53415940	G	A	intergenic	*GCLC,LOC101927136*	.	.	.
1	94379546	T	G	intergenic	*GCLM,ABCA4*	.	.	.
6	53092113	C	T	intergenic	*GCM1,ELOVL5*	.	.	.
15	58039700	C	A	intergenic	*GCOM1,ALDH1A2*	.	.	.
15	58104865	T	A	intergenic	*GCOM1,ALDH1A2*	.	.	.
15	58192499	G	A	intergenic	*GCOM1,ALDH1A2*	.	.	.

1	247716368	C	T	intronic	*GCSAML*	.	.	.
1	247734315	A	AGAGACTAG	intronic	*GCSAML*	.	.	.
9	74789354	T	C	intronic	*GDA*	.	.	.
8	75284940	T	A	intergenic	*GDAP1,MIR5681A*	.	.	.
8	75285423	C	T	intergenic	*GDAP1,MIR5681A*	.	.	.
8	75285424	A	G	intergenic	*GDAP1,MIR5681A*	.	.	.
8	75299150	T	G	intergenic	*GDAP1,MIR5681A*	.	.	.
8	75301967	G	A	intergenic	*GDAP1,MIR5681A*	.	.	.
10	48608985	G	A	intergenic	*GDF10,PTPN20*	.	.	.
10	48443969	T	C	intergenic	*GDF10,PTPN20*	.	.	.
10	48592130	CT	C	intergenic	*GDF10,PTPN20*	.	.	.
10	48522785	G	T	intergenic	*GDF10,PTPN20*	.	.	.
10	48528722	C	T	intergenic	*GDF10,PTPN20*	.	.	.
8	97212615	C	G	intergenic	*GDF6,UQCRB*	.	.	.
8	97203764	C	T	intergenic	*GDF6,UQCRB*	.	.	.
5	37984448	T	A	intergenic	*GDNF-AS1,EGFLAM*	.	.	.
5	38223086	G	A	intergenic	*GDNF-AS1,EGFLAM*	.	.	.
17	57302105	C	T	intronic	*GDPD1*	.	.	.
17	57346632	G	A	intronic	*GDPD1*	.	.	.
11	76929463	C	T	intronic	*GDPD4*	.	.	.
11	77007832	C	T	intergenic	*GDPD4,PAK1*	.	.	.
11	75261353	C	T	intergenic	*GDPD5,SERPINH1*	.	.	.
8	95342357	T	C	intergenic	*GEM,RAD54B*	.	.	.
8	95350116	A	G	intergenic	*GEM,RAD54B*	.	.	.
X	14210517	T	A	intergenic	*GEMIN8,UBE2E4P*	.	.	.
X	14210518	A	G	intergenic	*GEMIN8,UBE2E4P*	.	.	.
X	14219470	C	T	intergenic	*GEMIN8,UBE2E4P*	.	.	.
X	14222878	A	G	intergenic	*GEMIN8,UBE2E4P*	.	.	.
6	13428867	T	C	intronic	*GFOD1*	.	.	.
6	13444503	T	C	intronic	*GFOD1*	.	.	.
5	179876810	G	A	intergenic	*GFPT2,CNOT6*	.	.	.
8	21684448	G	A	intergenic	*GFRA2,DOK2*	.	.	.
8	21714865	T	C	intergenic	*GFRA2,DOK2*	.	.	.
6	55213035	AGTGT	A	intronic	*GFRAL*	.	.	.
6	55231234	T	C	intronic	*GFRAL*	.	.	.
13	101198643	T	A	intronic	*GGACT*	.	.	.

13	101226855	C	T	intronic	*GGACT*	.	.	.
7	30581120	C	G	intergenic	*GGCT,LOC401320*	.	.	.
7	30582259	A	T	intergenic	*GGCT,LOC401320*	.	.	.
2	85772170	T	A	UTR3	*GGCX,MAT2A*	.	.	.
8	63964495	T	G	intergenic	*GGH,TTPA*	.	.	.
22	25009609	C	T	intronic	*GGT1*	.	.	.
22	25009610	A	G	intronic	*GGT1*	.	.	.
22	18886397	C	G	intergenic	*GGT3P,DGCR6*	.	.	.
22	18848246	TTA	T	intergenic	*GGT3P,DGCR6*	.	.	.
22	24628577	T	G	intronic	*GGT5*	.	.	.
20	33431798	C	A	downstream	*GGT7*	.	.	.
2	92107617	T	TTG	intergenic	*GGT8P,ACTR3BP2*	.	.	.
2	92107618	CACAAAAAAAAAA	C	intergenic	*GGT8P,ACTR3BP2*	.	.	.
2	92085450	C	T	intergenic	*GGT8P,ACTR3BP2*	.	.	.
2	92104761	C	G	intergenic	*GGT8P,ACTR3BP2*	.	.	.
2	91971591	G	A	intergenic	*GGT8P,ACTR3BP2*	.	.	.
2	92096999	C	G	intergenic	*GGT8P,ACTR3BP2*	.	.	.
20	24050843	C	A	intergenic	*GGTLC1,FLJ33581*	.	.	.
20	24113884	G	C	intergenic	*GGTLC1,FLJ33581*	.	.	.
20	24004464	C	A	intergenic	*GGTLC1,FLJ33581*	.	.	.
17	61959231	G	T	UTR5	*GH2*	.	.	.
5	42562109	T	G	intronic	*GHR*	.	.	.
5	42479720	A	G	intronic	*GHR*	.	.	.
5	42480748	A	C	intronic	*GHR*	.	.	.
5	42663038	G	A	intronic	*GHR*	.	.	.
5	42663984	A	G	intronic	*GHR*	.	.	.
5	42664790	G	A	intronic	*GHR*	.	.	.
5	42664910	G	A	intronic	*GHR*	.	.	.

7	31020886	G	A	intergenic	*GHRHR,ADCYAP1R1*	.	.	.
7	31075213	G	A	intergenic	*GHRHR,ADCYAP1R1*	.	.	.
3	10339968	G	A	intergenic	*GHRLOS,SEC13*	.	.	.
17	17953513	A	G	intronic	*GID4*	.	.	.
7	100297560	T	G	intergenic	*GIGYF1,POP7*	.	.	.
2	233584961	GTA	G	intronic	*GIGYF2*	.	.	.
7	150457081	G	A	intergenic	*GIMAP1-GIMAP5,TMEM176B*	.	.	.
7	150472793	TATATATATGTATGTTATACATACACACATGTGTATGTATAAC	T	intergenic	*GIMAP1-GIMAP5,TMEM176B*	.	.	.
7	150272316	C	G	intergenic	*GIMAP4,GIMAP6*	.	.	.
7	150299380	T	C	intergenic	*GIMAP4,GIMAP6*	.	.	.
7	150299381	G	A	intergenic	*GIMAP4,GIMAP6*	.	.	.
7	150304158	G	A	intergenic	*GIMAP4,GIMAP6*	.	.	.
7	150304956	C	A	intergenic	*GIMAP4,GIMAP6*	.	.	.
7	150315784	T	C	intergenic	*GIMAP4,GIMAP6*	.	.	.
7	150348477	T	G	intergenic	*GIMAP6,GIMAP2*	.	.	.
7	150239134	T	C	intergenic	*GIMAP7,GIMAP4*	.	.	.
7	150240422	T	C	intergenic	*GIMAP7,GIMAP4*	.	.	.
7	150240423	G	T	intergenic	*GIMAP7,GIMAP4*	.	.	.
7	150242024	T	G	intergenic	*GIMAP7,GIMAP4*	.	.	.
7	150243112	T	C	intergenic	*GIMAP7,GIMAP4*	.	.	.
7	150243948	T	C	intergenic	*GIMAP7,GIMAP4*	.	.	.
7	150251670	G	A	intergenic	*GIMAP7,GIMAP4*	.	.	.
7	150187536	G	A	intergenic	*GIMAP8,GIMAP7*	.	.	.
7	150187719	A	G	intergenic	*GIMAP8,GIMAP7*	.	.	.
7	150189382	C	A	intergenic	*GIMAP8,GIMAP7*	.	.	.
4	107435503	A	G	intergenic	*GIMD1,DKK2*	.	.	.
4	107325282	A	C	intergenic	*GIMD1,DKK2*	.	.	.
4	107409502	C	T	intergenic	*GIMD1,DKK2*	.	.	.
4	107409504	T	C	intergenic	*GIMD1,DKK2*	.	.	.

4	107410437	A	G	intergenic	*GIMD1,DKK2*	.	.	.
4	107458047	C	A	intergenic	*GIMD1,DKK2*	.	.	.
4	107470835	T	A	intergenic	*GIMD1,DKK2*	.	.	.
4	107632953	G	A	intergenic	*GIMD1,DKK2*	.	.	.
4	107643163	A	T	intergenic	*GIMD1,DKK2*	.	.	.
4	107643464	C	T	intergenic	*GIMD1,DKK2*	.	.	.
4	107643465	A	G	intergenic	*GIMD1,DKK2*	.	.	.
4	107756386	G	T	intergenic	*GIMD1,DKK2*	.	.	.
4	107823628	T	C	intergenic	*GIMD1,DKK2*	.	.	.
20	25413227	C	T	intronic	*GINS1*	.	.	.
1	78548627	CACAT	C	intronic	*GIPC2*	.	.	.
1	78532306	A	T	intronic	*GIPC2*	.	.	.
1	78629057	T	A	intergenic	*GIPC2,MGC27382*	.	.	.
1	78653558	A	G	intergenic	*GIPC2,MGC27382*	.	.	.
1	78670952	T	C	intergenic	*GIPC2,MGC27382*	.	.	.
1	78673159	C	T	intergenic	*GIPC2,MGC27382*	.	.	.
6	122357937	G	A	intergenic	*GJA1,HSF2*	.	.	.
6	122048451	TACAC	T	intergenic	*GJA1,HSF2*	.	.	.
6	122377515	C	A	intergenic	*GJA1,HSF2*	.	.	.
6	122023733	A	G	intergenic	*GJA1,HSF2*	.	.	.
6	122135906	C	T	intergenic	*GJA1,HSF2*	.	.	.
6	121965281	T	A	intergenic	*GJA1,HSF2*	.	.	.
6	122028205	G	T	intergenic	*GJA1,HSF2*	.	.	.
6	122184847	G	T	intergenic	*GJA1,HSF2*	.	.	.
6	122184848	A	G	intergenic	*GJA1,HSF2*	.	.	.
6	122230172	A	G	intergenic	*GJA1,HSF2*	.	.	.
6	122549423	T	C	intergenic	*GJA1,HSF2*	.	.	.
6	122552051	A	G	intergenic	*GJA1,HSF2*	.	.	.
6	122552350	G	T	intergenic	*GJA1,HSF2*	.	.	.
6	122591645	C	A	intergenic	*GJA1,HSF2*	.	.	.
6	122646562	C	T	intergenic	*GJA1,HSF2*	.	.	.
6	122701634	A	C	intergenic	*GJA1,HSF2*	.	.	.
1	147336141	C	CAA	intergenic	*GJA5,GJA8*	.	.	.
1	147339138	T	C	intergenic	*GJA5,GJA8*	.	.	.
1	147339623	T	A	intergenic	*GJA5,GJA8*	.	.	.
13	20819425	C	T	intergenic	*GJB6,CRYL1*	.	.	.
13	20819426	A	G	intergenic	*GJB6,CRYL1*	.	.	.
6	87997879	G	A	intronic	*GJB7*	.	.	.

6	88012631	G	A	intronic	*GJB7*	.	.	.
6	88012633	G	A	intronic	*GJB7*	.	.	.
6	88013827	G	A	intronic	*GJB7*	.	.	.
6	88014325	T	C	intronic	*GJB7*	.	.	.
6	88015509	G	A	intronic	*GJB7*	.	.	.
6	88017174	C	A	intronic	*GJB7*	.	.	.
6	88033350	A	C	intronic	*GJB7,SMIM8*	.	.	.
X	30756947	G	A	intergenic	*GK,TAB3*	.	.	.
9	86371413	C	A	intronic	*GKAP1*	.	.	.
2	69205457	G	A	intronic	*GKN1*	.	.	.
7	8128114	T	C	UTR3	*GLCCI1*	.	.	.
7	8060479	A	T	intronic	*GLCCI1*	.	.	.
7	8035034	G	A	intronic	*GLCCI1*	.	.	.
7	8139443	T	C	intergenic	*GLCCI1,ICA1*	.	.	.
9	131300279	C	G	exonic	*GLE1*	synonymous_SNV	.	.
2	121700357	C	T	intronic	*GLI2*	.	.	.
2	121824452	A	G	intergenic	*GLI2,TFCP2L1*	.	.	.
2	121762171	C	G	intergenic	*GLI2,TFCP2L1*	.	.	.
7	42202535	A	G	intronic	*GLI3*	.	.	.
7	42289373	C	T	intergenic	*GLI3,LINC01448*	.	.	.
7	42448151	T	A	intergenic	*GLI3,LINC01448*	.	.	.
7	42631281	T	C	intergenic	*GLI3,LINC01448*	.	.	.
7	42400294	A	G	intergenic	*GLI3,LINC01448*	.	.	.
7	42409966	A	G	intergenic	*GLI3,LINC01448*	.	.	.
7	42531579	A	T	intergenic	*GLI3,LINC01448*	.	.	.
7	42404763	T	C	intergenic	*GLI3,LINC01448*	.	.	.
7	42408815	A	G	intergenic	*GLI3,LINC01448*	.	.	.
7	42569076	G	GA	intergenic	*GLI3,LINC01448*	.	.	.
7	42622921	G	A	intergenic	*GLI3,LINC01448*	.	.	.
7	42640569	G	A	intergenic	*GLI3,LINC01448*	.	.	.
12	75834487	G	A	intergenic	*GLIPR1L2,GLIPR1*	.	.	.
12	75865560	C	T	intergenic	*GLIPR1L2,GLIPR1*	.	.	.
1	53979780	C	T	intronic	*GLIS1*	.	.	.
9	3829165	A	T	intronic	*GLIS3*	.	.	.
9	4058668	A	G	intronic	*GLIS3*	.	.	.
9	4236204	A	AAAAAAAAG	intronic	*GLIS3*	.	.	.
9	3941423	T	A	intronic	*GLIS3*	.	.	.
9	4189045	G	A	intronic	*GLIS3*	.	.	.

9	4322622	G	A	intergenic	*GLIS3,SLC1A1*	.	.	.
9	4469740	C	G	intergenic	*GLIS3,SLC1A1*	.	.	.
1	92722735	A	G	intronic	*GLMN*	.	.	.
1	92733042	C	T	intronic	*GLMN*	.	.	.
1	92750182	T	TC	intronic	*GLMN*	.	.	.
17	9735996	G	A	intronic	*GLP2R*	.	.	.
5	151215665	T	C	intronic	*GLRA1*	.	.	.
5	151273254	T	C	intronic	*GLRA1*	.	.	.
X	14650101	C	A	intronic	*GLRA2*	.	.	.
X	14645150	A	G	intronic	*GLRA2*	.	.	.
X	14621285	A	T	intronic	*GLRA2*	.	.	.
X	14701584	A	G	intronic	*GLRA2*	.	.	.
X	14702844	C	T	intronic	*GLRA2*	.	.	.
X	14702845	C	G	intronic	*GLRA2*	.	.	.
X	14751914	C	T	intergenic	*GLRA2,FANCB*	.	.	.
X	14788446	T	C	intergenic	*GLRA2,FANCB*	.	.	.
X	14829951	G	A	intergenic	*GLRA2,FANCB*	.	.	.
4	175642179	TG	T	intronic	*GLRA3*	.	.	.
4	175642181	TG	T	intronic	*GLRA3*	.	.	.
X	102964710	GGTGT	G	intronic	*GLRA4*	.	.	.
4	158006850	T	C	intronic	*GLRB*	.	.	.
4	158079453	G	A	intronic	*GLRB*	.	.	.
4	158098961	C	T	intergenic	*GLRB,GRIA2*	.	.	.
10	131981416	T	G	intergenic	*GLRX3,MIR378C*	.	.	.
10	132195998	C	G	intergenic	*GLRX3,MIR378C*	.	.	.
10	132238621	T	C	intergenic	*GLRX3,MIR378C*	.	.	.
10	132487845	C	T	intergenic	*GLRX3,MIR378C*	.	.	.
10	132662824	C	T	intergenic	*GLRX3,MIR378C*	.	.	.
10	132202777	CAGAG	C	intergenic	*GLRX3,MIR378C*	.	.	.
10	132276946	C	T	intergenic	*GLRX3,MIR378C*	.	.	.
10	132317008	G	C	intergenic	*GLRX3,MIR378C*	.	.	.
2	191809634	C	T	intronic	*GLS*	.	.	.
2	191823621	A	G	intronic	*GLS*	.	.	.
12	129405595	T	C	intronic	*GLT1D1*	.	.	.
12	129411844	C	T	intronic	*GLT1D1*	.	.	.
12	129411846	T	C	intronic	*GLT1D1*	.	.	.
12	129448016	T	A	intronic	*GLT1D1*	.	.	.

12	129543429	T	C	intergenic	*GLT1D1,TMEM132D*	.	.	.
12	104432507	C	T	intronic	*GLT8D2*	.	.	.
19	48198629	T	C	exonic	*GLTSCR1*	synonymous_SNV	.	.
19	48164209	C	G	intronic	*GLTSCR1*	.	.	.
X	120473435	T	C	intergenic	*GLUD2,MIR3672*	.	.	.
X	120407747	T	A	intergenic	*GLUD2,MIR3672*	.	.	.
X	120408432	G	A	intergenic	*GLUD2,MIR3672*	.	.	.
11	58520000	G	C	intergenic	*GLYAT,GLYATL2*	.	.	.
11	58586280	T	C	intergenic	*GLYAT,GLYATL2*	.	.	.
11	58587893	T	C	intergenic	*GLYAT,GLYATL2*	.	.	.
11	58588176	T	C	intergenic	*GLYAT,GLYATL2*	.	.	.
11	58639462	A	G	intergenic	*GLYATL2,GLYATL1*	.	.	.
11	58642275	A	G	intergenic	*GLYATL2,GLYATL1*	.	.	.
11	58650367	A	G	intergenic	*GLYATL2,GLYATL1*	.	.	.
11	58651300	C	T	intergenic	*GLYATL2,GLYATL1*	.	.	.
11	58651301	A	G	intergenic	*GLYATL2,GLYATL1*	.	.	.
11	58651766	T	C	intergenic	*GLYATL2,GLYATL1*	.	.	.
12	55013541	C	G	intergenic	*GLYCAM1,LACRT*	.	.	.
12	55008241	CAGAG	C	intergenic	*GLYCAM1,LACRT*	.	.	.
3	52344232	A	G	intergenic	*GLYCTK,DNAH1*	.	.	.
1	181450202	A	T	intergenic	*GM140,CACNA1E*	.	.	.
1	181235906	A	G	intergenic	*GM140,CACNA1E*	.	.	.
1	181236081	G	A	intergenic	*GM140,CACNA1E*	.	.	.
1	181375595	C	T	intergenic	*GM140,CACNA1E*	.	.	.
6	1751371	G	A	intronic	*GMDS*	.	.	.
6	1753559	G	T	intronic	*GMDS*	.	.	.
6	1963268	G	A	intronic	*GMDS*	.	.	.
6	2050105	G	T	intronic	*GMDS*	.	.	.
6	2050224	A	C	intronic	*GMDS*	.	.	.
6	2301835	C	G	ncRNA_intronic	*GMDS-AS1*	.	.	.
19	19743044	G	A	intronic	*GMIP*	.	.	.

8	143933729	G	A	intergenic	*GML,CYP11B1*	.	.	.
8	143935383	C	A	intergenic	*GML,CYP11B1*	.	.	.
8	143935471	A	C	intergenic	*GML,CYP11B1*	.	.	.
8	143936208	A	G	intergenic	*GML,CYP11B1*	.	.	.
8	143936210	C	T	intergenic	*GML,CYP11B1*	.	.	.
6	16244704	C	T	intronic	*GMPR*	.	.	.
3	155780595	G	A	intergenic	*GMPS,KCNAB1*	.	.	.
3	155734896	CT	C	intergenic	*GMPS,KCNAB1*	.	.	.
7	2798931	A	G	intronic	*GNA12*	.	.	.
7	2785893	CTTG	C	intronic	*GNA12*	.	.	.
9	80253196	T	TG	intronic	*GNA14*	.	.	.
9	80300953	T	G	intergenic	*GNA14,GNAQ*	.	.	.
7	79780805	T	C	intronic	*GNAI1*	.	.	.
7	79824855	C	T	intronic	*GNAI1*	.	.	.
3	50295419	A	T	intronic	*GNAI2*	.	.	.
16	56321891	G	C	intronic	*GNAO1*	.	.	.
9	80596977	T	C	intronic	*GNAQ*	.	.	.
9	80512391	T	C	intronic	*GNAQ*	.	.	.
9	80585891	C	T	intronic	*GNAQ*	.	.	.
9	80811770	T	A	intergenic	*GNAQ,CEP78*	.	.	.
9	80693154	T	C	intergenic	*GNAQ,CEP78*	.	.	.
9	80693226	T	C	intergenic	*GNAQ,CEP78*	.	.	.
9	80694250	T	C	intergenic	*GNAQ,CEP78*	.	.	.
9	80702479	A	G	intergenic	*GNAQ,CEP78*	.	.	.
9	80726187	A	T	intergenic	*GNAQ,CEP78*	.	.	.
9	80753141	A	T	intergenic	*GNAQ,CEP78*	.	.	.
9	80820235	T	C	intergenic	*GNAQ,CEP78*	.	.	.
9	80820236	T	C	intergenic	*GNAQ,CEP78*	.	.	.
9	80824980	AG	A	intergenic	*GNAQ,CEP78*	.	.	.
20	57424633	C	G	ncRNA_intronic	*GNAS-AS1*	.	.	.
7	80127001	C	T	intronic	*GNAT3*	.	.	.
15	52433644	A	T	intronic	*GNB5*	.	.	.
1	68395650	C	A	ncRNA_intronic	*GNG12-AS1*	.	.	.
1	68472013	A	G	ncRNA_intronic	*GNG12-AS1*	.	.	.
19	2547339	C	A	intronic	*GNG7*	.	.	.
X	54569015	A	G	intronic	*GNL3L*	.	.	.
X	54597774	C	T	intergenic	*GNL3L,ITIH6*	.	.	.
X	54607069	C	T	intergenic	*GNL3L,ITIH6*	.	.	.

X	54702515	C	T	intergenic	*GNL3L,ITIH6*	.	.	.
X	54709568	G	A	intergenic	*GNL3L,ITIH6*	.	.	.
X	54726769	G	A	intergenic	*GNL3L,ITIH6*	.	.	.
X	54728239	G	A	intergenic	*GNL3L,ITIH6*	.	.	.
X	54745645	C	A	intergenic	*GNL3L,ITIH6*	.	.	.
X	54759273	G	C	intergenic	*GNL3L,ITIH6*	.	.	.
X	54759547	G	A	intergenic	*GNL3L,ITIH6*	.	.	.
X	54761867	C	T	intergenic	*GNL3L,ITIH6*	.	.	.
1	231412377	T	C	intronic	*GNPAT*	.	.	.
5	141422718	G	A	intergenic	*GNPDA1,NDFIP1*	.	.	.
4	45516303	G	T	intergenic	*GNPDA2,GABRG1*	.	.	.
4	45608890	G	T	intergenic	*GNPDA2,GABRG1*	.	.	.
4	45770879	T	A	intergenic	*GNPDA2,GABRG1*	.	.	.
4	44887330	C	A	intergenic	*GNPDA2,GABRG1*	.	.	.
4	44735064	T	C	intergenic	*GNPDA2,GABRG1*	.	.	.
4	45022704	G	T	intergenic	*GNPDA2,GABRG1*	.	.	.
4	45352709	A	G	intergenic	*GNPDA2,GABRG1*	.	.	.
4	45186586	A	T	intergenic	*GNPDA2,GABRG1*	.	.	.
4	45315572	A	G	intergenic	*GNPDA2,GABRG1*	.	.	.
4	45964502	C	A	intergenic	*GNPDA2,GABRG1*	.	.	.
4	44746675	T	C	intergenic	*GNPDA2,GABRG1*	.	.	.
4	44749633	T	G	intergenic	*GNPDA2,GABRG1*	.	.	.
4	44750483	A	C	intergenic	*GNPDA2,GABRG1*	.	.	.
4	44813363	C	T	intergenic	*GNPDA2,GABRG1*	.	.	.
4	44834083	C	A	intergenic	*GNPDA2,GABRG1*	.	.	.
4	44886899	G	A	intergenic	*GNPDA2,GABRG1*	.	.	.
4	45032714	A	G	intergenic	*GNPDA2,GABRG1*	.	.	.
4	45055852	G	A	intergenic	*GNPDA2,GABRG1*	.	.	.
4	45055854	C	G	intergenic	*GNPDA2,GABRG1*	.	.	.
4	45055973	C	A	intergenic	*GNPDA2,GABRG1*	.	.	.
4	45063455	A	T	intergenic	*GNPDA2,GABRG1*	.	.	.
4	45306542	G	A	intergenic	*GNPDA2,GABRG1*	.	.	.
4	45576354	T	A	intergenic	*GNPDA2,GABRG1*	.	.	.
4	45655833	T	C	intergenic	*GNPDA2,GABRG1*	.	.	.
4	45655834	G	A	intergenic	*GNPDA2,GABRG1*	.	.	.
4	45658219	G	C	intergenic	*GNPDA2,GABRG1*	.	.	.
4	45742428	T	C	intergenic	*GNPDA2,GABRG1*	.	.	.
4	45776075	G	C	intergenic	*GNPDA2,GABRG1*	.	.	.

4	45776076	G	T	intergenic	*GNPDA2,GABRG1*	.	.	.
4	45776694	C	T	intergenic	*GNPDA2,GABRG1*	.	.	.
4	45784324	G	A	intergenic	*GNPDA2,GABRG1*	.	.	.
4	45917734	G	A	intergenic	*GNPDA2,GABRG1*	.	.	.
4	45917736	T	A	intergenic	*GNPDA2,GABRG1*	.	.	.
4	45966235	G	A	intergenic	*GNPDA2,GABRG1*	.	.	.
4	45980156	T	C	intergenic	*GNPDA2,GABRG1*	.	.	.
4	45980157	G	A	intergenic	*GNPDA2,GABRG1*	.	.	.
4	46003999	A	T	intergenic	*GNPDA2,GABRG1*	.	.	.
4	68614444	A	AAAAT	intronic	*GNRHR*	.	.	.
12	65157700	A	G	intergenic	*GNS,TBC1D30*	.	.	.
9	127661687	C	T	exonic	*GOLGA1*	nonsynonymous_SNV	.	.
3	37343469	A	G	intronic	*GOLGA4*	.	.	.
14	93263096	A	ACC	intronic	*GOLGA5*	.	.	.
15	23478821	T	A	intronic	*GOLGA6L22*	.	.	.
15	23501900	G	GT	intronic	*GOLGA6L22*	.	.	.
15	23503635	T	C	intronic	*GOLGA6L22*	.	.	.
15	85802215	G	A	intergenic	*GOLGA6L3,MIR7706*	.	.	.
15	20756804	T	C	intergenic	*GOLGA6L6,GOLGA8CP*	.	.	.
15	34888931	GTTGTTGT	G	intergenic	*GOLGA8B,GJD2*	.	.	.
15	20854305	AAATAATAAT	A	intergenic	*GOLGA8CP,NBEAP1*	.	.	.
15	32884720	T	TTTAA	upstream	*GOLGA8N*	.	.	.
15	32897967	G	A	UTR3	*GOLGA8N*	.	.	.
15	32798567	A	T	intergenic	*GOLGA8O,WHAMMP1*	.	.	.
15	23613080	G	C	ncRNA_exonic	*GOLGA8S*	.	.	.
3	121393825	G	A	intronic	*GOLGB1*	.	.	.
9	88706535	A	G	intronic	*GOLM1*	.	.	.
9	88701106	A	G	intronic	*GOLM1*	.	.	.
9	88703232	C	G	intronic	*GOLM1*	.	.	.
9	88703234	G	T	intronic	*GOLM1*	.	.	.
6	117919928	A	G	intronic	*GOPC*	.	.	.
6	117968595	C	T	intergenic	*GOPC,LOC101927919*	.	.	.

6	117968596	G	C	intergenic	*GOPC,LOC101927919*	.	.	.
6	117944684	G	T	intergenic	*GOPC,LOC101927919*	.	.	.
1	170602150	A	G	intergenic	*GORAB,PRRX1*	.	.	.
1	170539889	G	T	intergenic	*GORAB,PRRX1*	.	.	.
1	170541193	T	A	intergenic	*GORAB,PRRX1*	.	.	.
1	170543498	A	T	intergenic	*GORAB,PRRX1*	.	.	.
1	170578136	A	G	intergenic	*GORAB,PRRX1*	.	.	.
17	44999491	T	C	upstream	*GOSR2*	.	.	.
17	45031635	T	C	intergenic	*GOSR2,MIR5089*	.	.	.
10	101170554	A	G	intronic	*GOT1*	.	.	.
10	101176852	C	A	intronic	*GOT1*	.	.	.
10	101253044	C	A	intergenic	*GOT1,LINC01475*	.	.	.
16	59249066	C	A	intergenic	*GOT2,APOOP5*	.	.	.
16	59342284	A	C	intergenic	*GOT2,APOOP5*	.	.	.
16	59307253	A	ACC	intergenic	*GOT2,APOOP5*	.	.	.
16	58875582	C	T	intergenic	*GOT2,APOOP5*	.	.	.
16	58909509	A	C	intergenic	*GOT2,APOOP5*	.	.	.
16	58910489	G	A	intergenic	*GOT2,APOOP5*	.	.	.
16	58949347	C	A	intergenic	*GOT2,APOOP5*	.	.	.
16	59035458	T	G	intergenic	*GOT2,APOOP5*	.	.	.
16	59758084	T	A	intergenic	*GOT2,APOOP5*	.	.	.
16	59758307	G	A	intergenic	*GOT2,APOOP5*	.	.	.
1	167030964	G	C	intronic	*GPA33*	.	.	.
1	167030965	G	C	intronic	*GPA33*	.	.	.
2	96762069	T	A	intergenic	*GPAT2,ADRA2B*	.	.	.
4	84842488	TATTTAATTTA	T	intergenic	*GPAT3,LOC101928978*	.	.	.
4	84741556	G	A	intergenic	*GPAT3,LOC101928978*	.	.	.
4	84657111	TCC	T	intergenic	*GPAT3,LOC101928978*	.	.	.
4	84874483	G	T	intergenic	*GPAT3,LOC101928978*	.	.	.
1	217723423	T	G	intronic	*GPATCH2*	.	.	.
1	217688410	C	A	intronic	*GPATCH2*	.	.	.
1	217724602	G	A	intronic	*GPATCH2*	.	.	.

1	217613085	G	A	intronic	*GPATCH2*	.	.	.
1	217613991	A	C	intronic	*GPATCH2*	.	.	.
1	217614170	A	C	intronic	*GPATCH2*	.	.	.
1	217705947	G	GAAATA	intronic	*GPATCH2*	.	.	.
1	217743015	C	T	intronic	*GPATCH2*	.	.	.
5	56495453	C	T	intronic	*GPBP1*	.	.	.
5	56722608	T	TATC	intergenic	*GPBP1,ACTBL2*	.	.	.
5	56684354	T	C	intergenic	*GPBP1,ACTBL2*	.	.	.
5	56651100	C	G	intergenic	*GPBP1,ACTBL2*	.	.	.
X	132827592	A	G	intronic	*GPC3*	.	.	.
X	133092411	A	T	intronic	*GPC3*	.	.	.
X	133055182	A	G	intronic	*GPC3*	.	.	.
X	132760402	A	AG	intronic	*GPC3*	.	.	.
X	132760404	A	C	intronic	*GPC3*	.	.	.
X	133280513	C	T	intergenic	*GPC3,MIR363*	.	.	.
X	133181107	C	T	intergenic	*GPC3,MIR363*	.	.	.
X	133181108	A	G	intergenic	*GPC3,MIR363*	.	.	.
X	133181406	C	T	intergenic	*GPC3,MIR363*	.	.	.
X	133187533	T	C	intergenic	*GPC3,MIR363*	.	.	.
X	133193354	C	T	intergenic	*GPC3,MIR363*	.	.	.
X	133216271	C	T	intergenic	*GPC3,MIR363*	.	.	.
X	133216273	C	T	intergenic	*GPC3,MIR363*	.	.	.
X	133216375	C	T	intergenic	*GPC3,MIR363*	.	.	.
X	133216376	A	G	intergenic	*GPC3,MIR363*	.	.	.
X	133218782	T	A	intergenic	*GPC3,MIR363*	.	.	.
X	133238018	T	C	intergenic	*GPC3,MIR363*	.	.	.
X	133256253	T	A	intergenic	*GPC3,MIR363*	.	.	.
X	132512159	C	T	intronic	*GPC4*	.	.	.
X	132509191	C	T	intronic	*GPC4*	.	.	.
13	92111386	C	A	intronic	*GPC5*	.	.	.
13	92280983	C	G	intronic	*GPC5*	.	.	.
13	92467367	C	A	intronic	*GPC5*	.	.	.
13	93263630	C	G	intronic	*GPC5*	.	.	.
13	92210957	C	T	intronic	*GPC5*	.	.	.
13	93031037	A	G	intronic	*GPC5*	.	.	.
13	93042156	G	T	intronic	*GPC5*	.	.	.
13	93314780	C	A	intronic	*GPC5*	.	.	.

13	92224049	GTATATATACACACACATACGTGTGTGTA	G	intronic	*GPC5*	.	.	.
13	92086415	G	C	intronic	*GPC5*	.	.	.
13	92086416	G	T	intronic	*GPC5*	.	.	.
13	92086496	A	T	intronic	*GPC5*	.	.	.
13	92086497	C	T	intronic	*GPC5*	.	.	.
13	92485053	T	C	intronic	*GPC5*	.	.	.
13	92486618	T	A	intronic	*GPC5*	.	.	.
13	92487194	T	A	intronic	*GPC5*	.	.	.
13	92503776	C	T	intronic	*GPC5*	.	.	.
13	92820864	C	G	intronic	*GPC5*	.	.	.
13	92886547	C	A	intronic	*GPC5*	.	.	.
13	92935155	C	T	intronic	*GPC5*	.	.	.
13	92937077	C	A	intronic	*GPC5*	.	.	.
13	92937079	C	T	intronic	*GPC5*	.	.	.
13	93049425	C	T	intronic	*GPC5*	.	.	.
13	93171061	T	G	intronic	*GPC5*	.	.	.
13	93172316	T	C	intronic	*GPC5*	.	.	.
13	93198690	T	A	intronic	*GPC5*	.	.	.
13	93282883	G	A	intronic	*GPC5*	.	.	.
13	93348919	A	T	intronic	*GPC5*	.	.	.
13	93349187	A	G	intronic	*GPC5*	.	.	.
13	93351936	T	G	intronic	*GPC5*	.	.	.
13	93351938	T	A	intronic	*GPC5*	.	.	.
13	93447558	C	T	intronic	*GPC5*	.	.	.
13	93447559	A	G	intronic	*GPC5*	.	.	.
13	94453813	C	G	intronic	*GPC6*	.	.	.
13	93918396	T	G	intronic	*GPC6*	.	.	.
13	94175268	ATAT	A	intronic	*GPC6*	.	.	.
13	94684722	A	G	intronic	*GPC6*	.	.	.
13	93966654	G	A	intronic	*GPC6*	.	.	.
13	94665472	GTATA	G	intronic	*GPC6*	.	.	.
13	94918405	C	CTTTTTTTTTT	intronic	*GPC6*	.	.	.
13	94333999	T	G	intronic	*GPC6*	.	.	.
13	94966337	G	A	intronic	*GPC6*	.	.	.
13	93899070	AG	A	intronic	*GPC6*	.	.	.

13	94093350	A	T	intronic	*GPC6*	.	.	.
13	94093634	C	A	intronic	*GPC6*	.	.	.
13	94423942	A	T	intronic	*GPC6*	.	.	.
13	94683364	ATGTG	A	intronic	*GPC6*	.	.	.
13	94756315	C	T	intronic	*GPC6*	.	.	.
13	94932084	C	T	intronic	*GPC6*	.	.	.
13	95027551	T	C	intronic	*GPC6*	.	.	.
13	95027552	T	G	intronic	*GPC6*	.	.	.
13	94487930	G	A	ncRNA_intronic	*GPC6-AS2*	.	.	.
20	5664742	C	G	intergenic	*GPCPD1,C20orf196*	.	.	.
3	32237761	A	G	intergenic	*GPD1L,CMTM8*	.	.	.
3	32242216	G	A	intergenic	*GPD1L,CMTM8*	.	.	.
2	157763989	C	A	intergenic	*GPD2,GALNT5*	.	.	.
2	157818872	A	T	intergenic	*GPD2,GALNT5*	.	.	.
2	157820859	G	T	intergenic	*GPD2,GALNT5*	.	.	.
2	157842557	C	T	intergenic	*GPD2,GALNT5*	.	.	.
2	157851316	C	G	intergenic	*GPD2,GALNT5*	.	.	.
2	157853586	T	C	intergenic	*GPD2,GALNT5*	.	.	.
2	157919060	A	G	intergenic	*GPD2,GALNT5*	.	.	.
2	157928202	G	A	intergenic	*GPD2,GALNT5*	.	.	.
2	157928478	T	C	intergenic	*GPD2,GALNT5*	.	.	.
2	157928995	G	C	intergenic	*GPD2,GALNT5*	.	.	.
2	157954171	A	T	intergenic	*GPD2,GALNT5*	.	.	.
2	157957594	G	A	intergenic	*GPD2,GALNT5*	.	.	.
2	157957595	A	C	intergenic	*GPD2,GALNT5*	.	.	.
14	67048821	A	G	intronic	*GPHN*	.	.	.
14	67338946	G	C	intronic	*GPHN*	.	.	.
14	67432240	T	A	intronic	*GPHN*	.	.	.
14	67482514	C	T	intronic	*GPHN*	.	.	.
14	67556548	T	C	intronic	*GPHN*	.	.	.
14	66993275	G	A	intronic	*GPHN*	.	.	.
14	67055551	T	C	intronic	*GPHN*	.	.	.
14	67132100	A	T	intronic	*GPHN*	.	.	.
14	67152533	C	T	intronic	*GPHN*	.	.	.
14	67179376	C	A	intronic	*GPHN*	.	.	.
14	67344196	T	A	intronic	*GPHN*	.	.	.
14	67520426	T	C	intronic	*GPHN*	.	.	.

14	67521570	T	C	intronic	*GPHN*	.	.	.
14	67521571	G	C	intronic	*GPHN*	.	.	.
14	67529821	A	G	intronic	*GPHN*	.	.	.
14	67532334	C	A	intronic	*GPHN*	.	.	.
14	67615270	A	T	intronic	*GPHN*	.	.	.
14	67642657	C	CTT	intronic	*GPHN*	.	.	.
19	34890714	G	A	intronic	*GPI*	.	.	.
X	48980932	G	T	upstream	*GPKOW*	.	.	.
X	48970129	T	C	downstream	*GPKOW*	.	.	.
4	176802440	G	A	intronic	*GPM6A*	.	.	.
4	176863690	C	G	intronic	*GPM6A*	.	.	.
4	176863692	G	A	intronic	*GPM6A*	.	.	.
4	176863979	A	T	intronic	*GPM6A*	.	.	.
4	176888207	ATATTAGC	A	intronic	*GPM6A*	.	.	.
X	13797452	A	G	intronic	*GPM6B*	.	.	.
X	13877176	T	A	intronic	*GPM6B*	.	.	.
X	13844666	TACATACAT ACATACAC	T	intronic	*GPM6B*	.	.	.
X	13990887	T	C	intergenic	*GPM6B,GEMIN8*	.	.	.
2	207098158	A	G	ncRNA_intronic	*GPR1-AS*	.	.	.
X	136256668	C	A	intergenic	*GPR101,ZIC3*	.	.	.
X	136363701	A	T	intergenic	*GPR101,ZIC3*	.	.	.
X	136207392	C	T	intergenic	*GPR101,ZIC3*	.	.	.
X	136214488	C	CTG	intergenic	*GPR101,ZIC3*	.	.	.
X	136216793	C	T	intergenic	*GPR101,ZIC3*	.	.	.
X	136216838	T	A	intergenic	*GPR101,ZIC3*	.	.	.
X	136436226	CGTGTGTG T	C	intergenic	*GPR101,ZIC3*	.	.	.
X	136521151	C	A	intergenic	*GPR101,ZIC3*	.	.	.
X	136593754	G	A	intergenic	*GPR101,ZIC3*	.	.	.
13	27614599	G	A	intergenic	*GPR12,USP12*	.	.	.
13	27351071	G	A	intergenic	*GPR12,USP12*	.	.	.
1	236353266	G	C	intronic	*GPR137B*	.	.	.
14	53037605	G	A	intronic	*GPR137C*	.	.	.
14	53042693	C	T	intronic	*GPR137C*	.	.	.
14	53060280	G	A	intronic	*GPR137C*	.	.	.
14	53061284	C	A	intronic	*GPR137C*	.	.	.

14	53061285	A	T	intronic	*GPR137C*	.	.	.
14	53105513	A	G	intergenic	*GPR137C,ERO1A*	.	.	.
7	37847036	A	T	intergenic	*GPR141,NME8*	.	.	.
7	37847782	T	C	intergenic	*GPR141,NME8*	.	.	.
7	37849355	T	G	intergenic	*GPR141,NME8*	.	.	.
7	37852758	C	T	intergenic	*GPR141,NME8*	.	.	.
7	37852759	C	T	intergenic	*GPR141,NME8*	.	.	.
7	37880844	G	T	intergenic	*GPR141,NME8*	.	.	.
2	131504091	A	G	intergenic	*GPR148,AMER3*	.	.	.
3	154240603	G	T	intergenic	*GPR149,MME*	.	.	.
3	154730681	G	T	intergenic	*GPR149,MME*	.	.	.
3	154783083	TCTCCCTTA	T	intergenic	*GPR149,MME*	.	.	.
2	175336166	T	A	intronic	*GPR155*	.	.	.
3	119965981	G	A	intergenic	*GPR156,LRRC58*	.	.	.
3	120018171	A	G	intergenic	*GPR156,LRRC58*	.	.	.
10	25846954	T	G	intronic	*GPR158*	.	.	.
10	25633084	C	A	intronic	*GPR158*	.	.	.
10	25859063	A	G	intronic	*GPR158*	.	.	.
10	25859064	G	T	intronic	*GPR158*	.	.	.
10	25674248	T	C	intronic	*GPR158*	.	.	.
10	25493410	C	T	intronic	*GPR158*	.	.	.
10	25493411	A	G	intronic	*GPR158*	.	.	.
10	25649140	T	C	intronic	*GPR158*	.	.	.
10	25660972	G	A	intronic	*GPR158*	.	.	.
10	25762352	T	G	intronic	*GPR158*	.	.	.
10	25900441	G	C	intergenic	*GPR158,LINC00836*	.	.	.
X	78427214	G	T	exonic	*GPR174*	nonsynonymous_SNV	.	.
X	78508013	C	T	intergenic	*GPR174,ITM2A*	.	.	.
X	78576073	AT	A	intergenic	*GPR174,ITM2A*	.	.	.
X	78462208	T	C	intergenic	*GPR174,ITM2A*	.	.	.
15	40131205	C	T	intronic	*GPR176*	.	.	.
13	95290210	A	G	intergenic	*GPR180,LOC101927248*	.	.	.
14	31961277	AGGTTGCCATGAGCC	A	intergenic	*GPR33,NUBPL*	.	.	.

14	31967108	T	C	intergenic	*GPR33,NUBPL*	.	.	.
14	31976827	C	T	intergenic	*GPR33,NUBPL*	.	.	.
2	133334692	G	A	intronic	*GPR39*	.	.	.
2	133228415	C	A	intronic	*GPR39*	.	.	.
2	133213859	T	C	intronic	*GPR39*	.	.	.
2	133289543	A	G	intronic	*GPR39*	.	.	.
2	133291617	T	A	intronic	*GPR39*	.	.	.
2	133294386	G	A	intronic	*GPR39*	.	.	.
X	150491317	T	C	intergenic	*GPR50,VMA21*	.	.	.
X	150370716	G	A	intergenic	*GPR50,VMA21*	.	.	.
6	110323398	T	A	intergenic	*GPR6,WASF1*	.	.	.
6	110310365	T	A	intergenic	*GPR6,WASF1*	.	.	.
6	110311219	T	C	intergenic	*GPR6,WASF1*	.	.	.
6	97267185	T	C	intronic	*GPR63*	.	.	.
6	97267187	T	C	intronic	*GPR63*	.	.	.
2	54069698	T	A	intronic	*GPR75-ASB3*	.	.	.
11	94122070	T	C	intronic	*GPR83*	.	.	.
11	94122743	T	C	intronic	*GPR83*	.	.	.
11	94124272	T	G	intronic	*GPR83*	.	.	.
11	94124274	G	A	intronic	*GPR83*	.	.	.
11	94124990	T	C	intronic	*GPR83*	.	.	.
11	94124991	T	A	intronic	*GPR83*	.	.	.
7	112729987	C	G	intergenic	*GPR85,LINC00998*	.	.	.
7	112729988	G	C	intergenic	*GPR85,LINC00998*	.	.	.
1	145774305	G	C	intronic	*GPR89A,NBPF10,NBPF20*	.	.	.
1	145784043	A	C	intronic	*GPR89A,NBPF10,NBPF20*	.	.	.
16	20037828	TG	T	intergenic	*GPRC5B,GPR139*	.	.	.
16	19966682	G	A	intergenic	*GPRC5B,GPR139*	.	.	.
6	117135139	G	T	intronic	*GPRC6A*	.	.	.
10	46994390	T	C	intronic	*GPRIN2*	.	.	.
10	47040358	G	A	intergenic	*GPRIN2,NPY4R*	.	.	.
10	47016617	A	T	intergenic	*GPRIN2,NPY4R*	.	.	.
4	90480663	A	T	intergenic	*GPRIN3,SNCA*	.	.	.
4	90311369	G	A	intergenic	*GPRIN3,SNCA*	.	.	.
4	90400535	C	A	intergenic	*GPRIN3,SNCA*	.	.	.
4	90401663	A	C	intergenic	*GPRIN3,SNCA*	.	.	.

4	90598243	T	G	intergenic	*GPRIN3,SNCA*	.	.	.
6	28515180	T	C	intergenic	*GPX5,ZBED9*	.	.	.
6	28478884	G	T	intronic	*GPX6*	.	.	.
11	123463532	G	A	intronic	*GRAMD1B*	.	.	.
5	125780739	G	A	intronic	*GRAMD3*	.	.	.
22	47048578	G	A	intronic	*GRAMD4*	.	.	.
7	50813351	T	C	intronic	*GRB10*	.	.	.
7	51035063	T	C	intergenic	*GRB10,COBL*	.	.	.
7	50946035	T	C	intergenic	*GRB10,COBL*	.	.	.
7	50946037	G	T	intergenic	*GRB10,COBL*	.	.	.
7	50946522	A	T	intergenic	*GRB10,COBL*	.	.	.
7	50958997	C	G	intergenic	*GRB10,COBL*	.	.	.
7	50958998	A	G	intergenic	*GRB10,COBL*	.	.	.
2	165444442	G	T	intronic	*GRB14*	.	.	.
2	165485281	G	A	intergenic	*GRB14,COBLL1*	.	.	.
17	37901310	AG	A	intronic	*GRB7*	.	.	.
2	11776930	A	G	intronic	*GREB1*	.	.	.
18	19041113	A	ATCGG	intronic	*GREB1L*	.	.	.
15	33020089	G	T	intronic	*GREM1*	.	.	.
8	102530993	C	T	intronic	*GRHL2*	.	.	.
8	102686616	C	A	intergenic	*GRHL2,NCALD*	.	.	.
8	102684653	A	G	intergenic	*GRHL2,NCALD*	.	.	.
8	102690780	G	A	intergenic	*GRHL2,NCALD*	.	.	.
5	153138555	T	C	intronic	*GRIA1*	.	.	.
5	152881011	G	A	intronic	*GRIA1*	.	.	.
5	153048099	A	C	intronic	*GRIA1*	.	.	.
5	153061653	A	T	intronic	*GRIA1*	.	.	.
5	153293239	G	A	intergenic	*GRIA1,FAM114A2*	.	.	.
5	153293241	T	C	intergenic	*GRIA1,FAM114A2*	.	.	.
5	153322547	T	C	intergenic	*GRIA1,FAM114A2*	.	.	.
5	153322548	G	A	intergenic	*GRIA1,FAM114A2*	.	.	.
4	158177393	T	TTAC	intronic	*GRIA2*	.	.	.
4	158418275	C	A	intergenic	*GRIA2,LOC340017*	.	.	.
X	122428493	G	A	intronic	*GRIA3*	.	.	.
X	122419584	T	A	intronic	*GRIA3*	.	.	.
X	122507886	G	A	intronic	*GRIA3*	.	.	.
X	122700019	T	C	intergenic	*GRIA3,THOC2*	.	.	.

X	122709554	T	C	intergenic	*GRIA3,THOC2*	.	.	.
X	122711459	C	T	intergenic	*GRIA3,THOC2*	.	.	.
X	122711460	A	G	intergenic	*GRIA3,THOC2*	.	.	.
X	122712072	C	A	intergenic	*GRIA3,THOC2*	.	.	.
X	122713428	A	T	intergenic	*GRIA3,THOC2*	.	.	.
11	105771679	AATAT	A	intronic	*GRIA4*	.	.	.
11	105825656	T	C	intronic	*GRIA4*	.	.	.
11	105825773	C	A	intronic	*GRIA4*	.	.	.
11	105518515	T	A	intronic	*GRIA4*	.	.	.
11	105671149	G	C	intronic	*GRIA4*	.	.	.
11	105527024	T	C	intronic	*GRIA4*	.	.	.
11	105596773	C	T	intronic	*GRIA4*	.	.	.
11	105599951	A	C	intronic	*GRIA4*	.	.	.
11	105599952	C	T	intronic	*GRIA4*	.	.	.
11	105744348	C	T	intronic	*GRIA4*	.	.	.
11	105744349	A	G	intronic	*GRIA4*	.	.	.
11	105744749	C	T	intronic	*GRIA4*	.	.	.
10	87403189	C	A	intronic	*GRID1*	.	.	.
10	88019415	CTGTG	C	intronic	*GRID1*	.	.	.
10	87464410	T	C	intronic	*GRID1*	.	.	.
10	87531562	T	C	intronic	*GRID1*	.	.	.
10	87582179	C	A	intronic	*GRID1*	.	.	.
10	87639023	T	C	intronic	*GRID1*	.	.	.
10	87639024	G	A	intronic	*GRID1*	.	.	.
10	87640704	A	G	intronic	*GRID1*	.	.	.
10	87934398	A	G	intronic	*GRID1*	.	.	.
10	88034063	G	T	intronic	*GRID1*	.	.	.
10	88137047	T	C	intergenic	*GRID1,WAPL*	.	.	.
4	94044297	T	G	intronic	*GRID2*	.	.	.
4	94490879	T	C	intronic	*GRID2*	.	.	.
4	93235310	T	C	intronic	*GRID2*	.	.	.
4	93378874	T	TA	intronic	*GRID2*	.	.	.
4	93226736	G	C	intronic	*GRID2*	.	.	.
4	93226737	C	T	intronic	*GRID2*	.	.	.
4	93578101	C	A	intronic	*GRID2*	.	.	.
4	93407619	T	G	intronic	*GRID2*	.	.	.
4	93830325	A	G	intronic	*GRID2*	.	.	.
4	94423858	TCC	T	intronic	*GRID2*	.	.	.

4	93396280	AAATAATA AT	A	intronic	*GRID2*	.	.	.
4	93563548	A	G	intronic	*GRID2*	.	.	.
4	93958981	T	G	intronic	*GRID2*	.	.	.
4	93959436	A	T	intronic	*GRID2*	.	.	.
4	94026351	C	G	intronic	*GRID2*	.	.	.
4	94026555	A	T	intronic	*GRID2*	.	.	.
4	94243944	G	C	intronic	*GRID2*	.	.	.
4	94245272	G	A	intronic	*GRID2*	.	.	.
4	94478442	C	A	intronic	*GRID2*	.	.	.
4	94480425	T	G	intronic	*GRID2*	.	.	.
4	94600922	T	C	intronic	*GRID2*	.	.	.
4	94602851	G	A	intronic	*GRID2*	.	.	.
4	94674608	T	TA	intronic	*GRID2*	.	.	.
4	94674610	T	C	intronic	*GRID2*	.	.	.
7	6542245	A	C	intronic	*GRID2IP*	.	.	.
7	6539354	G	A	intronic	*GRID2IP*	.	.	.
21	31238188	A	T	intronic	*GRIK1*	.	.	.
21	31189358	A	T	intronic	*GRIK1*	.	.	.
21	31273425	G	A	intronic	*GRIK1*	.	.	.
21	31465901	A	G	intergenic	*GRIK1,CLDN17*	.	.	.
6	102501674	G	A	intronic	*GRIK2*	.	.	.
6	102387908	GC	G	intronic	*GRIK2*	.	.	.
6	101995536	C	T	intronic	*GRIK2*	.	.	.
6	101996198	G	T	intronic	*GRIK2*	.	.	.
6	102027546	C	T	intronic	*GRIK2*	.	.	.
6	102237048	A	G	intronic	*GRIK2*	.	.	.
6	102239386	T	A	intronic	*GRIK2*	.	.	.
6	102240131	A	T	intronic	*GRIK2*	.	.	.
6	102461448	T	TCA	intronic	*GRIK2*	.	.	.
6	103306113	GTGTA	G	intergenic	*GRIK2,HACE1*	.	.	.
6	103497504	T	C	intergenic	*GRIK2,HACE1*	.	.	.
6	103945522	T	C	intergenic	*GRIK2,HACE1*	.	.	.
6	104338676	G	T	intergenic	*GRIK2,HACE1*	.	.	.
6	103359039	G	T	intergenic	*GRIK2,HACE1*	.	.	.
6	103479280	G	A	intergenic	*GRIK2,HACE1*	.	.	.
6	104176970	C	T	intergenic	*GRIK2,HACE1*	.	.	.
6	103531782	C	A	intergenic	*GRIK2,HACE1*	.	.	.

6	103582227	G	A	intergenic	*GRIK2,HACE1*	.	.	.
6	103275886	A	G	intergenic	*GRIK2,HACE1*	.	.	.
6	104289766	C	CTA	intergenic	*GRIK2,HACE1*	.	.	.
6	104582441	G	T	intergenic	*GRIK2,HACE1*	.	.	.
6	103110062	C	A	intergenic	*GRIK2,HACE1*	.	.	.
6	103101423	T	C	intergenic	*GRIK2,HACE1*	.	.	.
6	103175856	T	G	intergenic	*GRIK2,HACE1*	.	.	.
6	103252374	G	A	intergenic	*GRIK2,HACE1*	.	.	.
6	103253432	T	C	intergenic	*GRIK2,HACE1*	.	.	.
6	103253433	A	T	intergenic	*GRIK2,HACE1*	.	.	.
6	103254015	G	A	intergenic	*GRIK2,HACE1*	.	.	.
6	103363838	A	G	intergenic	*GRIK2,HACE1*	.	.	.
6	103455114	A	G	intergenic	*GRIK2,HACE1*	.	.	.
6	103455175	G	A	intergenic	*GRIK2,HACE1*	.	.	.
6	103463214	G	A	intergenic	*GRIK2,HACE1*	.	.	.
6	103671726	C	T	intergenic	*GRIK2,HACE1*	.	.	.
6	103672437	A	G	intergenic	*GRIK2,HACE1*	.	.	.
6	103696466	A	G	intergenic	*GRIK2,HACE1*	.	.	.
6	103699915	C	T	intergenic	*GRIK2,HACE1*	.	.	.
6	103763859	T	C	intergenic	*GRIK2,HACE1*	.	.	.
6	103819642	T	C	intergenic	*GRIK2,HACE1*	.	.	.
6	103824814	G	A	intergenic	*GRIK2,HACE1*	.	.	.
6	103824815	C	G	intergenic	*GRIK2,HACE1*	.	.	.
6	103908590	T	C	intergenic	*GRIK2,HACE1*	.	.	.
6	103909309	T	C	intergenic	*GRIK2,HACE1*	.	.	.
6	103909497	G	A	intergenic	*GRIK2,HACE1*	.	.	.
6	103971952	T	C	intergenic	*GRIK2,HACE1*	.	.	.
6	103975398	T	C	intergenic	*GRIK2,HACE1*	.	.	.
6	103975399	G	C	intergenic	*GRIK2,HACE1*	.	.	.
6	103976905	G	T	intergenic	*GRIK2,HACE1*	.	.	.
6	104029352	A	C	intergenic	*GRIK2,HACE1*	.	.	.
6	104093109	C	T	intergenic	*GRIK2,HACE1*	.	.	.
6	104093110	A	G	intergenic	*GRIK2,HACE1*	.	.	.
6	104165707	T	A	intergenic	*GRIK2,HACE1*	.	.	.
6	104242073	G	A	intergenic	*GRIK2,HACE1*	.	.	.
6	104254429	G	C	intergenic	*GRIK2,HACE1*	.	.	.
6	104254559	C	T	intergenic	*GRIK2,HACE1*	.	.	.
6	104254850	C	A	intergenic	*GRIK2,HACE1*	.	.	.

6	104255190	C	T	intergenic	*GRIK2,HACE1*	.	.	.
6	104255467	C	A	intergenic	*GRIK2,HACE1*	.	.	.
6	104464911	G	A	intergenic	*GRIK2,HACE1*	.	.	.
6	104552709	T	C	intergenic	*GRIK2,HACE1*	.	.	.
6	102649707	C	T	intergenic	*GRIK2,NONE*	.	.	.
6	103033678	T	C	intergenic	*GRIK2,NONE*	.	.	.
6	102701472	C	G	intergenic	*GRIK2,NONE*	.	.	.
6	102623408	T	A	intergenic	*GRIK2,NONE*	.	.	.
6	102623409	C	A	intergenic	*GRIK2,NONE*	.	.	.
6	102987491	G	A	intergenic	*GRIK2,NONE*	.	.	.
6	102620511	A	G	intergenic	*GRIK2,NONE*	.	.	.
6	102620772	C	T	intergenic	*GRIK2,NONE*	.	.	.
6	102622631	A	G	intergenic	*GRIK2,NONE*	.	.	.
6	102695254	T	C	intergenic	*GRIK2,NONE*	.	.	.
6	102725187	C	A	intergenic	*GRIK2,NONE*	.	.	.
6	102733304	T	C	intergenic	*GRIK2,NONE*	.	.	.
6	102984952	A	G	intergenic	*GRIK2,NONE*	.	.	.
11	120864836	T	A	intergenic	*GRIK4,TBCEL*	.	.	.
19	42577711	G	A	intergenic	*GRIK5,ZNF574*	.	.	.
9	140055610	G	A	exonic	*GRIN1*	nonsynonymous_SNV	.	.
16	9978938	T	C	intronic	*GRIN2A*	.	.	.
16	9852240	G	T	UTR3	*GRIN2A*	.	.	.
12	13883725	T	A	intronic	*GRIN2B*	.	.	.
12	13746622	G	A	intronic	*GRIN2B*	.	.	.
12	13746793	T	C	intronic	*GRIN2B*	.	.	.
12	13863923	C	T	intronic	*GRIN2B*	.	.	.
12	13864182	C	A	intronic	*GRIN2B*	.	.	.
12	14207048	A	G	intergenic	*GRIN2B,ATF7IP*	.	.	.
12	14156069	A	G	intergenic	*GRIN2B,ATF7IP*	.	.	.
12	14191406	G	A	intergenic	*GRIN2B,ATF7IP*	.	.	.
12	14191784	T	C	intergenic	*GRIN2B,ATF7IP*	.	.	.
12	14243889	A	G	intergenic	*GRIN2B,ATF7IP*	.	.	.
12	14497169	G	A	intergenic	*GRIN2B,ATF7IP*	.	.	.
12	14497170	T	A	intergenic	*GRIN2B,ATF7IP*	.	.	.
9	104377088	A	G	intronic	*GRIN3A*	.	.	.
9	104380455	A	C	intronic	*GRIN3A*	.	.	.

9	104651014	G	T	intergenic	*GRIN3A,LINC00587*	.	.	.
9	105094811	C	A	intergenic	*GRIN3A,LINC00587*	.	.	.
9	104599468	A	G	intergenic	*GRIN3A,LINC00587*	.	.	.
9	104614865	G	T	intergenic	*GRIN3A,LINC00587*	.	.	.
9	104701063	C	T	intergenic	*GRIN3A,LINC00587*	.	.	.
9	104701064	C	T	intergenic	*GRIN3A,LINC00587*	.	.	.
9	104824800	C	A	intergenic	*GRIN3A,LINC00587*	.	.	.
9	104824801	A	G	intergenic	*GRIN3A,LINC00587*	.	.	.
9	104891360	C	T	intergenic	*GRIN3A,LINC00587*	.	.	.
9	104892443	G	C	intergenic	*GRIN3A,LINC00587*	.	.	.
9	104946631	T	TC	intergenic	*GRIN3A,LINC00587*	.	.	.
9	105145335	G	T	intergenic	*GRIN3A,LINC00587*	.	.	.
9	105168639	A	G	intergenic	*GRIN3A,LINC00587*	.	.	.
12	66905545	C	A	intronic	*GRIP1*	.	.	.
12	66869860	T	G	intronic	*GRIP1*	.	.	.
12	66871967	G	A	intronic	*GRIP1*	.	.	.
12	66872993	A	G	intronic	*GRIP1*	.	.	.
12	66918238	C	T	intronic	*GRIP1*	.	.	.
12	66918472	C	A	intronic	*GRIP1*	.	.	.
12	66918679	T	C	intronic	*GRIP1*	.	.	.
12	67235231	C	T	intergenic	*GRIP1,LOC102724421*	.	.	.
12	67427051	GTATATA	G	intergenic	*GRIP1,LOC102724421*	.	.	.

12	67250000	G	T	intergenic	*GRIP1,LOC102724421*	.	.	.
3	14576192	G	T	intronic	*GRIP2*	.	.	.
4	2999914	G	A	intronic	*GRK4*	.	.	.
4	3058772	T	TC	intergenic	*GRK4,HTT-AS*	.	.	.
6	146511716	G	C	intronic	*GRM1*	.	.	.
6	146655018	A	G	intronic	*GRM1*	.	.	.
6	146629157	A	T	intronic	*GRM1*	.	.	.
6	146405083	T	C	intronic	*GRM1*	.	.	.
6	146442935	A	G	intronic	*GRM1*	.	.	.
6	146641203	C	T	intronic	*GRM1*	.	.	.
6	146641205	A	T	intronic	*GRM1*	.	.	.
6	146735695	C	T	intronic	*GRM1*	.	.	.
6	146832301	T	C	intergenic	*GRM1,RAB32*	.	.	.
7	86415960	C	T	exonic	*GRM3*	synonymous_SNV	.	.
7	86416872	CAGAG	C	intronic	*GRM3*	.	.	.
7	86409413	T	C	intronic	*GRM3*	.	.	.
7	86425652	A	T	intronic	*GRM3*	.	.	.
7	86426072	T	TC	intronic	*GRM3*	.	.	.
7	86426074	T	TTGAA	intronic	*GRM3*	.	.	.
6	34200071	G	T	intergenic	*GRM4,HMGA1*	.	.	.
11	88505686	T	C	intronic	*GRM5*	.	.	.
11	88660753	C	G	intronic	*GRM5*	.	.	.
11	88413624	C	T	intronic	*GRM5*	.	.	.
11	88413744	C	T	intronic	*GRM5*	.	.	.
11	88460953	A	G	intronic	*GRM5*	.	.	.
11	88509893	C	T	intronic	*GRM5*	.	.	.
11	88537812	G	A	intronic	*GRM5*	.	.	.
11	88600213	A	G	intronic	*GRM5*	.	.	.
11	88732147	A	C	intronic	*GRM5*	.	.	.
11	88899946	A	G	intergenic	*GRM5,TYR*	.	.	.
11	88809967	C	G	intergenic	*GRM5,TYR*	.	.	.
11	88810389	C	T	intergenic	*GRM5,TYR*	.	.	.
11	88851221	C	T	intergenic	*GRM5,TYR*	.	.	.
11	88879017	A	ATTTTAAT	intergenic	*GRM5,TYR*	.	.	.
3	6922936	G	T	intronic	*GRM7*	.	.	.
3	7591555	G	T	intronic	*GRM7*	.	.	.

3	7400907	TTGTGTGT GTGTGTG	T	intronic	*GRM7*	.	.	.
3	6743296	C	A	ncRNA_intronic	*GRM7-AS3*	.	.	.
3	6786879	C	A	ncRNA_intronic	*GRM7-AS3*	.	.	.
3	6685876	C	T	ncRNA_intronic	*GRM7-AS3*	.	.	.
3	6859952	G	A	intergenic	*GRM7-AS3,GRM7*	.	.	.
7	126090692	A	T	intronic	*GRM8*	.	.	.
7	126162961	T	C	intronic	*GRM8*	.	.	.
7	126222631	A	G	intronic	*GRM8*	.	.	.
7	126452444	C	T	intronic	*GRM8*	.	.	.
7	126622107	T	C	intronic	*GRM8*	.	.	.
7	126503526	T	G	intronic	*GRM8*	.	.	.
7	126607532	C	G	intronic	*GRM8*	.	.	.
7	126370343	A	T	intronic	*GRM8*	.	.	.
7	126225753	T	C	intronic	*GRM8*	.	.	.
7	126226800	A	T	intronic	*GRM8*	.	.	.
7	126257827	T	C	intronic	*GRM8*	.	.	.
7	126271491	C	T	intronic	*GRM8*	.	.	.
7	126297864	G	A	intronic	*GRM8*	.	.	.
7	126466867	A	T	intronic	*GRM8*	.	.	.
7	126467610	T	A	intronic	*GRM8*	.	.	.
7	126468774	C	T	intronic	*GRM8*	.	.	.
7	126667004	C	G	intronic	*GRM8*	.	.	.
7	126667289	C	T	intronic	*GRM8*	.	.	.
7	126667981	A	C	intronic	*GRM8*	.	.	.
7	126667982	C	T	intronic	*GRM8*	.	.	.
7	126773574	C	T	intronic	*GRM8*	.	.	.
7	126918297	G	C	intergenic	*GRM8,ZNF800*	.	.	.
7	126997900	CAGAG	C	intergenic	*GRM8,ZNF800*	.	.	.
18	56926646	G	T	intergenic	*GRP,RAX*	.	.	.
4	71753534	G	A	intergenic	*GRSF1,MOB1B*	.	.	.
4	43018650	TTGTGTG	T	intronic	*GRXCR1*	.	.	.
4	42930580	G	A	intronic	*GRXCR1*	.	.	.
4	42930581	A	T	intronic	*GRXCR1*	.	.	.
4	42998096	C	A	intronic	*GRXCR1*	.	.	.
4	42984125	A	G	intronic	*GRXCR1*	.	.	.
4	43234965	G	T	intergenic	*GRXCR1,LVCAT1*	.	.	.
4	43538189	A	C	intergenic	*GRXCR1,LVCAT1*	.	.	.

4	43266833	T	TACAC	intergenic	*GRXCR1,LVCAT1*	.	.	.
4	43378638	C	T	intergenic	*GRXCR1,LVCAT1*	.	.	.
4	43306553	T	C	intergenic	*GRXCR1,LVCAT1*	.	.	.
4	43439205	G	C	intergenic	*GRXCR1,LVCAT1*	.	.	.
4	43794151	T	C	intergenic	*GRXCR1,LVCAT1*	.	.	.
4	43871900	C	A	intergenic	*GRXCR1,LVCAT1*	.	.	.
4	43399175	T	G	intergenic	*GRXCR1,LVCAT1*	.	.	.
4	43628069	G	T	intergenic	*GRXCR1,LVCAT1*	.	.	.
4	43736652	G	T	intergenic	*GRXCR1,LVCAT1*	.	.	.
4	43097028	C	T	intergenic	*GRXCR1,LVCAT1*	.	.	.
4	43142960	C	T	intergenic	*GRXCR1,LVCAT1*	.	.	.
4	43143840	T	C	intergenic	*GRXCR1,LVCAT1*	.	.	.
4	43152716	T	C	intergenic	*GRXCR1,LVCAT1*	.	.	.
4	43154377	A	T	intergenic	*GRXCR1,LVCAT1*	.	.	.
4	43154826	C	T	intergenic	*GRXCR1,LVCAT1*	.	.	.
4	43174284	T	G	intergenic	*GRXCR1,LVCAT1*	.	.	.
4	43378432	C	A	intergenic	*GRXCR1,LVCAT1*	.	.	.
4	43529912	G	A	intergenic	*GRXCR1,LVCAT1*	.	.	.
4	43578572	G	A	intergenic	*GRXCR1,LVCAT1*	.	.	.
4	43616414	A	G	intergenic	*GRXCR1,LVCAT1*	.	.	.
4	43658688	G	A	intergenic	*GRXCR1,LVCAT1*	.	.	.
4	43726550	G	A	intergenic	*GRXCR1,LVCAT1*	.	.	.
4	43745354	T	C	intergenic	*GRXCR1,LVCAT1*	.	.	.
4	43746194	G	T	intergenic	*GRXCR1,LVCAT1*	.	.	.
4	43814561	T	G	intergenic	*GRXCR1,LVCAT1*	.	.	.
4	43860243	G	T	intergenic	*GRXCR1,LVCAT1*	.	.	.
4	43861087	G	T	intergenic	*GRXCR1,LVCAT1*	.	.	.
4	43861088	A	G	intergenic	*GRXCR1,LVCAT1*	.	.	.
4	43881784	G	A	intergenic	*GRXCR1,LVCAT1*	.	.	.
4	43894321	T	C	intergenic	*GRXCR1,LVCAT1*	.	.	.
4	43894630	C	T	intergenic	*GRXCR1,LVCAT1*	.	.	.
4	43916897	C	A	intergenic	*GRXCR1,LVCAT1*	.	.	.
5	145260915	A	G	intergenic	*GRXCR2,SH3RF2*	.	.	.
5	145306560	G	A	intergenic	*GRXCR2,SH3RF2*	.	.	.
5	145308117	T	C	intergenic	*GRXCR2,SH3RF2*	.	.	.
5	145308118	G	A	intergenic	*GRXCR2,SH3RF2*	.	.	.
7	66053440	T	C	ncRNA_intronic	*GS1-124K5.11*	.	.	.

8	6704346	A	G	intergenic	*GS1-24F4.2,DEFB1*	.	.	.
1	185351133	G	A	intergenic	*GS1-279B7.1,LINC01350*	.	.	.
1	185481443	A	T	intergenic	*GS1-279B7.1,LINC01350*	.	.	.
1	185481445	G	A	intergenic	*GS1-279B7.1,LINC01350*	.	.	.
1	185486569	C	T	intergenic	*GS1-279B7.1,LINC01350*	.	.	.
14	95290093	G	T	intergenic	*GSC,DICER1*	.	.	.
12	13261182	T	TTTC	intergenic	*GSG1,EMP1*	.	.	.
3	119827651	A	G	intergenic	*GSK3B,GPR156*	.	.	.
16	12002433	C	T	intronic	*GSPT1*	.	.	.
16	12006098	G	A	intronic	*GSPT1*	.	.	.
16	12028346	G	C	intergenic	*GSPT1,TNFRSF17*	.	.	.
16	12051305	C	T	intergenic	*GSPT1,TNFRSF17*	.	.	.
X	51512288	G	T	intergenic	*GSPT2,MAGED1*	.	.	.
X	51521264	G	C	intergenic	*GSPT2,MAGED1*	.	.	.
6	52685989	T	C	intergenic	*GSTA1,GSTA5*	.	.	.
6	52637788	T	C	intergenic	*GSTA2,GSTA1*	.	.	.
6	52708271	A	G	intronic	*GSTA5*	.	.	.
4	106654724	G	C	intronic	*GSTCD*	.	.	.
4	106692433	A	G	intronic	*GSTCD*	.	.	.
4	106694615	T	C	intronic	*GSTCD*	.	.	.
4	106694616	G	A	intronic	*GSTCD*	.	.	.
4	106712473	C	T	intronic	*GSTCD*	.	.	.
4	106785729	C	T	intergenic	*GSTCD,NPNT*	.	.	.
4	106785730	A	C	intergenic	*GSTCD,NPNT*	.	.	.
4	106785682	C	A	intergenic	*GSTCD,NPNT*	.	.	.
4	106806358	C	T	intergenic	*GSTCD,NPNT*	.	.	.
2	144864133	C	T	intronic	*GTDC1*	.	.	.
2	144864966	A	G	intronic	*GTDC1*	.	.	.
2	144867155	T	C	intronic	*GTDC1*	.	.	.

2	144867710	G	A	intronic	*GTDC1*	.	.	.
2	145095577	T	TA	intergenic	*GTDC1,ZEB2*	.	.	.
2	145095578	G	T	intergenic	*GTDC1,ZEB2*	.	.	.
1	89352609	ATTT	A	intronic	*GTF2B*	.	.	.
8	30482777	C	T	intronic	*GTF2E2*	.	.	.
5	70350149	G	A	intronic	*GTF2H2*	.	.	.
5	68869023	A	T	intronic	*GTF2H2C,GTF2H2C_2*	.	.	.
6	158596553	C	T	intronic	*GTF2H5*	.	.	.
6	158596554	A	G	intronic	*GTF2H5*	.	.	.
6	158600258	T	C	intronic	*GTF2H5*	.	.	.
6	158600421	G	A	intronic	*GTF2H5*	.	.	.
6	158601821	T	A	intronic	*GTF2H5*	.	.	.
6	158623800	G	A	intergenic	*GTF2H5,TULP4*	.	.	.
1	224150582	G	A	ncRNA_intronic	*GTF2IP20*	.	.	.
1	224161803	C	G	ncRNA_intronic	*GTF2IP20*	.	.	.
1	224150005	TA	T	ncRNA_intronic	*GTF2IP20*	.	.	.
1	224150599	T	C	ncRNA_intronic	*GTF2IP20*	.	.	.
1	224177112	A	C	ncRNA_intronic	*GTF2IP20*	.	.	.
1	224177114	T	C	ncRNA_intronic	*GTF2IP20*	.	.	.
1	224273263	A	G	intergenic	*GTF2IP20,FBXO28*	.	.	.
7	73870751	C	T	intronic	*GTF2IRD1*	.	.	.
7	74058564	G	A	intergenic	*GTF2IRD1,GTF2I*	.	.	.
7	74294975	T	C	intergenic	*GTF2IRD2,STAG3L2*	.	.	.
9	135576703	A	G	intergenic	*GTF3C4,AK8*	.	.	.
7	89989628	A	G	intronic	*GTPBP10*	.	.	.
7	89989630	G	A	intronic	*GTPBP10*	.	.	.
7	89990389	T	G	intronic	*GTPBP10*	.	.	.
7	90018889	C	T	UTR3	*GTPBP10*	.	.	.
18	68966900	T	C	intergenic	*GTSCR1,LINC01541*	.	.	.
18	68556832	C	G	intergenic	*GTSCR1,LINC01541*	.	.	.
18	69157286	T	C	intergenic	*GTSCR1,LINC01541*	.	.	.

18	68355571	TC	T	intergenic	*GTSCR1,LINC01541*	.	.	.
18	68355573	TC	T	intergenic	*GTSCR1,LINC01541*	.	.	.
18	68364576	T	A	intergenic	*GTSCR1,LINC01541*	.	.	.
18	68364577	G	T	intergenic	*GTSCR1,LINC01541*	.	.	.
18	68384877	T	G	intergenic	*GTSCR1,LINC01541*	.	.	.
18	68386415	C	A	intergenic	*GTSCR1,LINC01541*	.	.	.
18	68408058	C	A	intergenic	*GTSCR1,LINC01541*	.	.	.
18	68436111	T	G	intergenic	*GTSCR1,LINC01541*	.	.	.
18	68436113	A	G	intergenic	*GTSCR1,LINC01541*	.	.	.
18	68524492	T	C	intergenic	*GTSCR1,LINC01541*	.	.	.
18	68656623	A	T	intergenic	*GTSCR1,LINC01541*	.	.	.
18	68761004	T	C	intergenic	*GTSCR1,LINC01541*	.	.	.
20	42445556	T	C	intergenic	*GTSF1L,TOX2*	.	.	.
20	42391791	T	G	intergenic	*GTSF1L,TOX2*	.	.	.
20	42503507	T	C	intergenic	*GTSF1L,TOX2*	.	.	.
20	42503611	G	A	intergenic	*GTSF1L,TOX2*	.	.	.
6	42147118	G	A	exonic	*GUCA1A*	nonsynonymous_SNV	.	.
6	42167662	C	G	intergenic	*GUCA1B,MRPS10*	.	.	.
11	106804634	T	C	intronic	*GUCY1A2*	.	.	.
11	106597381	A	G	intronic	*GUCY1A2*	.	.	.
11	106609354	A	G	intronic	*GUCY1A2*	.	.	.
11	106609690	A	G	intronic	*GUCY1A2*	.	.	.
11	106623913	A	G	intronic	*GUCY1A2*	.	.	.
11	106625218	C	T	intronic	*GUCY1A2*	.	.	.
11	106625893	A	T	intronic	*GUCY1A2*	.	.	.

11	106625894	C	T	intronic	*GUCY1A2*	.	.	.
11	106668309	T	C	intronic	*GUCY1A2*	.	.	.
11	106709853	G	T	intronic	*GUCY1A2*	.	.	.
11	106821992	G	A	intronic	*GUCY1A2*	.	.	.
11	106821994	T	G	intronic	*GUCY1A2*	.	.	.
11	107002519	C	T	intergenic	*GUCY1A2,CWF19L2*	.	.	.
11	106968922	G	T	intergenic	*GUCY1A2,CWF19L2*	.	.	.
11	106979327	T	C	intergenic	*GUCY1A2,CWF19L2*	.	.	.
11	107013916	GACAC	G	intergenic	*GUCY1A2,CWF19L2*	.	.	.
11	107039999	G	GGTT	intergenic	*GUCY1A2,CWF19L2*	.	.	.
11	107040419	C	T	intergenic	*GUCY1A2,CWF19L2*	.	.	.
11	107040420	A	T	intergenic	*GUCY1A2,CWF19L2*	.	.	.
11	107065924	T	C	intergenic	*GUCY1A2,CWF19L2*	.	.	.
11	107066569	T	C	intergenic	*GUCY1A2,CWF19L2*	.	.	.
11	107152505	C	T	intergenic	*GUCY1A2,CWF19L2*	.	.	.
11	107152507	C	T	intergenic	*GUCY1A2,CWF19L2*	.	.	.
11	107167552	G	A	intergenic	*GUCY1A2,CWF19L2*	.	.	.
11	107169042	G	A	intergenic	*GUCY1A2,CWF19L2*	.	.	.
13	51648228	C	T	intergenic	*GUCY1B2,LINC00371*	.	.	.
12	14785201	G	A	intronic	*GUCY2C*	.	.	.
X	108689996	A	T	intronic	*GUCY2F*	.	.	.
X	108689997	C	G	intronic	*GUCY2F*	.	.	.
X	108690972	A	G	intronic	*GUCY2F*	.	.	.
2	189337882	TTCTC	T	intronic	*GULP1*	.	.	.

2	189431447	C	T	intronic	*GULP1*	.	.	.
2	189525896	A	G	intergenic	*GULP1,DIRC1*	.	.	.
2	189546949	T	C	intergenic	*GULP1,DIRC1*	.	.	.
2	189552344	T	A	intergenic	*GULP1,DIRC1*	.	.	.
7	65458702	T	C	intergenic	*GUSB,ASL*	.	.	.
7	65459097	T	G	intergenic	*GUSB,ASL*	.	.	.
5	21539482	ACAC	A	ncRNA_intronic	*GUSBP1*	.	.	.
5	21478330	C	T	ncRNA_intronic	*GUSBP1*	.	.	.
5	21568924	A	C	ncRNA_intronic	*GUSBP1*	.	.	.
5	21562471	A	G	ncRNA_intronic	*GUSBP1*	.	.	.
5	21599937	CGTGT	C	intergenic	*GUSBP1,CDH12*	.	.	.
5	21625458	G	A	intergenic	*GUSBP1,CDH12*	.	.	.
5	21696431	A	G	intergenic	*GUSBP1,CDH12*	.	.	.
5	21708436	G	A	intergenic	*GUSBP1,CDH12*	.	.	.
7	57447923	G	A	intergenic	*GUSBP10,MIR3147*	.	.	.
7	57354982	G	A	intergenic	*GUSBP10,MIR3147*	.	.	.
22	24008041	T	C	ncRNA_intronic	*GUSBP11*	.	.	.
22	24008263	G	C	ncRNA_intronic	*GUSBP11*	.	.	.
6	26914071	C	A	ncRNA_intronic	*GUSBP2*	.	.	.
6	26914441	C	T	ncRNA_intronic	*GUSBP2*	.	.	.
5	69612514	ATATATATATATGCATATG	A	ncRNA_intronic	*GUSBP3*	.	.	.
5	69826290	G	C	ncRNA_intronic	*GUSBP3,GUSBP9,SMA5*	.	.	.
5	69118383	A	AT	intergenic	*GUSBP3,SERF1A*	.	.	.
5	70518590	C	CAAA	ncRNA_intronic	*GUSBP9*	.	.	.
11	6753446	T	C	intergenic	*GVINP1,OR2AG2*	.	.	.
11	6782710	G	A	intergenic	*GVINP1,OR2AG2*	.	.	.
X	2775572	TA	T	intronic	*GYG2*	.	.	.
X	2775574	TGTATCTAGATCTAGATCTCTCTCTATATAGATCTA	T	intronic	*GYG2*	.	.	.
4	145070498	C	A	intergenic	*GYPA,HHIP-AS1*	.	.	.

4	145072467	A	T	intergenic	*GYPA,HHIP-AS1*	.	.	.
4	145077548	T	C	intergenic	*GYPA,HHIP-AS1*	.	.	.
4	145077550	A	G	intergenic	*GYPA,HHIP-AS1*	.	.	.
4	145120523	T	A	intergenic	*GYPA,HHIP-AS1*	.	.	.
4	145358604	A	G	intergenic	*GYPA,HHIP-AS1*	.	.	.
4	145405817	T	G	intergenic	*GYPA,HHIP-AS1*	.	.	.
4	145409001	T	C	intergenic	*GYPA,HHIP-AS1*	.	.	.
4	145447514	G	A	intergenic	*GYPA,HHIP-AS1*	.	.	.
4	145472271	G	A	intergenic	*GYPA,HHIP-AS1*	.	.	.
4	144968286	A	C	intergenic	*GYPB,GYPA*	.	.	.
2	127496481	G	A	intergenic	*GYPC,BIN1*	.	.	.
2	127550049	G	A	intergenic	*GYPC,BIN1*	.	.	.
2	127803808	C	T	intergenic	*GYPC,BIN1*	.	.	.
12	21689663	C	G	UTR3	*GYS2*	.	.	.
12	21725745	A	T	intronic	*GYS2*	.	.	.
12	21777577	C	T	intergenic	*GYS2,LDHB*	.	.	.
14	25272808	C	CAAATAAAT	intergenic	*GZMB,STXBP6*	.	.	.
14	25117624	G	C	intergenic	*GZMB,STXBP6*	.	.	.
14	25135994	C	T	intergenic	*GZMB,STXBP6*	.	.	.
14	25219977	C	T	intergenic	*GZMB,STXBP6*	.	.	.
14	25219978	A	G	intergenic	*GZMB,STXBP6*	.	.	.
14	25233394	G	C	intergenic	*GZMB,STXBP6*	.	.	.
14	25239224	G	A	intergenic	*GZMB,STXBP6*	.	.	.
14	25251727	G	A	intergenic	*GZMB,STXBP6*	.	.	.
14	25088950	A	G	intergenic	*GZMH,GZMB*	.	.	.
5	54372300	T	G	intergenic	*GZMK,GZMA*	.	.	.
5	54395355	A	G	intergenic	*GZMK,GZMA*	.	.	.
5	54395393	A	G	intergenic	*GZMK,GZMA*	.	.	.
5	54395487	T	C	intergenic	*GZMK,GZMA*	.	.	.
11	2068805	T	C	intergenic	*H19,IGF2*	.	.	.
5	134699245	T	C	intronic	*H2AFY*	.	.	.
5	134777115	CGTGT	C	intergenic	*H2AFY,DCANP1*	.	.	.
X	103278175	C	A	intergenic	*H2BFWT,H2BFM*	.	.	.
X	103278177	G	A	intergenic	*H2BFWT,H2BFM*	.	.	.
X	103244070	G	A	intergenic	*H2BFXP,H2BFWT*	.	.	.
X	103322564	C	T	intergenic	*H2BFXP,SLC25A53*	.	.	.

X	103326733	G	A	intergenic	*H2BFXP,SLC25A53*	.	.	.
2	43001319	C	A	intronic	*HAAO*	.	.	.
2	43131921	A	T	intergenic	*HAAO,LOC102723854*	.	.	.
10	115341953	C	A	intronic	*HABP2*	.	.	.
10	17671004	C	G	intergenic	*HACD1,STAM-AS1*	.	.	.
6	105202784	C	A	intronic	*HACE1*	.	.	.
6	105203500	T	C	intronic	*HACE1*	.	.	.
6	105209558	A	C	intronic	*HACE1*	.	.	.
6	105209601	T	C	intronic	*HACE1*	.	.	.
6	105252498	G	A	intronic	*HACE1*	.	.	.
6	105252724	C	T	intronic	*HACE1*	.	.	.
6	105252725	A	T	intronic	*HACE1*	.	.	.
6	105253342	G	A	intronic	*HACE1*	.	.	.
6	105253373	G	T	intronic	*HACE1*	.	.	.
6	105322747	T	A	intergenic	*HACE1,LINC00577*	.	.	.
4	108931151	C	T	intronic	*HADH*	.	.	.
2	26441906	T	A	intronic	*HADHA*	.	.	.
12	96365714	C	T	downstream	*HAL*	.	.	.
5	153868029	G	A	intergenic	*HAND1,MIR3141*	.	.	.
4	174631309	G	T	intergenic	*HAND2-AS1,LOC101928509*	.	.	.
4	174953304	C	A	intergenic	*HAND2-AS1,LOC101928509*	.	.	.
4	174662520	G	A	intergenic	*HAND2-AS1,LOC101928509*	.	.	.
4	174711688	G	A	intergenic	*HAND2-AS1,LOC101928509*	.	.	.
4	174712238	C	T	intergenic	*HAND2-AS1,LOC101928509*	.	.	.

4	174808364	A	T	intergenic	*HAND2-AS1,LOC101928509*	.	.	.
4	174885113	G	A	intergenic	*HAND2-AS1,LOC101928509*	.	.	.
20	7900075	GACAC	G	intronic	*HAO1*	.	.	.
20	7934882	G	A	intergenic	*HAO1,TMX4*	.	.	.
1	119943267	A	G	intergenic	*HAO2,HSD3B2*	.	.	.
1	119945274	T	C	intergenic	*HAO2,HSD3B2*	.	.	.
1	119950470	T	C	intergenic	*HAO2,HSD3B2*	.	.	.
5	83113744	T	A	intergenic	*HAPLN1,EDIL3*	.	.	.
5	83053204	G	A	intergenic	*HAPLN1,EDIL3*	.	.	.
5	83054064	A	C	intergenic	*HAPLN1,EDIL3*	.	.	.
5	83090644	T	C	intergenic	*HAPLN1,EDIL3*	.	.	.
5	83092325	T	C	intergenic	*HAPLN1,EDIL3*	.	.	.
5	83116099	T	C	intergenic	*HAPLN1,EDIL3*	.	.	.
5	83116100	G	A	intergenic	*HAPLN1,EDIL3*	.	.	.
5	83146481	G	C	intergenic	*HAPLN1,EDIL3*	.	.	.
19	52221913	C	G	intronic	*HAS1*	.	.	.
19	52239549	C	A	intergenic	*HAS1,FPR1*	.	.	.
8	123375256	G	T	intergenic	*HAS2-AS1,LOC105375734*	.	.	.
8	122924705	G	A	intergenic	*HAS2-AS1,LOC105375734*	.	.	.
8	122992960	T	C	intergenic	*HAS2-AS1,LOC105375734*	.	.	.
8	122992961	G	A	intergenic	*HAS2-AS1,LOC105375734*	.	.	.
8	123017967	T	C	intergenic	*HAS2-AS1,LOC105375734*	.	.	.

8	123019106	T	C	intergenic	*HAS2-AS1,LOC105375734*	.	.	.
8	123213962	C	A	intergenic	*HAS2-AS1,LOC105375734*	.	.	.
8	123213963	A	T	intergenic	*HAS2-AS1,LOC105375734*	.	.	.
8	123288003	G	C	intergenic	*HAS2-AS1,LOC105375734*	.	.	.
8	123288991	A	C	intergenic	*HAS2-AS1,LOC105375734*	.	.	.
2	172791605	T	C	intronic	*HAT1*	.	.	.
2	172778251	T	C	upstream	*HAT1*	.	.	.
2	172784247	G	A	intronic	*HAT1*	.	.	.
18	43700427	C	T	intronic	*HAUS1*	.	.	.
18	43741395	T	G	intergenic	*HAUS1,C18orf25*	.	.	.
9	19081636	T	C	intronic	*HAUS6*	.	.	.
9	19097197	G	T	intronic	*HAUS6*	.	.	.
19	24501159	C	A	intergenic	*HAVCR1P1,NONE*	.	.	.
19	24569253	G	T	intergenic	*HAVCR1P1,NONE*	.	.	.
19	24574159	G	A	intergenic	*HAVCR1P1,NONE*	.	.	.
19	24428669	C	T	intergenic	*HAVCR1P1,NONE*	.	.	.
19	24391379	A	T	intergenic	*HAVCR1P1,NONE*	.	.	.
19	24619718	T	C	intergenic	*HAVCR1P1,NONE*	.	.	.
19	24382331	T	C	intergenic	*HAVCR1P1,NONE*	.	.	.
19	24417159	C	A	intergenic	*HAVCR1P1,NONE*	.	.	.
19	24417973	G	C	intergenic	*HAVCR1P1,NONE*	.	.	.
19	24447656	T	C	intergenic	*HAVCR1P1,NONE*	.	.	.
19	24448592	C	T	intergenic	*HAVCR1P1,NONE*	.	.	.
19	24449099	C	A	intergenic	*HAVCR1P1,NONE*	.	.	.
19	24449100	A	G	intergenic	*HAVCR1P1,NONE*	.	.	.
19	24465651	T	C	intergenic	*HAVCR1P1,NONE*	.	.	.
19	24466389	C	A	intergenic	*HAVCR1P1,NONE*	.	.	.
19	24466390	A	G	intergenic	*HAVCR1P1,NONE*	.	.	.

19	24572423	G	C	intergenic	*HAVCR1P1,NONE*	.	.	.
19	24580543	G	C	intergenic	*HAVCR1P1,NONE*	.	.	.
19	24581665	G	A	intergenic	*HAVCR1P1,NONE*	.	.	.
19	24589629	C	A	intergenic	*HAVCR1P1,NONE*	.	.	.
19	24590151	T	C	intergenic	*HAVCR1P1,NONE*	.	.	.
19	24590152	G	A	intergenic	*HAVCR1P1,NONE*	.	.	.
19	24595421	C	A	intergenic	*HAVCR1P1,NONE*	.	.	.
19	24596434	T	G	intergenic	*HAVCR1P1,NONE*	.	.	.
1	154284003	G	A	intergenic	*HAX1,AQP10*	.	.	.
11	5264546	G	C	ncRNA_exonic	*HBBP1*	.	.	.
6	135474237	A	G	intergenic	*HBS1L,MYB*	.	.	.
6	135474592	C	T	intergenic	*HBS1L,MYB*	.	.	.
X	11153806	G	C	intergenic	*HCCS,ARHGAP6*	.	.	.
X	153236918	G	A	upstream	*HCFC1*	.	.	.
6	26526732	T	C	ncRNA_exonic	*HCG11*	.	.	.
6	26534470	G	T	intergenic	*HCG11,HMGN4*	.	.	.
6	30220393	C	A	ncRNA_intronic	*HCG17*	.	.	.
6	30226165	G	A	ncRNA_intronic	*HCG17*	.	.	.
6	31192472	A	T	intergenic	*HCG27,HLA-C*	.	.	.
5	45472191	T	C	intronic	*HCN1*	.	.	.
5	45627724	C	T	intronic	*HCN1*	.	.	.
5	45640598	A	AT	intronic	*HCN1*	.	.	.
5	45442137	T	C	intronic	*HCN1*	.	.	.
5	45317054	A	G	intronic	*HCN1*	.	.	.
5	45350376	T	C	intronic	*HCN1*	.	.	.
5	45379126	A	T	intronic	*HCN1*	.	.	.
5	45496675	C	T	intronic	*HCN1*	.	.	.
5	45505392	A	G	intronic	*HCN1*	.	.	.
5	45575622	G	A	intronic	*HCN1*	.	.	.
5	45584437	C	G	intronic	*HCN1*	.	.	.
5	45584478	G	T	intronic	*HCN1*	.	.	.
5	45996258	G	A	intergenic	*HCN1,NONE*	.	.	.
5	46075655	G	A	intergenic	*HCN1,NONE*	.	.	.
5	45876056	T	A	intergenic	*HCN1,NONE*	.	.	.
5	45745091	G	A	intergenic	*HCN1,NONE*	.	.	.
5	45746646	C	G	intergenic	*HCN1,NONE*	.	.	.
5	45792057	C	A	intergenic	*HCN1,NONE*	.	.	.
5	45833283	G	T	intergenic	*HCN1,NONE*	.	.	.

5	45834550	G	A	intergenic	*HCN1,NONE*	.	.	.
5	45876245	C	T	intergenic	*HCN1,NONE*	.	.	.
5	45881660	G	C	intergenic	*HCN1,NONE*	.	.	.
5	45883596	A	G	intergenic	*HCN1,NONE*	.	.	.
5	45922243	C	T	intergenic	*HCN1,NONE*	.	.	.
5	45923457	A	G	intergenic	*HCN1,NONE*	.	.	.
5	45925575	T	C	intergenic	*HCN1,NONE*	.	.	.
5	45954163	T	C	intergenic	*HCN1,NONE*	.	.	.
5	46107562	G	A	intergenic	*HCN1,NONE*	.	.	.
5	46108393	T	C	intergenic	*HCN1,NONE*	.	.	.
5	46156756	G	A	intergenic	*HCN1,NONE*	.	.	.
5	46157111	C	T	intergenic	*HCN1,NONE*	.	.	.
5	46171365	G	C	intergenic	*HCN1,NONE*	.	.	.
5	46171366	A	T	intergenic	*HCN1,NONE*	.	.	.
5	46195780	G	A	intergenic	*HCN1,NONE*	.	.	.
5	46270578	G	A	intergenic	*HCN1,NONE*	.	.	.
5	46315419	C	A	intergenic	*HCN1,NONE*	.	.	.
5	46327011	G	C	intergenic	*HCN1,NONE*	.	.	.
5	46342299	C	T	intergenic	*HCN1,NONE*	.	.	.
5	46342300	C	G	intergenic	*HCN1,NONE*	.	.	.
5	46353068	G	GA	intergenic	*HCN1,NONE*	.	.	.
5	46374329	G	A	intergenic	*HCN1,NONE*	.	.	.
5	46377181	A	T	intergenic	*HCN1,NONE*	.	.	.
5	46397163	G	C	intergenic	*HCN1,NONE*	.	.	.
19	596545	C	T	intronic	*HCN2*	.	.	.
6	55127563	AATATAT	A	intronic	*HCRTR2*	.	.	.
6	55156113	A	G	intergenic	*HCRTR2,GFRAL*	.	.	.
6	55177233	T	C	intergenic	*HCRTR2,GFRAL*	.	.	.
3	13562345	C	T	intergenic	*HDAC11,FBLN2*	.	.	.
2	240128090	GCACA	G	intronic	*HDAC4*	.	.	.
2	239997444	T	C	intronic	*HDAC4*	.	.	.
2	240640460	T	C	intergenic	*HDAC4,LOC150935*	.	.	.
17	42180592	C	T	intronic	*HDAC5*	.	.	.
17	42172118	GTGTGTA	G	intronic	*HDAC5*	.	.	.
12	48193951	C	G	intronic	*HDAC7*	.	.	.
X	71680568	A	G	intronic	*HDAC8*	.	.	.
X	71739079	T	C	intronic	*HDAC8*	.	.	.

X	71665528	T	C	intronic	*HDAC8*	.	.	.
X	71720587	A	C	intronic	*HDAC8*	.	.	.
7	18143074	T	C	intronic	*HDAC9*	.	.	.
7	18163891	A	G	intronic	*HDAC9*	.	.	.
7	18236770	G	A	intronic	*HDAC9*	.	.	.
7	18483827	T	C	intronic	*HDAC9*	.	.	.
7	18907534	C	T	intronic	*HDAC9*	.	.	.
7	18213644	C	G	intronic	*HDAC9*	.	.	.
7	18227767	G	A	intronic	*HDAC9*	.	.	.
7	18228216	C	T	intronic	*HDAC9*	.	.	.
7	18455867	C	T	intronic	*HDAC9*	.	.	.
7	18777888	G	A	intronic	*HDAC9*	.	.	.
7	19068427	A	C	intergenic	*HDAC9,TWIST1*	.	.	.
7	19075820	T	C	intergenic	*HDAC9,TWIST1*	.	.	.
7	19087726	T	G	intergenic	*HDAC9,TWIST1*	.	.	.
7	19088300	C	T	intergenic	*HDAC9,TWIST1*	.	.	.
7	19088302	T	C	intergenic	*HDAC9,TWIST1*	.	.	.
7	19088458	G	A	intergenic	*HDAC9,TWIST1*	.	.	.
7	19089855	T	G	intergenic	*HDAC9,TWIST1*	.	.	.
15	50554133	C	T	intronic	*HDC*	.	.	.
6	125679797	G	GTATA	intergenic	*HDDC2,LOC643623*	.	.	.
6	125718232	G	A	intergenic	*HDDC2,LOC643623*	.	.	.
6	125720539	C	A	intergenic	*HDDC2,LOC643623*	.	.	.
6	125722633	G	A	intergenic	*HDDC2,LOC643623*	.	.	.
6	125930389	A	G	intergenic	*HDDC2,LOC643623*	.	.	.
6	125930426	G	A	intergenic	*HDDC2,LOC643623*	.	.	.
6	125930826	T	C	intergenic	*HDDC2,LOC643623*	.	.	.
6	22946384	C	G	intergenic	*HDGFL1,NRSN1*	.	.	.
6	22767012	G	C	intergenic	*HDGFL1,NRSN1*	.	.	.
6	22715522	T	TA	intergenic	*HDGFL1,NRSN1*	.	.	.
6	22690205	C	T	intergenic	*HDGFL1,NRSN1*	.	.	.

6	22915741	C	A	intergenic	*HDGFL1,NRSN1*	.	.	.
6	22983946	A	AC	intergenic	*HDGFL1,NRSN1*	.	.	.
6	22989884	T	C	intergenic	*HDGFL1,NRSN1*	.	.	.
6	22992028	C	T	intergenic	*HDGFL1,NRSN1*	.	.	.
6	23063098	A	G	intergenic	*HDGFL1,NRSN1*	.	.	.
6	23110199	C	CAAAAAAAA	intergenic	*HDGFL1,NRSN1*	.	.	.
6	23121439	C	A	intergenic	*HDGFL1,NRSN1*	.	.	.
6	23124270	T	C	intergenic	*HDGFL1,NRSN1*	.	.	.
6	23124271	G	A	intergenic	*HDGFL1,NRSN1*	.	.	.
6	23153238	C	G	intergenic	*HDGFL1,NRSN1*	.	.	.
6	23153239	C	A	intergenic	*HDGFL1,NRSN1*	.	.	.
6	23362116	C	T	intergenic	*HDGFL1,NRSN1*	.	.	.
6	23362117	T	G	intergenic	*HDGFL1,NRSN1*	.	.	.
6	23431292	A	C	intergenic	*HDGFL1,NRSN1*	.	.	.
6	23431293	T	TTTTC	intergenic	*HDGFL1,NRSN1*	.	.	.
6	23665401	A	C	intergenic	*HDGFL1,NRSN1*	.	.	.
6	23665402	A	T	intergenic	*HDGFL1,NRSN1*	.	.	.
6	23665653	G	A	intergenic	*HDGFL1,NRSN1*	.	.	.
6	23758155	A	T	intergenic	*HDGFL1,NRSN1*	.	.	.
6	23777172	C	T	intergenic	*HDGFL1,NRSN1*	.	.	.
6	23798872	C	T	intergenic	*HDGFL1,NRSN1*	.	.	.
15	83892446	C	T	intergenic	*HDGFRP3,BNC1*	.	.	.
15	83893582	C	A	intergenic	*HDGFRP3,BNC1*	.	.	.
18	44660353	G	T	intronic	*HDHD2*	.	.	.
X	83655141	T	A	intronic	*HDX*	.	.	.
X	83621610	G	C	intronic	*HDX*	.	.	.
X	83628718	T	C	intronic	*HDX*	.	.	.
X	83664115	A	C	intronic	*HDX*	.	.	.
X	83719494	T	C	intronic	*HDX*	.	.	.
X	83719777	A	G	intronic	*HDX*	.	.	.
X	83945905	GCACA	G	intergenic	*HDX,UBE2DNL*	.	.	.
X	83772128	C	G	intergenic	*HDX,UBE2DNL*	.	.	.
X	83792788	G	A	intergenic	*HDX,UBE2DNL*	.	.	.
X	83803080	T	C	intergenic	*HDX,UBE2DNL*	.	.	.
X	83914962	G	A	intergenic	*HDX,UBE2DNL*	.	.	.
X	83914963	T	A	intergenic	*HDX,UBE2DNL*	.	.	.
X	83947357	C	T	intergenic	*HDX,UBE2DNL*	.	.	.
X	83948475	A	G	intergenic	*HDX,UBE2DNL*	.	.	.

2	37278530	T	A	intronic	*HEATR5B*	.	.	.
14	31750132	C	A	intergenic	*HECTD1,HEATR5A*	.	.	.
10	93210771	T	A	ncRNA_intronic	*HECTD2-AS1*	.	.	.
10	93084975	T	C	ncRNA_intronic	*HECTD2-AS1*	.	.	.
10	93085057	T	G	ncRNA_intronic	*HECTD2-AS1*	.	.	.
10	93121589	T	C	ncRNA_intronic	*HECTD2-AS1*	.	.	.
10	93121590	G	A	ncRNA_intronic	*HECTD2-AS1*	.	.	.
10	93122001	G	T	ncRNA_intronic	*HECTD2-AS1*	.	.	.
10	93122002	C	T	ncRNA_intronic	*HECTD2-AS1*	.	.	.
10	93209490	C	T	ncRNA_intronic	*HECTD2-AS1*	.	.	.
10	93209579	T	G	ncRNA_intronic	*HECTD2-AS1*	.	.	.
10	93210116	C	T	ncRNA_intronic	*HECTD2-AS1*	.	.	.
10	93291681	G	A	ncRNA_intronic	*HECTD2-AS1*	.	.	.
10	93292557	G	A	ncRNA_intronic	*HECTD2-AS1*	.	.	.
12	112697833	T	C	intronic	*HECTD4*	.	.	.
12	112634851	C	T	intronic	*HECTD4*	.	.	.
12	112638688	CGTGTGTGT	C	intronic	*HECTD4*	.	.	.
12	112827695	G	A	intergenic	*HECTD4,RPL6*	.	.	.
7	43376570	A	T	intronic	*HECW1*	.	.	.
7	43383200	C	A	intronic	*HECW1*	.	.	.
7	43386571	T	C	intronic	*HECW1*	.	.	.
7	43386572	G	A	intronic	*HECW1*	.	.	.
7	43423175	A	G	intronic	*HECW1*	.	.	.
7	43423176	C	T	intronic	*HECW1*	.	.	.
7	43613400	G	A	intergenic	*HECW1,STK17A*	.	.	.
2	197258339	G	C	intronic	*HECW2*	.	.	.
2	197386963	A	G	intronic	*HECW2*	.	.	.
3	124796558	A	G	intergenic	*HEG1,SLC12A8*	.	.	.
3	124776717	CAAT	C	intergenic	*HEG1,SLC12A8*	.	.	.
10	96311255	C	T	intronic	*HELLS*	.	.	.
17	65097251	T	A	intronic	*HELZ*	.	.	.
9	100689585	T	C	UTR3	*HEMGN*	.	.	.
9	100711599	T	G	intergenic	*HEMGN,ANP32B*	.	.	.
X	65487387	A	G	downstream	*HEPH*	.	.	.
X	65398876	A	G	intronic	*HEPH*	.	.	.
X	65432923	A	T	intronic	*HEPH*	.	.	.
X	65432924	A	G	intronic	*HEPH*	.	.	.

X	65444663	ATCTC	A	intronic	*HEPH*	.	.	.
X	65465623	A	T	intronic	*HEPH*	.	.	.
X	65704229	A	G	intergenic	*HEPH,EDA2R*	.	.	.
X	65505733	GTGTGTGT ATATATATA CACACATA TATACGTAT ATATGTGT GTGTATAT ATATACAC ATATATAC GTATATA	G	intergenic	*HEPH,EDA2R*	.	.	.
X	65756688	T	G	intergenic	*HEPH,EDA2R*	.	.	.
X	65526099	A	G	intergenic	*HEPH,EDA2R*	.	.	.
X	65527507	T	A	intergenic	*HEPH,EDA2R*	.	.	.
X	65554673	A	T	intergenic	*HEPH,EDA2R*	.	.	.
X	65555580	A	G	intergenic	*HEPH,EDA2R*	.	.	.
X	65591838	C	T	intergenic	*HEPH,EDA2R*	.	.	.
X	65594148	T	C	intergenic	*HEPH,EDA2R*	.	.	.
X	65597725	A	G	intergenic	*HEPH,EDA2R*	.	.	.
X	65597726	T	A	intergenic	*HEPH,EDA2R*	.	.	.
X	65610355	T	C	intergenic	*HEPH,EDA2R*	.	.	.
X	65610679	G	T	intergenic	*HEPH,EDA2R*	.	.	.
X	65641228	A	G	intergenic	*HEPH,EDA2R*	.	.	.
X	65650838	G	A	intergenic	*HEPH,EDA2R*	.	.	.
X	65679117	T	C	intergenic	*HEPH,EDA2R*	.	.	.
X	65714188	A	G	intergenic	*HEPH,EDA2R*	.	.	.
X	65714190	T	G	intergenic	*HEPH,EDA2R*	.	.	.
X	65780666	T	C	intergenic	*HEPH,EDA2R*	.	.	.
X	65784835	T	C	intergenic	*HEPH,EDA2R*	.	.	.
X	65785880	C	A	intergenic	*HEPH,EDA2R*	.	.	.
X	65785882	T	G	intergenic	*HEPH,EDA2R*	.	.	.
X	65785935	G	A	intergenic	*HEPH,EDA2R*	.	.	.
X	65805459	T	G	intergenic	*HEPH,EDA2R*	.	.	.
X	65805460	A	G	intergenic	*HEPH,EDA2R*	.	.	.
11	93831874	G	A	intronic	*HEPHL1*	.	.	.
11	93855365	G	A	intergenic	*HEPHL1,PANX1*	.	.	.

15	63922686	C	T	exonic	*HERC1*	stopgain	.	.
15	63998914	CTTTT	C	intronic	*HERC1*	.	.	.
15	28557972	G	A	intronic	*HERC2*	.	.	.
16	32234269	T	G	intergenic	*HERC2P4,TP53TG 3D*	.	.	.
16	32202956	G	A	intergenic	*HERC2P4,TP53TG 3D*	.	.	.
15	23392841	C	A	ncRNA_exonic	*HERC2P7*	.	.	.
15	28938999	T	C	intergenic	*HERC2P9,GOLGA8 M*	.	.	.
15	28939000	G	C	intergenic	*HERC2P9,GOLGA8 M*	.	.	.
4	89560784	T	C	intronic	*HERC3*	.	.	.
7	35681513	G	T	intronic	*HERPUD2*	.	.	.
7	35703068	A	G	intronic	*HERPUD2*	.	.	.
7	35723280	A	G	intronic	*HERPUD2*	.	.	.
5	74005610	T	A	intronic	*HEXB*	.	.	.
6	26094204	G	T	intronic	*HFE*	.	.	.
1	91905304	A	G	intergenic	*HFM1,CDC7*	.	.	.
1	91888922	A	G	intergenic	*HFM1,CDC7*	.	.	.
7	81380177	G	A	intronic	*HGF*	.	.	.
7	81439873	C	A	intergenic	*HGF,CACNA2D1*	.	.	.
7	81492940	CT	C	intergenic	*HGF,CACNA2D1*	.	.	.
7	81457048	C	T	intergenic	*HGF,CACNA2D1*	.	.	.
7	81493868	T	A	intergenic	*HGF,CACNA2D1*	.	.	.
7	81541289	T	G	intergenic	*HGF,CACNA2D1*	.	.	.
7	81541290	T	G	intergenic	*HGF,CACNA2D1*	.	.	.
8	43009855	A	T	intronic	*HGSNAT*	.	.	.
8	43040150	A	G	intronic	*HGSNAT*	.	.	.
8	43040151	C	G	intronic	*HGSNAT*	.	.	.
8	43042062	T	C	intronic	*HGSNAT*	.	.	.
8	43059437	C	T	intergenic	*HGSNAT,POTEA*	.	.	.
1	210617971	A	T	intronic	*HHAT*	.	.	.
1	210832428	G	A	intronic	*HHAT*	.	.	.
1	210832610	T	C	intronic	*HHAT*	.	.	.
4	145715825	G	A	intergenic	*HHIP,ANAPC10*	.	.	.
4	145716666	T	C	intergenic	*HHIP,ANAPC10*	.	.	.
4	145716667	G	A	intergenic	*HHIP,ANAPC10*	.	.	.

4	145788676	A	C	intergenic	*HHIP,ANAPC10*	.	.	.
4	145817429	G	A	intergenic	*HHIP,ANAPC10*	.	.	.
4	145879609	G	C	intergenic	*HHIP,ANAPC10*	.	.	.
4	145902230	C	A	intergenic	*HHIP,ANAPC10*	.	.	.
4	145902231	A	G	intergenic	*HHIP,ANAPC10*	.	.	.
3	108087564	T	C	intronic	*HHLA2*	.	.	.
3	108091935	T	C	intronic	*HHLA2*	.	.	.
7	27767100	C	G	intergenic	*HIBADH,TAX1BP1*	.	.	.
2	191089387	C	T	intronic	*HIBCH*	.	.	.
2	191087798	A	G	intronic	*HIBCH*	.	.	.
2	191090805	G	A	intronic	*HIBCH*	.	.	.
2	191132192	A	G	intronic	*HIBCH*	.	.	.
10	102462227	G	T	intergenic	*HIF1AN,PAX2*	.	.	.
10	102463291	G	T	intergenic	*HIF1AN,PAX2*	.	.	.
7	75339454	G	A	intronic	*HIP1*	.	.	.
7	75388486	T	C	intergenic	*HIP1,CCL26*	.	.	.
7	75388487	G	A	intergenic	*HIP1,CCL26*	.	.	.
1	228616464	C	G	intergenic	*HIST3H3,HIST3H2 A*	.	.	.
12	14925708	G	A	intergenic	*HIST4H4,H2AFJ*	.	.	.
6	12083443	T	C	intronic	*HIVEP1*	.	.	.
6	12156187	C	T	intronic	*HIVEP1*	.	.	.
6	12156188	C	G	intronic	*HIVEP1*	.	.	.
6	12237093	T	C	intergenic	*HIVEP1,EDN1*	.	.	.
6	143151691	A	G	intronic	*HIVEP2*	.	.	.
6	143284419	G	A	intergenic	*HIVEP2,LINC01277*	.	.	.
1	42017341	A	G	intronic	*HIVEP3*	.	.	.
1	42017979	T	C	intronic	*HIVEP3*	.	.	.
1	42369858	G	A	intronic	*HIVEP3*	.	.	.
1	42435013	A	G	intergenic	*HIVEP3,NONE*	.	.	.
6	29911815	C	A	intronic	*HLA-A*	.	.	.
6	31236598	C	T	UTR3	*HLA-C*	.	.	.
6	31250557	C	T	intergenic	*HLA-C,HLA-B*	.	.	.
6	31275973	T	TGCCGAGCC	intergenic	*HLA-C,HLA-B*	.	.	.
6	31274807	A	AGC	intergenic	*HLA-C,HLA-B*	.	.	.
6	31274808	T	TTG	intergenic	*HLA-C,HLA-B*	.	.	.

6	33066610	T	C	intergenic	*HLA-DPB1,HLA-DPB2*	.	.	.
6	32701074	T	C	intergenic	*HLA-DQB1,HLA-DQA2*	.	.	.
6	32673111	T	A	intergenic	*HLA-DQB1,HLA-DQA2*	.	.	.
6	32677653	TTCCTTCCTTCCTTCTC	T	intergenic	*HLA-DQB1,HLA-DQA2*	.	.	.
6	32771865	T	C	intergenic	*HLA-DQB2,HLA-DOB*	.	.	.
6	32436369	C	A	intergenic	*HLA-DRA,HLA-DRB5*	.	.	.
6	30507778	C	T	intergenic	*HLA-E,GNL1*	.	.	.
6	30501728	G	A	intergenic	*HLA-E,GNL1*	.	.	.
6	29977470	C	T	ncRNA_exonic	*HLA-J*	.	.	.
21	38208880	C	T	intronic	*HLCS*	.	.	.
1	221033825	A	G	ncRNA_intronic	*HLX-AS1*	.	.	.
1	221033980	A	G	ncRNA_intronic	*HLX-AS1*	.	.	.
1	221319060	A	G	intergenic	*HLX,C1orf140*	.	.	.
1	221073492	A	G	intergenic	*HLX,C1orf140*	.	.	.
1	221073493	T	C	intergenic	*HLX,C1orf140*	.	.	.
1	221142040	C	T	intergenic	*HLX,C1orf140*	.	.	.
1	221224038	C	A	intergenic	*HLX,C1orf140*	.	.	.
1	221224039	A	G	intergenic	*HLX,C1orf140*	.	.	.
1	221225793	T	C	intergenic	*HLX,C1orf140*	.	.	.
1	221225794	T	C	intergenic	*HLX,C1orf140*	.	.	.
1	221383783	A	T	intergenic	*HLX,C1orf140*	.	.	.
1	221384679	A	G	intergenic	*HLX,C1orf140*	.	.	.
1	221384680	T	C	intergenic	*HLX,C1orf140*	.	.	.
1	221385253	A	G	intergenic	*HLX,C1orf140*	.	.	.
1	221431198	A	T	intergenic	*HLX,C1orf140*	.	.	.
1	221433273	A	G	intergenic	*HLX,C1orf140*	.	.	.
1	221433557	C	T	intergenic	*HLX,C1orf140*	.	.	.
20	30178513	T	C	intergenic	*HM13-AS1,ID1*	.	.	.
20	30178514	G	A	intergenic	*HM13-AS1,ID1*	.	.	.
8	28854507	GGTGT	G	intronic	*HMBOX1*	.	.	.
1	185955775	A	G	intronic	*HMCN1*	.	.	.
1	185853352	T	C	intronic	*HMCN1*	.	.	.

1	185794825	A	G	intronic	*HMCN1*	.	.	.
1	185748716	G	C	intronic	*HMCN1*	.	.	.
1	185749219	A	G	intronic	*HMCN1*	.	.	.
1	185749909	G	A	intronic	*HMCN1*	.	.	.
1	185751718	A	T	intronic	*HMCN1*	.	.	.
1	185798776	T	A	intronic	*HMCN1*	.	.	.
9	133226121	A	AAT	intronic	*HMCN2*	.	.	.
12	66409947	C	T	intergenic	*HMGA2,MIR6074*	.	.	.
12	66373438	A	G	intergenic	*HMGA2,MIR6074*	.	.	.
12	66375584	T	C	intergenic	*HMGA2,MIR6074*	.	.	.
X	150186656	T	C	intergenic	*HMGB3,MIR4330*	.	.	.
X	150235087	C	A	intergenic	*HMGB3,MIR4330*	.	.	.
X	150235089	G	A	intergenic	*HMGB3,MIR4330*	.	.	.
X	150289786	T	C	intergenic	*HMGB3,MIR4330*	.	.	.
X	150291590	T	C	intergenic	*HMGB3,MIR4330*	.	.	.
6	55476909	A	C	intergenic	*HMGCLL1,BMP5*	.	.	.
6	55476910	G	A	intergenic	*HMGCLL1,BMP5*	.	.	.
6	55603561	T	C	intergenic	*HMGCLL1,BMP5*	.	.	.
5	43302784	A	G	intronic	*HMGCS1*	.	.	.
5	43327442	C	T	intergenic	*HMGCS1,CCL28*	.	.	.
5	43327443	A	G	intergenic	*HMGCS1,CCL28*	.	.	.
5	43343908	A	G	intergenic	*HMGCS1,CCL28*	.	.	.
5	43345043	T	A	intergenic	*HMGCS1,CCL28*	.	.	.
5	43345347	A	T	intergenic	*HMGCS1,CCL28*	.	.	.
6	79963982	G	A	intergenic	*HMGN3-AS1,LCAL1*	.	.	.
6	79964811	G	T	intergenic	*HMGN3-AS1,LCAL1*	.	.	.
6	79997478	G	A	intergenic	*HMGN3-AS1,LCAL1*	.	.	.
6	26579791	A	C	intergenic	*HMGN4,ABT1*	.	.	.
X	80415594	C	A	intronic	*HMGN5*	.	.	.
X	80455079	C	T	intronic	*HMGN5*	.	.	.
X	80403500	A	G	intronic	*HMGN5*	.	.	.
X	80404035	T	A	intronic	*HMGN5*	.	.	.
X	80407298	A	G	intronic	*HMGN5*	.	.	.
X	80434593	A	C	intronic	*HMGN5*	.	.	.
X	80435522	T	C	intronic	*HMGN5*	.	.	.

X	80454556	G	A	intronic	*HMGN5*	.	.	.
X	80456001	G	A	intronic	*HMGN5*	.	.	.
5	143436567	C	T	intergenic	*HMHB1,YIPF5*	.	.	.
5	143331751	T	A	intergenic	*HMHB1,YIPF5*	.	.	.
5	143431698	C	T	intergenic	*HMHB1,YIPF5*	.	.	.
5	143432586	A	G	intergenic	*HMHB1,YIPF5*	.	.	.
5	173614409	A	G	intergenic	*HMP19,LINC01411*	.	.	.
5	173724147	T	C	intergenic	*HMP19,LINC01411*	.	.	.
5	173724900	A	T	intergenic	*HMP19,LINC01411*	.	.	.
4	8918969	A	G	intergenic	*HMX1,LOC650293*	.	.	.
4	8937904	G	A	intergenic	*HMX1,LOC650293*	.	.	.
17	73158603	C	T	intergenic	*HN1,SUMO2*	.	.	.
8	76504227	C	T	intergenic	*HNF4G,LINC01111*	.	.	.
8	76676192	T	C	intergenic	*HNF4G,LINC01111*	.	.	.
8	76961685	G	T	intergenic	*HNF4G,LINC01111*	.	.	.
8	76564166	C	CA	intergenic	*HNF4G,LINC01111*	.	.	.
8	76690604	A	G	intergenic	*HNF4G,LINC01111*	.	.	.
8	77283819	T	TATAC	intergenic	*HNF4G,LINC01111*	.	.	.
8	76599102	C	A	intergenic	*HNF4G,LINC01111*	.	.	.
8	76488274	G	T	intergenic	*HNF4G,LINC01111*	.	.	.
8	76548296	T	C	intergenic	*HNF4G,LINC01111*	.	.	.
8	76676976	A	G	intergenic	*HNF4G,LINC01111*	.	.	.
8	76680390	A	T	intergenic	*HNF4G,LINC01111*	.	.	.
8	76787300	T	C	intergenic	*HNF4G,LINC01111*	.	.	.
8	76839259	G	A	intergenic	*HNF4G,LINC01111*	.	.	.
8	77021831	T	C	intergenic	*HNF4G,LINC01111*	.	.	.
8	77021832	T	C	intergenic	*HNF4G,LINC01111*	.	.	.
2	138754970	T	C	intronic	*HNMT*	.	.	.
2	138754971	G	A	intronic	*HNMT*	.	.	.
2	139088180	T	C	intergenic	*HNMT,SPOPL*	.	.	.
2	138899642	T	C	intergenic	*HNMT,SPOPL*	.	.	.
2	138900082	C	T	intergenic	*HNMT,SPOPL*	.	.	.
2	139046965	T	A	intergenic	*HNMT,SPOPL*	.	.	.
2	139219631	G	T	intergenic	*HNMT,SPOPL*	.	.	.
2	139219632	A	C	intergenic	*HNMT,SPOPL*	.	.	.
2	139241138	A	G	intergenic	*HNMT,SPOPL*	.	.	.
5	137121061	G	T	intergenic	*HNRNPA0,NPY6R*	.	.	.

10	44329712	C	T	intergenic	*HNRNPA3P1,LINC00619*	.	.	.
4	83311533	C	T	intergenic	*HNRNPD,HNRNPDL*	.	.	.
9	86594606	A	C	intronic	*HNRNPK*	.	.	.
19	39345305	T	C	intergenic	*HNRNPL,RINL*	.	.	.
19	8553715	G	A	exonic	*HNRNPM*	nonsynonymous_SNV	.	.
1	23634942	A	G	UTR3	*HNRNPR*	.	.	.
5	78681652	C	T	intronic	*HOMER1*	.	.	.
15	83595193	A	G	intronic	*HOMER2*	.	.	.
8	42799906	G	C	intronic	*HOOK3*	.	.	.
8	42791593	A	G	intronic	*HOOK3*	.	.	.
8	42792367	A	G	intronic	*HOOK3*	.	.	.
8	42793246	C	T	intronic	*HOOK3*	.	.	.
4	57583136	T	C	intergenic	*HOPX,SPINK2*	.	.	.
1	150699518	T	C	intergenic	*HORMAD1,CTSS*	.	.	.
22	30530396	ATATATATATTTATGATTATACTTAAAGTATAATCATAAATAAATATAT	A	intronic	*HORMAD2*	.	.	.
12	54368249	T	TA	ncRNA_intronic	*HOTAIR*	.	.	.
12	54343636	T	TAGA	intergenic	*HOXC13,HOXC12*	.	.	.
2	177065479	A	T	intergenic	*HOXD1,MTX2*	.	.	.
2	10573793	TGA	T	intergenic	*HPCAL1,ODC1*	.	.	.
12	122318090	G	C	intronic	*HPD*	.	.	.
X	133631883	G	A	intronic	*HPRT1*	.	.	.
22	26850533	C	T	intronic	*HPS4*	.	.	.
10	100509470	A	T	intronic	*HPSE2*	.	.	.
10	100747789	A	G	intronic	*HPSE2*	.	.	.
10	100259822	G	A	intronic	*HPSE2*	.	.	.
10	100280974	A	G	intronic	*HPSE2*	.	.	.
10	100304542	C	G	intronic	*HPSE2*	.	.	.

10	100327514	G	A	intronic	*HPSE2*	.	.	.
10	100464068	C	T	intronic	*HPSE2*	.	.	.
10	100483475	C	T	intronic	*HPSE2*	.	.	.
10	100484212	C	G	intronic	*HPSE2*	.	.	.
10	100545498	G	A	intronic	*HPSE2*	.	.	.
10	100692152	A	AT	intronic	*HPSE2*	.	.	.
10	100692154	AG	A	intronic	*HPSE2*	.	.	.
10	100715511	T	C	intronic	*HPSE2*	.	.	.
10	100864169	G	A	intronic	*HPSE2*	.	.	.
10	100936574	G	T	intronic	*HPSE2*	.	.	.
10	100939836	C	T	intronic	*HPSE2*	.	.	.
10	100941288	G	C	intronic	*HPSE2*	.	.	.
7	54364165	G	A	intergenic	*HPVC1,LINC01445*	.	.	.
7	54374704	T	C	intergenic	*HPVC1,LINC01445*	.	.	.
7	54339679	C	T	intergenic	*HPVC1,LINC01445*	.	.	.
7	54361731	T	C	intergenic	*HPVC1,LINC01445*	.	.	.
7	54363209	A	G	intergenic	*HPVC1,LINC01445*	.	.	.
7	54370921	C	T	intergenic	*HPVC1,LINC01445*	.	.	.
7	54374735	A	T	intergenic	*HPVC1,LINC01445*	.	.	.
7	54379233	A	G	intergenic	*HPVC1,LINC01445*	.	.	.
7	54389991	T	C	intergenic	*HPVC1,LINC01445*	.	.	.
11	63251848	C	T	intronic	*HRASLS5*	.	.	.
11	63261245	A	T	intergenic	*HRASLS5,LGALS12*	.	.	.
5	117766845	T	A	ncRNA_intronic	*HRAT56*	.	.	.
5	117697955	C	T	ncRNA_intronic	*HRAT56*	.	.	.
5	117739350	G	C	ncRNA_intronic	*HRAT56*	.	.	.
5	117739351	C	A	ncRNA_intronic	*HRAT56*	.	.	.
5	117830860	T	A	ncRNA_intronic	*HRAT56*	.	.	.
5	117835281	T	G	ncRNA_intronic	*HRAT56*	.	.	.
3	11186170	C	CCTATAGAC	intronic	*HRH1*	.	.	.
5	175126662	C	T	intergenic	*HRH2,CPLX2*	.	.	.
18	22132618	C	T	intergenic	*HRH4,LOC729950*	.	.	.
12	117332480	C	T	intergenic	*HRK,FBXW8*	.	.	.
1	152233585	T	C	intergenic	*HRNR,FLG*	.	.	.
1	152234419	G	A	intergenic	*HRNR,FLG*	.	.	.
1	152237665	A	T	intergenic	*HRNR,FLG*	.	.	.
1	152237666	T	C	intergenic	*HRNR,FLG*	.	.	.

1	152245339	C	T	intergenic	*HRNR,FLG*	.	.	.
1	152261680	G	A	intergenic	*HRNR,FLG*	.	.	.
1	152262340	G	GGCTC	intergenic	*HRNR,FLG*	.	.	.
1	152262385	C	T	intergenic	*HRNR,FLG*	.	.	.
1	152268301	G	A	intergenic	*HRNR,FLG*	.	.	.
1	152270650	G	A	intergenic	*HRNR,FLG*	.	.	.
1	87534079	C	T	intronic	*HS2ST1*	.	.	.
4	11734613	G	A	intergenic	*HS3ST1,LOC101929019*	.	.	.
4	11958109	G	T	intergenic	*HS3ST1,LOC101929019*	.	.	.
4	12018214	G	A	intergenic	*HS3ST1,LOC101929019*	.	.	.
4	12077505	A	G	intergenic	*HS3ST1,LOC101929019*	.	.	.
4	11947478	T	TGC	intergenic	*HS3ST1,LOC101929019*	.	.	.
4	12214480	A	C	intergenic	*HS3ST1,LOC101929019*	.	.	.
4	11562984	A	C	intergenic	*HS3ST1,LOC101929019*	.	.	.
4	11563340	C	A	intergenic	*HS3ST1,LOC101929019*	.	.	.
4	11582742	T	C	intergenic	*HS3ST1,LOC101929019*	.	.	.
4	11671611	TG	T	intergenic	*HS3ST1,LOC101929019*	.	.	.
4	11915151	ACACACACACACACACCC	A	intergenic	*HS3ST1,LOC101929019*	.	.	.
4	11963036	C	G	intergenic	*HS3ST1,LOC101929019*	.	.	.
4	11964567	A	T	intergenic	*HS3ST1,LOC101929019*	.	.	.
4	11965013	A	T	intergenic	*HS3ST1,LOC101929019*	.	.	.
4	11965015	A	T	intergenic	*HS3ST1,LOC101929019*	.	.	.

4	12094917	C	T	intergenic	*HS3ST1,LOC101929019*	.	.	.
16	22951566	C	G	intergenic	*HS3ST2,USP31*	.	.	.
17	13599530	C	T	intergenic	*HS3ST3A1,CDRT15P1*	.	.	.
17	13761482	C	A	intergenic	*HS3ST3A1,CDRT15P1*	.	.	.
17	13782491	C	G	intergenic	*HS3ST3A1,CDRT15P1*	.	.	.
17	13655640	T	A	intergenic	*HS3ST3A1,CDRT15P1*	.	.	.
17	13780348	A	C	intergenic	*HS3ST3A1,CDRT15P1*	.	.	.
17	14498513	T	C	intergenic	*HS3ST3B1,CDRT7*	.	.	.
17	14368925	C	T	intergenic	*HS3ST3B1,CDRT7*	.	.	.
17	14413389	C	A	intergenic	*HS3ST3B1,CDRT7*	.	.	.
17	14437204	G	C	intergenic	*HS3ST3B1,CDRT7*	.	.	.
17	14505710	T	A	intergenic	*HS3ST3B1,CDRT7*	.	.	.
17	14506522	G	C	intergenic	*HS3ST3B1,CDRT7*	.	.	.
16	25750777	T	G	intronic	*HS3ST4*	.	.	.
16	25914448	G	A	intronic	*HS3ST4*	.	.	.
16	26046712	C	A	intronic	*HS3ST4*	.	.	.
16	25742064	T	C	intronic	*HS3ST4*	.	.	.
16	25774237	T	A	intronic	*HS3ST4*	.	.	.
16	26109008	A	T	intronic	*HS3ST4*	.	.	.
16	26464933	G	A	intergenic	*HS3ST4,C16orf82*	.	.	.
16	26595407	C	A	intergenic	*HS3ST4,C16orf82*	.	.	.
16	26333134	G	T	intergenic	*HS3ST4,C16orf82*	.	.	.
16	26503574	G	A	intergenic	*HS3ST4,C16orf82*	.	.	.
16	26509286	TCACA	T	intergenic	*HS3ST4,C16orf82*	.	.	.
16	26967563	C	A	intergenic	*HS3ST4,C16orf82*	.	.	.
16	26322978	C	T	intergenic	*HS3ST4,C16orf82*	.	.	.
16	26420573	C	T	intergenic	*HS3ST4,C16orf82*	.	.	.
16	26433552	T	C	intergenic	*HS3ST4,C16orf82*	.	.	.
16	26646553	A	G	intergenic	*HS3ST4,C16orf82*	.	.	.
16	26860475	G	A	intergenic	*HS3ST4,C16orf82*	.	.	.
2	129248419	G	T	intergenic	*HS6ST1,LOC101927881*	.	.	.

2	129546694	T	TTG	intergenic	*HS6ST1,LOC101927881*	.	.	.
2	129421943	C	A	intergenic	*HS6ST1,LOC101927881*	.	.	.
2	129583899	G	A	intergenic	*HS6ST1,LOC101927881*	.	.	.
2	129583900	G	T	intergenic	*HS6ST1,LOC101927881*	.	.	.
2	129584247	T	C	intergenic	*HS6ST1,LOC101927881*	.	.	.
2	129616605	T	A	intergenic	*HS6ST1,LOC101927881*	.	.	.
X	132002231	A	G	intronic	*HS6ST2*	.	.	.
X	132002462	C	T	intronic	*HS6ST2*	.	.	.
X	132139935	T	C	intergenic	*HS6ST2,USP26*	.	.	.
X	132149144	G	A	intergenic	*HS6ST2,USP26*	.	.	.
13	97220560	A	G	intronic	*HS6ST3*	.	.	.
13	97247716	C	T	intronic	*HS6ST3*	.	.	.
13	96798339	C	T	intronic	*HS6ST3*	.	.	.
13	97306300	A	G	intronic	*HS6ST3*	.	.	.
13	97350666	G	A	intronic	*HS6ST3*	.	.	.
13	97350954	T	C	intronic	*HS6ST3*	.	.	.
13	97350955	G	A	intronic	*HS6ST3*	.	.	.
13	97351198	T	C	intronic	*HS6ST3*	.	.	.
13	97351199	G	A	intronic	*HS6ST3*	.	.	.
13	97556794	C	A	intergenic	*HS6ST3,LINC00359*	.	.	.
13	97576229	G	A	intergenic	*HS6ST3,LINC00359*	.	.	.
1	209917681	T	C	intergenic	*HSD11B1,TRAF3IP3*	.	.	.
16	67466193	T	G	intronic	*HSD11B2*	.	.	.
11	43736444	G	T	intronic	*HSD17B12*	.	.	.
11	43797211	A	G	intronic	*HSD17B12*	.	.	.
11	43798280	C	T	intronic	*HSD17B12*	.	.	.
11	43798662	T	C	intronic	*HSD17B12*	.	.	.
19	49330255	T	C	intronic	*HSD17B14*	.	.	.
19	49332993	C	T	intronic	*HSD17B14*	.	.	.

19	49332994	A	G	intronic	*HSD17B14*	.	.	.
16	82177081	G	A	intergenic	*HSD17B2,MPHOSPH6*	.	.	.
9	99018908	G	A	intronic	*HSD17B3*	.	.	.
9	99052913	T	C	intronic	*HSD17B3*	.	.	.
5	118888373	T	A	intergenic	*HSD17B4,FAM170A*	.	.	.
5	118889249	T	C	intergenic	*HSD17B4,FAM170A*	.	.	.
5	118889250	G	A	intergenic	*HSD17B4,FAM170A*	.	.	.
5	118938058	T	C	intergenic	*HSD17B4,FAM170A*	.	.	.
5	118940119	C	A	intergenic	*HSD17B4,FAM170A*	.	.	.
5	118940836	A	G	intergenic	*HSD17B4,FAM170A*	.	.	.
12	57241564	T	A	intergenic	*HSD17B6,SDR9C7*	.	.	.
12	57284093	C	A	intergenic	*HSD17B6,SDR9C7*	.	.	.
12	57285423	T	C	intergenic	*HSD17B6,SDR9C7*	.	.	.
12	57285424	G	A	intergenic	*HSD17B6,SDR9C7*	.	.	.
12	57307296	T	C	intergenic	*HSD17B6,SDR9C7*	.	.	.
1	162779109	T	C	intronic	*HSD17B7*	.	.	.
1	162810025	AGGGGGGAGGGAGGGAAGGAAGGAAGGAAGGAAGGAAG	A	intergenic	*HSD17B7,CCDC190*	.	.	.
10	38662417	G	T	ncRNA_intronic	*HSD17B7P2*	.	.	.
10	38664617	T	C	ncRNA_intronic	*HSD17B7P2*	.	.	.
1	59597491	GTATTAT	G	downstream	*HSD52*	.	.	.
1	59720904	A	C	intergenic	*HSD52,FGGY*	.	.	.
9	115171566	T	C	intronic	*HSDL2*	.	.	.
8	145523587	G	A	intronic	*HSF1*	.	.	.
21	44975840	A	C	intronic	*HSF2BP*	.	.	.
21	44995117	C	T	intronic	*HSF2BP*	.	.	.
22	17376539	C	T	intergenic	*HSFY1P1,GAB4*	.	.	.

22	17390081	G	A	intergenic	*HSFY1P1,GAB4*	.	.	.
22	17361255	C	CGT	intergenic	*HSFY1P1,GAB4*	.	.	.
22	17424092	A	T	intergenic	*HSFY1P1,GAB4*	.	.	.
21	15792838	A	T	intergenic	*HSPA13,SAMSN1*	.	.	.
10	14907825	C	A	intronic	*HSPA14*	.	.	.
5	132471925	A	G	intergenic	*HSPA4,FSTL4*	.	.	.
5	132472511	A	G	intergenic	*HSPA4,FSTL4*	.	.	.
5	132472513	G	A	intergenic	*HSPA4,FSTL4*	.	.	.
5	132504675	A	C	intergenic	*HSPA4,FSTL4*	.	.	.
1	161585626	T	C	intergenic	*HSPA7,FCGR3B*	.	.	.
5	138059786	A	G	intergenic	*HSPA9,CTNNA1*	.	.	.
5	138059787	G	T	intergenic	*HSPA9,CTNNA1*	.	.	.
5	53782680	T	C	intergenic	*HSPB3,SNX18*	.	.	.
2	198427981	A	G	intergenic	*HSPE1-MOB4,RFTN2*	.	.	.
2	198430818	T	C	intergenic	*HSPE1-MOB4,RFTN2*	.	.	.
4	70922286	A	G	intronic	*HTN1*	.	.	.
5	63271143	G	A	intergenic	*HTR1A,RNF180*	.	.	.
5	63404066	C	A	intergenic	*HTR1A,RNF180*	.	.	.
5	63271644	G	T	intergenic	*HTR1A,RNF180*	.	.	.
5	63274589	T	C	intergenic	*HTR1A,RNF180*	.	.	.
5	63354741	T	C	intergenic	*HTR1A,RNF180*	.	.	.
5	63388280	T	A	intergenic	*HTR1A,RNF180*	.	.	.
5	63397334	A	C	intergenic	*HTR1A,RNF180*	.	.	.
5	63399925	C	T	intergenic	*HTR1A,RNF180*	.	.	.
6	78340219	C	T	intergenic	*HTR1B,MEI4*	.	.	.
6	78249314	C	A	intergenic	*HTR1B,MEI4*	.	.	.
6	78295817	TCACACA	T	intergenic	*HTR1B,MEI4*	.	.	.
6	78368844	G	T	intergenic	*HTR1B,MEI4*	.	.	.
6	78337497	G	A	intergenic	*HTR1B,MEI4*	.	.	.
6	78210006	A	G	intergenic	*HTR1B,MEI4*	.	.	.
6	78247839	C	A	intergenic	*HTR1B,MEI4*	.	.	.
6	78247841	G	A	intergenic	*HTR1B,MEI4*	.	.	.
6	78248613	G	A	intergenic	*HTR1B,MEI4*	.	.	.
6	87746190	C	T	intergenic	*HTR1E,CGA*	.	.	.
6	87746191	A	G	intergenic	*HTR1E,CGA*	.	.	.
13	47447562	TTTATTA	T	intronic	*HTR2A*	.	.	.

13	48214824	T	C	intergenic	*HTR2A,LINC00562*	.	.	.
13	48175114	G	A	intergenic	*HTR2A,LINC00562*	.	.	.
13	48314050	G	T	intergenic	*HTR2A,LINC00562*	.	.	.
13	47601312	C	T	intergenic	*HTR2A,LINC00562*	.	.	.
13	47516236	G	A	intergenic	*HTR2A,LINC00562*	.	.	.
13	47516238	A	G	intergenic	*HTR2A,LINC00562*	.	.	.
13	47769518	A	G	intergenic	*HTR2A,LINC00562*	.	.	.
13	47916024	A	C	intergenic	*HTR2A,LINC00562*	.	.	.
13	48142044	T	C	intergenic	*HTR2A,LINC00562*	.	.	.
13	48143143	T	C	intergenic	*HTR2A,LINC00562*	.	.	.
13	48143480	T	C	intergenic	*HTR2A,LINC00562*	.	.	.
13	48204746	T	A	intergenic	*HTR2A,LINC00562*	.	.	.
13	48210821	T	C	intergenic	*HTR2A,LINC00562*	.	.	.
13	48210823	A	C	intergenic	*HTR2A,LINC00562*	.	.	.
13	48303580	C	T	intergenic	*HTR2A,LINC00562*	.	.	.
13	48328053	G	T	intergenic	*HTR2A,LINC00562*	.	.	.
13	48360444	G	A	intergenic	*HTR2A,LINC00562*	.	.	.
13	48484275	C	T	intergenic	*HTR2A,LINC00562*	.	.	.
X	113938072	A	G	intronic	*HTR2C*	.	.	.
X	114027260	A	G	intronic	*HTR2C*	.	.	.
X	114027261	T	A	intronic	*HTR2C*	.	.	.
X	114030181	A	G	intronic	*HTR2C*	.	.	.
X	114172392	A	T	intergenic	*HTR2C,IL13RA2*	.	.	.
X	114172573	C	T	intergenic	*HTR2C,IL13RA2*	.	.	.
X	114172574	A	G	intergenic	*HTR2C,IL13RA2*	.	.	.
X	114175038	T	A	intergenic	*HTR2C,IL13RA2*	.	.	.
X	114175040	A	C	intergenic	*HTR2C,IL13RA2*	.	.	.
11	113908657	G	A	intergenic	*HTR3A,ZBTB16*	.	.	.
11	113796960	T	C	intronic	*HTR3B*	.	.	.
5	147883993	A	G	intronic	*HTR4*	.	.	.
5	148149008	C	A	intergenic	*HTR4,ADRB2*	.	.	.
7	154897231	CAAA	C	intergenic	*HTR5A,INSIG1*	.	.	.
10	124313807	CTTATGTACCAGATATGGACTCTTTTTAAGAGTCCCATATCTG	C	intergenic	*HTRA1,DMBT1*	.	.	.

4	3163060	C	T	intronic	*HTT*	.	.	.
4	3166124	G	A	intronic	*HTT*	.	.	.
4	3188747	G	A	intronic	*HTT*	.	.	.
4	3238894	G	A	intronic	*HTT*	.	.	.
21	33256797	T	C	intronic	*HUNK*	.	.	.
21	33256798	G	A	intronic	*HUNK*	.	.	.
21	33413918	A	G	intergenic	*HUNK,LINC00159*	.	.	.
X	53704622	T	A	intronic	*HUWE1*	.	.	.
X	53602633	G	T	exonic	*HUWE1*	nonsynonymous_SNV	.	.
X	53587728	C	A	intronic	*HUWE1*	.	.	.
X	53673129	G	C	intronic	*HUWE1*	.	.	.
X	53645548	C	T	intronic	*HUWE1*	.	.	.
X	53717433	A	G	intergenic	*HUWE1,PHF8*	.	.	.
X	53876508	C	T	intergenic	*HUWE1,PHF8*	.	.	.
X	53877109	T	C	intergenic	*HUWE1,PHF8*	.	.	.
X	53877724	G	A	intergenic	*HUWE1,PHF8*	.	.	.
X	53877976	A	G	intergenic	*HUWE1,PHF8*	.	.	.
X	53917555	C	A	intergenic	*HUWE1,PHF8*	.	.	.
X	53918759	T	C	intergenic	*HUWE1,PHF8*	.	.	.
X	53919182	A	C	intergenic	*HUWE1,PHF8*	.	.	.
X	53919975	C	A	intergenic	*HUWE1,PHF8*	.	.	.
12	111144471	A	C	intergenic	*HVCN1,PPP1CC*	.	.	.
7	123493969	A	G	intronic	*HYAL4*	.	.	.
7	123479355	G	A	intergenic	*HYALP1,HYAL4*	.	.	.
7	123479375	C	A	intergenic	*HYALP1,HYAL4*	.	.	.
7	123480169	C	T	intergenic	*HYALP1,HYAL4*	.	.	.
16	70982205	C	T	intronic	*HYDIN*	.	.	.
X	37862368	C	T	intergenic	*HYPM,SYTL5*	.	.	.
6	82926559	A	G	intronic	*IBTK*	.	.	.
6	83003431	A	AATC	intergenic	*IBTK,TPBG*	.	.	.
6	83011464	T	C	intergenic	*IBTK,TPBG*	.	.	.
6	83012983	A	G	intergenic	*IBTK,TPBG*	.	.	.
6	83034735	G	A	intergenic	*IBTK,TPBG*	.	.	.
7	8179761	A	G	intronic	*ICA1*	.	.	.
7	8236593	C	T	intronic	*ICA1*	.	.	.
7	8283797	A	G	intronic	*ICA1*	.	.	.
2	203697967	A	G	intronic	*ICA1L*	.	.	.

2	203716491	C	T	intronic	*ICA1L*	.	.	.
2	203716805	C	A	intronic	*ICA1L*	.	.	.
2	203716826	T	A	intronic	*ICA1L*	.	.	.
2	203719048	G	T	intronic	*ICA1L*	.	.	.
2	203720970	T	C	intronic	*ICA1L*	.	.	.
19	10381171	T	C	upstream	*ICAM1*	.	.	.
17	62091690	G	GGAAAGAAA	intronic	*ICAM2*	.	.	.
5	5478016	A	G	intronic	*ICE1*	.	.	.
5	5478018	C	A	intronic	*ICE1*	.	.	.
5	5479435	G	A	intronic	*ICE1*	.	.	.
5	5877099	A	G	intergenic	*ICE1,FLJ33360*	.	.	.
5	5668145	T	G	intergenic	*ICE1,FLJ33360*	.	.	.
5	6130547	A	C	intergenic	*ICE1,FLJ33360*	.	.	.
5	6246931	G	T	intergenic	*ICE1,FLJ33360*	.	.	.
5	5658856	TTAGTATTATTGTAGCTATGCCTTTCTATTCTTTGGTAGTATTATCA	T	intergenic	*ICE1,FLJ33360*	.	.	.
5	6174131	A	G	intergenic	*ICE1,FLJ33360*	.	.	.
15	60735003	C	CACATAGGC	intronic	*ICE2*	.	.	.
2	204804762	C	G	intronic	*ICOS*	.	.	.
2	205101430	C	T	intergenic	*ICOS,PARD3B*	.	.	.
2	204960499	C	A	intergenic	*ICOS,PARD3B*	.	.	.
2	205329932	A	AAAAC	intergenic	*ICOS,PARD3B*	.	.	.
2	204955963	A	G	intergenic	*ICOS,PARD3B*	.	.	.
2	205168463	C	T	intergenic	*ICOS,PARD3B*	.	.	.
2	205152107	G	A	intergenic	*ICOS,PARD3B*	.	.	.
2	205291358	C	G	intergenic	*ICOS,PARD3B*	.	.	.
2	205291360	C	T	intergenic	*ICOS,PARD3B*	.	.	.
2	205330022	T	TG	intergenic	*ICOS,PARD3B*	.	.	.
6	19869527	G	T	intergenic	*ID4,MBOAT1*	.	.	.
6	19877044	A	G	intergenic	*ID4,MBOAT1*	.	.	.
6	19992069	G	A	intergenic	*ID4,MBOAT1*	.	.	.
10	94275039	C	T	intronic	*IDE*	.	.	.
10	94344977	C	T	intergenic	*IDE,KIF11*	.	.	.
15	78456629	TTCTC	T	intronic	*IDH3A*	.	.	.

8	39876911	C	T	intergenic	*IDO2,C8orf4*	.	.	.
8	39946561	A	ATTTATTTTA	intergenic	*IDO2,C8orf4*	.	.	.
X	148572110	T	TAAC	intronic	*IDS*	.	.	.
X	148590834	C	A	intergenic	*IDS,LINC00893*	.	.	.
X	148598381	C	T	intergenic	*IDS,LINC00893*	.	.	.
18	44716466	C	G	intergenic	*IER3IP1,SKOR2*	.	.	.
1	181116243	A	G	intergenic	*IER5,LOC101928973*	.	.	.
1	19378905	C	T	intergenic	*IFFO2,UBR4*	.	.	.
1	159012586	T	C	intronic	*IFI16*	.	.	.
1	158997231	A	G	intronic	*IFI16*	.	.	.
14	94614346	C	T	intergenic	*IFI27L2,PPP4R4*	.	.	.
14	94614414	A	T	intergenic	*IFI27L2,PPP4R4*	.	.	.
14	94616926	C	T	intergenic	*IFI27L2,PPP4R4*	.	.	.
14	94616927	A	G	intergenic	*IFI27L2,PPP4R4*	.	.	.
1	79156242	G	A	intergenic	*IFI44,ADGRL4*	.	.	.
1	79164357	T	C	intergenic	*IFI44,ADGRL4*	.	.	.
1	27999953	ATCTC	A	intergenic	*IFI6,FAM76A*	.	.	.
10	91081031	G	A	intergenic	*IFIT2,IFIT3*	.	.	.
10	91096518	G	A	intronic	*IFIT3*	.	.	.
6	29724006	GTGTGTGTGTGTGTGTGTGTGTT	G	intergenic	*IFITM4P,HCG4*	.	.	.
6	29752684	A	G	intergenic	*IFITM4P,HCG4*	.	.	.
9	21445378	GATAT	G	intergenic	*IFNA1,MIR31HG*	.	.	.
9	21393502	A	G	intergenic	*IFNA2,IFNA8*	.	.	.
9	21300453	A	G	intergenic	*IFNA22P,IFNA5*	.	.	.
9	21290946	T	A	intergenic	*IFNA22P,IFNA5*	.	.	.
9	21193808	T	C	intergenic	*IFNA4,IFNA7*	.	.	.
9	21325008	G	A	intergenic	*IFNA5,KLHL9*	.	.	.
9	21359552	A	AT	intergenic	*IFNA6,IFNA13*	.	.	.
9	21204650	A	G	intergenic	*IFNA7,IFNA10*	.	.	.
21	34759111	T	A	intergenic	*IFNAR1,IFNGR2*	.	.	.
21	34760456	T	C	intergenic	*IFNAR1,IFNGR2*	.	.	.
9	21127250	C	CA	intergenic	*IFNB1,IFNW1*	.	.	.
12	68464038	A	C	intergenic	*IFNG-AS1,IFNG*	.	.	.
12	68450371	G	A	intergenic	*IFNG-AS1,IFNG*	.	.	.
12	68450372	C	T	intergenic	*IFNG-AS1,IFNG*	.	.	.

12	68454048	A	T	intergenic	*IFNG-AS1,IFNG*	.	.	.
6	137749780	A	G	intergenic	*IFNGR1,OLIG3*	.	.	.
6	137545875	G	A	intergenic	*IFNGR1,OLIG3*	.	.	.
21	34795216	T	A	intronic	*IFNGR2*	.	.	.
7	112064657	TG	T	intronic	*IFRD1*	.	.	.
3	129241653	A	G	intergenic	*IFT122,RHO*	.	.	.
3	107981668	T	C	intergenic	*IFT57,HHLA2*	.	.	.
3	107994212	T	C	intergenic	*IFT57,HHLA2*	.	.	.
3	160080133	G	A	intronic	*IFT80*	.	.	.
12	110615313	T	C	intronic	*IFT81*	.	.	.
12	102841596	T	C	intronic	*IGF1*	.	.	.
12	102842637	T	C	intronic	*IGF1*	.	.	.
12	102842638	G	A	intronic	*IGF1*	.	.	.
12	102885230	C	G	intergenic	*IGF1,LINC00485*	.	.	.
12	103056983	G	T	intergenic	*IGF1,LINC00485*	.	.	.
12	102883682	G	A	intergenic	*IGF1,LINC00485*	.	.	.
12	103002339	C	A	intergenic	*IGF1,LINC00485*	.	.	.
12	103137714	G	T	intergenic	*IGF1,LINC00485*	.	.	.
6	160417711	G	A	intronic	*IGF2R*	.	.	.
16	1848238	C	CATCAAAAA	intergenic	*IGFALS,HAGH*	.	.	.
7	46324778	C	T	intergenic	*IGFBP3,TNS3*	.	.	.
7	46983017	T	C	intergenic	*IGFBP3,TNS3*	.	.	.
7	47244559	C	T	intergenic	*IGFBP3,TNS3*	.	.	.
7	46347607	GTGTATA	G	intergenic	*IGFBP3,TNS3*	.	.	.
7	46290685	C	G	intergenic	*IGFBP3,TNS3*	.	.	.
7	46667486	A	G	intergenic	*IGFBP3,TNS3*	.	.	.
7	45987144	C	A	intergenic	*IGFBP3,TNS3*	.	.	.
7	46126644	C	T	intergenic	*IGFBP3,TNS3*	.	.	.
7	46377275	C	T	intergenic	*IGFBP3,TNS3*	.	.	.
7	46379010	C	G	intergenic	*IGFBP3,TNS3*	.	.	.
7	46379871	C	T	intergenic	*IGFBP3,TNS3*	.	.	.
7	46411435	G	T	intergenic	*IGFBP3,TNS3*	.	.	.
7	46521598	A	G	intergenic	*IGFBP3,TNS3*	.	.	.
7	46539593	A	C	intergenic	*IGFBP3,TNS3*	.	.	.
7	46540115	C	T	intergenic	*IGFBP3,TNS3*	.	.	.
7	46603129	G	A	intergenic	*IGFBP3,TNS3*	.	.	.
7	46633626	T	A	intergenic	*IGFBP3,TNS3*	.	.	.
7	46634122	C	A	intergenic	*IGFBP3,TNS3*	.	.	.

7	46634124	T	C	intergenic	*IGFBP3,TNS3*	.	.	.
7	46635673	A	C	intergenic	*IGFBP3,TNS3*	.	.	.
7	46635685	T	C	intergenic	*IGFBP3,TNS3*	.	.	.
7	47137560	A	G	intergenic	*IGFBP3,TNS3*	.	.	.
7	47149196	A	ATC	intergenic	*IGFBP3,TNS3*	.	.	.
7	47202407	A	C	intergenic	*IGFBP3,TNS3*	.	.	.
7	47204832	A	G	intergenic	*IGFBP3,TNS3*	.	.	.
7	47204990	T	A	intergenic	*IGFBP3,TNS3*	.	.	.
4	58252982	G	A	intergenic	*IGFBP7-AS1,LOC101928851*	.	.	.
19	46688408	T	C	intergenic	*IGFL2,DKFZp434J0226*	.	.	.
1	201178977	C	T	exonic	*IGFN1*	synonymous_SNV	.	.
1	201207576	T	TGGC	intergenic	*IGFN1,PKP1*	.	.	.
1	201200857	T	C	intergenic	*IGFN1,PKP1*	.	.	.
1	201201181	A	G	intergenic	*IGFN1,PKP1*	.	.	.
11	68744099	C	G	intergenic	*IGHMBP2,MRGPRD*	.	.	.
22	25738036	A	G	intergenic	*IGLL3P,LRP5L*	.	.	.
22	25738598	C	T	intergenic	*IGLL3P,LRP5L*	.	.	.
22	23314680	A	G	intergenic	*IGLL5,RSPH14*	.	.	.
22	23314853	A	T	intergenic	*IGLL5,RSPH14*	.	.	.
22	23347861	T	C	intergenic	*IGLL5,RSPH14*	.	.	.
22	23347863	C	A	intergenic	*IGLL5,RSPH14*	.	.	.
22	23391474	T	A	intergenic	*IGLL5,RSPH14*	.	.	.
X	130421164	T	TAG	intronic	*IGSF1*	.	.	.
X	130470906	C	A	intergenic	*IGSF1,OR13H1*	.	.	.
X	130515149	A	G	intergenic	*IGSF1,OR13H1*	.	.	.
X	130572059	A	C	intergenic	*IGSF1,OR13H1*	.	.	.
X	130584868	T	A	intergenic	*IGSF1,OR13H1*	.	.	.
X	130599979	T	C	intergenic	*IGSF1,OR13H1*	.	.	.
X	130639274	G	A	intergenic	*IGSF1,OR13H1*	.	.	.
X	130660610	A	G	intergenic	*IGSF1,OR13H1*	.	.	.

3	151250749	A	AACTTCAGC	intergenic	*IGSF10,MIR5186*	.	.	.
1	18595773	C	A	intronic	*IGSF21*	.	.	.
21	41157304	T	C	intronic	*IGSF5*	.	.	.
21	41178555	G	A	intergenic	*IGSF5,PCP4*	.	.	.
21	41178722	A	G	intergenic	*IGSF5,PCP4*	.	.	.
7	50425059	CAAGTCCA CTGCTGA	C	intronic	*IKZF1*	.	.	.
1	206959971	A	G	intergenic	*IL10,IL19*	.	.	.
3	159831627	AG	A	ncRNA_intronic	*IL12A-AS1*	.	.	.
X	117896536	CAGCA	C	intronic	*IL13RA1*	.	.	.
X	117911592	G	T	intronic	*IL13RA1*	.	.	.
X	117940160	T	A	intergenic	*IL13RA1,ZCCHC12*	.	.	.
4	142619415	T	C	intronic	*IL15*	.	.	.
4	142758083	C	T	intergenic	*IL15,INPP4B*	.	.	.
4	142759730	T	A	intergenic	*IL15,INPP4B*	.	.	.
4	142804206	G	C	intergenic	*IL15,INPP4B*	.	.	.
4	142804306	G	T	intergenic	*IL15,INPP4B*	.	.	.
4	142889929	A	T	intergenic	*IL15,INPP4B*	.	.	.
4	142890059	G	C	intergenic	*IL15,INPP4B*	.	.	.
4	142891619	T	C	intergenic	*IL15,INPP4B*	.	.	.
4	142891620	G	A	intergenic	*IL15,INPP4B*	.	.	.
4	142909175	G	A	intergenic	*IL15,INPP4B*	.	.	.
4	142910875	G	A	intergenic	*IL15,INPP4B*	.	.	.
15	81600960	C	A	exonic	*IL16*	nonsynonymous_SNV	.	.
15	81494964	T	C	intronic	*IL16*	.	.	.
6	52117832	GGAGA	G	intergenic	*IL17F,MCM3*	.	.	.
2	113648610	C	T	intergenic	*IL1B,IL37*	.	.	.
2	113616401	A	C	intergenic	*IL1B,IL37*	.	.	.
2	102744092	G	T	intronic	*IL1R1*	.	.	.
2	102656006	T	C	intergenic	*IL1R2,IL1R1*	.	.	.
2	102656008	A	C	intergenic	*IL1R2,IL1R1*	.	.	.
2	102656249	G	A	intergenic	*IL1R2,IL1R1*	.	.	.
X	28791178	ATCTC	A	intronic	*IL1RAPL1*	.	.	.
X	29542598	G	T	intronic	*IL1RAPL1*	.	.	.
X	29926492	AATAT	A	intronic	*IL1RAPL1*	.	.	.
X	28640974	A	T	intronic	*IL1RAPL1*	.	.	.
X	28663318	G	T	intronic	*IL1RAPL1*	.	.	.

X	28858728	AT	A	intronic	*IL1RAPL1*	.	.	.
X	28769837	C	A	intronic	*IL1RAPL1*	.	.	.
X	28934231	C	G	intronic	*IL1RAPL1*	.	.	.
X	29500882	T	C	intronic	*IL1RAPL1*	.	.	.
X	28734773	T	A	intronic	*IL1RAPL1*	.	.	.
X	28956345	T	A	intronic	*IL1RAPL1*	.	.	.
X	28973614	G	T	intronic	*IL1RAPL1*	.	.	.
X	30180971	AATAT	A	intergenic	*IL1RAPL1,MAGEB2*	.	.	.
X	30000626	C	T	intergenic	*IL1RAPL1,MAGEB2*	.	.	.
X	30001047	T	C	intergenic	*IL1RAPL1,MAGEB2*	.	.	.
X	103940015	C	A	intronic	*IL1RAPL2*	.	.	.
X	104272682	A	G	intronic	*IL1RAPL2*	.	.	.
X	104713647	G	C	intronic	*IL1RAPL2*	.	.	.
X	103851414	A	G	intronic	*IL1RAPL2*	.	.	.
X	104078578	CTT	C	intronic	*IL1RAPL2*	.	.	.
X	104881490	G	A	intronic	*IL1RAPL2*	.	.	.
X	104008929	T	C	intronic	*IL1RAPL2*	.	.	.
X	103915603	T	C	intronic	*IL1RAPL2*	.	.	.
X	103850301	AGAGT	A	intronic	*IL1RAPL2*	.	.	.
X	104137781	T	C	intronic	*IL1RAPL2*	.	.	.
X	104407500	G	T	intronic	*IL1RAPL2*	.	.	.
X	104441552	C	A	intronic	*IL1RAPL2*	.	.	.
X	103816102	C	T	intronic	*IL1RAPL2*	.	.	.
X	103848683	C	T	intronic	*IL1RAPL2*	.	.	.
X	103848684	A	G	intronic	*IL1RAPL2*	.	.	.
X	103851062	G	C	intronic	*IL1RAPL2*	.	.	.
X	103851441	T	C	intronic	*IL1RAPL2*	.	.	.
X	103852009	C	G	intronic	*IL1RAPL2*	.	.	.
X	103878633	G	A	intronic	*IL1RAPL2*	.	.	.
X	103900632	A	T	intronic	*IL1RAPL2*	.	.	.
X	103901713	G	A	intronic	*IL1RAPL2*	.	.	.
X	103902211	C	A	intronic	*IL1RAPL2*	.	.	.
X	103902231	A	G	intronic	*IL1RAPL2*	.	.	.
X	103902302	G	T	intronic	*IL1RAPL2*	.	.	.
X	104066896	C	G	intronic	*IL1RAPL2*	.	.	.

X	104066980	A	G	intronic	*IL1RAPL2*	.	.	.
X	104070874	T	TTTAA	intronic	*IL1RAPL2*	.	.	.
X	104084512	G	A	intronic	*IL1RAPL2*	.	.	.
X	104136076	T	G	intronic	*IL1RAPL2*	.	.	.
X	104159647	A	T	intronic	*IL1RAPL2*	.	.	.
X	104187770	G	A	intronic	*IL1RAPL2*	.	.	.
X	104194063	C	T	intronic	*IL1RAPL2*	.	.	.
X	104194065	C	A	intronic	*IL1RAPL2*	.	.	.
X	104253442	T	A	intronic	*IL1RAPL2*	.	.	.
X	104253814	T	C	intronic	*IL1RAPL2*	.	.	.
X	104253815	G	A	intronic	*IL1RAPL2*	.	.	.
X	104284960	A	T	intronic	*IL1RAPL2*	.	.	.
X	104322220	C	T	intronic	*IL1RAPL2*	.	.	.
X	104325210	C	T	intronic	*IL1RAPL2*	.	.	.
X	104325583	T	G	intronic	*IL1RAPL2*	.	.	.
X	104460676	T	C	intronic	*IL1RAPL2*	.	.	.
X	104460678	A	G	intronic	*IL1RAPL2*	.	.	.
X	104627354	C	T	intronic	*IL1RAPL2*	.	.	.
X	104627436	A	T	intronic	*IL1RAPL2*	.	.	.
X	104658967	G	T	intronic	*IL1RAPL2*	.	.	.
X	104931069	A	G	intronic	*IL1RAPL2*	.	.	.
X	105051612	A	T	intergenic	*IL1RAPL2,NRK*	.	.	.
X	105045862	C	T	intergenic	*IL1RAPL2,NRK*	.	.	.
X	105050531	A	G	intergenic	*IL1RAPL2,NRK*	.	.	.
X	105052398	T	C	intergenic	*IL1RAPL2,NRK*	.	.	.
2	102901426	A	G	intergenic	*IL1RL2,IL1RL1*	.	.	.
4	123382015	A	AT	intergenic	*IL2,IL21*	.	.	.
4	123416770	T	C	intergenic	*IL2,IL21*	.	.	.
4	123417264	T	G	intergenic	*IL2,IL21*	.	.	.
4	123417947	T	G	intergenic	*IL2,IL21*	.	.	.
6	137436906	T	C	intergenic	*IL20RA,IL22RA2*	.	.	.
6	137392765	A	G	intergenic	*IL20RA,IL22RA2*	.	.	.
6	137393452	A	C	intergenic	*IL20RA,IL22RA2*	.	.	.
3	136705086	TAAATAAATAAATAAAA	T	intronic	*IL20RB*	.	.	.
3	137129935	A	G	intergenic	*IL20RB,SOX14*	.	.	.
3	137458344	G	T	intergenic	*IL20RB,SOX14*	.	.	.

3	137463029	C	T	intergenic	*IL20RB,SOX14*	.	.	.
3	137281472	C	T	intergenic	*IL20RB,SOX14*	.	.	.
3	136966800	GACACACA C	G	intergenic	*IL20RB,SOX14*	.	.	.
3	136795156	G	A	intergenic	*IL20RB,SOX14*	.	.	.
12	68682183	G	A	intergenic	*IL22,MDM1*	.	.	.
6	137465447	C	G	UTR3	*IL22RA2*	.	.	.
9	6258721	C	T	downstream	*IL33*	.	.	.
9	6310632	T	C	intergenic	*IL33,TPD52L3*	.	.	.
9	6297822	T	C	intergenic	*IL33,TPD52L3*	.	.	.
9	6297823	G	A	intergenic	*IL33,TPD52L3*	.	.	.
2	113766747	T	A	intergenic	*IL36A,IL36B*	.	.	.
X	1496891	G	A	intronic	*IL3RA*	.	.	.
X	1495610	C	T	intronic	*IL3RA*	.	.	.
16	27409284	T	A	intergenic	*IL4R,IL21R*	.	.	.
7	22792627	C	G	intergenic	*IL6,TOMM7*	.	.	.
7	22792628	A	G	intergenic	*IL6,TOMM7*	.	.	.
7	22793326	T	C	intergenic	*IL6,TOMM7*	.	.	.
7	22793327	G	C	intergenic	*IL6,TOMM7*	.	.	.
1	154382353	G	C	intronic	*IL6R*	.	.	.
8	79693859	G	A	intronic	*IL7*	.	.	.
8	79963933	A	G	intergenic	*IL7,STMN2*	.	.	.
8	80136847	A	T	intergenic	*IL7,STMN2*	.	.	.
8	80179946	T	C	intergenic	*IL7,STMN2*	.	.	.
8	80300312	T	C	intergenic	*IL7,STMN2*	.	.	.
8	80326469	A	G	intergenic	*IL7,STMN2*	.	.	.
8	80373441	G	A	intergenic	*IL7,STMN2*	.	.	.
8	80502176	A	ATATTTCC	intergenic	*IL7,STMN2*	.	.	.
X	155229810	A	AAAT	intronic	*IL9R*	.	.	.
1	166939314	C	T	intronic	*ILDR2*	.	.	.
2	239126709	G	T	intergenic	*ILKAP,LOC151174*	.	.	.
7	111173027	G	C	intronic	*IMMP2L*	.	.	.
7	111146930	C	T	intronic	*IMMP2L*	.	.	.
7	110688345	G	C	intronic	*IMMP2L*	.	.	.
7	110717294	G	C	intronic	*IMMP2L*	.	.	.
7	110621031	G	T	intronic	*IMMP2L*	.	.	.
7	110385295	G	A	intronic	*IMMP2L*	.	.	.
7	110385681	A	C	intronic	*IMMP2L*	.	.	.

7	110385933	G	A	intronic	*IMMP2L*	.	.	.
7	110388555	C	T	intronic	*IMMP2L*	.	.	.
7	110388557	T	C	intronic	*IMMP2L*	.	.	.
7	110455174	C	T	intronic	*IMMP2L*	.	.	.
7	110465337	C	A	intronic	*IMMP2L*	.	.	.
7	110465936	C	T	intronic	*IMMP2L*	.	.	.
7	111060832	A	T	intronic	*IMMP2L*	.	.	.
7	111063837	C	T	intronic	*IMMP2L*	.	.	.
7	111090218	C	T	intronic	*IMMP2L*	.	.	.
7	111110417	A	C	intronic	*IMMP2L*	.	.	.
7	111110904	T	A	intronic	*IMMP2L*	.	.	.
7	111307065	C	A	intergenic	*IMMP2L,DOCK4*	.	.	.
7	111216212	G	T	intergenic	*IMMP2L,DOCK4*	.	.	.
7	111341035	CG	C	intergenic	*IMMP2L,DOCK4*	.	.	.
7	111341037	G	A	intergenic	*IMMP2L,DOCK4*	.	.	.
7	110761998	G	A	intronic	*IMMP2L,LRRN3*	.	.	.
7	110761999	C	T	intronic	*IMMP2L,LRRN3*	.	.	.
8	82580215	C	T	intronic	*IMPA1*	.	.	.
18	12020443	C	T	intronic	*IMPA2*	.	.	.
18	11987995	A	T	intronic	*IMPA2*	.	.	.
18	12085784	G	C	intergenic	*IMPA2,ANKRD62*	.	.	.
8	57905255	A	C	intronic	*IMPAD1*	.	.	.
8	58120980	C	A	intergenic	*IMPAD1,LINC01606*	.	.	.
8	57937383	C	T	intergenic	*IMPAD1,LINC01606*	.	.	.
8	58009238	C	T	intergenic	*IMPAD1,LINC01606*	.	.	.
6	76757503	C	A	intronic	*IMPG1*	.	.	.
6	76668040	C	T	intronic	*IMPG1*	.	.	.
6	77917014	G	A	intergenic	*IMPG1,HTR1B*	.	.	.
6	76899189	C	A	intergenic	*IMPG1,HTR1B*	.	.	.
6	77273280	A	C	intergenic	*IMPG1,HTR1B*	.	.	.
6	77400222	G	A	intergenic	*IMPG1,HTR1B*	.	.	.
6	77541677	C	G	intergenic	*IMPG1,HTR1B*	.	.	.
6	76810475	G	A	intergenic	*IMPG1,HTR1B*	.	.	.
6	76810477	G	A	intergenic	*IMPG1,HTR1B*	.	.	.
6	76857534	A	G	intergenic	*IMPG1,HTR1B*	.	.	.

6	76879875	T	C	intergenic	*IMPG1,HTR1B*	.	.	.
6	76903684	T	C	intergenic	*IMPG1,HTR1B*	.	.	.
6	76903685	G	A	intergenic	*IMPG1,HTR1B*	.	.	.
6	76921753	C	T	intergenic	*IMPG1,HTR1B*	.	.	.
6	76921977	T	C	intergenic	*IMPG1,HTR1B*	.	.	.
6	76921978	G	A	intergenic	*IMPG1,HTR1B*	.	.	.
6	76991110	C	T	intergenic	*IMPG1,HTR1B*	.	.	.
6	76991111	A	G	intergenic	*IMPG1,HTR1B*	.	.	.
6	77006589	A	G	intergenic	*IMPG1,HTR1B*	.	.	.
6	77009500	TA	T	intergenic	*IMPG1,HTR1B*	.	.	.
6	77009903	C	T	intergenic	*IMPG1,HTR1B*	.	.	.
6	77009904	T	A	intergenic	*IMPG1,HTR1B*	.	.	.
6	77010168	A	G	intergenic	*IMPG1,HTR1B*	.	.	.
6	77011949	T	A	intergenic	*IMPG1,HTR1B*	.	.	.
6	77011951	A	C	intergenic	*IMPG1,HTR1B*	.	.	.
6	77045627	C	A	intergenic	*IMPG1,HTR1B*	.	.	.
6	77078025	A	G	intergenic	*IMPG1,HTR1B*	.	.	.
6	77078026	C	T	intergenic	*IMPG1,HTR1B*	.	.	.
6	77082640	A	G	intergenic	*IMPG1,HTR1B*	.	.	.
6	77086498	T	A	intergenic	*IMPG1,HTR1B*	.	.	.
6	77086499	A	G	intergenic	*IMPG1,HTR1B*	.	.	.
6	77455456	C	T	intergenic	*IMPG1,HTR1B*	.	.	.
6	77531928	C	T	intergenic	*IMPG1,HTR1B*	.	.	.
6	77534788	C	T	intergenic	*IMPG1,HTR1B*	.	.	.
6	77557140	C	A	intergenic	*IMPG1,HTR1B*	.	.	.
6	77600903	A	G	intergenic	*IMPG1,HTR1B*	.	.	.
6	77671093	T	A	intergenic	*IMPG1,HTR1B*	.	.	.
6	77739128	T	G	intergenic	*IMPG1,HTR1B*	.	.	.
6	77881218	C	T	intergenic	*IMPG1,HTR1B*	.	.	.
6	77881219	A	T	intergenic	*IMPG1,HTR1B*	.	.	.
6	77964038	T	C	intergenic	*IMPG1,HTR1B*	.	.	.
6	77964299	A	C	intergenic	*IMPG1,HTR1B*	.	.	.
6	78013368	C	T	intergenic	*IMPG1,HTR1B*	.	.	.
6	78135908	G	A	intergenic	*IMPG1,HTR1B*	.	.	.
11	61898031	C	T	exonic	*INCENP*	synonymous_SNV	.	.
11	61950867	G	A	intergenic	*INCENP,SCGB1D1*	.	.	.

2	121182578	TCGGGGCCACTTTGAACCAGCCAGTTCAAAGTGGCCCCGACCCTGGA	T	intergenic	*INHBB,LINC01101*	.	.	.
2	121170421	T	C	intergenic	*INHBB,LINC01101*	.	.	.
15	41315796	G	A	intronic	*INO80*	.	.	.
18	33167559	A	T	intergenic	*INO80C,MIR3975*	.	.	.
2	206897606	T	C	intronic	*INO80D*	.	.	.
4	143400610	T	G	intronic	*INPP4B*	.	.	.
4	143597249	C	T	intronic	*INPP4B*	.	.	.
4	143036282	C	T	intronic	*INPP4B*	.	.	.
4	143036283	C	G	intronic	*INPP4B*	.	.	.
4	143037907	T	A	intronic	*INPP4B*	.	.	.
4	143058125	C	T	intronic	*INPP4B*	.	.	.
4	143359578	T	A	intronic	*INPP4B*	.	.	.
4	143524883	C	CTGATGATC	intronic	*INPP4B*	.	.	.
4	143571584	T	A	intronic	*INPP4B*	.	.	.
4	143574485	G	A	intronic	*INPP4B*	.	.	.
4	143852623	C	A	intergenic	*INPP4B,USP38*	.	.	.
4	143916573	G	A	intergenic	*INPP4B,USP38*	.	.	.
4	144026099	G	A	intergenic	*INPP4B,USP38*	.	.	.
4	144065795	C	A	intergenic	*INPP4B,USP38*	.	.	.
4	144066025	A	G	intergenic	*INPP4B,USP38*	.	.	.
10	134532575	C	T	intronic	*INPP5A*	.	.	.
2	233959029	A	C	intronic	*INPP5D*	.	.	.
11	15160222	A	G	intronic	*INSC*	.	.	.
11	15622112	C	A	intergenic	*INSC,LOC102724957*	.	.	.
2	118850876	T	TGATGAG	intronic	*INSIG2*	.	.	.
2	118957931	T	C	intergenic	*INSIG2,LOC101927709*	.	.	.
2	118887506	G	T	intergenic	*INSIG2,LOC101927709*	.	.	.
2	119169317	C	A	intergenic	*INSIG2,LOC101927709*	.	.	.

2	119069803	G	T	intergenic	*INSIG2,LOC101927709*	.	.	.
2	119169845	T	C	intergenic	*INSIG2,LOC101927709*	.	.	.
2	119170118	T	C	intergenic	*INSIG2,LOC101927709*	.	.	.
2	119170119	G	A	intergenic	*INSIG2,LOC101927709*	.	.	.
2	119172214	T	A	intergenic	*INSIG2,LOC101927709*	.	.	.
2	119209591	C	T	intergenic	*INSIG2,LOC101927709*	.	.	.
2	119210699	G	A	intergenic	*INSIG2,LOC101927709*	.	.	.
2	119212749	C	T	intergenic	*INSIG2,LOC101927709*	.	.	.
2	119212750	A	G	intergenic	*INSIG2,LOC101927709*	.	.	.
2	119263493	CGTGT	C	intergenic	*INSIG2,LOC101927709*	.	.	.
9	5256113	T	C	intergenic	*INSL4,RLN2*	.	.	.
9	5296648	A	G	intergenic	*INSL4,RLN2*	.	.	.
9	5296649	A	G	intergenic	*INSL4,RLN2*	.	.	.
9	5215712	G	GT	intergenic	*INSL6,INSL4*	.	.	.
20	20349721	G	C	exonic	*INSM1*	nonsynonymous_SNV	.	.
19	7196193	G	A	intronic	*INSR*	.	.	.
19	7233090	C	T	intronic	*INSR*	.	.	.
19	7435200	G	T	intergenic	*INSR,ARHGEF18*	.	.	.
1	156825710	CGTGT	C	intronic	*INSRR,NTRK1*	.	.	.
8	19765573	A	T	intergenic	*INTS10,LPL*	.	.	.
8	19769541	T	C	intergenic	*INTS10,LPL*	.	.	.
8	19791051	G	A	intergenic	*INTS10,LPL*	.	.	.
8	19792012	T	C	intergenic	*INTS10,LPL*	.	.	.
11	77642767	A	G	intronic	*INTS4*	.	.	.
11	77663003	T	C	intronic	*INTS4*	.	.	.
7	65171527	T	C	ncRNA_intronic	*INTS4P2,LOC441242*	.	.	.

13	51997478	GCA	G	intronic	*INTS6*	.	.	.
13	52057536	G	T	intergenic	*INTS6-AS1,MIR4703*	.	.	.
13	52085255	G	A	intergenic	*INTS6-AS1,MIR4703*	.	.	.
13	52085610	A	G	intergenic	*INTS6-AS1,MIR4703*	.	.	.
13	52087219	A	C	intergenic	*INTS6-AS1,MIR4703*	.	.	.
13	52101841	C	T	intergenic	*INTS6-AS1,MIR4703*	.	.	.
13	52101842	A	G	intergenic	*INTS6-AS1,MIR4703*	.	.	.
1	212136637	A	G	intronic	*INTS7*	.	.	.
1	212136638	C	T	intronic	*INTS7*	.	.	.
8	95886191	T	A	intronic	*INTS8*	.	.	.
8	28673874	A	G	intronic	*INTS9*	.	.	.
4	128572298	A	G	intronic	*INTU*	.	.	.
4	128572604	A	T	intronic	*INTU*	.	.	.
4	128600585	C	G	intronic	*INTU*	.	.	.
4	128603057	T	A	intronic	*INTU*	.	.	.
9	103010750	A	G	intronic	*INVS*	.	.	.
9	103017573	C	T	intronic	*INVS*	.	.	.
9	103018544	T	C	intronic	*INVS*	.	.	.
9	103018804	A	G	intronic	*INVS*	.	.	.
3	48751688	C	T	intronic	*IP6K2*	.	.	.
3	48751689	A	G	intronic	*IP6K2*	.	.	.
6	33724268	A	T	intergenic	*IP6K3,LEMD2*	.	.	.
6	154495582	C	T	intronic	*IPCEF1,OPRM1*	.	.	.
10	59991783	A	G	intronic	*IPMK*	.	.	.
10	60005429	T	C	intronic	*IPMK*	.	.	.
10	60006693	C	T	intronic	*IPMK*	.	.	.
5	61804679	G	A	intronic	*IPO11*	.	.	.
5	61842037	T	A	intronic	*IPO11*	.	.	.
5	62803570	G	GA	intergenic	*IPO11,HTR1A*	.	.	.
5	63088126	A	G	intergenic	*IPO11,HTR1A*	.	.	.
5	63000092	TACAC	T	intergenic	*IPO11,HTR1A*	.	.	.
5	62489912	G	T	intergenic	*IPO11,HTR1A*	.	.	.

5	62103958	T	C	intergenic	*IPO11,HTR1A*	.	.	.
5	62458280	T	C	intergenic	*IPO11,HTR1A*	.	.	.
5	62880299	T	A	intergenic	*IPO11,HTR1A*	.	.	.
5	62806894	G	A	intergenic	*IPO11,HTR1A*	.	.	.
5	61954046	A	G	intergenic	*IPO11,HTR1A*	.	.	.
5	61972097	T	G	intergenic	*IPO11,HTR1A*	.	.	.
5	61972286	G	A	intergenic	*IPO11,HTR1A*	.	.	.
5	62051846	T	C	intergenic	*IPO11,HTR1A*	.	.	.
5	62053547	C	A	intergenic	*IPO11,HTR1A*	.	.	.
5	62053971	C	T	intergenic	*IPO11,HTR1A*	.	.	.
5	62053972	C	A	intergenic	*IPO11,HTR1A*	.	.	.
5	62103687	T	A	intergenic	*IPO11,HTR1A*	.	.	.
5	62138726	G	A	intergenic	*IPO11,HTR1A*	.	.	.
5	62161129	C	A	intergenic	*IPO11,HTR1A*	.	.	.
5	62187386	C	T	intergenic	*IPO11,HTR1A*	.	.	.
5	62189828	C	T	intergenic	*IPO11,HTR1A*	.	.	.
5	62189829	A	G	intergenic	*IPO11,HTR1A*	.	.	.
5	62189864	T	C	intergenic	*IPO11,HTR1A*	.	.	.
5	62247249	A	G	intergenic	*IPO11,HTR1A*	.	.	.
5	62247250	C	T	intergenic	*IPO11,HTR1A*	.	.	.
5	62574010	A	T	intergenic	*IPO11,HTR1A*	.	.	.
5	62574612	G	T	intergenic	*IPO11,HTR1A*	.	.	.
5	62657668	A	T	intergenic	*IPO11,HTR1A*	.	.	.
5	62674312	G	A	intergenic	*IPO11,HTR1A*	.	.	.
5	62676388	A	T	intergenic	*IPO11,HTR1A*	.	.	.
5	62759804	A	AC	intergenic	*IPO11,HTR1A*	.	.	.
5	62759806	A	G	intergenic	*IPO11,HTR1A*	.	.	.
5	62785239	A	G	intergenic	*IPO11,HTR1A*	.	.	.
5	62822915	T	C	intergenic	*IPO11,HTR1A*	.	.	.
5	62856373	A	G	intergenic	*IPO11,HTR1A*	.	.	.
5	62856541	A	C	intergenic	*IPO11,HTR1A*	.	.	.
5	62874958	A	G	intergenic	*IPO11,HTR1A*	.	.	.
5	62896658	T	G	intergenic	*IPO11,HTR1A*	.	.	.
5	62924970	A	G	intergenic	*IPO11,HTR1A*	.	.	.
5	62973790	A	C	intergenic	*IPO11,HTR1A*	.	.	.
5	62975540	G	C	intergenic	*IPO11,HTR1A*	.	.	.
5	62976158	T	G	intergenic	*IPO11,HTR1A*	.	.	.
5	63238362	T	G	intergenic	*IPO11,HTR1A*	.	.	.

14	24653629	G	C	intronic	*IPO4*	.	.	.
13	98675199	C	A	UTR3	*IPO5*	.	.	.
1	201827105	A	G	intronic	*IPO9*	.	.	.
1	46207290	A	T	intronic	*IPP*	.	.	.
2	237352955	T	C	intronic	*IQCA1*	.	.	.
2	237296207	G	C	intronic	*IQCA1*	.	.	.
2	237330010	T	A	intronic	*IQCA1*	.	.	.
2	237330032	C	T	intronic	*IQCA1*	.	.	.
2	237331689	C	T	intronic	*IQCA1*	.	.	.
2	237352784	T	C	intronic	*IQCA1*	.	.	.
2	237436537	C	A	intergenic	*IQCA1,ACKR3*	.	.	.
12	113650019	C	T	intronic	*IQCD*	.	.	.
3	51823100	A	C	intergenic	*IQCF6,IQCF4*	.	.	.
15	67593968	G	A	intronic	*IQCH*	.	.	.
15	67753797	C	A	ncRNA_intronic	*IQCH-AS1*	.	.	.
3	159397302	C	A	intronic	*IQCJ-SCHIP1,SCHIP1*	.	.	.
3	159468085	T	TACACAC	intronic	*IQCJ-SCHIP1,SCHIP1*	.	.	.
3	159110738	C	T	intronic	*IQCJ-SCHIP1,SCHIP1*	.	.	.
3	159110739	C	T	intronic	*IQCJ-SCHIP1,SCHIP1*	.	.	.
5	75902986	T	A	intronic	*IQGAP2*	.	.	.
5	75731224	A	AGC	intronic	*IQGAP2*	.	.	.
5	75731225	TTC	T	intronic	*IQGAP2*	.	.	.
5	75750415	G	A	intronic	*IQGAP2*	.	.	.
5	75968803	T	C	intronic	*IQGAP2*	.	.	.
3	13333733	C	G	intergenic	*IQSEC1,NUP210*	.	.	.
7	123116941	A	G	intronic	*IQUB*	.	.	.
7	123117443	G	T	intronic	*IQUB*	.	.	.
7	123118030	C	CT	intronic	*IQUB*	.	.	.
7	123118282	A	C	intronic	*IQUB*	.	.	.
7	123165171	A	G	intronic	*IQUB*	.	.	.
7	123166544	G	A	intronic	*IQUB*	.	.	.
6	79599116	T	G	intronic	*IRAK1BP1*	.	.	.
6	79633782	G	A	intergenic	*IRAK1BP1,PHIP*	.	.	.
6	79634038	G	A	intergenic	*IRAK1BP1,PHIP*	.	.	.

4	185461564	T	C	intergenic	*IRF2,LVCAT8*	.	.	.
14	77493830	CGCGGCGGCGGCGGCGGCCGCCGCTGCTGCCGCCGCCGCCGCCGCTTCCTTAGCCGACAGGGCCACTGTCTTGACCCCGACGG	C	exonic	*IRF2BPL*	frameshift_deletion	.	.
6	434520	G	A	intergenic	*IRF4,EXOC2*	.	.	.
6	446953	C	T	intergenic	*IRF4,EXOC2*	.	.	.
6	481904	A	G	intergenic	*IRF4,EXOC2*	.	.	.
6	482310	G	A	intergenic	*IRF4,EXOC2*	.	.	.
5	150237383	C	A	intergenic	*IRGM,ZNF300*	.	.	.
5	150266473	A	T	intergenic	*IRGM,ZNF300*	.	.	.
13	110476195	G	T	intergenic	*IRS2,LINC00396*	.	.	.
13	110479196	G	C	intergenic	*IRS2,LINC00396*	.	.	.
5	3733034	G	T	intergenic	*IRX1,LOC101929153*	.	.	.
5	4052690	T	A	intergenic	*IRX1,LOC101929153*	.	.	.
5	4095307	CATAT	C	intergenic	*IRX1,LOC101929153*	.	.	.
5	4503167	A	T	intergenic	*IRX1,LOC101929153*	.	.	.
5	4764233	C	A	intergenic	*IRX1,LOC101929153*	.	.	.
5	4770251	G	A	intergenic	*IRX1,LOC101929153*	.	.	.
5	4366095	G	T	intergenic	*IRX1,LOC101929153*	.	.	.
5	4604924	A	G	intergenic	*IRX1,LOC101929153*	.	.	.

5	4675224	C	G	intergenic	*IRX1,LOC101929153*	.	.	.
5	3900306	C	T	intergenic	*IRX1,LOC101929153*	.	.	.
5	3928967	A	G	intergenic	*IRX1,LOC101929153*	.	.	.
5	4607771	C	T	intergenic	*IRX1,LOC101929153*	.	.	.
5	4607773	T	TC	intergenic	*IRX1,LOC101929153*	.	.	.
5	4620374	T	C	intergenic	*IRX1,LOC101929153*	.	.	.
5	4688696	G	A	intergenic	*IRX1,LOC101929153*	.	.	.
5	4688697	T	C	intergenic	*IRX1,LOC101929153*	.	.	.
5	4713673	A	T	intergenic	*IRX1,LOC101929153*	.	.	.
5	4713674	T	C	intergenic	*IRX1,LOC101929153*	.	.	.
5	4715037	G	A	intergenic	*IRX1,LOC101929153*	.	.	.
16	54340672	T	TCCTCACAC	intergenic	*IRX3,CRNDE*	.	.	.
16	54416402	A	T	intergenic	*IRX3,CRNDE*	.	.	.
16	54369807	A	T	intergenic	*IRX3,CRNDE*	.	.	.
16	54369809	A	T	intergenic	*IRX3,CRNDE*	.	.	.
16	54637004	T	G	intergenic	*IRX3,CRNDE*	.	.	.
16	54700926	A	C	intergenic	*IRX3,CRNDE*	.	.	.
16	55079689	T	A	intergenic	*IRX5,IRX6*	.	.	.
16	55257611	G	A	intergenic	*IRX5,IRX6*	.	.	.
16	55090316	C	A	intergenic	*IRX5,IRX6*	.	.	.
16	55350569	CT	C	intergenic	*IRX5,IRX6*	.	.	.
15	89295198	A	G	intergenic	*ISG20,ACAN*	.	.	.
5	50719222	A	C	intergenic	*ISL1,PELO*	.	.	.
5	50908750	C	T	intergenic	*ISL1,PELO*	.	.	.
5	50775510	T	TCGGTGGTC	intergenic	*ISL1,PELO*	.	.	.
5	51487881	A	G	intergenic	*ISL1,PELO*	.	.	.
5	51673175	T	A	intergenic	*ISL1,PELO*	.	.	.

5	50808440	A	C	intergenic	*ISL1,PELO*	.	.	.
5	50808442	G	A	intergenic	*ISL1,PELO*	.	.	.
5	50925857	T	A	intergenic	*ISL1,PELO*	.	.	.
5	50933544	G	A	intergenic	*ISL1,PELO*	.	.	.
5	50935789	C	A	intergenic	*ISL1,PELO*	.	.	.
5	51036078	G	A	intergenic	*ISL1,PELO*	.	.	.
5	51081224	C	T	intergenic	*ISL1,PELO*	.	.	.
5	51081429	T	C	intergenic	*ISL1,PELO*	.	.	.
5	51082199	T	G	intergenic	*ISL1,PELO*	.	.	.
5	51084497	A	G	intergenic	*ISL1,PELO*	.	.	.
5	51084832	T	C	intergenic	*ISL1,PELO*	.	.	.
5	51084833	G	T	intergenic	*ISL1,PELO*	.	.	.
5	51252065	C	G	intergenic	*ISL1,PELO*	.	.	.
5	51253036	A	G	intergenic	*ISL1,PELO*	.	.	.
5	51354844	T	C	intergenic	*ISL1,PELO*	.	.	.
5	51394062	T	C	intergenic	*ISL1,PELO*	.	.	.
5	51395692	C	T	intergenic	*ISL1,PELO*	.	.	.
5	51395693	A	G	intergenic	*ISL1,PELO*	.	.	.
5	51397580	C	T	intergenic	*ISL1,PELO*	.	.	.
5	51456072	A	T	intergenic	*ISL1,PELO*	.	.	.
5	51457177	C	T	intergenic	*ISL1,PELO*	.	.	.
5	51457982	G	A	intergenic	*ISL1,PELO*	.	.	.
5	51463631	G	A	intergenic	*ISL1,PELO*	.	.	.
5	51463918	C	A	intergenic	*ISL1,PELO*	.	.	.
5	51513110	A	AC	intergenic	*ISL1,PELO*	.	.	.
5	51546017	A	G	intergenic	*ISL1,PELO*	.	.	.
5	51576985	G	C	intergenic	*ISL1,PELO*	.	.	.
5	51610866	C	G	intergenic	*ISL1,PELO*	.	.	.
5	51633177	C	A	intergenic	*ISL1,PELO*	.	.	.
5	51675495	A	G	intergenic	*ISL1,PELO*	.	.	.
5	51832155	T	C	intergenic	*ISL1,PELO*	.	.	.
5	51834577	A	G	intergenic	*ISL1,PELO*	.	.	.
5	51906563	A	T	intergenic	*ISL1,PELO*	.	.	.
5	51909398	T	C	intergenic	*ISL1,PELO*	.	.	.
5	51909399	G	T	intergenic	*ISL1,PELO*	.	.	.
5	51927861	A	T	intergenic	*ISL1,PELO*	.	.	.
5	52019013	G	A	intergenic	*ISL1,PELO*	.	.	.
20	13307397	T	C	intergenic	*ISM1,TASP1*	.	.	.

20	13308159	A	C	intergenic	*ISM1,TASP1*	.	.	.
20	13359344	C	G	intergenic	*ISM1,TASP1*	.	.	.
5	128470176	C	G	intergenic	*ISOC1,MIR4460*	.	.	.
5	128499085	G	A	intergenic	*ISOC1,MIR4460*	.	.	.
5	128495654	A	G	intergenic	*ISOC1,MIR4460*	.	.	.
5	128486582	T	C	intergenic	*ISOC1,MIR4460*	.	.	.
5	128491574	A	G	intergenic	*ISOC1,MIR4460*	.	.	.
5	128516842	G	A	intergenic	*ISOC1,MIR4460*	.	.	.
5	128544029	C	G	intergenic	*ISOC1,MIR4460*	.	.	.
5	128544030	A	G	intergenic	*ISOC1,MIR4460*	.	.	.
5	128546177	T	C	intergenic	*ISOC1,MIR4460*	.	.	.
5	128547044	T	C	intergenic	*ISOC1,MIR4460*	.	.	.
5	128621919	C	T	intergenic	*ISOC1,MIR4460*	.	.	.
5	128625016	C	T	intergenic	*ISOC1,MIR4460*	.	.	.
5	128651162	A	C	intergenic	*ISOC1,MIR4460*	.	.	.
7	16141991	T	C	intronic	*ISPD*	.	.	.
7	16141992	G	T	intronic	*ISPD*	.	.	.
7	16142565	G	C	intronic	*ISPD*	.	.	.
7	16201824	C	T	intronic	*ISPD*	.	.	.
7	16223728	C	G	intronic	*ISPD*	.	.	.
7	16390968	C	G	intronic	*ISPD*	.	.	.
7	16260987	C	T	ncRNA_intronic	*ISPD-AS1*	.	.	.
7	16295148	G	A	ncRNA_intronic	*ISPD-AS1*	.	.	.
7	16295480	A	G	ncRNA_intronic	*ISPD-AS1*	.	.	.
7	16492299	T	A	intergenic	*ISPD,SOSTDC1*	.	.	.
16	47245345	C	T	intronic	*ITFG1*	.	.	.
16	47398959	A	G	intronic	*ITFG1*	.	.	.
16	47385984	G	T	intronic	*ITFG1*	.	.	.
16	47358501	T	C	intronic	*ITFG1*	.	.	.
16	47457088	G	A	intronic	*ITFG1*	.	.	.
5	52265603	T	G	intergenic	*ITGA1,ITGA2*	.	.	.
5	52269273	A	G	intergenic	*ITGA1,ITGA2*	.	.	.
15	68791035	G	T	intergenic	*ITGA11,CORO2B*	.	.	.
5	52366741	G	A	intronic	*ITGA2*	.	.	.
2	182334447	A	G	intronic	*ITGA4*	.	.	.
2	173340648	AAAGC	A	intronic	*ITGA6*	.	.	.
2	173393112	C	T	intergenic	*ITGA6,PDK1*	.	.	.
10	15740849	G	A	intronic	*ITGA8*	.	.	.

3	37577492	T	C	intronic	*ITGA9*	.	.	.
3	37724751	G	T	intronic	*ITGA9*	.	.	.
3	37739224	T	C	intronic	*ITGA9*	.	.	.
3	37901032	G	T	ncRNA_intronic	*ITGA9-AS1*	.	.	.
3	37883161	A	G	ncRNA_intronic	*ITGA9-AS1*	.	.	.
3	37884607	A	G	ncRNA_intronic	*ITGA9-AS1*	.	.	.
3	37885513	G	T	ncRNA_intronic	*ITGA9-AS1*	.	.	.
16	30527739	C	T	intronic	*ITGAL*	.	.	.
16	30529559	G	A	intronic	*ITGAL*	.	.	.
16	31316376	T	C	intronic	*ITGAM*	.	.	.
16	31319775	C	T	intronic	*ITGAM*	.	.	.
16	31325562	T	C	intronic	*ITGAM*	.	.	.
16	31351134	A	G	intergenic	*ITGAM,ITGAX*	.	.	.
10	33197756	C	A	intronic	*ITGB1*	.	.	.
10	33263119	T	C	intergenic	*ITGB1,NRP1*	.	.	.
1	63971204	G	T	intronic	*ITGB3BP*	.	.	.
3	124495078	T	C	intronic	*ITGB5*	.	.	.
2	160992483	G	A	intronic	*ITGB6*	.	.	.
2	161007321	T	C	intronic	*ITGB6*	.	.	.
2	161007870	A	G	intronic	*ITGB6*	.	.	.
2	161042954	A	G	intronic	*ITGB6*	.	.	.
7	20399299	A	AGTGT	intronic	*ITGB8*	.	.	.
7	20464727	G	A	intergenic	*ITGB8,ABCB5*	.	.	.
7	20594737	T	G	intergenic	*ITGB8,ABCB5*	.	.	.
7	20608069	C	T	intergenic	*ITGB8,ABCB5*	.	.	.
7	20608070	A	G	intergenic	*ITGB8,ABCB5*	.	.	.
7	20611274	C	A	intergenic	*ITGB8,ABCB5*	.	.	.
7	20644649	G	A	intergenic	*ITGB8,ABCB5*	.	.	.
10	7777907	G	A	intronic	*ITIH2*	.	.	.
10	7781914	C	T	intronic	*ITIH2*	.	.	.
X	54790738	T	A	intronic	*ITIH6*	.	.	.
X	54819717	A	G	intronic	*ITIH6*	.	.	.
1	160867771	T	G	intergenic	*ITLN1,LOC101928372*	.	.	.
1	160868990	A	G	intergenic	*ITLN1,LOC101928372*	.	.	.
1	160869287	G	A	intergenic	*ITLN1,LOC101928372*	.	.	.

X	78635977	C	A	intergenic	*ITM2A,TBX22*	.	.	.
X	79096626	G	T	intergenic	*ITM2A,TBX22*	.	.	.
X	78995572	T	C	intergenic	*ITM2A,TBX22*	.	.	.
X	79165205	T	G	intergenic	*ITM2A,TBX22*	.	.	.
X	78644755	C	T	intergenic	*ITM2A,TBX22*	.	.	.
X	78757801	T	A	intergenic	*ITM2A,TBX22*	.	.	.
X	78645143	C	A	intergenic	*ITM2A,TBX22*	.	.	.
X	78673287	C	A	intergenic	*ITM2A,TBX22*	.	.	.
X	78754595	T	C	intergenic	*ITM2A,TBX22*	.	.	.
X	78764162	T	C	intergenic	*ITM2A,TBX22*	.	.	.
X	78906541	G	T	intergenic	*ITM2A,TBX22*	.	.	.
X	78909102	G	T	intergenic	*ITM2A,TBX22*	.	.	.
X	78927584	T	C	intergenic	*ITM2A,TBX22*	.	.	.
X	78935857	A	G	intergenic	*ITM2A,TBX22*	.	.	.
X	78935858	T	G	intergenic	*ITM2A,TBX22*	.	.	.
X	78947892	T	C	intergenic	*ITM2A,TBX22*	.	.	.
X	78951051	T	G	intergenic	*ITM2A,TBX22*	.	.	.
X	78964278	C	A	intergenic	*ITM2A,TBX22*	.	.	.
X	79095976	G	C	intergenic	*ITM2A,TBX22*	.	.	.
X	79151270	T	G	intergenic	*ITM2A,TBX22*	.	.	.
X	79157581	T	C	intergenic	*ITM2A,TBX22*	.	.	.
X	79159160	G	A	intergenic	*ITM2A,TBX22*	.	.	.
X	79240796	T	A	intergenic	*ITM2A,TBX22*	.	.	.
X	79256624	A	G	intergenic	*ITM2A,TBX22*	.	.	.
1	226843086	G	A	intronic	*ITPKB*	.	.	.
1	226857912	T	A	ncRNA_intronic	*ITPKB-IT1*	.	.	.
1	227041020	A	T	intergenic	*ITPKB,PSEN2*	.	.	.
1	227042409	G	T	intergenic	*ITPKB,PSEN2*	.	.	.
3	4762791	GATAT	G	intronic	*ITPR1*	.	.	.
3	4767840	G	A	intronic	*ITPR1*	.	.	.
12	26782677	TTCTC	T	intronic	*ITPR2*	.	.	.
12	26766496	GACACAC	G	intronic	*ITPR2*	.	.	.
12	27055939	T	C	intergenic	*ITPR2,ASUN*	.	.	.
2	24432673	C	T	intronic	*ITSN2*	.	.	.
2	24755043	C	T	intergenic	*ITSN2,NCOA1*	.	.	.
2	24756454	C	G	intergenic	*ITSN2,NCOA1*	.	.	.
15	40705396	C	T	intronic	*IVD*	.	.	.
6	150783225	G	A	intergenic	*IYD,PLEKHG1*	.	.	.

6	150796337	C	A	intergenic	*IYD,PLEKHG1*	.	.	.
6	150797021	C	T	intergenic	*IYD,PLEKHG1*	.	.	.
6	150797149	T	C	intergenic	*IYD,PLEKHG1*	.	.	.
11	94093580	A	G	intergenic	*IZUMO1R,GPR83*	.	.	.
11	94097014	A	G	intergenic	*IZUMO1R,GPR83*	.	.	.
11	94097015	A	T	intergenic	*IZUMO1R,GPR83*	.	.	.
11	94104954	T	A	intergenic	*IZUMO1R,GPR83*	.	.	.
11	94107659	T	C	intergenic	*IZUMO1R,GPR83*	.	.	.
11	94107807	T	C	intergenic	*IZUMO1R,GPR83*	.	.	.
9	24627066	A	G	intergenic	*IZUMO3,TUSC1*	.	.	.
9	24718145	G	T	intergenic	*IZUMO3,TUSC1*	.	.	.
9	24761919	AT	A	intergenic	*IZUMO3,TUSC1*	.	.	.
9	25514480	T	TGACTCTAC	intergenic	*IZUMO3,TUSC1*	.	.	.
9	24596003	A	G	intergenic	*IZUMO3,TUSC1*	.	.	.
9	24619226	G	A	intergenic	*IZUMO3,TUSC1*	.	.	.
9	25514480	T	TGACTCTAC	intergenic	*IZUMO3,TUSC1*	.	.	.
9	25152075	C	T	intergenic	*IZUMO3,TUSC1*	.	.	.
9	24635159	G	A	intergenic	*IZUMO3,TUSC1*	.	.	.
9	24635789	C	G	intergenic	*IZUMO3,TUSC1*	.	.	.
9	24635790	A	G	intergenic	*IZUMO3,TUSC1*	.	.	.
9	24752258	A	G	intergenic	*IZUMO3,TUSC1*	.	.	.
9	24752259	C	T	intergenic	*IZUMO3,TUSC1*	.	.	.
9	24792217	A	T	intergenic	*IZUMO3,TUSC1*	.	.	.
9	25106750	A	G	intergenic	*IZUMO3,TUSC1*	.	.	.
9	25107172	G	T	intergenic	*IZUMO3,TUSC1*	.	.	.
9	25229327	C	T	intergenic	*IZUMO3,TUSC1*	.	.	.
9	25230835	A	T	intergenic	*IZUMO3,TUSC1*	.	.	.
9	25318806	T	A	intergenic	*IZUMO3,TUSC1*	.	.	.
9	25609212	A	C	intergenic	*IZUMO3,TUSC1*	.	.	.
5	133883000	T	G	intronic	*JADE2*	.	.	.
X	46779271	TAA	T	intronic	*JADE3*	.	.	.
20	10627321	G	A	intronic	*JAG1*	.	.	.
20	10684830	A	G	intergenic	*JAG1,LOC101929395*	.	.	.
9	5042703	G	A	intronic	*JAK2*	.	.	.
9	5103439	T	C	intronic	*JAK2*	.	.	.
9	5137735	T	A	intergenic	*JAK2,INSL6*	.	.	.
9	5153763	G	A	intergenic	*JAK2,INSL6*	.	.	.

4	6109425	C	A	intronic	*JAKMIP1*	.	.	.
4	6172903	A	C	intronic	*JAKMIP1*	.	.	.
5	147072141	C	T	intronic	*JAKMIP2*	.	.	.
5	147098142	A	G	intronic	*JAKMIP2*	.	.	.
5	147099878	A	C	intronic	*JAKMIP2*	.	.	.
5	146986962	A	G	ncRNA_intronic	*JAKMIP2-AS1*	.	.	.
5	147033635	A	C	ncRNA_intronic	*JAKMIP2-AS1*	.	.	.
10	133968455	GTGATAATGGTTATCATGATGGTGATCATGATGGGGATGATGGTGATTATGATGATGA	G	intronic	*JAKMIP3*	.	.	.
11	133974251	T	C	intronic	*JAM3*	.	.	.
11	133975468	T	C	intronic	*JAM3*	.	.	.
11	133975717	G	C	intronic	*JAM3*	.	.	.
11	134022027	A	G	downstream	*JAM3,NCAPD3*	.	.	.
7	28050095	C	T	intronic	*JAZF1*	.	.	.
7	27893320	A	AT	intronic	*JAZF1*	.	.	.
7	27999518	CAAAAAA	C	intronic	*JAZF1*	.	.	.
7	28023784	T	A	intronic	*JAZF1*	.	.	.
7	28315949	A	G	intergenic	*JAZF1-AS1,CREB5*	.	.	.
7	28295524	A	G	intergenic	*JAZF1-AS1,CREB5*	.	.	.
7	28323671	G	A	intergenic	*JAZF1-AS1,CREB5*	.	.	.
4	71531113	A	ATATTCAG	intronic	*JCHAIN*	.	.	.
14	75983323	T	C	intergenic	*JDP2,BATF*	.	.	.
7	140022886	G	C	intergenic	*JHDM1D-AS1,SLC37A3*	.	.	.
10	64966540	T	C	exonic	*JMJD1C*	nonsynonymous_SNV	.	.
1	227922129	G	T	intronic	*JMJD4*	.	.	.
5	78544943	G	T	intronic	*JMY*	.	.	.
5	78632542	A	G	intergenic	*JMY,HOMER1*	.	.	.
8	75228601	A	G	intronic	*JPH1*	.	.	.
8	75165936	C	T	intronic	*JPH1*	.	.	.
8	75168947	G	A	intronic	*JPH1*	.	.	.

8	75168948	T	C	intronic	*JPH1*	.	.	.
8	75203236	C	A	intronic	*JPH1*	.	.	.
X	73200395	G	A	ncRNA_intronic	*JPX*	.	.	.
11	96224229	G	A	ncRNA_intronic	*JRKL-AS1*	.	.	.
11	98299328	C	A	intergenic	*JRKL-AS1,CNTN5*	.	.	.
11	96990477	G	C	intergenic	*JRKL-AS1,CNTN5*	.	.	.
11	97334857	A	C	intergenic	*JRKL-AS1,CNTN5*	.	.	.
11	97151413	A	G	intergenic	*JRKL-AS1,CNTN5*	.	.	.
11	97322936	TA	T	intergenic	*JRKL-AS1,CNTN5*	.	.	.
11	97357224	C	A	intergenic	*JRKL-AS1,CNTN5*	.	.	.
11	97417335	G	A	intergenic	*JRKL-AS1,CNTN5*	.	.	.
11	97425388	T	G	intergenic	*JRKL-AS1,CNTN5*	.	.	.
11	97518870	A	C	intergenic	*JRKL-AS1,CNTN5*	.	.	.
11	97788970	G	A	intergenic	*JRKL-AS1,CNTN5*	.	.	.
11	97890690	T	C	intergenic	*JRKL-AS1,CNTN5*	.	.	.
11	98283273	CTGTG	C	intergenic	*JRKL-AS1,CNTN5*	.	.	.
11	96926223	T	C	intergenic	*JRKL-AS1,CNTN5*	.	.	.
11	96974091	A	G	intergenic	*JRKL-AS1,CNTN5*	.	.	.
11	96977299	T	A	intergenic	*JRKL-AS1,CNTN5*	.	.	.
11	97065502	A	G	intergenic	*JRKL-AS1,CNTN5*	.	.	.
11	97066922	C	A	intergenic	*JRKL-AS1,CNTN5*	.	.	.
11	97097486	G	A	intergenic	*JRKL-AS1,CNTN5*	.	.	.
11	97173447	C	G	intergenic	*JRKL-AS1,CNTN5*	.	.	.
11	97173448	A	G	intergenic	*JRKL-AS1,CNTN5*	.	.	.
11	97311235	C	G	intergenic	*JRKL-AS1,CNTN5*	.	.	.
11	97389766	C	A	intergenic	*JRKL-AS1,CNTN5*	.	.	.
11	97410743	C	T	intergenic	*JRKL-AS1,CNTN5*	.	.	.
11	97411476	A	T	intergenic	*JRKL-AS1,CNTN5*	.	.	.
11	97511369	G	A	intergenic	*JRKL-AS1,CNTN5*	.	.	.
11	97581928	A	G	intergenic	*JRKL-AS1,CNTN5*	.	.	.
11	97607769	G	A	intergenic	*JRKL-AS1,CNTN5*	.	.	.
11	97612632	T	A	intergenic	*JRKL-AS1,CNTN5*	.	.	.
11	97795442	A	T	intergenic	*JRKL-AS1,CNTN5*	.	.	.
11	97795612	A	G	intergenic	*JRKL-AS1,CNTN5*	.	.	.
11	97797286	A	C	intergenic	*JRKL-AS1,CNTN5*	.	.	.
11	97885797	A	G	intergenic	*JRKL-AS1,CNTN5*	.	.	.
11	97887505	T	A	intergenic	*JRKL-AS1,CNTN5*	.	.	.
11	97888013	T	C	intergenic	*JRKL-AS1,CNTN5*	.	.	.

11	97965279	A	G	intergenic	*JRKL-AS1,CNTN5*	.	.	.
11	98048006	A	G	intergenic	*JRKL-AS1,CNTN5*	.	.	.
11	98200660	C	T	intergenic	*JRKL-AS1,CNTN5*	.	.	.
11	98202648	G	A	intergenic	*JRKL-AS1,CNTN5*	.	.	.
11	96281283	G	A	intergenic	*JRKL-AS1,NONE*	.	.	.
11	96484428	A	G	intergenic	*JRKL-AS1,NONE*	.	.	.
11	96594595	C	T	intergenic	*JRKL-AS1,NONE*	.	.	.
11	96684479	C	G	intergenic	*JRKL-AS1,NONE*	.	.	.
11	96781774	T	C	intergenic	*JRKL-AS1,NONE*	.	.	.
11	96781983	T	C	intergenic	*JRKL-AS1,NONE*	.	.	.
11	96782495	A	G	intergenic	*JRKL-AS1,NONE*	.	.	.
11	96782497	C	G	intergenic	*JRKL-AS1,NONE*	.	.	.
11	96782765	A	G	intergenic	*JRKL-AS1,NONE*	.	.	.
11	96784170	G	A	intergenic	*JRKL-AS1,NONE*	.	.	.
11	96140347	A	G	intergenic	*JRKL,JRKL-AS1*	.	.	.
3	124033441	T	A	intronic	*KALRN*	.	.	.
9	573280	ATTT	A	intronic	*KANK1*	.	.	.
9	545967	C	T	intronic	*KANK1*	.	.	.
9	772023	G	A	intergenic	*KANK1,DMRT1*	.	.	.
1	62898655	T	C	intergenic	*KANK4,USP1*	.	.	.
17	44365416	T	C	intergenic	*KANSL1,LRRC37A*	.	.	.
2	210967261	G	A	intronic	*KANSL1L*	.	.	.
2	210967856	T	C	intronic	*KANSL1L*	.	.	.
2	210976533	C	T	intronic	*KANSL1L*	.	.	.
2	211049452	A	C	intergenic	*KANSL1L,ACADL*	.	.	.
2	211050132	C	T	intergenic	*KANSL1L,ACADL*	.	.	.
2	97279009	G	A	intronic	*KANSL3*	.	.	.
2	97303181	C	T	intronic	*KANSL3*	.	.	.
X	53141272	A	G	ncRNA_intronic	*KANTR*	.	.	.
X	53176796	T	G	intergenic	*KANTR,KDM5C*	.	.	.
X	53176849	C	T	intergenic	*KANTR,KDM5C*	.	.	.
X	53177115	T	C	intergenic	*KANTR,KDM5C*	.	.	.
8	41844589	A	G	intronic	*KAT6A*	.	.	.
10	76720091	T	C	intronic	*KAT6B*	.	.	.
6	149924014	C	T	intronic	*KATNA1*	.	.	.
13	30820009	G	A	intronic	*KATNAL1*	.	.	.
15	34463278	A	T	intronic	*KATNBL1*	.	.	.
15	34463864	G	A	intronic	*KATNBL1*	.	.	.

15	34476918	C	T	intronic	*KATNBL1*	.	.	.
8	1935956	G	T	intronic	*KBTBD11*	.	.	.
3	127657614	A	C	intronic	*KBTBD12*	.	.	.
11	105922623	T	C	UTR3	*KBTBD3*	.	.	.
3	67102370	T	TTTCATTTTT	intergenic	*KBTBD8,MIR4272*	.	.	.
3	67188231	A	C	intergenic	*KBTBD8,MIR4272*	.	.	.
3	67188233	A	G	intergenic	*KBTBD8,MIR4272*	.	.	.
3	67189389	A	G	intergenic	*KBTBD8,MIR4272*	.	.	.
3	67191079	G	C	intergenic	*KBTBD8,MIR4272*	.	.	.
3	67210601	T	C	intergenic	*KBTBD8,MIR4272*	.	.	.
18	39170002	A	G	intergenic	*KC6,PIK3C3*	.	.	.
18	39353442	T	C	intergenic	*KC6,PIK3C3*	.	.	.
18	39232453	T	G	intergenic	*KC6,PIK3C3*	.	.	.
18	39380544	A	C	intergenic	*KC6,PIK3C3*	.	.	.
3	177926365	C	T	intergenic	*KCCAT211,LINC01014*	.	.	.
7	17444297	C	T	ncRNA_intronic	*KCCAT333*	.	.	.
12	5084146	T	C	intergenic	*KCNA1,KCNA5*	.	.	.
12	5091957	T	C	intergenic	*KCNA1,KCNA5*	.	.	.
1	111364597	A	G	intergenic	*KCNA3,CD53*	.	.	.
1	111364599	A	G	intergenic	*KCNA3,CD53*	.	.	.
1	111364642	T	G	intergenic	*KCNA3,CD53*	.	.	.
11	30083225	GA	G	intergenic	*KCNA4,FSHB*	.	.	.
11	30113422	T	C	intergenic	*KCNA4,FSHB*	.	.	.
11	30119240	A	C	intergenic	*KCNA4,FSHB*	.	.	.
11	30124554	G	A	intergenic	*KCNA4,FSHB*	.	.	.
11	30202448	G	T	intergenic	*KCNA4,FSHB*	.	.	.
11	30202449	G	A	intergenic	*KCNA4,FSHB*	.	.	.
11	30203044	T	C	intergenic	*KCNA4,FSHB*	.	.	.
11	30205143	T	G	intergenic	*KCNA4,FSHB*	.	.	.
12	5221089	C	G	intergenic	*KCNA5,LOC101929584*	.	.	.
12	5284823	C	T	intergenic	*KCNA5,LOC101929584*	.	.	.
3	156125424	C	A	intronic	*KCNAB1*	.	.	.
1	6090987	GGTGTGT	G	intronic	*KCNAB2*	.	.	.
20	48086738	A	G	intronic	*KCNB1*	.	.	.
8	73460677	C	T	intronic	*KCNB2*	.	.	.

8	73703031	A	G	intronic	*KCNB2*	.	.	.
8	73676338	G	A	intronic	*KCNB2*	.	.	.
8	73451534	A	G	intronic	*KCNB2*	.	.	.
8	73510842	C	T	intronic	*KCNB2*	.	.	.
8	73511985	T	G	intronic	*KCNB2*	.	.	.
8	73527548	G	A	intronic	*KCNB2*	.	.	.
8	73688101	G	A	intronic	*KCNB2*	.	.	.
8	73753391	G	A	intronic	*KCNB2*	.	.	.
8	73843692	G	A	intronic	*KCNB2*	.	.	.
8	73910780	C	T	intergenic	*KCNB2,TERF1*	.	.	.
12	75581640	A	T	intronic	*KCNC2*	.	.	.
12	75585386	A	T	intronic	*KCNC2*	.	.	.
12	75640956	G	A	intergenic	*KCNC2,CAPS2*	.	.	.
7	120115222	G	T	intronic	*KCND2*	.	.	.
7	120382330	G	T	intronic	*KCND2*	.	.	.
7	120174241	G	C	intronic	*KCND2*	.	.	.
7	119972651	C	T	intronic	*KCND2*	.	.	.
7	120405349	G	C	intergenic	*KCND2,TSPAN12*	.	.	.
7	120396020	G	A	intergenic	*KCND2,TSPAN12*	.	.	.
2	223994103	T	G	intergenic	*KCNE4,SCG2*	.	.	.
2	224457086	T	G	intergenic	*KCNE4,SCG2*	.	.	.
2	224457087	T	C	intergenic	*KCNE4,SCG2*	.	.	.
2	223931095	G	A	intergenic	*KCNE4,SCG2*	.	.	.
2	223999338	T	A	intergenic	*KCNE4,SCG2*	.	.	.
2	224043039	C	T	intergenic	*KCNE4,SCG2*	.	.	.
2	224154234	T	C	intergenic	*KCNE4,SCG2*	.	.	.
2	224197583	C	G	intergenic	*KCNE4,SCG2*	.	.	.
2	224197585	T	C	intergenic	*KCNE4,SCG2*	.	.	.
2	224198964	G	A	intergenic	*KCNE4,SCG2*	.	.	.
2	224280544	G	A	intergenic	*KCNE4,SCG2*	.	.	.
2	224435079	AAGAAAG	A	intergenic	*KCNE4,SCG2*	.	.	.

2	11232184	TATATAACATGTTATATATTATATAATATAGTATAATATATATGTTATATATTATATAATATAGTATAATATATA	T	intergenic	*KCNF1,FLJ33534*	.	.	.
20	49672132	G	A	intergenic	*KCNG1,NFATC2*	.	.	.
20	49885784	A	G	intergenic	*KCNG1,NFATC2*	.	.	.
2	42677178	G	T	intronic	*KCNG3*	.	.	.
1	211265073	TC	T	intronic	*KCNH1*	.	.	.
1	211012168	A	G	intronic	*KCNH1*	.	.	.
1	211018285	T	C	intronic	*KCNH1*	.	.	.
1	211044747	A	G	intronic	*KCNH1*	.	.	.
1	211044748	C	G	intronic	*KCNH1*	.	.	.
1	211061719	G	C	intronic	*KCNH1*	.	.	.
1	211061720	G	A	intronic	*KCNH1*	.	.	.
1	211128827	T	C	intronic	*KCNH1*	.	.	.
1	211129151	T	C	intronic	*KCNH1*	.	.	.
1	211129152	G	A	intronic	*KCNH1*	.	.	.
1	211142085	T	A	intronic	*KCNH1*	.	.	.
1	211143888	T	C	intronic	*KCNH1*	.	.	.
1	211158331	C	A	intronic	*KCNH1*	.	.	.
1	211158333	T	C	intronic	*KCNH1*	.	.	.
1	211174334	T	C	intronic	*KCNH1*	.	.	.
1	211174460	A	C	intronic	*KCNH1*	.	.	.
1	211175004	A	G	intronic	*KCNH1*	.	.	.
1	211202537	T	G	intronic	*KCNH1*	.	.	.
1	211206127	A	G	intronic	*KCNH1*	.	.	.
1	211217776	C	T	intronic	*KCNH1*	.	.	.
1	211252296	T	A	intronic	*KCNH1*	.	.	.
1	211254068	T	C	intronic	*KCNH1*	.	.	.
1	211295494	G	A	intronic	*KCNH1*	.	.	.
1	211355586	A	C	intergenic	*KCNH1,RCOR3*	.	.	.
17	40316619	G	A	intronic	*KCNH4*	.	.	.

14	63458154	G	C	intronic	*KCNH5*	.	.	.
14	63657271	A	G	intergenic	*KCNH5,RHOJ*	.	.	.
14	63606746	C	T	intergenic	*KCNH5,RHOJ*	.	.	.
14	63609761	C	T	intergenic	*KCNH5,RHOJ*	.	.	.
2	163393215	T	G	intronic	*KCNH7*	.	.	.
2	163310389	G	C	intronic	*KCNH7*	.	.	.
2	163310391	G	A	intronic	*KCNH7*	.	.	.
2	163310705	T	C	intronic	*KCNH7*	.	.	.
2	163311827	C	T	intronic	*KCNH7*	.	.	.
2	163311828	C	G	intronic	*KCNH7*	.	.	.
2	163312080	G	A	intronic	*KCNH7*	.	.	.
2	163312762	T	TG	intronic	*KCNH7*	.	.	.
2	163312764	A	G	intronic	*KCNH7*	.	.	.
2	163329670	C	T	intronic	*KCNH7*	.	.	.
2	163427661	T	C	intronic	*KCNH7*	.	.	.
2	163429541	T	A	intronic	*KCNH7*	.	.	.
2	163517642	C	T	intronic	*KCNH7*	.	.	.
2	163541160	G	A	intronic	*KCNH7*	.	.	.
2	163869411	C	G	intergenic	*KCNH7,FIGN*	.	.	.
2	164050460	A	C	intergenic	*KCNH7,FIGN*	.	.	.
2	164129918	T	C	intergenic	*KCNH7,FIGN*	.	.	.
2	164238168	C	T	intergenic	*KCNH7,FIGN*	.	.	.
2	164159333	A	T	intergenic	*KCNH7,FIGN*	.	.	.
2	164273715	T	C	intergenic	*KCNH7,FIGN*	.	.	.
2	163724683	A	G	intergenic	*KCNH7,FIGN*	.	.	.
2	163824318	TTGTGTG	T	intergenic	*KCNH7,FIGN*	.	.	.
2	164253991	A	G	intergenic	*KCNH7,FIGN*	.	.	.
2	164288747	T	C	intergenic	*KCNH7,FIGN*	.	.	.
2	163711945	C	T	intergenic	*KCNH7,FIGN*	.	.	.
2	163844245	C	G	intergenic	*KCNH7,FIGN*	.	.	.
2	164127711	A	C	intergenic	*KCNH7,FIGN*	.	.	.
2	164140433	C	G	intergenic	*KCNH7,FIGN*	.	.	.
2	164359824	T	A	intergenic	*KCNH7,FIGN*	.	.	.
3	19316576	T	C	intronic	*KCNH8*	.	.	.
3	19318076	G	T	intronic	*KCNH8*	.	.	.
3	19334601	G	T	intronic	*KCNH8*	.	.	.
3	19350719	A	T	intronic	*KCNH8*	.	.	.
3	19400922	C	A	intronic	*KCNH8*	.	.	.

3	19570455	T	G	intronic	*KCNH8*	.	.	.
3	19303546	C	CT	intronic	*KCNH8*	.	.	.
3	19305959	G	A	intronic	*KCNH8*	.	.	.
3	19364670	G	A	intronic	*KCNH8*	.	.	.
3	19421581	T	A	intronic	*KCNH8*	.	.	.
3	19332803	T	C	intronic	*KCNH8*	.	.	.
3	19394176	G	A	intronic	*KCNH8*	.	.	.
3	19394981	G	A	intronic	*KCNH8*	.	.	.
3	19711083	C	G	intergenic	*KCNH8,EFHB*	.	.	.
3	19678534	G	T	intergenic	*KCNH8,EFHB*	.	.	.
3	19692156	G	C	intergenic	*KCNH8,EFHB*	.	.	.
3	19794622	G	T	intergenic	*KCNH8,EFHB*	.	.	.
3	19884651	A	G	intergenic	*KCNH8,EFHB*	.	.	.
5	170141363	C	G	intronic	*KCNIP1*	.	.	.
5	170064908	G	A	intronic	*KCNIP1*	.	.	.
2	96042082	C	T	intronic	*KCNIP3*	.	.	.
4	21465581	G	T	intronic	*KCNIP4*	.	.	.
4	20924170	G	GTCTA	intronic	*KCNIP4*	.	.	.
4	21335135	T	C	intronic	*KCNIP4*	.	.	.
4	21517512	A	G	intronic	*KCNIP4*	.	.	.
4	21595857	A	C	intronic	*KCNIP4*	.	.	.
4	21012219	C	A	intronic	*KCNIP4*	.	.	.
4	21298725	GTATA	G	intronic	*KCNIP4*	.	.	.
4	21464151	T	C	intronic	*KCNIP4*	.	.	.
4	21890569	C	T	intronic	*KCNIP4*	.	.	.
4	21107392	C	A	intronic	*KCNIP4*	.	.	.
4	20744254	C	A	intronic	*KCNIP4*	.	.	.
4	20744255	T	G	intronic	*KCNIP4*	.	.	.
4	20745619	C	A	intronic	*KCNIP4*	.	.	.
4	20745620	C	A	intronic	*KCNIP4*	.	.	.
4	20746283	A	C	intronic	*KCNIP4*	.	.	.
4	20970160	T	A	intronic	*KCNIP4*	.	.	.
4	21073296	G	A	intronic	*KCNIP4*	.	.	.
4	21105891	A	T	intronic	*KCNIP4*	.	.	.
4	21201043	A	T	intronic	*KCNIP4*	.	.	.
4	21201495	G	T	intronic	*KCNIP4*	.	.	.
4	21240584	T	C	intronic	*KCNIP4*	.	.	.
4	21356708	T	G	intronic	*KCNIP4*	.	.	.

4	21392325	A	G	intronic	*KCNIP4*	.	.	.
4	21446180	T	C	intronic	*KCNIP4*	.	.	.
4	21448178	T	C	intronic	*KCNIP4*	.	.	.
4	21620225	A	T	intronic	*KCNIP4*	.	.	.
4	21670742	G	C	intronic	*KCNIP4*	.	.	.
4	21671967	C	T	intronic	*KCNIP4*	.	.	.
4	21772480	A	G	intronic	*KCNIP4*	.	.	.
4	21772481	G	A	intronic	*KCNIP4*	.	.	.
4	21773393	A	T	intronic	*KCNIP4*	.	.	.
4	21773410	A	G	intronic	*KCNIP4*	.	.	.
4	22149147	G	T	intergenic	*KCNIP4,LOC100505912*	.	.	.
4	22163506	A	G	intergenic	*KCNIP4,LOC100505912*	.	.	.
4	22196960	G	C	intergenic	*KCNIP4,LOC100505912*	.	.	.
4	22000735	T	A	intergenic	*KCNIP4,LOC100505912*	.	.	.
4	22001347	G	A	intergenic	*KCNIP4,LOC100505912*	.	.	.
4	22109376	T	C	intergenic	*KCNIP4,LOC100505912*	.	.	.
4	22127238	G	A	intergenic	*KCNIP4,LOC100505912*	.	.	.
4	22129024	A	T	intergenic	*KCNIP4,LOC100505912*	.	.	.
4	22129957	G	A	intergenic	*KCNIP4,LOC100505912*	.	.	.
4	22150933	C	T	intergenic	*KCNIP4,LOC100505912*	.	.	.
4	22153727	T	C	intergenic	*KCNIP4,LOC100505912*	.	.	.
4	22209306	C	A	intergenic	*KCNIP4,LOC100505912*	.	.	.
4	22209931	T	C	intergenic	*KCNIP4,LOC100505912*	.	.	.
4	22263489	T	C	intergenic	*KCNIP4,LOC100505912*	.	.	.

4	22263490	G	A	intergenic	*KCNIP4,LOC100505912*	.	.	.
1	160049487	T	C	intergenic	*KCNJ10,KCNJ9*	.	.	.
17	21351768	A	T	intergenic	*KCNJ12,C17orf51*	.	.	.
17	68100813	T	C	intronic	*KCNJ16*	.	.	.
17	68441343	G	T	intergenic	*KCNJ2,CASC17*	.	.	.
17	68810797	A	C	intergenic	*KCNJ2,CASC17*	.	.	.
17	69090472	T	A	intergenic	*KCNJ2,CASC17*	.	.	.
17	68272486	T	TAG	intergenic	*KCNJ2,CASC17*	.	.	.
17	68272488	T	G	intergenic	*KCNJ2,CASC17*	.	.	.
17	68842856	C	G	intergenic	*KCNJ2,CASC17*	.	.	.
17	68533051	C	G	intergenic	*KCNJ2,CASC17*	.	.	.
17	68777654	A	T	intergenic	*KCNJ2,CASC17*	.	.	.
17	68783887	T	A	intergenic	*KCNJ2,CASC17*	.	.	.
17	68827936	A	G	intergenic	*KCNJ2,CASC17*	.	.	.
17	68432504	C	T	intergenic	*KCNJ2,CASC17*	.	.	.
17	68432928	C	T	intergenic	*KCNJ2,CASC17*	.	.	.
17	68690494	C	T	intergenic	*KCNJ2,CASC17*	.	.	.
17	68707781	T	C	intergenic	*KCNJ2,CASC17*	.	.	.
2	155666400	TTATA	T	intronic	*KCNJ3*	.	.	.
2	155624455	G	A	intronic	*KCNJ3*	.	.	.
2	155625553	T	C	intronic	*KCNJ3*	.	.	.
2	155696105	T	C	intronic	*KCNJ3*	.	.	.
2	155697607	C	T	intronic	*KCNJ3*	.	.	.
2	155697608	A	G	intronic	*KCNJ3*	.	.	.
2	155897310	T	C	intergenic	*KCNJ3,LOC101929378*	.	.	.
2	155778509	T	G	intergenic	*KCNJ3,LOC101929378*	.	.	.
2	155837331	G	T	intergenic	*KCNJ3,LOC101929378*	.	.	.
2	156111881	TCTC	T	intergenic	*KCNJ3,LOC101929378*	.	.	.
2	156685507	A	C	intergenic	*KCNJ3,LOC101929378*	.	.	.
2	156685509	A	T	intergenic	*KCNJ3,LOC101929378*	.	.	.

2	155974277	T	G	intergenic	*KCNJ3,LOC101929378*	.	.	.
2	155975208	A	G	intergenic	*KCNJ3,LOC101929378*	.	.	.
2	156014800	G	A	intergenic	*KCNJ3,LOC101929378*	.	.	.
2	156202998	G	T	intergenic	*KCNJ3,LOC101929378*	.	.	.
2	156324324	T	G	intergenic	*KCNJ3,LOC101929378*	.	.	.
2	156333436	C	A	intergenic	*KCNJ3,LOC101929378*	.	.	.
2	156334912	A	C	intergenic	*KCNJ3,LOC101929378*	.	.	.
2	156609039	C	G	intergenic	*KCNJ3,LOC101929378*	.	.	.
2	156640042	A	AG	intergenic	*KCNJ3,LOC101929378*	.	.	.
2	156739761	C	A	intergenic	*KCNJ3,LOC101929378*	.	.	.
22	38835581	T	A	intronic	*KCNJ4*	.	.	.
21	39191187	A	T	intronic	*KCNJ6*	.	.	.
21	39180773	A	G	intronic	*KCNJ6*	.	.	.
21	39295409	C	T	intergenic	*KCNJ6,DSCR4*	.	.	.
21	39410163	ACC	A	intergenic	*KCNJ6,DSCR4*	.	.	.
12	21924360	A	G	intronic	*KCNJ8*	.	.	.
12	21943585	C	T	intergenic	*KCNJ8,ABCC9*	.	.	.
12	21944220	T	G	intergenic	*KCNJ8,ABCC9*	.	.	.
12	21944221	G	A	intergenic	*KCNJ8,ABCC9*	.	.	.
12	21945632	G	A	intergenic	*KCNJ8,ABCC9*	.	.	.
1	234001962	T	C	intergenic	*KCNK1,SLC35F3*	.	.	.
1	234001963	C	A	intergenic	*KCNK1,SLC35F3*	.	.	.
1	233885809	TCACACA	T	intergenic	*KCNK1,SLC35F3*	.	.	.
1	233823146	T	G	intergenic	*KCNK1,SLC35F3*	.	.	.
1	233993421	G	A	intergenic	*KCNK1,SLC35F3*	.	.	.
1	233901301	A	T	intergenic	*KCNK1,SLC35F3*	.	.	.
1	233901656	G	A	intergenic	*KCNK1,SLC35F3*	.	.	.
1	233902126	C	T	intergenic	*KCNK1,SLC35F3*	.	.	.

14	88840984	A	G	intergenic	*KCNK10,SPATA7*	.	.	.
2	47806320	T	A	intergenic	*KCNK12,MSH6*	.	.	.
2	47954868	G	C	intergenic	*KCNK12,MSH6*	.	.	.
2	47800106	G	A	intergenic	*KCNK12,MSH6*	.	.	.
14	90701887	C	T	intergenic	*KCNK13,PSMC1*	.	.	.
6	39288221	T	A	intronic	*KCNK16*	.	.	.
1	215307663	T	C	intronic	*KCNK2*	.	.	.
1	215327638	C	T	intronic	*KCNK2*	.	.	.
1	215327639	A	G	intronic	*KCNK2*	.	.	.
1	215337585	G	A	intronic	*KCNK2*	.	.	.
1	215341433	T	C	intronic	*KCNK2*	.	.	.
1	215341434	G	A	intronic	*KCNK2*	.	.	.
1	215347740	A	G	intronic	*KCNK2*	.	.	.
1	215716398	C	A	intergenic	*KCNK2,KCTD3*	.	.	.
1	215578058	AATAT	A	intergenic	*KCNK2,KCTD3*	.	.	.
1	215602229	T	G	intergenic	*KCNK2,KCTD3*	.	.	.
1	215707663	C	A	intergenic	*KCNK2,KCTD3*	.	.	.
1	215709844	C	A	intergenic	*KCNK2,KCTD3*	.	.	.
1	215709998	A	G	intergenic	*KCNK2,KCTD3*	.	.	.
8	140649609	A	G	intronic	*KCNK9*	.	.	.
8	140701530	G	C	intronic	*KCNK9*	.	.	.
8	140697996	A	G	intronic	*KCNK9*	.	.	.
10	78852074	A	G	intronic	*KCNMA1*	.	.	.
10	79266500	T	G	intronic	*KCNMA1*	.	.	.
10	79093281	G	A	intronic	*KCNMA1*	.	.	.
10	79520016	T	A	intergenic	*KCNMA1,DLG5*	.	.	.
3	178342527	G	T	ncRNA_intronic	*KCNMB2-AS1*	.	.	.
12	70848816	T	TC	intergenic	*KCNMB4,PTPRB*	.	.	.
12	70849065	T	A	intergenic	*KCNMB4,PTPRB*	.	.	.
12	70866147	C	T	intergenic	*KCNMB4,PTPRB*	.	.	.
12	70868458	A	G	intergenic	*KCNMB4,PTPRB*	.	.	.
12	70886541	T	G	intergenic	*KCNMB4,PTPRB*	.	.	.
1	154686950	G	T	intronic	*KCNN3*	.	.	.
1	154823667	T	C	intronic	*KCNN3*	.	.	.
11	2517265	T	TA	intronic	*KCNQ1*	.	.	.
11	2517266	T	TA	intronic	*KCNQ1*	.	.	.
11	2898892	A	C	intergenic	*KCNQ1DN,CDKN1C*	.	.	.

11	2662373	A	AT	ncRNA_exonic	*KCNQ1OT1*	.	.	.
11	2659120	A	G	ncRNA_exonic	*KCNQ1OT1*	.	.	.
11	2659245	A	G	ncRNA_exonic	*KCNQ1OT1*	.	.	.
20	62064666	G	GAGATCGGA	intronic	*KCNQ2*	.	.	.
8	133372492	C	A	intronic	*KCNQ3*	.	.	.
8	133207186	T	C	intronic	*KCNQ3*	.	.	.
8	133233631	C	T	intronic	*KCNQ3*	.	.	.
8	133233632	T	C	intronic	*KCNQ3*	.	.	.
6	73663663	C	T	intronic	*KCNQ5*	.	.	.
6	73389061	A	T	intronic	*KCNQ5*	.	.	.
6	73540459	T	G	intronic	*KCNQ5*	.	.	.
6	73552667	T	C	intronic	*KCNQ5*	.	.	.
6	73552668	G	A	intronic	*KCNQ5*	.	.	.
6	73553011	A	G	intronic	*KCNQ5*	.	.	.
6	73688733	A	T	intronic	*KCNQ5*	.	.	.
6	73689551	G	A	intronic	*KCNQ5*	.	.	.
6	73690879	A	G	intronic	*KCNQ5*	.	.	.
6	73766586	T	C	intronic	*KCNQ5*	.	.	.
6	73851145	C	T	ncRNA_intronic	*KCNQ5-AS1*	.	.	.
2	18086159	G	A	intronic	*KCNS3*	.	.	.
2	18088031	T	C	intronic	*KCNS3*	.	.	.
2	18088238	G	A	intronic	*KCNS3*	.	.	.
2	18426580	A	G	intergenic	*KCNS3,RDH14*	.	.	.
2	18537271	T	C	intergenic	*KCNS3,RDH14*	.	.	.
2	18584119	T	C	intergenic	*KCNS3,RDH14*	.	.	.
2	18167455	T	C	intergenic	*KCNS3,RDH14*	.	.	.
2	18196586	A	T	intergenic	*KCNS3,RDH14*	.	.	.
2	18321710	T	G	intergenic	*KCNS3,RDH14*	.	.	.
2	18467197	A	G	intergenic	*KCNS3,RDH14*	.	.	.
2	18495806	T	C	intergenic	*KCNS3,RDH14*	.	.	.
2	18575226	GA	G	intergenic	*KCNS3,RDH14*	.	.	.
2	18686352	A	G	intergenic	*KCNS3,RDH14*	.	.	.
1	196537499	G	A	intronic	*KCNT2*	.	.	.
1	196378620	T	C	intronic	*KCNT2*	.	.	.
1	196250947	C	T	intronic	*KCNT2*	.	.	.
1	196322152	C	T	intronic	*KCNT2*	.	.	.
1	196517480	C	T	intronic	*KCNT2*	.	.	.
1	196605843	G	A	intergenic	*KCNT2,CFH*	.	.	.

8	36751129	G	A	intronic	*KCNU1*	.	.	.
8	36911878	C	A	intergenic	*KCNU1,MIR1268A*	.	.	.
8	111422973	G	C	intergenic	*KCNV1,LINC01608*	.	.	.
8	111901480	A	G	intergenic	*KCNV1,LINC01608*	.	.	.
8	111009094	GATATAT	G	intergenic	*KCNV1,LINC01608*	.	.	.
8	111021292	A	G	intergenic	*KCNV1,LINC01608*	.	.	.
8	111054599	G	A	intergenic	*KCNV1,LINC01608*	.	.	.
8	111054600	A	G	intergenic	*KCNV1,LINC01608*	.	.	.
8	111080400	A	G	intergenic	*KCNV1,LINC01608*	.	.	.
8	111164254	A	G	intergenic	*KCNV1,LINC01608*	.	.	.
8	111232120	A	T	intergenic	*KCNV1,LINC01608*	.	.	.
8	111232122	C	T	intergenic	*KCNV1,LINC01608*	.	.	.
8	111257299	C	T	intergenic	*KCNV1,LINC01608*	.	.	.
8	111273462	T	C	intergenic	*KCNV1,LINC01608*	.	.	.
8	111344627	T	C	intergenic	*KCNV1,LINC01608*	.	.	.
8	111388258	C	T	intergenic	*KCNV1,LINC01608*	.	.	.
8	111401513	A	G	intergenic	*KCNV1,LINC01608*	.	.	.
8	111440990	T	C	intergenic	*KCNV1,LINC01608*	.	.	.
8	111568517	AGCAGCGG CAGCG	A	intergenic	*KCNV1,LINC01608*	.	.	.
18	24166182	C	T	intronic	*KCTD1*	.	.	.
16	29972183	AAT	A	intergenic	*KCTD13,TMEM219*	.	.	.
19	34453218	G	GGAAGGAA(	intergenic	*KCTD15,LSM14A*	.	.	.
19	34535698	A	AG	intergenic	*KCTD15,LSM14A*	.	.	.
19	34598158	G	A	intergenic	*KCTD15,LSM14A*	.	.	.
5	143820248	T	A	intronic	*KCTD16*	.	.	.
5	143623473	A	T	intronic	*KCTD16*	.	.	.
5	143777123	T	C	intronic	*KCTD16*	.	.	.
5	143790833	T	C	intronic	*KCTD16*	.	.	.
5	143826093	T	G	intronic	*KCTD16*	.	.	.
5	143826120	A	G	intronic	*KCTD16*	.	.	.
5	143867847	C	G	intergenic	*KCTD16,PRELID2*	.	.	.
5	144376692	C	T	intergenic	*KCTD16,PRELID2*	.	.	.
5	143975869	C	T	intergenic	*KCTD16,PRELID2*	.	.	.
5	144747317	A	T	intergenic	*KCTD16,PRELID2*	.	.	.
5	144896753	G	A	intergenic	*KCTD16,PRELID2*	.	.	.
5	143895882	G	T	intergenic	*KCTD16,PRELID2*	.	.	.
5	143895883	T	A	intergenic	*KCTD16,PRELID2*	.	.	.

5	143877997	T	G	intergenic	*KCTD16,PRELID2*	.	.	.
5	143880161	G	A	intergenic	*KCTD16,PRELID2*	.	.	.
5	143894637	T	G	intergenic	*KCTD16,PRELID2*	.	.	.
5	143894965	C	T	intergenic	*KCTD16,PRELID2*	.	.	.
5	143916062	G	GTATTTATTT	intergenic	*KCTD16,PRELID2*	.	.	.
5	143938962	T	C	intergenic	*KCTD16,PRELID2*	.	.	.
5	143939289	A	G	intergenic	*KCTD16,PRELID2*	.	.	.
5	143951207	G	T	intergenic	*KCTD16,PRELID2*	.	.	.
5	143951208	T	C	intergenic	*KCTD16,PRELID2*	.	.	.
5	143951784	C	A	intergenic	*KCTD16,PRELID2*	.	.	.
5	143969887	C	T	intergenic	*KCTD16,PRELID2*	.	.	.
5	143995235	G	A	intergenic	*KCTD16,PRELID2*	.	.	.
5	144004277	T	C	intergenic	*KCTD16,PRELID2*	.	.	.
5	144006628	T	C	intergenic	*KCTD16,PRELID2*	.	.	.
5	144006734	A	G	intergenic	*KCTD16,PRELID2*	.	.	.
5	144188192	T	C	intergenic	*KCTD16,PRELID2*	.	.	.
5	144188194	A	G	intergenic	*KCTD16,PRELID2*	.	.	.
5	144203781	C	T	intergenic	*KCTD16,PRELID2*	.	.	.
5	144203783	T	A	intergenic	*KCTD16,PRELID2*	.	.	.
5	144204682	C	G	intergenic	*KCTD16,PRELID2*	.	.	.
5	144205168	A	C	intergenic	*KCTD16,PRELID2*	.	.	.
5	144273189	A	C	intergenic	*KCTD16,PRELID2*	.	.	.
5	144311033	A	C	intergenic	*KCTD16,PRELID2*	.	.	.
5	144365533	G	A	intergenic	*KCTD16,PRELID2*	.	.	.
5	144423407	A	G	intergenic	*KCTD16,PRELID2*	.	.	.
5	144423408	T	C	intergenic	*KCTD16,PRELID2*	.	.	.
5	144476839	A	G	intergenic	*KCTD16,PRELID2*	.	.	.
5	144707774	C	T	intergenic	*KCTD16,PRELID2*	.	.	.
5	144707775	T	C	intergenic	*KCTD16,PRELID2*	.	.	.
5	144709493	G	T	intergenic	*KCTD16,PRELID2*	.	.	.
5	144795293	A	G	intergenic	*KCTD16,PRELID2*	.	.	.
5	144848434	C	T	intergenic	*KCTD16,PRELID2*	.	.	.
5	144894482	C	T	intergenic	*KCTD16,PRELID2*	.	.	.
5	144894483	A	G	intergenic	*KCTD16,PRELID2*	.	.	.
5	144896598	C	CT	intergenic	*KCTD16,PRELID2*	.	.	.
5	144897317	G	A	intergenic	*KCTD16,PRELID2*	.	.	.
5	144897319	A	G	intergenic	*KCTD16,PRELID2*	.	.	.
5	145021648	A	G	intergenic	*KCTD16,PRELID2*	.	.	.

5	145024288	T	C	intergenic	*KCTD16,PRELID2*	.	.	.
5	145024289	A	G	intergenic	*KCTD16,PRELID2*	.	.	.
5	145129187	T	A	intergenic	*KCTD16,PRELID2*	.	.	.
2	201366102	C	T	intronic	*KCTD18*	.	.	.
6	36448182	A	T	intronic	*KCTD20*	.	.	.
11	77871441	G	A	ncRNA_intronic	*KCTD21-AS1*	.	.	.
7	66112950	C	T	intergenic	*KCTD7,LOC100996437*	.	.	.
4	44210384	G	A	intronic	*KCTD8*	.	.	.
4	44262271	T	C	intronic	*KCTD8*	.	.	.
4	44321219	C	G	intronic	*KCTD8*	.	.	.
4	44268543	G	C	intronic	*KCTD8*	.	.	.
4	44271473	T	A	intronic	*KCTD8*	.	.	.
4	44305467	A	G	intronic	*KCTD8*	.	.	.
4	44339073	T	TA	intronic	*KCTD8*	.	.	.
4	44371560	C	T	intronic	*KCTD8*	.	.	.
4	44511386	C	T	intergenic	*KCTD8,YIPF7*	.	.	.
4	44476848	C	A	intergenic	*KCTD8,YIPF7*	.	.	.
4	44501509	T	G	intergenic	*KCTD8,YIPF7*	.	.	.
4	44501713	A	G	intergenic	*KCTD8,YIPF7*	.	.	.
4	44599104	T	C	intergenic	*KCTD8,YIPF7*	.	.	.
4	44599903	G	A	intergenic	*KCTD8,YIPF7*	.	.	.
4	44600498	T	C	intergenic	*KCTD8,YIPF7*	.	.	.
11	66902551	C	T	intronic	*KDM2A*	.	.	.
11	66964747	C	T	intronic	*KDM2A*	.	.	.
12	121965657	A	C	intronic	*KDM2B*	.	.	.
12	122000610	G	T	intronic	*KDM2B*	.	.	.
5	137766809	G	T	intronic	*KDM3B*	.	.	.
19	5100416	TTTGTTG	T	intronic	*KDM4B*	.	.	.
19	5201839	TACAA	T	intergenic	*KDM4B,PTPRS*	.	.	.
9	6724550	A	G	intronic	*KDM4C*	.	.	.
9	6798507	T	C	intronic	*KDM4C*	.	.	.
9	6798508	T	C	intronic	*KDM4C*	.	.	.
9	7156527	A	G	intronic	*KDM4C*	.	.	.
9	7159306	C	T	intronic	*KDM4C*	.	.	.
9	7381232	T	G	intergenic	*KDM4C,TMEM261*	.	.	.
9	7782375	C	G	intergenic	*KDM4C,TMEM261*	.	.	.
9	7205191	C	A	intergenic	*KDM4C,TMEM261*	.	.	.

9	7359382	C	T	intergenic	*KDM4C,TMEM261*	.	.	.
9	7630517	C	A	intergenic	*KDM4C,TMEM261*	.	.	.
9	7642452	T	C	intergenic	*KDM4C,TMEM261*	.	.	.
9	7253967	C	T	intergenic	*KDM4C,TMEM261*	.	.	.
9	7271668	GGAGA	G	intergenic	*KDM4C,TMEM261*	.	.	.
9	7280397	C	T	intergenic	*KDM4C,TMEM261*	.	.	.
9	7280398	T	C	intergenic	*KDM4C,TMEM261*	.	.	.
9	7281033	T	C	intergenic	*KDM4C,TMEM261*	.	.	.
9	7629316	G	A	intergenic	*KDM4C,TMEM261*	.	.	.
9	7629317	T	C	intergenic	*KDM4C,TMEM261*	.	.	.
12	409635	C	T	intronic	*KDM5A*	.	.	.
12	409636	A	G	intronic	*KDM5A*	.	.	.
16	27228410	A	G	intronic	*KDM8*	.	.	.
4	55995239	A	G	intergenic	*KDR,SRD5A3*	.	.	.
4	56034749	G	C	intergenic	*KDR,SRD5A3*	.	.	.
4	56076070	T	A	intergenic	*KDR,SRD5A3*	.	.	.
4	56077062	A	G	intergenic	*KDR,SRD5A3*	.	.	.
4	56077064	T	A	intergenic	*KDR,SRD5A3*	.	.	.
4	56168708	C	T	intergenic	*KDR,SRD5A3*	.	.	.
18	61011853	G	A	intronic	*KDSR*	.	.	.
7	142654909	C	G	intronic	*KEL*	.	.	.
7	142716334	T	C	intergenic	*KEL,OR9A2*	.	.	.
12	91477934	C	T	intergenic	*KERA,LUM*	.	.	.
12	91477935	C	G	intergenic	*KERA,LUM*	.	.	.
12	91479845	T	A	intergenic	*KERA,LUM*	.	.	.
12	91479846	A	T	intergenic	*KERA,LUM*	.	.	.
6	73950821	A	C	downstream	*KHDC1*	.	.	.
6	74022168	G	A	intergenic	*KHDC1,DPPA5*	.	.	.
6	62972804	A	T	intronic	*KHDRBS2*	.	.	.
6	62663323	G	C	intronic	*KHDRBS2*	.	.	.
6	62641926	C	A	intronic	*KHDRBS2*	.	.	.
6	62762119	T	A	intronic	*KHDRBS2*	.	.	.
6	62584795	A	G	intronic	*KHDRBS2*	.	.	.
6	62832155	G	T	intronic	*KHDRBS2*	.	.	.
6	62744430	C	T	intronic	*KHDRBS2*	.	.	.
6	62817853	A	T	intronic	*KHDRBS2*	.	.	.
6	62434960	T	TTG	intronic	*KHDRBS2*	.	.	.
6	62434961	C	G	intronic	*KHDRBS2*	.	.	.

6	62714490	A	G	intronic	*KHDRBS2*	.	.	.
6	62781165	A	G	intronic	*KHDRBS2*	.	.	.
6	62815360	G	T	intronic	*KHDRBS2*	.	.	.
6	62908088	G	A	intronic	*KHDRBS2*	.	.	.
6	63495286	G	T	intergenic	*KHDRBS2,LGSN*	.	.	.
6	63764701	T	C	intergenic	*KHDRBS2,LGSN*	.	.	.
6	63764797	A	G	intergenic	*KHDRBS2,LGSN*	.	.	.
6	63023559	C	A	intergenic	*KHDRBS2,LGSN*	.	.	.
6	63023560	C	A	intergenic	*KHDRBS2,LGSN*	.	.	.
6	63115479	AT	A	intergenic	*KHDRBS2,LGSN*	.	.	.
6	63385568	G	T	intergenic	*KHDRBS2,LGSN*	.	.	.
6	63351101	CT	C	intergenic	*KHDRBS2,LGSN*	.	.	.
6	63053996	C	A	intergenic	*KHDRBS2,LGSN*	.	.	.
6	63339769	G	A	intergenic	*KHDRBS2,LGSN*	.	.	.
6	63916470	A	ACAATG	intergenic	*KHDRBS2,LGSN*	.	.	.
6	63515653	T	A	intergenic	*KHDRBS2,LGSN*	.	.	.
6	63790724	G	T	intergenic	*KHDRBS2,LGSN*	.	.	.
6	63023464	C	G	intergenic	*KHDRBS2,LGSN*	.	.	.
6	63023465	T	A	intergenic	*KHDRBS2,LGSN*	.	.	.
6	63024772	T	A	intergenic	*KHDRBS2,LGSN*	.	.	.
6	63026720	T	A	intergenic	*KHDRBS2,LGSN*	.	.	.
6	63093635	G	T	intergenic	*KHDRBS2,LGSN*	.	.	.
6	63126519	C	T	intergenic	*KHDRBS2,LGSN*	.	.	.
6	63126520	A	C	intergenic	*KHDRBS2,LGSN*	.	.	.
6	63205087	G	C	intergenic	*KHDRBS2,LGSN*	.	.	.
6	63243639	G	A	intergenic	*KHDRBS2,LGSN*	.	.	.
6	63266939	C	A	intergenic	*KHDRBS2,LGSN*	.	.	.
6	63405060	TG	T	intergenic	*KHDRBS2,LGSN*	.	.	.
6	63406653	A	T	intergenic	*KHDRBS2,LGSN*	.	.	.
6	63525613	C	T	intergenic	*KHDRBS2,LGSN*	.	.	.
6	63526525	C	G	intergenic	*KHDRBS2,LGSN*	.	.	.
6	63539408	T	G	intergenic	*KHDRBS2,LGSN*	.	.	.
6	63660359	A	G	intergenic	*KHDRBS2,LGSN*	.	.	.
6	63660360	T	C	intergenic	*KHDRBS2,LGSN*	.	.	.
6	63660685	A	T	intergenic	*KHDRBS2,LGSN*	.	.	.
6	63660815	T	C	intergenic	*KHDRBS2,LGSN*	.	.	.
6	63695451	A	G	intergenic	*KHDRBS2,LGSN*	.	.	.
6	63738995	C	T	intergenic	*KHDRBS2,LGSN*	.	.	.

6	63740350	C	A	intergenic	*KHDRBS2,LGSN*	.	.	.
6	63751205	A	C	intergenic	*KHDRBS2,LGSN*	.	.	.
6	63766989	C	T	intergenic	*KHDRBS2,LGSN*	.	.	.
6	63767182	T	C	intergenic	*KHDRBS2,LGSN*	.	.	.
6	63782251	A	C	intergenic	*KHDRBS2,LGSN*	.	.	.
6	63809275	G	A	intergenic	*KHDRBS2,LGSN*	.	.	.
6	63809276	T	C	intergenic	*KHDRBS2,LGSN*	.	.	.
6	63880192	T	C	intergenic	*KHDRBS2,LGSN*	.	.	.
6	63881108	T	C	intergenic	*KHDRBS2,LGSN*	.	.	.
8	136869765	ATGTG	A	intergenic	*KHDRBS3,LOC101927915*	.	.	.
8	137300001	A	T	intergenic	*KHDRBS3,LOC101927915*	.	.	.
8	137723787	G	A	intergenic	*KHDRBS3,LOC101927915*	.	.	.
8	137853768	A	G	intergenic	*KHDRBS3,LOC101927915*	.	.	.
8	137961740	A	C	intergenic	*KHDRBS3,LOC101927915*	.	.	.
8	137367932	A	G	intergenic	*KHDRBS3,LOC101927915*	.	.	.
8	136827396	A	T	intergenic	*KHDRBS3,LOC101927915*	.	.	.
8	136827397	G	A	intergenic	*KHDRBS3,LOC101927915*	.	.	.
8	136861057	C	A	intergenic	*KHDRBS3,LOC101927915*	.	.	.
8	137358769	A	G	intergenic	*KHDRBS3,LOC101927915*	.	.	.
8	136845501	T	A	intergenic	*KHDRBS3,LOC101927915*	.	.	.
8	136902569	C	A	intergenic	*KHDRBS3,LOC101927915*	.	.	.
8	136700681	G	A	intergenic	*KHDRBS3,LOC101927915*	.	.	.
8	137205085	A	G	intergenic	*KHDRBS3,LOC101927915*	.	.	.

8	137205740	A	T	intergenic	*KHDRBS3,LOC101927915*	.	.	.
8	137336935	G	GCAGACACA	intergenic	*KHDRBS3,LOC101927915*	.	.	.
8	137577826	A	C	intergenic	*KHDRBS3,LOC101927915*	.	.	.
8	137624250	A	T	intergenic	*KHDRBS3,LOC101927915*	.	.	.
8	137624643	G	T	intergenic	*KHDRBS3,LOC101927915*	.	.	.
8	137674075	G	A	intergenic	*KHDRBS3,LOC101927915*	.	.	.
8	137774324	A	G	intergenic	*KHDRBS3,LOC101927915*	.	.	.
8	137903230	A	G	intergenic	*KHDRBS3,LOC101927915*	.	.	.
8	137930754	A	T	intergenic	*KHDRBS3,LOC101927915*	.	.	.
8	138236865	A	ATAAG	intergenic	*KHDRBS3,LOC101927915*	.	.	.
8	138255947	A	T	intergenic	*KHDRBS3,LOC101927915*	.	.	.
1	175178861	G	T	intergenic	*KIAA0040,TNR*	.	.	.
1	175235987	G	A	intergenic	*KIAA0040,TNR*	.	.	.
1	175260538	A	AC	intergenic	*KIAA0040,TNR*	.	.	.
1	175227182	A	G	intergenic	*KIAA0040,TNR*	.	.	.
1	175273378	C	T	intergenic	*KIAA0040,TNR*	.	.	.
7	26602961	C	A	intergenic	*KIAA0087,C7orf71*	.	.	.
7	26603847	A	G	intergenic	*KIAA0087,C7orf71*	.	.	.
14	106423140	T	C	intergenic	*KIAA0125,ADAM6*	.	.	.
14	106424734	A	T	intergenic	*KIAA0125,ADAM6*	.	.	.
14	106426190	A	T	intergenic	*KIAA0125,ADAM6*	.	.	.
4	6820558	G	A	intronic	*KIAA0232*	.	.	.
4	6820559	T	C	intronic	*KIAA0232*	.	.	.
4	6837942	A	G	intronic	*KIAA0232*	.	.	.
14	35601950	G	A	intronic	*KIAA0391*	.	.	.
14	35632766	C	T	intronic	*KIAA0391*	.	.	.
14	35644704	G	A	intronic	*KIAA0391*	.	.	.

14	35646387	T	A	intronic	*KIAA0391*	.	.	.
14	58974584	C	T	intronic	*KIAA0586*	.	.	.
14	58997435	A	T	intronic	*KIAA0586*	.	.	.
14	59047674	T	C	intergenic	*KIAA0586,DACT1*	.	.	.
14	59049349	T	C	intergenic	*KIAA0586,DACT1*	.	.	.
14	59056337	G	A	intergenic	*KIAA0586,DACT1*	.	.	.
14	59058224	C	G	intergenic	*KIAA0586,DACT1*	.	.	.
5	93607847	T	A	intronic	*KIAA0825*	.	.	.
5	93720408	G	C	intronic	*KIAA0825*	.	.	.
5	93490909	A	T	intronic	*KIAA0825*	.	.	.
5	93561802	G	T	intronic	*KIAA0825*	.	.	.
5	93882222	CAGAGAGAG	C	intronic	*KIAA0825*	.	.	.
5	93921801	T	C	intronic	*KIAA0825*	.	.	.
5	93923183	G	A	intronic	*KIAA0825*	.	.	.
5	93924167	A	G	intronic	*KIAA0825*	.	.	.
4	154389908	T	C	intronic	*KIAA0922*	.	.	.
15	79850252	G	T	intergenic	*KIAA1024,ST20-MTHFS*	.	.	.
15	80005887	T	C	intergenic	*KIAA1024,ST20-MTHFS*	.	.	.
15	80078748	G	T	intergenic	*KIAA1024,ST20-MTHFS*	.	.	.
15	80078750	G	A	intergenic	*KIAA1024,ST20-MTHFS*	.	.	.
5	129199612	T	A	intergenic	*KIAA1024L,CHSY3*	.	.	.
5	129161566	T	C	intergenic	*KIAA1024L,CHSY3*	.	.	.
5	129205319	T	C	intergenic	*KIAA1024L,CHSY3*	.	.	.
1	92645694	A	G	intronic	*KIAA1107*	.	.	.
1	92665328	A	T	intergenic	*KIAA1107,C1orf146*	.	.	.
4	123132610	C	T	intronic	*KIAA1109*	.	.	.
9	34370001	TTCTC	T	UTR3	*KIAA1161*	.	.	.
X	118314479	C	T	intergenic	*KIAA1210,PGRMC1*	.	.	.

4	57198895	T	C	intergenic	*KIAA1211,AASDH*	.	.	.
2	99606384	T	G	intergenic	*KIAA1211L,TSGA10*	.	.	.
2	99608576	C	T	intergenic	*KIAA1211L,TSGA10*	.	.	.
10	24137092	A	T	intronic	*KIAA1217*	.	.	.
10	24156520	G	C	intronic	*KIAA1217*	.	.	.
10	24624552	TATTG	T	intronic	*KIAA1217*	.	.	.
10	24676350	C	T	intronic	*KIAA1217*	.	.	.
10	24677139	C	T	intronic	*KIAA1217*	.	.	.
10	24839476	G	A	intergenic	*KIAA1217,ARHGAP21*	.	.	.
7	86520358	T	A	intronic	*KIAA1324L*	.	.	.
7	86672124	T	C	intronic	*KIAA1324L*	.	.	.
7	86726342	G	A	intergenic	*KIAA1324L,DMTF1*	.	.	.
18	34610087	A	C	intronic	*KIAA1328*	.	.	.
18	34454783	T	A	intronic	*KIAA1328*	.	.	.
18	34456732	A	G	intronic	*KIAA1328*	.	.	.
18	34456734	A	G	intronic	*KIAA1328*	.	.	.
18	34457391	G	C	intronic	*KIAA1328*	.	.	.
18	34549508	T	G	intronic	*KIAA1328*	.	.	.
18	34653056	T	C	intronic	*KIAA1328*	.	.	.
18	34679637	T	G	intronic	*KIAA1328*	.	.	.
18	34692868	G	A	intronic	*KIAA1328*	.	.	.
18	34694781	G	A	intronic	*KIAA1328*	.	.	.
18	34728743	G	C	intronic	*KIAA1328*	.	.	.
18	34728907	C	T	intronic	*KIAA1328*	.	.	.
18	34729454	C	T	intronic	*KIAA1328*	.	.	.
18	34729455	A	G	intronic	*KIAA1328*	.	.	.
18	34794300	A	G	intronic	*KIAA1328*	.	.	.
18	34794510	T	C	intronic	*KIAA1328*	.	.	.
18	34796368	T	C	intronic	*KIAA1328*	.	.	.
8	95626681	T	A	intergenic	*KIAA1429,LOC100288748*	.	.	.
10	30469653	T	A	intergenic	*KIAA1462,LOC101929279*	.	.	.
10	30439208	G	C	intergenic	*KIAA1462,LOC101929279*	.	.	.

10	30439210	A	G	intergenic	*KIAA1462,LOC101929279*	.	.	.
18	59991066	G	T	intergenic	*KIAA1468,TNFRSF11A*	.	.	.
7	138587469	C	T	intronic	*KIAA1549*	.	.	.
7	138633550	C	T	intronic	*KIAA1549*	.	.	.
12	32246061	G	T	intergenic	*KIAA1551,BICD1*	.	.	.
22	44698763	GGTGAGGAGGCAGCAGGGGCAAAGGCCCTGGGGTGGGTGCA	G	intronic	*KIAA1644*	.	.	.
2	176825421	T	A	intronic	*KIAA1715*	.	.	.
1	233732463	T	C	intergenic	*KIAA1804,KCNK1*	.	.	.
2	61339731	C	T	intronic	*KIAA1841*	.	.	.
2	61345869	T	G	intronic	*KIAA1841*	.	.	.
2	61345870	C	T	intronic	*KIAA1841*	.	.	.
6	111605898	A	G	intergenic	*KIAA1919,REV3L*	.	.	.
6	111592402	T	A	intergenic	*KIAA1919,REV3L*	.	.	.
9	115436529	T	A	intergenic	*KIAA1958,INIP*	.	.	.
X	73996110	A	C	intronic	*KIAA2022*	.	.	.
X	74038847	G	C	intronic	*KIAA2022*	.	.	.
X	74039699	G	T	intronic	*KIAA2022*	.	.	.
X	74095434	G	A	intronic	*KIAA2022*	.	.	.
X	74095982	C	T	intronic	*KIAA2022*	.	.	.
X	74140489	T	G	intronic	*KIAA2022*	.	.	.
X	74140490	T	C	intronic	*KIAA2022*	.	.	.
X	74159340	T	G	intergenic	*KIAA2022,ABCB7*	.	.	.
X	74158364	A	G	intergenic	*KIAA2022,ABCB7*	.	.	.
X	74205434	C	CT	intergenic	*KIAA2022,ABCB7*	.	.	.
X	74215204	T	C	intergenic	*KIAA2022,ABCB7*	.	.	.
X	74234566	G	A	intergenic	*KIAA2022,ABCB7*	.	.	.
X	74234947	T	G	intergenic	*KIAA2022,ABCB7*	.	.	.
X	74234948	G	C	intergenic	*KIAA2022,ABCB7*	.	.	.
X	74254212	T	G	intergenic	*KIAA2022,ABCB7*	.	.	.
X	74254622	T	C	intergenic	*KIAA2022,ABCB7*	.	.	.
X	74254623	G	A	intergenic	*KIAA2022,ABCB7*	.	.	.

10	94372161	T	C	intronic	*KIF11*	.	.	.
6	17916181	AAAATAAAAAT	A	intronic	*KIF13A*	.	.	.
6	17923042	A	G	intronic	*KIF13A*	.	.	.
6	17909370	G	A	intronic	*KIF13A*	.	.	.
6	18032556	A	G	intergenic	*KIF13A,NHLRC1*	.	.	.
3	44860487	C	T	intronic	*KIF15*	.	.	.
20	16269550	TTGGGATCCCATCATAAGTGGAGGAGTATCTGTTCTTAGTAAGTACATACTGCAGAGATGCTCCTCCACTTATGA	T	intronic	*KIF16B*	.	.	.
20	16504297	T	A	intronic	*KIF16B*	.	.	.
20	16638220	C	T	intergenic	*KIF16B,SNRPB2*	.	.	.
20	16681591	C	T	intergenic	*KIF16B,SNRPB2*	.	.	.
10	91524068	A	G	intronic	*KIF20B*	.	.	.
10	91580851	AAAACAAAC	A	intergenic	*KIF20B,LINC00865*	.	.	.
14	104683357	C	T	intergenic	*KIF26A,C14orf180*	.	.	.
14	104674876	T	C	intergenic	*KIF26A,C14orf180*	.	.	.
14	104819146	C	T	intergenic	*KIF26A,C14orf180*	.	.	.
14	104879719	G	C	intergenic	*KIF26A,C14orf180*	.	.	.
14	104880060	A	G	intergenic	*KIF26A,C14orf180*	.	.	.
14	104881090	A	G	intergenic	*KIF26A,C14orf180*	.	.	.
14	104881399	G	A	intergenic	*KIF26A,C14orf180*	.	.	.
1	245663257	G	A	intronic	*KIF26B*	.	.	.
1	245443808	G	T	intronic	*KIF26B*	.	.	.
1	245756770	T	A	intronic	*KIF26B*	.	.	.
1	245759735	A	G	intronic	*KIF26B*	.	.	.
1	245792802	A	G	intronic	*KIF26B*	.	.	.
1	245792804	C	T	intronic	*KIF26B*	.	.	.
1	245909915	T	C	intergenic	*KIF26B,SMYD3*	.	.	.
9	86476461	G	A	intronic	*KIF27*	.	.	.

9	86479161	A	G	intronic	*KIF27*	.	.	.
17	51968906	G	T	intergenic	*KIF2B,TOM1L1*	.	.	.
17	52429938	G	C	intergenic	*KIF2B,TOM1L1*	.	.	.
17	52489720	G	A	intergenic	*KIF2B,TOM1L1*	.	.	.
17	52090958	T	C	intergenic	*KIF2B,TOM1L1*	.	.	.
17	52626341	A	G	intergenic	*KIF2B,TOM1L1*	.	.	.
17	52003318	A	G	intergenic	*KIF2B,TOM1L1*	.	.	.
17	52757693	G	A	intergenic	*KIF2B,TOM1L1*	.	.	.
17	52807507	G	A	intergenic	*KIF2B,TOM1L1*	.	.	.
17	51912778	G	A	intergenic	*KIF2B,TOM1L1*	.	.	.
17	51912966	T	C	intergenic	*KIF2B,TOM1L1*	.	.	.
17	51919986	T	C	intergenic	*KIF2B,TOM1L1*	.	.	.
17	51920363	G	A	intergenic	*KIF2B,TOM1L1*	.	.	.
17	51995043	A	G	intergenic	*KIF2B,TOM1L1*	.	.	.
17	52147078	C	T	intergenic	*KIF2B,TOM1L1*	.	.	.
17	52148456	A	T	intergenic	*KIF2B,TOM1L1*	.	.	.
17	52166778	C	T	intergenic	*KIF2B,TOM1L1*	.	.	.
17	52166983	G	A	intergenic	*KIF2B,TOM1L1*	.	.	.
17	52167275	G	A	intergenic	*KIF2B,TOM1L1*	.	.	.
17	52203554	A	T	intergenic	*KIF2B,TOM1L1*	.	.	.
17	52239834	T	C	intergenic	*KIF2B,TOM1L1*	.	.	.
17	52240600	T	C	intergenic	*KIF2B,TOM1L1*	.	.	.
17	52284089	G	A	intergenic	*KIF2B,TOM1L1*	.	.	.
17	52515469	C	A	intergenic	*KIF2B,TOM1L1*	.	.	.
17	52540373	T	C	intergenic	*KIF2B,TOM1L1*	.	.	.
17	52540374	G	T	intergenic	*KIF2B,TOM1L1*	.	.	.
17	52641990	ACAAACAAC	A	intergenic	*KIF2B,TOM1L1*	.	.	.
17	52665924	T	TGG	intergenic	*KIF2B,TOM1L1*	.	.	.
17	52665925	CAA	C	intergenic	*KIF2B,TOM1L1*	.	.	.
17	52668311	A	G	intergenic	*KIF2B,TOM1L1*	.	.	.
17	52736034	T	C	intergenic	*KIF2B,TOM1L1*	.	.	.
17	52736036	T	A	intergenic	*KIF2B,TOM1L1*	.	.	.
17	52774560	A	G	intergenic	*KIF2B,TOM1L1*	.	.	.
17	52775025	C	T	intergenic	*KIF2B,TOM1L1*	.	.	.
17	52900261	C	T	intergenic	*KIF2B,TOM1L1*	.	.	.
5	132059489	A	G	intronic	*KIF3A*	.	.	.
20	30924492	G	A	intergenic	*KIF3B,ASXL1*	.	.	.

2	26246263	A	G	intergenic	*KIF3C,RAB10*	.	.	.
X	69586283	A	G	intronic	*KIF4A*	.	.	.
X	69617092	C	T	intronic	*KIF4A*	.	.	.
X	69631827	TACACAC	T	intronic	*KIF4A*	.	.	.
5	155012356	T	G	intergenic	*KIF4B,SGCD*	.	.	.
5	155719866	T	G	intergenic	*KIF4B,SGCD*	.	.	.
5	154917645	A	G	intergenic	*KIF4B,SGCD*	.	.	.
5	154972829	C	A	intergenic	*KIF4B,SGCD*	.	.	.
5	155703642	C	A	intergenic	*KIF4B,SGCD*	.	.	.
5	154487488	T	C	intergenic	*KIF4B,SGCD*	.	.	.
5	154636758	T	A	intergenic	*KIF4B,SGCD*	.	.	.
5	155128417	A	G	intergenic	*KIF4B,SGCD*	.	.	.
5	154865178	T	C	intergenic	*KIF4B,SGCD*	.	.	.
5	154928932	G	T	intergenic	*KIF4B,SGCD*	.	.	.
5	155656717	T	A	intergenic	*KIF4B,SGCD*	.	.	.
5	155715271	G	A	intergenic	*KIF4B,SGCD*	.	.	.
5	154727496	G	A	intergenic	*KIF4B,SGCD*	.	.	.
5	154552340	C	T	intergenic	*KIF4B,SGCD*	.	.	.
5	154606304	G	A	intergenic	*KIF4B,SGCD*	.	.	.
5	154757506	A	G	intergenic	*KIF4B,SGCD*	.	.	.
5	155019554	T	C	intergenic	*KIF4B,SGCD*	.	.	.
5	155020022	T	C	intergenic	*KIF4B,SGCD*	.	.	.
5	155021769	A	T	intergenic	*KIF4B,SGCD*	.	.	.
5	155098193	G	A	intergenic	*KIF4B,SGCD*	.	.	.
5	155492325	T	C	intergenic	*KIF4B,SGCD*	.	.	.
5	155492445	G	A	intergenic	*KIF4B,SGCD*	.	.	.
5	155497396	C	A	intergenic	*KIF4B,SGCD*	.	.	.
10	32491378	A	G	intergenic	*KIF5B,EPC1*	.	.	.
6	39346312	T	C	intronic	*KIF6*	.	.	.
6	39311978	C	T	intronic	*KIF6*	.	.	.
6	39472876	C	T	intronic	*KIF6*	.	.	.
6	39478758	A	G	intronic	*KIF6*	.	.	.
6	39478915	T	C	intronic	*KIF6*	.	.	.
6	39505909	T	A	intronic	*KIF6*	.	.	.
6	39609367	A	T	intronic	*KIF6*	.	.	.
6	39708519	C	T	intergenic	*KIF6,DAAM2*	.	.	.
15	90173005	C	A	intronic	*KIF7*	.	.	.
1	170037194	T	A	UTR5	*KIFAP3*	.	.	.

1	170020215	G	A	intronic	*KIFAP3*	.	.	.
1	170028162	C	T	intronic	*KIFAP3*	.	.	.
1	170028163	A	G	intronic	*KIFAP3*	.	.	.
1	170075153	A	G	intergenic	*KIFAP3,METTL11B*	.	.	.
6	33370296	T	C	intronic	*KIFC1*	.	.	.
10	7819351	T	C	intronic	*KIN*	.	.	.
19	55376642	C	T	intronic	*KIR3DL2*	.	.	.
11	126807674	A	T	intronic	*KIRREL3*	.	.	.
11	126807675	G	T	intronic	*KIRREL3*	.	.	.
11	126480071	C	T	intronic	*KIRREL3*	.	.	.
4	55554603	G	GGACAGCTA	intronic	*KIT*	.	.	.
4	55566225	C	T	intronic	*KIT*	.	.	.
4	55627297	A	T	intergenic	*KIT,KDR*	.	.	.
4	55677871	T	A	intergenic	*KIT,KDR*	.	.	.
4	55789387	G	C	intergenic	*KIT,KDR*	.	.	.
4	55835754	A	G	intergenic	*KIT,KDR*	.	.	.
12	89103589	T	A	intergenic	*KITLG,LOC728084*	.	.	.
12	88987072	T	C	intergenic	*KITLG,LOC728084*	.	.	.
12	88997656	C	A	intergenic	*KITLG,LOC728084*	.	.	.
12	89000834	A	C	intergenic	*KITLG,LOC728084*	.	.	.
12	89001523	A	G	intergenic	*KITLG,LOC728084*	.	.	.
12	89003957	C	G	intergenic	*KITLG,LOC728084*	.	.	.
12	89063738	T	C	intergenic	*KITLG,LOC728084*	.	.	.
12	89203855	T	C	intergenic	*KITLG,LOC728084*	.	.	.
12	89203856	G	A	intergenic	*KITLG,LOC728084*	.	.	.
12	89222744	T	C	intergenic	*KITLG,LOC728084*	.	.	.
20	21262766	G	A	intergenic	*KIZ,XRN2*	.	.	.
13	74529704	C	A	intronic	*KLF12*	.	.	.
13	74562804	C	T	intronic	*KLF12*	.	.	.
13	74632694	T	C	intronic	*KLF12*	.	.	.
13	74660615	C	T	intronic	*KLF12*	.	.	.
13	74758334	A	G	intergenic	*KLF12,LINC00381*	.	.	.
13	74917002	A	ATATGTAAT	intergenic	*KLF12,LINC00381*	.	.	.
13	74924717	TA	T	intergenic	*KLF12,LINC00381*	.	.	.
13	74924719	T	C	intergenic	*KLF12,LINC00381*	.	.	.
7	130450223	T	C	intergenic	*KLF14,MIR29A*	.	.	.
7	130450224	G	A	intergenic	*KLF14,MIR29A*	.	.	.

7	130481975	A	G	intergenic	*KLF14,MIR29A*	.	.	.
7	130484133	T	G	intergenic	*KLF14,MIR29A*	.	.	.
1	44588467	T	C	intronic	*KLF17*	.	.	.
1	44588468	G	A	intronic	*KLF17*	.	.	.
4	38739747	A	G	intergenic	*KLF3,TLR10*	.	.	.
4	38764891	A	C	intergenic	*KLF3,TLR10*	.	.	.
9	110432969	A	ATG	intergenic	*KLF4,ACTL7B*	.	.	.
9	110746745	ATCTC	A	intergenic	*KLF4,ACTL7B*	.	.	.
9	111360079	GTGGATGG ATGGA	G	intergenic	*KLF4,ACTL7B*	.	.	.
9	111515405	G	T	intergenic	*KLF4,ACTL7B*	.	.	.
9	110789401	G	C	intergenic	*KLF4,ACTL7B*	.	.	.
9	110831249	A	G	intergenic	*KLF4,ACTL7B*	.	.	.
9	111208816	A	G	intergenic	*KLF4,ACTL7B*	.	.	.
9	111479400	C	T	intergenic	*KLF4,ACTL7B*	.	.	.
9	111581471	A	T	intergenic	*KLF4,ACTL7B*	.	.	.
13	73643052	A	G	intronic	*KLF5*	.	.	.
13	73732615	T	C	intergenic	*KLF5,LINC00392*	.	.	.
13	74005906	G	GAGA	intergenic	*KLF5,LINC00392*	.	.	.
13	74029740	G	A	intergenic	*KLF5,LINC00392*	.	.	.
13	74029874	G	C	intergenic	*KLF5,LINC00392*	.	.	.
13	74032970	G	T	intergenic	*KLF5,LINC00392*	.	.	.
10	4020275	T	C	intergenic	*KLF6,MIR6078*	.	.	.
X	56321746	A	ACAAACAGA	intergenic	*KLF8,UBQLN2*	.	.	.
X	56587519	T	A	intergenic	*KLF8,UBQLN2*	.	.	.
X	56352795	G	C	intergenic	*KLF8,UBQLN2*	.	.	.
X	56353912	T	G	intergenic	*KLF8,UBQLN2*	.	.	.
X	56376582	T	A	intergenic	*KLF8,UBQLN2*	.	.	.
X	56416769	T	C	intergenic	*KLF8,UBQLN2*	.	.	.
X	56418199	T	C	intergenic	*KLF8,UBQLN2*	.	.	.
X	56427270	T	C	intergenic	*KLF8,UBQLN2*	.	.	.
X	56432289	G	A	intergenic	*KLF8,UBQLN2*	.	.	.
X	56432290	A	G	intergenic	*KLF8,UBQLN2*	.	.	.
X	56432459	C	G	intergenic	*KLF8,UBQLN2*	.	.	.
X	56444124	T	C	intergenic	*KLF8,UBQLN2*	.	.	.
X	56444125	G	A	intergenic	*KLF8,UBQLN2*	.	.	.
X	56446473	C	T	intergenic	*KLF8,UBQLN2*	.	.	.
X	56452431	C	A	intergenic	*KLF8,UBQLN2*	.	.	.

X	56452874	A	G	intergenic	*KLF8,UBQLN2*	.	.	.
X	56467871	G	A	intergenic	*KLF8,UBQLN2*	.	.	.
X	56476190	G	A	intergenic	*KLF8,UBQLN2*	.	.	.
X	56476612	A	G	intergenic	*KLF8,UBQLN2*	.	.	.
X	56481543	T	C	intergenic	*KLF8,UBQLN2*	.	.	.
X	56516368	T	C	intergenic	*KLF8,UBQLN2*	.	.	.
X	56516765	G	A	intergenic	*KLF8,UBQLN2*	.	.	.
X	56518980	C	T	intergenic	*KLF8,UBQLN2*	.	.	.
X	56536310	C	G	intergenic	*KLF8,UBQLN2*	.	.	.
X	56536805	T	A	intergenic	*KLF8,UBQLN2*	.	.	.
X	56550594	G	A	intergenic	*KLF8,UBQLN2*	.	.	.
6	42987529	C	G	intronic	*KLHDC3*	.	.	.
13	70418893	GTTTT	G	intronic	*KLHL1*	.	.	.
13	70316315	T	C	intronic	*KLHL1*	.	.	.
13	70534050	G	T	intronic	*KLHL1*	.	.	.
13	70347995	CTATATA	C	intronic	*KLHL1*	.	.	.
13	70376374	C	A	intronic	*KLHL1*	.	.	.
13	70452985	G	A	intronic	*KLHL1*	.	.	.
13	70490819	G	A	intronic	*KLHL1*	.	.	.
X	117045730	C	T	intronic	*KLHL13*	.	.	.
X	117073926	G	A	intronic	*KLHL13*	.	.	.
X	117076599	A	G	intronic	*KLHL13*	.	.	.
X	117085834	C	G	intronic	*KLHL13*	.	.	.
X	117100823	G	T	intronic	*KLHL13*	.	.	.
X	117143137	T	C	intronic	*KLHL13*	.	.	.
X	117170479	A	G	intronic	*KLHL13*	.	.	.
X	117229557	T	C	intronic	*KLHL13*	.	.	.
X	117229927	A	C	intronic	*KLHL13*	.	.	.
X	117453685	T	C	intergenic	*KLHL13,WDR44*	.	.	.
X	117380477	G	A	intergenic	*KLHL13,WDR44*	.	.	.
X	117380478	C	T	intergenic	*KLHL13,WDR44*	.	.	.
X	117380801	G	A	intergenic	*KLHL13,WDR44*	.	.	.
18	30376178	C	A	intergenic	*KLHL14,CCDC178*	.	.	.
X	24054330	G	A	intergenic	*KLHL15,EIF2S3*	.	.	.
3	47359718	A	G	intronic	*KLHL18*	.	.	.
1	173688060	G	A	intronic	*KLHL20*	.	.	.
1	173697027	C	T	intronic	*KLHL20*	.	.	.
1	173697171	T	C	intronic	*KLHL20*	.	.	.

1	173698299	T	A	intronic	*KLHL20*	.	.	.
1	173698650	G	A	intronic	*KLHL20*	.	.	.
1	173700499	A	T	intronic	*KLHL20*	.	.	.
15	86514516	T	A	intergenic	*KLHL25,LINC01584*	.	.	.
19	18769999	C	T	intronic	*KLHL26*	.	.	.
14	45431026	G	A	intronic	*KLHL28*	.	.	.
2	23962409	A	G	intergenic	*KLHL29,ATAD2B*	.	.	.
5	137049678	AG	A	intronic	*KLHL3*	.	.	.
6	53533980	A	G	intergenic	*KLHL31,LRRC1*	.	.	.
6	53641236	C	G	intergenic	*KLHL31,LRRC1*	.	.	.
6	53642674	C	T	intergenic	*KLHL31,LRRC1*	.	.	.
6	53642675	A	G	intergenic	*KLHL31,LRRC1*	.	.	.
6	53643305	T	A	intergenic	*KLHL31,LRRC1*	.	.	.
6	53643384	A	C	intergenic	*KLHL31,LRRC1*	.	.	.
6	97537389	T	G	intronic	*KLHL32*	.	.	.
6	97464444	T	C	intronic	*KLHL32*	.	.	.
X	21673476	C	T	UTR3	*KLHL34*	.	.	.
X	86871369	C	T	intronic	*KLHL4*	.	.	.
X	87450937	A	G	intergenic	*KLHL4,CPXCR1*	.	.	.
X	87129181	G	A	intergenic	*KLHL4,CPXCR1*	.	.	.
X	87257473	G	C	intergenic	*KLHL4,CPXCR1*	.	.	.
X	87218085	T	A	intergenic	*KLHL4,CPXCR1*	.	.	.
X	87679349	A	G	intergenic	*KLHL4,CPXCR1*	.	.	.
X	87750672	T	A	intergenic	*KLHL4,CPXCR1*	.	.	.
X	87760529	C	T	intergenic	*KLHL4,CPXCR1*	.	.	.
X	87969621	C	G	intergenic	*KLHL4,CPXCR1*	.	.	.
X	87020315	A	C	intergenic	*KLHL4,CPXCR1*	.	.	.
X	87020316	G	A	intergenic	*KLHL4,CPXCR1*	.	.	.
X	87046611	G	A	intergenic	*KLHL4,CPXCR1*	.	.	.
X	87046612	C	T	intergenic	*KLHL4,CPXCR1*	.	.	.
X	87080450	G	A	intergenic	*KLHL4,CPXCR1*	.	.	.
X	87084532	A	C	intergenic	*KLHL4,CPXCR1*	.	.	.
X	87115071	A	G	intergenic	*KLHL4,CPXCR1*	.	.	.
X	87172565	C	G	intergenic	*KLHL4,CPXCR1*	.	.	.
X	87172567	T	A	intergenic	*KLHL4,CPXCR1*	.	.	.
X	87242520	G	A	intergenic	*KLHL4,CPXCR1*	.	.	.
X	87243778	T	C	intergenic	*KLHL4,CPXCR1*	.	.	.

X	87263320	G	A	intergenic	*KLHL4,CPXCR1*	.	.	.
X	87263438	T	C	intergenic	*KLHL4,CPXCR1*	.	.	.
X	87345038	C	A	intergenic	*KLHL4,CPXCR1*	.	.	.
X	87479635	T	C	intergenic	*KLHL4,CPXCR1*	.	.	.
X	87479636	G	A	intergenic	*KLHL4,CPXCR1*	.	.	.
X	87534159	G	T	intergenic	*KLHL4,CPXCR1*	.	.	.
X	87534898	T	G	intergenic	*KLHL4,CPXCR1*	.	.	.
X	87535409	C	T	intergenic	*KLHL4,CPXCR1*	.	.	.
X	87572588	A	T	intergenic	*KLHL4,CPXCR1*	.	.	.
X	87576132	C	T	intergenic	*KLHL4,CPXCR1*	.	.	.
X	87744618	C	A	intergenic	*KLHL4,CPXCR1*	.	.	.
X	87792762	T	C	intergenic	*KLHL4,CPXCR1*	.	.	.
X	87864946	T	A	intergenic	*KLHL4,CPXCR1*	.	.	.
12	28016845	A	G	intergenic	*KLHL42,PTHLH*	.	.	.
12	27999683	G	A	intergenic	*KLHL42,PTHLH*	.	.	.
12	28063896	C	T	intergenic	*KLHL42,PTHLH*	.	.	.
7	23187749	G	A	intronic	*KLHL7*	.	.	.
7	23172772	G	A	intronic	*KLHL7*	.	.	.
19	51462011	C	A	UTR3	*KLK6*	.	.	.
19	51461566	C	T	downstream	*KLK6*	.	.	.
12	9759948	C	CT	intronic	*KLRB1*	.	.	.
12	9762453	C	T	intergenic	*KLRB1,LOC374443*	.	.	.
12	10712262	A	G	intergenic	*KLRC1,KLRAP1*	.	.	.
12	10618298	C	T	intergenic	*KLRC1,KLRAP1*	.	.	.
12	10626114	A	T	intergenic	*KLRC1,KLRAP1*	.	.	.
12	10627378	G	A	intergenic	*KLRC1,KLRAP1*	.	.	.
12	10630771	T	C	intergenic	*KLRC1,KLRAP1*	.	.	.
12	10637821	C	G	intergenic	*KLRC1,KLRAP1*	.	.	.
12	10670198	C	T	intergenic	*KLRC1,KLRAP1*	.	.	.
12	10478253	A	G	intergenic	*KLRD1,LOC101928100*	.	.	.
7	139189830	G	A	intergenic	*KLRG2,CLEC2L*	.	.	.
7	152111379	CAA	C	intronic	*KMT2C*	.	.	.
7	152111381	A	AGGGG	intronic	*KMT2C*	.	.	.
7	152085992	C	A	intronic	*KMT2C*	.	.	.
7	151916727	T	C	intronic	*KMT2C*	.	.	.
7	151978292	A	T	intronic	*KMT2C*	.	.	.

7	151986225	T	C	intronic	*KMT2C*	.	.	.
7	152076917	A	G	intronic	*KMT2C*	.	.	.
7	152088782	G	A	intronic	*KMT2C*	.	.	.
7	152091507	C	G	intronic	*KMT2C*	.	.	.
7	152111459	A	AG	intronic	*KMT2C*	.	.	.
7	104727931	C	T	intronic	*KMT2E*	.	.	.
12	123081995	T	C	intronic	*KNTC1*	.	.	.
13	50458175	G	A	intergenic	*KPNA3,CTAGE10P*	.	.	.
1	152746221	G	A	intergenic	*KPRP,LCE1F*	.	.	.
12	25617996	C	T	intergenic	*KRAS,LMNTD1*	.	.	.
X	46347629	TTGAATG	T	intergenic	*KRBOX4,ZNF674*	.	.	.
X	46342801	C	T	intergenic	*KRBOX4,ZNF674*	.	.	.
X	46342802	A	G	intergenic	*KRBOX4,ZNF674*	.	.	.
7	91862858	T	C	intronic	*KRIT1*	.	.	.
12	76213533	A	G	intergenic	*KRR1,PHLDA1*	.	.	.
12	76363436	A	C	intergenic	*KRR1,PHLDA1*	.	.	.
12	75913068	G	C	intergenic	*KRR1,PHLDA1*	.	.	.
12	75914733	A	G	intergenic	*KRR1,PHLDA1*	.	.	.
12	75914952	T	C	intergenic	*KRR1,PHLDA1*	.	.	.
12	75914989	T	G	intergenic	*KRR1,PHLDA1*	.	.	.
12	76012264	A	G	intergenic	*KRR1,PHLDA1*	.	.	.
12	76012295	T	C	intergenic	*KRR1,PHLDA1*	.	.	.
12	76383707	C	T	intergenic	*KRR1,PHLDA1*	.	.	.
17	39764303	A	C	intergenic	*KRT14,KRT16*	.	.	.
17	39764304	T	A	intergenic	*KRT14,KRT16*	.	.	.
17	16807944	C	T	intergenic	*KRT16P2,TNFRSF13B*	.	.	.
17	20477003	T	C	intergenic	*KRT16P3,CDRT15L2*	.	.	.
12	53377163	C	T	intergenic	*KRT18,EIF4B*	.	.	.
12	95319017	C	A	intergenic	*KRT19P2,NDUFA12*	.	.	.
12	95298952	T	G	intergenic	*KRT19P2,NDUFA12*	.	.	.
12	95277738	T	C	intergenic	*KRT19P2,NDUFA12*	.	.	.

12	95277739	G	A	intergenic	*KRT19P2,NDUFA12*	.	.	.
17	39062327	T	C	intergenic	*KRT20,KRT23*	.	.	.
17	39062328	G	A	intergenic	*KRT20,KRT23*	.	.	.
17	38876036	C	A	intergenic	*KRT24,KRT25*	.	.	.
17	38934038	A	T	intronic	*KRT27*	.	.	.
17	38971959	A	G	intergenic	*KRT28,KRT10*	.	.	.
17	39579698	G	A	intronic	*KRT37*	.	.	.
12	52982609	G	A	intronic	*KRT72*	.	.	.
12	53166783	T	G	intronic	*KRT76*	.	.	.
12	53277628	C	A	intergenic	*KRT78,KRT8*	.	.	.
12	53225586	G	A	intronic	*KRT79*	.	.	.
12	53313091	G	T	intronic	*KRT8*	.	.	.
12	52695418	G	A	upstream	*KRT86*	.	.	.
11	9147564	G	A	intergenic	*KRT8P41,DENND5A*	.	.	.
17	39488586	G	A	intergenic	*KRTAP17-1,KRT33A*	.	.	.
17	39475933	T	C	intergenic	*KRTAP17-1,KRT33A*	.	.	.
17	39475935	C	T	intergenic	*KRTAP17-1,KRT33A*	.	.	.
21	31852567	C	T	exonic	*KRTAP19-1*	nonsynonymous_SNV	.	.
21	31857834	G	T	intergenic	*KRTAP19-1,KRTAP19-2*	.	.	.
21	31879893	G	T	intergenic	*KRTAP19-5,KRTAP19-6*	.	.	.
21	31880409	C	A	intergenic	*KRTAP19-5,KRTAP19-6*	.	.	.
21	32433867	C	T	intergenic	*KRTAP19-8,TIAM1*	.	.	.
21	32031257	C	T	intergenic	*KRTAP20-3,KRTAP21-3*	.	.	.
21	32031258	A	T	intergenic	*KRTAP20-3,KRTAP21-3*	.	.	.
21	32176544	A	G	intergenic	*KRTAP21-1,KRTAP8-1*	.	.	.

21	31979682	T	C	intergenic	*KRTAP22-1,KRTAP6-1*	.	.	.
21	31741302	G	A	intergenic	*KRTAP23-1,KRTAP13-2*	.	.	.
21	31741986	T	G	intergenic	*KRTAP23-1,KRTAP13-2*	.	.	.
21	31741987	T	C	intergenic	*KRTAP23-1,KRTAP13-2*	.	.	.
17	39163919	GTATA	G	downstream	*KRTAP3-1*	.	.	.
17	39283465	G	A	intergenic	*KRTAP4-12,KRTAP4-6*	.	.	.
11	71333541	A	C	intergenic	*KRTAP5-11,FAM86C1*	.	.	.
11	71408085	T	C	intergenic	*KRTAP5-11,FAM86C1*	.	.	.
11	1648125	T	C	intergenic	*KRTAP5-4,KRTAP5-5*	.	.	.
21	31970715	T	A	downstream	*KRTAP6-2*	.	.	.
21	32211608	T	C	intergenic	*KRTAP7-1,KRTAP11-1*	.	.	.
21	32225723	T	C	intergenic	*KRTAP7-1,KRTAP11-1*	.	.	.
21	32244474	C	T	intergenic	*KRTAP7-1,KRTAP11-1*	.	.	.
21	32244475	C	G	intergenic	*KRTAP7-1,KRTAP11-1*	.	.	.
17	39420727	CAGAGAG	C	upstream	*KRTAP9-6*	.	.	.
17	39399567	C	T	intergenic	*KRTAP9-8,KRTAP9-4*	.	.	.
17	39400105	G	GT	intergenic	*KRTAP9-8,KRTAP9-4*	.	.	.
17	39401280	A	G	intergenic	*KRTAP9-8,KRTAP9-4*	.	.	.
17	25815923	G	A	intronic	*KSR1*	.	.	.
12	118195425	C	A	intronic	*KSR2*	.	.	.
12	118224850	T	A	intronic	*KSR2*	.	.	.
12	117901264	C	T	UTR3	*KSR2*	.	.	.
12	118033294	T	C	intronic	*KSR2*	.	.	.

12	118063513	T	C	intronic	*KSR2*	.	.	.
12	118063779	C	T	intronic	*KSR2*	.	.	.
12	118183951	T	A	intronic	*KSR2*	.	.	.
14	56193744	G	T	intergenic	*KTN1,RPL13AP3*	.	.	.
14	56194360	T	A	intergenic	*KTN1,RPL13AP3*	.	.	.
3	134408286	TA	T	intergenic	*KY,EPHB1*	.	.	.
3	134408288	TTA	T	intergenic	*KY,EPHB1*	.	.	.
3	134417270	A	C	intergenic	*KY,EPHB1*	.	.	.
2	143810804	T	C	intergenic	*KYNU,ARHGAP15*	.	.	.
2	143810805	G	A	intergenic	*KYNU,ARHGAP15*	.	.	.
2	143800934	T	A	intergenic	*KYNU,ARHGAP15*	.	.	.
2	143802115	T	C	intergenic	*KYNU,ARHGAP15*	.	.	.
6	130410636	G	A	intronic	*L3MBTL3*	.	.	.
18	6256668	T	C	intronic	*L3MBTL4*	.	.	.
18	6027663	A	G	intronic	*L3MBTL4*	.	.	.
18	6167096	T	G	intronic	*L3MBTL4*	.	.	.
18	6378554	A	G	intronic	*L3MBTL4*	.	.	.
13	44583516	TTCTC	T	intergenic	*LACC1,LINC00284*	.	.	.
6	108734671	G	C	intronic	*LACE1*	.	.	.
6	108771854	A	G	intronic	*LACE1*	.	.	.
6	108777950	G	A	intronic	*LACE1*	.	.	.
6	108835873	G	A	intronic	*LACE1*	.	.	.
6	108836382	G	A	intronic	*LACE1*	.	.	.
8	71539537	A	G	ncRNA_intronic	*LACTB2-AS1*	.	.	.
8	71540788	C	T	ncRNA_intronic	*LACTB2-AS1*	.	.	.
19	55032546	C	G	intergenic	*LAIR2,KIR3DX1*	.	.	.
12	48968651	T	C	intergenic	*LALBA,KANSL2*	.	.	.
12	48970119	C	T	intergenic	*LALBA,KANSL2*	.	.	.
12	48984865	T	C	intergenic	*LALBA,KANSL2*	.	.	.
18	7145820	G	A	intergenic	*LAMA1,LRRC30*	.	.	.
18	7182330	G	A	intergenic	*LAMA1,LRRC30*	.	.	.
6	129258014	C	G	intronic	*LAMA2*	.	.	.
6	129731859	TTAGATAGA	T	intronic	*LAMA2*	.	.	.

6	129372800	CATATCTATAGATCGATCTATAGATATCTATATCTATAGATCGATCTATAGATATCT	C	intronic	*LAMA2*	.	.	.
6	129435596	A	G	intronic	*LAMA2*	.	.	.
6	129526719	A	C	intronic	*LAMA2*	.	.	.
6	129229058	C	T	intronic	*LAMA2*	.	.	.
6	129229059	C	T	intronic	*LAMA2*	.	.	.
6	129544749	A	T	intronic	*LAMA2*	.	.	.
6	129708879	T	G	intronic	*LAMA2*	.	.	.
6	129862662	T	C	intergenic	*LAMA2,ARHGAP18*	.	.	.
6	129840122	C	A	intergenic	*LAMA2,ARHGAP18*	.	.	.
6	129894127	C	T	intergenic	*LAMA2,ARHGAP18*	.	.	.
6	129895016	G	A	intergenic	*LAMA2,ARHGAP18*	.	.	.
6	129895877	A	T	intergenic	*LAMA2,ARHGAP18*	.	.	.
7	107565454	G	T	intronic	*LAMB1*	.	.	.
3	49164558	A	G	intronic	*LAMB2*	.	.	.
1	209802048	AAGAG	A	intronic	*LAMB3*	.	.	.
1	183141218	C	A	intergenic	*LAMC1,LAMC2*	.	.	.
1	183169822	A	AAGATC	intronic	*LAMC2*	.	.	.
2	211198837	C	G	ncRNA_intronic	*LANCL1-AS1*	.	.	.
2	211198838	A	G	ncRNA_intronic	*LANCL1-AS1*	.	.	.
2	211199124	T	C	ncRNA_intronic	*LANCL1-AS1*	.	.	.
2	211211673	A	C	ncRNA_intronic	*LANCL1-AS1*	.	.	.
2	211267734	A	G	ncRNA_intronic	*LANCL1-AS1*	.	.	.
2	211268337	G	C	ncRNA_intronic	*LANCL1-AS1*	.	.	.
2	211270440	A	G	ncRNA_intronic	*LANCL1-AS1*	.	.	.
2	211315921	A	G	ncRNA_intronic	*LANCL1-AS1*	.	.	.
X	37527510	CTATA	C	intronic	*LANCL3*	.	.	.
X	37442168	A	G	intronic	*LANCL3*	.	.	.

X	37443204	T	G	intronic	*LANCL3*	.	.	.
X	37489506	C	T	intronic	*LANCL3*	.	.	.
4	17595543	TTGTTTTTTCACACAACAAAACCTACTGAGATTTTGATTGAGATAGATTTTGTGTGTGTGTAAAAATGTGC	T	intronic	*LAP3*	.	.	.
4	17596882	C	T	intronic	*LAP3*	.	.	.
4	17598208	C	T	intronic	*LAP3*	.	.	.
2	20298681	A	G	intergenic	*LAPTM4A,SDC1*	.	.	.
22	33795374	A	T	intronic	*LARGE*	.	.	.
22	34039211	G	A	intronic	*LARGE*	.	.	.
22	34889439	G	T	intergenic	*LARGE,ISX*	.	.	.
22	34560740	C	A	intergenic	*LARGE,ISX*	.	.	.
22	34694962	A	T	intergenic	*LARGE,ISX*	.	.	.
22	35241444	A	ATATATATAT	intergenic	*LARGE,ISX*	.	.	.
22	34953478	G	T	intergenic	*LARGE,ISX*	.	.	.
22	34953479	G	T	intergenic	*LARGE,ISX*	.	.	.
22	35025784	C	T	intergenic	*LARGE,ISX*	.	.	.
22	34577973	C	A	intergenic	*LARGE,ISX*	.	.	.
22	35241444	A	ATATATATAT	intergenic	*LARGE,ISX*	.	.	.
22	34540116	C	G	intergenic	*LARGE,ISX*	.	.	.
22	34648966	C	A	intergenic	*LARGE,ISX*	.	.	.
22	34648967	C	T	intergenic	*LARGE,ISX*	.	.	.
22	34763037	G	A	intergenic	*LARGE,ISX*	.	.	.
22	34783781	A	T	intergenic	*LARGE,ISX*	.	.	.
22	34785812	C	T	intergenic	*LARGE,ISX*	.	.	.
22	34956068	T	C	intergenic	*LARGE,ISX*	.	.	.
22	35009538	A	G	intergenic	*LARGE,ISX*	.	.	.
22	35143318	G	A	intergenic	*LARGE,ISX*	.	.	.
22	35145594	A	G	intergenic	*LARGE,ISX*	.	.	.
22	35422315	C	T	intergenic	*LARGE,ISX*	.	.	.
22	35422498	A	G	intergenic	*LARGE,ISX*	.	.	.
22	35424238	C	T	intergenic	*LARGE,ISX*	.	.	.

5	154116880	T	C	intronic	*LARP1*	.	.	.
5	154116881	G	A	intronic	*LARP1*	.	.	.
12	50866798	A	G	intronic	*LARP4*	.	.	.
10	936963	A	G	intronic	*LARP4B*	.	.	.
10	866437	C	T	intronic	*LARP4B*	.	.	.
4	113660631	G	A	intergenic	*LARP7,ANK2*	.	.	.
3	45471781	A	T	intronic	*LARS2*	.	.	.
11	111422737	GTGTATA	G	intronic	*LAYN*	.	.	.
20	37040267	G	A	intergenic	*LBP,SNHG17*	.	.	.
6	80323551	T	C	intergenic	*LCA5,SH3BGRL2*	.	.	.
6	80072528	T	A	intergenic	*LCAL1,LCA5*	.	.	.
6	80072530	C	G	intergenic	*LCAL1,LCA5*	.	.	.
6	80073041	A	C	intergenic	*LCAL1,LCA5*	.	.	.
6	80075482	C	T	intergenic	*LCAL1,LCA5*	.	.	.
6	80075483	A	G	intergenic	*LCAL1,LCA5*	.	.	.
6	80076118	C	G	intergenic	*LCAL1,LCA5*	.	.	.
6	80077783	A	G	intergenic	*LCAL1,LCA5*	.	.	.
1	152807040	A	C	intergenic	*LCE1A,LCE6A*	.	.	.
1	152784998	ACC	A	exonic	*LCE1B*	frameshift_deletion	.	.
1	152791724	C	A	intergenic	*LCE1B,LCE1A*	.	.	.
1	152662079	T	A	intergenic	*LCE2B,LCE2A*	.	.	.
1	152589216	G	A	intergenic	*LCE3B,LCE3A*	.	.	.
2	30691230	G	T	intronic	*LCLAT1*	.	.	.
2	30732003	A	G	intronic	*LCLAT1*	.	.	.
2	30941780	C	G	intergenic	*LCLAT1,CAPN13*	.	.	.
2	30942563	T	C	intergenic	*LCLAT1,CAPN13*	.	.	.
9	138436637	T	A	intergenic	*LCN1,OBP2A*	.	.	.
10	98734279	G	C	intergenic	*LCOR,C10orf12*	.	.	.
4	17948970	A	G	intronic	*LCORL*	.	.	.
4	17851992	C	T	intronic	*LCORL*	.	.	.
4	17851993	A	G	intronic	*LCORL*	.	.	.
4	19200472	T	A	intergenic	*LCORL,SLIT2*	.	.	.
4	19353431	A	G	intergenic	*LCORL,SLIT2*	.	.	.
4	19702734	C	G	intergenic	*LCORL,SLIT2*	.	.	.
4	18738556	C	T	intergenic	*LCORL,SLIT2*	.	.	.
4	18505574	G	C	intergenic	*LCORL,SLIT2*	.	.	.
4	18508487	TGGTCAGAAG	T	intergenic	*LCORL,SLIT2*	.	.	.

4	19611965	C	A	intergenic	*LCORL,SLIT2*	.	.	.
4	19709031	T	G	intergenic	*LCORL,SLIT2*	.	.	.
4	18343338	T	C	intergenic	*LCORL,SLIT2*	.	.	.
4	19822058	C	A	intergenic	*LCORL,SLIT2*	.	.	.
4	19920001	A	G	intergenic	*LCORL,SLIT2*	.	.	.
4	18964649	A	T	intergenic	*LCORL,SLIT2*	.	.	.
4	19731655	G	C	intergenic	*LCORL,SLIT2*	.	.	.
4	20094980	CTA	C	intergenic	*LCORL,SLIT2*	.	.	.
4	18212033	G	A	intergenic	*LCORL,SLIT2*	.	.	.
4	18212035	G	T	intergenic	*LCORL,SLIT2*	.	.	.
4	18222200	C	T	intergenic	*LCORL,SLIT2*	.	.	.
4	18493083	G	A	intergenic	*LCORL,SLIT2*	.	.	.
4	18493084	G	A	intergenic	*LCORL,SLIT2*	.	.	.
4	18500371	T	C	intergenic	*LCORL,SLIT2*	.	.	.
4	18500372	T	C	intergenic	*LCORL,SLIT2*	.	.	.
4	18550359	A	G	intergenic	*LCORL,SLIT2*	.	.	.
4	18591592	T	C	intergenic	*LCORL,SLIT2*	.	.	.
4	18650694	C	T	intergenic	*LCORL,SLIT2*	.	.	.
4	18673030	G	T	intergenic	*LCORL,SLIT2*	.	.	.
4	18673032	G	GT	intergenic	*LCORL,SLIT2*	.	.	.
4	18703762	T	C	intergenic	*LCORL,SLIT2*	.	.	.
4	18703764	C	G	intergenic	*LCORL,SLIT2*	.	.	.
4	18967355	A	G	intergenic	*LCORL,SLIT2*	.	.	.
4	19065167	T	A	intergenic	*LCORL,SLIT2*	.	.	.
4	19066285	G	A	intergenic	*LCORL,SLIT2*	.	.	.
4	19170267	C	T	intergenic	*LCORL,SLIT2*	.	.	.
4	19340296	T	C	intergenic	*LCORL,SLIT2*	.	.	.
4	19340298	G	A	intergenic	*LCORL,SLIT2*	.	.	.
4	19340711	C	T	intergenic	*LCORL,SLIT2*	.	.	.
4	19340713	C	T	intergenic	*LCORL,SLIT2*	.	.	.
4	19419652	C	T	intergenic	*LCORL,SLIT2*	.	.	.
4	19419902	T	A	intergenic	*LCORL,SLIT2*	.	.	.
4	19595751	G	C	intergenic	*LCORL,SLIT2*	.	.	.
4	19612159	T	C	intergenic	*LCORL,SLIT2*	.	.	.
4	19720357	C	T	intergenic	*LCORL,SLIT2*	.	.	.
4	19726601	G	C	intergenic	*LCORL,SLIT2*	.	.	.
4	19866828	A	G	intergenic	*LCORL,SLIT2*	.	.	.
4	19867511	C	T	intergenic	*LCORL,SLIT2*	.	.	.

4	19920928	A	G	intergenic	*LCORL,SLIT2*	.	.	.
4	19920939	A	G	intergenic	*LCORL,SLIT2*	.	.	.
4	20082147	A	C	intergenic	*LCORL,SLIT2*	.	.	.
4	20082333	A	G	intergenic	*LCORL,SLIT2*	.	.	.
5	169739984	A	C	intergenic	*LCP2,LINC01366*	.	.	.
2	21019241	C	T	intronic	*LDAH*	.	.	.
2	21105605	A	G	intergenic	*LDAH,APOB*	.	.	.
2	21109875	T	G	intergenic	*LDAH,APOB*	.	.	.
4	16675669	C	A	intronic	*LDB2*	.	.	.
4	16535504	C	A	intronic	*LDB2*	.	.	.
4	16563182	C	A	intronic	*LDB2*	.	.	.
4	16631622	C	G	intronic	*LDB2*	.	.	.
4	16631915	C	T	intronic	*LDB2*	.	.	.
4	16634827	C	G	intronic	*LDB2*	.	.	.
4	17060162	AGTATGTCTCCCTGGCCG	A	intergenic	*LDB2,LOC101929123*	.	.	.
4	17118383	GATAT	G	intergenic	*LDB2,LOC101929123*	.	.	.
12	21900929	A	C	intergenic	*LDHB,KCNJ8*	.	.	.
11	18434039	T	C	intronic	*LDHC*	.	.	.
19	11245184	G	A	downstream	*LDLR*	.	.	.
11	36195912	G	A	intronic	*LDLRAD3*	.	.	.
11	36234580	CTCTG	C	intronic	*LDLRAD3*	.	.	.
18	13334109	G	T	intronic	*LDLRAD4*	.	.	.
18	13343410	T	C	intronic	*LDLRAD4*	.	.	.
18	13351789	A	G	intronic	*LDLRAD4*	.	.	.
18	13351791	T	C	intronic	*LDLRAD4*	.	.	.
18	13352255	G	C	intronic	*LDLRAD4*	.	.	.
X	140345216	A	G	intergenic	*LDOC1,SPANXA2-OT1*	.	.	.
X	140410881	G	T	intergenic	*LDOC1,SPANXA2-OT1*	.	.	.
X	140539551	C	T	intergenic	*LDOC1,SPANXA2-OT1*	.	.	.
X	140540083	T	G	intergenic	*LDOC1,SPANXA2-OT1*	.	.	.

22	44942312	G	T	intergenic	*LDOC1L,LINC00207*	.	.	.
22	44952519	A	T	intergenic	*LDOC1L,LINC00207*	.	.	.
5	135340146	G	C	intergenic	*LECT2,TGFBI*	.	.	.
4	109161681	T	A	intergenic	*LEF1-AS1,RPL34-AS1*	.	.	.
4	109171866	T	C	intergenic	*LEF1-AS1,RPL34-AS1*	.	.	.
4	109358009	C	T	intergenic	*LEF1-AS1,RPL34-AS1*	.	.	.
4	109438113	A	G	intergenic	*LEF1-AS1,RPL34-AS1*	.	.	.
4	109438114	T	G	intergenic	*LEF1-AS1,RPL34-AS1*	.	.	.
1	65930462	A	T	intronic	*LEPR*	.	.	.
1	66011410	T	G	intronic	*LEPR*	.	.	.
1	66122416	T	TA	intergenic	*LEPR,PDE4B*	.	.	.
1	66120061	G	C	intergenic	*LEPR,PDE4B*	.	.	.
1	66244286	C	T	intergenic	*LEPR,PDE4B*	.	.	.
22	38078377	T	G	intergenic	*LGALS1,NOL12*	.	.	.
19	40119715	A	T	intergenic	*LGALS13,LOC100129935*	.	.	.
19	40205961	A	G	intergenic	*LGALS14,CLC*	.	.	.
19	40213337	G	T	intergenic	*LGALS14,CLC*	.	.	.
19	40215791	T	C	intergenic	*LGALS14,CLC*	.	.	.
19	40215792	G	A	intergenic	*LGALS14,CLC*	.	.	.
19	40158942	A	G	intergenic	*LGALS16,LGALS17A*	.	.	.
19	40160614	T	C	intergenic	*LGALS16,LGALS17A*	.	.	.
17	26009893	C	T	intergenic	*LGALS9,NOS2*	.	.	.
17	20371026	T	C	upstream	*LGALS9B*	.	.	.
10	95543006	G	A	intronic	*LGI1*	.	.	.
14	93225756	G	T	intergenic	*LGMN,GOLGA5*	.	.	.
11	27407392	A	G	intronic	*LGR4*	.	.	.
1	202260073	C	T	intronic	*LGR6*	.	.	.
6	64012858	C	G	intronic	*LGSN*	.	.	.

6	64016608	A	C	intronic	*LGSN*	.	.	.
6	64105224	A	G	intergenic	*LGSN,PTP4A1*	.	.	.
6	64035037	T	A	intergenic	*LGSN,PTP4A1*	.	.	.
6	64067129	C	G	intergenic	*LGSN,PTP4A1*	.	.	.
6	64074518	G	C	intergenic	*LGSN,PTP4A1*	.	.	.
6	64076995	A	G	intergenic	*LGSN,PTP4A1*	.	.	.
6	64195306	G	GT	intergenic	*LGSN,PTP4A1*	.	.	.
6	64270063	G	T	intergenic	*LGSN,PTP4A1*	.	.	.
2	48945849	C	T	intronic	*LHCGR,STON1-GTF2A1L*	.	.	.
2	48966305	A	G	intronic	*LHCGR,STON1-GTF2A1L*	.	.	.
13	40168169	T	A	intronic	*LHFP*	.	.	.
13	40208155	A	T	intergenic	*LHFP,COG6*	.	.	.
13	40219444	T	C	intergenic	*LHFP,COG6*	.	.	.
X	111943603	G	A	intergenic	*LHFPL1,AMOT*	.	.	.
X	111944167	C	A	intergenic	*LHFPL1,AMOT*	.	.	.
5	77970530	T	G	intergenic	*LHFPL2,ARSB*	.	.	.
5	77979920	C	T	intergenic	*LHFPL2,ARSB*	.	.	.
5	77982678	A	G	intergenic	*LHFPL2,ARSB*	.	.	.
5	78010663	C	T	intergenic	*LHFPL2,ARSB*	.	.	.
7	104041272	T	C	intronic	*LHFPL3*	.	.	.
7	104011236	G	A	intronic	*LHFPL3*	.	.	.
7	104050770	G	A	intronic	*LHFPL3*	.	.	.
7	104080518	C	T	intronic	*LHFPL3*	.	.	.
7	104223949	G	C	intronic	*LHFPL3*	.	.	.
7	104248513	A	G	intronic	*LHFPL3*	.	.	.
7	104248514	C	T	intronic	*LHFPL3*	.	.	.
7	104256933	C	T	intronic	*LHFPL3*	.	.	.
7	104257452	A	G	intronic	*LHFPL3*	.	.	.
7	104360817	T	C	intronic	*LHFPL3*	.	.	.
7	104371623	C	T	intronic	*LHFPL3*	.	.	.
7	104373849	T	G	intronic	*LHFPL3*	.	.	.
7	104471042	C	T	intronic	*LHFPL3*	.	.	.
7	104508427	T	C	intronic	*LHFPL3*	.	.	.
7	104516729	A	G	intronic	*LHFPL3*	.	.	.
3	9563524	A	G	intronic	*LHFPL4*	.	.	.
17	35301882	T	A	UTR3	*LHX1*	.	.	.

12	114139417	ATCATATATATATCATATATATATC	A	intergenic	*LHX5-AS1,LINC01234*	.	.	.
12	113979629	C	A	intergenic	*LHX5-AS1,LINC01234*	.	.	.
12	113979630	T	A	intergenic	*LHX5-AS1,LINC01234*	.	.	.
12	113989814	G	T	intergenic	*LHX5-AS1,LINC01234*	.	.	.
12	113971166	G	A	intergenic	*LHX5-AS1,LINC01234*	.	.	.
12	113976083	T	C	intergenic	*LHX5-AS1,LINC01234*	.	.	.
1	75642133	C	T	intergenic	*LHX8,SLC44A5*	.	.	.
1	75642135	G	T	intergenic	*LHX8,SLC44A5*	.	.	.
1	75644341	C	T	intergenic	*LHX8,SLC44A5*	.	.	.
1	197908973	A	T	intergenic	*LHX9,NEK7*	.	.	.
1	198107572	T	C	intergenic	*LHX9,NEK7*	.	.	.
1	198107574	A	C	intergenic	*LHX9,NEK7*	.	.	.
5	38516620	T	C	intronic	*LIFR*	.	.	.
5	38549888	G	A	intronic	*LIFR*	.	.	.
19	55093220	C	T	intronic	*LILRA2*	.	.	.
19	55143769	GTGGGATGATGT	G	intronic	*LILRB1*	.	.	.
19	54737679	A	G	intronic	*LILRB3*	.	.	.
19	54763156	T	TAATA	intergenic	*LILRB5,LILRB2*	.	.	.
4	41577901	T	C	intronic	*LIMCH1*	.	.	.
4	41390969	G	GT	intronic	*LIMCH1*	.	.	.
4	41475008	T	C	intronic	*LIMCH1*	.	.	.
4	41400293	A	G	intronic	*LIMCH1*	.	.	.
4	41403930	G	C	intronic	*LIMCH1*	.	.	.
4	41469933	C	T	intronic	*LIMCH1*	.	.	.
4	41551088	A	T	intronic	*LIMCH1*	.	.	.
7	73555074	C	A	intergenic	*LIMK1,EIF4H*	.	.	.
2	109213394	T	TAGACA	intronic	*LIMS1*	.	.	.
2	109265261	T	G	intronic	*LIMS1*	.	.	.
2	128416843	T	C	intronic	*LIMS2*	.	.	.

6	105495052	A	C	intronic	*LIN28B*	.	.	.
12	81194193	G	A	intronic	*LIN7A*	.	.	.
12	81447682	T	A	intergenic	*LIN7A,ACSS3*	.	.	.
12	81450838	A	G	intergenic	*LIN7A,ACSS3*	.	.	.
12	81451786	T	C	intergenic	*LIN7A,ACSS3*	.	.	.
12	81453072	T	G	intergenic	*LIN7A,ACSS3*	.	.	.
12	81453183	G	C	intergenic	*LIN7A,ACSS3*	.	.	.
12	81454060	T	C	intergenic	*LIN7A,ACSS3*	.	.	.
12	81461142	C	T	intergenic	*LIN7A,ACSS3*	.	.	.
12	81463107	T	A	intergenic	*LIN7A,ACSS3*	.	.	.
12	81463718	T	G	intergenic	*LIN7A,ACSS3*	.	.	.
1	226474593	C	G	intronic	*LIN9*	.	.	.
18	54766604	A	AG	intergenic	*LINC-ROR,BOD1L2*	.	.	.
18	54768735	T	G	intergenic	*LINC-ROR,BOD1L2*	.	.	.
18	54768737	C	T	intergenic	*LINC-ROR,BOD1L2*	.	.	.
9	27254306	T	C	ncRNA_intronic	*LINC00032*	.	.	.
9	27256215	C	A	ncRNA_intronic	*LINC00032*	.	.	.
15	88370675	C	A	intergenic	*LINC00052,NTRK3*	.	.	.
15	88382967	T	A	intergenic	*LINC00052,NTRK3*	.	.	.
21	29105052	G	T	ncRNA_intronic	*LINC00113,MIR5009*	.	.	.
2	110984686	TTTTTA	T	intergenic	*LINC00116,LOC100507334*	.	.	.
2	110984960	C	T	intergenic	*LINC00116,LOC100507334*	.	.	.
21	26807765	A	G	intergenic	*LINC00158,MIR155HG*	.	.	.
21	26807766	A	G	intergenic	*LINC00158,MIR155HG*	.	.	.
21	26808953	G	A	intergenic	*LINC00158,MIR155HG*	.	.	.
21	26815297	G	A	intergenic	*LINC00158,MIR155HG*	.	.	.
21	33456616	T	C	ncRNA_intronic	*LINC00159*	.	.	.

21	33595749	C	T	intergenic	*LINC00159,MIS18A*	.	.	.
21	33595750	T	A	intergenic	*LINC00159,MIS18A*	.	.	.
21	29924560	G	A	intergenic	*LINC00161,N6AMT1*	.	.	.
21	29972360	A	G	intergenic	*LINC00161,N6AMT1*	.	.	.
21	30001200	G	A	intergenic	*LINC00161,N6AMT1*	.	.	.
21	30206367	T	A	intergenic	*LINC00161,N6AMT1*	.	.	.
21	29992850	C	T	intergenic	*LINC00161,N6AMT1*	.	.	.
21	29993366	A	T	intergenic	*LINC00161,N6AMT1*	.	.	.
21	29993681	G	A	intergenic	*LINC00161,N6AMT1*	.	.	.
10	27275147	T	C	intergenic	*LINC00202-1,ANKRD26*	.	.	.
10	26945477	C	T	intergenic	*LINC00202-2,PDSS1*	.	.	.
1	218072416	T	C	ncRNA_intronic	*LINC00210*	.	.	.
1	218141173	A	G	intergenic	*LINC00210,LOC101929631*	.	.	.
1	218117867	CT	C	intergenic	*LINC00210,LOC101929631*	.	.	.
1	218117869	CT	C	intergenic	*LINC00210,LOC101929631*	.	.	.
1	218143324	C	T	intergenic	*LINC00210,LOC101929631*	.	.	.
14	106985730	G	A	intergenic	*LINC00221,MIR7641-2*	.	.	.
14	107011579	AC	A	intergenic	*LINC00221,MIR7641-2*	.	.	.
14	107011581	G	T	intergenic	*LINC00221,MIR7641-2*	.	.	.

6	109134202	A	T	intergenic	*LINC00222,ARMC2*	.	.	.
6	109134681	C	T	intergenic	*LINC00222,ARMC2*	.	.	.
6	109134683	T	A	intergenic	*LINC00222,ARMC2*	.	.	.
6	109138226	T	C	intergenic	*LINC00222,ARMC2*	.	.	.
14	106781423	C	T	intergenic	*LINC00226,LINC00221*	.	.	.
14	106830163	G	T	intergenic	*LINC00226,LINC00221*	.	.	.
14	106755800	A	C	intergenic	*LINC00226,LINC00221*	.	.	.
14	106818689	C	G	intergenic	*LINC00226,LINC00221*	.	.	.
22	45005098	G	T	ncRNA_intronic	*LINC00229*	.	.	.
22	45049016	AGATGAAAGTGAAGATGTAGATTGTAAAGAAGGTGAT	A	intergenic	*LINC00229,PRR5*	.	.	.
14	66953308	GTCTC	G	ncRNA_intronic	*LINC00238*	.	.	.
6	26978318	G	A	ncRNA_intronic	*LINC00240*	.	.	.
6	26979218	C	A	ncRNA_intronic	*LINC00240*	.	.	.
6	26980724	C	T	ncRNA_intronic	*LINC00240*	.	.	.
8	66348909	A	G	intergenic	*LINC00251,LINC01299*	.	.	.
8	66124913	T	C	intergenic	*LINC00251,LINC01299*	.	.	.
8	66374400	A	T	intergenic	*LINC00251,LINC01299*	.	.	.
8	66295134	T	C	intergenic	*LINC00251,LINC01299*	.	.	.
8	66295557	A	C	intergenic	*LINC00251,LINC01299*	.	.	.
8	66303825	G	A	intergenic	*LINC00251,LINC01299*	.	.	.

8	66342722	C	G	intergenic	*LINC00251,LINC01299*	.	.	.
8	66342723	A	G	intergenic	*LINC00251,LINC01299*	.	.	.
8	66344094	C	A	intergenic	*LINC00251,LINC01299*	.	.	.
8	66350741	A	C	intergenic	*LINC00251,LINC01299*	.	.	.
8	66350879	T	A	intergenic	*LINC00251,LINC01299*	.	.	.
8	66416611	A	ATTTAG	intergenic	*LINC00251,LINC01299*	.	.	.
16	1937074	G	A	intergenic	*LINC00254,HS3ST6*	.	.	.
7	39801087	A	T	ncRNA_intronic	*LINC00265*	.	.	.
7	39914298	T	C	intergenic	*LINC00265,CDK13*	.	.	.
7	39979872	G	A	intergenic	*LINC00265,CDK13*	.	.	.
20	62963146	C	T	intergenic	*LINC00266-1,NONE*	.	.	.
X	68422660	A	T	ncRNA_intronic	*LINC00269*	.	.	.
X	68434480	C	T	intergenic	*LINC00269,FAM155B*	.	.	.
X	68648177	T	C	intergenic	*LINC00269,FAM155B*	.	.	.
X	68509731	C	G	intergenic	*LINC00269,FAM155B*	.	.	.
X	68595216	T	A	intergenic	*LINC00269,FAM155B*	.	.	.
X	68573263	T	C	intergenic	*LINC00269,FAM155B*	.	.	.
X	68678725	A	T	intergenic	*LINC00269,FAM155B*	.	.	.
X	68678727	A	G	intergenic	*LINC00269,FAM155B*	.	.	.
X	68679249	C	T	intergenic	*LINC00269,FAM155B*	.	.	.
X	68679478	A	T	intergenic	*LINC00269,FAM155B*	.	.	.

X	68682395	G	T	intergenic	*LINC00269,FAM155B*	.	.	.
X	68703983	A	G	intergenic	*LINC00269,FAM155B*	.	.	.
X	68704802	A	G	intergenic	*LINC00269,FAM155B*	.	.	.
6	135932898	C	T	ncRNA_intronic	*LINC00271*	.	.	.
6	135880514	C	A	ncRNA_intronic	*LINC00271*	.	.	.
6	135881937	G	A	ncRNA_intronic	*LINC00271*	.	.	.
6	136063077	T	A	intergenic	*LINC00271,PDE7B*	.	.	.
1	182410632	T	C	intergenic	*LINC00272,RGSL1*	.	.	.
1	182391750	G	A	intergenic	*LINC00272,RGSL1*	.	.	.
1	182391752	G	A	intergenic	*LINC00272,RGSL1*	.	.	.
16	34175963	A	C	intergenic	*LINC00273,UBE2MP1*	.	.	.
16	34009977	G	C	intergenic	*LINC00273,UBE2MP1*	.	.	.
16	34009978	A	T	intergenic	*LINC00273,UBE2MP1*	.	.	.
16	33970405	A	C	intergenic	*LINC00273,UBE2MP1*	.	.	.
16	34180085	G	C	intergenic	*LINC00273,UBE2MP1*	.	.	.
16	33979553	C	T	intergenic	*LINC00273,UBE2MP1*	.	.	.
16	33996937	T	A	intergenic	*LINC00273,UBE2MP1*	.	.	.
16	33969283	G	C	intergenic	*LINC00273,UBE2MP1*	.	.	.
16	34393821	C	T	intergenic	*LINC00273,UBE2MP1*	.	.	.
2	14387900	G	A	ncRNA_intronic	*LINC00276*	.	.	.
2	14387901	T	C	ncRNA_intronic	*LINC00276*	.	.	.
2	14710855	TTAGATAGA	T	intergenic	*LINC00276,FAM84A*	.	.	.
2	14676997	A	C	intergenic	*LINC00276,FAM84A*	.	.	.

2	14565640	G	T	intergenic	*LINC00276,FAM84A*	.	.	.
2	14546243	C	A	intergenic	*LINC00276,FAM84A*	.	.	.
2	14579176	A	G	intergenic	*LINC00276,FAM84A*	.	.	.
2	14579506	G	T	intergenic	*LINC00276,FAM84A*	.	.	.
2	14630699	G	A	intergenic	*LINC00276,FAM84A*	.	.	.
2	14691669	T	C	intergenic	*LINC00276,FAM84A*	.	.	.
2	14691671	T	C	intergenic	*LINC00276,FAM84A*	.	.	.
13	44656556	T	C	intergenic	*LINC00284,LINC00390*	.	.	.
4	182014658	A	G	ncRNA_intronic	*LINC00290*	.	.	.
4	182547525	A	G	intergenic	*LINC00290,LOC90768*	.	.	.
4	182361797	A	G	intergenic	*LINC00290,LOC90768*	.	.	.
4	182194804	C	T	intergenic	*LINC00290,LOC90768*	.	.	.
4	182564073	C	G	intergenic	*LINC00290,LOC90768*	.	.	.
4	182702517	G	A	intergenic	*LINC00290,LOC90768*	.	.	.
4	182841635	A	C	intergenic	*LINC00290,LOC90768*	.	.	.
4	182853979	G	A	intergenic	*LINC00290,LOC90768*	.	.	.
4	182954889	G	A	intergenic	*LINC00290,LOC90768*	.	.	.
8	47989336	TAA	T	intergenic	*LINC00293,LOC100287846*	.	.	.
8	48018852	G	A	intergenic	*LINC00293,LOC100287846*	.	.	.

8	48022780	A	G	intergenic	*LINC00293,LOC100287846*	.	.	.
8	47807059	C	T	intergenic	*LINC00293,LOC100287846*	.	.	.
8	47809025	A	G	intergenic	*LINC00293,LOC100287846*	.	.	.
8	47810937	T	C	intergenic	*LINC00293,LOC100287846*	.	.	.
8	47856823	T	C	intergenic	*LINC00293,LOC100287846*	.	.	.
8	47859331	G	C	intergenic	*LINC00293,LOC100287846*	.	.	.
8	47934856	C	G	intergenic	*LINC00293,LOC100287846*	.	.	.
8	47935661	G	A	intergenic	*LINC00293,LOC100287846*	.	.	.
8	48066265	A	AT	intergenic	*LINC00293,LOC100287846*	.	.	.
8	48074732	C	G	intergenic	*LINC00293,LOC100287846*	.	.	.
2	8099001	G	T	ncRNA_intronic	*LINC00298*	.	.	.
2	8232342	G	GAAAGAAA[illegible]	ncRNA_intronic	*LINC00299*	.	.	.
2	8211204	A	T	ncRNA_intronic	*LINC00299*	.	.	.
2	8271272	G	A	ncRNA_intronic	*LINC00299*	.	.	.
2	8526158	A	T	intergenic	*LINC00299,LOC101929567*	.	.	.
11	60461328	C	G	intergenic	*LINC00301,MS4A8*	.	.	.
21	24113990	T	A	intergenic	*LINC00308,D21S2088E*	.	.	.
21	24083253	G	T	intergenic	*LINC00308,D21S2088E*	.	.	.
21	24125126	T	C	intergenic	*LINC00308,D21S2088E*	.	.	.
21	24223637	C	A	intergenic	*LINC00308,D21S2088E*	.	.	.
21	24223638	T	A	intergenic	*LINC00308,D21S2088E*	.	.	.

21	24729967	C	CA	intergenic	*LINC00308,D21S2088E*	.	.	.
21	24606338	T	C	intergenic	*LINC00308,D21S2088E*	.	.	.
21	24199825	A	G	intergenic	*LINC00308,D21S2088E*	.	.	.
21	24038927	C	T	intergenic	*LINC00308,D21S2088E*	.	.	.
21	24281870	A	G	intergenic	*LINC00308,D21S2088E*	.	.	.
21	24316534	A	T	intergenic	*LINC00308,D21S2088E*	.	.	.
21	24445441	T	C	intergenic	*LINC00308,D21S2088E*	.	.	.
2	64436578	C	G	intergenic	*LINC00309,LOC100507006*	.	.	.
2	64436579	T	G	intergenic	*LINC00309,LOC100507006*	.	.	.
3	8657534	G	T	intergenic	*LINC00312,SSUH2*	.	.	.
21	23101224	T	TA	ncRNA_intronic	*LINC00317*	.	.	.
21	23101225	C	A	ncRNA_intronic	*LINC00317*	.	.	.
21	23094701	TTTTTGTTTTG	T	downstream	*LINC00317*	.	.	.
21	22188580	A	C	intergenic	*LINC00320,NCAM2*	.	.	.
21	22263688	G	A	intergenic	*LINC00320,NCAM2*	.	.	.
21	22190593	A	C	intergenic	*LINC00320,NCAM2*	.	.	.
21	22296543	A	G	intergenic	*LINC00320,NCAM2*	.	.	.
21	22309564	T	C	intergenic	*LINC00320,NCAM2*	.	.	.
21	22309565	T	C	intergenic	*LINC00320,NCAM2*	.	.	.
21	22327565	T	C	intergenic	*LINC00320,NCAM2*	.	.	.

21	22327566	G	A	intergenic	*LINC00320,NCAM2*	.	.	.
21	22331669	A	T	intergenic	*LINC00320,NCAM2*	.	.	.
6	133439511	T	C	intergenic	*LINC00326,EYA4*	.	.	.
6	133439985	A	G	intergenic	*LINC00326,EYA4*	.	.	.
6	133439986	C	A	intergenic	*LINC00326,EYA4*	.	.	.
6	133440710	A	T	intergenic	*LINC00326,EYA4*	.	.	.
6	133442201	A	T	intergenic	*LINC00326,EYA4*	.	.	.
6	133443086	A	G	intergenic	*LINC00326,EYA4*	.	.	.
6	133464956	G	T	intergenic	*LINC00326,EYA4*	.	.	.
6	133473327	C	T	intergenic	*LINC00326,EYA4*	.	.	.
6	133473328	C	G	intergenic	*LINC00326,EYA4*	.	.	.
13	24064972	C	T	intergenic	*LINC00327,TNFRSF19*	.	.	.
13	79546584	A	G	intergenic	*LINC00331,RBM26*	.	.	.
13	79555842	T	C	intergenic	*LINC00331,RBM26*	.	.	.
13	79501464	G	C	intergenic	*LINC00331,RBM26*	.	.	.
13	79544241	A	C	intergenic	*LINC00331,RBM26*	.	.	.
13	79779689	T	A	intergenic	*LINC00331,RBM26*	.	.	.
13	79502510	C	A	intergenic	*LINC00331,RBM26*	.	.	.
13	79891125	T	TTTAA	intergenic	*LINC00331,RBM26*	.	.	.
13	79423991	G	A	intergenic	*LINC00331,RBM26*	.	.	.
13	79430143	C	A	intergenic	*LINC00331,RBM26*	.	.	.
13	79433361	T	A	intergenic	*LINC00331,RBM26*	.	.	.
13	79498626	T	C	intergenic	*LINC00331,RBM26*	.	.	.
13	79498725	A	G	intergenic	*LINC00331,RBM26*	.	.	.
13	79499150	G	C	intergenic	*LINC00331,RBM26*	.	.	.
13	79524110	C	T	intergenic	*LINC00331,RBM26*	.	.	.
13	79688110	C	T	intergenic	*LINC00331,RBM26*	.	.	.
13	79810096	A	C	intergenic	*LINC00331,RBM26*	.	.	.
13	79811456	G	A	intergenic	*LINC00331,RBM26*	.	.	.
13	79824301	G	A	intergenic	*LINC00331,RBM26*	.	.	.
13	79875179	G	A	intergenic	*LINC00331,RBM26*	.	.	.
13	84994150	T	C	ncRNA_intronic	*LINC00333*	.	.	.
13	84915217	A	T	ncRNA_intronic	*LINC00333*	.	.	.
13	84936058	A	G	ncRNA_intronic	*LINC00333*	.	.	.

13	85270476	A	G	intergenic	*LINC00333,SNORA107*	.	.	.
13	85272679	T	G	intergenic	*LINC00333,SNORA107*	.	.	.
2	96485882	G	T	ncRNA_intronic	*LINC00342*	.	.	.
13	106406067	T	C	ncRNA_intronic	*LINC00343*	.	.	.
13	106952654	A	G	intergenic	*LINC00343,LINC00460*	.	.	.
13	106831863	T	C	intergenic	*LINC00343,LINC00460*	.	.	.
13	106815221	A	G	intergenic	*LINC00343,LINC00460*	.	.	.
13	106474791	A	T	intergenic	*LINC00343,LINC00460*	.	.	.
13	106510164	G	T	intergenic	*LINC00343,LINC00460*	.	.	.
13	106781329	G	T	intergenic	*LINC00343,LINC00460*	.	.	.
13	106913678	G	A	intergenic	*LINC00343,LINC00460*	.	.	.
13	106964248	T	A	intergenic	*LINC00343,LINC00460*	.	.	.
13	106538492	T	C	intergenic	*LINC00343,LINC00460*	.	.	.
13	106539840	C	T	intergenic	*LINC00343,LINC00460*	.	.	.
13	106539842	A	G	intergenic	*LINC00343,LINC00460*	.	.	.
13	106568127	G	C	intergenic	*LINC00343,LINC00460*	.	.	.
13	106615936	A	G	intergenic	*LINC00343,LINC00460*	.	.	.
13	75366982	C	T	intergenic	*LINC00347,CTAGE11P*	.	.	.
13	75431783	T	C	intergenic	*LINC00347,CTAGE11P*	.	.	.
13	75507892	A	T	intergenic	*LINC00347,CTAGE11P*	.	.	.

13	75507893	G	C	intergenic	*LINC00347,CTAGE11P*	.	.	.
13	75508341	T	C	intergenic	*LINC00347,CTAGE11P*	.	.	.
13	75510475	A	T	intergenic	*LINC00347,CTAGE11P*	.	.	.
13	75538376	T	G	intergenic	*LINC00347,CTAGE11P*	.	.	.
13	75580271	T	A	intergenic	*LINC00347,CTAGE11P*	.	.	.
13	75592093	G	C	intergenic	*LINC00347,CTAGE11P*	.	.	.
13	75606484	C	T	intergenic	*LINC00347,CTAGE11P*	.	.	.
13	75607600	A	G	intergenic	*LINC00347,CTAGE11P*	.	.	.
13	75654601	C	A	intergenic	*LINC00347,CTAGE11P*	.	.	.
13	75682244	A	T	intergenic	*LINC00347,CTAGE11P*	.	.	.
13	75773025	C	T	intergenic	*LINC00347,CTAGE11P*	.	.	.
13	71610459	A	C	ncRNA_intronic	*LINC00348*	.	.	.
13	71702543	A	G	ncRNA_intronic	*LINC00348*	.	.	.
13	71705926	A	G	ncRNA_intronic	*LINC00348*	.	.	.
13	71917293	CTATATA	C	intergenic	*LINC00348,DACH1*	.	.	.
13	71779404	G	T	intergenic	*LINC00348,DACH1*	.	.	.
13	71751496	A	G	intergenic	*LINC00348,DACH1*	.	.	.
13	71751609	G	A	intergenic	*LINC00348,DACH1*	.	.	.
13	71751891	A	G	intergenic	*LINC00348,DACH1*	.	.	.
13	71760018	T	G	intergenic	*LINC00348,DACH1*	.	.	.
13	86103027	T	C	ncRNA_intronic	*LINC00351*	.	.	.
13	86058285	G	C	ncRNA_intronic	*LINC00351*	.	.	.
13	85963614	G	A	ncRNA_intronic	*LINC00351*	.	.	.
13	86025591	G	A	ncRNA_intronic	*LINC00351*	.	.	.
13	86033694	A	AG	ncRNA_intronic	*LINC00351*	.	.	.
13	86267580	G	C	intergenic	*LINC00351,SLITRK6*	.	.	.

13	86136523	AATAGATAG	A	intergenic	*LINC00351,SLITRK6*	.	.	.
13	86268055	G	A	intergenic	*LINC00351,SLITRK6*	.	.	.
13	86287890	A	AT	intergenic	*LINC00351,SLITRK6*	.	.	.
13	86345264	T	C	intergenic	*LINC00351,SLITRK6*	.	.	.
13	90325361	G	A	intergenic	*LINC00353,LINC00559*	.	.	.
13	90333073	C	A	intergenic	*LINC00353,LINC00559*	.	.	.
13	112618550	C	T	intergenic	*LINC00354,LINC00403*	.	.	.
13	62606390	A	T	intergenic	*LINC00358,LINC01075*	.	.	.
13	97631520	G	A	ncRNA_intronic	*LINC00359*	.	.	.
13	93820801	C	T	intergenic	*LINC00363,GPC6*	.	.	.
13	93783437	G	A	intergenic	*LINC00363,GPC6*	.	.	.
13	67974670	AACCCTTAC	A	intergenic	*LINC00364,LINC00550*	.	.	.
13	69062694	A	G	intergenic	*LINC00364,LINC00550*	.	.	.
13	68814279	T	A	intergenic	*LINC00364,LINC00550*	.	.	.
13	69238205	T	A	intergenic	*LINC00364,LINC00550*	.	.	.
13	69026988	A	C	intergenic	*LINC00364,LINC00550*	.	.	.
13	68100351	G	A	intergenic	*LINC00364,LINC00550*	.	.	.
13	68139256	C	A	intergenic	*LINC00364,LINC00550*	.	.	.
13	68166698	T	C	intergenic	*LINC00364,LINC00550*	.	.	.
13	68168264	A	G	intergenic	*LINC00364,LINC00550*	.	.	.

13	68254879	A	C	intergenic	*LINC00364,LINC00550*	.	.	.
13	68255654	C	T	intergenic	*LINC00364,LINC00550*	.	.	.
13	68473920	G	T	intergenic	*LINC00364,LINC00550*	.	.	.
13	68475070	A	C	intergenic	*LINC00364,LINC00550*	.	.	.
13	68569087	C	T	intergenic	*LINC00364,LINC00550*	.	.	.
13	68588294	A	G	intergenic	*LINC00364,LINC00550*	.	.	.
13	68902400	C	T	intergenic	*LINC00364,LINC00550*	.	.	.
13	68913627	T	C	intergenic	*LINC00364,LINC00550*	.	.	.
13	68913629	G	A	intergenic	*LINC00364,LINC00550*	.	.	.
13	68914015	T	C	intergenic	*LINC00364,LINC00550*	.	.	.
13	69338315	C	T	intergenic	*LINC00364,LINC00550*	.	.	.
13	69353061	G	A	intergenic	*LINC00364,LINC00550*	.	.	.
13	69353062	T	C	intergenic	*LINC00364,LINC00550*	.	.	.
13	69365510	T	G	intergenic	*LINC00364,LINC00550*	.	.	.
13	69367682	G	A	intergenic	*LINC00364,LINC00550*	.	.	.
13	69367684	G	A	intergenic	*LINC00364,LINC00550*	.	.	.
13	111758591	T	A	downstream	*LINC00368*	.	.	.
13	59761796	T	A	intergenic	*LINC00374,DIAPH3*	.	.	.
13	59772948	C	T	intergenic	*LINC00374,DIAPH3*	.	.	.

13	58971047	T	A	intergenic	*LINC00374,DIAPH3*	.	.	.
13	59223908	G	T	intergenic	*LINC00374,DIAPH3*	.	.	.
13	59846762	T	C	intergenic	*LINC00374,DIAPH3*	.	.	.
13	59951029	C	A	intergenic	*LINC00374,DIAPH3*	.	.	.
13	59955080	C	G	intergenic	*LINC00374,DIAPH3*	.	.	.
13	59955081	T	A	intergenic	*LINC00374,DIAPH3*	.	.	.
13	58925723	G	T	intergenic	*LINC00374,DIAPH3*	.	.	.
13	59324976	C	A	intergenic	*LINC00374,DIAPH3*	.	.	.
13	59928243	G	C	intergenic	*LINC00374,DIAPH3*	.	.	.
13	58878179	C	G	intergenic	*LINC00374,DIAPH3*	.	.	.
13	60024597	G	C	intergenic	*LINC00374,DIAPH3*	.	.	.
13	59361406	G	A	intergenic	*LINC00374,DIAPH3*	.	.	.
13	60166787	A	G	intergenic	*LINC00374,DIAPH3*	.	.	.
13	85696546	G	A	ncRNA_intronic	*LINC00375*	.	.	.
13	85696739	T	C	ncRNA_intronic	*LINC00375*	.	.	.
13	85730144	C	T	intergenic	*LINC00375,LINC00351*	.	.	.
13	85826639	G	T	intergenic	*LINC00375,LINC00351*	.	.	.
13	63837742	T	C	ncRNA_intronic	*LINC00376*	.	.	.
13	64077470	A	G	intergenic	*LINC00376,LINC00395*	.	.	.
13	64199081	A	T	intergenic	*LINC00376,LINC00395*	.	.	.

13	63990546	A	T	intergenic	*LINC00376,LINC00395*	.	.	.
13	63991717	T	C	intergenic	*LINC00376,LINC00395*	.	.	.
13	64074653	A	G	intergenic	*LINC00376,LINC00395*	.	.	.
13	64074654	A	G	intergenic	*LINC00376,LINC00395*	.	.	.
13	64228115	G	A	intergenic	*LINC00376,LINC00395*	.	.	.
13	64228481	T	A	intergenic	*LINC00376,LINC00395*	.	.	.
13	64229388	C	A	intergenic	*LINC00376,LINC00395*	.	.	.
13	81742226	T	TCTTTCTTTC	intergenic	*LINC00377,LINC00564*	.	.	.
13	81722666	C	G	intergenic	*LINC00377,LINC00564*	.	.	.
13	81742052	A	C	intergenic	*LINC00377,LINC00564*	.	.	.
13	81639145	C	A	intergenic	*LINC00377,LINC00564*	.	.	.
13	81639612	A	G	intergenic	*LINC00377,LINC00564*	.	.	.
13	61737828	C	G	intergenic	*LINC00378,MIR3169*	.	.	.
13	61632959	C	A	intergenic	*LINC00378,MIR3169*	.	.	.
13	61343569	A	G	intergenic	*LINC00378,MIR3169*	.	.	.
13	61354605	G	A	intergenic	*LINC00378,MIR3169*	.	.	.
13	91782388	A	G	ncRNA_intronic	*LINC00379*	.	.	.
13	91909529	G	T	intergenic	*LINC00379,MIR17HG*	.	.	.
13	91894089	A	ATCGG	intergenic	*LINC00379,MIR17HG*	.	.	.

13	91759313	T	C	intergenic	*LINC00380,LINC00379*	.	.	.
13	91774296	G	C	intergenic	*LINC00380,LINC00379*	.	.	.
13	75052643	ATGTGTG	A	intergenic	*LINC00381,LINC00347*	.	.	.
13	80465165	T	C	ncRNA_intronic	*LINC00382*	.	.	.
13	80465718	A	G	ncRNA_intronic	*LINC00382*	.	.	.
13	80466127	G	C	ncRNA_intronic	*LINC00382*	.	.	.
13	80495303	A	G	intergenic	*LINC00382,LINC01080*	.	.	.
13	80495304	C	T	intergenic	*LINC00382,LINC01080*	.	.	.
13	80553277	G	C	intergenic	*LINC00382,LINC01080*	.	.	.
13	80553784	A	C	intergenic	*LINC00382,LINC01080*	.	.	.
13	69908758	G	C	intergenic	*LINC00383,KLHL1*	.	.	.
13	70003369	T	C	intergenic	*LINC00383,KLHL1*	.	.	.
13	69975579	AT	A	intergenic	*LINC00383,KLHL1*	.	.	.
13	70044648	T	G	intergenic	*LINC00383,KLHL1*	.	.	.
13	70102670	C	T	intergenic	*LINC00383,KLHL1*	.	.	.
13	70250301	C	T	intergenic	*LINC00383,KLHL1*	.	.	.
13	69967121	C	T	intergenic	*LINC00383,KLHL1*	.	.	.
13	69967122	A	G	intergenic	*LINC00383,KLHL1*	.	.	.
13	70010020	T	C	intergenic	*LINC00383,KLHL1*	.	.	.
13	70243795	T	A	intergenic	*LINC00383,KLHL1*	.	.	.
13	74140381	T	C	ncRNA_intronic	*LINC00392*	.	.	.
13	110763590	A	T	intergenic	*LINC00396,COL4A1*	.	.	.
13	88520058	G	A	intergenic	*LINC00397,LOC105370306*	.	.	.
13	88630192	T	G	intergenic	*LINC00397,LOC105370306*	.	.	.
13	88687381	CT	C	intergenic	*LINC00397,LOC105370306*	.	.	.
13	88645940	C	T	intergenic	*LINC00397,LOC105370306*	.	.	.

13	88645941	T	G	intergenic	*LINC00397,LOC105370306*	.	.	.
13	110063559	G	T	intergenic	*LINC00399,LINC00676*	.	.	.
13	110326050	C	A	intergenic	*LINC00399,LINC00676*	.	.	.
13	110066775	C	T	intergenic	*LINC00399,LINC00676*	.	.	.
13	110094385	C	T	intergenic	*LINC00399,LINC00676*	.	.	.
13	110110632	T	C	intergenic	*LINC00399,LINC00676*	.	.	.
13	110156091	A	G	intergenic	*LINC00399,LINC00676*	.	.	.
13	110156092	C	T	intergenic	*LINC00399,LINC00676*	.	.	.
13	43703171	G	A	ncRNA_intronic	*LINC00400*	.	.	.
13	43769932	G	T	intergenic	*LINC00400,ENOX1*	.	.	.
13	112702188	C	T	ncRNA_intronic	*LINC00403*	.	.	.
13	112801581	G	A	intergenic	*LINC00403,LINC01070*	.	.	.
13	19488267	G	GTA	ncRNA_intronic	*LINC00408*	.	.	.
13	19495268	A	T	ncRNA_intronic	*LINC00408*	.	.	.
13	19560935	T	A	intergenic	*LINC00408,LINC00442*	.	.	.
13	19563583	G	C	intergenic	*LINC00408,LINC00442*	.	.	.
13	91683586	C	T	intergenic	*LINC00410,LINC00380*	.	.	.
13	19321373	T	C	intergenic	*LINC00417,ANKRD20A9P*	.	.	.
13	19400532	C	G	intergenic	*LINC00417,ANKRD20A9P*	.	.	.
13	19985458	TTGG	T	intergenic	*LINC00421,TPTE2*	.	.	.
13	33384447	C	T	ncRNA_intronic	*LINC00423*	.	.	.
13	22666722	C	A	intergenic	*LINC00424,LINC00540*	.	.	.

13	22748395	C	A	intergenic	*LINC00424,LINC00540*	.	.	.
13	22772195	C	A	intergenic	*LINC00424,LINC00540*	.	.	.
13	22688476	C	A	intergenic	*LINC00424,LINC00540*	.	.	.
13	22707068	CTCTCTA	C	intergenic	*LINC00424,LINC00540*	.	.	.
13	22767186	G	A	intergenic	*LINC00424,LINC00540*	.	.	.
13	87672287	A	T	intergenic	*LINC00430,MIR4500HG*	.	.	.
13	87728717	T	A	intergenic	*LINC00430,MIR4500HG*	.	.	.
13	87865382	C	A	intergenic	*LINC00430,MIR4500HG*	.	.	.
13	88006001	T	C	intergenic	*LINC00430,MIR4500HG*	.	.	.
13	87610906	G	C	intergenic	*LINC00430,MIR4500HG*	.	.	.
13	87935139	G	A	intergenic	*LINC00430,MIR4500HG*	.	.	.
13	88018755	G	C	intergenic	*LINC00430,MIR4500HG*	.	.	.
13	88018757	G	C	intergenic	*LINC00430,MIR4500HG*	.	.	.
13	88019128	A	G	intergenic	*LINC00430,MIR4500HG*	.	.	.
13	89215226	C	T	intergenic	*LINC00433,LINC01047*	.	.	.
13	89839060	C	T	intergenic	*LINC00433,LINC01047*	.	.	.
13	89249310	A	C	intergenic	*LINC00433,LINC01047*	.	.	.
13	89324534	G	A	intergenic	*LINC00433,LINC01047*	.	.	.
13	89466974	A	G	intergenic	*LINC00433,LINC01047*	.	.	.

13	89482900	G	A	intergenic	*LINC00433,LINC01047*	.	.	.
13	89484144	T	C	intergenic	*LINC00433,LINC01047*	.	.	.
13	89676050	C	T	intergenic	*LINC00433,LINC01047*	.	.	.
13	89729095	TA	T	intergenic	*LINC00433,LINC01047*	.	.	.
13	89746112	T	A	intergenic	*LINC00433,LINC01047*	.	.	.
13	60948941	C	A	intergenic	*LINC00434,TDRD3*	.	.	.
13	39188331	T	C	ncRNA_intronic	*LINC00437*	.	.	.
13	39166787	T	C	ncRNA_intronic	*LINC00437*	.	.	.
13	89934340	G	T	intergenic	*LINC00440,LINC01040*	.	.	.
13	90081552	T	C	intergenic	*LINC00440,LINC01040*	.	.	.
13	19641004	A	G	intergenic	*LINC00442,TUBA3C*	.	.	.
13	19657150	C	A	intergenic	*LINC00442,TUBA3C*	.	.	.
13	107592709	A	T	intergenic	*LINC00443,FAM155A*	.	.	.
13	107654593	T	C	intergenic	*LINC00443,FAM155A*	.	.	.
13	107576203	C	T	intergenic	*LINC00443,FAM155A*	.	.	.
13	107695811	G	C	intergenic	*LINC00443,FAM155A*	.	.	.
13	107673569	C	T	intergenic	*LINC00443,FAM155A*	.	.	.
13	107691790	A	T	intergenic	*LINC00443,FAM155A*	.	.	.
13	107691852	C	T	intergenic	*LINC00443,FAM155A*	.	.	.
13	107692313	T	C	intergenic	*LINC00443,FAM155A*	.	.	.

13	107692314	G	A	intergenic	*LINC00443,FAM155A*	.	.	.
13	107743030	C	T	intergenic	*LINC00443,FAM155A*	.	.	.
13	63336121	A	T	ncRNA_intronic	*LINC00448*	.	.	.
13	63354593	C	T	ncRNA_intronic	*LINC00448*	.	.	.
13	63556944	T	A	intergenic	*LINC00448,LINC00376*	.	.	.
13	63613877	G	A	intergenic	*LINC00448,LINC00376*	.	.	.
13	63658205	G	T	intergenic	*LINC00448,LINC00376*	.	.	.
13	63755775	A	G	intergenic	*LINC00448,LINC00376*	.	.	.
13	63756080	A	G	intergenic	*LINC00448,LINC00376*	.	.	.
13	35032422	A	T	ncRNA_intronic	*LINC00457*	.	.	.
13	35194981	G	A	ncRNA_intronic	*LINC00457*	.	.	.
13	35070779	CA	C	ncRNA_intronic	*LINC00457*	.	.	.
13	35126742	C	G	ncRNA_intronic	*LINC00457*	.	.	.
13	35356177	GTCTC	G	intergenic	*LINC00457,NBEA*	.	.	.
13	35222037	T	C	intergenic	*LINC00457,NBEA*	.	.	.
13	35226942	A	G	intergenic	*LINC00457,NBEA*	.	.	.
13	54745135	A	T	intergenic	*LINC00458,MIR1297*	.	.	.
13	54856742	A	C	intergenic	*LINC00458,MIR1297*	.	.	.
13	54857833	T	C	intergenic	*LINC00458,MIR1297*	.	.	.
5	87873155	C	A	ncRNA_intronic	*LINC00461*	.	.	.
5	87875577	T	C	ncRNA_intronic	*LINC00461*	.	.	.
5	87998773	C	A	intergenic	*LINC00461,MEF2C*	.	.	.
5	88001357	C	T	intergenic	*LINC00461,MEF2C*	.	.	.
1	63750977	A	G	ncRNA_intronic	*LINC00466*	.	.	.
1	211573165	T	C	ncRNA_intronic	*LINC00467*	.	.	.
1	211578748	T	C	ncRNA_intronic	*LINC00467*	.	.	.
1	211629132	C	A	intergenic	*LINC00467,RD3*	.	.	.
1	211632442	T	C	intergenic	*LINC00467,RD3*	.	.	.

17	71804110	A	G	ncRNA_intronic	*LINC00469*	.	.	.
17	71806093	G	A	ncRNA_intronic	*LINC00469*	.	.	.
17	72163789	C	CCACA	intergenic	*LINC00469,RPL38*	.	.	.
17	71921634	T	A	intergenic	*LINC00469,RPL38*	.	.	.
18	1337943	A	G	ncRNA_intronic	*LINC00470*	.	.	.
18	1740868	G	T	intergenic	*LINC00470,METTL4*	.	.	.
18	1905519	T	A	intergenic	*LINC00470,METTL4*	.	.	.
18	1617501	A	G	intergenic	*LINC00470,METTL4*	.	.	.
18	1717614	A	G	intergenic	*LINC00470,METTL4*	.	.	.
18	1770951	T	C	intergenic	*LINC00470,METTL4*	.	.	.
18	2331984	T	C	intergenic	*LINC00470,METTL4*	.	.	.
18	1695927	T	A	intergenic	*LINC00470,METTL4*	.	.	.
18	1388511	A	G	intergenic	*LINC00470,METTL4*	.	.	.
18	1631285	TATAC	T	intergenic	*LINC00470,METTL4*	.	.	.
18	1633149	A	C	intergenic	*LINC00470,METTL4*	.	.	.
18	1702267	A	G	intergenic	*LINC00470,METTL4*	.	.	.
18	1702490	A	C	intergenic	*LINC00470,METTL4*	.	.	.
18	1723067	G	C	intergenic	*LINC00470,METTL4*	.	.	.
18	1734112	C	T	intergenic	*LINC00470,METTL4*	.	.	.
18	2389698	C	T	intergenic	*LINC00470,METTL4*	.	.	.
18	2505443	ATTT	A	intergenic	*LINC00470,METTL4*	.	.	.

9	118779663	TTGTG	T	intergenic	*LINC00474,PAPPA*	.	.	.
9	118882477	G	A	intergenic	*LINC00474,PAPPA*	.	.	.
9	94920541	G	A	ncRNA_exonic	*LINC00475*	.	.	.
12	24954690	G	T	intergenic	*LINC00477,BCAT1*	.	.	.
12	24815375	A	T	intergenic	*LINC00477,BCAT1*	.	.	.
12	24773754	G	A	intergenic	*LINC00477,BCAT1*	.	.	.
12	24773939	G	T	intergenic	*LINC00477,BCAT1*	.	.	.
12	24838624	G	A	intergenic	*LINC00477,BCAT1*	.	.	.
12	24939783	AATAT	A	intergenic	*LINC00477,BCAT1*	.	.	.
3	108958194	T	TGGA	intergenic	*LINC00488,DPPA2*	.	.	.
3	108958978	T	C	intergenic	*LINC00488,DPPA2*	.	.	.
20	36280325	T	C	intergenic	*LINC00489,LOC100287792*	.	.	.
5	102103462	A	G	intergenic	*LINC00491,PAM*	.	.	.
5	102021774	C	T	intergenic	*LINC00491,PAM*	.	.	.
5	102021776	G	A	intergenic	*LINC00491,PAM*	.	.	.
5	102022187	A	G	intergenic	*LINC00491,PAM*	.	.	.
5	102089151	GACAC	G	intergenic	*LINC00491,PAM*	.	.	.
4	139273036	G	T	ncRNA_intronic	*LINC00499*	.	.	.
4	139429171	G	T	intergenic	*LINC00499,NOCT*	.	.	.
4	139710721	T	C	intergenic	*LINC00499,NOCT*	.	.	.
4	139366891	C	T	intergenic	*LINC00499,NOCT*	.	.	.
4	139423726	T	A	intergenic	*LINC00499,NOCT*	.	.	.
3	177147747	G	A	intergenic	*LINC00501,LINC00578*	.	.	.
10	92867132	C	A	intergenic	*LINC00502,NUDT9P1*	.	.	.
4	14473600	C	G	ncRNA_exonic	*LINC00504*	.	.	.
4	14527768	AT	A	ncRNA_intronic	*LINC00504*	.	.	.
4	14527770	A	G	ncRNA_intronic	*LINC00504*	.	.	.
4	14544406	G	A	ncRNA_intronic	*LINC00504*	.	.	.
4	14724189	T	C	ncRNA_intronic	*LINC00504*	.	.	.
4	14841624	A	C	ncRNA_intronic	*LINC00504*	.	.	.
3	87142176	C	T	ncRNA_intronic	*LINC00506*	.	.	.
3	87247453	C	A	intergenic	*LINC00506,MIR4795*	.	.	.

14	56250283	A	G	ncRNA_intronic	*LINC00520*	.	.	.
14	56460289	A	G	intergenic	*LINC00520,PELI2*	.	.	.
14	56276121	A	T	intergenic	*LINC00520,PELI2*	.	.	.
14	56475701	A	G	intergenic	*LINC00520,PELI2*	.	.	.
7	47808495	A	G	intergenic	*LINC00525,PKD1L1*	.	.	.
8	91339668	G	T	ncRNA_intronic	*LINC00534*	.	.	.
8	91281764	G	A	ncRNA_intronic	*LINC00534*	.	.	.
8	91347987	C	G	ncRNA_intronic	*LINC00534*	.	.	.
8	91348303	A	T	ncRNA_intronic	*LINC00534*	.	.	.
8	91390573	C	A	ncRNA_intronic	*LINC00534*	.	.	.
8	91390784	A	G	ncRNA_intronic	*LINC00534*	.	.	.
8	91392645	C	T	ncRNA_intronic	*LINC00534*	.	.	.
8	91404677	T	C	intergenic	*LINC00534,LINC01030*	.	.	.
8	91500153	T	A	intergenic	*LINC00534,LINC01030*	.	.	.
8	91500458	A	G	intergenic	*LINC00534,LINC01030*	.	.	.
8	91501552	A	G	intergenic	*LINC00534,LINC01030*	.	.	.
8	91512862	ATGTG	A	intergenic	*LINC00534,LINC01030*	.	.	.
8	94393804	C	T	ncRNA_intronic	*LINC00535*	.	.	.
8	94613670	G	T	ncRNA_intronic	*LINC00535*	.	.	.
8	94481099	A	G	ncRNA_intronic	*LINC00535*	.	.	.
8	94481356	A	C	ncRNA_intronic	*LINC00535*	.	.	.
8	94483659	G	C	ncRNA_intronic	*LINC00535*	.	.	.
8	94483788	T	A	ncRNA_intronic	*LINC00535*	.	.	.
8	117195958	C	T	ncRNA_intronic	*LINC00536*	.	.	.
8	117207093	T	C	ncRNA_intronic	*LINC00536*	.	.	.
8	117078548	A	G	ncRNA_intronic	*LINC00536*	.	.	.
8	117329892	G	A	ncRNA_intronic	*LINC00536*	.	.	.
8	117343696	C	T	intergenic	*LINC00536,EIF3H*	.	.	.
8	117408103	T	C	intergenic	*LINC00536,EIF3H*	.	.	.
8	117428325	A	T	intergenic	*LINC00536,EIF3H*	.	.	.
13	23338333	C	T	intergenic	*LINC00540,BASP1P1*	.	.	.

13	22937477	A	G	intergenic	*LINC00540,BASP1P1*	.	.	.
13	22937478	T	C	intergenic	*LINC00540,BASP1P1*	.	.	.
13	23050271	C	G	intergenic	*LINC00540,BASP1P1*	.	.	.
13	23050272	C	G	intergenic	*LINC00540,BASP1P1*	.	.	.
13	23050994	T	G	intergenic	*LINC00540,BASP1P1*	.	.	.
13	23102157	G	A	intergenic	*LINC00540,BASP1P1*	.	.	.
13	23351870	T	C	intergenic	*LINC00540,BASP1P1*	.	.	.
13	23351871	G	A	intergenic	*LINC00540,BASP1P1*	.	.	.
13	30534276	A	G	intergenic	*LINC00544,LINC00365*	.	.	.
13	30530410	C	A	intergenic	*LINC00544,LINC00365*	.	.	.
13	30543417	G	T	intergenic	*LINC00544,LINC00365*	.	.	.
13	38125956	G	A	downstream	*LINC00547*	.	.	.
13	40784256	C	T	ncRNA_intronic	*LINC00548*	.	.	.
13	69754015	A	G	intergenic	*LINC00550,LINC00383*	.	.	.
13	69469043	T	G	intergenic	*LINC00550,LINC00383*	.	.	.
13	69757312	A	C	intergenic	*LINC00550,LINC00383*	.	.	.
13	69520389	G	A	intergenic	*LINC00550,LINC00383*	.	.	.
13	69605157	T	C	intergenic	*LINC00550,LINC00383*	.	.	.
13	69626467	A	C	intergenic	*LINC00550,LINC00383*	.	.	.
13	69626633	A	G	intergenic	*LINC00550,LINC00383*	.	.	.

13	69627171	G	C	intergenic	*LINC00550,LINC00383*	.	.	.
13	69779619	G	A	intergenic	*LINC00550,LINC00383*	.	.	.
13	114455880	A	C	intergenic	*LINC00552,TMEM255B*	.	.	.
13	114455881	A	T	intergenic	*LINC00552,TMEM255B*	.	.	.
13	100684630	C	T	intergenic	*LINC00554,LOC105370333*	.	.	.
13	100713762	C	T	intergenic	*LINC00554,LOC105370333*	.	.	.
13	54480024	A	G	intergenic	*LINC00558,LINC00458*	.	.	.
13	54578815	C	T	intergenic	*LINC00558,LINC00458*	.	.	.
13	54637960	T	TCA	intergenic	*LINC00558,LINC00458*	.	.	.
13	54472594	C	T	intergenic	*LINC00558,LINC00458*	.	.	.
13	54582782	A	G	intergenic	*LINC00558,LINC00458*	.	.	.
13	90734206	C	A	ncRNA_intronic	*LINC00559*	.	.	.
13	46872223	T	C	upstream	*LINC00563*	.	.	.
13	81815403	C	CAGAG	intergenic	*LINC00564,NONE*	.	.	.
13	82022160	A	T	intergenic	*LINC00564,NONE*	.	.	.
13	82111438	G	T	intergenic	*LINC00564,NONE*	.	.	.
13	82206254	C	T	intergenic	*LINC00564,NONE*	.	.	.
13	82304618	GA	G	intergenic	*LINC00564,NONE*	.	.	.
13	82304620	T	G	intergenic	*LINC00564,NONE*	.	.	.
13	82305148	C	T	intergenic	*LINC00564,NONE*	.	.	.
13	82324590	A	G	intergenic	*LINC00564,NONE*	.	.	.
13	82671905	ATCTC	A	intergenic	*LINC00564,SLITRK1*	.	.	.
13	82426570	G	T	intergenic	*LINC00564,SLITRK1*	.	.	.
13	82455292	T	G	intergenic	*LINC00564,SLITRK1*	.	.	.

13	82424814	C	T	intergenic	*LINC00564,SLITRK1*	.	.	.
13	82593511	G	GA	intergenic	*LINC00564,SLITRK1*	.	.	.
13	82690850	A	G	intergenic	*LINC00564,SLITRK1*	.	.	.
13	82503382	T	C	intergenic	*LINC00564,SLITRK1*	.	.	.
13	82761905	C	T	intergenic	*LINC00564,SLITRK1*	.	.	.
13	82686345	A	G	intergenic	*LINC00564,SLITRK1*	.	.	.
13	82747935	A	ATAC	intergenic	*LINC00564,SLITRK1*	.	.	.
13	82806476	T	C	intergenic	*LINC00564,SLITRK1*	.	.	.
13	82977674	C	CAA	intergenic	*LINC00564,SLITRK1*	.	.	.
13	82979212	A	G	intergenic	*LINC00564,SLITRK1*	.	.	.
13	82994928	G	A	intergenic	*LINC00564,SLITRK1*	.	.	.
13	83359026	A	T	intergenic	*LINC00564,SLITRK1*	.	.	.
13	83382595	T	G	intergenic	*LINC00564,SLITRK1*	.	.	.
13	83431369	C	G	intergenic	*LINC00564,SLITRK1*	.	.	.
13	83815264	T	C	intergenic	*LINC00564,SLITRK1*	.	.	.
13	83815265	G	T	intergenic	*LINC00564,SLITRK1*	.	.	.
13	83816509	G	A	intergenic	*LINC00564,SLITRK1*	.	.	.
13	83879848	T	A	intergenic	*LINC00564,SLITRK1*	.	.	.
13	24919023	G	A	intergenic	*LINC00566,PARP4*	.	.	.
13	24919536	C	A	intergenic	*LINC00566,PARP4*	.	.	.

13	24919538	A	G	intergenic	*LINC00566,PARP4*	.	.	.
13	24920387	C	G	intergenic	*LINC00566,PARP4*	.	.	.
13	38691612	T	C	ncRNA_intronic	*LINC00571*	.	.	.
13	38641421	T	C	ncRNA_intronic	*LINC00571*	.	.	.
13	38641422	G	A	ncRNA_intronic	*LINC00571*	.	.	.
13	38689247	G	A	ncRNA_intronic	*LINC00571*	.	.	.
13	38719581	T	C	intergenic	*LINC00571,UFM1*	.	.	.
13	38810586	G	C	intergenic	*LINC00571,UFM1*	.	.	.
13	38843271	C	A	intergenic	*LINC00571,UFM1*	.	.	.
6	170383587	C	T	intergenic	*LINC00574,LOC102724511*	.	.	.
6	170386659	G	A	intergenic	*LINC00574,LOC102724511*	.	.	.
3	177503616	C	T	intergenic	*LINC00578,KCCAT211*	.	.	.
3	177501946	T	G	intergenic	*LINC00578,KCCAT211*	.	.	.
6	21520329	A	G	intergenic	*LINC00581,SOX4*	.	.	.
6	21543548	C	T	intergenic	*LINC00581,SOX4*	.	.	.
9	13948387	T	A	intergenic	*LINC00583,NFIB*	.	.	.
9	13998336	G	A	intergenic	*LINC00583,NFIB*	.	.	.
9	105386081	TC	T	ncRNA_intronic	*LINC00587*	.	.	.
9	105396659	ACTCT	A	ncRNA_intronic	*LINC00587*	.	.	.
9	105394076	C	T	ncRNA_intronic	*LINC00587*	.	.	.
9	105665098	C	A	intergenic	*LINC00587,CYLC2*	.	.	.
9	105579325	G	C	intergenic	*LINC00587,CYLC2*	.	.	.
9	105615858	A	G	intergenic	*LINC00587,CYLC2*	.	.	.
9	105667079	A	G	intergenic	*LINC00587,CYLC2*	.	.	.
8	58237243	T	C	intergenic	*LINC00588,LOC101929488*	.	.	.
15	70133153	C	A	ncRNA_exonic	*LINC00593*	.	.	.
10	80684042	A	C	intergenic	*LINC00595,ZMIZ1-AS1*	.	.	.
10	80371559	T	C	intergenic	*LINC00595,ZMIZ1-AS1*	.	.	.
10	80367052	C	T	intergenic	*LINC00595,ZMIZ1-AS1*	.	.	.
13	40990034	G	A	ncRNA_intronic	*LINC00598*	.	.	.

13	40993180	T	C	ncRNA_intronic	*LINC00598*	.	.	.
5	40176654	C	T	intergenic	*LINC00603,PTGER4*	.	.	.
5	40275404	C	T	intergenic	*LINC00603,PTGER4*	.	.	.
5	40328025	G	T	intergenic	*LINC00603,PTGER4*	.	.	.
5	40472167	G	C	intergenic	*LINC00603,PTGER4*	.	.	.
5	40061658	A	G	intergenic	*LINC00603,PTGER4*	.	.	.
5	40180417	T	C	intergenic	*LINC00603,PTGER4*	.	.	.
5	40298513	T	G	intergenic	*LINC00603,PTGER4*	.	.	.
5	40338398	T	C	intergenic	*LINC00603,PTGER4*	.	.	.
5	40338400	G	T	intergenic	*LINC00603,PTGER4*	.	.	.
5	40340926	A	G	intergenic	*LINC00603,PTGER4*	.	.	.
5	40418669	C	T	intergenic	*LINC00603,PTGER4*	.	.	.
5	40421561	T	C	intergenic	*LINC00603,PTGER4*	.	.	.
5	40448407	A	G	intergenic	*LINC00603,PTGER4*	.	.	.
5	40448409	A	G	intergenic	*LINC00603,PTGER4*	.	.	.
5	40449714	A	T	intergenic	*LINC00603,PTGER4*	.	.	.
5	40469605	C	A	intergenic	*LINC00603,PTGER4*	.	.	.
5	40470140	T	G	intergenic	*LINC00603,PTGER4*	.	.	.
5	40472891	C	T	intergenic	*LINC00603,PTGER4*	.	.	.

5	40565451	A	ACT	intergenic	*LINC00603,PTGER4*	.	.	.
5	40591330	A	T	intergenic	*LINC00603,PTGER4*	.	.	.
5	40636316	C	G	intergenic	*LINC00603,PTGER4*	.	.	.
5	40639881	G	A	intergenic	*LINC00603,PTGER4*	.	.	.
5	40639883	G	T	intergenic	*LINC00603,PTGER4*	.	.	.
2	216686914	A	T	ncRNA_intronic	*LINC00607*	.	.	.
2	216688383	C	A	ncRNA_intronic	*LINC00607*	.	.	.
14	36563786	T	C	ncRNA_intronic	*LINC00609*	.	.	.
4	136833098	TTAATCCTCTCAAGACCTGTATAAGGTCTTGAGAGGATTAATGGAA	T	ncRNA_intronic	*LINC00613*	.	.	.
4	137816634	C	CTATA	intergenic	*LINC00613,PCDH18*	.	.	.
4	137790575	G	C	intergenic	*LINC00613,PCDH18*	.	.	.
4	137134085	G	C	intergenic	*LINC00613,PCDH18*	.	.	.
4	137064603	A	G	intergenic	*LINC00613,PCDH18*	.	.	.
4	138226700	T	A	intergenic	*LINC00613,PCDH18*	.	.	.
4	136991826	G	A	intergenic	*LINC00613,PCDH18*	.	.	.
4	137282002	T	C	intergenic	*LINC00613,PCDH18*	.	.	.
4	137678709	G	A	intergenic	*LINC00613,PCDH18*	.	.	.
4	138274693	A	AAC	intergenic	*LINC00613,PCDH18*	.	.	.

4	138274694	T	TCTC	intergenic	*LINC00613,PCDH18*	.	.	.
14	97790024	A	G	intergenic	*LINC00618,LOC101929241*	.	.	.
14	97852151	T	G	intergenic	*LINC00618,LOC101929241*	.	.	.
14	97893415	A	T	intergenic	*LINC00618,LOC101929241*	.	.	.
14	97622158	C	T	intergenic	*LINC00618,LOC101929241*	.	.	.
14	97679230	C	A	intergenic	*LINC00618,LOC101929241*	.	.	.
3	13776516	T	C	ncRNA_intronic	*LINC00620*	.	.	.
1	149584899	C	T	ncRNA_intronic	*LINC00623,LINC00869,LOC103091866*	.	.	.
1	146914927	C	T	ncRNA_intronic	*LINC00624*	.	.	.
1	146914929	C	T	ncRNA_intronic	*LINC00624*	.	.	.
1	146921235	G	T	ncRNA_intronic	*LINC00624*	.	.	.
1	147005027	GTCTCTCTCTC	G	intergenic	*LINC00624,BCL9*	.	.	.
1	168783824	T	C	intergenic	*LINC00626,LINC00970*	.	.	.
1	168783825	A	C	intergenic	*LINC00626,LINC00970*	.	.	.
1	168853787	A	G	intergenic	*LINC00626,LINC00970*	.	.	.
X	102049016	A	G	ncRNA_intronic	*LINC00630*	.	.	.
X	102057218	T	G	ncRNA_intronic	*LINC00630*	.	.	.
X	102066946	A	G	ncRNA_intronic	*LINC00630*	.	.	.
X	102123175	C	T	ncRNA_intronic	*LINC00630*	.	.	.
X	102127519	G	A	ncRNA_intronic	*LINC00630*	.	.	.
X	102128764	G	A	ncRNA_intronic	*LINC00630*	.	.	.
X	102128765	G	A	ncRNA_intronic	*LINC00630*	.	.	.
X	102129608	A	G	ncRNA_intronic	*LINC00630*	.	.	.
X	102129626	C	T	ncRNA_intronic	*LINC00630*	.	.	.
X	102129627	A	G	ncRNA_intronic	*LINC00630*	.	.	.

X	102170663	T	A	intergenic	*LINC00630,RAB40AL*	.	.	.
X	139808616	C	T	ncRNA_intronic	*LINC00632*	.	.	.
14	104333842	G	A	intergenic	*LINC00637,C14orf2*	.	.	.
14	39236311	C	T	ncRNA_intronic	*LINC00639*	.	.	.
14	39252746	C	T	ncRNA_intronic	*LINC00639*	.	.	.
14	39437915	TG	T	intergenic	*LINC00639,SEC23A*	.	.	.
14	39393339	G	C	intergenic	*LINC00639,SEC23A*	.	.	.
14	39437405	G	T	intergenic	*LINC00639,SEC23A*	.	.	.
14	39437407	A	G	intergenic	*LINC00639,SEC23A*	.	.	.
14	39447436	T	G	intergenic	*LINC00639,SEC23A*	.	.	.
14	39489332	A	C	intergenic	*LINC00639,SEC23A*	.	.	.
14	39490658	G	A	intergenic	*LINC00639,SEC23A*	.	.	.
14	39491262	C	A	intergenic	*LINC00639,SEC23A*	.	.	.
14	51908542	G	T	intergenic	*LINC00640,FRMD6-AS2*	.	.	.
14	51872837	A	G	intergenic	*LINC00640,FRMD6-AS2*	.	.	.
14	51883190	C	T	intergenic	*LINC00640,FRMD6-AS2*	.	.	.
14	51883936	C	T	intergenic	*LINC00640,FRMD6-AS2*	.	.	.
14	51883937	T	C	intergenic	*LINC00640,FRMD6-AS2*	.	.	.
14	90936083	C	T	intergenic	*LINC00642,TTC7B*	.	.	.
14	62868170	T	A	intergenic	*LINC00644,KCNH5*	.	.	.
14	62635683	A	G	intergenic	*LINC00644,KCNH5*	.	.	.
14	62668011	T	C	intergenic	*LINC00644,KCNH5*	.	.	.
14	62668012	A	C	intergenic	*LINC00644,KCNH5*	.	.	.

14	62669504	T	C	intergenic	*LINC00644,KCNH5*	.	.	.
14	62772698	C	T	intergenic	*LINC00644,KCNH5*	.	.	.
14	62773076	C	T	intergenic	*LINC00644,KCNH5*	.	.	.
14	62794804	T	A	intergenic	*LINC00644,KCNH5*	.	.	.
14	62863324	C	A	intergenic	*LINC00644,KCNH5*	.	.	.
14	62989530	T	A	intergenic	*LINC00644,KCNH5*	.	.	.
14	63026559	G	T	intergenic	*LINC00644,KCNH5*	.	.	.
14	63105662	A	G	intergenic	*LINC00644,KCNH5*	.	.	.
14	28104356	CGTGT	C	ncRNA_intronic	*LINC00645*	.	.	.
14	28557130	G	A	intergenic	*LINC00645,FOXG1-AS1*	.	.	.
14	29116965	G	A	intergenic	*LINC00645,FOXG1-AS1*	.	.	.
14	28295195	C	T	intergenic	*LINC00645,FOXG1-AS1*	.	.	.
14	28389262	C	T	intergenic	*LINC00645,FOXG1-AS1*	.	.	.
14	28460152	A	T	intergenic	*LINC00645,FOXG1-AS1*	.	.	.
14	28679586	T	G	intergenic	*LINC00645,FOXG1-AS1*	.	.	.
14	29034891	T	A	intergenic	*LINC00645,FOXG1-AS1*	.	.	.
14	28890967	A	T	intergenic	*LINC00645,FOXG1-AS1*	.	.	.
14	28309110	T	C	intergenic	*LINC00645,FOXG1-AS1*	.	.	.
14	28712439	T	C	intergenic	*LINC00645,FOXG1-AS1*	.	.	.
14	28308727	C	T	intergenic	*LINC00645,FOXG1-AS1*	.	.	.
14	28392314	TACAC	T	intergenic	*LINC00645,FOXG1-AS1*	.	.	.
14	28118504	C	G	intergenic	*LINC00645,FOXG1-AS1*	.	.	.
14	28118505	A	G	intergenic	*LINC00645,FOXG1-AS1*	.	.	.

14	28147357	A	G	intergenic	*LINC00645,FOXG1-AS1*	.	.	.
14	28239684	A	AC	intergenic	*LINC00645,FOXG1-AS1*	.	.	.
14	28390112	C	A	intergenic	*LINC00645,FOXG1-AS1*	.	.	.
14	28412052	T	C	intergenic	*LINC00645,FOXG1-AS1*	.	.	.
14	28412053	T	C	intergenic	*LINC00645,FOXG1-AS1*	.	.	.
14	28435925	A	C	intergenic	*LINC00645,FOXG1-AS1*	.	.	.
14	28438960	T	C	intergenic	*LINC00645,FOXG1-AS1*	.	.	.
14	28438962	T	C	intergenic	*LINC00645,FOXG1-AS1*	.	.	.
14	28510091	C	T	intergenic	*LINC00645,FOXG1-AS1*	.	.	.
14	28544795	C	A	intergenic	*LINC00645,FOXG1-AS1*	.	.	.
14	28639340	T	C	intergenic	*LINC00645,FOXG1-AS1*	.	.	.
14	28639341	C	T	intergenic	*LINC00645,FOXG1-AS1*	.	.	.
14	28642098	T	G	intergenic	*LINC00645,FOXG1-AS1*	.	.	.
14	28661824	G	C	intergenic	*LINC00645,FOXG1-AS1*	.	.	.
14	28782814	A	T	intergenic	*LINC00645,FOXG1-AS1*	.	.	.
14	28975189	C	T	intergenic	*LINC00645,FOXG1-AS1*	.	.	.
14	28975190	A	G	intergenic	*LINC00645,FOXG1-AS1*	.	.	.
14	29015492	T	C	intergenic	*LINC00645,FOXG1-AS1*	.	.	.
14	29105613	C	T	intergenic	*LINC00645,FOXG1-AS1*	.	.	.

14	29107716	C	A	intergenic	*LINC00645,FOXG1-AS1*	.	.	.
14	48850761	G	T	intergenic	*LINC00648,RPS29*	.	.	.
14	48522841	G	A	intergenic	*LINC00648,RPS29*	.	.	.
14	49494459	T	TACTGAAAT(	intergenic	*LINC00648,RPS29*	.	.	.
14	48876324	C	T	intergenic	*LINC00648,RPS29*	.	.	.
14	48480245	A	G	intergenic	*LINC00648,RPS29*	.	.	.
14	49000307	G	T	intergenic	*LINC00648,RPS29*	.	.	.
14	48338567	T	A	intergenic	*LINC00648,RPS29*	.	.	.
14	48409656	C	A	intergenic	*LINC00648,RPS29*	.	.	.
14	48411119	G	A	intergenic	*LINC00648,RPS29*	.	.	.
14	48411334	C	T	intergenic	*LINC00648,RPS29*	.	.	.
14	48439617	A	G	intergenic	*LINC00648,RPS29*	.	.	.
14	48568916	C	T	intergenic	*LINC00648,RPS29*	.	.	.
14	48601022	G	T	intergenic	*LINC00648,RPS29*	.	.	.
14	48750951	C	A	intergenic	*LINC00648,RPS29*	.	.	.
14	48835864	G	A	intergenic	*LINC00648,RPS29*	.	.	.
14	48836821	G	A	intergenic	*LINC00648,RPS29*	.	.	.
14	48838409	G	C	intergenic	*LINC00648,RPS29*	.	.	.
14	48838410	A	T	intergenic	*LINC00648,RPS29*	.	.	.
14	48857186	C	T	intergenic	*LINC00648,RPS29*	.	.	.
14	48881388	C	G	intergenic	*LINC00648,RPS29*	.	.	.
14	48882583	T	G	intergenic	*LINC00648,RPS29*	.	.	.
14	48884801	C	A	intergenic	*LINC00648,RPS29*	.	.	.
14	48913114	G	A	intergenic	*LINC00648,RPS29*	.	.	.
14	49039845	C	G	intergenic	*LINC00648,RPS29*	.	.	.
14	49039846	A	G	intergenic	*LINC00648,RPS29*	.	.	.
14	49041155	A	G	intergenic	*LINC00648,RPS29*	.	.	.
14	49041701	G	T	intergenic	*LINC00648,RPS29*	.	.	.
14	49061667	T	C	intergenic	*LINC00648,RPS29*	.	.	.
14	49061843	C	T	intergenic	*LINC00648,RPS29*	.	.	.
14	49066276	C	T	intergenic	*LINC00648,RPS29*	.	.	.
14	49080857	T	C	intergenic	*LINC00648,RPS29*	.	.	.
14	49170298	A	G	intergenic	*LINC00648,RPS29*	.	.	.
14	49291928	A	G	intergenic	*LINC00648,RPS29*	.	.	.
14	49590534	C	T	intergenic	*LINC00648,RPS29*	.	.	.
14	49590535	A	G	intergenic	*LINC00648,RPS29*	.	.	.
14	49612979	A	G	intergenic	*LINC00648,RPS29*	.	.	.

14	49614683	A	AG	intergenic	*LINC00648,RPS29*	.	.	.
14	49648765	G	A	intergenic	*LINC00648,RPS29*	.	.	.
14	49649462	T	A	intergenic	*LINC00648,RPS29*	.	.	.
14	49650135	T	C	intergenic	*LINC00648,RPS29*	.	.	.
14	49714159	A	T	intergenic	*LINC00648,RPS29*	.	.	.
14	49714161	T	C	intergenic	*LINC00648,RPS29*	.	.	.
14	49714619	T	C	intergenic	*LINC00648,RPS29*	.	.	.
14	49816588	A	G	intergenic	*LINC00648,RPS29*	.	.	.
14	49852604	G	C	intergenic	*LINC00648,RPS29*	.	.	.
14	49852834	C	T	intergenic	*LINC00648,RPS29*	.	.	.
14	49860036	A	G	intergenic	*LINC00648,RPS29*	.	.	.
14	49861205	A	G	intergenic	*LINC00648,RPS29*	.	.	.
14	50028101	C	T	intergenic	*LINC00648,RPS29*	.	.	.
14	50028102	C	G	intergenic	*LINC00648,RPS29*	.	.	.
21	35401870	T	C	intergenic	*LINC00649,MRPS6*	.	.	.
21	35347846	C	T	intergenic	*LINC00649,MRPS6*	.	.	.
20	23241306	C	T	intergenic	*LINC00656,NXT1*	.	.	.
20	23299323	A	G	intergenic	*LINC00656,NXT1*	.	.	.
20	23162241	T	TC	intergenic	*LINC00656,NXT1*	.	.	.
20	23162243	C	T	intergenic	*LINC00656,NXT1*	.	.	.
20	23162330	G	A	intergenic	*LINC00656,NXT1*	.	.	.
20	23162331	C	G	intergenic	*LINC00656,NXT1*	.	.	.
20	23219549	G	T	intergenic	*LINC00656,NXT1*	.	.	.
20	23220263	A	G	intergenic	*LINC00656,NXT1*	.	.	.
20	5421284	T	A	ncRNA_intronic	*LINC00658*	.	.	.
20	5430614	G	A	intergenic	*LINC00658,LOC643406*	.	.	.
20	5438438	T	A	intergenic	*LINC00658,LOC643406*	.	.	.
20	5438639	T	A	intergenic	*LINC00658,LOC643406*	.	.	.
19	21673313	T	A	ncRNA_exonic	*LINC00664*	.	.	.
17	12462905	A	T	ncRNA_intronic	*LINC00670*	.	.	.
17	12558192	G	T	intergenic	*LINC00670,MYOCD*	.	.	.
17	41027169	C	A	ncRNA_intronic	*LINC00671*	.	.	.

17	70545434	TTCTC	T	ncRNA_intronic	*LINC00673*	.	.	.
17	70553392	A	G	ncRNA_intronic	*LINC00673*	.	.	.
17	70540903	C	T	ncRNA_intronic	*LINC00673*	.	.	.
17	70548766	C	T	ncRNA_intronic	*LINC00673*	.	.	.
6	58562126	C	G	intergenic	*LINC00680-GUSBP4,NONE*	.	.	.
6	58528185	C	T	intergenic	*LINC00680-GUSBP4,NONE*	.	.	.
6	58391333	G	A	intergenic	*LINC00680-GUSBP4,NONE*	.	.	.
6	58532911	G	A	intergenic	*LINC00680-GUSBP4,NONE*	.	.	.
6	58674622	ATTTTTTTTTT	A	intergenic	*LINC00680-GUSBP4,NONE*	.	.	.
6	58584568	T	C	intergenic	*LINC00680-GUSBP4,NONE*	.	.	.
6	58429512	A	C	intergenic	*LINC00680-GUSBP4,NONE*	.	.	.
6	58456727	A	G	intergenic	*LINC00680-GUSBP4,NONE*	.	.	.
6	58474089	C	T	intergenic	*LINC00680-GUSBP4,NONE*	.	.	.
6	58478496	A	G	intergenic	*LINC00680-GUSBP4,NONE*	.	.	.
6	58520884	C	T	intergenic	*LINC00680-GUSBP4,NONE*	.	.	.
6	58538465	C	G	intergenic	*LINC00680-GUSBP4,NONE*	.	.	.
6	58551703	T	C	intergenic	*LINC00680-GUSBP4,NONE*	.	.	.
6	58602702	G	A	intergenic	*LINC00680-GUSBP4,NONE*	.	.	.
6	58722181	A	T	intergenic	*LINC00680-GUSBP4,NONE*	.	.	.
6	58723520	C	T	intergenic	*LINC00680-GUSBP4,NONE*	.	.	.
6	58723706	T	C	intergenic	*LINC00680-GUSBP4,NONE*	.	.	.

6	58724261	A	ATCGGAAGA	intergenic	*LINC00680-GUSBP4,NONE*	.	.	.
8	12686776	C	A	intergenic	*LINC00681,KIAA1456*	.	.	.
4	41905732	C	CTG	intergenic	*LINC00682,TMEM33*	.	.	.
4	41906921	A	G	intergenic	*LINC00682,TMEM33*	.	.	.
4	41909329	A	C	intergenic	*LINC00682,TMEM33*	.	.	.
20	11865611	A	G	intergenic	*LINC00687,BTBD3*	.	.	.
3	16600930	A	C	intergenic	*LINC00690,DAZL*	.	.	.
3	26335740	T	A	intergenic	*LINC00692,LRRC3B*	.	.	.
3	26435085	A	G	intergenic	*LINC00692,LRRC3B*	.	.	.
3	25972886	C	A	intergenic	*LINC00692,LRRC3B*	.	.	.
3	26254068	A	G	intergenic	*LINC00692,LRRC3B*	.	.	.
3	26035958	A	G	intergenic	*LINC00692,LRRC3B*	.	.	.
3	26035959	T	G	intergenic	*LINC00692,LRRC3B*	.	.	.
3	26207105	C	T	intergenic	*LINC00692,LRRC3B*	.	.	.
3	26086848	G	A	intergenic	*LINC00692,LRRC3B*	.	.	.
3	26538415	A	G	intergenic	*LINC00692,LRRC3B*	.	.	.
3	26037484	C	T	intergenic	*LINC00692,LRRC3B*	.	.	.
3	26081330	A	G	intergenic	*LINC00692,LRRC3B*	.	.	.
3	26168785	A	G	intergenic	*LINC00692,LRRC3B*	.	.	.
3	26168955	G	A	intergenic	*LINC00692,LRRC3B*	.	.	.

3	26168957	A	C	intergenic	*LINC00692,LRRC3B*	.	.	.
3	26169506	T	C	intergenic	*LINC00692,LRRC3B*	.	.	.
3	26282473	G	A	intergenic	*LINC00692,LRRC3B*	.	.	.
3	26283385	A	G	intergenic	*LINC00692,LRRC3B*	.	.	.
3	26477884	A	C	intergenic	*LINC00692,LRRC3B*	.	.	.
3	26478564	T	C	intergenic	*LINC00692,LRRC3B*	.	.	.
3	26509804	G	A	intergenic	*LINC00692,LRRC3B*	.	.	.
3	26536524	C	T	intergenic	*LINC00692,LRRC3B*	.	.	.
3	29142627	A	G	intergenic	*LINC00693,RBMS3-AS3*	.	.	.
3	29039352	A	T	intergenic	*LINC00693,RBMS3-AS3*	.	.	.
3	29094558	A	G	intergenic	*LINC00693,RBMS3-AS3*	.	.	.
3	29096612	G	A	intergenic	*LINC00693,RBMS3-AS3*	.	.	.
3	29172329	C	G	intergenic	*LINC00693,RBMS3-AS3*	.	.	.
10	2357413	G	A	upstream	*LINC00701*	.	.	.
10	2801204	TTATA	T	intergenic	*LINC00701,PFKP*	.	.	.
10	2702487	A	G	intergenic	*LINC00701,PFKP*	.	.	.
10	2930696	G	A	intergenic	*LINC00701,PFKP*	.	.	.
10	2621921	A	G	intergenic	*LINC00701,PFKP*	.	.	.
10	2843181	G	A	intergenic	*LINC00701,PFKP*	.	.	.
10	2843182	G	A	intergenic	*LINC00701,PFKP*	.	.	.
10	2994442	A	G	intergenic	*LINC00701,PFKP*	.	.	.
10	3010961	TGAGAGA	T	intergenic	*LINC00701,PFKP*	.	.	.
10	6864792	C	T	ncRNA_intronic	*LINC00707*	.	.	.
10	6871994	C	T	ncRNA_intronic	*LINC00707*	.	.	.
10	6849194	G	T	ncRNA_intronic	*LINC00707*	.	.	.

10	7189605	C	T	intergenic	*LINC00707,SFMBT2*	.	.	.
10	7189606	A	G	intergenic	*LINC00707,SFMBT2*	.	.	.
10	7091590	G	A	intergenic	*LINC00707,SFMBT2*	.	.	.
10	7061244	T	C	intergenic	*LINC00707,SFMBT2*	.	.	.
10	7088307	T	C	intergenic	*LINC00707,SFMBT2*	.	.	.
10	7046531	C	G	intergenic	*LINC00707,SFMBT2*	.	.	.
10	7066775	AAATAAT	A	intergenic	*LINC00707,SFMBT2*	.	.	.
10	8531575	A	G	intergenic	*LINC00708,LOC105755953*	.	.	.
10	8916959	T	A	intergenic	*LINC00708,LOC105755953*	.	.	.
10	8360355	G	C	intergenic	*LINC00708,LOC105755953*	.	.	.
8	129437376	GTAAGCCCAGGGTGGTC	G	ncRNA_intronic	*LINC00824*	.	.	.
8	129588794	T	A	intergenic	*LINC00824,LINC00977*	.	.	.
8	130009292	A	G	intergenic	*LINC00824,LINC00977*	.	.	.
8	129583383	C	A	intergenic	*LINC00824,LINC00977*	.	.	.
8	129884571	A	G	intergenic	*LINC00824,LINC00977*	.	.	.
8	130084081	G	T	intergenic	*LINC00824,LINC00977*	.	.	.
8	129897422	C	T	intergenic	*LINC00824,LINC00977*	.	.	.
8	130086132	G	A	intergenic	*LINC00824,LINC00977*	.	.	.

8	129631222	C	G	intergenic	*LINC00824,LINC00977*	.	.	.
8	129885660	G	A	intergenic	*LINC00824,LINC00977*	.	.	.
8	129720857	G	A	intergenic	*LINC00824,LINC00977*	.	.	.
8	129816563	A	G	intergenic	*LINC00824,LINC00977*	.	.	.
10	34294335	T	A	intergenic	*LINC00838,PARD3*	.	.	.
10	34138435	A	T	intergenic	*LINC00838,PARD3*	.	.	.
10	34294241	A	C	intergenic	*LINC00838,PARD3*	.	.	.
10	44370447	G	T	ncRNA_intronic	*LINC00840*	.	.	.
10	44456102	A	G	ncRNA_intronic	*LINC00841*	.	.	.
10	44465865	C	A	downstream	*LINC00841*	.	.	.
10	44411051	A	G	ncRNA_intronic	*LINC00841*	.	.	.
10	44411053	C	A	ncRNA_intronic	*LINC00841*	.	.	.
10	44411216	G	A	ncRNA_intronic	*LINC00841*	.	.	.
10	44411365	C	T	ncRNA_intronic	*LINC00841*	.	.	.
10	44485469	A	G	intergenic	*LINC00841,C10orf142*	.	.	.
10	44572939	G	A	intergenic	*LINC00841,C10orf142*	.	.	.
10	44588811	A	T	intergenic	*LINC00841,C10orf142*	.	.	.
10	44590854	A	G	intergenic	*LINC00841,C10orf142*	.	.	.
10	44592101	A	C	intergenic	*LINC00841,C10orf142*	.	.	.
10	44603740	A	G	intergenic	*LINC00841,C10orf142*	.	.	.
10	44634544	A	G	intergenic	*LINC00841,C10orf142*	.	.	.
10	44634546	G	A	intergenic	*LINC00841,C10orf142*	.	.	.
10	44635140	C	T	intergenic	*LINC00841,C10orf142*	.	.	.
10	44643021	A	G	intergenic	*LINC00841,C10orf142*	.	.	.

10	44644939	A	G	intergenic	*LINC00841,C10orf142*	.	.	.
10	44685636	T	C	intergenic	*LINC00841,C10orf142*	.	.	.
10	44722419	T	A	intergenic	*LINC00841,C10orf142*	.	.	.
10	44742670	A	T	intergenic	*LINC00841,C10orf142*	.	.	.
10	44742971	G	C	intergenic	*LINC00841,C10orf142*	.	.	.
10	44768527	G	A	intergenic	*LINC00841,C10orf142*	.	.	.
10	44771069	G	T	intergenic	*LINC00841,C10orf142*	.	.	.
10	44771070	G	T	intergenic	*LINC00841,C10orf142*	.	.	.
10	60863351	G	A	intergenic	*LINC00844,CCEPR*	.	.	.
10	60835022	T	C	intergenic	*LINC00844,CCEPR*	.	.	.
10	60798092	C	T	intergenic	*LINC00844,CCEPR*	.	.	.
10	60798993	C	G	intergenic	*LINC00844,CCEPR*	.	.	.
10	60801361	A	G	intergenic	*LINC00844,CCEPR*	.	.	.
10	62815002	C	CACA	intergenic	*LINC00845,TMEM26*	.	.	.
10	62815003	T	A	intergenic	*LINC00845,TMEM26*	.	.	.
10	62854245	A	G	intergenic	*LINC00845,TMEM26*	.	.	.
10	63139445	T	C	intergenic	*LINC00845,TMEM26*	.	.	.
10	63140527	A	G	intergenic	*LINC00845,TMEM26*	.	.	.
10	63152380	T	C	intergenic	*LINC00845,TMEM26*	.	.	.

10	63152392	C	T	intergenic	*LINC00845,TMEM26*	.	.	.
10	81984245	C	G	intergenic	*LINC00857,MAT1A*	.	.	.
10	81986268	TTTGTTG	T	intergenic	*LINC00857,MAT1A*	.	.	.
10	82026962	A	C	intergenic	*LINC00857,MAT1A*	.	.	.
10	86042308	T	C	ncRNA_intronic	*LINC00858*	.	.	.
8	127006599	T	C	intergenic	*LINC00861,LOC101927657*	.	.	.
8	127289952	T	A	intergenic	*LINC00861,LOC101927657*	.	.	.
8	126966552	C	T	intergenic	*LINC00861,LOC101927657*	.	.	.
8	127038283	A	G	intergenic	*LINC00861,LOC101927657*	.	.	.
8	127206821	C	A	intergenic	*LINC00861,LOC101927657*	.	.	.
8	127216972	C	A	intergenic	*LINC00861,LOC101927657*	.	.	.
10	89182149	C	T	intergenic	*LINC00864,MIR4678*	.	.	.
10	91668244	G	A	intergenic	*LINC00865,LINC01375*	.	.	.
10	91669831	G	A	intergenic	*LINC00865,LINC01375*	.	.	.
10	120226366	T	C	intergenic	*LINC00867,PRLHR*	.	.	.
10	120295221	GCATTAAATCACCCAGCACCCTCCCAAGACAGTGCTAGGTGATTTAATGCTGCAT	G	intergenic	*LINC00867,PRLHR*	.	.	.
14	46558732	A	T	ncRNA_intronic	*LINC00871*	.	.	.
14	46662125	C	T	ncRNA_intronic	*LINC00871*	.	.	.
14	46880318	C	G	ncRNA_intronic	*LINC00871*	.	.	.
14	46670799	G	A	ncRNA_intronic	*LINC00871*	.	.	.
14	46567897	T	C	ncRNA_intronic	*LINC00871*	.	.	.
14	46631438	C	G	ncRNA_intronic	*LINC00871*	.	.	.

14	46633383	G	T	ncRNA_intronic	*LINC00871*	.	.	.
14	46634085	C	A	ncRNA_intronic	*LINC00871*	.	.	.
14	46650223	A	G	ncRNA_intronic	*LINC00871*	.	.	.
14	46695881	A	G	ncRNA_intronic	*LINC00871*	.	.	.
14	46733133	T	C	ncRNA_intronic	*LINC00871*	.	.	.
14	46785849	G	A	ncRNA_intronic	*LINC00871*	.	.	.
14	46909913	G	A	ncRNA_intronic	*LINC00871*	.	.	.
14	47079049	T	A	intergenic	*LINC00871,RPL10L*	.	.	.
14	47001735	C	T	intergenic	*LINC00871,RPL10L*	.	.	.
14	47001736	C	T	intergenic	*LINC00871,RPL10L*	.	.	.
3	72095891	G	A	ncRNA_intronic	*LINC00877*	.	.	.
3	72174204	C	T	intergenic	*LINC00877,LINC00870*	.	.	.
3	95183932	A	T	intergenic	*LINC00879,MTHFD2P1*	.	.	.
3	94738938	C	A	intergenic	*LINC00879,MTHFD2P1*	.	.	.
3	94942400	G	C	intergenic	*LINC00879,MTHFD2P1*	.	.	.
3	95049310	A	T	intergenic	*LINC00879,MTHFD2P1*	.	.	.
3	95024928	T	C	intergenic	*LINC00879,MTHFD2P1*	.	.	.
3	94718112	T	C	intergenic	*LINC00879,MTHFD2P1*	.	.	.
3	94750936	A	T	intergenic	*LINC00879,MTHFD2P1*	.	.	.
3	94779398	G	A	intergenic	*LINC00879,MTHFD2P1*	.	.	.
3	94782786	C	T	intergenic	*LINC00879,MTHFD2P1*	.	.	.
3	94829718	G	C	intergenic	*LINC00879,MTHFD2P1*	.	.	.
3	94901074	C	T	intergenic	*LINC00879,MTHFD2P1*	.	.	.

3	94921114	C	T	intergenic	*LINC00879,MTHFD2P1*	.	.	.
3	95367223	A	G	intergenic	*LINC00879,MTHFD2P1*	.	.	.
3	106865060	C	T	ncRNA_intronic	*LINC00882*	.	.	.
3	194258168	C	T	intergenic	*LINC00884,TMEM44-AS1*	.	.	.
3	195892071	GT	G	intergenic	*LINC00885,ZDHHC19*	.	.	.
3	156493536	G	A	ncRNA_intronic	*LINC00886*	.	.	.
X	149295299	G	T	intergenic	*LINC00894,MIR2114*	.	.	.
X	149357238	T	C	intergenic	*LINC00894,MIR2114*	.	.	.
X	149193859	C	T	intergenic	*LINC00894,MIR2114*	.	.	.
X	149235367	C	CT	intergenic	*LINC00894,MIR2114*	.	.	.
X	149237209	T	C	intergenic	*LINC00894,MIR2114*	.	.	.
X	149247019	G	T	intergenic	*LINC00894,MIR2114*	.	.	.
X	149339969	T	C	intergenic	*LINC00894,MIR2114*	.	.	.
X	149339970	G	A	intergenic	*LINC00894,MIR2114*	.	.	.
11	115730642	C	A	intergenic	*LINC00900,BUD13*	.	.	.
11	115941201	C	T	intergenic	*LINC00900,BUD13*	.	.	.
11	116014193	A	T	intergenic	*LINC00900,BUD13*	.	.	.
11	116105799	T	C	intergenic	*LINC00900,BUD13*	.	.	.
11	115728050	C	T	intergenic	*LINC00900,BUD13*	.	.	.
11	115878710	C	A	intergenic	*LINC00900,BUD13*	.	.	.
11	115881734	T	A	intergenic	*LINC00900,BUD13*	.	.	.
11	116433311	T	A	intergenic	*LINC00900,BUD13*	.	.	.
11	116433313	A	ATCGG	intergenic	*LINC00900,BUD13*	.	.	.
11	116490188	A	T	intergenic	*LINC00900,BUD13*	.	.	.
11	116490706	G	C	intergenic	*LINC00900,BUD13*	.	.	.
11	116503590	T	A	intergenic	*LINC00900,BUD13*	.	.	.

11	116540275	C	G	intergenic	*LINC00900,BUD13*	.	.	.
11	116540813	T	TA	intergenic	*LINC00900,BUD13*	.	.	.
11	116541575	A	G	intergenic	*LINC00900,BUD13*	.	.	.
11	116542880	T	C	intergenic	*LINC00900,BUD13*	.	.	.
3	116665561	GT	G	intergenic	*LINC00901,IGSF11*	.	.	.
3	117622340	G	T	intergenic	*LINC00901,IGSF11*	.	.	.
3	117981507	A	G	intergenic	*LINC00901,IGSF11*	.	.	.
3	118244207	G	C	intergenic	*LINC00901,IGSF11*	.	.	.
3	117137246	A	G	intergenic	*LINC00901,IGSF11*	.	.	.
3	117317025	T	C	intergenic	*LINC00901,IGSF11*	.	.	.
3	117875448	GA	G	intergenic	*LINC00901,IGSF11*	.	.	.
3	117611497	C	G	intergenic	*LINC00901,IGSF11*	.	.	.
3	118495415	CATATAT	C	intergenic	*LINC00901,IGSF11*	.	.	.
18	40251654	A	G	ncRNA_intronic	*LINC00907*	.	.	.
18	39851408	G	T	ncRNA_intronic	*LINC00907*	.	.	.
18	39845689	G	GGTTA	ncRNA_intronic	*LINC00907*	.	.	.
18	40127446	C	T	ncRNA_intronic	*LINC00907*	.	.	.
18	40127448	G	T	ncRNA_intronic	*LINC00907*	.	.	.
18	40244286	A	C	ncRNA_intronic	*LINC00907*	.	.	.
18	40245449	G	A	ncRNA_intronic	*LINC00907*	.	.	.
18	40246478	G	T	ncRNA_intronic	*LINC00907*	.	.	.
18	40296271	G	A	intergenic	*LINC00907,RIT2*	.	.	.
18	74320176	G	A	intergenic	*LINC00908,LINC00683*	.	.	.
18	72335127	G	A	intergenic	*LINC00909,ZNF407*	.	.	.
14	85911386	C	A	intergenic	*LINC00911,FLRT2*	.	.	.
14	85942607	G	A	intergenic	*LINC00911,FLRT2*	.	.	.

16	86412473	G	A	intergenic	*LINC00917,FENDRR*	.	.	.
16	65419651	A	T	ncRNA_intronic	*LINC00922*	.	.	.
16	65319879	AT	A	ncRNA_intronic	*LINC00922*	.	.	.
16	65382447	AT	A	ncRNA_intronic	*LINC00922*	.	.	.
16	65419744	T	A	ncRNA_intronic	*LINC00922*	.	.	.
16	65384336	C	T	ncRNA_intronic	*LINC00922*	.	.	.
16	65469547	A	G	ncRNA_intronic	*LINC00922*	.	.	.
16	66027953	C	T	intergenic	*LINC00922,CDH5*	.	.	.
16	66181785	T	C	intergenic	*LINC00922,CDH5*	.	.	.
16	65700343	G	T	intergenic	*LINC00922,CDH5*	.	.	.
16	65836649	A	T	intergenic	*LINC00922,CDH5*	.	.	.
16	65963695	A	C	intergenic	*LINC00922,CDH5*	.	.	.
15	98331812	GCTCT	G	ncRNA_intronic	*LINC00923*	.	.	.
15	96055497	G	A	intergenic	*LINC00924,NR2F2-AS1*	.	.	.
15	96171205	T	A	intergenic	*LINC00924,NR2F2-AS1*	.	.	.
15	96288767	A	C	intergenic	*LINC00924,NR2F2-AS1*	.	.	.
15	96319139	G	A	intergenic	*LINC00924,NR2F2-AS1*	.	.	.
15	90078498	G	A	intergenic	*LINC00928,TICRR*	.	.	.
15	26696499	T	C	intergenic	*LINC00929,GABRB3*	.	.	.
12	119768838	G	T	intergenic	*LINC00934,CCDC60*	.	.	.
12	90547287	C	T	intergenic	*LINC00936,LINC00615*	.	.	.
12	90756869	TACAC	T	intergenic	*LINC00936,LINC00615*	.	.	.
12	90786821	T	A	intergenic	*LINC00936,LINC00615*	.	.	.
12	90795603	A	T	intergenic	*LINC00936,LINC00615*	.	.	.
12	91194594	GTATATA	G	intergenic	*LINC00936,LINC00615*	.	.	.

12	91018783	T	G	intergenic	*LINC00936,LINC00615*	.	.	.
12	90703834	G	A	intergenic	*LINC00936,LINC00615*	.	.	.
12	91042237	TTATTCTTATTACCAAATAAGAATATATTCTTATTATATATATAAGAATA	T	intergenic	*LINC00936,LINC00615*	.	.	.
12	90840283	C	A	intergenic	*LINC00936,LINC00615*	.	.	.
12	90121822	G	A	intergenic	*LINC00936,LINC00615*	.	.	.
12	90121823	C	T	intergenic	*LINC00936,LINC00615*	.	.	.
12	90483298	G	A	intergenic	*LINC00936,LINC00615*	.	.	.
12	90531842	G	A	intergenic	*LINC00936,LINC00615*	.	.	.
12	90614070	T	G	intergenic	*LINC00936,LINC00615*	.	.	.
12	90634548	A	G	intergenic	*LINC00936,LINC00615*	.	.	.
12	91043195	T	G	intergenic	*LINC00936,LINC00615*	.	.	.
12	91145608	T	C	intergenic	*LINC00936,LINC00615*	.	.	.
12	91146362	G	A	intergenic	*LINC00936,LINC00615*	.	.	.
12	91146939	T	C	intergenic	*LINC00936,LINC00615*	.	.	.
12	8528080	C	T	ncRNA_intronic	*LINC00937*	.	.	.
12	8536353	T	C	ncRNA_intronic	*LINC00937*	.	.	.
12	126513902	T	A	intergenic	*LINC00939,LOC101927464*	.	.	.
12	126494428	C	T	intergenic	*LINC00939,LOC101927464*	.	.	.

12	126494429	T	C	intergenic	*LINC00939,LOC101927464*	.	.	.
12	126530917	A	G	intergenic	*LINC00939,LOC101927464*	.	.	.
12	2038135	C	T	downstream	*LINC00940*	.	.	.
12	2052221	A	C	intergenic	*LINC00940,DCP1B*	.	.	.
12	2054021	G	A	intergenic	*LINC00940,DCP1B*	.	.	.
12	30971983	TAGAG	T	intergenic	*LINC00941,TSPAN11*	.	.	.
12	31044663	T	C	intergenic	*LINC00941,TSPAN11*	.	.	.
12	1624830	C	CTTTTTTTTT	intergenic	*LINC00942,FBXL14*	.	.	.
12	127256763	G	A	ncRNA_exonic	*LINC00944*	.	.	.
12	127283977	G	A	intergenic	*LINC00944,LOC440117*	.	.	.
10	131871727	A	T	ncRNA_intronic	*LINC00959*	.	.	.
10	131891111	G	C	ncRNA_intronic	*LINC00959*	.	.	.
10	131893575	A	G	ncRNA_intronic	*LINC00959*	.	.	.
10	131894252	T	C	ncRNA_intronic	*LINC00959*	.	.	.
3	75755066	C	T	intergenic	*LINC00960,ZNF717*	.	.	.
8	7128881	A	G	upstream	*LINC00965*	.	.	.
8	57464728	A	G	ncRNA_intronic	*LINC00968*	.	.	.
8	57800363	GGTGT	G	intergenic	*LINC00968,IMPAD1*	.	.	.
8	57651426	C	A	intergenic	*LINC00968,IMPAD1*	.	.	.
8	57868603	AATATAT	A	intergenic	*LINC00968,IMPAD1*	.	.	.
8	57546779	A	C	intergenic	*LINC00968,IMPAD1*	.	.	.
8	57546781	A	G	intergenic	*LINC00968,IMPAD1*	.	.	.
8	57686744	A	G	intergenic	*LINC00968,IMPAD1*	.	.	.
8	57722133	T	C	intergenic	*LINC00968,IMPAD1*	.	.	.

8	57722652	T	G	intergenic	*LINC00968,IMPAD1*	.	.	.
8	57766212	G	T	intergenic	*LINC00968,IMPAD1*	.	.	.
8	57461797	G	C	ncRNA_intronic	*LINC00968,LOC101929415*	.	.	.
3	195437208	C	G	ncRNA_intronic	*LINC00969*	.	.	.
1	168962365	A	C	ncRNA_intronic	*LINC00970*	.	.	.
1	168962950	C	A	ncRNA_intronic	*LINC00970*	.	.	.
1	168965381	C	G	ncRNA_intronic	*LINC00970*	.	.	.
1	168965383	C	G	ncRNA_intronic	*LINC00970*	.	.	.
1	169071278	A	G	intergenic	*LINC00970,ATP1B1*	.	.	.
3	84718605	A	G	ncRNA_exonic	*LINC00971*	.	.	.
3	84784160	A	G	ncRNA_intronic	*LINC00971*	.	.	.
3	84827165	A	C	ncRNA_intronic	*LINC00971*	.	.	.
3	84862235	A	T	ncRNA_intronic	*LINC00971*	.	.	.
8	130323987	G	C	intergenic	*LINC00977,CCDC26*	.	.	.
8	130339250	C	T	intergenic	*LINC00977,CCDC26*	.	.	.
8	130341021	C	G	intergenic	*LINC00977,CCDC26*	.	.	.
12	9399450	C	T	intergenic	*LINC00987,LOC642846*	.	.	.
4	80414407	A	C	ncRNA_exonic	*LINC00989*	.	.	.
4	80579272	A	T	intergenic	*LINC00989,PCAT4*	.	.	.
5	116854367	C	A	ncRNA_intronic	*LINC00992*	.	.	.
5	116828265	A	C	ncRNA_intronic	*LINC00992*	.	.	.
7	32846829	C	T	intergenic	*LINC00997,DPY19L1P2*	.	.	.
7	32840641	CT	C	intergenic	*LINC00997,DPY19L1P2*	.	.	.
7	112878884	G	A	intergenic	*LINC00998,PPP1R3A*	.	.	.
7	113096960	A	T	intergenic	*LINC00998,PPP1R3A*	.	.	.

7	113128390	C	A	intergenic	*LINC00998,PPP1R3A*	.	.	.
7	113201916	G	A	intergenic	*LINC00998,PPP1R3A*	.	.	.
7	113399459	C	T	intergenic	*LINC00998,PPP1R3A*	.	.	.
7	113264419	T	A	intergenic	*LINC00998,PPP1R3A*	.	.	.
7	112987377	G	A	intergenic	*LINC00998,PPP1R3A*	.	.	.
7	113010889	C	T	intergenic	*LINC00998,PPP1R3A*	.	.	.
7	113025929	G	A	intergenic	*LINC00998,PPP1R3A*	.	.	.
7	113111673	C	G	intergenic	*LINC00998,PPP1R3A*	.	.	.
7	113113059	T	G	intergenic	*LINC00998,PPP1R3A*	.	.	.
7	113113602	T	C	intergenic	*LINC00998,PPP1R3A*	.	.	.
7	113243413	A	G	intergenic	*LINC00998,PPP1R3A*	.	.	.
7	113254938	T	C	intergenic	*LINC00998,PPP1R3A*	.	.	.
7	113387238	T	C	intergenic	*LINC00998,PPP1R3A*	.	.	.
7	113388686	G	A	intergenic	*LINC00998,PPP1R3A*	.	.	.
7	113397126	C	G	intergenic	*LINC00998,PPP1R3A*	.	.	.
7	113451998	G	A	intergenic	*LINC00998,PPP1R3A*	.	.	.
7	113500582	C	T	intergenic	*LINC00998,PPP1R3A*	.	.	.
7	113500664	G	A	intergenic	*LINC00998,PPP1R3A*	.	.	.
7	113508218	C	A	intergenic	*LINC00998,PPP1R3A*	.	.	.

10	38719408	A	G	ncRNA_intronic	*LINC00999*	.	.	.
10	38734686	G	GAAT	ncRNA_intronic	*LINC00999*	.	.	.
10	38964449	A	G	intergenic	*LINC00999,ACTR3BP5*	.	.	.
10	38959710	A	G	intergenic	*LINC00999,ACTR3BP5*	.	.	.
10	38929834	CTT	C	intergenic	*LINC00999,ACTR3BP5*	.	.	.
10	38814880	G	A	intergenic	*LINC00999,ACTR3BP5*	.	.	.
10	38906181	C	A	intergenic	*LINC00999,ACTR3BP5*	.	.	.
10	38955281	T	C	intergenic	*LINC00999,ACTR3BP5*	.	.	.
10	38955282	G	A	intergenic	*LINC00999,ACTR3BP5*	.	.	.
10	38956760	G	A	intergenic	*LINC00999,ACTR3BP5*	.	.	.
10	38957362	T	A	intergenic	*LINC00999,ACTR3BP5*	.	.	.
7	152206974	A	T	intergenic	*LINC01003,XRCC2*	.	.	.
7	152262254	T	C	intergenic	*LINC01003,XRCC2*	.	.	.
7	156294221	T	C	ncRNA_intronic	*LINC01006*	.	.	.
7	156294223	C	G	ncRNA_intronic	*LINC01006*	.	.	.
7	156392206	G	A	ncRNA_intronic	*LINC01006*	.	.	.
5	3498443	A	G	ncRNA_intronic	*LINC01017,LINC01019*	.	.	.
5	3416814	C	A	downstream	*LINC01019*	.	.	.
5	3417770	G	T	ncRNA_exonic	*LINC01019*	.	.	.
5	3480977	A	T	ncRNA_intronic	*LINC01019*	.	.	.
5	3480979	A	T	ncRNA_intronic	*LINC01019*	.	.	.
5	27493514	G	A	ncRNA_intronic	*LINC01021*	.	.	.
5	27633055	G	T	intergenic	*LINC01021,LSP1P3*	.	.	.
5	27984171	G	T	intergenic	*LINC01021,LSP1P3*	.	.	.
5	27990128	C	G	intergenic	*LINC01021,LSP1P3*	.	.	.

5	28711636	A	ATT	intergenic	*LINC01021,LSP1P3*	.	.	.
5	28557095	C	T	intergenic	*LINC01021,LSP1P3*	.	.	.
5	27996976	C	T	intergenic	*LINC01021,LSP1P3*	.	.	.
5	28710754	CTTTG	C	intergenic	*LINC01021,LSP1P3*	.	.	.
5	28815858	A	AT	intergenic	*LINC01021,LSP1P3*	.	.	.
5	27622725	G	T	intergenic	*LINC01021,LSP1P3*	.	.	.
5	27975146	G	A	intergenic	*LINC01021,LSP1P3*	.	.	.
5	28527844	T	TACAC	intergenic	*LINC01021,LSP1P3*	.	.	.
5	27787465	G	T	intergenic	*LINC01021,LSP1P3*	.	.	.
5	28696381	A	T	intergenic	*LINC01021,LSP1P3*	.	.	.
5	27852786	GA	G	intergenic	*LINC01021,LSP1P3*	.	.	.
5	27982814	TGAGA	T	intergenic	*LINC01021,LSP1P3*	.	.	.
5	28555063	C	A	intergenic	*LINC01021,LSP1P3*	.	.	.
5	27538915	A	G	intergenic	*LINC01021,LSP1P3*	.	.	.
5	27885432	A	G	intergenic	*LINC01021,LSP1P3*	.	.	.
5	27886394	A	G	intergenic	*LINC01021,LSP1P3*	.	.	.
5	27887337	T	C	intergenic	*LINC01021,LSP1P3*	.	.	.
5	27887338	G	T	intergenic	*LINC01021,LSP1P3*	.	.	.
5	28185549	C	T	intergenic	*LINC01021,LSP1P3*	.	.	.

5	28485076	A	G	intergenic	*LINC01021,LSP1P3*	.	.	.
5	28536530	T	TG	intergenic	*LINC01021,LSP1P3*	.	.	.
5	28758205	C	T	intergenic	*LINC01021,LSP1P3*	.	.	.
5	28803806	T	C	intergenic	*LINC01021,LSP1P3*	.	.	.
5	28845380	T	G	intergenic	*LINC01021,LSP1P3*	.	.	.
18	76611404	GCTATGGAATGTAAAATGGTTCACCCA	G	intergenic	*LINC01029,SALL3*	.	.	.
18	75993148	A	C	intergenic	*LINC01029,SALL3*	.	.	.
18	75993149	G	A	intergenic	*LINC01029,SALL3*	.	.	.
18	76034473	A	T	intergenic	*LINC01029,SALL3*	.	.	.
18	76072665	A	G	intergenic	*LINC01029,SALL3*	.	.	.
18	76659575	A	T	intergenic	*LINC01029,SALL3*	.	.	.
18	76168437	G	T	intergenic	*LINC01029,SALL3*	.	.	.
18	76629514	AATATAT	A	intergenic	*LINC01029,SALL3*	.	.	.
18	75706901	C	G	intergenic	*LINC01029,SALL3*	.	.	.
18	75830527	A	G	intergenic	*LINC01029,SALL3*	.	.	.
18	75850659	A	C	intergenic	*LINC01029,SALL3*	.	.	.
18	75896586	C	A	intergenic	*LINC01029,SALL3*	.	.	.
18	76448677	A	C	intergenic	*LINC01029,SALL3*	.	.	.
18	76599816	A	G	intergenic	*LINC01029,SALL3*	.	.	.
18	76599818	G	T	intergenic	*LINC01029,SALL3*	.	.	.
18	76119114	A	C	intergenic	*LINC01029,SALL3*	.	.	.
18	76600934	A	G	intergenic	*LINC01029,SALL3*	.	.	.
8	91616178	G	A	ncRNA_intronic	*LINC01030*	.	.	.
1	193306427	T	C	ncRNA_intronic	*LINC01031*	.	.	.
1	193306428	A	AC	ncRNA_intronic	*LINC01031*	.	.	.
1	194344335	T	A	intergenic	*LINC01031,KCNT2*	.	.	.
1	194800362	C	A	intergenic	*LINC01031,KCNT2*	.	.	.
1	194209500	ATT	A	intergenic	*LINC01031,KCNT2*	.	.	.
1	194590113	A	C	intergenic	*LINC01031,KCNT2*	.	.	.
1	194884277	T	C	intergenic	*LINC01031,KCNT2*	.	.	.

1	195223467	G	A	intergenic	*LINC01031,KCNT2*	.	.	.
1	195281024	C	A	intergenic	*LINC01031,KCNT2*	.	.	.
1	194959923	G	T	intergenic	*LINC01031,KCNT2*	.	.	.
1	194703173	A	T	intergenic	*LINC01031,KCNT2*	.	.	.
1	194835288	TTCTC	T	intergenic	*LINC01031,KCNT2*	.	.	.
1	194170353	T	C	intergenic	*LINC01031,KCNT2*	.	.	.
1	194171094	A	G	intergenic	*LINC01031,KCNT2*	.	.	.
1	194284338	G	C	intergenic	*LINC01031,KCNT2*	.	.	.
1	194462123	G	A	intergenic	*LINC01031,KCNT2*	.	.	.
1	194510717	A	G	intergenic	*LINC01031,KCNT2*	.	.	.
1	194577461	G	T	intergenic	*LINC01031,KCNT2*	.	.	.
1	194578334	G	T	intergenic	*LINC01031,KCNT2*	.	.	.
1	194578708	T	A	intergenic	*LINC01031,KCNT2*	.	.	.
1	194597355	C	A	intergenic	*LINC01031,KCNT2*	.	.	.
1	194597357	A	G	intergenic	*LINC01031,KCNT2*	.	.	.
1	194654748	T	C	intergenic	*LINC01031,KCNT2*	.	.	.
1	194729469	G	C	intergenic	*LINC01031,KCNT2*	.	.	.
1	195014958	A	T	intergenic	*LINC01031,KCNT2*	.	.	.
1	195147558	C	T	intergenic	*LINC01031,KCNT2*	.	.	.
1	195196303	C	T	intergenic	*LINC01031,KCNT2*	.	.	.
1	193456405	T	G	intergenic	*LINC01031,NONE*	.	.	.
1	193510216	T	A	intergenic	*LINC01031,NONE*	.	.	.
1	193754649	C	G	intergenic	*LINC01031,NONE*	.	.	.
1	193923821	T	A	intergenic	*LINC01031,NONE*	.	.	.
1	193480734	C	T	intergenic	*LINC01031,NONE*	.	.	.
1	193609071	A	G	intergenic	*LINC01031,NONE*	.	.	.
1	193611303	G	C	intergenic	*LINC01031,NONE*	.	.	.
1	193611642	T	TAGGGTG	intergenic	*LINC01031,NONE*	.	.	.
1	193613388	T	C	intergenic	*LINC01031,NONE*	.	.	.
1	193688470	T	C	intergenic	*LINC01031,NONE*	.	.	.
1	193718155	C	T	intergenic	*LINC01031,NONE*	.	.	.
1	193765637	C	T	intergenic	*LINC01031,NONE*	.	.	.
1	193773317	C	G	intergenic	*LINC01031,NONE*	.	.	.
1	193805610	G	A	intergenic	*LINC01031,NONE*	.	.	.
1	193840582	A	G	intergenic	*LINC01031,NONE*	.	.	.
1	193844340	C	T	intergenic	*LINC01031,NONE*	.	.	.
1	193987085	T	C	intergenic	*LINC01031,NONE*	.	.	.
1	193988154	A	T	intergenic	*LINC01031,NONE*	.	.	.

5	53679753	A	T	ncRNA_intronic	*LINC01033*	.	.	.
5	53632948	A	ATCTTTCTAC	ncRNA_intronic	*LINC01033*	.	.	.
5	53634964	TA	T	ncRNA_intronic	*LINC01033*	.	.	.
5	53634966	TTA	T	ncRNA_intronic	*LINC01033*	.	.	.
5	53690294	C	T	ncRNA_intronic	*LINC01033*	.	.	.
1	187178782	CATAT	C	ncRNA_intronic	*LINC01036*	.	.	.
1	187323559	C	T	ncRNA_intronic	*LINC01036*	.	.	.
1	187090239	G	T	ncRNA_intronic	*LINC01036*	.	.	.
1	187104422	A	G	ncRNA_intronic	*LINC01036*	.	.	.
1	187104729	C	T	ncRNA_intronic	*LINC01036*	.	.	.
1	187110439	C	CA	ncRNA_intronic	*LINC01036*	.	.	.
1	189084958	CTTT	C	intergenic	*LINC01037,BRINP3*	.	.	.
1	188861685	AATATAT	A	intergenic	*LINC01037,BRINP3*	.	.	.
1	189004958	A	AAAAT	intergenic	*LINC01037,BRINP3*	.	.	.
1	188999989	T	A	intergenic	*LINC01037,BRINP3*	.	.	.
1	188207636	C	CT	intergenic	*LINC01037,BRINP3*	.	.	.
1	188385960	G	A	intergenic	*LINC01037,BRINP3*	.	.	.
1	189227080	T	C	intergenic	*LINC01037,BRINP3*	.	.	.
1	189309087	T	A	intergenic	*LINC01037,BRINP3*	.	.	.
1	188435332	A	G	intergenic	*LINC01037,BRINP3*	.	.	.
1	188652885	C	T	intergenic	*LINC01037,BRINP3*	.	.	.
1	188115133	T	A	intergenic	*LINC01037,BRINP3*	.	.	.
1	188321977	G	A	intergenic	*LINC01037,BRINP3*	.	.	.
1	188489442	T	C	intergenic	*LINC01037,BRINP3*	.	.	.

1	188597498	G	A	intergenic	*LINC01037,BRINP3*	.	.	.
1	188723944	C	T	intergenic	*LINC01037,BRINP3*	.	.	.
1	188728169	T	C	intergenic	*LINC01037,BRINP3*	.	.	.
1	188781272	G	A	intergenic	*LINC01037,BRINP3*	.	.	.
1	188781273	G	A	intergenic	*LINC01037,BRINP3*	.	.	.
1	188868502	G	C	intergenic	*LINC01037,BRINP3*	.	.	.
1	188951074	G	T	intergenic	*LINC01037,BRINP3*	.	.	.
1	189001675	G	A	intergenic	*LINC01037,BRINP3*	.	.	.
1	189002015	A	C	intergenic	*LINC01037,BRINP3*	.	.	.
1	189134712	T	A	intergenic	*LINC01037,BRINP3*	.	.	.
1	189178969	A	G	intergenic	*LINC01037,BRINP3*	.	.	.
1	189267588	G	A	intergenic	*LINC01037,BRINP3*	.	.	.
1	189299044	T	G	intergenic	*LINC01037,BRINP3*	.	.	.
1	189368804	T	C	intergenic	*LINC01037,BRINP3*	.	.	.
1	189380417	A	T	intergenic	*LINC01037,BRINP3*	.	.	.
1	189380419	T	C	intergenic	*LINC01037,BRINP3*	.	.	.
1	189382045	C	T	intergenic	*LINC01037,BRINP3*	.	.	.
1	189436103	A	T	intergenic	*LINC01037,BRINP3*	.	.	.
1	187990923	C	A	intergenic	*LINC01037,NONE*	.	.	.
1	187585989	A	G	intergenic	*LINC01037,NONE*	.	.	.

1	187836484	G	A	intergenic	*LINC01037,NONE*	.	.	.
1	187957609	C	G	intergenic	*LINC01037,NONE*	.	.	.
1	187817174	GCTCT	G	intergenic	*LINC01037,NONE*	.	.	.
1	187923612	C	G	intergenic	*LINC01037,NONE*	.	.	.
1	187510513	A	G	intergenic	*LINC01037,NONE*	.	.	.
1	187511311	C	G	intergenic	*LINC01037,NONE*	.	.	.
1	187551157	C	T	intergenic	*LINC01037,NONE*	.	.	.
1	187726113	A	G	intergenic	*LINC01037,NONE*	.	.	.
1	187726503	A	G	intergenic	*LINC01037,NONE*	.	.	.
1	187777230	C	T	intergenic	*LINC01037,NONE*	.	.	.
1	187777231	A	G	intergenic	*LINC01037,NONE*	.	.	.
1	187843488	A	T	intergenic	*LINC01037,NONE*	.	.	.
1	187843595	C	A	intergenic	*LINC01037,NONE*	.	.	.
1	187951496	C	T	intergenic	*LINC01037,NONE*	.	.	.
13	80393354	G	A	intergenic	*LINC01038,LINC00382*	.	.	.
13	100250683	A	G	intergenic	*LINC01039,CLYBL*	.	.	.
13	38072677	G	A	intergenic	*LINC01048,LINC00547*	.	.	.
13	91181667	G	A	ncRNA_intronic	*LINC01049*	.	.	.
13	91373945	G	T	intergenic	*LINC01049,LINC00410*	.	.	.
13	91407305	A	G	intergenic	*LINC01049,LINC00410*	.	.	.
13	91489852	T	C	intergenic	*LINC01049,LINC00410*	.	.	.
13	66451072	T	C	ncRNA_intronic	*LINC01052*	.	.	.
13	66457908	G	C	intergenic	*LINC01052,MIR548X2*	.	.	.
13	115109112	T	C	intergenic	*LINC01054,NONE*	.	.	.
1	95176747	CTG	C	ncRNA_intronic	*LINC01057*	.	.	.
4	189400772	A	G	ncRNA_intronic	*LINC01060*	.	.	.
4	189479806	A	T	ncRNA_intronic	*LINC01060*	.	.	.
4	189932143	G	C	intergenic	*LINC01060,LINC01262*	.	.	.
4	190450841	C	A	intergenic	*LINC01060,LINC01262*	.	.	.

4	189654114	G	A	intergenic	*LINC01060,LINC01262*	.	.	.
4	189599471	G	C	intergenic	*LINC01060,LINC01262*	.	.	.
4	189599472	C	T	intergenic	*LINC01060,LINC01262*	.	.	.
4	189781116	T	C	intergenic	*LINC01060,LINC01262*	.	.	.
4	190146821	G	A	intergenic	*LINC01060,LINC01262*	.	.	.
4	190400615	T	G	intergenic	*LINC01060,LINC01262*	.	.	.
13	54149828	A	G	intergenic	*LINC01065,LINC00558*	.	.	.
13	54375798	T	C	intergenic	*LINC01065,LINC00558*	.	.	.
13	53871172	C	T	intergenic	*LINC01065,LINC00558*	.	.	.
13	53871304	C	A	intergenic	*LINC01065,LINC00558*	.	.	.
13	53900186	T	C	intergenic	*LINC01065,LINC00558*	.	.	.
13	53965902	G	T	intergenic	*LINC01065,LINC00558*	.	.	.
13	54171736	G	A	intergenic	*LINC01065,LINC00558*	.	.	.
13	54173365	A	G	intergenic	*LINC01065,LINC00558*	.	.	.
13	54194304	C	T	intergenic	*LINC01065,LINC00558*	.	.	.
13	80145865	T	C	downstream	*LINC01068*	.	.	.
13	80152806	G	A	intergenic	*LINC01068,LINC01038*	.	.	.
13	78575544	C	A	intergenic	*LINC01069,LINC00446*	.	.	.
13	78576636	A	T	intergenic	*LINC01069,LINC00446*	.	.	.

13	78576637	C	T		intergenic	*LINC01069,LINC00446*	.	.	.
13	62816859	G	T		ncRNA_intronic	*LINC01075*	.	.	.
13	63010684	G	A		intergenic	*LINC01075,LINC00448*	.	.	.
13	63108482	T	TGAG		intergenic	*LINC01075,LINC00448*	.	.	.
13	63178487	C	A		intergenic	*LINC01075,LINC00448*	.	.	.
13	63204368	C	T		intergenic	*LINC01075,LINC00448*	.	.	.
13	75818355	C	T		ncRNA_intronic	*LINC01078*	.	.	.
13	75849972	C	G		intergenic	*LINC01078,TBC1D4*	.	.	.
13	80821253	A	T		intergenic	*LINC01080,SPRY2*	.	.	.
13	80821255	T	C		intergenic	*LINC01080,SPRY2*	.	.	.
13	80627039	T	G		intergenic	*LINC01080,SPRY2*	.	.	.
4	14135675	T	C		ncRNA_intronic	*LINC01085*	.	.	.
4	14142535	C	A		downstream	*LINC01085*	.	.	.
4	14447175	A	C		intergenic	*LINC01085,LINC00504*	.	.	.
4	14220125	C	G		intergenic	*LINC01085,LINC00504*	.	.	.
4	14209619	C	T		intergenic	*LINC01085,LINC00504*	.	.	.
4	14423139	G	A		intergenic	*LINC01085,LINC00504*	.	.	.
4	14425923	T	C		intergenic	*LINC01085,LINC00504*	.	.	.
2	132456294	A	G		intergenic	*LINC01087,C2orf27A*	.	.	.
2	132441976	A	G		intergenic	*LINC01087,C2orf27A*	.	.	.
2	132451538	C	T		intergenic	*LINC01087,C2orf27A*	.	.	.
4	79898746	T	G		ncRNA_intronic	*LINC01088*	.	.	.
4	79900452	A	G		ncRNA_intronic	*LINC01088*	.	.	.
4	80026037	G	T		ncRNA_intronic	*LINC01088*	.	.	.

4	80089683	T	C	ncRNA_intronic	*LINC01088*	.	.	.
4	80143556	T	C	ncRNA_intronic	*LINC01088*	.	.	.
2	188953869	G	A	ncRNA_intronic	*LINC01090*	.	.	.
2	189092434	AG	A	ncRNA_intronic	*LINC01090*	.	.	.
2	189128394	G	T	ncRNA_intronic	*LINC01090*	.	.	.
4	124843649	C	T	ncRNA_intronic	*LINC01091*	.	.	.
4	124637059	T	C	ncRNA_intronic	*LINC01091*	.	.	.
4	124637320	A	C	ncRNA_intronic	*LINC01091*	.	.	.
4	124637321	G	A	ncRNA_intronic	*LINC01091*	.	.	.
4	124638511	A	AG	ncRNA_intronic	*LINC01091*	.	.	.
4	124638513	T	C	ncRNA_intronic	*LINC01091*	.	.	.
4	124931435	C	A	intergenic	*LINC01091,LOC101927087*	.	.	.
4	125048844	C	T	intergenic	*LINC01091,LOC101927087*	.	.	.
4	125050029	A	C	intergenic	*LINC01091,LOC101927087*	.	.	.
4	125050575	C	A	intergenic	*LINC01091,LOC101927087*	.	.	.
4	125161515	A	G	intergenic	*LINC01091,LOC101927087*	.	.	.
4	13553362	A	C	intergenic	*LINC01096,BOD1L1*	.	.	.
4	13557652	G	A	intergenic	*LINC01096,BOD1L1*	.	.	.
4	178869521	A	G	ncRNA_intronic	*LINC01098*	.	.	.
4	178670285	G	T	ncRNA_intronic	*LINC01098*	.	.	.
4	180055018	TA	T	intergenic	*LINC01098,LINC00290*	.	.	.
4	180253159	A	G	intergenic	*LINC01098,LINC00290*	.	.	.
4	180850952	C	A	intergenic	*LINC01098,LINC00290*	.	.	.
4	179953287	G	A	intergenic	*LINC01098,LINC00290*	.	.	.
4	180459419	G	A	intergenic	*LINC01098,LINC00290*	.	.	.

4	180562049	C	T	intergenic	*LINC01098,LINC00290*	.	.	.
4	180562050	T	C	intergenic	*LINC01098,LINC00290*	.	.	.
4	180601924	C	T	intergenic	*LINC01098,LINC00290*	.	.	.
4	180603672	A	C	intergenic	*LINC01098,LINC00290*	.	.	.
4	180605250	T	C	intergenic	*LINC01098,LINC00290*	.	.	.
4	180907403	T	G	intergenic	*LINC01098,LINC00290*	.	.	.
4	180966620	A	G	intergenic	*LINC01098,LINC00290*	.	.	.
4	179577173	TTCTC	T	intergenic	*LINC01098,NONE*	.	.	.
4	179668870	C	T	intergenic	*LINC01098,NONE*	.	.	.
4	179257696	A	G	intergenic	*LINC01098,NONE*	.	.	.
4	179375241	A	T	intergenic	*LINC01098,NONE*	.	.	.
4	179864928	TATATGG	T	intergenic	*LINC01098,NONE*	.	.	.
4	179210837	A	G	intergenic	*LINC01098,NONE*	.	.	.
4	179366892	C	T	intergenic	*LINC01098,NONE*	.	.	.
4	179433046	C	T	intergenic	*LINC01098,NONE*	.	.	.
4	179555864	G	A	intergenic	*LINC01098,NONE*	.	.	.
4	179561322	T	A	intergenic	*LINC01098,NONE*	.	.	.
4	179630273	A	C	intergenic	*LINC01098,NONE*	.	.	.
2	121246856	G	T	intergenic	*LINC01101,GLI2*	.	.	.
2	105090646	G	A	ncRNA_intronic	*LINC01102*	.	.	.
2	105164936	T	C	intergenic	*LINC01102,LINC01114*	.	.	.
2	105188093	A	G	intergenic	*LINC01102,LINC01114*	.	.	.
2	105345854	G	A	intergenic	*LINC01102,LINC01114*	.	.	.
2	105242033	AT	A	intergenic	*LINC01102,LINC01114*	.	.	.
2	105261463	A	C	intergenic	*LINC01102,LINC01114*	.	.	.
2	6087049	G	T	ncRNA_intronic	*LINC01105*	.	.	.

6	15120986	C	A	intergenic	*LINC01108,JARID2*	.	.	.
6	14592879	G	A	intergenic	*LINC01108,JARID2*	.	.	.
6	14419629	T	TAATA	intergenic	*LINC01108,JARID2*	.	.	.
6	14294165	T	G	intergenic	*LINC01108,JARID2*	.	.	.
8	77329284	A	G	ncRNA_intronic	*LINC01111*	.	.	.
8	77351263	A	C	ncRNA_intronic	*LINC01111*	.	.	.
2	105374591	CAGGGGATGGATGTCCACAGT	C	upstream	*LINC01114*	.	.	.
2	105382360	C	A	intergenic	*LINC01114,LINC01158*	.	.	.
2	865056	G	C	upstream	*LINC01115*	.	.	.
2	786672	C	T	ncRNA_intronic	*LINC01115*	.	.	.
2	177811402	T	C	intergenic	*LINC01117,HNRNPA3*	.	.	.
2	177959508	C	T	intergenic	*LINC01117,HNRNPA3*	.	.	.
2	177665459	G	A	intergenic	*LINC01117,HNRNPA3*	.	.	.
2	177666266	T	C	intergenic	*LINC01117,HNRNPA3*	.	.	.
2	177666378	A	G	intergenic	*LINC01117,HNRNPA3*	.	.	.
2	45609726	T	C	intergenic	*LINC01121,SRBD1*	.	.	.
2	58834163	G	A	ncRNA_intronic	*LINC01122*	.	.	.
2	59117816	T	C	ncRNA_intronic	*LINC01122*	.	.	.
2	58949122	T	A	ncRNA_intronic	*LINC01122*	.	.	.
2	59108167	C	T	ncRNA_intronic	*LINC01122*	.	.	.
2	59182494	A	G	ncRNA_intronic	*LINC01122*	.	.	.
2	58862160	A	C	ncRNA_intronic	*LINC01122*	.	.	.
2	59051360	T	C	ncRNA_intronic	*LINC01122*	.	.	.
2	59051999	G	T	ncRNA_intronic	*LINC01122*	.	.	.
2	59052495	C	A	ncRNA_intronic	*LINC01122*	.	.	.

2	59298494	C	G	intergenic	*LINC01122,LOC101927285*	.	.	.
2	59298442	A	G	intergenic	*LINC01122,LOC101927285*	.	.	.
1	235001724	C	CTTA	intergenic	*LINC01132,LOC101927851*	.	.	.
1	234967657	A	AAGATC	intergenic	*LINC01132,LOC101927851*	.	.	.
1	234871390	G	A	intergenic	*LINC01132,LOC101927851*	.	.	.
1	235086895	G	A	intergenic	*LINC01132,LOC101927851*	.	.	.
1	3998229	C	T	intergenic	*LINC01134,LINC01346*	.	.	.
1	59255235	G	T	ncRNA_intronic	*LINC01135*	.	.	.
1	238828310	AATATAT	A	intergenic	*LINC01139,CHRM3*	.	.	.
1	239169029	C	T	intergenic	*LINC01139,CHRM3*	.	.	.
1	239521162	C	T	intergenic	*LINC01139,CHRM3*	.	.	.
1	239382097	C	T	intergenic	*LINC01139,CHRM3*	.	.	.
1	238760528	TTTTATTTA	T	intergenic	*LINC01139,CHRM3*	.	.	.
1	239051048	TACACAC	T	intergenic	*LINC01139,CHRM3*	.	.	.
1	238889383	T	A	intergenic	*LINC01139,CHRM3*	.	.	.
1	239702942	T	G	intergenic	*LINC01139,CHRM3*	.	.	.
1	239219013	C	T	intergenic	*LINC01139,CHRM3*	.	.	.
1	239016076	T	C	intergenic	*LINC01139,CHRM3*	.	.	.
1	239066082	A	C	intergenic	*LINC01139,CHRM3*	.	.	.

1	239068151	T	C	intergenic	*LINC01139,CHRM3*	.	.	.
1	239069017	AT	A	intergenic	*LINC01139,CHRM3*	.	.	.
1	239073530	T	A	intergenic	*LINC01139,CHRM3*	.	.	.
1	239284626	T	C	intergenic	*LINC01139,CHRM3*	.	.	.
1	239284628	T	C	intergenic	*LINC01139,CHRM3*	.	.	.
1	239666372	C	A	intergenic	*LINC01139,CHRM3*	.	.	.
1	239666373	A	G	intergenic	*LINC01139,CHRM3*	.	.	.
1	170251787	T	C	ncRNA_intronic	*LINC01142*	.	.	.
1	170251788	G	A	ncRNA_intronic	*LINC01142*	.	.	.
1	170322347	C	T	intergenic	*LINC01142,LOC101928650*	.	.	.
1	170292942	A	G	intergenic	*LINC01142,LOC101928650*	.	.	.
1	170340020	C	T	intergenic	*LINC01142,LOC101928650*	.	.	.
1	170370178	G	A	intergenic	*LINC01142,LOC101928650*	.	.	.
14	88588659	A	T	intergenic	*LINC01146,KCNK10*	.	.	.
14	88589909	C	T	intergenic	*LINC01146,KCNK10*	.	.	.
11	1917627	A	AT	upstream	*LINC01150*	.	.	.
2	105542771	C	T	intergenic	*LINC01159,LOC102724691*	.	.	.
2	105542772	T	C	intergenic	*LINC01159,LOC102724691*	.	.	.
7	20944380	C	A	ncRNA_intronic	*LINC01162*	.	.	.
7	20979980	G	A	ncRNA_intronic	*LINC01162*	.	.	.
7	21043284	C	A	ncRNA_intronic	*LINC01162*	.	.	.
7	20924571	C	T	ncRNA_intronic	*LINC01162*	.	.	.
7	20963385	G	A	ncRNA_intronic	*LINC01162*	.	.	.

7	20965645	T	A	ncRNA_intronic	*LINC01162*	.	.	.
7	20965647	C	T	ncRNA_intronic	*LINC01162*	.	.	.
7	21195552	G	C	intergenic	*LINC01162,SP4*	.	.	.
7	21119925	T	C	intergenic	*LINC01162,SP4*	.	.	.
7	21122458	A	C	intergenic	*LINC01162,SP4*	.	.	.
7	21188889	A	T	intergenic	*LINC01162,SP4*	.	.	.
7	21397814	A	G	intergenic	*LINC01162,SP4*	.	.	.
7	21275363	T	C	intergenic	*LINC01162,SP4*	.	.	.
7	21380697	A	G	intergenic	*LINC01162,SP4*	.	.	.
7	21380698	T	G	intergenic	*LINC01162,SP4*	.	.	.
10	130331003	C	T	intergenic	*LINC01163,MGMT*	.	.	.
10	130776099	A	C	intergenic	*LINC01163,MGMT*	.	.	.
10	130278915	C	G	intergenic	*LINC01163,MGMT*	.	.	.
10	130373958	T	TA	intergenic	*LINC01163,MGMT*	.	.	.
10	130947053	A	AT	intergenic	*LINC01163,MGMT*	.	.	.
10	130989360	G	T	intergenic	*LINC01163,MGMT*	.	.	.
10	131047299	C	A	intergenic	*LINC01163,MGMT*	.	.	.
10	130591376	T	C	intergenic	*LINC01163,MGMT*	.	.	.
10	130178845	A	G	intergenic	*LINC01163,MGMT*	.	.	.
10	130777913	G	C	intergenic	*LINC01163,MGMT*	.	.	.
10	130353905	C	G	intergenic	*LINC01163,MGMT*	.	.	.
10	130453592	G	T	intergenic	*LINC01163,MGMT*	.	.	.
10	130732511	A	T	intergenic	*LINC01163,MGMT*	.	.	.
10	130240797	C	T	intergenic	*LINC01163,MGMT*	.	.	.
10	130664172	GACACACA CAC	G	intergenic	*LINC01163,MGMT*	.	.	.
10	133608978	G	A	ncRNA_intronic	*LINC01164*	.	.	.
10	133743200	C	T	intergenic	*LINC01164,PPP2R 2D*	.	.	.
5	123504094	G	A	ncRNA_intronic	*LINC01170*	.	.	.
5	123579948	A	T	ncRNA_intronic	*LINC01170*	.	.	.
5	123751725	A	T	ncRNA_intronic	*LINC01170*	.	.	.
5	123512028	C	CATATATAC/	ncRNA_intronic	*LINC01170*	.	.	.
5	123554078	G	T	ncRNA_intronic	*LINC01170*	.	.	.
5	123698400	A	G	ncRNA_intronic	*LINC01170*	.	.	.
5	123829685	A	G	intergenic	*LINC01170,ZNF608*	.	.	.

2	235686615	A	C	intergenic	*LINC01173,SH3BP4*	.	.	.
2	235688015	G	A	intergenic	*LINC01173,SH3BP4*	.	.	.
2	235688889	G	A	intergenic	*LINC01173,SH3BP4*	.	.	.
7	30457473	C	T	intergenic	*LINC01176,NOD1*	.	.	.
4	166713534	T	G	intergenic	*LINC01179,TLL1*	.	.	.
4	166745116	T	G	intergenic	*LINC01179,TLL1*	.	.	.
4	166745118	T	C	intergenic	*LINC01179,TLL1*	.	.	.
4	13742139	C	T	ncRNA_intronic	*LINC01182*	.	.	.
4	13742141	T	G	ncRNA_intronic	*LINC01182*	.	.	.
4	13743084	T	TGGTGAGAC	ncRNA_intronic	*LINC01182*	.	.	.
4	13817014	G	A	ncRNA_intronic	*LINC01182*	.	.	.
4	13736703	TACACAC	T	ncRNA_intronic	*LINC01182*	.	.	.
4	14038873	T	C	intergenic	*LINC01182,LINC01085*	.	.	.
5	127365603	T	C	ncRNA_intronic	*LINC01184*	.	.	.
X	46200968	G	T	intergenic	*LINC01186,KRBOX4*	.	.	.
X	46272147	G	T	intergenic	*LINC01186,KRBOX4*	.	.	.
5	169641488	G	T	intergenic	*LINC01187,C5orf58*	.	.	.
5	169641611	G	A	intergenic	*LINC01187,C5orf58*	.	.	.
2	114752671	CAGAGAGAGAGAG	C	ncRNA_intronic	*LINC01191*	.	.	.
2	114994024	G	A	intergenic	*LINC01191,DPP10*	.	.	.
2	114988542	G	C	intergenic	*LINC01191,DPP10*	.	.	.
2	115162343	G	A	intergenic	*LINC01191,DPP10*	.	.	.
2	114819028	G	A	intergenic	*LINC01191,DPP10*	.	.	.
2	114855383	G	A	intergenic	*LINC01191,DPP10*	.	.	.
2	114856690	G	A	intergenic	*LINC01191,DPP10*	.	.	.
2	114986389	T	C	intergenic	*LINC01191,DPP10*	.	.	.
2	115091071	C	A	intergenic	*LINC01191,DPP10*	.	.	.
2	115091692	G	T	intergenic	*LINC01191,DPP10*	.	.	.
2	115092090	T	C	intergenic	*LINC01191,DPP10*	.	.	.

2	115114439	G	T	intergenic	*LINC01191,DPP10*	.	.	.
2	115116356	G	A	intergenic	*LINC01191,DPP10*	.	.	.
2	115151187	T	C	intergenic	*LINC01191,DPP10*	.	.	.
2	115151256	G	A	intergenic	*LINC01191,DPP10*	.	.	.
3	162920704	AACAC	A	ncRNA_intronic	*LINC01192*	.	.	.
3	162930120	CTTATTTAT	C	ncRNA_intronic	*LINC01192*	.	.	.
3	163579352	A	C	intergenic	*LINC01192,MIR1263*	.	.	.
3	163809895	C	CAA	intergenic	*LINC01192,MIR1263*	.	.	.
3	163809896	T	TAATTACAAT	intergenic	*LINC01192,MIR1263*	.	.	.
3	163865512	T	C	intergenic	*LINC01192,MIR1263*	.	.	.
3	163345431	T	A	intergenic	*LINC01192,MIR1263*	.	.	.
3	163600929	T	A	intergenic	*LINC01192,MIR1263*	.	.	.
3	163418712	C	T	intergenic	*LINC01192,MIR1263*	.	.	.
15	21189613	A	G	ncRNA_intronic	*LINC01193*	.	.	.
15	21199460	T	C	downstream	*LINC01193*	.	.	.
15	21255228	GATAT	G	intergenic	*LINC01193,LOC646214*	.	.	.
15	21398050	T	C	intergenic	*LINC01193,LOC646214*	.	.	.
15	21315767	G	A	intergenic	*LINC01193,LOC646214*	.	.	.
15	21915818	C	T	intergenic	*LINC01193,LOC646214*	.	.	.
15	21925773	A	AGTGTGTGT	intergenic	*LINC01193,LOC646214*	.	.	.
15	21246125	C	G	intergenic	*LINC01193,LOC646214*	.	.	.
15	21378654	G	GC	intergenic	*LINC01193,LOC646214*	.	.	.
15	21378655	T	A	intergenic	*LINC01193,LOC646214*	.	.	.

5	12754549	C	CA	ncRNA_intronic	*LINC01194*	.	.	.
5	12672231	G	A	ncRNA_intronic	*LINC01194*	.	.	.
5	12622723	C	T	ncRNA_intronic	*LINC01194*	.	.	.
5	12597601	T	C	ncRNA_intronic	*LINC01194*	.	.	.
5	12601657	ACTCATACATCAGTGACCGCTG	A	ncRNA_intronic	*LINC01194*	.	.	.
5	12609016	A	G	ncRNA_intronic	*LINC01194*	.	.	.
5	12678702	G	GGTTTAC	ncRNA_intronic	*LINC01194*	.	.	.
5	13054173	CATAT	C	intergenic	*LINC01194,DNAH5*	.	.	.
5	13629891	G	A	intergenic	*LINC01194,DNAH5*	.	.	.
5	13202175	A	AG	intergenic	*LINC01194,DNAH5*	.	.	.
5	12852479	T	C	intergenic	*LINC01194,DNAH5*	.	.	.
5	12898648	A	T	intergenic	*LINC01194,DNAH5*	.	.	.
5	13096237	A	T	intergenic	*LINC01194,DNAH5*	.	.	.
5	13074776	A	G	intergenic	*LINC01194,DNAH5*	.	.	.
5	13074777	C	G	intergenic	*LINC01194,DNAH5*	.	.	.
5	13125247	A	G	intergenic	*LINC01194,DNAH5*	.	.	.
5	13137268	G	T	intergenic	*LINC01194,DNAH5*	.	.	.
5	13335407	A	G	intergenic	*LINC01194,DNAH5*	.	.	.
5	13670658	C	T	intergenic	*LINC01194,DNAH5*	.	.	.
5	13670659	A	T	intergenic	*LINC01194,DNAH5*	.	.	.
16	9544679	C	T	ncRNA_intronic	*LINC01195*	.	.	.
16	9825435	C	G	intergenic	*LINC01195,GRIN2A*	.	.	.
16	9743036	T	A	intergenic	*LINC01195,GRIN2A*	.	.	.
16	9682410	G	T	intergenic	*LINC01195,GRIN2A*	.	.	.
13	47044249	G	C	intergenic	*LINC01198,LRCH1*	.	.	.
13	47044250	G	A	intergenic	*LINC01198,LRCH1*	.	.	.
13	47081749	G	C	intergenic	*LINC01198,LRCH1*	.	.	.
5	161373348	A	G	ncRNA_intronic	*LINC01202*	.	.	.
5	161353961	C	A	ncRNA_intronic	*LINC01202*	.	.	.
5	161438364	A	C	intergenic	*LINC01202,GABRG2*	.	.	.
X	13424530	C	T	intergenic	*LINC01203,EGFL6*	.	.	.
X	13545439	A	G	intergenic	*LINC01203,EGFL6*	.	.	.

X	45536358	C	G	intergenic	*LINC01204,LOC392452*	.	.	.
X	45451066	G	T	intergenic	*LINC01204,LOC392452*	.	.	.
X	45516969	A	T	intergenic	*LINC01204,LOC392452*	.	.	.
3	109175782	A	T	ncRNA_intronic	*LINC01205*	.	.	.
3	109312501	T	C	intergenic	*LINC01205,MIR4445*	.	.	.
3	109229838	C	G	intergenic	*LINC01205,MIR4445*	.	.	.
3	109259492	T	C	intergenic	*LINC01205,MIR4445*	.	.	.
3	109259815	G	C	intergenic	*LINC01205,MIR4445*	.	.	.
3	109261959	C	A	intergenic	*LINC01205,MIR4445*	.	.	.
3	181676996	GTATATA	G	ncRNA_intronic	*LINC01206*	.	.	.
3	181869871	C	A	intergenic	*LINC01206,FLJ46066*	.	.	.
4	165677447	G	A	ncRNA_intronic	*LINC01207*	.	.	.
4	165783541	T	C	intergenic	*LINC01207,APELA*	.	.	.
3	176404375	T	A	intergenic	*LINC01208,LINC01209*	.	.	.
3	137540958	G	C	intergenic	*LINC01210,CLDN18*	.	.	.
3	137608656	T	A	intergenic	*LINC01210,CLDN18*	.	.	.
3	137700019	C	G	intergenic	*LINC01210,CLDN18*	.	.	.
3	70056990	A	G	ncRNA_intronic	*LINC01212*	.	.	.
3	70120263	GA	G	intergenic	*LINC01212,FOXP1*	.	.	.
3	70369561	A	G	intergenic	*LINC01212,FOXP1*	.	.	.
3	70708500	A	G	intergenic	*LINC01212,FOXP1*	.	.	.
3	70204914	G	T	intergenic	*LINC01212,FOXP1*	.	.	.
3	70653175	C	T	intergenic	*LINC01212,FOXP1*	.	.	.
3	70388508	A	T	intergenic	*LINC01212,FOXP1*	.	.	.
3	70503464	T	C	intergenic	*LINC01212,FOXP1*	.	.	.

3	70736637	G	T	intergenic	*LINC01212,FOXP1*	.	.	.
3	70942499	G	T	intergenic	*LINC01212,FOXP1*	.	.	.
3	70944248	A	G	intergenic	*LINC01212,FOXP1*	.	.	.
4	101589936	T	C	ncRNA_intronic	*LINC01216*	.	.	.
4	101589937	G	A	ncRNA_intronic	*LINC01216*	.	.	.
4	101590671	T	C	ncRNA_intronic	*LINC01216*	.	.	.
4	101686650	C	T	intergenic	*LINC01216,PPP3CA*	.	.	.
4	101787016	C	T	intergenic	*LINC01216,PPP3CA*	.	.	.
1	199033999	T	A	ncRNA_intronic	*LINC01221*	.	.	.
1	199325017	G	T	intergenic	*LINC01221,NR5A2*	.	.	.
1	199870626	G	T	intergenic	*LINC01221,NR5A2*	.	.	.
1	199383704	G	T	intergenic	*LINC01221,NR5A2*	.	.	.
1	199482986	T	A	intergenic	*LINC01221,NR5A2*	.	.	.
1	199104713	T	TTGG	intergenic	*LINC01221,NR5A2*	.	.	.
1	199438715	G	T	intergenic	*LINC01221,NR5A2*	.	.	.
1	199333940	A	C	intergenic	*LINC01221,NR5A2*	.	.	.
1	199335841	T	C	intergenic	*LINC01221,NR5A2*	.	.	.
1	199411924	A	G	intergenic	*LINC01221,NR5A2*	.	.	.
1	199573035	T	C	intergenic	*LINC01221,NR5A2*	.	.	.
1	199573569	T	G	intergenic	*LINC01221,NR5A2*	.	.	.
1	199573941	G	A	intergenic	*LINC01221,NR5A2*	.	.	.
1	199575412	T	C	intergenic	*LINC01221,NR5A2*	.	.	.
1	199575414	C	A	intergenic	*LINC01221,NR5A2*	.	.	.
1	199619539	G	A	intergenic	*LINC01221,NR5A2*	.	.	.
1	199621915	T	C	intergenic	*LINC01221,NR5A2*	.	.	.
1	199682155	C	T	intergenic	*LINC01221,NR5A2*	.	.	.
1	199854437	C	T	intergenic	*LINC01221,NR5A2*	.	.	.
1	199890431	T	TGTC	intergenic	*LINC01221,NR5A2*	.	.	.
1	199982068	T	A	intergenic	*LINC01221,NR5A2*	.	.	.
19	23591467	AT	A	ncRNA_intronic	*LINC01224*	.	.	.
19	23599315	G	A	upstream	*LINC01224*	.	.	.
19	23702459	C	A	intergenic	*LINC01224,ZNF675*	.	.	.
19	23637663	C	T	intergenic	*LINC01224,ZNF675*	.	.	.

19	23639195	G	A	intergenic	*LINC01224,ZNF675*	.	.	.
19	23639196	T	C	intergenic	*LINC01224,ZNF675*	.	.	.
1	32003373	A	C	intergenic	*LINC01226,TINAGL1*	.	.	.
1	32003374	C	G	intergenic	*LINC01226,TINAGL1*	.	.	.
16	79859597	A	T	intergenic	*LINC01229,LOC102724084*	.	.	.
16	80040497	C	T	intergenic	*LINC01229,LOC102724084*	.	.	.
16	80172555	T	C	intergenic	*LINC01229,LOC102724084*	.	.	.
9	22685256	T	TTCTC	ncRNA_intronic	*LINC01239*	.	.	.
9	22738480	G	A	ncRNA_intronic	*LINC01239*	.	.	.
9	22886595	T	C	intergenic	*LINC01239,LOC101929563*	.	.	.
9	22962236	C	T	intergenic	*LINC01239,LOC101929563*	.	.	.
9	23068525	A	T	intergenic	*LINC01239,LOC101929563*	.	.	.
9	23359774	C	T	intergenic	*LINC01239,LOC101929563*	.	.	.
9	25855747	A	C	intergenic	*LINC01241,LOC100506422*	.	.	.
9	25930474	C	G	intergenic	*LINC01241,LOC100506422*	.	.	.
9	25946650	G	A	intergenic	*LINC01241,LOC100506422*	.	.	.
9	25986976	T	C	intergenic	*LINC01241,LOC100506422*	.	.	.
9	26044934	C	T	intergenic	*LINC01241,LOC100506422*	.	.	.
9	26048368	T	C	intergenic	*LINC01241,LOC100506422*	.	.	.
9	30953787	G	A	intergenic	*LINC01242,ACO1*	.	.	.
9	31515360	T	C	intergenic	*LINC01242,ACO1*	.	.	.

9	30533442	T	C	intergenic	*LINC01242,ACO1*	.	.	.
9	31224284	G	T	intergenic	*LINC01242,ACO1*	.	.	.
9	31932531	T	TG	intergenic	*LINC01242,ACO1*	.	.	.
9	32009194	G	A	intergenic	*LINC01242,ACO1*	.	.	.
9	32115004	C	G	intergenic	*LINC01242,ACO1*	.	.	.
9	32341345	T	C	intergenic	*LINC01242,ACO1*	.	.	.
9	30740935	C	CAT	intergenic	*LINC01242,ACO1*	.	.	.
9	30893406	TC	T	intergenic	*LINC01242,ACO1*	.	.	.
9	31600468	T	C	intergenic	*LINC01242,ACO1*	.	.	.
9	30924224	T	C	intergenic	*LINC01242,ACO1*	.	.	.
9	32369067	CAAACAAACA	C	intergenic	*LINC01242,ACO1*	.	.	.
9	30665369	A	G	intergenic	*LINC01242,ACO1*	.	.	.
9	31008854	A	ATG	intergenic	*LINC01242,ACO1*	.	.	.
9	31539134	C	T	intergenic	*LINC01242,ACO1*	.	.	.
9	31920159	C	T	intergenic	*LINC01242,ACO1*	.	.	.
9	32260539	CCCCCCG	C	intergenic	*LINC01242,ACO1*	.	.	.
9	30540562	G	A	intergenic	*LINC01242,ACO1*	.	.	.
9	30585000	T	A	intergenic	*LINC01242,ACO1*	.	.	.
9	30952297	A	T	intergenic	*LINC01242,ACO1*	.	.	.
9	31057654	A	G	intergenic	*LINC01242,ACO1*	.	.	.
9	31060087	A	G	intergenic	*LINC01242,ACO1*	.	.	.
9	31157730	T	C	intergenic	*LINC01242,ACO1*	.	.	.
9	31157731	A	G	intergenic	*LINC01242,ACO1*	.	.	.
9	31158408	T	A	intergenic	*LINC01242,ACO1*	.	.	.
9	31158409	G	A	intergenic	*LINC01242,ACO1*	.	.	.
9	31181175	A	G	intergenic	*LINC01242,ACO1*	.	.	.
9	31225609	T	A	intergenic	*LINC01242,ACO1*	.	.	.
9	31300049	A	T	intergenic	*LINC01242,ACO1*	.	.	.
9	31685323	A	G	intergenic	*LINC01242,ACO1*	.	.	.
9	31934054	C	G	intergenic	*LINC01242,ACO1*	.	.	.
9	31993035	G	C	intergenic	*LINC01242,ACO1*	.	.	.
9	32023954	A	G	intergenic	*LINC01242,ACO1*	.	.	.
9	32023955	A	G	intergenic	*LINC01242,ACO1*	.	.	.
9	32050919	T	G	intergenic	*LINC01242,ACO1*	.	.	.
9	32101860	C	T	intergenic	*LINC01242,ACO1*	.	.	.
9	32123400	A	T	intergenic	*LINC01242,ACO1*	.	.	.
9	32152259	C	G	intergenic	*LINC01242,ACO1*	.	.	.

9	32152260	C	A	intergenic	*LINC01242,ACO1*	.	.	.
9	32215369	G	A	intergenic	*LINC01242,ACO1*	.	.	.
2	6508503	T	G	ncRNA_intronic	*LINC01247*	.	.	.
2	6716346	A	G	intergenic	*LINC01247,LINC01246*	.	.	.
2	6696617	A	G	intergenic	*LINC01247,LINC01246*	.	.	.
2	6751441	C	T	intergenic	*LINC01247,LINC01246*	.	.	.
2	5793172	G	T	ncRNA_intronic	*LINC01248*	.	.	.
2	5813096	G	A	ncRNA_intronic	*LINC01248*	.	.	.
2	5808066	GGA	G	ncRNA_intronic	*LINC01248*	.	.	.
2	4693763	A	C	ncRNA_intronic	*LINC01249*	.	.	.
2	5679240	G	T	intergenic	*LINC01249,LINC01248*	.	.	.
2	5462344	TTGTGTG	T	intergenic	*LINC01249,LINC01248*	.	.	.
2	4824373	TATA	T	intergenic	*LINC01249,LINC01248*	.	.	.
2	5719611	A	AT	intergenic	*LINC01249,LINC01248*	.	.	.
2	4710680	C	A	intergenic	*LINC01249,LINC01248*	.	.	.
2	4948412	T	G	intergenic	*LINC01249,LINC01248*	.	.	.
2	5282921	C	T	intergenic	*LINC01249,LINC01248*	.	.	.
2	5306926	T	C	intergenic	*LINC01249,LINC01248*	.	.	.
2	5306927	G	A	intergenic	*LINC01249,LINC01248*	.	.	.
2	5400198	A	C	intergenic	*LINC01249,LINC01248*	.	.	.
2	5401034	C	A	intergenic	*LINC01249,LINC01248*	.	.	.
2	5401394	T	TC	intergenic	*LINC01249,LINC01248*	.	.	.

2	5402259	T	C	intergenic	*LINC01249,LINC01248*	.	.	.
2	3089189	G	C	ncRNA_intronic	*LINC01250*	.	.	.
2	2900273	C	A	ncRNA_exonic	*LINC01250*	.	.	.
2	2986224	G	A	ncRNA_intronic	*LINC01250*	.	.	.
2	2992549	T	C	ncRNA_intronic	*LINC01250*	.	.	.
18	11597192	T	A	intergenic	*LINC01255,SLC35G4*	.	.	.
4	133537347	T	C	ncRNA_intronic	*LINC01256*	.	.	.
4	133550148	A	G	ncRNA_intronic	*LINC01256*	.	.	.
4	133799408	T	C	intergenic	*LINC01256,LOC101927359*	.	.	.
4	133931337	TTGTG	T	intergenic	*LINC01256,LOC101927359*	.	.	.
4	133975601	T	G	intergenic	*LINC01256,LOC101927359*	.	.	.
12	131797651	C	G	intergenic	*LINC01257,LOC338797*	.	.	.
12	131774682	G	A	intergenic	*LINC01257,LOC338797*	.	.	.
4	38437138	A	G	ncRNA_intronic	*LINC01258*	.	.	.
4	38480932	A	G	ncRNA_intronic	*LINC01258*	.	.	.
4	38596797	C	A	intergenic	*LINC01258,KLF3-AS1*	.	.	.
4	38575125	C	T	intergenic	*LINC01258,KLF3-AS1*	.	.	.
4	190588716	G	GTGT	intergenic	*LINC01262,LINC01596*	.	.	.
4	190797755	G	A	intergenic	*LINC01262,LINC01596*	.	.	.
4	190669303	A	G	intergenic	*LINC01262,LINC01596*	.	.	.
3	732717	C	G	ncRNA_intronic	*LINC01266*	.	.	.
3	652154	C	A	ncRNA_intronic	*LINC01266*	.	.	.
3	878372	G	A	ncRNA_intronic	*LINC01266*	.	.	.
3	736590	C	G	ncRNA_intronic	*LINC01266*	.	.	.
3	1028116	G	C	intergenic	*LINC01266,CNTN6*	.	.	.
3	1058970	A	ATT	intergenic	*LINC01266,CNTN6*	.	.	.

3	1074381	G	C	intergenic	*LINC01266,CNTN6*	.	.	.
3	1099922	G	A	intergenic	*LINC01266,CNTN6*	.	.	.
20	48975839	G	A	intergenic	*LINC01271,PTPN1*	.	.	.
20	48906661	G	A	intergenic	*LINC01272,LINC01270*	.	.	.
6	143313806	C	CAGAG	ncRNA_intronic	*LINC01277*	.	.	.
6	143314696	T	C	ncRNA_intronic	*LINC01277*	.	.	.
6	143318337	G	A	ncRNA_intronic	*LINC01277*	.	.	.
6	143351476	T	C	ncRNA_intronic	*LINC01277*	.	.	.
6	143351656	C	T	ncRNA_intronic	*LINC01277*	.	.	.
6	143352916	G	A	ncRNA_intronic	*LINC01277*	.	.	.
6	143353227	A	G	ncRNA_intronic	*LINC01277*	.	.	.
X	62705814	C	T	ncRNA_intronic	*LINC01278*	.	.	.
X	62667311	C	T	ncRNA_intronic	*LINC01278*	.	.	.
X	62668762	C	G	ncRNA_intronic	*LINC01278*	.	.	.
X	62683229	C	T	ncRNA_intronic	*LINC01278*	.	.	.
X	62700074	T	C	ncRNA_intronic	*LINC01278*	.	.	.
X	62700075	G	C	ncRNA_intronic	*LINC01278*	.	.	.
X	62702488	A	C	ncRNA_intronic	*LINC01278*	.	.	.
X	62741078	T	G	ncRNA_intronic	*LINC01278*	.	.	.
X	62741738	G	T	ncRNA_intronic	*LINC01278*	.	.	.
X	62759726	A	G	ncRNA_intronic	*LINC01278*	.	.	.
X	62769855	T	A	ncRNA_intronic	*LINC01278*	.	.	.
X	62769857	A	G	ncRNA_intronic	*LINC01278*	.	.	.
X	62822831	C	T	intergenic	*LINC01278,ARHGEF9*	.	.	.
X	62822832	G	T	intergenic	*LINC01278,ARHGEF9*	.	.	.
X	62784002	T	C	intergenic	*LINC01278,ARHGEF9*	.	.	.
X	62831435	C	T	intergenic	*LINC01278,ARHGEF9*	.	.	.
X	62836564	T	C	intergenic	*LINC01278,ARHGEF9*	.	.	.
X	39193450	A	ATTGTGT	intergenic	*LINC01281,LINC01282*	.	.	.
X	39273269	C	G	intergenic	*LINC01282,MIR3937*	.	.	.

X	39403919	T	C	intergenic	*LINC01282,MIR3937*	.	.	.
X	39404912	C	A	intergenic	*LINC01282,MIR3937*	.	.	.
X	50875192	T	C	ncRNA_intronic	*LINC01284*	.	.	.
X	51014440	G	A	intergenic	*LINC01284,NUDT10*	.	.	.
X	50957841	A	AT	intergenic	*LINC01284,NUDT10*	.	.	.
X	50991221	G	C	intergenic	*LINC01284,NUDT10*	.	.	.
X	50991401	A	C	intergenic	*LINC01284,NUDT10*	.	.	.
X	50995924	T	C	intergenic	*LINC01284,NUDT10*	.	.	.
X	51008190	A	G	intergenic	*LINC01284,NUDT10*	.	.	.
X	51033376	G	A	intergenic	*LINC01284,NUDT10*	.	.	.
X	118095905	G	A	intergenic	*LINC01285,LONRF3*	.	.	.
X	118044264	G	A	intergenic	*LINC01285,LONRF3*	.	.	.
7	153273124	C	CAAAAAAAA	intergenic	*LINC01287,DPP6*	.	.	.
7	153235640	T	A	intergenic	*LINC01287,DPP6*	.	.	.
7	153132322	T	A	intergenic	*LINC01287,DPP6*	.	.	.
7	153524364	G	T	intergenic	*LINC01287,DPP6*	.	.	.
7	153209563	G	A	intergenic	*LINC01287,DPP6*	.	.	.
7	153415566	G	A	intergenic	*LINC01287,DPP6*	.	.	.
7	153441307	G	A	intergenic	*LINC01287,DPP6*	.	.	.
7	153441308	C	A	intergenic	*LINC01287,DPP6*	.	.	.
7	153494345	G	T	intergenic	*LINC01287,DPP6*	.	.	.
7	153566550	A	G	intergenic	*LINC01287,DPP6*	.	.	.
8	34657531	CA	C	ncRNA_intronic	*LINC01288*	.	.	.
8	34674576	C	A	ncRNA_intronic	*LINC01288*	.	.	.
8	34656510	T	C	ncRNA_intronic	*LINC01288*	.	.	.
8	34936525	C	T	intergenic	*LINC01288,UNC5D*	.	.	.
8	34942593	C	A	intergenic	*LINC01288,UNC5D*	.	.	.

8	34889726	T	TA	intergenic	*LINC01288,UNC5D*	.	.	.
8	34848852	G	A	intergenic	*LINC01288,UNC5D*	.	.	.
8	34954225	T	C	intergenic	*LINC01288,UNC5D*	.	.	.
8	35085678	T	C	intergenic	*LINC01288,UNC5D*	.	.	.
8	35086276	C	T	intergenic	*LINC01288,UNC5D*	.	.	.
8	35086277	A	G	intergenic	*LINC01288,UNC5D*	.	.	.
8	64731116	T	G	intergenic	*LINC01289,LOC102724623*	.	.	.
8	142357908	ACACAGACACACACACACTCAAATACACAGACTCACATACT	A	intergenic	*LINC01300,GPR20*	.	.	.
8	61383511	A	G	intergenic	*LINC01301,RAB2A*	.	.	.
8	61354211	G	T	intergenic	*LINC01301,RAB2A*	.	.	.
8	61355141	A	G	intergenic	*LINC01301,RAB2A*	.	.	.
8	61355269	C	G	intergenic	*LINC01301,RAB2A*	.	.	.
8	61364074	C	A	intergenic	*LINC01301,RAB2A*	.	.	.
8	61365193	C	T	intergenic	*LINC01301,RAB2A*	.	.	.
8	61366034	A	T	intergenic	*LINC01301,RAB2A*	.	.	.
2	4387784	C	A	intergenic	*LINC01304,LINC01249*	.	.	.
2	4517885	A	G	intergenic	*LINC01304,LINC01249*	.	.	.
2	4075796	A	G	intergenic	*LINC01304,LINC01249*	.	.	.
2	4516200	T	C	intergenic	*LINC01304,LINC01249*	.	.	.
2	4519961	C	A	intergenic	*LINC01304,LINC01249*	.	.	.
2	4126829	A	G	intergenic	*LINC01304,LINC01249*	.	.	.
2	4127972	T	C	intergenic	*LINC01304,LINC01249*	.	.	.
2	4127973	G	A	intergenic	*LINC01304,LINC01249*	.	.	.

2	4129627	A	G	intergenic	*LINC01304,LINC01249*	.	.	.
2	4165026	T	A	intergenic	*LINC01304,LINC01249*	.	.	.
2	4165865	T	C	intergenic	*LINC01304,LINC01249*	.	.	.
2	4165866	G	A	intergenic	*LINC01304,LINC01249*	.	.	.
2	4243596	A	AAATAATTT	intergenic	*LINC01304,LINC01249*	.	.	.
2	4358990	T	C	intergenic	*LINC01304,LINC01249*	.	.	.
2	4622204	T	C	intergenic	*LINC01304,LINC01249*	.	.	.
2	4663831	C	G	intergenic	*LINC01304,LINC01249*	.	.	.
1	102231004	A	G	intergenic	*LINC01307,OLFM3*	.	.	.
1	102231005	T	G	intergenic	*LINC01307,OLFM3*	.	.	.
13	104153253	C	T	intergenic	*LINC01309,DAOA-AS1*	.	.	.
13	104819586	C	A	intergenic	*LINC01309,DAOA-AS1*	.	.	.
13	105663669	G	A	intergenic	*LINC01309,DAOA-AS1*	.	.	.
13	106062719	G	A	intergenic	*LINC01309,DAOA-AS1*	.	.	.
13	105756508	A	G	intergenic	*LINC01309,DAOA-AS1*	.	.	.
13	105038283	T	A	intergenic	*LINC01309,DAOA-AS1*	.	.	.
13	104712865	T	C	intergenic	*LINC01309,DAOA-AS1*	.	.	.
13	104867646	C	T	intergenic	*LINC01309,DAOA-AS1*	.	.	.
13	105015898	C	A	intergenic	*LINC01309,DAOA-AS1*	.	.	.
13	105049028	C	A	intergenic	*LINC01309,DAOA-AS1*	.	.	.

13	105305497	G	C	intergenic	*LINC01309,DAOA-AS1*	.	.	.
13	105662809	T	C	intergenic	*LINC01309,DAOA-AS1*	.	.	.
13	105978230	G	A	intergenic	*LINC01309,DAOA-AS1*	.	.	.
13	104381410	G	A	intergenic	*LINC01309,DAOA-AS1*	.	.	.
13	104158066	T	C	intergenic	*LINC01309,DAOA-AS1*	.	.	.
13	104400896	G	C	intergenic	*LINC01309,DAOA-AS1*	.	.	.
13	104477654	T	C	intergenic	*LINC01309,DAOA-AS1*	.	.	.
13	104477914	G	A	intergenic	*LINC01309,DAOA-AS1*	.	.	.
13	104478279	C	T	intergenic	*LINC01309,DAOA-AS1*	.	.	.
13	104478362	C	T	intergenic	*LINC01309,DAOA-AS1*	.	.	.
13	104733892	A	G	intergenic	*LINC01309,DAOA-AS1*	.	.	.
13	104735193	C	A	intergenic	*LINC01309,DAOA-AS1*	.	.	.
13	104836660	T	C	intergenic	*LINC01309,DAOA-AS1*	.	.	.
13	104837548	G	A	intergenic	*LINC01309,DAOA-AS1*	.	.	.
13	104982895	T	C	intergenic	*LINC01309,DAOA-AS1*	.	.	.
13	104984012	T	C	intergenic	*LINC01309,DAOA-AS1*	.	.	.
13	104985031	T	G	intergenic	*LINC01309,DAOA-AS1*	.	.	.
13	104996391	G	T	intergenic	*LINC01309,DAOA-AS1*	.	.	.
13	105091570	A	C	intergenic	*LINC01309,DAOA-AS1*	.	.	.

13	105097492	T	A	intergenic	*LINC01309,DAOA-AS1*	.	.	.
13	105097494	T	C	intergenic	*LINC01309,DAOA-AS1*	.	.	.
13	105146573	A	C	intergenic	*LINC01309,DAOA-AS1*	.	.	.
13	105146575	A	G	intergenic	*LINC01309,DAOA-AS1*	.	.	.
13	105586474	T	A	intergenic	*LINC01309,DAOA-AS1*	.	.	.
13	105609610	C	T	intergenic	*LINC01309,DAOA-AS1*	.	.	.
13	105611098	T	A	intergenic	*LINC01309,DAOA-AS1*	.	.	.
13	105803129	G	A	intergenic	*LINC01309,DAOA-AS1*	.	.	.
13	105803839	GT	G	intergenic	*LINC01309,DAOA-AS1*	.	.	.
13	106071744	A	G	intergenic	*LINC01309,DAOA-AS1*	.	.	.
13	106071745	C	T	intergenic	*LINC01309,DAOA-AS1*	.	.	.
22	49263070	C	T	ncRNA_intronic	*LINC01310*	.	.	.
22	49505986	C	A	intergenic	*LINC01310,NONE*	.	.	.
22	49729909	C	T	intergenic	*LINC01310,NONE*	.	.	.
6	134147717	T	C	ncRNA_intronic	*LINC01312,TARID*	.	.	.
2	33995115	T	G	ncRNA_intronic	*LINC01317*	.	.	.
2	34003019	G	T	ncRNA_intronic	*LINC01317*	.	.	.
2	34409736	A	T	ncRNA_intronic	*LINC01317*	.	.	.
2	34039335	C	G	ncRNA_intronic	*LINC01317*	.	.	.
2	34408173	A	G	ncRNA_intronic	*LINC01317*	.	.	.
2	34408174	T	C	ncRNA_intronic	*LINC01317*	.	.	.
2	34434261	T	A	ncRNA_intronic	*LINC01317*	.	.	.
2	34434263	GC	G	ncRNA_intronic	*LINC01317*	.	.	.
2	34657780	C	G	intergenic	*LINC01317,LINC01320*	.	.	.
2	34870678	T	C	intergenic	*LINC01317,LINC01320*	.	.	.

2	34578443	C	G	intergenic	*LINC01317,LINC01320*	.	.	.
2	34541501	C	A	intergenic	*LINC01317,LINC01320*	.	.	.
2	34787667	A	G	intergenic	*LINC01317,LINC01320*	.	.	.
2	34693980	A	G	intergenic	*LINC01317,LINC01320*	.	.	.
2	34780389	G	A	intergenic	*LINC01317,LINC01320*	.	.	.
2	34784477	T	C	intergenic	*LINC01317,LINC01320*	.	.	.
2	34826304	T	C	intergenic	*LINC01317,LINC01320*	.	.	.
2	34826305	G	A	intergenic	*LINC01317,LINC01320*	.	.	.
2	34864959	C	A	intergenic	*LINC01317,LINC01320*	.	.	.
2	34879288	C	T	intergenic	*LINC01317,LINC01320*	.	.	.
2	34879608	T	G	intergenic	*LINC01317,LINC01320*	.	.	.
2	35207536	T	C	intergenic	*LINC01320,LOC100288911*	.	.	.
2	35131315	T	C	intergenic	*LINC01320,LOC100288911*	.	.	.
2	35972667	A	G	intergenic	*LINC01320,LOC100288911*	.	.	.
2	35223604	G	T	intergenic	*LINC01320,LOC100288911*	.	.	.
2	35885118	C	A	intergenic	*LINC01320,LOC100288911*	.	.	.
2	34985962	T	C	intergenic	*LINC01320,LOC100288911*	.	.	.
2	35231241	A	T	intergenic	*LINC01320,LOC100288911*	.	.	.
2	35667375	G	T	intergenic	*LINC01320,LOC100288911*	.	.	.

2	35670804	T	A	intergenic	*LINC01320,LOC100288911*	.	.	.
2	36036609	C	A	intergenic	*LINC01320,LOC100288911*	.	.	.
2	35431995	C	G	intergenic	*LINC01320,LOC100288911*	.	.	.
2	34989697	A	T	intergenic	*LINC01320,LOC100288911*	.	.	.
2	34989699	C	T	intergenic	*LINC01320,LOC100288911*	.	.	.
2	35160574	C	T	intergenic	*LINC01320,LOC100288911*	.	.	.
2	35242073	T	C	intergenic	*LINC01320,LOC100288911*	.	.	.
2	35242146	C	A	intergenic	*LINC01320,LOC100288911*	.	.	.
2	35332518	C	T	intergenic	*LINC01320,LOC100288911*	.	.	.
2	35335267	T	C	intergenic	*LINC01320,LOC100288911*	.	.	.
2	35353111	C	T	intergenic	*LINC01320,LOC100288911*	.	.	.
2	35503874	T	TG	intergenic	*LINC01320,LOC100288911*	.	.	.
2	35546550	C	T	intergenic	*LINC01320,LOC100288911*	.	.	.
2	35639112	A	G	intergenic	*LINC01320,LOC100288911*	.	.	.
2	35642966	A	AAATCTCTG	intergenic	*LINC01320,LOC100288911*	.	.	.
2	35706077	T	C	intergenic	*LINC01320,LOC100288911*	.	.	.
2	35707689	A	G	intergenic	*LINC01320,LOC100288911*	.	.	.
2	35707751	T	G	intergenic	*LINC01320,LOC100288911*	.	.	.
2	35737796	C	T	intergenic	*LINC01320,LOC100288911*	.	.	.

2	35758102	G	C	intergenic	*LINC01320,LOC100288911*	.	.	.
2	35790943	C	T	intergenic	*LINC01320,LOC100288911*	.	.	.
2	35995483	G	A	intergenic	*LINC01320,LOC100288911*	.	.	.
2	36025333	GGT	G	intergenic	*LINC01320,LOC100288911*	.	.	.
2	36074242	A	T	intergenic	*LINC01320,LOC100288911*	.	.	.
2	36074720	C	G	intergenic	*LINC01320,LOC100288911*	.	.	.
2	36128373	C	T	intergenic	*LINC01320,LOC100288911*	.	.	.
2	36290821	G	A	intergenic	*LINC01320,LOC100288911*	.	.	.
2	36327433	G	T	intergenic	*LINC01320,LOC100288911*	.	.	.
2	36334861	G	A	intergenic	*LINC01320,LOC100288911*	.	.	.
2	36343275	A	T	intergenic	*LINC01320,LOC100288911*	.	.	.
2	36443801	A	G	intergenic	*LINC01320,LOC100288911*	.	.	.
2	36445219	A	G	intergenic	*LINC01320,LOC100288911*	.	.	.
3	164935919	T	A	ncRNA_intronic	*LINC01322*	.	.	.
3	165044930	A	G	ncRNA_intronic	*LINC01322*	.	.	.
3	165274860	A	T	intergenic	*LINC01322,BCHE*	.	.	.
3	164441465	C	G	ncRNA_intronic	*LINC01324*	.	.	.
5	73733321	C	A	ncRNA_intronic	*LINC01331*	.	.	.
5	73733323	A	G	ncRNA_intronic	*LINC01331*	.	.	.
5	73614675	C	T	intergenic	*LINC01335,LINC01333*	.	.	.
5	82211101	C	T	intergenic	*LINC01338,TMEM167A*	.	.	.
5	82230493	A	T	intergenic	*LINC01338,TMEM167A*	.	.	.

5	89474642	T	C	ncRNA_intronic	*LINC01339*	.	.	.
5	89474713	G	A	ncRNA_intronic	*LINC01339*	.	.	.
5	89507336	A	G	ncRNA_intronic	*LINC01339*	.	.	.
5	89535910	G	A	ncRNA_intronic	*LINC01339*	.	.	.
5	89538933	G	A	ncRNA_intronic	*LINC01339*	.	.	.
5	89653381	C	CATAA	intergenic	*LINC01339,CETN3*	.	.	.
5	96852351	A	G	ncRNA_intronic	*LINC01340*	.	.	.
5	97332534	C	CACT	intergenic	*LINC01340,RGMB*	.	.	.
5	97547917	TTCTCTC	T	intergenic	*LINC01340,RGMB*	.	.	.
5	97293140	G	T	intergenic	*LINC01340,RGMB*	.	.	.
5	97786676	A	G	intergenic	*LINC01340,RGMB*	.	.	.
5	97944838	C	A	intergenic	*LINC01340,RGMB*	.	.	.
5	97833768	A	C	intergenic	*LINC01340,RGMB*	.	.	.
5	97018769	T	A	intergenic	*LINC01340,RGMB*	.	.	.
5	97018829	T	C	intergenic	*LINC01340,RGMB*	.	.	.
5	97054590	T	G	intergenic	*LINC01340,RGMB*	.	.	.
5	97112223	A	G	intergenic	*LINC01340,RGMB*	.	.	.
5	97146614	G	T	intergenic	*LINC01340,RGMB*	.	.	.
5	97227445	A	G	intergenic	*LINC01340,RGMB*	.	.	.
5	97227446	T	A	intergenic	*LINC01340,RGMB*	.	.	.
5	97235245	C	T	intergenic	*LINC01340,RGMB*	.	.	.
5	97235246	A	G	intergenic	*LINC01340,RGMB*	.	.	.
5	97236192	C	T	intergenic	*LINC01340,RGMB*	.	.	.
5	97262810	G	A	intergenic	*LINC01340,RGMB*	.	.	.
5	97263744	C	T	intergenic	*LINC01340,RGMB*	.	.	.
5	97331457	C	T	intergenic	*LINC01340,RGMB*	.	.	.
5	97360638	G	A	intergenic	*LINC01340,RGMB*	.	.	.
5	97361524	G	A	intergenic	*LINC01340,RGMB*	.	.	.
5	97459515	C	G	intergenic	*LINC01340,RGMB*	.	.	.
5	97473261	C	G	intergenic	*LINC01340,RGMB*	.	.	.
5	97473685	C	A	intergenic	*LINC01340,RGMB*	.	.	.
5	97473687	G	A	intergenic	*LINC01340,RGMB*	.	.	.
5	97572251	C	T	intergenic	*LINC01340,RGMB*	.	.	.
5	97572253	C	G	intergenic	*LINC01340,RGMB*	.	.	.
5	97574843	A	G	intergenic	*LINC01340,RGMB*	.	.	.
5	97575128	A	G	intergenic	*LINC01340,RGMB*	.	.	.
5	97601819	C	T	intergenic	*LINC01340,RGMB*	.	.	.
5	97667455	T	C	intergenic	*LINC01340,RGMB*	.	.	.

5	97668160	A	G	intergenic	*LINC01340,RGMB*	.	.	.
5	97681857	C	T	intergenic	*LINC01340,RGMB*	.	.	.
5	97683419	A	G	intergenic	*LINC01340,RGMB*	.	.	.
5	97869413	T	G	intergenic	*LINC01340,RGMB*	.	.	.
5	97869679	GT	G	intergenic	*LINC01340,RGMB*	.	.	.
5	97878286	T	C	intergenic	*LINC01340,RGMB*	.	.	.
5	97926285	T	C	intergenic	*LINC01340,RGMB*	.	.	.
5	97926286	G	A	intergenic	*LINC01340,RGMB*	.	.	.
5	97930981	T	C	intergenic	*LINC01340,RGMB*	.	.	.
5	98020657	T	C	intergenic	*LINC01340,RGMB*	.	.	.
5	98020658	G	T	intergenic	*LINC01340,RGMB*	.	.	.
5	98021217	G	GC	intergenic	*LINC01340,RGMB*	.	.	.
5	98040278	G	A	intergenic	*LINC01340,RGMB*	.	.	.
5	98054754	G	T	intergenic	*LINC01340,RGMB*	.	.	.
5	98074630	T	C	intergenic	*LINC01340,RGMB*	.	.	.
5	98082099	C	T	intergenic	*LINC01340,RGMB*	.	.	.
1	39012163	AACACAC	A	intergenic	*LINC01343,RRAGC*	.	.	.
1	39210551	C	T	intergenic	*LINC01343,RRAGC*	.	.	.
1	39097052	A	G	intergenic	*LINC01343,RRAGC*	.	.	.
1	39098197	C	T	intergenic	*LINC01343,RRAGC*	.	.	.
1	39113525	G	A	intergenic	*LINC01343,RRAGC*	.	.	.
1	182292811	G	A	intergenic	*LINC01344,GLUL*	.	.	.
1	4072572	G	A	intergenic	*LINC01346,LOC284661*	.	.	.
1	4163635	A	G	intergenic	*LINC01346,LOC284661*	.	.	.
1	4306836	G	A	intergenic	*LINC01346,LOC284661*	.	.	.
1	243247583	A	G	ncRNA_intronic	*LINC01347*	.	.	.
1	243259872	G	C	ncRNA_intronic	*LINC01347*	.	.	.
1	101101598	A	T	ncRNA_intronic	*LINC01349*	.	.	.
1	101149150	T	C	intergenic	*LINC01349,VCAM1*	.	.	.

1	101130069	A	G	intergenic	*LINC01349,VCAM1*	.	.	.
1	101131576	G	C	intergenic	*LINC01349,VCAM1*	.	.	.
1	101143349	C	T	intergenic	*LINC01349,VCAM1*	.	.	.
1	185545176	G	T	ncRNA_intronic	*LINC01350*	.	.	.
1	185583360	T	C	ncRNA_intronic	*LINC01350*	.	.	.
1	185544790	T	C	ncRNA_intronic	*LINC01350*	.	.	.
1	190459573	C	G	intergenic	*LINC01351,LOC440704*	.	.	.
1	73796476	A	C	ncRNA_intronic	*LINC01360*	.	.	.
1	74371815	A	G	intergenic	*LINC01360,LRRIQ3*	.	.	.
1	73865595	G	A	intergenic	*LINC01360,LRRIQ3*	.	.	.
1	74044831	A	G	intergenic	*LINC01360,LRRIQ3*	.	.	.
1	73894646	G	T	intergenic	*LINC01360,LRRIQ3*	.	.	.
1	74152468	G	T	intergenic	*LINC01360,LRRIQ3*	.	.	.
1	74276674	T	C	intergenic	*LINC01360,LRRIQ3*	.	.	.
1	83940884	A	T	intergenic	*LINC01361,LOC101927587*	.	.	.
1	83510730	G	T	intergenic	*LINC01361,LOC101927587*	.	.	.
1	83912863	C	T	intergenic	*LINC01361,LOC101927587*	.	.	.
1	83927853	T	G	intergenic	*LINC01361,LOC101927587*	.	.	.
1	87822083	C	T	ncRNA_intronic	*LINC01364*	.	.	.
1	88708583	C	A	intergenic	*LINC01364,PKN2-AS1*	.	.	.
20	38866091	A	G	intergenic	*LINC01370,MAFB*	.	.	.
20	39140517	G	A	intergenic	*LINC01370,MAFB*	.	.	.
20	38687893	C	A	intergenic	*LINC01370,MAFB*	.	.	.

20	39060851	G	T	intergenic	*LINC01370,MAFB*	.	.	.
20	39281901	A	C	intergenic	*LINC01370,MAFB*	.	.	.
20	38671431	C	T	intergenic	*LINC01370,MAFB*	.	.	.
20	39250242	A	C	intergenic	*LINC01370,MAFB*	.	.	.
20	39251267	T	C	intergenic	*LINC01370,MAFB*	.	.	.
7	67120973	TGTTGCCTTAGTCTGTGTTGTGTTGCTACAAAGCATCCCTGTATAGCAACACAACACAGACTAAGACAACACTTA	T	intergenic	*LINC01372,LOC102723427*	.	.	.
7	67044214	T	C	intergenic	*LINC01372,LOC102723427*	.	.	.
7	67404196	G	C	intergenic	*LINC01372,LOC102723427*	.	.	.
10	91693408	A	T	ncRNA_intronic	*LINC01375*	.	.	.
10	91695026	T	C	ncRNA_intronic	*LINC01375*	.	.	.
10	91914751	G	T	intergenic	*LINC01375,LOC101926942*	.	.	.
10	92015445	G	A	intergenic	*LINC01375,LOC101926942*	.	.	.
10	92025501	A	G	intergenic	*LINC01375,LOC101926942*	.	.	.
10	92025502	A	G	intergenic	*LINC01375,LOC101926942*	.	.	.
10	92040761	C	T	intergenic	*LINC01375,LOC101926942*	.	.	.
10	92040762	A	G	intergenic	*LINC01375,LOC101926942*	.	.	.
10	92094367	G	A	intergenic	*LINC01375,LOC101926942*	.	.	.
10	92129214	A	G	intergenic	*LINC01375,LOC101926942*	.	.	.

5	3271475	C	A	intergenic	*LINC01377,LINC01019*	.	.	.
5	3336120	G	A	intergenic	*LINC01377,LINC01019*	.	.	.
5	3370793	A	T	intergenic	*LINC01377,LINC01019*	.	.	.
5	3406228	T	C	intergenic	*LINC01377,LINC01019*	.	.	.
5	3291123	G	A	intergenic	*LINC01377,LINC01019*	.	.	.
5	3405926	A	C	intergenic	*LINC01377,LINC01019*	.	.	.
4	118441059	G	A	ncRNA_intronic	*LINC01378*	.	.	.
4	118441895	T	G	ncRNA_intronic	*LINC01378*	.	.	.
4	118578194	A	G	ncRNA_intronic	*LINC01378*	.	.	.
4	118579916	C	G	ncRNA_intronic	*LINC01378*	.	.	.
4	118602250	A	G	ncRNA_intronic	*LINC01378*	.	.	.
4	118842719	C	A	intergenic	*LINC01378,NDST3*	.	.	.
4	118633303	A	G	intergenic	*LINC01378,NDST3*	.	.	.
4	118780355	C	T	intergenic	*LINC01378,NDST3*	.	.	.
4	118858170	A	C	intergenic	*LINC01378,NDST3*	.	.	.
4	118874904	T	A	intergenic	*LINC01378,NDST3*	.	.	.
20	22692331	T	A	intergenic	*LINC01384,SSTR4*	.	.	.
20	22994072	CT	C	intergenic	*LINC01384,SSTR4*	.	.	.
20	22971245	C	T	intergenic	*LINC01384,SSTR4*	.	.	.
18	6518903	C	T	ncRNA_intronic	*LINC01387*	.	.	.
18	6586635	A	T	ncRNA_intronic	*LINC01387*	.	.	.
18	6619475	C	A	intergenic	*LINC01387,ARHGAP28*	.	.	.
18	6800210	C	A	intergenic	*LINC01387,ARHGAP28*	.	.	.
7	114795853	T	G	ncRNA_intronic	*LINC01392*	.	.	.
7	115125643	T	G	intergenic	*LINC01392,TFEC*	.	.	.
7	114896332	AATATAT	A	intergenic	*LINC01392,TFEC*	.	.	.
7	114920646	T	C	intergenic	*LINC01392,TFEC*	.	.	.
7	115230084	T	G	intergenic	*LINC01392,TFEC*	.	.	.
7	115237032	C	A	intergenic	*LINC01392,TFEC*	.	.	.
7	115237033	C	T	intergenic	*LINC01392,TFEC*	.	.	.

7	115478483	G	A	intergenic	*LINC01392,TFEC*	.	.	.
7	114724462	G	A	ncRNA_intronic	*LINC01393*	.	.	.
11	129516288	T	A	intergenic	*LINC01395,TMEM45B*	.	.	.
11	129520514	C	T	intergenic	*LINC01395,TMEM45B*	.	.	.
11	129521163	T	C	intergenic	*LINC01395,TMEM45B*	.	.	.
11	129531355	C	T	intergenic	*LINC01395,TMEM45B*	.	.	.
11	129531356	A	G	intergenic	*LINC01395,TMEM45B*	.	.	.
11	129531381	T	C	intergenic	*LINC01395,TMEM45B*	.	.	.
11	129531382	G	A	intergenic	*LINC01395,TMEM45B*	.	.	.
11	129532030	T	C	intergenic	*LINC01395,TMEM45B*	.	.	.
11	129533336	G	A	intergenic	*LINC01395,TMEM45B*	.	.	.
11	129585258	A	C	intergenic	*LINC01395,TMEM45B*	.	.	.
11	129625819	C	G	intergenic	*LINC01395,TMEM45B*	.	.	.
11	129625820	A	G	intergenic	*LINC01395,TMEM45B*	.	.	.
22	35583438	T	A	ncRNA_intronic	*LINC01399*	.	.	.
22	35600380	A	T	ncRNA_intronic	*LINC01399*	.	.	.
12	111428058	T	C	intergenic	*LINC01405,CUX2*	.	.	.
9	66457192	TAGAG	T	upstream	*LINC01410*	.	.	.
5	173868328	C	CAAATT	ncRNA_intronic	*LINC01411*	.	.	.
5	173883880	G	A	ncRNA_intronic	*LINC01411*	.	.	.
5	173814775	A	G	ncRNA_intronic	*LINC01411*	.	.	.
5	173897986	A	G	ncRNA_intronic	*LINC01411*	.	.	.
2	145374944	G	T	intergenic	*LINC01412,TEX41*	.	.	.
18	53555937	C	A	ncRNA_intronic	*LINC01416*	.	.	.
8	84633445	G	GA	intergenic	*LINC01419,RALYL*	.	.	.
8	84356304	TA	T	intergenic	*LINC01419,RALYL*	.	.	.

8	84753659	A	G	intergenic	*LINC01419,RALYL*	.	.	.
8	84757432	A	C	intergenic	*LINC01419,RALYL*	.	.	.
8	84823341	C	G	intergenic	*LINC01419,RALYL*	.	.	.
8	84477018	A	G	intergenic	*LINC01419,RALYL*	.	.	.
8	85055696	C	T	intergenic	*LINC01419,RALYL*	.	.	.
8	84603590	T	C	intergenic	*LINC01419,RALYL*	.	.	.
8	84624838	CTGGGATTACATGTAGGAGTCACCAAGCCTGGCTA	C	intergenic	*LINC01419,RALYL*	.	.	.
8	84406513	C	A	intergenic	*LINC01419,RALYL*	.	.	.
8	84516744	C	T	intergenic	*LINC01419,RALYL*	.	.	.
8	84516746	C	T	intergenic	*LINC01419,RALYL*	.	.	.
8	84518803	C	G	intergenic	*LINC01419,RALYL*	.	.	.
8	84518804	T	G	intergenic	*LINC01419,RALYL*	.	.	.
8	84529904	A	C	intergenic	*LINC01419,RALYL*	.	.	.
8	84529905	A	C	intergenic	*LINC01419,RALYL*	.	.	.
8	84532599	A	C	intergenic	*LINC01419,RALYL*	.	.	.
8	84532726	A	G	intergenic	*LINC01419,RALYL*	.	.	.
8	84532728	A	G	intergenic	*LINC01419,RALYL*	.	.	.
8	84558413	T	A	intergenic	*LINC01419,RALYL*	.	.	.
8	84558414	T	A	intergenic	*LINC01419,RALYL*	.	.	.
8	84640090	C	A	intergenic	*LINC01419,RALYL*	.	.	.
8	84679352	A	G	intergenic	*LINC01419,RALYL*	.	.	.
8	84744805	A	C	intergenic	*LINC01419,RALYL*	.	.	.
8	84791060	T	G	intergenic	*LINC01419,RALYL*	.	.	.
8	84815056	G	A	intergenic	*LINC01419,RALYL*	.	.	.
8	84879735	T	A	intergenic	*LINC01419,RALYL*	.	.	.
8	84927461	C	T	intergenic	*LINC01419,RALYL*	.	.	.
8	84927462	A	C	intergenic	*LINC01419,RALYL*	.	.	.
8	84992062	G	T	intergenic	*LINC01419,RALYL*	.	.	.
X	56937947	T	C	intergenic	*LINC01420,SPIN3*	.	.	.
X	56895077	G	T	intergenic	*LINC01420,SPIN3*	.	.	.
X	56895078	C	T	intergenic	*LINC01420,SPIN3*	.	.	.
X	56953679	C	T	intergenic	*LINC01420,SPIN3*	.	.	.
X	56863154	T	C	intergenic	*LINC01420,SPIN3*	.	.	.
X	56864203	T	C	intergenic	*LINC01420,SPIN3*	.	.	.

X	56865237	A	AT	intergenic	*LINC01420,SPIN3*	.	.	.
X	56897931	G	A	intergenic	*LINC01420,SPIN3*	.	.	.
X	56906316	G	T	intergenic	*LINC01420,SPIN3*	.	.	.
X	56921775	A	G	intergenic	*LINC01420,SPIN3*	.	.	.
X	56922792	T	C	intergenic	*LINC01420,SPIN3*	.	.	.
X	56923275	A	G	intergenic	*LINC01420,SPIN3*	.	.	.
X	56926016	A	T	intergenic	*LINC01420,SPIN3*	.	.	.
X	56964665	A	T	intergenic	*LINC01420,SPIN3*	.	.	.
X	56968470	T	C	intergenic	*LINC01420,SPIN3*	.	.	.
X	56968471	G	A	intergenic	*LINC01420,SPIN3*	.	.	.
22	27381817	A	G	intergenic	*LINC01422,MN1*	.	.	.
22	27910126	T	C	intergenic	*LINC01422,MN1*	.	.	.
22	27771234	G	A	intergenic	*LINC01422,MN1*	.	.	.
21	23146430	A	AAAAAATTA	ncRNA_intronic	*LINC01425*	.	.	.
21	23162507	T	G	ncRNA_intronic	*LINC01425*	.	.	.
21	23220554	C	T	intergenic	*LINC01425,LOC101927843*	.	.	.
21	23227157	C	T	intergenic	*LINC01425,LOC101927843*	.	.	.
20	7203625	G	A	ncRNA_intronic	*LINC01428*	.	.	.
20	7269698	CAT	C	intergenic	*LINC01428,LOC101929312*	.	.	.
20	50470826	T	C	ncRNA_intronic	*LINC01429*	.	.	.
20	50470827	G	A	ncRNA_intronic	*LINC01429*	.	.	.
20	50671325	A	G	intergenic	*LINC01429,ZFP64*	.	.	.
20	22183258	C	A	intergenic	*LINC01432,LINC01427*	.	.	.
20	22063178	TGCATATATCATATAGGTATATATGATATATGCGCATATATCATATAGGTATATATGATATATGC	T	intergenic	*LINC01432,LINC01427*	.	.	.
20	22161069	C	T	intergenic	*LINC01432,LINC01427*	.	.	.

20	22114821	C	G	intergenic	*LINC01432,LINC01427*	.	.	.
20	22166950	A	C	intergenic	*LINC01432,LINC01427*	.	.	.
20	22177110	T	A	intergenic	*LINC01432,LINC01427*	.	.	.
20	22177342	T	A	intergenic	*LINC01432,LINC01427*	.	.	.
20	22177515	T	C	intergenic	*LINC01432,LINC01427*	.	.	.
20	22179372	T	A	intergenic	*LINC01432,LINC01427*	.	.	.
20	22191266	G	A	intergenic	*LINC01432,LINC01427*	.	.	.
20	22191268	C	T	intergenic	*LINC01432,LINC01427*	.	.	.
10	109684734	T	G	ncRNA_intronic	*LINC01435*	.	.	.
10	109737778	T	A	ncRNA_intronic	*LINC01435*	.	.	.
10	109782611	C	A	ncRNA_intronic	*LINC01435*	.	.	.
10	109783396	G	A	ncRNA_intronic	*LINC01435*	.	.	.
10	109785793	T	G	ncRNA_intronic	*LINC01435*	.	.	.
10	109787015	T	C	ncRNA_intronic	*LINC01435*	.	.	.
10	109787016	G	A	ncRNA_intronic	*LINC01435*	.	.	.
10	109997025	GTGTGTGTGTGTGAA	G	intergenic	*LINC01435,XPNPEP1*	.	.	.
10	110543859	G	T	intergenic	*LINC01435,XPNPEP1*	.	.	.
10	110123307	AG	A	intergenic	*LINC01435,XPNPEP1*	.	.	.
10	110080583	G	T	intergenic	*LINC01435,XPNPEP1*	.	.	.
10	110281920	A	C	intergenic	*LINC01435,XPNPEP1*	.	.	.
10	110716621	G	A	intergenic	*LINC01435,XPNPEP1*	.	.	.
10	111162508	G	C	intergenic	*LINC01435,XPNPEP1*	.	.	.

10	110132529	T	C	intergenic	*LINC01435,XPNPEP1*	.	.	.
10	110257136	C	G	intergenic	*LINC01435,XPNPEP1*	.	.	.
10	110295076	C	T	intergenic	*LINC01435,XPNPEP1*	.	.	.
10	110295169	A	T	intergenic	*LINC01435,XPNPEP1*	.	.	.
10	110297553	C	A	intergenic	*LINC01435,XPNPEP1*	.	.	.
10	110297554	A	G	intergenic	*LINC01435,XPNPEP1*	.	.	.
10	110365519	A	T	intergenic	*LINC01435,XPNPEP1*	.	.	.
10	110396453	A	G	intergenic	*LINC01435,XPNPEP1*	.	.	.
10	110603972	C	T	intergenic	*LINC01435,XPNPEP1*	.	.	.
10	110711176	A	C	intergenic	*LINC01435,XPNPEP1*	.	.	.
10	110712666	T	C	intergenic	*LINC01435,XPNPEP1*	.	.	.
10	110712668	C	T	intergenic	*LINC01435,XPNPEP1*	.	.	.
10	110755296	C	T	intergenic	*LINC01435,XPNPEP1*	.	.	.
10	110756233	C	A	intergenic	*LINC01435,XPNPEP1*	.	.	.
10	110853658	G	A	intergenic	*LINC01435,XPNPEP1*	.	.	.
10	110856394	A	G	intergenic	*LINC01435,XPNPEP1*	.	.	.
10	110857291	A	T	intergenic	*LINC01435,XPNPEP1*	.	.	.
10	110894562	G	A	intergenic	*LINC01435,XPNPEP1*	.	.	.
10	110896316	T	C	intergenic	*LINC01435,XPNPEP1*	.	.	.

10	110896455	G	A	intergenic	*LINC01435,XPNPEP1*	.	.	.
10	110956161	T	G	intergenic	*LINC01435,XPNPEP1*	.	.	.
10	110957157	T	G	intergenic	*LINC01435,XPNPEP1*	.	.	.
10	110986394	A	C	intergenic	*LINC01435,XPNPEP1*	.	.	.
10	111130045	G	T	intergenic	*LINC01435,XPNPEP1*	.	.	.
10	111297342	G	T	intergenic	*LINC01435,XPNPEP1*	.	.	.
10	111299136	A	G	intergenic	*LINC01435,XPNPEP1*	.	.	.
10	111308478	A	G	intergenic	*LINC01435,XPNPEP1*	.	.	.
10	111332324	G	A	intergenic	*LINC01435,XPNPEP1*	.	.	.
10	111414339	G	A	intergenic	*LINC01435,XPNPEP1*	.	.	.
10	111415255	G	A	intergenic	*LINC01435,XPNPEP1*	.	.	.
10	111417043	T	A	intergenic	*LINC01435,XPNPEP1*	.	.	.
10	111417251	C	T	intergenic	*LINC01435,XPNPEP1*	.	.	.
10	111445237	T	C	intergenic	*LINC01435,XPNPEP1*	.	.	.
10	111446054	A	T	intergenic	*LINC01435,XPNPEP1*	.	.	.
20	54554423	G	A	intergenic	*LINC01441,CBLN4*	.	.	.
20	54124495	C	A	intergenic	*LINC01441,CBLN4*	.	.	.
20	54469708	C	A	intergenic	*LINC01441,CBLN4*	.	.	.
20	54060154	T	A	intergenic	*LINC01441,CBLN4*	.	.	.
20	54143624	G	A	intergenic	*LINC01441,CBLN4*	.	.	.
20	54397613	AGTGT	A	intergenic	*LINC01441,CBLN4*	.	.	.
18	15200485	T	C	intergenic	*LINC01443,LOC644669*	.	.	.

18	15300036	A	T	intergenic	*LINC01443,LOC644669*	.	.	.
18	15223368	T	G	intergenic	*LINC01443,LOC644669*	.	.	.
18	15035863	T	A	intergenic	*LINC01443,LOC644669*	.	.	.
18	15157568	T	A	intergenic	*LINC01443,LOC644669*	.	.	.
18	15225572	T	C	intergenic	*LINC01443,LOC644669*	.	.	.
18	15295631	A	G	intergenic	*LINC01443,LOC644669*	.	.	.
7	54519391	C	T	intergenic	*LINC01445,VSTM2A*	.	.	.
7	54575996	T	C	intergenic	*LINC01445,VSTM2A*	.	.	.
7	54567421	A	T	intergenic	*LINC01445,VSTM2A*	.	.	.
7	54567802	A	G	intergenic	*LINC01445,VSTM2A*	.	.	.
7	54568532	G	T	intergenic	*LINC01445,VSTM2A*	.	.	.
7	54578184	T	C	intergenic	*LINC01445,VSTM2A*	.	.	.
7	54578185	T	C	intergenic	*LINC01445,VSTM2A*	.	.	.
7	53853382	G	A	ncRNA_intronic	*LINC01446*	.	.	.
7	54193505	A	G	intergenic	*LINC01446,HPVC1*	.	.	.
7	54205908	C	A	intergenic	*LINC01446,HPVC1*	.	.	.
7	42913495	A	AGAAGATCT	intergenic	*LINC01448,C7orf25*	.	.	.
7	42838858	A	AAGATC	intergenic	*LINC01448,C7orf25*	.	.	.
7	42839804	A	G	intergenic	*LINC01448,C7orf25*	.	.	.
7	42839806	G	A	intergenic	*LINC01448,C7orf25*	.	.	.

7	42840897	T	C	intergenic	*LINC01448,C7orf25*	.	.	.
7	42841380	A	G	intergenic	*LINC01448,C7orf25*	.	.	.
7	42841381	A	G	intergenic	*LINC01448,C7orf25*	.	.	.
7	42899846	A	T	intergenic	*LINC01448,C7orf25*	.	.	.
7	41604997	G	A	intergenic	*LINC01449,INHBA*	.	.	.
7	41625153	G	A	intergenic	*LINC01449,INHBA*	.	.	.
7	41397517	C	CCACCGAG	intergenic	*LINC01449,INHBA*	.	.	.
7	41527088	G	T	intergenic	*LINC01449,INHBA*	.	.	.
7	41107109	T	C	intergenic	*LINC01450,LINC01449*	.	.	.
5	79257996	T	A	ncRNA_intronic	*LINC01455*	.	.	.
X	18041381	G	T	ncRNA_intronic	*LINC01456*	.	.	.
X	18088378	T	G	ncRNA_intronic	*LINC01456*	.	.	.
X	18127719	C	A	intergenic	*LINC01456,BEND2*	.	.	.
14	83841161	G	A	intergenic	*LINC01467,LINC00911*	.	.	.
14	83808366	G	A	intergenic	*LINC01467,LINC00911*	.	.	.
14	84095239	G	C	intergenic	*LINC01467,LINC00911*	.	.	.
14	82519916	C	A	intergenic	*LINC01467,NONE*	.	.	.
14	83790376	T	G	intergenic	*LINC01467,NONE*	.	.	.
14	82208964	C	T	intergenic	*LINC01467,NONE*	.	.	.
14	82567540	T	A	intergenic	*LINC01467,NONE*	.	.	.
14	83694394	A	G	intergenic	*LINC01467,NONE*	.	.	.
14	83767156	G	T	intergenic	*LINC01467,NONE*	.	.	.
14	83767157	A	T	intergenic	*LINC01467,NONE*	.	.	.
14	83288981	C	G	intergenic	*LINC01467,NONE*	.	.	.
14	82772128	C	A	intergenic	*LINC01467,NONE*	.	.	.
14	82808457	C	A	intergenic	*LINC01467,NONE*	.	.	.
14	83159459	G	A	intergenic	*LINC01467,NONE*	.	.	.
14	83404064	C	T	intergenic	*LINC01467,NONE*	.	.	.
14	82970865	G	T	intergenic	*LINC01467,NONE*	.	.	.
14	82172964	A	T	intergenic	*LINC01467,NONE*	.	.	.

14	82172965	C	T	intergenic	*LINC01467,NONE*	.	.	.
14	82173889	T	C	intergenic	*LINC01467,NONE*	.	.	.
14	82328236	G	A	intergenic	*LINC01467,NONE*	.	.	.
14	82785003	T	C	intergenic	*LINC01467,NONE*	.	.	.
14	82842174	T	C	intergenic	*LINC01467,NONE*	.	.	.
14	82934667	G	A	intergenic	*LINC01467,NONE*	.	.	.
14	82935153	G	A	intergenic	*LINC01467,NONE*	.	.	.
14	83008467	T	C	intergenic	*LINC01467,NONE*	.	.	.
14	83008469	T	C	intergenic	*LINC01467,NONE*	.	.	.
14	83043675	A	G	intergenic	*LINC01467,NONE*	.	.	.
14	83645089	T	C	intergenic	*LINC01467,NONE*	.	.	.
14	83743362	T	TAGAGA	intergenic	*LINC01467,NONE*	.	.	.
10	54319222	G	A	intergenic	*LINC01468,MBL2*	.	.	.
10	54356912	CAAA	C	intergenic	*LINC01468,MBL2*	.	.	.
10	54479257	T	C	intergenic	*LINC01468,MBL2*	.	.	.
10	54479633	C	T	intergenic	*LINC01468,MBL2*	.	.	.
10	54490561	G	A	intergenic	*LINC01468,MBL2*	.	.	.
5	152241360	G	T	ncRNA_intronic	*LINC01470*	.	.	.
5	152177513	T	C	ncRNA_intronic	*LINC01470*	.	.	.
5	152177515	C	T	ncRNA_intronic	*LINC01470*	.	.	.
5	152001651	C	T	ncRNA_intronic	*LINC01470*	.	.	.
5	152233017	C	A	ncRNA_intronic	*LINC01470*	.	.	.
5	152351603	C	T	ncRNA_intronic	*LINC01470*	.	.	.
5	152204322	A	T	ncRNA_intronic	*LINC01470*	.	.	.
5	152124687	A	G	ncRNA_intronic	*LINC01470*	.	.	.
5	152137339	A	G	ncRNA_intronic	*LINC01470*	.	.	.
5	152153585	T	C	ncRNA_intronic	*LINC01470*	.	.	.
5	152168715	C	T	ncRNA_intronic	*LINC01470*	.	.	.
5	152172464	C	T	ncRNA_intronic	*LINC01470*	.	.	.
5	152295949	G	C	ncRNA_intronic	*LINC01470*	.	.	.
5	152394460	TTATATA	T	intergenic	*LINC01470,GRIA1*	.	.	.
5	152564297	G	T	intergenic	*LINC01470,GRIA1*	.	.	.
5	152667655	A	ACTAAAG	intergenic	*LINC01470,GRIA1*	.	.	.
5	152373737	G	T	intergenic	*LINC01470,GRIA1*	.	.	.
5	152540830	C	A	intergenic	*LINC01470,GRIA1*	.	.	.
5	152687723	A	G	intergenic	*LINC01470,GRIA1*	.	.	.
5	152560599	T	A	intergenic	*LINC01470,GRIA1*	.	.	.
5	152563381	A	T	intergenic	*LINC01470,GRIA1*	.	.	.

5	152597251	G	A	intergenic	*LINC01470,GRIA1*	.	.	.
5	152664360	C	T	intergenic	*LINC01470,GRIA1*	.	.	.
5	152665531	T	A	intergenic	*LINC01470,GRIA1*	.	.	.
5	152667279	A	G	intergenic	*LINC01470,GRIA1*	.	.	.
5	152667280	T	G	intergenic	*LINC01470,GRIA1*	.	.	.
5	152667633	C	T	intergenic	*LINC01470,GRIA1*	.	.	.
5	152671030	G	A	intergenic	*LINC01470,GRIA1*	.	.	.
5	152671592	T	C	intergenic	*LINC01470,GRIA1*	.	.	.
5	152683192	G	T	intergenic	*LINC01470,GRIA1*	.	.	.
5	152714610	T	A	intergenic	*LINC01470,GRIA1*	.	.	.
5	152714611	T	C	intergenic	*LINC01470,GRIA1*	.	.	.
5	152766685	T	C	intergenic	*LINC01470,GRIA1*	.	.	.
5	152766686	A	C	intergenic	*LINC01470,GRIA1*	.	.	.
5	152799074	A	T	intergenic	*LINC01470,GRIA1*	.	.	.
5	152804984	G	T	intergenic	*LINC01470,GRIA1*	.	.	.
2	187163992	A	G	intergenic	*LINC01473,ZC3H15*	.	.	.
2	187237543	T	C	intergenic	*LINC01473,ZC3H15*	.	.	.
2	187099027	C	T	intergenic	*LINC01473,ZC3H15*	.	.	.
2	187130663	C	T	intergenic	*LINC01473,ZC3H15*	.	.	.
2	187130664	T	G	intergenic	*LINC01473,ZC3H15*	.	.	.
2	187132902	G	A	intergenic	*LINC01473,ZC3H15*	.	.	.
2	187133296	G	A	intergenic	*LINC01473,ZC3H15*	.	.	.
2	187133298	T	C	intergenic	*LINC01473,ZC3H15*	.	.	.
2	187179959	G	C	intergenic	*LINC01473,ZC3H15*	.	.	.
2	187181112	G	GAA	intergenic	*LINC01473,ZC3H15*	.	.	.
2	187182107	C	G	intergenic	*LINC01473,ZC3H15*	.	.	.

2	187183313	T	C	intergenic	*LINC01473,ZC3H15*	.	.	.
2	187183314	G	A	intergenic	*LINC01473,ZC3H15*	.	.	.
2	187235905	G	A	intergenic	*LINC01473,ZC3H15*	.	.	.
2	187256445	A	G	intergenic	*LINC01473,ZC3H15*	.	.	.
2	187292045	C	T	intergenic	*LINC01473,ZC3H15*	.	.	.
17	57527617	C	T	ncRNA_intronic	*LINC01476*	.	.	.
18	39012325	A	G	intergenic	*LINC01477,KC6*	.	.	.
18	38442596	C	G	intergenic	*LINC01477,KC6*	.	.	.
18	37889931	C	A	intergenic	*LINC01477,KC6*	.	.	.
18	37884803	G	T	intergenic	*LINC01477,KC6*	.	.	.
18	38434381	G	T	intergenic	*LINC01477,KC6*	.	.	.
18	37966024	C	T	intergenic	*LINC01477,KC6*	.	.	.
18	38180350	A	G	intergenic	*LINC01477,KC6*	.	.	.
18	38467307	C	A	intergenic	*LINC01477,KC6*	.	.	.
18	38231131	G	T	intergenic	*LINC01477,KC6*	.	.	.
18	37908050	G	T	intergenic	*LINC01477,KC6*	.	.	.
18	38414540	C	A	intergenic	*LINC01477,KC6*	.	.	.
18	38435272	A	G	intergenic	*LINC01477,KC6*	.	.	.
18	38717212	A	C	intergenic	*LINC01477,KC6*	.	.	.
18	38900514	T	C	intergenic	*LINC01477,KC6*	.	.	.
18	37682292	G	T	intergenic	*LINC01477,KC6*	.	.	.
18	38534620	G	T	intergenic	*LINC01477,KC6*	.	.	.
18	38893996	T	A	intergenic	*LINC01477,KC6*	.	.	.
18	37738528	TGG	T	intergenic	*LINC01477,KC6*	.	.	.
18	37809863	A	G	intergenic	*LINC01477,KC6*	.	.	.
18	38051837	C	T	intergenic	*LINC01477,KC6*	.	.	.
18	38092604	T	C	intergenic	*LINC01477,KC6*	.	.	.
18	38309402	A	ATTTG	intergenic	*LINC01477,KC6*	.	.	.
18	38404923	T	C	intergenic	*LINC01477,KC6*	.	.	.
18	38668179	C	A	intergenic	*LINC01477,KC6*	.	.	.
18	38669109	C	T	intergenic	*LINC01477,KC6*	.	.	.
18	41989419	T	C	ncRNA_intronic	*LINC01478*	.	.	.
18	42103924	T	A	ncRNA_intronic	*LINC01478*	.	.	.

18	41930144	A	C	ncRNA_intronic	*LINC01478*	.	.	.
18	41949164	T	G	ncRNA_intronic	*LINC01478*	.	.	.
18	41998376	T	G	ncRNA_intronic	*LINC01478*	.	.	.
18	42011987	G	A	ncRNA_intronic	*LINC01478*	.	.	.
18	42012064	T	G	ncRNA_intronic	*LINC01478*	.	.	.
18	42012065	A	G	ncRNA_intronic	*LINC01478*	.	.	.
18	42026314	A	T	ncRNA_intronic	*LINC01478*	.	.	.
18	42027193	C	T	ncRNA_intronic	*LINC01478*	.	.	.
18	42027718	T	C	ncRNA_intronic	*LINC01478*	.	.	.
12	68367627	G	A	intergenic	*LINC01479,IFNG-AS1*	.	.	.
17	66854844	A	T	intergenic	*LINC01482,ABCA8*	.	.	.
17	66841801	T	C	intergenic	*LINC01482,ABCA8*	.	.	.
17	66841802	G	A	intergenic	*LINC01482,ABCA8*	.	.	.
17	67597132	ATT	A	ncRNA_intronic	*LINC01483*	.	.	.
17	67610226	C	T	ncRNA_intronic	*LINC01483*	.	.	.
17	67610564	C	A	ncRNA_intronic	*LINC01483*	.	.	.
17	67610565	A	G	ncRNA_intronic	*LINC01483*	.	.	.
17	67628529	T	C	ncRNA_intronic	*LINC01483*	.	.	.
17	67733060	G	A	ncRNA_intronic	*LINC01483*	.	.	.
17	67921652	T	C	intergenic	*LINC01483,LINC01497*	.	.	.
17	67923572	A	G	intergenic	*LINC01483,LINC01497*	.	.	.
5	173192723	A	G	intergenic	*LINC01484,LINC01485*	.	.	.
3	154963913	T	A	intergenic	*LINC01487,LOC100507537*	.	.	.
12	15223983	C	G	intergenic	*LINC01489,RERG*	.	.	.
12	15224042	T	C	intergenic	*LINC01489,RERG*	.	.	.
12	15224368	C	G	intergenic	*LINC01489,RERG*	.	.	.
15	48347566	C	A	intergenic	*LINC01491,SLC24A5*	.	.	.
9	106078543	A	T	ncRNA_intronic	*LINC01492*	.	.	.
9	105907247	T	C	ncRNA_intronic	*LINC01492*	.	.	.
9	106074974	G	T	ncRNA_intronic	*LINC01492*	.	.	.
9	106458214	C	CAT	intergenic	*LINC01492,LOC101928523*	.	.	.

9	106556032	T	C	intergenic	*LINC01492,LOC101928523*	.	.	.
9	106565294	C	CA	intergenic	*LINC01492,LOC101928523*	.	.	.
9	106196839	T	G	intergenic	*LINC01492,LOC101928523*	.	.	.
9	106199272	C	G	intergenic	*LINC01492,LOC101928523*	.	.	.
9	106279015	A	C	intergenic	*LINC01492,LOC101928523*	.	.	.
9	106355087	T	G	intergenic	*LINC01492,LOC101928523*	.	.	.
9	106368605	C	A	intergenic	*LINC01492,LOC101928523*	.	.	.
9	106413094	G	A	intergenic	*LINC01492,LOC101928523*	.	.	.
9	106515533	A	G	intergenic	*LINC01492,LOC101928523*	.	.	.
11	38670031	A	T	upstream	*LINC01493*	.	.	.
11	38836210	AT	A	intergenic	*LINC01493,LRRC4C*	.	.	.
11	38836212	AAT	A	intergenic	*LINC01493,LRRC4C*	.	.	.
11	39153030	T	TTA	intergenic	*LINC01493,LRRC4C*	.	.	.
11	39960538	AT	A	intergenic	*LINC01493,LRRC4C*	.	.	.
11	40010897	C	A	intergenic	*LINC01493,LRRC4C*	.	.	.
11	39061649	A	ACAGAAGTT	intergenic	*LINC01493,LRRC4C*	.	.	.
11	39399231	C	T	intergenic	*LINC01493,LRRC4C*	.	.	.
11	39664350	C	A	intergenic	*LINC01493,LRRC4C*	.	.	.
11	40117221	C	G	intergenic	*LINC01493,LRRC4C*	.	.	.

11	38966622	G	T	intergenic	*LINC01493,LRRC4C*	.	.	.
11	39423535	A	T	intergenic	*LINC01493,LRRC4C*	.	.	.
11	40076739	A	T	intergenic	*LINC01493,LRRC4C*	.	.	.
11	39374235	T	G	intergenic	*LINC01493,LRRC4C*	.	.	.
11	39122994	A	G	intergenic	*LINC01493,LRRC4C*	.	.	.
11	39280023	A	AT	intergenic	*LINC01493,LRRC4C*	.	.	.
11	39185542	T	C	intergenic	*LINC01493,LRRC4C*	.	.	.
11	39643886	T	A	intergenic	*LINC01493,LRRC4C*	.	.	.
11	39771804	G	T	intergenic	*LINC01493,LRRC4C*	.	.	.
11	39982716	TA	T	intergenic	*LINC01493,LRRC4C*	.	.	.
11	39906192	A	T	intergenic	*LINC01493,LRRC4C*	.	.	.
11	38750727	T	G	intergenic	*LINC01493,LRRC4C*	.	.	.
11	38750729	T	G	intergenic	*LINC01493,LRRC4C*	.	.	.
11	38750801	C	T	intergenic	*LINC01493,LRRC4C*	.	.	.
11	38750802	A	T	intergenic	*LINC01493,LRRC4C*	.	.	.
11	38766191	G	C	intergenic	*LINC01493,LRRC4C*	.	.	.
11	38766192	A	T	intergenic	*LINC01493,LRRC4C*	.	.	.
11	38783312	A	G	intergenic	*LINC01493,LRRC4C*	.	.	.
11	38783313	A	G	intergenic	*LINC01493,LRRC4C*	.	.	.

11	38784041	G	A	intergenic	*LINC01493,LRRC4C*	.	.	.
11	38784704	T	G	intergenic	*LINC01493,LRRC4C*	.	.	.
11	38799282	T	TCA	intergenic	*LINC01493,LRRC4C*	.	.	.
11	38928376	G	A	intergenic	*LINC01493,LRRC4C*	.	.	.
11	38937489	A	C	intergenic	*LINC01493,LRRC4C*	.	.	.
11	38940361	T	G	intergenic	*LINC01493,LRRC4C*	.	.	.
11	39329820	T	C	intergenic	*LINC01493,LRRC4C*	.	.	.
11	39329988	A	AG	intergenic	*LINC01493,LRRC4C*	.	.	.
11	39332364	C	T	intergenic	*LINC01493,LRRC4C*	.	.	.
11	39352088	A	G	intergenic	*LINC01493,LRRC4C*	.	.	.
11	39352148	T	A	intergenic	*LINC01493,LRRC4C*	.	.	.
11	39542254	T	C	intergenic	*LINC01493,LRRC4C*	.	.	.
11	39657059	C	A	intergenic	*LINC01493,LRRC4C*	.	.	.
11	39675489	A	G	intergenic	*LINC01493,LRRC4C*	.	.	.
11	39732653	C	T	intergenic	*LINC01493,LRRC4C*	.	.	.
11	39834365	G	A	intergenic	*LINC01493,LRRC4C*	.	.	.
11	39862517	C	T	intergenic	*LINC01493,LRRC4C*	.	.	.
11	39871116	C	T	intergenic	*LINC01493,LRRC4C*	.	.	.
11	39906313	G	T	intergenic	*LINC01493,LRRC4C*	.	.	.

11	39913766	C	T	intergenic	*LINC01493,LRRC4C*	.	.	.
11	40010680	T	C	intergenic	*LINC01493,LRRC4C*	.	.	.
11	40010681	G	A	intergenic	*LINC01493,LRRC4C*	.	.	.
11	40061888	A	T	intergenic	*LINC01493,LRRC4C*	.	.	.
11	40062491	T	A	intergenic	*LINC01493,LRRC4C*	.	.	.
11	40062692	T	C	intergenic	*LINC01493,LRRC4C*	.	.	.
11	40064593	T	A	intergenic	*LINC01493,LRRC4C*	.	.	.
11	22553149	A	AT	intergenic	*LINC01495,FANCF*	.	.	.
11	22547703	A	G	intergenic	*LINC01495,FANCF*	.	.	.
X	51378472	ATTTTTTTACT	A	intergenic	*LINC01496,CENPVP2*	.	.	.
X	51338795	T	A	intergenic	*LINC01496,CENPVP2*	.	.	.
X	51330538	G	C	intergenic	*LINC01496,CENPVP2*	.	.	.
X	51283843	T	A	intergenic	*LINC01496,CENPVP2*	.	.	.
X	51283844	T	C	intergenic	*LINC01496,CENPVP2*	.	.	.
X	51295619	A	G	intergenic	*LINC01496,CENPVP2*	.	.	.
X	51335209	G	C	intergenic	*LINC01496,CENPVP2*	.	.	.
X	51369289	A	C	intergenic	*LINC01496,CENPVP2*	.	.	.
X	51369680	T	C	intergenic	*LINC01496,CENPVP2*	.	.	.
X	51370293	G	A	intergenic	*LINC01496,CENPVP2*	.	.	.
X	51389830	G	T	intergenic	*LINC01496,CENPVP2*	.	.	.

X	51390649	A	G	intergenic	*LINC01496,CENPVP2*	.	.	.
X	51390700	A	G	intergenic	*LINC01496,CENPVP2*	.	.	.
11	41745436	A	G	ncRNA_intronic	*LINC01499*	.	.	.
11	41746823	A	G	ncRNA_intronic	*LINC01499*	.	.	.
11	42122178	T	A	intergenic	*LINC01499,LOC100507205*	.	.	.
11	41882346	T	C	intergenic	*LINC01499,LOC100507205*	.	.	.
11	41888403	T	C	intergenic	*LINC01499,LOC100507205*	.	.	.
11	41961449	CAGAG	C	intergenic	*LINC01499,LOC100507205*	.	.	.
11	41937658	C	T	intergenic	*LINC01499,LOC100507205*	.	.	.
11	42178179	T	A	intergenic	*LINC01499,LOC100507205*	.	.	.
11	42178456	C	T	intergenic	*LINC01499,LOC100507205*	.	.	.
14	59466035	G	GT	ncRNA_intronic	*LINC01500*	.	.	.
14	59502815	C	T	intergenic	*LINC01500,DAAM1*	.	.	.
14	59538771	A	G	intergenic	*LINC01500,DAAM1*	.	.	.
9	93326249	T	C	ncRNA_intronic	*LINC01501*	.	.	.
9	109576480	T	C	intergenic	*LINC01505,ZNF462*	.	.	.
9	109585722	G	A	intergenic	*LINC01505,ZNF462*	.	.	.
9	109585723	T	C	intergenic	*LINC01505,ZNF462*	.	.	.
9	71203670	T	A	intergenic	*LINC01506,PIP5K1B*	.	.	.
9	71204937	A	T	intergenic	*LINC01506,PIP5K1B*	.	.	.
9	82462461	A	G	ncRNA_intronic	*LINC01507*	.	.	.
9	82472154	A	T	ncRNA_intronic	*LINC01507*	.	.	.

9	82527697	T	C	ncRNA_intronic	*LINC01507*	.	.	.
9	82571740	G	A	ncRNA_intronic	*LINC01507*	.	.	.
9	82808162	T	C	intergenic	*LINC01507,TLE1*	.	.	.
9	82885053	C	T	intergenic	*LINC01507,TLE1*	.	.	.
9	82934039	T	C	intergenic	*LINC01507,TLE1*	.	.	.
9	83058196	T	C	intergenic	*LINC01507,TLE1*	.	.	.
9	82937975	AT	A	intergenic	*LINC01507,TLE1*	.	.	.
9	82963526	T	A	intergenic	*LINC01507,TLE1*	.	.	.
9	83269036	T	C	intergenic	*LINC01507,TLE1*	.	.	.
9	83269037	T	C	intergenic	*LINC01507,TLE1*	.	.	.
9	83883372	AATATAT	A	intergenic	*LINC01507,TLE1*	.	.	.
9	82733467	A	T	intergenic	*LINC01507,TLE1*	.	.	.
9	82734495	G	A	intergenic	*LINC01507,TLE1*	.	.	.
9	82736218	G	A	intergenic	*LINC01507,TLE1*	.	.	.
9	82911540	T	C	intergenic	*LINC01507,TLE1*	.	.	.
9	83194084	G	A	intergenic	*LINC01507,TLE1*	.	.	.
9	83224799	A	G	intergenic	*LINC01507,TLE1*	.	.	.
9	83271818	G	A	intergenic	*LINC01507,TLE1*	.	.	.
9	83277120	A	G	intergenic	*LINC01507,TLE1*	.	.	.
9	83433848	C	A	intergenic	*LINC01507,TLE1*	.	.	.
9	83435208	C	T	intergenic	*LINC01507,TLE1*	.	.	.
9	83532085	T	C	intergenic	*LINC01507,TLE1*	.	.	.
9	83835318	C	T	intergenic	*LINC01507,TLE1*	.	.	.
9	83837303	C	T	intergenic	*LINC01507,TLE1*	.	.	.
9	93125481	C	T	ncRNA_intronic	*LINC01508*	.	.	.
9	110243985	C	T	intergenic	*LINC01509,KLF4*	.	.	.
7	116225359	G	T	ncRNA_intronic	*LINC01510*	.	.	.
7	116208870	TTATATA	T	ncRNA_intronic	*LINC01510*	.	.	.
6	43914433	AAGAG	A	intergenic	*LINC01512,LOC101929705*	.	.	.
10	67348290	T	G	ncRNA_intronic	*LINC01515*	.	.	.
10	67349624	T	C	ncRNA_intronic	*LINC01515*	.	.	.
10	67377496	A	G	ncRNA_intronic	*LINC01515*	.	.	.
10	67407735	C	T	ncRNA_intronic	*LINC01515*	.	.	.
10	67407736	A	G	ncRNA_intronic	*LINC01515*	.	.	.
10	67579496	A	G	intergenic	*LINC01515,CTNNA3*	.	.	.

10	67597200	G	A	intergenic	*LINC01515,CTNNA3*	.	.	.
10	67601973	G	T	intergenic	*LINC01515,CTNNA3*	.	.	.
10	67581859	C	A	intergenic	*LINC01515,CTNNA3*	.	.	.
10	67572349	G	A	intergenic	*LINC01515,CTNNA3*	.	.	.
10	43250310	T	A	intergenic	*LINC01518,BMS1*	.	.	.
10	87052429	A	ACG	intergenic	*LINC01519,LOC101929646*	.	.	.
10	87035572	C	T	intergenic	*LINC01519,LOC101929646*	.	.	.
10	87261558	G	T	intergenic	*LINC01520,GRID1-AS1*	.	.	.
10	87276759	C	T	intergenic	*LINC01520,GRID1-AS1*	.	.	.
22	31771484	A	T	intergenic	*LINC01521,DRG1*	.	.	.
20	46956508	C	T	intergenic	*LINC01522,LINC00494*	.	.	.
20	46844603	C	A	intergenic	*LINC01522,LINC00494*	.	.	.
20	46776154	CAGAG	C	intergenic	*LINC01522,LINC00494*	.	.	.
20	51388664	G	T	intergenic	*LINC01524,TSHZ2*	.	.	.
1	117858263	A	G	ncRNA_intronic	*LINC01525*	.	.	.
1	117896572	T	C	intergenic	*LINC01525,MAN1A2*	.	.	.
1	117898474	T	C	intergenic	*LINC01525,MAN1A2*	.	.	.
6	82784298	A	G	intergenic	*LINC01526,IBTK*	.	.	.
6	82720416	G	GGT	intergenic	*LINC01526,IBTK*	.	.	.
6	82641720	T	G	intergenic	*LINC01526,IBTK*	.	.	.
6	82664850	A	G	intergenic	*LINC01526,IBTK*	.	.	.
6	82666758	A	G	intergenic	*LINC01526,IBTK*	.	.	.
6	82682237	T	C	intergenic	*LINC01526,IBTK*	.	.	.
6	82813035	C	A	intergenic	*LINC01526,IBTK*	.	.	.
6	82813037	T	C	intergenic	*LINC01526,IBTK*	.	.	.

6	82834572	C	T	intergenic	*LINC01526,IBTK*	.	.	.
6	82865715	T	G	intergenic	*LINC01526,IBTK*	.	.	.
19	29544118	GGAGA	G	intergenic	*LINC01532,UQCRFS1*	.	.	.
19	29671038	G	T	intergenic	*LINC01532,UQCRFS1*	.	.	.
19	29666250	A	G	intergenic	*LINC01532,UQCRFS1*	.	.	.
19	29625928	A	G	intergenic	*LINC01532,UQCRFS1*	.	.	.
19	29628491	G	A	intergenic	*LINC01532,UQCRFS1*	.	.	.
18	61895396	G	C	ncRNA_intronic	*LINC01538,LOC284294*	.	.	.
18	53800041	C	T	ncRNA_intronic	*LINC01539*	.	.	.
18	53800042	T	C	ncRNA_intronic	*LINC01539*	.	.	.
18	53812603	A	T	intergenic	*LINC01539,TXNL1*	.	.	.
18	54036203	T	C	intergenic	*LINC01539,TXNL1*	.	.	.
18	53982747	C	T	intergenic	*LINC01539,TXNL1*	.	.	.
18	54010920	G	T	intergenic	*LINC01539,TXNL1*	.	.	.
18	69198354	T	A	ncRNA_intronic	*LINC01541*	.	.	.
18	69297816	T	C	intergenic	*LINC01541,LOC102724913*	.	.	.
18	59457515	C	A	intergenic	*LINC01544,RNF152*	.	.	.
X	3192958	C	A	ncRNA_intronic	*LINC01546*	.	.	.
21	34558283	A	G	intergenic	*LINC01548,IFNAR2*	.	.	.
14	98390955	GCACACA	G	downstream	*LINC01550*	.	.	.
14	98450648	C	T	intergenic	*LINC01550,C14orf177*	.	.	.
14	98475822	A	T	intergenic	*LINC01550,C14orf177*	.	.	.
14	98512988	T	A	intergenic	*LINC01550,C14orf177*	.	.	.
14	98573678	A	C	intergenic	*LINC01550,C14orf177*	.	.	.

14	98877037	A	C	intergenic	*LINC01550,C14orf177*	.	.	.
14	99068245	C	T	intergenic	*LINC01550,C14orf177*	.	.	.
14	98469716	G	A	intergenic	*LINC01550,C14orf177*	.	.	.
14	29513570	G	A	intergenic	*LINC01551,MIR548AI*	.	.	.
14	29682769	G	T	intergenic	*LINC01551,MIR548AI*	.	.	.
14	29428372	A	T	intergenic	*LINC01551,MIR548AI*	.	.	.
14	29646481	A	G	intergenic	*LINC01551,MIR548AI*	.	.	.
14	29832715	A	G	intergenic	*LINC01551,MIR548AI*	.	.	.
14	29407457	G	A	intergenic	*LINC01551,MIR548AI*	.	.	.
14	29408664	A	G	intergenic	*LINC01551,MIR548AI*	.	.	.
14	29463088	G	GT	intergenic	*LINC01551,MIR548AI*	.	.	.
14	29471699	T	G	intergenic	*LINC01551,MIR548AI*	.	.	.
14	29472725	C	T	intergenic	*LINC01551,MIR548AI*	.	.	.
14	29497051	T	C	intergenic	*LINC01551,MIR548AI*	.	.	.
14	29497052	G	A	intergenic	*LINC01551,MIR548AI*	.	.	.
14	29588044	A	AGCACTGAG	intergenic	*LINC01551,MIR548AI*	.	.	.
14	29753853	A	C	intergenic	*LINC01551,MIR548AI*	.	.	.
14	29754133	T	C	intergenic	*LINC01551,MIR548AI*	.	.	.
14	29882484	A	T	intergenic	*LINC01551,MIR548AI*	.	.	.

10	23515261	G	A	ncRNA_intronic	*LINC01552*	.	.	.
10	23516765	T	C	ncRNA_intronic	*LINC01552*	.	.	.
12	13640790	G	A	intergenic	*LINC01559,GRIN2B*	.	.	.
12	13672223	A	T	intergenic	*LINC01559,GRIN2B*	.	.	.
16	34650359	G	T	intergenic	*LINC01566,FRG2DP*	.	.	.
16	34650854	A	G	intergenic	*LINC01566,FRG2DP*	.	.	.
16	34706198	A	G	intergenic	*LINC01566,FRG2DP*	.	.	.
16	34708675	A	G	intergenic	*LINC01566,FRG2DP*	.	.	.
16	74127416	G	A	intergenic	*LINC01568,LOC101928035*	.	.	.
16	73789838	G	A	intergenic	*LINC01568,LOC101928035*	.	.	.
16	73871519	ATTTTCTTTTC	A	intergenic	*LINC01568,LOC101928035*	.	.	.
16	73665084	C	G	intergenic	*LINC01568,LOC101928035*	.	.	.
16	73701480	C	T	intergenic	*LINC01568,LOC101928035*	.	.	.
16	73618612	T	G	intergenic	*LINC01568,LOC101928035*	.	.	.
16	73810235	C	T	intergenic	*LINC01568,LOC101928035*	.	.	.
16	73968253	C	G	intergenic	*LINC01568,LOC101928035*	.	.	.
16	5650745	G	A	downstream	*LINC01570*	.	.	.
16	51845570	A	G	intergenic	*LINC01571,C16orf97*	.	.	.
16	51922364	A	T	intergenic	*LINC01571,C16orf97*	.	.	.
16	52044477	T	C	intergenic	*LINC01571,C16orf97*	.	.	.
16	72362496	T	C	ncRNA_intronic	*LINC01572*	.	.	.

16	72362497	C	T	ncRNA_intronic	*LINC01572*	.	.	.
16	72560173	C	T	ncRNA_intronic	*LINC01572*	.	.	.
16	72580319	A	C	ncRNA_intronic	*LINC01572*	.	.	.
16	72581908	T	A	ncRNA_intronic	*LINC01572*	.	.	.
16	72726638	T	A	intergenic	*LINC01572,ZFHX3*	.	.	.
5	176204450	G	A	intergenic	*LINC01574,UNC5A*	.	.	.
15	94694295	G	T	intergenic	*LINC01581,MCTP2*	.	.	.
15	94752610	T	A	intergenic	*LINC01581,MCTP2*	.	.	.
15	94752813	G	C	intergenic	*LINC01581,MCTP2*	.	.	.
15	94756179	T	A	intergenic	*LINC01581,MCTP2*	.	.	.
15	94761133	A	T	intergenic	*LINC01581,MCTP2*	.	.	.
15	98915903	T	A	intergenic	*LINC01582,FAM169B*	.	.	.
4	5547697	C	T	intergenic	*LINC01587,EVC2*	.	.	.
22	46009768	C	T	intergenic	*LINC01589,ATXN10*	.	.	.
22	46019245	C	T	intergenic	*LINC01589,ATXN10*	.	.	.
8	136406393	A	T	intergenic	*LINC01591,KHDRBS3*	.	.	.
8	136350531	A	G	intergenic	*LINC01591,KHDRBS3*	.	.	.
8	136350815	T	C	intergenic	*LINC01591,KHDRBS3*	.	.	.
8	136351596	G	A	intergenic	*LINC01591,KHDRBS3*	.	.	.
8	136351597	T	C	intergenic	*LINC01591,KHDRBS3*	.	.	.
8	136395930	A	T	intergenic	*LINC01591,KHDRBS3*	.	.	.
8	69861508	C	T	ncRNA_intronic	*LINC01592*	.	.	.
8	69836459	T	C	ncRNA_intronic	*LINC01592*	.	.	.
8	69841199	A	C	ncRNA_intronic	*LINC01592*	.	.	.
8	69870359	T	C	ncRNA_intronic	*LINC01592*	.	.	.
8	69870360	G	C	ncRNA_intronic	*LINC01592*	.	.	.
8	69881240	A	G	ncRNA_intronic	*LINC01592*	.	.	.
8	70004072	G	T	ncRNA_intronic	*LINC01592*	.	.	.

8	70196333	G	A	intergenic	*LINC01592,LINC01603*	.	.	.
8	70267014	C	T	intergenic	*LINC01592,LINC01603*	.	.	.
2	108817883	A	G	ncRNA_intronic	*LINC01594*	.	.	.
2	108822273	A	G	ncRNA_intronic	*LINC01594*	.	.	.
2	108822951	C	A	ncRNA_intronic	*LINC01594*	.	.	.
2	108839917	G	C	intergenic	*LINC01594,SULT1C3*	.	.	.
4	190846194	C	A	intergenic	*LINC01596,FRG1*	.	.	.
4	190854096	A	AAAGTT	intergenic	*LINC01596,FRG1*	.	.	.
20	29600590	C	T	intergenic	*LINC01598,FRG1BP*	.	.	.
20	29606875	AAAAC	A	intergenic	*LINC01598,FRG1BP*	.	.	.
20	29590129	A	G	intergenic	*LINC01598,FRG1BP*	.	.	.
20	29585772	A	G	intergenic	*LINC01598,FRG1BP*	.	.	.
20	29596003	T	A	intergenic	*LINC01598,FRG1BP*	.	.	.
20	29602382	A	T	intergenic	*LINC01598,FRG1BP*	.	.	.
20	29582512	G	C	intergenic	*LINC01598,FRG1BP*	.	.	.
20	29585340	A	T	intergenic	*LINC01598,FRG1BP*	.	.	.
14	50543723	G	A	ncRNA_intronic	*LINC01599*	.	.	.
8	70365118	A	G	intergenic	*LINC01603,SULF1*	.	.	.
8	37320429	T	C	ncRNA_intronic	*LINC01605*	.	.	.
8	111955133	G	A	ncRNA_intronic	*LINC01608*	.	.	.
8	112760559	A	T	intergenic	*LINC01609,CSMD3*	.	.	.
8	112708955	G	T	intergenic	*LINC01609,CSMD3*	.	.	.
8	112634482	GTATA	G	intergenic	*LINC01609,CSMD3*	.	.	.

8	112751211	G	T	intergenic	*LINC01609,CSMD3*	.	.	.
8	112355450	A	ATCTC	intergenic	*LINC01609,CSMD3*	.	.	.
8	112403683	G	C	intergenic	*LINC01609,CSMD3*	.	.	.
8	112508269	T	C	intergenic	*LINC01609,CSMD3*	.	.	.
8	112508890	G	T	intergenic	*LINC01609,CSMD3*	.	.	.
8	112522735	T	C	intergenic	*LINC01609,CSMD3*	.	.	.
8	112522736	G	C	intergenic	*LINC01609,CSMD3*	.	.	.
8	112523083	C	A	intergenic	*LINC01609,CSMD3*	.	.	.
8	112568276	T	C	intergenic	*LINC01609,CSMD3*	.	.	.
8	112569502	A	C	intergenic	*LINC01609,CSMD3*	.	.	.
8	112587524	T	C	intergenic	*LINC01609,CSMD3*	.	.	.
8	112587525	A	T	intergenic	*LINC01609,CSMD3*	.	.	.
8	112647322	T	C	intergenic	*LINC01609,CSMD3*	.	.	.
8	112686404	A	C	intergenic	*LINC01609,CSMD3*	.	.	.
8	112686405	T	C	intergenic	*LINC01609,CSMD3*	.	.	.
8	112722010	G	A	intergenic	*LINC01609,CSMD3*	.	.	.
8	113098140	A	G	intergenic	*LINC01609,CSMD3*	.	.	.
8	113197419	A	G	intergenic	*LINC01609,CSMD3*	.	.	.
8	113224044	T	A	intergenic	*LINC01609,CSMD3*	.	.	.

8	113231654	T	C	intergenic	*LINC01609,CSMD3*	.	.	.
8	113231655	A	T	intergenic	*LINC01609,CSMD3*	.	.	.
8	113232876	A	G	intergenic	*LINC01609,CSMD3*	.	.	.
6	85146286	C	A	ncRNA_intronic	*LINC01611*	.	.	.
6	85184942	C	CTGTT	upstream	*LINC01611*	.	.	.
6	85185375	T	G	upstream	*LINC01611*	.	.	.
6	85275250	G	A	intergenic	*LINC01611,TBX18-AS1*	.	.	.
6	85286110	G	C	intergenic	*LINC01611,TBX18-AS1*	.	.	.
6	85346623	G	A	intergenic	*LINC01611,TBX18-AS1*	.	.	.
6	85294730	A	G	intergenic	*LINC01611,TBX18-AS1*	.	.	.
6	85313696	G	A	intergenic	*LINC01611,TBX18-AS1*	.	.	.
6	85338065	T	A	intergenic	*LINC01611,TBX18-AS1*	.	.	.
6	85339287	C	T	intergenic	*LINC01611,TBX18-AS1*	.	.	.
6	85340842	C	A	intergenic	*LINC01611,TBX18-AS1*	.	.	.
4	171187538	A	G	ncRNA_intronic	*LINC01612*	.	.	.
4	171526511	TCTG	T	intergenic	*LINC01612,LOC100506122*	.	.	.
4	171294812	G	T	intergenic	*LINC01612,LOC100506122*	.	.	.
4	171627565	G	T	intergenic	*LINC01612,LOC100506122*	.	.	.
4	171400448	G	T	intergenic	*LINC01612,LOC100506122*	.	.	.
4	171241759	A	G	intergenic	*LINC01612,LOC100506122*	.	.	.
4	171241761	A	T	intergenic	*LINC01612,LOC100506122*	.	.	.

4	171331541	C	T	intergenic	*LINC01612,LOC100506122*	.	.	.
4	171332644	C	T	intergenic	*LINC01612,LOC100506122*	.	.	.
4	171332646	G	A	intergenic	*LINC01612,LOC100506122*	.	.	.
4	171358033	A	G	intergenic	*LINC01612,LOC100506122*	.	.	.
4	171429320	T	A	intergenic	*LINC01612,LOC100506122*	.	.	.
4	171429321	G	A	intergenic	*LINC01612,LOC100506122*	.	.	.
4	171555707	A	C	intergenic	*LINC01612,LOC100506122*	.	.	.
4	171586745	C	A	intergenic	*LINC01612,LOC100506122*	.	.	.
4	171951026	A	G	intergenic	*LINC01612,LOC100506122*	.	.	.
9	122850364	C	T	intergenic	*LINC01613,MIR147A*	.	.	.
9	122886104	A	T	intergenic	*LINC01613,MIR147A*	.	.	.
9	122886105	G	A	intergenic	*LINC01613,MIR147A*	.	.	.
9	122922136	TTCTCTC	T	intergenic	*LINC01613,MIR147A*	.	.	.
4	53672993	G	C	ncRNA_intronic	*LINC01618*	.	.	.
12	92429347	T	C	ncRNA_intronic	*LINC01619*	.	.	.
12	92410803	T	C	ncRNA_intronic	*LINC01619*	.	.	.
12	92491806	A	G	ncRNA_intronic	*LINC01619*	.	.	.
6	80569210	T	C	intergenic	*LINC01621,ELOVL4*	.	.	.
6	985722	A	G	ncRNA_intronic	*LINC01622*	.	.	.
6	987085	A	G	ncRNA_intronic	*LINC01622*	.	.	.
6	1069089	GTCTC	G	ncRNA_intronic	*LINC01622*	.	.	.
6	1249486	A	G	intergenic	*LINC01622,FOXQ1*	.	.	.

6	139980218	C	A	intergenic	*LINC01625,LOC100132735*	.	.	.
6	139865707	G	T	intergenic	*LINC01625,LOC100132735*	.	.	.
6	139833024	G	A	intergenic	*LINC01625,LOC100132735*	.	.	.
6	139956128	T	TC	intergenic	*LINC01625,LOC100132735*	.	.	.
6	72561790	A	G	intergenic	*LINC01626,RIMS1*	.	.	.
6	72235283	A	AATT	intergenic	*LINC01626,RIMS1*	.	.	.
6	72450253	CGTGTGTGT	C	intergenic	*LINC01626,RIMS1*	.	.	.
6	72526383	C	T	intergenic	*LINC01626,RIMS1*	.	.	.
6	72538096	A	C	intergenic	*LINC01626,RIMS1*	.	.	.
6	72594313	T	C	intergenic	*LINC01626,RIMS1*	.	.	.
3	191358405	A	G	intergenic	*LINCR-0002,FGF12*	.	.	.
5	56883669	G	A	ncRNA_intronic	*LINCR-0003*	.	.	.
5	56883671	A	G	ncRNA_intronic	*LINCR-0003*	.	.	.
5	56929117	AACAC	A	intergenic	*LINCR-0003,LOC101928505*	.	.	.
15	77941016	C	A	ncRNA_exonic	*LINGO1-AS1*	.	.	.
9	28197331	T	A	intronic	*LINGO2*	.	.	.
9	28522491	A	G	intronic	*LINGO2*	.	.	.
9	28221800	T	C	intronic	*LINGO2*	.	.	.
9	28804562	CAAAAA	C	intronic	*LINGO2*	.	.	.
9	28088688	T	C	intronic	*LINGO2*	.	.	.
9	28091918	T	A	intronic	*LINGO2*	.	.	.
9	28092245	G	A	intronic	*LINGO2*	.	.	.
9	28481700	A	C	intronic	*LINGO2*	.	.	.
9	28481701	T	A	intronic	*LINGO2*	.	.	.
9	28495486	T	G	intronic	*LINGO2*	.	.	.
9	28993009	A	G	intronic	*LINGO2*	.	.	.
9	29124022	T	C	intronic	*LINGO2*	.	.	.
9	30196113	T	C	intergenic	*LINGO2,LINC01242*	.	.	.

9	30345069	C	A	intergenic	*LINGO2,LINC01242*	.	.	.
9	29291809	T	C	intergenic	*LINGO2,LINC01242*	.	.	.
9	29734110	G	T	intergenic	*LINGO2,LINC01242*	.	.	.
9	29801604	G	A	intergenic	*LINGO2,LINC01242*	.	.	.
9	29551761	T	C	intergenic	*LINGO2,LINC01242*	.	.	.
9	29924727	G	A	intergenic	*LINGO2,LINC01242*	.	.	.
9	29712471	T	C	intergenic	*LINGO2,LINC01242*	.	.	.
9	29622105	A	G	intergenic	*LINGO2,LINC01242*	.	.	.
9	30031165	C	T	intergenic	*LINGO2,LINC01242*	.	.	.
9	29525714	G	A	intergenic	*LINGO2,LINC01242*	.	.	.
9	29728914	C	T	intergenic	*LINGO2,LINC01242*	.	.	.
9	29891095	G	A	intergenic	*LINGO2,LINC01242*	.	.	.
9	30342376	A	T	intergenic	*LINGO2,LINC01242*	.	.	.
1	151771890	C	A	downstream	*LINGO4*	.	.	.
19	43103407	G	T	ncRNA_intronic	*LIPE-AS1*	.	.	.
19	42972013	G	A	ncRNA_intronic	*LIPE-AS1*	.	.	.
19	43140542	C	G	ncRNA_intronic	*LIPE-AS1*	.	.	.
19	43205951	T	A	intergenic	*LIPE-AS1,PSG3*	.	.	.
19	43221269	T	C	intergenic	*LIPE-AS1,PSG3*	.	.	.
19	43223182	T	C	intergenic	*LIPE-AS1,PSG3*	.	.	.
19	43223435	C	A	intergenic	*LIPE-AS1,PSG3*	.	.	.
18	47205972	A	T	intergenic	*LIPG,ACAA2*	.	.	.
18	47237967	A	G	intergenic	*LIPG,ACAA2*	.	.	.
18	47239342	T	G	intergenic	*LIPG,ACAA2*	.	.	.
18	47245884	G	T	intergenic	*LIPG,ACAA2*	.	.	.

10	90387806	C	T	intergenic	*LIPJ,LIPF*	.	.	.
10	90389447	G	A	intergenic	*LIPJ,LIPF*	.	.	.
10	90395032	C	T	intergenic	*LIPJ,LIPF*	.	.	.
11	74288884	ACG	A	intergenic	*LIPT2,POLD3*	.	.	.
11	74239512	A	G	intergenic	*LIPT2,POLD3*	.	.	.
11	74239514	C	T	intergenic	*LIPT2,POLD3*	.	.	.
16	11711472	G	A	intergenic	*LITAF,SNN*	.	.	.
22	22916240	T	C	intergenic	*LL22NC03-63E9.3,POM121L1P*	.	.	.
22	22926387	T	C	intergenic	*LL22NC03-63E9.3,POM121L1P*	.	.	.
5	176772658	G	A	intronic	*LMAN2*	.	.	.
7	156512378	T	C	intronic	*LMBR1*	.	.	.
7	156596147	C	T	intronic	*LMBR1*	.	.	.
7	156707393	C	T	intergenic	*LMBR1,NOM1*	.	.	.
7	156703648	G	A	intergenic	*LMBR1,NOM1*	.	.	.
7	156705068	T	G	intergenic	*LMBR1,NOM1*	.	.	.
7	156705069	G	T	intergenic	*LMBR1,NOM1*	.	.	.
6	70431783	G	C	intronic	*LMBRD1*	.	.	.
5	36129966	G	T	intronic	*LMBRD2*	.	.	.
3	8577967	A	G	intronic	*LMCD1*	.	.	.
3	8560999	A	G	intronic	*LMCD1*	.	.	.
3	8305604	G	A	ncRNA_intronic	*LMCD1-AS1*	.	.	.
3	8449938	G	A	ncRNA_intronic	*LMCD1-AS1*	.	.	.
3	8482209	T	C	ncRNA_intronic	*LMCD1-AS1*	.	.	.
3	8422429	T	C	ncRNA_intronic	*LMCD1-AS1*	.	.	.
3	8418649	C	G	ncRNA_intronic	*LMCD1-AS1*	.	.	.
16	920555	G	A	intronic	*LMF1*	.	.	.
5	126168211	C	T	intronic	*LMNB1*	.	.	.
5	126168212	T	G	intronic	*LMNB1*	.	.	.
12	25660795	G	C	intronic	*LMNTD1*	.	.	.
12	25692208	T	C	intronic	*LMNTD1*	.	.	.
12	25729027	A	T	intronic	*LMNTD1*	.	.	.
12	25729028	C	T	intronic	*LMNTD1*	.	.	.
12	25729354	C	T	intronic	*LMNTD1*	.	.	.
12	25729735	T	C	intronic	*LMNTD1*	.	.	.

12	25757596	C	T	intronic	*LMNTD1*	.	.	.
12	25757598	T	C	intronic	*LMNTD1*	.	.	.
12	25758799	A	G	intronic	*LMNTD1*	.	.	.
12	25760401	T	A	intronic	*LMNTD1*	.	.	.
12	26017979	T	C	intergenic	*LMNTD1,MIR4302*	.	.	.
12	25814033	T	C	intergenic	*LMNTD1,MIR4302*	.	.	.
12	25814536	G	C	intergenic	*LMNTD1,MIR4302*	.	.	.
12	25815076	C	T	intergenic	*LMNTD1,MIR4302*	.	.	.
12	25816678	C	T	intergenic	*LMNTD1,MIR4302*	.	.	.
12	25817557	C	T	intergenic	*LMNTD1,MIR4302*	.	.	.
11	8257440	C	G	intronic	*LMO1*	.	.	.
12	16886892	T	C	intergenic	*LMO3,SKP1P2*	.	.	.
12	16887043	C	T	intergenic	*LMO3,SKP1P2*	.	.	.
12	16922807	A	G	intergenic	*LMO3,SKP1P2*	.	.	.
12	16922808	C	T	intergenic	*LMO3,SKP1P2*	.	.	.
12	16922972	T	A	intergenic	*LMO3,SKP1P2*	.	.	.
12	17027880	C	T	intergenic	*LMO3,SKP1P2*	.	.	.
13	76357231	T	C	intronic	*LMO7*	.	.	.
13	76303184	G	A	intronic	*LMO7*	.	.	.
13	76896630	TGAGA	T	intergenic	*LMO7DN,KCTD12*	.	.	.
13	76640559	T	C	intergenic	*LMO7DN,KCTD12*	.	.	.
13	76640560	G	A	intergenic	*LMO7DN,KCTD12*	.	.	.
13	76653960	A	G	intergenic	*LMO7DN,KCTD12*	.	.	.
13	76653961	T	C	intergenic	*LMO7DN,KCTD12*	.	.	.
13	76665102	C	T	intergenic	*LMO7DN,KCTD12*	.	.	.
13	76683943	T	C	intergenic	*LMO7DN,KCTD12*	.	.	.
13	76814599	A	C	intergenic	*LMO7DN,KCTD12*	.	.	.
13	76814834	C	T	intergenic	*LMO7DN,KCTD12*	.	.	.
13	76919807	T	A	intergenic	*LMO7DN,KCTD12*	.	.	.
13	77155321	A	C	intergenic	*LMO7DN,KCTD12*	.	.	.
13	77180355	G	A	intergenic	*LMO7DN,KCTD12*	.	.	.
3	69189368	C	CTCCTTCCT	intergenic	*LMOD3,FRMD4B*	.	.	.
3	69199451	G	T	intergenic	*LMOD3,FRMD4B*	.	.	.
19	49038520	A	G	intergenic	*LMTK3,SULT2B1*	.	.	.
1	165266144	C	G	intronic	*LMX1A*	.	.	.
9	129503430	T	C	intergenic	*LMX1B,ZBTB43*	.	.	.
3	100165378	T	G	intronic	*LNP1*	.	.	.
3	100165379	T	C	intronic	*LNP1*	.	.	.

3	100199448	C	T	intergenic	*LNP1,TMEM45A*	.	.	.
5	96300307	T	C	intronic	*LNPEP*	.	.	.
4	54337890	G	A	intronic	*LNX1*	.	.	.
4	54533669	A	G	intergenic	*LNX1-AS2,LOC100506444*	.	.	.
4	54533671	C	T	intergenic	*LNX1-AS2,LOC100506444*	.	.	.
9	94902077	C	A	intergenic	*LOC100128076,LINC00475*	.	.	.
14	65684982	T	A	ncRNA_intronic	*LOC100128233*	.	.	.
14	65771529	A	G	intergenic	*LOC100128233,MIR4708*	.	.	.
7	81276890	A	C	ncRNA_intronic	*LOC100128317*	.	.	.
12	126978024	G	A	intergenic	*LOC100128554,LOC100996671*	.	.	.
12	127013217	C	T	intergenic	*LOC100128554,LOC100996671*	.	.	.
12	127040848	A	G	intergenic	*LOC100128554,LOC100996671*	.	.	.
19	5998703	C	T	ncRNA_intronic	*LOC100128568*	.	.	.
19	6015609	C	A	ncRNA_intronic	*LOC100128568*	.	.	.
15	26299108	T	C	downstream	*LOC100128714*	.	.	.
15	26167714	T	G	ncRNA_intronic	*LOC100128714*	.	.	.
7	64123250	A	G	intergenic	*LOC100128885,ZNF107*	.	.	.
10	38480652	C	T	ncRNA_intronic	*LOC100129055*	.	.	.
10	38480653	C	T	ncRNA_intronic	*LOC100129055*	.	.	.
10	38569774	A	ACAAATAGA	intergenic	*LOC100129055,HSD17B7P2*	.	.	.
10	38509596	T	A	intergenic	*LOC100129055,HSD17B7P2*	.	.	.
1	105022939	G	C	intergenic	*LOC100129138,LOC101928476*	.	.	.
1	104916246	G	A	intergenic	*LOC100129138,LOC101928476*	.	.	.

1	104971372	C	T	intergenic	*LOC100129138,LOC101928476*	.	.	.
1	105202505	T	G	intergenic	*LOC100129138,LOC101928476*	.	.	.
1	105361533	A	G	intergenic	*LOC100129138,LOC101928476*	.	.	.
1	105460478	T	TAA	intergenic	*LOC100129138,LOC101928476*	.	.	.
1	105733378	T	C	intergenic	*LOC100129138,LOC101928476*	.	.	.
1	105806724	T	C	intergenic	*LOC100129138,LOC101928476*	.	.	.
1	105834306	A	G	intergenic	*LOC100129138,LOC101928476*	.	.	.
1	105988992	T	C	intergenic	*LOC100129138,LOC101928476*	.	.	.
1	106121310	C	T	intergenic	*LOC100129138,LOC101928476*	.	.	.
11	94964274	G	C	ncRNA_intronic	*LOC100129203*	.	.	.
11	95201318	A	G	intergenic	*LOC100129203,FAM76B*	.	.	.
11	94972220	AT	A	intergenic	*LOC100129203,FAM76B*	.	.	.
11	95418989	GTTATTA	G	intergenic	*LOC100129203,FAM76B*	.	.	.
11	95285728	T	C	intergenic	*LOC100129203,FAM76B*	.	.	.
11	95133950	T	C	intergenic	*LOC100129203,FAM76B*	.	.	.
11	95142706	C	A	intergenic	*LOC100129203,FAM76B*	.	.	.
11	95142708	G	A	intergenic	*LOC100129203,FAM76B*	.	.	.
11	95143890	T	C	intergenic	*LOC100129203,FAM76B*	.	.	.
11	95143892	T	G	intergenic	*LOC100129203,FAM76B*	.	.	.

9	93895490	A	AAT	intergenic	*LOC100129316,AUH*	.	.	.
14	98258666	A	T	intergenic	*LOC100129345,LINC01550*	.	.	.
14	98219742	A	G	intergenic	*LOC100129345,LINC01550*	.	.	.
14	98364161	C	T	intergenic	*LOC100129345,LINC01550*	.	.	.
14	98206276	T	C	intergenic	*LOC100129345,LINC01550*	.	.	.
14	98211336	C	T	intergenic	*LOC100129345,LINC01550*	.	.	.
14	98211338	A	G	intergenic	*LOC100129345,LINC01550*	.	.	.
14	98283717	T	C	intergenic	*LOC100129345,LINC01550*	.	.	.
2	56401634	G	C	ncRNA_exonic	*LOC100129434*	.	.	.
X	124549040	C	A	intergenic	*LOC100129520,LOC101928495*	.	.	.
X	124632782	A	G	intergenic	*LOC100129520,LOC101928495*	.	.	.
X	124770005	C	T	intergenic	*LOC100129520,LOC101928495*	.	.	.
X	124613822	G	GT	intergenic	*LOC100129520,LOC101928495*	.	.	.
X	125130820	C	A	intergenic	*LOC100129520,LOC101928495*	.	.	.
X	125155529	G	T	intergenic	*LOC100129520,LOC101928495*	.	.	.
X	124498158	G	C	intergenic	*LOC100129520,LOC101928495*	.	.	.
X	124498160	G	C	intergenic	*LOC100129520,LOC101928495*	.	.	.
X	124508889	T	C	intergenic	*LOC100129520,LOC101928495*	.	.	.
X	124940998	A	C	intergenic	*LOC100129520,LOC101928495*	.	.	.

X	124964384	C	A	intergenic	*LOC100129520,LOC101928495*	.	.	.
X	124976761	T	G	intergenic	*LOC100129520,LOC101928495*	.	.	.
X	125051050	T	G	intergenic	*LOC100129520,LOC101928495*	.	.	.
X	125052056	A	G	intergenic	*LOC100129520,LOC101928495*	.	.	.
X	125053129	A	G	intergenic	*LOC100129520,LOC101928495*	.	.	.
X	125094730	C	T	intergenic	*LOC100129520,LOC101928495*	.	.	.
X	125154630	G	GT	intergenic	*LOC100129520,LOC101928495*	.	.	.
X	125155015	G	A	intergenic	*LOC100129520,LOC101928495*	.	.	.
X	125155768	A	G	intergenic	*LOC100129520,LOC101928495*	.	.	.
X	125174467	C	T	intergenic	*LOC100129520,LOC101928495*	.	.	.
X	125240501	C	T	intergenic	*LOC100129520,LOC101928495*	.	.	.
1	99484767	A	G	ncRNA_intronic	*LOC100129620*	.	.	.
4	7046700	C	A	ncRNA_intronic	*LOC100129931*	.	.	.
20	19257780	G	T	ncRNA_intronic	*LOC100130264*	.	.	.
8	61951018	C	CAT	intergenic	*LOC100130298,CLVS1*	.	.	.
8	62182113	A	G	intergenic	*LOC100130298,CLVS1*	.	.	.
8	61958979	T	C	intergenic	*LOC100130298,CLVS1*	.	.	.
8	61959976	A	G	intergenic	*LOC100130298,CLVS1*	.	.	.
8	62029117	C	G	intergenic	*LOC100130298,CLVS1*	.	.	.
8	62029118	A	G	intergenic	*LOC100130298,CLVS1*	.	.	.

8	62053185	G	A	intergenic	*LOC100130298,CLVS1*	.	.	.
8	62092747	G	A	intergenic	*LOC100130298,CLVS1*	.	.	.
8	62134511	G	A	intergenic	*LOC100130298,CLVS1*	.	.	.
1	238044017	C	T	ncRNA_intronic	*LOC100130331*	.	.	.
1	238074838	G	A	ncRNA_intronic	*LOC100130331*	.	.	.
1	238356078	T	C	intergenic	*LOC100130331,LINC01139*	.	.	.
1	238293744	A	T	intergenic	*LOC100130331,LINC01139*	.	.	.
1	238448353	A	C	intergenic	*LOC100130331,LINC01139*	.	.	.
1	238542816	G	T	intergenic	*LOC100130331,LINC01139*	.	.	.
1	238620345	G	T	intergenic	*LOC100130331,LINC01139*	.	.	.
1	238264147	T	C	intergenic	*LOC100130331,LINC01139*	.	.	.
1	238166524	G	C	intergenic	*LOC100130331,LINC01139*	.	.	.
1	238180553	TTCTC	T	intergenic	*LOC100130331,LINC01139*	.	.	.
1	238373207	A	T	intergenic	*LOC100130331,LINC01139*	.	.	.
1	238384776	T	C	intergenic	*LOC100130331,LINC01139*	.	.	.
2	178235001	T	C	ncRNA_intronic	*LOC100130691*	.	.	.
2	178235737	A	G	ncRNA_intronic	*LOC100130691*	.	.	.
8	39422268	T	G	ncRNA_intronic	*LOC100130964*	.	.	.
6	38917582	A	G	ncRNA_intronic	*LOC100131047*	.	.	.
7	7164880	G	A	intergenic	*LOC100131257,C1GALT1*	.	.	.
7	7179939	G	T	intergenic	*LOC100131257,C1GALT1*	.	.	.
7	7179941	A	T	intergenic	*LOC100131257,C1GALT1*	.	.	.

7	7182352	C	G	intergenic	*LOC100131257,C1GALT1*	.	.	.
7	7182823	G	C	intergenic	*LOC100131257,C1GALT1*	.	.	.
7	7182825	T	C	intergenic	*LOC100131257,C1GALT1*	.	.	.
7	7183815	A	G	intergenic	*LOC100131257,C1GALT1*	.	.	.
9	97135156	C	G	intergenic	*LOC100132077,MFSD14B*	.	.	.
11	111304115	C	T	intergenic	*LOC100132078,BTG4*	.	.	.
11	111304117	A	C	intergenic	*LOC100132078,BTG4*	.	.	.
17	75738544	A	G	intergenic	*LOC100132174,FLJ45079*	.	.	.
17	75845477	C	T	intergenic	*LOC100132174,FLJ45079*	.	.	.
6	140135015	G	A	ncRNA_intronic	*LOC100132735*	.	.	.
6	140195314	T	A	intergenic	*LOC100132735,LOC100507477*	.	.	.
6	140293123	T	C	intergenic	*LOC100132735,LOC100507477*	.	.	.
9	99655921	T	A	intergenic	*LOC100132781,LOC441454*	.	.	.
X	40871554	G	T	intergenic	*LOC100132831,USP9X*	.	.	.
5	99716974	C	T	ncRNA_exonic	*LOC100133050*	.	.	.
5	99845945	T	C	intergenic	*LOC100133050,FAM174A*	.	.	.
5	99730887	A	G	intergenic	*LOC100133050,FAM174A*	.	.	.
5	99794940	T	C	intergenic	*LOC100133050,FAM174A*	.	.	.
5	99839900	A	C	intergenic	*LOC100133050,FAM174A*	.	.	.
5	99842689	C	G	intergenic	*LOC100133050,FAM174A*	.	.	.

5	99849996	A	G	intergenic	*LOC100133050,FAM174A*	.	.	.
7	76192352	C	T	ncRNA_intronic	*LOC100133091*	.	.	.
7	76587611	T	TTTTG	intergenic	*LOC100133091,DTX2P1-UPK3BP1-PMS2P11*	.	.	.
11	71598429	A	G	ncRNA_intronic	*LOC100133315*	.	.	.
1	692424	C	T	intergenic	*LOC100133331,LOC100288069*	.	.	.
1	695007	T	C	intergenic	*LOC100133331,LOC100288069*	.	.	.
8	144090712	A	T	ncRNA_intronic	*LOC100133669*	.	.	.
9	69737653	A	AATG	intergenic	*LOC100133920,LOC101928381*	.	.	.
9	69690180	G	T	intergenic	*LOC100133920,LOC101928381*	.	.	.
2	155347748	C	T	intergenic	*LOC100144595,KCNJ3*	.	.	.
2	155538258	T	C	intergenic	*LOC100144595,KCNJ3*	.	.	.
2	155332586	G	GAGA	intergenic	*LOC100144595,KCNJ3*	.	.	.
2	155332587	T	TCGG	intergenic	*LOC100144595,KCNJ3*	.	.	.
1	165750015	A	G	intergenic	*LOC100147773,UCK2*	.	.	.
2	105010762	A	T	ncRNA_intronic	*LOC100287010*	.	.	.
2	105014510	C	T	ncRNA_intronic	*LOC100287010*	.	.	.
2	105014511	A	G	ncRNA_intronic	*LOC100287010*	.	.	.
2	105045346	A	G	intergenic	*LOC100287010,LINC01102*	.	.	.
18	49396270	C	T	intergenic	*LOC100287225,DCC*	.	.	.
18	49707443	C	A	intergenic	*LOC100287225,DCC*	.	.	.
18	49710679	G	C	intergenic	*LOC100287225,DCC*	.	.	.

18	49622206	C	T	intergenic	*LOC100287225,DCC*	.	.	.
18	49732107	TTTGTTG	T	intergenic	*LOC100287225,DCC*	.	.	.
18	49496536	A	G	intergenic	*LOC100287225,DCC*	.	.	.
18	49620890	C	G	intergenic	*LOC100287225,DCC*	.	.	.
18	49620892	G	A	intergenic	*LOC100287225,DCC*	.	.	.
18	49621200	T	C	intergenic	*LOC100287225,DCC*	.	.	.
18	49833469	G	A	intergenic	*LOC100287225,DCC*	.	.	.
18	49833470	A	C	intergenic	*LOC100287225,DCC*	.	.	.
5	50575512	T	C	intergenic	*LOC100287592,LOC642366*	.	.	.
5	50438693	T	A	intergenic	*LOC100287592,LOC642366*	.	.	.
5	50646067	G	T	intergenic	*LOC100287592,LOC642366*	.	.	.
5	50646905	A	C	intergenic	*LOC100287592,LOC642366*	.	.	.
5	50648940	C	T	intergenic	*LOC100287592,LOC642366*	.	.	.
5	50648941	A	G	intergenic	*LOC100287592,LOC642366*	.	.	.
7	62970802	G	C	intergenic	*LOC100287834,MIR4283-2*	.	.	.
7	62942203	C	T	intergenic	*LOC100287834,MIR4283-2*	.	.	.
7	63041222	C	T	intergenic	*LOC100287834,MIR4283-2*	.	.	.
8	48148007	A	T	intergenic	*LOC100287846,SPIDR*	.	.	.
12	107144290	T	A	ncRNA_intronic	*LOC100287944*	.	.	.
12	106943090	A	G	ncRNA_intronic	*LOC100287944*	.	.	.

12	106943159	T	C	ncRNA_intronic	*LOC100287944*	.	.	.
12	106943161	A	G	ncRNA_intronic	*LOC100287944*	.	.	.
1	735147	T	G	intergenic	*LOC100288069,FAM87B*	.	.	.
16	18448286	T	C	intergenic	*LOC100288162,MIR6770-2*	.	.	.
16	18464096	A	G	intergenic	*LOC100288162,MIR6770-2*	.	.	.
16	18464312	A	G	intergenic	*LOC100288162,MIR6770-2*	.	.	.
15	30939821	G	C	ncRNA_intronic	*LOC100288637*	.	.	.
15	31097297	A	G	intergenic	*LOC100288637,HERC2P10*	.	.	.
12	46870893	G	GTTTC	ncRNA_intronic	*LOC100288798*	.	.	.
12	46818767	C	G	ncRNA_intronic	*LOC100288798*	.	.	.
12	46975551	G	T	ncRNA_intronic	*LOC100288798*	.	.	.
12	46976864	C	G	ncRNA_intronic	*LOC100288798*	.	.	.
12	46979260	C	T	ncRNA_intronic	*LOC100288798*	.	.	.
12	46983129	A	T	ncRNA_intronic	*LOC100288798*	.	.	.
12	46983130	A	G	ncRNA_intronic	*LOC100288798*	.	.	.
12	46991314	T	C	ncRNA_intronic	*LOC100288798*	.	.	.
12	47142390	G	T	intergenic	*LOC100288798,SLC38A4*	.	.	.
5	98471915	G	GTCTGTCTA	intergenic	*LOC100289230,CTD-2151A2.1*	.	.	.
5	98685060	T	A	intergenic	*LOC100289230,CTD-2151A2.1*	.	.	.
5	98509865	G	A	intergenic	*LOC100289230,CTD-2151A2.1*	.	.	.
5	98785209	A	T	intergenic	*LOC100289230,CTD-2151A2.1*	.	.	.
5	98783238	C	T	intergenic	*LOC100289230,CTD-2151A2.1*	.	.	.
5	98376297	T	C	intergenic	*LOC100289230,CTD-2151A2.1*	.	.	.
5	98376298	G	A	intergenic	*LOC100289230,CTD-2151A2.1*	.	.	.

5	98379149	A	G	intergenic	*LOC100289230,CTD-2151A2.1*	.	.	.
5	98494098	C	A	intergenic	*LOC100289230,CTD-2151A2.1*	.	.	.
5	98494171	A	C	intergenic	*LOC100289230,CTD-2151A2.1*	.	.	.
5	98494172	G	A	intergenic	*LOC100289230,CTD-2151A2.1*	.	.	.
5	98519272	T	C	intergenic	*LOC100289230,CTD-2151A2.1*	.	.	.
5	98519273	G	A	intergenic	*LOC100289230,CTD-2151A2.1*	.	.	.
5	98519884	T	G	intergenic	*LOC100289230,CTD-2151A2.1*	.	.	.
5	98626334	C	T	intergenic	*LOC100289230,CTD-2151A2.1*	.	.	.
5	98633197	A	G	intergenic	*LOC100289230,CTD-2151A2.1*	.	.	.
5	98675658	C	T	intergenic	*LOC100289230,CTD-2151A2.1*	.	.	.
5	98678174	G	A	intergenic	*LOC100289230,CTD-2151A2.1*	.	.	.
5	98791699	T	C	intergenic	*LOC100289230,CTD-2151A2.1*	.	.	.
5	109528255	C	T	intergenic	*LOC100289673,TMEM232*	.	.	.
5	109377577	T	TTGTG	intergenic	*LOC100289673,TMEM232*	.	.	.
5	109377578	AGCAG	A	intergenic	*LOC100289673,TMEM232*	.	.	.
5	109467302	A	T	intergenic	*LOC100289673,TMEM232*	.	.	.
5	109573595	T	A	intergenic	*LOC100289673,TMEM232*	.	.	.
5	109577361	A	G	intergenic	*LOC100289673,TMEM232*	.	.	.
5	109616583	C	CA	intergenic	*LOC100289673,TMEM232*	.	.	.

5	109617087	G	A	intergenic	*LOC100289673,TMEM232*	.	.	.
19	28981605	C	G	ncRNA_intronic	*LOC100420587*	.	.	.
19	29117120	A	G	ncRNA_intronic	*LOC100420587*	.	.	.
19	29117122	T	C	ncRNA_intronic	*LOC100420587*	.	.	.
19	29169707	C	T	ncRNA_intronic	*LOC100420587*	.	.	.
19	29174510	C	T	ncRNA_intronic	*LOC100420587*	.	.	.
19	29390138	C	A	intergenic	*LOC100420587,LINC00906*	.	.	.
19	29252998	G	T	intergenic	*LOC100420587,LINC00906*	.	.	.
19	29282053	C	A	intergenic	*LOC100420587,LINC00906*	.	.	.
19	29426674	G	T	intergenic	*LOC100420587,LINC00906*	.	.	.
19	29424251	G	T	intergenic	*LOC100420587,LINC00906*	.	.	.
19	29336110	C	G	intergenic	*LOC100420587,LINC00906*	.	.	.
19	29381085	G	T	intergenic	*LOC100420587,LINC00906*	.	.	.
19	29252867	T	A	intergenic	*LOC100420587,LINC00906*	.	.	.
19	29259293	A	C	intergenic	*LOC100420587,LINC00906*	.	.	.
19	29297778	C	T	intergenic	*LOC100420587,LINC00906*	.	.	.
X	55308880	T	C	ncRNA_intronic	*LOC100421746*	.	.	.
X	55318453	C	G	intergenic	*LOC100421746,MIR4536-2*	.	.	.
X	55318313	T	A	intergenic	*LOC100421746,MIR4536-2*	.	.	.
X	55324464	T	C	intergenic	*LOC100421746,MIR4536-2*	.	.	.
X	55345187	A	T	intergenic	*LOC100421746,MIR4536-2*	.	.	.
X	55345189	T	C	intergenic	*LOC100421746,MIR4536-2*	.	.	.

X	55347061	A	G	intergenic	*LOC100421746,MIR4536-2*	.	.	.
X	55347668	T	A	intergenic	*LOC100421746,MIR4536-2*	.	.	.
X	55347979	T	C	intergenic	*LOC100421746,MIR4536-2*	.	.	.
X	55348518	C	T	intergenic	*LOC100421746,MIR4536-2*	.	.	.
X	55373263	C	CT	intergenic	*LOC100421746,MIR4536-2*	.	.	.
X	55373518	G	T	intergenic	*LOC100421746,MIR4536-2*	.	.	.
X	55387990	G	A	intergenic	*LOC100421746,MIR4536-2*	.	.	.
X	55428395	T	C	intergenic	*LOC100421746,MIR4536-2*	.	.	.
X	55428697	T	A	intergenic	*LOC100421746,MIR4536-2*	.	.	.
X	55457357	T	C	intergenic	*LOC100421746,MIR4536-2*	.	.	.
1	164441302	CCTCT	C	intergenic	*LOC100422212,PBX1*	.	.	.
1	164123150	C	G	intergenic	*LOC100422212,PBX1*	.	.	.
1	163945059	G	T	intergenic	*LOC100422212,PBX1*	.	.	.
1	163450681	C	T	intergenic	*LOC100422212,PBX1*	.	.	.
1	163400357	T	G	intergenic	*LOC100422212,PBX1*	.	.	.
1	164369315	A	G	intergenic	*LOC100422212,PBX1*	.	.	.
1	163734594	G	A	intergenic	*LOC100422212,PBX1*	.	.	.
1	163729906	C	T	intergenic	*LOC100422212,PBX1*	.	.	.
1	163819177	C	T	intergenic	*LOC100422212,PBX1*	.	.	.

1	163819988	T	C	intergenic	*LOC100422212,PBX1*	.	.	.
1	163821536	C	T	intergenic	*LOC100422212,PBX1*	.	.	.
1	163872984	G	A	intergenic	*LOC100422212,PBX1*	.	.	.
1	163961381	C	A	intergenic	*LOC100422212,PBX1*	.	.	.
1	163962809	C	T	intergenic	*LOC100422212,PBX1*	.	.	.
1	163963985	G	C	intergenic	*LOC100422212,PBX1*	.	.	.
1	163970722	T	C	intergenic	*LOC100422212,PBX1*	.	.	.
1	163974677	G	A	intergenic	*LOC100422212,PBX1*	.	.	.
1	164019044	CACACACACACACATATAT	C	intergenic	*LOC100422212,PBX1*	.	.	.
1	164086964	C	T	intergenic	*LOC100422212,PBX1*	.	.	.
1	164092291	C	T	intergenic	*LOC100422212,PBX1*	.	.	.
6	107180345	C	G	ncRNA_intronic	*LOC100422737*	.	.	.
9	100098176	T	A	ncRNA_intronic	*LOC100499484-C9ORF174*	.	.	.
9	100099570	C	T	ncRNA_intronic	*LOC100499484-C9ORF174*	.	.	.
10	22797682	G	C	intergenic	*LOC100499489,PIP4K2A*	.	.	.
10	22813177	T	G	intergenic	*LOC100499489,PIP4K2A*	.	.	.
3	180205685	T	G	intergenic	*LOC100505609,TTC14*	.	.	.
18	70998435	A	G	ncRNA_intronic	*LOC100505817*	.	.	.
18	71342148	T	G	intergenic	*LOC100505817,FBXO15*	.	.	.

18	71099428	C	A	intergenic	*LOC100505817,FBXO15*	.	.	.
18	71448066	G	T	intergenic	*LOC100505817,FBXO15*	.	.	.
18	71451155	T	C	intergenic	*LOC100505817,FBXO15*	.	.	.
18	71711534	A	G	intergenic	*LOC100505817,FBXO15*	.	.	.
18	71725189	C	T	intergenic	*LOC100505817,FBXO15*	.	.	.
18	71725929	G	A	intergenic	*LOC100505817,FBXO15*	.	.	.
5	121514068	C	T	intronic	*LOC100505841*	.	.	.
5	121514069	T	G	intronic	*LOC100505841*	.	.	.
5	121586286	A	G	intergenic	*LOC100505841,SNCAIP*	.	.	.
5	121611980	G	T	intergenic	*LOC100505841,SNCAIP*	.	.	.
5	86344295	T	A	intergenic	*LOC100505878,MIR4280*	.	.	.
5	86071019	G	C	intergenic	*LOC100505878,MIR4280*	.	.	.
5	86071021	T	C	intergenic	*LOC100505878,MIR4280*	.	.	.
5	86071351	G	A	intergenic	*LOC100505878,MIR4280*	.	.	.
5	86283733	A	C	intergenic	*LOC100505878,MIR4280*	.	.	.
5	86314163	G	A	intergenic	*LOC100505878,MIR4280*	.	.	.
5	86320990	T	G	intergenic	*LOC100505878,MIR4280*	.	.	.
5	86384575	T	C	intergenic	*LOC100505878,MIR4280*	.	.	.
4	22337668	C	T	ncRNA_intronic	*LOC100505912*	.	.	.
4	22355626	A	G	intergenic	*LOC100505912,ADGRA3*	.	.	.
16	15196986	T	A	ncRNA_intronic	*LOC100505915*	.	.	.

7	8393533	C	T	intergenic	*LOC100505938,NXPH1*	.	.	.
1	173203621	G	A	downstream	*LOC100506023*	.	.	.
1	173204048	G	A	downstream	*LOC100506023*	.	.	.
1	173241105	G	A	ncRNA_intronic	*LOC100506023*	.	.	.
1	173290505	C	A	ncRNA_intronic	*LOC100506023*	.	.	.
1	173294651	A	G	ncRNA_intronic	*LOC100506023*	.	.	.
11	4226852	C	T	intergenic	*LOC100506082,OR52B4*	.	.	.
11	4322223	C	A	intergenic	*LOC100506082,OR52B4*	.	.	.
11	4373555	C	T	intergenic	*LOC100506082,OR52B4*	.	.	.
11	4373556	A	G	intergenic	*LOC100506082,OR52B4*	.	.	.
11	4373820	CA	C	intergenic	*LOC100506082,OR52B4*	.	.	.
11	4374615	G	T	intergenic	*LOC100506082,OR52B4*	.	.	.
4	171968214	T	C	ncRNA_intronic	*LOC100506122*	.	.	.
11	76143266	C	A	intergenic	*LOC100506127,EMSY*	.	.	.
7	96311194	T	C	intergenic	*LOC100506136,SHFM1*	.	.	.
6	57133509	T	C	intergenic	*LOC100506188,PRIM2*	.	.	.
6	57137481	C	T	intergenic	*LOC100506188,PRIM2*	.	.	.
6	57150960	T	C	intergenic	*LOC100506188,PRIM2*	.	.	.
6	8504509	G	A	ncRNA_intronic	*LOC100506207*	.	.	.
6	8445398	T	C	ncRNA_intronic	*LOC100506207*	.	.	.
6	8489858	C	T	ncRNA_intronic	*LOC100506207*	.	.	.
6	8490394	T	G	ncRNA_intronic	*LOC100506207*	.	.	.
6	8497713	T	A	ncRNA_intronic	*LOC100506207*	.	.	.
6	8497715	T	G	ncRNA_intronic	*LOC100506207*	.	.	.
6	8585504	C	T	ncRNA_intronic	*LOC100506207*	.	.	.
6	8585571	C	T	ncRNA_intronic	*LOC100506207*	.	.	.

6	8615541	A	G	ncRNA_intronic	*LOC100506207*	.	.	.
6	8615543	T	G	ncRNA_intronic	*LOC100506207*	.	.	.
6	8616695	A	G	ncRNA_intronic	*LOC100506207*	.	.	.
6	10202510	C	T	intergenic	*LOC100506207,TFAP2A*	.	.	.
6	8850696	T	C	intergenic	*LOC100506207,TFAP2A*	.	.	.
6	8850697	G	A	intergenic	*LOC100506207,TFAP2A*	.	.	.
6	9114780	A	T	intergenic	*LOC100506207,TFAP2A*	.	.	.
6	9216939	C	A	intergenic	*LOC100506207,TFAP2A*	.	.	.
6	9294735	C	T	intergenic	*LOC100506207,TFAP2A*	.	.	.
6	9445690	T	C	intergenic	*LOC100506207,TFAP2A*	.	.	.
6	9445692	A	G	intergenic	*LOC100506207,TFAP2A*	.	.	.
6	9445859	T	A	intergenic	*LOC100506207,TFAP2A*	.	.	.
6	9447244	T	C	intergenic	*LOC100506207,TFAP2A*	.	.	.
6	9447245	G	A	intergenic	*LOC100506207,TFAP2A*	.	.	.
6	9447403	G	A	intergenic	*LOC100506207,TFAP2A*	.	.	.
6	9448474	G	A	intergenic	*LOC100506207,TFAP2A*	.	.	.
6	9448475	A	C	intergenic	*LOC100506207,TFAP2A*	.	.	.
6	9449598	T	C	intergenic	*LOC100506207,TFAP2A*	.	.	.
6	9513020	T	C	intergenic	*LOC100506207,TFAP2A*	.	.	.
6	9572777	A	G	intergenic	*LOC100506207,TFAP2A*	.	.	.

6	9755377	C	T	intergenic	*LOC100506207,TFAP2A*	.	.	.
6	9885360	C	T	intergenic	*LOC100506207,TFAP2A*	.	.	.
6	10108410	T	C	intergenic	*LOC100506207,TFAP2A*	.	.	.
6	10108412	T	C	intergenic	*LOC100506207,TFAP2A*	.	.	.
6	10138596	T	A	intergenic	*LOC100506207,TFAP2A*	.	.	.
6	10334880	G	A	intergenic	*LOC100506207,TFAP2A*	.	.	.
4	188772987	T	C	intergenic	*LOC100506272,ZFP42*	.	.	.
4	188759840	A	G	intergenic	*LOC100506272,ZFP42*	.	.	.
4	188765139	T	C	intergenic	*LOC100506272,ZFP42*	.	.	.
4	188767798	T	C	intergenic	*LOC100506272,ZFP42*	.	.	.
4	188779469	G	A	intergenic	*LOC100506272,ZFP42*	.	.	.
4	188780722	A	T	intergenic	*LOC100506272,ZFP42*	.	.	.
4	188794794	G	A	intergenic	*LOC100506272,ZFP42*	.	.	.
4	188845862	G	A	intergenic	*LOC100506272,ZFP42*	.	.	.
2	7901825	C	A	intergenic	*LOC100506274,LOC101929551*	.	.	.
2	7901826	T	A	intergenic	*LOC100506274,LOC101929551*	.	.	.
2	7742404	A	G	intergenic	*LOC100506274,LOC101929551*	.	.	.
2	7623701	T	C	intergenic	*LOC100506274,LOC101929551*	.	.	.
2	7623702	G	A	intergenic	*LOC100506274,LOC101929551*	.	.	.

2	7687770	A	T	intergenic	*LOC100506274,LOC101929551*	.	.	.
2	7918402	G	A	intergenic	*LOC100506274,LOC101929551*	.	.	.
11	86681745	A	G	ncRNA_intronic	*LOC100506368*	.	.	.
11	86673775	T	C	ncRNA_intronic	*LOC100506368*	.	.	.
11	86675001	G	A	ncRNA_intronic	*LOC100506368*	.	.	.
11	86675483	G	A	ncRNA_intronic	*LOC100506368*	.	.	.
11	86676611	A	G	ncRNA_intronic	*LOC100506368*	.	.	.
12	20186387	T	A	ncRNA_intronic	*LOC100506393*	.	.	.
12	20214119	C	T	ncRNA_intronic	*LOC100506393*	.	.	.
12	20224167	T	C	ncRNA_intronic	*LOC100506393*	.	.	.
12	20224451	C	T	ncRNA_intronic	*LOC100506393*	.	.	.
12	20322944	A	G	intergenic	*LOC100506393,PDE3A*	.	.	.
12	20322863	A	G	intergenic	*LOC100506393,PDE3A*	.	.	.
12	20484738	G	A	intergenic	*LOC100506393,PDE3A*	.	.	.
12	20516009	C	T	intergenic	*LOC100506393,PDE3A*	.	.	.
21	36759873	G	A	ncRNA_intronic	*LOC100506403*	.	.	.
21	36790281	A	G	ncRNA_intronic	*LOC100506403*	.	.	.
21	36790282	T	C	ncRNA_intronic	*LOC100506403*	.	.	.
21	36870084	C	T	ncRNA_intronic	*LOC100506403*	.	.	.
21	36975301	ATGGCGG	A	intergenic	*LOC100506403,MIR802*	.	.	.
21	36982466	A	G	intergenic	*LOC100506403,MIR802*	.	.	.
9	26524598	G	A	intergenic	*LOC100506422,CAAP1*	.	.	.
9	26560181	T	A	intergenic	*LOC100506422,CAAP1*	.	.	.
9	26370754	A	T	intergenic	*LOC100506422,CAAP1*	.	.	.
9	26349914	C	A	intergenic	*LOC100506422,CAAP1*	.	.	.

9	26350670	A	T	intergenic	*LOC100506422,CAAP1*	.	.	.
9	26350672	G	GTTTCATTTA	intergenic	*LOC100506422,CAAP1*	.	.	.
9	26480700	C	T	intergenic	*LOC100506422,CAAP1*	.	.	.
9	26558709	A	G	intergenic	*LOC100506422,CAAP1*	.	.	.
9	26558971	C	T	intergenic	*LOC100506422,CAAP1*	.	.	.
9	26588348	G	A	intergenic	*LOC100506422,CAAP1*	.	.	.
9	26837130	C	A	intergenic	*LOC100506422,CAAP1*	.	.	.
4	54591531	G	A	ncRNA_intronic	*LOC100506444*	.	.	.
4	54691161	C	A	intergenic	*LOC100506444,RPL21P44*	.	.	.
4	54702997	T	C	intergenic	*LOC100506444,RPL21P44*	.	.	.
4	54704293	C	T	intergenic	*LOC100506444,RPL21P44*	.	.	.
2	14089171	C	A	intergenic	*LOC100506474,LINC00276*	.	.	.
2	14161844	G	T	intergenic	*LOC100506474,LINC00276*	.	.	.
2	14194359	G	T	intergenic	*LOC100506474,LINC00276*	.	.	.
2	13714849	G	T	intergenic	*LOC100506474,LINC00276*	.	.	.

2	14143117	TATATATAT ATACACAT ACATATGT ATATATACA CACATATAT ATGTGTAT ATATACATA TGTATGTG TATATATAT ATATAC	T	intergenic	*LOC100506474,LINC00276*	.	.	.
2	13241636	C	CGT	intergenic	*LOC100506474,LINC00276*	.	.	.
2	13451980	C	A	intergenic	*LOC100506474,LINC00276*	.	.	.
2	13526307	C	T	intergenic	*LOC100506474,LINC00276*	.	.	.
2	13691188	G	C	intergenic	*LOC100506474,LINC00276*	.	.	.
2	13805341	A	G	intergenic	*LOC100506474,LINC00276*	.	.	.
2	13808920	A	T	intergenic	*LOC100506474,LINC00276*	.	.	.
2	14097381	TTCTC	T	intergenic	*LOC100506474,LINC00276*	.	.	.
2	14143098	TTATATATA TATATATGT ATATATATA TATACACAT ACATATGT ATATATACA CACATATAT ATGTGTAT ATATACATA TGTATGTG TA	T	intergenic	*LOC100506474,LINC00276*	.	.	.
2	13613238	G	A	intergenic	*LOC100506474,LINC00276*	.	.	.

2	13377334	T	C	intergenic	*LOC100506474,LINC00276*	.	.	.
2	13413572	C	T	intergenic	*LOC100506474,LINC00276*	.	.	.
2	13451874	C	T	intergenic	*LOC100506474,LINC00276*	.	.	.
2	13563678	C	T	intergenic	*LOC100506474,LINC00276*	.	.	.
2	13564727	A	C	intergenic	*LOC100506474,LINC00276*	.	.	.
2	13826002	C	CTCTCTA	intergenic	*LOC100506474,LINC00276*	.	.	.
2	13858868	C	T	intergenic	*LOC100506474,LINC00276*	.	.	.
2	13859943	T	A	intergenic	*LOC100506474,LINC00276*	.	.	.
2	13860319	C	A	intergenic	*LOC100506474,LINC00276*	.	.	.
2	13888523	T	A	intergenic	*LOC100506474,LINC00276*	.	.	.
2	13901467	C	T	intergenic	*LOC100506474,LINC00276*	.	.	.
2	14007429	G	A	intergenic	*LOC100506474,LINC00276*	.	.	.
2	14007575	G	A	intergenic	*LOC100506474,LINC00276*	.	.	.
2	14135728	C	A	intergenic	*LOC100506474,LINC00276*	.	.	.
2	14135729	A	G	intergenic	*LOC100506474,LINC00276*	.	.	.
2	14242037	T	C	intergenic	*LOC100506474,LINC00276*	.	.	.
2	14345960	G	A	intergenic	*LOC100506474,LINC00276*	.	.	.
2	14346310	T	A	intergenic	*LOC100506474,LINC00276*	.	.	.
14	74315362	C	T	intergenic	*LOC100506476,PTGR2*	.	.	.

5	61056750	T	C	intergenic	*LOC100506526,KIF2A*	.	.	.
5	61154314	A	G	intergenic	*LOC100506526,KIF2A*	.	.	.
5	61154316	T	G	intergenic	*LOC100506526,KIF2A*	.	.	.
5	61187270	C	T	intergenic	*LOC100506526,KIF2A*	.	.	.
5	61214126	T	C	intergenic	*LOC100506526,KIF2A*	.	.	.
5	61319701	T	C	intergenic	*LOC100506526,KIF2A*	.	.	.
5	61323561	G	A	intergenic	*LOC100506526,KIF2A*	.	.	.
5	61328882	T	G	intergenic	*LOC100506526,KIF2A*	.	.	.
5	61333556	T	TAGATC	intergenic	*LOC100506526,KIF2A*	.	.	.
5	61524067	G	T	intergenic	*LOC100506526,KIF2A*	.	.	.
5	61542598	T	A	intergenic	*LOC100506526,KIF2A*	.	.	.
5	61590805	C	T	intergenic	*LOC100506526,KIF2A*	.	.	.
7	127130109	C	T	intergenic	*LOC100506682,GCC1*	.	.	.
7	127130600	G	C	intergenic	*LOC100506682,GCC1*	.	.	.
7	127131399	T	C	intergenic	*LOC100506682,GCC1*	.	.	.
7	127153553	TGCCGCCGCC	T	intergenic	*LOC100506682,GCC1*	.	.	.
7	35780167	G	A	intergenic	*LOC100506725,SEPT7-AS1*	.	.	.
7	35783120	A	G	intergenic	*LOC100506725,SEPT7-AS1*	.	.	.
7	35783121	C	T	intergenic	*LOC100506725,SEPT7-AS1*	.	.	.

X	134548292	T	G	intergenic	*LOC100506790,SMIM10L2A*	.	.	.
5	2705798	G	T	intergenic	*LOC100506858,IRX2*	.	.	.
5	2330594	T	C	intergenic	*LOC100506858,IRX2*	.	.	.
5	2357477	G	T	intergenic	*LOC100506858,IRX2*	.	.	.
5	2419311	A	T	intergenic	*LOC100506858,IRX2*	.	.	.
5	2483887	G	A	intergenic	*LOC100506858,IRX2*	.	.	.
5	2319069	A	G	intergenic	*LOC100506858,IRX2*	.	.	.
12	59187042	A	G	ncRNA_intronic	*LOC100506869*	.	.	.
12	59177888	T	G	ncRNA_intronic	*LOC100506869*	.	.	.
12	59181649	A	T	ncRNA_intronic	*LOC100506869*	.	.	.
12	59193291	A	G	ncRNA_intronic	*LOC100506869*	.	.	.
12	58992793	C	G	ncRNA_intronic	*LOC100506869,LOC101927653*	.	.	.
12	59071044	G	A	ncRNA_intronic	*LOC100506869,LOC101927653*	.	.	.
12	59101567	G	A	ncRNA_intronic	*LOC100506869,LOC101927653*	.	.	.
12	59142490	A	G	ncRNA_intronic	*LOC100506869,LOC101927653*	.	.	.
12	59232557	T	A	intergenic	*LOC100506869,LRIG3*	.	.	.
12	59232329	A	C	intergenic	*LOC100506869,LRIG3*	.	.	.
12	59233595	C	T	intergenic	*LOC100506869,LRIG3*	.	.	.
8	12365028	C	T	ncRNA_intronic	*LOC100506990*	.	.	.
8	12418337	ATG	A	ncRNA_intronic	*LOC100506990,LOC729732*	.	.	.
8	12410133	G	A	ncRNA_intronic	*LOC100506990,LOC729732*	.	.	.
4	100178344	G	T	ncRNA_intronic	*LOC100507053*	.	.	.

4	100039041	T	C	ncRNA_intronic	*LOC100507053*	.	.	.
4	100112822	T	A	ncRNA_intronic	*LOC100507053*	.	.	.
12	65927139	ACTCT	A	ncRNA_intronic	*LOC100507065*	.	.	.
12	66095309	AATATAT	A	intergenic	*LOC100507065,RPSAP52*	.	.	.
2	66922431	A	G	ncRNA_exonic	*LOC100507073*	.	.	.
2	66838215	A	G	ncRNA_intronic	*LOC100507073*	.	.	.
2	66851157	T	C	ncRNA_intronic	*LOC100507073*	.	.	.
2	66851425	C	T	ncRNA_intronic	*LOC100507073*	.	.	.
2	66854474	C	T	ncRNA_intronic	*LOC100507073*	.	.	.
2	66856991	C	T	ncRNA_intronic	*LOC100507073*	.	.	.
2	66857305	G	A	ncRNA_intronic	*LOC100507073*	.	.	.
2	66857489	G	C	ncRNA_intronic	*LOC100507073*	.	.	.
2	66871591	G	A	ncRNA_intronic	*LOC100507073*	.	.	.
2	66873753	G	A	ncRNA_intronic	*LOC100507073*	.	.	.
8	23205476	T	A	ncRNA_intronic	*LOC100507156*	.	.	.
6	3198813	C	T	intergenic	*LOC100507194,TUBB2B*	.	.	.
12	68929599	C	A	intergenic	*LOC100507195,RAP1B*	.	.	.
2	82262758	C	A	intergenic	*LOC100507201,LOC1720*	.	.	.
2	82838546	T	C	intergenic	*LOC100507201,LOC1720*	.	.	.
2	82069546	C	A	intergenic	*LOC100507201,LOC1720*	.	.	.
2	82242898	GT	G	intergenic	*LOC100507201,LOC1720*	.	.	.
2	82362040	T	C	intergenic	*LOC100507201,LOC1720*	.	.	.
2	82295785	T	C	intergenic	*LOC100507201,LOC1720*	.	.	.
2	82522004	A	T	intergenic	*LOC100507201,LOC1720*	.	.	.
2	82414050	C	CATGTATAC,	intergenic	*LOC100507201,LOC1720*	.	.	.
2	82261883	A	T	intergenic	*LOC100507201,LOC1720*	.	.	.

2	82418407	T	A	intergenic	*LOC100507201,LOC1720*	.	.	.
2	82142293	C	A	intergenic	*LOC100507201,LOC1720*	.	.	.
2	81714826	G	C	intergenic	*LOC100507201,LOC1720*	.	.	.
2	81784480	C	T	intergenic	*LOC100507201,LOC1720*	.	.	.
2	81784481	G	C	intergenic	*LOC100507201,LOC1720*	.	.	.
2	81865633	C	A	intergenic	*LOC100507201,LOC1720*	.	.	.
2	81866432	C	T	intergenic	*LOC100507201,LOC1720*	.	.	.
2	81866433	T	G	intergenic	*LOC100507201,LOC1720*	.	.	.
2	81867111	T	A	intergenic	*LOC100507201,LOC1720*	.	.	.
2	81952267	G	C	intergenic	*LOC100507201,LOC1720*	.	.	.
2	82071390	T	C	intergenic	*LOC100507201,LOC1720*	.	.	.
2	82072192	A	C	intergenic	*LOC100507201,LOC1720*	.	.	.
2	82173376	T	C	intergenic	*LOC100507201,LOC1720*	.	.	.
2	82175479	T	C	intergenic	*LOC100507201,LOC1720*	.	.	.
2	82470835	C	A	intergenic	*LOC100507201,LOC1720*	.	.	.
2	82502532	G	T	intergenic	*LOC100507201,LOC1720*	.	.	.
2	82502534	C	G	intergenic	*LOC100507201,LOC1720*	.	.	.
2	82641327	G	A	intergenic	*LOC100507201,LOC1720*	.	.	.
2	82742996	G	A	intergenic	*LOC100507201,LOC1720*	.	.	.

2	82743718	A	G	intergenic	*LOC100507201,LOC1720*	.	.	.
2	82744719	T	C	intergenic	*LOC100507201,LOC1720*	.	.	.
2	82744721	A	G	intergenic	*LOC100507201,LOC1720*	.	.	.
2	82872221	C	T	intergenic	*LOC100507201,LOC1720*	.	.	.
2	82949457	A	G	intergenic	*LOC100507201,LOC1720*	.	.	.
2	82975995	C	T	intergenic	*LOC100507201,LOC1720*	.	.	.
2	83061357	C	T	intergenic	*LOC100507201,LOC1720*	.	.	.
11	43026451	C	G	intergenic	*LOC100507205,HNRNPKP3*	.	.	.
11	42386668	C	T	intergenic	*LOC100507205,HNRNPKP3*	.	.	.
11	42519513	C	A	intergenic	*LOC100507205,HNRNPKP3*	.	.	.
11	42898838	T	A	intergenic	*LOC100507205,HNRNPKP3*	.	.	.
11	43061479	T	C	intergenic	*LOC100507205,HNRNPKP3*	.	.	.
11	43279250	A	G	intergenic	*LOC100507205,HNRNPKP3*	.	.	.
11	42486460	G	T	intergenic	*LOC100507205,HNRNPKP3*	.	.	.
11	43022764	G	A	intergenic	*LOC100507205,HNRNPKP3*	.	.	.
11	43078614	C	A	intergenic	*LOC100507205,HNRNPKP3*	.	.	.
11	43102160	G	A	intergenic	*LOC100507205,HNRNPKP3*	.	.	.
11	42737621	A	C	intergenic	*LOC100507205,HNRNPKP3*	.	.	.
11	42313303	C	T	intergenic	*LOC100507205,HNRNPKP3*	.	.	.

11	42314409	T	C	intergenic	*LOC100507205,HNRNPKP3*	.	.	.
11	42314410	G	T	intergenic	*LOC100507205,HNRNPKP3*	.	.	.
11	42334558	G	A	intergenic	*LOC100507205,HNRNPKP3*	.	.	.
11	42366755	C	CAG	intergenic	*LOC100507205,HNRNPKP3*	.	.	.
11	42401455	T	G	intergenic	*LOC100507205,HNRNPKP3*	.	.	.
11	42451878	A	T	intergenic	*LOC100507205,HNRNPKP3*	.	.	.
11	42452295	T	C	intergenic	*LOC100507205,HNRNPKP3*	.	.	.
11	42452296	G	A	intergenic	*LOC100507205,HNRNPKP3*	.	.	.
11	42479007	C	T	intergenic	*LOC100507205,HNRNPKP3*	.	.	.
11	42479008	A	G	intergenic	*LOC100507205,HNRNPKP3*	.	.	.
11	42541339	C	A	intergenic	*LOC100507205,HNRNPKP3*	.	.	.
11	42594886	C	T	intergenic	*LOC100507205,HNRNPKP3*	.	.	.
11	42640081	T	A	intergenic	*LOC100507205,HNRNPKP3*	.	.	.
11	42677017	T	C	intergenic	*LOC100507205,HNRNPKP3*	.	.	.
11	42677018	G	A	intergenic	*LOC100507205,HNRNPKP3*	.	.	.
11	42700982	T	C	intergenic	*LOC100507205,HNRNPKP3*	.	.	.
11	42711680	G	A	intergenic	*LOC100507205,HNRNPKP3*	.	.	.
11	42712011	C	T	intergenic	*LOC100507205,HNRNPKP3*	.	.	.
11	42712906	A	T	intergenic	*LOC100507205,HNRNPKP3*	.	.	.

11	42712908	G	C	intergenic	*LOC100507205,HNRNPKP3*	.	.	.
11	42919235	C	T	intergenic	*LOC100507205,HNRNPKP3*	.	.	.
11	42938590	A	G	intergenic	*LOC100507205,HNRNPKP3*	.	.	.
11	43022457	A	T	intergenic	*LOC100507205,HNRNPKP3*	.	.	.
11	43022459	T	G	intergenic	*LOC100507205,HNRNPKP3*	.	.	.
11	43028295	G	A	intergenic	*LOC100507205,HNRNPKP3*	.	.	.
11	43118390	C	T	intergenic	*LOC100507205,HNRNPKP3*	.	.	.
11	43215126	G	A	intergenic	*LOC100507205,HNRNPKP3*	.	.	.
11	43215789	T	A	intergenic	*LOC100507205,HNRNPKP3*	.	.	.
11	43277006	G	A	intergenic	*LOC100507205,HNRNPKP3*	.	.	.
3	139254598	G	T	ncRNA_intronic	*LOC100507291*	.	.	.
3	139246732	A	AT	ncRNA_intronic	*LOC100507291*	.	.	.
3	139214762	C	A	ncRNA_intronic	*LOC100507291*	.	.	.
17	75660180	C	G	intergenic	*LOC100507351,LOC100132174*	.	.	.
12	74569009	T	C	ncRNA_intronic	*LOC100507377*	.	.	.
12	74612256	A	T	ncRNA_intronic	*LOC100507377*	.	.	.
12	74889759	TTAAAC	T	intergenic	*LOC100507377,ATXN7L3B*	.	.	.
12	74865016	A	G	intergenic	*LOC100507377,ATXN7L3B*	.	.	.
12	74865017	T	C	intergenic	*LOC100507377,ATXN7L3B*	.	.	.
3	194514331	A	C	intergenic	*LOC100507391,XXYLT1*	.	.	.
3	194654294	T	C	intergenic	*LOC100507391,XXYLT1*	.	.	.

3	194690865	T	A	intergenic	*LOC100507391,XXYLT1*	.	.	.
6	138110227	G	A	intergenic	*LOC100507406,LOC100130476*	.	.	.
11	130719182	C	T	ncRNA_intronic	*LOC100507431*	.	.	.
11	130731573	G	A	ncRNA_intronic	*LOC100507431*	.	.	.
15	101849315	C	G	ncRNA_exonic	*LOC100507472*	.	.	.
6	140452457	G	A	intergenic	*LOC100507477,MIR3668*	.	.	.
6	140440501	T	G	intergenic	*LOC100507477,MIR3668*	.	.	.
6	140440182	C	T	intergenic	*LOC100507477,MIR3668*	.	.	.
6	140483148	C	T	intergenic	*LOC100507477,MIR3668*	.	.	.
6	140483754	G	A	intergenic	*LOC100507477,MIR3668*	.	.	.
6	140483755	T	G	intergenic	*LOC100507477,MIR3668*	.	.	.
6	146061502	C	T	ncRNA_intronic	*LOC100507557*	.	.	.
6	146061503	A	G	ncRNA_intronic	*LOC100507557*	.	.	.
6	146096318	T	C	ncRNA_intronic	*LOC100507557*	.	.	.
6	146173642	C	T	ncRNA_intronic	*LOC100507557*	.	.	.
6	146192705	G	A	ncRNA_intronic	*LOC100507557*	.	.	.
4	142551206	A	G	intergenic	*LOC100507639,IL15*	.	.	.
4	142284224	A	T	intergenic	*LOC100507639,IL15*	.	.	.
4	142287494	C	T	intergenic	*LOC100507639,IL15*	.	.	.
4	142301908	G	T	intergenic	*LOC100507639,IL15*	.	.	.
4	142321451	C	A	intergenic	*LOC100507639,IL15*	.	.	.
4	142426769	G	T	intergenic	*LOC100507639,IL15*	.	.	.
4	142426851	C	T	intergenic	*LOC100507639,IL15*	.	.	.

4	142433326	TGCAGCAG<br>CAGCA	T	intergenic	*LOC100507639,IL1<br>5*	.	.	.
4	142472990	G	C	intergenic	*LOC100507639,IL1<br>5*	.	.	.
7	102055880	G	A	ncRNA_intronic	*LOC100630923*	.	.	.
1	113577416	CAACAACA<br>ACA	C	ncRNA_intronic	*LOC100996251*	.	.	.
4	153031580	C	A	intergenic	*LOC100996286,FB<br>XW7*	.	.	.
4	153181889	T	C	intergenic	*LOC100996286,FB<br>XW7*	.	.	.
17	76272432	A	AG	ncRNA_intronic	*LOC100996291*	.	.	.
16	54296526	T	C	ncRNA_intronic	*LOC100996338*	.	.	.
5	175484125	C	A	ncRNA_intronic	*LOC100996385*	.	.	.
5	175484127	G	A	ncRNA_intronic	*LOC100996385*	.	.	.
5	175489423	C	T	upstream	*LOC100996385*	.	.	.
3	28226626	A	T	intergenic	*LOC100996624,CM<br>C1*	.	.	.
3	28143082	T	G	intergenic	*LOC100996624,CM<br>C1*	.	.	.
1	96030532	A	G	intergenic	*LOC100996635,LO<br>C102723661*	.	.	.
1	96343262	A	T	intergenic	*LOC100996635,LO<br>C102723661*	.	.	.
1	96255780	AGTGT	A	intergenic	*LOC100996635,LO<br>C102723661*	.	.	.
1	96265873	T	G	intergenic	*LOC100996635,LO<br>C102723661*	.	.	.
1	96358632	G	A	intergenic	*LOC100996635,LO<br>C102723661*	.	.	.
1	96434972	C	T	intergenic	*LOC100996635,LO<br>C102723661*	.	.	.
7	55068480	CAGATAGA<br>T	C	intergenic	*LOC100996654,EG<br>FR*	.	.	.
7	55010121	A	C	intergenic	*LOC100996654,EG<br>FR*	.	.	.
7	54990207	C	T	intergenic	*LOC100996654,EG<br>FR*	.	.	.

7	54991563	A	G	intergenic	*LOC100996654,EGFR*	.	.	.
7	54992682	A	C	intergenic	*LOC100996654,EGFR*	.	.	.
12	127154641	T	C	ncRNA_intronic	*LOC100996671*	.	.	.
4	120897508	C	T	intergenic	*LOC100996694,MAD2L1*	.	.	.
11	101020411	T	C	ncRNA_intronic	*LOC101054525*	.	.	.
11	101147191	G	A	intergenic	*LOC101054525,TRPC6*	.	.	.
11	101180332	G	A	intergenic	*LOC101054525,TRPC6*	.	.	.
11	101236725	CT	C	intergenic	*LOC101054525,TRPC6*	.	.	.
11	101237516	C	T	intergenic	*LOC101054525,TRPC6*	.	.	.
11	101238794	T	TTGGC	intergenic	*LOC101054525,TRPC6*	.	.	.
11	101246698	A	T	intergenic	*LOC101054525,TRPC6*	.	.	.
11	101248007	A	G	intergenic	*LOC101054525,TRPC6*	.	.	.
11	101249362	T	C	intergenic	*LOC101054525,TRPC6*	.	.	.
11	101266989	G	T	intergenic	*LOC101054525,TRPC6*	.	.	.
2	67138068	A	G	ncRNA_intronic	*LOC101060019*	.	.	.
2	67246860	T	C	intergenic	*LOC101060019,LOC102800447*	.	.	.
18	68254674	TTCTCTC	T	intergenic	*LOC101060542,GTSCR1*	.	.	.
18	68233036	C	G	intergenic	*LOC101060542,GTSCR1*	.	.	.
18	68296568	G	A	intergenic	*LOC101060542,GTSCR1*	.	.	.
2	46700179	G	A	intergenic	*LOC101805491,TMEM247*	.	.	.

2	46700240	T	A	intergenic	*LOC101805491,TMEM247*	.	.	.
2	46701001	C	T	intergenic	*LOC101805491,TMEM247*	.	.	.
5	140441621	G	A	ncRNA_intronic	*LOC101926905*	.	.	.
15	91589685	T	G	intergenic	*LOC101926911,SV2B*	.	.	.
15	91636882	T	C	intergenic	*LOC101926911,SV2B*	.	.	.
15	91636883	G	A	intergenic	*LOC101926911,SV2B*	.	.	.
6	46300778	C	G	ncRNA_intronic	*LOC101926915*	.	.	.
20	25831974	CATACATACATACATACATACATACATAT	C	ncRNA_intronic	*LOC101926935*	.	.	.
20	25877483	G	A	intergenic	*LOC101926935,LOC101926955*	.	.	.
20	25915979	C	T	intergenic	*LOC101926935,LOC101926955*	.	.	.
5	39890559	CATAT	C	intergenic	*LOC101926940,LINC00603*	.	.	.
5	39795437	GTA	G	intergenic	*LOC101926940,LINC00603*	.	.	.
5	39688379	C	T	intergenic	*LOC101926940,LINC00603*	.	.	.
5	39845834	TTGTG	T	intergenic	*LOC101926940,LINC00603*	.	.	.
5	39594287	C	T	intergenic	*LOC101926940,LINC00603*	.	.	.
5	39954617	C	T	intergenic	*LOC101926940,LINC00603*	.	.	.
5	39954619	G	C	intergenic	*LOC101926940,LINC00603*	.	.	.
5	39955273	A	G	intergenic	*LOC101926940,LINC00603*	.	.	.
5	40012362	A	G	intergenic	*LOC101926940,LINC00603*	.	.	.

10	92183048	G	A	ncRNA_intronic	*LOC101926942*	.	.	.
10	92276169	G	A	ncRNA_intronic	*LOC101926942*	.	.	.
10	92407875	G	A	intergenic	*LOC101926942,HTR7*	.	.	.
20	25972206	G	A	intergenic	*LOC101926955,LOC100134868*	.	.	.
5	42321851	T	C	intergenic	*LOC101926960,GHR*	.	.	.
5	42254868	G	T	intergenic	*LOC101926960,GHR*	.	.	.
5	42255479	G	A	intergenic	*LOC101926960,GHR*	.	.	.
5	42256459	C	T	intergenic	*LOC101926960,GHR*	.	.	.
5	42355967	T	C	intergenic	*LOC101926960,GHR*	.	.	.
5	42372063	T	A	intergenic	*LOC101926960,GHR*	.	.	.
5	42413852	G	C	intergenic	*LOC101926960,GHR*	.	.	.
5	42415559	T	A	intergenic	*LOC101926960,GHR*	.	.	.
1	61238929	G	T	ncRNA_intronic	*LOC101926964*	.	.	.
2	15899451	T	A	intergenic	*LOC101926966,MYCNUT*	.	.	.
7	1203631	T	TTTG	ncRNA_intronic	*LOC101927021*	.	.	.
8	80756634	G	A	ncRNA_intronic	*LOC101927040*	.	.	.
8	80760051	T	C	ncRNA_intronic	*LOC101927040*	.	.	.
8	80760053	G	A	ncRNA_intronic	*LOC101927040*	.	.	.
2	47590173	G	A	intergenic	*LOC101927043,EPCAM*	.	.	.
14	24917180	T	G	ncRNA_intronic	*LOC101927045*	.	.	.
14	24964919	C	G	ncRNA_intronic	*LOC101927045*	.	.	.
14	24965833	T	A	ncRNA_intronic	*LOC101927045*	.	.	.
12	43038602	C	T	ncRNA_intronic	*LOC101927058*	.	.	.
12	43264367	T	A	intergenic	*LOC101927058,ADAMTS20*	.	.	.

12	43264368	G	T	intergenic	*LOC101927058,ADAMTS20*	.	.	.
12	43389802	ATGTG	A	intergenic	*LOC101927058,ADAMTS20*	.	.	.
12	43495936	G	A	intergenic	*LOC101927058,ADAMTS20*	.	.	.
12	43114976	C	T	intergenic	*LOC101927058,ADAMTS20*	.	.	.
12	43115207	A	G	intergenic	*LOC101927058,ADAMTS20*	.	.	.
12	43117103	A	G	intergenic	*LOC101927058,ADAMTS20*	.	.	.
12	43292730	G	A	intergenic	*LOC101927058,ADAMTS20*	.	.	.
14	27266126	T	A	ncRNA_intronic	*LOC101927062*	.	.	.
14	27285223	A	G	ncRNA_intronic	*LOC101927062,LOC102724890*	.	.	.
14	27285629	TC	T	ncRNA_intronic	*LOC101927062,LOC102724890*	.	.	.
14	27285631	TATC	T	ncRNA_intronic	*LOC101927062,LOC102724890*	.	.	.
8	98016911	A	AT	ncRNA_intronic	*LOC101927066*	.	.	.
8	98015945	G	C	ncRNA_intronic	*LOC101927066*	.	.	.
8	98001719	T	G	ncRNA_intronic	*LOC101927066*	.	.	.
8	97966884	C	T	ncRNA_intronic	*LOC101927066*	.	.	.
8	97967339	T	C	ncRNA_intronic	*LOC101927066*	.	.	.
8	97968244	C	T	ncRNA_intronic	*LOC101927066*	.	.	.
8	98032048	C	G	ncRNA_intronic	*LOC101927066*	.	.	.
8	98053103	G	T	ncRNA_intronic	*LOC101927066*	.	.	.
8	98056885	T	G	ncRNA_intronic	*LOC101927066*	.	.	.
8	98344130	A	C	ncRNA_intronic	*LOC101927066*	.	.	.
8	98497873	A	G	intergenic	*LOC101927066,MTDH*	.	.	.
8	98536335	A	T	intergenic	*LOC101927066,MTDH*	.	.	.
8	98547923	C	T	intergenic	*LOC101927066,MTDH*	.	.	.

8	98547924	T	G	intergenic	*LOC101927066,MTDH*	.	.	.
5	114017691	T	A	ncRNA_intronic	*LOC101927078*	.	.	.
5	113867773	A	T	ncRNA_intronic	*LOC101927078*	.	.	.
5	113867790	G	C	ncRNA_intronic	*LOC101927078*	.	.	.
5	113869442	C	A	ncRNA_intronic	*LOC101927078*	.	.	.
5	113869443	T	G	ncRNA_intronic	*LOC101927078*	.	.	.
5	113909639	C	G	ncRNA_intronic	*LOC101927078*	.	.	.
5	114111594	G	C	intergenic	*LOC101927078,TRIM36*	.	.	.
5	114448576	TG	T	intergenic	*LOC101927078,TRIM36*	.	.	.
5	114115624	T	C	intergenic	*LOC101927078,TRIM36*	.	.	.
5	114132943	A	G	intergenic	*LOC101927078,TRIM36*	.	.	.
5	114184898	T	C	intergenic	*LOC101927078,TRIM36*	.	.	.
5	114206917	T	G	intergenic	*LOC101927078,TRIM36*	.	.	.
5	114214544	A	G	intergenic	*LOC101927078,TRIM36*	.	.	.
5	114227464	T	C	intergenic	*LOC101927078,TRIM36*	.	.	.
5	114228216	G	T	intergenic	*LOC101927078,TRIM36*	.	.	.
5	114228520	A	G	intergenic	*LOC101927078,TRIM36*	.	.	.
5	114395957	G	T	intergenic	*LOC101927078,TRIM36*	.	.	.
5	114396393	T	G	intergenic	*LOC101927078,TRIM36*	.	.	.
15	22381690	AATAGGATACAATAATGTAAGTGTAGAAATTCTCCTATTATTGCTTTC	A	ncRNA_intronic	*LOC101927079*	.	.	.

15	22318583	T	C	ncRNA_intronic	*LOC101927079,LOC727924*	.	.	.
15	22312036	A	G	ncRNA_intronic	*LOC101927079,LOC727924*	.	.	.
15	22312345	A	G	ncRNA_intronic	*LOC101927079,LOC727924*	.	.	.
15	22362322	C	T	ncRNA_intronic	*LOC101927079,LOC727924*	.	.	.
15	22362323	A	G	ncRNA_intronic	*LOC101927079,LOC727924*	.	.	.
4	125509747	C	T	intergenic	*LOC101927087,ANKRD50*	.	.	.
4	125523558	T	C	intergenic	*LOC101927087,ANKRD50*	.	.	.
4	125534119	C	T	intergenic	*LOC101927087,ANKRD50*	.	.	.
4	125535682	C	T	intergenic	*LOC101927087,ANKRD50*	.	.	.
11	56703346	T	A	intergenic	*LOC101927120,OR5AK2*	.	.	.
11	56687941	T	G	intergenic	*LOC101927120,OR5AK2*	.	.	.
11	56688323	G	A	intergenic	*LOC101927120,OR5AK2*	.	.	.
11	56707659	G	A	intergenic	*LOC101927120,OR5AK2*	.	.	.
11	56708229	G	T	intergenic	*LOC101927120,OR5AK2*	.	.	.
11	56732780	C	A	intergenic	*LOC101927120,OR5AK2*	.	.	.
16	47929143	G	A	ncRNA_intronic	*LOC101927132*	.	.	.
16	47946853	G	A	intergenic	*LOC101927132,ABCC12*	.	.	.
6	53466819	C	G	intergenic	*LOC101927136,LINC01564*	.	.	.
19	28644926	T	C	intergenic	*LOC101927151,LOC100420587*	.	.	.

19	28756146	G	A	intergenic	*LOC101927151,LOC100420587*	.	.	.
19	28756604	G	A	intergenic	*LOC101927151,LOC100420587*	.	.	.
19	28827646	C	T	intergenic	*LOC101927151,LOC100420587*	.	.	.
19	28341845	G	A	intergenic	*LOC101927151,LOC100420587*	.	.	.
19	28354452	T	A	intergenic	*LOC101927151,LOC100420587*	.	.	.
19	28436261	A	C	intergenic	*LOC101927151,LOC100420587*	.	.	.
19	28437518	T	C	intergenic	*LOC101927151,LOC100420587*	.	.	.
19	28438612	T	A	intergenic	*LOC101927151,LOC100420587*	.	.	.
19	28580078	A	G	intergenic	*LOC101927151,LOC100420587*	.	.	.
19	28581536	A	T	intergenic	*LOC101927151,LOC100420587*	.	.	.
19	28581537	A	G	intergenic	*LOC101927151,LOC100420587*	.	.	.
2	182098878	C	T	ncRNA_intronic	*LOC101927156*	.	.	.
2	182107446	C	CAGAG	ncRNA_intronic	*LOC101927156*	.	.	.
2	182134417	C	A	ncRNA_intronic	*LOC101927156*	.	.	.
2	182045709	G	A	ncRNA_intronic	*LOC101927156*	.	.	.
2	182315693	A	G	intergenic	*LOC101927156,ITGA4*	.	.	.
4	47922509	C	A	ncRNA_intronic	*LOC101927157*	.	.	.
4	47922765	T	C	ncRNA_intronic	*LOC101927157*	.	.	.
5	115773749	AACAC	A	intergenic	*LOC101927190,SEMA6A*	.	.	.
5	115764857	G	A	intergenic	*LOC101927190,SEMA6A*	.	.	.
18	52822581	A	G	intergenic	*LOC101927229,TCF4*	.	.	.
18	52823972	A	G	intergenic	*LOC101927229,TCF4*	.	.	.

18	52824699	T	A	intergenic	*LOC101927229,TCF4*	.	.	.
1	71207078	A	G	ncRNA_intronic	*LOC101927244*	.	.	.
4	130918784	T	C	intergenic	*LOC101927282,LOC101927305*	.	.	.
4	130972167	A	T	intergenic	*LOC101927282,LOC101927305*	.	.	.
4	131124916	C	A	intergenic	*LOC101927282,LOC101927305*	.	.	.
4	131577163	C	CCATCCCAT	intergenic	*LOC101927282,LOC101927305*	.	.	.
4	131576252	G	T	intergenic	*LOC101927282,LOC101927305*	.	.	.
4	132596778	T	TA	intergenic	*LOC101927282,LOC101927305*	.	.	.
4	132664052	G	T	intergenic	*LOC101927282,LOC101927305*	.	.	.
4	132482507	C	T	intergenic	*LOC101927282,LOC101927305*	.	.	.
4	131607631	G	A	intergenic	*LOC101927282,LOC101927305*	.	.	.
4	132659380	C	A	intergenic	*LOC101927282,LOC101927305*	.	.	.
4	130799405	T	A	intergenic	*LOC101927282,LOC101927305*	.	.	.
4	130799407	C	T	intergenic	*LOC101927282,LOC101927305*	.	.	.
4	131128302	A	G	intergenic	*LOC101927282,LOC101927305*	.	.	.
4	131165405	T	C	intergenic	*LOC101927282,LOC101927305*	.	.	.
4	131361109	G	A	intergenic	*LOC101927282,LOC101927305*	.	.	.
4	131376813	T	C	intergenic	*LOC101927282,LOC101927305*	.	.	.
4	131898371	A	C	intergenic	*LOC101927282,LOC101927305*	.	.	.

4	132065391	C	A	intergenic	*LOC101927282,LOC101927305*	.	.	.
4	132321519	C	T	intergenic	*LOC101927282,LOC101927305*	.	.	.
4	132342950	C	T	intergenic	*LOC101927282,LOC101927305*	.	.	.
4	132397655	G	A	intergenic	*LOC101927282,LOC101927305*	.	.	.
4	132648928	G	C	intergenic	*LOC101927282,LOC101927305*	.	.	.
13	95524700	C	T	ncRNA_intronic	*LOC101927284*	.	.	.
13	95490419	A	C	ncRNA_intronic	*LOC101927284*	.	.	.
13	95525941	T	C	ncRNA_intronic	*LOC101927284*	.	.	.
13	95528251	G	A	ncRNA_intronic	*LOC101927284*	.	.	.
2	59453575	T	A	ncRNA_intronic	*LOC101927285*	.	.	.
2	59489384	T	C	ncRNA_intronic	*LOC101927285*	.	.	.
2	60004212	A	AACACACAC	intergenic	*LOC101927285,MIR4432HG*	.	.	.
2	59639538	C	A	intergenic	*LOC101927285,MIR4432HG*	.	.	.
2	59851815	C	A	intergenic	*LOC101927285,MIR4432HG*	.	.	.
2	59716808	C	T	intergenic	*LOC101927285,MIR4432HG*	.	.	.
2	59665706	G	T	intergenic	*LOC101927285,MIR4432HG*	.	.	.
2	59789139	T	G	intergenic	*LOC101927285,MIR4432HG*	.	.	.
2	60262221	T	C	intergenic	*LOC101927285,MIR4432HG*	.	.	.
2	59556525	A	G	intergenic	*LOC101927285,MIR4432HG*	.	.	.
2	59558392	A	G	intergenic	*LOC101927285,MIR4432HG*	.	.	.
2	59576391	A	G	intergenic	*LOC101927285,MIR4432HG*	.	.	.
2	59577710	A	G	intergenic	*LOC101927285,MIR4432HG*	.	.	.

2	59658543	C	T	intergenic	*LOC101927285,MIR4432HG*	.	.	.
2	59658544	A	G	intergenic	*LOC101927285,MIR4432HG*	.	.	.
2	59658950	C	G	intergenic	*LOC101927285,MIR4432HG*	.	.	.
2	59658951	A	G	intergenic	*LOC101927285,MIR4432HG*	.	.	.
2	59659371	G	A	intergenic	*LOC101927285,MIR4432HG*	.	.	.
2	59925510	G	T	intergenic	*LOC101927285,MIR4432HG*	.	.	.
2	60186382	T	A	intergenic	*LOC101927285,MIR4432HG*	.	.	.
2	60265560	A	G	intergenic	*LOC101927285,MIR4432HG*	.	.	.
2	60267494	C	T	intergenic	*LOC101927285,MIR4432HG*	.	.	.
2	60345003	C	T	intergenic	*LOC101927285,MIR4432HG*	.	.	.
2	60419133	G	A	intergenic	*LOC101927285,MIR4432HG*	.	.	.
2	60421610	A	G	intergenic	*LOC101927285,MIR4432HG*	.	.	.
2	60429145	A	T	intergenic	*LOC101927285,MIR4432HG*	.	.	.
2	60429147	A	T	intergenic	*LOC101927285,MIR4432HG*	.	.	.
2	60429820	T	A	intergenic	*LOC101927285,MIR4432HG*	.	.	.
2	60431206	T	C	intergenic	*LOC101927285,MIR4432HG*	.	.	.
2	60443329	G	A	intergenic	*LOC101927285,MIR4432HG*	.	.	.
15	97919279	T	C	ncRNA_intronic	*LOC101927286*	.	.	.
15	97957616	T	C	ncRNA_intronic	*LOC101927286*	.	.	.
15	98056021	G	A	ncRNA_intronic	*LOC101927286*	.	.	.

15	98084475	C	A	intergenic	*LOC101927286,LOC101927310*	.	.	.
15	98089460	GGATAGATA	G	intergenic	*LOC101927286,LOC101927310*	.	.	.
3	73584141	C	G	ncRNA_intronic	*LOC101927296*	.	.	.
3	73574079	CGATTCATATATATGAAAATCATAGATCT	C	ncRNA_intronic	*LOC101927296*	.	.	.
4	133112768	G	T	intergenic	*LOC101927305,LINC01256*	.	.	.
4	133013348	C	A	intergenic	*LOC101927305,LINC01256*	.	.	.
4	132943543	A	G	intergenic	*LOC101927305,LINC01256*	.	.	.
4	133360662	T	G	intergenic	*LOC101927305,LINC01256*	.	.	.
4	132788023	T	C	intergenic	*LOC101927305,LINC01256*	.	.	.
4	133058030	T	C	intergenic	*LOC101927305,LINC01256*	.	.	.
4	133088400	C	T	intergenic	*LOC101927305,LINC01256*	.	.	.
4	133088758	A	G	intergenic	*LOC101927305,LINC01256*	.	.	.
4	133180947	T	C	intergenic	*LOC101927305,LINC01256*	.	.	.
4	133382265	G	A	intergenic	*LOC101927305,LINC01256*	.	.	.
15	98120665	CTGTGTG	C	intergenic	*LOC101927310,LINC00923*	.	.	.
15	98205164	GA	G	intergenic	*LOC101927310,LINC00923*	.	.	.
15	98114090	T	A	intergenic	*LOC101927310,LINC00923*	.	.	.
15	98122514	C	T	intergenic	*LOC101927310,LINC00923*	.	.	.
6	97959636	T	C	ncRNA_intronic	*LOC101927314*	.	.	.

6	98314400	A	T	intergenic	*LOC101927314,MIR2113*	.	.	.
6	98286537	C	T	intergenic	*LOC101927314,MIR2113*	.	.	.
6	98231031	G	A	intergenic	*LOC101927314,MIR2113*	.	.	.
6	98245411	T	C	intergenic	*LOC101927314,MIR2113*	.	.	.
6	98457493	A	G	intergenic	*LOC101927314,MIR2113*	.	.	.
6	97766183	T	A	ncRNA_intronic	*LOC101927314,MIR548H3*	.	.	.
7	7352716	C	T	intergenic	*LOC101927354,COL28A1*	.	.	.
7	81649085	T	C	ncRNA_intronic	*LOC101927356*	.	.	.
4	134021351	T	C	ncRNA_intronic	*LOC101927359*	.	.	.
7	84199551	T	C	ncRNA_intronic	*LOC101927378*	.	.	.
7	84211178	T	C	ncRNA_intronic	*LOC101927378*	.	.	.
7	84396757	G	C	intergenic	*LOC101927378,SEMA3D*	.	.	.
7	84550884	TCACACACACA	T	intergenic	*LOC101927378,SEMA3D*	.	.	.
7	84574860	C	T	intergenic	*LOC101927378,SEMA3D*	.	.	.
7	84220523	A	G	intergenic	*LOC101927378,SEMA3D*	.	.	.
7	84585092	G	A	intergenic	*LOC101927378,SEMA3D*	.	.	.
7	84617782	T	G	intergenic	*LOC101927378,SEMA3D*	.	.	.
5	121967489	G	T	ncRNA_intronic	*LOC101927379*	.	.	.
5	122021608	T	C	ncRNA_intronic	*LOC101927379*	.	.	.
7	7600351	G	A	ncRNA_intronic	*LOC101927391*	.	.	.
3	8002271	A	G	ncRNA_intronic	*LOC101927394*	.	.	.
3	8102922	C	A	intergenic	*LOC101927394,LMCD1-AS1*	.	.	.
3	8105869	G	C	intergenic	*LOC101927394,LMCD1-AS1*	.	.	.

2	195239280	C	A	ncRNA_intronic	*LOC101927406*	.	.	.
2	195256381	T	C	ncRNA_intronic	*LOC101927406*	.	.	.
2	195313321	G	T	intergenic	*LOC101927406,LOC101927431*	.	.	.
2	195525606	A	T	intergenic	*LOC101927406,LOC101927431*	.	.	.
2	195547709	C	G	intergenic	*LOC101927406,LOC101927431*	.	.	.
2	195322996	A	AAAAT	intergenic	*LOC101927406,LOC101927431*	.	.	.
2	195314792	T	TAGATC	intergenic	*LOC101927406,LOC101927431*	.	.	.
2	195287506	C	T	intergenic	*LOC101927406,LOC101927431*	.	.	.
2	195402538	G	GTA	intergenic	*LOC101927406,LOC101927431*	.	.	.
2	195311764	A	G	intergenic	*LOC101927406,LOC101927431*	.	.	.
2	195327602	A	T	intergenic	*LOC101927406,LOC101927431*	.	.	.
2	195340707	G	A	intergenic	*LOC101927406,LOC101927431*	.	.	.
2	195370117	A	G	intergenic	*LOC101927406,LOC101927431*	.	.	.
2	195507845	T	C	intergenic	*LOC101927406,LOC101927431*	.	.	.
2	195508490	A	C	intergenic	*LOC101927406,LOC101927431*	.	.	.
2	195508612	C	T	intergenic	*LOC101927406,LOC101927431*	.	.	.
19	32771684	C	T	intergenic	*LOC101927411,ZNF507*	.	.	.
19	32817476	A	T	intergenic	*LOC101927411,ZNF507*	.	.	.
1	81523496	T	G	intergenic	*LOC101927412,LOC101927434*	.	.	.
1	81360596	C	A	intergenic	*LOC101927412,LOC101927434*	.	.	.

1	81741898	T	C	intergenic	*LOC101927412,LOC101927434*	.	.	.
1	81131609	CAGAG	C	intergenic	*LOC101927412,LOC101927434*	.	.	.
1	81838129	A	G	intergenic	*LOC101927412,LOC101927434*	.	.	.
1	81151098	GGAGA	G	intergenic	*LOC101927412,LOC101927434*	.	.	.
1	81254343	T	G	intergenic	*LOC101927412,LOC101927434*	.	.	.
5	124602749	A	G	ncRNA_intronic	*LOC101927421*	.	.	.
5	124426629	T	C	ncRNA_intronic	*LOC101927421*	.	.	.
5	124642125	C	G	ncRNA_intronic	*LOC101927421*	.	.	.
5	124728588	A	G	intergenic	*LOC101927421,LOC101927460*	.	.	.
5	124794815	C	T	intergenic	*LOC101927421,LOC101927460*	.	.	.
5	124798635	G	A	intergenic	*LOC101927421,LOC101927460*	.	.	.
2	195597681	A	G	ncRNA_intronic	*LOC101927431*	.	.	.
2	195599547	T	C	ncRNA_intronic	*LOC101927431*	.	.	.
2	195600148	G	T	ncRNA_intronic	*LOC101927431*	.	.	.
2	195631615	T	C	intergenic	*LOC101927431,SLC39A10*	.	.	.
2	195760342	C	G	intergenic	*LOC101927431,SLC39A10*	.	.	.
2	196109807	C	A	intergenic	*LOC101927431,SLC39A10*	.	.	.
2	196152498	C	G	intergenic	*LOC101927431,SLC39A10*	.	.	.
2	195939603	A	C	intergenic	*LOC101927431,SLC39A10*	.	.	.
2	195939605	G	A	intergenic	*LOC101927431,SLC39A10*	.	.	.
2	196194863	C	T	intergenic	*LOC101927431,SLC39A10*	.	.	.
2	196258593	A	G	intergenic	*LOC101927431,SLC39A10*	.	.	.

2	196273995	AAACATGAG	A	intergenic	*LOC101927431,SLC39A10*	.	.	.
2	195671418	T	C	intergenic	*LOC101927431,SLC39A10*	.	.	.
2	195673457	C	T	intergenic	*LOC101927431,SLC39A10*	.	.	.
2	195718021	C	T	intergenic	*LOC101927431,SLC39A10*	.	.	.
2	195804668	A	G	intergenic	*LOC101927431,SLC39A10*	.	.	.
2	195806000	T	G	intergenic	*LOC101927431,SLC39A10*	.	.	.
2	195806530	T	C	intergenic	*LOC101927431,SLC39A10*	.	.	.
2	195839296	A	T	intergenic	*LOC101927431,SLC39A10*	.	.	.
2	195839755	T	C	intergenic	*LOC101927431,SLC39A10*	.	.	.
2	195934990	G	C	intergenic	*LOC101927431,SLC39A10*	.	.	.
2	196089073	G	T	intergenic	*LOC101927431,SLC39A10*	.	.	.
2	196092094	G	C	intergenic	*LOC101927431,SLC39A10*	.	.	.
2	196092095	G	A	intergenic	*LOC101927431,SLC39A10*	.	.	.
2	196092557	C	T	intergenic	*LOC101927431,SLC39A10*	.	.	.
2	196392553	C	G	intergenic	*LOC101927431,SLC39A10*	.	.	.
2	196392587	C	A	intergenic	*LOC101927431,SLC39A10*	.	.	.
13	100601827	C	A	ncRNA_intronic	*LOC101927437*	.	.	.
2	65120476	A	G	intergenic	*LOC101927438,LOC400958*	.	.	.
2	65121218	G	T	intergenic	*LOC101927438,LOC400958*	.	.	.
7	89964054	C	T	upstream	*LOC101927446*	.	.	.

9	81844254	C	A	intergenic	*LOC101927450,TLE4*	.	.	.
9	81875212	G	A	intergenic	*LOC101927450,TLE4*	.	.	.
9	82016011	GTATA	G	intergenic	*LOC101927450,TLE4*	.	.	.
9	82034314	T	G	intergenic	*LOC101927450,TLE4*	.	.	.
9	82034315	C	T	intergenic	*LOC101927450,TLE4*	.	.	.
9	82055948	G	T	intergenic	*LOC101927450,TLE4*	.	.	.
9	82056582	T	A	intergenic	*LOC101927450,TLE4*	.	.	.
9	82161627	G	A	intergenic	*LOC101927450,TLE4*	.	.	.
9	82161859	C	T	intergenic	*LOC101927450,TLE4*	.	.	.
5	124904278	C	T	ncRNA_intronic	*LOC101927460*	.	.	.
5	124904389	A	G	ncRNA_intronic	*LOC101927460*	.	.	.
5	124905056	A	G	ncRNA_intronic	*LOC101927460*	.	.	.
5	125306524	C	G	intergenic	*LOC101927460,LOC102546228*	.	.	.
5	125511588	A	G	intergenic	*LOC101927460,LOC102546228*	.	.	.
5	125060953	A	AATAT	intergenic	*LOC101927460,LOC102546228*	.	.	.
5	125074839	G	T	intergenic	*LOC101927460,LOC102546228*	.	.	.
5	124960684	C	G	intergenic	*LOC101927460,LOC102546228*	.	.	.
5	124961105	G	A	intergenic	*LOC101927460,LOC102546228*	.	.	.
5	124961467	C	T	intergenic	*LOC101927460,LOC102546228*	.	.	.
5	124961975	G	A	intergenic	*LOC101927460,LOC102546228*	.	.	.

5	125071731	T	C	intergenic	*LOC101927460,LOC102546228*	.	.	.
5	125071732	C	A	intergenic	*LOC101927460,LOC102546228*	.	.	.
5	125072907	G	A	intergenic	*LOC101927460,LOC102546228*	.	.	.
5	125077433	G	A	intergenic	*LOC101927460,LOC102546228*	.	.	.
5	125078123	T	A	intergenic	*LOC101927460,LOC102546228*	.	.	.
5	125178399	A	T	intergenic	*LOC101927460,LOC102546228*	.	.	.
5	125180113	T	C	intergenic	*LOC101927460,LOC102546228*	.	.	.
5	125207776	C	T	intergenic	*LOC101927460,LOC102546228*	.	.	.
5	125333988	A	C	intergenic	*LOC101927460,LOC102546228*	.	.	.
5	125407321	C	T	intergenic	*LOC101927460,LOC102546228*	.	.	.
5	125409648	T	C	intergenic	*LOC101927460,LOC102546228*	.	.	.
5	125426665	C	A	intergenic	*LOC101927460,LOC102546228*	.	.	.
5	125486101	G	C	intergenic	*LOC101927460,LOC102546228*	.	.	.
5	125510946	C	A	intergenic	*LOC101927460,LOC102546228*	.	.	.
5	125512288	A	C	intergenic	*LOC101927460,LOC102546228*	.	.	.
12	126674898	T	A	intergenic	*LOC101927464,LOC100128554*	.	.	.
12	126768393	C	G	intergenic	*LOC101927464,LOC100128554*	.	.	.
12	126768740	T	C	intergenic	*LOC101927464,LOC100128554*	.	.	.
12	126806356	C	A	intergenic	*LOC101927464,LOC100128554*	.	.	.

1	229647742	T	C	ncRNA_intronic	*LOC101927478*	.	.	.
1	229647743	T	A	ncRNA_intronic	*LOC101927478*	.	.	.
5	125645347	T	C	intergenic	*LOC101927488,GRAMD3*	.	.	.
5	125628122	T	G	intergenic	*LOC101927488,GRAMD3*	.	.	.
5	125628123	T	C	intergenic	*LOC101927488,GRAMD3*	.	.	.
7	92514799	T	C	ncRNA_intronic	*LOC101927497*	.	.	.
7	92523182	T	A	ncRNA_intronic	*LOC101927497*	.	.	.
7	92524748	G	T	ncRNA_intronic	*LOC101927497*	.	.	.
7	92545165	G	A	ncRNA_intronic	*LOC101927497*	.	.	.
7	92599447	C	T	intergenic	*LOC101927497,SAMD9*	.	.	.
7	92551027	G	C	intergenic	*LOC101927497,SAMD9*	.	.	.
7	92551387	G	A	intergenic	*LOC101927497,SAMD9*	.	.	.
7	92551581	A	ATG	intergenic	*LOC101927497,SAMD9*	.	.	.
7	92587961	T	C	intergenic	*LOC101927497,SAMD9*	.	.	.
7	92588374	A	AG	intergenic	*LOC101927497,SAMD9*	.	.	.
7	92699722	T	G	intergenic	*LOC101927497,SAMD9*	.	.	.
7	92710176	T	C	intergenic	*LOC101927497,SAMD9*	.	.	.
X	43142469	T	C	intergenic	*LOC101927501,MAOA*	.	.	.
X	43241776	A	T	intergenic	*LOC101927501,MAOA*	.	.	.
X	43393170	TG	T	intergenic	*LOC101927501,MAOA*	.	.	.
X	43227632	G	GCATTAATC,	intergenic	*LOC101927501,MAOA*	.	.	.
X	43347851	A	G	intergenic	*LOC101927501,MAOA*	.	.	.

9	84364018	C	T	ncRNA_intronic	*LOC101927502*	.	.	.
9	84401044	T	C	intergenic	*LOC101927502,SPATA31D5P*	.	.	.
10	106379156	TAACAAC	T	intergenic	*LOC101927523,SORCS3*	.	.	.
10	106288896	C	T	intergenic	*LOC101927523,SORCS3*	.	.	.
10	107521825	C	T	ncRNA_intronic	*LOC101927549*	.	.	.
10	107498380	A	G	ncRNA_intronic	*LOC101927549*	.	.	.
10	107527935	C	T	ncRNA_intronic	*LOC101927549*	.	.	.
10	107571437	A	T	ncRNA_intronic	*LOC101927549*	.	.	.
10	107727125	A	G	intergenic	*LOC101927549,SORCS1*	.	.	.
10	107749490	C	T	intergenic	*LOC101927549,SORCS1*	.	.	.
10	107855473	G	T	intergenic	*LOC101927549,SORCS1*	.	.	.
10	108011929	C	A	intergenic	*LOC101927549,SORCS1*	.	.	.
10	108007726	G	T	intergenic	*LOC101927549,SORCS1*	.	.	.
10	107855493	TTGTG	T	intergenic	*LOC101927549,SORCS1*	.	.	.
10	107611492	T	C	intergenic	*LOC101927549,SORCS1*	.	.	.
10	107611493	C	T	intergenic	*LOC101927549,SORCS1*	.	.	.
10	107651446	C	A	intergenic	*LOC101927549,SORCS1*	.	.	.
10	107829796	T	C	intergenic	*LOC101927549,SORCS1*	.	.	.
10	107863380	T	G	intergenic	*LOC101927549,SORCS1*	.	.	.
10	107863382	A	G	intergenic	*LOC101927549,SORCS1*	.	.	.
10	107983091	G	A	intergenic	*LOC101927549,SORCS1*	.	.	.

10	108088624	T	G	intergenic	*LOC101927549,SORCS1*	.	.	.
10	108088625	A	G	intergenic	*LOC101927549,SORCS1*	.	.	.
10	108159794	T	C	intergenic	*LOC101927549,SORCS1*	.	.	.
10	108159795	G	A	intergenic	*LOC101927549,SORCS1*	.	.	.
10	108180666	T	C	intergenic	*LOC101927549,SORCS1*	.	.	.
10	108182040	C	A	intergenic	*LOC101927549,SORCS1*	.	.	.
10	108190625	G	A	intergenic	*LOC101927549,SORCS1*	.	.	.
10	108230098	G	A	intergenic	*LOC101927549,SORCS1*	.	.	.
10	108230321	G	T	intergenic	*LOC101927549,SORCS1*	.	.	.
10	108231399	C	T	intergenic	*LOC101927549,SORCS1*	.	.	.
10	108231400	C	G	intergenic	*LOC101927549,SORCS1*	.	.	.
18	20341181	T	G	ncRNA_intronic	*LOC101927571*	.	.	.
18	20341351	T	G	ncRNA_intronic	*LOC101927571*	.	.	.
18	20341440	C	G	ncRNA_intronic	*LOC101927571*	.	.	.
18	20468758	T	C	intergenic	*LOC101927571,RBBP8*	.	.	.
18	20473943	G	A	intergenic	*LOC101927571,RBBP8*	.	.	.
18	20474271	G	A	intergenic	*LOC101927571,RBBP8*	.	.	.
9	86862751	C	A	intergenic	*LOC101927575,SLC28A3*	.	.	.
2	67027014	G	C	intergenic	*LOC101927577,LOC101060019*	.	.	.
16	59934521	A	T	ncRNA_intronic	*LOC101927580*	.	.	.
16	60003990	A	G	ncRNA_intronic	*LOC101927580*	.	.	.
16	60017243	A	G	ncRNA_intronic	*LOC101927580*	.	.	.

16	60072875	A	T	ncRNA_intronic	*LOC101927580*	.	.	.
16	59916054	T	C	ncRNA_intronic	*LOC101927580*	.	.	.
16	60110034	C	T	intergenic	*LOC101927580,LOC729159*	.	.	.
16	60110035	A	G	intergenic	*LOC101927580,LOC729159*	.	.	.
16	60182502	A	C	intergenic	*LOC101927580,LOC729159*	.	.	.
1	84194864	A	G	ncRNA_intronic	*LOC101927587*	.	.	.
1	84087351	T	C	ncRNA_intronic	*LOC101927587*	.	.	.
1	84126791	T	A	ncRNA_intronic	*LOC101927587*	.	.	.
1	84151882	A	G	ncRNA_intronic	*LOC101927587*	.	.	.
8	125215423	G	T	ncRNA_intronic	*LOC101927588*	.	.	.
12	127446238	C	T	ncRNA_intronic	*LOC101927592*	.	.	.
12	127401071	G	T	ncRNA_intronic	*LOC101927592*	.	.	.
12	127444628	TA	T	ncRNA_intronic	*LOC101927592*	.	.	.
12	127533787	G	C	ncRNA_intronic	*LOC101927592*	.	.	.
12	127667490	C	T	intergenic	*LOC101927592,LOC101927616*	.	.	.
12	127667491	C	A	intergenic	*LOC101927592,LOC101927616*	.	.	.
12	127656880	T	G	intergenic	*LOC101927592,LOC101927616*	.	.	.
12	127721626	C	T	intergenic	*LOC101927592,LOC101927616*	.	.	.
12	127657184	G	A	intergenic	*LOC101927592,LOC101927616*	.	.	.
12	127719181	G	T	intergenic	*LOC101927592,LOC101927616*	.	.	.
12	127758865	CAT	C	intergenic	*LOC101927592,LOC101927616*	.	.	.
12	127583583	AT	A	intergenic	*LOC101927592,LOC101927616*	.	.	.
12	127622682	C	T	intergenic	*LOC101927592,LOC101927616*	.	.	.
12	127724920	T	TTGTG	intergenic	*LOC101927592,LOC101927616*	.	.	.

12	127899061	A	G	intergenic	*LOC101927616,LOC101927637*	.	.	.
12	128068491	GTTTAATT	G	intergenic	*LOC101927616,LOC101927637*	.	.	.
12	128079821	C	T	intergenic	*LOC101927616,LOC101927637*	.	.	.
12	127894851	C	G	intergenic	*LOC101927616,LOC101927637*	.	.	.
12	127826178	T	C	intergenic	*LOC101927616,LOC101927637*	.	.	.
12	127877846	A	AATT	intergenic	*LOC101927616,LOC101927637*	.	.	.
2	199163237	C	A	downstream	*LOC101927619*	.	.	.
2	199313326	T	C	intergenic	*LOC101927619,SATB2*	.	.	.
2	199706822	A	G	intergenic	*LOC101927619,SATB2*	.	.	.
2	199811093	G	T	intergenic	*LOC101927619,SATB2*	.	.	.
2	199293076	G	A	intergenic	*LOC101927619,SATB2*	.	.	.
2	199394236	A	T	intergenic	*LOC101927619,SATB2*	.	.	.
2	199440817	C	T	intergenic	*LOC101927619,SATB2*	.	.	.
2	199535429	A	G	intergenic	*LOC101927619,SATB2*	.	.	.
2	199554217	T	C	intergenic	*LOC101927619,SATB2*	.	.	.
2	199555095	A	C	intergenic	*LOC101927619,SATB2*	.	.	.
2	199694294	A	G	intergenic	*LOC101927619,SATB2*	.	.	.
2	199872775	T	C	intergenic	*LOC101927619,SATB2*	.	.	.
2	199873110	C	T	intergenic	*LOC101927619,SATB2*	.	.	.

2	199873623	T	G	intergenic	*LOC101927619,SATB2*	.	.	.
2	199873624	G	A	intergenic	*LOC101927619,SATB2*	.	.	.
2	199896094	T	G	intergenic	*LOC101927619,SATB2*	.	.	.
2	199896821	T	C	intergenic	*LOC101927619,SATB2*	.	.	.
2	199897256	A	G	intergenic	*LOC101927619,SATB2*	.	.	.
2	199898352	T	C	intergenic	*LOC101927619,SATB2*	.	.	.
2	199917656	T	A	intergenic	*LOC101927619,SATB2*	.	.	.
2	200021751	G	A	intergenic	*LOC101927619,SATB2*	.	.	.
2	200108865	T	C	intergenic	*LOC101927619,SATB2*	.	.	.
14	53875175	A	ACTCCTGCC	intergenic	*LOC101927620,MIR5580*	.	.	.
14	53766286	G	A	intergenic	*LOC101927620,MIR5580*	.	.	.
14	53922498	C	G	intergenic	*LOC101927620,MIR5580*	.	.	.
14	53758185	A	G	intergenic	*LOC101927620,MIR5580*	.	.	.
14	53748871	C	T	intergenic	*LOC101927620,MIR5580*	.	.	.
14	53895390	C	A	intergenic	*LOC101927620,MIR5580*	.	.	.
14	53895825	G	C	intergenic	*LOC101927620,MIR5580*	.	.	.
14	53896231	T	A	intergenic	*LOC101927620,MIR5580*	.	.	.
14	54268152	T	A	intergenic	*LOC101927620,MIR5580*	.	.	.
14	54344230	A	T	intergenic	*LOC101927620,MIR5580*	.	.	.

9	88773972	C	A	intergenic	*LOC101927623,C9orf153*	.	.	.
7	17553814	C	A	ncRNA_intronic	*LOC101927630*	.	.	.
7	17603376	G	A	intergenic	*LOC101927630,SNX13*	.	.	.
7	17609652	T	C	intergenic	*LOC101927630,SNX13*	.	.	.
7	17622235	A	G	intergenic	*LOC101927630,SNX13*	.	.	.
7	17622236	A	G	intergenic	*LOC101927630,SNX13*	.	.	.
7	17776435	G	A	intergenic	*LOC101927630,SNX13*	.	.	.
7	17776436	T	A	intergenic	*LOC101927630,SNX13*	.	.	.
7	17777953	T	C	intergenic	*LOC101927630,SNX13*	.	.	.
12	128202944	A	G	intergenic	*LOC101927637,FLJ37505*	.	.	.
12	128331388	C	T	intergenic	*LOC101927637,FLJ37505*	.	.	.
12	128359676	A	G	intergenic	*LOC101927637,FLJ37505*	.	.	.
8	127356676	G	A	intergenic	*LOC101927657,FAM84B*	.	.	.
8	127474426	C	G	intergenic	*LOC101927657,FAM84B*	.	.	.
7	20064721	A	AC	ncRNA_intronic	*LOC101927668*	.	.	.
7	20064722	T	A	ncRNA_intronic	*LOC101927668*	.	.	.
7	20095924	G	A	ncRNA_intronic	*LOC101927668*	.	.	.
7	20110077	G	A	ncRNA_intronic	*LOC101927668*	.	.	.
7	20110781	G	C	ncRNA_intronic	*LOC101927668*	.	.	.
12	128644031	T	C	intergenic	*LOC101927694,MIR4419B*	.	.	.
12	128673747	A	ATGTGTG	intergenic	*LOC101927694,MIR4419B*	.	.	.
12	128695862	G	T	intergenic	*LOC101927694,MIR4419B*	.	.	.

5	157793905	G	T	ncRNA_intronic	*LOC101927697*	.	.	.
5	157823850	G	T	ncRNA_intronic	*LOC101927697*	.	.	.
5	157977000	GCACA	G	intergenic	*LOC101927697,EBF1*	.	.	.
5	157866505	C	T	intergenic	*LOC101927697,EBF1*	.	.	.
5	157866506	A	G	intergenic	*LOC101927697,EBF1*	.	.	.
5	157869244	A	T	intergenic	*LOC101927697,EBF1*	.	.	.
5	157976131	T	G	intergenic	*LOC101927697,EBF1*	.	.	.
5	158006138	A	G	intergenic	*LOC101927697,EBF1*	.	.	.
5	158093809	A	AG	intergenic	*LOC101927697,EBF1*	.	.	.
2	68232935	C	T	intergenic	*LOC101927701,C1D*	.	.	.
2	119477720	CAGAGAG	C	intergenic	*LOC101927709,EN1*	.	.	.
2	119431603	T	A	intergenic	*LOC101927709,EN1*	.	.	.
10	1011402	G	A	intergenic	*LOC101927762,GTPBP4*	.	.	.
5	159221800	C	A	ncRNA_intronic	*LOC101927766*	.	.	.
5	159280468	G	A	ncRNA_intronic	*LOC101927766*	.	.	.
5	159279009	A	T	ncRNA_intronic	*LOC101927766*	.	.	.
5	159279418	T	C	ncRNA_intronic	*LOC101927766*	.	.	.
5	159300062	G	T	intergenic	*LOC101927766,ADRA1B*	.	.	.
6	114527591	C	T	ncRNA_intronic	*LOC101927768*	.	.	.
6	114484391	C	A	ncRNA_intronic	*LOC101927768*	.	.	.
6	114310133	C	CT	ncRNA_intronic	*LOC101927768*	.	.	.
6	114311647	A	G	ncRNA_intronic	*LOC101927768*	.	.	.
6	114394042	G	A	ncRNA_intronic	*LOC101927768*	.	.	.
6	114394500	T	C	ncRNA_intronic	*LOC101927768*	.	.	.
6	115440574	C	T	intergenic	*LOC101927768,FRK*	.	.	.

6	115759046	G	A	intergenic	*LOC101927768,FRK*	.	.	.
6	115270161	G	T	intergenic	*LOC101927768,FRK*	.	.	.
6	115514663	G	A	intergenic	*LOC101927768,FRK*	.	.	.
6	115838011	G	T	intergenic	*LOC101927768,FRK*	.	.	.
6	116155824	T	C	intergenic	*LOC101927768,FRK*	.	.	.
6	115906509	T	C	intergenic	*LOC101927768,FRK*	.	.	.
6	115216613	C	G	intergenic	*LOC101927768,FRK*	.	.	.
6	115003829	T	C	intergenic	*LOC101927768,FRK*	.	.	.
6	115217907	C	T	intergenic	*LOC101927768,FRK*	.	.	.
6	115265294	T	C	intergenic	*LOC101927768,FRK*	.	.	.
6	114965904	C	T	intergenic	*LOC101927768,FRK*	.	.	.
6	115140305	T	C	intergenic	*LOC101927768,FRK*	.	.	.
6	114695998	C	T	intergenic	*LOC101927768,FRK*	.	.	.
6	114745043	G	A	intergenic	*LOC101927768,FRK*	.	.	.
6	114876014	A	G	intergenic	*LOC101927768,FRK*	.	.	.
6	114951373	A	G	intergenic	*LOC101927768,FRK*	.	.	.
6	114952660	A	G	intergenic	*LOC101927768,FRK*	.	.	.
6	115124089	C	T	intergenic	*LOC101927768,FRK*	.	.	.
6	115245742	G	A	intergenic	*LOC101927768,FRK*	.	.	.

6	115245744	T	C	intergenic	*LOC101927768,FRK*	.	.	.
6	115267465	T	A	intergenic	*LOC101927768,FRK*	.	.	.
6	115291597	T	A	intergenic	*LOC101927768,FRK*	.	.	.
6	115294452	T	C	intergenic	*LOC101927768,FRK*	.	.	.
6	115294453	G	A	intergenic	*LOC101927768,FRK*	.	.	.
6	115483937	A	G	intergenic	*LOC101927768,FRK*	.	.	.
6	115484066	C	T	intergenic	*LOC101927768,FRK*	.	.	.
6	115547702	G	A	intergenic	*LOC101927768,FRK*	.	.	.
6	115616138	G	C	intergenic	*LOC101927768,FRK*	.	.	.
6	115712557	A	G	intergenic	*LOC101927768,FRK*	.	.	.
6	115712558	C	T	intergenic	*LOC101927768,FRK*	.	.	.
6	115713039	A	G	intergenic	*LOC101927768,FRK*	.	.	.
6	115800815	A	G	intergenic	*LOC101927768,FRK*	.	.	.
6	115868551	G	A	intergenic	*LOC101927768,FRK*	.	.	.
6	116180889	C	G	intergenic	*LOC101927768,FRK*	.	.	.
6	116260832	C	A	intergenic	*LOC101927768,FRK*	.	.	.
6	116260833	A	G	intergenic	*LOC101927768,FRK*	.	.	.
20	52175469	A	ACATATATAT	ncRNA_intronic	*LOC101927770*	.	.	.
1	234810466	T	A	intergenic	*LOC101927787,LINC01132*	.	.	.

8	134829909	G	A	intergenic	*LOC101927798,LOC101927822*	.	.	.
8	134878690	G	A	intergenic	*LOC101927798,LOC101927822*	.	.	.
8	134879532	C	T	intergenic	*LOC101927798,LOC101927822*	.	.	.
8	134880776	G	A	intergenic	*LOC101927798,LOC101927822*	.	.	.
8	134889460	C	T	intergenic	*LOC101927798,LOC101927822*	.	.	.
8	134890031	C	G	intergenic	*LOC101927798,LOC101927822*	.	.	.
8	2734872	CTGTG	C	intergenic	*LOC101927815,CSMD1*	.	.	.
8	2608714	G	T	intergenic	*LOC101927815,CSMD1*	.	.	.
8	2658979	C	G	intergenic	*LOC101927815,CSMD1*	.	.	.
8	2659508	CGTGT	C	intergenic	*LOC101927815,CSMD1*	.	.	.
8	2772041	GCACA	G	intergenic	*LOC101927815,CSMD1*	.	.	.
8	134901351	A	G	ncRNA_intronic	*LOC101927822*	.	.	.
8	135395227	C	G	intergenic	*LOC101927822,ZFAT*	.	.	.
8	135101391	A	G	intergenic	*LOC101927822,ZFAT*	.	.	.
8	135417435	G	T	intergenic	*LOC101927822,ZFAT*	.	.	.
8	134979152	CAACCTA	C	intergenic	*LOC101927822,ZFAT*	.	.	.
8	135320447	C	T	intergenic	*LOC101927822,ZFAT*	.	.	.
8	135322624	A	G	intergenic	*LOC101927822,ZFAT*	.	.	.
8	135361498	G	A	intergenic	*LOC101927822,ZFAT*	.	.	.

8	135361887	C	T	intergenic	*LOC101927822,ZFAT*	.	.	.
8	135363380	A	T	intergenic	*LOC101927822,ZFAT*	.	.	.
3	20481314	T	A	intergenic	*LOC101927829,VENTXP7*	.	.	.
3	20888812	A	G	intergenic	*LOC101927829,VENTXP7*	.	.	.
3	21189734	TACACACATATATATGTATGTGTGTATATATATGTGTGGTGTGTATATATATATACACAC	T	intergenic	*LOC101927829,VENTXP7*	.	.	.
3	21217814	T	A	intergenic	*LOC101927829,VENTXP7*	.	.	.
3	21380750	T	A	intergenic	*LOC101927829,VENTXP7*	.	.	.
3	20396456	C	A	intergenic	*LOC101927829,VENTXP7*	.	.	.
3	20469503	T	A	intergenic	*LOC101927829,VENTXP7*	.	.	.
3	20670092	G	A	intergenic	*LOC101927829,VENTXP7*	.	.	.
3	20962663	C	T	intergenic	*LOC101927829,VENTXP7*	.	.	.
3	21169066	G	A	intergenic	*LOC101927829,VENTXP7*	.	.	.
3	21169067	G	C	intergenic	*LOC101927829,VENTXP7*	.	.	.
3	21169755	T	C	intergenic	*LOC101927829,VENTXP7*	.	.	.
3	21172087	T	A	intergenic	*LOC101927829,VENTXP7*	.	.	.
3	21394094	A	G	intergenic	*LOC101927829,VENTXP7*	.	.	.

3	21394095	A	T	intergenic	*LOC101927829,VENTXP7*	.	.	.
21	23450690	CATAT	C	ncRNA_intronic	*LOC101927843*	.	.	.
21	23402067	A	G	ncRNA_intronic	*LOC101927843*	.	.	.
21	23462042	A	G	ncRNA_intronic	*LOC101927843*	.	.	.
21	23462043	A	G	ncRNA_intronic	*LOC101927843*	.	.	.
8	135923165	T	G	intergenic	*LOC101927845,LINC01591*	.	.	.
8	135993131	G	A	intergenic	*LOC101927845,LINC01591*	.	.	.
8	136026067	T	G	intergenic	*LOC101927845,LINC01591*	.	.	.
8	136163058	G	A	intergenic	*LOC101927845,LINC01591*	.	.	.
4	150145338	G	C	ncRNA_intronic	*LOC101927849*	.	.	.
4	150152285	G	A	ncRNA_intronic	*LOC101927849*	.	.	.
4	150164989	T	C	ncRNA_intronic	*LOC101927849*	.	.	.
4	150383109	G	A	intergenic	*LOC101927849,DCLK2*	.	.	.
4	150626744	T	C	intergenic	*LOC101927849,DCLK2*	.	.	.
4	150258573	C	G	intergenic	*LOC101927849,DCLK2*	.	.	.
4	150258574	C	T	intergenic	*LOC101927849,DCLK2*	.	.	.
4	150340815	T	C	intergenic	*LOC101927849,DCLK2*	.	.	.
4	150470605	A	G	intergenic	*LOC101927849,DCLK2*	.	.	.
4	150515486	T	G	intergenic	*LOC101927849,DCLK2*	.	.	.
4	150674819	T	A	intergenic	*LOC101927849,DCLK2*	.	.	.
4	150737461	T	A	intergenic	*LOC101927849,DCLK2*	.	.	.
4	150739751	T	A	intergenic	*LOC101927849,DCLK2*	.	.	.

4	150866074	C	T	intergenic	*LOC101927849,DCLK2*	.	.	.
4	150900148	A	G	intergenic	*LOC101927849,DCLK2*	.	.	.
4	150996251	T	A	intergenic	*LOC101927849,DCLK2*	.	.	.
4	150996253	A	T	intergenic	*LOC101927849,DCLK2*	.	.	.
21	25688983	G	T	ncRNA_intronic	*LOC101927869*	.	.	.
21	25692159	CGTTGAACTTCT	C	ncRNA_intronic	*LOC101927869*	.	.	.
21	25850245	T	A	intergenic	*LOC101927869,LOC339622*	.	.	.
21	25957306	T	C	intergenic	*LOC101927869,LOC339622*	.	.	.
21	26004239	A	G	intergenic	*LOC101927869,LOC339622*	.	.	.
21	25862520	T	C	intergenic	*LOC101927869,LOC339622*	.	.	.
21	25725398	A	G	intergenic	*LOC101927869,LOC339622*	.	.	.
21	25928968	TG	T	intergenic	*LOC101927869,LOC339622*	.	.	.
7	103097840	G	A	ncRNA_intronic	*LOC101927870*	.	.	.
7	103089775	A	G	ncRNA_intronic	*LOC101927870*	.	.	.
7	103089777	T	A	ncRNA_intronic	*LOC101927870*	.	.	.
7	103090308	T	C	ncRNA_intronic	*LOC101927870*	.	.	.
7	103096019	C	T	ncRNA_intronic	*LOC101927870*	.	.	.
7	103146610	T	G	ncRNA_intronic	*LOC101927870*	.	.	.
7	103148332	G	A	ncRNA_intronic	*LOC101927870*	.	.	.
7	103149030	T	C	ncRNA_intronic	*LOC101927870*	.	.	.
7	103149031	A	T	ncRNA_intronic	*LOC101927870*	.	.	.
2	129865055	G	A	intergenic	*LOC101927881,LOC151121*	.	.	.
2	129934217	C	G	intergenic	*LOC101927881,LOC151121*	.	.	.
2	129996791	C	T	intergenic	*LOC101927881,LOC151121*	.	.	.

2	129698067	T	C	intergenic	*LOC101927881,LOC151121*	.	.	.
2	129795689	C	A	intergenic	*LOC101927881,LOC151121*	.	.	.
2	129798103	A	G	intergenic	*LOC101927881,LOC151121*	.	.	.
2	129798776	G	C	intergenic	*LOC101927881,LOC151121*	.	.	.
2	129799529	A	G	intergenic	*LOC101927881,LOC151121*	.	.	.
2	129803510	A	G	intergenic	*LOC101927881,LOC151121*	.	.	.
2	129805550	T	A	intergenic	*LOC101927881,LOC151121*	.	.	.
2	129805552	C	G	intergenic	*LOC101927881,LOC151121*	.	.	.
2	129989979	T	C	intergenic	*LOC101927881,LOC151121*	.	.	.
12	68184959	T	C	intergenic	*LOC101927901,LINC01479*	.	.	.
7	157279727	G	T	ncRNA_intronic	*LOC101927914*	.	.	.
7	157279728	T	A	ncRNA_intronic	*LOC101927914*	.	.	.
7	157304553	T	C	intergenic	*LOC101927914,PTPRN2*	.	.	.
8	138983467	A	G	intergenic	*LOC101927915,FAM135B*	.	.	.
8	138593066	G	T	intergenic	*LOC101927915,FAM135B*	.	.	.
8	138444528	G	A	intergenic	*LOC101927915,FAM135B*	.	.	.
8	138666451	G	A	intergenic	*LOC101927915,FAM135B*	.	.	.
8	138833714	A	G	intergenic	*LOC101927915,FAM135B*	.	.	.
8	138833716	A	T	intergenic	*LOC101927915,FAM135B*	.	.	.
8	139140475	G	A	intergenic	*LOC101927915,FAM135B*	.	.	.

8	138626308	G	A	intergenic	*LOC101927915,FAM135B*	.	.	.
8	138829914	G	A	intergenic	*LOC101927915,FAM135B*	.	.	.
8	138830061	C	T	intergenic	*LOC101927915,FAM135B*	.	.	.
8	138840038	G	C	intergenic	*LOC101927915,FAM135B*	.	.	.
8	139059550	G	A	intergenic	*LOC101927915,FAM135B*	.	.	.
8	139133734	C	T	intergenic	*LOC101927915,FAM135B*	.	.	.
20	57450919	C	T	ncRNA_intronic	*LOC101927932*	.	.	.
5	133850709	G	A	intergenic	*LOC101927934,JADE2*	.	.	.
6	5688693	G	A	ncRNA_intronic	*LOC101927950*	.	.	.
2	78381914	C	G	ncRNA_intronic	*LOC101927967*	.	.	.
2	78235082	A	G	ncRNA_intronic	*LOC101927967*	.	.	.
2	78425960	C	T	ncRNA_intronic	*LOC101927967*	.	.	.
2	78515957	C	T	ncRNA_intronic	*LOC101927967*	.	.	.
2	78037696	A	G	ncRNA_intronic	*LOC101927967*	.	.	.
2	78038166	A	G	ncRNA_intronic	*LOC101927967*	.	.	.
2	78038167	C	G	ncRNA_intronic	*LOC101927967*	.	.	.
2	78072816	T	A	ncRNA_intronic	*LOC101927967*	.	.	.
2	78390609	G	A	ncRNA_intronic	*LOC101927967*	.	.	.
2	78419895	A	AGACTTATT	ncRNA_intronic	*LOC101927967*	.	.	.
2	78474364	T	A	ncRNA_intronic	*LOC101927967*	.	.	.
2	78474983	G	A	ncRNA_intronic	*LOC101927967*	.	.	.
2	78863019	C	T	intergenic	*LOC101927967,REG3G*	.	.	.
2	78760510	GTATA	G	intergenic	*LOC101927967,REG3G*	.	.	.
2	78850820	A	C	intergenic	*LOC101927967,REG3G*	.	.	.
2	78646143	A	T	intergenic	*LOC101927967,REG3G*	.	.	.
2	78553931	AG	A	intergenic	*LOC101927967,REG3G*	.	.	.

2	78923012	C	T	intergenic	*LOC101927967,REG3G*	.	.	.
2	78950625	A	G	intergenic	*LOC101927967,REG3G*	.	.	.
21	29548374	T	C	ncRNA_intronic	*LOC101927973*	.	.	.
21	29722924	GGAGAGA	G	intergenic	*LOC101927973,LINC00161*	.	.	.
21	29780556	C	T	intergenic	*LOC101927973,LINC00161*	.	.	.
21	29672493	TTATTTATG	T	intergenic	*LOC101927973,LINC00161*	.	.	.
12	70130385	TCTCGAA	T	ncRNA_intronic	*LOC101928002*	.	.	.
11	9946844	TA	T	ncRNA_intronic	*LOC101928008*	.	.	.
11	9899440	A	G	ncRNA_intronic	*LOC101928008*	.	.	.
11	9914371	T	G	ncRNA_intronic	*LOC101928008*	.	.	.
11	9914372	T	C	ncRNA_intronic	*LOC101928008*	.	.	.
11	9931158	G	A	ncRNA_intronic	*LOC101928008*	.	.	.
1	153158720	C	G	ncRNA_intronic	*LOC101928009*	.	.	.
1	153158920	C	A	ncRNA_intronic	*LOC101928009*	.	.	.
7	112305178	C	T	ncRNA_intronic	*LOC101928012*	.	.	.
7	112298276	C	T	ncRNA_intronic	*LOC101928012*	.	.	.
7	112357661	T	G	intergenic	*LOC101928012,TMEM168*	.	.	.
7	112377340	G	A	intergenic	*LOC101928012,TMEM168*	.	.	.
14	71085986	TG	T	ncRNA_intronic	*LOC101928075*	.	.	.
X	84464931	C	T	upstream	*LOC101928128*	.	.	.
3	35306334	T	C	ncRNA_intronic	*LOC101928135*	.	.	.
3	35120748	C	T	ncRNA_intronic	*LOC101928135*	.	.	.
3	34948813	A	G	ncRNA_intronic	*LOC101928135*	.	.	.
3	34950474	G	A	ncRNA_intronic	*LOC101928135*	.	.	.
3	34966028	A	G	ncRNA_intronic	*LOC101928135*	.	.	.
3	35018121	T	C	ncRNA_intronic	*LOC101928135*	.	.	.
3	35018122	G	A	ncRNA_intronic	*LOC101928135*	.	.	.
3	35169298	C	T	ncRNA_intronic	*LOC101928135*	.	.	.
3	35169370	A	G	ncRNA_intronic	*LOC101928135*	.	.	.
3	35634359	G	A	intergenic	*LOC101928135,ARPP21*	.	.	.

3	35521287	T	C	intergenic	*LOC101928135,ARPP21*	.	.	.
3	35521288	G	A	intergenic	*LOC101928135,ARPP21*	.	.	.
3	35635390	T	A	intergenic	*LOC101928135,ARPP21*	.	.	.
3	35652018	C	T	intergenic	*LOC101928135,ARPP21*	.	.	.
3	35652902	C	T	intergenic	*LOC101928135,ARPP21*	.	.	.
3	35654848	A	G	intergenic	*LOC101928135,ARPP21*	.	.	.
3	35661639	C	A	intergenic	*LOC101928135,ARPP21*	.	.	.
12	73569398	A	C	ncRNA_intronic	*LOC101928137*	.	.	.
12	73602089	A	G	ncRNA_exonic	*LOC101928137*	.	.	.
12	73891706	A	AAAAAAAAA	intergenic	*LOC101928137,LOC100507377*	.	.	.
12	74380131	T	A	intergenic	*LOC101928137,LOC100507377*	.	.	.
12	74475400	C	T	intergenic	*LOC101928137,LOC100507377*	.	.	.
12	73612651	C	T	intergenic	*LOC101928137,LOC100507377*	.	.	.
12	73665731	T	A	intergenic	*LOC101928137,LOC100507377*	.	.	.
12	73665733	CA	C	intergenic	*LOC101928137,LOC100507377*	.	.	.
12	73698791	A	G	intergenic	*LOC101928137,LOC100507377*	.	.	.
12	73764322	T	C	intergenic	*LOC101928137,LOC100507377*	.	.	.
12	73936850	T	C	intergenic	*LOC101928137,LOC100507377*	.	.	.
12	73969360	G	A	intergenic	*LOC101928137,LOC100507377*	.	.	.
12	74030925	G	A	intergenic	*LOC101928137,LOC100507377*	.	.	.

12	74070157	G	A	intergenic	*LOC101928137,LOC100507377*	.	.	.
12	74128152	A	T	intergenic	*LOC101928137,LOC100507377*	.	.	.
12	74320655	G	T	intergenic	*LOC101928137,LOC100507377*	.	.	.
12	74320980	G	C	intergenic	*LOC101928137,LOC100507377*	.	.	.
12	74366045	T	C	intergenic	*LOC101928137,LOC100507377*	.	.	.
12	74366046	G	A	intergenic	*LOC101928137,LOC100507377*	.	.	.
12	74374879	C	T	intergenic	*LOC101928137,LOC100507377*	.	.	.
12	74375556	C	T	intergenic	*LOC101928137,LOC100507377*	.	.	.
12	74485523	C	T	intergenic	*LOC101928137,LOC100507377*	.	.	.
12	74520825	G	T	intergenic	*LOC101928137,LOC100507377*	.	.	.
10	6741871	A	G	intergenic	*LOC101928150,LINC00706*	.	.	.
15	35098190	T	C	ncRNA_intronic	*LOC101928174*	.	.	.
6	10941824	G	A	ncRNA_intronic	*LOC101928191*	.	.	.
X	5290167	T	A	intergenic	*LOC101928201,NLGN4X*	.	.	.
X	5458718	C	A	intergenic	*LOC101928201,NLGN4X*	.	.	.
X	5033519	G	T	intergenic	*LOC101928201,NLGN4X*	.	.	.
X	5720068	T	A	intergenic	*LOC101928201,NLGN4X*	.	.	.
X	5587307	G	A	intergenic	*LOC101928201,NLGN4X*	.	.	.
X	4620348	T	TAG	intergenic	*LOC101928201,NLGN4X*	.	.	.
X	4861036	T	C	intergenic	*LOC101928201,NLGN4X*	.	.	.

X	5378615	G	A	intergenic	*LOC101928201,NLGN4X*	.	.	.
X	5500473	G	T	intergenic	*LOC101928201,NLGN4X*	.	.	.
X	4751322	T	A	intergenic	*LOC101928201,NLGN4X*	.	.	.
X	4695917	TTCTC	T	intergenic	*LOC101928201,NLGN4X*	.	.	.
X	5326514	A	C	intergenic	*LOC101928201,NLGN4X*	.	.	.
X	5718235	C	A	intergenic	*LOC101928201,NLGN4X*	.	.	.
X	4729539	A	T	intergenic	*LOC101928201,NLGN4X*	.	.	.
X	4729541	A	G	intergenic	*LOC101928201,NLGN4X*	.	.	.
X	5258959	ATGTG	A	intergenic	*LOC101928201,NLGN4X*	.	.	.
X	5519151	A	G	intergenic	*LOC101928201,NLGN4X*	.	.	.
X	5683623	A	G	intergenic	*LOC101928201,NLGN4X*	.	.	.
X	5684675	G	A	intergenic	*LOC101928201,NLGN4X*	.	.	.
X	5745429	C	T	intergenic	*LOC101928201,NLGN4X*	.	.	.
16	76726901	C	T	intergenic	*LOC101928203,MIR4719*	.	.	.
7	124297607	C	A	intergenic	*LOC101928211,GPR37*	.	.	.
7	124297608	C	A	intergenic	*LOC101928211,GPR37*	.	.	.
7	124113670	C	T	intergenic	*LOC101928211,GPR37*	.	.	.
7	124220235	A	G	intergenic	*LOC101928211,GPR37*	.	.	.
7	124264030	A	T	intergenic	*LOC101928211,GPR37*	.	.	.

7	124264032	G	A	intergenic	*LOC101928211,GPR37*	.	.	.
1	248888135	T	C	intergenic	*LOC101928226,LYPD8*	.	.	.
21	44018972	G	A	downstream	*LOC101928233*	.	.	.
1	96830469	C	T	ncRNA_intronic	*LOC101928241*	.	.	.
1	96935562	C	A	intergenic	*LOC101928241,PTBP2*	.	.	.
1	97141435	A	T	intergenic	*LOC101928241,PTBP2*	.	.	.
1	100032723	A	G	intergenic	*LOC101928270,PALMD*	.	.	.
10	9782957	A	C	intergenic	*LOC101928272,LOC101928298*	.	.	.
10	9957923	G	T	intergenic	*LOC101928272,LOC101928298*	.	.	.
10	9725455	ACTCTGACACTCACATTCACT	A	intergenic	*LOC101928272,LOC101928298*	.	.	.
6	69036795	C	G	ncRNA_intronic	*LOC101928280*	.	.	.
6	68996506	G	A	ncRNA_intronic	*LOC101928280*	.	.	.
6	68996844	T	C	ncRNA_intronic	*LOC101928280*	.	.	.
6	68996941	A	G	ncRNA_intronic	*LOC101928280*	.	.	.
6	68999561	A	G	ncRNA_intronic	*LOC101928280*	.	.	.
6	69011787	T	A	ncRNA_intronic	*LOC101928280*	.	.	.
6	69240926	C	A	intergenic	*LOC101928280,LOC101928307*	.	.	.
6	69065386	G	A	intergenic	*LOC101928280,LOC101928307*	.	.	.
6	69165297	T	A	intergenic	*LOC101928280,LOC101928307*	.	.	.
6	69166775	A	G	intergenic	*LOC101928280,LOC101928307*	.	.	.
6	69173992	G	A	intergenic	*LOC101928280,LOC101928307*	.	.	.
6	69311507	T	G	intergenic	*LOC101928280,LOC101928307*	.	.	.

6	69332168	G	A	intergenic	*LOC101928280,LOC101928307*	.	.	.
7	125002706	C	A	ncRNA_intronic	*LOC101928283*	.	.	.
7	124915401	C	G	ncRNA_intronic	*LOC101928283*	.	.	.
7	124972740	T	A	ncRNA_intronic	*LOC101928283*	.	.	.
7	125955833	A	T	intergenic	*LOC101928283,GRM8*	.	.	.
7	125911795	G	T	intergenic	*LOC101928283,GRM8*	.	.	.
7	126034246	G	T	intergenic	*LOC101928283,GRM8*	.	.	.
7	125473118	A	C	intergenic	*LOC101928283,GRM8*	.	.	.
7	125090871	T	G	intergenic	*LOC101928283,GRM8*	.	.	.
7	125278214	G	T	intergenic	*LOC101928283,GRM8*	.	.	.
7	125854977	C	A	intergenic	*LOC101928283,GRM8*	.	.	.
7	125412026	C	A	intergenic	*LOC101928283,GRM8*	.	.	.
7	125118892	T	TAC	intergenic	*LOC101928283,GRM8*	.	.	.
7	125425198	T	C	intergenic	*LOC101928283,GRM8*	.	.	.
7	125478547	T	A	intergenic	*LOC101928283,GRM8*	.	.	.
7	125918485	T	A	intergenic	*LOC101928283,GRM8*	.	.	.
7	125920127	A	T	intergenic	*LOC101928283,GRM8*	.	.	.
7	125920741	T	C	intergenic	*LOC101928283,GRM8*	.	.	.
7	125936477	T	C	intergenic	*LOC101928283,GRM8*	.	.	.
7	125936478	G	A	intergenic	*LOC101928283,GRM8*	.	.	.

10	10159450	T	C	intergenic	*LOC101928298,LOC101928322*	.	.	.
10	10166796	TTTAC	T	intergenic	*LOC101928298,LOC101928322*	.	.	.
10	10330233	A	G	intergenic	*LOC101928298,LOC101928322*	.	.	.
4	4931079	T	C	ncRNA_intronic	*LOC101928306*	.	.	.
4	4931080	G	A	ncRNA_intronic	*LOC101928306*	.	.	.
4	4931957	A	T	ncRNA_intronic	*LOC101928306*	.	.	.
4	5014577	A	C	intergenic	*LOC101928306,CYTL1*	.	.	.
4	4999499	A	G	intergenic	*LOC101928306,CYTL1*	.	.	.
4	4999500	C	T	intergenic	*LOC101928306,CYTL1*	.	.	.
4	5000351	G	A	intergenic	*LOC101928306,CYTL1*	.	.	.
4	5000352	C	T	intergenic	*LOC101928306,CYTL1*	.	.	.
4	173603755	A	G	ncRNA_intronic	*LOC101928314*	.	.	.
10	10497326	C	A	ncRNA_intronic	*LOC101928322*	.	.	.
10	10600675	C	A	intergenic	*LOC101928322,SFTA1P*	.	.	.
2	217817148	C	G	intergenic	*LOC101928327,DIRC3-AS1*	.	.	.
2	217842636	G	A	intergenic	*LOC101928327,DIRC3-AS1*	.	.	.
2	218099905	T	C	intergenic	*LOC101928327,DIRC3-AS1*	.	.	.
X	107146132	C	G	ncRNA_intronic	*LOC101928335*	.	.	.
X	107187508	A	C	intergenic	*LOC101928335,TEX13B*	.	.	.
X	107187559	C	T	intergenic	*LOC101928335,TEX13B*	.	.	.
X	107188513	T	A	intergenic	*LOC101928335,TEX13B*	.	.	.
X	107197830	G	T	intergenic	*LOC101928335,TEX13B*	.	.	.

X	107199078	C	CAT	intergenic	*LOC101928335,TEX13B*	.	.	.
X	107201212	C	T	intergenic	*LOC101928335,TEX13B*	.	.	.
X	108176570	G	C	intergenic	*LOC101928358,MIR6087*	.	.	.
X	108047291	A	ATAAACTGC	intergenic	*LOC101928358,MIR6087*	.	.	.
X	108098956	C	T	intergenic	*LOC101928358,MIR6087*	.	.	.
X	108149324	T	C	intergenic	*LOC101928358,MIR6087*	.	.	.
X	108150735	T	G	intergenic	*LOC101928358,MIR6087*	.	.	.
X	108197485	T	C	intergenic	*LOC101928358,MIR6087*	.	.	.
X	108236136	A	G	intergenic	*LOC101928358,MIR6087*	.	.	.
X	108257232	A	C	intergenic	*LOC101928358,MIR6087*	.	.	.
X	108265863	C	T	intergenic	*LOC101928358,MIR6087*	.	.	.
X	108265865	C	A	intergenic	*LOC101928358,MIR6087*	.	.	.
X	108278678	G	A	intergenic	*LOC101928358,MIR6087*	.	.	.
1	160907073	A	G	ncRNA_intronic	*LOC101928372*	.	.	.
9	69804355	G	T	intergenic	*LOC101928381,FOXD4L5*	.	.	.
9	69782846	G	T	intergenic	*LOC101928381,FOXD4L5*	.	.	.
9	69779640	T	A	intergenic	*LOC101928381,FOXD4L5*	.	.	.
9	69967563	T	A	intergenic	*LOC101928381,FOXD4L5*	.	.	.
9	70102626	A	AAAT	intergenic	*LOC101928381,FOXD4L5*	.	.	.

9	69813073	T	C	intergenic	*LOC101928381,FOXD4L5*	.	.	.
9	69814805	A	C	intergenic	*LOC101928381,FOXD4L5*	.	.	.
9	69986465	G	T	intergenic	*LOC101928381,FOXD4L5*	.	.	.
9	66945248	A	G	intergenic	*LOC101928381,LOC286297*	.	.	.
7	56873493	C	G	intergenic	*LOC101928401,LOC401357*	.	.	.
7	56873495	C	T	intergenic	*LOC101928401,LOC401357*	.	.	.
7	56670950	A	G	intergenic	*LOC101928401,LOC401357*	.	.	.
12	132950494	C	T	intergenic	*LOC101928416,FBRSL1*	.	.	.
21	40473640	A	G	intergenic	*LOC101928435,PSMG1*	.	.	.
21	40505258	C	G	intergenic	*LOC101928435,PSMG1*	.	.	.
1	104029052	A	G	intronic	*LOC101928436*	.	.	.
1	104029173	G	A	intronic	*LOC101928436*	.	.	.
1	104035562	C	T	intronic	*LOC101928436*	.	.	.
1	104035757	C	G	intronic	*LOC101928436*	.	.	.
1	104053061	A	G	intronic	*LOC101928436*	.	.	.
X	112687563	A	C	ncRNA_intronic	*LOC101928437*	.	.	.
X	112648783	C	T	ncRNA_intronic	*LOC101928437*	.	.	.
X	112343123	T	C	ncRNA_intronic	*LOC101928437*	.	.	.
X	112343124	G	A	ncRNA_intronic	*LOC101928437*	.	.	.
X	112403116	A	T	ncRNA_intronic	*LOC101928437*	.	.	.
X	112443697	T	A	ncRNA_intronic	*LOC101928437*	.	.	.
X	112452046	G	A	ncRNA_intronic	*LOC101928437*	.	.	.
X	112452606	T	A	ncRNA_intronic	*LOC101928437*	.	.	.

X	112536820	CTAACCTGCATATTGTGCACATGTACCCTAAAACTTAATGTATAA	C	ncRNA_intronic	*LOC101928437*	.	.	.
X	112582220	G	A	ncRNA_intronic	*LOC101928437*	.	.	.
X	112616210	T	TAC	ncRNA_intronic	*LOC101928437*	.	.	.
X	112691559	A	G	ncRNA_intronic	*LOC101928437*	.	.	.
X	112848270	A	G	intergenic	*LOC101928437,XACT*	.	.	.
X	112813145	T	TG	intergenic	*LOC101928437,XACT*	.	.	.
9	102459051	C	T	ncRNA_intronic	*LOC101928438*	.	.	.
12	23571644	G	A	intergenic	*LOC101928441,SOX5*	.	.	.
12	23543104	A	G	intergenic	*LOC101928441,SOX5*	.	.	.
12	23546287	C	A	intergenic	*LOC101928441,SOX5*	.	.	.
12	23571501	A	G	intergenic	*LOC101928441,SOX5*	.	.	.
12	23601314	C	T	intergenic	*LOC101928441,SOX5*	.	.	.
16	82862269	G	GTGTA	ncRNA_intronic	*LOC101928446*	.	.	.
12	82363744	T	C	ncRNA_intronic	*LOC101928449*	.	.	.
12	82653136	T	G	intergenic	*LOC101928449,CCDC59*	.	.	.
12	82645092	C	A	intergenic	*LOC101928449,CCDC59*	.	.	.
12	82661172	A	G	intergenic	*LOC101928449,CCDC59*	.	.	.
12	82704290	T	C	intergenic	*LOC101928449,CCDC59*	.	.	.
12	82681372	G	T	intergenic	*LOC101928449,CCDC59*	.	.	.
12	82455992	C	A	intergenic	*LOC101928449,CCDC59*	.	.	.

12	82442289	A	G	intergenic	*LOC101928449,CCDC59*	.	.	.
12	82444027	C	A	intergenic	*LOC101928449,CCDC59*	.	.	.
12	82668313	A	C	intergenic	*LOC101928449,CCDC59*	.	.	.
12	82668315	G	T	intergenic	*LOC101928449,CCDC59*	.	.	.
12	82668560	A	G	intergenic	*LOC101928449,CCDC59*	.	.	.
12	82707288	T	C	intergenic	*LOC101928449,CCDC59*	.	.	.
1	30233810	G	T	intergenic	*LOC101928460,LOC101929406*	.	.	.
1	29749918	C	A	intergenic	*LOC101928460,LOC101929406*	.	.	.
1	30382120	A	T	intergenic	*LOC101928460,LOC101929406*	.	.	.
1	30238299	C	T	intergenic	*LOC101928460,LOC101929406*	.	.	.
1	30470539	T	C	intergenic	*LOC101928460,LOC101929406*	.	.	.
1	106137163	G	A	ncRNA_intronic	*LOC101928476*	.	.	.
1	106640256	T	A	intergenic	*LOC101928476,PRMT6*	.	.	.
1	106556888	G	A	intergenic	*LOC101928476,PRMT6*	.	.	.
1	106702171	G	A	intergenic	*LOC101928476,PRMT6*	.	.	.
1	107169216	C	CT	intergenic	*LOC101928476,PRMT6*	.	.	.
1	107490315	G	C	intergenic	*LOC101928476,PRMT6*	.	.	.
1	106412885	C	A	intergenic	*LOC101928476,PRMT6*	.	.	.
1	106976588	C	A	intergenic	*LOC101928476,PRMT6*	.	.	.

1	107160249	T	C	intergenic	*LOC101928476,PRMT6*	.	.	.
1	106579156	T	C	intergenic	*LOC101928476,PRMT6*	.	.	.
1	107163981	T	A	intergenic	*LOC101928476,PRMT6*	.	.	.
1	106364637	G	A	intergenic	*LOC101928476,PRMT6*	.	.	.
1	106424303	A	T	intergenic	*LOC101928476,PRMT6*	.	.	.
1	106489170	T	C	intergenic	*LOC101928476,PRMT6*	.	.	.
1	106491319	A	C	intergenic	*LOC101928476,PRMT6*	.	.	.
1	106509373	T	A	intergenic	*LOC101928476,PRMT6*	.	.	.
1	106709936	A	G	intergenic	*LOC101928476,PRMT6*	.	.	.
1	106714609	C	A	intergenic	*LOC101928476,PRMT6*	.	.	.
1	106756087	C	T	intergenic	*LOC101928476,PRMT6*	.	.	.
5	57145634	G	A	intergenic	*LOC101928505,LOC101928539*	.	.	.
5	57164039	T	C	intergenic	*LOC101928505,LOC101928539*	.	.	.
6	75010330	CTATATATACACACATATGTATGTGTATATATGTTTATGTACTATATATACACATACATATGTGTG	C	ncRNA_intronic	*LOC101928516*	.	.	.
6	75309272	T	A	ncRNA_intronic	*LOC101928516*	.	.	.
6	75115198	G	GATCCCCTC	ncRNA_intronic	*LOC101928516*	.	.	.
6	75205730	T	C	ncRNA_intronic	*LOC101928516*	.	.	.

6	75124234	ATGTG	A	ncRNA_intronic	*LOC101928516*	.	.	.
6	75268954	G	T	ncRNA_intronic	*LOC101928516*	.	.	.
6	74789658	C	T	ncRNA_intronic	*LOC101928516*	.	.	.
6	74789909	A	G	ncRNA_intronic	*LOC101928516*	.	.	.
6	74792521	C	T	ncRNA_intronic	*LOC101928516*	.	.	.
6	74899459	C	A	ncRNA_intronic	*LOC101928516*	.	.	.
6	74918728	T	C	ncRNA_intronic	*LOC101928516*	.	.	.
6	74971740	C	T	ncRNA_intronic	*LOC101928516*	.	.	.
6	75049773	T	A	ncRNA_intronic	*LOC101928516*	.	.	.
6	75050303	A	T	ncRNA_intronic	*LOC101928516*	.	.	.
6	75086292	C	A	ncRNA_intronic	*LOC101928516*	.	.	.
6	75091699	T	TC	ncRNA_intronic	*LOC101928516*	.	.	.
6	75091700	T	A	ncRNA_intronic	*LOC101928516*	.	.	.
6	75111322	C	T	ncRNA_intronic	*LOC101928516*	.	.	.
6	75114596	G	A	ncRNA_intronic	*LOC101928516*	.	.	.
6	75115311	G	T	ncRNA_intronic	*LOC101928516*	.	.	.
6	75197682	T	C	ncRNA_intronic	*LOC101928516*	.	.	.
6	75206651	C	G	ncRNA_intronic	*LOC101928516*	.	.	.
6	75226278	TA	T	ncRNA_intronic	*LOC101928516*	.	.	.
6	75436397	C	G	intergenic	*LOC101928516,COL12A1*	.	.	.
6	75497074	C	T	intergenic	*LOC101928516,COL12A1*	.	.	.
6	75497075	A	G	intergenic	*LOC101928516,COL12A1*	.	.	.
6	75546431	A	G	intergenic	*LOC101928516,COL12A1*	.	.	.
6	75596185	T	G	intergenic	*LOC101928516,COL12A1*	.	.	.
6	75596374	C	T	intergenic	*LOC101928516,COL12A1*	.	.	.
6	75596520	A	G	intergenic	*LOC101928516,COL12A1*	.	.	.
6	75742053	C	G	intergenic	*LOC101928516,COL12A1*	.	.	.
6	75742657	T	G	intergenic	*LOC101928516,COL12A1*	.	.	.
6	19147326	G	A	ncRNA_intronic	*LOC101928519*	.	.	.

6	19188415	C	T	intergenic	*LOC101928519,ID4*	.	.	.
6	19453998	A	G	intergenic	*LOC101928519,ID4*	.	.	.
6	19377368	T	A	intergenic	*LOC101928519,ID4*	.	.	.
6	19350312	A	G	intergenic	*LOC101928519,ID4*	.	.	.
6	19458461	G	T	intergenic	*LOC101928519,ID4*	.	.	.
6	19500281	T	C	intergenic	*LOC101928519,ID4*	.	.	.
6	19242376	C	T	intergenic	*LOC101928519,ID4*	.	.	.
6	19263547	C	T	intergenic	*LOC101928519,ID4*	.	.	.
6	19485284	A	G	intergenic	*LOC101928519,ID4*	.	.	.
6	19486359	G	A	intergenic	*LOC101928519,ID4*	.	.	.
6	19486706	C	A	intergenic	*LOC101928519,ID4*	.	.	.
6	19487347	G	A	intergenic	*LOC101928519,ID4*	.	.	.
6	19508933	A	G	intergenic	*LOC101928519,ID4*	.	.	.
6	19526989	G	T	intergenic	*LOC101928519,ID4*	.	.	.
6	19586244	T	A	intergenic	*LOC101928519,ID4*	.	.	.
6	19621479	A	G	intergenic	*LOC101928519,ID4*	.	.	.
6	19621481	G	A	intergenic	*LOC101928519,ID4*	.	.	.
6	19624121	C	T	intergenic	*LOC101928519,ID4*	.	.	.
6	19702636	G	A	intergenic	*LOC101928519,ID4*	.	.	.

6	19745526	G	A	intergenic	*LOC101928519,ID4*	.	.	.
6	19746352	T	A	intergenic	*LOC101928519,ID4*	.	.	.
9	106820681	T	C	intergenic	*LOC101928523,SMC2-AS1*	.	.	.
9	106820884	G	A	intergenic	*LOC101928523,SMC2-AS1*	.	.	.
5	57263140	G	C	intergenic	*LOC101928539,LOC101928569*	.	.	.
5	57264139	A	G	intergenic	*LOC101928539,LOC101928569*	.	.	.
5	57264845	T	C	intergenic	*LOC101928539,LOC101928569*	.	.	.
5	57264846	A	G	intergenic	*LOC101928539,LOC101928569*	.	.	.
5	57295072	A	G	intergenic	*LOC101928539,LOC101928569*	.	.	.
5	57295742	C	T	intergenic	*LOC101928539,LOC101928569*	.	.	.
5	57348168	T	A	intergenic	*LOC101928539,LOC101928569*	.	.	.
5	57396665	C	T	intergenic	*LOC101928539,LOC101928569*	.	.	.
6	76120437	T	C	ncRNA_intronic	*LOC101928540*	.	.	.
6	76144300	G	T	ncRNA_intronic	*LOC101928540*	.	.	.
6	76144664	C	T	ncRNA_intronic	*LOC101928540*	.	.	.
4	175794635	C	A	ncRNA_intronic	*LOC101928551*	.	.	.
1	168507397	T	C	intergenic	*LOC101928565,XCL2*	.	.	.
5	57496048	A	G	intergenic	*LOC101928569,PLK2*	.	.	.
5	57597695	A	AATATATATA	intergenic	*LOC101928569,PLK2*	.	.	.
5	57666791	A	C	intergenic	*LOC101928569,PLK2*	.	.	.
5	57714597	C	A	intergenic	*LOC101928569,PLK2*	.	.	.

5	57724273	G	A	intergenic	*LOC101928569,PLK2*	.	.	.
5	57490344	G	A	intergenic	*LOC101928569,PLK2*	.	.	.
5	57518383	A	G	intergenic	*LOC101928569,PLK2*	.	.	.
5	57588773	C	A	intergenic	*LOC101928569,PLK2*	.	.	.
5	57588775	T	C	intergenic	*LOC101928569,PLK2*	.	.	.
7	36162044	A	G	intergenic	*LOC101928618,EEPD1*	.	.	.
7	36164276	G	C	intergenic	*LOC101928618,EEPD1*	.	.	.
4	34032776	C	A	ncRNA_intronic	*LOC101928622*	.	.	.
4	33991901	T	C	ncRNA_intronic	*LOC101928622*	.	.	.
4	34010817	G	T	ncRNA_intronic	*LOC101928622*	.	.	.
4	34037028	A	C	ncRNA_intronic	*LOC101928622*	.	.	.
4	34019489	G	T	ncRNA_intronic	*LOC101928622*	.	.	.
4	34027017	C	T	ncRNA_intronic	*LOC101928622*	.	.	.
4	34027019	A	G	ncRNA_intronic	*LOC101928622*	.	.	.
4	34693998	C	T	intergenic	*LOC101928622,ARAP2*	.	.	.
4	35516913	G	A	intergenic	*LOC101928622,ARAP2*	.	.	.
4	34470073	T	A	intergenic	*LOC101928622,ARAP2*	.	.	.
4	34850489	C	T	intergenic	*LOC101928622,ARAP2*	.	.	.
4	35257496	AATAT	A	intergenic	*LOC101928622,ARAP2*	.	.	.
4	35383431	TA	T	intergenic	*LOC101928622,ARAP2*	.	.	.
4	35643595	C	A	intergenic	*LOC101928622,ARAP2*	.	.	.
4	36026051	C	T	intergenic	*LOC101928622,ARAP2*	.	.	.

4	34492164	T	C	intergenic	*LOC101928622,ARAP2*	.	.	.
4	34510821	T	A	intergenic	*LOC101928622,ARAP2*	.	.	.
4	34580109	T	C	intergenic	*LOC101928622,ARAP2*	.	.	.
4	34614893	C	A	intergenic	*LOC101928622,ARAP2*	.	.	.
4	34642813	C	T	intergenic	*LOC101928622,ARAP2*	.	.	.
4	34828443	C	T	intergenic	*LOC101928622,ARAP2*	.	.	.
4	34867317	G	A	intergenic	*LOC101928622,ARAP2*	.	.	.
4	35474389	G	A	intergenic	*LOC101928622,ARAP2*	.	.	.
4	35542524	T	C	intergenic	*LOC101928622,ARAP2*	.	.	.
4	35054871	C	A	intergenic	*LOC101928622,ARAP2*	.	.	.
4	34394673	G	T	intergenic	*LOC101928622,ARAP2*	.	.	.
4	34583577	T	A	intergenic	*LOC101928622,ARAP2*	.	.	.
4	35219853	T	TATATTACA(	intergenic	*LOC101928622,ARAP2*	.	.	.
4	35346221	G	T	intergenic	*LOC101928622,ARAP2*	.	.	.
4	35814109	G	T	intergenic	*LOC101928622,ARAP2*	.	.	.
4	34100493	G	T	intergenic	*LOC101928622,ARAP2*	.	.	.
4	34107625	T	A	intergenic	*LOC101928622,ARAP2*	.	.	.
4	34199192	C	T	intergenic	*LOC101928622,ARAP2*	.	.	.
4	34397237	C	CAG	intergenic	*LOC101928622,ARAP2*	.	.	.

4	34418313	C	A	intergenic	*LOC101928622,ARAP2*	.	.	.
4	34570604	A	C	intergenic	*LOC101928622,ARAP2*	.	.	.
4	34932774	G	A	intergenic	*LOC101928622,ARAP2*	.	.	.
4	35053823	A	G	intergenic	*LOC101928622,ARAP2*	.	.	.
4	35078983	T	A	intergenic	*LOC101928622,ARAP2*	.	.	.
4	35219356	G	T	intergenic	*LOC101928622,ARAP2*	.	.	.
4	35225610	C	A	intergenic	*LOC101928622,ARAP2*	.	.	.
4	35488294	C	T	intergenic	*LOC101928622,ARAP2*	.	.	.
4	35634928	C	A	intergenic	*LOC101928622,ARAP2*	.	.	.
4	34113305	G	C	intergenic	*LOC101928622,ARAP2*	.	.	.
4	34384335	A	T	intergenic	*LOC101928622,ARAP2*	.	.	.
4	34443143	C	G	intergenic	*LOC101928622,ARAP2*	.	.	.
4	34983638	T	C	intergenic	*LOC101928622,ARAP2*	.	.	.
4	36016010	G	A	intergenic	*LOC101928622,ARAP2*	.	.	.
4	34862021	TTCTC	T	intergenic	*LOC101928622,ARAP2*	.	.	.
4	35492472	T	TAG	intergenic	*LOC101928622,ARAP2*	.	.	.
4	34050805	TACAC	T	intergenic	*LOC101928622,ARAP2*	.	.	.
4	34251732	C	T	intergenic	*LOC101928622,ARAP2*	.	.	.
4	34290135	G	T	intergenic	*LOC101928622,ARAP2*	.	.	.

4	34290136	A	G	intergenic	*LOC101928622,ARAP2*	.	.	.
4	34476432	G	C	intergenic	*LOC101928622,ARAP2*	.	.	.
4	34624552	C	T	intergenic	*LOC101928622,ARAP2*	.	.	.
4	34624553	A	G	intergenic	*LOC101928622,ARAP2*	.	.	.
4	34839825	T	C	intergenic	*LOC101928622,ARAP2*	.	.	.
4	34839827	T	C	intergenic	*LOC101928622,ARAP2*	.	.	.
4	34845879	C	A	intergenic	*LOC101928622,ARAP2*	.	.	.
4	34876979	G	A	intergenic	*LOC101928622,ARAP2*	.	.	.
4	35036826	C	CTTTTTCTTT	intergenic	*LOC101928622,ARAP2*	.	.	.
4	35127001	T	C	intergenic	*LOC101928622,ARAP2*	.	.	.
4	35127002	G	C	intergenic	*LOC101928622,ARAP2*	.	.	.
4	35289929	T	C	intergenic	*LOC101928622,ARAP2*	.	.	.
4	35289930	G	A	intergenic	*LOC101928622,ARAP2*	.	.	.
4	35324241	A	C	intergenic	*LOC101928622,ARAP2*	.	.	.
4	35352789	C	T	intergenic	*LOC101928622,ARAP2*	.	.	.
4	35586694	T	A	intergenic	*LOC101928622,ARAP2*	.	.	.
4	35607167	T	A	intergenic	*LOC101928622,ARAP2*	.	.	.
4	35613892	TAAAAATCCATTTTTGATTCATC	T	intergenic	*LOC101928622,ARAP2*	.	.	.

4	35634910	A	G	intergenic	*LOC101928622,ARAP2*	.	.	.
4	35665415	C	T	intergenic	*LOC101928622,ARAP2*	.	.	.
4	35665417	G	C	intergenic	*LOC101928622,ARAP2*	.	.	.
4	35833604	T	A	intergenic	*LOC101928622,ARAP2*	.	.	.
4	35883304	C	A	intergenic	*LOC101928622,ARAP2*	.	.	.
4	35897297	A	G	intergenic	*LOC101928622,ARAP2*	.	.	.
4	35897299	T	C	intergenic	*LOC101928622,ARAP2*	.	.	.
4	36042813	C	A	intergenic	*LOC101928622,ARAP2*	.	.	.
16	87274735	G	A	intergenic	*LOC101928708,LOC101928682*	.	.	.
13	112921644	T	C	ncRNA_intronic	*LOC101928730*	.	.	.
13	112921646	A	T	ncRNA_intronic	*LOC101928730*	.	.	.
3	178821383	T	A	ncRNA_intronic	*LOC101928739*	.	.	.
1	175860012	G	A	intergenic	*LOC101928751,RFWD2*	.	.	.
14	86433580	T	C	ncRNA_intronic	*LOC101928767*	.	.	.
14	86412501	G	A	ncRNA_intronic	*LOC101928767*	.	.	.
14	86510521	C	T	ncRNA_intronic	*LOC101928767*	.	.	.
14	86511007	A	G	ncRNA_intronic	*LOC101928767*	.	.	.
14	86511009	G	A	ncRNA_intronic	*LOC101928767*	.	.	.
14	86511902	T	A	ncRNA_intronic	*LOC101928767*	.	.	.
14	86543267	G	A	ncRNA_intronic	*LOC101928767*	.	.	.
14	86560059	G	A	ncRNA_intronic	*LOC101928767*	.	.	.
14	86620790	A	G	intergenic	*LOC101928767,LOC283585*	.	.	.
14	86875947	G	T	intergenic	*LOC101928767,LOC283585*	.	.	.
14	87355225	G	T	intergenic	*LOC101928767,LOC283585*	.	.	.

14	86650373	A	G	intergenic	*LOC101928767,LOC283585*	.	.	.
14	86727584	G	T	intergenic	*LOC101928767,LOC283585*	.	.	.
14	86756368	C	A	intergenic	*LOC101928767,LOC283585*	.	.	.
14	87270137	T	TTATA	intergenic	*LOC101928767,LOC283585*	.	.	.
14	87337186	T	C	intergenic	*LOC101928767,LOC283585*	.	.	.
14	86619430	CA	C	intergenic	*LOC101928767,LOC283585*	.	.	.
14	86800176	T	G	intergenic	*LOC101928767,LOC283585*	.	.	.
14	86662762	G	A	intergenic	*LOC101928767,LOC283585*	.	.	.
14	86613564	A	C	intergenic	*LOC101928767,LOC283585*	.	.	.
14	87064817	G	T	intergenic	*LOC101928767,LOC283585*	.	.	.
14	87306767	C	T	intergenic	*LOC101928767,LOC283585*	.	.	.
14	87318121	T	C	intergenic	*LOC101928767,LOC283585*	.	.	.
14	87111521	G	C	intergenic	*LOC101928767,LOC283585*	.	.	.
14	87339347	C	A	intergenic	*LOC101928767,LOC283585*	.	.	.
14	86654074	C	T	intergenic	*LOC101928767,LOC283585*	.	.	.
14	86667914	G	T	intergenic	*LOC101928767,LOC283585*	.	.	.
14	86667916	A	C	intergenic	*LOC101928767,LOC283585*	.	.	.
14	86732928	G	A	intergenic	*LOC101928767,LOC283585*	.	.	.
14	86733820	C	G	intergenic	*LOC101928767,LOC283585*	.	.	.

14	86733821	A	G	intergenic	*LOC101928767,LOC283585*	.	.	.
14	86781852	G	A	intergenic	*LOC101928767,LOC283585*	.	.	.
14	86938087	G	C	intergenic	*LOC101928767,LOC283585*	.	.	.
14	86940636	C	A	intergenic	*LOC101928767,LOC283585*	.	.	.
14	86940957	G	A	intergenic	*LOC101928767,LOC283585*	.	.	.
14	87076073	A	T	intergenic	*LOC101928767,LOC283585*	.	.	.
14	87078062	A	G	intergenic	*LOC101928767,LOC283585*	.	.	.
14	87161730	G	T	intergenic	*LOC101928767,LOC283585*	.	.	.
14	87161856	A	C	intergenic	*LOC101928767,LOC283585*	.	.	.
14	87214777	C	G	intergenic	*LOC101928767,LOC283585*	.	.	.
14	87215518	A	G	intergenic	*LOC101928767,LOC283585*	.	.	.
5	65827810	G	T	intergenic	*LOC101928769,MAST4*	.	.	.
9	118530706	G	A	intergenic	*LOC101928775,LINC00474*	.	.	.
9	118531023	C	T	intergenic	*LOC101928775,LINC00474*	.	.	.
9	118562883	A	G	intergenic	*LOC101928775,LINC00474*	.	.	.
9	118563099	T	C	intergenic	*LOC101928775,LINC00474*	.	.	.
1	177846606	G	T	intergenic	*LOC101928778,SEC16B*	.	.	.
1	177688824	T	C	intergenic	*LOC101928778,SEC16B*	.	.	.
1	177684519	C	T	intergenic	*LOC101928778,SEC16B*	.	.	.

1	177684521	G	A	intergenic	*LOC101928778,SEC16B*	.	.	.
1	177733420	A	G	intergenic	*LOC101928778,SEC16B*	.	.	.
1	177774002	A	T	intergenic	*LOC101928778,SEC16B*	.	.	.
1	177861615	T	A	intergenic	*LOC101928778,SEC16B*	.	.	.
7	131600185	G	A	ncRNA_intronic	*LOC101928782*	.	.	.
7	131782727	G	A	intergenic	*LOC101928782,PLXNA4*	.	.	.
7	131665053	G	A	intergenic	*LOC101928782,PLXNA4*	.	.	.
9	120438355	C	T	intergenic	*LOC101928797,TLR4*	.	.	.
4	59908329	A	G	intergenic	*LOC101928851,MIR548AG1*	.	.	.
4	60072371	G	T	intergenic	*LOC101928851,MIR548AG1*	.	.	.
4	59845019	A	G	intergenic	*LOC101928851,MIR548AG1*	.	.	.
4	59718600	C	T	intergenic	*LOC101928851,MIR548AG1*	.	.	.
4	59718765	C	T	intergenic	*LOC101928851,MIR548AG1*	.	.	.
4	59795427	A	G	intergenic	*LOC101928851,MIR548AG1*	.	.	.
4	59947723	A	G	intergenic	*LOC101928851,MIR548AG1*	.	.	.
4	59975489	T	A	intergenic	*LOC101928851,MIR548AG1*	.	.	.
4	59976083	A	G	intergenic	*LOC101928851,MIR548AG1*	.	.	.
4	60058243	A	G	intergenic	*LOC101928851,MIR548AG1*	.	.	.
4	60277473	A	G	intergenic	*LOC101928851,MIR548AG1*	.	.	.

4	60288186	A	T	intergenic	*LOC101928851,MIR548AG1*	.	.	.
4	60296713	C	T	intergenic	*LOC101928851,MIR548AG1*	.	.	.
4	60322235	A	G	intergenic	*LOC101928851,MIR548AG1*	.	.	.
4	60354542	C	T	intergenic	*LOC101928851,MIR548AG1*	.	.	.
4	60398075	A	G	intergenic	*LOC101928851,MIR548AG1*	.	.	.
4	59231361	T	C	intergenic	*LOC101928851,NONE*	.	.	.
4	59476350	G	A	intergenic	*LOC101928851,NONE*	.	.	.
4	58798843	C	T	intergenic	*LOC101928851,NONE*	.	.	.
4	59529918	G	T	intergenic	*LOC101928851,NONE*	.	.	.
4	59663851	C	G	intergenic	*LOC101928851,NONE*	.	.	.
4	58735327	CATAT	C	intergenic	*LOC101928851,NONE*	.	.	.
4	58335838	A	T	intergenic	*LOC101928851,NONE*	.	.	.
4	58384893	T	C	intergenic	*LOC101928851,NONE*	.	.	.
4	58399206	T	G	intergenic	*LOC101928851,NONE*	.	.	.
4	58485835	C	T	intergenic	*LOC101928851,NONE*	.	.	.
4	58489288	A	G	intergenic	*LOC101928851,NONE*	.	.	.
4	58582540	C	T	intergenic	*LOC101928851,NONE*	.	.	.
4	58621178	G	A	intergenic	*LOC101928851,NONE*	.	.	.
4	58677844	G	A	intergenic	*LOC101928851,NONE*	.	.	.

4	58788158	T	C	intergenic	*LOC101928851,NONE*	.	.	.
4	58789162	G	A	intergenic	*LOC101928851,NONE*	.	.	.
4	58893704	C	A	intergenic	*LOC101928851,NONE*	.	.	.
4	58966100	C	T	intergenic	*LOC101928851,NONE*	.	.	.
4	59018610	T	C	intergenic	*LOC101928851,NONE*	.	.	.
4	59019991	A	G	intergenic	*LOC101928851,NONE*	.	.	.
4	59019992	A	G	intergenic	*LOC101928851,NONE*	.	.	.
4	59206275	T	C	intergenic	*LOC101928851,NONE*	.	.	.
4	59240490	T	C	intergenic	*LOC101928851,NONE*	.	.	.
4	59248433	T	G	intergenic	*LOC101928851,NONE*	.	.	.
4	59248899	T	G	intergenic	*LOC101928851,NONE*	.	.	.
4	59270940	T	G	intergenic	*LOC101928851,NONE*	.	.	.
4	59271879	T	G	intergenic	*LOC101928851,NONE*	.	.	.
4	59448827	C	T	intergenic	*LOC101928851,NONE*	.	.	.
4	59448952	T	C	intergenic	*LOC101928851,NONE*	.	.	.
4	59575100	A	G	intergenic	*LOC101928851,NONE*	.	.	.
5	67454070	A	C	intergenic	*LOC101928858,LOC102467655*	.	.	.
5	67428081	C	T	intergenic	*LOC101928858,LOC102467655*	.	.	.
5	67133156	T	C	intergenic	*LOC101928858,LOC102467655*	.	.	.

5	67162538	A	G	intergenic	*LOC101928858,LOC102467655*	.	.	.
5	67188195	A	G	intergenic	*LOC101928858,LOC102467655*	.	.	.
5	67225129	A	G	intergenic	*LOC101928858,LOC102467655*	.	.	.
5	67231172	G	A	intergenic	*LOC101928858,LOC102467655*	.	.	.
5	67359996	T	C	intergenic	*LOC101928858,LOC102467655*	.	.	.
5	67438867	T	A	intergenic	*LOC101928858,LOC102467655*	.	.	.
5	67438868	G	A	intergenic	*LOC101928858,LOC102467655*	.	.	.
16	88300166	C	T	intergenic	*LOC101928880,ZNF469*	.	.	.
16	88292714	A	G	intergenic	*LOC101928880,ZNF469*	.	.	.
5	68263524	TGAC	T	downstream	*LOC101928885*	.	.	.
5	68316343	G	A	ncRNA_intronic	*LOC101928885*	.	.	.
6	88611326	G	A	ncRNA_intronic	*LOC101928911*	.	.	.
6	88553303	C	G	ncRNA_intronic	*LOC101928911*	.	.	.
6	89237282	T	TAGA	ncRNA_exonic	*LOC101928936*	.	.	.
6	89290253	G	C	intergenic	*LOC101928936,RNGTT*	.	.	.
11	80970888	A	T	intergenic	*LOC101928944,MIR4300HG*	.	.	.
11	80854655	C	T	intergenic	*LOC101928944,MIR4300HG*	.	.	.
11	80883720	G	A	intergenic	*LOC101928944,MIR4300HG*	.	.	.
11	81496034	C	G	intergenic	*LOC101928944,MIR4300HG*	.	.	.
11	81356375	A	C	intergenic	*LOC101928944,MIR4300HG*	.	.	.
11	80705812	AACAC	A	intergenic	*LOC101928944,MIR4300HG*	.	.	.

11	81090344	T	G	intergenic	*LOC101928944,MIR4300HG*	.	.	.
11	81418152	A	C	intergenic	*LOC101928944,MIR4300HG*	.	.	.
11	80547878	G	T	intergenic	*LOC101928944,MIR4300HG*	.	.	.
11	80847127	T	A	intergenic	*LOC101928944,MIR4300HG*	.	.	.
11	80847128	T	A	intergenic	*LOC101928944,MIR4300HG*	.	.	.
11	81047286	G	A	intergenic	*LOC101928944,MIR4300HG*	.	.	.
11	81113378	T	A	intergenic	*LOC101928944,MIR4300HG*	.	.	.
11	81207320	T	A	intergenic	*LOC101928944,MIR4300HG*	.	.	.
11	81548114	G	T	intergenic	*LOC101928944,MIR4300HG*	.	.	.
11	80478780	C	A	intergenic	*LOC101928944,MIR4300HG*	.	.	.
11	80502724	G	A	intergenic	*LOC101928944,MIR4300HG*	.	.	.
11	80503766	G	A	intergenic	*LOC101928944,MIR4300HG*	.	.	.
11	80504199	T	G	intergenic	*LOC101928944,MIR4300HG*	.	.	.
11	80569255	AT	A	intergenic	*LOC101928944,MIR4300HG*	.	.	.
11	80805417	C	T	intergenic	*LOC101928944,MIR4300HG*	.	.	.
11	80805772	C	G	intergenic	*LOC101928944,MIR4300HG*	.	.	.
11	81119519	T	C	intergenic	*LOC101928944,MIR4300HG*	.	.	.
11	81119520	G	A	intergenic	*LOC101928944,MIR4300HG*	.	.	.
11	81119637	C	T	intergenic	*LOC101928944,MIR4300HG*	.	.	.

11	81165266	C	T	intergenic	*LOC101928944,MIR4300HG*	.	.	.
11	81212637	A	G	intergenic	*LOC101928944,MIR4300HG*	.	.	.
11	81311610	G	A	intergenic	*LOC101928944,MIR4300HG*	.	.	.
1	181167164	G	T	intergenic	*LOC101928973,GM140*	.	.	.
1	181169304	A	G	intergenic	*LOC101928973,GM140*	.	.	.
1	116509865	G	T	ncRNA_intronic	*LOC101928977*	.	.	.
4	85148294	TTCTCTC	T	ncRNA_intronic	*LOC101928978*	.	.	.
4	85063046	C	T	ncRNA_intronic	*LOC101928978*	.	.	.
4	84924889	G	A	ncRNA_intronic	*LOC101928978*	.	.	.
4	85037669	C	T	ncRNA_intronic	*LOC101928978*	.	.	.
4	85048747	C	T	ncRNA_intronic	*LOC101928978*	.	.	.
4	85051043	T	TCGGA	ncRNA_intronic	*LOC101928978*	.	.	.
4	85125671	T	G	ncRNA_intronic	*LOC101928978*	.	.	.
4	85212273	T	C	ncRNA_intronic	*LOC101928978*	.	.	.
4	85363657	G	A	intergenic	*LOC101928978,NKX6-1*	.	.	.
4	85245501	CT	C	intergenic	*LOC101928978,NKX6-1*	.	.	.
4	85245503	CT	C	intergenic	*LOC101928978,NKX6-1*	.	.	.
4	85235848	C	T	intergenic	*LOC101928978,NKX6-1*	.	.	.
4	85236042	G	A	intergenic	*LOC101928978,NKX6-1*	.	.	.
4	12534431	A	T	intergenic	*LOC101929019,RAB28*	.	.	.
4	12927167	AT	A	intergenic	*LOC101929019,RAB28*	.	.	.
4	12927169	AGAC	A	intergenic	*LOC101929019,RAB28*	.	.	.
4	12756084	G	T	intergenic	*LOC101929019,RAB28*	.	.	.

4	12772228	C	A	intergenic	*LOC101929019,RAB28*	.	.	.
4	12787224	C	T	intergenic	*LOC101929019,RAB28*	.	.	.
4	13019212	A	C	intergenic	*LOC101929019,RAB28*	.	.	.
4	13230346	A	G	intergenic	*LOC101929019,RAB28*	.	.	.
4	13291576	A	G	intergenic	*LOC101929019,RAB28*	.	.	.
4	12518566	G	T	intergenic	*LOC101929019,RAB28*	.	.	.
4	12947544	A	AAGGGAGA(	intergenic	*LOC101929019,RAB28*	.	.	.
4	12341248	T	A	intergenic	*LOC101929019,RAB28*	.	.	.
4	12344805	A	G	intergenic	*LOC101929019,RAB28*	.	.	.
4	12347621	C	T	intergenic	*LOC101929019,RAB28*	.	.	.
4	12600855	C	T	intergenic	*LOC101929019,RAB28*	.	.	.
4	12717485	C	A	intergenic	*LOC101929019,RAB28*	.	.	.
4	12717487	C	T	intergenic	*LOC101929019,RAB28*	.	.	.
4	12740872	G	A	intergenic	*LOC101929019,RAB28*	.	.	.
4	12887298	A	C	intergenic	*LOC101929019,RAB28*	.	.	.
4	13218420	G	A	intergenic	*LOC101929019,RAB28*	.	.	.
4	13267100	G	A	intergenic	*LOC101929019,RAB28*	.	.	.
4	13273175	A	C	intergenic	*LOC101929019,RAB28*	.	.	.
4	13336786	A	C	intergenic	*LOC101929019,RAB28*	.	.	.

4	13364567	T	TAGA	intergenic	*LOC101929019,RAB28*	.	.	.
4	13364568	T	TC	intergenic	*LOC101929019,RAB28*	.	.	.
12	103680809	G	A	intergenic	*LOC101929058,C12orf42*	.	.	.
12	103690805	A	G	intergenic	*LOC101929058,C12orf42*	.	.	.
4	87094808	G	C	ncRNA_intronic	*LOC101929064*	.	.	.
4	15071599	G	A	ncRNA_intronic	*LOC101929095*	.	.	.
4	15158502	T	C	ncRNA_intronic	*LOC101929095*	.	.	.
4	15115497	A	G	ncRNA_intronic	*LOC101929095*	.	.	.
4	15116052	A	G	ncRNA_intronic	*LOC101929095*	.	.	.
4	15264598	T	C	ncRNA_intronic	*LOC101929095*	.	.	.
4	15358254	C	G	ncRNA_intronic	*LOC101929095*	.	.	.
4	15358255	A	G	ncRNA_intronic	*LOC101929095*	.	.	.
4	15358757	A	G	ncRNA_intronic	*LOC101929095*	.	.	.
9	129081126	G	A	intergenic	*LOC101929116,MVB12B*	.	.	.
4	17179846	A	T	ncRNA_intronic	*LOC101929123*	.	.	.
4	17247944	A	C	intergenic	*LOC101929123,QDPR*	.	.	.
4	17228469	A	G	intergenic	*LOC101929123,QDPR*	.	.	.
4	17384808	C	G	intergenic	*LOC101929123,QDPR*	.	.	.
4	17392609	T	C	intergenic	*LOC101929123,QDPR*	.	.	.
19	23241693	G	C	ncRNA_intronic	*LOC101929144*	.	.	.
19	23276349	G	T	intergenic	*LOC101929144,ZNF730*	.	.	.
19	23292853	G	C	intergenic	*LOC101929144,ZNF730*	.	.	.
1	119698696	G	T	ncRNA_intronic	*LOC101929147*	.	.	.
1	119697783	G	T	ncRNA_intronic	*LOC101929147*	.	.	.
1	119754384	A	AG	ncRNA_intronic	*LOC101929147*	.	.	.
1	119890217	C	A	intergenic	*LOC101929147,HAO2*	.	.	.

5	4895174	GTAGGTAG ATAGA	G	intergenic	*LOC101929153,LINC01020*	.	.	.
5	4843589	G	C	intergenic	*LOC101929153,LINC01020*	.	.	.
5	4903903	T	C	intergenic	*LOC101929153,LINC01020*	.	.	.
5	4812227	G	C	intergenic	*LOC101929153,LINC01020*	.	.	.
5	4812579	G	GA	intergenic	*LOC101929153,LINC01020*	.	.	.
5	4888971	T	G	intergenic	*LOC101929153,LINC01020*	.	.	.
5	4889410	G	T	intergenic	*LOC101929153,LINC01020*	.	.	.
5	4979946	T	G	intergenic	*LOC101929153,LINC01020*	.	.	.
5	4979948	A	G	intergenic	*LOC101929153,LINC01020*	.	.	.
5	5029863	A	C	intergenic	*LOC101929153,LINC01020*	.	.	.
5	5030354	G	C	intergenic	*LOC101929153,LINC01020*	.	.	.
5	77213760	T	C	ncRNA_intronic	*LOC101929154*	.	.	.
5	77228690	G	A	ncRNA_intronic	*LOC101929154*	.	.	.
8	21293180	G	A	intergenic	*LOC101929172,GFRA2*	.	.	.
8	21294094	C	T	intergenic	*LOC101929172,GFRA2*	.	.	.
4	27242206	A	C	ncRNA_intronic	*LOC101929199*	.	.	.
4	28023488	T	C	intergenic	*LOC101929199,MIR4275*	.	.	.
4	28090293	TAAC	T	intergenic	*LOC101929199,MIR4275*	.	.	.
4	28106043	G	T	intergenic	*LOC101929199,MIR4275*	.	.	.
4	28144700	T	A	intergenic	*LOC101929199,MIR4275*	.	.	.

4	28215635	C	CAAAAAAAA	intergenic	*LOC101929199,MIR4275*	.	.	.
4	28286997	C	A	intergenic	*LOC101929199,MIR4275*	.	.	.
4	28304647	G	A	intergenic	*LOC101929199,MIR4275*	.	.	.
4	28505962	G	T	intergenic	*LOC101929199,MIR4275*	.	.	.
4	28159545	G	T	intergenic	*LOC101929199,MIR4275*	.	.	.
4	28699032	C	T	intergenic	*LOC101929199,MIR4275*	.	.	.
4	27475120	G	A	intergenic	*LOC101929199,MIR4275*	.	.	.
4	27782779	A	G	intergenic	*LOC101929199,MIR4275*	.	.	.
4	28650787	T	TC	intergenic	*LOC101929199,MIR4275*	.	.	.
4	28771322	C	G	intergenic	*LOC101929199,MIR4275*	.	.	.
4	27729315	G	A	intergenic	*LOC101929199,MIR4275*	.	.	.
4	27583675	T	C	intergenic	*LOC101929199,MIR4275*	.	.	.
4	27583676	T	C	intergenic	*LOC101929199,MIR4275*	.	.	.
4	27583888	G	T	intergenic	*LOC101929199,MIR4275*	.	.	.
4	28069654	T	C	intergenic	*LOC101929199,MIR4275*	.	.	.
4	28083266	A	C	intergenic	*LOC101929199,MIR4275*	.	.	.
4	28276059	C	G	intergenic	*LOC101929199,MIR4275*	.	.	.
4	28276707	A	G	intergenic	*LOC101929199,MIR4275*	.	.	.

4	28314274	CGTGTGTGTGTGTGCACCTGCACATGTGTGTGTGTGCTCGTGTGTGTGTGCTCGTGTGTGTGTGTGCACCTGCACATGTGTGTGTGTGCTCGT	C	intergenic	*LOC101929199,MIR4275*	.	.	.
4	28354579	G	C	intergenic	*LOC101929199,MIR4275*	.	.	.
4	28359079	A	G	intergenic	*LOC101929199,MIR4275*	.	.	.
4	28413341	A	G	intergenic	*LOC101929199,MIR4275*	.	.	.
4	28494284	T	C	intergenic	*LOC101929199,MIR4275*	.	.	.
4	28496434	T	C	intergenic	*LOC101929199,MIR4275*	.	.	.
4	28685685	T	C	intergenic	*LOC101929199,MIR4275*	.	.	.
4	28685686	G	A	intergenic	*LOC101929199,MIR4275*	.	.	.
4	28778957	G	A	intergenic	*LOC101929199,MIR4275*	.	.	.
11	120760520	T	C	ncRNA_intronic	*LOC101929227*	.	.	.
2	150492973	A	G	ncRNA_intronic	*LOC101929231*	.	.	.
2	150494842	A	G	ncRNA_intronic	*LOC101929231*	.	.	.
2	150570879	T	C	ncRNA_intronic	*LOC101929231*	.	.	.
2	150570881	T	C	ncRNA_intronic	*LOC101929231*	.	.	.
2	150610663	A	C	ncRNA_intronic	*LOC101929231*	.	.	.
2	150648840	A	G	ncRNA_intronic	*LOC101929231*	.	.	.
2	150658991	G	A	ncRNA_intronic	*LOC101929231*	.	.	.
2	150677469	C	T	ncRNA_intronic	*LOC101929231*	.	.	.

2	151125865	T	C	intergenic	*LOC101929231,RND3*	.	.	.
2	150916399	TAAATAAAC	T	intergenic	*LOC101929231,RND3*	.	.	.
2	151320045	ATGTG	A	intergenic	*LOC101929231,RND3*	.	.	.
2	150729916	A	G	intergenic	*LOC101929231,RND3*	.	.	.
2	150933458	GATAT	G	intergenic	*LOC101929231,RND3*	.	.	.
2	151175337	A	G	intergenic	*LOC101929231,RND3*	.	.	.
2	150735825	A	G	intergenic	*LOC101929231,RND3*	.	.	.
2	150769238	A	C	intergenic	*LOC101929231,RND3*	.	.	.
2	150816081	C	T	intergenic	*LOC101929231,RND3*	.	.	.
2	150825027	G	A	intergenic	*LOC101929231,RND3*	.	.	.
2	150825647	G	A	intergenic	*LOC101929231,RND3*	.	.	.
2	150826948	G	A	intergenic	*LOC101929231,RND3*	.	.	.
2	150836847	C	A	intergenic	*LOC101929231,RND3*	.	.	.
2	150836849	T	C	intergenic	*LOC101929231,RND3*	.	.	.
2	150866414	T	C	intergenic	*LOC101929231,RND3*	.	.	.
2	151051304	A	G	intergenic	*LOC101929231,RND3*	.	.	.
2	151054139	A	G	intergenic	*LOC101929231,RND3*	.	.	.
2	151096951	G	A	intergenic	*LOC101929231,RND3*	.	.	.
2	151172694	A	C	intergenic	*LOC101929231,RND3*	.	.	.

2	151278257	C	T	intergenic	*LOC101929231,RND3*	.	.	.
14	98042628	C	A	ncRNA_intronic	*LOC101929241*	.	.	.
14	98075095	TTGTG	T	intergenic	*LOC101929241,LOC100129345*	.	.	.
14	98051342	G	T	intergenic	*LOC101929241,LOC100129345*	.	.	.
14	98060440	G	A	intergenic	*LOC101929241,LOC100129345*	.	.	.
10	30535599	A	G	intergenic	*LOC101929279,MTPAP*	.	.	.
10	30565808	G	A	intergenic	*LOC101929279,MTPAP*	.	.	.
2	151494182	C	T	intergenic	*LOC101929282,RBM43*	.	.	.
2	151532320	A	T	intergenic	*LOC101929282,RBM43*	.	.	.
2	151651722	A	G	intergenic	*LOC101929282,RBM43*	.	.	.
2	151652402	T	A	intergenic	*LOC101929282,RBM43*	.	.	.
2	152089555	A	T	intergenic	*LOC101929282,RBM43*	.	.	.
5	8990328	C	T	intergenic	*LOC101929284,SEMA5A*	.	.	.
5	8935414	T	C	intergenic	*LOC101929284,SEMA5A*	.	.	.
5	8930692	A	G	intergenic	*LOC101929284,SEMA5A*	.	.	.
5	9004702	A	G	intergenic	*LOC101929284,SEMA5A*	.	.	.
5	9004703	C	T	intergenic	*LOC101929284,SEMA5A*	.	.	.
8	24189059	C	CT	ncRNA_intronic	*LOC101929294*	.	.	.
8	24184750	G	T	ncRNA_intronic	*LOC101929294*	.	.	.
8	24229930	C	A	ncRNA_intronic	*LOC101929294*	.	.	.
8	24258521	A	G	ncRNA_intronic	*LOC101929294*	.	.	.
8	24307190	G	T	ncRNA_intronic	*LOC101929294*	.	.	.

8	24413854	A	G	intergenic	*LOC101929294,NEFM*	.	.	.
8	24440683	A	AC	intergenic	*LOC101929294,NEFM*	.	.	.
8	24494550	T	C	intergenic	*LOC101929294,NEFM*	.	.	.
8	24494551	G	T	intergenic	*LOC101929294,NEFM*	.	.	.
8	24504456	T	A	intergenic	*LOC101929294,NEFM*	.	.	.
8	24698619	C	G	intergenic	*LOC101929294,NEFM*	.	.	.
2	152235024	C	T	ncRNA_intronic	*LOC101929319*	.	.	.
9	132059061	G	A	intergenic	*LOC101929331,C9orf106*	.	.	.
9	132060458	G	A	intergenic	*LOC101929331,C9orf106*	.	.	.
4	101225421	T	A	intergenic	*LOC101929353,EMCN*	.	.	.
4	101184046	A	G	intergenic	*LOC101929353,EMCN*	.	.	.
4	101302488	G	A	intergenic	*LOC101929353,EMCN*	.	.	.
4	101302490	C	T	intergenic	*LOC101929353,EMCN*	.	.	.
20	9990017	C	A	intergenic	*LOC101929371,SNAP25-AS1*	.	.	.
20	9998324	T	C	intergenic	*LOC101929371,SNAP25-AS1*	.	.	.
2	157023759	A	T	ncRNA_intronic	*LOC101929378*	.	.	.
2	157048844	G	A	ncRNA_intronic	*LOC101929378*	.	.	.
2	156935986	A	G	ncRNA_intronic	*LOC101929378*	.	.	.
2	156997769	C	G	ncRNA_intronic	*LOC101929378*	.	.	.
2	157028267	C	A	ncRNA_intronic	*LOC101929378*	.	.	.
2	157028268	A	G	ncRNA_intronic	*LOC101929378*	.	.	.
2	157080631	A	T	ncRNA_intronic	*LOC101929378*	.	.	.
5	86496036	T	G	ncRNA_intronic	*LOC101929380*	.	.	.
5	86509495	T	C	ncRNA_intronic	*LOC101929380*	.	.	.

20	10800109	G	C	intergenic	*LOC101929395,LOC101929413*	.	.	.
1	30629113	T	G	intergenic	*LOC101929406,MATN1*	.	.	.
1	30821100	C	T	intergenic	*LOC101929406,MATN1*	.	.	.
1	30969233	G	A	intergenic	*LOC101929406,MATN1*	.	.	.
1	31040687	C	T	intergenic	*LOC101929406,MATN1*	.	.	.
5	10523555	C	T	intergenic	*LOC101929412,ANKRD33B*	.	.	.
20	11121454	G	T	intergenic	*LOC101929413,LOC339593*	.	.	.
20	11003047	C	A	intergenic	*LOC101929413,LOC339593*	.	.	.
20	11094052	T	A	intergenic	*LOC101929413,LOC339593*	.	.	.
20	10954070	G	A	intergenic	*LOC101929413,LOC339593*	.	.	.
20	10990531	G	A	intergenic	*LOC101929413,LOC339593*	.	.	.
20	11009254	C	T	intergenic	*LOC101929413,LOC339593*	.	.	.
20	11131362	A	G	intergenic	*LOC101929413,LOC339593*	.	.	.
4	104379857	C	T	intergenic	*LOC101929448,TACR3*	.	.	.
4	104409321	A	G	intergenic	*LOC101929448,TACR3*	.	.	.
8	29620875	C	T	ncRNA_intronic	*LOC101929450*	.	.	.
2	7269103	A	G	intergenic	*LOC101929452,LOC100506274*	.	.	.
2	7396234	A	ATT	intergenic	*LOC101929452,LOC100506274*	.	.	.
2	7460017	T	C	intergenic	*LOC101929452,LOC100506274*	.	.	.

5	15355572	T	C	intergenic	*LOC101929454,FBXL7*	.	.	.
5	15275973	C	A	intergenic	*LOC101929454,FBXL7*	.	.	.
5	15273975	C	T	intergenic	*LOC101929454,FBXL7*	.	.	.
5	15359328	C	CA	intergenic	*LOC101929454,FBXL7*	.	.	.
5	15360041	C	G	intergenic	*LOC101929454,FBXL7*	.	.	.
5	15360042	A	G	intergenic	*LOC101929454,FBXL7*	.	.	.
5	15399009	C	T	intergenic	*LOC101929454,FBXL7*	.	.	.
4	105520400	T	C	ncRNA_intronic	*LOC101929468*	.	.	.
4	105416093	CCTCT	C	ncRNA_intronic	*LOC101929468*	.	.	.
4	105462717	C	T	ncRNA_intronic	*LOC101929468*	.	.	.
4	105462718	A	G	ncRNA_intronic	*LOC101929468*	.	.	.
4	105640964	C	T	intergenic	*LOC101929468,TET2*	.	.	.
4	105633034	C	G	intergenic	*LOC101929468,TET2*	.	.	.
4	105775054	C	T	intergenic	*LOC101929468,TET2*	.	.	.
4	105775056	G	C	intergenic	*LOC101929468,TET2*	.	.	.
4	105854947	A	G	intergenic	*LOC101929468,TET2*	.	.	.
4	105951517	C	A	intergenic	*LOC101929468,TET2*	.	.	.
4	105951518	T	A	intergenic	*LOC101929468,TET2*	.	.	.
4	105975345	T	C	intergenic	*LOC101929468,TET2*	.	.	.
8	29771216	T	C	intergenic	*LOC101929470,FAM183CP*	.	.	.
8	29771218	C	T	intergenic	*LOC101929470,FAM183CP*	.	.	.

11	127018705	GGAGA	G	intergenic	*LOC101929473,LOC101929497*	.	.	.
11	127046670	T	C	intergenic	*LOC101929473,LOC101929497*	.	.	.
20	12924050	C	T	ncRNA_intronic	*LOC101929486*	.	.	.
20	12978314	C	A	intergenic	*LOC101929486,SPTLC3*	.	.	.
8	58391681	A	G	intergenic	*LOC101929488,LINC01602*	.	.	.
8	58415495	A	C	intergenic	*LOC101929488,LINC01602*	.	.	.
8	58722752	T	C	intergenic	*LOC101929488,LINC01602*	.	.	.
8	58785495	A	G	intergenic	*LOC101929488,LINC01602*	.	.	.
8	58399140	T	A	intergenic	*LOC101929488,LINC01602*	.	.	.
8	58466101	A	T	intergenic	*LOC101929488,LINC01602*	.	.	.
8	58578161	G	A	intergenic	*LOC101929488,LINC01602*	.	.	.
8	58610235	A	G	intergenic	*LOC101929488,LINC01602*	.	.	.
8	58611435	A	C	intergenic	*LOC101929488,LINC01602*	.	.	.
8	58782671	A	G	intergenic	*LOC101929488,LINC01602*	.	.	.
8	58873815	A	T	intergenic	*LOC101929488,LINC01602*	.	.	.
11	127185047	C	A	ncRNA_intronic	*LOC101929497*	.	.	.
11	127185049	A	G	ncRNA_intronic	*LOC101929497*	.	.	.
11	127520514	GTTATTA	G	intergenic	*LOC101929497,ETS1*	.	.	.
11	127531396	G	A	intergenic	*LOC101929497,ETS1*	.	.	.
11	127640993	G	A	intergenic	*LOC101929497,ETS1*	.	.	.

11	127621238	C	T	intergenic	*LOC101929497,ETS1*	.	.	.
11	127846909	T	G	intergenic	*LOC101929497,ETS1*	.	.	.
11	127946814	A	G	intergenic	*LOC101929497,ETS1*	.	.	.
11	127979502	T	C	intergenic	*LOC101929497,ETS1*	.	.	.
11	128006473	C	T	intergenic	*LOC101929497,ETS1*	.	.	.
11	128014987	T	A	intergenic	*LOC101929497,ETS1*	.	.	.
11	128015246	T	C	intergenic	*LOC101929497,ETS1*	.	.	.
11	128078077	TACAC	T	intergenic	*LOC101929497,ETS1*	.	.	.
6	169581631	T	C	ncRNA_intronic	*LOC101929504*	.	.	.
5	16396097	G	A	ncRNA_intronic	*LOC101929505*	.	.	.
5	16396846	T	C	ncRNA_intronic	*LOC101929505*	.	.	.
5	16397408	A	G	ncRNA_intronic	*LOC101929505*	.	.	.
5	16398298	G	T	ncRNA_intronic	*LOC101929505*	.	.	.
5	16398813	G	A	ncRNA_intronic	*LOC101929505*	.	.	.
2	162136450	A	G	intergenic	*LOC101929512,PSMD14*	.	.	.
5	16636225	C	T	intergenic	*LOC101929524,MYO10*	.	.	.
5	16653223	G	A	intergenic	*LOC101929524,MYO10*	.	.	.
8	59290969	T	A	intergenic	*LOC101929528,UBXN2B*	.	.	.
8	59201656	A	G	intergenic	*LOC101929528,UBXN2B*	.	.	.
8	59202262	G	C	intergenic	*LOC101929528,UBXN2B*	.	.	.
8	59276647	A	G	intergenic	*LOC101929528,UBXN2B*	.	.	.
8	59276648	A	G	intergenic	*LOC101929528,UBXN2B*	.	.	.

2	162988676	T	C	ncRNA_intronic	*LOC101929532*	.	.	.
6	40865215	G	A	ncRNA_intronic	*LOC101929555*	.	.	.
6	40944212	C	CA	ncRNA_intronic	*LOC101929555*	.	.	.
6	40944214	T	C	ncRNA_intronic	*LOC101929555*	.	.	.
9	23627134	C	A	ncRNA_intronic	*LOC101929563*	.	.	.
9	23627136	A	G	ncRNA_intronic	*LOC101929563*	.	.	.
4	106927117	T	C	ncRNA_intronic	*LOC101929577*	.	.	.
12	5343599	A	G	ncRNA_intronic	*LOC101929584*	.	.	.
12	5520459	C	T	intergenic	*LOC101929584,NTF3*	.	.	.
2	38771548	TTCTC	T	intergenic	*LOC101929596,HNRNPLL*	.	.	.
2	38760711	T	C	intergenic	*LOC101929596,HNRNPLL*	.	.	.
3	107168524	A	C	ncRNA_intronic	*LOC101929607*	.	.	.
3	107173459	CTATA	C	ncRNA_intronic	*LOC101929607*	.	.	.
3	107194239	C	A	intergenic	*LOC101929607,BBX*	.	.	.
20	21616699	A	G	ncRNA_intronic	*LOC101929608*	.	.	.
1	218309131	T	C	intergenic	*LOC101929631,RRP15*	.	.	.
1	218408788	C	G	intergenic	*LOC101929631,RRP15*	.	.	.
1	218407430	C	T	intergenic	*LOC101929631,RRP15*	.	.	.
1	218407431	T	G	intergenic	*LOC101929631,RRP15*	.	.	.
5	29065705	TG	T	ncRNA_exonic	*LOC101929645*	.	.	.
11	131410144	C	T	ncRNA_intronic	*LOC101929653*	.	.	.
5	29369513	G	A	intergenic	*LOC101929660,LOC101929681*	.	.	.
5	29188612	T	TTTTCTA	intergenic	*LOC101929660,LOC101929681*	.	.	.
5	29295947	A	G	intergenic	*LOC101929660,LOC101929681*	.	.	.
5	29188616	T	TCGGAAG	intergenic	*LOC101929660,LOC101929681*	.	.	.

5	29237446	C	T	intergenic	*LOC101929660,LOC101929681*	.	.	.
5	29316345	G	C	intergenic	*LOC101929660,LOC101929681*	.	.	.
5	29317581	A	G	intergenic	*LOC101929660,LOC101929681*	.	.	.
2	166954123	G	T	ncRNA_intronic	*LOC101929680*	.	.	.
2	167061654	A	G	ncRNA_intronic	*LOC101929680*	.	.	.
5	29954941	T	TC	intergenic	*LOC101929681,CDH6*	.	.	.
5	30243695	C	CA	intergenic	*LOC101929681,CDH6*	.	.	.
5	30275594	C	A	intergenic	*LOC101929681,CDH6*	.	.	.
5	30544526	A	C	intergenic	*LOC101929681,CDH6*	.	.	.
5	30560237	A	T	intergenic	*LOC101929681,CDH6*	.	.	.
5	30912207	A	T	intergenic	*LOC101929681,CDH6*	.	.	.
5	31051294	G	A	intergenic	*LOC101929681,CDH6*	.	.	.
5	29900831	C	G	intergenic	*LOC101929681,CDH6*	.	.	.
5	30378055	G	A	intergenic	*LOC101929681,CDH6*	.	.	.
5	30211287	G	A	intergenic	*LOC101929681,CDH6*	.	.	.
5	29555642	C	T	intergenic	*LOC101929681,CDH6*	.	.	.
5	30838646	G	A	intergenic	*LOC101929681,CDH6*	.	.	.
5	31080077	T	C	intergenic	*LOC101929681,CDH6*	.	.	.
5	29411857	G	T	intergenic	*LOC101929681,CDH6*	.	.	.
5	29474234	G	GATATATAT	intergenic	*LOC101929681,CDH6*	.	.	.

5	29927380	C	T	intergenic	*LOC101929681,CDH6*	.	.	.
5	30308664	G	C	intergenic	*LOC101929681,CDH6*	.	.	.
5	30929475	C	CAAGAGAGA	intergenic	*LOC101929681,CDH6*	.	.	.
5	29949995	G	T	intergenic	*LOC101929681,CDH6*	.	.	.
5	29527617	A	G	intergenic	*LOC101929681,CDH6*	.	.	.
5	29537637	C	T	intergenic	*LOC101929681,CDH6*	.	.	.
5	29538769	T	G	intergenic	*LOC101929681,CDH6*	.	.	.
5	29650146	T	A	intergenic	*LOC101929681,CDH6*	.	.	.
5	30167342	G	T	intergenic	*LOC101929681,CDH6*	.	.	.
5	30361038	A	G	intergenic	*LOC101929681,CDH6*	.	.	.
5	30361039	C	T	intergenic	*LOC101929681,CDH6*	.	.	.
5	30456817	C	T	intergenic	*LOC101929681,CDH6*	.	.	.
5	30485900	A	G	intergenic	*LOC101929681,CDH6*	.	.	.
5	30780661	T	C	intergenic	*LOC101929681,CDH6*	.	.	.
5	30834033	G	A	intergenic	*LOC101929681,CDH6*	.	.	.
5	30952403	G	A	intergenic	*LOC101929681,CDH6*	.	.	.
5	31110141	G	A	intergenic	*LOC101929681,CDH6*	.	.	.
5	31111098	G	A	intergenic	*LOC101929681,CDH6*	.	.	.
3	112457972	TATAC	T	ncRNA_intronic	*LOC101929694*	.	.	.

5	139153557	TAATGTCCCAATGACATTGAGGGACATTGTCCCTC	T	intergenic	*LOC101929696,PSD2*	.	.	.
6	44012557	ACATTCATATACATC	A	ncRNA_intronic	*LOC101929705*	.	.	.
8	90728808	GTCTC	G	downstream	*LOC101929709*	.	.	.
5	95516671	T	A	ncRNA_intronic	*LOC101929710*	.	.	.
5	95517506	T	C	ncRNA_intronic	*LOC101929710*	.	.	.
5	95950462	A	AAT	ncRNA_intronic	*LOC101929710*	.	.	.
5	95415899	C	T	ncRNA_intronic	*LOC101929710*	.	.	.
5	95643712	A	G	ncRNA_intronic	*LOC101929710*	.	.	.
5	95692324	G	T	ncRNA_intronic	*LOC101929710*	.	.	.
5	95692767	C	G	ncRNA_intronic	*LOC101929710*	.	.	.
5	95699310	A	G	ncRNA_intronic	*LOC101929710*	.	.	.
5	95787358	C	A	ncRNA_intronic	*LOC101929710*	.	.	.
5	95832912	A	G	ncRNA_intronic	*LOC101929710*	.	.	.
5	95887646	A	G	ncRNA_intronic	*LOC101929710*	.	.	.
5	95888216	A	G	ncRNA_intronic	*LOC101929710*	.	.	.
5	95889627	A	G	ncRNA_intronic	*LOC101929710*	.	.	.
5	95893474	A	C	ncRNA_intronic	*LOC101929710*	.	.	.
5	95894343	C	T	ncRNA_intronic	*LOC101929710*	.	.	.
5	95895527	G	A	ncRNA_intronic	*LOC101929710*	.	.	.
3	114223134	A	G	ncRNA_intronic	*LOC101929754*	.	.	.
1	222054732	C	CA	intergenic	*LOC101929771,HHIPL2*	.	.	.
1	222464064	C	T	intergenic	*LOC101929771,HHIPL2*	.	.	.
1	222652980	A	T	intergenic	*LOC101929771,HHIPL2*	.	.	.
1	222081517	TA	T	intergenic	*LOC101929771,HHIPL2*	.	.	.
1	222569636	G	T	intergenic	*LOC101929771,HHIPL2*	.	.	.
1	222569637	G	T	intergenic	*LOC101929771,HHIPL2*	.	.	.

1	222323451	A	C	intergenic	*LOC101929771,HHIPL2*	.	.	.
1	222323722	A	C	intergenic	*LOC101929771,HHIPL2*	.	.	.
1	222350229	T	G	intergenic	*LOC101929771,HHIPL2*	.	.	.
1	222394228	G	A	intergenic	*LOC101929771,HHIPL2*	.	.	.
1	222676252	G	A	intergenic	*LOC101929771,HHIPL2*	.	.	.
8	42125988	T	C	ncRNA_intronic	*LOC101929897*	.	.	.
12	9549066	A	T	ncRNA_exonic	*LOC101930452*	.	.	.
12	9568784	A	AGGGGCT	intergenic	*LOC101930452,DDX12P*	.	.	.
16	52234588	G	T	intergenic	*LOC102467079,TOX3*	.	.	.
16	52461817	A	ACAAG	intergenic	*LOC102467079,TOX3*	.	.	.
16	52135883	G	A	intergenic	*LOC102467079,TOX3*	.	.	.
16	52230144	C	T	intergenic	*LOC102467079,TOX3*	.	.	.
16	52416824	GCACA	G	intergenic	*LOC102467079,TOX3*	.	.	.
5	54027138	T	C	ncRNA_intronic	*LOC102467080*	.	.	.
5	53981447	G	A	ncRNA_intronic	*LOC102467080*	.	.	.
5	54193025	C	T	intergenic	*LOC102467080,ESM1*	.	.	.
5	54086222	G	A	intergenic	*LOC102467080,ESM1*	.	.	.
5	54172123	T	C	intergenic	*LOC102467080,ESM1*	.	.	.
5	54215370	T	A	intergenic	*LOC102467080,ESM1*	.	.	.
5	54216568	C	T	intergenic	*LOC102467080,ESM1*	.	.	.
5	54216684	C	G	intergenic	*LOC102467080,ESM1*	.	.	.

5	54217182	C	T	intergenic	*LOC102467080,ESM1*	.	.	.
5	102868448	T	G	ncRNA_intronic	*LOC102467212*	.	.	.
5	106165120	G	GTA	ncRNA_intronic	*LOC102467213*	.	.	.
5	106157480	A	G	ncRNA_intronic	*LOC102467213*	.	.	.
5	106332839	C	CATTTTTTAT	ncRNA_intronic	*LOC102467213*	.	.	.
5	106177618	T	C	ncRNA_intronic	*LOC102467213*	.	.	.
5	106331026	T	A	ncRNA_intronic	*LOC102467213*	.	.	.
5	106333467	G	C	ncRNA_intronic	*LOC102467213*	.	.	.
5	106496809	C	T	intergenic	*LOC102467213,EFNA5*	.	.	.
5	115012532	G	A	intergenic	*LOC102467217,CDO1*	.	.	.
5	115088747	A	AGC	intergenic	*LOC102467217,CDO1*	.	.	.
8	20816381	G	C	ncRNA_intronic	*LOC102467222*	.	.	.
8	20816612	G	A	ncRNA_intronic	*LOC102467222*	.	.	.
5	116256165	T	A	intergenic	*LOC102467223,LINC00992*	.	.	.
5	116108213	A	G	intergenic	*LOC102467223,LINC00992*	.	.	.
5	116374344	T	G	intergenic	*LOC102467223,LINC00992*	.	.	.
5	116374346	C	A	intergenic	*LOC102467223,LINC00992*	.	.	.
5	116384929	G	C	intergenic	*LOC102467223,LINC00992*	.	.	.
5	116458634	G	A	intergenic	*LOC102467223,LINC00992*	.	.	.
5	116217847	GGAGA	G	intergenic	*LOC102467223,LINC00992*	.	.	.
5	116448363	G	A	intergenic	*LOC102467223,LINC00992*	.	.	.
5	116533976	G	C	intergenic	*LOC102467223,LINC00992*	.	.	.
5	116676256	C	T	intergenic	*LOC102467223,LINC00992*	.	.	.

5	116676695	C	G	intergenic	*LOC102467223,LINC00992*	.	.	.
5	117602187	C	A	downstream	*LOC102467224*	.	.	.
5	117082795	G	A	ncRNA_intronic	*LOC102467224*	.	.	.
5	117546847	T	C	ncRNA_intronic	*LOC102467224*	.	.	.
5	117341925	T	C	ncRNA_intronic	*LOC102467224*	.	.	.
5	117243386	G	A	ncRNA_intronic	*LOC102467224*	.	.	.
5	117266776	C	A	ncRNA_intronic	*LOC102467224*	.	.	.
5	117582247	C	T	ncRNA_intronic	*LOC102467224*	.	.	.
5	117602864	A	T	intergenic	*LOC102467224,HNCAT21*	.	.	.
5	118058002	G	T	intergenic	*LOC102467225,DTWD2*	.	.	.
5	120911356	C	A	intergenic	*LOC102467226,FTMT*	.	.	.
5	120693755	C	G	intergenic	*LOC102467226,FTMT*	.	.	.
5	120909399	T	A	intergenic	*LOC102467226,FTMT*	.	.	.
5	121150489	T	C	intergenic	*LOC102467226,FTMT*	.	.	.
5	120675612	T	C	intergenic	*LOC102467226,FTMT*	.	.	.
5	120803448	C	T	intergenic	*LOC102467226,FTMT*	.	.	.
5	120892579	A	T	intergenic	*LOC102467226,FTMT*	.	.	.
5	120984055	C	T	intergenic	*LOC102467226,FTMT*	.	.	.
5	120984056	A	G	intergenic	*LOC102467226,FTMT*	.	.	.
5	120986936	A	C	intergenic	*LOC102467226,FTMT*	.	.	.
5	120997552	G	A	intergenic	*LOC102467226,FTMT*	.	.	.
5	121000015	G	A	intergenic	*LOC102467226,FTMT*	.	.	.

5	121010270	C	T	intergenic	*LOC102467226,FTMT*	.	.	.
5	121015588	C	T	intergenic	*LOC102467226,FTMT*	.	.	.
5	121016660	T	A	intergenic	*LOC102467226,FTMT*	.	.	.
5	121016662	T	C	intergenic	*LOC102467226,FTMT*	.	.	.
5	121058867	A	G	intergenic	*LOC102467226,FTMT*	.	.	.
5	121140651	A	G	intergenic	*LOC102467226,FTMT*	.	.	.
5	71934468	T	C	ncRNA_intronic	*LOC102477328*	.	.	.
5	72086113	ACTCT	A	intergenic	*LOC102477328,TNPO1*	.	.	.
5	72026781	T	C	intergenic	*LOC102477328,TNPO1*	.	.	.
5	72027816	T	A	intergenic	*LOC102477328,TNPO1*	.	.	.
5	72103105	T	C	intergenic	*LOC102477328,TNPO1*	.	.	.
5	72103106	G	A	intergenic	*LOC102477328,TNPO1*	.	.	.
5	71865250	C	T	ncRNA_intronic	*LOC102503427*	.	.	.
5	87810287	G	GAGAGAGA(	intergenic	*LOC102546226,LINC00461*	.	.	.
5	87707685	C	T	ncRNA_intronic	*LOC102546226,TMEM161B-AS1*	.	.	.
5	87711335	G	T	ncRNA_intronic	*LOC102546226,TMEM161B-AS1*	.	.	.
5	133807808	T	C	intergenic	*LOC102546229,LOC101927934*	.	.	.
5	133776337	T	C	intergenic	*LOC102546229,LOC101927934*	.	.	.
5	147654821	A	G	ncRNA_intronic	*LOC102546294*	.	.	.
5	147656731	A	G	ncRNA_intronic	*LOC102546294*	.	.	.
5	147698659	G	C	ncRNA_intronic	*LOC102546294*	.	.	.
5	147739945	C	T	ncRNA_intronic	*LOC102546294*	.	.	.

5	165215406	A	G	intergenic	*LOC102546299,CTB-7E3.1*	.	.	.
5	164355624	A	G	intergenic	*LOC102546299,CTB-7E3.1*	.	.	.
5	165873456	T	C	intergenic	*LOC102546299,CTB-7E3.1*	.	.	.
5	164813619	C	T	intergenic	*LOC102546299,CTB-7E3.1*	.	.	.
5	165704799	G	C	intergenic	*LOC102546299,CTB-7E3.1*	.	.	.
5	164668823	C	A	intergenic	*LOC102546299,CTB-7E3.1*	.	.	.
5	165049790	A	T	intergenic	*LOC102546299,CTB-7E3.1*	.	.	.
5	165522113	C	A	intergenic	*LOC102546299,CTB-7E3.1*	.	.	.
5	165700277	T	A	intergenic	*LOC102546299,CTB-7E3.1*	.	.	.
5	165754862	T	A	intergenic	*LOC102546299,CTB-7E3.1*	.	.	.
5	164358562	T	C	intergenic	*LOC102546299,CTB-7E3.1*	.	.	.
5	164469334	T	A	intergenic	*LOC102546299,CTB-7E3.1*	.	.	.
5	164545098	G	A	intergenic	*LOC102546299,CTB-7E3.1*	.	.	.
5	164841917	G	A	intergenic	*LOC102546299,CTB-7E3.1*	.	.	.
5	165040555	T	C	intergenic	*LOC102546299,CTB-7E3.1*	.	.	.
5	165041705	A	G	intergenic	*LOC102546299,CTB-7E3.1*	.	.	.
5	165115144	G	A	intergenic	*LOC102546299,CTB-7E3.1*	.	.	.
5	165116106	A	G	intergenic	*LOC102546299,CTB-7E3.1*	.	.	.
5	165346981	C	G	intergenic	*LOC102546299,CTB-7E3.1*	.	.	.

5	165392911	C	A	intergenic	*LOC102546299,CTB-7E3.1*	.	.	.
5	165437621	G	A	intergenic	*LOC102546299,CTB-7E3.1*	.	.	.
5	165726971	C	T	intergenic	*LOC102546299,CTB-7E3.1*	.	.	.
5	165808394	G	T	intergenic	*LOC102546299,CTB-7E3.1*	.	.	.
5	165867795	A	G	intergenic	*LOC102546299,CTB-7E3.1*	.	.	.
5	165871890	G	A	intergenic	*LOC102546299,CTB-7E3.1*	.	.	.
5	165872636	A	G	intergenic	*LOC102546299,CTB-7E3.1*	.	.	.
5	165872966	T	G	intergenic	*LOC102546299,CTB-7E3.1*	.	.	.
5	165975520	G	A	intergenic	*LOC102546299,CTB-7E3.1*	.	.	.
5	164173521	C	G	intergenic	*LOC102546299,NONE*	.	.	.
5	164083112	T	A	intergenic	*LOC102546299,NONE*	.	.	.
5	164202141	C	A	intergenic	*LOC102546299,NONE*	.	.	.
5	164021899	C	T	intergenic	*LOC102546299,NONE*	.	.	.
5	163975657	T	TC	intergenic	*LOC102546299,NONE*	.	.	.
5	164013430	T	A	intergenic	*LOC102546299,NONE*	.	.	.
1	101579253	C	G	intergenic	*LOC102606465,LOC101928370*	.	.	.
1	101696933	C	G	intergenic	*LOC102606465,LOC101928370*	.	.	.
14	105580114	C	T	intergenic	*LOC102723354,JAG2*	.	.	.
2	22936947	T	G	intergenic	*LOC102723362,KLHL29*	.	.	.

2	23141854	G	GAATA	intergenic	*LOC102723362,KLHL29*	.	.	.
2	22770868	T	C	intergenic	*LOC102723362,KLHL29*	.	.	.
2	22770869	G	A	intergenic	*LOC102723362,KLHL29*	.	.	.
2	22866659	T	C	intergenic	*LOC102723362,KLHL29*	.	.	.
2	22866661	T	C	intergenic	*LOC102723362,KLHL29*	.	.	.
2	22927142	CAG	C	intergenic	*LOC102723362,KLHL29*	.	.	.
2	23268852	C	T	intergenic	*LOC102723362,KLHL29*	.	.	.
2	23332517	A	T	intergenic	*LOC102723362,KLHL29*	.	.	.
2	23395979	T	G	intergenic	*LOC102723362,KLHL29*	.	.	.
2	23398290	T	A	intergenic	*LOC102723362,KLHL29*	.	.	.
2	23398292	G	A	intergenic	*LOC102723362,KLHL29*	.	.	.
2	23398711	C	T	intergenic	*LOC102723362,KLHL29*	.	.	.
2	23398995	T	C	intergenic	*LOC102723362,KLHL29*	.	.	.
18	107764	T	A	intergenic	*LOC102723376,ROCK1P1*	.	.	.
7	68289618	G	C	intergenic	*LOC102723427,LOC100507468*	.	.	.
7	67626335	A	G	intergenic	*LOC102723427,LOC100507468*	.	.	.
7	67690812	C	T	intergenic	*LOC102723427,LOC100507468*	.	.	.
7	67815485	T	G	intergenic	*LOC102723427,LOC100507468*	.	.	.
7	68260301	G	A	intergenic	*LOC102723427,LOC100507468*	.	.	.

7	68313263	A	T	intergenic	*LOC102723427,LOC100507468*	.	.	.
7	68357588	A	G	intergenic	*LOC102723427,LOC100507468*	.	.	.
7	68615893	G	T	intergenic	*LOC102723427,LOC100507468*	.	.	.
7	68855702	C	T	intergenic	*LOC102723427,LOC100507468*	.	.	.
7	68456362	G	C	intergenic	*LOC102723427,LOC100507468*	.	.	.
7	68652673	T	C	intergenic	*LOC102723427,LOC100507468*	.	.	.
7	67809406	G	A	intergenic	*LOC102723427,LOC100507468*	.	.	.
7	68145330	AAGGAAGGACGGC	A	intergenic	*LOC102723427,LOC100507468*	.	.	.
3	66083	T	TTC	ncRNA_exonic	*LOC102723448*	.	.	.
3	198001	G	T	intergenic	*LOC102723448,CHL1*	.	.	.
17	70025652	G	A	intergenic	*LOC102723505,LINC01152*	.	.	.
6	137994827	G	T	ncRNA_intronic	*LOC102723649*	.	.	.
1	96631067	T	A	intergenic	*LOC102723661,LOC101928241*	.	.	.
1	143155973	G	A	ncRNA_intronic	*LOC102723769*	.	.	.
1	143183378	T	C	ncRNA_intronic	*LOC102723769*	.	.	.
1	143141152	T	A	ncRNA_intronic	*LOC102723769*	.	.	.
1	143147707	G	T	ncRNA_intronic	*LOC102723769*	.	.	.
1	143147708	GT	G	ncRNA_intronic	*LOC102723769*	.	.	.
1	143158344	A	G	ncRNA_intronic	*LOC102723769*	.	.	.
1	143175140	C	T	ncRNA_intronic	*LOC102723769*	.	.	.
1	143160315	A	C	ncRNA_intronic	*LOC102723769*	.	.	.
1	143362067	C	T	intergenic	*LOC102723769,MIR6077*	.	.	.
1	143444075	T	C	intergenic	*LOC102723769,MIR6077*	.	.	.
1	143531466	C	T	intergenic	*LOC102723769,MIR6077*	.	.	.

1	143257738	A	AATAATG	intergenic	*LOC102723769,MIR6077*	.	.	.
1	143269881	A	G	intergenic	*LOC102723769,MIR6077*	.	.	.
1	143391922	G	GAT	intergenic	*LOC102723769,MIR6077*	.	.	.
1	143467182	C	A	intergenic	*LOC102723769,MIR6077*	.	.	.
1	143217128	C	CT	intergenic	*LOC102723769,MIR6077*	.	.	.
1	143530689	CT	C	intergenic	*LOC102723769,MIR6077*	.	.	.
1	143274945	C	T	intergenic	*LOC102723769,MIR6077*	.	.	.
1	143521643	C	T	intergenic	*LOC102723769,MIR6077*	.	.	.
4	31173208	ACC	A	ncRNA_intronic	*LOC102723778*	.	.	.
4	31602317	G	T	intergenic	*LOC102723778,LOC102723828*	.	.	.
4	31949454	A	G	intergenic	*LOC102723778,LOC102723828*	.	.	.
4	31523582	A	T	intergenic	*LOC102723778,LOC102723828*	.	.	.
4	31845368	T	A	intergenic	*LOC102723778,LOC102723828*	.	.	.
4	31303905	T	A	intergenic	*LOC102723778,LOC102723828*	.	.	.
4	31900408	C	A	intergenic	*LOC102723778,LOC102723828*	.	.	.
4	31283698	C	A	intergenic	*LOC102723778,LOC102723828*	.	.	.
4	31402210	C	T	intergenic	*LOC102723778,LOC102723828*	.	.	.
4	31438174	T	A	intergenic	*LOC102723778,LOC102723828*	.	.	.
4	31624525	C	T	intergenic	*LOC102723778,LOC102723828*	.	.	.

4	31647740	C	T	intergenic	*LOC102723778,LOC102723828*	.	.	.
4	31998484	G	C	upstream	*LOC102723828*	.	.	.
4	32051735	T	G	ncRNA_intronic	*LOC102723828*	.	.	.
4	32051736	C	A	ncRNA_intronic	*LOC102723828*	.	.	.
4	32144912	A	ATAGGG	ncRNA_intronic	*LOC102723828*	.	.	.
4	32071001	G	GAAAAATAC	ncRNA_intronic	*LOC102723828*	.	.	.
4	32015838	T	C	ncRNA_intronic	*LOC102723828*	.	.	.
4	32016119	T	C	ncRNA_intronic	*LOC102723828*	.	.	.
4	32047748	T	A	ncRNA_intronic	*LOC102723828*	.	.	.
4	32144702	C	T	ncRNA_intronic	*LOC102723828*	.	.	.
4	33024168	C	T	intergenic	*LOC102723828,LOC101928622*	.	.	.
4	33805527	G	T	intergenic	*LOC102723828,LOC101928622*	.	.	.
4	33162086	G	A	intergenic	*LOC102723828,LOC101928622*	.	.	.
4	33397728	G	T	intergenic	*LOC102723828,LOC101928622*	.	.	.
4	33604694	A	T	intergenic	*LOC102723828,LOC101928622*	.	.	.
4	33796889	C	A	intergenic	*LOC102723828,LOC101928622*	.	.	.
4	33888785	G	C	intergenic	*LOC102723828,LOC101928622*	.	.	.
4	32663307	GACACAC	G	intergenic	*LOC102723828,LOC101928622*	.	.	.
4	33167306	C	T	intergenic	*LOC102723828,LOC101928622*	.	.	.
4	33700249	C	A	intergenic	*LOC102723828,LOC101928622*	.	.	.
4	33711946	C	A	intergenic	*LOC102723828,LOC101928622*	.	.	.
4	33746343	G	T	intergenic	*LOC102723828,LOC101928622*	.	.	.
4	32942533	T	C	intergenic	*LOC102723828,LOC101928622*	.	.	.

4	33325163	T	G	intergenic	*LOC102723828,LOC101928622*	.	.	.
4	33586584	C	A	intergenic	*LOC102723828,LOC101928622*	.	.	.
4	32566293	C	T	intergenic	*LOC102723828,LOC101928622*	.	.	.
4	32817905	G	T	intergenic	*LOC102723828,LOC101928622*	.	.	.
4	33130024	C	G	intergenic	*LOC102723828,LOC101928622*	.	.	.
4	33264795	GA	G	intergenic	*LOC102723828,LOC101928622*	.	.	.
4	33279184	C	T	intergenic	*LOC102723828,LOC101928622*	.	.	.
4	33384509	C	G	intergenic	*LOC102723828,LOC101928622*	.	.	.
4	33449249	A	T	intergenic	*LOC102723828,LOC101928622*	.	.	.
4	33523109	A	T	intergenic	*LOC102723828,LOC101928622*	.	.	.
4	33647250	T	C	intergenic	*LOC102723828,LOC101928622*	.	.	.
4	33647251	C	A	intergenic	*LOC102723828,LOC101928622*	.	.	.
4	32643305	CTATATATACTATATATACACTATATATATACTATATAGTGTATATATAGTATATATATATACTATATATACTATATATACACTATATAGTATATATATAGTGTA	C	intergenic	*LOC102723828,LOC101928622*	.	.	.

4	32219533	ATATATATATATATATATATAT	A	intergenic	*LOC102723828,LOC101928622*	.	.	.
4	32385960	A	G	intergenic	*LOC102723828,LOC101928622*	.	.	.
4	32413631	T	C	intergenic	*LOC102723828,LOC101928622*	.	.	.
4	32519304	C	T	intergenic	*LOC102723828,LOC101928622*	.	.	.
4	32519955	C	T	intergenic	*LOC102723828,LOC101928622*	.	.	.
4	32942106	C	T	intergenic	*LOC102723828,LOC101928622*	.	.	.
4	32950198	C	T	intergenic	*LOC102723828,LOC101928622*	.	.	.
4	33232022	A	G	intergenic	*LOC102723828,LOC101928622*	.	.	.
4	33290833	A	G	intergenic	*LOC102723828,LOC101928622*	.	.	.
4	33310402	C	T	intergenic	*LOC102723828,LOC101928622*	.	.	.
4	33458353	C	T	intergenic	*LOC102723828,LOC101928622*	.	.	.
4	33553931	G	A	intergenic	*LOC102723828,LOC101928622*	.	.	.
4	33554797	C	A	intergenic	*LOC102723828,LOC101928622*	.	.	.
4	33664198	C	G	intergenic	*LOC102723828,LOC101928622*	.	.	.
4	33666934	A	C	intergenic	*LOC102723828,LOC101928622*	.	.	.
4	33695876	C	A	intergenic	*LOC102723828,LOC101928622*	.	.	.
4	33696092	A	G	intergenic	*LOC102723828,LOC101928622*	.	.	.
4	33755938	C	T	intergenic	*LOC102723828,LOC101928622*	.	.	.

4	33809096	G	A	intergenic	*LOC102723828,LOC101928622*	.	.	.
4	33868047	T	C	intergenic	*LOC102723828,LOC101928622*	.	.	.
4	33869398	C	T	intergenic	*LOC102723828,LOC101928622*	.	.	.
4	33870342	T	C	intergenic	*LOC102723828,LOC101928622*	.	.	.
4	33871553	A	G	intergenic	*LOC102723828,LOC101928622*	.	.	.
6	68823767	AG	A	intergenic	*LOC102723883,LOC101928280*	.	.	.
6	68823769	A	T	intergenic	*LOC102723883,LOC101928280*	.	.	.
6	68887010	G	A	intergenic	*LOC102723883,LOC101928280*	.	.	.
11	104476427	A	G	ncRNA_intronic	*LOC102723895*	.	.	.
11	104555236	G	C	intergenic	*LOC102723895,CASP12*	.	.	.
11	104574630	T	C	intergenic	*LOC102723895,CASP12*	.	.	.
11	104487744	A	G	intergenic	*LOC102723895,CASP12*	.	.	.
11	104519410	G	C	intergenic	*LOC102723895,CASP12*	.	.	.
11	104740507	G	GTGTATA	intergenic	*LOC102723895,CASP12*	.	.	.
11	104487544	A	G	intergenic	*LOC102723895,CASP12*	.	.	.
11	104547639	GGTGTGTGTGT	G	intergenic	*LOC102723895,CASP12*	.	.	.
13	64823199	T	C	intergenic	*LOC102723968,LINC01052*	.	.	.
13	66205147	A	T	intergenic	*LOC102723968,LINC01052*	.	.	.
13	66281773	C	T	intergenic	*LOC102723968,LINC01052*	.	.	.

13	64541143	A	G	intergenic	*LOC102723968,LINC01052*	.	.	.
13	65000416	A	G	intergenic	*LOC102723968,LINC01052*	.	.	.
13	65876361	TTATATA	T	intergenic	*LOC102723968,LINC01052*	.	.	.
13	65410950	A	G	intergenic	*LOC102723968,LINC01052*	.	.	.
13	65659307	A	T	intergenic	*LOC102723968,LINC01052*	.	.	.
13	65834708	A	T	intergenic	*LOC102723968,LINC01052*	.	.	.
13	65346060	T	C	intergenic	*LOC102723968,LINC01052*	.	.	.
13	65199731	T	A	intergenic	*LOC102723968,LINC01052*	.	.	.
13	64690540	G	T	intergenic	*LOC102723968,LINC01052*	.	.	.
13	64785537	C	G	intergenic	*LOC102723968,LINC01052*	.	.	.
13	65294064	G	A	intergenic	*LOC102723968,LINC01052*	.	.	.
13	65294065	G	C	intergenic	*LOC102723968,LINC01052*	.	.	.
13	65294888	A	G	intergenic	*LOC102723968,LINC01052*	.	.	.
13	65294890	A	T	intergenic	*LOC102723968,LINC01052*	.	.	.
13	65305047	T	C	intergenic	*LOC102723968,LINC01052*	.	.	.
13	65347962	G	A	intergenic	*LOC102723968,LINC01052*	.	.	.
13	65406637	T	A	intergenic	*LOC102723968,LINC01052*	.	.	.
13	65433656	A	T	intergenic	*LOC102723968,LINC01052*	.	.	.
13	65551693	T	A	intergenic	*LOC102723968,LINC01052*	.	.	.

13	65560707	G	A	intergenic	*LOC102723968,LINC01052*	.	.	.
13	65593328	A	AT	intergenic	*LOC102723968,LINC01052*	.	.	.
13	65612173	C	T	intergenic	*LOC102723968,LINC01052*	.	.	.
13	65680139	T	C	intergenic	*LOC102723968,LINC01052*	.	.	.
13	65682045	T	A	intergenic	*LOC102723968,LINC01052*	.	.	.
13	65795136	A	G	intergenic	*LOC102723968,LINC01052*	.	.	.
13	65795138	C	T	intergenic	*LOC102723968,LINC01052*	.	.	.
13	65795706	T	C	intergenic	*LOC102723968,LINC01052*	.	.	.
13	65796721	T	A	intergenic	*LOC102723968,LINC01052*	.	.	.
13	66011398	A	C	intergenic	*LOC102723968,LINC01052*	.	.	.
13	66012206	A	T	intergenic	*LOC102723968,LINC01052*	.	.	.
13	66065631	C	T	intergenic	*LOC102723968,LINC01052*	.	.	.
13	66065632	A	G	intergenic	*LOC102723968,LINC01052*	.	.	.
13	66122332	C	T	intergenic	*LOC102723968,LINC01052*	.	.	.
13	66138331	C	G	intergenic	*LOC102723968,LINC01052*	.	.	.
13	66247431	A	AG	intergenic	*LOC102723968,LINC01052*	.	.	.
13	66280535	T	A	intergenic	*LOC102723968,LINC01052*	.	.	.
6	160095410	T	C	intergenic	*LOC102724053,SOD2*	.	.	.
16	80553622	G	A	ncRNA_intronic	*LOC102724084*	.	.	.
16	80277245	C	G	ncRNA_intronic	*LOC102724084*	.	.	.

16	80487776	T	A	ncRNA_intronic	*LOC102724084*	.	.	.
16	80256638	T	C	ncRNA_intronic	*LOC102724084*	.	.	.
16	80256747	A	G	ncRNA_intronic	*LOC102724084*	.	.	.
6	4390097	G	A	intergenic	*LOC102724096,MIR7641-2*	.	.	.
6	4334631	G	A	intergenic	*LOC102724096,MIR7641-2*	.	.	.
6	4202210	G	A	intergenic	*LOC102724096,MIR7641-2*	.	.	.
6	4329040	T	C	intergenic	*LOC102724096,MIR7641-2*	.	.	.
6	4330410	T	C	intergenic	*LOC102724096,MIR7641-2*	.	.	.
6	4409053	G	A	intergenic	*LOC102724096,MIR7641-2*	.	.	.
6	4411437	C	T	intergenic	*LOC102724096,MIR7641-2*	.	.	.
21	14949537	T	C	intergenic	*LOC102724188,POTED*	.	.	.
11	119965223	T	C	intergenic	*LOC102724301,TRIM29*	.	.	.
11	119825798	C	CTAA	intergenic	*LOC102724301,TRIM29*	.	.	.
11	119686951	C	T	intergenic	*LOC102724301,TRIM29*	.	.	.
11	119707343	A	G	intergenic	*LOC102724301,TRIM29*	.	.	.
12	67564657	GA	G	intergenic	*LOC102724421,CAND1*	.	.	.
12	67640883	C	T	intergenic	*LOC102724421,CAND1*	.	.	.
12	67641288	C	T	intergenic	*LOC102724421,CAND1*	.	.	.
7	116120611	T	C	intergenic	*LOC102724434,CAV2*	.	.	.
7	116120612	G	C	intergenic	*LOC102724434,CAV2*	.	.	.

10	116834920	A	G	intergenic	*LOC102724589,ATRNL1*	.	.	.
1	174115815	T	C	ncRNA_intronic	*LOC102724601*	.	.	.
8	64558231	C	G	intergenic	*LOC102724612,LINC01289*	.	.	.
8	64455028	A	G	intergenic	*LOC102724612,LINC01289*	.	.	.
8	64531057	T	G	intergenic	*LOC102724612,LINC01289*	.	.	.
8	64418010	C	G	intergenic	*LOC102724612,LINC01289*	.	.	.
8	64527906	T	C	intergenic	*LOC102724612,LINC01289*	.	.	.
8	64527907	G	A	intergenic	*LOC102724612,LINC01289*	.	.	.
8	64528416	G	A	intergenic	*LOC102724612,LINC01289*	.	.	.
8	64668556	A	G	intergenic	*LOC102724612,LINC01289*	.	.	.
8	65179754	G	A	ncRNA_intronic	*LOC102724623*	.	.	.
8	64790341	G	T	ncRNA_intronic	*LOC102724623*	.	.	.
8	65111651	G	C	ncRNA_intronic	*LOC102724623*	.	.	.
8	65224243	A	G	ncRNA_intronic	*LOC102724623*	.	.	.
8	64826493	CTGAACTACCAGGCTCAGTGGATTTCAGATG	C	ncRNA_intronic	*LOC102724623*	.	.	.
8	65015491	C	A	ncRNA_intronic	*LOC102724623*	.	.	.
8	65060044	A	G	ncRNA_intronic	*LOC102724623*	.	.	.
8	64982464	C	A	ncRNA_intronic	*LOC102724623*	.	.	.
8	65000013	C	T	ncRNA_intronic	*LOC102724623*	.	.	.
8	65018060	G	A	ncRNA_intronic	*LOC102724623*	.	.	.
8	65020463	A	G	ncRNA_intronic	*LOC102724623*	.	.	.
8	65155682	G	A	ncRNA_intronic	*LOC102724623*	.	.	.
8	65230931	C	G	ncRNA_intronic	*LOC102724623*	.	.	.
8	65232070	A	G	ncRNA_intronic	*LOC102724623*	.	.	.
8	65233110	C	T	ncRNA_intronic	*LOC102724623*	.	.	.
18	51118268	G	A	ncRNA_intronic	*LOC102724651*	.	.	.

18	51373641	G	T	intergenic	*LOC102724651,MBD2*	.	.	.
18	51132383	G	T	intergenic	*LOC102724651,MBD2*	.	.	.
18	51167129	T	C	intergenic	*LOC102724651,MBD2*	.	.	.
18	51333339	A	T	intergenic	*LOC102724651,MBD2*	.	.	.
18	51334838	A	G	intergenic	*LOC102724651,MBD2*	.	.	.
18	51334840	G	A	intergenic	*LOC102724651,MBD2*	.	.	.
18	51335034	A	G	intergenic	*LOC102724651,MBD2*	.	.	.
18	51350045	T	G	intergenic	*LOC102724651,MBD2*	.	.	.
18	51350253	C	T	intergenic	*LOC102724651,MBD2*	.	.	.
18	51357379	A	G	intergenic	*LOC102724651,MBD2*	.	.	.
18	51357381	C	T	intergenic	*LOC102724651,MBD2*	.	.	.
18	51358529	GT	G	intergenic	*LOC102724651,MBD2*	.	.	.
18	51358740	C	T	intergenic	*LOC102724651,MBD2*	.	.	.
18	51383188	A	G	intergenic	*LOC102724651,MBD2*	.	.	.
18	51502516	A	G	intergenic	*LOC102724651,MBD2*	.	.	.
1	177505503	C	T	intergenic	*LOC102724661,LOC101928778*	.	.	.
1	177385945	A	G	intergenic	*LOC102724661,LOC101928778*	.	.	.
1	177461454	G	T	intergenic	*LOC102724661,LOC101928778*	.	.	.
1	177479133	A	G	intergenic	*LOC102724661,LOC101928778*	.	.	.

1	177641891	C	T	intergenic	*LOC102724661,LOC101928778*	.	.	.
1	177664437	G	T	intergenic	*LOC102724661,LOC101928778*	.	.	.
2	105642387	G	A	ncRNA_intronic	*LOC102724691*	.	.	.
2	105596013	G	GA	ncRNA_intronic	*LOC102724691*	.	.	.
2	105571611	A	C	ncRNA_intronic	*LOC102724691*	.	.	.
2	105571811	C	T	ncRNA_intronic	*LOC102724691*	.	.	.
2	105571934	G	T	ncRNA_intronic	*LOC102724691*	.	.	.
2	105574753	T	C	ncRNA_intronic	*LOC102724691*	.	.	.
8	93720022	AATAT	A	intergenic	*LOC102724710,FLJ46284*	.	.	.
8	93716471	T	C	intergenic	*LOC102724710,FLJ46284*	.	.	.
8	97398106	TTCTCTC	T	ncRNA_intronic	*LOC102724804*	.	.	.
2	216414916	T	C	intergenic	*LOC102724849,LINC00607*	.	.	.
8	78610474	G	A	intergenic	*LOC102724874,PKIA*	.	.	.
8	78970764	G	T	intergenic	*LOC102724874,PKIA*	.	.	.
8	79050121	T	C	intergenic	*LOC102724874,PKIA*	.	.	.
8	78437748	G	T	intergenic	*LOC102724874,PKIA*	.	.	.
8	78599241	ACT	A	intergenic	*LOC102724874,PKIA*	.	.	.
8	78913759	T	A	intergenic	*LOC102724874,PKIA*	.	.	.
8	79256827	AAGAG	A	intergenic	*LOC102724874,PKIA*	.	.	.
8	78516274	G	T	intergenic	*LOC102724874,PKIA*	.	.	.
8	78517068	T	G	intergenic	*LOC102724874,PKIA*	.	.	.
8	78608379	G	A	intergenic	*LOC102724874,PKIA*	.	.	.

8	78766465	C	T	intergenic	*LOC102724874,PKIA*	.	.	.
8	78829324	A	T	intergenic	*LOC102724874,PKIA*	.	.	.
8	78887272	G	A	intergenic	*LOC102724874,PKIA*	.	.	.
8	78887274	C	G	intergenic	*LOC102724874,PKIA*	.	.	.
8	78932724	T	C	intergenic	*LOC102724874,PKIA*	.	.	.
8	78933137	C	T	intergenic	*LOC102724874,PKIA*	.	.	.
8	78967838	G	C	intergenic	*LOC102724874,PKIA*	.	.	.
8	79016472	C	T	intergenic	*LOC102724874,PKIA*	.	.	.
8	79294461	A	G	intergenic	*LOC102724874,PKIA*	.	.	.
8	79359720	T	G	intergenic	*LOC102724874,PKIA*	.	.	.
8	79361840	T	A	intergenic	*LOC102724874,PKIA*	.	.	.
18	69464794	A	G	intergenic	*LOC102724913,CBLN2*	.	.	.
18	69961553	A	G	intergenic	*LOC102724913,CBLN2*	.	.	.
18	69758184	T	A	intergenic	*LOC102724913,CBLN2*	.	.	.
18	69954439	T	G	intergenic	*LOC102724913,CBLN2*	.	.	.
18	70050606	TG	T	intergenic	*LOC102724913,CBLN2*	.	.	.
18	70050608	TTTGTGTGTGTG	T	intergenic	*LOC102724913,CBLN2*	.	.	.
18	69500247	A	G	intergenic	*LOC102724913,CBLN2*	.	.	.
18	69552642	T	C	intergenic	*LOC102724913,CBLN2*	.	.	.

18	70096224	C	G	intergenic	*LOC102724913,CBLN2*	.	.	.
18	69574014	C	T	intergenic	*LOC102724913,CBLN2*	.	.	.
18	69623394	G	A	intergenic	*LOC102724913,CBLN2*	.	.	.
18	69802068	C	G	intergenic	*LOC102724913,CBLN2*	.	.	.
18	69943641	A	G	intergenic	*LOC102724913,CBLN2*	.	.	.
18	69943642	T	A	intergenic	*LOC102724913,CBLN2*	.	.	.
1	186408124	C	T	ncRNA_intronic	*LOC102724919,MIR548F1*	.	.	.
1	186435827	C	T	ncRNA_intronic	*LOC102724919,MIR548F1*	.	.	.
11	15682782	A	G	intronic	*LOC102724957*	.	.	.
11	15782866	A	G	intergenic	*LOC102724957,SOX6*	.	.	.
2	213672844	C	T	ncRNA_intronic	*LOC102725079*	.	.	.
2	213756492	TTAATTTATTAATTTAAATA	T	intergenic	*LOC102725079,MIR4776-2*	.	.	.
2	213725482	A	G	intergenic	*LOC102725079,MIR4776-2*	.	.	.
2	213726031	T	C	intergenic	*LOC102725079,MIR4776-2*	.	.	.
2	213742999	T	G	intergenic	*LOC102725079,MIR4776-2*	.	.	.
2	213743202	G	A	intergenic	*LOC102725079,MIR4776-2*	.	.	.
2	213743297	T	C	intergenic	*LOC102725079,MIR4776-2*	.	.	.
2	213744303	G	A	intergenic	*LOC102725079,MIR4776-2*	.	.	.
2	213745085	C	T	intergenic	*LOC102725079,MIR4776-2*	.	.	.

2	213750523	G	A	intergenic	*LOC102725079,MIR4776-2*	.	.	.
2	213750638	A	G	intergenic	*LOC102725079,MIR4776-2*	.	.	.
2	67346641	A	G	ncRNA_intronic	*LOC102800447*	.	.	.
2	67586150	T	A	intergenic	*LOC102800447,ETAA1*	.	.	.
2	67589356	T	C	intergenic	*LOC102800447,ETAA1*	.	.	.
2	67366638	A	G	ncRNA_intronic	*LOC102800447,LOC644838*	.	.	.
2	67419960	C	T	ncRNA_intronic	*LOC102800447,LOC644838*	.	.	.
1	149656844	C	T	ncRNA_intronic	*LOC103091866*	.	.	.
1	149689129	A	AAG	intergenic	*LOC103091866,HIST2H2BF*	.	.	.
10	114701976	G	A	intergenic	*LOC103344931,TCF7L2*	.	.	.
9	44801632	G	T	intergenic	*LOC103908605,FAM27C*	.	.	.
9	44873060	C	T	intergenic	*LOC103908605,FAM27C*	.	.	.
9	44872347	T	C	intergenic	*LOC103908605,FAM27C*	.	.	.
9	46909360	CAAATAAATAAAT	C	intergenic	*LOC103908605,NONE*	.	.	.
9	47216644	TAGTC	T	intergenic	*LOC103908605,NONE*	.	.	.
13	88901454	T	C	intergenic	*LOC105370306,LINC00433*	.	.	.
13	88901455	T	C	intergenic	*LOC105370306,LINC00433*	.	.	.
8	123500852	T	A	ncRNA_intronic	*LOC105375734*	.	.	.
10	3489286	C	A	ncRNA_intronic	*LOC105376360*	.	.	.
10	3673912	GCTCT	G	ncRNA_intronic	*LOC105376360*	.	.	.
10	3723120	G	A	ncRNA_intronic	*LOC105376360*	.	.	.
10	3745667	ATCTC	A	ncRNA_intronic	*LOC105376360*	.	.	.
2	168731567	C	G	ncRNA_intronic	*LOC105616981*	.	.	.

2	168693972	C	A	ncRNA_intronic	*LOC105616981*	.	.	.
2	168694335	C	G	ncRNA_intronic	*LOC105616981*	.	.	.
2	192593620	A	G	intergenic	*LOC105747689,SDPR*	.	.	.
2	192640939	C	T	intergenic	*LOC105747689,SDPR*	.	.	.
10	8948537	T	C	ncRNA_intronic	*LOC105755953*	.	.	.
15	57196722	A	G	ncRNA_intronic	*LOC145783*	.	.	.
15	57196723	A	G	ncRNA_intronic	*LOC145783*	.	.	.
1	208028027	C	T	intergenic	*LOC148696,CD34*	.	.	.
2	240760150	G	A	intergenic	*LOC150935,MIR4786*	.	.	.
2	130008726	A	C	ncRNA_intronic	*LOC151121*	.	.	.
2	130046871	A	ATAT	intergenic	*LOC151121,LOC389033*	.	.	.
2	130307583	A	C	intergenic	*LOC151121,LOC389033*	.	.	.
2	130668905	T	C	intergenic	*LOC151121,LOC389033*	.	.	.
2	130368513	G	A	intergenic	*LOC151121,LOC389033*	.	.	.
2	130397754	T	A	intergenic	*LOC151121,LOC389033*	.	.	.
2	130443842	G	A	intergenic	*LOC151121,LOC389033*	.	.	.
2	130259077	A	C	intergenic	*LOC151121,LOC389033*	.	.	.
2	130330924	A	G	intergenic	*LOC151121,LOC389033*	.	.	.
2	130330925	G	T	intergenic	*LOC151121,LOC389033*	.	.	.
2	130600206	G	A	intergenic	*LOC151121,LOC389033*	.	.	.
3	101755975	T	C	intergenic	*LOC152225,ZPLD1*	.	.	.
3	101764022	A	T	intergenic	*LOC152225,ZPLD1*	.	.	.
6	142903466	A	G	ncRNA_intronic	*LOC153910*	.	.	.

7	149048141	G	T	intergenic	*LOC155060,ZNF777*	.	.	.
7	149040689	C	T	intergenic	*LOC155060,ZNF777*	.	.	.
8	9285311	C	T	intergenic	*LOC157273,TNKS*	.	.	.
8	9300540	T	C	intergenic	*LOC157273,TNKS*	.	.	.
2	83515924	C	CCA	intergenic	*LOC1720,FUNDC2P2*	.	.	.
2	84505591	C	G	intergenic	*LOC1720,FUNDC2P2*	.	.	.
2	83129937	C	G	intergenic	*LOC1720,FUNDC2P2*	.	.	.
2	83339442	G	C	intergenic	*LOC1720,FUNDC2P2*	.	.	.
2	83780020	G	C	intergenic	*LOC1720,FUNDC2P2*	.	.	.
2	83915738	A	T	intergenic	*LOC1720,FUNDC2P2*	.	.	.
2	83915739	T	G	intergenic	*LOC1720,FUNDC2P2*	.	.	.
2	83102722	G	A	intergenic	*LOC1720,FUNDC2P2*	.	.	.
2	83855839	T	G	intergenic	*LOC1720,FUNDC2P2*	.	.	.
2	84022980	T	G	intergenic	*LOC1720,FUNDC2P2*	.	.	.
2	83301413	G	A	intergenic	*LOC1720,FUNDC2P2*	.	.	.
2	83793172	G	C	intergenic	*LOC1720,FUNDC2P2*	.	.	.
2	84220353	G	T	intergenic	*LOC1720,FUNDC2P2*	.	.	.
2	83577518	C	T	intergenic	*LOC1720,FUNDC2P2*	.	.	.
2	83577786	C	T	intergenic	*LOC1720,FUNDC2P2*	.	.	.
2	83628926	A	C	intergenic	*LOC1720,FUNDC2P2*	.	.	.

2	83716808	A	G	intergenic	*LOC1720,FUNDC2P2*	.	.	.
2	83724536	T	C	intergenic	*LOC1720,FUNDC2P2*	.	.	.
2	83724792	C	G	intergenic	*LOC1720,FUNDC2P2*	.	.	.
2	83724794	T	C	intergenic	*LOC1720,FUNDC2P2*	.	.	.
2	84063879	T	A	intergenic	*LOC1720,FUNDC2P2*	.	.	.
2	84206303	G	A	intergenic	*LOC1720,FUNDC2P2*	.	.	.
2	84206604	A	G	intergenic	*LOC1720,FUNDC2P2*	.	.	.
2	84482499	A	G	intergenic	*LOC1720,FUNDC2P2*	.	.	.
2	84490425	A	G	intergenic	*LOC1720,FUNDC2P2*	.	.	.
2	84491762	G	A	intergenic	*LOC1720,FUNDC2P2*	.	.	.
2	84494660	C	T	intergenic	*LOC1720,FUNDC2P2*	.	.	.
5	177062505	C	T	ncRNA_intronic	*LOC202181*	.	.	.
3	197384038	G	A	intergenic	*LOC220729,RUBCN*	.	.	.
5	148457783	A	G	ncRNA_intronic	*LOC255187*	.	.	.
4	101077961	A	T	intergenic	*LOC256880,DDIT4L*	.	.	.
5	52416341	TTCTC	T	intergenic	*LOC257396,FST*	.	.	.
5	52481557	A	G	intergenic	*LOC257396,FST*	.	.	.
5	52481559	T	G	intergenic	*LOC257396,FST*	.	.	.
5	52482614	C	A	intergenic	*LOC257396,FST*	.	.	.
5	52482880	T	C	intergenic	*LOC257396,FST*	.	.	.
5	52567195	C	T	intergenic	*LOC257396,FST*	.	.	.
5	52567196	A	G	intergenic	*LOC257396,FST*	.	.	.
5	52601685	A	G	intergenic	*LOC257396,FST*	.	.	.
5	52604151	A	C	intergenic	*LOC257396,FST*	.	.	.
5	52666654	A	AT	intergenic	*LOC257396,FST*	.	.	.

11	112155428	T	C	ncRNA_intronic	*LOC283140*	.	.	.
11	112196734	T	C	ncRNA_intronic	*LOC283140*	.	.	.
11	112222482	G	A	ncRNA_intronic	*LOC283140*	.	.	.
11	112242735	C	T	intergenic	*LOC283140,LOC101928823*	.	.	.
11	112243665	T	G	intergenic	*LOC283140,LOC101928823*	.	.	.
11	112297854	T	C	intergenic	*LOC283140,LOC101928823*	.	.	.
11	112300956	A	G	intergenic	*LOC283140,LOC101928823*	.	.	.
11	112301425	G	A	intergenic	*LOC283140,LOC101928823*	.	.	.
11	134322314	T	C	ncRNA_intronic	*LOC283177*	.	.	.
11	134363049	A	G	ncRNA_intronic	*LOC283177*	.	.	.
11	134363191	G	T	ncRNA_intronic	*LOC283177*	.	.	.
11	134737538	G	A	intergenic	*LOC283177,NONE*	.	.	.
11	134413793	C	T	intergenic	*LOC283177,NONE*	.	.	.
11	134567299	G	C	intergenic	*LOC283177,NONE*	.	.	.
11	134651419	C	T	intergenic	*LOC283177,NONE*	.	.	.
11	134849348	A	AT	intergenic	*LOC283177,NONE*	.	.	.
11	134714414	A	C	intergenic	*LOC283177,NONE*	.	.	.
11	134806099	C	T	intergenic	*LOC283177,NONE*	.	.	.
11	134896205	AT	A	intergenic	*LOC283177,NONE*	.	.	.
11	134803940	A	G	intergenic	*LOC283177,NONE*	.	.	.
11	134909942	C	T	intergenic	*LOC283177,NONE*	.	.	.
11	134420191	A	C	intergenic	*LOC283177,NONE*	.	.	.
11	134592748	G	A	intergenic	*LOC283177,NONE*	.	.	.
11	134883821	C	T	intergenic	*LOC283177,NONE*	.	.	.
11	134883822	A	T	intergenic	*LOC283177,NONE*	.	.	.
11	58792524	A	C	ncRNA_intronic	*LOC283194*	.	.	.
11	58741317	C	T	ncRNA_intronic	*LOC283194*	.	.	.
11	58845065	C	A	intergenic	*LOC283194,FAM111B*	.	.	.
12	2892325	A	G	intergenic	*LOC283440,FKBP4*	.	.	.
14	87378854	T	C	ncRNA_intronic	*LOC283585*	.	.	.
14	88294636	T	G	intergenic	*LOC283585,GALC*	.	.	.

14	88027220	AACACAC	A	intergenic	*LOC283585,GALC*	.	.	.
14	87472280	G	T	intergenic	*LOC283585,GALC*	.	.	.
14	87649357	A	T	intergenic	*LOC283585,GALC*	.	.	.
14	87501189	C	T	intergenic	*LOC283585,GALC*	.	.	.
14	87699966	G	A	intergenic	*LOC283585,GALC*	.	.	.
14	87981046	AT	A	intergenic	*LOC283585,GALC*	.	.	.
14	87894650	CA	C	intergenic	*LOC283585,GALC*	.	.	.
14	87471348	GTCTC	G	intergenic	*LOC283585,GALC*	.	.	.
14	87482899	A	G	intergenic	*LOC283585,GALC*	.	.	.
14	87824282	T	G	intergenic	*LOC283585,GALC*	.	.	.
14	87882058	A	G	intergenic	*LOC283585,GALC*	.	.	.
14	87890837	C	T	intergenic	*LOC283585,GALC*	.	.	.
14	87894533	T	A	intergenic	*LOC283585,GALC*	.	.	.
14	88245571	G	T	intergenic	*LOC283585,GALC*	.	.	.
14	88378683	C	T	intergenic	*LOC283585,GALC*	.	.	.
14	88379757	T	A	intergenic	*LOC283585,GALC*	.	.	.
14	88379758	A	G	intergenic	*LOC283585,GALC*	.	.	.
14	88397814	C	T	intergenic	*LOC283585,GALC*	.	.	.
15	23159535	C	T	intergenic	*LOC283683,WHAMMP3*	.	.	.
18	62296723	T	TTTCCTTCCT	intergenic	*LOC284294,CDH7*	.	.	.
18	62158035	T	G	intergenic	*LOC284294,CDH7*	.	.	.
18	62392033	T	C	intergenic	*LOC284294,CDH7*	.	.	.
18	62766421	C	A	intergenic	*LOC284294,CDH7*	.	.	.
18	62785879	A	G	intergenic	*LOC284294,CDH7*	.	.	.
18	62853251	C	A	intergenic	*LOC284294,CDH7*	.	.	.
18	62939075	A	T	intergenic	*LOC284294,CDH7*	.	.	.
18	62849371	A	G	intergenic	*LOC284294,CDH7*	.	.	.
18	62291138	GTCTC	G	intergenic	*LOC284294,CDH7*	.	.	.
18	62124280	T	C	intergenic	*LOC284294,CDH7*	.	.	.
18	62242588	C	T	intergenic	*LOC284294,CDH7*	.	.	.
18	62245586	A	T	intergenic	*LOC284294,CDH7*	.	.	.
18	62837732	T	A	intergenic	*LOC284294,CDH7*	.	.	.
18	62838482	T	C	intergenic	*LOC284294,CDH7*	.	.	.
18	63066865	G	A	intergenic	*LOC284294,CDH7*	.	.	.
19	43747103	C	T	ncRNA_intronic	*LOC284344*	.	.	.
19	43750162	A	G	ncRNA_intronic	*LOC284344*	.	.	.
19	29814998	G	A	ncRNA_intronic	*LOC284395*	.	.	.

19	29974466	A	ACCCCCCCC	ncRNA_intronic	*LOC284395*	.	.	.
19	29907629	A	G	ncRNA_intronic	*LOC284395*	.	.	.
19	29842329	T	A	ncRNA_intronic	*LOC284395*	.	.	.
19	37763936	T	TGCC	intergenic	*LOC284412,HKR1*	.	.	.
19	37764278	G	A	intergenic	*LOC284412,HKR1*	.	.	.
1	205879411	C	T	intergenic	*LOC284581,SLC26A9*	.	.	.
1	4710150	C	T	intergenic	*LOC284661,AJAP1*	.	.	.
1	4636879	T	C	intergenic	*LOC284661,AJAP1*	.	.	.
1	4637830	T	C	intergenic	*LOC284661,AJAP1*	.	.	.
20	22513654	GAC	G	intergenic	*LOC284788,LINC00261*	.	.	.
20	25149532	G	A	intergenic	*LOC284798,LOC101926889*	.	.	.
20	25161843	C	T	intergenic	*LOC284798,LOC101926889*	.	.	.
22	48042925	C	T	ncRNA_intronic	*LOC284930*	.	.	.
22	48091941	C	T	ncRNA_intronic	*LOC284930*	.	.	.
22	48486040	G	C	intergenic	*LOC284930,MIR3201*	.	.	.
22	48644756	T	C	intergenic	*LOC284930,MIR3201*	.	.	.
22	48379261	G	T	intergenic	*LOC284930,MIR3201*	.	.	.
22	48432067	G	T	intergenic	*LOC284930,MIR3201*	.	.	.
22	48472913	G	T	intergenic	*LOC284930,MIR3201*	.	.	.
2	30646954	T	G	intergenic	*LOC285043,LCLAT1*	.	.	.
2	30646955	T	G	intergenic	*LOC285043,LCLAT1*	.	.	.
2	87352846	T	TCTC	intergenic	*LOC285074,MIR4771-2*	.	.	.

5	158956633	G	C	intergenic	*LOC285627,LOC101927766*	.	.	.
5	158951594	C	T	intergenic	*LOC285627,LOC101927766*	.	.	.
5	159077472	T	C	intergenic	*LOC285627,LOC101927766*	.	.	.
5	159077474	G	A	intergenic	*LOC285627,LOC101927766*	.	.	.
5	159976425	A	G	intergenic	*LOC285628,ATP10B*	.	.	.
5	160394352	T	C	intergenic	*LOC285629,GABRB2*	.	.	.
5	160369092	G	A	intergenic	*LOC285629,GABRB2*	.	.	.
5	160371460	C	T	intergenic	*LOC285629,GABRB2*	.	.	.
5	160391307	T	G	intergenic	*LOC285629,GABRB2*	.	.	.
5	160487961	C	T	intergenic	*LOC285629,GABRB2*	.	.	.
5	160597863	A	G	intergenic	*LOC285629,GABRB2*	.	.	.
5	160672148	CA	C	intergenic	*LOC285629,GABRB2*	.	.	.
5	9674441	G	T	ncRNA_intronic	*LOC285692*	.	.	.
5	9643863	A	C	ncRNA_intronic	*LOC285692*	.	.	.
5	9746927	A	G	ncRNA_intronic	*LOC285692*	.	.	.
5	9754502	C	G	ncRNA_intronic	*LOC285692*	.	.	.
5	9755605	G	A	ncRNA_intronic	*LOC285692*	.	.	.
5	9919327	T	A	intergenic	*LOC285692,FAM173B*	.	.	.
5	9936048	G	A	intergenic	*LOC285692,FAM173B*	.	.	.
5	9936050	A	C	intergenic	*LOC285692,FAM173B*	.	.	.
5	9936600	A	G	intergenic	*LOC285692,FAM173B*	.	.	.

5	9960273	C	G	intergenic	*LOC285692,FAM173B*	.	.	.
5	9960275	A	G	intergenic	*LOC285692,FAM173B*	.	.	.
5	9992670	A	G	intergenic	*LOC285692,FAM173B*	.	.	.
5	9993144	C	T	intergenic	*LOC285692,FAM173B*	.	.	.
5	10007634	T	TA	intergenic	*LOC285692,FAM173B*	.	.	.
5	10015162	T	C	intergenic	*LOC285692,FAM173B*	.	.	.
5	10181713	G	A	intergenic	*LOC285692,FAM173B*	.	.	.
5	10181714	C	T	intergenic	*LOC285692,FAM173B*	.	.	.
5	17130733	G	A	ncRNA_exonic	*LOC285696*	.	.	.
6	119788950	A	G	ncRNA_intronic	*LOC285762*	.	.	.
6	120202712	T	C	intergenic	*LOC285762,MIR3144*	.	.	.
6	120236948	C	G	intergenic	*LOC285762,MIR3144*	.	.	.
6	119926997	C	T	intergenic	*LOC285762,MIR3144*	.	.	.
6	120272870	C	T	intergenic	*LOC285762,MIR3144*	.	.	.
6	120043534	T	C	intergenic	*LOC285762,MIR3144*	.	.	.
6	120069494	G	A	intergenic	*LOC285762,MIR3144*	.	.	.
6	120330678	T	A	intergenic	*LOC285762,MIR3144*	.	.	.
8	1263330	T	A	intergenic	*LOC286083,DLGAP2*	.	.	.
8	1277011	G	T	intergenic	*LOC286083,DLGAP2*	.	.	.
8	1395938	C	T	intergenic	*LOC286083,DLGAP2*	.	.	.

8	1396089	G	A	intergenic	*LOC286083,DLGAP2*	.	.	.
8	1396464	G	A	intergenic	*LOC286083,DLGAP2*	.	.	.
8	20991894	T	C	intergenic	*LOC286114,LOC101929172*	.	.	.
8	20993277	T	A	intergenic	*LOC286114,LOC101929172*	.	.	.
9	67227816	T	C	intergenic	*LOC286297,AQP7P1*	.	.	.
12	131944889	C	G	intergenic	*LOC338797,SFSWAP*	.	.	.
12	132166812	C	G	intergenic	*LOC338797,SFSWAP*	.	.	.
12	131988784	A	G	intergenic	*LOC338797,SFSWAP*	.	.	.
12	132019681	AACAC	A	intergenic	*LOC338797,SFSWAP*	.	.	.
17	5720582	G	A	ncRNA_intronic	*LOC339166*	.	.	.
17	5926159	C	T	intergenic	*LOC339166,WSCD1*	.	.	.
17	20841578	C	T	upstream	*LOC339260*	.	.	.
17	20889513	A	G	intergenic	*LOC339260,USP22*	.	.	.
1	244100073	A	G	ncRNA_intronic	*LOC339529*	.	.	.
20	37882909	A	C	intergenic	*LOC339568,LINC01370*	.	.	.
20	37919173	G	T	intergenic	*LOC339568,LINC01370*	.	.	.
20	38199074	C	T	intergenic	*LOC339568,LINC01370*	.	.	.
20	38579435	G	C	intergenic	*LOC339568,LINC01370*	.	.	.
20	38164867	TAAAC	T	intergenic	*LOC339568,LINC01370*	.	.	.
20	38245275	TACAC	T	intergenic	*LOC339568,LINC01370*	.	.	.

20	38316911	A	C	intergenic	*LOC339568,LINC01370*	.	.	.
20	38328542	C	A	intergenic	*LOC339568,LINC01370*	.	.	.
20	38099724	G	A	intergenic	*LOC339568,LINC01370*	.	.	.
20	38115112	C	T	intergenic	*LOC339568,LINC01370*	.	.	.
20	38550265	C	A	intergenic	*LOC339568,LINC01370*	.	.	.
20	38550267	T	C	intergenic	*LOC339568,LINC01370*	.	.	.
20	38600592	T	G	intergenic	*LOC339568,LINC01370*	.	.	.
20	11248314	ATGTG	A	ncRNA_exonic	*LOC339593*	.	.	.
20	11251916	A	G	ncRNA_intronic	*LOC339593*	.	.	.
20	11656712	TTCTC	T	intergenic	*LOC339593,LINC00687*	.	.	.
20	11418716	TG	T	intergenic	*LOC339593,LINC00687*	.	.	.
20	11342202	T	G	intergenic	*LOC339593,LINC00687*	.	.	.
20	11342879	C	T	intergenic	*LOC339593,LINC00687*	.	.	.
20	11363984	C	T	intergenic	*LOC339593,LINC00687*	.	.	.
20	11363985	A	G	intergenic	*LOC339593,LINC00687*	.	.	.
20	11713860	T	G	intergenic	*LOC339593,LINC00687*	.	.	.
20	11744897	A	G	intergenic	*LOC339593,LINC00687*	.	.	.
20	11760815	G	A	intergenic	*LOC339593,LINC00687*	.	.	.
21	26370433	G	A	ncRNA_intronic	*LOC339622*	.	.	.
21	26371888	C	G	ncRNA_intronic	*LOC339622*	.	.	.
21	26234202	T	C	ncRNA_intronic	*LOC339622*	.	.	.
21	26244371	G	A	ncRNA_intronic	*LOC339622*	.	.	.

21	26288722	A	G	ncRNA_intronic	*LOC339622*	.	.	.
21	26288723	C	T	ncRNA_intronic	*LOC339622*	.	.	.
21	26464841	G	A	intergenic	*LOC339622,LINC00158*	.	.	.
21	26719667	T	A	intergenic	*LOC339622,LINC00158*	.	.	.
21	26720025	A	T	intergenic	*LOC339622,LINC00158*	.	.	.
3	18382630	T	C	intergenic	*LOC339862,SATB1*	.	.	.
3	18348650	T	C	intergenic	*LOC339862,SATB1*	.	.	.
3	18349186	T	C	intergenic	*LOC339862,SATB1*	.	.	.
3	18369478	C	T	intergenic	*LOC339862,SATB1*	.	.	.
3	18371946	T	G	intergenic	*LOC339862,SATB1*	.	.	.
4	188444479	C	T	intergenic	*LOC339975,LOC100506272*	.	.	.
4	158732630	C	T	intergenic	*LOC340017,FAM198B*	.	.	.
4	159004888	C	T	intergenic	*LOC340017,FAM198B*	.	.	.
4	158861924	C	CATATATATA	intergenic	*LOC340017,FAM198B*	.	.	.
4	158617142	A	C	intergenic	*LOC340017,FAM198B*	.	.	.
5	135103760	C	T	intergenic	*LOC340074,SLC25A48*	.	.	.
5	24840033	G	A	ncRNA_intronic	*LOC340107*	.	.	.
5	25387241	G	T	intergenic	*LOC340107,CDH9*	.	.	.
5	25958515	C	A	intergenic	*LOC340107,CDH9*	.	.	.
5	26652536	G	GA	intergenic	*LOC340107,CDH9*	.	.	.
5	24884025	C	G	intergenic	*LOC340107,CDH9*	.	.	.
5	25375338	A	C	intergenic	*LOC340107,CDH9*	.	.	.
5	25617151	C	T	intergenic	*LOC340107,CDH9*	.	.	.
5	25629343	T	A	intergenic	*LOC340107,CDH9*	.	.	.

5	25694207	C	A	intergenic	*LOC340107,CDH9*	.	.	.
5	25267590	T	C	intergenic	*LOC340107,CDH9*	.	.	.
5	25308218	C	A	intergenic	*LOC340107,CDH9*	.	.	.
5	25595239	A	G	intergenic	*LOC340107,CDH9*	.	.	.
5	25733301	C	A	intergenic	*LOC340107,CDH9*	.	.	.
5	26373830	C	A	intergenic	*LOC340107,CDH9*	.	.	.
5	25513321	A	T	intergenic	*LOC340107,CDH9*	.	.	.
5	24961147	T	TTATA	intergenic	*LOC340107,CDH9*	.	.	.
5	25451271	G	A	intergenic	*LOC340107,CDH9*	.	.	.
5	25856309	C	T	intergenic	*LOC340107,CDH9*	.	.	.
5	26088159	G	A	intergenic	*LOC340107,CDH9*	.	.	.
5	26420290	CA	C	intergenic	*LOC340107,CDH9*	.	.	.
5	26638487	G	A	intergenic	*LOC340107,CDH9*	.	.	.
5	26815720	G	T	intergenic	*LOC340107,CDH9*	.	.	.
5	26451103	T	A	intergenic	*LOC340107,CDH9*	.	.	.
5	24977501	A	G	intergenic	*LOC340107,CDH9*	.	.	.
5	25003658	C	T	intergenic	*LOC340107,CDH9*	.	.	.
5	25031933	C	A	intergenic	*LOC340107,CDH9*	.	.	.
5	25031935	A	G	intergenic	*LOC340107,CDH9*	.	.	.
5	25078572	A	G	intergenic	*LOC340107,CDH9*	.	.	.
5	25226355	G	A	intergenic	*LOC340107,CDH9*	.	.	.
5	25227752	G	A	intergenic	*LOC340107,CDH9*	.	.	.
5	25289624	C	A	intergenic	*LOC340107,CDH9*	.	.	.
5	25360267	T	C	intergenic	*LOC340107,CDH9*	.	.	.
5	25381579	C	T	intergenic	*LOC340107,CDH9*	.	.	.
5	25409174	T	C	intergenic	*LOC340107,CDH9*	.	.	.
5	25633203	T	C	intergenic	*LOC340107,CDH9*	.	.	.
5	25639162	C	G	intergenic	*LOC340107,CDH9*	.	.	.
5	26151974	C	A	intergenic	*LOC340107,CDH9*	.	.	.
5	26151975	A	G	intergenic	*LOC340107,CDH9*	.	.	.
5	26288626	T	C	intergenic	*LOC340107,CDH9*	.	.	.
5	26307151	A	T	intergenic	*LOC340107,CDH9*	.	.	.
5	26307307	T	C	intergenic	*LOC340107,CDH9*	.	.	.
5	26317316	C	T	intergenic	*LOC340107,CDH9*	.	.	.
5	26396909	A	G	intergenic	*LOC340107,CDH9*	.	.	.
5	26436904	A	C	intergenic	*LOC340107,CDH9*	.	.	.
5	26525950	T	A	intergenic	*LOC340107,CDH9*	.	.	.
5	33084605	G	A	intergenic	*LOC340113,TARS*	.	.	.

5	33098305	A	G	intergenic	*LOC340113,TARS*	.	.	.
5	33262503	C	T	intergenic	*LOC340113,TARS*	.	.	.
5	33317789	ATTAAACTACATAGAACAAGATGGTCAGCTC	A	intergenic	*LOC340113,TARS*	.	.	.
5	33349647	T	C	intergenic	*LOC340113,TARS*	.	.	.
9	109795864	C	G	ncRNA_intronic	*LOC340512,MIR548Q*	.	.	.
11	122892337	G	A	intergenic	*LOC341056,HSPA8*	.	.	.
11	122908916	T	G	intergenic	*LOC341056,HSPA8*	.	.	.
7	136746084	C	T	ncRNA_intronic	*LOC349160*	.	.	.
7	136791192	G	T	ncRNA_intronic	*LOC349160*	.	.	.
7	136818834	T	C	ncRNA_intronic	*LOC349160*	.	.	.
7	136821134	T	A	ncRNA_intronic	*LOC349160*	.	.	.
7	136871289	A	G	intergenic	*LOC349160,PTN*	.	.	.
7	136869803	A	G	intergenic	*LOC349160,PTN*	.	.	.
12	9786427	C	A	ncRNA_intronic	*LOC374443*	.	.	.
12	9786428	C	A	ncRNA_intronic	*LOC374443*	.	.	.
12	9782608	C	G	ncRNA_intronic	*LOC374443*	.	.	.
12	9783200	A	C	ncRNA_intronic	*LOC374443*	.	.	.
11	112406243	C	CTA	ncRNA_exonic	*LOC387810*	.	.	.
11	112630046	C	G	intergenic	*LOC387810,LOC101928847*	.	.	.
11	112608915	T	C	intergenic	*LOC387810,LOC101928847*	.	.	.
11	112552455	A	G	intergenic	*LOC387810,LOC101928847*	.	.	.
11	112554525	C	T	intergenic	*LOC387810,LOC101928847*	.	.	.
11	112699804	C	T	intergenic	*LOC387810,LOC101928847*	.	.	.
11	112711453	A	T	intergenic	*LOC387810,LOC101928847*	.	.	.
16	57891502	T	C	intergenic	*LOC388282,CNGB1*	.	.	.

1	149331102	GTA	G	intergenic	*LOC388692,FCGR 1C*	.	.	.
21	15979986	C	T	intronic	*LOC388813*	.	.	.
21	15996013	T	C	intronic	*LOC388813*	.	.	.
21	15996261	A	T	intronic	*LOC388813*	.	.	.
21	15998350	G	A	intronic	*LOC388813*	.	.	.
21	16307044	C	T	intergenic	*LOC388813,NRIP1*	.	.	.
21	16314672	G	T	intergenic	*LOC388813,NRIP1*	.	.	.
21	16306493	G	A	intergenic	*LOC388813,NRIP1*	.	.	.
21	16306494	C	T	intergenic	*LOC388813,NRIP1*	.	.	.
21	16156179	C	CATA	intergenic	*LOC388813,NRIP1*	.	.	.
21	16076219	C	T	intergenic	*LOC388813,NRIP1*	.	.	.
21	16154653	G	C	intergenic	*LOC388813,NRIP1*	.	.	.
21	16176503	T	C	intergenic	*LOC388813,NRIP1*	.	.	.
21	16220692	T	A	intergenic	*LOC388813,NRIP1*	.	.	.
21	16220693	T	A	intergenic	*LOC388813,NRIP1*	.	.	.
21	16307159	T	G	intergenic	*LOC388813,NRIP1*	.	.	.
21	16307572	G	A	intergenic	*LOC388813,NRIP1*	.	.	.
21	16309938	C	T	intergenic	*LOC388813,NRIP1*	.	.	.
22	23889193	A	G	intergenic	*LOC388882,IGLL1*	.	.	.
2	42126159	T	A	intergenic	*LOC388942,LOC10 1929723*	.	.	.
7	155863552	C	A	intergenic	*LOC389602,LOC28 5889*	.	.	.
7	155892862	C	T	intergenic	*LOC389602,LOC28 5889*	.	.	.
7	155843644	G	T	intergenic	*LOC389602,LOC28 5889*	.	.	.
7	156225373	G	A	intergenic	*LOC389602,LOC28 5889*	.	.	.
7	156186519	G	A	intergenic	*LOC389602,LOC28 5889*	.	.	.
9	14997179	T	C	ncRNA_exonic	*LOC389705*	.	.	.
9	15116846	A	C	intergenic	*LOC389705,TTC39 B*	.	.	.
9	88435388	G	GAAAAA	ncRNA_intronic	*LOC389765*	.	.	.
9	88477375	C	T	intergenic	*LOC389765,NAA35*	.	.	.

9	88477376	A	G	intergenic	*LOC389765,NAA35*	.	.	.
X	139241565	G	C	intergenic	*LOC389895,SOX3*	.	.	.
X	139541513	G	A	intergenic	*LOC389895,SOX3*	.	.	.
X	139567321	G	A	intergenic	*LOC389895,SOX3*	.	.	.
X	139436527	A	C	intergenic	*LOC389895,SOX3*	.	.	.
X	139234191	A	G	intergenic	*LOC389895,SOX3*	.	.	.
X	139318096	C	T	intergenic	*LOC389895,SOX3*	.	.	.
X	139320164	T	C	intergenic	*LOC389895,SOX3*	.	.	.
X	139351133	A	G	intergenic	*LOC389895,SOX3*	.	.	.
X	139352798	A	G	intergenic	*LOC389895,SOX3*	.	.	.
X	139353068	A	G	intergenic	*LOC389895,SOX3*	.	.	.
X	139367416	C	T	intergenic	*LOC389895,SOX3*	.	.	.
X	139367417	A	G	intergenic	*LOC389895,SOX3*	.	.	.
X	139372238	A	G	intergenic	*LOC389895,SOX3*	.	.	.
X	139372240	C	CAGTT	intergenic	*LOC389895,SOX3*	.	.	.
X	139415145	G	C	intergenic	*LOC389895,SOX3*	.	.	.
X	139416151	C	T	intergenic	*LOC389895,SOX3*	.	.	.
X	139416153	G	C	intergenic	*LOC389895,SOX3*	.	.	.
X	139417097	T	A	intergenic	*LOC389895,SOX3*	.	.	.
X	139418122	C	T	intergenic	*LOC389895,SOX3*	.	.	.
X	139418124	C	A	intergenic	*LOC389895,SOX3*	.	.	.
X	3737541	C	T	ncRNA_intronic	*LOC389906*	.	.	.
X	3976835	T	G	intergenic	*LOC389906,LOC101928201*	.	.	.
X	4203615	C	T	intergenic	*LOC389906,LOC101928201*	.	.	.
X	4403350	C	A	intergenic	*LOC389906,LOC101928201*	.	.	.
X	4006929	T	A	intergenic	*LOC389906,LOC101928201*	.	.	.
X	4145156	TATATATATATGTATAC	T	intergenic	*LOC389906,LOC101928201*	.	.	.
X	4242112	C	A	intergenic	*LOC389906,LOC101928201*	.	.	.
X	4256849	C	T	intergenic	*LOC389906,LOC101928201*	.	.	.

X	4166841	A	AGATCGG	intergenic	*LOC389906,LOC101928201*	.	.	.
X	3972312	T	G	intergenic	*LOC389906,LOC101928201*	.	.	.
X	3972313	A	G	intergenic	*LOC389906,LOC101928201*	.	.	.
X	3972731	G	T	intergenic	*LOC389906,LOC101928201*	.	.	.
X	4282995	G	T	intergenic	*LOC389906,LOC101928201*	.	.	.
X	4397533	G	A	intergenic	*LOC389906,LOC101928201*	.	.	.
16	33540867	T	C	intergenic	*LOC390705,ENPP7P13*	.	.	.
16	33416188	C	A	intergenic	*LOC390705,ENPP7P13*	.	.	.
16	33416189	T	G	intergenic	*LOC390705,ENPP7P13*	.	.	.
16	33438606	C	CAAAAAA	intergenic	*LOC390705,ENPP7P13*	.	.	.
16	33421815	GGTGT	G	intergenic	*LOC390705,ENPP7P13*	.	.	.
16	33326950	A	G	intergenic	*LOC390705,ENPP7P13*	.	.	.
16	33380439	T	C	intergenic	*LOC390705,ENPP7P13*	.	.	.
16	33396903	G	A	intergenic	*LOC390705,ENPP7P13*	.	.	.
16	33481889	C	A	intergenic	*LOC390705,ENPP7P13*	.	.	.
16	33491485	C	T	intergenic	*LOC390705,ENPP7P13*	.	.	.
16	33552786	T	C	intergenic	*LOC390705,ENPP7P13*	.	.	.
16	33552787	G	C	intergenic	*LOC390705,ENPP7P13*	.	.	.
16	33564911	T	C	intergenic	*LOC390705,ENPP7P13*	.	.	.

16	33565785	C	T	intergenic	*LOC390705,ENPP7P13*	.	.	.
16	33565786	A	G	intergenic	*LOC390705,ENPP7P13*	.	.	.
16	32459162	C	T	intergenic	*LOC390705,TP53TG3*	.	.	.
16	32396179	G	T	intergenic	*LOC390705,TP53TG3*	.	.	.
16	32352084	C	A	intergenic	*LOC390705,TP53TG3*	.	.	.
16	32368986	G	T	intergenic	*LOC390705,TP53TG3*	.	.	.
16	32379559	G	A	intergenic	*LOC390705,TP53TG3*	.	.	.
16	32382775	A	T	intergenic	*LOC390705,TP53TG3*	.	.	.
16	32387271	A	T	intergenic	*LOC390705,TP53TG3*	.	.	.
16	32393809	G	T	intergenic	*LOC390705,TP53TG3*	.	.	.
16	32500363	G	C	intergenic	*LOC390705,TP53TG3*	.	.	.
16	32519723	G	T	intergenic	*LOC390705,TP53TG3*	.	.	.
16	32562357	C	G	intergenic	*LOC390705,TP53TG3*	.	.	.
16	32393860	C	T	intergenic	*LOC390705,TP53TG3*	.	.	.
16	32551717	G	GCTATCTGA	intergenic	*LOC390705,TP53TG3*	.	.	.
16	32559591	G	T	intergenic	*LOC390705,TP53TG3*	.	.	.
16	32603764	T	A	intergenic	*LOC390705,TP53TG3*	.	.	.
16	32498097	A	G	intergenic	*LOC390705,TP53TG3*	.	.	.
16	32327781	T	C	intergenic	*LOC390705,TP53TG3*	.	.	.

16	32381782	G	A	intergenic	*LOC390705,TP53TG3*	.	.	.
16	32381981	T	A	intergenic	*LOC390705,TP53TG3*	.	.	.
16	32422506	A	G	intergenic	*LOC390705,TP53TG3*	.	.	.
16	32423804	T	C	intergenic	*LOC390705,TP53TG3*	.	.	.
16	32531324	T	C	intergenic	*LOC390705,TP53TG3*	.	.	.
16	32531326	T	A	intergenic	*LOC390705,TP53TG3*	.	.	.
16	32572888	A	C	intergenic	*LOC390705,TP53TG3*	.	.	.
16	32588970	C	T	intergenic	*LOC390705,TP53TG3*	.	.	.
8	73425427	A	T	intergenic	*LOC392232,KCNB2*	.	.	.
8	73186171	A	G	intergenic	*LOC392232,KCNB2*	.	.	.
8	73283203	C	A	intergenic	*LOC392232,KCNB2*	.	.	.
8	73173024	T	C	intergenic	*LOC392232,KCNB2*	.	.	.
8	73223217	C	T	intergenic	*LOC392232,KCNB2*	.	.	.
8	73223218	A	G	intergenic	*LOC392232,KCNB2*	.	.	.
8	73236286	G	C	intergenic	*LOC392232,KCNB2*	.	.	.
8	73237151	G	C	intergenic	*LOC392232,KCNB2*	.	.	.
8	73237837	A	T	intergenic	*LOC392232,KCNB2*	.	.	.
10	6441901	C	T	intergenic	*LOC399715,PRKCQ*	.	.	.
11	45445402	A	C	intergenic	*LOC399886,CHST1*	.	.	.

11	45496060	G	A	intergenic	*LOC399886,CHST1*	.	.	.
11	45445396	A	C	intergenic	*LOC399886,CHST1*	.	.	.
11	45437089	T	C	intergenic	*LOC399886,CHST1*	.	.	.
11	45652858	C	T	intergenic	*LOC399886,CHST1*	.	.	.
11	45653071	T	A	intergenic	*LOC399886,CHST1*	.	.	.
11	45653247	C	T	intergenic	*LOC399886,CHST1*	.	.	.
11	45653249	A	C	intergenic	*LOC399886,CHST1*	.	.	.
16	85227190	C	T	intergenic	*LOC400548,LINC00311*	.	.	.
16	88207722	A	T	intergenic	*LOC400553,LOC101928880*	.	.	.
16	88176941	TAAG	T	intergenic	*LOC400553,LOC101928880*	.	.	.
16	88184839	C	T	intergenic	*LOC400553,LOC101928880*	.	.	.
18	70833607	T	C	ncRNA_intronic	*LOC400655*	.	.	.
19	46598313	A	T	intergenic	*LOC400706,IGFL3*	.	.	.
1	165465568	C	A	ncRNA_intronic	*LOC400794*	.	.	.
1	165546528	G	C	ncRNA_intronic	*LOC400794*	.	.	.
1	165551445	C	T	upstream	*LOC400794*	.	.	.
1	165576914	TACAC	T	intergenic	*LOC400794,MGST3*	.	.	.
1	165587981	C	T	intergenic	*LOC400794,MGST3*	.	.	.
2	6352293	T	A	intergenic	*LOC400940,LINC01247*	.	.	.
2	6447880	C	A	intergenic	*LOC400940,LINC01247*	.	.	.
2	6157701	G	A	intergenic	*LOC400940,LINC01247*	.	.	.

2	6471820	C	T	intergenic	*LOC400940,LINC01247*	.	.	.
4	66030296	C	A	intergenic	*LOC401134,EPHA5*	.	.	.
4	65892003	T	C	intergenic	*LOC401134,EPHA5*	.	.	.
4	65918977	A	T	intergenic	*LOC401134,EPHA5*	.	.	.
4	66138917	A	G	intergenic	*LOC401134,EPHA5*	.	.	.
4	66158718	G	A	intergenic	*LOC401134,EPHA5*	.	.	.
5	17844307	G	GTATA	intergenic	*LOC401177,CDH18*	.	.	.
5	17708349	A	G	intergenic	*LOC401177,CDH18*	.	.	.
5	18176879	CT	C	intergenic	*LOC401177,CDH18*	.	.	.
5	18548210	G	C	intergenic	*LOC401177,CDH18*	.	.	.
5	18615039	C	A	intergenic	*LOC401177,CDH18*	.	.	.
5	19148457	G	GTGTGTGTA	intergenic	*LOC401177,CDH18*	.	.	.
5	18184387	T	C	intergenic	*LOC401177,CDH18*	.	.	.
5	18875018	T	C	intergenic	*LOC401177,CDH18*	.	.	.
5	19358531	T	A	intergenic	*LOC401177,CDH18*	.	.	.
5	17460384	C	T	intergenic	*LOC401177,CDH18*	.	.	.
5	17460843	A	G	intergenic	*LOC401177,CDH18*	.	.	.

5	17967840	GGATATATAGATATATCTCTGAAGATATATAGATATATATATCTTCAGA	G	intergenic	*LOC401177,CDH18*	.	.	.
5	18030982	G	A	intergenic	*LOC401177,CDH18*	.	.	.
5	18121860	G	A	intergenic	*LOC401177,CDH18*	.	.	.
5	18283404	C	T	intergenic	*LOC401177,CDH18*	.	.	.
5	18476649	C	T	intergenic	*LOC401177,CDH18*	.	.	.
5	18872740	A	C	intergenic	*LOC401177,CDH18*	.	.	.
5	19017775	T	A	intergenic	*LOC401177,CDH18*	.	.	.
5	19052591	T	C	intergenic	*LOC401177,CDH18*	.	.	.
5	19078026	T	A	intergenic	*LOC401177,CDH18*	.	.	.
5	19150998	A	G	intergenic	*LOC401177,CDH18*	.	.	.
5	19253776	GAAGGAAGGAAGC	G	intergenic	*LOC401177,CDH18*	.	.	.
7	22703199	G	T	ncRNA_exonic	*LOC401312*	.	.	.
7	35360374	A	G	ncRNA_intronic	*LOC401324*	.	.	.
7	35538354	G	A	intergenic	*LOC401324,HERPUD2*	.	.	.
7	56901833	A	AACC	intergenic	*LOC401357,LOC100130849*	.	.	.
9	138218483	A	C	intergenic	*LOC401557,C9orf62*	.	.	.
X	45738840	C	CAGCAA	intergenic	*LOC401585,LINC01186*	.	.	.

X	45917808	A	T	intergenic	*LOC401585,LINC01186*	.	.	.
X	46168747	G	A	intergenic	*LOC401585,LINC01186*	.	.	.
X	45859135	C	A	intergenic	*LOC401585,LINC01186*	.	.	.
X	45914588	C	T	intergenic	*LOC401585,LINC01186*	.	.	.
X	45920112	C	T	intergenic	*LOC401585,LINC01186*	.	.	.
X	45936674	G	C	intergenic	*LOC401585,LINC01186*	.	.	.
X	45951717	C	T	intergenic	*LOC401585,LINC01186*	.	.	.
X	45962526	T	C	intergenic	*LOC401585,LINC01186*	.	.	.
X	45965603	A	G	intergenic	*LOC401585,LINC01186*	.	.	.
X	45965728	A	G	intergenic	*LOC401585,LINC01186*	.	.	.
X	45965790	C	T	intergenic	*LOC401585,LINC01186*	.	.	.
X	45966021	T	C	intergenic	*LOC401585,LINC01186*	.	.	.
X	45966533	T	C	intergenic	*LOC401585,LINC01186*	.	.	.
X	45968097	A	G	intergenic	*LOC401585,LINC01186*	.	.	.
X	45968099	C	T	intergenic	*LOC401585,LINC01186*	.	.	.
X	45968171	G	C	intergenic	*LOC401585,LINC01186*	.	.	.
9	66520858	CTGTGTGTG	C	ncRNA_intronic	*LOC403323*	.	.	.
11	49637220	A	T	ncRNA_intronic	*LOC440040*	.	.	.
11	49595322	T	A	ncRNA_intronic	*LOC440040*	.	.	.
11	49646683	A	T	ncRNA_intronic	*LOC440040*	.	.	.
11	49647518	A	G	ncRNA_intronic	*LOC440040*	.	.	.

11	49647608	A	G	ncRNA_intronic	*LOC440040*	.	.	.
11	49889460	C	G	intergenic	*LOC440040,OR4C13*	.	.	.
11	49889461	A	G	intergenic	*LOC440040,OR4C13*	.	.	.
11	49892161	A	G	intergenic	*LOC440040,OR4C13*	.	.	.
11	49932726	C	T	intergenic	*LOC440040,OR4C13*	.	.	.
11	49932727	C	G	intergenic	*LOC440040,OR4C13*	.	.	.
11	49935099	A	T	intergenic	*LOC440040,OR4C13*	.	.	.
11	49949568	C	T	intergenic	*LOC440040,OR4C13*	.	.	.
11	49968497	C	T	intergenic	*LOC440040,OR4C13*	.	.	.
11	49970123	G	A	intergenic	*LOC440040,OR4C13*	.	.	.
11	49970731	A	G	intergenic	*LOC440040,OR4C13*	.	.	.
12	127380686	C	A	intergenic	*LOC440117,LOC101927592*	.	.	.
15	95622835	G	T	intergenic	*LOC440311,LINC01197*	.	.	.
15	95404652	C	A	intergenic	*LOC440311,LINC01197*	.	.	.
15	95698831	G	A	intergenic	*LOC440311,LINC01197*	.	.	.
16	87226907	CATATATATATATATAT	C	intergenic	*LOC440390,LOC101928708*	.	.	.
17	49603574	A	T	intergenic	*LOC440446,CA10*	.	.	.
17	49445990	C	T	intergenic	*LOC440446,CA10*	.	.	.
17	49647578	T	C	intergenic	*LOC440446,CA10*	.	.	.
17	49656847	G	C	intergenic	*LOC440446,CA10*	.	.	.
17	49656945	T	C	intergenic	*LOC440446,CA10*	.	.	.
17	49660824	A	C	intergenic	*LOC440446,CA10*	.	.	.

17	49701228	G	C	intergenic	*LOC440446,CA10*	.	.	.
1	190695309	T	G	ncRNA_intronic	*LOC440704*	.	.	.
1	190736232	GAGTTTTTAAAA	G	ncRNA_intronic	*LOC440704*	.	.	.
1	191250914	T	A	intergenic	*LOC440704,RGS18*	.	.	.
1	191815977	C	T	intergenic	*LOC440704,RGS18*	.	.	.
1	190827589	C	G	intergenic	*LOC440704,RGS18*	.	.	.
1	191317078	C	A	intergenic	*LOC440704,RGS18*	.	.	.
1	191361341	A	T	intergenic	*LOC440704,RGS18*	.	.	.
1	191956588	CAAA	C	intergenic	*LOC440704,RGS18*	.	.	.
1	191366007	G	T	intergenic	*LOC440704,RGS18*	.	.	.
1	191070208	TTATA	T	intergenic	*LOC440704,RGS18*	.	.	.
1	192019747	AT	A	intergenic	*LOC440704,RGS18*	.	.	.
1	190943777	C	T	intergenic	*LOC440704,RGS18*	.	.	.
1	190964808	G	C	intergenic	*LOC440704,RGS18*	.	.	.
1	191077054	A	T	intergenic	*LOC440704,RGS18*	.	.	.
1	191216403	C	A	intergenic	*LOC440704,RGS18*	.	.	.
1	191297681	G	A	intergenic	*LOC440704,RGS18*	.	.	.
1	191244957	G	C	intergenic	*LOC440704,RGS18*	.	.	.
1	191839685	CAGAGAG	C	intergenic	*LOC440704,RGS18*	.	.	.
1	191020801	TA	T	intergenic	*LOC440704,RGS18*	.	.	.

1	190784469	G	A	intergenic	*LOC440704,RGS18*	.	.	.
1	190785006	T	C	intergenic	*LOC440704,RGS18*	.	.	.
1	190902282	T	C	intergenic	*LOC440704,RGS18*	.	.	.
1	190902652	C	G	intergenic	*LOC440704,RGS18*	.	.	.
1	190970452	A	G	intergenic	*LOC440704,RGS18*	.	.	.
1	191029540	A	G	intergenic	*LOC440704,RGS18*	.	.	.
1	191030449	C	T	intergenic	*LOC440704,RGS18*	.	.	.
1	191171579	T	C	intergenic	*LOC440704,RGS18*	.	.	.
1	191188672	CCACACACA	C	intergenic	*LOC440704,RGS18*	.	.	.
1	191236171	A	G	intergenic	*LOC440704,RGS18*	.	.	.
1	191344848	C	T	intergenic	*LOC440704,RGS18*	.	.	.
1	191412824	G	C	intergenic	*LOC440704,RGS18*	.	.	.
1	191417089	T	TC	intergenic	*LOC440704,RGS18*	.	.	.
1	191417091	G	T	intergenic	*LOC440704,RGS18*	.	.	.
1	191441868	A	T	intergenic	*LOC440704,RGS18*	.	.	.
1	191442359	G	A	intergenic	*LOC440704,RGS18*	.	.	.
1	191444243	T	C	intergenic	*LOC440704,RGS18*	.	.	.
1	191520773	T	C	intergenic	*LOC440704,RGS18*	.	.	.
1	191794688	A	C	intergenic	*LOC440704,RGS18*	.	.	.

1	191986873	T	G	intergenic	*LOC440704,RGS18*	.	.	.
1	191987142	T	A	intergenic	*LOC440704,RGS18*	.	.	.
1	191987534	C	A	intergenic	*LOC440704,RGS18*	.	.	.
1	191987536	G	A	intergenic	*LOC440704,RGS18*	.	.	.
1	192118895	A	T	intergenic	*LOC440704,RGS18*	.	.	.
2	132053129	G	A	ncRNA_intronic	*LOC440910*	.	.	.
3	147152102	C	A	ncRNA_intronic	*LOC440982*	.	.	.
3	147357167	G	A	intergenic	*LOC440982,AGTR1*	.	.	.
3	147539804	A	G	intergenic	*LOC440982,AGTR1*	.	.	.
3	147810407	GTATATA	G	intergenic	*LOC440982,AGTR1*	.	.	.
3	147424290	A	G	intergenic	*LOC440982,AGTR1*	.	.	.
3	147975875	G	T	intergenic	*LOC440982,AGTR1*	.	.	.
3	147975876	G	T	intergenic	*LOC440982,AGTR1*	.	.	.
3	147952159	A	C	intergenic	*LOC440982,AGTR1*	.	.	.
3	147884912	C	T	intergenic	*LOC440982,AGTR1*	.	.	.
3	147716821	GTCTC	G	intergenic	*LOC440982,AGTR1*	.	.	.
3	148282162	A	G	intergenic	*LOC440982,AGTR1*	.	.	.
4	76332371	C	T	intergenic	*LOC441025,RCHY1*	.	.	.
4	76388949	G	C	intergenic	*LOC441025,RCHY1*	.	.	.
4	76389096	C	A	intergenic	*LOC441025,RCHY1*	.	.	.

4	76389098	A	G	intergenic	*LOC441025,RCHY1*	.	.	.
7	26541319	A	G	intergenic	*LOC441204,KIAA0087*	.	.	.
11	50345855	G	A	intergenic	*LOC441601,LOC646813*	.	.	.
11	50347276	A	G	intergenic	*LOC441601,LOC646813*	.	.	.
11	50347747	G	T	intergenic	*LOC441601,LOC646813*	.	.	.
2	95615518	G	A	intergenic	*LOC442028,MAL*	.	.	.
2	95618559	C	G	intergenic	*LOC442028,MAL*	.	.	.
7	517150	G	A	intergenic	*LOC442497,PDGFA*	.	.	.
5	131682737	A	G	ncRNA_intronic	*LOC553103*	.	.	.
14	19455722	G	A	intergenic	*LOC642426,POTEG*	.	.	.
14	19455723	G	C	intergenic	*LOC642426,POTEG*	.	.	.
12	9465649	G	A	ncRNA_intronic	*LOC642846*	.	.	.
12	9453174	C	T	ncRNA_intronic	*LOC642846*	.	.	.
12	9453175	C	G	ncRNA_intronic	*LOC642846*	.	.	.
12	9505790	A	G	intergenic	*LOC642846,LOC101930452*	.	.	.
12	9507752	A	G	intergenic	*LOC642846,LOC101930452*	.	.	.
9	43452937	T	A	intergenic	*LOC642929,FAM74A7*	.	.	.
9	95664946	A	G	intergenic	*LOC642943,FGD3*	.	.	.
9	95665965	G	C	intergenic	*LOC642943,FGD3*	.	.	.
12	93603499	A	G	ncRNA_intronic	*LOC643339*	.	.	.
12	93704551	G	A	ncRNA_intronic	*LOC643339*	.	.	.
1	112702097	T	C	intergenic	*LOC643355,CTTNBP2NL*	.	.	.
1	112745637	T	C	intergenic	*LOC643355,CTTNBP2NL*	.	.	.
1	112758406	G	T	intergenic	*LOC643355,CTTNBP2NL*	.	.	.

1	112759670	T	C	intergenic	*LOC643355,CTTNBP2NL*	.	.	.
1	112784652	G	A	intergenic	*LOC643355,CTTNBP2NL*	.	.	.
1	112833056	C	A	intergenic	*LOC643355,CTTNBP2NL*	.	.	.
1	113860752	T	A	intergenic	*LOC643441,MAGI3*	.	.	.
1	113861430	T	A	intergenic	*LOC643441,MAGI3*	.	.	.
1	113889388	G	A	intergenic	*LOC643441,MAGI3*	.	.	.
18	65443773	G	T	ncRNA_intronic	*LOC643542*	.	.	.
18	65442412	A	G	ncRNA_intronic	*LOC643542*	.	.	.
18	65274041	A	G	ncRNA_intronic	*LOC643542*	.	.	.
18	65378733	T	A	ncRNA_intronic	*LOC643542*	.	.	.
18	65379353	C	G	ncRNA_intronic	*LOC643542*	.	.	.
18	65379354	A	G	ncRNA_intronic	*LOC643542*	.	.	.
18	66324040	G	A	intergenic	*LOC643542,TMX3*	.	.	.
18	66295989	C	T	intergenic	*LOC643542,TMX3*	.	.	.
18	65581314	C	A	intergenic	*LOC643542,TMX3*	.	.	.
18	65582645	C	T	intergenic	*LOC643542,TMX3*	.	.	.
18	65677315	C	T	intergenic	*LOC643542,TMX3*	.	.	.
18	65805868	A	G	intergenic	*LOC643542,TMX3*	.	.	.
18	65972505	G	T	intergenic	*LOC643542,TMX3*	.	.	.
18	65972927	A	G	intergenic	*LOC643542,TMX3*	.	.	.
18	65972928	T	G	intergenic	*LOC643542,TMX3*	.	.	.
18	66053974	G	A	intergenic	*LOC643542,TMX3*	.	.	.
18	66301245	G	C	intergenic	*LOC643542,TMX3*	.	.	.
18	66319661	A	AT	intergenic	*LOC643542,TMX3*	.	.	.
12	98109795	A	G	ncRNA_intronic	*LOC643711*	.	.	.
12	98135212	C	A	ncRNA_intronic	*LOC643711*	.	.	.
12	98121909	G	C	ncRNA_intronic	*LOC643711*	.	.	.
12	98329673	TCTCA	T	intergenic	*LOC643711,MIR4495*	.	.	.
12	98182813	C	T	intergenic	*LOC643711,MIR4495*	.	.	.

12	98203130	G	A	intergenic	*LOC643711,MIR4495*	.	.	.
12	98285496	AATAT	A	intergenic	*LOC643711,MIR4495*	.	.	.
11	104793792	A	C	intergenic	*LOC643733,CASP4*	.	.	.
4	56710764	T	C	intergenic	*LOC644145,EXOC1*	.	.	.
18	15315105	C	T	ncRNA_intronic	*LOC644669*	.	.	.
18	15391617	G	A	intergenic	*LOC644669,NONE*	.	.	.
18	15381536	A	T	intergenic	*LOC644669,NONE*	.	.	.
18	15396882	G	A	intergenic	*LOC644669,NONE*	.	.	.
18	15369903	T	C	intergenic	*LOC644669,NONE*	.	.	.
18	15369904	C	T	intergenic	*LOC644669,NONE*	.	.	.
14	41465126	TAC	T	ncRNA_intronic	*LOC644919*	.	.	.
14	41428231	C	G	ncRNA_intronic	*LOC644919*	.	.	.
14	41471849	G	GTGTATA	ncRNA_intronic	*LOC644919*	.	.	.
14	41576853	T	C	ncRNA_intronic	*LOC644919*	.	.	.
14	41574687	T	C	ncRNA_intronic	*LOC644919*	.	.	.
14	41717959	G	T	intergenic	*LOC644919,LRFN5*	.	.	.
14	41679739	T	C	intergenic	*LOC644919,LRFN5*	.	.	.
14	41760381	C	T	intergenic	*LOC644919,LRFN5*	.	.	.
14	41760478	C	G	intergenic	*LOC644919,LRFN5*	.	.	.
14	41794493	C	G	intergenic	*LOC644919,LRFN5*	.	.	.
14	41794494	A	G	intergenic	*LOC644919,LRFN5*	.	.	.
14	41795038	T	G	intergenic	*LOC644919,LRFN5*	.	.	.
14	41795268	A	G	intergenic	*LOC644919,LRFN5*	.	.	.
14	41805471	C	T	intergenic	*LOC644919,LRFN5*	.	.	.

14	41805800	C	G	intergenic	*LOC644919,LRFN5*	.	.	.
14	41841416	G	A	intergenic	*LOC644919,LRFN5*	.	.	.
14	41962797	A	G	intergenic	*LOC644919,LRFN5*	.	.	.
14	41995921	A	C	intergenic	*LOC644919,LRFN5*	.	.	.
14	41995922	G	A	intergenic	*LOC644919,LRFN5*	.	.	.
14	42035829	T	C	intergenic	*LOC644919,LRFN5*	.	.	.
14	42035831	G	A	intergenic	*LOC644919,LRFN5*	.	.	.
14	42052247	C	T	intergenic	*LOC644919,LRFN5*	.	.	.
1	148944761	A	T	ncRNA_intronic	*LOC645166,NBPF25P*	.	.	.
2	21917838	C	A	ncRNA_intronic	*LOC645949*	.	.	.
2	22089994	A	G	intergenic	*LOC645949,LOC102723362*	.	.	.
2	22205372	G	T	intergenic	*LOC645949,LOC102723362*	.	.	.
2	22031093	G	T	intergenic	*LOC645949,LOC102723362*	.	.	.
2	22481119	C	A	intergenic	*LOC645949,LOC102723362*	.	.	.
2	22322629	A	ATATATACA(	intergenic	*LOC645949,LOC102723362*	.	.	.
2	22014732	G	T	intergenic	*LOC645949,LOC102723362*	.	.	.
2	22173987	T	C	intergenic	*LOC645949,LOC102723362*	.	.	.
2	22371673	C	A	intergenic	*LOC645949,LOC102723362*	.	.	.
2	22378567	G	A	intergenic	*LOC645949,LOC102723362*	.	.	.

2	22097683	T	G	intergenic	*LOC645949,LOC102723362*	.	.	.
2	22154490	C	T	intergenic	*LOC645949,LOC102723362*	.	.	.
2	22528850	C	A	intergenic	*LOC645949,LOC102723362*	.	.	.
2	22648304	G	C	intergenic	*LOC645949,LOC102723362*	.	.	.
2	22648305	A	T	intergenic	*LOC645949,LOC102723362*	.	.	.
15	21957847	G	A	intergenic	*LOC646214,CXADRP2*	.	.	.
2	227292094	TTGTGTG	T	intergenic	*LOC646736,MIR5702*	.	.	.
2	227159522	A	T	intergenic	*LOC646736,MIR5702*	.	.	.
2	227187095	T	A	intergenic	*LOC646736,MIR5702*	.	.	.
2	227485591	A	G	intergenic	*LOC646736,MIR5702*	.	.	.
2	227117580	C	T	intergenic	*LOC646736,MIR5702*	.	.	.
2	227117582	C	T	intergenic	*LOC646736,MIR5702*	.	.	.
2	227117633	T	A	intergenic	*LOC646736,MIR5702*	.	.	.
2	227119436	G	A	intergenic	*LOC646736,MIR5702*	.	.	.
2	227322831	A	T	intergenic	*LOC646736,MIR5702*	.	.	.
2	227322832	T	C	intergenic	*LOC646736,MIR5702*	.	.	.
2	227454273	A	T	intergenic	*LOC646736,MIR5702*	.	.	.
2	227467317	AACACACACAC	A	intergenic	*LOC646736,MIR5702*	.	.	.
11	50369906	G	T	ncRNA_intronic	*LOC646813*	.	.	.

11	50596577	C	T	intergenic	*LOC646813,OR4A5*	.	.	.
11	50447838	A	G	intergenic	*LOC646813,OR4A5*	.	.	.
11	50709903	G	C	intergenic	*LOC646813,OR4A5*	.	.	.
11	50497760	A	G	intergenic	*LOC646813,OR4A5*	.	.	.
11	50738479	G	T	intergenic	*LOC646813,OR4A5*	.	.	.
11	50691910	A	G	intergenic	*LOC646813,OR4A5*	.	.	.
11	50419030	A	T	intergenic	*LOC646813,OR4A5*	.	.	.
11	50432960	A	G	intergenic	*LOC646813,OR4A5*	.	.	.
11	50458434	T	C	intergenic	*LOC646813,OR4A5*	.	.	.
11	50665332	C	T	intergenic	*LOC646813,OR4A5*	.	.	.
11	50665333	T	A	intergenic	*LOC646813,OR4A5*	.	.	.
11	50703190	T	G	intergenic	*LOC646813,OR4A5*	.	.	.
11	50708160	T	G	intergenic	*LOC646813,OR4A5*	.	.	.
11	50760634	G	C	intergenic	*LOC646813,OR4A5*	.	.	.
11	51224701	A	T	intergenic	*LOC646813,OR4A5*	.	.	.
11	51248310	T	C	intergenic	*LOC646813,OR4A5*	.	.	.
11	51276734	A	G	intergenic	*LOC646813,OR4A5*	.	.	.
11	51278785	T	C	intergenic	*LOC646813,OR4A5*	.	.	.
11	51285333	C	G	intergenic	*LOC646813,OR4A5*	.	.	.

11	51316243	G	A	intergenic	*LOC646813,OR4A5*	.	.	.
11	51319436	A	G	intergenic	*LOC646813,OR4A5*	.	.	.
11	51336063	T	C	intergenic	*LOC646813,OR4A5*	.	.	.
11	51375528	G	A	intergenic	*LOC646813,OR4A5*	.	.	.
11	51375927	G	A	intergenic	*LOC646813,OR4A5*	.	.	.
11	51377244	T	C	intergenic	*LOC646813,OR4A5*	.	.	.
11	51407795	C	T	intergenic	*LOC646813,OR4A5*	.	.	.
11	51407796	A	G	intergenic	*LOC646813,OR4A5*	.	.	.
11	51408703	T	TC	intergenic	*LOC646813,OR4A5*	.	.	.
11	51409735	G	A	intergenic	*LOC646813,OR4A5*	.	.	.
3	149811511	A	C	intergenic	*LOC646903,LINC01213*	.	.	.
5	43056564	G	A	ncRNA_intronic	*LOC648987*	.	.	.
5	43056693	C	T	ncRNA_intronic	*LOC648987*	.	.	.
5	43059606	C	T	ncRNA_intronic	*LOC648987*	.	.	.
5	43059608	A	G	ncRNA_intronic	*LOC648987*	.	.	.
7	56520455	T	A	intergenic	*LOC650226,LOC100240728*	.	.	.
7	56521184	A	G	intergenic	*LOC650226,LOC100240728*	.	.	.
4	8986816	C	G	intergenic	*LOC650293,USP17L10*	.	.	.
4	9004385	G	C	intergenic	*LOC650293,USP17L10*	.	.	.
4	9009264	A	T	intergenic	*LOC650293,USP17L10*	.	.	.
4	8980540	G	A	intergenic	*LOC650293,USP17L10*	.	.	.

4	9011431	T	C	intergenic	*LOC650293,USP17L10*	.	.	.
4	9060465	A	G	intergenic	*LOC650293,USP17L10*	.	.	.
4	9085121	A	G	intergenic	*LOC650293,USP17L10*	.	.	.
4	9087310	C	A	intergenic	*LOC650293,USP17L10*	.	.	.
4	9087311	A	G	intergenic	*LOC650293,USP17L10*	.	.	.
4	9099711	A	C	intergenic	*LOC650293,USP17L10*	.	.	.
4	9120360	CGAAAGAAAGAAA	C	intergenic	*LOC650293,USP17L10*	.	.	.
2	14783485	G	T	intronic	*LOC653602*	.	.	.
2	14994576	C	A	intergenic	*LOC653602,NBAS*	.	.	.
2	15174799	A	G	intergenic	*LOC653602,NBAS*	.	.	.
2	14890887	A	G	intergenic	*LOC653602,NBAS*	.	.	.
2	15201675	C	T	intergenic	*LOC653602,NBAS*	.	.	.
2	14829899	C	A	intergenic	*LOC653602,NBAS*	.	.	.
17	58188909	G	GT	intergenic	*LOC653653,CA4*	.	.	.
16	22656981	C	T	intergenic	*LOC653786,MIR548AA2*	.	.	.
2	91843033	G	T	ncRNA_intronic	*LOC654342*	.	.	.
2	91910327	A	ACACACACA	intergenic	*LOC654342,GGT8P*	.	.	.
4	74419051	G	A	intergenic	*LOC728040,RASSF6*	.	.	.
12	89418183	CATATAT	C	intergenic	*LOC728084,DUSP6*	.	.	.
12	89542143	G	C	intergenic	*LOC728084,DUSP6*	.	.	.
12	89548441	A	G	intergenic	*LOC728084,DUSP6*	.	.	.
3	81182971	A	T	intergenic	*LOC728290,GBE1*	.	.	.
3	81295873	A	G	intergenic	*LOC728290,GBE1*	.	.	.
3	81442425	C	T	intergenic	*LOC728290,GBE1*	.	.	.
3	81154276	G	T	intergenic	*LOC728290,GBE1*	.	.	.

3	81440720	C	A	intergenic	*LOC728290,GBE1*	.	.	.
3	81452506	C	T	intergenic	*LOC728290,GBE1*	.	.	.
2	243072550	C	G	ncRNA_intronic	*LOC728323*	.	.	.
5	177354748	C	A	intergenic	*LOC728554,PROP1*	.	.	.
5	1645924	C	T	intergenic	*LOC728613,MIR4277*	.	.	.
5	1646465	C	T	intergenic	*LOC728613,MIR4277*	.	.	.
9	66855566	T	A	intergenic	*LOC728673,LOC101928381*	.	.	.
9	66770362	C	T	intergenic	*LOC728673,LOC101928381*	.	.	.
9	66816343	C	A	intergenic	*LOC728673,LOC101928381*	.	.	.
9	66816344	A	C	intergenic	*LOC728673,LOC101928381*	.	.	.
9	66845794	A	AC	intergenic	*LOC728673,LOC101928381*	.	.	.
2	39725309	T	A	ncRNA_intronic	*LOC728730*	.	.	.
2	39816730	C	T	ncRNA_intronic	*LOC728730*	.	.	.
2	39883441	T	A	intergenic	*LOC728730,TMEM178A*	.	.	.
12	108424023	T	C	intergenic	*LOC728739,WSCD2*	.	.	.
12	108316856	A	G	intergenic	*LOC728739,WSCD2*	.	.	.
16	60620841	G	T	intergenic	*LOC729159,MIR4426*	.	.	.
16	60918534	GTATACCATATATATGATATATATGATATATATGGTATACGTATACCATA	G	intergenic	*LOC729159,MIR4426*	.	.	.
16	60400902	G	A	intergenic	*LOC729159,MIR4426*	.	.	.

16	60629120	A	C	intergenic	*LOC729159,MIR4426*	.	.	.
16	60742293	G	A	intergenic	*LOC729159,MIR4426*	.	.	.
16	60833852	A	C	intergenic	*LOC729159,MIR4426*	.	.	.
16	60834653	A	G	intergenic	*LOC729159,MIR4426*	.	.	.
16	60876998	T	C	intergenic	*LOC729159,MIR4426*	.	.	.
16	60973185	T	C	intergenic	*LOC729159,MIR4426*	.	.	.
4	119515708	G	GTTTTTTTT	ncRNA_intronic	*LOC729218*	.	.	.
5	8434576	G	A	ncRNA_intronic	*LOC729506*	.	.	.
8	12505703	A	T	ncRNA_intronic	*LOC729732*	.	.	.
8	12519512	G	A	ncRNA_intronic	*LOC729732*	.	.	.
1	247867	T	C	intergenic	*LOC729737,LOC100133331*	.	.	.
1	254775	A	G	intergenic	*LOC729737,LOC100133331*	.	.	.
1	254806	T	C	intergenic	*LOC729737,LOC100133331*	.	.	.
1	89795894	T	C	intergenic	*LOC729930,GBP6*	.	.	.
1	89796160	G	A	intergenic	*LOC729930,GBP6*	.	.	.
18	22219982	A	G	ncRNA_intronic	*LOC729950*	.	.	.
18	22323262	A	AATATATATA	intergenic	*LOC729950,ZNF521*	.	.	.
18	22616443	G	A	intergenic	*LOC729950,ZNF521*	.	.	.
18	22416409	C	T	intergenic	*LOC729950,ZNF521*	.	.	.
1	98715945	A	C	ncRNA_intronic	*LOC729987*	.	.	.
1	98785006	A	T	intergenic	*LOC729987,SNX7*	.	.	.
1	99012568	T	C	intergenic	*LOC729987,SNX7*	.	.	.
1	98750416	C	T	intergenic	*LOC729987,SNX7*	.	.	.
1	98750714	A	T	intergenic	*LOC729987,SNX7*	.	.	.
1	99079668	T	A	intergenic	*LOC729987,SNX7*	.	.	.
1	99085826	C	T	intergenic	*LOC729987,SNX7*	.	.	.

1	99086535	C	T	intergenic	*LOC729987,SNX7*	.	.	.
20	57217178	C	T	intergenic	*LOC79160,STX16*	.	.	.
3	128276590	C	T	intergenic	*LOC90246,LINC01565*	.	.	.
4	6693295	G	T	intergenic	*LOC93622,S100P*	.	.	.
2	100957255	A	G	intergenic	*LONRF2,CHST10*	.	.	.
18	44111150	A	T	intronic	*LOXHD1*	.	.	.
8	23171297	T	A	intronic	*LOXL2*	.	.	.
10	100074844	C	T	intergenic	*LOXL4,PYROXD2*	.	.	.
6	161075096	ATTATATATAATATATATT	A	intronic	*LPA*	.	.	.
6	161002597	G	A	intronic	*LPA*	.	.	.
6	160987047	T	G	intronic	*LPA*	.	.	.
6	160988642	A	C	intronic	*LPA*	.	.	.
6	160991371	T	C	intronic	*LPA*	.	.	.
6	160991372	G	A	intronic	*LPA*	.	.	.
6	161101875	C	T	intergenic	*LPA,PLG*	.	.	.
6	160938679	T	G	intergenic	*LPAL2,LPA*	.	.	.
6	160951043	A	G	intergenic	*LPAL2,LPA*	.	.	.
9	113719541	G	C	intronic	*LPAR1*	.	.	.
9	113744386	T	C	intronic	*LPAR1*	.	.	.
1	85311728	C	T	intronic	*LPAR3*	.	.	.
X	78128375	T	C	intergenic	*LPAR4,MIR4328*	.	.	.
X	78136881	G	T	intergenic	*LPAR4,MIR4328*	.	.	.
X	78059787	G	T	intergenic	*LPAR4,MIR4328*	.	.	.
X	78060788	A	T	intergenic	*LPAR4,MIR4328*	.	.	.
X	78111341	A	G	intergenic	*LPAR4,MIR4328*	.	.	.
X	78120383	G	T	intergenic	*LPAR4,MIR4328*	.	.	.
X	78132004	T	C	intergenic	*LPAR4,MIR4328*	.	.	.
X	78136953	T	C	intergenic	*LPAR4,MIR4328*	.	.	.
X	78137519	T	TC	intergenic	*LPAR4,MIR4328*	.	.	.
X	78137520	A	T	intergenic	*LPAR4,MIR4328*	.	.	.
X	78137974	A	G	intergenic	*LPAR4,MIR4328*	.	.	.
13	48990555	C	T	UTR5	*LPAR6*	.	.	.
13	48994575	C	G	intronic	*LPAR6,RB1*	.	.	.
13	48994576	A	G	intronic	*LPAR6,RB1*	.	.	.
16	55652668	T	C	intergenic	*LPCAT2,SLC6A2*	.	.	.

1	212054677	T	C	intergenic	*LPGAT1,INTS7*	.	.	.
2	11872169	T	A	intronic	*LPIN1*	.	.	.
2	11877499	C	A	intronic	*LPIN1*	.	.	.
18	3014439	C	G	intergenic	*LPIN2,MYOM1*	.	.	.
8	19884568	C	CA	intergenic	*LPL,SLC18A1*	.	.	.
8	19895665	A	G	intergenic	*LPL,SLC18A1*	.	.	.
8	19896716	A	G	intergenic	*LPL,SLC18A1*	.	.	.
3	188223016	C	T	intronic	*LPP*	.	.	.
3	188158814	ATGTG	A	intronic	*LPP*	.	.	.
3	188303515	G	GC	intronic	*LPP*	.	.	.
3	188303516	G	A	intronic	*LPP*	.	.	.
3	188581594	A	G	intronic	*LPP*	.	.	.
4	155694678	G	GCTCT	intergenic	*LRAT,RBM46*	.	.	.
4	151423510	A	C	intronic	*LRBA*	.	.	.
4	151741552	C	T	intronic	*LRBA*	.	.	.
4	151196776	C	T	intronic	*LRBA*	.	.	.
4	151327473	T	TTTCATTAAA	intronic	*LRBA*	.	.	.
4	151428575	G	A	intronic	*LRBA*	.	.	.
4	151485014	T	C	intronic	*LRBA*	.	.	.
4	151609455	C	T	intronic	*LRBA*	.	.	.
13	47156151	G	C	intronic	*LRCH1*	.	.	.
13	47221832	C	G	intronic	*LRCH1*	.	.	.
X	114354424	C	T	intronic	*LRCH2*	.	.	.
X	114354425	A	G	intronic	*LRCH2*	.	.	.
X	114476936	A	C	intergenic	*LRCH2,LUZP4*	.	.	.
6	40432322	G	T	intronic	*LRFN2*	.	.	.
6	40533667	C	T	intronic	*LRFN2*	.	.	.
6	40833127	T	C	intergenic	*LRFN2,LOC101929555*	.	.	.
6	40597919	TTGTGTG	T	intergenic	*LRFN2,LOC101929555*	.	.	.
14	42108527	G	A	intronic	*LRFN5*	.	.	.
14	42323788	G	T	intronic	*LRFN5*	.	.	.
14	42205137	T	C	intronic	*LRFN5*	.	.	.
14	42206839	G	A	intronic	*LRFN5*	.	.	.
14	42221329	A	G	intronic	*LRFN5*	.	.	.
14	42221677	A	G	intronic	*LRFN5*	.	.	.
14	42221841	C	G	intronic	*LRFN5*	.	.	.

14	42355244	T	A	intronic	*LRFN5*	.	.	.
14	43119190	CATATATATACGTGTATATATATACGTATACGTATATATATACGTATATATATACGTATACGT	C	intergenic	*LRFN5,FSCB*	.	.	.
14	43162073	C	G	intergenic	*LRFN5,FSCB*	.	.	.
14	43515983	G	T	intergenic	*LRFN5,FSCB*	.	.	.
14	43319864	G	T	intergenic	*LRFN5,FSCB*	.	.	.
14	42905735	A	T	intergenic	*LRFN5,FSCB*	.	.	.
14	43040694	A	T	intergenic	*LRFN5,FSCB*	.	.	.
14	43188397	T	C	intergenic	*LRFN5,FSCB*	.	.	.
14	43191596	G	T	intergenic	*LRFN5,FSCB*	.	.	.
14	43939521	C	T	intergenic	*LRFN5,FSCB*	.	.	.
14	43265787	T	C	intergenic	*LRFN5,FSCB*	.	.	.
14	43913767	A	G	intergenic	*LRFN5,FSCB*	.	.	.
14	43736210	A	G	intergenic	*LRFN5,FSCB*	.	.	.
14	43616030	A	T	intergenic	*LRFN5,FSCB*	.	.	.
14	43965008	C	A	intergenic	*LRFN5,FSCB*	.	.	.
14	43965009	T	C	intergenic	*LRFN5,FSCB*	.	.	.
14	42903545	G	C	intergenic	*LRFN5,FSCB*	.	.	.
14	42970096	A	T	intergenic	*LRFN5,FSCB*	.	.	.
14	43243306	G	A	intergenic	*LRFN5,FSCB*	.	.	.
14	43250289	G	GT	intergenic	*LRFN5,FSCB*	.	.	.
14	43292860	T	A	intergenic	*LRFN5,FSCB*	.	.	.
14	43385773	G	A	intergenic	*LRFN5,FSCB*	.	.	.
14	43425095	G	A	intergenic	*LRFN5,FSCB*	.	.	.
14	43440125	C	G	intergenic	*LRFN5,FSCB*	.	.	.
14	43663709	C	A	intergenic	*LRFN5,FSCB*	.	.	.
14	43663711	A	T	intergenic	*LRFN5,FSCB*	.	.	.
14	43700306	T	C	intergenic	*LRFN5,FSCB*	.	.	.
14	43724945	C	G	intergenic	*LRFN5,FSCB*	.	.	.
14	43724947	C	G	intergenic	*LRFN5,FSCB*	.	.	.
14	43814160	A	G	intergenic	*LRFN5,FSCB*	.	.	.
14	43895450	T	G	intergenic	*LRFN5,FSCB*	.	.	.

14	43895847	A	G	intergenic	*LRFN5,FSCB*	.	.	.
14	43927087	T	C	intergenic	*LRFN5,FSCB*	.	.	.
14	43967681	C	G	intergenic	*LRFN5,FSCB*	.	.	.
14	43969149	T	A	intergenic	*LRFN5,FSCB*	.	.	.
14	44170870	T	A	intergenic	*LRFN5,FSCB*	.	.	.
14	44185123	A	AAGGGGAT(	intergenic	*LRFN5,FSCB*	.	.	.
14	44185125	C	G	intergenic	*LRFN5,FSCB*	.	.	.
14	44297627	A	C	intergenic	*LRFN5,FSCB*	.	.	.
14	44374072	A	G	intergenic	*LRFN5,FSCB*	.	.	.
14	44374419	T	C	intergenic	*LRFN5,FSCB*	.	.	.
14	42585308	C	A	intergenic	*LRFN5,NONE*	.	.	.
14	42420205	C	T	intergenic	*LRFN5,NONE*	.	.	.
14	42555150	A	G	intergenic	*LRFN5,NONE*	.	.	.
14	42376103	A	T	intergenic	*LRFN5,NONE*	.	.	.
14	42376795	T	G	intergenic	*LRFN5,NONE*	.	.	.
14	42434408	T	G	intergenic	*LRFN5,NONE*	.	.	.
14	42497420	G	A	intergenic	*LRFN5,NONE*	.	.	.
14	42620756	A	T	intergenic	*LRFN5,NONE*	.	.	.
14	42621987	G	A	intergenic	*LRFN5,NONE*	.	.	.
14	42622928	C	A	intergenic	*LRFN5,NONE*	.	.	.
14	42638392	G	A	intergenic	*LRFN5,NONE*	.	.	.
14	42674509	T	G	intergenic	*LRFN5,NONE*	.	.	.
14	42752229	A	G	intergenic	*LRFN5,NONE*	.	.	.
14	42752230	T	C	intergenic	*LRFN5,NONE*	.	.	.
14	42831804	AGTGTGTG TGTGT	A	intergenic	*LRFN5,NONE*	.	.	.
7	133928696	T	A	intronic	*LRGUK*	.	.	.
7	133929005	T	G	intronic	*LRGUK*	.	.	.
7	133929136	T	G	intronic	*LRGUK*	.	.	.
1	111646944	A	AT	intergenic	*LRIF1,DRAM2*	.	.	.
1	111619786	C	T	intergenic	*LRIF1,DRAM2*	.	.	.
1	111619787	C	G	intergenic	*LRIF1,DRAM2*	.	.	.
3	66739925	C	T	intergenic	*LRIG1,KBTBD8*	.	.	.
3	66956986	A	G	intergenic	*LRIG1,KBTBD8*	.	.	.
3	66815219	C	T	intergenic	*LRIG1,KBTBD8*	.	.	.
3	66815516	A	G	intergenic	*LRIG1,KBTBD8*	.	.	.
3	66897804	A	C	intergenic	*LRIG1,KBTBD8*	.	.	.
1	113620780	A	C	intronic	*LRIG2*	.	.	.

12	59946464	A	G	intergenic	*LRIG3,SLC16A7*	.	.	.
12	59503693	T	C	intergenic	*LRIG3,SLC16A7*	.	.	.
12	59980246	T	G	intergenic	*LRIG3,SLC16A7*	.	.	.
12	59533420	C	T	intergenic	*LRIG3,SLC16A7*	.	.	.
12	59533421	A	G	intergenic	*LRIG3,SLC16A7*	.	.	.
12	59694802	G	T	intergenic	*LRIG3,SLC16A7*	.	.	.
12	59696221	T	G	intergenic	*LRIG3,SLC16A7*	.	.	.
12	59818661	TTATTTATTTATG	T	intergenic	*LRIG3,SLC16A7*	.	.	.
12	59873098	T	C	intergenic	*LRIG3,SLC16A7*	.	.	.
12	25246270	G	A	intronic	*LRMP*	.	.	.
8	105591462	A	T	intronic	*LRP12*	.	.	.
8	106294772	G	A	intergenic	*LRP12,ZFPM2*	.	.	.
8	105864181	A	G	intergenic	*LRP12,ZFPM2*	.	.	.
8	106304156	A	G	intergenic	*LRP12,ZFPM2*	.	.	.
8	105779232	AGTGTGTGTGTGT	A	intergenic	*LRP12,ZFPM2*	.	.	.
8	105839520	C	T	intergenic	*LRP12,ZFPM2*	.	.	.
8	106011756	A	AAAG	intergenic	*LRP12,ZFPM2*	.	.	.
8	106012271	T	C	intergenic	*LRP12,ZFPM2*	.	.	.
8	106050656	T	C	intergenic	*LRP12,ZFPM2*	.	.	.
8	106070147	A	G	intergenic	*LRP12,ZFPM2*	.	.	.
8	106213581	A	T	intergenic	*LRP12,ZFPM2*	.	.	.
8	106213583	C	A	intergenic	*LRP12,ZFPM2*	.	.	.
8	106270930	C	T	intergenic	*LRP12,ZFPM2*	.	.	.
8	106270932	T	C	intergenic	*LRP12,ZFPM2*	.	.	.
8	106271755	A	C	intergenic	*LRP12,ZFPM2*	.	.	.
2	142200822	C	A	intronic	*LRP1B*	.	.	.
2	141332787	A	G	intronic	*LRP1B*	.	.	.
2	142193811	T	C	intronic	*LRP1B*	.	.	.
2	142338564	C	G	intronic	*LRP1B*	.	.	.
2	141888976	TTATATA	T	intronic	*LRP1B*	.	.	.
2	142714974	T	G	intronic	*LRP1B*	.	.	.
2	141275567	T	A	intronic	*LRP1B*	.	.	.
2	142395161	C	G	intronic	*LRP1B*	.	.	.
2	141061101	A	C	intronic	*LRP1B*	.	.	.
2	141298738	G	A	intronic	*LRP1B*	.	.	.
2	141302583	C	T	intronic	*LRP1B*	.	.	.

2	141792142	G	A	intronic	*LRP1B*	.	.	.
2	142200617	C	A	intronic	*LRP1B*	.	.	.
2	142203324	A	G	intronic	*LRP1B*	.	.	.
2	142203909	C	T	intronic	*LRP1B*	.	.	.
2	142377540	A	T	intronic	*LRP1B*	.	.	.
2	142546438	T	C	intronic	*LRP1B*	.	.	.
2	142773134	C	T	intronic	*LRP1B*	.	.	.
2	143393919	C	A	intergenic	*LRP1B,KYNU*	.	.	.
2	142986623	C	T	intergenic	*LRP1B,KYNU*	.	.	.
2	143404537	G	T	intergenic	*LRP1B,KYNU*	.	.	.
2	143577751	A	T	intergenic	*LRP1B,KYNU*	.	.	.
2	143203738	C	A	intergenic	*LRP1B,KYNU*	.	.	.
2	143251428	T	C	intergenic	*LRP1B,KYNU*	.	.	.
2	143190346	G	A	intergenic	*LRP1B,KYNU*	.	.	.
2	143257429	A	T	intergenic	*LRP1B,KYNU*	.	.	.
2	143412206	A	C	intergenic	*LRP1B,KYNU*	.	.	.
2	143412255	T	G	intergenic	*LRP1B,KYNU*	.	.	.
2	143569084	G	T	intergenic	*LRP1B,KYNU*	.	.	.
2	143619267	C	G	intergenic	*LRP1B,KYNU*	.	.	.
2	170095599	T	C	intronic	*LRP2*	.	.	.
2	170199524	A	G	intronic	*LRP2*	.	.	.
2	170200416	T	C	intronic	*LRP2*	.	.	.
2	170224008	A	T	intergenic	*LRP2,BBS5*	.	.	.
2	170224346	A	C	intergenic	*LRP2,BBS5*	.	.	.
2	170225453	C	T	intergenic	*LRP2,BBS5*	.	.	.
2	170226150	A	G	intergenic	*LRP2,BBS5*	.	.	.
2	170260057	G	A	intergenic	*LRP2,BBS5*	.	.	.
2	170287431	A	C	intergenic	*LRP2,BBS5*	.	.	.
2	170287941	T	C	intergenic	*LRP2,BBS5*	.	.	.
2	170312702	G	A	intergenic	*LRP2,BBS5*	.	.	.
4	186300966	TTCTC	T	upstream	*LRP2BP*	.	.	.
11	46869680	G	A	ncRNA_intronic	*LRP4-AS1*	.	.	.
11	68096912	G	A	intronic	*LRP5*	.	.	.
11	68154679	C	T	intronic	*LRP5*	.	.	.
11	68155451	G	A	intronic	*LRP5*	.	.	.
22	25819194	T	G	intergenic	*LRP5L,CRYBB2P1*	.	.	.
2	44171113	C	A	intronic	*LRPPRC*	.	.	.

5	194261	ACTGTCCTGTGGTCTCACTGCCCCCCGCCGTCTGCTTTCTGAGCAGACATT	A	intronic	*LRRC14B*	.	.	.
6	25410323	G	C	intronic	*LRRC16A*	.	.	.
6	25445330	C	A	intronic	*LRRC16A*	.	.	.
6	25445331	C	T	intronic	*LRRC16A*	.	.	.
6	25570383	G	A	intronic	*LRRC16A*	.	.	.
15	99857276	C	T	intronic	*LRRC28*	.	.	.
15	99958415	A	G	intergenic	*LRRC28,MEF2A*	.	.	.
15	99958417	A	C	intergenic	*LRRC28,MEF2A*	.	.	.
15	99958695	T	C	intergenic	*LRRC28,MEF2A*	.	.	.
15	99960617	A	C	intergenic	*LRRC28,MEF2A*	.	.	.
15	99970081	G	A	intergenic	*LRRC28,MEF2A*	.	.	.
21	45901375	G	A	intergenic	*LRRC3,TSPEAR*	.	.	.
21	45907737	G	A	intergenic	*LRRC3,TSPEAR*	.	.	.
21	45907738	G	T	intergenic	*LRRC3,TSPEAR*	.	.	.
18	7327937	G	T	intergenic	*LRRC30,PTPRM*	.	.	.
18	7316113	C	T	intergenic	*LRRC30,PTPRM*	.	.	.
3	169560247	T	C	intronic	*LRRC31*	.	.	.
3	169604138	A	G	intergenic	*LRRC31,SAMD7*	.	.	.
17	62892919	C	A	exonic	*LRRC37A3*	nonsynonymous_SNV	.	.
17	43643507	A	G	intergenic	*LRRC37A4P,LOC644172*	.	.	.
9	114365671	G	A	ncRNA_intronic	*LRRC37A5P*	.	.	.
17	30374338	C	T	intronic	*LRRC37B*	.	.	.
17	30350075	G	A	intronic	*LRRC37B*	.	.	.
1	100649783	A	T	intergenic	*LRRC39,DBT*	.	.	.

3	26718123	T	C	intronic	*LRRC3B*	.	.	.
3	26718124	G	T	intronic	*LRRC3B*	.	.	.
3	26801350	A	G	intergenic	*LRRC3B,NEK10*	.	.	.
3	26876168	T	A	intergenic	*LRRC3B,NEK10*	.	.	.
3	26969717	G	A	intergenic	*LRRC3B,NEK10*	.	.	.
3	27033833	G	A	intergenic	*LRRC3B,NEK10*	.	.	.
1	54463460	G	T	intergenic	*LRRC42,LDLRAD1*	.	.	.
1	54463462	A	G	intergenic	*LRRC42,LDLRAD1*	.	.	.
1	3725096	T	A	intergenic	*LRRC47,CEP104*	.	.	.
15	71321860	G	A	intronic	*LRRC49*	.	.	.
11	40712386	G	T	intronic	*LRRC4C*	.	.	.
11	40728135	C	G	intronic	*LRRC4C*	.	.	.
11	40937099	G	C	intronic	*LRRC4C*	.	.	.
11	41034629	G	T	intronic	*LRRC4C*	.	.	.
11	41194909	T	A	intronic	*LRRC4C*	.	.	.
11	41399957	T	C	intronic	*LRRC4C*	.	.	.
11	40365407	G	T	intronic	*LRRC4C*	.	.	.
11	40890207	G	T	intronic	*LRRC4C*	.	.	.
11	40168247	A	G	intronic	*LRRC4C*	.	.	.
11	41155036	G	C	intronic	*LRRC4C*	.	.	.
11	40468335	T	C	intronic	*LRRC4C*	.	.	.
11	41046339	AT	A	intronic	*LRRC4C*	.	.	.
11	41211500	G	T	intronic	*LRRC4C*	.	.	.
11	40215580	A	T	intronic	*LRRC4C*	.	.	.
11	40473822	G	A	intronic	*LRRC4C*	.	.	.
11	40473823	T	C	intronic	*LRRC4C*	.	.	.
11	40495286	C	A	intronic	*LRRC4C*	.	.	.
11	40495287	C	T	intronic	*LRRC4C*	.	.	.
11	40539426	A	G	intronic	*LRRC4C*	.	.	.
11	40560085	A	T	intronic	*LRRC4C*	.	.	.
11	40730165	T	G	intronic	*LRRC4C*	.	.	.
11	40758580	G	T	intronic	*LRRC4C*	.	.	.
11	40869428	G	T	intronic	*LRRC4C*	.	.	.
11	40870213	G	C	intronic	*LRRC4C*	.	.	.
11	40936107	C	A	intronic	*LRRC4C*	.	.	.
11	40986924	G	T	intronic	*LRRC4C*	.	.	.
11	40987130	C	T	intronic	*LRRC4C*	.	.	.
11	41088938	T	C	intronic	*LRRC4C*	.	.	.

11	41089551	A	G	intronic	*LRRC4C*	.	.	.
11	41191774	A	T	intronic	*LRRC4C*	.	.	.
11	41193102	A	G	intronic	*LRRC4C*	.	.	.
11	41252313	C	A	intronic	*LRRC4C*	.	.	.
11	41650978	T	G	intergenic	*LRRC4C,LINC01499*	.	.	.
11	41501629	A	T	intergenic	*LRRC4C,LINC01499*	.	.	.
11	41536806	C	A	intergenic	*LRRC4C,LINC01499*	.	.	.
11	41568695	C	A	intergenic	*LRRC4C,LINC01499*	.	.	.
11	41569428	G	A	intergenic	*LRRC4C,LINC01499*	.	.	.
11	41570063	T	C	intergenic	*LRRC4C,LINC01499*	.	.	.
11	41570064	G	A	intergenic	*LRRC4C,LINC01499*	.	.	.
11	41571284	C	T	intergenic	*LRRC4C,LINC01499*	.	.	.
11	41670288	A	T	intergenic	*LRRC4C,LINC01499*	.	.	.
13	46790878	C	T	intronic	*LRRC63*	.	.	.
8	92204954	T	A	intronic	*LRRC69*	.	.	.
8	92206558	A	C	intronic	*LRRC69*	.	.	.
1	70274793	A	G	intronic	*LRRC7*	.	.	.
1	70321969	A	G	intronic	*LRRC7*	.	.	.
1	70322838	A	T	intronic	*LRRC7*	.	.	.
7	16613472	G	A	intronic	*LRRC72*	.	.	.
14	77320480	G	A	intronic	*LRRC74A*	.	.	.
1	90170309	G	A	intronic	*LRRC8C*	.	.	.
19	7956410	GTGTGTGTGTGTGTGTGTGTGTT	G	intronic	*LRRC8E*	.	.	.
14	60512167	C	G	ncRNA_intronic	*LRRC9*	.	.	.
14	60419776	C	T	ncRNA_intronic	*LRRC9*	.	.	.
14	60504765	G	C	ncRNA_intronic	*LRRC9*	.	.	.
14	60541799	T	C	intergenic	*LRRC9,PCNXL4*	.	.	.

14	60544811	G	A	intergenic	*LRRC9,PCNXL4*	.	.	.
12	85567440	AAGAG	A	intronic	*LRRIQ1*	.	.	.
12	85479915	T	C	intronic	*LRRIQ1*	.	.	.
12	85611434	A	G	intronic	*LRRIQ1*	.	.	.
12	85588365	A	G	intronic	*LRRIQ1*	.	.	.
12	85618334	C	A	intronic	*LRRIQ1*	.	.	.
12	85621350	G	A	intronic	*LRRIQ1*	.	.	.
12	85621904	A	T	intronic	*LRRIQ1*	.	.	.
12	85656439	T	C	intergenic	*LRRIQ1,ALX1*	.	.	.
1	74519457	A	T	intronic	*LRRIQ3*	.	.	.
1	74496025	T	C	intronic	*LRRIQ3*	.	.	.
1	74496026	G	A	intronic	*LRRIQ3*	.	.	.
1	74510509	G	T	intronic	*LRRIQ3*	.	.	.
1	74511912	A	C	intronic	*LRRIQ3*	.	.	.
1	74511913	C	A	intronic	*LRRIQ3*	.	.	.
1	74603441	T	G	intronic	*LRRIQ3*	.	.	.
15	101537256	CATATCTATAGATATGATTATTAATATAATCATATCTATAGATCTT	C	intronic	*LRRK1*	.	.	.
15	101493713	CTATATATATA	C	intronic	*LRRK1*	.	.	.
15	101548103	T	C	intronic	*LRRK1*	.	.	.
15	101548104	G	C	intronic	*LRRK1*	.	.	.
3	3868751	C	A	intronic	*LRRN1*	.	.	.
3	4018904	T	A	intergenic	*LRRN1,SETMAR*	.	.	.
3	4269849	T	A	intergenic	*LRRN1,SETMAR*	.	.	.
3	4137890	C	T	intergenic	*LRRN1,SETMAR*	.	.	.
3	3909155	G	A	intergenic	*LRRN1,SETMAR*	.	.	.
3	3931755	T	A	intergenic	*LRRN1,SETMAR*	.	.	.
3	3980711	A	G	intergenic	*LRRN1,SETMAR*	.	.	.
3	4016735	G	A	intergenic	*LRRN1,SETMAR*	.	.	.
3	4016737	A	G	intergenic	*LRRN1,SETMAR*	.	.	.
3	4115421	T	A	intergenic	*LRRN1,SETMAR*	.	.	.
3	4117511	T	G	intergenic	*LRRN1,SETMAR*	.	.	.
3	4118976	A	C	intergenic	*LRRN1,SETMAR*	.	.	.

3	4221578	C	G	intergenic	*LRRN1,SETMAR*	.	.	.
1	204650102	A	T	intronic	*LRRN2*	.	.	.
1	204715359	C	T	intergenic	*LRRN2,NFASC*	.	.	.
2	77371199	G	A	intronic	*LRRTM4*	.	.	.
2	77001918	T	C	intronic	*LRRTM4*	.	.	.
2	77003717	T	A	intronic	*LRRTM4*	.	.	.
2	77278981	C	T	intronic	*LRRTM4*	.	.	.
2	77656283	A	T	intronic	*LRRTM4*	.	.	.
2	77652367	G	A	intronic	*LRRTM4*	.	.	.
2	77652368	A	T	intronic	*LRRTM4*	.	.	.
2	77365686	G	T	intronic	*LRRTM4*	.	.	.
2	77595899	C	T	intronic	*LRRTM4*	.	.	.
2	77064612	G	A	intronic	*LRRTM4*	.	.	.
2	77143548	T	A	intronic	*LRRTM4*	.	.	.
2	77365478	A	C	intronic	*LRRTM4*	.	.	.
2	77392089	T	C	intronic	*LRRTM4*	.	.	.
2	77393959	C	G	intronic	*LRRTM4*	.	.	.
2	77410534	G	A	intronic	*LRRTM4*	.	.	.
2	77694014	G	A	intronic	*LRRTM4*	.	.	.
2	77785025	A	T	intergenic	*LRRTM4,LOC101927967*	.	.	.
2	77821436	G	A	intergenic	*LRRTM4,LOC101927967*	.	.	.
3	115522077	C	T	UTR3	*LSAMP*	.	.	.
3	115955818	TATAC	T	intronic	*LSAMP*	.	.	.
3	116004932	C	A	intronic	*LSAMP*	.	.	.
3	115724758	C	G	intronic	*LSAMP*	.	.	.
3	115883909	T	C	intronic	*LSAMP*	.	.	.
3	115781936	TTGTG	T	intronic	*LSAMP*	.	.	.
3	115809947	G	T	intronic	*LSAMP*	.	.	.
3	116330432	G	A	intergenic	*LSAMP,TUSC7*	.	.	.
5	157192245	ATTGACATTAAAATGTCT	A	intergenic	*LSM11,CLINT1*	.	.	.
3	14231134	T	C	intronic	*LSM3*	.	.	.
3	14231289	A	C	intronic	*LSM3*	.	.	.
3	14232049	A	T	intronic	*LSM3*	.	.	.
7	112126569	C	T	intronic	*LSMEM1*	.	.	.

7	112141451	C	T	intergenic	*LSMEM1,LOC100996249*	.	.	.
7	112183172	G	T	intergenic	*LSMEM1,LOC100996249*	.	.	.
7	112184784	A	G	intergenic	*LSMEM1,LOC100996249*	.	.	.
7	112252178	T	A	intergenic	*LSMEM1,LOC100996249*	.	.	.
5	28951979	T	A	intergenic	*LSP1P3,LOC101929645*	.	.	.
5	28984712	C	G	intergenic	*LSP1P3,LOC101929645*	.	.	.
5	28984713	A	G	intergenic	*LSP1P3,LOC101929645*	.	.	.
5	28997250	A	T	intergenic	*LSP1P3,LOC101929645*	.	.	.
5	29040246	C	CT	intergenic	*LSP1P3,LOC101929645*	.	.	.
5	29056571	C	T	intergenic	*LSP1P3,LOC101929645*	.	.	.
5	29057483	C	G	intergenic	*LSP1P3,LOC101929645*	.	.	.
21	47640017	A	G	intronic	*LSS*	.	.	.
2	33591458	G	A	intronic	*LTBP1*	.	.	.
2	33321882	G	A	intronic	*LTBP1*	.	.	.
2	33269723	A	T	intronic	*LTBP1*	.	.	.
2	33269880	G	T	intronic	*LTBP1*	.	.	.
2	33269881	A	G	intronic	*LTBP1*	.	.	.
2	33272891	T	A	intronic	*LTBP1*	.	.	.
2	33460026	A	C	intronic	*LTBP1*	.	.	.
2	33460270	T	A	intronic	*LTBP1*	.	.	.
2	33460271	GA	G	intronic	*LTBP1*	.	.	.
2	33460430	T	C	intronic	*LTBP1*	.	.	.
2	33619185	C	T	intronic	*LTBP1*	.	.	.
2	33619186	T	G	intronic	*LTBP1*	.	.	.
21	30316305	GTC	G	intronic	*LTN1*	.	.	.
21	30345705	T	A	intronic	*LTN1*	.	.	.
6	144174361	T	A	intronic	*LTV1*	.	.	.

6	144176238	T	A	intronic	*LTV1*	.	.	.
1	46707135	G	A	intergenic	*LURAP1,RAD54L*	.	.	.
9	12703875	A	G	ncRNA_intronic	*LURAP1L-AS1*	.	.	.
9	12704233	C	T	ncRNA_intronic	*LURAP1L-AS1*	.	.	.
9	12957645	G	A	intergenic	*LURAP1L,SNORD137*	.	.	.
11	25016802	G	C	intronic	*LUZP2*	.	.	.
11	24529414	T	C	intronic	*LUZP2*	.	.	.
11	24589800	A	G	intronic	*LUZP2*	.	.	.
11	24680037	A	T	intronic	*LUZP2*	.	.	.
11	24688543	C	T	intronic	*LUZP2*	.	.	.
11	24856625	T	C	intronic	*LUZP2*	.	.	.
11	24856724	C	T	intronic	*LUZP2*	.	.	.
11	24856725	C	G	intronic	*LUZP2*	.	.	.
11	24871101	C	CTT	intronic	*LUZP2*	.	.	.
11	24939626	G	T	intronic	*LUZP2*	.	.	.
11	24980474	A	G	intronic	*LUZP2*	.	.	.
11	24982596	A	T	intronic	*LUZP2*	.	.	.
11	25432289	C	G	intergenic	*LUZP2,ANO3*	.	.	.
11	25705521	A	C	intergenic	*LUZP2,ANO3*	.	.	.
11	25832276	A	G	intergenic	*LUZP2,ANO3*	.	.	.
11	25328057	C	A	intergenic	*LUZP2,ANO3*	.	.	.
11	25675613	T	C	intergenic	*LUZP2,ANO3*	.	.	.
11	25869085	C	A	intergenic	*LUZP2,ANO3*	.	.	.
11	25119324	T	TATA	intergenic	*LUZP2,ANO3*	.	.	.
11	25560387	C	G	intergenic	*LUZP2,ANO3*	.	.	.
11	25223966	T	TTTTG	intergenic	*LUZP2,ANO3*	.	.	.
11	25163744	A	G	intergenic	*LUZP2,ANO3*	.	.	.
11	25212801	G	A	intergenic	*LUZP2,ANO3*	.	.	.
11	25212920	C	T	intergenic	*LUZP2,ANO3*	.	.	.
11	25213829	T	C	intergenic	*LUZP2,ANO3*	.	.	.
11	25252884	C	T	intergenic	*LUZP2,ANO3*	.	.	.
11	25252885	A	G	intergenic	*LUZP2,ANO3*	.	.	.
11	25519015	C	T	intergenic	*LUZP2,ANO3*	.	.	.
11	25644412	T	A	intergenic	*LUZP2,ANO3*	.	.	.
11	25667201	T	G	intergenic	*LUZP2,ANO3*	.	.	.
11	25769046	A	C	intergenic	*LUZP2,ANO3*	.	.	.
11	25769048	A	G	intergenic	*LUZP2,ANO3*	.	.	.

11	25782044	G	A	intergenic	*LUZP2,ANO3*	.	.	.
11	25782665	T	C	intergenic	*LUZP2,ANO3*	.	.	.
11	25782667	T	C	intergenic	*LUZP2,ANO3*	.	.	.
11	25784840	G	A	intergenic	*LUZP2,ANO3*	.	.	.
11	25837039	T	C	intergenic	*LUZP2,ANO3*	.	.	.
11	25854514	G	T	intergenic	*LUZP2,ANO3*	.	.	.
11	25899382	C	T	intergenic	*LUZP2,ANO3*	.	.	.
11	25899593	T	C	intergenic	*LUZP2,ANO3*	.	.	.
11	25901214	C	A	intergenic	*LUZP2,ANO3*	.	.	.
11	26137243	T	C	intergenic	*LUZP2,ANO3*	.	.	.
11	26138937	AG	A	intergenic	*LUZP2,ANO3*	.	.	.
11	26140744	T	C	intergenic	*LUZP2,ANO3*	.	.	.
11	26193764	G	A	intergenic	*LUZP2,ANO3*	.	.	.
11	26194602	T	A	intergenic	*LUZP2,ANO3*	.	.	.
11	26194604	A	G	intergenic	*LUZP2,ANO3*	.	.	.
11	26194643	A	C	intergenic	*LUZP2,ANO3*	.	.	.
X	114665613	CTATA	C	intergenic	*LUZP4,PLS3-AS1*	.	.	.
4	44029653	C	T	intergenic	*LVCAT1,KCTD8*	.	.	.
4	44126751	A	G	intergenic	*LVCAT1,KCTD8*	.	.	.
4	44051873	T	C	intergenic	*LVCAT1,KCTD8*	.	.	.
4	44052654	C	T	intergenic	*LVCAT1,KCTD8*	.	.	.
4	44052655	A	G	intergenic	*LVCAT1,KCTD8*	.	.	.
4	44052743	T	C	intergenic	*LVCAT1,KCTD8*	.	.	.
4	44053970	T	G	intergenic	*LVCAT1,KCTD8*	.	.	.
7	119285092	T	G	ncRNA_intronic	*LVCAT5*	.	.	.
7	119436512	C	T	ncRNA_intronic	*LVCAT5*	.	.	.
7	119436767	C	T	ncRNA_intronic	*LVCAT5*	.	.	.
7	119500605	T	A	ncRNA_intronic	*LVCAT5*	.	.	.
7	119521895	A	G	ncRNA_intronic	*LVCAT5*	.	.	.
7	119694095	G	A	intergenic	*LVCAT5,KCND2*	.	.	.
7	119877590	C	A	intergenic	*LVCAT5,KCND2*	.	.	.
7	119756338	C	T	intergenic	*LVCAT5,KCND2*	.	.	.
7	119850622	A	G	intergenic	*LVCAT5,KCND2*	.	.	.
7	119859515	C	T	intergenic	*LVCAT5,KCND2*	.	.	.
4	185510945	G	A	ncRNA_intronic	*LVCAT8*	.	.	.
4	185526316	T	C	ncRNA_intronic	*LVCAT8*	.	.	.
8	143868311	G	C	upstream	*LY6D*	.	.	.
2	160745911	A	T	intronic	*LY75,LY75-CD302*	.	.	.

6	6639261	C	A	intronic	*LY86*	.	.	.
6	6442926	G	A	ncRNA_intronic	*LY86-AS1*	.	.	.
6	6583553	T	TAAC	ncRNA_intronic	*LY86-AS1*	.	.	.
6	6427614	G	A	ncRNA_intronic	*LY86-AS1*	.	.	.
6	6740815	T	C	intergenic	*LY86,RREB1*	.	.	.
6	6669972	T	G	intergenic	*LY86,RREB1*	.	.	.
6	6671488	G	A	intergenic	*LY86,RREB1*	.	.	.
6	6777773	T	A	intergenic	*LY86,RREB1*	.	.	.
6	6801631	T	C	intergenic	*LY86,RREB1*	.	.	.
8	75037109	T	C	intergenic	*LY96,JPH1*	.	.	.
8	74995663	T	C	intergenic	*LY96,JPH1*	.	.	.
8	75128175	T	A	intergenic	*LY96,JPH1*	.	.	.
8	75128176	G	A	intergenic	*LY96,JPH1*	.	.	.
8	75128378	T	TC	intergenic	*LY96,JPH1*	.	.	.
2	99863484	C	T	intronic	*LYG2*	.	.	.
2	99864879	C	G	intronic	*LYG2*	.	.	.
8	56915450	T	G	intronic	*LYN*	.	.	.
8	56943645	C	A	intergenic	*LYN,RPS20*	.	.	.
8	143862160	C	G	intergenic	*LYNX1,LY6D*	.	.	.
2	150261992	A	C	intronic	*LYPD6*	.	.	.
2	150333530	G	A	intergenic	*LYPD6,MMADHC*	.	.	.
2	150351739	C	G	intergenic	*LYPD6,MMADHC*	.	.	.
2	150353730	T	C	intergenic	*LYPD6,MMADHC*	.	.	.
2	150353731	G	A	intergenic	*LYPD6,MMADHC*	.	.	.
2	150358204	T	C	intergenic	*LYPD6,MMADHC*	.	.	.
2	150360607	A	C	intergenic	*LYPD6,MMADHC*	.	.	.
2	150380095	T	C	intergenic	*LYPD6,MMADHC*	.	.	.
2	150404687	T	A	intergenic	*LYPD6,MMADHC*	.	.	.
2	149911093	T	TGAAACAC	intronic	*LYPD6B*	.	.	.
2	149973716	A	G	intronic	*LYPD6B*	.	.	.
2	149923042	G	A	intronic	*LYPD6B*	.	.	.
2	150111335	G	A	intergenic	*LYPD6B,LYPD6*	.	.	.
2	150111336	C	A	intergenic	*LYPD6B,LYPD6*	.	.	.
1	249097954	C	T	intergenic	*LYPD8,SH3BP5L*	.	.	.
1	249060792	T	C	intergenic	*LYPD8,SH3BP5L*	.	.	.
1	249061031	C	A	intergenic	*LYPD8,SH3BP5L*	.	.	.
1	249061032	A	G	intergenic	*LYPD8,SH3BP5L*	.	.	.
1	219371081	T	G	intronic	*LYPLAL1*	.	.	.

1	219904850	C	T	intergenic	*LYPLAL1,RNU5F-1*	.	.	.
1	219478469	T	C	intergenic	*LYPLAL1,RNU5F-1*	.	.	.
1	219846150	A	G	intergenic	*LYPLAL1,RNU5F-1*	.	.	.
1	219848323	AACAC	A	intergenic	*LYPLAL1,RNU5F-1*	.	.	.
1	219884778	C	T	intergenic	*LYPLAL1,RNU5F-1*	.	.	.
1	219901519	C	G	intergenic	*LYPLAL1,RNU5F-1*	.	.	.
1	219902175	T	TC	intergenic	*LYPLAL1,RNU5F-1*	.	.	.
1	219921030	GCACACAC ACA	G	intergenic	*LYPLAL1,RNU5F-1*	.	.	.
1	219940336	A	G	intergenic	*LYPLAL1,RNU5F-1*	.	.	.
1	219948999	C	T	intergenic	*LYPLAL1,RNU5F-1*	.	.	.
1	220005441	G	A	intergenic	*LYPLAL1,RNU5F-1*	.	.	.
1	220014776	A	G	intergenic	*LYPLAL1,RNU5F-1*	.	.	.
1	220017076	A	T	intergenic	*LYPLAL1,RNU5F-1*	.	.	.
1	220018131	G	A	intergenic	*LYPLAL1,RNU5F-1*	.	.	.
1	220028645	C	T	intergenic	*LYPLAL1,RNU5F-1*	.	.	.
1	220028887	C	T	intergenic	*LYPLAL1,RNU5F-1*	.	.	.
1	220028888	A	G	intergenic	*LYPLAL1,RNU5F-1*	.	.	.
16	20925735	C	T	intronic	*LYRM1*	.	.	.
16	20925736	A	G	intronic	*LYRM1*	.	.	.
6	5254478	C	T	intronic	*LYRM4*	.	.	.
6	5097658	G	GCCACCAGT	ncRNA_intronic	*LYRM4-AS1*	.	.	.
5	130561958	T	C	intergenic	*LYRM7,CDC42SE2*	.	.	.
17	26291478	T	C	intergenic	*LYRM9,NLK*	.	.	.
17	26228721	T	G	intergenic	*LYRM9,NLK*	.	.	.
17	26228905	C	A	intergenic	*LYRM9,NLK*	.	.	.
1	151137195	A	T	intronic	*LYSMD1,TNFAIP8L2-SCNM1*	.	.	.
15	52023338	T	C	intronic	*LYSMD2*	.	.	.
1	236047588	A	G	upstream	*LYST*	.	.	.
10	29622203	T	TTA	intergenic	*LYZL1,SVIL-AS1*	.	.	.
10	30920061	T	G	intergenic	*LYZL2,SVILP1*	.	.	.
17	34301424	GTCTC	G	intergenic	*LYZL6,CCL16*	.	.	.
3	45897617	C	CA	intronic	*LZTFL1*	.	.	.
3	45897618	T	TCACGCTAC	intronic	*LZTFL1*	.	.	.
3	45897843	A	G	intronic	*LZTFL1*	.	.	.

8	20455217	A	AT	intergenic	*LZTS1-AS1,LOC102467222*	.	.	.
8	20709616	A	T	intergenic	*LZTS1-AS1,LOC102467222*	.	.	.
8	20750427	G	A	intergenic	*LZTS1-AS1,LOC102467222*	.	.	.
8	20304237	A	C	intergenic	*LZTS1-AS1,LOC102467222*	.	.	.
8	20678647	T	A	intergenic	*LZTS1-AS1,LOC102467222*	.	.	.
8	20722629	TATATATAAAA	T	intergenic	*LZTS1-AS1,LOC102467222*	.	.	.
8	20755774	C	G	intergenic	*LZTS1-AS1,LOC102467222*	.	.	.
8	20602742	A	G	intergenic	*LZTS1-AS1,LOC102467222*	.	.	.
8	20603029	A	G	intergenic	*LZTS1-AS1,LOC102467222*	.	.	.
2	74833728	CATAT	C	intronic	*M1AP*	.	.	.
1	116880181	C	T	intergenic	*MAB21L3,ATP1A1*	.	.	.
20	14808141	T	C	intronic	*MACROD2*	.	.	.
20	14926531	T	G	intronic	*MACROD2*	.	.	.
20	15664904	A	T	intronic	*MACROD2*	.	.	.
20	14422871	C	T	intronic	*MACROD2*	.	.	.
20	14422872	C	A	intronic	*MACROD2*	.	.	.
20	14724330	TAATACACGCGATAGTAGGTTG	T	intronic	*MACROD2*	.	.	.
20	15460623	A	G	intronic	*MACROD2*	.	.	.

20	14462797	G	A	intronic	*MACROD2*	.	.	.
20	14212683	G	A	intronic	*MACROD2*	.	.	.
20	14218234	G	A	intronic	*MACROD2*	.	.	.
20	14253997	T	C	intronic	*MACROD2*	.	.	.
20	14792237	G	A	intronic	*MACROD2*	.	.	.
20	15115360	A	G	intronic	*MACROD2*	.	.	.
20	15123989	A	C	intronic	*MACROD2*	.	.	.
20	15123990	G	T	intronic	*MACROD2*	.	.	.
20	15176083	C	T	intronic	*MACROD2*	.	.	.
20	15419044	C	T	intronic	*MACROD2*	.	.	.
20	15655399	TTGTGTG	T	intronic	*MACROD2*	.	.	.
20	14541786	C	T	ncRNA_intronic	*MACROD2-IT1*	.	.	.
20	16247595	A	G	intergenic	*MACROD2,KIF16B*	.	.	.
20	16174679	G	T	intergenic	*MACROD2,KIF16B*	.	.	.
20	16137926	G	C	intergenic	*MACROD2,KIF16B*	.	.	.
20	16098389	G	A	intergenic	*MACROD2,KIF16B*	.	.	.
7	2210703	G	A	intronic	*MAD1L1*	.	.	.
4	121137469	CAT	C	intergenic	*MAD2L1,PRDM5*	.	.	.
4	121397615	CATAT	C	intergenic	*MAD2L1,PRDM5*	.	.	.
4	121173656	T	A	intergenic	*MAD2L1,PRDM5*	.	.	.
4	121010510	A	C	intergenic	*MAD2L1,PRDM5*	.	.	.
4	121554195	G	A	intergenic	*MAD2L1,PRDM5*	.	.	.
4	121128910	G	A	intergenic	*MAD2L1,PRDM5*	.	.	.
4	121399660	G	A	intergenic	*MAD2L1,PRDM5*	.	.	.
4	121415775	C	T	intergenic	*MAD2L1,PRDM5*	.	.	.
4	121432955	A	C	intergenic	*MAD2L1,PRDM5*	.	.	.
4	121497370	T	A	intergenic	*MAD2L1,PRDM5*	.	.	.
16	79715463	T	A	intergenic	*MAF,MAFTRR*	.	.	.
20	39532833	A	C	intergenic	*MAFB,TOP1*	.	.	.
16	79794924	CACAG	C	ncRNA_intronic	*MAFTRR*	.	.	.
X	151113718	G	A	intergenic	*MAGEA4,GABRE*	.	.	.
X	148902708	C	G	intergenic	*MAGEA9B,LINC00850*	.	.	.
X	30265381	A	C	intronic	*MAGEB1*	.	.	.
X	30299726	G	C	intergenic	*MAGEB1,NR0B1*	.	.	.
X	35838906	T	C	intergenic	*MAGEB16,CFAP47*	.	.	.
X	16366968	T	C	intergenic	*MAGEB17,CTPS2*	.	.	.

X	16288834	T	A	intergenic	*MAGEB17,CTPS2*	.	.	.
X	16342421	G	C	intergenic	*MAGEB17,CTPS2*	.	.	.
X	16342857	A	G	intergenic	*MAGEB17,CTPS2*	.	.	.
X	16380910	G	T	intergenic	*MAGEB17,CTPS2*	.	.	.
X	26180325	A	T	intergenic	*MAGEB18,MAGEB6*	.	.	.
X	26201656	C	T	intergenic	*MAGEB18,MAGEB6*	.	.	.
X	26202188	T	G	intergenic	*MAGEB18,MAGEB6*	.	.	.
X	26202588	A	C	intergenic	*MAGEB18,MAGEB6*	.	.	.
X	26304286	C	A	intergenic	*MAGEB5,VENTXP1*	.	.	.
X	26353183	C	A	intergenic	*MAGEB5,VENTXP1*	.	.	.
X	26531733	G	C	intergenic	*MAGEB5,VENTXP1*	.	.	.
X	26340723	C	A	intergenic	*MAGEB5,VENTXP1*	.	.	.
X	26300111	A	G	intergenic	*MAGEB5,VENTXP1*	.	.	.
X	26300171	A	G	intergenic	*MAGEB5,VENTXP1*	.	.	.
X	26353623	C	T	intergenic	*MAGEB5,VENTXP1*	.	.	.
X	26354904	C	T	intergenic	*MAGEB5,VENTXP1*	.	.	.
X	26505586	A	G	intergenic	*MAGEB5,VENTXP1*	.	.	.
X	26505587	C	T	intergenic	*MAGEB5,VENTXP1*	.	.	.
X	26510332	C	T	intergenic	*MAGEB5,VENTXP1*	.	.	.
X	141083637	T	C	intergenic	*MAGEC1,MAGEC2*	.	.	.
X	141242375	C	G	intergenic	*MAGEC1,MAGEC2*	.	.	.
X	141251473	G	C	intergenic	*MAGEC1,MAGEC2*	.	.	.
X	141266970	A	G	intergenic	*MAGEC1,MAGEC2*	.	.	.

X	141348003	CCTCT	C	intergenic	*MAGEC2,SPANXN4*	.	.	.
X	141525560	G	T	intergenic	*MAGEC2,SPANXN4*	.	.	.
X	141530163	C	T	intergenic	*MAGEC2,SPANXN4*	.	.	.
X	141932459	CGTATATATACATAGACATATATGTATATATACATATATGTATATATACATATATGTCTATGTATATATACGTATAT	C	intergenic	*MAGEC2,SPANXN4*	.	.	.
X	141971951	A	G	intergenic	*MAGEC2,SPANXN4*	.	.	.
X	141555624	C	A	intergenic	*MAGEC2,SPANXN4*	.	.	.
X	141556814	AAATAATAAT	A	intergenic	*MAGEC2,SPANXN4*	.	.	.
X	141869153	T	G	intergenic	*MAGEC2,SPANXN4*	.	.	.
X	141427505	T	A	intergenic	*MAGEC2,SPANXN4*	.	.	.
X	141427506	T	TCGG	intergenic	*MAGEC2,SPANXN4*	.	.	.
X	141493593	C	T	intergenic	*MAGEC2,SPANXN4*	.	.	.
X	141494592	C	T	intergenic	*MAGEC2,SPANXN4*	.	.	.
X	141521865	C	T	intergenic	*MAGEC2,SPANXN4*	.	.	.
X	141554652	G	A	intergenic	*MAGEC2,SPANXN4*	.	.	.
X	141556932	T	C	intergenic	*MAGEC2,SPANXN4*	.	.	.

X	141577661	G	T	intergenic	*MAGEC2,SPANXN4*	.	.	.
X	141605394	C	G	intergenic	*MAGEC2,SPANXN4*	.	.	.
X	141605396	T	G	intergenic	*MAGEC2,SPANXN4*	.	.	.
X	141761172	A	G	intergenic	*MAGEC2,SPANXN4*	.	.	.
X	141761673	G	C	intergenic	*MAGEC2,SPANXN4*	.	.	.
X	141761709	T	G	intergenic	*MAGEC2,SPANXN4*	.	.	.
X	141771925	A	T	intergenic	*MAGEC2,SPANXN4*	.	.	.
X	141807548	T	A	intergenic	*MAGEC2,SPANXN4*	.	.	.
X	141853026	A	T	intergenic	*MAGEC2,SPANXN4*	.	.	.
X	141853074	C	T	intergenic	*MAGEC2,SPANXN4*	.	.	.
X	141878108	C	T	intergenic	*MAGEC2,SPANXN4*	.	.	.
X	141998737	T	C	intergenic	*MAGEC2,SPANXN4*	.	.	.
X	141999224	G	A	intergenic	*MAGEC2,SPANXN4*	.	.	.
X	142038884	T	C	intergenic	*MAGEC2,SPANXN4*	.	.	.
X	142063357	G	T	intergenic	*MAGEC2,SPANXN4*	.	.	.
X	142063359	A	G	intergenic	*MAGEC2,SPANXN4*	.	.	.
X	140962926	G	A	intronic	*MAGEC3*	.	.	.
X	140978451	C	A	intronic	*MAGEC3*	.	.	.
X	51615337	G	A	intronic	*MAGED1*	.	.	.
X	51720820	T	C	intergenic	*MAGED1,MAGED4*	.	.	.
X	51649039	AC	A	intergenic	*MAGED1,MAGED4*	.	.	.
X	51684812	T	C	intergenic	*MAGED1,MAGED4*	.	.	.

X	51684813	G	T	intergenic	*MAGED1,MAGED4*	.	.	.
X	51705227	G	T	intergenic	*MAGED1,MAGED4*	.	.	.
X	51767484	T	C	intergenic	*MAGED1,MAGED4*	.	.	.
X	51771470	C	T	intergenic	*MAGED1,MAGED4*	.	.	.
X	51771993	A	C	intergenic	*MAGED1,MAGED4*	.	.	.
X	54925870	C	A	intergenic	*MAGED2,TRO*	.	.	.
X	54855840	C	A	intergenic	*MAGED2,TRO*	.	.	.
X	54873141	T	C	intergenic	*MAGED2,TRO*	.	.	.
X	54880680	C	A	intergenic	*MAGED2,TRO*	.	.	.
X	54882762	T	C	intergenic	*MAGED2,TRO*	.	.	.
X	51837779	C	A	intergenic	*MAGED4,MAGED4*	.	.	.
X	51838049	C	A	intergenic	*MAGED4,MAGED4*	.	.	.
X	51844004	C	T	intergenic	*MAGED4,MAGED4*	.	.	.
X	51844616	C	G	intergenic	*MAGED4,MAGED4*	.	.	.
X	51857875	T	C	intergenic	*MAGED4,MAGED4*	.	.	.
X	51864148	C	T	intergenic	*MAGED4,MAGED4*	.	.	.
X	51865717	C	G	intergenic	*MAGED4,MAGED4*	.	.	.
X	51865719	T	C	intergenic	*MAGED4,MAGED4*	.	.	.
X	51867424	T	C	intergenic	*MAGED4,MAGED4*	.	.	.
X	51918563	G	A	intergenic	*MAGED4,MAGED4*	.	.	.
X	51963018	T	G	intergenic	*MAGED4,MIR8088*	.	.	.
X	51970060	T	C	intergenic	*MAGED4,MIR8088*	.	.	.
X	51987531	A	T	intergenic	*MAGED4,MIR8088*	.	.	.
X	51987532	C	T	intergenic	*MAGED4,MIR8088*	.	.	.
X	52037283	C	G	intergenic	*MAGED4,MIR8088*	.	.	.
X	52037285	T	C	intergenic	*MAGED4,MIR8088*	.	.	.
X	52040204	C	A	intergenic	*MAGED4,MIR8088*	.	.	.
X	52040206	T	A	intergenic	*MAGED4,MIR8088*	.	.	.
X	75667292	C	A	intergenic	*MAGEE1,MIR325HG*	.	.	.
X	75803329	GT	G	intergenic	*MAGEE1,MIR325HG*	.	.	.
X	75671769	C	A	intergenic	*MAGEE1,MIR325HG*	.	.	.
X	75765645	T	C	intergenic	*MAGEE1,MIR325HG*	.	.	.
X	75684525	G	A	intergenic	*MAGEE1,MIR325HG*	.	.	.

X	75685326	TTTCCTC	T	intergenic	*MAGEE1,MIR325HG*	.	.	.
X	75704719	C	T	intergenic	*MAGEE1,MIR325HG*	.	.	.
X	75705658	T	C	intergenic	*MAGEE1,MIR325HG*	.	.	.
X	75706914	G	C	intergenic	*MAGEE1,MIR325HG*	.	.	.
X	75716813	T	C	intergenic	*MAGEE1,MIR325HG*	.	.	.
X	75744926	A	G	intergenic	*MAGEE1,MIR325HG*	.	.	.
X	75746470	C	T	intergenic	*MAGEE1,MIR325HG*	.	.	.
X	75749979	G	A	intergenic	*MAGEE1,MIR325HG*	.	.	.
X	75751310	T	C	intergenic	*MAGEE1,MIR325HG*	.	.	.
X	75752264	C	T	intergenic	*MAGEE1,MIR325HG*	.	.	.
X	75756317	A	G	intergenic	*MAGEE1,MIR325HG*	.	.	.
X	75756969	G	A	intergenic	*MAGEE1,MIR325HG*	.	.	.
X	75756970	T	C	intergenic	*MAGEE1,MIR325HG*	.	.	.
X	75757172	C	T	intergenic	*MAGEE1,MIR325HG*	.	.	.
X	75757361	T	G	intergenic	*MAGEE1,MIR325HG*	.	.	.
X	75766292	G	C	intergenic	*MAGEE1,MIR325HG*	.	.	.
X	75781458	T	C	intergenic	*MAGEE1,MIR325HG*	.	.	.
X	75784946	C	T	intergenic	*MAGEE1,MIR325HG*	.	.	.
X	75796640	C	G	intergenic	*MAGEE1,MIR325HG*	.	.	.

X	75797912	C	T	intergenic	*MAGEE1,MIR325HG*	.	.	.
X	75801419	G	T	intergenic	*MAGEE1,MIR325HG*	.	.	.
X	75806241	A	G	intergenic	*MAGEE1,MIR325HG*	.	.	.
X	75807050	T	TC	intergenic	*MAGEE1,MIR325HG*	.	.	.
X	75821779	T	C	intergenic	*MAGEE1,MIR325HG*	.	.	.
X	75821969	T	G	intergenic	*MAGEE1,MIR325HG*	.	.	.
X	75854507	G	T	intergenic	*MAGEE1,MIR325HG*	.	.	.
X	75871213	G	A	intergenic	*MAGEE1,MIR325HG*	.	.	.
X	75872213	G	A	intergenic	*MAGEE1,MIR325HG*	.	.	.
X	75872215	G	T	intergenic	*MAGEE1,MIR325HG*	.	.	.
X	75388762	G	T	intergenic	*MAGEE2,PBDC1*	.	.	.
X	75203289	G	A	intergenic	*MAGEE2,PBDC1*	.	.	.
X	75352447	G	C	intergenic	*MAGEE2,PBDC1*	.	.	.
X	75375997	T	C	intergenic	*MAGEE2,PBDC1*	.	.	.
X	75129825	AATAT	A	intergenic	*MAGEE2,PBDC1*	.	.	.
X	75014232	T	C	intergenic	*MAGEE2,PBDC1*	.	.	.
X	75025066	G	A	intergenic	*MAGEE2,PBDC1*	.	.	.
X	75026071	C	T	intergenic	*MAGEE2,PBDC1*	.	.	.
X	75028370	G	A	intergenic	*MAGEE2,PBDC1*	.	.	.
X	75050636	C	T	intergenic	*MAGEE2,PBDC1*	.	.	.
X	75056770	G	A	intergenic	*MAGEE2,PBDC1*	.	.	.
X	75065779	A	T	intergenic	*MAGEE2,PBDC1*	.	.	.
X	75075053	A	G	intergenic	*MAGEE2,PBDC1*	.	.	.
X	75079858	T	G	intergenic	*MAGEE2,PBDC1*	.	.	.
X	75142401	C	T	intergenic	*MAGEE2,PBDC1*	.	.	.
X	75143002	T	C	intergenic	*MAGEE2,PBDC1*	.	.	.
X	75156828	G	T	intergenic	*MAGEE2,PBDC1*	.	.	.
X	75157065	G	A	intergenic	*MAGEE2,PBDC1*	.	.	.

X	75181358	C	A	intergenic	*MAGEE2,PBDC1*	.	.	.
X	75181966	C	T	intergenic	*MAGEE2,PBDC1*	.	.	.
X	75194815	T	C	intergenic	*MAGEE2,PBDC1*	.	.	.
X	75195136	T	C	intergenic	*MAGEE2,PBDC1*	.	.	.
X	75213152	T	G	intergenic	*MAGEE2,PBDC1*	.	.	.
X	75215330	G	A	intergenic	*MAGEE2,PBDC1*	.	.	.
X	75215332	G	A	intergenic	*MAGEE2,PBDC1*	.	.	.
X	75240525	T	A	intergenic	*MAGEE2,PBDC1*	.	.	.
X	75240903	T	C	intergenic	*MAGEE2,PBDC1*	.	.	.
X	75271834	C	T	intergenic	*MAGEE2,PBDC1*	.	.	.
X	75292774	G	A	intergenic	*MAGEE2,PBDC1*	.	.	.
X	75293320	G	A	intergenic	*MAGEE2,PBDC1*	.	.	.
X	75298068	C	A	intergenic	*MAGEE2,PBDC1*	.	.	.
X	75298069	C	T	intergenic	*MAGEE2,PBDC1*	.	.	.
X	75305059	T	G	intergenic	*MAGEE2,PBDC1*	.	.	.
X	75305061	A	T	intergenic	*MAGEE2,PBDC1*	.	.	.
X	75309753	T	C	intergenic	*MAGEE2,PBDC1*	.	.	.
X	75309754	G	T	intergenic	*MAGEE2,PBDC1*	.	.	.
X	75329357	T	G	intergenic	*MAGEE2,PBDC1*	.	.	.
X	75383446	G	C	intergenic	*MAGEE2,PBDC1*	.	.	.
3	65682586	C	T	intronic	*MAGI1*	.	.	.
3	65528605	T	A	intronic	*MAGI1*	.	.	.
7	77661111	G	T	intronic	*MAGI2*	.	.	.
7	78940264	GCACA	G	intronic	*MAGI2*	.	.	.
7	78338218	TC	T	intronic	*MAGI2*	.	.	.
7	77791222	A	G	intronic	*MAGI2*	.	.	.
7	77837795	A	T	intronic	*MAGI2*	.	.	.
7	77885787	C	A	exonic	*MAGI2*	nonsynonymous_SNV	.	.
7	78040086	C	A	intronic	*MAGI2*	.	.	.
7	78102236	C	T	intronic	*MAGI2*	.	.	.
7	78106626	G	T	intronic	*MAGI2*	.	.	.
7	78690162	G	C	intronic	*MAGI2*	.	.	.
7	78993886	A	T	intronic	*MAGI2*	.	.	.
7	78620633	TACAC	T	intronic	*MAGI2*	.	.	.
7	77899289	C	T	intronic	*MAGI2*	.	.	.
7	77900954	G	T	intronic	*MAGI2*	.	.	.
7	77919503	A	G	intronic	*MAGI2*	.	.	.

7	77920166	T	C	intronic	*MAGI2*	.	.	.
7	77921153	C	T	intronic	*MAGI2*	.	.	.
7	77951706	G	A	intronic	*MAGI2*	.	.	.
7	77951708	T	A	intronic	*MAGI2*	.	.	.
7	77971030	A	G	intronic	*MAGI2*	.	.	.
7	78033325	C	T	intronic	*MAGI2*	.	.	.
7	78033556	G	C	intronic	*MAGI2*	.	.	.
7	78181487	G	T	intronic	*MAGI2*	.	.	.
7	78277073	G	C	intronic	*MAGI2*	.	.	.
7	78518816	T	G	intronic	*MAGI2*	.	.	.
7	78519129	A	G	intronic	*MAGI2*	.	.	.
7	79074290	C	T	intronic	*MAGI2*	.	.	.
7	79196516	G	A	intergenic	*MAGI2-AS3,MIR548M*	.	.	.
7	79228224	T	C	intergenic	*MAGI2-AS3,MIR548M*	.	.	.
7	79156716	T	C	intergenic	*MAGI2-AS3,MIR548M*	.	.	.
1	113967566	A	G	intronic	*MAGI3*	.	.	.
1	114014917	C	T	intronic	*MAGI3*	.	.	.
1	114055857	G	C	intronic	*MAGI3*	.	.	.
1	114056329	C	A	intronic	*MAGI3*	.	.	.
1	114234342	A	G	intergenic	*MAGI3,PHTF1*	.	.	.
X	49021893	G	T	intronic	*MAGIX*	.	.	.
X	77099701	A	C	intronic	*MAGT1*	.	.	.
X	77100222	C	T	intronic	*MAGT1*	.	.	.
X	77100347	T	A	intronic	*MAGT1*	.	.	.
X	77118762	T	C	intronic	*MAGT1*	.	.	.
2	95703471	C	G	intronic	*MAL*	.	.	.
8	120249230	C	T	intronic	*MAL2*	.	.	.
8	120245725	C	T	intronic	*MAL2*	.	.	.
8	120245869	T	G	intronic	*MAL2*	.	.	.
8	120296566	C	A	intergenic	*MAL2,NOV*	.	.	.
8	120405747	C	T	intergenic	*MAL2,NOV*	.	.	.
8	120297133	A	C	intergenic	*MAL2,NOV*	.	.	.
8	120307690	C	T	intergenic	*MAL2,NOV*	.	.	.
10	19454171	A	AATATATATA	intronic	*MALRD1*	.	.	.
10	19946474	A	T	intronic	*MALRD1*	.	.	.

10	19481516	C	G	intronic	*MALRD1*	.	.	.
10	19454195	T	TATATATATA	intronic	*MALRD1*	.	.	.
10	19367128	G	A	intronic	*MALRD1*	.	.	.
10	19670283	A	G	intronic	*MALRD1*	.	.	.
10	19732335	C	T	intronic	*MALRD1*	.	.	.
10	19732336	A	G	intronic	*MALRD1*	.	.	.
10	19753778	C	T	intronic	*MALRD1*	.	.	.
10	20026259	A	T	intergenic	*MALRD1,PLXDC2*	.	.	.
9	72741459	T	C	intronic	*MAMDC2*	.	.	.
9	72754324	A	T	intronic	*MAMDC2*	.	.	.
11	96082599	C	T	intergenic	*MAML2,CCDC82*	.	.	.
4	141105784	A	T	intergenic	*MAML3,SCOC*	.	.	.
X	149537309	C	T	intronic	*MAMLD1*	.	.	.
X	149537310	A	G	intronic	*MAMLD1*	.	.	.
6	119571813	CTGTGTG	C	intronic	*MAN1A1*	.	.	.
6	119769494	ATGTGTGTG	A	intergenic	*MAN1A1,LOC285762*	.	.	.
6	119737788	T	C	intergenic	*MAN1A1,LOC285762*	.	.	.
6	119694832	G	A	intergenic	*MAN1A1,LOC285762*	.	.	.
6	119695526	G	A	intergenic	*MAN1A1,LOC285762*	.	.	.
6	119723200	G	C	intergenic	*MAN1A1,LOC285762*	.	.	.
6	119726300	G	A	intergenic	*MAN1A1,LOC285762*	.	.	.
6	119743688	G	T	intergenic	*MAN1A1,LOC285762*	.	.	.
1	118018947	A	G	intronic	*MAN1A2*	.	.	.
1	118020662	A	T	intronic	*MAN1A2*	.	.	.
1	118025265	G	C	intronic	*MAN1A2*	.	.	.
1	118025266	C	T	intronic	*MAN1A2*	.	.	.
1	118026193	G	A	intronic	*MAN1A2*	.	.	.
1	118089420	G	T	intergenic	*MAN1A2,LOC100996263*	.	.	.
1	118117987	TACAC	T	intergenic	*MAN1A2,LOC100996263*	.	.	.

19	12769848	T	G	intronic	*MAN2B1*	.	.	.
19	12776293	C	A	exonic	*MAN2B1*	synonymous_SNV	.	.
4	6637827	A	G	intergenic	*MAN2B2,MRFAP1*	.	.	.
4	6637829	T	C	intergenic	*MAN2B2,MRFAP1*	.	.	.
4	103575970	A	AGTTAGCTG	intronic	*MANBA*	.	.	.
4	103575972	A	ATGAAAGAA	intronic	*MANBA*	.	.	.
6	96170383	T	A	intergenic	*MANEA,FUT9*	.	.	.
6	96086292	G	T	intergenic	*MANEA,FUT9*	.	.	.
6	96093994	C	A	intergenic	*MANEA,FUT9*	.	.	.
6	96157437	A	T	intergenic	*MANEA,FUT9*	.	.	.
6	96168357	C	A	intergenic	*MANEA,FUT9*	.	.	.
6	96169187	T	C	intergenic	*MANEA,FUT9*	.	.	.
X	43530102	A	G	intronic	*MAOA*	.	.	.
X	43630466	T	C	intronic	*MAOB*	.	.	.
X	43636278	T	C	intronic	*MAOB*	.	.	.
X	43781568	A	G	intergenic	*MAOB,NDP*	.	.	.
X	43782538	C	G	intergenic	*MAOB,NDP*	.	.	.
12	117116659	G	T	intergenic	*MAP1LC3B2,C12orf49*	.	.	.
2	210427378	G	A	intronic	*MAP2*	.	.	.
2	210509421	T	A	intronic	*MAP2*	.	.	.
17	21244375	C	CGT	intergenic	*MAP2K3,KCNJ12*	.	.	.
17	21277146	G	T	intergenic	*MAP2K3,KCNJ12*	.	.	.
17	21236680	T	C	intergenic	*MAP2K3,KCNJ12*	.	.	.
17	12006724	T	C	intronic	*MAP2K4*	.	.	.
17	12023099	T	C	intronic	*MAP2K4*	.	.	.
17	12384309	G	T	intergenic	*MAP2K4,LINC00670*	.	.	.
17	12272521	C	T	intergenic	*MAP2K4,LINC00670*	.	.	.
17	12310590	C	G	intergenic	*MAP2K4,LINC00670*	.	.	.
17	12201517	A	T	intergenic	*MAP2K4,LINC00670*	.	.	.
17	12324739	C	A	intergenic	*MAP2K4,LINC00670*	.	.	.
17	12213662	A	G	intergenic	*MAP2K4,LINC00670*	.	.	.

17	12213664	T	A	intergenic	*MAP2K4,LINC00670*	.	.	.
X	72765435	C	G	ncRNA_intronic	*MAP2K4P1*	.	.	.
X	72765436	A	G	ncRNA_intronic	*MAP2K4P1*	.	.	.
X	72766967	G	A	ncRNA_intronic	*MAP2K4P1*	.	.	.
X	72766969	G	T	ncRNA_intronic	*MAP2K4P1*	.	.	.
17	67428601	TA	T	intronic	*MAP2K6*	.	.	.
5	56187536	T	G	intronic	*MAP3K1*	.	.	.
5	56187922	C	T	intronic	*MAP3K1*	.	.	.
17	43460625	G	C	intergenic	*MAP3K14,ARHGAP27*	.	.	.
X	19413610	G	A	intronic	*MAP3K15*	.	.	.
X	19465095	A	G	intronic	*MAP3K15*	.	.	.
X	19440489	A	G	intronic	*MAP3K15*	.	.	.
X	19487282	G	A	intronic	*MAP3K15*	.	.	.
6	137098451	C	T	intronic	*MAP3K5*	.	.	.
6	136938996	A	G	intronic	*MAP3K5*	.	.	.
6	137007914	G	A	intronic	*MAP3K5*	.	.	.
6	137071361	G	A	intronic	*MAP3K5*	.	.	.
6	91291393	A	T	intronic	*MAP3K7*	.	.	.
6	91279194	ACTTCTTT	A	intronic	*MAP3K7*	.	.	.
6	92133111	T	G	intergenic	*MAP3K7,MIR4643*	.	.	.
6	91496597	T	C	intergenic	*MAP3K7,MIR4643*	.	.	.
6	91605652	T	A	intergenic	*MAP3K7,MIR4643*	.	.	.
6	91873321	T	C	intergenic	*MAP3K7,MIR4643*	.	.	.
6	92098095	A	T	intergenic	*MAP3K7,MIR4643*	.	.	.
6	92214092	A	G	intergenic	*MAP3K7,MIR4643*	.	.	.
6	91506573	AAGAG	A	intergenic	*MAP3K7,MIR4643*	.	.	.
6	91338583	T	C	intergenic	*MAP3K7,MIR4643*	.	.	.
6	91339046	G	A	intergenic	*MAP3K7,MIR4643*	.	.	.
6	91339047	T	C	intergenic	*MAP3K7,MIR4643*	.	.	.
6	91523966	A	G	intergenic	*MAP3K7,MIR4643*	.	.	.
6	91524439	A	G	intergenic	*MAP3K7,MIR4643*	.	.	.
6	91757789	G	A	intergenic	*MAP3K7,MIR4643*	.	.	.
6	92007090	T	C	intergenic	*MAP3K7,MIR4643*	.	.	.
6	92048639	G	A	intergenic	*MAP3K7,MIR4643*	.	.	.
6	92063278	A	T	intergenic	*MAP3K7,MIR4643*	.	.	.
6	92079319	C	T	intergenic	*MAP3K7,MIR4643*	.	.	.

6	92082723	T	G	intergenic	*MAP3K7,MIR4643*	.	.	.
6	92083922	C	T	intergenic	*MAP3K7,MIR4643*	.	.	.
6	92112740	T	C	intergenic	*MAP3K7,MIR4643*	.	.	.
3	48005571	C	G	intronic	*MAP4*	.	.	.
2	39558868	GA	G	intronic	*MAP4K3*	.	.	.
2	39544385	A	C	intronic	*MAP4K3*	.	.	.
2	39544386	A	T	intronic	*MAP4K3*	.	.	.
2	102390743	A	G	intronic	*MAP4K4*	.	.	.
2	102556009	G	A	intergenic	*MAP4K4,LINC01127*	.	.	.
14	50994961	G	C	intronic	*MAP4K5*	.	.	.
14	50974967	C	T	intronic	*MAP4K5*	.	.	.
6	136722850	G	A	intronic	*MAP7*	.	.	.
6	136816591	TACACACAC	T	intronic	*MAP7*	.	.	.
1	36654533	T	C	intergenic	*MAP7D1,THRAP3*	.	.	.
X	135379204	T	C	intergenic	*MAP7D3,ADGRG4*	.	.	.
X	135379205	G	A	intergenic	*MAP7D3,ADGRG4*	.	.	.
X	135380226	A	G	intergenic	*MAP7D3,ADGRG4*	.	.	.
4	156303780	C	A	intergenic	*MAP9,GUCY1A3*	.	.	.
4	156495813	A	G	intergenic	*MAP9,GUCY1A3*	.	.	.
4	87145671	G	A	intronic	*MAPK10*	.	.	.
4	86963102	G	A	intronic	*MAPK10*	.	.	.
4	87031192	A	G	intronic	*MAPK10*	.	.	.
4	87159759	T	C	intronic	*MAPK10*	.	.	.
4	87159760	G	A	intronic	*MAPK10*	.	.	.
4	87294410	C	T	intronic	*MAPK10*	.	.	.
14	55520914	G	T	intronic	*MAPK1IP1L*	.	.	.
18	48172084	A	G	intronic	*MAPK4*	.	.	.
22	51057200	C	T	intergenic	*MAPK8IP2,ARSA*	.	.	.
12	112301660	C	T	intronic	*MAPKAPK5*	.	.	.
18	32673658	T	C	intronic	*MAPRE2*	.	.	.
18	32614079	C	T	intronic	*MAPRE2*	.	.	.
18	32688449	T	C	intronic	*MAPRE2*	.	.	.
18	32731801	G	A	intergenic	*MAPRE2,ZNF397*	.	.	.
18	32727530	C	T	intergenic	*MAPRE2,ZNF397*	.	.	.
18	32727532	A	G	intergenic	*MAPRE2,ZNF397*	.	.	.
2	27239620	T	C	intronic	*MAPRE3*	.	.	.

17	43981006	G	A	intronic	*MAPT*	.	.	.
5	16247922	C	T	intergenic	*MARCH11,LOC101929505*	.	.	.
5	16297724	G	A	intergenic	*MARCH11,LOC101929505*	.	.	.
5	16297726	T	C	intergenic	*MARCH11,LOC101929505*	.	.	.
5	16357892	G	A	intergenic	*MARCH11,LOC101929505*	.	.	.
10	94137374	T	A	intergenic	*MARCH5,MARK2P9*	.	.	.
2	119801507	G	GTA	intergenic	*MARCO,C1QL2*	.	.	.
2	119758736	G	A	intergenic	*MARCO,C1QL2*	.	.	.
2	119790146	C	T	intergenic	*MARCO,C1QL2*	.	.	.
2	119790587	A	C	intergenic	*MARCO,C1QL2*	.	.	.
2	119803146	T	C	intergenic	*MARCO,C1QL2*	.	.	.
2	119803147	T	C	intergenic	*MARCO,C1QL2*	.	.	.
1	220812936	G	A	intronic	*MARK1*	.	.	.
1	220711984	C	A	intronic	*MARK1*	.	.	.
1	220780135	T	G	intronic	*MARK1*	.	.	.
1	220782272	G	A	intronic	*MARK1*	.	.	.
1	220782274	G	A	intronic	*MARK1*	.	.	.
1	220818647	G	A	intronic	*MARK1*	.	.	.
5	68766525	G	C	intergenic	*MARVELD2,OCLN*	.	.	.
6	29474186	G	A	intergenic	*MAS1L,LINC01015*	.	.	.
19	12964129	G	T	intronic	*MAST1*	.	.	.
1	46427558	A	AG	intronic	*MAST2*	.	.	.
19	18232241	A	G	intronic	*MAST3*	.	.	.
5	65971179	A	C	intronic	*MAST4*	.	.	.
5	66389487	A	G	intronic	*MAST4*	.	.	.
5	66351827	GATAT	G	intronic	*MAST4*	.	.	.
5	66469306	A	T	intergenic	*MAST4,CD180*	.	.	.
10	82069233	C	T	intergenic	*MAT1A,DYDC1*	.	.	.
5	162998072	T	A	intergenic	*MAT2B,LOC101927835*	.	.	.
5	163090858	C	A	intergenic	*MAT2B,LOC101927835*	.	.	.

5	163221039	T	C	intergenic	*MAT2B,LOC101927835*	.	.	.
5	163095466	A	G	intergenic	*MAT2B,LOC101927835*	.	.	.
5	163164692	G	A	intergenic	*MAT2B,LOC101927835*	.	.	.
5	163088995	C	G	intergenic	*MAT2B,LOC101927835*	.	.	.
5	163071721	G	GTTAT	intergenic	*MAT2B,LOC101927835*	.	.	.
5	163204352	A	G	intergenic	*MAT2B,LOC101927835*	.	.	.
5	163280810	T	C	intergenic	*MAT2B,LOC101927835*	.	.	.
5	163363276	T	C	intergenic	*MAT2B,LOC101927835*	.	.	.
5	163364169	G	A	intergenic	*MAT2B,LOC101927835*	.	.	.
5	163389203	T	G	intergenic	*MAT2B,LOC101927835*	.	.	.
5	163389204	G	T	intergenic	*MAT2B,LOC101927835*	.	.	.
5	163516468	A	G	intergenic	*MAT2B,LOC101927835*	.	.	.
5	163548934	T	C	intergenic	*MAT2B,LOC101927835*	.	.	.
5	163588334	A	T	intergenic	*MAT2B,LOC101927835*	.	.	.
8	99051834	A	G	intergenic	*MATN2,RPL30*	.	.	.
6	74158008	C	T	intronic	*MB21D1*	.	.	.
3	192861326	A	C	intergenic	*MB21D2,HRASLS*	.	.	.
3	192830783	C	G	intergenic	*MB21D2,HRASLS*	.	.	.
18	51759934	C	A	intergenic	*MBD2,POLI*	.	.	.
2	149088224	T	C	intronic	*MBD5*	.	.	.
2	149191457	CCTCCCCG	C	intronic	*MBD5*	.	.	.
2	148886290	A	T	intronic	*MBD5*	.	.	.
2	148909308	G	A	intronic	*MBD5*	.	.	.
2	148927560	G	A	intronic	*MBD5*	.	.	.

2	148962450	T	TA	intronic	*MBD5*	.	.	.
2	148962452	A	ATCGG	intronic	*MBD5*	.	.	.
2	148986516	C	G	intronic	*MBD5*	.	.	.
2	149066619	G	A	intronic	*MBD5*	.	.	.
2	149068563	T	C	intronic	*MBD5*	.	.	.
2	149083345	A	C	intronic	*MBD5*	.	.	.
2	149083441	A	T	intronic	*MBD5*	.	.	.
2	149083865	A	G	intronic	*MBD5*	.	.	.
2	149112743	A	C	intronic	*MBD5*	.	.	.
2	149124328	A	C	intronic	*MBD5*	.	.	.
2	149142283	T	C	intronic	*MBD5*	.	.	.
2	149142633	C	A	intronic	*MBD5*	.	.	.
2	149181683	C	G	intronic	*MBD5*	.	.	.
2	149184358	G	A	intronic	*MBD5*	.	.	.
2	149186118	G	C	intronic	*MBD5*	.	.	.
2	149186848	G	A	intronic	*MBD5*	.	.	.
2	149318924	GC	G	intergenic	*MBD5,EPC2*	.	.	.
2	149318926	T	G	intergenic	*MBD5,EPC2*	.	.	.
2	149378444	G	T	intergenic	*MBD5,EPC2*	.	.	.
14	36884148	A	G	intergenic	*MBIP,SFTA3*	.	.	.
14	36885795	A	G	intergenic	*MBIP,SFTA3*	.	.	.
14	36886001	A	T	intergenic	*MBIP,SFTA3*	.	.	.
14	36886002	A	C	intergenic	*MBIP,SFTA3*	.	.	.
10	55348214	C	CTTTAG	intergenic	*MBL2,PCDH15*	.	.	.
10	54826215	C	A	intergenic	*MBL2,PCDH15*	.	.	.
10	55140612	T	C	intergenic	*MBL2,PCDH15*	.	.	.
10	55176596	G	A	intergenic	*MBL2,PCDH15*	.	.	.
10	55264254	G	T	intergenic	*MBL2,PCDH15*	.	.	.
10	55057076	C	A	intergenic	*MBL2,PCDH15*	.	.	.
10	54550953	T	G	intergenic	*MBL2,PCDH15*	.	.	.
10	54551302	G	A	intergenic	*MBL2,PCDH15*	.	.	.
10	54601334	T	C	intergenic	*MBL2,PCDH15*	.	.	.
10	54601527	G	T	intergenic	*MBL2,PCDH15*	.	.	.
10	54602539	C	A	intergenic	*MBL2,PCDH15*	.	.	.
10	54607276	A	T	intergenic	*MBL2,PCDH15*	.	.	.
10	54622198	C	T	intergenic	*MBL2,PCDH15*	.	.	.
10	54846503	T	C	intergenic	*MBL2,PCDH15*	.	.	.
10	54846505	T	A	intergenic	*MBL2,PCDH15*	.	.	.

10	54874406	T	A	intergenic	*MBL2,PCDH15*	.	.	.
10	54958394	G	A	intergenic	*MBL2,PCDH15*	.	.	.
10	55100632	A	G	intergenic	*MBL2,PCDH15*	.	.	.
10	55101782	T	C	intergenic	*MBL2,PCDH15*	.	.	.
10	55134157	A	G	intergenic	*MBL2,PCDH15*	.	.	.
10	55192430	T	C	intergenic	*MBL2,PCDH15*	.	.	.
10	55330604	T	C	intergenic	*MBL2,PCDH15*	.	.	.
10	55330605	G	A	intergenic	*MBL2,PCDH15*	.	.	.
10	55348365	G	T	intergenic	*MBL2,PCDH15*	.	.	.
10	55348367	C	T	intergenic	*MBL2,PCDH15*	.	.	.
10	55455597	A	G	intergenic	*MBL2,PCDH15*	.	.	.
10	55542490	C	T	intergenic	*MBL2,PCDH15*	.	.	.
10	55556264	C	T	intergenic	*MBL2,PCDH15*	.	.	.
3	152489275	A	G	intergenic	*MBNL1,P2RY1*	.	.	.
3	152298583	C	T	intergenic	*MBNL1,P2RY1*	.	.	.
3	152428932	A	C	intergenic	*MBNL1,P2RY1*	.	.	.
13	97932420	A	C	intronic	*MBNL2*	.	.	.
X	131580945	T	A	intronic	*MBNL3*	.	.	.
X	131583006	C	A	intronic	*MBNL3*	.	.	.
X	131583592	C	T	intronic	*MBNL3*	.	.	.
X	131641581	CAA	C	intergenic	*MBNL3,HS6ST2*	.	.	.
6	20147642	G	A	intronic	*MBOAT1*	.	.	.
6	20384980	A	G	intergenic	*MBOAT1,E2F3*	.	.	.
2	9071377	T	G	intronic	*MBOAT2*	.	.	.
2	9110556	T	C	intronic	*MBOAT2*	.	.	.
2	9110997	G	A	intronic	*MBOAT2*	.	.	.
2	9111452	G	A	intronic	*MBOAT2*	.	.	.
2	9112097	G	A	intronic	*MBOAT2*	.	.	.
2	9233606	A	G	intergenic	*MBOAT2,ASAP2*	.	.	.
2	9181350	A	G	intergenic	*MBOAT2,ASAP2*	.	.	.
18	74821247	A	T	intronic	*MBP*	.	.	.
18	74752467	T	C	intronic	*MBP*	.	.	.
18	74933252	A	C	intergenic	*MBP,GALR1*	.	.	.
17	49327239	A	T	intronic	*MBTD1*	.	.	.
X	21864897	C	G	intronic	*MBTPS2*	.	.	.
18	13905024	C	A	intronic	*MC2R*	.	.	.
18	13905026	G	A	intronic	*MC2R*	.	.	.
18	14037953	G	A	intergenic	*MC2R,ZNF519*	.	.	.

18	14038283	T	A	intergenic	*MC2R,ZNF519*	.	.	.
18	14041586	A	AG	intergenic	*MC2R,ZNF519*	.	.	.
18	14042289	C	T	intergenic	*MC2R,ZNF519*	.	.	.
18	14067779	A	C	intergenic	*MC2R,ZNF519*	.	.	.
18	14067993	A	G	intergenic	*MC2R,ZNF519*	.	.	.
18	58186463	ATGTG	A	intergenic	*MC4R,CDH20*	.	.	.
18	58267971	T	C	intergenic	*MC4R,CDH20*	.	.	.
18	59063462	C	A	intergenic	*MC4R,CDH20*	.	.	.
18	58189248	C	A	intergenic	*MC4R,CDH20*	.	.	.
18	58209343	A	T	intergenic	*MC4R,CDH20*	.	.	.
18	58209344	C	T	intergenic	*MC4R,CDH20*	.	.	.
18	58465028	C	T	intergenic	*MC4R,CDH20*	.	.	.
18	58465029	A	G	intergenic	*MC4R,CDH20*	.	.	.
18	58465438	A	G	intergenic	*MC4R,CDH20*	.	.	.
18	58465460	C	A	intergenic	*MC4R,CDH20*	.	.	.
18	58469997	A	C	intergenic	*MC4R,CDH20*	.	.	.
18	58524475	G	A	intergenic	*MC4R,CDH20*	.	.	.
18	13835989	A	T	intergenic	*MC5R,MC2R*	.	.	.
18	13858972	C	T	intergenic	*MC5R,MC2R*	.	.	.
18	13859112	C	T	intergenic	*MC5R,MC2R*	.	.	.
18	13860001	A	T	intergenic	*MC5R,MC2R*	.	.	.
5	112412274	A	G	intronic	*MCC*	.	.	.
5	112412276	T	G	intronic	*MCC*	.	.	.
5	112415341	A	T	intronic	*MCC*	.	.	.
5	112436153	A	T	intronic	*MCC*	.	.	.
5	112481446	CACACACACACACACACACACACACACACACACAA	C	intronic	*MCC*	.	.	.
5	112749786	T	A	intronic	*MCC*	.	.	.
5	112751055	A	G	intronic	*MCC*	.	.	.
X	138754046	G	C	intronic	*MCF2*	.	.	.
13	113644652	G	T	intronic	*MCF2L*	.	.	.
13	113733338	C	A	intronic	*MCF2L*	.	.	.
3	183123445	C	T	intronic	*MCF2L2*	.	.	.
6	100375428	G	A	intronic	*MCHR2*	.	.	.
6	100478919	C	T	ncRNA_intronic	*MCHR2-AS1*	.	.	.

6	100478920	A	G	ncRNA_intronic	*MCHR2-AS1*	.	.	.
6	100488312	G	A	ncRNA_intronic	*MCHR2-AS1*	.	.	.
6	100488313	T	C	ncRNA_intronic	*MCHR2-AS1*	.	.	.
6	100492142	G	A	ncRNA_intronic	*MCHR2-AS1*	.	.	.
6	100519577	T	C	ncRNA_intronic	*MCHR2-AS1*	.	.	.
6	100578120	G	T	intergenic	*MCHR2-AS1,SIM1*	.	.	.
6	100743263	G	C	intergenic	*MCHR2-AS1,SIM1*	.	.	.
22	35839265	C	T	intergenic	*MCM5,RASD2*	.	.	.
2	136614230	T	G	intronic	*MCM6*	.	.	.
6	119189632	C	A	intronic	*MCM9*	.	.	.
10	121611546	T	C	intronic	*MCMBP*	.	.	.
8	6349067	G	A	intronic	*MCPH1*	.	.	.
8	6510861	A	T	ncRNA_intronic	*MCPH1-AS1*	.	.	.
8	6512209	G	A	ncRNA_intronic	*MCPH1-AS1*	.	.	.
5	94456449	AC	A	intronic	*MCTP1*	.	.	.
5	94328810	T	A	intronic	*MCTP1*	.	.	.
5	94053466	T	C	intronic	*MCTP1*	.	.	.
5	94053963	A	G	intronic	*MCTP1*	.	.	.
5	94160802	T	C	intronic	*MCTP1*	.	.	.
5	94160804	A	C	intronic	*MCTP1*	.	.	.
5	94161222	A	T	intronic	*MCTP1*	.	.	.
5	94295289	G	A	intronic	*MCTP1*	.	.	.
5	94430684	A	T	intronic	*MCTP1*	.	.	.
5	94504847	T	A	intronic	*MCTP1*	.	.	.
5	94505441	A	T	intronic	*MCTP1*	.	.	.
5	94506262	A	G	intronic	*MCTP1*	.	.	.
5	94545695	T	A	intronic	*MCTP1*	.	.	.
5	94582913	A	T	intronic	*MCTP1*	.	.	.
5	94583350	T	G	intronic	*MCTP1*	.	.	.
5	94583755	A	C	intronic	*MCTP1*	.	.	.
5	94583757	T	C	intronic	*MCTP1*	.	.	.
5	94676317	C	A	intergenic	*MCTP1,FAM81B*	.	.	.
5	94676855	T	C	intergenic	*MCTP1,FAM81B*	.	.	.
5	94678891	A	G	intergenic	*MCTP1,FAM81B*	.	.	.
5	94692070	G	C	intergenic	*MCTP1,FAM81B*	.	.	.
X	119746076	A	G	exonic	*MCTS1*	nonsynonymous_SNV	.	.
X	119740669	T	A	intronic	*MCTS1*	.	.	.

6	41641784	C	T	intergenic	*MDFI,TFEB*	.	.	.
7	114565390	C	T	intronic	*MDFIC*	.	.	.
7	114630865	A	G	intronic	*MDFIC*	.	.	.
7	114633560	T	C	intronic	*MDFIC*	.	.	.
7	114692233	C	A	intergenic	*MDFIC,LINC01393*	.	.	.
7	114665825	A	T	intergenic	*MDFIC,LINC01393*	.	.	.
6	37621373	A	T	intronic	*MDGA1*	.	.	.
6	37701991	G	A	intergenic	*MDGA1,ZFAND3*	.	.	.
14	47315732	G	A	intronic	*MDGA2*	.	.	.
14	47523524	TC	T	intronic	*MDGA2*	.	.	.
14	47523526	TTA	T	intronic	*MDGA2*	.	.	.
14	47338572	TG	T	intronic	*MDGA2*	.	.	.
14	47884993	C	T	intronic	*MDGA2*	.	.	.
14	47751330	G	A	intronic	*MDGA2*	.	.	.
14	47771289	T	C	intronic	*MDGA2*	.	.	.
14	47452150	T	C	intronic	*MDGA2*	.	.	.
14	47452151	G	A	intronic	*MDGA2*	.	.	.
14	47643551	T	C	intronic	*MDGA2*	.	.	.
14	47643716	A	T	intronic	*MDGA2*	.	.	.
14	47643941	G	A	intronic	*MDGA2*	.	.	.
14	47643943	C	T	intronic	*MDGA2*	.	.	.
14	47646650	G	T	intronic	*MDGA2*	.	.	.
14	47943335	C	G	intronic	*MDGA2*	.	.	.
14	47944585	A	G	intronic	*MDGA2*	.	.	.
14	48186041	G	A	intergenic	*MDGA2,MIR548Y*	.	.	.
14	48164318	C	A	intergenic	*MDGA2,MIR548Y*	.	.	.
14	48185760	C	T	intergenic	*MDGA2,MIR548Y*	.	.	.
2	63899340	T	G	intergenic	*MDH1,UGP2*	.	.	.
2	63846303	A	G	intergenic	*MDH1,UGP2*	.	.	.
2	63848124	C	T	intergenic	*MDH1,UGP2*	.	.	.
2	63850753	C	T	intergenic	*MDH1,UGP2*	.	.	.
2	63850754	A	T	intergenic	*MDH1,UGP2*	.	.	.
2	63931499	T	C	intergenic	*MDH1,UGP2*	.	.	.
2	63953387	A	G	intergenic	*MDH1,UGP2*	.	.	.
2	63953389	C	T	intergenic	*MDH1,UGP2*	.	.	.
1	204509783	C	T	intronic	*MDM4*	.	.	.
1	204550357	TA	T	intergenic	*MDM4,LRRN2*	.	.	.
6	90527680	T	A	intronic	*MDN1*	.	.	.

6	90462715	G	A	intronic	*MDN1*	.	.	.
6	83978447	GTTATTA	G	intronic	*ME1*	.	.	.
6	84102287	C	T	intronic	*ME1*	.	.	.
6	83966381	C	A	intronic	*ME1*	.	.	.
6	83998590	G	A	intronic	*ME1*	.	.	.
6	84008726	T	TA	intronic	*ME1*	.	.	.
6	84027057	C	A	intronic	*ME1*	.	.	.
6	84034280	A	ATG	intronic	*ME1*	.	.	.
6	84036094	G	GC	intronic	*ME1*	.	.	.
6	84085565	A	G	intronic	*ME1*	.	.	.
6	84208430	T	C	intergenic	*ME1,PRSS35*	.	.	.
11	86342029	G	T	intronic	*ME3*	.	.	.
11	86240226	A	G	intronic	*ME3*	.	.	.
11	86242662	A	C	intronic	*ME3*	.	.	.
11	86359709	A	C	intronic	*ME3*	.	.	.
11	86363154	G	A	intronic	*ME3*	.	.	.
6	165254152	A	T	intergenic	*MEAT6,C6orf118*	.	.	.
6	165436352	G	A	intergenic	*MEAT6,C6orf118*	.	.	.
6	165403840	T	A	intergenic	*MEAT6,C6orf118*	.	.	.
6	165551084	G	T	intergenic	*MEAT6,C6orf118*	.	.	.
6	165372773	A	C	intergenic	*MEAT6,C6orf118*	.	.	.
6	165431446	T	G	intergenic	*MEAT6,C6orf118*	.	.	.
6	165571607	A	T	intergenic	*MEAT6,C6orf118*	.	.	.
3	169350609	A	G	intronic	*MECOM*	.	.	.
3	168818673	CTG	C	exonic	*MECOM*	frameshift_deletion	.	.
X	153372164	G	A	intergenic	*MECP2,OPN1LW*	.	.	.
17	37613585	C	A	intergenic	*MED1,CDK12*	.	.	.
3	150891272	A	G	intronic	*MED12L*	.	.	.
3	150996886	T	C	intronic	*MED12L*	.	.	.
17	60019700	A	G	downstream	*MED13*	.	.	.
17	60030861	A	C	intronic	*MED13*	.	.	.
17	60080824	G	T	intronic	*MED13*	.	.	.
17	60138620	T	C	intronic	*MED13*	.	.	.
17	60185646	T	A	intergenic	*MED13,TBC1D3P2*	.	.	.
17	60218921	G	A	intergenic	*MED13,TBC1D3P2*	.	.	.
12	116689940	T	TTTTAG	intronic	*MED13L*	.	.	.
X	40580441	T	C	intronic	*MED14*	.	.	.
22	20941478	C	T	UTR3	*MED15*	.	.	.

22	20971142	G	A	intergenic	*MED15,POM121L4P*	.	.	.
12	27225809	A	G	intergenic	*MED21,C12orf71*	.	.	.
17	38200665	C	T	intronic	*MED24*	.	.	.
9	134861103	T	C	intronic	*MED27*	.	.	.
8	118649641	C	T	intergenic	*MED30,EXT1*	.	.	.
8	118678901	T	C	intergenic	*MED30,EXT1*	.	.	.
8	118799481	A	T	intergenic	*MED30,EXT1*	.	.	.
8	118799798	T	G	intergenic	*MED30,EXT1*	.	.	.
13	48777860	G	A	intergenic	*MED4,ITM2B*	.	.	.
13	48778049	G	C	intergenic	*MED4,ITM2B*	.	.	.
5	88115892	TTATA	T	intronic	*MEF2C*	.	.	.
5	89180195	G	C	intergenic	*MEF2C-AS1,MIR3660*	.	.	.
5	88969750	C	A	intergenic	*MEF2C-AS1,MIR3660*	.	.	.
5	88987800	T	A	intergenic	*MEF2C-AS1,MIR3660*	.	.	.
5	88460378	A	G	intergenic	*MEF2C-AS1,MIR3660*	.	.	.
5	88518677	G	A	intergenic	*MEF2C-AS1,MIR3660*	.	.	.
5	88518782	T	C	intergenic	*MEF2C-AS1,MIR3660*	.	.	.
5	88627998	T	C	intergenic	*MEF2C-AS1,MIR3660*	.	.	.
5	88760774	C	T	intergenic	*MEF2C-AS1,MIR3660*	.	.	.
5	88908111	T	A	intergenic	*MEF2C-AS1,MIR3660*	.	.	.
5	88928607	C	T	intergenic	*MEF2C-AS1,MIR3660*	.	.	.
5	88928608	A	G	intergenic	*MEF2C-AS1,MIR3660*	.	.	.
5	88929026	G	A	intergenic	*MEF2C-AS1,MIR3660*	.	.	.
5	89034637	C	G	intergenic	*MEF2C-AS1,MIR3660*	.	.	.

5	89040837	G	A	intergenic	*MEF2C-AS1,MIR3660*	.	.	.
5	89041675	C	T	intergenic	*MEF2C-AS1,MIR3660*	.	.	.
5	89099567	G	C	intergenic	*MEF2C-AS1,MIR3660*	.	.	.
5	89101594	A	G	intergenic	*MEF2C-AS1,MIR3660*	.	.	.
5	89101793	A	G	intergenic	*MEF2C-AS1,MIR3660*	.	.	.
14	101732238	G	A	intergenic	*MEG9,LINC00524*	.	.	.
14	101756628	TACAC	T	intergenic	*MEG9,LINC00524*	.	.	.
14	101671434	G	T	intergenic	*MEG9,LINC00524*	.	.	.
14	101735182	G	A	intergenic	*MEG9,LINC00524*	.	.	.
5	126813215	A	AGAGATCGC	intergenic	*MEGF10,PRRC1*	.	.	.
1	3531390	A	G	intergenic	*MEGF6,TPRG1L*	.	.	.
6	78534880	T	A	intronic	*MEI4*	.	.	.
6	78407831	C	G	intronic	*MEI4*	.	.	.
6	78439865	G	A	intronic	*MEI4*	.	.	.
6	78448520	G	T	intronic	*MEI4*	.	.	.
6	78455709	A	G	intronic	*MEI4*	.	.	.
6	78461700	A	G	intronic	*MEI4*	.	.	.
6	78475636	G	C	intronic	*MEI4*	.	.	.
6	78498480	G	C	intronic	*MEI4*	.	.	.
6	78499048	A	G	intronic	*MEI4*	.	.	.
6	78526319	G	C	intronic	*MEI4*	.	.	.
6	78620218	C	T	intronic	*MEI4*	.	.	.
6	78784639	T	A	intergenic	*MEI4,IRAK1BP1*	.	.	.
6	79008363	T	A	intergenic	*MEI4,IRAK1BP1*	.	.	.
6	79077973	G	T	intergenic	*MEI4,IRAK1BP1*	.	.	.
6	78832474	A	C	intergenic	*MEI4,IRAK1BP1*	.	.	.
6	78833523	C	T	intergenic	*MEI4,IRAK1BP1*	.	.	.
6	79551177	T	A	intergenic	*MEI4,IRAK1BP1*	.	.	.
6	78779999	C	A	intergenic	*MEI4,IRAK1BP1*	.	.	.
6	78780096	G	T	intergenic	*MEI4,IRAK1BP1*	.	.	.
6	79081372	T	C	intergenic	*MEI4,IRAK1BP1*	.	.	.
6	79179891	C	T	intergenic	*MEI4,IRAK1BP1*	.	.	.
6	79182769	G	A	intergenic	*MEI4,IRAK1BP1*	.	.	.

6	79281287	A	C	intergenic	*MEI4,IRAK1BP1*	.	.	.
6	79354441	C	G	intergenic	*MEI4,IRAK1BP1*	.	.	.
6	79355747	T	C	intergenic	*MEI4,IRAK1BP1*	.	.	.
6	79359134	C	A	intergenic	*MEI4,IRAK1BP1*	.	.	.
6	79359350	T	A	intergenic	*MEI4,IRAK1BP1*	.	.	.
6	79359509	A	C	intergenic	*MEI4,IRAK1BP1*	.	.	.
6	79390603	G	A	intergenic	*MEI4,IRAK1BP1*	.	.	.
6	79391459	A	G	intergenic	*MEI4,IRAK1BP1*	.	.	.
6	79391734	A	T	intergenic	*MEI4,IRAK1BP1*	.	.	.
6	79406915	G	T	intergenic	*MEI4,IRAK1BP1*	.	.	.
6	79438856	G	A	intergenic	*MEI4,IRAK1BP1*	.	.	.
6	79448414	T	C	intergenic	*MEI4,IRAK1BP1*	.	.	.
6	79451172	A	C	intergenic	*MEI4,IRAK1BP1*	.	.	.
6	79472124	T	C	intergenic	*MEI4,IRAK1BP1*	.	.	.
6	79472125	G	A	intergenic	*MEI4,IRAK1BP1*	.	.	.
6	79495369	C	T	intergenic	*MEI4,IRAK1BP1*	.	.	.
6	79501428	A	ACCT	intergenic	*MEI4,IRAK1BP1*	.	.	.
6	79514619	C	T	intergenic	*MEI4,IRAK1BP1*	.	.	.
6	79514620	A	G	intergenic	*MEI4,IRAK1BP1*	.	.	.
6	79530606	A	G	intergenic	*MEI4,IRAK1BP1*	.	.	.
10	15058735	A	G	intergenic	*MEIG1,OLAH*	.	.	.
5	131224218	C	T	intronic	*MEIKIN*	.	.	.
5	131229721	G	T	intronic	*MEIKIN*	.	.	.
5	131232267	A	G	intronic	*MEIKIN*	.	.	.
16	1896578	A	T	intronic	*MEIOB*	.	.	.
2	66713641	C	T	intronic	*MEIS1*	.	.	.
15	37267801	C	T	intronic	*MEIS2*	.	.	.
15	37522838	C	A	intergenic	*MEIS2,TMCO5A*	.	.	.
15	37553452	G	A	intergenic	*MEIS2,TMCO5A*	.	.	.
15	37658658	A	T	intergenic	*MEIS2,TMCO5A*	.	.	.
15	37690983	T	C	intergenic	*MEIS2,TMCO5A*	.	.	.
15	37565077	C	A	intergenic	*MEIS2,TMCO5A*	.	.	.
15	37506262	T	C	intergenic	*MEIS2,TMCO5A*	.	.	.
15	37595535	A	T	intergenic	*MEIS2,TMCO5A*	.	.	.
15	37646439	C	T	intergenic	*MEIS2,TMCO5A*	.	.	.
15	37646440	A	G	intergenic	*MEIS2,TMCO5A*	.	.	.
15	37646736	C	A	intergenic	*MEIS2,TMCO5A*	.	.	.
15	37723159	T	A	intergenic	*MEIS2,TMCO5A*	.	.	.

15	38216182	A	G	intergenic	*MEIS2,TMCO5A*	.	.	.
17	15699591	C	T	intergenic	*MEIS3P1,LOC101928567*	.	.	.
2	32123051	A	G	intronic	*MEMO1*	.	.	.
7	15873259	A	G	intergenic	*MEOX2-AS1,ISPD*	.	.	.
7	15746131	A	G	intergenic	*MEOX2-AS1,ISPD*	.	.	.
7	16000509	T	G	intergenic	*MEOX2-AS1,ISPD*	.	.	.
7	16000510	A	C	intergenic	*MEOX2-AS1,ISPD*	.	.	.
7	16030396	A	G	intergenic	*MEOX2-AS1,ISPD*	.	.	.
7	16032484	C	T	intergenic	*MEOX2-AS1,ISPD*	.	.	.
7	16032486	C	T	intergenic	*MEOX2-AS1,ISPD*	.	.	.
4	88799727	G	C	intergenic	*MEPE,SPP1*	.	.	.
4	88809948	T	C	intergenic	*MEPE,SPP1*	.	.	.
2	112728315	A	C	intronic	*MERTK*	.	.	.
2	112692144	C	T	intronic	*MERTK*	.	.	.
15	90297879	G	A	intergenic	*MESP1,MESP2*	.	.	.
7	116361797	T	C	intronic	*MET*	.	.	.
7	116390246	T	C	intronic	*MET*	.	.	.
7	116390248	G	A	intronic	*MET*	.	.	.
7	116390412	T	C	intronic	*MET*	.	.	.
4	99926434	G	A	intronic	*METAP1*	.	.	.
2	172907752	A	G	intronic	*METAP1D*	.	.	.
17	81058340	AGGGGGTGTGGCGGGTCTGTGGGAGGGGGACAAGGTTGGCAGGACCGTGAGGGGGTGGCGGGTCTGTGGGAGGGGGACAAGGTTGGCAGGACCGTGAG	A	intergenic	*METRNL,RPL23AP87*	.	.	.
17	81083285	T	G	intergenic	*METRNL,RPL23AP87*	.	.	.

17	81171719	G	C	intergenic	*METRNL,RPL23AP87*	.	.	.
1	170142176	G	T	intergenic	*METTL11B,LINC01142*	.	.	.
1	170142177	G	A	intergenic	*METTL11B,LINC01142*	.	.	.
11	28205291	T	C	intronic	*METTL15*	.	.	.
11	28239142	C	G	intronic	*METTL15*	.	.	.
11	28257192	T	C	intronic	*METTL15*	.	.	.
11	28348396	G	A	intronic	*METTL15*	.	.	.
11	28348397	G	C	intronic	*METTL15*	.	.	.
11	28366238	T	C	intergenic	*METTL15,MIR8068*	.	.	.
11	28413446	A	G	intergenic	*METTL15,MIR8068*	.	.	.
11	28448586	A	G	intergenic	*METTL15,MIR8068*	.	.	.
17	2361021	G	T	intronic	*METTL16*	.	.	.
17	2417655	G	T	intergenic	*METTL16,PAFAH1B1*	.	.	.
17	2478440	C	T	intergenic	*METTL16,PAFAH1B1*	.	.	.
17	2478441	A	G	intergenic	*METTL16,PAFAH1B1*	.	.	.
2	208504167	A	G	intergenic	*METTL21A,CCNYL1*	.	.	.
2	208528058	A	G	intergenic	*METTL21A,CCNYL1*	.	.	.
13	103359021	T	C	intergenic	*METTL21C,CCDC168*	.	.	.
13	103683883	C	G	intergenic	*METTL21EP,SLC10A2*	.	.	.
13	103658685	G	A	intergenic	*METTL21EP,SLC10A2*	.	.	.
16	8740096	T	C	downstream	*METTL22*	.	.	.
16	8755927	TG	T	intergenic	*METTL22,ABAT*	.	.	.
6	110584150	T	C	intronic	*METTL24*	.	.	.
6	110626476	C	A	intronic	*METTL24*	.	.	.
6	110704495	G	A	intergenic	*METTL24,DDO*	.	.	.
6	110704715	T	C	intergenic	*METTL24,DDO*	.	.	.
6	110704716	G	A	intergenic	*METTL24,DDO*	.	.	.

12	82854314	G	A	intronic	*METTL25*	.	.	.
12	82757172	G	A	intronic	*METTL25*	.	.	.
12	82815541	T	C	intronic	*METTL25*	.	.	.
12	82942982	C	T	intergenic	*METTL25,TMTC2*	.	.	.
12	82982255	A	G	intergenic	*METTL25,TMTC2*	.	.	.
12	83024835	T	C	intergenic	*METTL25,TMTC2*	.	.	.
12	83027545	C	A	intergenic	*METTL25,TMTC2*	.	.	.
7	128166878	C	G	intergenic	*METTL2B,LINC01000*	.	.	.
18	2551830	G	T	intronic	*METTL4*	.	.	.
18	2552132	C	T	intronic	*METTL4*	.	.	.
2	170673188	C	G	intronic	*METTL5*	.	.	.
12	51338787	C	G	intergenic	*METTL7A,HIGD1C*	.	.	.
12	56075656	A	C	exonic	*METTL7B*	nonsynonymous_SNV	.	.
2	172220828	A	C	intronic	*METTL8*	.	.	.
2	172207212	C	T	intronic	*METTL8*	.	.	.
2	172207608	A	T	intronic	*METTL8*	.	.	.
2	172208021	C	T	intronic	*METTL8*	.	.	.
18	48705904	CTTTGAGCATTATGCTGGTGCTCAAAAAGCCTCAGATTTTGGAGCTCTAAAACAAAATCGGAGGCTT	C	intronic	*MEX3C*	.	.	.
18	48886513	TATTGAATG	T	intergenic	*MEX3C,LOC100287225*	.	.	.
18	48899428	T	C	intergenic	*MEX3C,LOC100287225*	.	.	.
15	89525080	C	T	intergenic	*MFGE8,ABHD2*	.	.	.
17	74752361	C	T	intronic	*MFSD11*	.	.	.
1	100511907	C	T	intronic	*MFSD14A*	.	.	.
9	97167737	T	C	intronic	*MFSD14B*	.	.	.
9	97193744	T	G	intronic	*MFSD14B*	.	.	.
9	97198136	A	ATGT	intronic	*MFSD14B*	.	.	.

9	97232703	A	G	intergenic	*MFSD14B,PCAT7*	.	.	.
9	97232908	C	G	intergenic	*MFSD14B,PCAT7*	.	.	.
9	97268820	T	C	intergenic	*MFSD14B,PCAT7*	.	.	.
9	99780960	G	T	intergenic	*MFSD14C,CTSV*	.	.	.
1	40461279	T	C	intergenic	*MFSD2A,CAP1*	.	.	.
1	205572806	G	A	downstream	*MFSD4*	.	.	.
2	191338082	A	G	intronic	*MFSD6*	.	.	.
2	191341133	T	A	intronic	*MFSD6*	.	.	.
15	42055276	A	T	intronic	*MGA*	.	.	.
7	141726582	G	C	intronic	*MGAM*	.	.	.
7	141781613	G	T	intronic	*MGAM*	.	.	.
7	141730618	CTGTG	C	intronic	*MGAM*	.	.	.
7	141878101	G	T	intronic	*MGAM2*	.	.	.
7	141891092	C	A	intronic	*MGAM2*	.	.	.
5	180229775	G	C	UTR5	*MGAT1*	.	.	.
2	99240641	A	T	UTR3	*MGAT4A*	.	.	.
2	99322302	G	A	intronic	*MGAT4A*	.	.	.
2	99322303	G	T	intronic	*MGAT4A*	.	.	.
12	86426027	G	T	intronic	*MGAT4C*	.	.	.
12	87218957	T	C	intronic	*MGAT4C*	.	.	.
12	86413877	G	A	intronic	*MGAT4C*	.	.	.
12	86432902	A	G	intronic	*MGAT4C*	.	.	.
12	86457541	G	A	intronic	*MGAT4C*	.	.	.
12	86515386	T	A	intronic	*MGAT4C*	.	.	.
12	86555716	T	C	intronic	*MGAT4C*	.	.	.
12	86681783	T	G	intronic	*MGAT4C*	.	.	.
12	86739717	C	T	intronic	*MGAT4C*	.	.	.
12	86751677	T	C	intronic	*MGAT4C*	.	.	.
12	86751679	T	C	intronic	*MGAT4C*	.	.	.
12	86753161	T	C	intronic	*MGAT4C*	.	.	.
12	86779739	C	T	intronic	*MGAT4C*	.	.	.
12	86887345	T	G	intronic	*MGAT4C*	.	.	.
12	86903230	A	G	intronic	*MGAT4C*	.	.	.
12	86903231	C	G	intronic	*MGAT4C*	.	.	.
12	86980897	G	A	intronic	*MGAT4C*	.	.	.
12	86981453	G	T	intronic	*MGAT4C*	.	.	.
12	87024455	G	A	intronic	*MGAT4C*	.	.	.
12	87024974	A	G	intronic	*MGAT4C*	.	.	.

12	87078710	G	T	intronic	*MGAT4C*	.	.	.
12	87159740	T	C	intronic	*MGAT4C*	.	.	.
12	87163397	A	G	intronic	*MGAT4C*	.	.	.
12	87411339	T	G	intergenic	*MGAT4C,MKRN9P*	.	.	.
12	88018481	C	G	intergenic	*MGAT4C,MKRN9P*	.	.	.
12	88151591	A	G	intergenic	*MGAT4C,MKRN9P*	.	.	.
12	87915802	G	A	intergenic	*MGAT4C,MKRN9P*	.	.	.
12	88034312	T	A	intergenic	*MGAT4C,MKRN9P*	.	.	.
12	87367144	T	C	intergenic	*MGAT4C,MKRN9P*	.	.	.
12	87368129	G	A	intergenic	*MGAT4C,MKRN9P*	.	.	.
12	87368528	C	A	intergenic	*MGAT4C,MKRN9P*	.	.	.
12	87368790	C	T	intergenic	*MGAT4C,MKRN9P*	.	.	.
12	87710096	AACACACA C	A	intergenic	*MGAT4C,MKRN9P*	.	.	.
12	87790423	G	A	intergenic	*MGAT4C,MKRN9P*	.	.	.
12	87927077	A	C	intergenic	*MGAT4C,MKRN9P*	.	.	.
12	87937630	G	A	intergenic	*MGAT4C,MKRN9P*	.	.	.
12	87957064	T	C	intergenic	*MGAT4C,MKRN9P*	.	.	.
12	88059253	C	T	intergenic	*MGAT4C,MKRN9P*	.	.	.
12	88059402	T	C	intergenic	*MGAT4C,MKRN9P*	.	.	.
12	88075135	C	T	intergenic	*MGAT4C,MKRN9P*	.	.	.
12	88091101	C	T	intergenic	*MGAT4C,MKRN9P*	.	.	.
12	88091814	G	A	intergenic	*MGAT4C,MKRN9P*	.	.	.
12	88099330	G	A	intergenic	*MGAT4C,MKRN9P*	.	.	.
12	88099332	T	C	intergenic	*MGAT4C,MKRN9P*	.	.	.
12	88129283	G	C	intergenic	*MGAT4C,MKRN9P*	.	.	.
4	141405659	T	A	intronic	*MGAT4D*	.	.	.
4	141435027	G	C	intergenic	*MGAT4D,ELMOD2*	.	.	.
4	141442694	A	T	intergenic	*MGAT4D,ELMOD2*	.	.	.
2	135144757	G	A	intronic	*MGAT5*	.	.	.
7	127937184	A	C	downstream	*MGC27345*	.	.	.
1	78749087	C	T	ncRNA_intronic	*MGC27382*	.	.	.
1	78780084	T	A	ncRNA_intronic	*MGC27382*	.	.	.
1	78944891	C	T	intergenic	*MGC27382,PTGFR*	.	.	.
5	121853983	T	C	intergenic	*MGC32805,LOC101927379*	.	.	.
5	121930707	T	C	intergenic	*MGC32805,LOC101927379*	.	.	.

7	97607356	A	C	intergenic	*MGC72080,OCM2*	.	.	.
10	103550044	TTTTG	T	intronic	*MGEA5*	.	.	.
3	127538422	A	G	intronic	*MGLL*	.	.	.
10	131403165	G	T	intronic	*MGMT*	.	.	.
10	131374164	T	C	intronic	*MGMT*	.	.	.
10	131536936	A	G	intronic	*MGMT*	.	.	.
10	131577469	C	G	intergenic	*MGMT,EBF3*	.	.	.
16	4677747	C	T	intronic	*MGRN1*	.	.	.
14	39729539	C	T	intergenic	*MIA2,CTAGE5*	.	.	.
14	39729540	A	G	intergenic	*MIA2,CTAGE5*	.	.	.
14	39731624	T	C	intergenic	*MIA2,CTAGE5*	.	.	.
18	19439880	T	A	intronic	*MIB1*	.	.	.
6	31415864	T	G	intergenic	*MICA,HCP5*	.	.	.
11	12161391	T	C	intronic	*MICAL2*	.	.	.
22	18333368	A	G	intronic	*MICAL3*	.	.	.
22	18415183	G	A	intronic	*MICAL3*	.	.	.
11	12389945	C	A	intergenic	*MICALCL,PARVA*	.	.	.
11	12390031	T	G	intergenic	*MICALCL,PARVA*	.	.	.
11	12392155	G	A	intergenic	*MICALCL,PARVA*	.	.	.
10	74300972	A	G	intronic	*MICU1*	.	.	.
10	74157451	C	T	intronic	*MICU1*	.	.	.
10	74197980	T	C	intronic	*MICU1*	.	.	.
10	74198475	G	T	intronic	*MICU1*	.	.	.
10	74200337	T	C	intronic	*MICU1*	.	.	.
10	74200338	G	A	intronic	*MICU1*	.	.	.
10	74324941	C	T	intronic	*MICU1*	.	.	.
10	74439485	C	G	intergenic	*MICU1,MCU*	.	.	.
10	74435301	G	A	intergenic	*MICU1,MCU*	.	.	.
13	22126829	T	C	intronic	*MICU2*	.	.	.
13	22126831	C	T	intronic	*MICU2*	.	.	.
13	22128116	A	G	intronic	*MICU2*	.	.	.
13	22129105	T	C	intronic	*MICU2*	.	.	.
13	22167468	A	G	intronic	*MICU2*	.	.	.
X	10620382	T	A	intronic	*MID1*	.	.	.
X	10609125	C	T	intronic	*MID1*	.	.	.
X	10610185	C	T	intronic	*MID1*	.	.	.
X	10685953	A	C	intronic	*MID1*	.	.	.
X	10686924	A	T	intronic	*MID1*	.	.	.

X	10688404	G	C	intronic	*MID1*	.	.	.
X	10892158	G	T	intergenic	*MID1,HCCS*	.	.	.
X	11058872	A	G	intergenic	*MID1,HCCS*	.	.	.
X	10904684	A	T	intergenic	*MID1,HCCS*	.	.	.
X	10890770	T	C	intergenic	*MID1,HCCS*	.	.	.
X	10975921	T	A	intergenic	*MID1,HCCS*	.	.	.
X	11039683	A	G	intergenic	*MID1,HCCS*	.	.	.
X	38900771	T	A	intergenic	*MID1IP1,LINC01281*	.	.	.
X	39152009	G	T	intergenic	*MID1IP1,LINC01281*	.	.	.
X	39056254	C	G	intergenic	*MID1IP1,LINC01281*	.	.	.
X	39118064	C	A	intergenic	*MID1IP1,LINC01281*	.	.	.
X	39033232	T	A	intergenic	*MID1IP1,LINC01281*	.	.	.
X	38734441	GTATA	G	intergenic	*MID1IP1,LINC01281*	.	.	.
X	38685539	T	C	intergenic	*MID1IP1,LINC01281*	.	.	.
X	38951572	C	G	intergenic	*MID1IP1,LINC01281*	.	.	.
X	39042762	T	C	intergenic	*MID1IP1,LINC01281*	.	.	.
X	107129567	C	A	intronic	*MID2*	.	.	.
1	67445462	A	T	intronic	*MIER1*	.	.	.
5	56295023	G	GT	intergenic	*MIER3,GPBP1*	.	.	.
5	56328931	T	C	intergenic	*MIER3,GPBP1*	.	.	.
5	56375794	C	T	intergenic	*MIER3,GPBP1*	.	.	.
22	24263089	T	C	intergenic	*MIF-AS1,GSTT2B*	.	.	.
19	57520298	C	T	intergenic	*MIMT1,USP29*	.	.	.
19	57469789	A	AC	intergenic	*MIMT1,USP29*	.	.	.
19	57552430	A	G	intergenic	*MIMT1,USP29*	.	.	.
19	57362017	T	A	intergenic	*MIMT1,USP29*	.	.	.
19	57376319	G	T	intergenic	*MIMT1,USP29*	.	.	.
19	57391627	T	C	intergenic	*MIMT1,USP29*	.	.	.
19	57391629	T	C	intergenic	*MIMT1,USP29*	.	.	.

19	57466833	A	G	intergenic	*MIMT1,USP29*	.	.	.
17	4774531	G	A	intronic	*MINK1*	.	.	.
10	89287717	G	A	intronic	*MINPP1*	.	.	.
10	89288030	T	C	intronic	*MINPP1*	.	.	.
10	89340544	T	C	intergenic	*MINPP1,PAPSS2*	.	.	.
22	50934452	C	G	intergenic	*MIOX,LMF2*	.	.	.
13	24359718	T	C	intronic	*MIPEP*	.	.	.
14	38019700	CATAT	C	UTR3	*MIPOL1*	.	.	.
14	37961999	G	T	intronic	*MIPOL1*	.	.	.
14	37791992	A	G	intronic	*MIPOL1*	.	.	.
14	37792544	A	C	intronic	*MIPOL1*	.	.	.
14	37823281	G	A	intronic	*MIPOL1*	.	.	.
14	37830119	A	G	intronic	*MIPOL1*	.	.	.
14	37830362	A	G	intronic	*MIPOL1*	.	.	.
14	37830363	G	T	intronic	*MIPOL1*	.	.	.
14	37956304	T	G	intronic	*MIPOL1*	.	.	.
20	61190339	T	C	intergenic	*MIR1-1HG,SLCO4A1*	.	.	.
11	122114906	G	T	intergenic	*MIR100HG,UBASH3B*	.	.	.
11	122133166	C	T	intergenic	*MIR100HG,UBASH3B*	.	.	.
11	122236496	GCACACACA	G	intergenic	*MIR100HG,UBASH3B*	.	.	.
11	122260775	T	C	intergenic	*MIR100HG,UBASH3B*	.	.	.
11	122316273	G	A	intergenic	*MIR100HG,UBASH3B*	.	.	.
X	133327575	C	T	intergenic	*MIR106A,CCDC160*	.	.	.
14	101495605	G	A	upstream	*MIR1193,MIR494*	.	.	.
6	156333614	A	T	intergenic	*MIR1202,ARID1B*	.	.	.
6	156928516	C	T	intergenic	*MIR1202,ARID1B*	.	.	.
6	156928671	T	C	intergenic	*MIR1202,ARID1B*	.	.	.
6	156987706	C	CT	intergenic	*MIR1202,ARID1B*	.	.	.
6	156989998	C	A	intergenic	*MIR1202,ARID1B*	.	.	.
6	156990120	A	T	intergenic	*MIR1202,ARID1B*	.	.	.

8	129325554	A	C	intergenic	*MIR1208,LINC00824*	.	.	.
8	129353578	CAGAG	C	intergenic	*MIR1208,LINC00824*	.	.	.
8	129360844	C	T	intergenic	*MIR1208,LINC00824*	.	.	.
8	9781055	A	G	intergenic	*MIR124-1,MSRA*	.	.	.
8	9806278	G	A	intergenic	*MIR124-1,MSRA*	.	.	.
8	9851486	A	G	intergenic	*MIR124-1,MSRA*	.	.	.
8	65339033	A	G	intergenic	*MIR124-2HG,LOC401463*	.	.	.
8	65317134	A	T	intergenic	*MIR124-2HG,LOC401463*	.	.	.
8	65448045	A	G	intergenic	*MIR124-2HG,LOC401463*	.	.	.
8	65313338	G	A	intergenic	*MIR124-2HG,LOC401463*	.	.	.
8	65316376	T	C	intergenic	*MIR124-2HG,LOC401463*	.	.	.
8	65475831	T	C	intergenic	*MIR124-2HG,LOC401463*	.	.	.
8	65477081	C	A	intergenic	*MIR124-2HG,LOC401463*	.	.	.
8	65477108	C	G	intergenic	*MIR124-2HG,LOC401463*	.	.	.
8	65478335	CT	C	intergenic	*MIR124-2HG,LOC401463*	.	.	.
8	65478427	T	A	intergenic	*MIR124-2HG,LOC401463*	.	.	.
20	61808952	AACG	A	upstream	*MIR124-3*	.	.	.
2	177487954	T	C	intergenic	*MIR1246,LINC01116*	.	.	.
4	36490286	G	A	intergenic	*MIR1255B1,MIR4801*	.	.	.
4	36605734	C	A	intergenic	*MIR1255B1,MIR4801*	.	.	.
4	36847211	G	T	intergenic	*MIR1255B1,MIR4801*	.	.	.

4	37112266	C	T	intergenic	*MIR1255B1,MIR4801*	.	.	.
4	36805608	T	G	intergenic	*MIR1255B1,MIR4801*	.	.	.
4	36784970	C	T	intergenic	*MIR1255B1,MIR4801*	.	.	.
4	37170244	G	A	intergenic	*MIR1255B1,MIR4801*	.	.	.
4	37219705	A	C	intergenic	*MIR1255B1,MIR4801*	.	.	.
4	36584309	G	C	intergenic	*MIR1255B1,MIR4801*	.	.	.
4	36513149	G	A	intergenic	*MIR1255B1,MIR4801*	.	.	.
4	36590837	T	G	intergenic	*MIR1255B1,MIR4801*	.	.	.
4	36590838	A	G	intergenic	*MIR1255B1,MIR4801*	.	.	.
4	36660662	C	A	intergenic	*MIR1255B1,MIR4801*	.	.	.
4	36780944	G	T	intergenic	*MIR1255B1,MIR4801*	.	.	.
4	36861040	T	G	intergenic	*MIR1255B1,MIR4801*	.	.	.
4	36889056	A	G	intergenic	*MIR1255B1,MIR4801*	.	.	.
4	36938500	A	G	intergenic	*MIR1255B1,MIR4801*	.	.	.
4	37030906	C	G	intergenic	*MIR1255B1,MIR4801*	.	.	.
4	37030907	A	G	intergenic	*MIR1255B1,MIR4801*	.	.	.
20	60541983	G	A	intergenic	*MIR1257,TAF4*	.	.	.
3	164391097	A	G	intergenic	*MIR1263,LINC01324*	.	.	.
3	163984492	G	A	intergenic	*MIR1263,LINC01324*	.	.	.

3	164367494	G	T	intergenic	*MIR1263,LINC01324*	.	.	.
8	37213514	T	TATAGATGG	ncRNA_intronic	*MIR1268A*	.	.	.
19	3763947	G	A	ncRNA_intronic	*MIR1268A*	.	.	.
19	3791561	G	A	ncRNA_intronic	*MIR1268A*	.	.	.
8	37139607	T	C	ncRNA_intronic	*MIR1268A*	.	.	.
8	37140899	G	A	ncRNA_intronic	*MIR1268A*	.	.	.
8	37161875	CTGTGTGTGTG	C	ncRNA_intronic	*MIR1268A*	.	.	.
4	67145621	TAAGATATATATTTCTATTTGAAATATAAGATATATATTTCTATTTGAAATATAAGATATATATTTCTATTTGA	T	intergenic	*MIR1269A,LOC101927237*	.	.	.
4	67363357	G	GTA	intergenic	*MIR1269A,LOC101927237*	.	.	.
4	67448882	C	T	intergenic	*MIR1269A,LOC101927237*	.	.	.
4	67314255	A	G	intergenic	*MIR1269A,LOC101927237*	.	.	.
4	67747550	GCTCT	G	intergenic	*MIR1269A,LOC101927237*	.	.	.
4	67563147	C	G	intergenic	*MIR1269A,LOC101927237*	.	.	.
4	67279349	C	A	intergenic	*MIR1269A,LOC101927237*	.	.	.
4	67324869	A	G	intergenic	*MIR1269A,LOC101927237*	.	.	.
4	67536476	C	T	intergenic	*MIR1269A,LOC101927237*	.	.	.
4	67833923	A	G	intergenic	*MIR1269A,LOC101927237*	.	.	.
4	67835098	T	G	intergenic	*MIR1269A,LOC101927237*	.	.	.

4	67981320	T	C	intergenic	*MIR1269A,LOC101927237*	.	.	.
4	67982673	A	G	intergenic	*MIR1269A,LOC101927237*	.	.	.
4	68120043	A	C	intergenic	*MIR1269A,LOC101927237*	.	.	.
4	68163358	A	G	intergenic	*MIR1269A,LOC101927237*	.	.	.
4	68276155	C	A	intergenic	*MIR1269A,LOC101927237*	.	.	.
6	155333708	G	A	intergenic	*MIR1273C,TIAM2*	.	.	.
4	39507955	G	A	ncRNA_intronic	*MIR1273H*	.	.	.
4	39157515	G	A	ncRNA_intronic	*MIR1273H*	.	.	.
4	39199024	A	T	ncRNA_intronic	*MIR1273H*	.	.	.
4	39199025	A	G	ncRNA_intronic	*MIR1273H*	.	.	.
4	39253174	C	T	ncRNA_intronic	*MIR1273H*	.	.	.
4	39253176	A	T	ncRNA_intronic	*MIR1273H*	.	.	.
4	39485910	CACACACACACACACAA	C	ncRNA_intronic	*MIR1273H*	.	.	.
11	43678669	T	C	intergenic	*MIR129-2,HSD17B12*	.	.	.
13	55603846	G	A	intergenic	*MIR1297,MIR5007*	.	.	.
13	55044827	A	T	intergenic	*MIR1297,MIR5007*	.	.	.
13	55733294	GATAT	G	intergenic	*MIR1297,MIR5007*	.	.	.
13	55662614	G	A	intergenic	*MIR1297,MIR5007*	.	.	.
13	55399488	A	G	intergenic	*MIR1297,MIR5007*	.	.	.
13	55403370	A	G	intergenic	*MIR1297,MIR5007*	.	.	.
13	54977612	A	G	intergenic	*MIR1297,MIR5007*	.	.	.
13	55078494	A	T	intergenic	*MIR1297,MIR5007*	.	.	.
13	55142807	A	G	intergenic	*MIR1297,MIR5007*	.	.	.
13	55647176	G	A	intergenic	*MIR1297,MIR5007*	.	.	.
13	55228723	T	A	intergenic	*MIR1297,MIR5007*	.	.	.
13	55288568	C	T	intergenic	*MIR1297,MIR5007*	.	.	.
13	55291343	A	C	intergenic	*MIR1297,MIR5007*	.	.	.
13	55315413	G	A	intergenic	*MIR1297,MIR5007*	.	.	.
13	55354716	C	T	intergenic	*MIR1297,MIR5007*	.	.	.
13	55397705	C	A	intergenic	*MIR1297,MIR5007*	.	.	.

13	55397706	C	T	intergenic	*MIR1297,MIR5007*	.	.	.
13	55496162	A	G	intergenic	*MIR1297,MIR5007*	.	.	.
13	55496164	T	C	intergenic	*MIR1297,MIR5007*	.	.	.
2	208228321	T	C	intergenic	*MIR1302-4,CREB1*	.	.	.
2	208216852	CGTGTGTG TGT	C	intergenic	*MIR1302-4,CREB1*	.	.	.
2	208242267	T	G	intergenic	*MIR1302-4,CREB1*	.	.	.
2	208327316	T	C	intergenic	*MIR1302-4,CREB1*	.	.	.
2	208327317	G	A	intergenic	*MIR1302-4,CREB1*	.	.	.
3	75688776	G	A	intergenic	*MIR1324,FRG2EP*	.	.	.
12	98092026	C	A	intergenic	*MIR135A2,LOC643711*	.	.	.
12	97976834	G	A	intergenic	*MIR135A2,LOC643711*	.	.	.
12	98069794	C	T	intergenic	*MIR135A2,LOC643711*	.	.	.
12	98071292	A	C	intergenic	*MIR135A2,LOC643711*	.	.	.
12	98071878	A	G	intergenic	*MIR135A2,LOC643711*	.	.	.
12	98086343	A	G	intergenic	*MIR135A2,LOC643711*	.	.	.
3	44190831	A	G	intergenic	*MIR138-1,TOPAZ1*	.	.	.
X	63011020	G	T	intergenic	*MIR1468,AMER1*	.	.	.
X	63187064	T	C	intergenic	*MIR1468,AMER1*	.	.	.
X	63080174	C	A	intergenic	*MIR1468,AMER1*	.	.	.
X	63248027	G	C	intergenic	*MIR1468,AMER1*	.	.	.
X	63121983	A	G	intergenic	*MIR1468,AMER1*	.	.	.
X	63064224	T	A	intergenic	*MIR1468,AMER1*	.	.	.
X	63101870	A	G	intergenic	*MIR1468,AMER1*	.	.	.
X	63122475	G	A	intergenic	*MIR1468,AMER1*	.	.	.
X	63126875	T	A	intergenic	*MIR1468,AMER1*	.	.	.
X	63190977	G	C	intergenic	*MIR1468,AMER1*	.	.	.
X	63191910	G	A	intergenic	*MIR1468,AMER1*	.	.	.
X	63234568	T	C	intergenic	*MIR1468,AMER1*	.	.	.
X	63234569	G	A	intergenic	*MIR1468,AMER1*	.	.	.
X	63278432	A	G	intergenic	*MIR1468,AMER1*	.	.	.
X	63279777	C	G	intergenic	*MIR1468,AMER1*	.	.	.

X	63280107	G	T	intergenic	*MIR1468,AMER1*	.	.	.
X	63301551	T	C	intergenic	*MIR1468,AMER1*	.	.	.
X	63308007	T	G	intergenic	*MIR1468,AMER1*	.	.	.
X	63308009	T	C	intergenic	*MIR1468,AMER1*	.	.	.
X	63308632	A	G	intergenic	*MIR1468,AMER1*	.	.	.
X	63331916	T	A	intergenic	*MIR1468,AMER1*	.	.	.
X	63353636	C	T	intergenic	*MIR1468,AMER1*	.	.	.
X	63355511	A	AT	intergenic	*MIR1468,AMER1*	.	.	.
X	63364310	T	C	intergenic	*MIR1468,AMER1*	.	.	.
X	63376946	T	G	intergenic	*MIR1468,AMER1*	.	.	.
X	63376947	T	G	intergenic	*MIR1468,AMER1*	.	.	.
X	63377223	G	A	intergenic	*MIR1468,AMER1*	.	.	.
X	63377596	T	C	intergenic	*MIR1468,AMER1*	.	.	.
9	123035444	C	T	intergenic	*MIR147A,CDK5RAP2*	.	.	.
9	123035446	G	C	intergenic	*MIR147A,CDK5RAP2*	.	.	.
7	26133347	TAC	T	intergenic	*MIR148A,NFE2L3*	.	.	.
7	26054479	G	A	intergenic	*MIR148A,NFE2L3*	.	.	.
7	26139330	G	A	intergenic	*MIR148A,NFE2L3*	.	.	.
1	198822511	GTATA	G	ncRNA_intronic	*MIR181A1HG*	.	.	.
1	198899540	G	A	ncRNA_intronic	*MIR181A1HG*	.	.	.
1	198931453	A	G	intergenic	*MIR181A1HG,LINC01222*	.	.	.
1	198933568	A	T	intergenic	*MIR181A1HG,LINC01222*	.	.	.
1	198933569	A	T	intergenic	*MIR181A1HG,LINC01222*	.	.	.
12	100590220	A	T	intergenic	*MIR1827,ACTR6*	.	.	.
4	117679616	A	T	intergenic	*MIR1973,TRAM1L1*	.	.	.
4	117712242	T	C	intergenic	*MIR1973,TRAM1L1*	.	.	.
4	117712243	G	T	intergenic	*MIR1973,TRAM1L1*	.	.	.
4	117325760	C	CA	intergenic	*MIR1973,TRAM1L1*	.	.	.

4	117355370	C	G	intergenic	*MIR1973,TRAM1L1*	.	.	.
4	117355372	A	T	intergenic	*MIR1973,TRAM1L1*	.	.	.
4	117362003	C	G	intergenic	*MIR1973,TRAM1L1*	.	.	.
4	117363092	T	C	intergenic	*MIR1973,TRAM1L1*	.	.	.
4	117698341	G	A	intergenic	*MIR1973,TRAM1L1*	.	.	.
4	117699786	A	C	intergenic	*MIR1973,TRAM1L1*	.	.	.
4	117730188	C	T	intergenic	*MIR1973,TRAM1L1*	.	.	.
4	117741359	A	G	intergenic	*MIR1973,TRAM1L1*	.	.	.
4	117770220	C	G	intergenic	*MIR1973,TRAM1L1*	.	.	.
4	117809290	G	A	intergenic	*MIR1973,TRAM1L1*	.	.	.
4	117859334	AGG	A	intergenic	*MIR1973,TRAM1L1*	.	.	.
4	117953949	G	A	intergenic	*MIR1973,TRAM1L1*	.	.	.
10	135074066	G	GAA	intergenic	*MIR202HG,ADAM8*	.	.	.
10	135074067	T	C	intergenic	*MIR202HG,ADAM8*	.	.	.
8	75650209	TTATC	T	ncRNA_intronic	*MIR2052HG*	.	.	.
8	75611651	GTATATA	G	ncRNA_intronic	*MIR2052HG*	.	.	.
8	75656626	C	T	ncRNA_intronic	*MIR2052HG*	.	.	.
8	75656902	C	A	ncRNA_intronic	*MIR2052HG*	.	.	.
8	75734031	A	T	intergenic	*MIR2052HG,PI15*	.	.	.
4	128040995	A	AATAT	intergenic	*MIR2054,INTU*	.	.	.
4	127563466	C	G	intergenic	*MIR2054,INTU*	.	.	.
4	126786128	G	A	intergenic	*MIR2054,INTU*	.	.	.
4	126786129	G	T	intergenic	*MIR2054,INTU*	.	.	.
4	126803286	A	C	intergenic	*MIR2054,INTU*	.	.	.
4	126816679	T	C	intergenic	*MIR2054,INTU*	.	.	.
4	126818510	C	A	intergenic	*MIR2054,INTU*	.	.	.

4	126818512	C	A	intergenic	*MIR2054,INTU*	.	.	.
4	126850888	C	A	intergenic	*MIR2054,INTU*	.	.	.
4	126954169	G	A	intergenic	*MIR2054,INTU*	.	.	.
4	126955025	A	C	intergenic	*MIR2054,INTU*	.	.	.
4	127005282	T	C	intergenic	*MIR2054,INTU*	.	.	.
4	127005283	G	A	intergenic	*MIR2054,INTU*	.	.	.
4	127115682	A	G	intergenic	*MIR2054,INTU*	.	.	.
4	127115683	C	T	intergenic	*MIR2054,INTU*	.	.	.
4	127127796	C	CTT	intergenic	*MIR2054,INTU*	.	.	.
4	127477178	T	C	intergenic	*MIR2054,INTU*	.	.	.
4	127477761	A	G	intergenic	*MIR2054,INTU*	.	.	.
4	127595727	T	C	intergenic	*MIR2054,INTU*	.	.	.
4	127596008	A	T	intergenic	*MIR2054,INTU*	.	.	.
4	127783117	A	C	intergenic	*MIR2054,INTU*	.	.	.
4	127841484	A	G	intergenic	*MIR2054,INTU*	.	.	.
4	128144126	C	CTT	intergenic	*MIR2054,INTU*	.	.	.
4	128145425	C	T	intergenic	*MIR2054,INTU*	.	.	.
4	128185297	A	G	intergenic	*MIR2054,INTU*	.	.	.
4	126491351	A	G	intergenic	*MIR2054,NONE*	.	.	.
4	126491353	G	A	intergenic	*MIR2054,NONE*	.	.	.
1	209696600	A	G	intergenic	*MIR205HG,CAMK1G*	.	.	.
1	209748845	A	ATGC	intergenic	*MIR205HG,CAMK1G*	.	.	.
1	209743327	TCACACA	T	intergenic	*MIR205HG,CAMK1G*	.	.	.
1	209627004	C	T	intergenic	*MIR205HG,CAMK1G*	.	.	.
17	57926930	G	A	intergenic	*MIR21,TUBD1*	.	.	.
6	98725162	C	T	intergenic	*MIR2113,POU3F2*	.	.	.
6	99130455	GGTGTGTGT	G	intergenic	*MIR2113,POU3F2*	.	.	.
6	99030250	A	G	intergenic	*MIR2113,POU3F2*	.	.	.
6	98672379	C	CAAAAAAAA	intergenic	*MIR2113,POU3F2*	.	.	.
6	99046477	T	C	intergenic	*MIR2113,POU3F2*	.	.	.
6	98508904	T	C	intergenic	*MIR2113,POU3F2*	.	.	.
6	98508905	G	T	intergenic	*MIR2113,POU3F2*	.	.	.
6	98510107	G	A	intergenic	*MIR2113,POU3F2*	.	.	.

6	98510476	G	A	intergenic	*MIR2113,POU3F2*	.	.	.
6	98533884	A	G	intergenic	*MIR2113,POU3F2*	.	.	.
6	98682132	G	A	intergenic	*MIR2113,POU3F2*	.	.	.
6	98682960	A	C	intergenic	*MIR2113,POU3F2*	.	.	.
6	98756613	C	T	intergenic	*MIR2113,POU3F2*	.	.	.
6	98775667	GTATA	G	intergenic	*MIR2113,POU3F2*	.	.	.
6	98837302	C	T	intergenic	*MIR2113,POU3F2*	.	.	.
6	98839179	G	A	intergenic	*MIR2113,POU3F2*	.	.	.
6	98861910	T	C	intergenic	*MIR2113,POU3F2*	.	.	.
6	98872398	G	A	intergenic	*MIR2113,POU3F2*	.	.	.
6	98929301	A	G	intergenic	*MIR2113,POU3F2*	.	.	.
6	98968268	A	C	intergenic	*MIR2113,POU3F2*	.	.	.
6	98968269	G	A	intergenic	*MIR2113,POU3F2*	.	.	.
6	99155560	C	T	intergenic	*MIR2113,POU3F2*	.	.	.
6	99156095	G	A	intergenic	*MIR2113,POU3F2*	.	.	.
2	56211047	CATAT	C	ncRNA_intronic	*MIR217HG*	.	.	.
2	56385145	T	TATAC	intergenic	*MIR217HG,LOC100129434*	.	.	.
2	56313867	G	GAGA	intergenic	*MIR217HG,LOC100129434*	.	.	.
2	56313868	T	TCGG	intergenic	*MIR217HG,LOC100129434*	.	.	.
2	56323332	A	G	intergenic	*MIR217HG,LOC100129434*	.	.	.
2	56324078	G	A	intergenic	*MIR217HG,LOC100129434*	.	.	.
2	56365984	T	C	intergenic	*MIR217HG,LOC100129434*	.	.	.
2	56365985	G	A	intergenic	*MIR217HG,LOC100129434*	.	.	.
X	45702342	G	T	intergenic	*MIR222,LOC401585*	.	.	.
X	45629386	T	G	intergenic	*MIR222,LOC401585*	.	.	.
19	13951385	G	A	intergenic	*MIR23A,MIR181C*	.	.	.
11	7264558	G	C	intergenic	*MIR302E,SYT9*	.	.	.
18	28307348	T	C	intergenic	*MIR302F,DSC3*	.	.	.
18	28134396	CCTCT	C	intergenic	*MIR302F,DSC3*	.	.	.

18	27906072	C	A	intergenic	*MIR302F,DSC3*	.	.	.
18	28438393	ATATCC	A	intergenic	*MIR302F,DSC3*	.	.	.
18	28254106	C	CTA	intergenic	*MIR302F,DSC3*	.	.	.
18	28315705	G	A	intergenic	*MIR302F,DSC3*	.	.	.
18	28315706	C	G	intergenic	*MIR302F,DSC3*	.	.	.
18	28424728	T	TG	intergenic	*MIR302F,DSC3*	.	.	.
18	28466837	G	A	intergenic	*MIR302F,DSC3*	.	.	.
18	28558245	A	C	intergenic	*MIR302F,DSC3*	.	.	.
8	135820418	T	TGCACAC	intergenic	*MIR30D,NCRNA00250*	.	.	.
9	114797551	C	T	ncRNA_intronic	*MIR3134*	.	.	.
5	154052010	A	G	intergenic	*MIR3141,MIR1303*	.	.	.
5	153995880	G	A	intergenic	*MIR3141,MIR1303*	.	.	.
6	27166422	G	A	intergenic	*MIR3143,PRSS16*	.	.	.
6	121037572	A	G	intergenic	*MIR3144,TBC1D32*	.	.	.
6	120772176	C	T	intergenic	*MIR3144,TBC1D32*	.	.	.
6	120496457	A	G	intergenic	*MIR3144,TBC1D32*	.	.	.
6	120526726	C	T	intergenic	*MIR3144,TBC1D32*	.	.	.
6	120565422	C	G	intergenic	*MIR3144,TBC1D32*	.	.	.
6	120573582	T	G	intergenic	*MIR3144,TBC1D32*	.	.	.
6	120573583	A	C	intergenic	*MIR3144,TBC1D32*	.	.	.
6	120587326	A	G	intergenic	*MIR3144,TBC1D32*	.	.	.
6	120687176	G	C	intergenic	*MIR3144,TBC1D32*	.	.	.
6	120841554	A	T	intergenic	*MIR3144,TBC1D32*	.	.	.
6	121074567	A	G	intergenic	*MIR3144,TBC1D32*	.	.	.
6	121332667	G	A	intergenic	*MIR3144,TBC1D32*	.	.	.
8	77886160	G	A	intergenic	*MIR3149,PEX2*	.	.	.
21	14881210	A	T	intergenic	*MIR3156-3,LOC102724188*	.	.	.
11	87976421	T	TAACA	intergenic	*MIR3166,CTSC*	.	.	.
11	87984579	GTATA	G	intergenic	*MIR3166,CTSC*	.	.	.
11	87926916	G	A	intergenic	*MIR3166,CTSC*	.	.	.
11	87976875	T	C	intergenic	*MIR3166,CTSC*	.	.	.
13	61960365	C	A	intergenic	*MIR3169,PCDH20*	.	.	.
13	61941949	T	C	intergenic	*MIR3169,PCDH20*	.	.	.
13	61842811	A	C	intergenic	*MIR3169,PCDH20*	.	.	.
13	61899853	G	C	intergenic	*MIR3169,PCDH20*	.	.	.
13	61901656	G	A	intergenic	*MIR3169,PCDH20*	.	.	.

13	61902587	C	T	intergenic	*MIR3169,PCDH20*	.	.	.
16	15393722	A	T	intergenic	*MIR3180-4,NPIPA5*	.	.	.
9	21698799	C	CG	intergenic	*MIR31HG,MTAP*	.	.	.
9	21587542	A	G	intergenic	*MIR31HG,MTAP*	.	.	.
9	21760777	G	C	intergenic	*MIR31HG,MTAP*	.	.	.
9	21785490	C	CTGA	intergenic	*MIR31HG,MTAP*	.	.	.
1	117215247	AAGAGAG	A	downstream	*MIR320B1*	.	.	.
X	140080246	C	T	intergenic	*MIR320D2,SPANXB1*	.	.	.
X	140040303	G	T	intergenic	*MIR320D2,SPANXB1*	.	.	.
X	140070341	G	C	intergenic	*MIR320D2,SPANXB1*	.	.	.
X	75911731	C	A	ncRNA_intronic	*MIR325HG*	.	.	.
X	75929997	A	T	ncRNA_intronic	*MIR325HG*	.	.	.
X	75914724	C	G	ncRNA_intronic	*MIR325HG*	.	.	.
X	75884117	A	T	ncRNA_intronic	*MIR325HG*	.	.	.
X	75896272	A	C	ncRNA_intronic	*MIR325HG*	.	.	.
X	75896273	T	C	ncRNA_intronic	*MIR325HG*	.	.	.
X	75896414	T	G	ncRNA_intronic	*MIR325HG*	.	.	.
X	75897755	A	G	ncRNA_intronic	*MIR325HG*	.	.	.
X	75943061	G	C	ncRNA_intronic	*MIR325HG*	.	.	.
X	75943571	A	G	ncRNA_intronic	*MIR325HG*	.	.	.
X	75979626	T	A	ncRNA_intronic	*MIR325HG*	.	.	.
X	75995605	G	T	ncRNA_intronic	*MIR325HG*	.	.	.
X	75996758	A	G	ncRNA_intronic	*MIR325HG*	.	.	.
X	76004789	G	C	ncRNA_intronic	*MIR325HG*	.	.	.
X	76005953	T	A	ncRNA_intronic	*MIR325HG*	.	.	.
X	76038348	G	A	ncRNA_intronic	*MIR325HG*	.	.	.
X	76065336	G	A	ncRNA_intronic	*MIR325HG*	.	.	.
X	76068385	T	C	ncRNA_intronic	*MIR325HG*	.	.	.
X	76106386	T	C	ncRNA_intronic	*MIR325HG*	.	.	.
X	76107868	G	A	ncRNA_intronic	*MIR325HG*	.	.	.
X	76111808	A	G	ncRNA_intronic	*MIR325HG*	.	.	.
X	76119533	A	C	ncRNA_intronic	*MIR325HG*	.	.	.
X	76148593	G	T	ncRNA_intronic	*MIR325HG*	.	.	.
X	76157697	T	G	ncRNA_intronic	*MIR325HG*	.	.	.

X	76160210	T	C	ncRNA_intronic	*MIR325HG*	.	.	.
X	76174219	T	A	ncRNA_intronic	*MIR325HG*	.	.	.
X	76174826	T	C	ncRNA_intronic	*MIR325HG*	.	.	.
X	76175023	C	A	ncRNA_intronic	*MIR325HG*	.	.	.
X	76202235	A	G	ncRNA_intronic	*MIR325HG*	.	.	.
X	76540800	A	G	intergenic	*MIR325HG,FGF16*	.	.	.
X	76288423	T	A	intergenic	*MIR325HG,FGF16*	.	.	.
X	76249220	T	C	intergenic	*MIR325HG,FGF16*	.	.	.
X	76249222	C	T	intergenic	*MIR325HG,FGF16*	.	.	.
X	76259429	G	A	intergenic	*MIR325HG,FGF16*	.	.	.
X	76260055	G	A	intergenic	*MIR325HG,FGF16*	.	.	.
X	76271662	C	A	intergenic	*MIR325HG,FGF16*	.	.	.
X	76312829	T	A	intergenic	*MIR325HG,FGF16*	.	.	.
X	76357435	G	A	intergenic	*MIR325HG,FGF16*	.	.	.
X	76373140	GA	G	intergenic	*MIR325HG,FGF16*	.	.	.
X	76407731	G	A	intergenic	*MIR325HG,FGF16*	.	.	.
X	76407984	A	T	intergenic	*MIR325HG,FGF16*	.	.	.
X	76407985	G	A	intergenic	*MIR325HG,FGF16*	.	.	.
X	76420564	A	G	intergenic	*MIR325HG,FGF16*	.	.	.
X	76423110	A	T	intergenic	*MIR325HG,FGF16*	.	.	.
X	76429778	G	A	intergenic	*MIR325HG,FGF16*	.	.	.
X	76448414	T	C	intergenic	*MIR325HG,FGF16*	.	.	.
X	76451367	A	T	intergenic	*MIR325HG,FGF16*	.	.	.
X	76481754	T	C	intergenic	*MIR325HG,FGF16*	.	.	.
X	76508627	A	C	intergenic	*MIR325HG,FGF16*	.	.	.
X	76511325	C	G	intergenic	*MIR325HG,FGF16*	.	.	.
X	76541526	T	C	intergenic	*MIR325HG,FGF16*	.	.	.
X	76542061	C	T	intergenic	*MIR325HG,FGF16*	.	.	.
X	76549969	G	A	intergenic	*MIR325HG,FGF16*	.	.	.
X	76557503	C	G	intergenic	*MIR325HG,FGF16*	.	.	.
X	76559619	T	G	intergenic	*MIR325HG,FGF16*	.	.	.
X	76600134	C	A	intergenic	*MIR325HG,FGF16*	.	.	.
X	76615111	A	G	intergenic	*MIR325HG,FGF16*	.	.	.
X	76626585	G	A	intergenic	*MIR325HG,FGF16*	.	.	.
X	76627240	T	C	intergenic	*MIR325HG,FGF16*	.	.	.
8	27578135	C	CCA	intergenic	*MIR3622B,CCDC25*	.	.	.

21	9825913	C	A	ncRNA_exonic	*MIR3648-1,MIR3648-2*	.	.	.
1	38562823	CAGAG	C	intergenic	*MIR3659,LINC01343*	.	.	.
17	29992807	T	A	intergenic	*MIR365B,COPRS*	.	.	.
17	29992808	T	A	intergenic	*MIR365B,COPRS*	.	.	.
17	29992993	C	A	intergenic	*MIR365B,COPRS*	.	.	.
17	29993691	A	T	intergenic	*MIR365B,COPRS*	.	.	.
17	29995187	G	T	intergenic	*MIR365B,COPRS*	.	.	.
17	30109070	T	C	intergenic	*MIR365B,COPRS*	.	.	.
17	30109838	G	T	intergenic	*MIR365B,COPRS*	.	.	.
17	30109839	T	C	intergenic	*MIR365B,COPRS*	.	.	.
17	30144523	T	C	intergenic	*MIR365B,COPRS*	.	.	.
17	30144525	A	C	intergenic	*MIR365B,COPRS*	.	.	.
5	89331005	A	G	intergenic	*MIR3660,LINC01339*	.	.	.
5	89355023	C	T	intergenic	*MIR3660,LINC01339*	.	.	.
5	89355024	A	G	intergenic	*MIR3660,LINC01339*	.	.	.
5	89371852	C	T	intergenic	*MIR3660,LINC01339*	.	.	.
5	89400577	A	C	intergenic	*MIR3660,LINC01339*	.	.	.
5	89448318	T	A	intergenic	*MIR3660,LINC01339*	.	.	.
22	49956870	T	C	intergenic	*MIR3667,C22orf34*	.	.	.
6	140532774	TTGTGTG	T	intergenic	*MIR3668,MIR4465*	.	.	.
6	140555327	A	G	intergenic	*MIR3668,MIR4465*	.	.	.
6	140556847	T	C	intergenic	*MIR3668,MIR4465*	.	.	.
6	140690452	G	A	intergenic	*MIR3668,MIR4465*	.	.	.
6	140694403	G	A	intergenic	*MIR3668,MIR4465*	.	.	.
6	140934990	G	C	intergenic	*MIR3668,MIR4465*	.	.	.
X	120597826	A	G	intergenic	*MIR3672,GRIA3*	.	.	.
X	120781638	T	A	intergenic	*MIR3672,GRIA3*	.	.	.
X	122161740	G	T	intergenic	*MIR3672,GRIA3*	.	.	.
X	122238524	A	G	intergenic	*MIR3672,GRIA3*	.	.	.
X	122261292	G	A	intergenic	*MIR3672,GRIA3*	.	.	.

X	121276459	A	G	intergenic	*MIR3672,GRIA3*	.	.	.
X	121591882	A	G	intergenic	*MIR3672,GRIA3*	.	.	.
X	121835721	TA	T	intergenic	*MIR3672,GRIA3*	.	.	.
X	120606852	T	C	intergenic	*MIR3672,GRIA3*	.	.	.
X	120607216	A	G	intergenic	*MIR3672,GRIA3*	.	.	.
X	120668565	C	T	intergenic	*MIR3672,GRIA3*	.	.	.
X	120825220	G	T	intergenic	*MIR3672,GRIA3*	.	.	.
X	120829471	G	T	intergenic	*MIR3672,GRIA3*	.	.	.
X	120954955	C	T	intergenic	*MIR3672,GRIA3*	.	.	.
X	121162945	T	A	intergenic	*MIR3672,GRIA3*	.	.	.
X	121162946	G	T	intergenic	*MIR3672,GRIA3*	.	.	.
X	121197819	G	A	intergenic	*MIR3672,GRIA3*	.	.	.
X	121295503	T	C	intergenic	*MIR3672,GRIA3*	.	.	.
X	121297331	A	G	intergenic	*MIR3672,GRIA3*	.	.	.
X	121346407	G	A	intergenic	*MIR3672,GRIA3*	.	.	.
X	121430268	A	T	intergenic	*MIR3672,GRIA3*	.	.	.
X	121441051	T	C	intergenic	*MIR3672,GRIA3*	.	.	.
X	121530072	A	G	intergenic	*MIR3672,GRIA3*	.	.	.
X	121627726	A	G	intergenic	*MIR3672,GRIA3*	.	.	.
X	121627728	T	A	intergenic	*MIR3672,GRIA3*	.	.	.
X	121628064	G	T	intergenic	*MIR3672,GRIA3*	.	.	.
X	121661184	G	C	intergenic	*MIR3672,GRIA3*	.	.	.
X	121751956	C	T	intergenic	*MIR3672,GRIA3*	.	.	.
X	121956165	T	C	intergenic	*MIR3672,GRIA3*	.	.	.
X	121962089	G	A	intergenic	*MIR3672,GRIA3*	.	.	.
X	121962091	A	T	intergenic	*MIR3672,GRIA3*	.	.	.
X	121987419	T	A	intergenic	*MIR3672,GRIA3*	.	.	.
X	122044836	C	T	intergenic	*MIR3672,GRIA3*	.	.	.
X	122044837	A	G	intergenic	*MIR3672,GRIA3*	.	.	.
X	122049730	T	A	intergenic	*MIR3672,GRIA3*	.	.	.
X	122049731	A	G	intergenic	*MIR3672,GRIA3*	.	.	.
X	122051314	G	A	intergenic	*MIR3672,GRIA3*	.	.	.
X	122052953	T	G	intergenic	*MIR3672,GRIA3*	.	.	.
X	122053804	G	A	intergenic	*MIR3672,GRIA3*	.	.	.
X	122189859	G	A	intergenic	*MIR3672,GRIA3*	.	.	.
X	122202442	T	C	intergenic	*MIR3672,GRIA3*	.	.	.
X	122236024	A	G	intergenic	*MIR3672,GRIA3*	.	.	.
1	17224310	T	G	intergenic	*MIR3675,CROCC*	.	.	.

1	17193078	T	G	intergenic	*MIR3675,CROCC*	.	.	.
2	134936821	A	G	intergenic	*MIR3679,MGAT5*	.	.	.
2	134910025	T	C	intergenic	*MIR3679,MGAT5*	.	.	.
2	12277846	G	T	ncRNA_intronic	*MIR3681HG*	.	.	.
2	12253213	C	T	ncRNA_intronic	*MIR3681HG*	.	.	.
2	12652241	T	G	ncRNA_intronic	*MIR3681HG*	.	.	.
2	12652243	A	G	ncRNA_intronic	*MIR3681HG*	.	.	.
2	12847536	A	T	intergenic	*MIR3681HG,TRIB2*	.	.	.
12	95740208	T	C	intergenic	*MIR3685,METAP2*	.	.	.
12	95784380	A	T	intergenic	*MIR3685,METAP2*	.	.	.
4	160167447	A	G	intergenic	*MIR3688-1,RAPGEF2*	.	.	.
10	132831258	G	T	intergenic	*MIR378C,TCERG1L*	.	.	.
9	94434335	C	T	intergenic	*MIR3910-1,ROR2*	.	.	.
10	59925506	C	T	intergenic	*MIR3924,IPMK*	.	.	.
10	59290707	G	T	intergenic	*MIR3924,IPMK*	.	.	.
10	59194610	G	T	intergenic	*MIR3924,IPMK*	.	.	.
10	59531303	T	C	intergenic	*MIR3924,IPMK*	.	.	.
10	59128081	C	A	intergenic	*MIR3924,IPMK*	.	.	.
10	59176925	T	C	intergenic	*MIR3924,IPMK*	.	.	.
10	59177153	A	G	intergenic	*MIR3924,IPMK*	.	.	.
10	59202997	CCT	C	intergenic	*MIR3924,IPMK*	.	.	.
10	59203950	T	C	intergenic	*MIR3924,IPMK*	.	.	.
10	59208429	G	C	intergenic	*MIR3924,IPMK*	.	.	.
10	59247973	A	C	intergenic	*MIR3924,IPMK*	.	.	.
10	59247974	T	C	intergenic	*MIR3924,IPMK*	.	.	.
10	59249710	T	A	intergenic	*MIR3924,IPMK*	.	.	.
10	59306926	G	C	intergenic	*MIR3924,IPMK*	.	.	.
10	59307754	C	T	intergenic	*MIR3924,IPMK*	.	.	.
10	59348457	G	T	intergenic	*MIR3924,IPMK*	.	.	.
10	59478186	C	G	intergenic	*MIR3924,IPMK*	.	.	.
10	59478188	T	C	intergenic	*MIR3924,IPMK*	.	.	.
10	59480890	A	C	intergenic	*MIR3924,IPMK*	.	.	.
10	59480892	G	A	intergenic	*MIR3924,IPMK*	.	.	.
10	59542911	G	A	intergenic	*MIR3924,IPMK*	.	.	.
10	59656272	T	C	intergenic	*MIR3924,IPMK*	.	.	.
10	59656922	G	C	intergenic	*MIR3924,IPMK*	.	.	.

10	59656923	T	G	intergenic	*MIR3924,IPMK*	.	.	.
10	59693637	A	T	intergenic	*MIR3924,IPMK*	.	.	.
10	59694962	G	A	intergenic	*MIR3924,IPMK*	.	.	.
10	59695085	C	A	intergenic	*MIR3924,IPMK*	.	.	.
10	59736322	T	A	intergenic	*MIR3924,IPMK*	.	.	.
10	59736323	G	A	intergenic	*MIR3924,IPMK*	.	.	.
10	59793228	G	A	intergenic	*MIR3924,IPMK*	.	.	.
10	59793602	C	T	intergenic	*MIR3924,IPMK*	.	.	.
10	59794239	T	C	intergenic	*MIR3924,IPMK*	.	.	.
10	59794240	G	A	intergenic	*MIR3924,IPMK*	.	.	.
10	59794439	A	G	intergenic	*MIR3924,IPMK*	.	.	.
10	59889625	C	T	intergenic	*MIR3924,IPMK*	.	.	.
10	59889626	A	C	intergenic	*MIR3924,IPMK*	.	.	.
X	39785350	G	C	intergenic	*MIR3937,BCOR*	.	.	.
4	185783788	T	C	intergenic	*MIR3945HG,LINC01093*	.	.	.
12	18013070	C	G	intergenic	*MIR3974,RERGL*	.	.	.
12	18132739	G	T	intergenic	*MIR3974,RERGL*	.	.	.
12	18195180	A	G	intergenic	*MIR3974,RERGL*	.	.	.
12	18198810	G	C	intergenic	*MIR3974,RERGL*	.	.	.
12	17935195	A	G	intergenic	*MIR3974,RERGL*	.	.	.
12	17957609	G	A	intergenic	*MIR3974,RERGL*	.	.	.
12	17960084	G	C	intergenic	*MIR3974,RERGL*	.	.	.
12	17976776	C	G	intergenic	*MIR3974,RERGL*	.	.	.
18	5874728	GGTGTGT	G	intergenic	*MIR3976,TMEM200C*	.	.	.
18	5767988	A	T	ncRNA_intronic	*MIR3976HG*	.	.	.
18	5808830	C	T	intergenic	*MIR3976HG,MIR3976*	.	.	.
18	5808831	A	G	intergenic	*MIR3976HG,MIR3976*	.	.	.
5	82143643	C	A	intergenic	*MIR3977,LINC01338*	.	.	.
5	82143644	T	A	intergenic	*MIR3977,LINC01338*	.	.	.
5	82139276	G	T	intergenic	*MIR3977,LINC01338*	.	.	.

5	82140918	A	G	intergenic	*MIR3977,LINC01338*	.	.	.
15	64167491	C	T	intergenic	*MIR422A,DAPK2*	.	.	.
1	37876778	G	T	intergenic	*MIR4255,LINC01137*	.	.	.
1	37729075	CCTCT	C	intergenic	*MIR4255,LINC01137*	.	.	.
1	37678791	A	G	intergenic	*MIR4255,LINC01137*	.	.	.
1	37797956	A	ACTCT	intergenic	*MIR4255,LINC01137*	.	.	.
1	37841370	A	G	intergenic	*MIR4255,LINC01137*	.	.	.
2	12067186	A	C	intergenic	*MIR4262,MIR3681HG*	.	.	.
2	12021958	C	A	intergenic	*MIR4262,MIR3681HG*	.	.	.
2	12025374	T	A	intergenic	*MIR4262,MIR3681HG*	.	.	.
2	12100627	T	G	intergenic	*MIR4262,MIR3681HG*	.	.	.
2	222015133	G	A	intergenic	*MIR4268,EPHA4*	.	.	.
2	221351727	AGAGAG	A	intergenic	*MIR4268,EPHA4*	.	.	.
2	221780039	G	A	intergenic	*MIR4268,EPHA4*	.	.	.
2	221093591	G	T	intergenic	*MIR4268,EPHA4*	.	.	.
2	221401594	TAATATATATATTATAATATATATAATATAATATATAA	T	intergenic	*MIR4268,EPHA4*	.	.	.
2	222086160	T	C	intergenic	*MIR4268,EPHA4*	.	.	.

2	221952627	GTGTGCATGCATATGAACGTATGTACGCACATGTGCGTGCATATGAACGTATGTACGCACA	G	intergenic	*MIR4268,EPHA4*	.	.	.
2	220801446	T	C	intergenic	*MIR4268,EPHA4*	.	.	.
2	221116729	G	A	intergenic	*MIR4268,EPHA4*	.	.	.
2	221247435	T	C	intergenic	*MIR4268,EPHA4*	.	.	.
2	221250202	A	G	intergenic	*MIR4268,EPHA4*	.	.	.
2	221272200	T	C	intergenic	*MIR4268,EPHA4*	.	.	.
2	221401962	T	C	intergenic	*MIR4268,EPHA4*	.	.	.
2	221401963	G	A	intergenic	*MIR4268,EPHA4*	.	.	.
2	221694139	G	A	intergenic	*MIR4268,EPHA4*	.	.	.
2	221955753	C	G	intergenic	*MIR4268,EPHA4*	.	.	.
2	222072093	T	A	intergenic	*MIR4268,EPHA4*	.	.	.
2	222121933	G	T	intergenic	*MIR4268,EPHA4*	.	.	.
4	28822183	G	T	downstream	*MIR4275*	.	.	.
4	30090337	A	T	intergenic	*MIR4275,PCDH7*	.	.	.
4	29106809	CGTGTGTGTGTGT	C	intergenic	*MIR4275,PCDH7*	.	.	.
4	29404924	C	G	intergenic	*MIR4275,PCDH7*	.	.	.
4	29819067	T	C	intergenic	*MIR4275,PCDH7*	.	.	.
4	29435769	CATAT	C	intergenic	*MIR4275,PCDH7*	.	.	.
4	29987297	C	CTCAGGCCC	intergenic	*MIR4275,PCDH7*	.	.	.
4	30620168	CTATATATATATATATATATATATATATA	C	intergenic	*MIR4275,PCDH7*	.	.	.
4	28919561	C	A	intergenic	*MIR4275,PCDH7*	.	.	.
4	29629478	T	A	intergenic	*MIR4275,PCDH7*	.	.	.
4	29879305	C	T	intergenic	*MIR4275,PCDH7*	.	.	.
4	30634430	C	A	intergenic	*MIR4275,PCDH7*	.	.	.
4	30405616	C	A	intergenic	*MIR4275,PCDH7*	.	.	.
4	29987299	C	CAGGCCCT	intergenic	*MIR4275,PCDH7*	.	.	.
4	30675346	A	T	intergenic	*MIR4275,PCDH7*	.	.	.

4	29185432	T	C	intergenic	*MIR4275,PCDH7*	.	.	.
4	30403141	T	A	intergenic	*MIR4275,PCDH7*	.	.	.
4	28854200	G	T	intergenic	*MIR4275,PCDH7*	.	.	.
4	28970060	C	G	intergenic	*MIR4275,PCDH7*	.	.	.
4	29132263	T	G	intergenic	*MIR4275,PCDH7*	.	.	.
4	29148406	A	C	intergenic	*MIR4275,PCDH7*	.	.	.
4	29148407	G	A	intergenic	*MIR4275,PCDH7*	.	.	.
4	29149126	T	A	intergenic	*MIR4275,PCDH7*	.	.	.
4	29233730	T	C	intergenic	*MIR4275,PCDH7*	.	.	.
4	29319246	C	T	intergenic	*MIR4275,PCDH7*	.	.	.
4	29319481	T	G	intergenic	*MIR4275,PCDH7*	.	.	.
4	29380141	T	C	intergenic	*MIR4275,PCDH7*	.	.	.
4	29526259	A	G	intergenic	*MIR4275,PCDH7*	.	.	.
4	29678647	G	C	intergenic	*MIR4275,PCDH7*	.	.	.
4	29679688	A	T	intergenic	*MIR4275,PCDH7*	.	.	.
4	29734640	TTTTTGTTG TTGTTGTTG	T	intergenic	*MIR4275,PCDH7*	.	.	.
4	29823015	T	C	intergenic	*MIR4275,PCDH7*	.	.	.
4	29874476	T	C	intergenic	*MIR4275,PCDH7*	.	.	.
4	29875615	C	G	intergenic	*MIR4275,PCDH7*	.	.	.
4	29927157	A	G	intergenic	*MIR4275,PCDH7*	.	.	.
4	29975464	A	G	intergenic	*MIR4275,PCDH7*	.	.	.
4	30048147	C	G	intergenic	*MIR4275,PCDH7*	.	.	.
4	30079391	A	AT	intergenic	*MIR4275,PCDH7*	.	.	.
4	30253487	A	G	intergenic	*MIR4275,PCDH7*	.	.	.
4	30320014	A	T	intergenic	*MIR4275,PCDH7*	.	.	.
4	30522635	C	T	intergenic	*MIR4275,PCDH7*	.	.	.
4	30626529	C	T	intergenic	*MIR4275,PCDH7*	.	.	.
5	7149085	G	C	intergenic	*MIR4278,MIR4454*	.	.	.
5	7234266	A	G	intergenic	*MIR4278,MIR4454*	.	.	.
5	7158674	G	C	intergenic	*MIR4278,MIR4454*	.	.	.
7	63116029	C	T	intergenic	*MIR4283-2,LINC01005*	.	.	.
7	63359143	G	A	intergenic	*MIR4283-2,LINC01005*	.	.	.
7	63304080	T	A	intergenic	*MIR4283-2,LINC01005*	.	.	.

7	63210211	A	C	intergenic	*MIR4283-2,LINC01005*	.	.	.
7	63433529	C	T	intergenic	*MIR4283-2,LINC01005*	.	.	.
7	63468200	T	A	intergenic	*MIR4283-2,LINC01005*	.	.	.
7	63468711	T	A	intergenic	*MIR4283-2,LINC01005*	.	.	.
7	57083535	T	A	intergenic	*MIR4283-2,ZNF479*	.	.	.
7	57108718	C	A	intergenic	*MIR4283-2,ZNF479*	.	.	.
9	91524152	A	G	intergenic	*MIR4289,C9orf47*	.	.	.
9	92823984	AT	A	intergenic	*MIR4290HG,LINC01508*	.	.	.
9	93035491	G	T	intergenic	*MIR4290HG,LINC01508*	.	.	.
9	96590014	C	A	intergenic	*MIR4291,BARX1*	.	.	.
10	14461715	A	G	intergenic	*MIR4293,MIR1265*	.	.	.
11	11754629	A	G	intergenic	*MIR4299,MIR8070*	.	.	.
11	11759363	AAATAATAAT	A	intergenic	*MIR4299,MIR8070*	.	.	.
11	11713379	C	T	intergenic	*MIR4299,MIR8070*	.	.	.
11	11718576	C	A	intergenic	*MIR4299,MIR8070*	.	.	.
11	11737350	C	T	intergenic	*MIR4299,MIR8070*	.	.	.
11	81987615	A	T	ncRNA_intronic	*MIR4300HG*	.	.	.
11	81829510	C	G	ncRNA_intronic	*MIR4300HG*	.	.	.
11	81597582	A	T	ncRNA_intronic	*MIR4300HG*	.	.	.
11	81738920	C	A	ncRNA_intronic	*MIR4300HG*	.	.	.
11	81948094	T	A	ncRNA_intronic	*MIR4300HG*	.	.	.
11	81614051	T	C	ncRNA_intronic	*MIR4300HG*	.	.	.
11	81833489	C	A	ncRNA_intronic	*MIR4300HG*	.	.	.
11	81989213	A	G	ncRNA_intronic	*MIR4300HG*	.	.	.
11	81991336	A	G	ncRNA_intronic	*MIR4300HG*	.	.	.
11	82055704	T	G	ncRNA_intronic	*MIR4300HG*	.	.	.
11	82056914	C	T	ncRNA_intronic	*MIR4300HG*	.	.	.
11	82056915	A	G	ncRNA_intronic	*MIR4300HG*	.	.	.

11	82435101	T	C	intergenic	*MIR4300HG,FAM181B*	.	.	.
11	82393552	CAGAG	C	intergenic	*MIR4300HG,FAM181B*	.	.	.
11	82124119	TA	T	intergenic	*MIR4300HG,FAM181B*	.	.	.
11	82350344	A	G	intergenic	*MIR4300HG,FAM181B*	.	.	.
11	82228746	T	C	intergenic	*MIR4300HG,FAM181B*	.	.	.
12	98707342	C	T	intergenic	*MIR4303,SLC9A7P1*	.	.	.
12	98586801	A	T	intergenic	*MIR4303,SLC9A7P1*	.	.	.
12	98598449	T	G	intergenic	*MIR4303,SLC9A7P1*	.	.	.
12	98599639	T	A	intergenic	*MIR4303,SLC9A7P1*	.	.	.
12	98706433	C	T	intergenic	*MIR4303,SLC9A7P1*	.	.	.
12	98706946	A	G	intergenic	*MIR4303,SLC9A7P1*	.	.	.
12	98707354	G	A	intergenic	*MIR4303,SLC9A7P1*	.	.	.
12	98775817	C	A	intergenic	*MIR4303,SLC9A7P1*	.	.	.
14	27358846	C	A	ncRNA_intronic	*MIR4307HG*	.	.	.
14	27902095	A	G	intergenic	*MIR4307HG,LINC00645*	.	.	.
14	27437089	G	T	intergenic	*MIR4307HG,LINC00645*	.	.	.
14	27550142	A	C	intergenic	*MIR4307HG,LINC00645*	.	.	.
14	27872403	C	A	intergenic	*MIR4307HG,LINC00645*	.	.	.
14	27486841	A	C	intergenic	*MIR4307HG,LINC00645*	.	.	.

14	27449067	T	A	intergenic	*MIR4307HG,LINC00645*	.	.	.
14	27664030	T	A	intergenic	*MIR4307HG,LINC00645*	.	.	.
14	27851644	C	G	intergenic	*MIR4307HG,LINC00645*	.	.	.
14	27418983	T	C	intergenic	*MIR4307HG,LINC00645*	.	.	.
14	27516269	T	A	intergenic	*MIR4307HG,LINC00645*	.	.	.
14	27581751	A	G	intergenic	*MIR4307HG,LINC00645*	.	.	.
14	27652487	CATACACAT	C	intergenic	*MIR4307HG,LINC00645*	.	.	.
14	27662705	G	A	intergenic	*MIR4307HG,LINC00645*	.	.	.
14	27693812	C	A	intergenic	*MIR4307HG,LINC00645*	.	.	.
14	27826794	C	T	intergenic	*MIR4307HG,LINC00645*	.	.	.
14	27828540	G	C	intergenic	*MIR4307HG,LINC00645*	.	.	.
14	27831280	T	G	intergenic	*MIR4307HG,LINC00645*	.	.	.
14	27831282	G	T	intergenic	*MIR4307HG,LINC00645*	.	.	.
14	27900332	T	A	intergenic	*MIR4307HG,LINC00645*	.	.	.
14	27931160	C	T	intergenic	*MIR4307HG,LINC00645*	.	.	.
14	27962769	G	A	intergenic	*MIR4307HG,LINC00645*	.	.	.
18	35438670	T	C	intergenic	*MIR4318,MIR924HG*	.	.	.
18	36369677	CTATA	C	intergenic	*MIR4318,MIR924HG*	.	.	.
18	35240481	C	A	intergenic	*MIR4318,MIR924HG*	.	.	.

18	35664158	T	G	intergenic	*MIR4318,MIR924HG*	.	.	.
18	35900101	T	A	intergenic	*MIR4318,MIR924HG*	.	.	.
18	36550444	GGATAGATAGATAGATA	G	intergenic	*MIR4318,MIR924HG*	.	.	.
18	35760207	A	G	intergenic	*MIR4318,MIR924HG*	.	.	.
18	35716651	G	T	intergenic	*MIR4318,MIR924HG*	.	.	.
18	35750545	A	T	intergenic	*MIR4318,MIR924HG*	.	.	.
18	35982376	T	C	intergenic	*MIR4318,MIR924HG*	.	.	.
18	36209273	A	G	intergenic	*MIR4318,MIR924HG*	.	.	.
18	36351732	T	C	intergenic	*MIR4318,MIR924HG*	.	.	.
18	36510566	T	G	intergenic	*MIR4318,MIR924HG*	.	.	.
18	36775743	C	T	intergenic	*MIR4318,MIR924HG*	.	.	.
18	36599814	T	A	intergenic	*MIR4318,MIR924HG*	.	.	.
18	36674983	T	A	intergenic	*MIR4318,MIR924HG*	.	.	.
18	36769571	ATGTG	A	intergenic	*MIR4318,MIR924HG*	.	.	.
18	35273684	C	T	intergenic	*MIR4318,MIR924HG*	.	.	.
18	35273686	A	T	intergenic	*MIR4318,MIR924HG*	.	.	.
18	35407589	T	G	intergenic	*MIR4318,MIR924HG*	.	.	.
18	35411095	C	T	intergenic	*MIR4318,MIR924HG*	.	.	.

18	35421857	T	G	intergenic	*MIR4318,MIR924HG*	.	.	.
18	35520273	A	G	intergenic	*MIR4318,MIR924HG*	.	.	.
18	35523450	A	G	intergenic	*MIR4318,MIR924HG*	.	.	.
18	35847054	C	G	intergenic	*MIR4318,MIR924HG*	.	.	.
18	36495824	T	C	intergenic	*MIR4318,MIR924HG*	.	.	.
18	36495884	T	G	intergenic	*MIR4318,MIR924HG*	.	.	.
18	36496780	T	G	intergenic	*MIR4318,MIR924HG*	.	.	.
18	36496781	A	G	intergenic	*MIR4318,MIR924HG*	.	.	.
18	36702689	A	T	intergenic	*MIR4318,MIR924HG*	.	.	.
18	36702792	T	C	intergenic	*MIR4318,MIR924HG*	.	.	.
18	36702793	G	A	intergenic	*MIR4318,MIR924HG*	.	.	.
21	31758782	CAT	C	intergenic	*MIR4327,KRTAP13-1*	.	.	.
X	78157393	T	A	upstream	*MIR4328*	.	.	.
X	78168642	C	T	intergenic	*MIR4328,P2RY10*	.	.	.
X	78162165	G	T	intergenic	*MIR4328,P2RY10*	.	.	.
X	78193473	A	G	intergenic	*MIR4328,P2RY10*	.	.	.
1	5715700	A	G	intergenic	*MIR4417,MIR4689*	.	.	.
12	128731570	C	A	intergenic	*MIR4419B,TMEM132C*	.	.	.
1	55799540	T	C	intergenic	*MIR4422,PLPP3*	.	.	.
1	55760856	T	C	intergenic	*MIR4422,PLPP3*	.	.	.
1	55901132	C	G	intergenic	*MIR4422,PLPP3*	.	.	.
1	55905766	C	T	intergenic	*MIR4422,PLPP3*	.	.	.
1	55945076	G	A	intergenic	*MIR4422,PLPP3*	.	.	.
1	56711143	CT	C	intergenic	*MIR4422,PLPP3*	.	.	.
1	56737296	A	C	intergenic	*MIR4422,PLPP3*	.	.	.

16	61297459	C	A	intergenic	*MIR4426,CDH8*	.	.	.
16	61190091	G	A	intergenic	*MIR4426,CDH8*	.	.	.
16	61496084	C	T	intergenic	*MIR4426,CDH8*	.	.	.
16	61496586	T	C	intergenic	*MIR4426,CDH8*	.	.	.
16	61516453	G	A	intergenic	*MIR4426,CDH8*	.	.	.
1	192748149	G	GTATATA	intergenic	*MIR4426,RGS2*	.	.	.
1	192749489	T	C	intergenic	*MIR4426,RGS2*	.	.	.
2	53890204	A	G	intergenic	*MIR4431,ASB3*	.	.	.
2	53093092	C	T	intergenic	*MIR4431,ASB3*	.	.	.
2	53278337	C	T	intergenic	*MIR4431,ASB3*	.	.	.
2	53336911	C	G	intergenic	*MIR4431,ASB3*	.	.	.
2	53348545	A	G	intergenic	*MIR4431,ASB3*	.	.	.
2	53477183	C	A	intergenic	*MIR4431,ASB3*	.	.	.
2	53024891	C	T	intergenic	*MIR4431,ASB3*	.	.	.
2	53024892	C	G	intergenic	*MIR4431,ASB3*	.	.	.
2	53135138	G	A	intergenic	*MIR4431,ASB3*	.	.	.
2	53150287	G	A	intergenic	*MIR4431,ASB3*	.	.	.
2	53159228	C	T	intergenic	*MIR4431,ASB3*	.	.	.
2	53216113	G	A	intergenic	*MIR4431,ASB3*	.	.	.
2	53238408	C	T	intergenic	*MIR4431,ASB3*	.	.	.
2	53238409	T	C	intergenic	*MIR4431,ASB3*	.	.	.
2	53275829	G	A	intergenic	*MIR4431,ASB3*	.	.	.
2	53288782	T	G	intergenic	*MIR4431,ASB3*	.	.	.
2	53306463	C	T	intergenic	*MIR4431,ASB3*	.	.	.
2	53389958	C	G	intergenic	*MIR4431,ASB3*	.	.	.
2	53389959	G	C	intergenic	*MIR4431,ASB3*	.	.	.
2	53582605	A	T	intergenic	*MIR4431,ASB3*	.	.	.
2	60677095	C	T	intergenic	*MIR4432HG,BCL11A*	.	.	.
2	64657350	TTTG	T	intergenic	*MIR4433B,LGALSL*	.	.	.
2	112223981	A	G	ncRNA_intronic	*MIR4435-2HG*	.	.	.
2	112254641	T	TG	intergenic	*MIR4435-2HG,ANAPC1*	.	.	.
2	112480939	T	C	intergenic	*MIR4435-2HG,ANAPC1*	.	.	.
2	112346668	C	T	intergenic	*MIR4435-2HG,ANAPC1*	.	.	.

2	90224129	C	A	intergenic	*MIR4436A,LOC654342*	.	.	.
2	90417530	G	T	intergenic	*MIR4436A,LOC654342*	.	.	.
2	90008718	G	A	intergenic	*MIR4436A,LOC654342*	.	.	.
2	90126286	G	C	intergenic	*MIR4436A,LOC654342*	.	.	.
2	90477454	TAGC	T	intergenic	*MIR4436A,LOC654342*	.	.	.
2	89853004	T	C	intergenic	*MIR4436A,LOC654342*	.	.	.
2	90045552	C	A	intergenic	*MIR4436A,LOC654342*	.	.	.
2	90441330	A	G	intergenic	*MIR4436A,LOC654342*	.	.	.
2	90438706	T	C	intergenic	*MIR4436A,LOC654342*	.	.	.
2	89831837	T	A	intergenic	*MIR4436A,LOC654342*	.	.	.
2	89833835	A	T	intergenic	*MIR4436A,LOC654342*	.	.	.
2	89833836	A	T	intergenic	*MIR4436A,LOC654342*	.	.	.
2	89838307	T	TTGATGATG	intergenic	*MIR4436A,LOC654342*	.	.	.
2	89841190	T	C	intergenic	*MIR4436A,LOC654342*	.	.	.
2	89841192	C	A	intergenic	*MIR4436A,LOC654342*	.	.	.
2	89845851	G	A	intergenic	*MIR4436A,LOC654342*	.	.	.
2	89848717	G	A	intergenic	*MIR4436A,LOC654342*	.	.	.
2	89848718	G	A	intergenic	*MIR4436A,LOC654342*	.	.	.
2	89853001	C	T	intergenic	*MIR4436A,LOC654342*	.	.	.

2	89857362	TCCATTCCATC	T	intergenic	*MIR4436A,LOC654342*	.	.	.
2	89864520	CCATGT	C	intergenic	*MIR4436A,LOC654342*	.	.	.
2	89877273	G	T	intergenic	*MIR4436A,LOC654342*	.	.	.
2	90054722	T	G	intergenic	*MIR4436A,LOC654342*	.	.	.
2	90372364	C	T	intergenic	*MIR4436A,LOC654342*	.	.	.
2	90420961	G	C	intergenic	*MIR4436A,LOC654342*	.	.	.
2	89353966	T	C	intergenic	*MIR4436A,NONE*	.	.	.
2	89600020	C	G	intergenic	*MIR4436A,NONE*	.	.	.
2	89365779	T	A	intergenic	*MIR4436A,NONE*	.	.	.
2	89118256	A	T	intergenic	*MIR4436A,NONE*	.	.	.
2	89482861	A	G	intergenic	*MIR4436A,NONE*	.	.	.
2	89482863	C	T	intergenic	*MIR4436A,NONE*	.	.	.
3	25740118	G	T	intergenic	*MIR4442,NGLY1*	.	.	.
3	75396732	C	G	intergenic	*MIR4444-1,FAM86DP*	.	.	.
3	75392653	T	G	intergenic	*MIR4444-1,FAM86DP*	.	.	.
3	75326432	T	C	intergenic	*MIR4444-1,FAM86DP*	.	.	.
3	109467402	G	T	intergenic	*MIR4445,PVRL3-AS1*	.	.	.
3	110593969	T	G	intergenic	*MIR4445,PVRL3-AS1*	.	.	.
3	109372455	G	A	intergenic	*MIR4445,PVRL3-AS1*	.	.	.
3	109406352	TAAATAAATAAAA	T	intergenic	*MIR4445,PVRL3-AS1*	.	.	.
3	110038061	T	C	intergenic	*MIR4445,PVRL3-AS1*	.	.	.
3	110411790	T	C	intergenic	*MIR4445,PVRL3-AS1*	.	.	.

3	110066328	G	C	intergenic	*MIR4445,PVRL3-AS1*	.	.	.
3	109370788	G	A	intergenic	*MIR4445,PVRL3-AS1*	.	.	.
3	109386381	G	C	intergenic	*MIR4445,PVRL3-AS1*	.	.	.
3	109429914	G	A	intergenic	*MIR4445,PVRL3-AS1*	.	.	.
3	109541954	G	C	intergenic	*MIR4445,PVRL3-AS1*	.	.	.
3	109718298	T	G	intergenic	*MIR4445,PVRL3-AS1*	.	.	.
3	109920588	C	T	intergenic	*MIR4445,PVRL3-AS1*	.	.	.
3	109921368	T	A	intergenic	*MIR4445,PVRL3-AS1*	.	.	.
3	109921370	G	T	intergenic	*MIR4445,PVRL3-AS1*	.	.	.
3	109921580	G	A	intergenic	*MIR4445,PVRL3-AS1*	.	.	.
3	109922889	G	A	intergenic	*MIR4445,PVRL3-AS1*	.	.	.
3	109975354	G	T	intergenic	*MIR4445,PVRL3-AS1*	.	.	.
3	110217298	A	G	intergenic	*MIR4445,PVRL3-AS1*	.	.	.
3	110217300	T	C	intergenic	*MIR4445,PVRL3-AS1*	.	.	.
3	110354278	G	A	intergenic	*MIR4445,PVRL3-AS1*	.	.	.
3	110354279	A	T	intergenic	*MIR4445,PVRL3-AS1*	.	.	.
3	110633380	T	G	intergenic	*MIR4445,PVRL3-AS1*	.	.	.
3	116584728	G	C	intergenic	*MIR4447,LINC00901*	.	.	.
3	116589558	G	C	intergenic	*MIR4447,LINC00901*	.	.	.

X	92675129	T	A	intergenic	*MIR4454,NAP1L3*	.	.	.
X	92752970	T	A	intergenic	*MIR4454,NAP1L3*	.	.	.
X	92787520	C	A	intergenic	*MIR4454,NAP1L3*	.	.	.
X	92312318	C	G	intergenic	*MIR4454,NAP1L3*	.	.	.
X	92628855	A	T	intergenic	*MIR4454,NAP1L3*	.	.	.
X	92772090	C	A	intergenic	*MIR4454,NAP1L3*	.	.	.
X	92540586	GGTGTGT	G	intergenic	*MIR4454,NAP1L3*	.	.	.
X	92591959	G	T	intergenic	*MIR4454,NAP1L3*	.	.	.
X	92693153	C	A	intergenic	*MIR4454,NAP1L3*	.	.	.
X	92146332	G	A	intergenic	*MIR4454,NAP1L3*	.	.	.
X	92319348	C	T	intergenic	*MIR4454,NAP1L3*	.	.	.
X	92446482	T	C	intergenic	*MIR4454,NAP1L3*	.	.	.
X	92542929	G	T	intergenic	*MIR4454,NAP1L3*	.	.	.
X	92731197	T	C	intergenic	*MIR4454,NAP1L3*	.	.	.
X	92732080	A	G	intergenic	*MIR4454,NAP1L3*	.	.	.
X	92754332	T	C	intergenic	*MIR4454,NAP1L3*	.	.	.
X	92900681	G	C	intergenic	*MIR4454,NAP1L3*	.	.	.
X	92906189	C	A	intergenic	*MIR4454,NAP1L3*	.	.	.
X	109141759	T	C	intergenic	*MIR4454,TMEM164*	.	.	.
X	109229892	T	G	intergenic	*MIR4454,TMEM164*	.	.	.
X	109230415	G	C	intergenic	*MIR4454,TMEM164*	.	.	.
5	553687	A	G	intergenic	*MIR4456,LOC100996325*	.	.	.
5	8807538	G	A	intergenic	*MIR4458HG,LOC101929284*	.	.	.
5	8763560	G	T	intergenic	*MIR4458HG,LOC101929284*	.	.	.
5	8587732	G	A	intergenic	*MIR4458HG,LOC101929284*	.	.	.
5	8698358	G	A	intergenic	*MIR4458HG,LOC101929284*	.	.	.
5	8698994	G	A	intergenic	*MIR4458HG,LOC101929284*	.	.	.
5	8698996	A	G	intergenic	*MIR4458HG,LOC101929284*	.	.	.

5	8713939	A	T	intergenic	*MIR4458HG,LOC101929284*	.	.	.
5	8736105	C	T	intergenic	*MIR4458HG,LOC101929284*	.	.	.
6	141642921	C	G	intergenic	*MIR4465,NMBR*	.	.	.
6	141927014	A	G	intergenic	*MIR4465,NMBR*	.	.	.
6	141190286	T	C	intergenic	*MIR4465,NMBR*	.	.	.
6	141294674	G	A	intergenic	*MIR4465,NMBR*	.	.	.
6	141464804	GA	G	intergenic	*MIR4465,NMBR*	.	.	.
6	141678324	G	A	intergenic	*MIR4465,NMBR*	.	.	.
6	142140039	G	C	intergenic	*MIR4465,NMBR*	.	.	.
6	142241231	A	G	intergenic	*MIR4465,NMBR*	.	.	.
6	142264078	C	A	intergenic	*MIR4465,NMBR*	.	.	.
6	142264324	C	G	intergenic	*MIR4465,NMBR*	.	.	.
6	142293594	A	G	intergenic	*MIR4465,NMBR*	.	.	.
6	142295827	T	G	intergenic	*MIR4465,NMBR*	.	.	.
6	142337715	G	A	intergenic	*MIR4465,NMBR*	.	.	.
7	137870915	C	T	intergenic	*MIR4468,TRIM24*	.	.	.
7	138110912	A	G	intergenic	*MIR4468,TRIM24*	.	.	.
7	138002960	T	TG	intergenic	*MIR4468,TRIM24*	.	.	.
7	138099717	TTGTGTGTGTGTGTGTATG	T	intergenic	*MIR4468,TRIM24*	.	.	.
8	62778928	GTATATA	G	intergenic	*MIR4470,NKAIN3*	.	.	.
8	62664919	C	T	intergenic	*MIR4470,NKAIN3*	.	.	.
8	62891653	C	G	intergenic	*MIR4470,NKAIN3*	.	.	.
8	63083249	TA	T	intergenic	*MIR4470,NKAIN3*	.	.	.
8	62659452	AAGAG	A	intergenic	*MIR4470,NKAIN3*	.	.	.
8	62757615	G	T	intergenic	*MIR4470,NKAIN3*	.	.	.
8	62760397	T	G	intergenic	*MIR4470,NKAIN3*	.	.	.
8	62879188	T	C	intergenic	*MIR4470,NKAIN3*	.	.	.
8	62879189	G	A	intergenic	*MIR4470,NKAIN3*	.	.	.
8	62879679	G	A	intergenic	*MIR4470,NKAIN3*	.	.	.
8	62934977	G	A	intergenic	*MIR4470,NKAIN3*	.	.	.
8	63151086	T	C	intergenic	*MIR4470,NKAIN3*	.	.	.
8	101501145	G	A	intergenic	*MIR4471,ANKRD46*	.	.	.

9	68422846	C	T	intergenic	*MIR4477A,FRG1JP*	.	.	.
9	124883096	C	T	upstream	*MIR4478*	.	.	.
12	47834205	C	G	intergenic	*MIR4494,RPAP3*	.	.	.
12	47825994	G	A	intergenic	*MIR4494,RPAP3*	.	.	.
12	47894746	G	A	intergenic	*MIR4494,RPAP3*	.	.	.
12	48020579	T	C	intergenic	*MIR4494,RPAP3*	.	.	.
12	48022488	T	C	intergenic	*MIR4494,RPAP3*	.	.	.
12	98370460	G	T	intergenic	*MIR4495,MIR4303*	.	.	.
13	88270385	A	T	ncRNA_intronic	*MIR4500HG*	.	.	.
13	88257713	T	C	ncRNA_intronic	*MIR4500HG*	.	.	.
15	28759488	G	A	intergenic	*MIR4509-1,GOLGA8G*	.	.	.
15	28733393	C	T	intergenic	*MIR4509-1,MIR4509-1*	.	.	.
15	36452102	GTGTA	G	intergenic	*MIR4510,C15orf41*	.	.	.
15	36549859	C	G	intergenic	*MIR4510,C15orf41*	.	.	.
18	45149952	C	CA	intergenic	*MIR4527,SMAD2*	.	.	.
18	45066684	C	T	intergenic	*MIR4527,SMAD2*	.	.	.
18	45255115	G	GAAAAT	intergenic	*MIR4527,SMAD2*	.	.	.
22	49254472	G	A	intergenic	*MIR4535,LINC01310*	.	.	.
22	49217732	G	T	intergenic	*MIR4535,LINC01310*	.	.	.
14	106348812	C	T	intergenic	*MIR4539,KIAA0125*	.	.	.
8	143222435	G	A	intergenic	*MIR4539,MIR4472-1*	.	.	.
8	143198511	G	T	intergenic	*MIR4539,MIR4472-1*	.	.	.
5	174247369	A	G	intergenic	*MIR4634,FLJ16171*	.	.	.
5	174287109	A	C	intergenic	*MIR4634,FLJ16171*	.	.	.
5	174312551	G	T	intergenic	*MIR4634,FLJ16171*	.	.	.
5	174334138	C	T	intergenic	*MIR4634,FLJ16171*	.	.	.

6	92237460	C	G	intergenic	*MIR4643,CASC6*	.	.	.
6	92237886	G	C	intergenic	*MIR4643,CASC6*	.	.	.
7	93424940	A	AT	intergenic	*MIR4652,TFPI2*	.	.	.
3	31468814	C	G	intergenic	*MIR466,STT3B*	.	.	.
3	31301881	TACACAC	T	intergenic	*MIR466,STT3B*	.	.	.
3	31440317	A	C	intergenic	*MIR466,STT3B*	.	.	.
1	228665780	AAATAAT	A	intergenic	*MIR4666A,RNF187*	.	.	.
10	20848104	G	A	intergenic	*MIR4675,NEBL*	.	.	.
10	20848652	T	A	intergenic	*MIR4675,NEBL*	.	.	.
10	90868104	T	C	intergenic	*MIR4679-2,CH25H*	.	.	.
10	121790010	T	C	intergenic	*MIR4682,PLPP4*	.	.	.
10	121864092	G	A	intergenic	*MIR4682,PLPP4*	.	.	.
10	122177529	C	A	intergenic	*MIR4682,PLPP4*	.	.	.
10	121885284	C	T	intergenic	*MIR4682,PLPP4*	.	.	.
10	121909778	T	C	intergenic	*MIR4682,PLPP4*	.	.	.
10	121910418	T	G	intergenic	*MIR4682,PLPP4*	.	.	.
10	122174127	A	T	intergenic	*MIR4682,PLPP4*	.	.	.
10	122207604	T	G	intergenic	*MIR4682,PLPP4*	.	.	.
13	66856315	G	A	intergenic	*MIR4704,PCDH9*	.	.	.
13	66864161	T	TA	intergenic	*MIR4704,PCDH9*	.	.	.
14	65857039	C	T	intergenic	*MIR4708,FUT8*	.	.	.
16	77053061	A	C	intergenic	*MIR4719,MON1B*	.	.	.
16	77007897	G	C	intergenic	*MIR4719,MON1B*	.	.	.
17	29902361	G	GAGA	ncRNA_exonic	*MIR4725*	.	.	.
X	6440512	A	G	intergenic	*MIR4770,VCX3A*	.	.	.
2	87656188	C	A	intergenic	*MIR4771-2,LINC00152*	.	.	.
2	87623836	C	G	intergenic	*MIR4771-2,LINC00152*	.	.	.
2	87593015	G	A	intergenic	*MIR4771-2,LINC00152*	.	.	.
2	87655715	C	G	intergenic	*MIR4771-2,LINC00152*	.	.	.
2	87588337	A	C	intergenic	*MIR4771-2,LINC00152*	.	.	.
2	87569617	G	C	intergenic	*MIR4771-2,LINC00152*	.	.	.

2	87625543	C	G	intergenic	*MIR4771-2,LINC00152*	.	.	.
2	87624058	T	A	intergenic	*MIR4771-2,LINC00152*	.	.	.
2	87627976	C	T	intergenic	*MIR4771-2,LINC00152*	.	.	.
2	87641926	TGCCAGCCAAGCCAGCCAGGCAGCCAA	T	intergenic	*MIR4771-2,LINC00152*	.	.	.
2	87643073	A	T	intergenic	*MIR4771-2,LINC00152*	.	.	.
2	87499411	T	C	intergenic	*MIR4771-2,LINC00152*	.	.	.
2	87499412	G	A	intergenic	*MIR4771-2,LINC00152*	.	.	.
3	6211833	C	A	intergenic	*MIR4790,GRM7-AS3*	.	.	.
3	5476740	G	A	intergenic	*MIR4790,GRM7-AS3*	.	.	.
3	5553010	C	T	intergenic	*MIR4790,GRM7-AS3*	.	.	.
3	5615439	C	G	intergenic	*MIR4790,GRM7-AS3*	.	.	.
3	5933825	G	A	intergenic	*MIR4790,GRM7-AS3*	.	.	.
3	6411324	A	G	intergenic	*MIR4790,GRM7-AS3*	.	.	.
3	6587070	T	C	intergenic	*MIR4790,GRM7-AS3*	.	.	.
3	6623838	G	C	intergenic	*MIR4790,GRM7-AS3*	.	.	.
3	5458151	T	A	intergenic	*MIR4790,GRM7-AS3*	.	.	.
3	5602637	C	T	intergenic	*MIR4790,GRM7-AS3*	.	.	.
3	6554934	C	T	intergenic	*MIR4790,GRM7-AS3*	.	.	.

3	5629994	A	G	intergenic	*MIR4790,GRM7-AS3*	.	.	.
3	6028148	A	G	intergenic	*MIR4790,GRM7-AS3*	.	.	.
3	6299701	T	TACAC	intergenic	*MIR4790,GRM7-AS3*	.	.	.
3	6404307	C	T	intergenic	*MIR4790,GRM7-AS3*	.	.	.
3	6495085	A	AAT	intergenic	*MIR4790,GRM7-AS3*	.	.	.
3	6550483	C	T	intergenic	*MIR4790,GRM7-AS3*	.	.	.
3	6638993	TA	T	intergenic	*MIR4790,GRM7-AS3*	.	.	.
3	5416053	T	G	intergenic	*MIR4790,GRM7-AS3*	.	.	.
3	5421717	C	T	intergenic	*MIR4790,GRM7-AS3*	.	.	.
3	5423011	T	A	intergenic	*MIR4790,GRM7-AS3*	.	.	.
3	5423013	A	G	intergenic	*MIR4790,GRM7-AS3*	.	.	.
3	5458811	T	C	intergenic	*MIR4790,GRM7-AS3*	.	.	.
3	5555975	G	T	intergenic	*MIR4790,GRM7-AS3*	.	.	.
3	5559187	T	C	intergenic	*MIR4790,GRM7-AS3*	.	.	.
3	5635254	G	A	intergenic	*MIR4790,GRM7-AS3*	.	.	.
3	5845500	A	C	intergenic	*MIR4790,GRM7-AS3*	.	.	.
3	5955051	A	G	intergenic	*MIR4790,GRM7-AS3*	.	.	.
3	5960926	C	G	intergenic	*MIR4790,GRM7-AS3*	.	.	.
3	5977288	G	A	intergenic	*MIR4790,GRM7-AS3*	.	.	.

3	5980697	A	G	intergenic	*MIR4790,GRM7-AS3*	.	.	.
3	5982644	C	CA	intergenic	*MIR4790,GRM7-AS3*	.	.	.
3	6578377	A	G	intergenic	*MIR4790,GRM7-AS3*	.	.	.
3	24713068	C	T	intergenic	*MIR4792,RARB*	.	.	.
3	24765585	TTGTGTGTG	T	intergenic	*MIR4792,RARB*	.	.	.
3	24861687	A	AT	intergenic	*MIR4792,RARB*	.	.	.
3	24610599	G	A	intergenic	*MIR4792,RARB*	.	.	.
3	24611694	T	C	intergenic	*MIR4792,RARB*	.	.	.
3	24675459	C	T	intergenic	*MIR4792,RARB*	.	.	.
3	24737788	G	T	intergenic	*MIR4792,RARB*	.	.	.
14	101500078	G	T	upstream	*MIR495*	.	.	.
13	57284390	G	T	intergenic	*MIR5007,PRR20D*	.	.	.
13	57328356	C	A	intergenic	*MIR5007,PRR20D*	.	.	.
13	57573243	T	A	intergenic	*MIR5007,PRR20D*	.	.	.
13	57081217	CAGAG	C	intergenic	*MIR5007,PRR20D*	.	.	.
13	56676712	C	A	intergenic	*MIR5007,PRR20D*	.	.	.
13	56744047	C	A	intergenic	*MIR5007,PRR20D*	.	.	.
13	56906096	T	C	intergenic	*MIR5007,PRR20D*	.	.	.
13	57278767	T	C	intergenic	*MIR5007,PRR20D*	.	.	.
13	57289309	G	A	intergenic	*MIR5007,PRR20D*	.	.	.
13	57481066	T	G	intergenic	*MIR5007,PRR20D*	.	.	.
13	55886703	C	T	intergenic	*MIR5007,PRR20D*	.	.	.
13	57294918	G	A	intergenic	*MIR5007,PRR20D*	.	.	.
13	57081197	CAGAGAG	C	intergenic	*MIR5007,PRR20D*	.	.	.
13	56339278	T	C	intergenic	*MIR5007,PRR20D*	.	.	.
13	56340995	C	T	intergenic	*MIR5007,PRR20D*	.	.	.
13	57049337	G	GT	intergenic	*MIR5007,PRR20D*	.	.	.
13	57628186	G	T	intergenic	*MIR5007,PRR20D*	.	.	.
13	55780770	C	T	intergenic	*MIR5007,PRR20D*	.	.	.
13	55847710	C	T	intergenic	*MIR5007,PRR20D*	.	.	.
13	55848417	A	C	intergenic	*MIR5007,PRR20D*	.	.	.
13	56131064	T	A	intergenic	*MIR5007,PRR20D*	.	.	.
13	56524179	G	C	intergenic	*MIR5007,PRR20D*	.	.	.
13	56524181	G	A	intergenic	*MIR5007,PRR20D*	.	.	.

13	56590968	G	A	intergenic	*MIR5007,PRR20D*	.	.	.
13	56610823	T	C	intergenic	*MIR5007,PRR20D*	.	.	.
13	56757278	A	C	intergenic	*MIR5007,PRR20D*	.	.	.
13	56761224	C	G	intergenic	*MIR5007,PRR20D*	.	.	.
13	56984578	C	A	intergenic	*MIR5007,PRR20D*	.	.	.
13	57084473	G	A	intergenic	*MIR5007,PRR20D*	.	.	.
13	57205991	G	A	intergenic	*MIR5007,PRR20D*	.	.	.
13	57207685	T	A	intergenic	*MIR5007,PRR20D*	.	.	.
13	57235600	A	T	intergenic	*MIR5007,PRR20D*	.	.	.
13	57235601	G	A	intergenic	*MIR5007,PRR20D*	.	.	.
13	57339803	G	A	intergenic	*MIR5007,PRR20D*	.	.	.
13	57340694	G	T	intergenic	*MIR5007,PRR20D*	.	.	.
13	57342385	T	A	intergenic	*MIR5007,PRR20D*	.	.	.
13	57482891	G	A	intergenic	*MIR5007,PRR20D*	.	.	.
13	57567980	G	A	intergenic	*MIR5007,PRR20D*	.	.	.
13	57655447	G	GA	intergenic	*MIR5007,PRR20D*	.	.	.
21	28744174	A	G	ncRNA_intronic	*MIR5009*	.	.	.
21	28937734	A	G	ncRNA_intronic	*MIR5009*	.	.	.
21	28716229	A	G	ncRNA_intronic	*MIR5009*	.	.	.
21	28915119	CT	C	ncRNA_intronic	*MIR5009*	.	.	.
21	28950165	C	G	ncRNA_intronic	*MIR5009*	.	.	.
21	28950532	G	T	ncRNA_intronic	*MIR5009*	.	.	.
21	28988466	A	T	ncRNA_intronic	*MIR5009*	.	.	.
21	29033888	A	G	ncRNA_intronic	*MIR5009*	.	.	.
21	29083821	C	T	ncRNA_intronic	*MIR5009*	.	.	.
21	29141030	C	T	ncRNA_intronic	*MIR5009*	.	.	.
21	29163260	T	A	ncRNA_intronic	*MIR5009*	.	.	.
21	29163261	T	C	ncRNA_intronic	*MIR5009*	.	.	.
18	65015624	T	A	intergenic	*MIR5011,DSEL*	.	.	.
18	65114579	T	C	intergenic	*MIR5011,DSEL*	.	.	.
16	48796212	T	G	intergenic	*MIR5095,CBLN1*	.	.	.
16	48675153	C	A	intergenic	*MIR5095,CBLN1*	.	.	.
16	49220911	G	T	intergenic	*MIR5095,CBLN1*	.	.	.
16	49259460	C	T	intergenic	*MIR5095,CBLN1*	.	.	.
16	48696619	C	G	intergenic	*MIR5095,CBLN1*	.	.	.
16	48795734	A	C	intergenic	*MIR5095,CBLN1*	.	.	.
16	48797262	C	T	intergenic	*MIR5095,CBLN1*	.	.	.
16	49068512	T	C	intergenic	*MIR5095,CBLN1*	.	.	.

1	235685540	C	T	ncRNA_intronic	*MIR5096*	.	.	.
1	235780973	C	T	ncRNA_intronic	*MIR5096*	.	.	.
1	235824110	A	AGATCGGAA	ncRNA_intronic	*MIR5096*	.	.	.
1	235867763	C	T	ncRNA_intronic	*MIR5096*	.	.	.
10	43512469	A	C	intergenic	*MIR5100,RET*	.	.	.
X	146283944	G	C	intergenic	*MIR513B,MIR513A1*	.	.	.
X	146286755	A	G	intergenic	*MIR513B,MIR513A1*	.	.	.
X	146984063	C	A	intergenic	*MIR514A2,FMR1-AS1*	.	.	.
X	146436392	C	T	intergenic	*MIR514A2,FMR1-AS1*	.	.	.
X	146629735	A	G	intergenic	*MIR514A2,FMR1-AS1*	.	.	.
X	146906448	G	A	intergenic	*MIR514A2,FMR1-AS1*	.	.	.
X	146911631	G	A	intergenic	*MIR514A2,FMR1-AS1*	.	.	.
X	146946060	T	C	intergenic	*MIR514A2,FMR1-AS1*	.	.	.
14	107278848	G	A	intergenic	*MIR5195,NONE*	.	.	.
14	107265073	C	T	intergenic	*MIR5195,NONE*	.	.	.
6	18682046	G	A	intergenic	*MIR548A1,LOC101928519*	.	.	.
6	18678802	T	C	intergenic	*MIR548A1,LOC101928519*	.	.	.
6	18718902	T	A	intergenic	*MIR548A1,LOC101928519*	.	.	.
6	18871088	A	T	intergenic	*MIR548A1,LOC101928519*	.	.	.
6	18871089	G	GA	intergenic	*MIR548A1,LOC101928519*	.	.	.
6	18968017	A	T	intergenic	*MIR548A1,LOC101928519*	.	.	.
3	104442180	G	T	intergenic	*MIR548A3,ALCAM*	.	.	.
3	104462130	T	C	intergenic	*MIR548A3,ALCAM*	.	.	.
3	104541670	C	T	intergenic	*MIR548A3,ALCAM*	.	.	.

3	103960236	C	T	intergenic	*MIR548A3,ALCAM*	.	.	.
3	104027370	G	T	intergenic	*MIR548A3,ALCAM*	.	.	.
3	104028481	T	C	intergenic	*MIR548A3,ALCAM*	.	.	.
3	104093170	T	G	intergenic	*MIR548A3,ALCAM*	.	.	.
3	104183707	G	T	intergenic	*MIR548A3,ALCAM*	.	.	.
3	104186372	A	G	intergenic	*MIR548A3,ALCAM*	.	.	.
3	104370009	C	T	intergenic	*MIR548A3,ALCAM*	.	.	.
3	104936449	C	G	intergenic	*MIR548A3,ALCAM*	.	.	.
3	105027918	C	T	intergenic	*MIR548A3,ALCAM*	.	.	.
3	105028478	C	T	intergenic	*MIR548A3,ALCAM*	.	.	.
3	105030390	A	AAACT	intergenic	*MIR548A3,ALCAM*	.	.	.
16	22779005	T	A	ncRNA_intronic	*MIR548AA2,MIR548D2*	.	.	.
16	22779006	G	T	ncRNA_intronic	*MIR548AA2,MIR548D2*	.	.	.
3	103353838	C	CAAAAAAAA	intergenic	*MIR548AB,MIR548A3*	.	.	.
3	103566148	T	C	intergenic	*MIR548AB,MIR548A3*	.	.	.
3	103835678	C	CAAA	intergenic	*MIR548AB,MIR548A3*	.	.	.
3	103295418	G	T	intergenic	*MIR548AB,MIR548A3*	.	.	.
3	103486278	C	A	intergenic	*MIR548AB,MIR548A3*	.	.	.
3	103477841	G	A	intergenic	*MIR548AB,MIR548A3*	.	.	.
3	103600778	C	T	intergenic	*MIR548AB,MIR548A3*	.	.	.
3	103650140	A	T	intergenic	*MIR548AB,MIR548A3*	.	.	.
3	103652087	A	C	intergenic	*MIR548AB,MIR548A3*	.	.	.
3	103742169	C	G	intergenic	*MIR548AB,MIR548A3*	.	.	.
3	103803042	A	G	intergenic	*MIR548AB,MIR548A3*	.	.	.

3	103839151	G	T	intergenic	*MIR548AB,MIR548A3*	.	.	.
3	23513424	A	G	ncRNA_intronic	*MIR548AC*	.	.	.
3	23493493	T	C	ncRNA_intronic	*MIR548AC*	.	.	.
3	23458129	C	CAG	ncRNA_intronic	*MIR548AC*	.	.	.
3	23602524	C	A	ncRNA_intronic	*MIR548AC*	.	.	.
3	23624905	C	T	ncRNA_intronic	*MIR548AC*	.	.	.
2	185360704	C	A	intergenic	*MIR548AE1,ZNF804A*	.	.	.
16	48315775	C	A	ncRNA_intronic	*MIR548AE2*	.	.	.
4	62242023	T	C	intergenic	*MIR548AG1,ADGRL3*	.	.	.
4	61880657	A	T	intergenic	*MIR548AG1,ADGRL3*	.	.	.
20	59522346	ATT	A	intergenic	*MIR548AG2,LOC100506470*	.	.	.
14	29922217	G	C	ncRNA_intronic	*MIR548AI*	.	.	.
14	29900542	ATCTCAAAAAAAAAAAAAAAAAGAGAATGAATACTTCTGCACCAGGAAGCAGCAGTTGTTTTTATCCAC	A	ncRNA_intronic	*MIR548AI*	.	.	.
14	29923005	C	T	ncRNA_intronic	*MIR548AI*	.	.	.
4	23275167	T	C	ncRNA_intronic	*MIR548AJ2*	.	.	.
4	23393802	A	G	ncRNA_intronic	*MIR548AJ2*	.	.	.
4	23294549	A	T	ncRNA_intronic	*MIR548AJ2*	.	.	.
4	23420141	A	G	ncRNA_intronic	*MIR548AJ2*	.	.	.
4	23646361	T	C	intergenic	*MIR548AJ2,PPARGC1A*	.	.	.
4	23660926	C	T	intergenic	*MIR548AJ2,PPARGC1A*	.	.	.
13	99965360	C	T	ncRNA_intronic	*MIR548AN*	.	.	.
13	100054494	C	T	ncRNA_intronic	*MIR548AN*	.	.	.
12	122036250	G	A	intergenic	*MIR548AQ,ORAI1*	.	.	.

13	104026427	C	G	intergenic	*MIR548AS,LINC01309*	.	.	.
13	104053733	C	G	intergenic	*MIR548AS,LINC01309*	.	.	.
13	104073380	A	C	intergenic	*MIR548AS,LINC01309*	.	.	.
13	104027478	A	T	intergenic	*MIR548AS,LINC01309*	.	.	.
13	104027606	T	C	intergenic	*MIR548AS,LINC01309*	.	.	.
13	104070586	A	G	intergenic	*MIR548AS,LINC01309*	.	.	.
2	107913381	C	A	ncRNA_intronic	*MIR548AU*	.	.	.
2	107929101	A	T	ncRNA_intronic	*MIR548AU*	.	.	.
2	108341953	A	T	intergenic	*MIR548AU,GACAT1*	.	.	.
2	108147786	A	T	intergenic	*MIR548AU,GACAT1*	.	.	.
2	108184236	CATAT	C	intergenic	*MIR548AU,GACAT1*	.	.	.
2	108108035	T	C	intergenic	*MIR548AU,GACAT1*	.	.	.
2	108358403	G	T	intergenic	*MIR548AU,GACAT1*	.	.	.
2	108226049	C	A	intergenic	*MIR548AU,GACAT1*	.	.	.
2	108161032	A	G	intergenic	*MIR548AU,GACAT1*	.	.	.
2	108284613	G	A	intergenic	*MIR548AU,GACAT1*	.	.	.
4	16616093	A	G	ncRNA_intronic	*MIR548AX*	.	.	.
4	16616095	C	G	ncRNA_intronic	*MIR548AX*	.	.	.
14	64761393	C	T	ncRNA_intronic	*MIR548AZ*	.	.	.
14	64640977	T	A	ncRNA_intronic	*MIR548AZ*	.	.	.
1	186075244	C	A	ncRNA_intronic	*MIR548F1*	.	.	.
1	186129489	C	T	ncRNA_intronic	*MIR548F1*	.	.	.
1	186185505	A	G	ncRNA_intronic	*MIR548F1*	.	.	.
1	186230435	G	A	ncRNA_intronic	*MIR548F1*	.	.	.

1	186237755	CAACAACA ACAAT	C	ncRNA_intronic	*MIR548F1*	.	.	.
1	186538725	TA	T	intergenic	*MIR548F1,PTGS2*	.	.	.
1	186479996	C	T	intergenic	*MIR548F1,PTGS2*	.	.	.
1	186516792	G	C	intergenic	*MIR548F1,PTGS2*	.	.	.
1	186516793	G	A	intergenic	*MIR548F1,PTGS2*	.	.	.
1	186637291	T	C	intergenic	*MIR548F1,PTGS2*	.	.	.
1	218930842	A	G	ncRNA_intronic	*MIR548F3*	.	.	.
1	219034033	T	C	ncRNA_intronic	*MIR548F3*	.	.	.
1	218733403	C	T	ncRNA_intronic	*MIR548F3*	.	.	.
1	218734010	T	G	ncRNA_intronic	*MIR548F3*	.	.	.
1	218802122	C	T	ncRNA_intronic	*MIR548F3*	.	.	.
1	218803313	T	C	ncRNA_intronic	*MIR548F3*	.	.	.
1	218803314	T	C	ncRNA_intronic	*MIR548F3*	.	.	.
1	218898876	A	T	ncRNA_intronic	*MIR548F3*	.	.	.
1	218955748	C	T	ncRNA_intronic	*MIR548F3*	.	.	.
1	218994450	T	C	ncRNA_intronic	*MIR548F3*	.	.	.
1	218994451	G	A	ncRNA_intronic	*MIR548F3*	.	.	.
1	218994770	T	C	ncRNA_intronic	*MIR548F3*	.	.	.
1	218995193	C	T	ncRNA_intronic	*MIR548F3*	.	.	.
13	36179340	A	G	ncRNA_intronic	*MIR548F5*	.	.	.
3	99311503	G	C	ncRNA_intronic	*MIR548G*	.	.	.
3	99312706	G	A	ncRNA_intronic	*MIR548G*	.	.	.
3	99313465	A	T	ncRNA_intronic	*MIR548G*	.	.	.
3	99324054	A	G	ncRNA_intronic	*MIR548G*	.	.	.
3	99400021	T	G	ncRNA_intronic	*MIR548G*	.	.	.
3	99711403	T	A	ncRNA_intronic	*MIR548G*	.	.	.
9	78255877	T	A	ncRNA_intronic	*MIR548H3*	.	.	.
6	97605422	T	C	ncRNA_intronic	*MIR548H3*	.	.	.
9	78168968	G	T	ncRNA_intronic	*MIR548H3*	.	.	.
9	78317430	C	T	ncRNA_intronic	*MIR548H3*	.	.	.
9	78432638	T	TTTTC	intergenic	*MIR548H3,PCSK5*	.	.	.
9	78499405	C	T	intergenic	*MIR548H3,PCSK5*	.	.	.
9	78383968	T	C	intergenic	*MIR548H3,PCSK5*	.	.	.
15	69209253	T	C	ncRNA_intronic	*MIR548H4*	.	.	.
3	125515813	G	A	intergenic	*MIR548I1,LOC1019 27056*	.	.	.
4	9651608	A	G	intergenic	*MIR548I2,DRD5*	.	.	.

4	9723069	G	T	intergenic	*MIR548I2,DRD5*	.	.	.
4	9604928	C	A	intergenic	*MIR548I2,DRD5*	.	.	.
4	9612921	G	A	intergenic	*MIR548I2,DRD5*	.	.	.
4	9622371	G	A	intergenic	*MIR548I2,DRD5*	.	.	.
4	9623216	A	C	intergenic	*MIR548I2,DRD5*	.	.	.
8	8049062	A	T	intergenic	*MIR548I3,FAM86B3P*	.	.	.
8	8017165	A	T	intergenic	*MIR548I3,FAM86B3P*	.	.	.
7	147010271	C	T	ncRNA_intronic	*MIR548I4*	.	.	.
7	147050951	T	C	ncRNA_intronic	*MIR548I4*	.	.	.
7	79607183	GGGCCGGCAGGGCC	G	intergenic	*MIR548M,GNAI1*	.	.	.
7	79584109	CA	C	intergenic	*MIR548M,GNAI1*	.	.	.
7	79664050	C	T	intergenic	*MIR548M,GNAI1*	.	.	.
7	79409461	C	T	intergenic	*MIR548M,GNAI1*	.	.	.
7	79504989	C	A	intergenic	*MIR548M,GNAI1*	.	.	.
7	79505208	C	A	intergenic	*MIR548M,GNAI1*	.	.	.
7	79515475	C	G	intergenic	*MIR548M,GNAI1*	.	.	.
7	79515476	A	C	intergenic	*MIR548M,GNAI1*	.	.	.
7	79547403	C	G	intergenic	*MIR548M,GNAI1*	.	.	.
7	79568989	A	C	intergenic	*MIR548M,GNAI1*	.	.	.
7	79625639	A	T	intergenic	*MIR548M,GNAI1*	.	.	.
2	179524101	G	C	ncRNA_intronic	*MIR548N*	.	.	.
8	29958365	CTGTG	C	ncRNA_intronic	*MIR548O2*	.	.	.
8	29977383	A	G	ncRNA_intronic	*MIR548O2*	.	.	.
7	147917243	G	T	ncRNA_intronic	*MIR548T*	.	.	.
7	147649938	C	T	ncRNA_intronic	*MIR548T*	.	.	.
7	147710945	G	C	ncRNA_intronic	*MIR548T*	.	.	.
7	147692341	G	A	ncRNA_intronic	*MIR548T*	.	.	.
7	148031665	T	C	ncRNA_intronic	*MIR548T*	.	.	.
7	147682208	AAAT	A	ncRNA_intronic	*MIR548T*	.	.	.
17	60901950	ATGTG	A	ncRNA_intronic	*MIR548W*	.	.	.
17	61023690	G	GCACCACCA	ncRNA_intronic	*MIR548W*	.	.	.
17	60862490	C	T	ncRNA_intronic	*MIR548W*	.	.	.
17	60862668	G	A	ncRNA_intronic	*MIR548W*	.	.	.
17	60885039	G	A	ncRNA_intronic	*MIR548W*	.	.	.
17	61167946	G	A	ncRNA_intronic	*MIR548W*	.	.	.

17	61168709	T	A	ncRNA_intronic	*MIR548W*	.	.	.
16	9506562	A	G	intergenic	*MIR548X,LINC01177*	.	.	.
16	9388051	G	A	intergenic	*MIR548X,LINC01177*	.	.	.
16	9441566	T	C	intergenic	*MIR548X,LINC01177*	.	.	.
13	66637344	C	T	intergenic	*MIR548X2,MIR4704*	.	.	.
21	20119712	A	T	ncRNA_intronic	*MIR548XHG*	.	.	.
21	19961113	T	G	ncRNA_intronic	*MIR548XHG*	.	.	.
21	20089115	T	C	ncRNA_intronic	*MIR548XHG*	.	.	.
21	20103298	T	C	ncRNA_intronic	*MIR548XHG*	.	.	.
21	19969174	T	G	ncRNA_intronic	*MIR548XHG*	.	.	.
21	20037170	C	T	ncRNA_intronic	*MIR548XHG*	.	.	.
21	21045326	A	ATTTT	intergenic	*MIR548XHG,LINC00320*	.	.	.
21	21678419	T	C	intergenic	*MIR548XHG,LINC00320*	.	.	.
21	20435925	G	A	intergenic	*MIR548XHG,LINC00320*	.	.	.
21	20942291	T	C	intergenic	*MIR548XHG,LINC00320*	.	.	.
21	21285466	C	G	intergenic	*MIR548XHG,LINC00320*	.	.	.
21	21479648	AATTTTCAAGTT	A	intergenic	*MIR548XHG,LINC00320*	.	.	.
21	21719807	T	C	intergenic	*MIR548XHG,LINC00320*	.	.	.
21	21887171	G	T	intergenic	*MIR548XHG,LINC00320*	.	.	.
21	21523758	G	T	intergenic	*MIR548XHG,LINC00320*	.	.	.
21	21565863	A	C	intergenic	*MIR548XHG,LINC00320*	.	.	.
21	20713621	G	T	intergenic	*MIR548XHG,LINC00320*	.	.	.

21	21142467	C	A	intergenic	*MIR548XHG,LINC00320*	.	.	.
21	21499613	G	A	intergenic	*MIR548XHG,LINC00320*	.	.	.
21	21384500	C	G	intergenic	*MIR548XHG,LINC00320*	.	.	.
21	20161026	T	G	intergenic	*MIR548XHG,LINC00320*	.	.	.
21	20161606	C	T	intergenic	*MIR548XHG,LINC00320*	.	.	.
21	20206790	G	C	intergenic	*MIR548XHG,LINC00320*	.	.	.
21	20237284	T	C	intergenic	*MIR548XHG,LINC00320*	.	.	.
21	20347404	C	T	intergenic	*MIR548XHG,LINC00320*	.	.	.
21	20444837	T	C	intergenic	*MIR548XHG,LINC00320*	.	.	.
21	20522038	G	A	intergenic	*MIR548XHG,LINC00320*	.	.	.
21	20522514	T	C	intergenic	*MIR548XHG,LINC00320*	.	.	.
21	20888199	G	A	intergenic	*MIR548XHG,LINC00320*	.	.	.
21	20908587	C	T	intergenic	*MIR548XHG,LINC00320*	.	.	.
21	21187778	C	T	intergenic	*MIR548XHG,LINC00320*	.	.	.
21	21188042	G	A	intergenic	*MIR548XHG,LINC00320*	.	.	.
21	21274255	T	C	intergenic	*MIR548XHG,LINC00320*	.	.	.
21	21316638	T	C	intergenic	*MIR548XHG,LINC00320*	.	.	.
21	21394150	T	C	intergenic	*MIR548XHG,LINC00320*	.	.	.
21	21420301	A	G	intergenic	*MIR548XHG,LINC00320*	.	.	.

21	21420302	A	ATTCTGTTAT	intergenic	*MIR548XHG,LINC00320*	.	.	.
21	21531152	A	G	intergenic	*MIR548XHG,LINC00320*	.	.	.
21	21609525	A	G	intergenic	*MIR548XHG,LINC00320*	.	.	.
21	21609527	T	A	intergenic	*MIR548XHG,LINC00320*	.	.	.
21	21764505	G	A	intergenic	*MIR548XHG,LINC00320*	.	.	.
21	22058461	T	C	intergenic	*MIR548XHG,LINC00320*	.	.	.
21	22058462	G	A	intergenic	*MIR548XHG,LINC00320*	.	.	.
1	35171667	G	T	intergenic	*MIR552,GJB5*	.	.	.
3	15949866	A	T	intergenic	*MIR563,GALNT15*	.	.	.
3	16201461	C	T	intergenic	*MIR563,GALNT15*	.	.	.
8	103148402	C	T	intergenic	*MIR5680,RRM2B*	.	.	.
4	165594031	G	GA	ncRNA_intronic	*MIR5684*	.	.	.
4	165437152	C	T	ncRNA_intronic	*MIR5684*	.	.	.
12	79890307	G	C	intergenic	*MIR5692B,PAWR*	.	.	.
12	79901723	T	TC	intergenic	*MIR5692B,PAWR*	.	.	.
12	79901725	G	C	intergenic	*MIR5692B,PAWR*	.	.	.
3	195224577	T	A	intergenic	*MIR5692C1,PPP1R2*	.	.	.
3	195225679	A	C	intergenic	*MIR5692C1,PPP1R2*	.	.	.
3	195231939	C	G	intergenic	*MIR5692C1,PPP1R2*	.	.	.
10	122473637	G	T	ncRNA_intronic	*MIR5694*	.	.	.
10	122374362	G	T	ncRNA_intronic	*MIR5694*	.	.	.
10	122634016	C	T	ncRNA_intronic	*MIR5694*	.	.	.
10	122433167	T	C	ncRNA_intronic	*MIR5694*	.	.	.
10	122433169	A	T	ncRNA_intronic	*MIR5694*	.	.	.
10	122488436	G	T	ncRNA_intronic	*MIR5694*	.	.	.
10	122672628	A	G	ncRNA_intronic	*MIR5694*	.	.	.
10	123073091	C	A	intergenic	*MIR5694,FGFR2*	.	.	.
10	123151926	T	A	intergenic	*MIR5694,FGFR2*	.	.	.

10	122989115	G	A	intergenic	*MIR5694,FGFR2*	.	.	.
10	122902507	C	T	intergenic	*MIR5694,FGFR2*	.	.	.
10	122967094	C	T	intergenic	*MIR5694,FGFR2*	.	.	.
10	122986764	A	G	intergenic	*MIR5694,FGFR2*	.	.	.
10	123008774	C	A	intergenic	*MIR5694,FGFR2*	.	.	.
10	123008775	A	G	intergenic	*MIR5694,FGFR2*	.	.	.
6	126454682	G	C	intergenic	*MIR5695,CENPW*	.	.	.
6	126559307	T	G	intergenic	*MIR5695,CENPW*	.	.	.
6	126564399	C	T	intergenic	*MIR5695,CENPW*	.	.	.
6	126652462	G	A	intergenic	*MIR5695,CENPW*	.	.	.
2	101934976	G	A	intergenic	*MIR5696,CREG2*	.	.	.
2	101935214	A	G	intergenic	*MIR5696,CREG2*	.	.	.
2	101936830	C	T	intergenic	*MIR5696,CREG2*	.	.	.
2	101937181	A	C	intergenic	*MIR5696,CREG2*	.	.	.
8	81280966	A	G	intergenic	*MIR5708,ZBTB10*	.	.	.
8	81345952	C	G	intergenic	*MIR5708,ZBTB10*	.	.	.
8	81386590	A	T	intergenic	*MIR5708,ZBTB10*	.	.	.
4	11371624	G	A	intergenic	*MIR572,HS3ST1*	.	.	.
6	127028166	C	CTA	intergenic	*MIR588,RSPO3*	.	.	.
6	127062981	AG	A	intergenic	*MIR588,RSPO3*	.	.	.
6	126829409	T	C	intergenic	*MIR588,RSPO3*	.	.	.
6	126882859	G	A	intergenic	*MIR588,RSPO3*	.	.	.
6	126883722	G	A	intergenic	*MIR588,RSPO3*	.	.	.
6	126916581	T	C	intergenic	*MIR588,RSPO3*	.	.	.
6	126917990	G	T	intergenic	*MIR588,RSPO3*	.	.	.
6	127048869	T	C	intergenic	*MIR588,RSPO3*	.	.	.
6	127048870	G	A	intergenic	*MIR588,RSPO3*	.	.	.
6	127185371	G	A	intergenic	*MIR588,RSPO3*	.	.	.
6	127185424	C	T	intergenic	*MIR588,RSPO3*	.	.	.
6	127185708	T	G	intergenic	*MIR588,RSPO3*	.	.	.
6	127291630	C	T	intergenic	*MIR588,RSPO3*	.	.	.
6	127291631	A	G	intergenic	*MIR588,RSPO3*	.	.	.
6	127305490	G	C	intergenic	*MIR588,RSPO3*	.	.	.
6	127345137	G	A	intergenic	*MIR588,RSPO3*	.	.	.
6	127368801	G	A	intergenic	*MIR588,RSPO3*	.	.	.
10	2209345	C	T	intergenic	*MIR6072,LINC00701*	.	.	.

4	172573857	A	G	intergenic	*MIR6082,GALNTL6*	.	.	.
4	172693727	A	T	intergenic	*MIR6082,GALNTL6*	.	.	.
4	172180762	C	G	intergenic	*MIR6082,GALNTL6*	.	.	.
4	172234013	C	A	intergenic	*MIR6082,GALNTL6*	.	.	.
4	172234800	A	T	intergenic	*MIR6082,GALNTL6*	.	.	.
4	172309843	C	T	intergenic	*MIR6082,GALNTL6*	.	.	.
4	172685186	T	C	intergenic	*MIR6082,GALNTL6*	.	.	.
4	172721057	C	A	intergenic	*MIR6082,GALNTL6*	.	.	.
4	172721830	A	G	intergenic	*MIR6082,GALNTL6*	.	.	.
X	108526435	T	C	intergenic	*MIR6087,GUCY2F*	.	.	.
X	108376531	A	G	intergenic	*MIR6087,GUCY2F*	.	.	.
X	108438138	C	CTTG	intergenic	*MIR6087,GUCY2F*	.	.	.
X	108438140	C	T	intergenic	*MIR6087,GUCY2F*	.	.	.
X	108311773	T	C	intergenic	*MIR6087,GUCY2F*	.	.	.
X	108311774	G	A	intergenic	*MIR6087,GUCY2F*	.	.	.
X	108312664	A	G	intergenic	*MIR6087,GUCY2F*	.	.	.
X	108328334	A	AT	intergenic	*MIR6087,GUCY2F*	.	.	.
X	108328475	A	G	intergenic	*MIR6087,GUCY2F*	.	.	.
X	108329626	T	TC	intergenic	*MIR6087,GUCY2F*	.	.	.
X	108329627	A	T	intergenic	*MIR6087,GUCY2F*	.	.	.
X	108330270	C	A	intergenic	*MIR6087,GUCY2F*	.	.	.
X	108335822	A	G	intergenic	*MIR6087,GUCY2F*	.	.	.
X	108395688	CTTTTTGCATTGTGATTACCATGCTGATTACATAAAATATCATAGTTTTAT	C	intergenic	*MIR6087,GUCY2F*	.	.	.

X	108406795	C	T	intergenic	*MIR6087,GUCY2F*	.	.	.
X	108437398	A	T	intergenic	*MIR6087,GUCY2F*	.	.	.
X	108438666	C	A	intergenic	*MIR6087,GUCY2F*	.	.	.
X	108438667	G	A	intergenic	*MIR6087,GUCY2F*	.	.	.
X	108439831	T	A	intergenic	*MIR6087,GUCY2F*	.	.	.
X	108440479	T	G	intergenic	*MIR6087,GUCY2F*	.	.	.
X	108440480	T	A	intergenic	*MIR6087,GUCY2F*	.	.	.
X	108449499	A	G	intergenic	*MIR6087,GUCY2F*	.	.	.
X	108487021	C	T	intergenic	*MIR6087,GUCY2F*	.	.	.
X	108487022	A	T	intergenic	*MIR6087,GUCY2F*	.	.	.
X	108488466	T	C	intergenic	*MIR6087,GUCY2F*	.	.	.
X	108490455	C	T	intergenic	*MIR6087,GUCY2F*	.	.	.
X	108560978	T	C	intergenic	*MIR6087,GUCY2F*	.	.	.
X	108560979	T	C	intergenic	*MIR6087,GUCY2F*	.	.	.
X	108562698	G	GT	intergenic	*MIR6087,GUCY2F*	.	.	.
X	108574447	A	C	intergenic	*MIR6087,GUCY2F*	.	.	.
X	108574764	A	C	intergenic	*MIR6087,GUCY2F*	.	.	.
X	108576270	T	A	intergenic	*MIR6087,GUCY2F*	.	.	.
11	56555587	A	G	intergenic	*MIR6128,LOC101927120*	.	.	.
11	56527625	G	A	intergenic	*MIR6128,LOC101927120*	.	.	.
11	56561085	A	T	intergenic	*MIR6128,LOC101927120*	.	.	.
11	56588642	A	G	intergenic	*MIR6128,LOC101927120*	.	.	.
9	76411247	A	C	ncRNA_intronic	*MIR6130*	.	.	.
9	76440061	A	G	ncRNA_intronic	*MIR6130*	.	.	.
9	76892885	C	G	ncRNA_intronic	*MIR6130*	.	.	.
9	76781909	A	C	ncRNA_intronic	*MIR6130*	.	.	.
9	76529806	T	A	ncRNA_intronic	*MIR6130*	.	.	.
9	76744632	G	A	ncRNA_intronic	*MIR6130*	.	.	.
9	76744633	G	C	ncRNA_intronic	*MIR6130*	.	.	.
9	76774872	C	CAAAAAAA	ncRNA_intronic	*MIR6130*	.	.	.
9	76783190	A	AT	ncRNA_intronic	*MIR6130*	.	.	.
X	28554232	TTGTG	T	intergenic	*MIR6134,IL1RAPL1*	.	.	.

13	91131327	A	G	intergenic	*MIR622,LINC01049*	.	.	.
13	90988799	G	C	intergenic	*MIR622,LINC01049*	.	.	.
13	91101065	C	T	intergenic	*MIR622,LINC01049*	.	.	.
13	91095840	C	G	intergenic	*MIR622,LINC01049*	.	.	.
13	91093496	C	T	intergenic	*MIR622,LINC01049*	.	.	.
20	58745750	G	T	ncRNA_intronic	*MIR646HG*	.	.	.
20	58756948	C	T	ncRNA_intronic	*MIR646HG*	.	.	.
20	59021380	C	A	intergenic	*MIR646HG,LOC101928048*	.	.	.
20	58942949	A	G	intergenic	*MIR646HG,LOC101928048*	.	.	.
20	58944641	T	C	intergenic	*MIR646HG,LOC101928048*	.	.	.
22	23173374	T	A	intergenic	*MIR650,MIR5571*	.	.	.
20	26177587	A	G	ncRNA_intronic	*MIR663AHG*	.	.	.
20	26183190	T	G	ncRNA_intronic	*MIR663AHG*	.	.	.
20	26245643	T	A	intergenic	*MIR663AHG,NONE*	.	.	.
20	26252681	CA	C	intergenic	*MIR663AHG,NONE*	.	.	.
3	94459657	ATGTGTG	A	intergenic	*MIR6730,LINC00879*	.	.	.
3	94460764	A	G	intergenic	*MIR6730,LINC00879*	.	.	.
3	94510435	A	T	intergenic	*MIR6730,LINC00879*	.	.	.
3	94470711	A	G	intergenic	*MIR6730,LINC00879*	.	.	.
3	94353845	A	G	intergenic	*MIR6730,LINC00879*	.	.	.
3	94354225	T	C	intergenic	*MIR6730,LINC00879*	.	.	.
11	104294193	A	G	intergenic	*MIR7641-1,LOC102723895*	.	.	.

11	104399795	G	C	intergenic	*MIR7641-1,LOC102723895*	.	.	.
11	104413914	A	G	intergenic	*MIR7641-1,LOC102723895*	.	.	.
11	104156185	C	T	intergenic	*MIR7641-1,LOC102723895*	.	.	.
11	104303589	G	T	intergenic	*MIR7641-1,LOC102723895*	.	.	.
11	104370457	A	C	intergenic	*MIR7641-1,LOC102723895*	.	.	.
11	104370459	G	A	intergenic	*MIR7641-1,LOC102723895*	.	.	.
11	104415353	G	A	intergenic	*MIR7641-1,LOC102723895*	.	.	.
1	228762162	C	A	upstream	*MIR7641-2*	.	.	.
1	228762163	G	A	upstream	*MIR7641-2*	.	.	.
6	4507655	C	G	intergenic	*MIR7641-2,KU-MEL-3*	.	.	.
6	4507656	T	A	intergenic	*MIR7641-2,KU-MEL-3*	.	.	.
6	4573183	G	A	intergenic	*MIR7641-2,KU-MEL-3*	.	.	.
8	93285649	C	A	intergenic	*MIR7641-2,LOC102724710*	.	.	.
8	93492111	G	T	intergenic	*MIR7641-2,LOC102724710*	.	.	.
8	93212744	C	A	intergenic	*MIR7641-2,LOC102724710*	.	.	.
8	93229293	C	T	intergenic	*MIR7641-2,LOC102724710*	.	.	.
8	93229479	T	G	intergenic	*MIR7641-2,LOC102724710*	.	.	.
8	93234646	G	T	intergenic	*MIR7641-2,LOC102724710*	.	.	.
8	93322629	C	T	intergenic	*MIR7641-2,LOC102724710*	.	.	.
8	93323679	A	C	intergenic	*MIR7641-2,LOC102724710*	.	.	.

8	93324677	G	C	intergenic	*MIR7641-2,LOC102724710*	.	.	.
8	93365817	G	A	intergenic	*MIR7641-2,LOC102724710*	.	.	.
8	93376476	A	T	intergenic	*MIR7641-2,LOC102724710*	.	.	.
8	93377434	T	C	intergenic	*MIR7641-2,LOC102724710*	.	.	.
8	93419085	A	G	intergenic	*MIR7641-2,LOC102724710*	.	.	.
14	107171274	A	G	intergenic	*MIR7641-2,MIR5195*	.	.	.
14	107105067	C	T	intergenic	*MIR7641-2,MIR5195*	.	.	.
14	107140089	T	C	intergenic	*MIR7641-2,MIR5195*	.	.	.
6	153756435	C	G	intergenic	*MIR7641-2,OPRM1*	.	.	.
6	153756436	C	T	intergenic	*MIR7641-2,OPRM1*	.	.	.
6	153852877	C	T	intergenic	*MIR7641-2,OPRM1*	.	.	.
6	153958131	A	G	intergenic	*MIR7641-2,OPRM1*	.	.	.
6	154013082	A	G	intergenic	*MIR7641-2,OPRM1*	.	.	.
6	154014700	A	G	intergenic	*MIR7641-2,OPRM1*	.	.	.
6	154051802	G	A	intergenic	*MIR7641-2,OPRM1*	.	.	.
6	154052541	T	A	intergenic	*MIR7641-2,OPRM1*	.	.	.
6	154052542	A	G	intergenic	*MIR7641-2,OPRM1*	.	.	.
6	154068081	C	T	intergenic	*MIR7641-2,OPRM1*	.	.	.
6	154106532	G	A	intergenic	*MIR7641-2,OPRM1*	.	.	.
6	154189042	C	A	intergenic	*MIR7641-2,OPRM1*	.	.	.
6	154257659	C	T	intergenic	*MIR7641-2,OPRM1*	.	.	.
6	154257660	C	T	intergenic	*MIR7641-2,OPRM1*	.	.	.
6	154257962	C	T	intergenic	*MIR7641-2,OPRM1*	.	.	.
6	154257963	C	T	intergenic	*MIR7641-2,OPRM1*	.	.	.
6	154258603	C	T	intergenic	*MIR7641-2,OPRM1*	.	.	.
6	154258605	C	T	intergenic	*MIR7641-2,OPRM1*	.	.	.
2	133846010	G	A	ncRNA_intronic	*MIR7853*	.	.	.
2	133713345	A	C	ncRNA_intronic	*MIR7853*	.	.	.
2	133960723	G	C	ncRNA_intronic	*MIR7853*	.	.	.
2	134057565	C	T	ncRNA_intronic	*MIR7853*	.	.	.

2	134065723	G	C	ncRNA_intronic	*MIR7853*	.	.	.
2	134143885	A	G	ncRNA_intronic	*MIR7853*	.	.	.
2	134143887	T	C	ncRNA_intronic	*MIR7853*	.	.	.
2	134161458	ATGTGTG	A	ncRNA_intronic	*MIR7853*	.	.	.
21	37237944	TAAAAGA	T	intergenic	*MIR802,LOC101928269*	.	.	.
21	37275012	C	T	intergenic	*MIR802,LOC101928269*	.	.	.
11	24033467	G	A	intergenic	*MIR8054,LUZP2*	.	.	.
11	24248100	G	T	intergenic	*MIR8054,LUZP2*	.	.	.
11	23729010	T	C	intergenic	*MIR8054,LUZP2*	.	.	.
11	23818429	T	G	intergenic	*MIR8054,LUZP2*	.	.	.
11	24490081	G	T	intergenic	*MIR8054,LUZP2*	.	.	.
11	23618576	CGAGA	C	intergenic	*MIR8054,LUZP2*	.	.	.
11	23456028	G	C	intergenic	*MIR8054,LUZP2*	.	.	.
11	23456112	A	T	intergenic	*MIR8054,LUZP2*	.	.	.
11	23466936	G	A	intergenic	*MIR8054,LUZP2*	.	.	.
11	23589207	C	T	intergenic	*MIR8054,LUZP2*	.	.	.
11	23697310	G	A	intergenic	*MIR8054,LUZP2*	.	.	.
11	23813573	G	C	intergenic	*MIR8054,LUZP2*	.	.	.
11	24242521	T	A	intergenic	*MIR8054,LUZP2*	.	.	.
11	24243009	C	A	intergenic	*MIR8054,LUZP2*	.	.	.
11	24289754	C	T	intergenic	*MIR8054,LUZP2*	.	.	.
11	24305490	T	C	intergenic	*MIR8054,LUZP2*	.	.	.
11	24312372	C	G	intergenic	*MIR8054,LUZP2*	.	.	.
5	172894208	A	T	intergenic	*MIR8056,LOC285593*	.	.	.
17	48885698	T	A	intergenic	*MIR8059,WFIKKN2*	.	.	.
3	96159430	T	C	intergenic	*MIR8060,EPHA6*	.	.	.
3	96135348	T	C	intergenic	*MIR8060,EPHA6*	.	.	.
3	96208879	C	T	intergenic	*MIR8060,EPHA6*	.	.	.
3	96345329	T	C	intergenic	*MIR8060,EPHA6*	.	.	.
3	96518870	T	G	intergenic	*MIR8060,EPHA6*	.	.	.
20	7580957	A	T	intergenic	*MIR8062,HAO1*	.	.	.
20	7437844	C	T	intergenic	*MIR8062,HAO1*	.	.	.
20	7649807	G	T	intergenic	*MIR8062,HAO1*	.	.	.
20	7389043	G	T	intergenic	*MIR8062,HAO1*	.	.	.
20	7761578	C	T	intergenic	*MIR8062,HAO1*	.	.	.

20	7720628	GAA	G	intergenic	*MIR8062,HAO1*	.	.	.
20	7384042	C	T	intergenic	*MIR8062,HAO1*	.	.	.
20	7576073	T	G	intergenic	*MIR8062,HAO1*	.	.	.
16	5860357	G	T	intergenic	*MIR8065,RBFOX1*	.	.	.
16	5797285	G	T	intergenic	*MIR8065,RBFOX1*	.	.	.
16	6031259	T	A	intergenic	*MIR8065,RBFOX1*	.	.	.
16	5709260	C	G	intergenic	*MIR8065,RBFOX1*	.	.	.
16	5795560	G	A	intergenic	*MIR8065,RBFOX1*	.	.	.
16	5801920	C	A	intergenic	*MIR8065,RBFOX1*	.	.	.
16	5858796	A	C	intergenic	*MIR8065,RBFOX1*	.	.	.
11	29366032	AATAT	A	intergenic	*MIR8068,LINC01616*	.	.	.
11	28601039	G	T	intergenic	*MIR8068,LINC01616*	.	.	.
11	28636381	G	A	intergenic	*MIR8068,LINC01616*	.	.	.
11	29135862	T	C	intergenic	*MIR8068,LINC01616*	.	.	.
11	29038980	C	A	intergenic	*MIR8068,LINC01616*	.	.	.
11	29305836	A	G	intergenic	*MIR8068,LINC01616*	.	.	.
11	29513781	C	T	intergenic	*MIR8068,LINC01616*	.	.	.
11	28976855	A	C	intergenic	*MIR8068,LINC01616*	.	.	.
11	28654786	CAGAGAG	C	intergenic	*MIR8068,LINC01616*	.	.	.
11	28662360	C	T	intergenic	*MIR8068,LINC01616*	.	.	.
11	28663128	T	C	intergenic	*MIR8068,LINC01616*	.	.	.
11	28665237	G	A	intergenic	*MIR8068,LINC01616*	.	.	.
11	28665460	G	C	intergenic	*MIR8068,LINC01616*	.	.	.
11	28811498	G	C	intergenic	*MIR8068,LINC01616*	.	.	.

11	28903511	T	C	intergenic	*MIR8068,LINC01616*	.	.	.
11	28904047	C	T	intergenic	*MIR8068,LINC01616*	.	.	.
11	28904049	T	A	intergenic	*MIR8068,LINC01616*	.	.	.
11	28906077	G	A	intergenic	*MIR8068,LINC01616*	.	.	.
11	28974281	T	C	intergenic	*MIR8068,LINC01616*	.	.	.
11	29070453	G	A	intergenic	*MIR8068,LINC01616*	.	.	.
11	29076137	A	G	intergenic	*MIR8068,LINC01616*	.	.	.
11	29384498	C	T	intergenic	*MIR8068,LINC01616*	.	.	.
11	29391125	C	T	intergenic	*MIR8068,LINC01616*	.	.	.
11	29408187	A	G	intergenic	*MIR8068,LINC01616*	.	.	.
11	29422058	C	A	intergenic	*MIR8068,LINC01616*	.	.	.
11	29440498	C	A	intergenic	*MIR8068,LINC01616*	.	.	.
11	29440499	T	G	intergenic	*MIR8068,LINC01616*	.	.	.
11	29441029	G	C	intergenic	*MIR8068,LINC01616*	.	.	.
11	29450281	A	T	intergenic	*MIR8068,LINC01616*	.	.	.
11	29595070	A	G	intergenic	*MIR8068,LINC01616*	.	.	.
11	29688518	A	C	intergenic	*MIR8068,LINC01616*	.	.	.
11	29688790	C	T	intergenic	*MIR8068,LINC01616*	.	.	.
11	29688791	A	C	intergenic	*MIR8068,LINC01616*	.	.	.

11	29689581	C	CA	intergenic	*MIR8068,LINC01616*	.	.	.
11	29792381	A	G	intergenic	*MIR8068,LINC01616*	.	.	.
11	29811015	C	T	intergenic	*MIR8068,LINC01616*	.	.	.
11	29820221	T	C	intergenic	*MIR8068,LINC01616*	.	.	.
11	29820222	C	T	intergenic	*MIR8068,LINC01616*	.	.	.
11	29890308	T	A	intergenic	*MIR8068,LINC01616*	.	.	.
21	15211152	A	T	intergenic	*MIR8069-1,CYP4F29P*	.	.	.
21	15202609	A	G	intergenic	*MIR8069-1,CYP4F29P*	.	.	.
11	11819125	G	A	intergenic	*MIR8070,USP47*	.	.	.
11	11826484	T	C	intergenic	*MIR8070,USP47*	.	.	.
14	106128840	T	C	intergenic	*MIR8071-2,ELK2AP*	.	.	.
14	106124191	A	G	intergenic	*MIR8071-2,ELK2AP*	.	.	.
13	44903058	A	G	intergenic	*MIR8079,SERP2*	.	.	.
9	109370010	G	C	intergenic	*MIR8081,LINC01505*	.	.	.
9	109370984	G	A	intergenic	*MIR8081,LINC01505*	.	.	.
X	52087164	G	A	intergenic	*MIR8088,XAGE2*	.	.	.
X	52092998	A	T	intergenic	*MIR8088,XAGE2*	.	.	.
X	145414873	TTGTG	T	intergenic	*MIR891A,CXorf51A*	.	.	.
X	145293738	A	ATTG	intergenic	*MIR891A,CXorf51A*	.	.	.
X	145293740	T	G	intergenic	*MIR891A,CXorf51A*	.	.	.
X	145853382	T	C	intergenic	*MIR891A,CXorf51A*	.	.	.

X	145297402	C	T	intergenic	*MIR891A,CXorf51A*	.	.	.
X	145548074	A	T	intergenic	*MIR891A,CXorf51A*	.	.	.
X	145199934	A	C	intergenic	*MIR891A,CXorf51A*	.	.	.
X	145203217	C	T	intergenic	*MIR891A,CXorf51A*	.	.	.
X	145203713	A	G	intergenic	*MIR891A,CXorf51A*	.	.	.
X	145293525	C	CA	intergenic	*MIR891A,CXorf51A*	.	.	.
X	145311317	C	A	intergenic	*MIR891A,CXorf51A*	.	.	.
X	145313234	G	C	intergenic	*MIR891A,CXorf51A*	.	.	.
X	145401964	C	T	intergenic	*MIR891A,CXorf51A*	.	.	.
X	145401965	A	G	intergenic	*MIR891A,CXorf51A*	.	.	.
X	145700097	A	G	intergenic	*MIR891A,CXorf51A*	.	.	.
18	37227048	C	A	ncRNA_intronic	*MIR924HG*	.	.	.
18	36928409	C	A	ncRNA_intronic	*MIR924HG*	.	.	.
18	37090309	T	A	ncRNA_intronic	*MIR924HG*	.	.	.
18	36795625	A	G	ncRNA_intronic	*MIR924HG*	.	.	.
18	36849659	C	T	ncRNA_intronic	*MIR924HG*	.	.	.
18	37145912	G	C	ncRNA_intronic	*MIR924HG*	.	.	.
18	37284961	A	G	ncRNA_intronic	*MIR924HG*	.	.	.
18	37145259	G	T	ncRNA_intronic	*MIR924HG*	.	.	.
18	37206274	C	A	ncRNA_intronic	*MIR924HG*	.	.	.
18	36972484	GAGCA	G	ncRNA_intronic	*MIR924HG*	.	.	.
18	36975409	A	G	ncRNA_intronic	*MIR924HG*	.	.	.
18	37345923	T	TA	intergenic	*MIR924HG,LINC01477*	.	.	.
18	37523339	T	C	intergenic	*MIR924HG,LINC01477*	.	.	.

18	37351196	G	T	intergenic	*MIR924HG,LINC01477*	.	.	.
18	37504832	G	T	intergenic	*MIR924HG,LINC01477*	.	.	.
18	37623015	T	G	intergenic	*MIR924HG,LINC01477*	.	.	.
18	37575555	C	A	intergenic	*MIR924HG,LINC01477*	.	.	.
18	37389414	A	G	intergenic	*MIR924HG,LINC01477*	.	.	.
21	17593907	T	C	ncRNA_intronic	*MIR99AHG*	.	.	.
21	17686364	T	TA	ncRNA_intronic	*MIR99AHG*	.	.	.
21	17506251	C	A	ncRNA_intronic	*MIR99AHG*	.	.	.
21	17681555	C	A	ncRNA_intronic	*MIR99AHG*	.	.	.
21	17599405	A	T	ncRNA_intronic	*MIR99AHG*	.	.	.
21	17600267	G	A	ncRNA_intronic	*MIR99AHG*	.	.	.
21	17602077	T	C	ncRNA_intronic	*MIR99AHG*	.	.	.
21	17602079	T	A	ncRNA_intronic	*MIR99AHG*	.	.	.
21	17602414	T	C	ncRNA_intronic	*MIR99AHG*	.	.	.
21	17602415	G	A	ncRNA_intronic	*MIR99AHG*	.	.	.
21	17783084	C	T	ncRNA_intronic	*MIR99AHG*	.	.	.
21	17783086	G	T	ncRNA_intronic	*MIR99AHG*	.	.	.
21	17875719	A	G	ncRNA_intronic	*MIR99AHG*	.	.	.
21	17875720	G	T	ncRNA_intronic	*MIR99AHG*	.	.	.
21	18330646	C	G	intergenic	*MIR99AHG,LINC01549*	.	.	.
21	18265873	CCCCAATACAA	C	intergenic	*MIR99AHG,LINC01549*	.	.	.
21	18572889	C	A	intergenic	*MIR99AHG,LINC01549*	.	.	.
21	18106144	C	A	intergenic	*MIR99AHG,LINC01549*	.	.	.
21	18106621	G	A	intergenic	*MIR99AHG,LINC01549*	.	.	.
21	18255823	A	T	intergenic	*MIR99AHG,LINC01549*	.	.	.
21	18411314	A	G	intergenic	*MIR99AHG,LINC01549*	.	.	.

21	18411552	C	T	intergenic	*MIR99AHG,LINC01549*	.	.	.
21	18411553	A	T	intergenic	*MIR99AHG,LINC01549*	.	.	.
21	18481142	T	A	intergenic	*MIR99AHG,LINC01549*	.	.	.
21	18481143	G	A	intergenic	*MIR99AHG,LINC01549*	.	.	.
21	18528659	G	A	intergenic	*MIR99AHG,LINC01549*	.	.	.
21	18537323	G	A	intergenic	*MIR99AHG,LINC01549*	.	.	.
21	18538179	A	T	intergenic	*MIR99AHG,LINC01549*	.	.	.
21	18538523	C	T	intergenic	*MIR99AHG,LINC01549*	.	.	.
21	18545513	A	G	intergenic	*MIR99AHG,LINC01549*	.	.	.
21	18570199	G	GGGAGGA	intergenic	*MIR99AHG,LINC01549*	.	.	.
21	18617261	A	G	intergenic	*MIR99AHG,LINC01549*	.	.	.
21	18618574	G	A	intergenic	*MIR99AHG,LINC01549*	.	.	.
21	18648009	A	G	intergenic	*MIR99AHG,LINC01549*	.	.	.
21	18658555	G	A	intergenic	*MIR99AHG,LINC01549*	.	.	.
21	18684599	T	C	intergenic	*MIR99AHG,LINC01549*	.	.	.
21	18684600	C	T	intergenic	*MIR99AHG,LINC01549*	.	.	.
21	18684672	A	C	intergenic	*MIR99AHG,LINC01549*	.	.	.
21	18747220	A	G	intergenic	*MIR99AHG,LINC01549*	.	.	.
21	18747222	A	T	intergenic	*MIR99AHG,LINC01549*	.	.	.

21	18754521	T	C	intergenic	*MIR99AHG,LINC01549*	.	.	.
9	96981782	A	C	intergenic	*MIRLET7DHG,ZNF169*	.	.	.
9	97002995	C	T	intergenic	*MIRLET7DHG,ZNF169*	.	.	.
14	45983973	T	C	intergenic	*MIS18BP1,LINC00871*	.	.	.
14	46034052	CCTCCCATCTTACCTACTGAGTAA	C	intergenic	*MIS18BP1,LINC00871*	.	.	.
14	46156880	A	G	intergenic	*MIS18BP1,LINC00871*	.	.	.
14	45791282	C	T	intergenic	*MIS18BP1,LINC00871*	.	.	.
14	45792255	T	A	intergenic	*MIS18BP1,LINC00871*	.	.	.
14	45792257	C	G	intergenic	*MIS18BP1,LINC00871*	.	.	.
14	45882494	T	A	intergenic	*MIS18BP1,LINC00871*	.	.	.
14	45885878	G	C	intergenic	*MIS18BP1,LINC00871*	.	.	.
14	45944834	C	A	intergenic	*MIS18BP1,LINC00871*	.	.	.
14	45945145	C	G	intergenic	*MIS18BP1,LINC00871*	.	.	.
14	45945147	T	C	intergenic	*MIS18BP1,LINC00871*	.	.	.
14	46060972	T	C	intergenic	*MIS18BP1,LINC00871*	.	.	.
14	46060974	T	C	intergenic	*MIS18BP1,LINC00871*	.	.	.
14	46067041	A	G	intergenic	*MIS18BP1,LINC00871*	.	.	.
14	46138645	C	T	intergenic	*MIS18BP1,LINC00871*	.	.	.

14	46220088	A	G	intergenic	*MIS18BP1,LINC00871*	.	.	.
14	46220438	A	C	intergenic	*MIS18BP1,LINC00871*	.	.	.
14	46469473	G	A	intergenic	*MIS18BP1,LINC00871*	.	.	.
14	46469675	T	TA	intergenic	*MIS18BP1,LINC00871*	.	.	.
19	780787	C	T	intergenic	*MISP,PTBP1*	.	.	.
10	130058617	A	G	intergenic	*MKI67,LINC01163*	.	.	.
10	130023869	G	A	intergenic	*MKI67,LINC01163*	.	.	.
10	130023871	A	G	intergenic	*MKI67,LINC01163*	.	.	.
10	130025477	T	C	intergenic	*MKI67,LINC01163*	.	.	.
20	10394310	T	C	UTR5	*MKKS*	.	.	.
22	40837294	T	C	intronic	*MKL1*	.	.	.
22	41014705	C	T	intronic	*MKL1*	.	.	.
16	14165405	G	C	intronic	*MKL2*	.	.	.
16	14225601	T	A	intronic	*MKL2*	.	.	.
16	14261269	T	G	intronic	*MKL2*	.	.	.
16	14261270	C	T	intronic	*MKL2*	.	.	.
7	130971265	A	G	intronic	*MKLN1*	.	.	.
7	130919264	G	C	intronic	*MKLN1*	.	.	.
7	130905092	A	AT	intronic	*MKLN1*	.	.	.
7	131102925	C	T	intronic	*MKLN1*	.	.	.
7	131102926	T	G	intronic	*MKLN1*	.	.	.
7	131004106	C	CAT	ncRNA_intronic	*MKLN1-AS*	.	.	.
7	131005324	A	C	ncRNA_intronic	*MKLN1-AS*	.	.	.
7	140190743	C	T	intergenic	*MKRN1,DENND2A*	.	.	.
7	140190744	C	G	intergenic	*MKRN1,DENND2A*	.	.	.
20	45099335	T	C	intergenic	*MKRN7P,ZNF334*	.	.	.
12	88313534	A	C	intergenic	*MKRN9P,C12orf50*	.	.	.
12	88232815	G	A	intergenic	*MKRN9P,C12orf50*	.	.	.
12	88268922	G	A	intergenic	*MKRN9P,C12orf50*	.	.	.
12	88268923	T	C	intergenic	*MKRN9P,C12orf50*	.	.	.
12	88339101	T	A	intergenic	*MKRN9P,C12orf50*	.	.	.
12	88339102	A	G	intergenic	*MKRN9P,C12orf50*	.	.	.
12	88340310	A	T	intergenic	*MKRN9P,C12orf50*	.	.	.
12	88341517	A	G	intergenic	*MKRN9P,C12orf50*	.	.	.

10	27973250	GAA	G	intronic	*MKX*	.	.	.
9	5901702	G	A	intronic	*MLANA*	.	.	.
6	54023365	A	C	intronic	*MLIP*	.	.	.
6	54163478	A	G	intergenic	*MLIP,TINAG*	.	.	.
6	54163934	G	A	intergenic	*MLIP,TINAG*	.	.	.
2	174077092	A	G	ncRNA_intronic	*MLK7-AS1*	.	.	.
2	174193554	T	C	intergenic	*MLK7-AS1,CDCA7*	.	.	.
2	174188666	T	C	intergenic	*MLK7-AS1,CDCA7*	.	.	.
20	29642349	A	G	intergenic	*MLLT10P1,DEFB115*	.	.	.
9	20374333	G	A	intronic	*MLLT3*	.	.	.
6	33850578	T	C	intergenic	*MLN,LINC01016*	.	.	.
12	122605876	A	T	intronic	*MLXIP*	.	.	.
17	53667418	A	G	intergenic	*MMD,TMEM100*	.	.	.
17	53541894	C	A	intergenic	*MMD,TMEM100*	.	.	.
17	53633938	A	C	intergenic	*MMD,TMEM100*	.	.	.
14	23337503	T	C	intergenic	*MMP14,LRP10*	.	.	.
8	89078596	G	A	intronic	*MMP16*	.	.	.
8	89206193	C	CA	intronic	*MMP16*	.	.	.
8	89196001	A	G	intronic	*MMP16*	.	.	.
8	89234753	C	G	intronic	*MMP16*	.	.	.
8	89299005	C	T	intronic	*MMP16*	.	.	.
8	89509864	G	A	intergenic	*MMP16,LOC101929709*	.	.	.
8	90030276	A	G	intergenic	*MMP16,LOC101929709*	.	.	.
8	90515227	C	T	intergenic	*MMP16,LOC101929709*	.	.	.
8	90662322	A	C	intergenic	*MMP16,LOC101929709*	.	.	.
8	89548840	T	A	intergenic	*MMP16,LOC101929709*	.	.	.
8	89862634	G	T	intergenic	*MMP16,LOC101929709*	.	.	.
8	89993995	C	A	intergenic	*MMP16,LOC101929709*	.	.	.
8	89450112	T	C	intergenic	*MMP16,LOC101929709*	.	.	.

8	89913181	T	TCATA	intergenic	*MMP16,LOC101929709*	.	.	.
8	89496481	A	G	intergenic	*MMP16,LOC101929709*	.	.	.
8	89496483	T	G	intergenic	*MMP16,LOC101929709*	.	.	.
8	89504305	T	G	intergenic	*MMP16,LOC101929709*	.	.	.
8	89511813	C	T	intergenic	*MMP16,LOC101929709*	.	.	.
8	89511814	A	G	intergenic	*MMP16,LOC101929709*	.	.	.
8	89512262	A	G	intergenic	*MMP16,LOC101929709*	.	.	.
8	89539628	G	T	intergenic	*MMP16,LOC101929709*	.	.	.
8	89544883	C	G	intergenic	*MMP16,LOC101929709*	.	.	.
8	89544884	A	G	intergenic	*MMP16,LOC101929709*	.	.	.
8	89590911	A	C	intergenic	*MMP16,LOC101929709*	.	.	.
8	89659300	T	C	intergenic	*MMP16,LOC101929709*	.	.	.
8	89839090	G	T	intergenic	*MMP16,LOC101929709*	.	.	.
8	89839222	A	G	intergenic	*MMP16,LOC101929709*	.	.	.
8	89913407	T	C	intergenic	*MMP16,LOC101929709*	.	.	.
8	89913408	G	C	intergenic	*MMP16,LOC101929709*	.	.	.
8	89942358	CA	C	intergenic	*MMP16,LOC101929709*	.	.	.
8	90035526	G	A	intergenic	*MMP16,LOC101929709*	.	.	.
8	90164668	T	A	intergenic	*MMP16,LOC101929709*	.	.	.

8	90177883	G	A	intergenic	*MMP16,LOC101929709*	.	.	.
8	90371018	G	A	intergenic	*MMP16,LOC101929709*	.	.	.
8	90390777	GA	G	intergenic	*MMP16,LOC101929709*	.	.	.
8	90402853	C	T	intergenic	*MMP16,LOC101929709*	.	.	.
8	90472906	T	A	intergenic	*MMP16,LOC101929709*	.	.	.
8	90523563	G	T	intergenic	*MMP16,LOC101929709*	.	.	.
8	90523728	T	C	intergenic	*MMP16,LOC101929709*	.	.	.
8	90673048	C	G	intergenic	*MMP16,LOC101929709*	.	.	.
8	90673049	C	T	intergenic	*MMP16,LOC101929709*	.	.	.
8	90699641	T	A	intergenic	*MMP16,LOC101929709*	.	.	.
8	90699642	C	A	intergenic	*MMP16,LOC101929709*	.	.	.
8	90723203	C	T	intergenic	*MMP16,LOC101929709*	.	.	.
8	90723498	G	A	intergenic	*MMP16,LOC101929709*	.	.	.
12	56282004	T	C	intergenic	*MMP19,PYM1*	.	.	.
11	102521141	C	T	intergenic	*MMP20,MMP27*	.	.	.
11	102418113	T	C	intergenic	*MMP7,MMP20*	.	.	.
11	102639146	A	G	intergenic	*MMP8,MMP10*	.	.	.
4	90901983	G	A	intergenic	*MMRN1,CCSER1*	.	.	.
4	90955986	G	T	intergenic	*MMRN1,CCSER1*	.	.	.
14	61319702	G	C	intronic	*MNAT1*	.	.	.
14	61319704	C	T	intronic	*MNAT1*	.	.	.
14	61324796	T	G	intronic	*MNAT1*	.	.	.
14	61356164	T	C	intronic	*MNAT1*	.	.	.
14	61375539	T	C	intronic	*MNAT1*	.	.	.
14	61376944	C	T	intronic	*MNAT1*	.	.	.

14	61406611	G	T	intronic	*MNAT1*	.	.	.
4	154351679	G	A	intergenic	*MND1,KIAA0922*	.	.	.
1	158833785	C	T	intergenic	*MNDA,PYHIN1*	.	.	.
1	158837531	G	A	intergenic	*MNDA,PYHIN1*	.	.	.
1	158838889	T	C	intergenic	*MNDA,PYHIN1*	.	.	.
1	158838891	T	A	intergenic	*MNDA,PYHIN1*	.	.	.
1	158839274	G	C	intergenic	*MNDA,PYHIN1*	.	.	.
15	56856572	T	C	intergenic	*MNS1,ZNF280D*	.	.	.
15	56856573	G	A	intergenic	*MNS1,ZNF280D*	.	.	.
15	56866419	C	T	intergenic	*MNS1,ZNF280D*	.	.	.
7	156898676	A	T	intergenic	*MNX1-AS1,UBE3C*	.	.	.
9	27431750	T	C	intronic	*MOB3B*	.	.	.
3	39529042	T	C	intronic	*MOBP*	.	.	.
3	39597656	G	A	intergenic	*MOBP,MYRIP*	.	.	.
3	39657624	T	A	intergenic	*MOBP,MYRIP*	.	.	.
3	39658242	T	A	intergenic	*MOBP,MYRIP*	.	.	.
3	39658243	G	A	intergenic	*MOBP,MYRIP*	.	.	.
3	39659501	C	A	intergenic	*MOBP,MYRIP*	.	.	.
3	39659503	C	A	intergenic	*MOBP,MYRIP*	.	.	.
3	39780546	G	A	intergenic	*MOBP,MYRIP*	.	.	.
18	33863867	CAA	C	intergenic	*MOCOS,FHOD3*	.	.	.
6	39876964	A	G	intronic	*MOCS1*	.	.	.
6	39876966	A	G	intronic	*MOCS1*	.	.	.
5	52398701	T	C	intronic	*MOCS2*	.	.	.
2	223575977	G	A	intergenic	*MOGAT1,ACSL3*	.	.	.
2	223650708	C	T	intergenic	*MOGAT1,ACSL3*	.	.	.
3	108681585	A	G	intronic	*MORC1*	.	.	.
X	106211729	A	C	intronic	*MORC4*	.	.	.
X	102952427	G	C	intergenic	*MORF4L2-AS1,GLRA4*	.	.	.
9	124929325	A	C	intronic	*MORN5*	.	.	.
9	124944157	C	T	intronic	*MORN5*	.	.	.
8	57052358	A	G	intergenic	*MOS,PLAG1*	.	.	.
8	57060247	A	G	intergenic	*MOS,PLAG1*	.	.	.
X	134071597	A	G	intergenic	*MOSPD1,SMIM10*	.	.	.
X	14974796	T	C	intergenic	*MOSPD2,ASB9*	.	.	.
X	15228561	TTGTGTG	T	intergenic	*MOSPD2,ASB9*	.	.	.
X	14966288	G	A	intergenic	*MOSPD2,ASB9*	.	.	.

X	14989824	G	T	intergenic	*MOSPD2,ASB9*	.	.	.
X	15017731	T	C	intergenic	*MOSPD2,ASB9*	.	.	.
X	15074607	C	A	intergenic	*MOSPD2,ASB9*	.	.	.
6	132718775	CAGAAAGAA	C	intronic	*MOXD1*	.	.	.
6	132634926	C	T	intronic	*MOXD1*	.	.	.
9	13305535	G	T	intergenic	*MPDZ,FLJ41200*	.	.	.
11	59006188	G	T	intergenic	*MPEG1,OR5AN1*	.	.	.
11	59083426	T	C	intergenic	*MPEG1,OR5AN1*	.	.	.
11	59083783	G	A	intergenic	*MPEG1,OR5AN1*	.	.	.
11	59084161	C	A	intergenic	*MPEG1,OR5AN1*	.	.	.
11	59085044	G	T	intergenic	*MPEG1,OR5AN1*	.	.	.
11	59086729	A	C	intergenic	*MPEG1,OR5AN1*	.	.	.
11	59111488	A	G	intergenic	*MPEG1,OR5AN1*	.	.	.
11	59113336	C	T	intergenic	*MPEG1,OR5AN1*	.	.	.
16	82454923	C	T	intergenic	*MPHOSPH6,CDH13*	.	.	.
13	20213123	A	T	intronic	*MPHOSPH8*	.	.	.
14	67713557	C	T	intronic	*MPP5*	.	.	.
14	67741059	C	G	intronic	*MPP5*	.	.	.
14	67774588	T	C	intronic	*MPP5*	.	.	.
14	67774590	T	C	intronic	*MPP5*	.	.	.
7	24624309	T	G	intronic	*MPP6*	.	.	.
7	24668013	A	C	intronic	*MPP6*	.	.	.
7	24685864	G	A	intronic	*MPP6*	.	.	.
18	11917926	G	A	intergenic	*MPPE1,IMPA2*	.	.	.
18	11918398	A	G	intergenic	*MPPE1,IMPA2*	.	.	.
11	30578708	C	G	intronic	*MPPED2*	.	.	.
11	30581859	T	TAAA	intronic	*MPPED2*	.	.	.
11	30410822	A	G	intronic	*MPPED2*	.	.	.
11	30647446	A	C	intergenic	*MPPED2,DCDC5*	.	.	.
11	30698195	A	G	intergenic	*MPPED2,DCDC5*	.	.	.
11	30789808	G	A	intergenic	*MPPED2,DCDC5*	.	.	.
11	30698486	G	A	intergenic	*MPPED2,DCDC5*	.	.	.
11	30700224	C	A	intergenic	*MPPED2,DCDC5*	.	.	.
11	30700564	C	A	intergenic	*MPPED2,DCDC5*	.	.	.
11	30700769	T	A	intergenic	*MPPED2,DCDC5*	.	.	.
1	167741553	T	A	exonic	*MPZL1*	stopgain	.	.

11	118119748	C	T	intronic	*MPZL3*	.	.	.
1	181026874	C	T	UTR3	*MR1*	.	.	.
6	84823982	A	G	intergenic	*MRAP2,CEP162*	.	.	.
11	94161196	G	C	intronic	*MRE11A*	.	.	.
2	216842352	C	A	intronic	*MREG*	.	.	.
2	216855263	A	T	intronic	*MREG*	.	.	.
2	216855265	G	A	intronic	*MREG*	.	.	.
2	216855845	T	C	intronic	*MREG*	.	.	.
2	216857651	C	T	intronic	*MREG*	.	.	.
2	216857652	A	G	intronic	*MREG*	.	.	.
4	6656180	C	T	intergenic	*MRFAP1,LOC93622*	.	.	.
4	6656505	T	A	intergenic	*MRFAP1,LOC93622*	.	.	.
4	6656998	T	C	intergenic	*MRFAP1,LOC93622*	.	.	.
11	19017104	C	T	intergenic	*MRGPRX1,MRGPRX2*	.	.	.
11	18963917	A	G	intergenic	*MRGPRX1,MRGPRX2*	.	.	.
11	18969927	T	G	intergenic	*MRGPRX1,MRGPRX2*	.	.	.
11	19085753	T	A	intergenic	*MRGPRX2,ZDHHC13*	.	.	.
11	19087968	T	A	intergenic	*MRGPRX2,ZDHHC13*	.	.	.
11	19088264	C	T	intergenic	*MRGPRX2,ZDHHC13*	.	.	.
11	19088265	A	G	intergenic	*MRGPRX2,ZDHHC13*	.	.	.
11	18147438	G	A	intronic	*MRGPRX3*	.	.	.
17	35241059	C	T	intergenic	*MRM1,LHX1*	.	.	.
5	41025260	C	A	intronic	*MROH2B*	.	.	.
5	41136664	T	C	intergenic	*MROH2B,C6*	.	.	.
5	41137280	C	T	intergenic	*MROH2B,C6*	.	.	.
8	142636392	C	G	intergenic	*MROH5,MIR1302-7*	.	.	.

8	142630520	GGGGCCTT CAGGTGCC GAGGGTG GTGCGCAC CCAGGGAT GCTGGTGG GGATTGGG CAAAGACG TCAACTCC AGGGCTGT GGAAGGA GGCGA	G	intergenic	*MROH5,MIR1302-7*	.	.	.
1	55194282	T	A	ncRNA_intronic	*MROH7-TTC4*	.	.	.
20	35735826	TATAC	T	intronic	*MROH8*	.	.	.
1	170968667	CTATA	C	intronic	*MROH9*	.	.	.
1	170950480	G	A	intronic	*MROH9*	.	.	.
1	170950481	A	G	intronic	*MROH9*	.	.	.
1	170991206	C	T	intronic	*MROH9*	.	.	.
1	171000033	A	C	intronic	*MROH9*	.	.	.
1	171056475	C	CAAAAAAAA	intergenic	*MROH9,FMO3*	.	.	.
4	78839102	G	C	intronic	*MRPL1*	.	.	.
4	78893570	C	A	intergenic	*MRPL1,FRAS1*	.	.	.
6	44080797	T	A	downstream	*MRPL14*	.	.	.
8	55318294	C	T	intergenic	*MRPL15,SOX17*	.	.	.
8	55274078	A	C	intergenic	*MRPL15,SOX17*	.	.	.
5	154330162	C	T	intronic	*MRPL22*	.	.	.
5	154378152	C	A	intergenic	*MRPL22,KIF4B*	.	.	.
2	99809071	A	G	intronic	*MRPL30*	.	.	.
2	99851435	A	G	intergenic	*MRPL30,LYG2*	.	.	.
7	43074970	A	AGACGGAGT	intergenic	*MRPL32,HECW1*	.	.	.
7	43062566	C	T	intergenic	*MRPL32,HECW1*	.	.	.
7	43103149	C	T	intergenic	*MRPL32,HECW1*	.	.	.
12	93953062	A	G	intergenic	*MRPL42,SOCS2-AS1*	.	.	.
12	93954279	C	CAA	intergenic	*MRPL42,SOCS2-AS1*	.	.	.
17	45536571	C	T	ncRNA_intronic	*MRPL45P2*	.	.	.
11	73527418	A	ATTTTTTTTTT	intronic	*MRPL48*	.	.	.

13	21766346	T	TTTTTTTTTT	intergenic	*MRPL57,LINC01046*	.	.	.
1	175026149	G	A	intergenic	*MRPS14,TNN*	.	.	.
1	175029430	A	AATAT	intergenic	*MRPS14,TNN*	.	.	.
20	3046083	C	T	intergenic	*MRPS26,OXT*	.	.	.
5	71596838	A	ACAACACAG	intronic	*MRPS27*	.	.	.
8	80907742	A	G	intronic	*MRPS28*	.	.	.
8	80833438	C	T	intronic	*MRPS28*	.	.	.
8	80917824	C	T	intronic	*MRPS28*	.	.	.
8	80918228	T	G	intronic	*MRPS28*	.	.	.
8	80921366	C	T	intronic	*MRPS28*	.	.	.
5	45190135	T	C	intergenic	*MRPS30,HCN1*	.	.	.
5	44986416	C	T	intergenic	*MRPS30,HCN1*	.	.	.
5	44827638	G	T	intergenic	*MRPS30,HCN1*	.	.	.
5	44817133	C	G	intergenic	*MRPS30,HCN1*	.	.	.
5	45060306	C	G	intergenic	*MRPS30,HCN1*	.	.	.
5	44997928	G	A	intergenic	*MRPS30,HCN1*	.	.	.
5	45048229	C	A	intergenic	*MRPS30,HCN1*	.	.	.
5	45119695	C	A	intergenic	*MRPS30,HCN1*	.	.	.
5	45120221	C	T	intergenic	*MRPS30,HCN1*	.	.	.
5	45121386	A	G	intergenic	*MRPS30,HCN1*	.	.	.
5	45241833	T	G	intergenic	*MRPS30,HCN1*	.	.	.
5	45242528	A	G	intergenic	*MRPS30,HCN1*	.	.	.
13	52843433	T	C	intergenic	*MRPS31P5,LOC103191607*	.	.	.
13	52865361	C	T	intergenic	*MRPS31P5,LOC103191607*	.	.	.
13	52836917	T	C	intergenic	*MRPS31P5,LOC103191607*	.	.	.
13	52789002	T	C	intergenic	*MRPS31P5,LOC103191607*	.	.	.
13	52789003	C	T	intergenic	*MRPS31P5,LOC103191607*	.	.	.
13	52834528	T	C	intergenic	*MRPS31P5,LOC103191607*	.	.	.
7	140771309	A	C	intergenic	*MRPS33,TMEM178B*	.	.	.
2	95760900	C	G	intronic	*MRPS5*	.	.	.

11	10628531	G	A	intronic	*MRVI1*	.	.	.
11	60577942	A	G	intergenic	*MS4A10,CCDC86*	.	.	.
11	60259257	G	GGGGTCAG(	upstream	*MS4A12*	.	.	.
11	60370481	G	A	intergenic	*MS4A13,LINC0030 1*	.	.	.
11	60526413	A	G	intronic	*MS4A15*	.	.	.
11	59853273	C	T	intergenic	*MS4A3,MS4A2*	.	.	.
11	60096828	A	G	intergenic	*MS4A4A,MS4A6E*	.	.	.
11	60142471	C	T	intergenic	*MS4A6E,MS4A7*	.	.	.
11	60142472	T	G	intergenic	*MS4A6E,MS4A7*	.	.	.
4	3290922	A	G	intergenic	*MSANTD1,RGS12*	.	.	.
9	103324270	G	C	intronic	*MSANTD3-TMEFF1,TMEFF1*	.	.	.
9	103264119	A	G	intronic	*MSANTD3-TMEFF1,TMEFF1*	.	.	.
11	105912211	A	T	intergenic	*MSANTD4,KBTBD3*	.	.	.
8	72787193	T	C	ncRNA_intronic	*MSC-AS1*	.	.	.
8	72792351	C	T	ncRNA_intronic	*MSC-AS1*	.	.	.
8	72901310	A	G	ncRNA_intronic	*MSC-AS1*	.	.	.
8	72841509	A	C	ncRNA_intronic	*MSC-AS1*	.	.	.
8	72849566	C	G	ncRNA_intronic	*MSC-AS1*	.	.	.
8	72849567	A	G	ncRNA_intronic	*MSC-AS1*	.	.	.
8	72851300	C	G	ncRNA_intronic	*MSC-AS1*	.	.	.
8	72851301	T	A	ncRNA_intronic	*MSC-AS1*	.	.	.
2	18012536	T	TC	intergenic	*MSGN1,KCNS3*	.	.	.
2	47654713	C	A	intronic	*MSH2*	.	.	.
5	80137100	T	C	intronic	*MSH3*	.	.	.
1	76275524	C	T	intronic	*MSH4*	.	.	.
1	76275525	A	G	intronic	*MSH4*	.	.	.
1	76289588	C	T	intronic	*MSH4*	.	.	.
1	76296310	A	G	intronic	*MSH4*	.	.	.
1	76297485	G	A	intronic	*MSH4*	.	.	.
1	76297677	A	C	intronic	*MSH4*	.	.	.
1	76299751	A	T	intronic	*MSH4*	.	.	.
1	76325116	A	G	intronic	*MSH4*	.	.	.

12	120835427	AATACTTTTTGAGTGTAAGTATGTCCCATGCAATATTTGGGACATACTT	A	intergenic	*MSI1,COX6A1*	.	.	.
17	55503983	C	G	intronic	*MSI2*	.	.	.
17	55624402	T	C	intronic	*MSI2*	.	.	.
X	11900582	T	G	intergenic	*MSL3,FRMPD4*	.	.	.
X	12028171	T	C	intergenic	*MSL3,FRMPD4*	.	.	.
X	12036186	C	A	intergenic	*MSL3,FRMPD4*	.	.	.
X	11899302	A	G	intergenic	*MSL3,FRMPD4*	.	.	.
X	12128458	T	C	intergenic	*MSL3,FRMPD4*	.	.	.
4	166297598	G	T	intergenic	*MSMO1,CPE*	.	.	.
X	64934235	G	T	intronic	*MSN*	.	.	.
X	65194439	A	G	intergenic	*MSN,MIR223*	.	.	.
X	65208012	G	C	intergenic	*MSN,MIR223*	.	.	.
X	65196480	T	A	intergenic	*MSN,MIR223*	.	.	.
X	64981884	T	C	intergenic	*MSN,MIR223*	.	.	.
X	64998701	CA	C	intergenic	*MSN,MIR223*	.	.	.
X	65011512	C	T	intergenic	*MSN,MIR223*	.	.	.
X	65045270	C	A	intergenic	*MSN,MIR223*	.	.	.
X	65053638	T	C	intergenic	*MSN,MIR223*	.	.	.
X	65074887	G	C	intergenic	*MSN,MIR223*	.	.	.
X	65109702	C	T	intergenic	*MSN,MIR223*	.	.	.
X	65110724	C	T	intergenic	*MSN,MIR223*	.	.	.
X	65157977	T	A	intergenic	*MSN,MIR223*	.	.	.
X	65172086	G	A	intergenic	*MSN,MIR223*	.	.	.
X	65184441	G	T	intergenic	*MSN,MIR223*	.	.	.
X	65197218	A	T	intergenic	*MSN,MIR223*	.	.	.
X	65198260	T	C	intergenic	*MSN,MIR223*	.	.	.
X	65203483	C	G	intergenic	*MSN,MIR223*	.	.	.
X	65203484	A	G	intergenic	*MSN,MIR223*	.	.	.
X	65203825	G	A	intergenic	*MSN,MIR223*	.	.	.
X	65203826	C	T	intergenic	*MSN,MIR223*	.	.	.
8	16219478	ACTCTCT	A	intergenic	*MSR1,FGF20*	.	.	.
8	16422512	T	C	intergenic	*MSR1,FGF20*	.	.	.
8	16837515	T	C	intergenic	*MSR1,FGF20*	.	.	.

8	16339195	T	C	intergenic	*MSR1,FGF20*	.	.	.
8	16063535	A	G	intergenic	*MSR1,FGF20*	.	.	.
8	16064694	G	A	intergenic	*MSR1,FGF20*	.	.	.
8	16064696	G	T	intergenic	*MSR1,FGF20*	.	.	.
8	16064801	C	A	intergenic	*MSR1,FGF20*	.	.	.
8	16194656	C	T	intergenic	*MSR1,FGF20*	.	.	.
8	16196069	C	T	intergenic	*MSR1,FGF20*	.	.	.
8	16196301	A	G	intergenic	*MSR1,FGF20*	.	.	.
8	16275581	C	A	intergenic	*MSR1,FGF20*	.	.	.
8	16287788	G	A	intergenic	*MSR1,FGF20*	.	.	.
8	16288082	A	G	intergenic	*MSR1,FGF20*	.	.	.
8	16542585	A	T	intergenic	*MSR1,FGF20*	.	.	.
8	16729625	C	G	intergenic	*MSR1,FGF20*	.	.	.
8	16833226	C	A	intergenic	*MSR1,FGF20*	.	.	.
8	16834258	T	G	intergenic	*MSR1,FGF20*	.	.	.
8	16835626	C	T	intergenic	*MSR1,FGF20*	.	.	.
8	10127319	G	A	intronic	*MSRA*	.	.	.
8	10330509	T	C	intergenic	*MSRA,LINCR-0001*	.	.	.
10	23479491	TACACACACACACACAC	T	intergenic	*MSRB2,PTF1A*	.	.	.
12	65742622	G	T	intronic	*MSRB3*	.	.	.
12	65794462	G	A	intronic	*MSRB3*	.	.	.
1	17124915	T	TTTTTC	intergenic	*MST1L,MIR3675*	.	.	.
2	190919440	G	A	downstream	*MSTN*	.	.	.
2	42722329	C	A	UTR5	*MTA3*	.	.	.
2	42905601	T	C	intronic	*MTA3*	.	.	.
2	42957230	C	G	intronic	*MTA3*	.	.	.
9	21828566	C	T	intronic	*MTAP*	.	.	.
9	21820977	G	A	intronic	*MTAP*	.	.	.
9	21821997	C	T	intronic	*MTAP*	.	.	.
9	21821998	A	G	intronic	*MTAP*	.	.	.
9	21898154	A	G	intergenic	*MTAP,CDKN2A-AS1*	.	.	.
8	121488783	A	G	intronic	*MTBP*	.	.	.
11	47641761	C	T	intronic	*MTCH2*	.	.	.
7	91560675	A	G	intergenic	*MTERF1,AKAP9*	.	.	.
7	91536896	C	A	intergenic	*MTERF1,AKAP9*	.	.	.

7	91538349	C	G	intergenic	*MTERF1,AKAP9*	.	.	.
8	66558637	A	ACTG	intronic	*MTFR1*	.	.	.
6	151355607	G	A	intronic	*MTHFD1L*	.	.	.
4	75075994	C	T	intronic	*MTHFD2L*	.	.	.
4	75075995	A	G	intronic	*MTHFD2L*	.	.	.
3	95523449	C	T	intergenic	*MTHFD2P1,MIR8060*	.	.	.
3	95553485	G	A	intergenic	*MTHFD2P1,MIR8060*	.	.	.
3	95582512	C	T	intergenic	*MTHFD2P1,MIR8060*	.	.	.
3	95582513	A	G	intergenic	*MTHFD2P1,MIR8060*	.	.	.
3	95583968	A	T	intergenic	*MTHFD2P1,MIR8060*	.	.	.
3	95595569	A	AC	intergenic	*MTHFD2P1,MIR8060*	.	.	.
3	95874435	C	G	intergenic	*MTHFD2P1,MIR8060*	.	.	.
3	95874436	C	T	intergenic	*MTHFD2P1,MIR8060*	.	.	.
11	68478022	A	C	intronic	*MTL5*	.	.	.
X	149848512	A	C	intergenic	*MTM1,MTMR1*	.	.	.
X	149891836	G	A	intronic	*MTMR1*	.	.	.
5	32226834	C	T	downstream	*MTMR12*	.	.	.
5	32226836	G	T	downstream	*MTMR12*	.	.	.
11	95631873	A	T	intronic	*MTMR2*	.	.	.
11	95604630	C	G	intronic	*MTMR2*	.	.	.
22	30292115	G	A	intronic	*MTMR3*	.	.	.
8	17162310	C	A	intronic	*MTMR7*	.	.	.
8	17280986	T	A	intergenic	*MTMR7,SLC7A2*	.	.	.
X	63500333	A	G	intronic	*MTMR8*	.	.	.
X	63504683	C	T	intronic	*MTMR8*	.	.	.
X	63504979	T	A	intronic	*MTMR8*	.	.	.
X	63505636	G	A	intronic	*MTMR8*	.	.	.
X	63507608	C	T	intronic	*MTMR8*	.	.	.
X	63531536	G	T	intronic	*MTMR8*	.	.	.
X	63532158	A	G	intronic	*MTMR8*	.	.	.

X	63539443	T	C	intronic	*MTMR8*	.	.	.
X	63587764	A	C	intronic	*MTMR8*	.	.	.
X	63795984	G	T	intergenic	*MTMR8,ZC4H2*	.	.	.
X	63652456	G	T	intergenic	*MTMR8,ZC4H2*	.	.	.
X	63807720	T	A	intergenic	*MTMR8,ZC4H2*	.	.	.
X	63621206	A	G	intergenic	*MTMR8,ZC4H2*	.	.	.
X	63629180	T	C	intergenic	*MTMR8,ZC4H2*	.	.	.
X	63629181	G	A	intergenic	*MTMR8,ZC4H2*	.	.	.
X	63704949	C	A	intergenic	*MTMR8,ZC4H2*	.	.	.
X	63704951	C	G	intergenic	*MTMR8,ZC4H2*	.	.	.
X	63727882	G	A	intergenic	*MTMR8,ZC4H2*	.	.	.
X	63727884	C	G	intergenic	*MTMR8,ZC4H2*	.	.	.
X	63732675	A	T	intergenic	*MTMR8,ZC4H2*	.	.	.
X	63766900	C	T	intergenic	*MTMR8,ZC4H2*	.	.	.
X	63784571	G	C	intergenic	*MTMR8,ZC4H2*	.	.	.
X	63793636	A	C	intergenic	*MTMR8,ZC4H2*	.	.	.
X	63794119	A	G	intergenic	*MTMR8,ZC4H2*	.	.	.
X	63794321	G	A	intergenic	*MTMR8,ZC4H2*	.	.	.
X	63822097	C	T	intergenic	*MTMR8,ZC4H2*	.	.	.
X	63822098	A	G	intergenic	*MTMR8,ZC4H2*	.	.	.
X	63822330	C	G	intergenic	*MTMR8,ZC4H2*	.	.	.
X	63822594	T	G	intergenic	*MTMR8,ZC4H2*	.	.	.
X	63822595	A	C	intergenic	*MTMR8,ZC4H2*	.	.	.
X	63823075	A	C	intergenic	*MTMR8,ZC4H2*	.	.	.
X	63824144	G	T	intergenic	*MTMR8,ZC4H2*	.	.	.
X	63836797	A	T	intergenic	*MTMR8,ZC4H2*	.	.	.
X	63838420	T	C	intergenic	*MTMR8,ZC4H2*	.	.	.
X	63854425	G	A	intergenic	*MTMR8,ZC4H2*	.	.	.
X	63888296	G	A	intergenic	*MTMR8,ZC4H2*	.	.	.
X	63910930	A	T	intergenic	*MTMR8,ZC4H2*	.	.	.
X	63912504	A	G	intergenic	*MTMR8,ZC4H2*	.	.	.
X	63938509	T	C	intergenic	*MTMR8,ZC4H2*	.	.	.
X	63980204	T	C	intergenic	*MTMR8,ZC4H2*	.	.	.
X	63980205	T	C	intergenic	*MTMR8,ZC4H2*	.	.	.
X	64024235	T	G	intergenic	*MTMR8,ZC4H2*	.	.	.
X	64036990	T	C	intergenic	*MTMR8,ZC4H2*	.	.	.
X	64048344	C	T	intergenic	*MTMR8,ZC4H2*	.	.	.
X	64054247	T	C	intergenic	*MTMR8,ZC4H2*	.	.	.

X	64100928	G	A	intergenic	*MTMR8,ZC4H2*	.	.	.
X	64100929	T	C	intergenic	*MTMR8,ZC4H2*	.	.	.
X	64101021	A	T	intergenic	*MTMR8,ZC4H2*	.	.	.
X	64101986	C	T	intergenic	*MTMR8,ZC4H2*	.	.	.
X	64131069	A	T	intergenic	*MTMR8,ZC4H2*	.	.	.
X	64131071	A	C	intergenic	*MTMR8,ZC4H2*	.	.	.
4	187488640	T	C	intergenic	*MTNR1A,FAT1*	.	.	.
11	92818584	G	A	intergenic	*MTNR1B,SLC36A4*	.	.	.
11	92826279	G	T	intergenic	*MTNR1B,SLC36A4*	.	.	.
11	92736950	C	T	intergenic	*MTNR1B,SLC36A4*	.	.	.
11	92736951	A	G	intergenic	*MTNR1B,SLC36A4*	.	.	.
11	92780527	G	A	intergenic	*MTNR1B,SLC36A4*	.	.	.
11	92781105	A	G	intergenic	*MTNR1B,SLC36A4*	.	.	.
11	92844678	A	G	intergenic	*MTNR1B,SLC36A4*	.	.	.
7	135914163	AATAT	A	intergenic	*MTPN,CHRM2*	.	.	.
7	136506731	AT	A	intergenic	*MTPN,CHRM2*	.	.	.
7	136506733	AAT	A	intergenic	*MTPN,CHRM2*	.	.	.
7	135837576	T	C	intergenic	*MTPN,CHRM2*	.	.	.
7	135762179	C	T	intergenic	*MTPN,CHRM2*	.	.	.
7	135797831	T	C	intergenic	*MTPN,CHRM2*	.	.	.
7	135797832	G	A	intergenic	*MTPN,CHRM2*	.	.	.
7	135929835	T	C	intergenic	*MTPN,CHRM2*	.	.	.
7	135929836	G	A	intergenic	*MTPN,CHRM2*	.	.	.
7	136117957	T	A	intergenic	*MTPN,CHRM2*	.	.	.
7	136189030	C	T	intergenic	*MTPN,CHRM2*	.	.	.
7	136223958	C	T	intergenic	*MTPN,CHRM2*	.	.	.
7	136450179	A	G	intergenic	*MTPN,CHRM2*	.	.	.
7	136462109	A	G	intergenic	*MTPN,CHRM2*	.	.	.
7	136498835	G	A	intergenic	*MTPN,CHRM2*	.	.	.
7	136498836	T	G	intergenic	*MTPN,CHRM2*	.	.	.
7	136500476	T	G	intergenic	*MTPN,CHRM2*	.	.	.
7	136500478	G	A	intergenic	*MTPN,CHRM2*	.	.	.
1	237097439	G	C	intergenic	*MTR,MT1HL1*	.	.	.
6	153323798	C	T	exonic	*MTRF1L*	nonsynonymous_SNV	.	.
17	22050998	A	T	intergenic	*MTRNR2L1,NONE*	.	.	.
17	22220989	A	G	intergenic	*MTRNR2L1,NONE*	.	.	.
17	22227065	C	A	intergenic	*MTRNR2L1,NONE*	.	.	.

17	22240182	G	A	intergenic	*MTRNR2L1,NONE*	.	.	.
10	57457391	TA	T	intergenic	*MTRNR2L5,ZWINT*	.	.	.
10	58012872	T	A	intergenic	*MTRNR2L5,ZWINT*	.	.	.
10	57601642	T	C	intergenic	*MTRNR2L5,ZWINT*	.	.	.
10	57507048	G	T	intergenic	*MTRNR2L5,ZWINT*	.	.	.
10	57603947	T	C	intergenic	*MTRNR2L5,ZWINT*	.	.	.
10	57752124	C	A	intergenic	*MTRNR2L5,ZWINT*	.	.	.
10	57831486	T	G	intergenic	*MTRNR2L5,ZWINT*	.	.	.
[truncated: 681,010 more chars]
